# Systematic Assessment of the Catalytic Reactivity of Frustrated Lewis Pairs in C-H Bond Activation

**DOI:** 10.3390/molecules29010024

**Published:** 2023-12-19

**Authors:** Yongjie Guo, Xueqi Lian, Hao Zhang, Xueling Zhang, Jun Chen, Changzhong Chen, Xiaobing Lan, Youxiang Shao

**Affiliations:** 1 Hunan Provincial Key Laboratory of Xiangnan Rare-Precious Metals Compounds Research and Application, School of Chemistry and Environmental Science, Xiangnan University, Chenzhou 423000, China; 15973261335@163.com (Y.G.); zh15874402983@163.com (H.Z.); jchen4174@xnu.edu.cn (J.C.); czchen@xnu.edu.cn (C.C.); 2 Key Laboratory of Electronic Functional Materials and Devices of Guangdong Province, School of Chemistry and Materials Engineering, Huizhou University, Huizhou 516007, China; 2006070302129@stu.hzu.edu.cn (X.L.); zxl@ahnu.edu.cn (X.Z.)

**Keywords:** C-H activation, frustrated Lewis pairs, catalytic reactivity, systematic assessment, DFT calculations

## Abstract

Unreactive C-H bond activation is a new horizon for frustrated Lewis pair (FLP) chemistry. This study provides a systematic assessment of the catalytic reactivity of recently reported intra-molecular FLPs on the activation of typical inert C-H bonds, including 1-methylpyrrole, methane, benzyl, propylene, and benzene, in terms of density functional theory (DFT) calculations. The reactivity of FLPs is evaluated according to the calculated reaction thermodynamic and energy barriers of C-H bond activation processes in the framework of concerted C-H activation mechanisms. As for 1-methylpyrrole, 14 types of N-B-based and 15 types of P-B-based FLPs are proposed to be active. Although none of the evaluated FLPs are able to catalyze the C-H activation of methane, benzyl, or propylene, four types of N-B-based FLPs are suggested to be capable of catalyzing the activation of benzene. Moreover, the influence of the strength of Lewis acid (LA) and Lewis base (LB), and the differences between the influences of LA and LB on the catalytic reactivity of FLPs, are also discussed briefly. This systematic assessment of the catalytic activity of FLPs should provide valuable guidelines to aid the development of efficient FLP-based metal-free catalysts for C-H bond activation.

## 1. Introduction

Exploring cost-efficient, resource-abundant, and highly active metal-free catalysts as a potential alternative to metallic catalysts is a contemporary challenge in the context of organic synthesis and other important chemical processes. Several strategies in developing metal-free catalysts have been proposed during recent decades, including unsaturated heavier main-group compounds [1,2], the singlet carbenes [3], and frustrated Lewis pairs (FLPs) [4,5,6,7,8]. Among these prominent approaches, FLPs have attracted great attention as they make the classic Lewis acid-based concept shine with vitality. It is well-known that the Lewis acid (LA) and Lewis base (LB) could contact strongly with the formation of classical LA–LB covalent adducts. Different to the classical LA–LB covalent adduct, the LA and LB moieties in FLPs are independent without the formation of a strong dative covalent bond due to the electronic or steric encumbrance originating from very bulky substituents at the LA and LB centers. As a result, FLPs could play the role of an electronic donor and electronic accepter simultaneously, like bifunctional transition-metal complexes, which leads to the unprecedented catalytic reactivity of FLPs.

Ever since Stephan discovered that *p*-(Mes_2_PH)C_6_F_4_(BH(C_6_F_5_)_2_) could efficiently catalyze the splitting of hydrogen reversibly under mild conditions [8], these FLP-based metal-free bifunctional catalysts have attracted incredible attention and burgeoned into an intriguing alternative for transition metal-based catalysts. On one hand, a large number of original FLPs have been designed for different purposes up until now; for example, the aluminum-based FLPs [9,10,11,12], the N-heterocyclic carbene-based FLPs [13], the carbon-based FLPs [14], and even the solid FLPs [15,16]. On the other hand, the applications of FLPs as versatile catalysts have extended to multiple aspects of chemistry. In the beginning, research on the applications of FLPs paid attention mainly to the activation of the E-H (E = O [17,18,19], S [20], N [21,22], Si [23,24], B [25,26], etc.) bond and their interaction with a variety of small molecules [27,28], which are often thought to be the typical domain of transition metal-containing chemistry. Later, the ability to cleave molecular hydrogen under mild conditions reversibly inspired researches to uncover whether FLPs are able to catalyze C-H bond activation, which has been considered as one of the holy grails of organic chemistry. Initially, studies in this field focused on the activation of the C(sp)-H bond [29,30,31,32], and this has been extended expeditiously to include the relative inactive C(sp^2^)-H bond [33,34,35,36,37]. In contrast, the activation of the most inert C(sp^3^)-H bond is still elusive and only a few metal-free systems for this have been reported [38,39,40]. In particular, Fontaine and coworkers recently reported that an *ansa*-aminoborane-based FLP (2-NMe_2_-C_6_H_4_)_2_BH is able to activate the C(sp^3^)-H bond of a methyl group [41]. Although such pioneering research has verified the paradigm of C-H bond activation using FLP-based metal-free catalysts, there is lack of guidelines for the rational design of more cost-efficient FLPs. As a result, it is necessary to evaluate the reactivity of various previously reported FLPs towards C-H bond activation systemically, which will provide helpful information for developing cost-efficient FLPs for the future of C-H bond activation.

Generally speaking, FLPs could be divided into intermolecular FLPs and intra-molecular FLPs according to whether their LA and LB sites are closely connected by sufficiently flexible bridges. Compared to intermolecular FLPs, intra-molecular FLPs with different covalent linkers show more variety and controllability in tuning the cooperativity between the LA and LB sites. Moreover, these covalent linkers allow the usage of less bulkier acidic and basic fragments. These advantages are beneficial for controlling the typical behaviors of FLPs. Therefore, the present study mainly focuses on the catalytic reactivity of an assortment of intra-molecular FLPs. In fact, the rational design of intermolecular FLPs, especially concerning the influence of LA and LB on the reactivity of intermolecular FLPs towards H_2_ activation, has been evaluated by Pápai’s research group [42].

In an effort to obtain deeper insights into the factors that determine the reactivity of FLP-catalyzed C-H bond activation and provide rational guidelines for developing more cost-efficient FLPs, the present study performed systemic DFT calculations on the thermodynamics and kinetics of FLP-catalyzed typical inert C-H bond activation, including 1-methylpyrrole, methane, benzyl, propylene, and benzene. Moreover, the catalytic activity of several candidates was discussed briefly based on the systematic assessment.

## 2. Results and Discussion

### 2.1. The Scope of FLPs and the Framework of the C-H Activation Mechanism

**Figure 1:**
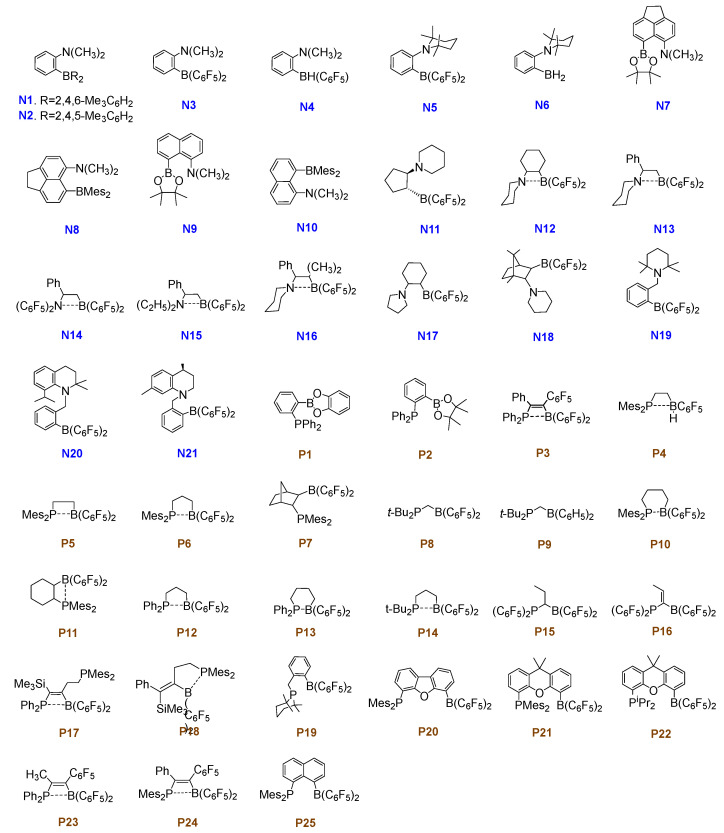
The structure of previously reported N-B- and P-B-type FLPs. The detailed references are available in the Supplementary Materials.

Aiming to obtain a comprehensive picture of the FLP-catalyzed C-H bond activation, we collected a large set of FLPs which have been reported for their success in versatile catalytic applications. As the scope of FLPs has expanded extensively since the notion of FLPs was first proposed by Stephan [8], only the intra-molecular FLPs are taken into account in this study. Owing to the remarkable contributions of many researchers, various phosphine- and nitrogen-based types of LB have been developed for FLP chemistry, while research on LA has mainly been focused on boron-based compounds, although several aluminum- and transition metal-based types of LA have been described [43,44,45,46]. Due to the complexity of FLPs, only the previously reported FLPs comprising phosphine- and nitrogen-based LBs and boron-based LA with different linking fragments were considered in this study. The structure of these FLPs discussed in present study are depicted in Figure 1 (more details see Table S1 in the Supplementary Materials).

**Scheme 1:**
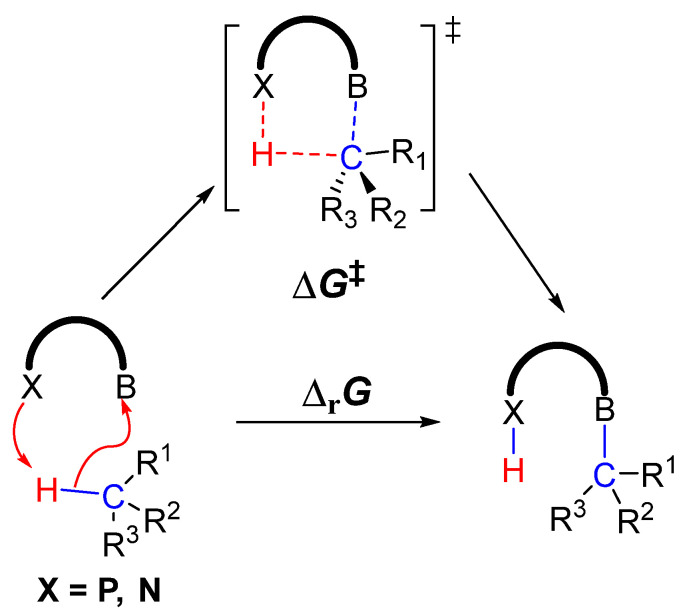
The concerted mechanism of intra-molecular FLP-catalyzed C-H bond activation.

As unreactive C-H bond activation is a new horizon for FLP chemistry, the mechanisms behind FLP-catalyzed C-H bond activation have not been explored extensively. Only Fontaine and coworkers [36,41] have proposed a concerted heterolysis mechanism, similar to that in H_2_ activation, by studying the C-H bond activation of 1-methylpyrrole. Homolysis or radical mechanisms [47,48,49], proposed recently for FLP-catalyzed H_2_ activation, are scarcely reported in relation to C-H bond activation. As a result, in order to simplify the discussions on FLPs and to compare the reactivity of different FLPs, we calculated all of the overall solvent-phase Gibbs free energies (∆_r_*G*) and the free energy barrier (∆*G*^‡^) of the C-H bond activation reaction according to the concerted heterolysis mechanism, as shown in Scheme 1. It is expected that the calculated ∆_r_*G* and ∆*G*^‡^ should vary across a remarkably wide range; thus, a reasonable standard is of crucial importance to evaluate the reactivity of FLPs. Of note, although it has been proposed that experimentally unreactive FLPs are characterized by ∆_r_*G* values of typically more than 10 kcal/mol in the activation of hydrogen, the *t*Bu_3_P/BPh_3_ FLP was found to be reactive with ∆_r_*G* values as high as 18.2 kcal/mol [41]. Therefore, all systems with ∆_r_*G* under 20 kcal/mol were considered to be potential candidates for catalyzed C-H bond activation in view of thermodynamics. On the other hand, those with a free energy barrier (∆*G*^‡^) under 30 kcal/mol were utilized to appraise the catalytic reactivity of FLPs in view of kinetics. Indeed, the free energy barrier of the active FLPs in [32,38,39,40] is calculated to be 24.4 kcal/mol and 25.5 kcal/mol, respectively. It is necessary to point out that the values of 30 kcal/mol and 20 kcal/mol are merely reference values rather than absolute benchmarks for the reactivity of FLPs.

### 2.2. The Performance of FLPs on the C-H Bond Activation of 1-Methylpyrrole

**Figure 2:**
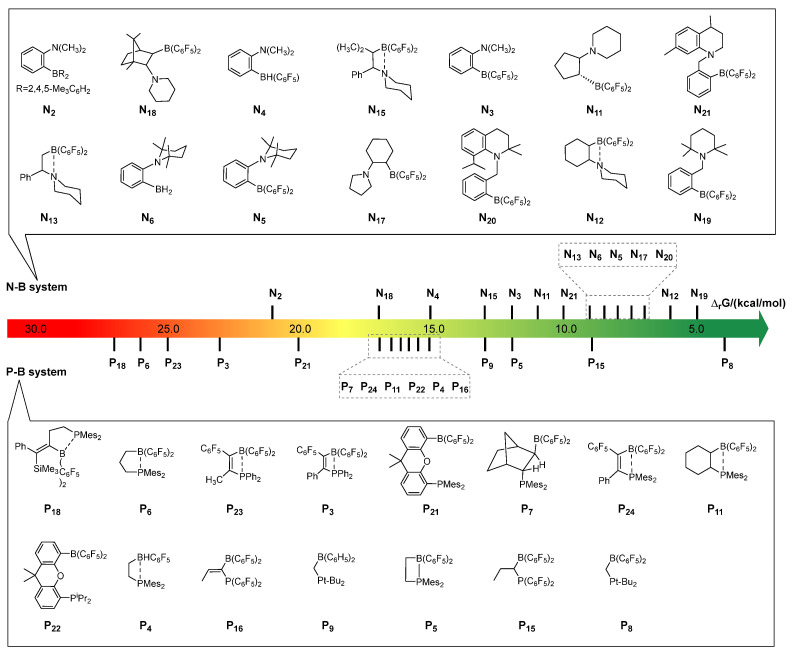
The calculated free energies (∆_r_*G*) of the FLP-catalyzed C-H bond activation of 1-methylpyrrole.

**Figure 3:**
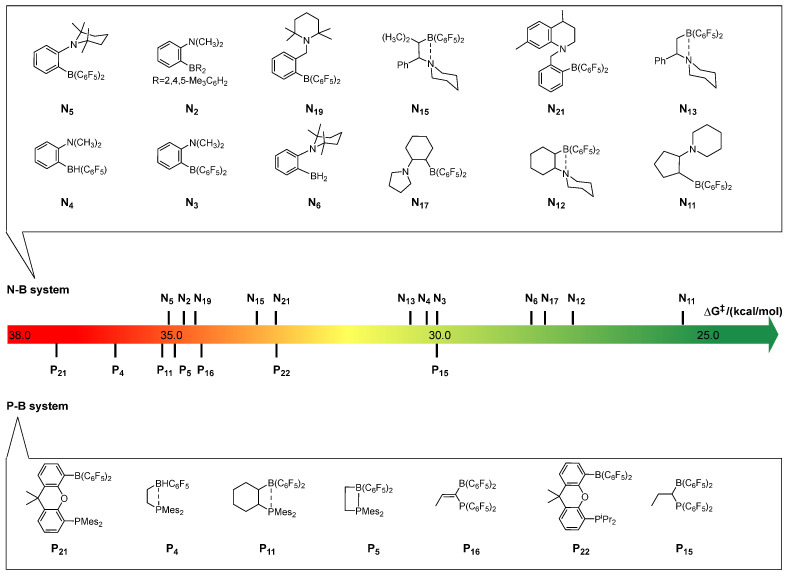
The calculated free energy barrier (∆*G*^‡^) of the FLP-catalyzed C-H bond activation of 1-methylpyrrole.

The catalyzed C-H bond activation of 1-methylpyrrole was evaluated firstly because Fontaine and coworkers [20] have confirmed that *ansa*-aminoborane-based FLPs (2-NMe_2_-C_6_H_4_)_2_BH are capable of catalyzing the C-H bond activation of 1-methylpyrrole. It is necessary to evaluate whether this C-H bond activation of 1-methylpyrrole is a common reactivity pattern similar to those previously reported for other FLPs. The calculated thermodynamic (∆_r_*G*) and kinetic (∆*G*^‡^) results are collected in Figure 2 and Figure 3 (more details see Figure S1 in the Supplementary Materials). To our delight, the overall performance of these FLPs on the C-H activation of 1-methylpyrrole is acceptable. As can be seen in Figure 2, there are 14 types of N-B-based FLPs and 15 types of P-B-based FLPs that fall into the region in which the ∆_r_*G* is less than 30 kcal/mol. Obviously, these FLPs should afford the C-H activation of 1-methylpyrrole. It is worth noting that the reactivity of N-B-based FLPs may be more efficient than that of P-B-based FLPs; this is probably due to the fact that the electronegativity of a N atom is stronger than that of a P atom.

The origin of the differences in catalytic reactivity between these FLPs is complicated; however, the influencing factors mainly include the electronic effect and the distance effect. The electronic effect is induced by the LA and LB comprising the FLPs, while the distance effect is caused by the linkers. For example, although the structures of **N1** and **N3** are very similar except the substituent of the LA site, the catalytic reactivities of these two FLPs are different from each other. This could be attributed to the electronic effect evoked by the LA site. In the case of **N1**, the electron-donating group 2,4,6-Me_3_C_6_H_2_ reduces the electrophilicity of the LA site, which weakens the strength of the LA, and thus depresses the catalytic activity of **N1**. Contrarily, the electron-withdrawing group C_6_F_5_ in **N3** could reinforce the strength of LA, which is beneficial for the catalytic activity of **N3**. Another good example is the results of **N13**, **N14**, and **N15**. Although these three FLPs possess similar backbone scaffolds, their catalytic reactivities are quite distinct from one another, as the substituents on LB center N are different. The electron-withdrawing group of the N center weakens the strength of LB, which leads to the diminishing of catalytic activity of **N14**. On the contrary, the activity of **N13** and **N15** is strengthened, attributed to the electron-donating group on the N center. Briefly, the substituents of the LA and LB sites are of crucial importance to the catalytic reactivity of FLPs.

**Figure 4:**
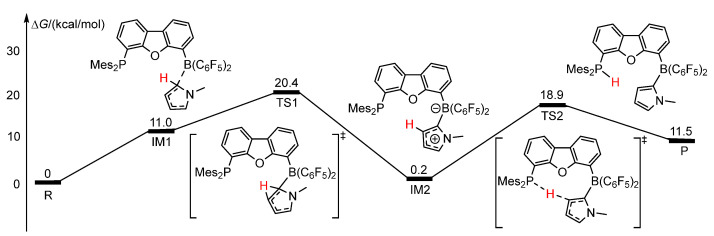
The proposed stepwise mechanism for **P20**-catalyzed C-H bond activation of 1-methylpyrrole.

As for the distance effect, the comparison of di-benzofuran-derived **P20** and xanthene-based system **P21** provides a concise example. The thermodynamic reactions are almost identical for **P20** and **P21**; however, a corresponding transition state of **P20** similar to that of **P21** could not be located. This could be attributed to the distance effect, i.e., the P–B distance is 5.669 Å for **P20** and 4.243 Å for **P21** according to the XRD experiment [50]. The distance between the LA and LB sites in **P20** is so long that the cooperative interaction of substrates with LA and LB sites is prohibitive, making the concerted C-H bond activation mechanism unfavorable. Fortunately, we found an alternative mechanism for **P20**-catalyzed C-H bond activation of 1-methylpyrrole, as shown in Figure 4. It is obvious that the distance between the LA and LB sites could affect the mechanism of FLP-catalyzed C-H activation of 1-methylpyrrole. This distance-controlled stepwise mechanism is in accordance with our previous work [51].

### 2.3. The Performance of FLPs on C-H Bond Activation in Methane, Methylbenzene, Propylene, and Benzene

**Figure 5:**
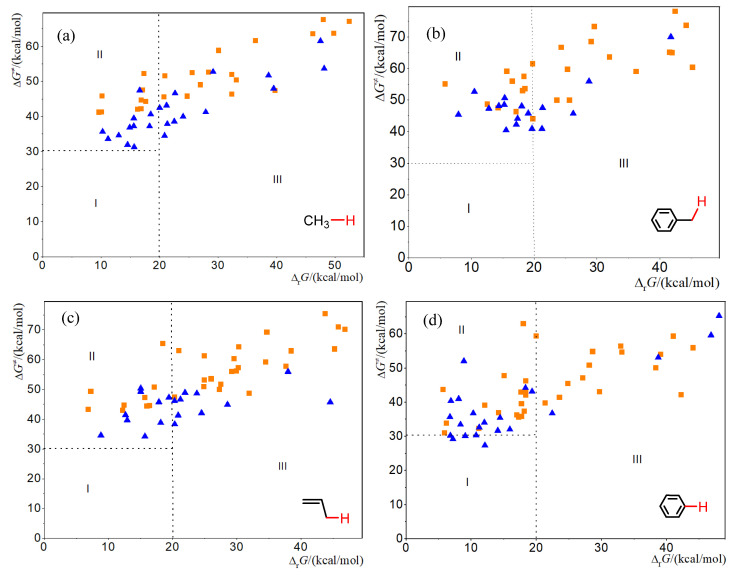
The calculated free energies (∆_r_*G*) and the free energy barrier (∆*G*^‡^) of the C-H bond activation of (**a**) methane, (**b**) methylbenzene, (**c**) propylene, and (**d**) benzene. ■: FLPs **P1**–**P25**; ▲: FLPs **N1**–**N21**. I: ∆_r_*G* < 20 kcal/mol and ∆*G*^‡^ < 30 kcal/mol; II: ∆_r_*G* < 20 kcal/mol and ∆*G*^‡^ > 30 kcal/mol; III: ∆_r_*G* > 20 kcal/mol and ∆*G*^‡^ > 30 kcal/mol.

Motivated by the results of the FLP-catalyzed C-H bond activation of 1-methylpyrrole discussed above, we continued to evaluate the catalytic performance of FLPs on the activation of more inert C-H bonds, i.e., methane, methylbenzene, propylene with sp^3^ hybrid C-H bonds, and benzene with sp^2^ C-H bonds. The calculated results are collected in Figure 5. The catalyzed C-H bond activation of methane was discussed as a model system because methane has long been considered as one of the most important hydrocarbon feedstocks of fuels and chemicals in the excessive development and usage of traditional fossil energy reserves. Although this resource is abundant, the efficient usage of methane is a great challenge since the activation of methane under ambient conditions is extremely difficult, which could be attributed to the extremely strong sp^3^ C-H bonds in methane. The results of various intra-molecular FLP-catalyzed C-H bond activations of methane are exhibited in Figure 5a. Unfortunately, the overall performance of these FLPs on the C-H activation of methane is unsatisfactory. The reaction is forbidden both kinetically and thermodynamically as both the ∆_r_*G* and ∆*G*^‡^ values are very large. Obtaining these results is not surprising, as the C-H bond of methane is extremely inert. It is worth noting that there are several N-B-type FLPs located in zone II with an energy barrier of about 30 kcal/mol. As a result, if the energy barrier could be reduced slightly, these FLPs could become the candidates for catalyzing the C-H activation of methane. However, there may be alternative reaction mechanisms, as the present study only considers the concerted heterolysis mechanism. Moreover, the catalytic reactivity of these FLPs could be modified by tuning the electronic structure of the LA, LB, and linker moieties.

In order to obtain a systemic understanding of the catalytic reactivity of FLPs, propylene and methylbenzene with more active (sp^3^) C-H bonds than methane were examined as well. The calculated kinetic and thermodynamic data are collected in Figure 5b and Figure 5c, respectively. It is discouraging that the total performance of all FLPs discussed in the present study is unsatisfactory, similar to the results of methane. The sp^2^ hybrid C-H bond in benzene should be more active than that of methane, methylbenzene, and propylene, thus it could be catalyzed by FLPs. In fact, Chernichenko and coworkers [35] revealed very recently that aminohydroborane is able to catalyze the C-H bond activation of benzene. As a result, the catalyzed C-H bond activation of benzene was evaluated here. These calculated thermodynamic and kinetic data are depicted in Figure 4d. It is obvious that although the overall performance of the various studied FLPs is not satisfactory, the catalytic reactivity of N-B-based FLPs has improved, as there are four N-B-type FLPs located in zone I (Figure 5d). The distinct differences between benzene and methylbenzene should be attributed to the distinguishing reactivity of the sp^2^ and sp^3^ hybrid C-H bonds. It is worth pointing out that the performance of aminohydroborane-based FLP **N6** is in good accordance with the results found by Chernichenko and coworkers [31]. In summary, the catalytic reactivity of FLPs on the C(sp^3^)-H bond activation of methane, methylbenzene, and propylene is unsatisfactory, yet there are four N-B-based FLPs (Figure 5d) that are potential catalysts for the C(sp^2^)-H bond activation of benzene.

### 2.4. The Influence on the Reactivity of FLPs

**Scheme 2:**
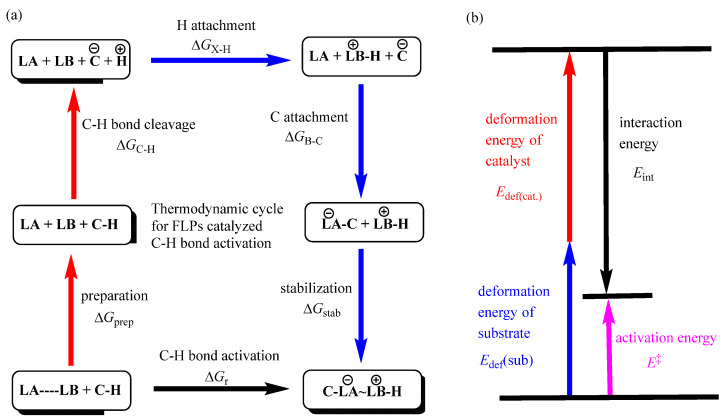
Partitioning of the overall free energy of FLP-catalyzed C-H bond activation. (**a**) Thermodynamic cyclefor FLPs catalyzedC-H bond activation. (**b**) activation distortion-interaction energy decomposition analysis of transition states.

In order to understand the intrinsic relationship between the structure of FLPs and their thermodynamic performance in C-H bond activation, and to provide useful guidelines for rationally improving the catalytic efficiency of FLPs, it is necessary to analyze the composition of the overall free energy. According to the framework of the concerted heterolysis C-H bond activation mechanism, a catalyzed C-H bond activation involves the preparation of frustrated LA and LB; in cases where active LA and LB sites are quenched, the cleavage of the C-H bond, the attachment of H^+^ to the LB site, the attachment of C to the LA site, and the stabilization of LA–C and LB–H moieties. Thus, it is reasonable to partition the overall free energy (Δ*G_r_*) into five components, i.e., the preparation energy (Δ*G*_prep_), the formation energy of newly formed H-X (X = N, P) and B-C bonds (Δ*G*_X-H_ and Δ*G*_B-C_), the deprotonation energy of the broken C-H bond (Δ*G*_C-H_), and the stabilization energy (Δ*G*_stab_), as depicted in Scheme 2. Therefore, the overall thermodynamics of the C-H bond activation reaction could be described as Equation (1):

Δ*G_r_* = Δ*G*_prep_ + Δ*G*_C-H_ + Δ*G*_X-H_ + Δ*G*_B-C_ + Δ*G*_stab_ (1)

In Equation (1), Δ*G*_prep_ is always negligible, as by definition, FLPs require the LA and LB to be separated. Δ*G*_C-H_ can be measured with the bond dissociation energy (BDE), which could be obtained from experiment results [52]. For the same substrate, Δ*G*_C-H_ is constant for all FLPs. Moreover, Δ*G*_stab_ has been suggested to vary insignificantly [38,39,40]. As a result, only Δ*G*_X-H_ and Δ*G*_B-C_ are thought to change remarkably with variations in the FLPs. In other words, the thermodynamic performance of intra-molecular FLPs in C-H bond activation should depend on the magnitude of Δ*G*_X-H_ and Δ*G*_B-C_.

Generally, Δ*G*_X-H_ represents the ability of LB to accept a proton, which could be judged according to proton affinity (PA). A large PA results in a large Δ*G*_X-H_. In theory, the value of PA could be obtained from reference [53]. Considering the fact that the FLPs discussed in the present study comprise nitride- or phosphine-based LBs, it is reasonable to judge the relative strength of the LB by comparing the electronic properties of the substituents of the LB. For example, although the frameworks of **N13**, **N14**, and **N15** are identical, the catalytic reactivity of these FLPs on the C-H bond activation of 1-methylpyrrole are different owing to the fact that the substituents of boron are different. The electron-withdrawing substituents C_6_F_5_ could decrease the electron density of the LB center, which leads to the decreases in the PA. As a result, the value of Δ*G*_X-H_ becomes small. Accordingly, the overall thermodynamics of **N14**-catalyzed C-H bond activation become unfavorable. In order to improve the activity of FLPs, it is necessary to employ a stronger LB; for example, the nitride- or phosphine-based LB with electron-donating substituents.

On the other hand, Δ*G*_B-C_ stands for the ability of LA to accept electrons; in other words, the strength of the LA determines the value of Δ*G*_B-C_. In principle, the LA strength could be measured via the Gutmann–Beckett method [54,55,56]. As the FLPs considered in the present study are all combined by boron-based LA, the relative strength of the LA could be estimated according to the electronic properties of the boron substituents, as discussed above. The electron-withdrawing groups weaken the electron density of B, which is beneficial for the interaction of C with B, leading to the decreased Δ*G*_B-C_. Therefore, the overall thermodynamics are favorable. In summary, electron-withdrawing groups are essential for improving the catalytic activity of FLPs.

It is noteworthy that the nature of the linker has a great influence on the catalytic reactivity of FLPs, as discussed regarding the differences between **P20** and **P21** due to the distance between their two active sites. However, the impact of the linker structure on the catalytic reactivity of FLPs is not easily predictable, as proposed by Ashley and coworkers [57]. More detailed and systemic research is required to undercover the mystery of how linkers affect the electronic properties and catalytic reactivity of FLPs.

## 3. Materials and Methods

### Computational Methods

According to previous research in the literature, in which DFT methods were commonly used in assessing the performance of FLPs [58,59,60,61], the geometries of all reactants, intermediates, transition states, and products were fully optimized at the M06-2X/6-31G(d,p) level of theory [62] in the gas phase. Harmonic vibrational frequencies were calculated at the same level of theory for the characterization of stationary points (minimum or transition states) and for the zero-point energy (ZPE) corrections. Intrinsic reaction coordinate (IRC) [63,64,65] calculations were carried out to verify the predicted transition states connecting the designated reactants and products. In order to obtain more-accurate thermodynamic energies, the M06-2X/6-311++G(d,p) method was employed to calculate the single-point energies based on the geometries optimized at the M06-2X/6-31G(d,p) level of theory. The continuum polarized solvent model SMD [66] was employed to evaluate the solvent effect of toluene during the single-point energy calculations. The final Gibbs free energy with the SMD solvent model was calculated according to previous research [67]. All DFT calculations in this work were carried out using the Gaussian09 software package (Revision B.01) [68].

## 4. Conclusions

In summary, the catalytic reactivity of a series of recently reported intra-molecular FLPs in C-H bond activation was systemically evaluated using DFT methods. The overall performance of these FLPs on the C-H bond activation of 1-methylpyrrole is very excellent. In total, 14 types of N-B-based FLPs and 15 types of P-B-based FLPs are proposed to be of a good catalytic reactivity. In contrast, our results are unsatisfactory in the cases of methane, benzyl, propylene, and benzene. There are only four types of N-B-based FLPs that were shown to be active in the C-H activation of benzene. Moreover, the electronic effect on the reactivity of FLPs was briefly analyzed in relation to thermodynamics. An LA with electron-withdrawing groups and an LB with electron-withdrawing groups are required to develop more efficient FLP-based metal-free catalysts. This evaluation of the catalytic reactivity of FLPs and insight into the related influencing factors will provide useful guidelines for the rational design of novel FLPs.

## Data Availability

Data are contained within the article and Supplementary Materials.

## Supplementary Materials

The following supporting information can be downloaded at: https://www.mdpi.com/article/10.3390/molecules29010024/s1, Table S1: The detailed information of FLPs discussed in the manuscript; Figure S1: The calculated free energies (ΔrG) and the free energy barrier (ΔG^‡^) of the C-H bond activation of (A) methane, (B) methylbenzene, (C) propylene and (D) benzene. the detailed information of FLPs, additional references, optimized coordinates of collected FLPs, coordinates of intermediates and transition states involved in Figure 1, Figure 2, Figure 3, Figure 4 and Figure 5. Refs [69,70,71,72,73,74,75,76,77,78,79,80,81,82,83,84,85,86,87,88,89,90,91,92,93] are cited in the Supplementary Materials.
