# Supplementary material for: Systematic Assessment of the Catalytic Reactivity of Frustrated Lewis Pairs in C-H Bond Activation
Source: Molecules. 2023 Dec 19;29(1):24. doi: 10.3390/molecules29010024 (PMC10780200; doi:10.3390/molecules29010024)
Supplement: Supplementary file 1 [file molecules-29-00024-s001.zip › molecules-2761477-supplementary.pdf]

Supporting Information

Systematic Assessment of the Catalytic Reactivity of Frustrated Lewis Pairs on C-H Bond Activation

Yongjie Guo [1,†], Xueqi Lian [2,†], Hao Zhang [1], Xueling Zhang [2], Jun Chen [1], Changzhong Chen [1], Xiaobing Lan [1,*] and Youxiang Shao [2,*]

1 Hunan Provincial Key Laboratory of Xiangnan Rare-Precious Metals Compounds Research and Application, School of Chemistry and Environmental Science, Xiangnan University, Chenzhou 423000, China

2 School of Chemistry and Materials Engineering, Key Laboratory of Electronic Functional Materials and Devices of Guangdong Province, Huizhou University, Huizhou 516007, China

* Correspondence: xblan@xnu.edu.cn; shaoyx@whu.edu.cn

[†] These authors contributed equally to this work.

Table of Contents

1. Table S1. The detailed information of FLPs discussed in the manuscript…………2

2. References……………………………………………………………………..6

3. Figure S1. The calculated free energies ($\Delta_r G$) and the free energy barrier ($\Delta G^{‡}$) of the C-H bond activation……………………………………………………………11

4. Optimized coordinates of collected FLPs……….……………………………………11

5. Coordinates of intermediates and transition states involved in Figure 2 and Figure 3....……………………………………………………………………………………44

6. Coordinates of intermediates and transition states involved in Figure 4...…….…117

7. Coordinates of intermediates and transition states involved in Figure 5.....…….123

Table S1. The detailed information of FLPs discussed in the manuscript.

number	structure	reference
**N1**	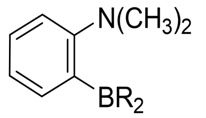 R=2,4,6-$Me_3C_6H_2$	[1]
**N2**	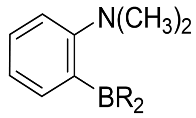 R=2,4,5-$Me_3C_6H_2$	[1]
**N3**	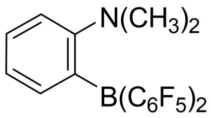	[2-3]
**N4**	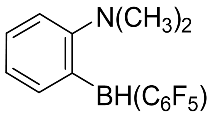	[3]
**N5**	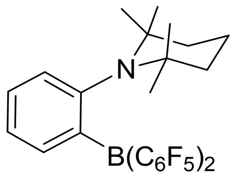	[2]
**N6**	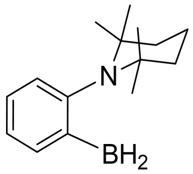	[1]
**N7**	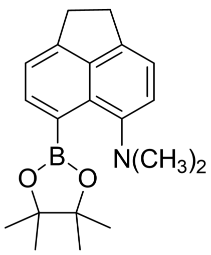	[4]
**N8**	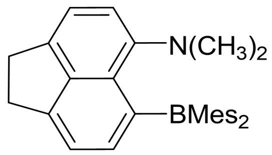	[4]
**N9**	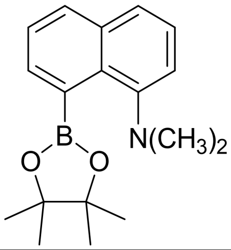	[4]

**N10**	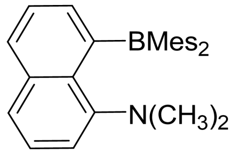	[4]
**N11**	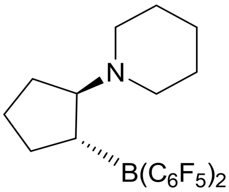	[5]
**N12**	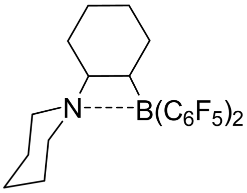	[6]
**N13**	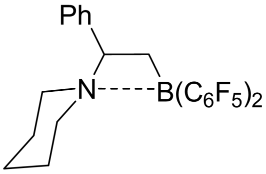	[6]
**N14**	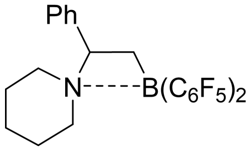	[6]
**N15**	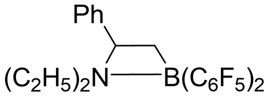	[6]
**N16**	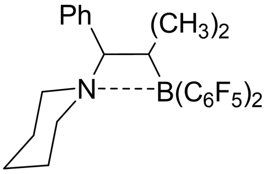	[6]
**N17**	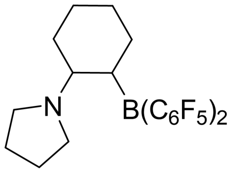	[6]
**N18**	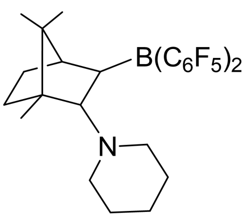	[7-8]

**N19**	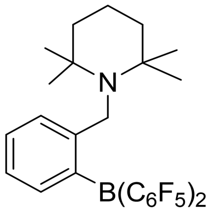	[9]
**N20**	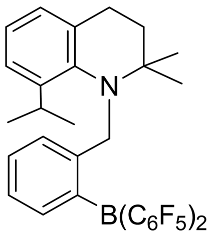	[10]
**N21**	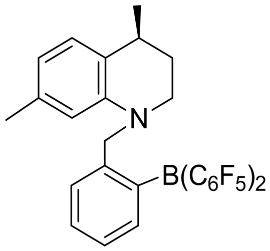	[10]
**P1**	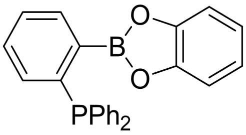	[11]
**P2**	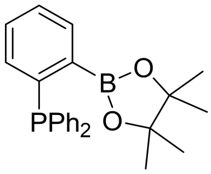	[12]
**P3**	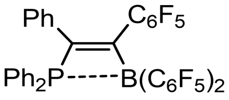	[13]
**P4**	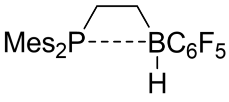	[14]
**P5**	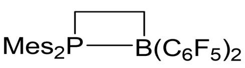	[15]
**P6**	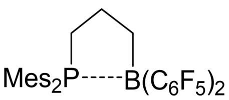	[16]
**P7**	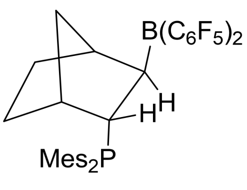	[17]

**P8**	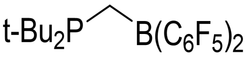	[18]
**P9**	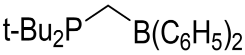	[18]
**P10**	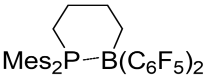	[19]
**P11**	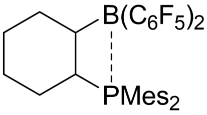	[20]
**P12**	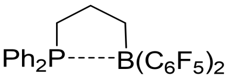	[21]
**P13**	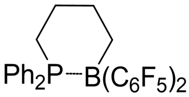	[21]
**P14**	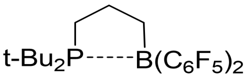	[21]
**P15**	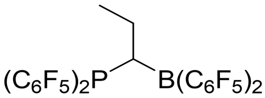	[22]
**P16**	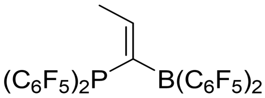	[23]
**P17**	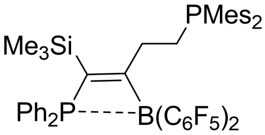	[24]
**P18**	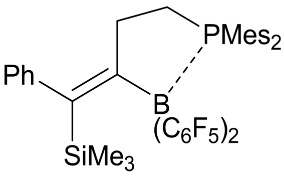	[24]
**P19**	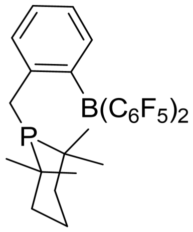	[25]
**P20**	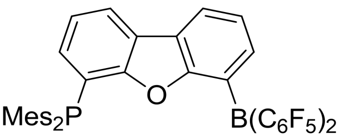	[26]

**P21**	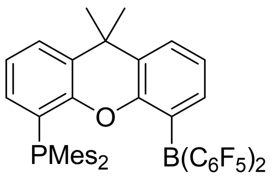	[26]
**P22**	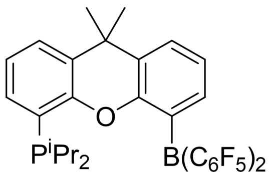	[26]
**P23**	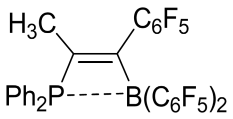	[13]
**P24**	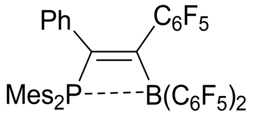	[13, 27]
**P25**	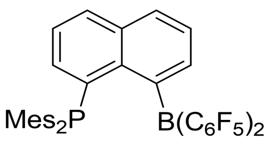	[28]

References

1. Chernichenko, K.; Kótai, B.; Pápai, I.; Zhivonitko, V.; Nieger, M.; Leskelä, M.; Repo, T., Intramolecular Frustrated Lewis Pair with the Smallest Boryl Site: Reversible H_2 Addition and Kinetic Analysis. *Angew. Chem., Int. Ed.* **2015,** *54*, 1749-1753.

2. Chernichenko, K.; Nieger, M.; Leskelä, M.; Repo, T., Hydrogen activation by 2-boryl-N,N-dialkylanilines: a revision of Piers' ansa-aminoborane. *Dalton Trans.* **2012,** *41*, 9029-9032.

3. Chernichenko, K.; Madarász, Á.; Pápai, I.; Nieger, M.; Leskelä, M.; Repo, T., A frustrated-Lewis-pair approach to catalytic reduction of alkynes to cis-alkenes. *Nat. Chem.* **2013,** *5*, 718-723.

4. Pla, D.; Sadek, O.; Cadet, S.; Mestre-Voegtlé, B.; Gras, E., Naphthylaminoborane: from structural switches to frustrated Lewis pair reactivity. *Dalton Trans.* **2015,** *44*, 18340-18346.

5. Xu, B.-H.; Bussmann, K.; Fröhlich, R.; Daniliuc, C. G.; Brandenburg, J. G.; Grimme, S.; Kehr, G.; Erker, G., An Enamine/HB(C_6F_5)$_2$ Adduct as a Dormant State in Frustrated Lewis Pair Chemistry. *Organometallics* **2013,** *32*, 6745-6752.

6. Schwendemann, S.; Fröhlich, R.; Kehr, G.; Erker, G., Intramolecular frustrated N/B lewis pairs by enamine hydroboration. *Chem. Sci.* **2011,** *2*, 1842-1849.

7. Schwendemann, S.; Oishi, S.; Saito, S.; Fröhlich, R.; Kehr, G.; Erker, G., Reaction of an "Invisible" Frustrated N/B Lewis Pair with Dihydrogen. *Chem. Asian J.* **2013,** *8*, 212-217.

8. Lindqvist, M.; Axenov, K.; Nieger, M.; Räisänen, M.; Leskelä, M.; Repo, T., Frustrated Lewis Pair Chemistry of Chiral (+)-Camphor-Based Aminoboranes. *Chem. Eur. J.* **2013,** *19*, 10412-10418.

9. Sumerin, V.; Schulz, F.; Atsumi, M.; Wang, C.; Nieger, M.; Leskelä, M.; Repo, T.; Pyykkö, P.; Rieger, B., Molecular Tweezers for Hydrogen: Synthesis, Characterization, and Reactivity. *J. Am. Chem. Soc.* **2008,** *130*, 14117-14119.

10. Sumerin, V.; Chernichenko, K.; Nieger, M.; Leskelä, M.; Rieger, B.; Repo, T., Highly Active Metal-Free Catalysts for Hydrogenation of Unsaturated Nitrogen-Containing Compounds. *Adv. Synth. Catal.* **2011,** *353*, 2093-2110.

11. Courtemanche, M.-A.; Légaré, M.-A.; Maron, L.; Fontaine, F.-G., A Highly Active Phosphine–Borane Organocatalyst for the Reduction of CO_2 to Methanol Using Hydroboranes. *J. Am. Chem. Soc.* **2013,** *135*, 9326-9329.

12. Porcel, S.; Bouhadir, G.; Saffon, N.; Maron, L.; Bourissou, D., Reaction of Singlet Dioxygen with Phosphine–Borane Derivatives: From Transient Phosphine Peroxides to Crystalline Peroxoboronates. *Angew. Chem., Int. Ed.* **2010,** *49*, 6186-6189.

13. Wiegand, T.; Eckert, H.; Ekkert, O.; Fröhlich, R.; Kehr, G.; Erker, G.; Grimme, S., New Insights into Frustrated Lewis Pairs: Structural Investigations of Intramolecular Phosphane–Borane Adducts by Using Modern Solid-State NMR Techniques and DFT Calculations. *J. Am. Chem. Soc.* **2012,** *134*, 4236-4249.

14. Erdmann, M.; Rösener, C.; Holtrichter-Rößmann, T.; Daniliuc, C. G.; Fröhlich,

R.; Uhl, W.; Würthwein, E.-U.; Kehr, G.; Erker, G., Functional group chemistry at intramolecular frustrated Lewis pairs: substituent exchange at the Lewis acid site with 9-BBN. *Dalton Trans.* **2013,** *42*, 709-718.

15. Spies, P.; Erker, G.; Kehr, G.; Bergander, K.; Fröhlich, R.; Grimme, S.; Stephan, D. W., Rapid intramolecular heterolytic dihydrogen activation by a four-membered heterocyclic phosphane–borane adduct. *Chem. Commun.* **2007**, 5072-5074.

16. Spies, P.; Kehr, G.; Bergander, K.; Wibbeling, B.; Fröhlich, R.; Erker, G., Metal-free dihydrogen activation chemistry: structural and dynamic features of intramolecular P/B pairs. *Dalton Trans.* **2009**, 1534-1541.

17. Sajid, M.; Kehr, G.; Wiegand, T.; Eckert, H.; Schwickert, C.; Pöttgen, R.; Cardenas, A. J. P.; Warren, T. H.; Fröhlich, R.; Daniliuc, C. G.; Erker, G., Noninteracting, Vicinal Frustrated P/B-Lewis Pair at the Norbornane Framework: Synthesis, Characterization, and Reactions. *J. Am. Chem. Soc.* **2013,** *135*, 8882-8895.

18. Bertini, F.; Lyaskovskyy, V.; Timmer, B. J. J.; de Kanter, F. J. J.; Lutz, M.; Ehlers, A. W.; Slootweg, J. C.; Lammertsma, K., Preorganized Frustrated Lewis Pairs. *J. Am. Chem. Soc.* **2012,** *134*, 201-204.

19. Wang, X.; Kehr, G.; Daniliuc, C. G.; Erker, G., Internal Adduct Formation of Active Intramolecular C4-bridged Frustrated Phosphane/Borane Lewis Pairs. *J. Am. Chem. Soc.* **2014,** *136*, 3293-3303.

20. Axenov, K. V.; Mömming, C. M.; Kehr, G.; Fröhlich, R.; Erker, G., Structure and Dynamic Features of an Intramolecular Frustrated Lewis Pair. *Chem. Eur. J.* **2010,** *16*, 14069-14073.

21. Spies, P.; Fröhlich, R.; Kehr, G.; Erker, G.; Grimme, S., Structural Importance of Secondary Interactions in Molecules: Origin of Unconventional Conformations of Phosphine–Borane Adducts. *Chem. Eur. J.* **2008,** *14*, 333-343.

22. Stute, A.; Kehr, G.; Fröhlich, R.; Erker, G., Chemistry of a geminal frustrated Lewis pair featuring electron withdrawing C_6F_5 substituents at both phosphorus and boron. *Chem. Commun.* **2011,** *47*, 4288-4290.

23. Rosorius, C.; Kehr, G.; Fröhlich, R.; Grimme, S.; Erker, G., Electronic Control

of Frustrated Lewis Pair Behavior: Chemistry of a Geminal Alkylidene-Bridged Per-pentafluorophenylated P/B Pair. *Organometallics* **2011,** *30*, 4211-4219.

24. Liedtke, R.; Scheidt, F.; Ren, J.; Schirmer, B.; Cardenas, A. J. P.; Daniliuc, C. G.; Eckert, H.; Warren, T. H.; Grimme, S.; Kehr, G.; Erker, G., Frustrated Lewis Pair Modification by 1,1-Carboboration: Disclosure of a Phosphine Oxide Triggered Nitrogen Monoxide Addition to an Intramolecular P/B Frustrated Lewis Pair. *J. Am. Chem. Soc.* **2014,** *136*, 9014-9027.

25. Stute, A.; Kehr, G.; Daniliuc, C. G.; Fröhlich, R.; Erker, G., Electronic control in frustrated Lewis pair chemistry: adduct formation of intramolecular FLP systems with –$P(C_6F_5)_2$ Lewis base components. *Dalton Trans.* **2013,** *42*, 4487-4499.

26. Mo, Z.; Kolychev, E. L.; Rit, A.; Campos, J.; Niu, H.; Aldridge, S., Facile Reversibility by Design: Tuning Small Molecule Capture and Activation by Single Component Frustrated Lewis Pairs. *J. Am. Chem. Soc.* **2015,** *137*, 12227-12230.

27. Ekkert, O.; Kehr, G.; Fröhlich, R.; Erker, G., P−C Bond Activation Chemistry: Evidence for 1,1-Carboboration Reactions Proceeding with Phosphorus−Carbon Bond Cleavage. *J. Am. Chem. Soc.* **2011,** *133*, 4610-4616.

28. Beckmann, J.; Hupf, E.; Lork, E.; Mebs, S., Peri-Substituted (Ace)Naphthylphosphinoboranes. (Frustrated) Lewis Pairs. *Inorg. Chem.* **2013,** *52*, 11881-11888.

(A)

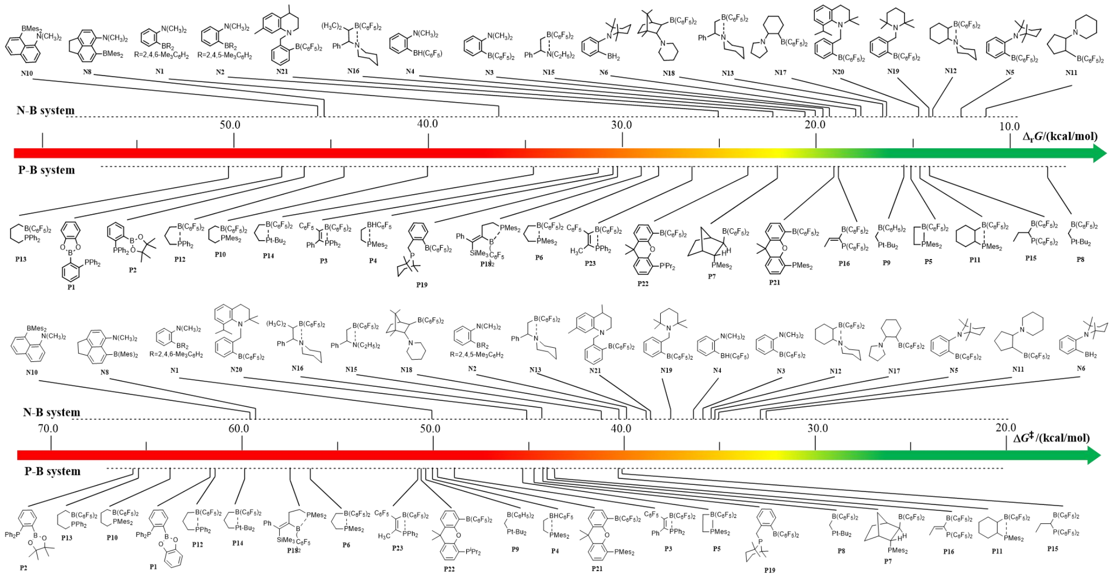
N-B system
50.0
40.0
30.0
20.0
10.0
$\Delta_r G$/(kcal/mol)
P-B system
N-B system
70.0
60.0
50.0
40.0
30.0
20.0
$\Delta G^{\ddagger}$/(kcal/mol)
P-B system

(B)

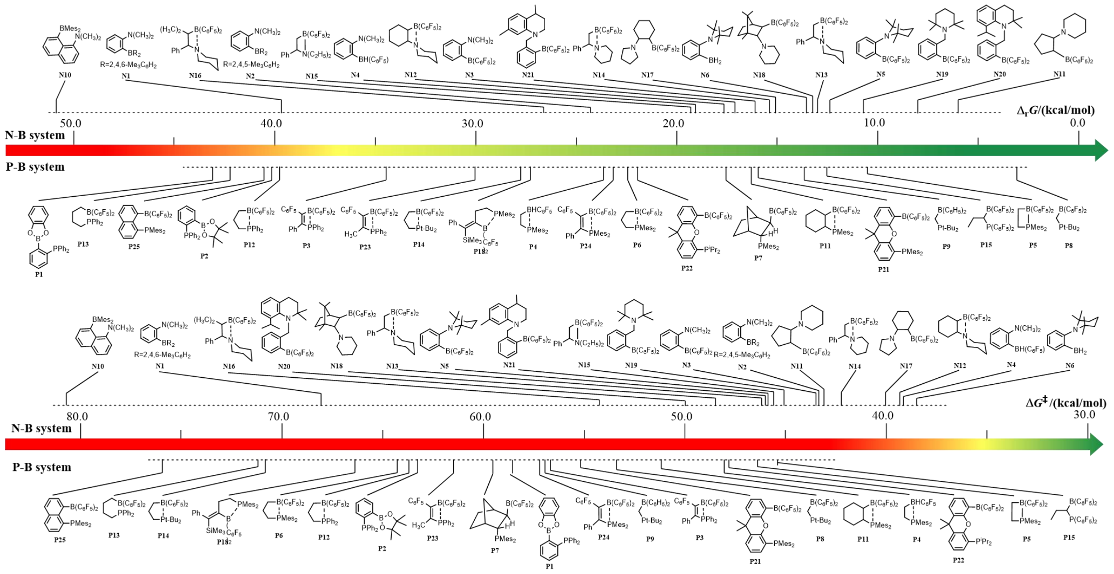
50.0
40.0
30.0
20.0
10.0
0.0
$\Delta_r G$/(kcal/mol)
N-B system
P-B system
$\Delta G^{\ddagger}$/(kcal/mol)
80.0
70.0
60.0
50.0
40.0
30.0
N-B system
P-B system

(C)

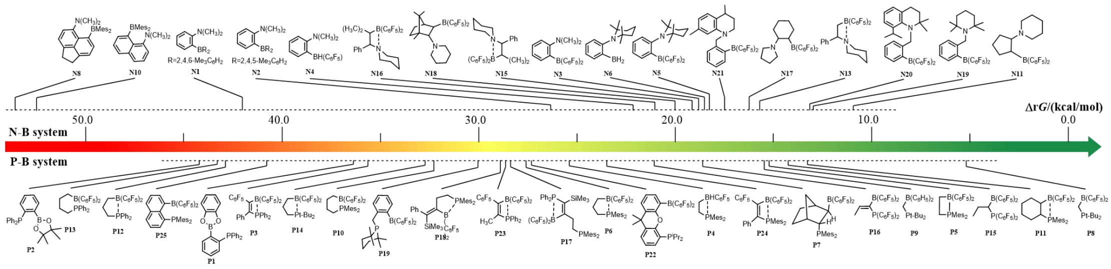
ΔrG/(kcal/mol)
50.0
40.0
30.0
20.0
10.0
0.0
N-B system
P-B system

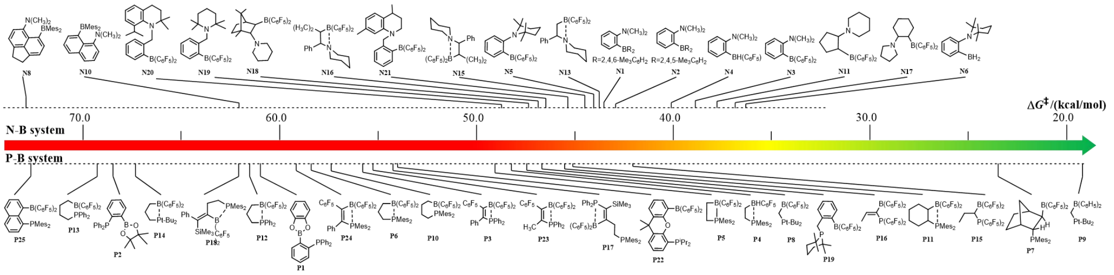

(D)

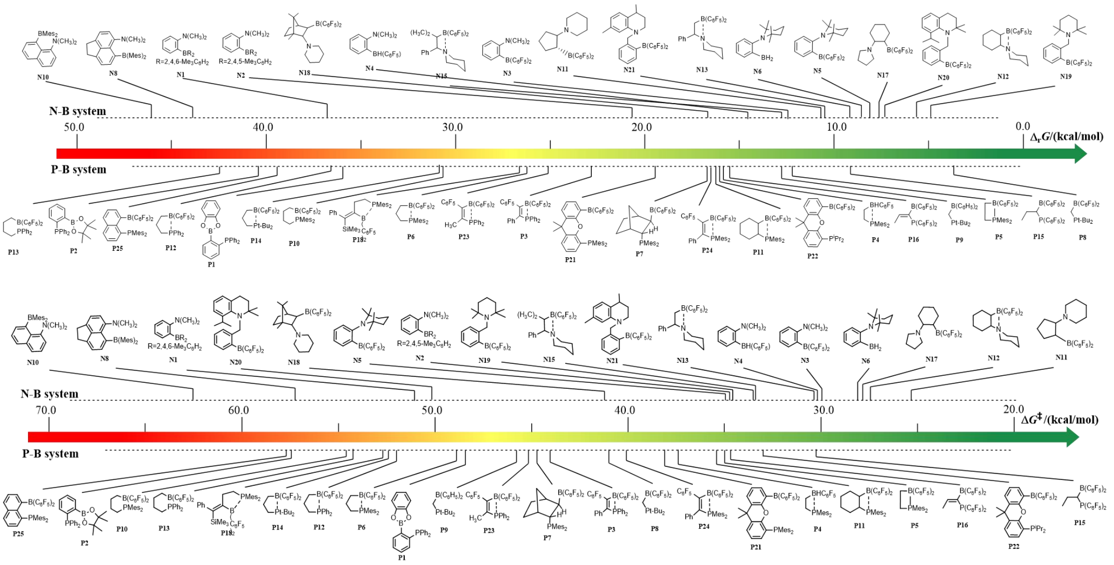

Figure S1. The calculated free energies ($\Delta_r G$) and the free energy barrier ($\Delta G^{\ddagger}$) of the C-H bond activation of (A) methane, (B) methylbenzene, (C) propylene and (D) benzene.

4. Optimized coordinates of collected FLPs.

N1

H	-0.82324700	3.90975700	2.98843400
C	-0.32679000	3.48894000	2.11942200
C	-0.56980900	2.17088800	1.74186000
C	0.54147700	4.27068400	1.36208000
C	0.06489500	1.59824300	0.63138900
H	-1.27117000	1.56966500	2.31761900
C	1.15662900	3.73239800	0.23423000
H	0.73022800	5.30372200	1.63875200
C	0.92314400	2.40593300	-0.13256600
H	1.81470900	4.35182600	-0.36931900
B	-0.19519200	0.08380400	0.26665800
C	-1.68808400	-0.39857400	0.09814800
C	-2.17615000	-1.55060800	0.75852600
C	-2.58092500	0.32747700	-0.72493400
C	-3.51350400	-1.92301600	0.61553400
C	-3.90220300	-0.09359500	-0.86953000
C	-4.39396900	-1.20920200	-0.19486300
H	-3.87488500	-2.80375100	1.14337000
H	-4.56547500	0.46535700	-1.52691400
C	1.02242100	-0.91306600	0.16771700
C	1.91788000	-1.08481200	1.24530100
C	1.25570700	-1.62811000	-1.02386000
C	2.98409300	-1.97564900	1.12950700
C	2.34698600	-2.49506400	-1.11492900
C	3.21879900	-2.68979300	-0.04632100

H	3.65611500	-2.11201000	1.97552900
H	2.51915600	-3.03167300	-2.04609900
C	2.89644900	1.45690100	-1.14512900
H	2.99881500	0.81850400	-0.26542700
H	3.23058300	0.88981900	-2.02032400
H	3.55730000	2.33327300	-1.02632500
C	1.35697500	2.69141800	-2.50330400
H	2.01090300	3.57952700	-2.48760800
H	1.61482900	2.10938600	-3.39412500
H	0.32207500	3.02998800	-2.59760000
N	1.49956500	1.84071700	-1.32788600
C	4.38365700	-3.63990100	-0.14511200
H	5.33529900	-3.11425100	-0.01482800
H	4.33158500	-4.40978500	0.63147700
H	4.40683200	-4.14152400	-1.11545900
C	0.37458900	-1.42508100	-2.23256200
H	0.72259400	-2.02452400	-3.07770700
H	-0.66533300	-1.69719900	-2.02756100
H	0.38742700	-0.37068000	-2.53466100
C	1.76785500	-0.30429100	2.53142600
H	2.13892500	0.72054700	2.41468700
H	0.72644800	-0.22297900	2.85678800
H	2.33380600	-0.77526500	3.33941800
C	-1.29608300	-2.42091800	1.62733300
H	-0.62638400	-3.03874700	1.02039200
H	-0.66749500	-1.83658400	2.30372200
H	-1.90797500	-3.08985300	2.23800700
C	-5.83708200	-1.61929800	-0.32212900
H	-5.94901400	-2.70546600	-0.26729800
H	-6.43709200	-1.18668000	0.48613700
H	-6.26445600	-1.27716300	-1.26818100
C	-2.13459600	1.54094200	-1.50905300
H	-2.08250600	2.43166300	-0.87399400
H	-1.14200400	1.39602700	-1.94624100
H	-2.83577200	1.74797300	-2.32184300

N2

H	1.22576600	4.37777400	-2.57676400
C	0.80191400	3.86039200	-1.72136500
C	0.79430600	2.46897100	-1.68176800
C	0.27756700	4.58797000	-0.65430600
C	0.24069300	1.77928200	-0.59537500
H	1.21969000	1.90800400	-2.51140000
C	-0.24208900	3.92110900	0.45167700
H	0.28724200	5.67355700	-0.67783300
C	-0.25460600	2.52495900	0.48128500
H	-0.63004500	4.48376900	1.29742300
B	0.14783500	0.20414300	-0.52021900
C	1.48262100	-0.61305100	-0.43232400
C	1.67694300	-1.92232600	-0.92712400
C	2.56359600	0.00020600	0.22840100
C	2.90764900	-2.54652800	-0.71311500
C	3.78052000	-0.63171600	0.46448200
C	3.95315800	-1.93972200	-0.01769100
H	3.06058900	-3.54888700	-1.10955900
C	-1.26068500	-0.48991800	-0.46912200
C	-2.36496500	-0.13399900	-1.26785500
C	-1.45051600	-1.47426000	0.51381400
C	-3.58864600	-0.77268900	-1.05275000
C	-2.67695700	-2.09139300	0.74843000
C	-3.77113500	-1.73249300	-0.05730600
H	-4.43698900	-0.50849600	-1.68186400
C	-2.18064100	1.87808600	1.78788400
H	-2.67801600	1.59012400	0.85772100
H	-2.49607400	1.18044900	2.57058000
H	-2.51728900	2.89071800	2.07088800
C	-0.03224100	2.10471100	2.85367500
H	-0.23584500	3.12285700	3.22787600
H	-0.33567100	1.39567500	3.63056600
H	1.04532600	2.00384400	2.69965600
N	-0.73699400	1.78441100	1.62123600
C	-5.11221300	-2.38736900	0.13919000
H	-5.48526500	-2.23939600	1.15874000
H	-5.85340700	-1.98255700	-0.55364800
H	-5.05504900	-3.46971500	-0.02359800
C	-2.26266700	0.91402100	-2.35097400
H	-2.17363500	1.92015200	-1.92679500
H	-1.38565000	0.75799200	-2.98713500
H	-3.14777000	0.89632100	-2.99239600
C	0.62902900	-2.68033000	-1.70904100
H	-0.03696100	-3.24278100	-1.04638800
H	-0.00150200	-2.01820600	-2.30548000
H	1.10787600	-3.39609500	-2.38313300
C	5.25419900	-2.66509300	0.19436700
H	5.20785800	-3.68335900	-0.19794200
H	6.08303500	-2.14816500	-0.30246400
H	5.50851300	-2.72157800	1.25846400
H	2.43987800	1.02273200	0.58106200

H	-0.59957900	-1.76279500	1.13015400
C	-2.82698800	-3.12619900	1.83223000
H	-3.55992700	-2.81492400	2.58535000
H	-3.17258600	-4.08526400	1.43040900
H	-1.87599000	-3.29756100	2.34187400
C	4.88878400	0.07079400	1.20312400
H	5.14754000	-0.45082200	2.13158500
H	5.80444800	0.12263100	0.60345100
H	4.59948700	1.09191500	1.46236600

N3

B	0.01184400	0.70805800	0.27092100
C	0.05421200	2.20245800	-0.34144000
C	0.06873200	3.05461600	-1.44427100
H	0.03969600	2.67150800	-2.45854800
C	0.12678800	4.42887600	-1.20974300
H	0.13905700	5.11380900	-2.05224100
C	0.17160800	4.95658100	0.08749900
H	0.21745000	6.03226400	0.22417100
C	0.15885000	4.12174000	1.20514300
H	0.19334500	4.51767000	2.21559600
C	0.09843500	2.76795700	0.91361400
N	0.06493500	1.58464500	1.78527400
C	-1.12753800	1.60701300	2.66571600
H	-1.10338400	2.51837900	3.26986600
H	-1.12087200	0.73462100	3.31555500
H	-2.02697900	1.60850500	2.04858300
C	1.29323000	1.47835700	2.60687400
H	2.16415900	1.50148300	1.95142500
H	1.27179100	0.54573100	3.16891100
H	1.33740300	2.32523600	3.29747800
C	1.32040000	-0.21766600	0.03810400
C	1.74128500	-1.21198000	0.91565600
F	1.07859100	-1.41396800	2.06675800
C	2.83410800	-2.03363300	0.68250500
F	3.19599500	-2.95813900	1.57240300
C	3.54880800	-1.89089400	-0.49787800
F	4.59755300	-2.67105800	-0.74437600
C	3.15468900	-0.93345100	-1.41818700
F	3.82490800	-0.79597200	-2.56217300
C	2.05928400	-0.12486800	-1.13836100
F	1.72476700	0.75724100	-2.08811200
C	-1.34983600	-0.13849700	0.03052600
C	-1.97686400	-0.99124100	0.93361700

F	-1.49591300	-1.14612000	2.17760400
C	-3.11826300	-1.72309300	0.63216700
F	-3.68491900	-2.50593700	1.55101600
C	-3.66601200	-1.64032400	-0.63803000
F	-4.75866300	-2.33413700	-0.94549700
C	-3.05928400	-0.83159600	-1.58597300
F	-3.56535400	-0.75691300	-2.81689500
C	-1.92554700	-0.10988100	-1.23841300
F	-1.36895900	0.62223800	-2.21373600

N4

B	-0.89179000	0.37601200	-0.97506300
C	-2.12227300	-0.59691900	-0.61141800
C	-2.80329300	-1.80099200	-0.76229800
H	-2.40325800	-2.61364000	-1.36163400
C	-4.03062200	-1.94910400	-0.11004400
H	-4.58078800	-2.88003700	-0.21211300
C	-4.57492700	-0.92902600	0.67904500
H	-5.53091700	-1.08636300	1.16852700
C	-3.90534100	0.28484900	0.85204000
H	-4.31425800	1.08108200	1.46702400
C	-2.69512600	0.37737000	0.18607400
N	-1.68890700	1.44632400	0.11021300
C	-1.04745100	1.71728400	1.41289700
H	-1.77542700	2.15947200	2.09933500
H	-0.21832300	2.41044600	1.26145900
H	-0.67705700	0.78015900	1.82991100
C	-2.17917300	2.69539200	-0.50522900
H	-2.69553100	2.45155800	-1.43373400
H	-1.32193800	3.33524600	-0.72329100
H	-2.86391700	3.20957100	0.17631300
C	0.60972600	0.04268800	-0.51265900
C	1.54920200	1.06155100	-0.40910400
F	1.16773700	2.33040300	-0.65377300
C	2.87062500	0.85743700	-0.04558300
F	3.72683300	1.87618000	0.05141100
C	3.29883500	-0.43643000	0.22051600
F	4.56343000	-0.66218700	0.57082200
C	2.40180100	-1.48878400	0.12279500
F	2.81148700	-2.73283200	0.37518900
C	1.08280900	-1.23378800	-0.23705600
F	0.27043200	-2.29626600	-0.32019800
H	-0.92530200	0.90133300	-2.05888700

N5

B	-0.65587027	-0.04626210	0.51741946
C	-0.01053240	-1.23660925	1.28990584
C	-0.66411204	-1.65300561	2.46014173
H	-1.61051289	-1.18924146	2.73036051
C	-0.12454564	-2.62508093	3.29053379
H	-0.63541229	-2.91328299	4.20315561
C	1.06429852	-3.24259282	2.91778781
H	1.48673918	-4.03140196	3.53261575
C	1.70860524	-2.87081098	1.74161168
H	2.60768499	-3.39526023	1.44569305
C	1.20787323	-1.84662873	0.92551515
N	1.87327607	-1.43387084	-0.28487522
C	3.28270930	-0.94923981	-0.11980230
C	3.70843539	-0.28918954	-1.44157007
H	3.06468083	0.58244858	-1.61933607
H	4.73298295	0.08119674	-1.32784478
C	3.57986799	-1.23416036	-2.62533243
H	4.21047116	-2.12057789	-2.48168308
H	3.92442533	-0.74929582	-3.54410238
C	2.11290610	-1.61479742	-2.75478585
H	1.95224704	-2.30570696	-3.59027590
H	1.54065072	-0.70529137	-2.96805084
C	1.52876679	-2.26534812	-1.48869423
C	1.98753071	-3.73654026	-1.41145583
H	3.06909044	-3.85118529	-1.50095933
H	1.53649989	-4.29404337	-2.23838532
H	1.65859809	-4.20771656	-0.48190926
C	0.00700169	-2.28025577	-1.66453485
H	-0.37742047	-1.25603807	-1.72563472
H	-0.50032440	-2.81059925	-0.85752883
H	-0.23856775	-2.77962368	-2.60678313
C	4.34453466	-2.00397861	0.28051481
H	4.29070780	-2.23280526	1.34716262
H	5.34095639	-1.58955628	0.09564953
H	4.26777925	-2.93837392	-0.27492976
C	3.32155488	0.13750544	0.96047579
H	2.78352100	1.02757115	0.63561739
H	4.36298480	0.42038363	1.14300864
H	2.89682959	-0.20880779	1.90708183
C	-2.17812519	-0.03945109	0.12110816
C	-2.95700675	-1.20697426	0.06372462
F	-2.43646198	-2.40073188	0.34104405
C	-4.29818465	-1.20904127	-0.28979371
F	-4.98728584	-2.34491017	-0.33193459
C	-4.92501602	-0.00949266	-0.59903195
F	-6.20634376	0.00357978	-0.92902345
C	-4.20371589	1.17483661	-0.55701831
F	-4.80135765	2.32582750	-0.84752826
C	-2.86065862	1.14169008	-0.20892216
F	-2.24570716	2.32299764	-0.18550379
C	0.18546447	1.27714506	0.34019610
C	0.49474493	2.02396739	1.46827815
F	0.03422101	1.62757963	2.66239267
C	1.25835473	3.18199137	1.41535814
F	1.52663960	3.87608806	2.51783741
C	1.75316778	3.60592593	0.18849154
F	2.49926578	4.70231670	0.11416971
C	1.45421856	2.88778093	-0.96389568
F	1.93722360	3.28259430	-2.13961011
C	0.65975722	1.75467861	-0.86997008
F	0.36585502	1.10484409	-2.00505558

N6

C	-1.08329500	-1.29709700	-0.08292900
N	-0.39180600	0.00000400	0.14359200
C	-1.08333800	1.29709400	-0.08288700
C	-2.45919400	1.23684000	0.60146900
C	-3.26000500	-0.00004200	0.21880600
C	-2.45915500	-1.23691200	0.60142600
C	1.02330500	0.00001900	-0.06589800
C	1.89274600	-0.00000600	1.05680400
C	3.28083100	0.00000800	0.82738600
C	3.81886600	0.00003500	-0.45394000
C	2.95541000	0.00004200	-1.54466500
C	1.57399600	0.00003200	-1.35198100
B	1.40120600	-0.00005700	2.52226900
C	-1.25323700	1.70134300	-1.56105100
C	-0.28118300	2.41098400	0.60201800
C	-1.25316400	-1.70130200	-1.56110800
C	-0.28109400	-2.41097100	0.60194700
H	0.24888100	-0.00012600	2.82057000
H	3.94678600	-0.00000800	1.68640600
H	4.89372200	0.00004100	-0.60335500
H	3.35422100	0.00005500	-2.55507500
H	0.91553800	0.00003300	-2.21310700
H	-2.29780800	1.22676200	1.68736900
H	-3.00789900	2.15461100	0.36067200

H	-4.22416100	-0.00006600	0.73798700
H	-3.48421600	-0.00002700	-0.85517200
H	-3.00783100	-2.15469300	0.36059600
H	-2.29777100	-1.22686700	1.68732700
H	-0.28331000	-1.79470200	-2.05675400
H	-1.86447200	-0.99771300	-2.12922500
H	-1.74138300	-2.68030700	-1.61883200
H	0.68162900	-2.58617700	0.11298600
H	-0.85276600	-3.34365000	0.56166200
H	-0.09678200	-2.16778100	1.65295800
H	-1.86452000	0.99774800	-2.12918800
H	-0.28339400	1.79480600	-2.05670300
H	-1.74150100	2.68032800	-1.61873600
H	0.68152500	2.58625700	0.11305100
H	-0.09684600	2.16776700	1.65301800
H	-0.85290300	3.34363600	0.56177300
H	2.22294900	-0.00006800	3.39091300

N7

C	-4.62187200	-1.91695000	0.05788100
C	-5.15126600	-0.45394200	0.19416100
C	-3.72320400	1.76986900	0.21152700
C	-2.40241400	2.28825200	0.11654800
C	-1.29063800	1.47982400	-0.01822700
C	-0.40610200	-0.90568400	-0.09266800
C	-0.76191100	-2.24450100	-0.13129900
C	-2.10517400	-2.70513800	-0.10783800
C	-3.91008600	0.41541400	0.13981100
C	-1.45382400	0.06058700	-0.04234300
C	-3.11366800	-1.78056600	-0.02257800
C	-2.77445500	-0.41268700	0.01196100
C	0.28002400	3.24923800	0.52193500
C	0.40307300	2.10394500	-1.58437800
C	3.30571800	-0.64143400	-0.61367100
C	3.17541800	-0.21884600	0.88475800
C	4.24598100	0.21577900	-1.44513900
C	3.64887700	-2.12073700	-0.77859600
C	4.10001400	-0.95201200	1.84258300
C	3.29044800	1.29068100	1.07268800
O	1.95766200	-0.46915500	-1.09615700
O	1.81306000	-0.57834800	1.18244700
B	1.12205500	-0.56508500	-0.00457700
N	0.03813300	1.99230300	-0.17031000
H	-5.02035300	-2.40332700	-0.83791600
H	-4.91905500	-2.53379600	0.91121100
H	-5.84967900	-0.20277400	-0.60979900
H	-5.68925300	-0.31175300	1.13646500
H	-4.55669400	2.45728700	0.32329000
H	-2.27299900	3.36567400	0.14046500
H	0.02709700	-2.99357500	-0.16329400
H	-2.30941000	-3.77148600	-0.13823300
H	1.35030700	3.47213400	0.49741000
H	-0.24300800	4.10365500	0.06162400
H	-0.03487200	3.16092700	1.56374000
H	1.47114200	2.33168100	-1.66618800
H	-0.17249400	2.89857500	-2.08688200
H	0.22517800	1.15493900	-2.09206500
H	4.25670400	-0.14831200	-2.47551700
H	5.26567900	0.16036900	-1.05027300
H	3.92906600	1.26000800	-1.45502200
H	3.50758900	-2.40038600	-1.82530000
H	4.68499200	-2.32654900	-0.49547200
H	2.98916900	-2.74137500	-0.16465700
H	3.93383200	-0.58690200	2.85918200
H	5.14758600	-0.77187900	1.58006500
H	3.91192800	-2.02665100	1.83113500
H	2.95055200	1.54774200	2.07906700
H	4.31986900	1.63741800	0.94481800
H	2.64450200	1.80114400	0.35419800

N8

C	-4.51329200	-2.49779400	-1.70300000
H	-4.68366900	-3.56273200	-1.51811200
H	-4.71231200	-2.32521000	-2.76500700
C	-5.41731600	-1.60839900	-0.79653500
H	-6.06679800	-0.96012200	-1.39282700
H	-6.07075700	-2.21402700	-0.16143900
C	-4.64277200	0.13882800	1.02541100
H	-5.63734900	0.42873900	1.35183000
C	-3.50521300	0.70373900	1.65098400
H	-3.65900700	1.40541800	2.46727900
C	-2.20545100	0.38920400	1.29073700
C	-0.70302200	-0.94017600	-0.30964900
C	-0.70405600	-1.96630800	-1.24754500
H	0.25415500	-2.31068400	-1.63185500
C	-1.87944800	-2.55765100	-1.78534800
H	-1.79529300	-3.33985200	-2.53441700
C	-4.44869800	-0.79079100	0.03428500
C	-1.97343600	-0.52860100	0.22285700

C	-3.09481900	-2.10696800	-1.34446100
C	-3.12713400	-1.11055600	-0.33993100
C	-1.04407000	2.37130500	2.04394900
H	-1.05201000	2.75156700	1.02050500
H	-0.11725900	2.69847500	2.52631400
H	-1.88792200	2.81452500	2.59852900
C	-1.09213000	0.40718800	3.40902200
H	-1.92080800	0.82053200	4.00824500
H	-0.15055800	0.68024100	3.89833400
H	-1.18476600	-0.68117300	3.40763900
C	1.98100200	-1.07401700	-0.04208700
C	3.00982800	-0.97019900	-1.00300100
C	4.14937800	-1.77291200	-0.89511400
H	4.92735200	-1.68801200	-1.65156400
C	4.31682400	-2.67505500	0.15109200
C	3.29756300	-2.77445400	1.09928600
H	3.40824000	-3.47339700	1.92688400
C	2.13984900	-2.00527100	1.00867300
C	2.92686200	-0.02243500	-2.17933100
H	3.14442300	1.00914200	-1.88379900
H	1.93381100	-0.02057400	-2.63831200
H	3.64784600	-0.30977400	-2.94942100
C	5.54149200	-3.54667000	0.24642400
H	5.93856400	-3.56407700	1.26566600
H	6.33245900	-3.19666000	-0.42125600
H	5.30684500	-4.58119800	-0.02687300
C	1.07350000	-2.16474800	2.06508500
H	1.45945000	-2.70320900	2.93472200
H	0.21103900	-2.71742800	1.67729300
H	0.70358500	-1.19227200	2.40165900
C	0.80484700	1.36583700	-0.39754800
C	1.65961100	2.12797700	0.43506400
C	1.84105900	3.48910200	0.20321900
H	2.49124600	4.05848600	0.86597700
C	1.20623200	4.14395800	-0.85309900
C	0.36506800	3.39467300	-1.66929900
H	-0.13867400	3.88480500	-2.50001300
C	0.14923700	2.02799600	-1.45940500
C	2.35425900	1.51153200	1.62743200
H	3.22702000	0.92141400	1.33063900
H	1.67891400	0.84414400	2.17336300
H	2.69440800	2.28898100	2.31761800
C	1.41604900	5.61790900	-1.08107700
H	0.95756000	6.20861100	-0.28076800
H	0.97714100	5.94118000	-2.02811800
H	2.48137800	5.86760500	-1.09762800
C	-0.77586100	1.32056100	-2.42484500
H	-1.00983800	1.97307400	-3.27003600
H	-1.72040800	1.04105500	-1.94769900
H	-0.34040900	0.40034600	-2.82413400
B	0.66823400	-0.19127100	-0.15884900
N	-1.08634700	0.91108300	2.03593800

N9

C	-4.32162000	-1.16712300	-0.18752400
C	-3.07380300	-1.82608100	-0.09011600
C	-1.90510900	-1.10870200	0.00577300
C	-0.73318600	1.08296200	0.01450700
C	-0.83072500	2.45902000	0.02878300
C	-2.07991000	3.11980100	-0.00176200
C	-4.38486800	0.19983500	-0.16638900
C	-1.93539400	0.31894200	-0.01412600
C	-3.23825300	2.39108000	-0.06489800
C	-3.19749200	0.97339200	-0.07744200
C	-0.47457100	-2.95916000	-0.61492100
C	-0.32850500	-1.97565300	1.57876900
C	2.88163700	0.40433800	0.64254500
C	2.76119800	-0.07660600	-0.83913300
C	3.73460800	-0.47784900	1.54042500
C	3.32952000	1.86101600	0.75111200
C	3.76062100	0.54529100	-1.80137700
C	2.78572500	-1.59714400	-0.95296100
O	1.51393900	0.35414700	1.09187500
O	1.43762300	0.36358000	-1.19453100
B	0.71460300	0.47391400	-0.02899600
N	-0.62139700	-1.73451800	0.16317600
H	-5.22971000	-1.75686800	-0.26219900
H	-3.04940600	-2.91069500	-0.07671700
H	0.07559900	3.06028200	0.04980000
H	-2.11538700	4.20462100	0.01024900
H	0.55997500	-3.30323000	-0.55339500
H	-1.11397900	-3.77916900	-0.25206200
H	-0.71477800	-2.75716000	-1.66057700
H	0.70863200	-2.31080900	1.68082200
H	-0.99953700	-2.73748800	2.00657500
H	-0.43445000	-1.04492400	2.13848900
H	3.75858700	-0.05714200	2.54886300
H	4.76203400	-0.53013500	1.16594700

H	3.33141700	-1.49004300	1.60354400
H	3.17711500	2.19964500	1.77858100
H	4.38605200	1.97931900	0.49431400
H	2.73562700	2.49730700	0.08784600
H	3.60081400	0.14023300	-2.80384200
H	4.78593800	0.31156800	-1.49639600
H	3.64357000	1.62890900	-1.84956300
H	2.43785900	-1.88404800	-1.94859000
H	3.79189900	-1.99720200	-0.79852100
H	2.11270600	-2.03569900	-0.21272100
H	-4.20512200	2.88532200	-0.10883700
H	-5.34041700	0.71350400	-0.22521200

N10

B	-0.40739200	-0.15249700	-0.21529400
C	1.97096900	-1.42742300	-0.19888500
C	0.60803400	-1.28168000	-0.63302400
C	2.49039700	-0.78178600	0.96605500
C	2.85512500	-2.26962900	-0.94064400
C	3.82213200	-0.91628400	1.29672100
H	4.20191000	-0.42235000	2.18668400
C	0.17046500	-2.07360100	-1.68022100
H	-0.86823100	-2.00331600	-1.99517500
C	2.04517600	1.32832800	2.05177800
H	2.97785400	1.37641600	2.63603000
H	1.27344200	1.86867800	2.60796300
H	2.19754700	1.83191900	1.09564900
C	2.35737300	-3.00268300	-2.05363900
H	3.04630000	-3.63650200	-2.60554700
C	4.21781700	-2.38227400	-0.56676200
H	4.86872000	-3.02081900	-1.15755600
C	1.46395500	-0.72187600	3.12536100
H	1.18260300	-1.76611700	2.98063100
H	0.68469800	-0.22343200	3.71157200
H	2.39936700	-0.69366900	3.70809700
C	4.69844100	-1.70247200	0.52122500
H	5.74238900	-1.78418200	0.80660500
C	1.03724100	-2.92981600	-2.40047500
H	0.65211800	-3.51117800	-3.23238400
N	1.59712900	-0.04773500	1.83191100
C	-0.05321100	1.38717800	-0.30520900
C	-0.58338100	2.27315700	0.66493800
C	0.73987600	1.93479300	-1.33864600
C	-0.31302200	3.63793600	0.59886600
H	-0.71434500	4.29548400	1.36848500
C	0.97926400	3.31312400	-1.37959900
H	1.58652100	3.71682100	-2.18721500
C	0.46583000	4.18243500	-0.42248600
C	1.34121600	1.10055200	-2.44882000
H	0.60582000	0.44342500	-2.92117300
H	2.14898700	0.45863400	-2.08440600
H	1.75623700	1.74917800	-3.22431600
C	-1.40221900	1.77369500	1.83425500
H	-1.43455000	2.52658100	2.62685400
H	-0.97515200	0.85619600	2.25269700
H	-2.43330600	1.54770600	1.54434800
C	0.72732100	5.66436000	-0.48480100
H	-0.18713500	6.21426500	-0.73136300
H	1.47663900	5.90454000	-1.24285400
H	1.08310400	6.04437000	0.47766200
C	-1.93027400	-0.57673800	-0.04518100
C	-2.90738400	-0.05286800	-0.92538300
C	-2.35028800	-1.50317200	0.93216800
C	-3.69749500	-1.85099700	1.04633500
H	-3.99736700	-2.55304700	1.82217600
C	-4.24261000	-0.43846200	-0.79739300
H	-4.97733200	-0.03269100	-1.49058200
C	-4.66287400	-1.32725700	0.19087700
C	-1.37776900	-2.08938700	1.92266500
H	-1.17810700	-1.38355600	2.73681100
H	-0.42027400	-2.32836700	1.45338200
H	-1.77787500	-3.00192800	2.37262000
C	-2.55970800	0.91382300	-2.03646300
H	-3.37015700	0.95636700	-2.76887900
H	-1.64890500	0.62112700	-2.56776500
H	-2.39613000	1.92655800	-1.65501300
C	-6.10825700	-1.73703300	0.29943500
H	-6.77506800	-0.91470700	0.02635100
H	-6.35598900	-2.05477900	1.31543800
H	-6.33011000	-2.57465500	-0.37101800

N11

B	0.28342300	0.08666700	-0.86084000
N	-0.90263500	2.11483500	0.59485500
C	0.16320500	2.45368100	-0.33789500
H	1.09992000	2.31925600	0.22375200
C	0.22375600	1.48707400	-1.55443800
H	-0.68191300	1.63579800	-2.14717700

C	1.40517800	2.08664400	-2.32383400
H	2.35012500	1.78780100	-1.85539500
H	1.43918300	1.75585800	-3.36420100
C	1.20885300	3.62501700	-2.19198000
H	2.16494200	4.12465500	-2.01672900
H	0.79817200	4.04284100	-3.11456900
C	0.22833200	3.83786400	-1.01145100
H	-0.76626400	4.11102300	-1.38072700
H	0.55477600	4.62262400	-0.32327800
C	-0.75946300	2.74026300	1.90605000
H	-0.99940400	3.82032800	1.86506200
H	0.28413600	2.64354700	2.22072000
C	-1.67952800	2.05076700	2.90996600
H	-1.57132000	2.51792400	3.89425100
H	-1.36005800	1.00477400	2.99857900
C	-3.13244700	2.11196500	2.43633600
H	-3.78620000	1.55728600	3.11694600
H	-3.46954000	3.15761100	2.44869400
C	-3.25063300	1.56979600	1.01151700
H	-3.02741600	0.49453000	1.00339500
H	-4.26828700	1.69860700	0.62817000
C	-2.26215100	2.27625500	0.08610100
H	-2.32767500	1.85361400	-0.92113100
H	-2.53822100	3.34492700	0.01006200
C	-0.97700700	-0.83336200	-0.66632000
F	-2.06498000	-0.05355600	-2.63530000
C	-2.07932700	-0.80205100	-1.52691600
F	-4.25399100	-1.46678500	-2.16634100
C	-3.23842200	-1.53097700	-1.30841100
F	-4.42941800	-3.04826900	0.03568500
C	-3.33005500	-2.34210600	-0.18642800
F	-2.34536200	-3.19179500	1.77380900
C	-2.26343500	-2.41392700	0.69738400
F	-0.14293800	-1.78469000	1.34674400
C	-1.11758300	-1.67420900	0.44287100
C	1.68747300	-0.35204800	-0.26216900
F	2.28856600	-1.06191800	-2.41867700
C	2.62497900	-0.89358200	-1.12963100
F	4.76824000	-1.78492500	-1.59527100
C	3.90109300	-1.26246700	-0.73015200
F	5.48572000	-1.41408000	1.00451900
C	4.26743800	-1.07388900	0.59492300
F	3.72073200	-0.33367500	2.76102800
C	3.36255900	-0.52270900	1.49224900
F	1.25622900	0.37048400	1.94419600
C	2.09405300	-0.17254900	1.05227300

N12

N	0.03104700	1.97033800	0.93738400
B	-0.08146300	0.61372600	-0.18456700
C	-0.90562500	2.61639200	-0.05319000
H	-1.90050500	2.26919100	0.26197500
C	-0.47100400	1.79733300	-1.24858700
H	0.44288800	2.24623200	-1.65268600
C	-0.49872000	2.10234900	2.32470700
H	-1.49700200	1.67038000	2.34426700
H	-0.59388900	3.18211300	2.51120200
C	0.40972800	1.48290400	3.37470000
H	0.49701200	0.40922400	3.19041100
H	-0.06163900	1.61101400	4.35409100
C	1.78917700	2.13471500	3.33447300
H	1.70548600	3.19357800	3.61376000
H	2.46408900	1.66255900	4.05393200
C	2.35301000	2.02226200	1.92100700
H	2.56362000	0.97376100	1.69031500
H	3.29889600	2.56550800	1.82867900
C	1.38940600	2.59981500	0.88989700
H	1.25339900	3.67100500	1.08608000
H	1.78856700	2.48784300	-0.11966900
C	-0.98776200	4.11030400	-0.30125300
H	-0.02377200	4.49669800	-0.65264400
H	-1.28506400	4.68460200	0.58385700
C	-2.04403600	4.25714700	-1.41776400
H	-3.02210400	3.96886000	-1.01037100
H	-2.12506200	5.30826500	-1.71165000
C	-1.73874300	3.39428800	-2.65495500
H	-0.82382100	3.77536300	-3.12837600
H	-2.54397200	3.52018600	-3.38670800
C	-1.53098300	1.90065600	-2.33847600
H	-1.23623700	1.36524500	-3.24453800
H	-2.47577700	1.45554400	-1.99335600
C	-1.29059300	-0.45796700	0.04775800
C	-1.69386400	-1.12292700	-1.11423100
F	-1.08013800	-0.85761500	-2.27805100
C	-2.67815700	-2.09872800	-1.15651000
F	-3.01240300	-2.68055600	-2.30875400
C	-3.30423800	-2.47984300	0.02040700
F	-4.25351400	-3.41225100	0.01008900

C	-2.90850500	-1.89171600	1.20862400
F	-3.47334200	-2.26684500	2.35758700
C	-1.91053800	-0.92300700	1.20169400
F	-1.55804800	-0.47146000	2.41672600
C	1.30184400	-0.22009000	-0.33447900
C	1.66305700	-1.16812400	0.62102700
F	0.91156000	-1.31497000	1.72567000
C	2.77969000	-1.98289500	0.52391300
F	3.07488500	-2.86315700	1.48066900
C	3.59701400	-1.87281400	-0.59228400
F	4.67467600	-2.64290700	-0.71327000
C	3.27903300	-0.95425900	-1.57859400
F	4.05663600	-0.84330100	-2.65615400
C	2.14826200	-0.15622900	-1.43746700
F	1.91303700	0.69577800	-2.44561400

N13

B	0.14184300	-0.07716500	-0.35387900
N	1.52436000	-0.02616100	0.66470500
C	2.19356100	-0.82391000	-0.46256800
H	1.94895300	-1.86362600	-0.21801500
C	1.24227100	-0.32913100	-1.54171200
H	1.02621000	-1.07592600	-2.30406100
H	1.60805800	0.56831200	-2.03751600
C	2.07509200	1.36757000	0.73069100
H	1.91886500	1.84331700	-0.24087500
H	3.15511900	1.27680500	0.89106500
C	1.47872200	2.20419300	1.85930700
H	1.99058700	3.17192200	1.84882300
H	0.41946800	2.40141100	1.67303000
C	1.64229100	1.51187300	3.20968600
H	2.70768300	1.45279200	3.46866900
H	1.14861400	2.08875700	3.99674900
C	1.05820900	0.10447900	3.12163200
H	1.21194200	-0.44928100	4.05324600
H	-0.02046900	0.15408200	2.94580900
C	1.72132100	-0.66962500	1.99189200
H	1.35520600	-1.69292100	1.92716800
H	2.80775000	-0.70501400	2.15736900
C	3.69293100	-0.70550000	-0.58627500
C	4.51374900	-1.61834400	0.08345100
H	4.05759300	-2.41111200	0.67200500
C	5.90042000	-1.53544900	-0.00968800
H	6.52174400	-2.25608100	0.51256800
C	6.48526400	-0.53507900	-0.78102500
H	7.56582500	-0.46915400	-0.86034400
C	5.67829900	0.37520700	-1.45977100
H	6.12883300	1.15176600	-2.06997000
C	4.29283300	0.28869900	-1.36528200
H	3.67465500	0.99847600	-1.90673900
C	-0.76908500	1.26811500	-0.29104300
C	-1.78278700	1.40506600	0.65822200
F	-1.95376200	0.45954500	1.59718800
C	-2.63492800	2.49668700	0.73532900
F	-3.57741400	2.56460500	1.67547800
C	-2.49238400	3.53122700	-0.17813700
F	-3.29623200	4.58947700	-0.12487200
C	-1.49769700	3.45218000	-1.13795000
F	-1.34169000	4.44402700	-2.01553500
C	-0.66603100	2.33817600	-1.17575900
F	0.28307200	2.36961300	-2.12452600
C	-0.84496300	-1.38143300	-0.24002800
C	-1.23938200	-2.14841200	0.85118400
F	-0.79052000	-1.88268500	2.08900100
C	-2.13251500	-3.21107400	0.77459600
F	-2.44599900	-3.90684600	1.86943400
C	-2.71181000	-3.53474100	-0.43941600
F	-3.57463800	-4.54380600	-0.53127100
C	-2.39414200	-2.77579000	-1.55473600
F	-2.96358400	-3.04912100	-2.72931500
C	-1.49105200	-1.72999800	-1.43118900
F	-1.28539900	-1.00986200	-2.54569300

N14

B	0.14184300	-0.07716500	-0.35387900
N	1.52436000	-0.02616100	0.66470500
C	2.19356100	-0.82391000	-0.46256800
H	1.94895200	-1.86362600	-0.21801500
C	1.24227000	-0.32913100	-1.54171200
H	1.02621000	-1.07592600	-2.30406100
H	1.60805800	0.56831200	-2.03751600
C	2.07509200	1.36757000	0.73069100
H	1.91886500	1.84331700	-0.24087500
H	3.15511900	1.27680500	0.89106500
C	1.47872200	2.20419300	1.85930700
H	1.99058800	3.17192200	1.84882300
H	0.41946800	2.40141100	1.67303000
C	1.64229100	1.51187300	3.20968500

H	2.70768300	1.45279200	3.46866900
H	1.14861400	2.08875700	3.99674900
C	1.05820900	0.10447900	3.12163200
H	1.21194100	-0.44928100	4.05324600
H	-0.02047000	0.15408200	2.94580900
C	1.72132100	-0.66962600	1.99189200
H	1.35520500	-1.69292100	1.92716800
H	2.80775000	-0.70501500	2.15736900
C	3.69293100	-0.70550000	-0.58627500
C	4.51374800	-1.61834500	0.08345100
H	4.05759200	-2.41111200	0.67200500
C	5.90042000	-1.53545000	-0.00968800
H	6.52174300	-2.25608200	0.51256800
C	6.48526400	-0.53508000	-0.78102500
H	7.56582500	-0.46915500	-0.86034400
C	5.67829900	0.37520600	-1.45977100
H	6.12883300	1.15176600	-2.06997000
C	4.29283300	0.28869800	-1.36528200
H	3.67465500	0.99847600	-1.90673900
C	-0.76908500	1.26811500	-0.29104300
C	-1.78278700	1.40506700	0.65822200
F	-1.95376200	0.45954600	1.59718800
C	-2.63492800	2.49668800	0.73532900
F	-3.57741400	2.56460600	1.67547800
C	-2.49238400	3.53122700	-0.17813700
F	-3.29623100	4.58947800	-0.12487200
C	-1.49769600	3.45218000	-1.13795000
F	-1.34168900	4.44402700	-2.01553500
C	-0.66603000	2.33817600	-1.17575900
F	0.28307200	2.36961200	-2.12452600
C	-0.84496400	-1.38143300	-0.24002800
C	-1.23938200	-2.14841200	0.85118500
F	-0.79052000	-1.88268500	2.08900100
C	-2.13251500	-3.21107400	0.77459600
F	-2.44599900	-3.90684600	1.86943400
C	-2.71181000	-3.53474100	-0.43941600
F	-3.57463900	-4.54380500	-0.53127100
C	-2.39414300	-2.77579000	-1.55473600
F	-2.96358400	-3.04912100	-2.72931500
C	-1.49105200	-1.72999800	-1.43118900
F	-1.28539900	-1.00986200	-2.54569300

N15

N	-1.40166500	-0.11970600	0.77875800
B	-0.07641400	-0.04252900	-0.29999300
C	-2.23435200	0.28938300	-0.45620400
H	-2.20719700	1.38197400	-0.44153300
C	-1.20944800	-0.21467700	-1.45987100
H	-1.15924600	0.35084800	-2.38663200
H	-1.36576100	-1.26893700	-1.70841600
C	-1.69310300	-1.52093900	1.22750000
H	-0.80679500	-1.87503400	1.74910600
H	-1.79897900	-2.12019700	0.32000000
C	-2.90291400	-1.71281500	2.13744500
H	-2.75154500	-1.26102600	3.12101800
H	-3.02746400	-2.78815700	2.29401700
H	-3.83340400	-1.32872500	1.71945100
C	-1.52992500	0.85962800	1.89028700
H	-2.57671200	0.85234200	2.21518900
H	-1.32775200	1.84395400	1.46016400
C	-0.59256200	0.60681600	3.05740500
H	-0.76936500	-0.35934300	3.53601300
H	-0.75385600	1.38545000	3.80765800
H	0.45210600	0.64028500	2.74784500
C	-3.67859500	-0.13959700	-0.48239500
C	-4.64800800	0.69542100	0.08307700
H	-4.34770800	1.65845800	0.48970400
C	-5.98324800	0.30720600	0.13090300
H	-6.72317400	0.96508400	0.57583000
C	-6.36743300	-0.92197800	-0.40052100
H	-7.40801100	-1.22892600	-0.36602300
C	-5.41522100	-1.74725600	-0.99389600
H	-5.71400300	-2.69724600	-1.42592200
C	-4.07992300	-1.35594600	-1.03915700
H	-3.34743500	-2.00345100	-1.51146700
C	0.64519500	1.42846900	-0.20518000
C	0.26876900	2.54700700	-0.95428500
F	-0.78564700	2.51450500	-1.78677700
C	0.91673700	3.77553000	-0.90346400
F	0.48636400	4.79450700	-1.64787700
C	2.01240400	3.94039700	-0.07395300
F	2.64306800	5.10884700	-0.01101600
C	2.43365500	2.86840900	0.69471300
F	3.48079900	3.00260000	1.50828800
C	1.75495600	1.66017800	0.61132400
F	2.23653800	0.69044300	1.40538200
C	1.01753300	-1.25134700	-0.25294200
C	1.57369200	-1.89959200	0.84559900

F	1.21761100	-1.56116900	2.09835500
C	2.50979300	-2.92059100	0.75170000
F	2.98673300	-3.50842800	1.85047000
C	2.96098500	-3.32135900	-0.49566600
F	3.86531200	-4.29158400	-0.60619200
C	2.46243900	-2.69303500	-1.62477400
F	2.89607800	-3.05277600	-2.83388600
C	1.51949000	-1.68374200	-1.48114400
F	1.10809300	-1.10474100	-2.61967500

N16

N	-1.08164000	-1.38263900	-0.35948500
B	0.00943600	-0.31761000	0.41724700
C	-1.98186800	-1.25067300	0.87936600
H	-2.18826400	-2.26080900	1.24507300
C	-0.92378100	-0.54089800	1.78142300
C	-1.46739100	0.68341500	2.52785000
H	-2.36042600	0.41649200	3.10653300
H	-1.74167800	1.51448500	1.87517100
H	-0.71777600	1.05082100	3.23088900
C	-0.47633800	-1.53262500	2.86863900
H	0.16522300	-1.04667900	3.60411900
H	0.07172400	-2.39837500	2.48436100
H	-1.35941400	-1.91030700	3.40178100
C	-1.73148400	-1.13604800	-1.68646400
H	-0.92301500	-1.11221100	-2.41837400
H	-2.18904100	-0.15374500	-1.67049900
C	-2.76139300	-2.19382200	-2.07294300
H	-3.60631600	-2.16849100	-1.37605800
H	-3.15435100	-1.91722600	-3.05645200
C	-2.14951000	-3.58985100	-2.09947100
H	-1.39122500	-3.64531800	-2.89125100
H	-2.90842100	-4.34529900	-2.32226900
C	-1.49259400	-3.86224000	-0.75089800
H	-0.94969600	-4.81263000	-0.75528600
H	-2.26114000	-3.94469600	0.02689500
C	-0.48994500	-2.76678400	-0.40876400
H	-0.00221900	-2.95892900	0.54651800
H	0.28275300	-2.73628800	-1.18149600
C	-3.32342100	-0.58236500	0.68990000
C	-3.48644500	0.71234400	0.17978900
H	-2.61775800	1.28301400	-0.13790300
C	-4.74960400	1.28471700	0.08088200
H	-4.85347000	2.28664300	-0.32323900
C	-5.87561700	0.58126700	0.50427700
H	-6.86000300	1.03200100	0.42845600
C	-5.72859500	-0.69921600	1.02682300
H	-6.59749300	-1.25614100	1.36298700
C	-4.46241800	-1.27288900	1.11456300
H	-4.35468800	-2.27630200	1.51964100
C	1.56011100	-0.83988600	0.27570600
C	2.49196000	-1.00345800	1.29570100
F	2.16598800	-0.82693200	2.58041900
C	3.82450900	-1.33941600	1.06903500
F	4.66214100	-1.49121100	2.09576900
C	4.28509900	-1.51129000	-0.22448400
F	5.55420800	-1.84065400	-0.45207400
C	3.40460000	-1.32939300	-1.28129000
F	3.82518400	-1.47766400	-2.53837700
C	2.09179500	-0.98696800	-1.00448100
F	1.28937200	-0.78995100	-2.07015100
C	0.11684800	1.24974700	-0.08306600
C	0.74819500	2.08003300	0.85210900
F	1.17345600	1.55958000	2.01456400
C	1.01930700	3.42461700	0.66190900
F	1.61191400	4.14341800	1.61551500
C	0.68787900	4.01727900	-0.54730400
F	0.93581100	5.30730000	-0.75894100
C	0.11193200	3.23662400	-1.53115700
F	-0.18866800	3.77261900	-2.71542200
C	-0.13851400	1.88656100	-1.29397900
F	-0.64928600	1.23391900	-2.35035400

N17

B	-0.10628300	0.66971600	0.00328900
N	-0.18949000	1.82343400	1.31671100
C	-1.03252900	2.60944200	0.35353600
H	-2.04554000	2.20378500	0.49106600
C	-0.43178200	2.00037500	-0.89359000
H	0.52286400	2.51622100	-1.07248600
C	-0.79967700	1.71154400	2.67016400
H	-1.08875500	2.73088200	2.95630200
H	-1.69196300	1.09338300	2.63787000
C	0.31841300	1.19777500	3.57287700
H	0.30087100	0.10932900	3.61731100
H	0.20614100	1.58399700	4.58743100
C	1.61503100	1.69573400	2.88938500
H	2.26459400	0.85644600	2.63456100

H 2.19017000 2.37244600 3.52412900
C 1.14101400 2.42999900 1.62922500
H 1.81815100 2.34290100 0.77939700
H 0.97810100 3.49086400 1.84500900
C -1.11738800 4.12310700 0.34119400
H -0.12261900 4.56091400 0.19473400
H -1.54659500 4.54550200 1.25719300
C -2.00898200 4.45451100 -0.87481500
H -3.02721700 4.09919900 -0.66720100
H -2.07542900 5.54016800 -0.99578000
C -1.50982100 3.81537900 -2.18293100
H -0.54370500 4.26688100 -2.44653800
H -2.20369600 4.06799300 -2.99209800
C -1.32198800 2.28873900 -2.09322000
H -0.88725100 1.90775500 -3.02114000
H -2.29878600 1.79987300 -1.96968800
C -1.23610900 -0.50409500 0.00596900
C -1.60602700 -0.98370400 -1.25347200
F -1.04261700 -0.46391500 -2.35425800
C -2.49921300 -2.02389700 -1.46213000
F -2.80883500 -2.42039900 -2.69698300
C -3.05743800 -2.66654500 -0.36776600
F -3.92033600 -3.66499700 -0.53828600
C -2.68412000 -2.26965900 0.90434400
F -3.18138400 -2.89908600 1.97024800
C -1.77877100 -1.22615000 1.06395800
F -1.43729800 -0.96888900 2.33829300
C 1.34279600 -0.03030300 -0.18270100
C 2.10958700 0.12310100 -1.33464000
F 1.70558700 0.91712300 -2.33596900
C 3.31658600 -0.53738300 -1.53697700
F 4.01451700 -0.34831400 -2.65735100
C 3.79436800 -1.40336000 -0.56637700
F 4.94918000 -2.04005100 -0.74423000
C 3.05335500 -1.60957800 0.58848400
F 3.49610200 -2.44991500 1.52475800
C 1.85199400 -0.93481400 0.74342900
F 1.15316400 -1.19072100 1.86298300

N18

C 1.98081900 -2.48325900 -1.22314200
C 1.75985500 -1.17256300 -0.37439900
H 1.85138300 -0.32525000 -1.07076000
C 0.26586700 -1.29684200 0.05923600
H 0.19569700 -1.54852300 1.12700100
C -0.22241900 -2.55157600 -0.70715800
H -1.16733800 -2.94860800 -0.32638100
C -0.23193400 -2.20911300 -2.21250900
H -0.71344000 -1.24372300 -2.40568600
H -0.77943400 -2.95730400 -2.79180500
C 1.27960700 -2.19733000 -2.57111300
H 1.60432300 -1.24188100 -2.99378600
H 1.52360900 -2.97389800 -3.30262800
C 0.99158400 -3.49967100 -0.57094000
C 3.41511400 -2.95568100 -1.36268000
H 3.91285000 -2.99163800 -0.38867600
H 3.99608100 -2.30274100 -2.02291600
H 3.44192700 -3.95789900 -1.80449700
C 1.34776100 -3.87677400 0.87099000
H 1.73369700 -3.04090900 1.45546000
H 2.12725400 -4.64649100 0.87215700
H 0.47452900 -4.29498500 1.38598000
C 0.80859600 -4.80866200 -1.34603100
H 1.73500600 -5.39219600 -1.34768000
H 0.50176800 -4.67108300 -2.38461000
H 0.04391900 -5.42146100 -0.85503300
N 2.75590000 -0.96385700 0.69631300
C 3.85237300 -0.13944600 0.18209600
H 3.49544200 0.89898700 0.01193500
H 4.15871600 -0.53001000 -0.79094800
C 5.04280100 -0.11547800 1.13314100
H 5.83665900 0.50648600 0.70649800
H 5.43619800 -1.13487800 1.22933300
C 4.61473800 0.40366100 2.50301600
H 4.32832300 1.46082900 2.41486600
H 5.44065400 0.35261200 3.21960900
C 3.41548500 -0.40061700 2.99686300
H 3.71918200 -1.43929800 3.17783000
H 3.03465300 0.00262800 3.94109400
C 2.28831800 -0.40660200 1.96525600
H 1.47382500 -1.01390300 2.36520100
H 1.90261400 0.62546000 1.84548800
B -0.64044200 -0.02645100 -0.03787700
C -0.00909300 1.41195100 -0.23196300
F -0.44214600 1.92692100 2.02329500
C 0.05250700 2.29865500 0.83298700
F 0.71518600 4.35726600 1.79120200
C 0.65587800 3.54457100 0.73874400

F	1.77860100	5.13036700	-0.58805700
C	1.19928200	3.93996800	-0.47546700
F	1.66395200	3.46601900	-2.73552700
C	1.14494800	3.08687800	-1.57045500
F	0.50154400	1.04244100	-2.50255600
C	0.54270200	1.84589100	-1.42874300
C	-2.19837900	-0.11938600	0.15259600
F	-2.06692700	-1.95981700	1.64901400
C	-2.80588800	-1.07632700	0.96989500
F	-4.70973500	-2.07373100	1.95119100
C	-4.17767200	-1.15374200	1.15105700
F	-6.31580500	-0.32721900	0.63673600
C	-5.00137200	-0.25798300	0.48368400
F	-5.23790100	1.55893100	-0.99238800
C	-4.44750200	0.70683000	-0.34570700
F	-2.59392100	1.70270500	-1.31367600
C	-3.06944300	0.76497600	-0.49007800

N19

B	0.73300500	-0.00297600	0.70732200
C	-0.07602500	-0.91786600	1.67632000
C	0.70888100	-1.57476700	2.64070700
H	1.79234300	-1.53615200	2.55844000
C	0.14936500	-2.23706100	3.72927400
H	0.78855200	-2.71867600	4.46223900
C	-1.23160900	-2.26018400	3.86820500
H	-1.69231100	-2.75638700	4.71690800
C	-2.03040900	-1.65489900	2.90031400
H	-3.11153300	-1.69950700	3.00212300
C	-1.48421400	-1.00745900	1.79275200
C	-2.45303400	-0.40978700	0.78618000
H	-2.23413800	0.65713700	0.71662200
H	-3.45480200	-0.47459100	1.23575800
N	-2.43488500	-0.94177600	-0.58980000
C	-2.83672800	-2.39099900	-0.68105700
C	-2.97474700	-2.82109900	-2.15440400
H	-1.97221700	-2.83230600	-2.60282300
H	-3.33906600	-3.85379900	-2.16179300
C	-3.85858700	-1.91180300	-2.98414900
H	-4.88341200	-1.88929700	-2.59420500
H	-3.91938400	-2.27255800	-4.01606900
C	-3.22824700	-0.53473800	-2.93750200
H	-3.78642200	0.18731400	-3.54314200
H	-2.21853400	-0.60077100	-3.36380300
C	-3.11798200	0.03561800	-1.51372900
C	-2.24009900	1.28636300	-1.65933200
H	-1.22744500	0.99207300	-1.94519700
H	-2.19346500	1.90161400	-0.75590100
H	-2.64684700	1.92218700	-2.45267600
C	-4.51589800	0.48767900	-1.04562900
H	-4.48409700	0.96939300	-0.06458900
H	-5.23262300	-0.33451500	-0.99486300
H	-4.91200100	1.22458700	-1.75180300
C	-4.13735100	-2.74651500	0.06281500
H	-4.08134300	-2.50490200	1.12721100
H	-4.30273700	-3.82662800	-0.00675100
H	-5.01886100	-2.25256800	-0.35222000
C	-1.71226000	-3.28461100	-0.13011700
H	-0.75318600	-2.95836900	-0.53303900
H	-1.89293700	-4.31425900	-0.45812900
H	-1.64827200	-3.29999200	0.95746400
C	2.12336600	-0.50437300	0.14228000
C	2.36054800	-1.84765500	-0.16757900
F	1.40062900	-2.76263200	-0.02756300
C	3.57590400	-2.31840100	-0.64491300
F	3.73600700	-3.60698300	-0.93717700
C	4.63100500	-1.43479100	-0.80806900
F	5.80261800	-1.86729300	-1.25285800
C	4.45527800	-0.09355000	-0.49956400
F	5.46895600	0.75740400	-0.64022000
C	3.22303600	0.34235900	-0.03930400
F	3.13852600	1.64420700	0.25007700
C	0.32815500	1.50512400	0.47732800
C	-0.34730800	2.25255700	1.44522600
F	-0.69870400	1.70858700	2.61167700
C	-0.69277200	3.58603500	1.26773900
F	-1.32851200	4.25712000	2.22593600
C	-0.36108300	4.22373200	0.08200800
F	-0.68651400	5.49570900	-0.10225600
C	0.31324600	3.52460700	-0.91060500
F	0.62027500	4.12777000	-2.05676200
C	0.65044500	2.20075500	-0.69016900
F	1.26657000	1.56973900	-1.69506700

N20

B	1.09144200	0.08433300	0.80837400
C	-0.04148700	0.17664500	1.88322200
C	0.14408300	1.08301200	2.93936500
H	1.03861000	1.69669200	2.97363700

C	-0.77518100	1.18928600	3.98034700
H	-0.58805200	1.87617700	4.79949700
C	-1.94068200	0.43412000	3.94465700
H	-2.68035000	0.52701800	4.73380900
C	-2.16682600	-0.43441100	2.87756400
H	-3.08998200	-1.00665900	2.83230800
C	-1.22663700	-0.59291400	1.86204000
C	-1.51655000	-1.57264200	0.74641700
H	-2.31372700	-2.25116300	1.08914500
H	-0.63123600	-2.18572900	0.57165700
N	-1.87921600	-0.91334800	-0.52575600
C	-1.82519500	-1.83275600	-1.70117300
C	-2.74417600	-3.04082400	-1.45427700
H	-2.79166700	-3.65303800	-2.36098700
H	-2.28286900	-3.67135700	-0.68608300
C	-4.16448500	-2.62957300	-1.01777800
C	-4.31516100	-1.19574600	-0.53991500
C	-5.59697400	-0.68228500	-0.34207600
C	-5.77905900	0.65229400	-0.00170100
H	-6.77905100	1.04355600	0.15900100
C	-4.67693600	1.49444900	0.09150100
H	-4.82682800	2.54844400	0.30962800
C	-3.37904600	1.01820400	-0.11275500
C	-3.20548000	-0.34984400	-0.38601200
C	-2.21295100	1.98800400	-0.13942700
H	-1.30481700	1.38812200	-0.10912400
C	-2.19613600	2.94608700	1.05141800
H	-1.25613100	3.50963100	1.06724300
H	-2.28508500	2.40455000	1.99692200
H	-3.01135300	3.67641300	0.99717400
C	-2.21945800	2.76152900	-1.46340400
H	-1.35902500	3.43674800	-1.52904100
H	-3.12817600	3.36805100	-1.55159900
H	-2.18372700	2.08253700	-2.31987300
C	-0.38936000	-2.29760100	-1.94900300
H	0.01681400	-2.90663200	-1.13533600
H	0.25874100	-1.42990600	-2.10385000
H	-0.35883200	-2.91128600	-2.85459700
C	-2.25636700	-1.04719500	-2.94545900
H	-1.62706700	-0.15784700	-3.05081000
H	-3.29971600	-0.72602000	-2.90825300
H	-2.12638000	-1.66968400	-3.83587200
C	1.59887400	1.44998900	0.16809300
C	1.12638100	1.90325200	-1.05727100
F	0.30195600	1.12658200	-1.77176500
C	1.47739800	3.13478500	-1.58745900
F	0.97143200	3.54544800	-2.74880400
C	2.36313000	3.94657600	-0.89098100
F	2.71119700	5.13000600	-1.38525300
C	2.88932900	3.51292400	0.31659300
F	3.75672500	4.27526400	0.97890600
C	2.50275400	2.27725200	0.81700100
F	3.04010100	1.88703700	1.98538200
C	1.82817800	-1.24446600	0.42248000
C	1.83376700	-2.36140400	1.26829200
F	1.17329100	-2.34130000	2.42472300
C	2.50101900	-3.53823100	0.96420400
F	2.48292000	-4.56924200	1.80433900
C	3.19808300	-3.63482500	-0.23256800
F	3.83222600	-4.75567200	-0.53996600
C	3.22010500	-2.55893900	-1.10849600
F	3.87106800	-2.65655700	-2.26405100
C	2.55454300	-1.39287400	-0.76490000
F	2.57992700	-0.41761300	-1.67399200
H	-6.45579100	-1.33756200	-0.47022800
H	-4.85938500	-2.75470200	-1.85565300
H	-4.52220800	-3.31142300	-0.23718700

N21

B	0.22434800	0.85869700	0.82856000
C	-0.33344900	0.15957700	2.11260100
C	-0.98232100	0.95757900	3.06458700
H	-1.11492200	2.01928300	2.86831400
C	-1.45668400	0.42841000	4.26263500
H	-1.94735500	1.07210900	4.98575600
C	-1.31558700	-0.93132300	4.51134400
H	-1.70021200	-1.36283100	5.43032500
C	-0.69947900	-1.74782300	3.56410400
H	-0.61832700	-2.81688000	3.74379000
C	-0.19935200	-1.21706100	2.37859000
C	0.47787700	-2.14378400	1.38787700
H	0.23098700	-3.18367100	1.64395800
H	1.56206200	-2.03498400	1.51339600
N	0.17376000	-1.85120600	-0.02365600
C	1.30316200	-2.08778300	-0.93129900
C	1.38447500	-3.53170300	-1.39957400
H	1.41140600	-4.20183700	-0.52904400
H	2.30442100	-3.69970400	-1.96916800

C	0.16506000	-3.84275300	-2.26312500
H	0.28884900	-3.29568200	-3.20970100
C	-1.09496400	-3.32729400	-1.58007500
C	-2.35116500	-3.76659000	-2.01171400
H	-2.40637900	-4.44549800	-2.85730400
C	-3.52943500	-3.37506500	-1.39187000
H	-4.48520600	-3.73462400	-1.76311000
C	-3.48472200	-2.52400300	-0.28699400
C	-2.24251400	-2.07695500	0.15085600
H	-2.20157800	-1.40386300	0.99664300
C	-1.05153000	-2.42769800	-0.49443200
C	0.10015300	-5.33699100	-2.58588200
H	1.06246700	-5.67489300	-2.98330600
H	-0.66079800	-5.57291200	-3.33321300
H	-0.11916400	-5.91789600	-1.68423100
C	-4.73771800	-2.03446600	0.39067500
H	-4.63044500	-2.04514100	1.47911300
H	-5.60246200	-2.64731500	0.12438900
H	-4.95474000	-1.00141000	0.09715500
C	1.74301100	0.73011200	0.41803800
C	2.73880700	0.38406300	1.33284400
F	2.45121100	0.19539500	2.62302500
C	4.06977900	0.20897700	0.97481200
F	4.98135800	-0.11692800	1.88873400
C	4.44853300	0.38474700	-0.34725400
F	5.71511100	0.22068500	-0.70615600
C	3.49514000	0.73158400	-1.29485100
F	3.85017000	0.88185000	-2.56922600
C	2.17836600	0.89465300	-0.89939100
F	1.29925600	1.16885100	-1.86879800
C	-0.68721300	1.92566800	0.09551600
C	-0.17554900	3.09561300	-0.47797600
F	1.13262900	3.36467500	-0.42790100
C	-0.96317200	4.04959400	-1.10220500
F	-0.41755600	5.14553200	-1.62578800
C	-2.33540100	3.85790000	-1.17436500
F	-3.10315900	4.75726700	-1.77439700
C	-2.89722800	2.72381800	-0.61010300
F	-4.21386200	2.53315200	-0.66835000
C	-2.07839700	1.79789500	0.02117500
F	-2.70452500	0.74658200	0.55158400
H	2.21992400	-1.78308000	-0.41996800
H	1.17680100	-1.43114700	-1.80279800

P1

B	0.07361600	0.79876900	0.67292800
C	-0.73328000	-0.71546400	2.68418300
C	-0.23402300	0.55804000	2.26183500
C	-0.98637900	-1.72049100	1.71284800
C	-1.05141600	-0.98675800	4.04286100
C	-1.61510300	-2.89716700	2.05171600
H	-1.88109600	-3.62078900	1.28563800
C	-0.05039900	1.50556100	3.24690100
H	0.32232800	2.49038200	2.99620100
C	-0.81698100	0.02441300	5.01111800
H	-1.04669300	-0.17670200	6.05373800
C	-1.63087400	-2.23725300	4.37478400
H	-1.87061300	-2.43941000	5.41537200
C	-1.92229800	-3.16516900	3.40273800
H	-2.39859700	-4.10348500	3.66702600
C	-0.32590900	1.23817500	4.61343000
H	-0.15227400	2.02062700	5.34587400
C	-0.66250600	2.13045600	0.07012400
C	-1.70020700	2.19791900	-0.85186300
C	-0.19813700	3.38517300	0.47351500
C	-2.29287000	3.38313500	-1.27768300
C	-0.75156200	4.59159900	0.07955200
C	-1.82389400	4.59253100	-0.80056100
C	1.67077800	0.78027100	0.31617500
C	2.30171600	1.58385900	-0.63644300
C	2.53182800	-0.12467200	0.93890700
C	3.87293800	-0.27851900	0.62252700
C	3.64821900	1.48513600	-0.96662700
C	4.44195800	0.54309500	-0.33620400
P	-0.64668900	-1.15121600	0.02286200
C	0.81890600	-1.91065600	-0.78185400
C	1.63166400	-2.91938500	-0.22565000
C	1.23483400	-1.23974300	-1.95573400
C	2.88059600	-3.16058100	-0.80521200
C	2.48429500	-1.52708900	-2.49911800
C	3.34288600	-2.45504300	-1.91214300
H	3.51947900	-3.91821600	-0.35837900
H	2.80282700	-0.99069700	-3.39017000
C	-2.21625400	-1.65796400	-0.82477400
C	-2.30036000	-2.65388300	-1.82654000
C	-3.40654500	-1.06187700	-0.34007800
C	-3.55781600	-2.95767400	-2.36098800
C	-4.62931100	-1.41174700	-0.90627300

C	-4.72913000	-2.34520400	-1.93349200
H	-3.61498100	-3.71730500	-3.13653600
H	-5.53081500	-0.93584400	-0.52650500
C	1.24597600	-3.77957300	0.95106200
H	1.17819500	-3.20712500	1.87768100
H	0.28181800	-4.26972500	0.79271000
H	1.99426700	-4.56223600	1.09176700
C	4.74376600	-2.64577500	-2.42473300
H	5.41601400	-1.92502200	-1.94485400
H	5.12001400	-3.64711900	-2.20253100
H	4.80263100	-2.48258500	-3.50366200
C	0.36960500	-0.21502000	-2.64877500
H	-0.64644800	-0.58194000	-2.82523200
H	0.28110800	0.71067500	-2.07218900
H	0.80347800	0.04870500	-3.61559700
C	-1.15045000	-3.48180200	-2.35625600
H	-0.45386900	-2.90362600	-2.96820600
H	-0.56836900	-3.94294400	-1.55578700
H	-1.54884600	-4.28436100	-2.98008300
C	-6.05869300	-2.67327200	-2.55653100
H	-6.84492800	-2.73787500	-1.79961500
H	-6.35337300	-1.89459200	-3.26789800
H	-6.02174500	-3.62164500	-3.09735100
C	-3.46167700	-0.08268000	0.80819200
H	-3.66346700	-0.61136400	1.74676600
H	-2.54784900	0.49131400	0.95682400
H	-4.27278200	0.63148100	0.64581000
F	0.90300700	3.47774900	1.24072500
F	-0.24671800	5.74655300	0.51471200
F	-2.37417600	5.73817100	-1.19479200
F	-3.29995000	3.35450100	-2.15213600
F	-2.18819400	1.08559900	-1.42249600
F	2.05281200	-0.98354700	1.85116200
F	4.60635700	-1.22614600	1.20773000
F	5.72403400	0.40119900	-0.67078500
F	4.17326300	2.27813500	-1.90246400
F	1.62106500	2.49294100	-1.35476600

P2

C	-1.97421000	2.32858800	-0.42922100
C	-0.91819700	1.50331400	-0.03416300
C	0.40753700	1.87942000	-0.34977600
C	0.62032500	3.05548200	-1.07759200
C	-0.43630900	3.87725500	-1.46085400
C	-1.73500100	3.51123300	-1.12825400
H	-2.99786700	2.05001100	-0.19936200
H	1.63884300	3.32811400	-1.34191800
H	-0.24653000	4.79094700	-2.01545000
H	-2.57251500	4.13748600	-1.42129700
B	1.67896900	1.06319000	0.05559000
P	-1.21324300	-0.05229200	0.93361300
C	-3.02882000	-0.29768100	0.68766000
C	-3.61479500	-0.84674800	-0.45819500
C	-3.85837100	0.10451100	1.74107000
C	-4.99716400	-0.98412300	-0.54794400
H	-2.98909400	-1.17400200	-1.28353100
C	-5.24249500	-0.02100400	1.64734300
H	-3.41359400	0.51862000	2.64285900
C	-5.81364800	-0.56858200	0.50213100
H	-5.43836400	-1.41625000	-1.44105800
H	-5.87185100	0.29885400	2.47213400
H	-6.89151100	-0.67686000	0.42919100
C	-0.51156200	-1.30881900	-0.22592900
C	-0.11092200	-2.53140000	0.32487700
C	-0.37103500	-1.11539300	-1.60582100
C	0.39768600	-3.54537400	-0.48391100
H	-0.19247600	-2.68510300	1.39833000
C	0.13830600	-2.12902600	-2.41591500
H	-0.66125300	-0.16721300	-2.05088700
C	0.52027800	-3.34653100	-1.85774400
H	0.70345900	-4.48829500	-0.04034500
H	0.23856000	-1.96368500	-3.48435900
H	0.91956300	-4.13394800	-2.48953900
O	2.84105100	1.11706000	-0.67162600
O	1.78408100	0.27745600	1.17270600
C	3.17977600	-0.07729400	1.31496700
C	3.26888800	-1.48478400	1.87871300
H	4.30988800	-1.82247300	1.91180900
H	2.87405300	-1.49672000	2.89830900
H	2.68572000	-2.18636400	1.27879300
C	3.79563800	0.93260300	2.27899900
H	3.74892300	1.94585700	1.86842800
H	3.23215900	0.91898100	3.21530700
H	4.83952700	0.69147000	2.49867200
C	3.70617900	0.08076700	-0.14647200
C	5.15021000	0.53514700	-0.26324000
H	5.81984400	-0.18790400	0.21347600
H	5.42777700	0.60838900	-1.31840500

H	5.30216900	1.51251900	0.19877900
C	3.45541400	-1.16476000	-0.99047600
H	2.42233300	-1.50869600	-0.87648900
H	3.62022900	-0.92112400	-2.04345300
H	4.12851100	-1.98111600	-0.71230900

P3

C	-0.34869266	1.74286393	-0.16544390
C	-0.89691420	0.52962913	0.10313607
C	-0.92541673	3.08556118	-0.37083440
C	-0.49117719	3.88166287	-1.43993013
C	-1.88842418	3.59699696	0.50851295
C	-1.03683138	5.14416579	-1.64417283
H	0.26502561	3.50412782	-2.12207219
C	-2.42722919	4.86313166	0.30246971
H	-2.18973363	3.01546519	1.37173922
C	-2.00979566	5.63668705	-0.77697139
H	-0.70134725	5.74491570	-2.48351112
H	-3.17004617	5.24755650	0.99414125
H	-2.43379098	6.62306259	-0.93595649
C	-2.36753930	0.31611219	0.16669897
C	-3.13879729	0.60629888	1.28586531
C	-3.03575245	-0.20202756	-0.93700459
C	-4.51187461	0.40813022	1.30715038
C	-4.40451443	-0.41542345	-0.94485016
C	-5.14638394	-0.10788400	0.18724016
F	-2.34196904	-0.52161341	-2.03654061
F	-5.00817206	-0.91156955	-2.02268961
F	-6.46147609	-0.30245651	0.19622211
F	-5.22021518	0.70707982	2.39442401
F	-2.56517544	1.11012424	2.38390571
B	0.37052849	-0.52472280	0.20376212
P	1.32875921	1.14503807	-0.42963345
C	2.59330301	2.06229327	0.48480566
C	3.93463258	1.66000416	0.43438099
C	2.21498666	3.14616667	1.28249998
C	4.88904391	2.35121347	1.17081179
H	4.22527254	0.80545990	-0.16948906
C	3.17805339	3.83005135	2.02022950
H	1.17560916	3.45794861	1.32769171
C	4.51094746	3.43495811	1.96291062
H	5.92758504	2.03942916	1.13284128
H	2.88381580	4.67230738	2.63750913
H	5.25972389	3.96930234	2.53902446
C	1.69028249	1.26386201	-2.21401018
C	0.75584173	0.71316436	-3.10166438
C	2.83736586	1.88651003	-2.70968222
C	0.98341579	0.77287317	-4.47156684
H	-0.15551278	0.25510272	-2.72601301
C	3.05651292	1.94214138	-4.08434498
H	3.55731135	2.33454134	-2.03319119
C	2.13582743	1.38207156	-4.96438164
H	0.25663024	0.34623106	-5.15501811
H	3.95054961	2.42554379	-4.46471997
H	2.31174628	1.42532623	-6.03442630
C	0.49110457	-1.76067973	-0.83129044
C	1.64819403	-2.06402549	-1.54337075
C	-0.55882404	-2.65510057	-1.00278482
C	1.74823345	-3.14871874	-2.40488040
C	-0.50262517	-3.75265859	-1.84898016
C	0.66410215	-3.99888103	-2.55829798
C	0.79363118	-0.98093343	1.69737736
C	2.12048780	-1.16867936	2.06967055
C	-0.14218321	-1.31291952	2.67000327
C	2.51015904	-1.60081859	3.32900937
C	0.20011185	-1.75294246	3.94162442
C	1.53851640	-1.89004396	4.27536022
F	2.87712725	-3.38447448	-3.07383864
F	2.74319616	-1.29328760	-1.43153447
F	-1.69595348	-2.47869404	-0.31175147
F	-1.54475026	-4.57344804	-1.97753609
F	0.74374685	-5.04653658	-3.37498196
F	-1.45184807	-1.24996675	2.39160153
F	-0.74199805	-2.05501604	4.83470316
F	1.88859091	-2.31151492	5.48804798
F	3.79974577	-1.74977740	3.63318508
F	3.09942433	-0.94853261	1.17738006

P4

C	-0.65593000	-0.59541900	2.08463300
H	-1.27456000	0.04307200	2.72149700
H	-0.73265000	-1.62449200	2.43697800
C	0.82272300	-0.17735700	2.11139800
H	0.94005200	0.85838300	1.77837400
H	1.18869500	-0.18698300	3.15222400
P	-1.13118000	-0.52704800	0.27622700
B	1.70746500	-1.21120600	1.32309400
C	3.09018700	-0.91277100	0.65572000

C	3.74252000	-1.90113300	-0.09084300
C	3.76371500	0.30869600	0.76030000
C	4.96984400	-1.69420300	-0.70641500
C	4.99142300	0.55103800	0.16421000
C	5.59448900	-0.46139700	-0.57253700
C	-2.84382400	-1.16260100	-0.05583800
C	-3.34208500	-0.87071600	-1.34708900
C	-3.63579600	-1.94998300	0.80854800
C	-4.62106400	-1.29715500	-1.70975500
C	-4.90463700	-2.35835900	0.39754400
C	-5.42712100	-2.03350300	-0.85067500
H	-4.99018300	-1.04684800	-2.70226300
H	-5.50634600	-2.95224800	1.08247900
C	-1.35908600	1.31287500	0.10399600
C	-2.45728500	2.01201800	0.64958100
C	-0.39523700	2.03016700	-0.64101000
C	-2.56711400	3.38859600	0.43456400
C	-0.54873300	3.40297200	-0.83043600
C	-1.63244100	4.10232500	-0.30645100
H	-3.41726100	3.91575100	0.86283000
H	0.20218500	3.94061100	-1.40557300
C	-3.56868400	1.35948500	1.43827300
H	-4.32141200	0.92756200	0.77041700
H	-3.21408500	0.54987000	2.07719500
H	-4.06136400	2.10231000	2.07005400
C	-1.79245000	5.57930400	-0.55578800
H	-2.48535600	6.02901000	0.15887400
H	-0.83371000	6.09879500	-0.47902000
H	-2.18533900	5.76272300	-1.56131300
C	0.82671700	1.37612700	-1.24265400
H	1.55092100	1.11850900	-0.46428800
H	0.58619800	0.45210000	-1.77289100
H	1.31859500	2.06106100	-1.93691200
C	-3.19107100	-2.39114100	2.18229500
H	-2.29443800	-3.01572500	2.13316300
H	-2.97191200	-1.54602700	2.84026900
H	-3.97660200	-2.98268400	2.65671700
C	-6.79443300	-2.50696900	-1.26945400
H	-7.47451600	-2.55543800	-0.41522700
H	-7.23101100	-1.84176000	-2.01816700
H	-6.74281400	-3.50964400	-1.70682700
C	-2.55325800	-0.10332900	-2.38293300
H	-2.55322700	0.97248000	-2.18239200
H	-1.50836700	-0.42883600	-2.41451800

H	-2.98508100	-0.26424800	-3.37303100
F	5.55244700	-2.65600200	-1.41668900
F	6.76588800	-0.24662900	-1.15332400
F	5.59597000	1.72957100	0.28827700
F	3.22621800	1.31726300	1.45018000
F	3.19101600	-3.10110400	-0.25082600
H	1.35193200	-2.34883700	1.29882700

P5

P	0.06911100	1.22715700	0.73606700
B	0.41902300	-0.89694300	1.08446200
C	0.52138100	1.14383200	2.52037400
C	0.38123500	-0.39224900	2.65051600
C	0.90323600	2.65006200	-0.08464900
C	1.24847300	5.04307900	-0.23743500
C	2.18036800	4.87520700	-1.26467000
C	2.45813100	3.58269000	-1.68405900
C	1.84147100	2.46138900	-1.11532900
C	-1.74215700	1.36156000	0.40242500
C	-2.75525700	1.42266500	1.38650200
C	-4.08460500	1.22450500	0.99918200
C	-4.45691000	0.98448300	-0.31816900
C	-3.45356300	0.98952200	-1.28320200
C	-2.11212100	1.16412900	-0.95232800
C	1.85981800	-1.50251400	0.67662200
C	3.03339200	-0.75711100	0.73002100
C	4.29394800	-1.26399500	0.45340900
C	4.42880500	-2.60854300	0.14609400
C	3.29657300	-3.40729300	0.10742800
C	2.05245300	-2.84881200	0.37012200
C	-0.86492300	-1.58564800	0.37944700
C	-2.13390500	-1.69340000	0.94859600
C	-3.26915300	-2.06694500	0.23987100
C	-3.17009000	-2.37245500	-1.10569800
C	-1.92912500	-2.30617100	-1.72008600
C	-0.82484200	-1.92551800	-0.97390500
C	2.84359100	6.06943000	-1.89596800
C	2.24115900	1.12573600	-1.69280300
C	-2.52112300	1.67308400	2.85653800
C	-5.87816100	0.65680100	-0.68330200
C	-1.10950900	1.10201200	-2.07778200
F	2.97969300	0.55757500	1.01838800
F	5.36615700	-0.47015900	0.47899700
F	5.62767800	-3.12363600	-0.11501200

F	3.41061500	-4.70470800	-0.17872800
F	1.01214900	-3.69178700	0.31332600
F	-2.35418100	-1.38937100	2.23795300
F	-4.46271900	-2.08456200	0.83733800
F	-4.25780200	-2.68912000	-1.80597300
F	-1.81430000	-2.56836500	-3.02352400
F	0.33011900	-1.81987100	-1.65720100
H	-0.02103800	1.75324000	3.23861100
H	1.57189600	1.44282900	2.54324900
H	-0.54507300	-0.65843400	3.15676900
H	1.19983300	-0.81434800	3.24182300
H	1.01585000	6.04769800	0.10881500
H	3.18123700	3.42420500	-2.48144900
H	-3.71498500	0.81623000	-2.32508100
H	3.61457300	5.76637300	-2.60776300
H	2.11128700	6.68341300	-2.43009900
H	3.30962400	6.70598400	-1.13792300
H	2.03168900	1.10542200	-2.76728800
H	1.73085800	0.27832600	-1.24027400
H	3.31863000	0.97433600	-1.56813700
H	-3.48114100	1.78848600	3.36359100
H	-2.00108100	0.84147800	3.33716200
H	-1.94840200	2.58643000	3.03052700
H	-6.13783100	1.04567500	-1.67125400
H	-6.58376700	1.06138100	0.04616600
H	-6.01234900	-0.43054000	-0.70884300
H	-1.58627700	0.72318200	-2.98505100
H	-0.69102600	2.08875300	-2.30117000
H	-0.27466400	0.43429100	-1.85131800
C	0.60652700	3.96379700	0.35874100
C	-0.38270800	4.24523800	1.46055700
H	-1.37398500	3.85054600	1.21804400
H	-0.06351800	3.80140500	2.40892500
H	-0.47947700	5.32122500	1.61869600
H	-4.85377300	1.24441400	1.76752000

P6

P	0.41113600	1.19190400	-0.76643400
C	-0.26150700	1.78898100	-2.38323500
H	-1.05951500	2.48328000	-2.11092400
H	0.47074200	2.35079100	-2.95267900
C	-0.80319300	0.55520600	-3.15198500
H	-1.89710400	0.58475200	-3.13533200
H	-0.50652200	0.61413200	-4.20435100
C	-0.31182800	-0.74495500	-2.49569700
H	0.74030500	-0.90999600	-2.74164400
H	-0.85669400	-1.61082900	-2.89157000
B	-0.56840300	-0.64865300	-0.88319200
C	2.21566400	0.76617700	-0.82508500
C	3.00661800	0.69192200	-1.99844700
C	2.60742700	1.18915400	-3.37041100
H	3.40264000	0.95891200	-4.08158800
H	2.46995900	2.27480400	-3.38669500
H	1.69519100	0.72353900	-3.74729200
C	4.28155600	0.12491900	-1.91887400
H	4.87227300	0.05948500	-2.82894300
C	4.81322000	-0.37483400	-0.73644700
C	6.15205300	-1.05805300	-0.69505500
H	6.78474200	-0.64412200	0.09549700
H	6.68097100	-0.96066900	-1.64575700
H	6.02224000	-2.12527000	-0.48666600
C	4.04776400	-0.24286900	0.41608500
H	4.44234200	-0.60874700	1.36170700
C	2.77414600	0.32284000	0.40014100
C	2.06910500	0.41753200	1.73095500
H	1.01256200	0.15789600	1.67140600
H	2.13764800	1.43005200	2.14240000
H	2.53204500	-0.26643600	2.44650100
C	0.26375400	2.61307000	0.40933700
C	0.92600400	3.82761000	0.09746300
C	1.71057200	4.05582100	-1.17146700
H	2.32088200	4.95592500	-1.07462000
H	1.04462100	4.20746900	-2.02687400
H	2.38163900	3.22509300	-1.39907700
C	0.84726600	4.89348600	0.98978800
H	1.36582300	5.81741700	0.74362500
C	0.13353000	4.81341000	2.18540000
C	0.05716600	5.99228200	3.11756800
H	-0.62841900	6.75164100	2.72704300
H	1.03563700	6.46598900	3.23598100
H	-0.30233900	5.69470200	4.10515800
C	-0.51209600	3.61903100	2.47131800
H	-1.07191600	3.52337700	3.39951100
C	-0.46271400	2.51677000	1.61199300
C	-1.19244700	1.28553100	2.08351800
H	-1.11592100	0.44529200	1.40152600
H	-2.25678400	1.50471700	2.21563600
H	-0.80403400	0.96451600	3.05612100

C	0.16153100	-1.74849900	0.07326800
C	-0.37011900	-2.17646500	1.29424400
F	-1.57460800	-1.74880700	1.71247600
C	0.27693100	-3.03322100	2.17336100
F	-0.29605300	-3.37811300	3.32791600
C	1.53061600	-3.52696100	1.84898300
F	2.17662600	-4.33597100	2.68556600
C	2.09482000	-3.16383900	0.63924700
F	3.30576100	-3.61588000	0.30456200
C	1.40087300	-2.32072300	-0.22110800
F	2.03433700	-2.06488000	-1.37355700
C	-2.18918700	-0.57602400	-0.67818100
C	-2.90312400	-1.76907000	-0.79446400
F	-2.24102800	-2.92350000	-0.97034500
C	-4.28450900	-1.85841100	-0.72887300
F	-4.89884500	-3.03735100	-0.83617300
C	-5.03248700	-0.70318300	-0.55303600
F	-6.36116800	-0.76216400	-0.48788600
C	-4.37740900	0.51109000	-0.44784700
F	-5.07678700	1.63589600	-0.28546600
C	-2.98926800	0.54691900	-0.52160500
F	-2.43739100	1.77400500	-0.41939600

P7

P	2.13382400	-0.96777900	1.35687700
B	-1.44470100	0.15602200	0.51723900
C	0.03859800	-2.63186100	2.41702300
H	0.48881100	-3.60273400	2.19980500
C	0.34700000	-1.55138200	1.34427000
H	0.05952500	-1.94763300	0.36905800
C	-0.65584500	-0.39447200	1.75683200
H	-0.10874200	0.41678500	2.24069400
C	-1.56698700	-1.10081000	2.81449000
H	-2.56799700	-0.67192400	2.89735400
C	-0.78367200	-1.11348200	4.13612700
H	-1.41710200	-1.46932200	4.95501100
H	-0.42163700	-0.11723200	4.40766600
C	0.36641200	-2.11867900	3.83604700
H	1.35553400	-1.65784900	3.88963700
H	0.35519600	-2.95282200	4.54310500
C	-1.49601700	-2.58526600	2.41871300
H	-1.94559300	-3.23636300	3.17572200
H	-1.93461800	-2.81520700	1.44320900
C	3.17504100	-1.83831000	0.07719300
C	2.84082700	-2.95893600	-0.71766900
C	3.77920000	-3.46929100	-1.61879500
H	3.49817400	-4.32853100	-2.22433400
C	5.05037800	-2.92563100	-1.77086800
C	5.38899500	-1.85951200	-0.94583600
H	6.38840800	-1.43409300	-1.01029900
C	4.49229000	-1.31957200	-0.02196100
C	1.50817100	-3.66841000	-0.69014400
H	1.59506900	-4.63227400	-1.19699400
H	0.72826400	-3.10480800	-1.21192400
H	1.16131900	-3.86753600	0.32395300
C	6.01627200	-3.46734600	-2.79039600
H	5.87266800	-4.54003400	-2.94438100
H	7.05234700	-3.30040700	-2.48465000
H	5.87630300	-2.97570600	-3.75948600
C	5.00498100	-0.18551800	0.83556600
H	6.09727500	-0.17102600	0.81857300
H	4.68617700	-0.28947200	1.87746000
H	4.65188400	0.78828400	0.48138700
C	2.02983500	0.74111000	0.62880800
C	2.25255000	1.82518100	1.51247200
C	2.31701600	3.12383700	1.00938300
H	2.47315900	3.94944200	1.70082800
C	2.18821500	3.39211900	-0.35465400
C	1.94111900	2.32274300	-1.20781900
H	1.80276900	2.51077100	-2.27156600
C	1.84411100	1.00724600	-0.74188000
C	2.42979700	1.63741200	3.00094200
H	3.32969000	1.05747900	3.22925900
H	1.59004800	1.10220400	3.45580300
H	2.51480500	2.60851800	3.49426700
C	2.26456500	4.80513400	-0.86963100
H	1.60028800	5.46689100	-0.30491800
H	1.98838800	4.86143700	-1.92577400
H	3.27864500	5.20525900	-0.76704700
C	1.49891400	-0.05736700	-1.75267500
H	0.90581600	0.37401100	-2.56422900
H	0.91768500	-0.86838100	-1.31074500
H	2.39708000	-0.50409300	-2.19260400
C	-2.58368700	-0.75009900	-0.12951600
F	-4.11103700	0.27707600	1.33762200
C	-3.87086000	-0.62938900	0.37479300
F	-6.14750800	-1.26618800	0.45944200
C	-4.93131700	-1.41045100	-0.06082700

F	-5.70775100	-3.10655400	-1.49703100
C	-4.70834600	-2.34832300	-1.05879200
F	-3.22671000	-3.37856500	-2.57046400
C	-3.43882700	-2.48802400	-1.60438800
F	-1.19978000	-1.83821400	-1.70263700
C	-2.40799100	-1.68653100	-1.13683500
C	-1.26654300	1.60010800	-0.06425900
F	-0.74887300	2.56032600	2.04861500
C	-0.93232900	2.69700800	0.73377700
F	-0.46686800	4.99263600	1.02585900
C	-0.81159100	3.98288100	0.22877500
F	-0.83307600	5.42329600	-1.62708200
C	-0.99235500	4.20446900	-1.12694300
F	-1.45155700	3.34912700	-3.27223600
C	-1.30653300	3.14293900	-1.96498400
F	-1.71826000	0.88561000	-2.28101200
C	-1.44650500	1.87581000	-1.42348500

P8

C	-0.83439300	-1.17451900	-0.89891300
H	-1.07398600	-0.83841800	-1.91994200
H	-0.50562900	-2.21375600	-0.97143000
P	-2.44734300	-0.93202700	0.02665900
B	0.28567400	-0.16579500	-0.48097000
C	-3.66759500	-1.97940300	-0.97922500
C	-3.50350700	-1.55578300	-2.44746900
H	-2.60759000	-1.99257000	-2.89910000
H	-4.36667900	-1.90966400	-3.02348900
H	-3.45267700	-0.46643900	-2.55982800
C	-5.07932600	-1.56909100	-0.53251500
H	-5.82560000	-2.12175400	-1.11672200
H	-5.26362200	-1.78342200	0.52377800
H	-5.24796300	-0.49957300	-0.69219600
C	-3.52199800	-3.49879500	-0.88753400
H	-3.86009300	-3.87994800	0.08013000
H	-4.14484400	-3.97288200	-1.65711400
H	-2.48926400	-3.82618900	-1.04734200
C	-2.05583500	-1.78014100	1.67759000
C	-0.99503700	-0.87920500	2.33276900
H	-0.83639800	-1.19322100	3.37138400
H	-0.02061600	-0.96589900	1.83348700
H	-1.29382500	0.17444900	2.33981800
C	-3.32138000	-1.74724700	2.54479100
H	-3.76669000	-0.74732800	2.58466300
H	-4.07673600	-2.44786300	2.17584300
H	-3.07287500	-2.04768000	3.57002600
C	-1.51167800	-3.21221800	1.64664100
H	-1.11880500	-3.46659700	2.63980700
H	-2.28437200	-3.94431300	1.40952000
H	-0.69247400	-3.33403900	0.93603100
C	1.81140000	-0.56433200	-0.40517000
C	2.24965100	-1.84675100	-0.06569900
C	2.83120100	0.34438200	-0.71110400
C	3.58702300	-2.21096800	-0.02049000
C	4.17723800	0.01357700	-0.69342000
C	4.55677000	-1.27371700	-0.34182100
F	1.37587400	-2.79929100	0.27419000
F	3.94470600	-3.44528700	0.32490400
F	5.10326500	0.91510100	-1.00857100
F	2.54314600	1.59899000	-1.06664200
F	5.83938800	-1.60539900	-0.31086000
C	-0.10664700	1.34424300	-0.21799300
C	0.44959000	2.06195200	0.83934700
C	-1.05714900	2.02654700	-0.97426300
C	0.08503700	3.36251000	1.14860200
C	-1.43347800	3.33503300	-0.71103400
C	-0.86084700	4.00359200	0.36123300
F	-1.63998800	1.43605100	-2.02508700
F	-2.33342200	3.95201400	-1.47374700
F	-1.21678900	5.25305000	0.63287900
F	0.63079400	3.99833700	2.18324600
F	1.36283500	1.48404400	1.62987500

P9

C	-0.29546200	-0.31606800	-1.15952600
H	-0.28805100	-1.29785100	-1.63416500
H	-0.71233700	0.39832000	-1.87639300
B	1.08219900	0.14916000	-0.56527100
C	2.25923000	-0.85597100	-0.29453200
C	3.38826300	-0.47724200	0.45316300
C	2.25467000	-2.15488300	-0.83288600
C	4.44059200	-1.35821800	0.68221400
C	3.30659900	-3.03974700	-0.62187300
C	4.39974000	-2.64311200	0.14598500
C	1.24919800	1.67699900	-0.21401700
C	1.22491500	2.64306100	-1.23001700
C	1.36709200	2.12589600	1.11015300
C	1.32014600	4.00183300	-0.94082300

C 1.44068000 3.48488700 1.40883900
C 1.42079800 4.42607500 0.38238000
P -1.40906500 -0.35657200 0.37504700
C -2.05434200 -2.14698400 0.33209000
C -3.29384200 -2.26067400 1.22714500
H -4.18500900 -1.84859200 0.74479500
H -3.49423500 -3.31929000 1.43393100
H -3.15219900 -1.75604800 2.18900100
C -2.37587600 -2.75006400 -1.03959300
H -3.09664000 -2.15777800 -1.60658900
H -1.48196300 -2.87925200 -1.65642700
H -2.80680400 -3.74960200 -0.89799400
C -0.92742200 -2.97054600 0.97469400
H 0.01685400 -2.86779600 0.43014400
H -0.74799500 -2.66325000 2.00953900
H -1.19943900 -4.03361200 0.97426400
C -2.80546700 0.84308800 -0.10261700
C -3.70559000 1.05297800 1.12421600
H -4.42612600 1.85194200 0.90961100
H -4.27697800 0.16157600 1.38839400
H -3.12043200 1.35544900 1.99847500
C -3.65407400 0.41843900 -1.30164900
H -4.27982600 -0.44869700 -1.06988200
H -4.32558400 1.23833700 -1.58788200
H -3.03698600 0.17672600 -2.17423100
C -2.14701500 2.19789400 -0.40854700
H -1.47893000 2.52236300 0.39675700
H -1.57169000 2.18817300 -1.33751400
H -2.93342500 2.95425300 -0.52043600
H 1.12130000 2.32855700 -2.26715600
H 1.30379600 4.73099300 -1.74566400
H 1.48070500 5.48560900 0.61226300
H 1.51311100 3.80886800 2.44290400
H 1.37608600 1.40026300 1.92035600
H 3.44342100 0.52906100 0.85840700
H 5.29616600 -1.04296800 1.27165900
H 5.22124000 -3.33211700 0.31869100
H 3.27615000 -4.03553100 -1.05341100
H 1.41379700 -2.48117800 -1.43959600

P10

P 0.46766900 1.20513000 0.74075100
B -0.58485200 -0.61377100 0.85870300
C -0.16138500 2.12969700 2.23075400
H -1.08343000 2.53910200 1.80898000
H 0.48227400 2.98719000 2.42739400
C -0.50497000 1.40098200 3.53842100
H -1.16109500 2.07447100 4.10197900
H 0.39182600 1.28202700 4.14674900
C -1.17597800 0.03677500 3.38511200
H -1.26488100 -0.40358900 4.38479700
H -2.20155400 0.16502700 3.01965200
C -0.38803600 -0.89868000 2.46118600
H 0.66501600 -0.88224200 2.76379700
H -0.71866600 -1.93434600 2.61629500
C 0.38572700 2.58368600 -0.51205600
C 1.14888700 3.76012300 -0.28818400
C 1.03516700 4.82509600 -1.18050200
H 1.62826800 5.71847800 -0.99363400
C 0.19607800 4.78875100 -2.29160100
C -0.51643200 3.61954900 -2.51450900
H -1.15934400 3.54249800 -3.38900700
C -0.43394000 2.51815700 -1.65787800
C 2.11483600 3.98695400 0.85515000
H 1.65354600 4.58760100 1.64667800
H 2.49954800 3.07007300 1.29544000
H 2.97591200 4.55218900 0.49023700
C 0.09962300 5.96680400 -3.22307700
H -0.63621100 5.79219800 -4.01103000
H -0.19079100 6.87194600 -2.68135800
H 1.06445100 6.16850600 -3.69908500
C -1.20855900 1.30178600 -2.09160800
H -0.84135000 0.95256200 -3.06293100
H -1.13586400 0.47429200 -1.39578000
H -2.26918000 1.54190600 -2.21290800
C 2.25740000 0.69843400 0.73859700
C 3.09500400 0.62590800 1.87351300
C 4.35120300 0.02408300 1.76470000
H 4.97304800 -0.03113300 2.65511500
C 4.82108800 -0.51706600 0.57574000
C 4.01597700 -0.37870700 -0.54937900
H 4.36902400 -0.76334600 -1.50387000
C 2.76091400 0.22625600 -0.50163600
C 2.74576900 1.13963300 3.24731200
H 3.66300800 1.33304400 3.80814800
H 2.17557400 2.06782200 3.22909900
H 2.17640300 0.39438000 3.81044300
C 6.13482800 -1.24396200 0.49937800

H	5.96140800	-2.32426000	0.44763800
H	6.69675500	-0.95871100	-0.39426400
H	6.75471700	-1.04389100	1.37632500
C	2.03305500	0.33535600	-1.81943000
H	2.43841500	-0.39278300	-2.52647300
H	0.96424800	0.14846700	-1.73881000
H	2.16017800	1.33200100	-2.25513000
C	-2.20127200	-0.53938700	0.59410000
C	-3.00753200	0.57691400	0.42045400
F	-2.46762500	1.81064600	0.33849400
C	-4.39197700	0.53269700	0.29865000
F	-5.09113900	1.65527500	0.12072900
C	-5.04298600	-0.68579700	0.36959500
F	-6.36779100	-0.75366600	0.25616900
C	-4.29132900	-1.83617300	0.55727100
F	-4.89742400	-3.02207900	0.62587600
C	-2.91342500	-1.73803300	0.67079700
C	0.14617800	-1.72142400	-0.10576800
C	1.35444100	-2.33622400	0.22890600
F	1.97362100	-2.07713200	1.38860600
C	2.03674300	-3.22368500	-0.59539200
F	3.22305000	-3.71016800	-0.22255400
C	1.48975200	-3.59077100	-1.81117600
F	2.12418600	-4.44164000	-2.61439600
C	0.26485900	-3.05537500	-2.17635500
F	-0.29377100	-3.40334300	-3.33727900
C	-0.36973500	-2.15521200	-1.33201600
F	-1.54695700	-1.69659400	-1.79315700
F	-2.25635700	-2.89601600	0.84599300

P11

P	1.29863100	0.10669100	0.25158900
B	-0.69891600	-0.71510200	0.67686700
C	1.11840500	0.04516100	2.10095900
H	0.57460900	0.93791600	2.41088700
C	2.27956600	-0.20309300	3.05047300
H	2.95918700	0.65648000	3.10138100
H	2.86845600	-1.06022100	2.69886100
C	1.69660200	-0.50178900	4.44116500
H	1.19502800	0.40196800	4.81384800
H	2.50748900	-0.72539800	5.14240700
C	0.68993500	-1.65920900	4.41534700
H	1.21647100	-2.57760900	4.12050100
H	0.30233900	-1.83031900	5.42547500
C	-0.47125700	-1.41711300	3.43870500
H	-1.11360000	-2.30178100	3.40190600
H	-1.09074300	-0.58857300	3.79343000
C	0.09699100	-1.11030100	2.04935700
H	0.67394900	-2.01144100	1.77678300
C	2.92062200	-0.67517400	-0.17592700
C	3.01389300	-2.03451000	-0.53395100
C	4.26465300	-2.57440000	-0.85602900
H	4.31542400	-3.62651100	-1.12817100
C	5.42705000	-1.81891500	-0.83874100
C	5.32310200	-0.48199900	-0.45322200
H	6.22404400	0.12549700	-0.40456000
C	4.10687300	0.10493700	-0.11658100
C	1.85589100	-2.99727000	-0.60527400
H	2.11094200	-3.91720600	-0.07097300
H	0.94087500	-2.60668200	-0.17222200
H	1.64594600	-3.26645800	-1.64439300
C	6.75840400	-2.40711900	-1.21981900
H	7.07497700	-2.04293700	-2.20290000
H	7.53431400	-2.12394300	-0.50299000
H	6.71383600	-3.49750300	-1.26496300
C	4.14465500	1.55503500	0.29810800
H	3.50578900	1.75418600	1.15971400
H	5.16466400	1.83412400	0.57088100
H	3.81853500	2.21733800	-0.50905700
C	1.20882300	1.77323100	-0.52028700
C	1.10364200	1.77525500	-1.93088200
C	0.66260900	2.92543800	-2.58430100
H	0.54951500	2.90062100	-3.66538500
C	0.33551700	4.08631100	-1.88929100
C	0.54425200	4.09689700	-0.51277800
H	0.34409000	5.00998700	0.04529300
C	0.97702900	2.97210000	0.19335600
C	1.45762100	0.57258600	-2.76874900
H	1.07276200	0.69195300	-3.78365400
H	2.54443800	0.44872300	-2.82974000
H	1.04231500	-0.35277200	-2.36768000
C	-0.27457500	5.27245100	-2.58410300
H	0.10860300	6.21404500	-2.18203400
H	-0.08314500	5.24993500	-3.65929800
H	-1.36033800	5.26804600	-2.43705800
C	1.16955700	3.17043600	1.68186700
H	0.24168600	3.01285600	2.24385500
H	1.93098800	2.52048300	2.11265700

H	1.47744600	4.20269800	1.86543000
C	-1.49617600	-1.89569600	-0.09003200
C	-2.57820300	-2.42615900	0.61076000
F	-2.88230200	-1.93360700	1.82308100
C	-3.40106800	-3.43099100	0.12414900
F	-4.41890800	-3.89472100	0.85020900
C	-3.15964400	-3.94578500	-1.14051500
F	-3.93106100	-4.91251500	-1.63290100
C	-2.11200000	-3.43518500	-1.88946200
F	-1.87769100	-3.91091600	-3.11369500
C	-1.31857300	-2.42262600	-1.36333200
F	-0.34619000	-1.97244800	-2.16960500
C	-1.50572900	0.68712100	0.50726700
C	-1.87907900	1.57849100	1.50522000
F	-1.63087000	1.31618700	2.79975400
C	-2.45498000	2.82055600	1.25599600
F	-2.73239700	3.64670700	2.26688400
C	-2.69323400	3.21532800	-0.04811500
F	-3.19917200	4.41808400	-0.31263500
C	-2.35994500	2.35602300	-1.08535200
F	-2.54213500	2.74261700	-2.34884000
C	-1.79325000	1.12873700	-0.78926200
F	-1.43301100	0.37451200	-1.83744600

P12

P	-0.05756100	1.50645500	0.75235500
C	0.05768300	2.09627900	2.48713600
H	0.66816300	2.99858900	2.57845500
H	-0.96469200	2.33187900	2.80344100
C	0.58229300	0.88951900	3.28951600
H	1.65730200	0.76075500	3.11887700
H	0.45356100	1.08290100	4.35945200
C	-0.16973800	-0.36600500	2.82786300
H	-1.21112200	-0.28268300	3.15674300
H	0.23668600	-1.25777700	3.31108900
B	-0.11745500	-0.51561000	1.20043200
C	-1.42790600	2.33272900	-0.10664800
C	-2.02215200	1.70875100	-1.20991200
H	-1.66519700	0.73778500	-1.54328500
C	-3.07347100	2.33353200	-1.87552000
H	-3.53543400	1.84705700	-2.72842900
C	-3.53516700	3.57422700	-1.44273700
H	-4.35964100	4.05578700	-1.95870500
C	-2.94133100	4.19870800	-0.34844800
H	-3.29841900	5.16665500	-0.01221100
C	-1.88803500	3.58192900	0.31900700
H	-1.42671500	4.07604700	1.16928400
C	1.45843900	2.02336200	-0.12129000
C	2.68494800	2.01893300	0.55318600
H	2.72607200	1.82472500	1.62119400
C	3.86718100	2.24972700	-0.14311300
H	4.81375500	2.23972700	0.38777900
C	3.83547300	2.48167800	-1.51670300
H	4.75966800	2.65023600	-2.06012300
C	2.61685300	2.49853000	-2.18949400
H	2.58608600	2.68398200	-3.25818900
C	1.43101700	2.27173500	-1.49670100
H	0.48724200	2.27778700	-2.03296200
C	1.29468900	-1.01564500	0.56492600
C	2.42799300	-1.39080400	1.27768200
F	2.40744600	-1.51589100	2.61555000
C	3.66315400	-1.63358200	0.68119200
F	4.71278100	-1.98812800	1.42590800
C	3.80344300	-1.50408500	-0.68879700
F	4.97948300	-1.72613800	-1.27198800
C	2.70089200	-1.13795700	-1.45043800
F	2.82075500	-0.98397500	-2.76930200
C	1.49327100	-0.92270900	-0.81277700
F	0.46651700	-0.52874400	-1.59204100
C	-1.45855000	-1.18226700	0.57703700
C	-2.71055000	-0.64010500	0.86744400
F	-2.79887600	0.46228300	1.63747200
C	-3.90938300	-1.14072100	0.38323500
F	-5.06890700	-0.55722100	0.68928300
C	-3.89108100	-2.26383600	-0.43041400
F	-5.02677400	-2.76890200	-0.90785300
C	-2.67674200	-2.85396600	-0.73916900
F	-2.64579500	-3.94015600	-1.51311100
C	-1.49923000	-2.31415900	-0.23378900
F	-0.37558500	-2.96146400	-0.57320100

P13

P	-1.10459000	-1.27893700	-0.54449300
B	0.27279500	0.13304200	-0.94981900
C	-0.78544500	-2.65877100	-1.70856000
H	0.15027100	-3.13477900	-1.40087200
H	-1.58911200	-3.39650400	-1.61375400
C	-0.67004000	-2.13641300	-3.14743000

H	-0.47169000	-2.99557100	-3.79694900
H	-1.63631900	-1.72017800	-3.46029700
C	0.42244200	-1.07778400	-3.32874800
H	0.52484400	-0.87621500	-4.40074900
H	1.38405000	-1.49622800	-3.00020100
C	0.13661100	0.23679900	-2.58318500
H	-0.84103800	0.60316000	-2.91485100
H	0.85508200	0.99420000	-2.91436000
C	-1.39360900	-1.99153200	1.11031000
C	-1.05823700	-3.31067000	1.42195600
H	-0.56975700	-3.94405800	0.68786900
C	-1.34200500	-3.81972500	2.68706200
H	-1.08135300	-4.84638400	2.92320900
C	-1.95385900	-3.01601000	3.64375200
H	-2.17086900	-3.41533700	4.62947100
C	-2.29006100	-1.69859200	3.33611200
H	-2.76840800	-1.06856000	4.07888500
C	-2.01770900	-1.18974400	2.07271200
H	-2.29876300	-0.16753900	1.82926100
C	-2.79485100	-0.71665100	-0.98616000
C	-3.83774000	-1.64500900	-0.85683800
H	-3.63234700	-2.64898500	-0.49274700
C	-5.14292600	-1.28809500	-1.17128700
H	-5.93970200	-2.01760400	-1.06817300
C	-5.42536400	0.00504300	-1.60850500
H	-6.44548200	0.28633900	-1.85013700
C	-4.39816300	0.93373800	-1.73123100
H	-4.61105100	1.94328600	-2.06758600
C	-3.08557600	0.57710300	-1.42393400
H	-2.30125100	1.31393100	-1.54128700
C	1.81012700	-0.30759000	-0.59166100
C	2.27042000	-1.47236400	0.00354900
F	1.40046800	-2.42510300	0.39331200
C	3.61071800	-1.75422600	0.23824300
F	3.97195500	-2.90555500	0.80712400
C	4.57151000	-0.82447200	-0.11944600
F	5.86271100	-1.06906800	0.09349000
C	4.17000900	0.36757900	-0.70381400
F	5.08152400	1.27785300	-1.04745900
C	2.81936800	0.59930600	-0.92097400
F	2.50698600	1.78162600	-1.47667300
C	-0.05990600	1.46025900	-0.05879000
C	-0.42509300	2.70572900	-0.55808700
F	-0.65248300	2.87500600	-1.87161600
C	-0.61403300	3.82825900	0.23837400
F	-0.97434400	4.99317400	-0.30216100
C	-0.41421600	3.73565200	1.60655900
F	-0.58782500	4.80054700	2.38559200
C	-0.04188900	2.51855200	2.15705000
F	0.15339500	2.41457200	3.47228800
C	0.13501400	1.42548500	1.32128900
F	0.51258700	0.27814100	1.90830800

P14

P	0.33407400	2.00399200	0.14746100
B	-0.04431100	0.09540400	0.84062900
C	1.43562900	2.34694200	1.57915100
H	2.35521000	1.78717700	1.38660100
H	1.70201800	3.40200100	1.68058300
C	0.74020700	1.75697700	2.81492300
H	0.06030500	2.50639800	3.23423000
H	1.49256000	1.56506700	3.58433000
C	-0.03682500	0.46466900	2.44915200
H	-1.07125300	0.55636200	2.79489300
H	0.37470600	-0.38196300	3.00174300
C	-1.10692900	3.24126600	0.29361900
C	-0.62363200	4.56863100	0.89787600
H	-1.47703200	5.25501100	0.94225500
H	0.15662000	5.05228400	0.30651900
H	-0.25574100	4.44212300	1.92010200
C	-1.79432500	3.51756900	-1.05279200
H	-2.20412900	2.61119300	-1.49984700
H	-1.13969900	3.99984700	-1.77971200
H	-2.62924400	4.20216700	-0.86515400
C	-2.17449200	2.65931500	1.23565600
H	-1.80282800	2.51745900	2.25158700
H	-2.57330900	1.70841600	0.87503200
H	-3.00479400	3.37301700	1.28646500
C	1.38921200	2.35613800	-1.38300200
C	2.77141400	1.69167100	-1.23503800
H	2.72156100	0.61349300	-1.08106500
H	3.35977100	2.13772300	-0.42891900
H	3.31914300	1.86375300	-2.16845300
C	1.65470600	3.86070600	-1.54415300
H	2.34858900	3.99499100	-2.38202400
H	2.13061700	4.28681300	-0.65510700
H	0.75968800	4.44020400	-1.77218100
C	0.71971100	1.79346300	-2.64320300

H	-0.26654800	2.22378300	-2.82607400
H	0.60569800	0.70868700	-2.58589000
H	1.35358400	2.02761800	-3.50602900
C	1.22335100	-0.82726300	0.35873200
C	1.22255700	-1.50925900	-0.86054800
F	0.18700000	-1.39089400	-1.70939800
C	2.26919900	-2.29795600	-1.31687500
F	2.19594200	-2.90809000	-2.49981300
C	3.40377400	-2.44369200	-0.53383700
F	4.41994100	-3.19380000	-0.95102000
C	3.46356100	-1.78972200	0.68502800
F	4.54837400	-1.90998700	1.45102300
C	2.39069800	-1.00941500	1.10092400
F	2.57399700	-0.39092500	2.28091800
C	-1.44584500	-0.61263500	0.40088400
C	-2.15495000	-0.37374300	-0.76989300
F	-1.67811900	0.50480000	-1.67140800
C	-3.34947500	-0.99549000	-1.10077300
F	-3.97970200	-0.70317100	-2.24059900
C	-3.87899800	-1.94423400	-0.23918300
F	-5.02245000	-2.55947900	-0.53432500
C	-3.19902500	-2.24725000	0.92876500
F	-3.68546700	-3.17013400	1.76063200
C	-2.00965200	-1.58960000	1.21830600
F	-1.39998900	-1.95977000	2.35496100

P15

P	1.24812500	-0.66651800	0.22878200
B	-1.57715400	-0.36621300	0.32717700
C	-0.32080800	-1.12651000	-0.53286800
H	-0.31388500	-2.18957600	-0.24419100
C	-0.42643000	-1.06366100	-2.06379500
H	-0.55549000	-0.03189600	-2.38985400
H	-1.36636900	-1.57048700	-2.29512600
C	0.70159900	-1.72955900	-2.84744400
H	1.62853900	-1.15053900	-2.80093700
H	0.43257500	-1.81008100	-3.90340000
H	0.90085100	-2.74224300	-2.47868300
C	1.93399000	0.99903000	0.06996400
C	1.81574100	1.77891600	-1.07623400
F	1.27974600	1.26932200	-2.18034100
C	2.24365600	3.09673600	-1.10314100
F	2.08423200	3.83261900	-2.19228400
C	2.81335700	3.65289100	0.03733300
F	3.21452300	4.90957900	0.02307600
C	2.96846100	2.88961600	1.18849400
F	3.51381200	3.42243300	2.26986500
C	2.52744400	1.57541700	1.19393400
F	2.67618200	0.86180700	2.30620400
C	2.62447400	-1.80572100	-0.10990800
C	3.62169600	-1.55785800	-1.05167400
F	3.60973600	-0.43535400	-1.76995600
C	4.63628900	-2.47180000	-1.29356800
F	5.57094400	-2.21619400	-2.19668100
C	4.66513700	-3.66560500	-0.58030500
F	5.62653500	-4.54205400	-0.80677000
C	3.68267800	-3.94369600	0.36267900
F	3.71072000	-5.08300100	1.03638900
C	2.67957200	-3.01209800	0.58533700
F	1.74405800	-3.29239000	1.49031900
C	-3.02382700	-0.84038500	-0.30586600
C	-4.15497300	-0.02576400	-0.30644900
F	-4.07628800	1.25419500	0.09615500
C	-5.41812900	-0.45208800	-0.69536000
F	-6.45236400	0.38857600	-0.68087900
C	-5.60568000	-1.76793600	-1.09010800
F	-6.80932300	-2.19646200	-1.46337600
C	-4.52154500	-2.63085000	-1.08274500
F	-4.67805900	-3.90506300	-1.44434100
C	-3.27958200	-2.15568800	-0.68270000
F	-2.28482200	-3.07019700	-0.67950600
C	-2.43534800	-0.09992300	2.78913900
C	-2.64749300	-0.51404900	4.09709100
C	-2.10469700	-1.71764600	4.54795600
C	-1.36957800	-2.49963900	3.66711000
C	-1.17108800	-2.07370500	2.35198400
C	-1.67678800	-0.85659000	1.87940800
F	-0.49329211	-2.89885176	1.52601453
F	-0.85533181	-3.67944453	4.07467566
F	-2.30395944	-2.12334963	5.82004053
F	-3.38312163	0.24981681	4.93247088
F	-2.98379856	1.06128767	2.37285572

P16

C	-1.43431600	-0.23882700	2.79069800
H	-2.26606700	-0.33623700	2.09401100
H	-1.56554100	0.69067900	3.35821900
H	-1.48810000	-1.06036500	3.51198400

C	-0.10004800	-0.23814500	2.11935400
H	0.74716000	-0.30893400	2.80826600
C	0.18233800	-0.15103600	0.81240100
P	-1.00609800	0.02506900	-0.58886400
C	-1.96583000	1.53505600	-0.09573700
C	-3.22094300	1.62662800	0.50116800
F	-3.91286900	0.53532700	0.84574500
C	-3.82472200	2.84571300	0.78241500
F	-5.02055600	2.88080200	1.36389100
C	-3.17705200	4.02825400	0.45991400
F	-3.74376800	5.19629400	0.73347200
C	-1.93209000	3.98115700	-0.15005700
F	-1.30211100	5.10597400	-0.47484900
C	-1.36491400	2.74853400	-0.42807800
F	-0.17709400	2.74816100	-1.04448300
C	-2.25384100	-1.32605800	-0.41114300
C	-3.35461100	-1.23558200	-1.26371000
F	-3.51384800	-0.12765000	-1.99690000
C	-4.31504800	-2.22443100	-1.37699200
F	-5.35110200	-2.07677300	-2.19772100
C	-4.18055700	-3.38183400	-0.62251500
F	-5.08454700	-4.34927000	-0.71573400
C	-3.10147900	-3.51441900	0.23532900
F	-2.97019000	-4.61701700	0.96906000
C	-2.15555700	-2.49882900	0.33449600
F	-1.15872800	-2.71355100	1.19462700
B	1.68244300	-0.14346200	0.34172200
C	2.43505100	1.18855100	0.01231800
C	3.56385100	1.23062100	-0.81846100
F	4.05899700	0.11154700	-1.34875000
C	4.21099300	2.40646800	-1.15870200
F	5.26949800	2.39508200	-1.96448500
C	3.74019700	3.61158200	-0.65339300
F	4.34736400	4.74449900	-0.96986800
C	2.63281100	3.62543600	0.18103800
F	2.17666700	4.77703500	0.66556600
C	2.00742500	2.43030700	0.49825500
F	0.95296900	2.52596700	1.30904200
C	2.40769100	-1.53476400	0.24252200
C	1.75302000	-2.66076700	-0.25547100
F	0.53029300	-2.54761400	-0.78984000
C	2.33183400	-3.91957500	-0.28392300
F	1.67716700	-4.96177200	-0.78750700
C	3.61550600	-4.08349400	0.21857100
F	4.18338600	-5.28120200	0.21005500
C	4.30814200	-2.99089700	0.72323100
F	5.53873600	-3.14977100	1.20305400
C	3.70300500	-1.74472200	0.71588300
F	4.40936800	-0.72200500	1.20622700

P18

P	-1.16627000	1.19580900	-0.56083400
Si	3.39267800	-2.21039200	-0.32196200
B	0.43875400	-0.40535600	0.13445300
C	-0.36853800	1.05109200	-2.21471200
H	-0.58460900	0.07326400	-2.64043800
H	-0.69785000	1.82293300	-2.90938600
C	1.13025100	1.11128200	-1.93588300
H	1.67715400	1.09469900	-2.88147700
H	1.38843000	2.05460100	-1.43535900
C	1.49853500	-0.06838400	-1.04956700
C	2.67953200	-0.69953500	-1.22468300
C	4.55916500	-3.09347900	-1.51195200
H	4.96441000	-3.98723900	-1.02343900
H	5.40268100	-2.46599400	-1.81477300
H	4.04518400	-3.42235000	-2.42121600
C	4.43166100	-1.66025800	1.15440300
H	3.82265900	-1.39283500	2.02223600
H	5.05510000	-0.79718700	0.89696100
H	5.10245300	-2.47111100	1.46053100
C	2.13428900	-3.50903100	0.19640300
H	2.68208900	-4.35553600	0.62786000
H	1.59261300	-3.88348100	-0.67850300
H	1.40532900	-3.18139600	0.93787900
C	3.65591700	-0.15799800	-2.22706200
C	3.75256800	-0.67763200	-3.52391000
H	3.08347800	-1.48118100	-3.82207800
C	4.68504500	-0.17219400	-4.42551200
H	4.74448300	-0.58493000	-5.42832100
C	5.54372400	0.85713100	-4.04357100
H	6.27411300	1.24727700	-4.74547800
C	5.45581200	1.38404300	-2.75801300
H	6.11500300	2.19176700	-2.45403500
C	4.51731300	0.88191500	-1.85860100
H	4.43348400	1.30381100	-0.85968500
C	-1.33292400	3.04091300	-0.37211000
C	-2.01476000	3.79365400	-1.36560900
C	-2.02684700	5.18366400	-1.27812000

H	-2.55472000	5.74422900	-2.04711600
C	-1.39882900	5.87564800	-0.24353500
C	-0.79021300	5.12423200	0.75106400
H	-0.33059300	5.62949400	1.59811000
C	-0.75934000	3.72637500	0.71826400
C	-2.77725600	3.20391400	-2.53002600
H	-3.65688200	3.81979100	-2.73333600
H	-3.12860200	2.19199100	-2.33910000
H	-2.17824500	3.19850900	-3.44711100
C	-1.39471900	7.37986300	-0.21302900
H	-2.37613200	7.78270400	-0.47842300
H	-0.67301600	7.77814600	-0.93404800
H	-1.12217000	7.75772100	0.77493900
C	-0.16583200	3.06169000	1.93304000
H	-0.76697100	3.31343400	2.81352100
H	0.85092100	3.42019300	2.11631300
H	-0.13914500	1.97915900	1.84921700
C	-2.88228800	0.49123800	-0.54179100
C	-3.58731900	0.63196700	0.67926200
C	-4.76706200	-0.08114900	0.88627800
H	-5.27182500	0.01646200	1.84437900
C	-5.29361000	-0.93287300	-0.07851000
C	-4.63883900	-0.99499500	-1.30334300
H	-5.05515200	-1.62334700	-2.08794100
C	-3.45034000	-0.30726500	-1.56782200
C	-3.11210800	1.50895300	1.81090100
H	-3.70292500	1.31065400	2.70747000
H	-3.21118500	2.57134500	1.56720800
H	-2.06946300	1.31416300	2.06432100
C	-6.48851300	-1.79900400	0.20748700
H	-7.07063200	-1.99156200	-0.69723400
H	-7.14484900	-1.34553700	0.95410300
H	-6.15559300	-2.76637600	0.60002200
C	-2.91505600	-0.48034000	-2.97533500
H	-2.09157000	-1.19909800	-3.03676800
H	-2.57506600	0.45655400	-3.41875800
H	-3.71394500	-0.86657800	-3.61193200
C	1.13631700	-0.20549300	1.58069400
F	2.12014900	1.80809900	0.80411400
C	1.98625700	0.88873600	1.77074600
F	3.41709700	2.25718200	3.05525700
C	2.68139000	1.15100600	2.93980000
F	3.23632600	0.49091000	5.13503300
C	2.58342600	0.26301100	4.00023400
F	1.70181700	-1.74190300	4.85636800
C	1.79350500	-0.86537400	3.85650400
F	0.40388000	-2.22623000	2.63715400
C	1.09782300	-1.07964000	2.67071700
C	-0.63222800	-1.59893600	-0.01903700
F	0.09340000	-2.28290100	-2.18391600
C	-0.75086600	-2.40522100	-1.14710200
F	-1.84110900	-4.04691000	-2.44976100
C	-1.76016700	-3.34726400	-1.31583200
F	-3.71891100	-4.37012200	-0.49467100
C	-2.71598200	-3.51157100	-0.32956500
F	-3.58826100	-2.85300700	1.75516300
C	-2.64550600	-2.73523800	0.81863400
F	-1.67994000	-1.00937500	2.01872700
C	-1.63100500	-1.80307200	0.93936400

P19

P	1.65845200	-1.11722400	-1.51001700
B	-2.13349400	-0.11692000	-0.79295400
C	-0.04166000	-1.46152300	-0.82114300
H	-0.09101000	-1.09188800	0.20949100
C	-1.02110100	-0.67223100	-1.75178800
H	-1.35622500	-1.32194400	-2.55952800
H	-0.51034800	0.18214400	-2.21152800
C	-0.33410000	-2.96195400	-0.83288700
H	-1.32657400	-3.15711700	-0.41765300
H	0.38669500	-3.51989000	-0.23155000
H	-0.31407900	-3.35491200	-1.85390200
C	2.86267900	-1.86820800	-0.31269400
C	2.62751600	-2.39089500	0.95734900
C	3.63091500	-2.97847400	1.71687700
C	4.92462800	-3.03979400	1.22112100
C	5.20403400	-2.52014300	-0.03398000
C	4.17855100	-1.94884800	-0.77131900
C	1.83232000	0.65652100	-0.99908600
C	1.81641200	1.09499000	0.32457700
C	2.00091500	2.42410600	0.66781000
C	2.15783100	3.37377900	-0.33416600
C	2.14164800	2.98363600	-1.66443800
C	1.98709400	1.63729900	-1.97608000
C	-3.50324900	-0.81959900	-0.50460000
C	-3.88605400	-2.01553000	-1.13166100
C	-5.09940600	-2.64467400	-0.90034400
C	-6.00693200	-2.07272400	-0.02106700

C	-5.68851200	-0.88358700	0.61832000
C	-4.46357500	-0.28486000	0.36943600
C	-1.70313000	1.19388500	-0.01298400
C	-1.58315400	1.22219100	1.37268200
C	-1.12481100	2.32942400	2.06898900
C	-0.78378600	3.47594900	1.36140000
C	-0.88323100	3.48913200	-0.02198000
C	-1.31446500	2.34816900	-0.68396100
F	1.41277600	-2.36082100	1.50971500
F	3.35882600	-3.47206300	2.92194300
F	5.89011500	-3.58978000	1.94566800
F	6.44225900	-2.56907100	-0.51689000
F	4.49281800	-1.44760000	-1.96903400
F	1.60898100	0.23105600	1.32293200
F	1.94513600	2.81177900	1.94135400
F	2.27217800	4.65431800	-0.01442100
F	2.26512100	3.89307900	-2.62507100
F	1.97620300	1.30548700	-3.26637600
F	-3.08306500	-2.63202400	-2.00074900
F	-5.40111900	-3.78312200	-1.51754500
F	-7.17060600	-2.65916800	0.20729100
F	-6.55960400	-0.32477300	1.45269600
F	-4.24572400	0.86280300	1.00985900
F	-1.90507200	0.12893900	2.07430200
F	-0.99397300	2.30408000	3.39133500
F	-0.30621200	4.53541300	2.00032800
F	-0.48158000	4.56069100	-0.70574600
F	-1.35768500	2.39637200	-2.02232500

P20

P	2.81794500	0.21205200	-0.30407400
C	2.52073000	1.95912500	0.17597500
C	3.51257300	2.90884100	0.43992300
C	3.21925700	4.27744600	0.55125400
C	1.92709400	4.76052400	0.39382600
C	0.91187200	3.83727500	0.12946900
C	1.23976900	2.48407600	0.03140900
O	0.13324500	1.71400400	-0.21121200
C	-0.93078100	2.56327600	-0.27828300
C	-0.52392400	3.89389100	-0.09126000
C	-1.47864500	4.90722300	-0.14894700
C	-2.80899400	4.55852800	-0.37005800
C	-3.18315800	3.22170500	-0.50344100
C	-2.25855500	2.15970400	-0.46092600
B	-2.70937600	0.68219400	-0.51730900
C	-1.73832100	-0.51261100	-0.81712700
C	-0.75740500	-0.46161400	-1.80850600
F	-0.60205600	0.63996300	-2.54337200
C	0.06065600	-1.53979700	-2.11054400
F	0.98311300	-1.45433100	-3.06855000
C	-0.09281700	-2.72395200	-1.40435300
F	0.67833300	-3.76966700	-1.67715300
C	-1.04860200	-2.81953500	-0.40388500
C	-1.84820200	-1.72334200	-0.12951200
F	-2.74475100	-1.86126200	0.85445800
F	-1.18305800	-3.95622900	0.27770900
C	-4.22451200	0.32895000	-0.24678700
C	-4.92345800	-0.53816300	-1.08463900
F	-4.32048500	-1.05888300	-2.15899200
C	-6.25038500	-0.88151200	-0.87589800
F	-6.88688600	-1.69530700	-1.71491300
C	-6.91651300	-0.36609000	0.22728800
F	-8.18496100	-0.68907400	0.44679400
C	-6.25475000	0.48637500	1.09939100
C	-4.93157500	0.82100000	0.84861200
F	-4.33232100	1.62523800	1.73182300
F	-6.88976800	0.97007700	2.16469000
C	2.11146200	-0.96894200	0.93573500
C	2.30692500	-2.32696200	0.57277800
C	3.18925100	-2.73729400	-0.58269800
C	1.66030800	-3.33899600	1.28287100
C	0.79664000	-3.06020000	2.33753500
C	0.03640100	-4.14810200	3.04773400
C	0.64182100	-1.72779100	2.70583500
C	1.28935300	-0.67689300	2.04821000
C	1.05101200	0.70106700	2.62178000
C	4.65718500	0.09241900	-0.14636600
C	5.37831600	0.10575100	-1.36208900
C	4.69642700	0.28279700	-2.69810600
C	6.76615700	-0.02968500	-1.33722000
C	7.46912900	-0.17903800	-0.14328900
C	8.96346900	-0.36300600	-0.14519700
C	6.74782800	-0.16530400	1.04746200
C	5.35734800	-0.02669800	1.07171900
C	4.67282600	0.01127700	2.41363600
H	4.54090300	2.57840300	0.55317000
H	4.02807100	4.97034900	0.75864500
H	1.71299000	5.82136700	0.47276100

H	-1.19201600	5.94669500	-0.02128000
H	-3.56578300	5.33360100	-0.42499400
H	-4.23328800	2.98822000	-0.65509300
H	3.12755000	-3.81507100	-0.74329400
H	2.89096800	-2.24227900	-1.51464300
H	4.23777200	-2.48363100	-0.40153200
H	1.81145700	-4.37151600	0.97552300
H	0.30802500	-4.19712200	4.10706000
H	-1.04062500	-3.96223100	2.99220700
H	0.23237800	-5.12519000	2.60038800
H	-0.00431300	-1.48622700	3.54809900
H	0.81468400	0.61343100	3.68520200
H	0.20066500	1.19623200	2.14225200
H	1.91259100	1.36092300	2.52699600
H	5.43391700	0.27142700	-3.50390700
H	4.15589600	1.23354600	-2.74922300
H	3.96257500	-0.50599000	-2.89052200
H	7.31405400	-0.01572600	-2.27700700
H	9.22416400	-1.41716000	-0.29061900
H	9.40472200	-0.04117500	0.80140900
H	9.43248100	0.20392100	-0.95375900
H	7.28069600	-0.25637600	1.99173000
H	5.40112000	0.20709000	3.20422800
H	3.91355500	0.79661700	2.45009800
H	4.17283400	-0.93498800	2.64115700

P21

P	-1.14225000	0.18525100	-0.90781000
C	-0.73191200	-1.10261000	-2.16968300
C	-1.33851900	-1.22229400	-3.42474900
C	-0.81935300	-2.08505100	-4.38657800
C	0.31979900	-2.84049000	-4.12115000
C	0.95841000	-2.74840500	-2.88516800
C	0.41204100	-1.87325100	-1.94737400
O	1.01096800	-1.74489800	-0.71515000
C	2.38817000	-1.81211700	-0.70895400
C	3.03266200	-0.96411400	0.18764000
C	4.43111300	-0.96700000	0.16915400
C	5.12923800	-1.81295800	-0.68908100
C	4.44047900	-2.64752500	-1.56684800
C	3.04501200	-2.66093600	-1.59388100
C	2.17761400	-3.56709600	-2.46304700
C	2.94106400	-4.12202300	-3.66371700
C	1.69098100	-4.74106200	-1.58451900
B	2.19362200	0.02484200	1.06630500
C	1.10620700	-0.43782500	2.08923600
C	0.28132600	0.45725000	2.78607700
F	0.39757600	1.77661600	2.62057400
C	-0.71620700	0.05535600	3.65974700
F	-1.47815400	0.95543200	4.27778000
C	-0.94749600	-1.29715600	3.85343800
F	-1.90347700	-1.70151600	4.67730000
C	-0.17726700	-2.22765700	3.17222900
C	0.82974000	-1.79446600	2.32606900
F	1.53300100	-2.75918100	1.73249700
F	-0.42894500	-3.52701100	3.31785000
C	2.50879700	1.54979100	0.81478200
C	2.87607100	2.46022300	1.79898600
F	2.97843200	2.05397600	3.06917300
C	3.14798700	3.79100400	1.52358300
F	3.50769500	4.63214000	2.49167400
C	3.03245700	4.24973900	0.21699700
F	3.26604600	5.52752800	-0.06103800
C	2.67025900	3.37355800	-0.79560000
C	2.43987400	2.04060700	-0.48669100
F	2.09341700	1.23093000	-1.49257300
F	2.53163100	3.81549800	-2.04484500
C	-2.37111000	1.20687700	-1.85229200
C	-1.91724800	2.46780500	-2.30322500
C	-0.49553900	2.93408500	-2.09769000
C	-2.79954000	3.32111000	-2.96416400
C	-4.12616200	2.96703600	-3.20267200
C	-5.06714500	3.92093900	-3.88881600
C	-4.55165100	1.71194600	-2.77906400
C	-3.70436000	0.82305400	-2.11023600
C	-4.28102200	-0.51706000	-1.71951600
C	-2.20287800	-0.50832700	0.45544600
C	-2.46133800	-1.86626600	0.74648600
C	-1.85155900	-3.02337200	-0.00569400
C	-3.31112900	-2.18742000	1.80894400
C	-3.91848200	-1.22012100	2.60489800
C	-4.83647000	-1.59533600	3.73685100
C	-3.61058000	0.11125400	2.34336800
C	-2.75166400	0.48254400	1.30735700
C	-2.43420400	1.95504600	1.17606800
H	-2.20969100	-0.62021200	-3.66171900
H	-1.30323300	-2.16320300	-5.35476300
H	0.71147100	-3.50282900	-4.88591600

H	4.97833500	-0.29878500	0.82929300
H	6.21418100	-1.81291700	-0.68673100
H	5.00074300	-3.28727900	-2.24106900
H	3.79914100	-4.71280200	-3.33254200
H	3.29887000	-3.32358400	-4.32091700
H	2.30492900	-4.79370800	-4.24621800
H	2.54378600	-5.34497300	-1.25838000
H	1.17171800	-4.37620400	-0.69294600
H	1.00429900	-5.37814100	-2.15097500
H	-0.35070100	3.91986600	-2.54597800
H	-0.25114000	3.00540800	-1.03171900
H	0.22739700	2.24863600	-2.54753700
H	-2.43868900	4.28886900	-3.30589900
H	-5.91698200	3.39428900	-4.32998400
H	-5.46449200	4.65389900	-3.17817000
H	-4.55929000	4.47702500	-4.68152200
H	-5.57622500	1.40465700	-2.97926400
H	-5.12466500	-0.76131400	-2.37060000
H	-4.64568600	-0.51182700	-0.68684900
H	-3.54768000	-1.32295400	-1.79944300
H	-2.22452000	-3.96686400	0.39939600
H	-0.76131300	-3.02834300	0.09403500
H	-2.08672500	-3.00042900	-1.07341900
H	-3.49669400	-3.23782400	2.02284500
H	-5.88192200	-1.39833300	3.47628600
H	-4.60728800	-1.01441900	4.63461500
H	-4.74736400	-2.65572900	3.98493900
H	-4.02227900	0.88966700	2.98252500
H	-2.58519700	2.45241600	2.13767700
H	-1.39549600	2.12408400	0.87528400
H	-3.07350700	2.44663900	0.43525800

P22

P	1.90796700	-1.54610900	1.08811000
C	3.00804400	-0.05737400	1.10678400
C	4.20815100	0.05153500	1.81615500
C	4.97077400	1.21474500	1.76715300
C	4.55984000	2.28839800	0.98227500
C	3.38569000	2.21498000	0.23422600
C	2.62839200	1.04623100	0.33270700
O	1.44226400	0.93562300	-0.36016500
C	0.73450700	2.07680300	-0.62916400
C	-0.65629500	1.94135200	-0.77476900
C	-1.38349700	3.12480200	-0.98833000
C	-0.75636900	4.35952900	-1.07296600
C	0.62945400	4.43905900	-0.95309600
C	1.40229200	3.29829800	-0.74188700
C	2.92650900	3.27116700	-0.76408500
C	3.54371700	4.64121700	-0.48851500
C	3.35318600	2.80039200	-2.17415200
B	-1.40459300	0.57820900	-0.73962200
C	-2.87842000	0.55846900	-0.17496600
C	-3.89655000	-0.19146200	-0.76412000
F	-3.64568400	-0.91041600	-1.86276100
C	-5.18882700	-0.23457000	-0.26713600
F	-6.13050400	-0.95691200	-0.87005500
C	-5.49495900	0.48098000	0.88297500
F	-6.72390400	0.44407200	1.38212800
C	-4.51249300	1.22915700	1.51386500
C	-3.23288200	1.26251500	0.97721800
F	-2.32358200	1.97737000	1.64483500
F	-4.80180900	1.90033500	2.62691700
C	-0.77078800	-0.76523100	-1.25211800
C	-1.10151800	-2.00186000	-0.69322400
F	-2.01557500	-2.08560700	0.28160300
C	-0.47203500	-3.18595100	-1.04033600
F	-0.77633900	-4.32894700	-0.42430900
C	0.53068400	-3.16353100	-1.99515700
F	1.17481800	-4.28150200	-2.30863700
C	0.87696600	-1.96594100	-2.60454500
C	0.22681600	-0.80191600	-2.22973800
F	0.60048700	0.31466100	-2.85909900
F	1.82845400	-1.94706800	-3.53616200
C	3.05711900	-2.91334600	1.63013000
C	4.01703600	-3.23084100	0.48127600
C	2.20940900	-4.13973800	1.98144100
C	0.88107000	-1.24273200	2.63488300
C	1.67237700	-1.05779200	3.92587100
C	-0.06963900	-0.06851300	2.39484400
H	4.54885600	-0.78024600	2.42523200
H	5.89247900	1.28224500	2.33585700
H	5.16870500	3.18520800	0.94497900
H	-2.46302100	3.06655500	-1.09993600
H	-1.33761700	5.25967400	-1.24191900
H	1.11296900	5.40686800	-1.03698100
H	3.21837300	5.36628000	-1.23892000
H	4.63330700	4.59087600	-0.55890600
H	3.27408800	5.01841700	0.50256500

H	3.02298600	3.52365400	-2.92687600
H	4.44230600	2.70459500	-2.22844300
H	2.90705500	1.83013500	-2.41378000
H	3.63556500	-2.62765800	2.51619400
H	4.69581900	-4.04350400	0.76186100
H	4.62451400	-2.36420300	0.20255900
H	3.46169000	-3.55068800	-0.40839100
H	2.85258900	-5.01030100	2.14924600
H	1.51689900	-4.38971500	1.16783900
H	1.61875400	-3.98452900	2.88959200
H	0.26975000	-2.15217700	2.71483500
H	0.98916400	-0.93090800	4.77358200
H	2.31365600	-1.91665700	4.14762300
H	2.30357000	-0.16437800	3.87420100
H	-0.71224500	0.09126300	3.26736300
H	0.47321400	0.86505600	2.20829200
H	-0.72189600	-0.26711500	1.53898800

P23

C	0.93216100	-0.41581600	-3.47076700
H	2.02256800	-0.37539200	-3.52971100
H	0.52650000	0.34249100	-4.14808600
H	0.60178900	-1.39534900	-3.83361900
C	0.45656200	-0.17925700	-2.07683600
C	1.10581300	0.00671800	-0.90377100
P	-1.20655000	-0.08667200	-1.40129300
C	-2.29806900	1.16073600	-2.12026400
C	-2.43567700	1.28867700	-3.50638300
H	-1.89521000	0.62144300	-4.17288400
C	-3.27221200	2.27012000	-4.02661800
H	-3.37815100	2.37578600	-5.10141700
C	-3.97390500	3.11524300	-3.16813800
H	-4.62530800	3.88049000	-3.57806800
C	-3.84764600	2.97988900	-1.78813200
H	-4.39957400	3.63554000	-1.12266200
C	-3.00807800	2.00342100	-1.25895200
H	-2.90299500	1.89280500	-0.18242600
C	-2.03422200	-1.69807500	-1.50685600
C	-3.42282800	-1.79457800	-1.38180400
H	-4.03429400	-0.89649300	-1.37035600
C	-4.01867000	-3.04590400	-1.25223700
H	-5.09612000	-3.12066200	-1.14740900
C	-3.23400500	-4.19702500	-1.24817200
H	-3.70177200	-5.17021500	-1.13874200
C	-1.84974400	-4.10259500	-1.38278800
H	-1.23852000	-4.99933700	-1.38154300
C	-1.24610400	-2.85591300	-1.50822000
H	-0.16350300	-2.77921300	-1.58530700
B	-0.04612600	0.18451700	0.25578700
C	2.57988400	-0.02600500	-0.78542400
F	2.68885100	-2.16371200	-1.78989000
C	3.31570300	-1.12231200	-1.23300600
F	5.36461800	-2.25605400	-1.53425700
C	4.69354700	-1.19108100	-1.10075100
F	6.69706400	-0.19049700	-0.37687500
C	5.37568000	-0.13842900	-0.50582700
F	5.32677600	1.98030000	0.51718900
C	4.67417300	0.96647800	-0.04670500
F	2.65226500	2.08509100	0.26889000
C	3.29402500	1.00597400	-0.18415800
C	-0.23059800	1.62237600	0.95901800
F	0.17139000	2.73663100	-1.09060700
C	-0.11494000	2.80021500	0.21863900
F	-0.17874800	5.15229700	-0.02070900
C	-0.29750600	4.06803300	0.74600000
F	-0.79247800	5.40904200	2.62088800
C	-0.60949100	4.20215000	2.09149300
F	-1.03160500	3.18179300	4.16852000
C	-0.73370600	3.06691000	2.87373700
F	-0.67636500	0.77423400	3.13850900
C	-0.54374100	1.81259000	2.30307900
C	-0.35941200	-1.12847800	1.14317600
F	1.80098100	-2.09017600	0.98624400
C	0.51470300	-2.18256900	1.36096500
F	1.01106400	-4.36844800	2.12043000
C	0.12575800	-3.38740100	1.93729500
F	-1.58435800	-4.72013100	2.85813800
C	-1.19417800	-3.56964100	2.31468900
F	-3.38772900	-2.71345900	2.45578200
C	-2.10968100	-2.54280000	2.11781700
F	-2.59204900	-0.39987000	1.34592600
C	-1.66919700	-1.35627900	1.55975200

P24

P	-1.09161200	1.11359900	-0.23901300
B	0.09210600	-0.46574200	0.53775800
C	0.40714200	1.11054300	-1.27648300
C	1.05762700	0.09620800	-0.65586400

C	-1.49133400	2.80486800	0.38054700
C	-2.74287900	3.43611200	0.17131900
C	-2.97121800	4.67888800	0.77018700
H	-3.93724100	5.15317400	0.61610200
C	-2.01463400	5.33609500	1.53476500
C	-0.76463500	4.73627600	1.65807000
H	0.02218500	5.24753600	2.20778800
C	-0.47976200	3.49485700	1.09521300
C	-3.86105800	2.91561700	-0.70312300
H	-4.58962700	3.71322600	-0.86126700
H	-4.39055700	2.06808300	-0.26080600
H	-3.50048700	2.59813800	-1.68341900
C	-2.31557700	6.64888700	2.20384200
H	-2.59001300	6.48944300	3.25213400
H	-3.14765300	7.16289900	1.71699000
H	-1.44515700	7.30998800	2.19095300
C	0.94624800	3.01836100	1.20727700
H	1.50895300	3.30455200	0.31130100
H	1.04898000	1.94132800	1.31079400
H	1.42932100	3.48503800	2.06887900
C	-2.55433700	0.27603000	-0.95100500
C	-2.72344600	0.01950000	-2.32015200
C	-3.66869300	-0.93228500	-2.71198600
H	-3.77339000	-1.15562300	-3.77130500
C	-4.45711800	-1.61392500	-1.79080000
C	-4.34241100	-1.26730600	-0.44287000
H	-4.98260000	-1.75383200	0.29007400
C	-3.40541200	-0.33813000	-0.00214100
C	-1.92292200	0.70983600	-3.39013300
H	-0.96593300	0.20557100	-3.55054100
H	-1.70958700	1.75350700	-3.14259700
H	-2.46956300	0.69333100	-4.33601000
C	-5.35773800	-2.73944200	-2.21901700
H	-6.27914800	-2.76459300	-1.63146200
H	-4.84680700	-3.69810600	-2.07105500
H	-5.62031500	-2.66411200	-3.27685000
C	-3.33987500	-0.00500900	1.46691800
H	-3.52082100	1.06116400	1.64654600
H	-2.36704600	-0.23576600	1.90934700
H	-4.09405200	-0.57123400	2.01806500
C	0.82955100	1.96038700	-2.40555700
C	0.56557800	3.33469600	-2.41716300
H	0.03313500	3.78964300	-1.58680200
C	0.98543700	4.12354100	-3.48487300
H	0.77956000	5.18922500	-3.47758000
C	1.66349200	3.55053700	-4.55690900
H	1.98718800	4.16677400	-5.38959000
C	1.91419700	2.17941200	-4.56222300
H	2.42637600	1.72187300	-5.40277900
C	1.49832700	1.38936500	-3.49716300
H	1.67041100	0.31639300	-3.51281400
C	2.44476500	-0.31555400	-0.97094100
C	3.48461700	0.61512400	-1.03940300
F	3.24603700	1.91307600	-0.84004200
C	4.79547200	0.24419400	-1.29875100
F	5.75826100	1.16242600	-1.35231600
C	5.10409800	-1.09381800	-1.49392800
F	6.35665500	-1.46225900	-1.73936100
C	4.09939900	-2.04715900	-1.42076600
F	4.39234500	-3.33367300	-1.59724700
C	2.79657800	-1.65223500	-1.15773200
F	1.87002500	-2.60593700	-1.08058800
C	0.69152500	-0.37812000	2.03950000
C	0.22717300	0.41199100	3.08484600
F	-0.79645900	1.26113500	2.89649500
C	0.76924700	0.39005400	4.36257900
F	0.28568600	1.18350700	5.31919200
C	1.81941000	-0.46940400	4.64478200
F	2.35393300	-0.50081900	5.86271500
C	2.30542700	-1.29525700	3.64322700
F	3.30356500	-2.14059900	3.90003600
C	1.73320900	-1.23505800	2.38088000
F	2.21633700	-2.08875600	1.46333800
C	-0.70320500	-1.85075300	0.25979600
C	-1.33445100	-2.57903500	1.26855100
F	-1.21726300	-2.22009000	2.55779400
C	-2.16138200	-3.67140400	1.03477300
F	-2.75148400	-4.30340800	2.05075900
C	-2.38715400	-4.08741300	-0.26561200
F	-3.20894000	-5.10442700	-0.52084200
C	-1.76935300	-3.41299200	-1.30704800
F	-2.00557100	-3.78339900	-2.56665500
C	-0.94009700	-2.33828700	-1.02832900
F	-0.39662200	-1.73851700	-2.09886400

P25

B	-0.06995500	-0.79947400	0.68256000
C	0.72266400	0.74231400	2.68063900

C	0.23052400	-0.53686000	2.26924300
C	0.97195800	1.73953100	1.70159600
C	1.03433200	1.02942800	4.03715400
C	1.59921700	2.92065200	2.02803900
H	1.86381700	3.63516700	1.25235900
C	0.03816700	-1.47187900	3.26411100
H	-0.33577600	-2.45761400	3.02037400
C	0.79348400	0.03043700	5.01528500
H	1.01652800	0.24351100	6.05698500
C	1.61242800	2.28295400	4.35619600
H	1.84980700	2.49652200	5.39516400
C	1.90665000	3.20149200	3.37575400
H	2.38305100	4.14187800	3.63213100
C	0.30354000	-1.18716800	4.62902200
H	0.12406500	-1.95965600	5.37045200
C	0.68469600	-2.12120700	0.08406700
C	1.71165400	-2.18061300	-0.85074000
C	0.24305300	-3.37904000	0.50370200
C	2.31838200	-3.36051100	-1.27285500
C	0.81177000	-4.58062300	0.11588300
C	1.87430300	-4.57299000	-0.77725400
C	-1.66331400	-0.79876400	0.32234100
C	-2.28093500	-1.61387300	-0.62890900
C	-2.53157700	0.10505700	0.93627300
C	-3.87011300	0.25310300	0.60645300
C	-3.62455600	-1.51983800	-0.97388000
C	-4.42562700	-0.57514200	-0.35539000
P	0.63359000	1.15401400	0.01864900
C	-0.83560600	1.89954900	-0.79103500
C	-1.66031400	2.90008700	-0.23956200
C	-1.24543700	1.21542400	-1.95852600
C	-2.91579900	3.11615000	-0.81284400
C	-2.50232500	1.47800000	-2.49615200
C	-3.37483400	2.39032100	-1.90730200
H	-3.56608400	3.86448500	-0.36654400
H	-2.81697200	0.92968500	-3.38149500
C	2.19842300	1.67076900	-0.82927100
C	2.27622500	2.66039100	-1.83607100
C	3.39135800	1.08323700	-0.34165300

5. Coordinates of intermediates and transition states involved in Figure 2 and Figure 3.

1-methylpyrrole

C	3.53147600	2.97128500	-2.37030100
C	4.61047000	1.43500200	-0.91279200
C	4.70492400	2.36506800	-1.94281000
H	3.58553100	3.72865200	-3.14842600
H	5.51535800	0.96494100	-0.53380200
C	-1.28193800	3.77099900	0.93232800
H	-1.20149300	3.19884800	1.85811000
H	-0.32427200	4.27208800	0.76795000
H	-2.04137400	4.54244200	1.07293000
C	-4.78766000	2.54339800	-2.40083800
H	-5.43947800	1.83986600	-1.87031200
H	-5.16884400	3.55085300	-2.21920100
H	-4.86510200	2.32727200	-3.46888300
C	-0.36578300	0.20143600	-2.64997200
H	0.64894400	0.57759900	-2.81454100
H	-0.27468800	-0.72508300	-2.07479200
H	-0.79116100	-0.06051600	-3.62076400
C	1.11910200	3.47450600	-2.37433400
H	0.41667700	2.88080100	-2.96336500
H	0.54404900	3.95089300	-1.57751700
H	1.51011800	4.26281000	-3.02011500
C	6.03269900	2.69537300	-2.57008800
H	6.81948800	2.76569200	-1.81459900
H	6.32757600	1.91524000	-3.27931200
H	5.99101800	3.64189500	-3.11334500
C	3.45095900	0.11641100	0.81730000
H	3.61275800	0.66079100	1.75458700
H	2.55132600	-0.48339300	0.95072300
H	4.28576800	-0.57439500	0.67749900
F	-0.84893400	-3.47989100	1.28189500
F	0.33153300	-5.73771000	0.56831900
F	2.43951300	-5.71242500	-1.16364100
F	3.31308300	-3.32395500	-2.15927800
F	2.17653600	-1.06554700	-1.43751400
F	-2.05916800	0.96806300	1.84835200
F	-4.61281000	1.20315300	1.17396400
F	-5.70393100	-0.43556800	-0.70460700
F	-4.13865100	-2.31598000	-1.91107600
F	-1.58767700	-2.52317500	-1.33326100

N1_TS

C	0.83433000	2.57940600	-0.74319400
C	0.57604700	1.23561000	-1.04785800
C	0.97720100	0.83996100	-2.33879500

C	1.53890000	1.70876000	-3.26381600
C	1.74872400	3.04536000	-2.92668800
C	1.39879400	3.47539300	-1.65578700
H	0.84722100	-0.20358000	-2.61767400
H	1.82388500	1.34378900	-4.24593100
H	2.18922400	3.73666400	-3.63785400
H	1.57109900	4.50801000	-1.36221900
H	0.20561300	1.72634000	1.27290000
B	-0.02051100	0.03353000	-0.07725600
N	0.51010700	3.05606300	0.59141200
C	-0.27649000	0.64526600	1.63191200
C	-1.58697000	1.08287500	1.99984700
C	-1.80783600	0.91905200	3.35682500
H	-2.29675300	1.47401500	1.28336300
H	-2.69478600	1.15806600	3.92375400
C	-0.64189900	0.34167200	3.85594000
H	-0.41053400	0.02194200	4.86392200
N	0.25215900	0.17710400	2.87402300
C	1.51179900	-0.50825600	3.13062000
H	2.35278000	0.18624700	3.11166300
H	1.45072400	-0.96249800	4.12015800
H	1.67328200	-1.28553200	2.38496200
C	-1.58030300	-0.43522400	-0.41396000
C	-2.15212400	-1.46401500	0.38894400
C	-2.44671300	0.14715600	-1.37279600
C	-3.46223600	-1.89493200	0.20930800
C	-3.76834500	-0.30983700	-1.51703700
C	-4.29962100	-1.33228700	-0.75009600
C	1.17467600	-1.12544800	-0.20211400
C	1.01511000	-2.39578600	-0.82168700
C	2.50722600	-0.80857200	0.18448200
C	2.07608000	-3.30949900	-0.87279100
C	3.53816000	-1.74776700	0.12870600
C	3.33827400	-3.02900500	-0.37040300
C	-0.24238400	-2.86821400	-1.52774700
H	-0.97526200	-3.29819000	-0.83950800
H	-0.75159300	-2.06717200	-2.06421500
H	0.02619300	-3.64525800	-2.24851200
C	4.44316800	-4.05227600	-0.38397200
H	4.29202400	-4.78791600	-1.17803600
H	5.41853000	-3.58162700	-0.53462500
H	4.48602400	-4.59788600	0.56511800
C	-2.08691200	1.27154900	-2.32912400
H	-1.56565800	2.10309100	-1.85430600
H	-1.43820400	0.92859900	-3.13973900
H	-2.99911400	1.66564400	-2.78441300
C	-5.71052200	-1.82367700	-0.94339400
H	-6.22725400	-1.93287900	0.01484900
H	-6.28824500	-1.13547400	-1.56541000
H	-5.72273500	-2.80388900	-1.43195000
C	-1.40479200	-2.15608700	1.51232700
H	-0.32664600	-2.18431600	1.35891600
H	-1.60267900	-1.65495500	2.46603500
H	-1.75677400	-3.18709400	1.61384900
H	1.90219000	-4.27709600	-1.34057700
H	-3.84355700	-2.69446600	0.84375400
H	-4.40162500	0.16273700	-2.26571500
C	1.57232100	3.80501400	1.26491600
H	1.35256300	3.84037200	2.33682000
H	2.52906600	3.30517800	1.10928000
H	1.64818500	4.83640000	0.89505300
C	-0.75571000	3.78962400	0.63041600
H	-1.06560100	3.92796900	1.67163700
H	-0.65796500	4.77034400	0.14540800
H	-1.52197400	3.21592500	0.10606000
H	4.53282000	-1.45746800	0.46444400
C	2.94122500	0.57784600	0.61267100
H	3.19276500	1.17789700	-0.26943800
H	2.17145200	1.12348300	1.15695400
H	3.83296300	0.52279800	1.24553100

N1_P

C	0.00068600	-2.63584200	-0.82676700
C	-0.25897100	-1.27564400	-1.01185400
C	-0.89369000	-1.00579700	-2.24604000
C	-1.20803400	-1.97525300	-3.18494200
C	-0.91125700	-3.31683000	-2.93892400
C	-0.30096200	-3.64744500	-1.74175500
H	-1.15403500	0.02688700	-2.46162100
H	-1.69235800	-1.68867700	-4.11346100
H	-1.15575300	-4.08736400	-3.66215200
H	-0.06416700	-4.68486700	-1.51747500
H	0.75043400	-2.19334800	1.01754200
B	-0.00791600	0.05504200	0.00272000
N	0.66006000	-3.07219700	0.42371600
C	0.33167700	-0.39284500	1.59190900
C	1.58897000	-0.87242300	1.98490000
C	1.60383500	-1.11739800	3.38333500

H	2.44230200	-0.91738200	1.31772100
H	2.42940400	-1.46838400	3.98558300
C	0.36474100	-0.75122500	3.83793000
H	-0.04535600	-0.73646600	4.83780200
N	-0.39467100	-0.32397000	2.77453400
C	-1.67165200	0.33490500	3.02434100
H	-2.51835500	-0.34289000	2.89750700
H	-1.66335700	0.69936900	4.05293600
H	-1.79938800	1.17972000	2.35122200
C	1.40564000	0.88461500	-0.36521700
C	1.79053800	1.93120200	0.52574300
C	2.33309000	0.59859100	-1.39844000
C	2.99474600	2.61426500	0.38735900
C	3.54983700	1.29676500	-1.49245200
C	3.90910400	2.30476300	-0.61526600
C	-1.45769600	0.87128700	-0.17198200
C	-1.61562600	2.17160600	-0.71537900
C	-2.67108600	0.19611400	0.15226400
C	-2.87918600	2.77913300	-0.77902100
C	-3.90926700	0.83043600	0.07762800
C	-4.03865700	2.14428600	-0.36414400
C	-0.49142300	2.98866400	-1.32222100
H	0.12913100	3.48342900	-0.57006600
H	0.18135300	2.37761300	-1.92591500
H	-0.91541900	3.76677900	-1.96275400
C	-5.38312500	2.82209600	-0.41686400
H	-5.31057300	3.80590100	-0.88673600
H	-6.10488300	2.22730200	-0.98544400
H	-5.79697700	2.96026400	0.58738300
C	2.12534400	-0.40254600	-2.52292200
H	1.94029200	-1.42442400	-2.18533800
H	1.27676500	-0.13100300	-3.15561200
H	3.01259000	-0.42364100	-3.16068400
C	5.21310900	3.04872000	-0.74171700
H	5.78882900	3.00084000	0.18802900
H	5.82965600	2.63378200	-1.54293300
H	5.04375200	4.10766000	-0.96245400
C	0.92808900	2.38950000	1.68616000
H	-0.13854000	2.29329600	1.47572000
H	1.14753100	1.81381100	2.59340700
H	1.13196600	3.44194400	1.90572300
H	-2.94989000	3.78671700	-1.18516200
H	3.22889900	3.41203800	1.09152600
H	4.23452200	1.04110700	-2.29915200
C	-0.13319300	-4.02612500	1.23801200
H	0.32432700	-4.08602200	2.22776700
H	-1.15100100	-3.64576200	1.31739400
H	-0.13781600	-5.00756200	0.76161800
C	2.05131500	-3.54091700	0.20824300
H	2.51791300	-3.69817300	1.18278700
H	2.03771200	-4.46764400	-0.36694800
H	2.58820500	-2.76663200	-0.34095000
H	-4.80393200	0.27641300	0.36025600
C	-2.73088800	-1.26671200	0.55468600
H	-2.75585500	-1.90805700	-0.33377600
H	-1.87203900	-1.57353000	1.15478900
H	-3.64008800	-1.46474600	1.13096000

N2_TS

C	1.73143100	2.06520800	-0.82767500
C	0.45799600	1.51246700	-1.05039800
C	-0.22679800	1.99535600	-2.17441300
C	0.30906800	2.96215100	-3.01934200
C	1.57026700	3.49230400	-2.76001800
C	2.28544800	3.04023500	-1.65702100
H	-1.21777100	1.59530000	-2.38133300
H	-0.25796000	3.30444700	-3.87982000
H	1.99711600	4.24714900	-3.41254300
H	3.27601100	3.43877300	-1.45457300
H	1.35493700	1.05643700	1.11716600
B	-0.14158200	0.36293100	-0.04987600
N	2.49798700	1.54579000	0.30901000
C	0.11062600	1.06828900	1.52842600
C	-0.03843200	2.44345300	1.83493600
C	-0.51945400	2.60505600	3.13304700
H	0.19815900	3.23517500	1.13541400
H	-0.72824600	3.52517500	3.65762500
C	-0.71873900	1.32015300	3.61969400
H	-1.12861700	0.99555400	4.56749800
N	-0.33966200	0.41572500	2.70061000
C	-0.61310100	-1.00628700	2.87737500
H	0.23721200	-1.52471200	3.32757400
H	-1.48420200	-1.10947800	3.52625100
H	-0.83813000	-1.45588500	1.91210000
C	-1.74568400	0.03308400	-0.17963500
C	-2.14817400	-1.30988800	-0.31264200
C	-2.79415600	0.98026100	-0.12417500
C	-3.47021000	-1.73954500	-0.40174000

C	-4.12363100	0.55390000	-0.22716600
C	-4.49014900	-0.77991700	-0.36253100
C	0.80593800	-0.97882500	-0.22335000
C	0.93019700	-1.64612600	-1.46537500
C	1.60503700	-1.48251600	0.81340300
C	1.79281400	-2.74031600	-1.58727300
C	2.46794800	-2.57174300	0.70123400
C	2.56447800	-3.22380600	-0.53358800
C	0.19784900	-1.19910400	-2.70923300
H	0.13757600	-2.01698200	-3.43262500
H	-0.81627700	-0.86108700	-2.48814500
H	0.72027500	-0.36464000	-3.18968400
C	3.47769300	-4.40747400	-0.71698200
H	3.44986900	-4.76841300	-1.74745100
H	4.51648400	-4.15589200	-0.47512200
H	3.19254800	-5.23834500	-0.06172000
C	-2.59101600	2.46431900	0.08543800
H	-1.64668900	2.82551900	-0.31831400
H	-3.39994900	3.02670300	-0.39066000
H	-2.60503500	2.70728800	1.15336600
C	-5.93724700	-1.18230400	-0.47195800
H	-6.22217800	-1.87577300	0.32726900
H	-6.59352900	-0.31107600	-0.41293600
H	-6.13912100	-1.69341900	-1.42012000
H	1.86884200	-3.23517000	-2.55471100
H	-4.91092800	1.30658300	-0.19525300
C	3.54861200	0.61492300	-0.15428700
H	3.96714400	0.08536400	0.70567300
H	3.12411500	-0.11045200	-0.84645800
H	4.34415000	1.18080300	-0.65713600
C	3.11118300	2.57794000	1.15491000
H	3.57344000	2.08545000	2.01468000
H	3.88499000	3.13918600	0.61927500
H	2.35006000	3.26990100	1.51418300
C	-3.79641400	-3.20345200	-0.54917900
H	-4.35337900	-3.39986800	-1.47242200
H	-2.88552000	-3.80574800	-0.57173100
H	-4.41968800	-3.56318300	0.27780200
H	-1.37653600	-2.07685400	-0.36372600
C	3.29347600	-3.02372800	1.87836300
H	4.36767500	-2.95202000	1.67025600
H	3.08114400	-2.41596100	2.76217700
H	3.09247000	-4.07017600	2.13381100
H	1.60804900	-0.96935600	1.77333200

N2_P

C	-1.16048700	-2.09798100	1.10067100
C	-0.13384600	-1.14254000	1.14726400
C	0.53415600	-1.11459900	2.38814700
C	0.24057100	-1.97824900	3.43856100
C	-0.76086000	-2.93660700	3.30802200
C	-1.47968800	-2.98757900	2.12213100
H	1.31860100	-0.38077100	2.53027600
H	0.79955500	-1.90333900	4.36642600
H	-0.98967900	-3.62158200	4.11704100
H	-2.28461500	-3.70863000	2.00367700
H	-1.58859900	-1.45528600	-0.76849700
B	0.17063800	-0.08184300	-0.11456800
N	-2.02004800	-2.14294800	-0.11119900
C	0.10923300	-0.92152600	-1.55204700
C	-0.50823800	-0.67697000	-2.78133100
C	-0.16702800	-1.69802600	-3.70703000
H	-1.11041100	0.18726300	-3.01668800
H	-0.48491300	-1.78192600	-4.73605900
C	0.65990600	-2.56089000	-3.03910900
H	1.16351200	-3.45984800	-3.36665800
N	0.81728700	-2.10925700	-1.75230300
C	1.78306800	-2.73285700	-0.86342500
H	1.92358900	-3.76874600	-1.18261700
H	2.74172500	-2.21021400	-0.90315000
H	1.42581200	-2.72969100	0.16653700
C	-1.05865100	1.02295200	0.08773000
C	-2.21501200	1.02987300	-0.71156200
C	-1.05882900	1.97488300	1.13951500
C	-3.30589900	1.88679700	-0.54992600
C	-2.14052000	2.84681200	1.29601800
C	-3.26752400	2.83122000	0.47944200
C	1.67929700	0.58553000	-0.11374300
C	1.97315300	1.76590200	-0.83868500
C	2.77701600	-0.02552600	0.51379300
C	3.27684300	2.26789100	-0.85526800
C	4.08283300	0.46739000	0.49424100
C	4.34052100	1.65480300	-0.19832500
C	5.72695100	2.24143600	-0.24344900
H	6.44418900	1.54456200	-0.69207600
H	5.74364600	3.16386600	-0.82834300
H	6.09763200	2.47253000	0.76183900
C	-4.40057200	3.80011300	0.69349300

H -4.20552600 4.44664900 1.55189300
H -4.55043400 4.44078100 -0.18308100
H -5.34898100 3.28013800 0.87069200
C -1.97162200 -3.44013400 -0.84222600
H -2.48128500 -3.31024700 -1.79844400
H -0.92698700 -3.68864300 -1.02068200
H -2.46213400 -4.20956200 -0.24557700
C -3.42008400 -1.72699000 0.18210100
H -3.94964400 -1.59789100 -0.76406700
H -3.89616000 -2.50578900 0.77932100
H -3.38976100 -0.78271900 0.72339700
C 0.92976100 2.53017300 -1.62046000
H 0.30711700 1.85741400 -2.21463100
H 0.25753600 3.09165800 -0.96410100
H 1.41205800 3.23858900 -2.29968700
C 0.05106000 2.11162400 2.15753700
H -0.07949500 1.38675200 2.96875100
H 1.04013700 1.95414600 1.72641900
H 0.02799400 3.10785700 2.60767600
C -4.49611200 1.79715900 -1.47041500
H -5.41637100 1.56361600 -0.92134800
H -4.67435700 2.74441200 -1.99136700
H -4.34957600 1.02446300 -2.23034700
C 5.19251600 -0.26565700 1.20391500
H 5.99032700 -0.55761500 0.51119000
H 5.65887700 0.35755000 1.97575200
H 4.81753200 -1.17204600 1.68616600
H 3.47297100 3.18127000 -1.41551700
H 2.62587100 -0.95516400 1.05618300
H -2.10006900 3.57667900 2.10366900
H -2.29882800 0.32437600 -1.53279700

N3_TS
C 1.26941300 -2.47784500 0.98107500
C 0.30604800 -1.47172100 1.16239000
C -0.27752600 -1.40087500 2.43465400
C 0.05371000 -2.28455700 3.45458200
C 0.99314200 -3.28818100 3.23107900
C 1.60649700 -3.38172900 1.98832200
H -1.01500200 -0.62575700 2.62339800
H -0.42380300 -2.19400600 4.42518100
H 1.25449200 -3.98583100 4.01999100
H 2.35768500 -4.14692200 1.81182700
H 1.04639600 -1.78872100 -1.12850800
B -0.06023700 -0.42076400 -0.03074500
N 1.98015400 -2.52689300 -0.30096000
C -0.14300500 -1.34526900 -1.44720600
C -0.93460600 -2.52963500 -1.45293300
C -1.62209800 -2.64186300 -2.65200800
H -0.98708700 -3.20900200 -0.61232400
H -2.29226800 -3.42689600 -2.96763000
C -1.29010600 -1.50289100 -3.38554200
H -1.63350800 -1.19042400 -4.36341800
N -0.42640700 -0.74730500 -2.69828200
C -0.01318800 0.57377900 -3.15712200
H -0.03909400 1.27640200 -2.32345300
H 0.99059000 0.54579100 -3.57924800
H -0.72126100 0.90811000 -3.91609600
C -1.54461700 0.27024000 0.10651000
C -1.83520300 1.60117900 -0.18761700
C -2.66753300 -0.48714300 0.44729900
C -3.10883400 2.15427600 -0.13386200
C -3.95847400 0.02175700 0.51949900
C -4.18333000 1.35847400 0.22909200
C 1.16050800 0.68875400 -0.01044100
C 1.20509200 1.59212000 1.05309600
C 2.24199000 0.78963200 -0.87529900
C 2.20602700 2.53344100 1.23542200
C 3.27618400 1.71038700 -0.73545700
C 3.25683000 2.59495600 0.32904200
C 3.35814600 -2.02154800 -0.15236300
H 3.79826200 -1.86498100 -1.13874700
H 3.33766900 -1.07870400 0.39309800
H 3.96348000 -2.74084200 0.41303800
C 2.00318100 -3.85451300 -0.93047200
H 0.98463500 -4.22378800 -1.05086200
H 2.46529400 -3.75956500 -1.91653700
H 2.58002000 -4.57388400 -0.33939000
F 4.28386600 1.73193700 -1.61017000
F 4.23198800 3.48635900 0.48570700
F 2.17427100 3.37454600 2.26750200
F 0.22083900 1.59077300 1.96254200
F 2.37546100 -0.06160700 -1.91967100
F -0.86544300 2.45333300 -0.57527300
F -3.30196800 3.43933800 -0.43240400
F -5.41197600 1.86635500 0.28660700
F -4.97936800 -0.76472500 0.86177600
F -2.54889400 -1.79465600 0.72756800

N3_P

C	1.34216200	2.24624200	-1.25107700
C	0.24316700	1.38060600	-1.20232900
C	-0.48277900	1.31274300	-2.40358300
C	-0.15665400	2.05746500	-3.53021000
C	0.93607800	2.92057300	-3.51177500
C	1.70167700	3.00995700	-2.35856500
H	-1.33292300	0.63968600	-2.45104100
H	-0.75793300	1.96517400	-4.42904600
H	1.19707200	3.50852400	-4.38480300
H	2.57191900	3.65973700	-2.33522300
H	1.74567900	1.81232800	0.68932700
B	-0.12883200	0.42923900	0.10299900
N	2.25611900	2.29965200	-0.07468600
C	-0.16408500	1.32119900	1.46203000
C	-0.35129400	2.69396500	1.58639300
C	-0.51240100	3.01823400	2.95884900
H	-0.46542600	3.36765100	0.74596500
H	-0.68783500	3.99291200	3.39051100
C	-0.43847700	1.83091100	3.64488700
H	-0.51377100	1.61780700	4.70197400
N	-0.24025500	0.81816100	2.74626800
C	-0.14277000	-0.57585500	3.13788500
H	0.86129700	-0.81673100	3.49918000
H	-0.86622900	-0.77673500	3.93084000
H	-0.37480300	-1.21764700	2.28830900
C	-1.65202200	-0.19255200	-0.02532500
C	-2.03180100	-1.51434500	0.18795900
C	-2.72220100	0.67347200	-0.26042300
C	-3.34766600	-1.96249200	0.15091600
C	-4.04984200	0.27191600	-0.31664900
C	-4.36746500	-1.06237500	-0.11014700
C	1.04387200	-0.74418500	0.06277400
C	1.03406800	-1.69363100	-0.96061200
C	2.15123700	-0.83235400	0.89619400
C	2.00397100	-2.67126100	-1.12466000
C	3.15692400	-1.78632100	0.77351100
C	3.07913100	-2.72065600	-0.24502400
C	3.54269400	1.59654300	-0.35990600
H	4.06857000	1.42820600	0.58063400
H	3.31718000	0.65025500	-0.84723800
H	4.13305600	2.22500100	-1.02730400
C	2.51736400	3.67419500	0.44364300
H	3.09302300	3.58355900	1.36567800
H	3.08386300	4.23443300	-0.29876800
H	1.56336600	4.15165600	0.64982900
F	4.20367300	-1.77635000	1.60253700
F	4.02831600	-3.64111900	-0.39197700
F	1.92465100	-3.55568600	-2.11668300
F	0.03429200	-1.69442700	-1.85026000
F	2.36682300	0.09891100	1.86175500
F	-1.11582000	-2.45948200	0.47880800
F	-3.63389900	-3.24811600	0.36223800
F	-5.63380000	-1.47073200	-0.15864800
F	-5.01911600	1.15436400	-0.56327600
F	-2.49960000	1.98056200	-0.46376300

N4_TS

C	-2.90388300	-0.87641000	-0.35543000
C	-1.51955000	-1.08317500	-0.47394500
C	-1.10187200	-2.42039600	-0.47416500
C	-1.99724400	-3.47896800	-0.35810900
C	-3.36129000	-3.23113400	-0.22592800
C	-3.81938300	-1.91875500	-0.22445200
H	-0.04045600	-2.63503100	-0.56854500
H	-1.63077200	-4.50092100	-0.36284400
H	-4.06482500	-4.05158600	-0.12859100
H	-4.88193600	-1.71330200	-0.12961800
H	-2.15328300	1.13588400	0.08032900
B	-0.51905000	0.17799900	-0.61208300
N	-3.35598000	0.50810600	-0.38647500
C	-0.98351400	1.33883500	0.55317000
C	-0.99615200	1.11312600	1.95205700
C	-0.51301800	2.23982900	2.60893500
H	-1.32704300	0.18599000	2.40233100
H	-0.39680000	2.39509100	3.67111100
C	-0.19677400	3.16551000	1.61178500
H	0.20980400	4.16473000	1.70033300
N	-0.48384400	2.64600800	0.41243000
C	-0.22732200	3.29388900	-0.86236800
H	0.56485700	2.77188800	-1.40261500
H	-1.13095000	3.27868400	-1.47534000
H	0.06412400	4.32844900	-0.67646200
C	1.05649200	-0.14380500	-0.39153100
C	2.03606400	0.28385700	-1.28223600
C	1.54289500	-0.81548700	0.72795600
C	3.39631100	0.06401600	-1.09621600

C	2.89047000	-1.06168200	0.95616800
C	3.82611100	-0.61754100	0.03199500
C	-3.67567000	0.96596200	-1.74461600
H	-3.82211500	2.05041400	-1.73313600
H	-2.84401900	0.71844200	-2.40457400
H	-4.59075200	0.48396600	-2.11225900
C	-4.40464300	0.84598500	0.57516700
H	-4.10793400	0.48947800	1.56354500
H	-4.51322300	1.93366300	0.60588600
H	-5.37279100	0.40701600	0.30667800
F	1.70191400	0.96913600	-2.39130500
F	4.28879600	0.50118600	-1.98722900
F	5.12484600	-0.83883000	0.22886500
F	3.29437400	-1.71237500	2.04876800
F	0.69012800	-1.26504400	1.66281000
H	-0.67655400	0.71975600	-1.68410000

N4_P

C	-2.20753800	-1.19624000	0.13334300
C	-1.39065300	-0.83643800	-0.94180600
C	-1.52359500	-1.66792200	-2.06726200
C	-2.37878100	-2.76280100	-2.10313600
C	-3.16559100	-3.07613000	-0.99550800
C	-3.08090500	-2.28079100	0.13875800
H	-0.92352500	-1.42978900	-2.94126100
H	-2.43711300	-3.37535400	-2.99761200
H	-3.83673900	-3.92791900	-1.01368900
H	-3.68393800	-2.51201900	1.01349600
H	-1.49400300	0.43356200	1.12232200
B	-0.34834100	0.43572000	-1.00429200
N	-2.12271700	-0.37865300	1.36719700
C	-0.87538900	1.69138700	-0.12456200
C	-0.46696600	2.21487100	1.10125000
C	-1.37661800	3.24010800	1.49167300
H	0.42507300	1.90188400	1.63041800
H	-1.33001500	3.86077000	2.37489600
C	-2.32074600	3.32213300	0.49697900
H	-3.17275900	3.97729100	0.38037600
N	-2.02346700	2.38187800	-0.45757400
C	-2.75530700	2.19884700	-1.69751000
H	-3.04992000	1.15232400	-1.81759800
H	-3.64810500	2.82607500	-1.67653100
H	-2.13455500	2.47881900	-2.55128800
C	1.16515100	0.05107600	-0.51605000
C	2.19065400	0.96380700	-0.77050800
C	1.57854200	-1.10663700	0.12916900
C	3.51755400	0.75137300	-0.42394400
C	2.89525400	-1.36534700	0.49842400
C	3.87458300	-0.42874400	0.21601900
C	-3.41284700	0.25098000	1.75669400
H	-3.20673800	1.00302400	2.51991300
H	-3.83027200	0.73313300	0.87351600
H	-4.09391000	-0.51200400	2.13569800
C	-1.48102500	-1.09231800	2.50248700
H	-1.30903500	-0.37197900	3.30466800
H	-2.14405600	-1.88873600	2.84400000
H	-0.54179100	-1.51360100	2.14851500
F	1.91137700	2.11941200	-1.38400400
F	4.45379300	1.66181600	-0.69655900
F	5.14263000	-0.65041100	0.56104500
F	3.21662200	-2.50308400	1.12081400
F	0.69193200	-2.07498400	0.45762800
H	-0.28544900	0.76131500	-2.17312000

N5_TS

B	-0.37502400	-0.02457900	-0.06478700
C	0.62145300	-0.46510400	-1.30480600
C	-0.03634100	-0.80500000	-2.50179400
H	-1.11395900	-0.66286700	-2.55016300
C	0.60873500	-1.31462600	-3.61643100
H	0.04418600	-1.55757600	-4.51106700
C	1.98409600	-1.52090300	-3.56996300
H	2.51525200	-1.93463200	-4.42116200
C	2.67838800	-1.17392600	-2.42142700
H	3.75149600	-1.30837500	-2.39911300
C	2.01967900	-0.63960800	-1.30325400
N	2.80517300	-0.20194300	-0.14569100
C	3.65522100	-1.24253500	0.55513100
C	4.09258700	-0.61826400	1.89224000
H	3.19401600	-0.41454800	2.49390600
H	4.68387600	-1.35999900	2.44011700
C	4.86964600	0.67763700	1.70610100
H	5.79513700	0.48567900	1.14977200
H	5.16984400	1.08413700	2.67686500
C	3.98747900	1.68265400	0.97391500
H	4.53157900	2.61350500	0.77832200
H	3.14494900	1.93552700	1.62408100
C	3.45857200	1.15991100	-0.37338300

C	4.61282100	1.20212500	-1.39715300
H	5.55930800	0.81997300	-1.01570200
H	4.77927600	2.25355600	-1.65122400
H	4.35857100	0.68585500	-2.32331000
C	2.39672600	2.11919600	-0.92217600
H	1.44876200	2.03537900	-0.39582500
H	2.19223500	1.92906700	-1.97753600
H	2.76285700	3.14536200	-0.81948100
C	4.88822500	-1.77793800	-0.20402900
H	4.59169700	-2.49133600	-0.97507400
H	5.51126800	-2.32376900	0.51186700
H	5.50882800	-1.01301800	-0.66191000
C	2.79391000	-2.46447900	0.86071700
H	2.39704700	-2.91519700	-0.04991500
H	1.96785700	-2.19868500	1.51671300
H	3.40971300	-3.20663100	1.37748600
C	-1.07294600	1.46106000	-0.26485100
C	-0.95477200	2.31538500	-1.35555100
F	-0.23533200	1.98639100	-2.43861400
C	-1.53541000	3.58362800	-1.40782200
F	-1.36870600	4.35496100	-2.48319800
C	-2.28004400	4.04386500	-0.33855700
F	-2.83966100	5.25077300	-0.37044000
C	-2.43456200	3.22806900	0.77683900
F	-3.15516300	3.65399800	1.81548600
C	-1.84047600	1.98005400	0.78247100
F	-2.04021800	1.23221700	1.88139600
C	-1.55102900	-1.21507700	-0.02798000
C	-2.93860500	-1.04367000	0.00545000
F	-3.51819300	0.16015400	0.07512200
C	-3.85964700	-2.09046900	-0.05850600
F	-5.16569700	-1.83027000	-0.01039300
C	-3.42374200	-3.39519200	-0.18903200
F	-4.28831400	-4.40315100	-0.25093500
C	-2.05839200	-3.62923600	-0.25241200
F	-1.59936100	-4.87577200	-0.37196000
C	-1.18412100	-2.55682100	-0.18599000
F	0.11780600	-2.90059100	-0.22345400
H	1.60477300	-0.04349200	0.85310800
C	0.48590900	0.13790200	1.43889200
C	0.76671600	1.41449300	2.03016000
C	0.78569000	1.33753300	3.40812600
C	0.45835900	0.01570400	3.71105700
N	0.27917100	-0.68641200	2.59118700
H	0.96757600	2.30930100	1.45882200
H	0.99519300	2.12274300	4.11836700
H	0.33294300	-0.45581400	4.67761600
C	-0.28078600	-2.03129100	2.65709400
H	-0.02806100	-2.45633200	3.62975200
H	-1.36690500	-1.98349000	2.54861100
H	0.13090500	-2.66178400	1.87599100

N5_P

B	-0.45844400	-0.01883900	0.05955200
C	0.51780800	-0.52267100	-1.22104200
C	-0.19543400	-0.76302300	-2.41698100
H	-1.26087600	-0.55153700	-2.41887900
C	0.37113100	-1.23875100	-3.58583200
H	-0.24741900	-1.39641200	-4.46366400
C	1.73461400	-1.51761800	-3.62472900
H	2.20339800	-1.90884600	-4.52120900
C	2.49385200	-1.27297200	-2.49450900
H	3.55630300	-1.46965900	-2.52102000
C	1.89502900	-0.77130800	-1.33108700
N	2.80667500	-0.46468200	-0.19241800
C	3.47401900	-1.69425100	0.49029900
C	4.08768100	-1.17312900	1.79775000
H	3.27289600	-0.85955400	2.46469800
H	4.58898100	-2.01774000	2.28126700
C	5.04868200	-0.00618900	1.60071500
H	5.91464500	-0.31073200	1.00098900
H	5.44111500	0.31289400	2.57047900
C	4.31053300	1.15744900	0.94584300
H	4.98020600	2.00888700	0.78634800
H	3.51471900	1.49368000	1.61926100
C	3.69444800	0.80349400	-0.41645300
C	4.79585900	0.66000600	-1.46878300
H	5.60798200	-0.00567100	-1.18294400
H	5.22975100	1.65735100	-1.58440800
H	4.40506300	0.37176700	-2.44355000
C	2.76223500	1.91892800	-0.88879800
H	1.87129300	2.00347000	-0.26609100
H	2.43561600	1.76325700	-1.91777000
H	3.30843900	2.86463800	-0.83251500
C	4.51132200	-2.43119600	-0.36108800
H	4.04392400	-2.95749700	-1.19367800
H	4.95611800	-3.19108500	0.28780200
H	5.32412400	-1.81505200	-0.73915700

C	2.36407100	-2.68775200	0.81716300
H	1.58812600	-2.22476800	1.42801100
H	2.80667600	-3.51020100	1.38653900
H	1.90907900	-3.09363600	-0.08678900
C	-0.89067300	1.57291900	-0.15497000
C	-0.57430800	2.41396400	-1.21705100
F	0.11016300	1.97970200	-2.29184900
C	-0.90203500	3.76941500	-1.26193200
F	-0.54141100	4.51389100	-2.31081100
C	-1.59746500	4.34174900	-0.21449700
F	-1.91999800	5.63340600	-0.23900700
C	-1.95506600	3.54620600	0.86755100
F	-2.63716100	4.07843100	1.88260200
C	-1.60248600	2.20773300	0.87077800
F	-2.00886400	1.50056500	1.93272600
C	-1.79792800	-1.01087500	-0.04772200
C	-3.14113800	-0.63181400	-0.06685400
F	-3.52500000	0.64828900	0.00921200
C	-4.20062000	-1.53168400	-0.18924500
F	-5.45687100	-1.08695400	-0.19810200
C	-3.95173500	-2.88590100	-0.32004900
F	-4.95326200	-3.75404700	-0.43746600
C	-2.63415300	-3.31984300	-0.32930900
F	-2.35634100	-4.61955200	-0.45090500
C	-1.61818500	-2.38636800	-0.20821200
F	-0.37201000	-2.90343700	-0.20582300
H	2.15460200	-0.14311700	0.55439600
C	0.21197800	-0.02834400	1.57182800
C	1.00244900	0.99506100	2.09646000
C	1.33217900	0.71186800	3.44626600
C	0.72509200	-0.48151000	3.73715100
N	0.05290900	-0.92069000	2.62321600
H	1.22212700	1.92091800	1.58342300
H	1.91595800	1.31750600	4.12454500
H	0.69356100	-1.05505000	4.65227200
C	-0.79951400	-2.09786800	2.69539500
H	-0.72518800	-2.50269600	3.70529200
H	-1.83947100	-1.83026800	2.50067200
H	-0.48325300	-2.86605900	1.98880500

N6_TS

C	-1.42174700	0.48244300	-0.00613700
C	-1.07368400	-0.59153700	0.83011700
C	-2.02670600	-1.61516500	0.92612100
C	-3.23806600	-1.57443100	0.24073200
C	-3.53482300	-0.49749500	-0.59163500
C	-2.61786800	0.54089400	-0.71914500
H	-1.79413500	-2.47439100	1.55055700
H	-3.94928700	-2.38872300	0.34373400
H	-4.47209800	-0.46359500	-1.13781000
H	-2.83865900	1.38714000	-1.36397500
H	0.74452500	0.74817300	0.19499300
B	0.35950700	-0.66928000	1.58208400
N	-0.43053700	1.53833600	-0.11327400
H	0.63364100	-1.81105000	1.88560000
C	1.60108500	-0.12682600	0.52628000
C	2.81309900	0.47421800	0.93155600
C	3.85357200	0.02959100	0.11724800
H	2.88499800	1.16979500	1.75775600
H	4.89842200	0.29850600	0.16219500
H	0.42432300	0.08283100	2.53345600
C	3.27949700	-0.85332900	-0.79692000
H	3.74070200	-1.42307700	-1.59341200
N	1.96082900	-0.93249400	-0.56964400
C	1.04119000	-1.82169100	-1.25523700
H	1.58112500	-2.35432700	-2.03922700
H	0.62118500	-2.53272700	-0.53967100
H	0.21739100	-1.25570300	-1.69714900
C	-0.28464100	2.15829000	-1.42705700
H	0.64998100	2.72622700	-1.44217500
H	-0.24145600	1.37934200	-2.19107900
H	-1.11141000	2.84197200	-1.65874500
C	-0.53217900	2.53406000	0.96021500
H	0.36603800	3.15932200	0.95253700
H	-1.41765200	3.16751800	0.81974900
H	-0.59957400	2.01413500	1.91584300

N6_P

C	-1.11546300	-1.01535800	0.18593600
C	-1.56443500	0.30741700	0.12799200
C	-2.85586700	0.70583200	-0.19879600
C	-3.78633300	-0.27832700	-0.51149000
C	-3.39670200	-1.61644800	-0.48610700
C	-2.09360500	-1.96631400	-0.14615900
H	-3.14463400	1.75291900	-0.21501400
H	-4.80155100	-0.00095800	-0.77428900
H	-4.11747300	-2.39005900	-0.73400400
H	-1.80547100	-3.01336900	-0.13049700

N	-0.55625700	1.33779700	0.49340900
B	0.39465200	-1.41834100	0.64930800
H	0.35607700	0.85889200	0.27371200
H	0.53943100	-2.62277800	0.57544900
H	0.53286600	-1.08680100	1.82680500
C	1.50571200	-0.63161800	-0.23852600
C	1.43360500	0.06315300	-1.44628500
C	2.72301500	0.57097600	-1.77051300
H	0.54074200	0.12618900	-2.05866500
C	3.55991900	0.17614900	-0.75611300
H	3.00913600	1.13345300	-2.64770200
H	4.61827600	0.33710600	-0.60591000
N	2.82385200	-0.53358100	0.15694100
C	3.34994300	-1.11605800	1.37377900
H	3.05545400	-0.53489000	2.25327800
H	2.96356700	-2.12985700	1.49095400
H	4.43933800	-1.14636600	1.31235200
C	-0.57532700	2.56128600	-0.34429300
H	0.32663400	3.13537400	-0.12862900
H	-0.58025000	2.26058600	-1.39072600
H	-1.45884800	3.15488300	-0.10979800
C	-0.59952600	1.65080800	1.94695900
H	0.22577000	2.32259100	2.19011700
H	-1.55713300	2.12530500	2.16775100
H	-0.49798200	0.71098400	2.48751400

N7_TS

C	4.64814900	-2.00178300	0.91651400
C	5.30039500	-0.95858500	-0.03964300
C	4.13147300	1.04059400	-1.28376700
C	2.88578800	1.67735300	-1.49745600
C	1.70824100	1.20687800	-0.95475000
C	0.52698800	-0.62326100	0.44300000
C	0.80230100	-1.70622400	1.26603200
C	2.09228800	-2.24430500	1.51351900
C	4.15521300	-0.07327400	-0.48701100
C	1.67048600	0.01791500	-0.15893000
C	3.16898600	-1.67665400	0.88976400
C	2.94616400	-0.55408200	0.06306800
C	-0.07214900	1.79355600	-2.51395600
C	0.59920200	3.38337600	-0.80505700
C	-2.57861600	-2.04913700	0.03787700
C	-2.63669200	-1.21363000	-1.28361800
C	-3.93786200	-2.44454200	0.60252400
C	-1.71930100	-3.30819600	-0.12669900
C	-2.56245200	-2.03914100	-2.56365600
C	-3.87954300	-0.32116600	-1.33704800
O	-1.94907400	-1.15433400	0.94968300
O	-1.46319600	-0.40932700	-1.20139100
B	-1.06691100	-0.29183300	0.19342000
N	0.48520600	1.96293900	-1.16191900
H	5.05025900	-1.91753300	1.93105700
H	4.83840900	-3.02723300	0.58598900
H	6.08108700	-0.37860900	0.46154900
H	5.77167300	-1.44478700	-0.89960600
H	5.03250700	1.44875000	-1.73121600
H	2.85907500	2.57931800	-2.10301400
H	-0.04776100	-2.17988400	1.74807200
H	2.20240700	-3.09802100	2.17721000
H	-1.06569800	2.25120800	-2.53988100
H	0.57486500	2.28039900	-3.25526100
H	-0.17571900	0.73028300	-2.71936800
H	-0.41153500	3.80068900	-0.75213200
H	1.18475100	3.94918300	-1.53781700
H	1.06791600	3.46704700	0.17749400
H	-3.79330600	-3.03962000	1.50861800
H	-4.50198700	-3.04841500	-0.11668900
H	-4.52565200	-1.56455500	0.86670800
H	-1.55999700	-3.76023600	0.85671600
H	-2.21306500	-4.04665300	-0.76627000
H	-0.74313000	-3.06618400	-0.55492100
H	-2.64200000	-1.37556700	-3.43025200
H	-3.38325200	-2.76275500	-2.61349200
H	-1.61336600	-2.57328500	-2.63049200
H	-3.75046200	0.41466200	-2.13782400
H	-4.77967000	-0.90595200	-1.55152300
H	-4.02467100	0.20713300	-0.39275900
H	-0.47118800	1.57566600	-0.18810000
C	-1.39015300	1.39881000	0.68069800
C	-2.49539800	2.18250700	0.29095500
C	-2.87998300	3.01351800	1.34738900
C	-2.00542900	2.73584700	2.39100600
N	-1.11612600	1.80762700	1.99211700
H	-2.94132900	2.14166500	-0.69346300
H	-3.68576600	3.73213600	1.37140100
H	-1.96662500	3.14107700	3.39358500
C	-0.10560200	1.22887100	2.86252800
H	-0.08345400	1.79939500	3.79235200

H	-0.34522500	0.18459000	3.07276700
H	0.87609600	1.26910300	2.38603600

N7_P

C	4.64790400	-1.82424300	0.93931100
C	5.21935400	-0.84239800	-0.12618700
C	3.91503600	0.92632100	-1.56133800
C	2.63390700	1.47031800	-1.80858300
C	1.50987200	1.02431500	-1.15046300
C	0.46242300	-0.61776500	0.56259600
C	0.81476000	-1.62813300	1.44607900
C	2.13140000	-2.10386500	1.66958900
C	4.01723900	-0.06895000	-0.62533300
C	1.54394800	-0.02500300	-0.17731700
C	3.15908700	-1.54753500	0.95726500
C	2.85565600	-0.51988600	0.03877900
C	-0.17272200	1.52911100	-2.89349300
C	0.26689900	3.14611000	-1.05842500
C	-2.50329400	-2.10410100	0.00410900
C	-2.66248000	-1.09079600	-1.17625100
C	-3.81262100	-2.72437000	0.47877400
C	-1.51041900	-3.22091500	-0.34422900
C	-2.59485100	-1.71672100	-2.56490700
C	-3.94591400	-0.26763100	-1.04531400
O	-1.98552900	-1.29558000	1.04553000
O	-1.53221300	-0.24463300	-1.00500000
B	-1.15232800	-0.24969800	0.45352600
N	0.24233800	1.71428200	-1.47535300
H	5.09533300	-1.65125900	1.92285100
H	4.85546800	-2.86598000	0.67714200
H	5.96747700	-0.16820500	0.30166000
H	5.70778800	-1.37544500	-0.94750900
H	4.77648900	1.30413000	-2.10220500
H	2.54335700	2.26913900	-2.53962700
H	-0.00072400	-2.09181700	1.99584700
H	2.30391500	-2.90116900	2.38780600
H	-1.16772500	1.96167300	-3.00854200
H	0.54182600	2.02296800	-3.55174800
H	-0.21804800	0.45740400	-3.07720100
H	-0.71260200	3.58012300	-1.25248600
H	1.04866000	3.66250600	-1.61533500
H	0.45856000	3.18141400	0.01461000
H	-3.60560200	-3.44169000	1.27791800
H	-4.31829200	-3.25612100	-0.33504400
H	-4.48199600	-1.95989000	0.87627700
H	-1.26596600	-3.76985100	0.56980400
H	-1.93419400	-3.92687800	-1.06617000
H	-0.58257800	-2.81101900	-0.75154900
H	-2.74390000	-0.94346900	-3.32629800
H	-3.37687300	-2.47221400	-2.69661300
H	-1.62293900	-2.18467500	-2.73476900
H	-3.91410500	0.55518200	-1.76795000
H	-4.83471800	-0.87035100	-1.25712800
H	-4.02893500	0.15506200	-0.04087900
H	-0.52630400	1.26295100	-0.94542300
C	-1.47061700	1.23861300	1.06720500
C	-2.25966700	2.24685700	0.52230800
C	-2.20918900	3.38888700	1.36664700
C	-1.38893700	3.05523100	2.41730200
N	-0.94705700	1.77079300	2.22841600
H	-2.82233700	2.15039500	-0.39785600
H	-2.72195100	4.33152200	1.23660000
H	-1.09880000	3.61312700	3.29670700
C	-0.11000300	1.06254400	3.17914000
H	-0.09316700	1.62406300	4.11509500
H	-0.52331800	0.06972500	3.36721600
H	0.91219300	0.94736000	2.80792400

N8_TS

C	4.18902200	-2.96200600	-2.22138400
H	4.35934800	-2.77525100	-3.28642500
H	4.17126400	-4.04791400	-2.08981300
C	5.28383800	-2.29021600	-1.34598900
H	5.78994400	-3.01952700	-0.70563000
H	6.05822600	-1.81222300	-1.95338500
C	4.98972000	-0.39579500	0.43434100
H	6.03890200	-0.32075300	0.70268600
C	4.03667800	0.43675300	1.06120900
H	4.39667200	1.15752500	1.78815900
C	2.68611800	0.39319300	0.78213400
C	0.76613800	-0.79601000	-0.54766800
C	0.58282000	-1.80926200	-1.48211600
H	-0.43798100	-2.04920200	-1.76347200
C	1.59604900	-2.56899800	-2.10864700
H	1.32675800	-3.33244900	-2.83359800
C	4.53759800	-1.27279000	-0.51028700
C	2.14609800	-0.53285000	-0.18333100
C	2.89508900	-2.32667100	-1.76371900

C	3.15270700	-1.33080100	-0.79725000
C	2.12487800	1.56254800	2.88153200
H	2.12672300	0.59518700	3.38373300
H	1.33554700	2.18628900	3.30964900
H	3.08418800	2.06230300	3.04941300
C	2.04632900	2.68202600	0.76021500
H	3.10407200	2.95956600	0.84995200
H	1.43188300	3.45157400	1.22600300
H	1.77819900	2.59456200	-0.29248000
C	-0.62260000	1.55761400	-0.43195000
C	-0.83782200	2.73404900	0.33549300
C	-0.83617900	4.00932900	-0.25427900
H	-1.01729500	4.87303600	0.38505800
C	-0.58396600	4.21962400	-1.59625400
C	-0.37495900	3.08017400	-2.36608400
H	-0.20535900	3.19199800	-3.43601400
C	-0.42287200	1.79486600	-1.82956100
C	-1.07536600	2.84445300	1.83836400
H	-0.77072400	1.98934300	2.43073600
H	-2.13424600	3.03158400	2.04926800
H	-0.53687100	3.71515700	2.22793600
C	-0.55474800	5.59664300	-2.20503900
H	-1.32659800	5.70431400	-2.97348400
H	0.40897200	5.79528500	-2.68437300
H	-0.72205800	6.36761000	-1.44904700
C	-0.35006200	0.69678400	-2.87245400
H	-0.65155600	1.10191800	-3.84255200
H	-1.02114200	-0.13068800	-2.63595300
H	0.65632100	0.28580000	-2.97991000
C	-2.02203400	-0.88814100	-0.28381800
C	-3.18479400	-0.31452900	-0.88644700
C	-4.32744500	-1.08634400	-1.13990500
H	-5.18478000	-0.60313300	-1.60417800
C	-4.41992000	-2.43205500	-0.82330800
C	-3.31565200	-2.99347900	-0.20127600
H	-3.35149900	-4.04223700	0.09006500
C	-2.15534000	-2.26421500	0.07600600
C	-3.37448700	1.14126100	-1.28362800
H	-2.78948200	1.41961400	-2.16239400
H	-3.10180000	1.84899600	-0.49956800
H	-4.42636700	1.30881200	-1.52670600
C	-5.64577500	-3.24359300	-1.15017500
H	-6.53998300	-2.61565200	-1.18257200
H	-5.80720900	-4.03261500	-0.41070400

H	-5.54695400	-3.72627100	-2.12846200
C	-1.12140500	-3.07478400	0.83559400
H	-1.24218400	-4.13507100	0.59627400
H	-1.27682800	-2.96823400	1.91421200
H	-0.09321500	-2.80163200	0.62229300
B	-0.59905300	-0.05411100	0.06331900
N	1.83241500	1.38420700	1.44553900
C	-0.35900100	-0.26956100	1.79272600
C	0.52218400	-1.13298000	2.49430500
C	-0.00964200	-1.46937100	3.73931900
C	-1.25983600	-0.86915900	3.78918100
N	-1.46596900	-0.15878700	2.66731700
H	1.49122000	-1.43800500	2.12242500
H	0.43520200	-2.08674900	4.50513600
H	-2.03101300	-0.92927900	4.54677500
C	-2.78524000	0.36504100	2.33428400
H	-3.16348600	0.95054800	3.17618100
H	-2.71577900	0.99313700	1.45152300
H	-3.46500700	-0.46017700	2.10572900
H	0.49941500	0.69101300	1.58012800

N8_P

C	-4.15379300	3.39290800	-1.65565500
H	-4.36143200	3.41745600	-2.73016800
H	-4.07856000	4.43228700	-1.32361800
C	-5.25901300	2.61677600	-0.88476200
H	-5.67977500	3.22099500	-0.07482400
H	-6.09271600	2.33021300	-1.53219700
C	-5.05260900	0.33271000	0.37130500
H	-6.10516500	0.23453900	0.61485000
C	-4.14332200	-0.68793100	0.73082600
H	-4.53351600	-1.58080100	1.20843900
C	-2.79939900	-0.59161300	0.46212100
C	-0.78783000	0.81240800	-0.46294500
C	-0.59626500	1.93538500	-1.26861900
H	0.42344400	2.18367400	-1.54079400
C	-1.58455100	2.82791700	-1.74006100
H	-1.29021800	3.67399000	-2.35498100
C	-4.55544500	1.40146400	-0.32100100
C	-2.18685600	0.53262900	-0.19143500
C	-2.88417100	2.62224300	-1.37086500
C	-3.16977600	1.47607200	-0.60465000
C	-2.17938900	-2.47201900	2.02053300
H	-1.97496300	-1.74550100	2.80621900

H	-1.46449600	-3.29422900	2.07017000
H	-3.19303400	-2.86400100	2.08489800
C	-2.20038200	-2.77182100	-0.41896100
H	-3.22499100	-3.13857500	-0.34968500
H	-1.47349200	-3.57937300	-0.32530800
H	-2.04619800	-2.24102800	-1.35644700
C	0.73536100	-1.52773500	-0.40852600
C	0.97875600	-2.70066100	0.36935900
C	1.07180700	-3.96711300	-0.23594500
H	1.28021600	-4.83012900	0.39469500
C	0.91896100	-4.16347500	-1.60024800
C	0.65390400	-3.03304700	-2.36651200
H	0.53072000	-3.14321700	-3.44310600
C	0.56859500	-1.75753200	-1.80789600
C	1.18236700	-2.75043100	1.87954200
H	0.50744600	-2.11441800	2.45230800
H	2.19701800	-2.45053000	2.15415400
H	1.05799400	-3.78137100	2.22495300
C	1.05778800	-5.52558200	-2.22872700
H	0.30697700	-5.68177200	-3.00836100
H	0.95075800	-6.31915800	-1.48499900
H	2.04103700	-5.64036400	-2.69646700
C	0.35438600	-0.63843700	-2.80545800
H	0.64344100	-0.97484300	-3.80510400
H	0.94896200	0.24149500	-2.55439700
H	-0.69047900	-0.31627100	-2.84864600
C	1.97072700	1.01892200	-0.21456200
C	3.12849600	0.61522800	-0.94017400
C	4.17224800	1.51301800	-1.20088600
H	5.03164200	1.15963000	-1.76879900
C	4.16452700	2.82683700	-0.75765800
C	3.07506200	3.21466900	0.00884200
H	3.04810300	4.22648400	0.41027100
C	2.01506100	2.35053000	0.30217600
C	3.41828400	-0.79217800	-1.43653400
H	2.86445000	-1.05129400	-2.34196800
H	3.18815300	-1.56304500	-0.69859500
H	4.48144700	-0.87296500	-1.67689400
C	5.30541300	3.76472500	-1.05662300
H	6.20027600	3.49534500	-0.48556800
H	5.04613800	4.79535200	-0.80188800
H	5.57591400	3.73454900	-2.11632100
C	0.99087700	2.94811000	1.25396400
H	1.04684100	4.03979700	1.21177500
H	1.20553000	2.64542400	2.28639000
H	-0.03445400	2.65309200	1.04905300
B	0.63000400	0.06820000	0.16717600
N	-1.98881700	-1.80268400	0.70225800
C	0.50209700	0.14889300	1.80235500
C	-0.59682100	0.35596500	2.63190400
C	-0.17197400	0.37985900	3.98753800
C	1.18828900	0.20140000	3.96551600
N	1.58764800	0.07476900	2.66029200
H	-1.60280400	0.56341500	2.29566500
H	-0.78087100	0.53348800	4.86727900
H	1.91286000	0.16031600	4.76663400
C	2.99084000	-0.06851800	2.31191500
H	3.50410200	-0.56365700	3.14074100
H	3.09810400	-0.67483000	1.41315000
H	3.45542700	0.90330300	2.12010900
H	-0.99518500	-1.51250500	0.65849300

N9_TS

C	4.56253500	-0.25748300	-1.16732000
C	3.50689100	0.62220000	-1.47627600
C	2.28153300	0.50350900	-0.86417400
C	0.70465200	-0.77138600	0.68093000
C	0.65701900	-1.73082700	1.67557400
C	1.76721600	-2.50711200	2.07726300
C	4.36699300	-1.21699500	-0.21206400
C	1.99736700	-0.53382000	0.07992200
C	2.96687700	-2.35062200	1.44312700
C	3.11169000	-1.36675800	0.43160700
C	0.76909400	1.48390300	-2.51849800
C	1.73813900	2.87394500	-0.78013100
C	-2.54994700	-1.66545700	0.06581400
C	-2.32093100	-1.00789200	-1.33464100
C	-4.00880500	-1.75146500	0.49728700
C	-1.91830800	-3.05987600	0.14831500
C	-2.26605300	-1.99094400	-2.49891600
C	-3.36800800	0.06664300	-1.63749900
O	-1.86622900	-0.77697100	0.94581200
O	-1.03524800	-0.40649400	-1.19607600
B	-0.76922300	-0.18621200	0.21382600
N	1.28219800	1.52279200	-1.14054200
H	5.52145700	-0.14944100	-1.66285300
H	3.66941400	1.42133100	-2.19427700
H	-0.30443400	-1.91451300	2.14546500

H	1.65158100	-3.24688400	2.86390200
H	-0.09471600	2.15295700	-2.58064100
H	1.53544400	1.81977600	-3.22881000
H	0.44402200	0.47016700	-2.74105800
H	0.87051000	3.54165200	-0.79929100
H	2.50156400	3.24817600	-1.47080800
H	2.14697800	2.85232500	0.23225500
H	-4.07022500	-2.24379300	1.47182900
H	-4.59541900	-2.33742700	-0.21864100
H	-4.45044700	-0.75859000	0.59197400
H	-1.93937200	-3.39786100	1.18849900
H	-2.47187500	-3.78398300	-0.45775400
H	-0.87743100	-3.04191300	-0.18432900
H	-2.13340200	-1.44019600	-3.43520400
H	-3.19621200	-2.56487100	-2.57249200
H	-1.42945500	-2.68284200	-2.39052000
H	-3.02870400	0.65761600	-2.49488500
H	-4.33419300	-0.38093100	-1.89119200
H	-3.50647300	0.73196000	-0.78304100
H	0.17655700	1.45341900	-0.24556300
C	-0.83943100	1.58584900	0.49574900
C	-1.73665400	2.49097500	-0.10386000
C	-2.07813500	3.48927900	0.81423100
C	-1.38568500	3.19151400	1.98129800
N	-0.63873500	2.08830700	1.78586800
H	-2.08037500	2.40998100	-1.12581600
H	-2.74220400	4.32831700	0.66886100
H	-1.38498800	3.69528000	2.93884700
C	0.14789900	1.44757200	2.82621200
H	0.16128700	2.09907200	3.70121800
H	-0.29342500	0.48371300	3.08757800
H	1.17053200	1.27713300	2.48299900
H	5.17501800	-1.88426400	0.07532800
H	3.82698100	-2.96276900	1.70041700

N9_P

C	4.41936700	-0.43829500	-1.27056100
C	3.34257500	0.38150200	-1.65706300
C	2.14009600	0.31117900	-0.99953500
C	0.61937300	-0.74105800	0.76909400
C	0.60586700	-1.63717000	1.82333500
C	1.72567800	-2.38561400	2.24430700
C	4.25301500	-1.28598600	-0.21064000
C	1.88201000	-0.58148300	0.08947800
C	2.91117500	-2.26660100	1.57483200
C	3.01780800	-1.37332500	0.48074300
C	0.71976300	1.11551000	-2.86772000
C	1.51290500	2.68359900	-1.11202000
C	-2.53888000	-1.67075800	0.04466200
C	-2.36919200	-0.83431400	-1.26576100
C	-3.98803200	-1.93354000	0.43776200
C	-1.78349700	-3.00287400	-0.03764400
C	-2.31379300	-1.66053400	-2.54574900
C	-3.44992500	0.24151300	-1.39285700
O	-1.95300900	-0.83334400	1.02722700
O	-1.10181900	-0.21680200	-1.07199800
B	-0.87198300	-0.07161400	0.40393800
N	1.11667000	1.28295600	-1.44127700
H	5.36296700	-0.37771600	-1.80092200
H	3.47781500	1.08325200	-2.47450800
H	-0.34384800	-1.78843600	2.32866400
H	1.63410600	-3.06982100	3.08283300
H	-0.13711800	1.76590100	-3.04882000
H	1.55432500	1.38932800	-3.51277400
H	0.42331900	0.07737700	-3.00054800
H	0.70394500	3.34813600	-1.41050900
H	2.44138900	2.92021400	-1.63079800
H	1.63940100	2.74882300	-0.03057400
H	-4.01332500	-2.54200700	1.34623500
H	-4.52093800	-2.47518500	-0.35165700
H	-4.50980400	-0.99714000	0.64294700
H	-1.74265500	-3.44707600	0.96117300
H	-2.28517300	-3.70784100	-0.70875100
H	-0.75781700	-2.85308000	-0.38472600
H	-2.21580000	-0.99438800	-3.40975700
H	-3.22918900	-2.24749600	-2.67687100
H	-1.45866800	-2.33952400	-2.53528800
H	-3.18033700	0.91866100	-2.21059500
H	-4.42792000	-0.19387800	-1.62088200
H	-3.51985200	0.82222100	-0.46971400
H	0.24155200	1.11251900	-0.91841000
C	-0.94880400	1.52266700	0.78514800
C	-1.40745800	2.57880900	0.00382100
C	-1.25088300	3.79425300	0.72351400
C	-0.70278200	3.45561100	1.93726800
N	-0.51900900	2.09701500	1.96344300
H	-1.83125100	2.46284700	-0.98627400
H	-1.52606000	4.79054400	0.40742500

H	-0.44664300	4.06173400	2.79500800
C	-0.03337000	1.36849700	3.12076900
H	-0.06904100	2.02699400	3.99055100
H	-0.67457400	0.50356700	3.30223800
H	0.99227200	1.01694000	2.97642300
H	3.78355700	-2.84895900	1.85738800
H	5.07218300	-1.91659700	0.12371000

N10_TS

C	4.71234300	-2.33221800	-0.56689000
H	5.72789000	-2.68972900	-0.43609400
C	4.16808100	-1.40298400	0.33870500
H	4.79820200	-1.03997600	1.14280600
C	2.88423000	-0.92310300	0.21568500
C	0.59531200	-1.01685100	-1.00159100
C	-0.04333600	-1.60937400	-2.08011100
H	-1.09832300	-1.40227500	-2.22317200
C	0.55917000	-2.47360500	-3.01200100
H	-0.02815200	-2.87338000	-3.83302500
C	3.93952500	-2.74758000	-1.60983800
C	1.99969700	-1.36264200	-0.83816100
C	1.86996700	-2.81360800	-2.85379900
C	2.60408100	-2.29153900	-1.76277500
C	2.94173500	-0.10640200	2.54438100
H	2.66566200	-1.11197900	2.86192600
H	2.44221300	0.62208500	3.18751000
H	4.02215900	0.02892100	2.65519600
C	3.08809300	1.39679600	0.67837400
H	4.17757600	1.27612800	0.63374000
H	2.84320400	2.20443100	1.36684500
H	2.70317800	1.63982900	-0.31182000
C	0.11162300	1.55934600	-0.17478700
C	0.39323300	2.50891200	0.84406200
C	0.77800800	3.82579400	0.53886600
H	0.97165300	4.51024100	1.36449300
C	0.95297900	4.28076500	-0.75343400
C	0.67745300	3.36754600	-1.76610700
H	0.76842200	3.68857900	-2.80265500
C	0.24224500	2.06970900	-1.50677400
C	0.36307400	2.30782800	2.35600900
H	0.35832400	1.28106200	2.70192400
H	-0.51157600	2.80509300	2.79057900
H	1.23614000	2.79552700	2.80378800
C	1.39194300	5.68792800	-1.06086800
H	2.29834900	5.69362400	-1.67400400
H	1.59850900	6.24731700	-0.14526200
H	0.61992200	6.22731900	-1.61856100
C	-0.16693200	1.30576200	-2.75056200
H	-0.39185900	2.01573300	-3.55125800
H	-1.06215800	0.70595000	-2.57932400
H	0.61756100	0.63568200	-3.10929400
C	-2.02135700	-0.28386400	-0.28901700
C	-2.97133300	0.75711200	-0.53698300
C	-4.33437000	0.47209500	-0.69973100
H	-5.01672300	1.29874900	-0.88708100
C	-4.85682500	-0.80934100	-0.62826200
C	-3.95340400	-1.82564700	-0.36258700
H	-4.31926600	-2.84804700	-0.27939000
C	-2.58554500	-1.59244200	-0.18931800
C	-2.68417200	2.24868200	-0.62116400
H	-2.14628200	2.52304000	-1.53077600
H	-2.09141300	2.63254600	0.20991800
H	-3.63173000	2.79217400	-0.62915800
C	-6.32147200	-1.08406000	-0.84635600
H	-6.52806800	-1.29696000	-1.90074000
H	-6.93287300	-0.22446000	-0.55950900
H	-6.65249800	-1.94995900	-0.26668800
C	-1.82363300	-2.86006400	0.15557500
H	-2.29936200	-3.71323900	-0.33670500
H	-1.86763800	-3.04352500	1.23423800
H	-0.77973300	-2.85210300	-0.13554600
B	-0.36977500	-0.04898300	-0.02990900
N	2.48655000	0.12810000	1.15868500
C	-0.08984200	-0.75157100	1.56352800
C	0.47791000	-1.99881300	1.92806200
C	-0.04957800	-2.43972800	3.14228800
C	-0.99775400	-1.49468700	3.50621100
N	-1.01701400	-0.50066900	2.60181600
H	1.24862300	-2.50180900	1.35937500
H	0.20139300	-3.33757500	3.68660200
H	-1.68116300	-1.48150600	4.34565400
C	-2.07945300	0.49783800	2.59975200
H	-2.15889900	0.93989500	3.59616200
H	-1.85247300	1.27190200	1.87301600
H	-3.02548300	0.03227600	2.31117800
H	1.01641600	-0.09582400	1.38675700
H	2.36827100	-3.49629600	-3.53628500
H	4.32393200	-3.45250900	-2.34159900

N10_P

C	4.36247100	-3.00918000	-0.30925700
H	5.32152100	-3.48107800	-0.12966200
C	4.09952800	-1.72453900	0.20765700
H	4.88382800	-1.19988300	0.74119000
C	2.87818200	-1.13801400	0.01033700
C	0.43693600	-1.20753200	-0.82958900
C	-0.33564300	-1.88845800	-1.76368700
H	-1.34053300	-1.53089300	-1.95330100
C	0.03710700	-3.06298200	-2.44458400
H	-0.66238000	-3.51954600	-3.13810000
C	3.40489500	-3.60789300	-1.07500100
C	1.78485200	-1.74208600	-0.69743000
C	1.24110400	-3.64853100	-2.17877600
C	2.14336600	-2.99658700	-1.30935200
C	3.34774600	0.65107600	1.72129900
H	2.91920400	0.00651200	2.48812700
H	3.08181200	1.69227900	1.90530000
H	4.43105300	0.55620400	1.66988500
C	3.29744200	1.14781600	-0.68191500
H	4.37494500	0.98505300	-0.72557400
H	3.05145200	2.18570800	-0.45309200
H	2.82853200	0.85005400	-1.61790400
C	0.19727200	1.53334400	-0.21461500
C	0.60749500	2.50403800	0.75056900
C	1.10134500	3.75943800	0.35132400
H	1.39020000	4.47230200	1.12185800
C	1.21880700	4.13683300	-0.97826500
C	0.83397600	3.19670800	-1.92869200
H	0.90592900	3.45414200	-2.98434400
C	0.34260400	1.93959600	-1.57626800
C	0.54400700	2.34968100	2.26613300
H	0.87339500	1.38197200	2.64310900
H	-0.47418700	2.49746800	2.63469800
H	1.15884900	3.12607800	2.73142300
C	1.71034700	5.50360300	-1.37758900
H	2.39190800	5.44764700	-2.23097500
H	2.23203400	5.99504700	-0.55280900
H	0.87438100	6.14657800	-1.67131000
C	-0.07399300	1.06958700	-2.74287500
H	-0.22697200	1.68956700	-3.63045300
H	-1.00226300	0.53605800	-2.53386400
H	0.67915900	0.31452800	-2.98774800
C	-2.06131600	-0.18426800	-0.19564600
C	-2.94610500	0.82104200	-0.67256800
C	-4.30321700	0.54550100	-0.90379300
H	-4.93845700	1.34719900	-1.27627300
C	-4.87066400	-0.69404800	-0.66383000
C	-4.03453700	-1.66437900	-0.12323300
H	-4.45323100	-2.63730000	0.12953400
C	-2.68102400	-1.43406800	0.12870000
C	-2.59290400	2.28222300	-0.90330100
H	-2.07686600	2.45429900	-1.85126800
H	-1.95735700	2.69973300	-0.12097200
H	-3.51395300	2.86992800	-0.92967700
C	-6.31958800	-0.97956000	-0.96046800
H	-6.77372900	-1.58621700	-0.17182800
H	-6.42784100	-1.53294700	-1.89953700
H	-6.89432200	-0.05463400	-1.05381500
C	-1.98686600	-2.59015000	0.83141200
H	-2.54369000	-3.51403400	0.65089400
H	-1.96692600	-2.42081200	1.91486100
H	-0.95748500	-2.74970600	0.52230700
B	-0.41272900	-0.02182600	0.11156200
N	2.75273400	0.27701800	0.40742600
C	-0.23290900	-0.45565200	1.68599400
C	0.69777900	-1.26826400	2.32893900
C	0.39400700	-1.33684700	3.71475200
C	-0.73148900	-0.57482200	3.89790000
N	-1.10905100	-0.05959800	2.68488400
H	1.47544200	-1.83931000	1.84378400
H	0.91906700	-1.89415500	4.47741500
H	-1.30616700	-0.35801200	4.78721800
C	-2.30378200	0.75856500	2.55962100
H	-2.48634300	1.24779300	3.52002800
H	-2.16686000	1.52154400	1.79417900
H	-3.17420500	0.15641700	2.28360500
H	1.73999700	0.49023700	0.47108100
H	1.53404500	-4.59164500	-2.63044600
H	3.59150700	-4.57414800	-1.53493100

N11_TS

B	0.10648100	-0.01476700	0.24700300
N	-2.41089400	-1.95205600	0.45337900
C	-1.41217400	-1.87993500	1.54825100
H	-0.66590200	-2.65014800	1.32588200
C	-0.75259900	-0.49060500	1.57113200

H	-1.61643200	0.18580400	1.59713800
C	-0.11946300	-0.37979400	2.98208600
H	0.97220300	-0.37074400	2.95550200
H	-0.42969500	0.55389400	3.46070900
C	-0.64147000	-1.60751200	3.77240100
H	0.11938000	-2.39473300	3.76351100
H	-0.86809500	-1.38133700	4.81692300
C	-1.87350100	-2.09351900	2.99384400
H	-2.73347200	-1.44824300	3.21111100
H	-2.15275400	-3.12655400	3.22420500
C	-2.63236100	-3.31747300	-0.04904500
H	-3.17877200	-3.90533100	0.70583900
H	-1.65759800	-3.79077800	-0.19230100
C	-3.40033000	-3.28300200	-1.36604200
H	-3.55220500	-4.30614200	-1.72332600
H	-2.78020000	-2.76630600	-2.11186100
C	-4.73251600	-2.55477900	-1.19869100
H	-5.24960800	-2.46931200	-2.15903600
H	-5.38278300	-3.13972100	-0.53461100
C	-4.49645800	-1.17476700	-0.58653700
H	-3.93379400	-0.54743700	-1.28817900
H	-5.44442900	-0.66754200	-0.38372200
C	-3.70000800	-1.28636200	0.71038000
H	-3.51034900	-0.29799800	1.12982600
H	-4.27192700	-1.86837600	1.45103300
C	-0.20214200	1.60311600	0.16858300
F	1.94737300	2.34743300	0.87619600
C	0.68077000	2.61687300	0.53718300
F	1.23119800	4.87707200	0.95797900
C	0.32879400	3.96255100	0.60600700
F	-1.32284700	5.63462900	0.39333800
C	-0.97001900	4.35340800	0.32533100
F	-3.16357300	3.72663900	-0.27964300
C	-1.90098300	3.38329300	-0.01655800
F	-2.47072000	1.16396800	-0.37245100
C	-1.49826500	2.05851400	-0.06214600
C	1.71183900	-0.35881200	0.19371300
F	2.04769300	1.25277400	-1.51343200
C	2.54109100	0.27400200	-0.73018900
F	4.61244000	0.61372500	-1.82345700
C	3.87472000	-0.04740600	-0.93047400
F	5.71594900	-1.41939900	-0.37981400
C	4.43901600	-1.08567800	-0.20206200
F	4.15933700	-2.81099700	1.37692700
C	3.64858300	-1.78307000	0.69660400
F	1.59740700	-2.21353500	1.68176200
C	2.31759700	-1.41593900	0.86083800
C	-0.46102800	-0.92114200	-1.13371300
C	0.12853600	-2.21185600	-1.33537800
C	0.34372700	-2.45217200	-2.67872100
C	-0.08441400	-1.29691600	-3.33948400
N	-0.54643800	-0.40848400	-2.45659200
H	0.38695200	-2.88015000	-0.52305800
H	0.76148500	-3.33222000	-3.14362800
H	-0.08109800	-1.06920300	-4.39758700
C	-0.96731400	0.92494900	-2.86382400
H	-0.97277900	0.96119800	-3.95345000
H	-1.96615000	1.14026200	-2.49001500
H	-0.26100600	1.66298500	-2.48141200
H	-1.56812300	-1.26082900	-0.56960300

N11_P

B	0.43240800	-0.34403900	0.02906200
N	-2.37554700	-1.65668400	0.64276200
C	-1.19741000	-1.99036000	1.53872700
H	-0.80711600	-2.92194700	1.12586700
C	-0.08863300	-0.93815500	1.51953500
H	-0.50803900	-0.07426900	2.05785300
C	0.84992100	-1.57677900	2.56045900
H	1.42846900	-2.38470100	2.11306300
H	1.55673300	-0.84290800	2.95441500
C	-0.09070200	-2.14767900	3.65829800
H	0.23930800	-3.13484900	3.98985600
H	-0.11237100	-1.50552600	4.54149000
C	-1.50644200	-2.21675900	3.01961800
H	-2.12391100	-1.40495600	3.41391600
H	-2.02069700	-3.16108100	3.22162700
C	-3.15680100	-2.87877200	0.26227700
H	-3.57994700	-3.28208200	1.18825000
H	-2.43691900	-3.58745700	-0.14998400
C	-4.23398200	-2.52898100	-0.75430300
H	-4.76953000	-3.44552400	-1.01667600
H	-3.74379800	-2.16805800	-1.66809200
C	-5.18338100	-1.46587500	-0.20490800
H	-5.93597500	-1.20261000	-0.95225600
H	-5.71772700	-1.86784800	0.66592200
C	-4.38548700	-0.22718600	0.20018600
H	-3.96510400	0.24421000	-0.69131900

H	-5.02624300	0.51691000	0.68249400
C	-3.27880800	-0.58813100	1.18537200
H	-2.65159700	0.26891600	1.44056900
H	-3.71475600	-0.99514000	2.10162000
C	-0.47882100	1.05490900	-0.08348100
F	0.69623300	1.96325000	1.75539300
C	-0.28523000	2.08005400	0.84895600
F	-0.81400300	4.15624400	1.85440100
C	-1.06593700	3.22211300	0.93881300
F	-2.91775100	4.46044800	0.14872800
C	-2.14197000	3.38182600	0.07209600
F	-3.46270200	2.47964500	-1.66231500
C	-2.40120600	2.38903700	-0.85423300
F	-1.99989700	0.31436300	-1.77782100
C	-1.58204500	1.26300200	-0.90093000
C	2.06488600	-0.05885300	0.02927200
F	2.03952700	2.28247100	-0.43531700
C	2.72411300	1.15128700	-0.17356600
F	4.65728400	2.49784100	-0.39235100
C	4.10734600	1.29850400	-0.19263100
F	6.24219800	0.30311900	-0.03737100
C	4.91533900	0.18803100	-0.02163600
F	5.07594700	-2.14232300	0.27316400
C	4.31757200	-1.05265300	0.14072000
F	2.44776500	-2.39841800	0.24634400
C	2.93299700	-1.14801600	0.14531800
C	0.17285400	-1.31538100	-1.26145500
C	-0.13436400	-2.66889200	-1.38796800
C	-0.19014800	-3.01625400	-2.76512100
C	0.09982500	-1.87143400	-3.45934700
N	0.33063000	-0.86444100	-2.56073000
H	-0.16829300	-3.38456000	-0.57959100
H	-0.37918800	-3.99148000	-3.18982900
H	0.17409400	-1.68380100	-4.52121400
C	0.63474600	0.48592100	-2.99700100
H	1.35078600	0.44180800	-3.82088600
H	-0.26886500	1.00096400	-3.33881100
H	1.07706200	1.06014700	-2.18558700
H	-1.93479600	-1.32297000	-0.23631000

N12_TS

N	2.77289500	-1.20410400	0.28704300
H	1.62190300	-1.07532200	-0.67158800
B	-0.10233600	-0.12138700	0.15622300
C	2.24081400	-0.42109800	1.44164800
H	2.48900800	0.62452000	1.20970000
C	0.70254700	-0.52483300	1.54044900
H	0.48056000	-1.55879800	1.83914400
C	4.07371300	-0.71778900	-0.20637800
H	4.86357900	-0.96967000	0.52098000
H	4.02673800	0.36715000	-0.29126800
C	4.39476600	-1.35688600	-1.55428300
H	3.61973500	-1.06141000	-2.27318800
H	5.34947500	-0.96934700	-1.92233600
C	4.43677400	-2.88006900	-1.42669000
H	5.28108600	-3.16453000	-0.78456500
H	4.60194900	-3.34852300	-2.40125100
C	3.13493500	-3.38556000	-0.80244500
H	2.31008800	-3.21340100	-1.50565100
H	3.18561400	-4.46262100	-0.61072100
C	2.84863300	-2.65735100	0.51097200
H	1.90695300	-2.99079900	0.95471500
H	3.64821700	-2.88471800	1.23274200
C	2.92780300	-0.78621100	2.76549100
H	2.70131100	-1.83219900	3.01133200
H	4.01745900	-0.70610400	2.66474000
C	2.42935100	0.09390400	3.91209400
H	2.92533900	-0.19340100	4.84491900
H	2.70313600	1.13924700	3.71484500
C	0.91062700	-0.01170300	4.04100800
H	0.55096200	0.63886400	4.84523800
H	0.63736000	-1.03994200	4.31517300
C	0.24092900	0.35761800	2.71844300
H	0.48333400	1.40588200	2.49378700
H	-0.84774400	0.30611700	2.81101500
C	0.12712300	1.49208300	-0.10848300
C	-0.77138900	2.45237200	0.36251200
F	-1.87953400	2.08883700	1.02475600
C	-0.59513600	3.82247100	0.21507500
F	-1.50346000	4.67546900	0.68670200
C	0.53950500	4.30720300	-0.41836000
F	0.72605400	5.61617600	-0.56585600
C	1.47928800	3.40272000	-0.88294300
F	2.59098500	3.83366500	-1.48158600
C	1.25120000	2.04317600	-0.70956000
F	2.23380800	1.23670900	-1.17899700
C	-1.71242400	-0.47036600	0.15400300
C	-2.51329500	-0.00994400	-0.89443400

F	-1.98531300	0.77657300	-1.84542500
C	-3.86250000	-0.29994300	-1.03225900
F	-4.56246400	0.17658100	-2.06173600
C	-4.48849700	-1.09605900	-0.08313300
F	-5.78117300	-1.39236100	-0.19744500
C	-3.74490200	-1.58282900	0.97755700
F	-4.32342900	-2.35853300	1.89607500
C	-2.39344200	-1.26238700	1.07274400
F	-1.75863200	-1.81117000	2.13117000
C	0.48214100	-1.03073300	-1.21051600
C	0.54213600	-0.51141000	-2.53586600
C	0.07167300	-1.44514000	-3.44541200
C	-0.30325200	-2.55918500	-2.69307600
N	-0.06389100	-2.33378200	-1.39927900
H	0.90652600	0.47448300	-2.78167300
H	-0.01030600	-1.34734000	-4.51732700
H	-0.72523800	-3.50225000	-3.01849300
C	-0.37938900	-3.31779900	-0.37496300
H	-1.36944100	-3.73662100	-0.56941900
H	0.35997200	-4.12496200	-0.37781800
H	-0.39887700	-2.84519800	0.60342900

NB12_P

N	2.80825700	-1.09628700	0.38751900
H	2.23388800	-0.85089400	-0.44653100
B	-0.16017200	-0.15401500	0.04422200
C	2.11485500	-0.40011100	1.55037700
H	2.36574600	0.65773800	1.40201000
C	0.58688900	-0.55679000	1.48392900
H	0.37237800	-1.60220400	1.74337700
C	4.20005500	-0.60334700	0.13420800
H	4.82560000	-0.95767600	0.95933900
H	4.16489800	0.48524000	0.15128700
C	4.69616500	-1.13831700	-1.20256600
H	4.04121900	-0.75783600	-1.99743200
H	5.69882700	-0.74247900	-1.38608600
C	4.69005100	-2.66717500	-1.20664300
H	5.41634000	-3.03774600	-0.47110100
H	4.99934900	-3.04582600	-2.18416900
C	3.29018200	-3.17677300	-0.86357100
H	2.59249200	-2.90154700	-1.66332600
H	3.27525500	-4.26760100	-0.77887500
C	2.79432300	-2.59156300	0.45329700
H	1.77031500	-2.89761900	0.67261800
H	3.44122200	-2.88271900	1.28606900
C	2.69371700	-0.86490700	2.88870300
H	2.44902600	-1.92354100	3.04210500
H	3.78651500	-0.77170400	2.90497000
C	2.08258500	-0.05613400	4.03699700
H	2.48346400	-0.41665700	4.98926200
H	2.38850900	0.99396200	3.93946900
C	0.55689600	-0.14124700	4.00934900
H	0.13280300	0.48063100	4.80442300
H	0.24081300	-1.17420700	4.20781100
C	0.02713900	0.29448800	2.64472200
H	0.30617300	1.34500900	2.47877300
H	-1.06453300	0.26251800	2.62822000
C	0.07065100	1.48771000	-0.14967100
C	-0.88121800	2.44955200	0.20222800
F	-2.06593300	2.09629800	0.71993500
C	-0.69211700	3.82201500	0.08740300
F	-1.66001200	4.66667900	0.44044700
C	0.51594400	4.31856500	-0.37751800
F	0.71723900	5.62934800	-0.48786300
C	1.51434400	3.41849300	-0.70457200
F	2.70587600	3.85256200	-1.12306700
C	1.26651800	2.05798900	-0.57229800
F	2.33928700	1.27433300	-0.87368000
C	-1.78641300	-0.49703600	0.08165000
C	-2.58324700	-0.04012600	-0.97153600
F	-2.04901900	0.75294000	-1.91076300
C	-3.92938300	-0.33512300	-1.12018800
F	-4.62632000	0.14371200	-2.15113700
C	-4.55927400	-1.13714700	-0.17756000
F	-5.85105900	-1.43703900	-0.30059400
C	-3.82065800	-1.62336100	0.88571200
F	-4.39929000	-2.40966300	1.79692700
C	-2.47077400	-1.29667500	0.98928600
F	-1.83847700	-1.87040800	2.03982100
C	0.35087100	-0.97921300	-1.28876700
C	0.90548100	-0.53713600	-2.48704200
C	0.96621100	-1.61333800	-3.40977600
C	0.44484800	-2.70666400	-2.76516000
N	0.09447900	-2.33484800	-1.49286900
H	1.19481500	0.48181600	-2.69582900
H	1.33687400	-1.58649900	-4.42415400
H	0.30036200	-3.72608700	-3.09580900
C	-0.60533900	-3.25038600	-0.61030000

H	-1.67226000	-3.29380100	-0.85165900
H	-0.17569000	-4.25029300	-0.72147500
H	-0.50914800	-2.93827900	0.42868600

NB13_TS

N	-2.48556500	-0.75664900	0.68224500
H	-1.13353800	-0.74090000	1.29897300
B	0.42331900	-0.10967400	-0.03369600
C	-2.16876000	-0.10683000	-0.63172400
H	-2.14616900	0.96396400	-0.40010500
C	-0.76454100	-0.52626400	-1.08865100
H	-0.57432600	-0.03154300	-2.04945500
H	-0.76875300	-1.59300900	-1.32751400
C	-2.82282600	-2.18809900	0.59099500
H	-2.08092800	-2.67928900	-0.04380200
H	-3.80040400	-2.30695000	0.09991800
C	-2.86023600	-2.81492600	1.98450000
H	-1.86571700	-2.74507100	2.44503800
H	-3.10681600	-3.87781900	1.89118800
C	-3.87358700	-2.09332500	2.87284200
H	-4.88435800	-2.25865500	2.47676500
H	-3.85388700	-2.50020800	3.88799700
C	-3.57591200	-0.59407400	2.88084000
H	-4.33580600	-0.04736300	3.44720300
H	-2.60755700	-0.40740900	3.36292900
C	-3.52503500	-0.05289800	1.45573200
H	-4.50164100	-0.19071800	0.96353600
H	-3.28944300	1.01155400	1.45189700
C	-3.27324400	-0.31276800	-1.65764900
C	-4.32564100	0.60383300	-1.73801300
H	-4.32197500	1.47516500	-1.08680700
C	-5.36363600	0.42813500	-2.64921900
H	-6.16661000	1.15661300	-2.70098400
C	-5.36327400	-0.67361200	-3.49957800
H	-6.16711000	-0.81157900	-4.21537700
C	-4.31793600	-1.59089600	-3.43528000
H	-4.30420500	-2.44715200	-4.10210000
C	-3.28160000	-1.40930000	-2.52477800
H	-2.46438100	-2.12413300	-2.50139100
C	1.92864800	-0.64946100	-0.40545500
C	3.02192800	-0.21774900	0.34754800
F	2.84536800	0.67762100	1.33213400
C	4.32572400	-0.64396300	0.13919800
F	5.32339000	-0.18800000	0.89696600
C	4.59083700	-1.55284200	-0.87578300
F	5.83376800	-1.97845800	-1.09132100
C	3.54318100	-2.01488400	-1.65459800
F	3.77490400	-2.89687800	-2.62857800
C	2.25381200	-1.55312200	-1.41036400
F	1.29916500	-2.07354700	-2.20893000
C	0.43505700	1.53786100	0.04637300
C	-0.36108900	2.32004400	0.87412900
F	-1.22810000	1.73562300	1.73744500
C	-0.37052800	3.70885000	0.88874600
F	-1.16613200	4.37386300	1.72801400
C	0.44708700	4.39751500	0.00866400
F	0.45869100	5.72815000	-0.00532900
C	1.24033600	3.67490700	-0.87004400
F	2.01532700	4.31845200	-1.74138800
C	1.21072600	2.28590000	-0.84250900
F	1.97755100	1.67733200	-1.75845800
C	0.12148500	-0.81854300	1.52253500
C	0.47543900	-0.21636300	2.76308600
C	1.04158200	-1.15290200	3.61459100
C	1.06172800	-2.35406700	2.90571300
N	0.52786100	-2.17491400	1.69456800
H	0.31715400	0.82599500	2.99269300
H	1.41105400	-1.00321000	4.61769100
H	1.42717800	-3.32803400	3.20717400
C	0.43750700	-3.25797100	0.72665600
H	1.34977900	-3.85627900	0.77465100
H	-0.42271100	-3.89868800	0.94389800
H	0.34971900	-2.85649200	-0.27925700

NB_P13

N	2.55578500	-0.57955500	-0.61863000
H	1.76609700	-0.32977100	-1.25066600
B	-0.49657600	-0.14322400	-0.09270900
C	2.07533300	-0.14809800	0.76990000
H	2.06203400	0.94341700	0.69311100
C	0.65052500	-0.64567400	1.00462700
H	0.36830700	-0.26765700	1.99525100
H	0.66831100	-1.73181900	1.12811700
C	2.75215600	-2.05391500	-0.78421100
H	1.85066500	-2.54458000	-0.41403500
H	3.60087800	-2.33397700	-0.15321200
C	2.99834300	-2.38481700	-2.25138000
H	2.10119100	-2.12994200	-2.82881400

H	3.14346800	-3.46600600	-2.33635500
C	4.20221900	-1.61967200	-2.80036500
H	5.11965600	-1.95230400	-2.29717800
H	4.32567000	-1.82404000	-3.86694700
C	4.00710100	-0.12198600	-2.56430700
H	4.87898400	0.45122700	-2.89137900
H	3.14241800	0.23820900	-3.13706500
C	3.76718400	0.16317700	-1.08747900
H	4.60564900	-0.16883600	-0.46707200
H	3.58383500	1.22219100	-0.90417700
C	3.10082000	-0.51994800	1.82360300
C	4.05482100	0.42395400	2.21380100
H	4.03892300	1.41406300	1.76367300
C	5.00932100	0.12142200	3.18058300
H	5.73649500	0.87097100	3.47550500
C	5.01991300	-1.13684000	3.77428300
H	5.75921800	-1.37571100	4.53187800
C	4.06793500	-2.08288900	3.40305000
H	4.06109900	-3.06088600	3.87330900
C	3.11239100	-1.77522900	2.43962400
H	2.36127500	-2.51480800	2.17869200
C	-2.00579000	-0.69711500	0.29243000
C	-3.09188500	-0.24781300	-0.46064600
F	-2.90162100	0.67729600	-1.41222900
C	-4.39628200	-0.68169400	-0.27940100
F	-5.38878700	-0.20455000	-1.03169300
C	-4.67048300	-1.62045300	0.70656000
F	-5.91625600	-2.05194400	0.89823500
C	-3.63017300	-2.10322200	1.48138000
F	-3.86819700	-3.01753800	2.42528800
C	-2.33876600	-1.63215700	1.26282300
F	-1.38907100	-2.19349100	2.04207300
C	-0.49380300	1.52357000	-0.02173500
C	0.43157800	2.34262700	-0.66105000
F	1.44239900	1.80294700	-1.39878700
C	0.46871600	3.72919500	-0.59286400
F	1.40488000	4.41745200	-1.25074000
C	-0.46607300	4.38929400	0.18503300
F	-0.45648600	5.71657900	0.27954300
C	-1.39423100	3.63445500	0.88445100
F	-2.28371700	4.24466700	1.66605600
C	-1.38262300	2.24757900	0.78035700
F	-2.29234500	1.62762800	1.54434700
C	-0.25977700	-0.70150700	-1.62449600
C	-0.07772600	-0.05385800	-2.84298100
C	-0.11283000	-1.00398500	-3.89717800
C	-0.31616000	-2.22736200	-3.31060000
N	-0.38595800	-2.05108300	-1.95185700
H	0.02427100	1.01340000	-2.96800700
H	-0.00805900	-0.81247600	-4.95525700
H	-0.41288500	-3.21544000	-3.73913600
C	-0.76023700	-3.14442700	-1.07278800
H	-1.84488600	-3.29275000	-1.06744900
H	-0.27831300	-4.06212200	-1.42199100
H	-0.44651800	-2.94683900	-0.04882800

N14_TS

B	0.42334000	-0.10963400	-0.03364300
N	-2.48577600	-0.75643100	0.68225900
H	-1.13338000	-0.74073300	1.29920800
C	-2.16883200	-0.10668500	-0.63168300
H	-2.14627700	0.96410900	-0.40008100
C	-0.76457700	-0.52611400	-1.08856100
H	-0.57434100	-0.03137500	-2.04935400
H	-0.76881900	-1.59284900	-1.32746800
C	-2.82288800	-2.18790200	0.59109800
H	-2.08089000	-2.67910700	-0.04357900
H	-3.80041700	-2.30690500	0.09994900
C	-2.86041600	-2.81462100	1.98465100
H	-3.10687100	-3.87754800	1.89139100
H	-1.86596800	-2.74461400	2.44531500
C	-3.87398100	-2.09304100	2.87277200
H	-4.88466300	-2.25848500	2.47651200
H	-3.85445200	-2.49984300	3.88796400
C	-3.57642600	-0.59376000	2.88069100
H	-4.33642700	-0.04706000	3.44692000
H	-2.60814400	-0.40699700	3.36289000
C	-3.52537800	-0.05268500	1.45554600
H	-3.28980800	1.01177800	1.45168900
H	-4.50192700	-0.19055200	0.96324600
C	-3.27319000	-0.31265300	-1.65775300
C	-4.32556100	0.60395700	-1.73840000
H	-4.32203300	1.47533100	-1.08724600
C	-5.36338000	0.42818200	-2.64979700
H	-6.16634600	1.15665300	-2.70177900
C	-5.36286200	-0.67363900	-3.50006100
H	-6.16655100	-0.81165400	-4.21601700
C	-4.31756200	-1.59094400	-3.43545700

H	-4.30370100	-2.44726000	-4.10219800
C	-3.28141400	-1.40927800	-2.52476300
H	-2.46422200	-2.12413100	-2.50112600
C	1.92860200	-0.64963400	-0.40535200
C	3.02191400	-0.21807800	0.34769400
F	2.84540400	0.67721500	1.33236000
C	4.32567400	-0.64438100	0.13931200
F	5.32338000	-0.18855700	0.89712100
C	4.59071100	-1.55320200	-0.87574100
F	5.83360400	-1.97892800	-1.09129500
C	3.54301900	-2.01510200	-1.65459300
F	3.77468000	-2.89708700	-2.62860200
C	2.25368700	-1.55325300	-1.41033100
F	1.29898000	-2.07360000	-2.20888100
C	0.43528400	1.53790100	0.04639400
C	-0.36090300	2.32017400	0.87402800
F	-1.22807700	1.73583500	1.73722400
C	-0.37022100	3.70898200	0.88859700
F	-1.16587300	4.37410400	1.72773400
C	0.44755200	4.39754700	0.00857400
F	0.45926200	5.72818600	-0.00548000
C	1.24082000	3.67484300	-0.87002900
F	2.01592900	4.31828400	-1.74134700
C	1.21108900	2.28584200	-0.84245100
F	1.97794000	1.67719300	-1.75833200
C	1.06092200	-2.35401400	2.90615300
C	1.04081400	-1.15280200	3.61498300
C	0.47516600	-0.21617700	2.76327000
C	0.12143100	-0.81834200	1.52262400
N	0.52750200	-2.17479700	1.69484100
H	1.42608600	-3.32804600	3.20776000
H	1.40996200	-1.00316400	4.61821000
H	0.31694600	0.82620900	2.99280600
C	0.43748600	-3.25778700	0.72680800
H	0.34889100	-2.85620600	-0.27898600
H	1.35021300	-3.85545100	0.77424300
H	-0.42218200	-3.89913800	0.94437500

N14_P

B	0.49637000	-0.14335700	0.09279100
N	-2.55602300	-0.57865000	0.61876200
H	-1.76630400	-0.32868100	1.25064100
C	-2.07555300	-0.14792100	-0.76996600
H	-2.06215800	0.94363100	-0.69368900
C	-0.65079900	-0.64581000	-1.00443000
H	-0.36850500	-0.26807000	-1.99514200
H	-0.66881700	-1.73197300	-1.12768100
C	-2.75259400	-2.05290300	0.78502300
H	-1.85112800	-2.54381100	0.41510100
H	-3.60132500	-2.33313800	0.15413000
C	-2.99889400	-2.38309800	2.25233300
H	-3.14420000	-3.46422800	2.33779400
H	-2.10172100	-2.12815200	2.82969700
C	-4.20264000	-1.61748500	2.80094100
H	-5.12014200	-1.95018200	2.29792400
H	-4.32611500	-1.82136600	3.86761500
C	-4.00725000	-0.11993300	2.56419100
H	-4.87901000	0.45362200	2.89099300
H	-3.14249100	0.24035600	3.13677400
C	-3.76728000	0.16451600	1.08723800
H	-3.58372400	1.22341300	0.90343500
H	-4.60580200	-0.16762500	0.46696600
C	-3.10098200	-0.52027300	-1.82353700
C	-4.05461200	0.42358000	-2.21475400
H	-4.03859000	1.41402300	-1.76538200
C	-5.00884400	0.12056700	-3.18165700
H	-5.73567100	0.87009000	-3.47746700
C	-5.01958800	-1.13814000	-3.77439200
H	-5.75867300	-1.37736300	-4.53208000
C	-4.06803400	-2.08419600	-3.40207400
H	-4.06135200	-3.06257200	-3.87152200
C	-3.11273500	-1.77604400	-2.43858900
H	-2.36193500	-2.51563100	-2.17681600
C	2.00551800	-0.69751100	-0.29221500
C	3.09161400	-0.24818100	0.46083500
F	2.90139300	0.67714000	1.41223200
C	4.39598300	-0.68221200	0.27973300
F	5.38848500	-0.20503200	1.03200300
C	4.67015400	-1.62118900	-0.70603500
F	5.91590200	-2.05277200	-0.89762200
C	3.62983700	-2.10395600	-1.48085300
F	3.86780900	-3.01837500	-2.42466900
C	2.33845200	-1.63274600	-1.26241700
F	1.38877100	-2.19407300	-2.04168300
C	0.49398800	1.52345200	0.02136100
C	-0.43104500	2.34296300	0.66057500
F	-1.44205800	1.80381300	1.39844600
C	-0.46764700	3.72954000	0.59217800

F	-1.40348000	4.41827000	1.25002400
C	0.46726800	4.38914000	-0.18598900
F	0.45819100	5.71640800	-0.28073900
C	1.39500400	3.63381700	-0.88543800
F	2.28453300	4.24353800	-1.66738300
C	1.38285900	2.24696600	-0.78111700
F	2.29205100	1.62650500	-1.54532200
C	0.31583100	-2.22672500	3.31115200
C	0.11267800	-1.00322200	3.89750400
C	0.07758100	-0.05330100	2.84311800
C	0.25939800	-0.70118100	1.62473800
N	0.38542300	-2.05074000	1.95235800
H	0.41252000	-3.21473500	3.73985600
H	0.00796000	-0.81151100	4.95555100
H	-0.02424800	1.01398700	2.96797900
C	0.75941200	-3.14430900	1.07346000
H	0.44511700	-2.94718100	0.04957400
H	1.84406700	-3.29249100	1.06765200
H	0.27775400	-4.06194300	1.42320400

N15_TS

N	-2.46237900	-0.72395400	1.03456800
B	0.38497000	-0.13409000	0.06455300
C	-2.24251300	-0.24452700	-0.37178000
H	-2.23516700	0.84788500	-0.28418500
C	-0.85683200	-0.69421700	-0.85317400
H	-0.73091900	-0.32241000	-1.87816800
H	-0.85089600	-1.78197700	-0.95654600
C	-2.71649900	-2.17743100	1.16353500
C	-4.17641800	-2.63134300	1.16204700
H	-4.71012500	-2.29162600	2.05312600
H	-4.20063600	-3.72427800	1.16206400
C	-3.21458600	-0.12717300	3.31721900
H	-3.87800000	0.53678700	3.87620000
H	-2.18310500	0.09395100	3.60599100
C	-3.40568600	0.09017000	1.82137200
C	-3.40070000	-0.61008700	-1.28739700
C	-4.55339800	0.18257000	-1.28095100
H	-4.57173000	1.08779600	-0.67848800
C	-5.66898600	-0.16603000	-2.03595700
H	-6.55349600	0.46267100	-2.01726100
C	-5.64459000	-1.31548800	-2.82211400
H	-6.51177000	-1.59119500	-3.41335900
C	-4.49380500	-2.09778600	-2.85960800
H	-4.46047200	-2.98439500	-3.48490800
C	-3.37950200	-1.74514800	-2.10196100
H	-2.48598200	-2.35967300	-2.15405700
C	1.87190300	-0.71154700	-0.32197500
C	3.00480400	-0.17153100	0.28977000
F	2.87972700	0.85228400	1.14905000
C	4.29861400	-0.61665300	0.05891900
F	5.33684800	-0.05470800	0.67855300
C	4.51150500	-1.65826400	-0.83327300
F	5.74423400	-2.10242700	-1.06886200
C	3.42287000	-2.23069000	-1.46981000
F	3.60477000	-3.23550800	-2.32857700
C	2.14501400	-1.74726300	-1.20825700
F	1.15047100	-2.37856700	-1.86586900
C	0.37505100	1.50783600	-0.08338700
C	-0.37563100	2.39417500	0.67857500
F	-1.16795400	1.93279300	1.67792300
C	-0.41309600	3.77014900	0.49127700
F	-1.15900200	4.54335700	1.28249000
C	0.32263000	4.33050500	-0.53950300
F	0.30167700	5.64424900	-0.74933900
C	1.06493500	3.49502300	-1.36088200
F	1.75945800	4.01012200	-2.37395800
C	1.06793400	2.12522000	-1.12759700
F	1.77793800	1.39612600	-2.00079700
H	-4.71422000	-2.28029500	0.27913000
H	-3.44234700	-1.15402300	3.61743700
H	-2.23677400	-2.50626900	2.09524300
H	-2.18527000	-2.67150600	0.34625100
H	-3.20924200	1.13506400	1.58050100
H	-4.44012600	-0.12541900	1.52298500
H	-1.07874300	-0.58951400	1.54635800
C	0.18320800	-0.62490700	1.72195300
C	0.62775000	0.15143100	2.82949200
C	1.27539300	-0.64980700	3.75682300
C	1.25288600	-1.94134700	3.23116100
N	0.61599700	-1.94274400	2.05777400
H	0.47644000	1.21597300	2.91875800
H	1.72232100	-0.35391600	4.69383500
H	1.65690300	-2.86018400	3.63805400
C	0.50317200	-3.14531700	1.24470000
H	1.46052300	-3.67130400	1.24870900
H	-0.27375300	-3.80808300	1.63466200
H	0.27036400	-2.87934400	0.21768100

N15_P

N	-2.60364100	-0.32949000	0.93004100
B	0.46184200	-0.08023500	0.16210200
C	-2.19527700	-0.15750400	-0.54433700
H	-2.19442600	0.92902600	-0.67300400
C	-0.78065600	-0.69369300	-0.75871800
H	-0.55838800	-0.50994700	-1.81722500
H	-0.80710700	-1.78243000	-0.67717800
C	-2.80764000	-1.76106400	1.36874700
C	-4.23530000	-2.28050300	1.27203500
H	-4.89707800	-1.80576900	2.00002400
H	-4.21327200	-3.34861100	1.50126700
C	-3.73980100	0.69730000	2.88590200
H	-4.48858100	1.43856500	3.17242000
H	-2.76548300	1.02407500	3.26065900
C	-3.71090300	0.57578000	1.37036200
C	-3.26315400	-0.73989700	-1.45266700
C	-4.39500000	0.01991100	-1.76598200
H	-4.46956300	1.04235600	-1.40320300
C	-5.41932500	-0.50866700	-2.54563500
H	-6.28924000	0.09660400	-2.77889200
C	-5.31799000	-1.80730000	-3.03741000
H	-6.11277600	-2.22266300	-3.64834700
C	-4.18049100	-2.56110900	-2.76185900
H	-4.08278700	-3.56442200	-3.16353400
C	-3.15847400	-2.03007000	-1.98036700
H	-2.27031700	-2.62451900	-1.79258800
C	1.89568600	-0.82111800	-0.20200300
C	3.04437900	-0.39401300	0.46631600
F	2.97399000	0.66007000	1.29164800
C	4.29301700	-0.97977000	0.32241100
F	5.35008300	-0.51520600	0.98964600
C	4.44133800	-2.06031300	-0.53764700
F	5.62926300	-2.64328300	-0.69151500
C	3.33565500	-2.52497800	-1.22795900
F	3.45303700	-3.56956600	-2.05167200
C	2.10484800	-1.89875400	-1.05206900
F	1.08405900	-2.45607800	-1.73995500
C	0.57263100	1.54818100	-0.17009300
C	-0.31800000	2.50079700	0.31603200
F	-1.36490000	2.12888000	1.10452800
C	-0.27505500	3.86306000	0.05108900
F	-1.18729400	4.68548000	0.57507600
C	0.71722400	4.35451000	-0.77882000
F	0.79109600	5.65327600	-1.05859400
C	1.62067100	3.45839500	-1.32749200
F	2.57129000	3.90188500	-2.14880200
C	1.52646500	2.10269400	-1.03046000
F	2.42814800	1.33891100	-1.66188700
H	-4.66148900	-2.16162200	0.27450400
H	-3.99510700	-0.24666100	3.37356500
H	-2.44018300	-1.81045000	2.39732500
H	-2.13377200	-2.36331900	0.75804800
H	-3.51044800	1.54721900	0.91876100
H	-4.64612100	0.19113800	0.96037200
H	-1.75293300	-0.01837800	1.44278800
C	0.24595200	-0.37662200	1.76368600
C	0.03298800	0.45468700	2.85821500
C	-0.11307600	-0.33253100	4.03221000
C	0.02792200	-1.63995900	3.64257800
N	0.23409900	-1.66913300	2.28592900
H	0.00627700	1.53338300	2.81891000
H	-0.28321100	0.01875200	5.03972400
H	0.01534000	-2.55754900	4.21418600
C	0.58973000	-2.90386500	1.60890000
H	1.67358700	-3.05776100	1.61007900
H	0.11200100	-3.73717400	2.13029400
H	0.25352800	-2.89914700	0.57271900

N17_TS

B	0.02740100	-0.17796600	0.02746000
N	-2.70656600	-1.53432700	-0.28912800
H	-1.49043100	-1.15398100	-1.09971700
C	-2.40551200	-0.81155000	0.97653900
H	-2.72541100	0.22297900	0.79255000
C	-0.88768500	-0.80253500	1.25109800
H	-0.61161900	-1.84723600	1.45415400
C	-4.00369600	-1.17144100	-0.91612100
H	-4.63016400	-0.65387800	-0.18037000
H	-3.83460700	-0.48764500	-1.75064700
C	-4.64986400	-2.49183200	-1.35132300
H	-4.33784900	-2.75009000	-2.36862800
H	-5.74027400	-2.43993500	-1.33783600
C	-4.07103400	-3.50324000	-0.35979300
H	-4.13968900	-4.53822000	-0.70206600
H	-4.57684000	-3.42427900	0.60844800
C	-2.63091300	-3.01268100	-0.23967800

H	-2.05007500	-3.35326900	-1.10886600
H	-2.11287800	-3.35039900	0.66031600
C	-3.21589900	-1.34514000	2.16756600
H	-2.97169400	-2.40337500	2.33291300
H	-4.28934800	-1.29578400	1.94496100
C	-2.90567800	-0.57588700	3.45269100
H	-3.22703100	0.46825300	3.33818000
H	-3.48133200	-0.99485800	4.28458600
C	-1.40698100	-0.60868800	3.74765400
H	-1.09095100	-1.64528700	3.92984500
H	-1.18613600	-0.04350300	4.65929100
C	-0.62678500	-0.03937900	2.56480000
H	0.44591100	-0.03283800	2.77874100
H	-0.92244100	1.01090200	2.43226700
C	-0.29922100	1.43669600	-0.06909200
C	0.48546300	2.39247400	0.57984300
F	1.56797100	2.02990500	1.28422900
C	0.21299400	3.75480400	0.57613600
F	1.01358800	4.60733200	1.21405300
C	-0.90916200	4.23050600	-0.08516300
F	-1.18775000	5.53151700	-0.09662500
C	-1.74050400	3.32554200	-0.72327500
F	-2.83752600	3.74965200	-1.35283100
C	-1.42162100	1.97365000	-0.68757300
F	-2.30418100	1.16093800	-1.31596400
C	1.64597700	-0.43296800	0.17140500
C	2.27538800	-1.28887900	1.06997500
F	1.57001500	-2.00215700	1.97229700
C	3.64991300	-1.50845900	1.10539700
F	4.17739300	-2.35039800	1.99621300
C	4.46993300	-0.85492600	0.20221200
F	5.78613600	-1.05348900	0.21533200
C	3.89702900	0.00774800	-0.72221000
F	4.67055300	0.64758900	-1.59933200
C	2.52306500	0.19625100	-0.71589900
F	2.04871700	1.06171500	-1.62547900
C	0.79167500	-2.13956700	-3.06500400
C	0.40396500	-0.95503000	-3.69495700
C	-0.29135900	-0.22599500	-2.74464700
C	-0.34486600	-0.93874100	-1.51241700
N	0.34847400	-2.15010100	-1.80735600
H	1.35749500	-2.97649400	-3.45630500
H	0.61821700	-0.68318600	-4.71746100
H	-0.73323200	0.74783800	-2.89484400
C	0.66985700	-3.22157400	-0.87560400
H	0.29323100	-2.97194900	0.11318300
H	0.22136500	-4.16107800	-1.20875600
H	1.75430500	-3.34023900	-0.80825100

N17_P

B	0.09846000	-0.16035000	-0.07634800
N	-2.83361900	-1.25472100	-0.14854700
H	-2.19870800	-0.87805200	-0.88139500
C	-2.30969300	-0.66601500	1.15261800
H	-2.62170600	0.38353500	1.10297100
C	-0.77559100	-0.73313000	1.22844500
H	-0.52873700	-1.79149100	1.39462100
C	-4.24870600	-0.84721700	-0.48604800
H	-4.70256700	-0.44338300	0.42047900
H	-4.20437700	-0.05940500	-1.23661600
C	-4.94273900	-2.12770600	-0.94734500
H	-4.78920500	-2.27267900	-2.02106800
H	-6.01644200	-2.09603600	-0.75565200
C	-4.21119000	-3.22108800	-0.16429500
H	-4.34755400	-4.21761800	-0.58744700
H	-4.54311300	-3.24035400	0.87869000
C	-2.76364300	-2.76036100	-0.26523400
H	-2.35313800	-2.97218700	-1.25509900
H	-2.08505500	-3.15437900	0.49089900
C	-2.98129700	-1.31874400	2.36357100
H	-2.69607500	-2.37775000	2.41799500
H	-4.07494800	-1.27970900	2.28570600
C	-2.53212700	-0.62242900	3.65276800
H	-2.90215800	0.41137800	3.64508700
H	-2.99276900	-1.11761100	4.51306900
C	-1.00831400	-0.60773800	3.77027800
H	-0.63389400	-1.63363700	3.88581100
H	-0.70968600	-0.05635600	4.66781600
C	-0.38705800	0.01466000	2.52136700
H	0.70099800	0.05594200	2.61055600
H	-0.72675700	1.05727400	2.43778100
C	-0.14803300	1.48937100	-0.12183400
C	0.77211200	2.42818000	0.35616700
F	1.94507700	2.04985100	0.88326000
C	0.56207100	3.80280900	0.36432500
F	1.50284600	4.62311200	0.83047100
C	-0.63643900	4.32380600	-0.09657100
F	-0.85852800	5.63605900	-0.08781600

C	-1.60548700	3.44395200	-0.54584400
F	-2.78760100	3.89963500	-0.96748300
C	-1.33846100	2.08082300	-0.53451400
F	-2.38560700	1.31546100	-0.95040900
C	1.71717100	-0.49436300	0.08484900
C	2.33768700	-1.33651300	0.99950500
F	1.63063900	-1.98587900	1.95344500
C	3.69676900	-1.64050300	0.98818500
F	4.21201700	-2.47092200	1.89833600
C	4.51039900	-1.08880400	0.01514300
F	5.81235600	-1.36726500	-0.01982800
C	3.94559500	-0.24372000	-0.93163300
F	4.71507300	0.29720300	-1.87684200
C	2.58779400	0.02842900	-0.87472900
F	2.11427700	0.86574300	-1.80803300
C	-0.46557800	-2.52718600	-3.05211200
C	-1.02932200	-1.41174100	-3.61698200
C	-0.91857900	-0.37346000	-2.65381500
C	-0.30192500	-0.86659600	-1.50668400
N	-0.04086600	-2.20573400	-1.78716900
H	-0.31984300	-3.52522000	-3.44145800
H	-1.45111200	-1.34164100	-4.60909300
H	-1.23413300	0.64917000	-2.79480600
C	0.69315800	-3.15821200	-0.97279500
H	0.50377100	-2.99722800	0.08850400
H	0.36881800	-4.16737500	-1.23917100
H	1.77111300	-3.07627400	-1.14305200

N18_TS

C	0.00000000	0.00000000	0.00000000
C	0.00000000	0.00000000	1.58856437
H	1.05605083	0.00000000	1.86544482
C	-0.59432959	1.37063824	1.98434035
H	-1.67796844	1.23957891	2.03378199
C	-0.37150474	2.14258115	0.66298860
H	-0.81589215	3.14074112	0.66324435
C	1.12639598	2.12845096	0.25338616
H	1.78557642	2.33768128	1.09483560
H	1.33348758	2.88962360	-0.50269080
C	1.34741322	0.69265446	-0.30605139
H	2.18639152	0.17435845	0.17061147
H	1.54235186	0.69478682	-1.38268530
C	-0.98285669	1.15762923	-0.37991790
C	-0.18208622	-1.32596816	-0.71420527
H	-1.13822418	-1.80331010	-0.47631214
H	0.62092495	-2.02742965	-0.46580446
H	-0.15197060	-1.17532194	-1.79884650
C	-2.47085951	0.82817941	-0.20265017
H	-2.68290163	0.14379253	0.61561992
H	-2.85951953	0.35777580	-1.11209984
H	-3.04448577	1.74642211	-0.03262425
C	-0.84950647	1.66545783	-1.82472927
H	-1.01013827	0.85228546	-2.54164882
H	0.11038184	2.12882838	-2.05212633
H	-1.62464712	2.41606973	-2.01088791
N	-0.50940448	-1.10039512	2.45364420
H	-0.48512343	-0.31516104	3.70046956
C	-1.91954476	-1.50939573	2.36503157
H	-2.10443829	-2.04364491	1.42177561
H	-2.53914780	-0.61261266	2.36636705
C	-2.30463094	-2.40109089	3.54796333
H	-3.35185265	-2.69983791	3.43261658
H	-2.22810641	-1.82043742	4.47665238
C	-1.39149030	-3.62082779	3.64941813
H	-1.53965523	-4.26433413	2.77169371
H	-1.64282380	-4.21588084	4.53202911
C	0.06339006	-3.15860084	3.69512427
H	0.24332644	-2.57800412	4.60922205
H	0.74796760	-4.01197795	3.70599885
C	0.37304205	-2.28455437	2.48475010
H	1.40545239	-1.93331834	2.50929689
H	0.23219419	-2.86939177	1.56315740
B	-0.13627099	1.86711479	3.48169198
C	-0.92121871	3.18790728	4.07084611
F	-2.51687201	3.40720429	2.31002254
C	-2.00127725	3.83549468	3.48354000
F	-3.70277259	5.48042232	3.42653963
C	-2.66463453	4.92025836	4.05006018
F	-2.86098928	6.44828670	5.83699292
C	-2.24375501	5.41152399	5.27387511
F	-0.75584313	5.25998005	7.09155315
C	-1.17201781	4.80064641	5.91110983
F	0.48645489	3.18776586	5.97823150
C	-0.54791807	3.71927308	5.30746716
C	1.49557314	2.09348995	3.56327316
F	1.28411658	4.43068884	3.17345879
C	2.06113450	3.35920119	3.38761972
F	3.88935148	4.84401663	3.19217543

C	3.42732838	3.60707849	3.36737597
F	5.63232645	2.77202568	3.49132047
C	4.31977603	2.55502567	3.51177035
F	4.65008245	0.23969721	3.80189058
C	3.81859667	1.27426737	3.66938677
F	2.05678894	-0.21260222	3.82238232
C	2.44230296	1.08234744	3.68531200
C	-0.61772199	0.61344112	4.60253398
C	0.12564385	0.23850039	5.75876065
C	-0.71907061	0.01609561	6.83639873
C	-2.00664529	0.27652304	6.37231788
H	1.19905386	0.13362087	5.77728448
H	-0.45301062	-0.28332145	7.83879007
H	-2.95343171	0.22888539	6.89635255
N	-1.96350189	0.62441106	5.08220135
C	-3.17859338	0.97954869	4.36174209
H	-2.93728610	1.45606535	3.41613871
H	-3.78670430	0.08800089	4.17651511
H	-3.75578785	1.68908811	4.95955393

N18_P

C	13.40924200	3.53606200	5.04060600
C	12.07215700	2.89772900	4.50265000
H	11.34688900	2.85906400	5.32402400
C	11.56363500	3.88129500	3.40390800
H	11.94073200	3.49334900	2.45402300
C	12.45328700	5.11571800	3.72067800
H	12.38670000	5.88882800	2.95732800
C	12.16287600	5.62200500	5.15560000
H	11.09930700	5.68396600	5.37120000
H	12.58199800	6.61945800	5.31097400
C	12.84962100	4.56451000	6.05833900
H	12.15704900	4.08891900	6.75991400
H	13.66011500	4.98334200	6.65989000
C	13.86850200	4.50674800	3.89389900
C	14.50112100	2.67952600	5.66546400
H	14.89208400	1.89782500	5.00607800
H	14.17060400	2.21266500	6.59880500
H	15.34399800	3.32971500	5.92010100
C	14.47945500	3.88097000	2.63149400
H	13.77391700	3.36768200	1.98277100
H	15.28679400	3.18445200	2.89091000
H	14.92889200	4.67259400	2.02468000
C	14.92337800	5.53019100	4.35568400
H	15.90899100	5.05787300	4.43661300
H	14.70930100	6.01471800	5.30712600
H	15.00649000	6.31779300	3.60021400
N	12.06164800	1.42417000	4.06849700
H	11.07543500	1.31150000	3.75799300
C	12.94959800	1.04030900	2.92922000
H	13.97779100	1.25232200	3.23124900
H	12.69381100	1.68995300	2.09436600
C	12.77386800	-0.42039300	2.53467800
H	13.48396600	-0.63346900	1.73032400
H	11.76433800	-0.55609400	2.12939500
C	12.97511400	-1.35555100	3.72319800
H	14.00729700	-1.27672300	4.08999600
H	12.81725700	-2.39446100	3.42235500
C	11.99777300	-0.96402400	4.82853600
H	10.96907100	-1.10224700	4.47930900
H	12.13277200	-1.58284700	5.72049600
C	12.19722900	0.48872100	5.23663200
H	11.44882400	0.80743100	5.96622100
H	13.19016800	0.63324200	5.65972500
B	9.90343100	3.94479800	3.12998400
C	9.51762800	4.58500600	1.64322000
F	11.70780500	5.10383700	0.85956100
C	10.36890700	5.10866800	0.67885100
F	10.82725100	6.07121600	-1.43628000
C	9.94588600	5.60114300	-0.55033800
F	8.16699700	6.05860300	-2.03135300
C	8.59466000	5.59193800	-0.85958700
F	6.39289900	5.06012800	-0.21705300
C	7.69550900	5.08246800	0.06670100
F	7.24639400	4.10368200	2.11670400
C	8.16992300	4.59934600	1.28029500
C	9.05226500	4.97068200	4.14298700
F	10.27222000	6.76022000	3.16076500
C	9.30311000	6.34192300	3.99508200
F	8.98084500	8.63458800	4.47841100
C	8.64534500	7.35761600	4.67168500
F	6.98137900	7.98271800	6.22906500
C	7.63065100	7.03301500	5.55905400
F	6.33214900	5.35404800	6.56919000
C	7.31237000	5.69976100	5.73187000
F	7.55774900	3.47610100	5.26398900
C	8.00382800	4.71710200	5.02438000
C	9.42712300	2.36643600	3.09327800

C	8.98898300	1.49366400	4.08880600
C	8.85792300	0.18195300	3.55828200
C	9.25105300	0.25355700	2.24645700
H	8.74860800	1.78229300	5.09825600
H	8.49207800	-0.69798300	4.06861200
H	9.28455200	-0.49953800	1.47150400
N	9.61828500	1.55073900	1.97248900
C	10.11146500	1.91025600	0.64941000
H	10.98491500	2.56256300	0.71540600
H	10.40259000	0.98937700	0.13895300
H	9.34850800	2.41864600	0.05512400

N19_TS

B	0.46812400	-0.38789800	0.20652700
C	-0.16937700	-0.89923300	1.64141200
C	0.58150700	-1.74822400	2.47241400
H	1.56436100	-2.06972100	2.14066600
C	0.13685400	-2.21853500	3.70388800
H	0.77267800	-2.86931800	4.29645400
C	-1.10949400	-1.82987400	4.17547300
H	-1.47061700	-2.16041800	5.14398900
C	-1.89061800	-1.00735600	3.37540700
H	-2.87471900	-0.70466400	3.72878600
C	-1.46290000	-0.57065100	2.11633900
C	-2.42825900	0.35495500	1.39420300
H	-1.90620900	1.28873800	1.19922400
H	-3.24192300	0.59921100	2.08551900
N	-2.99356100	-0.08677300	0.08582200
C	-3.96412200	-1.25847400	0.17063100
C	-4.36317100	-1.64216600	-1.26428800
H	-3.46048300	-2.00112400	-1.77980200
H	-5.05821900	-2.48665800	-1.20231800
C	-4.97203000	-0.49999900	-2.06023000
H	-5.93308700	-0.19722200	-1.62891100
H	-5.18458600	-0.82512400	-3.08339000
C	-3.99596800	0.66502500	-2.07960200
H	-4.43564600	1.53379200	-2.58198500
H	-3.11169900	0.37366500	-2.65699300
C	-3.54313400	1.12034300	-0.68194300
C	-2.44288800	2.17651700	-0.88615200
H	-1.48141000	1.73728400	-1.13902900
H	-2.30112300	2.81543700	-0.01076900
H	-2.74934200	2.82753900	-1.70986800
C	-4.70084600	1.85350400	0.02687000
H	-4.51403600	1.98574400	1.09534100
H	-5.66909700	1.37331900	-0.10002500
H	-4.78096800	2.85280300	-0.41106700
C	-5.21748400	-1.00121600	1.02924500
H	-4.96787600	-0.53789500	1.98756300
H	-5.67283100	-1.97028100	1.25334500
H	-5.97804400	-0.39596000	0.54081600
C	-3.28966200	-2.49873200	0.77118600
H	-2.30699000	-2.65516700	0.34011100
H	-3.91023000	-3.36571400	0.52445600
H	-3.19285300	-2.45200200	1.85437100
C	2.05275700	-0.85804300	0.03210400
C	2.63993800	-1.52113700	-1.03931600
F	1.89807700	-1.98500000	-2.06196300
C	4.00606800	-1.76305500	-1.15178700
F	4.49104500	-2.41911300	-2.20754000
C	4.86511300	-1.30944900	-0.16572700
F	6.17472600	-1.53004300	-0.25533100
C	4.33960400	-0.61715700	0.91638600
F	5.15131200	-0.16087900	1.87042600
C	2.97112900	-0.39916300	0.98189800
F	2.53841100	0.30919200	2.03564300
C	0.63139200	1.25608100	0.06841300
C	0.43052000	2.19034900	1.08437100
F	0.00859500	1.81933800	2.30064100
C	0.62310300	3.55986700	0.92833300
F	0.37649400	4.39527500	1.93770300
C	1.08582100	4.05762800	-0.27886800
F	1.27461300	5.36407000	-0.44477300
C	1.36909700	3.16767700	-1.30391700
F	1.85674300	3.61653700	-2.46089700
C	1.16563200	1.80924400	-1.09955700
F	1.53274000	1.01648700	-2.12265800
C	-0.51813800	-0.98117900	-1.03713500
C	-0.70204500	-0.47544400	-2.36305800
C	-0.94390100	-1.50562100	-3.25524500
C	-0.83719200	-2.68222600	-2.51259600
N	-0.58799200	-2.39802500	-1.23427900
H	-0.72476500	0.57675600	-2.60798800
H	-1.16915900	-1.43479100	-4.30860200
H	-0.91234100	-3.71127100	-2.84159900
C	-0.11923800	-3.42250900	-0.30878700
H	-0.47961600	-4.39139900	-0.65850500
H	0.97380800	-3.42327000	-0.29447300

H	-0.47960000	-3.23726700	0.69976600
H	-1.70633200	-0.58522500	-0.59197000

N19_P

B	0.60659800	-0.46999500	0.04576700
C	-0.05697400	-1.17578100	1.39527700
C	0.68680700	-2.15917700	2.07043800
H	1.67868200	-2.40183300	1.70018800
C	0.23828300	-2.84325900	3.19515000
H	0.87500500	-3.58827400	3.66287400
C	-1.00621000	-2.54277000	3.73250700
H	-1.36557600	-3.03329600	4.63116500
C	-1.77808900	-1.58168500	3.09663000
H	-2.74649100	-1.32284600	3.52061300
C	-1.34738200	-0.93435200	1.93066700
C	-2.30011000	0.12509200	1.41539400
H	-1.75092400	1.04163100	1.23582300
H	-3.04562200	0.33073000	2.18254900
N	-3.07707400	-0.11202800	0.12657600
C	-3.97455700	-1.37885700	0.09056700
C	-4.65884500	-1.44411100	-1.28648700
H	-3.88937800	-1.69052400	-2.02976400
H	-5.35109700	-2.29172800	-1.24846400
C	-5.37695500	-0.17570700	-1.71468700
H	-6.23235400	0.03750800	-1.06366700
H	-5.78327500	-0.30752200	-2.72142900
C	-4.38497100	0.97646700	-1.70718000
H	-4.86351600	1.91320400	-2.01079500
H	-3.58522400	0.77167000	-2.43118300
C	-3.74550400	1.22094100	-0.33416300
C	-2.66610000	2.30413900	-0.49300900
H	-1.75401300	1.92431300	-0.95479800
H	-2.40343700	2.77991000	0.45444400
H	-3.07527100	3.08140500	-1.14349000
C	-4.78095200	1.74445400	0.66544600
H	-4.38626600	1.81492500	1.68074300
H	-5.70727400	1.17503000	0.68506400
H	-5.03289700	2.76160500	0.35234000
C	-4.99688700	-1.39943400	1.22715400
H	-4.53930300	-1.20444400	2.19889000
H	-5.41088000	-2.41049300	1.26798900
H	-5.83018000	-0.71357100	1.08278400
C	-3.09585600	-2.63295400	0.18573800
H	-2.23022600	-2.55363500	-0.47372900
H	-3.70391500	-3.47343200	-0.16262400
H	-2.76109100	-2.85050600	1.19789400
C	2.23813700	-0.76265900	-0.04888300
C	2.93366600	-1.23945000	-1.15643800
F	2.29064900	-1.65158200	-2.25864700
C	4.32173700	-1.34399900	-1.20645800
F	4.92576900	-1.82848200	-2.29342500
C	5.08054400	-0.94081500	-0.12099100
F	6.40870400	-1.03546700	-0.15264100
C	4.43906300	-0.43775800	1.00267100
F	5.15758500	-0.03401800	2.05189100
C	3.05486100	-0.35147400	1.00703700
F	2.49495100	0.17314700	2.10896500
C	0.61245500	1.19013000	0.05397300
C	0.41240500	2.01407400	1.15983400
F	0.08510900	1.50188000	2.35821700
C	0.48686500	3.40371200	1.11905600
F	0.23539800	4.12847000	2.21127800
C	0.82900600	4.03623900	-0.06461800
F	0.90686000	5.36391800	-0.12501300
C	1.10953200	3.26238400	-1.18199100
F	1.48312700	3.85149700	-2.31828700
C	1.02699800	1.87851000	-1.09128700
F	1.39861800	1.20495800	-2.18830800
C	-0.20282900	-1.02730400	-1.26090800
C	-0.94657400	-0.37113000	-2.24089300
C	-1.46000100	-1.32674200	-3.16311200
C	-1.02418100	-2.55215000	-2.72752900
N	-0.28245700	-2.36729300	-1.58825400
H	-0.99079400	0.70166100	-2.36830400
H	-2.03623300	-1.13347800	-4.05724900
H	-1.16709700	-3.54315500	-3.13417700
C	0.32747900	-3.46945600	-0.86913600
H	0.36447500	-4.33570100	-1.53138400
H	1.34536100	-3.21144300	-0.57630500
H	-0.23928900	-3.72225400	0.03232100
H	-2.34826900	-0.29982300	-0.60026700

N20_TS

B	1.06627000	0.02579300	0.06627100
C	0.05341900	-0.20911200	-1.21515200
C	0.53985300	-0.91477600	-2.32895400
H	1.52036900	-1.37581900	-2.26794900
C	-0.16180900	-1.02676800	-3.52346300

H	0.26969800	-1.58099300	-4.35178300
C	-1.40408400	-0.41589300	-3.65784400
H	-1.96091100	-0.47977900	-4.58735800
C	-1.91944900	0.28776300	-2.57829600
H	-2.89069100	0.77085100	-2.66644800
C	-1.21804800	0.38929100	-1.37026800
C	-1.87462200	1.19626100	-0.27445300
H	-2.42814900	2.02508100	-0.72662700
H	-1.11948900	1.61219600	0.39022800
N	-2.82738400	0.42059100	0.60566800
C	-3.37680600	1.28463000	1.73527900
C	-4.25824600	2.39776000	1.15946900
H	-4.63048700	3.00285500	1.99275900
H	-3.63651900	3.06566900	0.55599100
C	-5.41454300	1.85244600	0.34134700
C	-5.08659700	0.58588700	-0.41667300
C	-6.07012900	0.11453700	-1.28905500
C	-5.92581300	-1.09830000	-1.93650800
H	-6.68826400	-1.45529600	-2.62111300
C	-4.79924600	-1.86624700	-1.67668700
H	-4.69423100	-2.83094400	-2.15908700
C	-3.78928800	-1.43646000	-0.81542600
C	-3.91935100	-0.16822100	-0.20316300
C	-2.65025400	-2.39226100	-0.51758800
H	-1.74307200	-1.80264400	-0.41158300
C	-2.35041300	-3.39518800	-1.63423600
H	-1.39580800	-3.88359800	-1.42255400
H	-2.26328200	-2.89114200	-2.60029600
H	-3.11452300	-4.17652300	-1.70736200
C	-2.95418900	-3.13108300	0.79345300
H	-2.11978300	-3.78265100	1.07527500
H	-3.84771200	-3.75225400	0.67163100
H	-3.14496100	-2.43261500	1.61612100
C	-2.26333400	1.96135600	2.54085100
H	-1.77174400	2.75952200	1.98395100
H	-1.51005600	1.26161700	2.90070700
H	-2.73232800	2.41775900	3.41768600
C	-4.16285100	0.35674800	2.66652300
H	-3.48380100	-0.36296100	3.13360800
H	-4.94564400	-0.19760600	2.14394700
H	-4.62865300	0.94615200	3.46138800
C	2.21512200	-1.17094800	0.14664700
C	3.57354400	-0.97733600	0.38347400
F	4.07964000	0.26165300	0.52973700
C	4.50097400	-2.00701800	0.49881200
F	5.78772600	-1.73780000	0.71832700
C	4.07964700	-3.32177400	0.38310600
F	4.94823400	-4.32409200	0.48991800
C	2.73484900	-3.57746900	0.16149100
F	2.30662100	-4.83548500	0.05295300
C	1.84721900	-2.51521200	0.05785600
F	0.56056000	-2.86331700	-0.14818300
C	1.70241400	1.51916700	-0.24156900
C	1.32471400	2.70922600	0.36548500
F	0.45762200	2.70516400	1.40782400
C	1.78360300	3.96734800	-0.00931000
F	1.36964800	5.06008400	0.63581200
C	2.67741000	4.07426000	-1.06041700
F	3.13705600	5.26416700	-1.44030700
C	3.09117500	2.91864100	-1.71002800
F	3.95639700	3.00353000	-2.71949000
C	2.59830300	1.68946200	-1.29892900
F	3.04713700	0.62181500	-1.97238400
H	-6.96058400	0.71951400	-1.44074400
H	-6.25461800	1.61549800	1.00688500
H	-5.78772500	2.61175700	-0.35308500
C	0.36495400	-0.17066600	1.53390100
C	-0.77868100	-1.00122200	1.81542500
C	-0.84015900	-1.14772800	3.26439600
C	0.14139300	-0.37822900	3.78134000
N	0.86140300	0.19814400	2.72519400
H	-0.88615700	-1.87909600	1.19008000
H	-1.54679100	-1.74491000	3.82420100
H	0.42429600	-0.16094400	4.80173400
C	2.04996900	1.00257700	2.98019500
H	2.61610600	0.53345500	3.78710300
H	2.66978700	1.03480200	2.08667500
H	1.76915600	2.01726600	3.26745700
H	-1.87259400	-0.37850600	1.26714600

N21_TS

B	-0.76776800	-0.29938500	0.17167200
C	-0.05085200	-0.79297000	1.57588100
C	-0.67288400	-1.78565400	2.34770300
H	-1.65745700	-2.13646000	2.05396600
C	-0.08351600	-2.38374200	3.45849300
H	-0.62089500	-3.15151700	4.00710400
C	1.19013000	-1.99820000	3.85216100

H	1.67452900	-2.45658500	4.70829400
C	1.83113600	-0.99512700	3.13381100
H	2.82275500	-0.66992100	3.43943200
C	1.23524800	-0.38752600	2.02427800
C	2.01446700	0.76133600	1.41135500
H	2.90299200	0.96035100	2.02218100
H	1.40175100	1.66241200	1.43081800
N	2.43340200	0.56478600	-0.00245500
C	2.45901700	1.80986200	-0.78407900
C	3.69977700	2.62976000	-0.47003300
H	3.74459700	2.82194700	0.61021100
H	3.65338300	3.60477300	-0.96466400
C	4.95165700	1.87281700	-0.91984100
H	5.02324300	1.97256300	-2.01292700
C	4.83570200	0.38657500	-0.60855700
C	5.93827200	-0.45761300	-0.78005000
H	6.86867500	-0.03892800	-1.15228100
C	5.88361000	-1.81160800	-0.48304700
H	6.76513900	-2.43121000	-0.62402300
C	4.70561700	-2.38338100	0.00358300
C	3.59644100	-1.56021000	0.15598700
H	2.66668200	-1.96986100	0.53159300
C	3.64489100	-0.19965500	-0.15433500
C	6.20110500	2.50556500	-0.30024800
H	6.21564800	3.58071500	-0.50230000
H	7.12541700	2.08174100	-0.69778500
H	6.20052400	2.36022000	0.78435500
C	4.61440900	-3.85421700	0.31757400
H	3.84718600	-4.04650600	1.07209500
H	5.56604700	-4.24013300	0.69075000
H	4.35495200	-4.42985900	-0.57749000
C	-1.06368700	1.32605000	0.05593500
C	-0.96933000	2.24588500	1.10171400
F	-0.55195600	1.87426600	2.32014300
C	-1.29052600	3.59468900	0.98248400
F	-1.13959800	4.41896100	2.01909500
C	-1.79508200	4.07823800	-0.21384700
F	-2.11165800	5.36357600	-0.34530700
C	-1.98414800	3.19460300	-1.26519400
F	-2.51225200	3.62549900	-2.41103600
C	-1.64583000	1.85764900	-1.09903000
F	-1.94999900	1.05829600	-2.13626500
C	-2.27911200	-0.94372100	-0.00635900
C	-3.25353800	-0.58353900	0.92769400
F	-2.91268600	0.18395400	1.97435600
C	-4.58318900	-0.97162300	0.85626600
F	-5.45860100	-0.59610900	1.78848600
C	-5.00185700	-1.75884300	-0.20827800
F	-6.27275700	-2.14296600	-0.30317000
C	-4.07976300	-2.13763100	-1.16860500
F	-4.46464000	-2.89371300	-2.19849600
C	-2.75555400	-1.72379900	-1.05285300
F	-1.94153400	-2.14020200	-2.03992400
C	0.27080100	-0.76586400	-1.08263000
C	0.40743900	-0.21546900	-2.39952000
C	0.84028900	-1.18313000	-3.28564500
C	0.92722600	-2.36649600	-2.54704700
N	0.60749700	-2.14149900	-1.27573900
H	0.26606300	0.83253100	-2.62282200
H	1.07681400	-1.07032500	-4.33257100
H	1.20946500	-3.35881200	-2.87547200
C	0.41671500	-3.21754100	-0.31598200
H	0.78318700	-4.14218100	-0.76440800
H	0.94797800	-3.02307900	0.61546800
H	-0.64763600	-3.31957000	-0.08801100
H	1.28663600	-0.21341200	-0.60903000
H	1.53054900	2.35093900	-0.57775500
H	2.45495300	1.52718400	-1.84509200

P1_TS

P	0.75231618	-0.52826829	0.14960028
C	-0.03564892	-1.05872422	-1.39753878
C	-1.44013570	-0.97002072	-1.43123148
B	-2.30374423	-0.40300572	-0.18086519
C	0.70307804	-1.55607064	-2.47690565
C	0.04166182	-1.96817115	-3.62651606
C	-1.34982142	-1.87758114	-3.69032036
C	-2.07371612	-1.38374723	-2.61024650
O	-3.75311029	-0.39930327	-0.45475635
O	-1.94052516	1.01616068	0.11792117
C	-3.12378921	1.66293741	0.27218986
C	-3.33258332	2.96475153	0.68049917
C	-4.65945670	3.41919535	0.75998785
C	-5.72114798	2.58496530	0.43584464
C	-5.50255074	1.26199166	0.01804952
C	-4.19753597	0.81818457	-0.05996112
H	-2.49183978	3.60391012	0.92832195
H	-4.85290426	4.43739197	1.08205056

H -6.73825121 2.95699168 0.50635952
H -6.32247507 0.60109544 -0.24045885
C 1.47174370 1.11722586 -0.10418222
C 2.10765501 -1.68814587 0.49583918
C 3.37111629 -1.58261848 -0.09718233
C 4.34540327 -2.54024263 0.16541806
C 4.06560133 -3.60641738 1.01816181
C 2.81293644 -3.71298634 1.61588248
C 1.83642732 -2.75414165 1.36119299
H 3.59298258 -0.74535394 -0.75371200
H 5.32435114 -2.45435928 -0.29499555
H 4.82771521 -4.35190392 1.22111559
H 2.59791277 -4.53797325 2.28706149
H 0.86113251 -2.82634788 1.84077409
C 2.56586306 1.54360392 0.65685238
C 3.05584234 2.83617950 0.50324794
C 2.44984707 3.70975332 -0.39665085
C 1.35048464 3.29090322 -1.14170924
C 0.85642591 1.99861806 -0.99942988
H 3.03701181 0.86605059 1.36372828
H 3.90850367 3.16161960 1.09035607
H 2.83207143 4.71885463 -0.51291772
H 0.87168645 3.97321493 -1.83627207
H -0.01487895 1.67593908 -1.56065137
H 1.78601077 -1.62928984 -2.41500806
H 0.60513279 -2.35704164 -4.46869177
H -1.86826383 -2.19759388 -4.58960945
H -3.15702053 -1.31266442 -2.65825106
C -2.10162546 -2.94087023 2.70097657
C -2.19775835 -1.78000166 3.45638759
C -2.13235588 -0.71400053 2.55023435
C -1.96768281 -1.22187917 1.24596085
N -1.97064347 -2.61231726 1.39636796
H -2.12625353 -3.97805135 3.00871743
H -2.30520322 -1.73219407 4.52975006
H -2.15859572 0.34421075 2.77544132
C -1.89374084 -3.56904489 0.30545151
H -2.66215960 -3.34580832 -0.43732785
H -0.91531160 -3.53052307 -0.18358340
H -2.05695563 -4.57002325 0.70751279
H -0.61141024 -0.81547060 1.08199611

P1_P

P -4.21277866 2.34052804 -1.29731743
C -4.90499764 0.91510019 -0.43923964
C -4.22737993 0.32639217 0.64330725
B -2.75123541 0.76462422 1.18935000
C -6.17179647 0.47691028 -0.86626663
C -6.79961584 -0.55889269 -0.19425750
C -6.16037886 -1.15368449 0.89439321
C -4.90312337 -0.71947012 1.28966880
O -2.88570681 1.23473985 2.61669896
O -2.30170246 2.00913880 0.44592183
C -1.95772180 2.91542617 1.39655288
C -1.40234365 4.16397957 1.20735523
C -1.23610988 4.98509966 2.33397940
C -1.61887250 4.54613610 3.59547989
C -2.17504671 3.27048425 3.78358073
C -2.33477369 2.46112494 2.67240480
H -1.12430117 4.49768903 0.21281224
H -0.80190132 5.97246558 2.21362331
H -1.48023040 5.19456518 4.45510044
H -2.47807194 2.91859489 4.76370375
C -4.34954742 3.86878376 -0.35795383
C -5.13155640 2.58492258 -2.84260965
C -6.21343870 3.46635398 -2.91901826
C -6.92112255 3.59025962 -4.11106636
C -6.55124113 2.83881888 -5.22357237
C -5.47258033 1.96000668 -5.15010893
C -4.76210127 1.83211354 -3.96165920
H -6.49541589 4.05589226 -2.05132590
H -7.75914419 4.27660191 -4.17122006
H -7.10336019 2.93932311 -6.15232166
H -5.18368262 1.37668349 -6.01784574
H -3.92279043 1.14315865 -3.90388641
C -3.99497585 5.08014376 -0.95972283
C -3.99873201 6.24681331 -0.20431118
C -4.35022040 6.20193057 1.14384198
C -4.71178866 4.99673575 1.73715387
C -4.71290843 3.82389448 0.98990557
H -3.71321365 5.11246391 -2.00939900
H -3.71492579 7.18742742 -0.66434234
H -4.33097549 7.11069614 1.73655617
H -4.95674444 4.95649096 2.79266191
H -4.94661303 2.87331286 1.46301505
H -6.66400809 0.94019669 -1.71544008
H -7.77655303 -0.90208854 -0.51752822
H -6.64738631 -1.96380400 1.42939720

H	-4.41674603	-1.19905396	2.13467543
C	-0.43197179	-2.26844604	1.22319039
C	-0.10447447	-1.74885786	-0.00638585
C	-0.87721270	-0.56993184	-0.16488724
C	-1.65958947	-0.39332647	0.96411772
N	-1.36850431	-1.45118053	1.80089059
H	-0.06604336	-3.13946605	1.74883770
H	0.62059837	-2.15976215	-0.69454755
H	-0.85499701	0.11183280	-1.00497612
C	-1.93808559	-1.69597643	3.11061258
H	-2.39923763	-0.77746836	3.47341550
H	-2.68445986	-2.49862912	3.07826022
H	-1.14792450	-1.99117806	3.80643950
H	-2.91212041	2.11753692	-1.75153705

P2_TS

C	-1.44941513	-0.06871429	2.64630812
C	-0.72598560	-0.11731808	1.44805814
C	0.63250270	-0.48202587	1.40261024
C	1.23531674	-0.79082117	2.63103756
C	0.53172215	-0.74219146	3.82959911
C	-0.81622751	-0.37900537	3.84217685
H	-2.50356507	0.19741210	2.64092566
H	2.28244017	-1.08378738	2.62197711
H	1.02927965	-0.99360604	4.76230764
H	-1.36791009	-0.34502487	4.77636727
B	1.49030131	-0.54057893	-0.00301140
P	-1.50690975	0.19694050	-0.16174198
C	-3.19917471	-0.46885658	-0.05954638
C	-4.27338147	0.23370653	0.49836608
C	-3.39865727	-1.77142508	-0.52972888
C	-5.52506860	-0.36634138	0.59407666
H	-4.12948161	1.25214029	0.84998208
C	-4.65076611	-2.37044807	-0.42648441
H	-2.56968236	-2.31225583	-0.98444170
C	-5.71359267	-1.66837480	0.13500446
H	-6.35445749	0.18265343	1.02843277
H	-4.79769063	-3.38093058	-0.79391796
H	-6.69155224	-2.13292276	0.21020666
C	-1.64522502	1.99350292	-0.39526521
C	-2.64058397	2.53173403	-1.21830044
C	-0.68422080	2.83488685	0.17503563
C	-2.68643222	3.90285661	-1.44955887
H	-3.38322664	1.88186346	-1.67343939
C	-0.73342614	4.20357807	-0.06692328
H	0.10836451	2.40686654	0.78063982
C	-1.73409915	4.74018933	-0.87355991
H	-3.46474810	4.31584443	-2.08325767
H	0.01677373	4.85150100	0.37531093
H	-1.76958566	5.80930209	-1.05748359
O	2.89491304	-0.85480484	0.19538245
O	1.44146678	0.77093330	-0.66659742
C	2.76940000	1.14687863	-1.00958575
C	2.88529054	2.66343462	-0.90376656
H	3.91262562	2.99525653	-1.09082392
H	2.23093556	3.12954628	-1.64669907
H	2.57764339	3.01436999	0.08366571
C	3.06654000	0.71485700	-2.44840149
H	3.04920216	-0.37488616	-2.53405677
H	2.29735301	1.13278355	-3.10559144
H	4.04207057	1.07763614	-2.78717257
C	3.63445140	0.34783082	0.02268191
C	5.03453402	-0.00453743	-0.46585250
H	5.60219551	0.89863097	-0.71604641
H	5.57179269	-0.53841647	0.32311937
H	4.99121430	-0.65143507	-1.34344932
C	3.73834656	1.08325473	1.36395282
H	2.75140909	1.38898022	1.72342983
H	4.17622799	0.40642950	2.10340623
H	4.37588452	1.97014423	1.29014593
H	-0.39560255	-0.83852879	-0.98896804
C	0.73223342	-1.64385088	-1.04804508
C	0.65069593	-1.64083714	-2.45900529
C	0.23442129	-2.89869441	-2.90938014
C	0.09450906	-3.68689464	-1.77504308
N	0.38104371	-2.94910713	-0.68171963
H	0.81185731	-0.75350114	-3.05560884
H	0.05055636	-3.21220042	-3.92609012
H	-0.18199224	-4.72892417	-1.68033931
C	0.41162901	-3.47529593	0.67449187
H	0.27786068	-4.55714153	0.62860295
H	1.37595252	-3.24538786	1.13115518
H	-0.38025885	-3.03352680	1.28491868

P2_P

C	-1.36066600	-0.30050300	2.58096200
C	-0.57785600	-0.28985000	1.41712200
C	0.79628900	-0.58407700	1.40970000

C	1.35753300	-0.87368200	2.66303100
C	0.60430700	-0.88167400	3.83234700
C	-0.76281700	-0.59902800	3.79649600
H	-2.42517400	-0.08145300	2.53929700
H	2.42434700	-1.08122900	2.70098700
H	1.08083800	-1.10668400	4.78276300
H	-1.35386800	-0.61045700	4.70640000
B	1.71316800	-0.56693600	0.03217400
P	-1.38323100	0.17844000	-0.12153700
C	-2.99772600	-0.61814900	-0.25895600
C	-4.16213500	-0.05003000	0.26774900
C	-3.03792400	-1.87008900	-0.88258700
C	-5.36628300	-0.74033900	0.17167000
H	-4.13007600	0.92927900	0.73832500
C	-4.24749200	-2.55106600	-0.97502000
H	-2.12928400	-2.30778200	-1.29173200
C	-5.40738800	-1.98745000	-0.44859000
H	-6.27265900	-0.30300000	0.57718300
H	-4.28310800	-3.52024900	-1.46122700
H	-6.34975100	-2.52065000	-0.52487600
C	-1.59025100	1.96615100	-0.25251800
C	-2.51742900	2.50318700	-1.15196600
C	-0.78319700	2.80252600	0.52096400
C	-2.63596100	3.88354500	-1.27052800
H	-3.14312800	1.85036100	-1.75425500
C	-0.91255600	4.18245600	0.39850900
H	-0.05273600	2.36995000	1.19846600
C	-1.83597100	4.72069900	-0.49460700
H	-3.35162600	4.30428000	-1.96896500
H	-0.28646200	4.83592400	0.99716800
H	-1.93219000	5.79781300	-0.58885300
O	3.14873600	-0.46193600	0.34043600
O	1.36929000	0.68581600	-0.69819800
C	2.58887900	1.32233800	-1.05724000
C	2.36132600	2.82664000	-1.13545200
H	3.29321400	3.35496900	-1.36670400
H	1.63877000	3.04912200	-1.92769200
H	1.96226900	3.22035800	-0.19830400
C	3.03959000	0.80032900	-2.42478400
H	3.24592700	-0.27227600	-2.38034200
H	2.23355100	0.96177800	-3.14750100
H	3.93230900	1.32083300	-2.78735100
C	3.55871300	0.86982600	0.08494100
C	5.03128200	0.86164700	-0.30841700
H	5.36518700	1.85328900	-0.63388400
H	5.63963600	0.56611100	0.55223400
H	5.21496400	0.14516200	-1.11149300
C	3.37510100	1.71050200	1.35388400
H	2.31646500	1.79026400	1.62097800
H	3.89453500	1.21584200	2.18034700
H	3.78746100	2.71942800	1.24525200
H	-0.65043500	-0.32405500	-1.19278700
C	1.38803900	-1.88529300	-0.87453500
C	0.81306700	-1.99437100	-2.13591000
C	0.70888300	-3.37272900	-2.47790400
C	1.23002500	-4.07660700	-1.41722000
N	1.63410300	-3.17649500	-0.46622600
H	0.54976100	-1.15064100	-2.76324000
H	0.32789400	-3.79802700	-3.39649500
H	1.36418300	-5.13857500	-1.26378000
C	2.24935000	-3.53760400	0.79599600
H	2.73270700	-4.51191900	0.69479500
H	3.00448700	-2.78978500	1.04700900
H	1.51130600	-3.58930400	1.60331700

P3_TS

C	1.42801573	-0.45525537	-0.23735703
C	0.07249712	-0.46158026	-0.21460409
C	2.23808858	-0.85283216	-1.43327448
C	2.86537110	-2.09452422	-1.57240470
C	2.40841973	0.10726222	-2.43943267
C	3.65838227	-2.36011410	-2.68692919
H	2.72270952	-2.86330906	-0.82232951
C	3.19932849	-0.16061346	-3.55071802
H	1.91214321	1.06862853	-2.33565811
C	3.83406667	-1.39447492	-3.67325584
H	4.13576126	-3.32995168	-2.78464753
H	3.32170211	0.59540051	-4.31957532
H	4.45460601	-1.60564521	-4.53819549
C	-0.50932577	-1.16401645	-1.41134068
C	-0.93295010	-0.50143831	-2.55897496
C	-0.48907815	-2.55764640	-1.48176529
C	-1.45466812	-1.17131013	-3.65751638
C	-1.01022526	-3.25525700	-2.56153288
C	-1.51186316	-2.55658417	-3.65090216
F	0.11520418	-3.27712060	-0.52821650
F	-0.99295111	-4.58627275	-2.57665217
F	-2.00579734	-3.21099246	-4.69664737

F	-1.85583469	-0.49363611	-4.72971766
F	-0.75620520	0.81751104	-2.67911671
B	-0.94388774	0.32756293	0.87452846
P	2.37951815	0.22204343	1.16299503
C	3.65521001	1.33102808	0.46415633
C	3.53015364	2.72110522	0.54042492
C	4.74631486	0.76978269	-0.21580687
C	4.48273871	3.53879383	-0.06416410
H	2.69882873	3.17319384	1.06532559
C	5.69263702	1.59266496	-0.81335491
H	4.84645629	-0.30957589	-0.28946603
C	5.56128929	2.97835081	-0.73933949
H	4.37358946	4.61656181	-0.00304904
H	6.52919280	1.14911612	-1.34316246
H	6.30060198	3.61949592	-1.20886646
C	3.30614323	-1.18101506	1.86291026
C	2.81279595	-2.48638283	1.77219178
C	4.47115288	-0.93591865	2.59945548
C	3.48016092	-3.53247059	2.40367608
H	1.91553155	-2.68774007	1.19309633
C	5.13504963	-1.98404420	3.22673650
H	4.86385227	0.07454786	2.67263142
C	4.63987752	-3.28306317	3.13120985
H	3.09272595	-4.54277356	2.32248992
H	6.03973580	-1.78579409	3.79195084
H	5.15873379	-4.09950324	3.62289582
C	-2.47243744	-0.29072237	0.98136943
C	-2.69029468	-1.66356797	1.06314627
C	-3.63067051	0.46126280	1.17835946
C	-3.92976141	-2.26039674	1.25199228
C	-4.89354013	-0.08383572	1.37522564
C	-5.04997228	-1.46031639	1.40607279
C	-0.95595328	1.88082119	0.29509856
C	-0.07745902	2.88234817	0.68848179
C	-1.80116262	2.27009008	-0.74447572
C	0.01310942	4.13854388	0.10366740
C	-1.76196914	3.51281560	-1.36023792
C	-0.83837396	4.45974794	-0.93971910
F	-4.04215074	-3.58767855	1.30684822
F	-1.65108671	-2.51105336	0.98184826
F	-3.59467401	1.80838845	1.20525720
F	-5.95246196	0.70853081	1.54389810
F	-6.25039395	-2.00237793	1.59390228
F	-2.71724546	1.41866482	-1.22313511
F	-2.59530398	3.80163578	-2.35679570
F	-0.77794867	5.65344598	-1.52261227
F	0.91276307	5.02333891	0.54663096
F	0.76565393	2.68493559	1.73285139
C	-0.65566409	0.16552512	4.65552574
C	-0.17710675	-1.10015003	4.34657165
C	-0.02693436	-1.12426188	2.96198795
C	-0.35907970	0.14271766	2.41456111
N	-0.78408416	0.89136375	3.52731887
H	-0.90870565	0.59672409	5.61495859
H	0.02887987	-1.89278121	5.04962198
H	0.29404742	-1.96357324	2.36365264
C	-1.36337983	2.22857693	3.52542719
H	-1.96840896	2.33772195	4.42620005
H	-0.58735880	2.99480175	3.51564318
H	-2.00906871	2.34911166	2.65669310
H	1.04932387	0.46326883	2.10481840

P3_P

C	-1.34240000	-0.40864100	0.25433500
C	0.01255000	-0.37196600	0.25298600
C	-2.14925600	-0.77427500	1.46960900
C	-2.78761900	-2.00881400	1.62221600
C	-2.29028300	0.19410500	2.47215900
C	-3.55429000	-2.26647000	2.75722500
H	-2.67553700	-2.77588900	0.86490400
C	-3.05817900	-0.06446200	3.60198800
H	-1.78972600	1.15200100	2.35250000
C	-3.69841200	-1.29453200	3.74334300
H	-4.03739500	-3.23203400	2.86905000
H	-3.15938400	0.69543800	4.37026600
H	-4.29870400	-1.49889400	4.62426800
C	0.59988500	-0.99239100	1.49694100
C	1.16859200	-0.27854300	2.54858300
C	0.50601500	-2.37371800	1.69094500
C	1.70699900	-0.89539500	3.67073800
C	1.03964200	-3.01615000	2.79633800
C	1.65708400	-2.27330900	3.79133200
F	-0.16247700	-3.14015500	0.82204000
F	0.93329800	-4.33791700	2.92248700
F	2.16429500	-2.87489900	4.86295300
F	2.23367600	-0.15906300	4.64737100
F	1.14079800	1.05785700	2.56671000
B	1.03372700	0.38391000	-0.84419000

P	-2.46025000	0.05732800	-1.10717000
C	-3.84519300	1.05562200	-0.49443400
C	-3.93370100	2.38330400	-0.92527900
C	-4.83211900	0.52831200	0.34830000
C	-4.98876600	3.18480900	-0.49813400
H	-3.18119800	2.79570200	-1.59052100
C	-5.88059700	1.33550400	0.77177100
H	-4.78220300	-0.50582500	0.67580900
C	-5.95781800	2.66294000	0.35265000
H	-5.05013400	4.21500000	-0.83242400
H	-6.63844800	0.92584300	1.43123900
H	-6.77923100	3.28907200	0.68636900
C	-3.17000400	-1.45161300	-1.81462000
C	-2.50263000	-2.67437400	-1.69964600
C	-4.37389700	-1.36661900	-2.51894600
C	-3.04646500	-3.81147800	-2.28710000
H	-1.56882200	-2.73560000	-1.14687600
C	-4.90569600	-2.50777800	-3.11204800
H	-4.89569800	-0.41727400	-2.60289200
C	-4.24400300	-3.72746100	-2.99456600
H	-2.53207100	-4.76202900	-2.19310400
H	-5.83922200	-2.44332200	-3.66106000
H	-4.66423700	-4.61686000	-3.45322100
C	2.46455000	-0.38029500	-1.13885800
C	2.65007100	-1.75505400	-1.11192800
C	3.53162100	0.31332700	-1.70909600
C	3.81396300	-2.39893900	-1.51767200
C	4.71312600	-0.27638100	-2.12789000
C	4.86165300	-1.65193600	-2.02410400
C	1.23773100	1.86286200	-0.12277800
C	0.18109400	2.76161700	-0.03780600
C	2.37971100	2.29645000	0.54992700
C	0.23239500	4.00175100	0.58068000
C	2.48977900	3.52903400	1.18011600
C	1.40836600	4.39524300	1.19633400
F	3.91595200	-3.72852400	-1.44067100
F	1.65149700	-2.56779500	-0.71779000
F	3.44658000	1.64425700	-1.89843200
F	5.69970000	0.46093100	-2.64366300
F	5.98739000	-2.24281400	-2.42447900
F	3.44323200	1.49380800	0.69247700
F	3.61962600	3.87047800	1.80175300
F	1.49308300	5.57793900	1.80218600
F	-0.83300700	4.80833800	0.57768900
F	-1.00857900	2.46226900	-0.61156400
C	-0.65412800	0.96395200	-4.25693500
C	-0.77675000	-0.40117600	-4.14314400
C	-0.13489000	-0.75777700	-2.93198400
C	0.34333600	0.39274100	-2.31282900
N	0.03936800	1.43407400	-3.16470400
H	-0.94989800	1.64041400	-5.04672500
H	-1.26407800	-1.05952100	-4.84887900
H	-0.01807600	-1.75467600	-2.52801500
C	0.49066000	2.81541500	-3.08065600
H	-0.24109600	3.45431300	-2.57904400
H	1.43943600	2.85636600	-2.54733700
H	0.64945000	3.19046700	-4.09326100
H	-1.96011100	0.86226500	-2.12960400

P4_TS

C	0.50960855	0.06083150	-1.97147237
H	0.94891230	0.68343848	-2.75913999
H	0.51288678	-0.97811807	-2.31293694
C	-0.93276723	0.45130187	-1.59403291
H	-0.93364554	1.44743253	-1.12891433
H	-1.49098857	0.57057935	-2.53072907
P	1.49536065	0.16307365	-0.41697630
B	-1.70215002	-0.70705417	-0.71756059
C	-3.27250782	-0.32443870	-0.50639833
C	-4.31176777	-1.15496191	-0.91366216
C	-3.68118919	0.87023773	0.07414307
C	-5.65592614	-0.83228824	-0.75674087
C	-5.00657813	1.23764658	0.25623424
C	-6.00584830	0.37369135	-0.16896104
C	2.84591770	-1.07169708	-0.25071536
C	3.30837352	-1.25597532	1.07769260
C	3.35550411	-1.89647574	-1.27507835
C	4.26854493	-2.22708391	1.34249108
C	4.31323210	-2.86352298	-0.95310293
C	4.78515109	-3.04669616	0.34025505
H	4.62059669	-2.35037403	2.36436705
H	4.70378881	-3.48973608	-1.75209524
C	2.17139235	1.86363778	-0.26913060
C	3.23671322	2.33294952	-1.06840306
C	1.57914510	2.72143042	0.68092818
C	3.69449139	3.63577313	-0.88836474
C	2.07146219	4.02068981	0.82055068
C	3.12913046	4.49497045	0.05245597

H	4.51491373	3.99220126	-1.50734285
H	1.60829841	4.67905891	1.55132217
C	3.88488313	1.48960736	-2.13484033
H	4.29228428	0.55944402	-1.73013931
H	3.16213574	1.22782001	-2.91391943
H	4.69812131	2.03801466	-2.61378287
C	3.66253334	5.89162497	0.23307939
H	3.81437307	6.38160965	-0.73234119
H	2.97974933	6.50392217	0.82559853
H	4.62917522	5.87304780	0.74672888
C	0.42043714	2.29680662	1.55014501
H	-0.45555389	2.02370676	0.95472377
H	0.67070846	1.42843682	2.16796219
H	0.12904497	3.11234856	2.21513977
C	2.92583145	-1.83341454	-2.72179829
H	2.03275237	-2.44377648	-2.89708295
H	2.70149901	-0.82174835	-3.05477138
H	3.71812324	-2.23102433	-3.35981535
C	5.82388857	-4.08877500	0.66004293
H	6.73077316	-3.62550093	1.06006146
H	5.45555977	-4.78856023	1.41607426
H	6.09913825	-4.66161462	-0.22772184
C	2.77832087	-0.44349119	2.23358618
H	2.89292461	0.63171117	2.06291438
H	1.71187695	-0.64339867	2.39952197
H	3.31368360	-0.69690852	3.15064506
F	-6.61411001	-1.66812396	-1.16530557
F	-7.28923858	0.69898130	-0.00974868
F	-5.32927282	2.40044822	0.82900115
F	-2.75557475	1.75103879	0.52301849
F	-4.05554005	-2.33929103	-1.48775175
H	0.11540003	-0.36540153	0.53226328
C	-1.02162823	-1.03562588	0.79579512
C	-1.36132860	-0.50232983	2.06772967
C	-1.02465028	-1.40968063	3.07150734
C	-0.49540486	-2.52515054	2.42587219
N	-0.50609005	-2.31669713	1.10007591
H	-1.80212347	0.47619078	2.20302378
H	-1.14155869	-1.29315474	4.13875046
H	-0.10542640	-3.44674675	2.83777042
C	-0.05169654	-3.27868710	0.11308680
H	0.48003710	-4.08222389	0.62502098
H	-0.90038361	-3.67895456	-0.44430264
H	0.63242183	-2.79892540	-0.59189520
H	-1.62301868	-1.74325415	-1.34263007

P4_P

C	0.61648900	-0.47140700	-2.09748700
H	0.99283700	0.19961800	-2.87755700
H	1.16721200	-1.41112500	-2.13896700
C	-0.89694700	-0.74217000	-2.19333200
H	-1.44088100	0.20713200	-2.07600300
H	-1.07491700	-1.03093800	-3.23642000
P	0.97161700	0.33356500	-0.49760900
B	-1.46174700	-1.94627700	-1.16034200
C	-2.53210600	-1.27628800	-0.09750300
C	-3.75011500	-0.85791400	-0.64629600
C	-2.42412400	-0.99318200	1.25665900
C	-4.76457500	-0.23420500	0.06612500
C	-3.40594300	-0.36788000	2.01847200
C	-4.59380000	0.01138200	1.42092800
C	2.50504000	-0.04798900	0.40915600
C	2.52989800	0.33367900	1.76935000
C	3.61586500	-0.70027300	-0.16753400
C	3.67952000	0.08202000	2.51763900
C	4.74008100	-0.91781900	0.62645200
C	4.79718000	-0.53495200	1.96537600
H	3.69414200	0.37875400	3.56322500
H	5.59871300	-1.41440100	0.18084900
C	0.75695400	2.13691900	-0.68912000
C	1.74861000	2.88900300	-1.36045000
C	-0.39534600	2.77648000	-0.18164600
C	1.57253200	4.26256100	-1.49749600
C	-0.51811300	4.15810600	-0.34729200
C	0.44875900	4.91836900	-0.99601500
H	2.33814700	4.83743700	-2.01323000
H	-1.40507700	4.64947600	0.04370500
C	2.98745500	2.26991000	-1.95619900
H	3.59296600	1.75521900	-1.20487000
H	2.73865300	1.54976900	-2.74250800
H	3.60986700	3.04068700	-2.41439400
C	0.29723100	6.40538500	-1.16338400
H	1.11637700	6.93765700	-0.67004500
H	0.32027600	6.68193300	-2.22190700
H	-0.64389400	6.76134900	-0.73945800
C	-1.51940500	2.05346900	0.51927800
H	-2.01711200	1.34165400	-0.14629400
H	-1.17750100	1.49997100	1.40038400

H	-2.27019900	2.77000100	0.85793100
C	3.65378900	-1.23299700	-1.57716700
H	3.08213800	-2.16740200	-1.63317800
H	3.25296100	-0.53637200	-2.31535800
H	4.68463000	-1.45711400	-1.85915900
C	6.02598100	-0.81185100	2.78774300
H	6.09232200	-1.87682500	3.03341000
H	6.93386000	-0.54590100	2.23942500
H	6.01302100	-0.25173600	3.72517900
C	1.35446000	0.97232500	2.46512500
H	1.05143600	1.90762900	1.98411000
H	0.48887200	0.29983100	2.48632800
H	1.61137700	1.20376000	3.50008400
F	-5.90110600	0.13192400	-0.53418200
F	-5.54904600	0.61815500	2.12835000
F	-3.19264100	-0.09857200	3.31146400
F	-1.27294400	-1.22970500	1.92859000
F	-3.98694000	-1.02618500	-1.95768100
H	-0.05817800	-0.06848300	0.35185300
C	-0.21590200	-2.83553200	-0.61548900
C	0.64403000	-2.84301400	0.48014200
C	1.69283800	-3.77787100	0.25745300
C	1.46544200	-4.33085900	-0.97825600
N	0.32997700	-3.76129400	-1.49722000
H	0.52658600	-2.25040700	1.37409500
H	2.50895700	-4.01751200	0.92551000
H	2.00019900	-5.09259200	-1.52881500
C	-0.21232200	-4.13101400	-2.79104600
H	0.46777700	-4.84534000	-3.25894400
H	-1.19732100	-4.59090900	-2.68803900
H	-0.30917100	-3.25889100	-3.44353400
H	-2.16109000	-2.68384800	-1.82499800

P5_TS

P	-1.70175600	-0.63784000	-0.40940900
B	1.45175500	-0.48774800	-0.29206900
C	-0.71007800	-1.12489000	-1.89210000
C	0.80933500	-0.82002500	-1.77914400
C	-3.09071600	-1.83983000	-0.35861800
C	-5.02094000	-2.88446900	-1.38427600
C	-5.15236400	-3.76976000	-0.31222100
C	-4.24868800	-3.66146700	0.73362200
C	-3.22049500	-2.71045900	0.74114900
C	-2.40977400	1.06569100	-0.43786300
C	-2.16744400	2.02731800	-1.44732200
C	-2.64373200	3.32826900	-1.26836300
C	-3.34252200	3.72516800	-0.13370100
C	-3.59343200	2.76078700	0.83585700
C	-3.13884800	1.44899100	0.71488100
C	3.09786400	-0.59738100	-0.34646000
C	3.72191400	-1.73503900	-0.86551000
C	5.09819600	-1.90303200	-0.94903300
C	5.93900200	-0.90317800	-0.48555800
C	5.37840200	0.23766400	0.06357700
C	3.99454400	0.36177200	0.12293900
C	0.94129400	1.03523600	0.15379900
C	1.24656900	2.11474700	-0.68076900
C	0.78566000	3.40882700	-0.49654000
C	-0.00871600	3.70032900	0.60465700
C	-0.30655000	2.68797200	1.49545800
C	0.16596300	1.40310900	1.24909600
C	-6.24738500	-4.80307300	-0.30457700
C	-2.35847800	-2.69112900	1.98494700
C	-1.43169600	1.76383600	-2.74069600
C	-3.76280300	5.15659600	0.06044000
C	-3.42478600	0.51013100	1.86108200
F	2.99746300	-2.77225400	-1.32045800
F	5.61612100	-3.01699800	-1.46915000
F	7.26093700	-1.04402600	-0.55701300
F	6.16564000	1.20569100	0.53533400
F	3.55832300	1.48915400	0.71655100
F	2.03649500	1.92412900	-1.75065300
F	1.07567800	4.36961500	-1.37494600
F	-0.49884900	4.92744400	0.77936200
F	-1.08862000	2.92521100	2.55099700
F	-0.25402800	0.48439000	2.15138300
H	-1.13282800	-0.75694500	-2.82429100
H	-0.88088100	-2.20738400	-1.90094000
H	1.05203500	0.01672200	-2.43816500
H	1.32609900	-1.68036200	-2.21080500
H	-5.72891300	-2.94166400	-2.20843200
H	-4.33990600	-4.33002900	1.58738700
H	-4.13508300	3.04047300	1.73602000
H	-7.22377000	-4.33967200	-0.47228900
H	-6.09365600	-5.53774800	-1.10096800
H	-6.28192400	-5.33844600	0.64637400
H	-1.56041400	-1.95140500	1.96800300
H	-1.89607300	-3.67016400	2.13739200

H	-2.98746900	-2.48236600	2.85699600
H	-1.27794400	2.70673400	-3.26892200
H	-0.44887200	1.31958800	-2.58362700
H	-2.00378300	1.10554000	-3.40326500
H	-3.96482300	5.64469500	-0.89584500
H	-2.96072100	5.71182000	0.55650300
H	-4.65772700	5.22734900	0.68297100
H	-2.50615100	0.04968900	2.23210700
H	-3.87425200	1.06194300	2.68862500
H	-4.11384900	-0.28854700	1.57075400
C	-4.01302700	-1.92899600	-1.43105600
C	-3.94943000	-1.02816500	-2.64085200
H	-3.77640400	0.01514000	-2.36470700
H	-3.14983600	-1.33776200	-3.32155200
H	-4.88776400	-1.07825100	-3.19645000
H	-2.43672800	4.06376600	-2.04260900
H	-0.36933000	-1.17002800	0.57679400
C	1.39938800	-3.04940400	2.49855100
C	0.93414000	-3.86948400	1.47762800
C	0.67289000	-3.02614600	0.40041900
C	0.93261100	-1.67781600	0.75980600
N	1.40247900	-1.76900100	2.08637500
H	1.74150200	-3.30602600	3.49290600
H	0.81253500	-4.94118700	1.52678100
H	0.30600200	-3.33395100	-0.56930100
C	2.03458800	-0.70991800	2.86555100
H	2.96451200	-1.09406700	3.28926100
H	2.27485400	0.13203400	2.21878200
H	1.37661600	-0.36803600	3.66591700

P5_P

P	-1.89817800	-0.66039000	-0.41419500
B	1.57001800	-0.69835100	-0.09697500
C	-0.68108200	-1.46712600	-1.51069200
C	0.78891500	-1.01870100	-1.52590300
C	-3.38737000	-1.70046800	-0.45929600
C	-5.44058100	-2.38687400	-1.51471900
C	-5.65363500	-3.35095200	-0.52879000
C	-4.70568900	-3.48347900	0.48235000
C	-3.56748700	-2.67767600	0.54358400
C	-2.34211700	1.10190800	-0.51040800
C	-2.02928100	1.95157700	-1.58528300
C	-2.43727200	3.28822800	-1.52084100
C	-3.12809900	3.80358000	-0.43180300
C	-3.42630300	2.94100800	0.62549100
C	-3.05112800	1.60260500	0.61385100
C	3.20164400	-0.57353600	-0.42154500
C	3.86434000	-1.63147500	-1.04900900
C	5.22593100	-1.65571700	-1.31932400
C	6.01774500	-0.58323800	-0.94317100
C	5.42249000	0.48613900	-0.29803300
C	4.05381600	0.46561400	-0.05421200
C	0.96259900	0.76479500	0.42860000
C	1.11158700	1.89465800	-0.37832700
C	0.56530000	3.13810100	-0.10854400
C	-0.16631700	3.31910500	1.05449100
C	-0.32533100	2.24782600	1.91297100
C	0.22531700	1.01326300	1.58309000
C	-6.88048500	-4.22121600	-0.56721800
C	-2.59739800	-2.88860700	1.68048600
C	-1.23983300	1.55630200	-2.80729500
C	-3.49023800	5.26023400	-0.35792700
C	-3.37104300	0.76240900	1.82612200
F	3.19574900	-2.73585100	-1.42627500
F	5.78016600	-2.70863800	-1.92822700
F	7.32952200	-0.58763200	-1.18685200
F	6.16725800	1.52477400	0.09422700
F	3.59299200	1.54120100	0.61749000
F	1.83444100	1.82105700	-1.51285600
F	0.69762800	4.14554800	-0.97548400
F	-0.73753400	4.49351800	1.32405600
F	-1.07818300	2.38716300	3.00972800
F	-0.07117200	0.01980600	2.45255000
H	-1.12682500	-1.51540100	-2.51054500
H	-0.76662100	-2.48220800	-1.10500500
H	0.88160800	-0.14578600	-2.17883800
H	1.30287500	-1.83002300	-2.05064900
H	-6.16387700	-2.27995200	-2.31978700
H	-4.84947700	-4.23906900	1.25008600
H	-3.95483400	3.32714800	1.49340200
H	-6.83786700	-5.00304300	0.19391800
H	-7.78281200	-3.62664100	-0.39146800
H	-6.99183400	-4.69927900	-1.54466500
H	-2.57319800	-2.02535400	2.35616900
H	-1.56980000	-3.06425700	1.34275100
H	-2.89843800	-3.75511100	2.27197500
H	-0.23186100	1.98198100	-2.75222500
H	-1.13647800	0.48271300	-2.94092500

H	-1.71672700	1.96003900	-3.70472500
H	-4.42723800	5.41120700	0.18366000
H	-3.58777500	5.69890300	-1.35354100
H	-2.70484500	5.80646900	0.17437800
H	-3.92906700	1.35443000	2.55306200
H	-3.97714700	-0.11296000	1.57288100
H	-2.46014900	0.41538200	2.32675300
C	-4.32368200	-1.55543500	-1.50443800
C	-4.17170500	-0.53937500	-2.60944900
H	-4.42102600	0.46921300	-2.26515400
H	-3.15456800	-0.50794700	-3.01278800
H	-4.83943100	-0.78556000	-3.43760700
H	-2.18311800	3.94307000	-2.35132600
H	-1.37388000	-0.83942000	0.86609200
C	1.78766600	-3.12167300	2.84801500
C	1.18867500	-3.97160100	1.95102800
C	0.97347700	-3.21564300	0.76613600
C	1.43121700	-1.91740800	0.96285500
N	1.93976900	-1.90039100	2.24841800
H	2.12453000	-3.28009200	3.86354100
H	0.94465600	-5.01085300	2.12289300
H	0.59520300	-3.60979300	-0.16784100
C	2.61048800	-0.79339300	2.90223300
H	3.63387600	-1.07482400	3.16797700
H	2.65694400	0.06340500	2.23220800
H	2.07480100	-0.49712000	3.80855800

P6_TS

P	1.85892266	-0.37483903	0.65201024
C	1.11487389	-0.22816987	2.33545181
H	1.91449475	-0.18860143	3.07949766
H	0.53048081	0.68724508	2.40047402
C	0.17921231	-1.42379246	2.57455476
H	0.61482452	-2.32333592	2.11865309
H	0.18044178	-1.62754519	3.65161356
C	-1.27437861	-1.18738788	2.12174425
H	-1.68502378	-0.41834389	2.78650325
H	-1.82322613	-2.09726773	2.37472143
B	-1.64629912	-0.74614576	0.56638291
C	2.43694862	1.21352052	-0.08054125
C	2.36737652	2.49159370	0.52036598
C	1.70908080	2.79458170	1.84616931
H	1.82030702	3.85605242	2.07512729
H	2.13219043	2.23243931	2.68084405
H	0.63715539	2.58207606	1.81199212
C	2.90002183	3.58356197	-0.16536029
H	2.84211294	4.56426296	0.30129974
C	3.47457665	3.47241354	-1.42835836
C	3.98847405	4.68892870	-2.14946034
H	4.67480421	4.41475297	-2.95347734
H	4.50796218	5.36530951	-1.46602279
H	3.15562316	5.24417402	-2.59319190
C	3.49704670	2.21566831	-2.01931685
H	3.91291041	2.10246922	-3.01755791
C	2.97979030	1.09046543	-1.37940397
C	2.98974157	-0.21558221	-2.13581972
H	1.98973908	-0.66386754	-2.14928649
H	3.68135017	-0.94269526	-1.69878528
H	3.29144549	-0.04653325	-3.17101918
C	3.42299207	-1.33876727	0.91665920
C	4.51056507	-0.77178448	1.62700675
C	4.52849442	0.63153229	2.18051790
H	5.34391750	0.73720275	2.89843303
H	3.60319186	0.89470279	2.69234709
H	4.67765807	1.36629087	1.38378473
C	5.67120999	-1.52311365	1.80650880
H	6.49540174	-1.07773529	2.35900827
C	5.81681839	-2.80906462	1.29435637
C	7.07751961	-3.59821974	1.52633765
H	7.04491354	-4.09864480	2.49954820
H	7.95668942	-2.94944279	1.51922455
H	7.20934920	-4.36693024	0.76181615
C	4.75351076	-3.33905099	0.57539220
H	4.84262698	-4.33483876	0.14769114
C	3.56396219	-2.63447366	0.37921226
C	2.48002866	-3.32890647	-0.39835576
H	1.92954648	-2.65887570	-1.05973563
H	1.74957073	-3.77927199	0.27790470
H	2.90100874	-4.13317754	-1.00600481
C	-1.35220242	0.88009409	0.36246573
C	-0.58071137	1.49182369	-0.61811312
F	0.11975358	0.75504941	-1.50931833
C	-0.46093935	2.86731574	-0.79851287
F	0.31773142	3.35418494	-1.76542417
C	-1.14021560	3.72231605	0.04896545
F	-1.03523938	5.04279515	-0.08825685
C	-1.93812126	3.17700753	1.04861472
F	-2.63014195	3.98375357	1.85280942

C	-2.02263577	1.79873301	1.18078928
F	-2.88812586	1.35958836	2.10715402
C	-3.26347978	-1.04651182	0.27529188
C	-3.83763873	-2.28567128	0.57992836
F	-3.10960999	-3.27759798	1.12285588
C	-5.16705924	-2.61742360	0.34795201
F	-5.63152564	-3.82313747	0.68092500
C	-6.01375291	-1.69125637	-0.23836969
F	-7.28994382	-1.98933326	-0.47398094
C	-5.49953965	-0.45526703	-0.58948455
F	-6.28421759	0.44806994	-1.17983321
C	-4.16359615	-0.16812289	-0.33185546
F	-3.77454541	1.05061695	-0.76388309
C	-1.00914476	-3.01831193	-2.37016056
C	-0.69784361	-3.89149876	-1.33952289
C	-0.62445370	-3.10498434	-0.18981789
C	-0.84392944	-1.74129844	-0.49996967
N	-1.10856774	-1.76243778	-1.89230077
H	-1.18314999	-3.21872999	-3.41966837
H	-0.54949940	-4.95737813	-1.42805753
H	-0.44802848	-3.46878147	0.81235252
C	-1.70214209	-0.70346865	-2.70251834
H	-2.15941588	0.04313285	-2.05691763
H	-0.95775207	-0.21964866	-3.33651802
H	-2.48486510	-1.14185941	-3.32438924
H	0.53788310	-1.17162973	-0.14149363

P6_P

P	2.08791700	-0.35841300	0.84262300
C	1.37647500	-0.82761500	2.46365700
H	2.19431900	-1.33505800	2.98437700
H	1.07738300	0.02647000	3.06231100
C	0.19442600	-1.77914700	2.22672100
H	0.49073200	-2.55458300	1.51341700
H	0.05506100	-2.29917800	3.18190100
C	-1.13413500	-1.09885800	1.83000400
H	-1.18616200	-0.15076200	2.37858200
H	-1.91332100	-1.71020100	2.29941700
B	-1.68292400	-0.86750100	0.26196700
C	2.41472800	1.39185400	0.44394100
C	1.92076000	2.47066100	1.21288300
C	1.03186900	2.34717000	2.42745400
H	0.58935100	3.31982100	2.65160500
H	1.58884700	2.03124400	3.31621100
H	0.20326400	1.65366400	2.26898000
C	2.22943800	3.77038900	0.80666800
H	1.84097100	4.59947700	1.39287600
C	2.97878500	4.04301200	-0.33299600
C	3.23601500	5.45646200	-0.77289300
H	4.18857500	5.54172700	-1.30120600
H	3.24385500	6.14404500	0.07585900
H	2.44438900	5.77853600	-1.45739000
C	3.41361400	2.96472600	-1.09915800
H	3.96109400	3.15251400	-2.01931700
C	3.14571000	1.64509100	-0.74370200
C	3.60683100	0.56808300	-1.69581100
H	2.82513700	-0.17245400	-1.88590300
H	4.49274600	0.04372300	-1.32680600
H	3.85668500	1.01925500	-2.65753600
C	3.55260900	-1.40322600	0.65720100
C	4.70740900	-1.07968900	1.39934200
C	4.79504000	0.15553300	2.26257300
H	5.62041700	0.06335800	2.97137600
H	3.88422000	0.33301900	2.84513000
H	4.97028700	1.05082900	1.65651600
C	5.81512700	-1.91482900	1.30079500
H	6.71043100	-1.67024800	1.86772500
C	5.80423900	-3.05356900	0.49278100
C	7.01672500	-3.94114200	0.41593400
H	7.19339500	-4.43811500	1.37517400
H	7.91309700	-3.36039700	0.18027600
H	6.89792800	-4.71310100	-0.34702900
C	4.64510300	-3.35256500	-0.21936400
H	4.62347600	-4.24115400	-0.84463000
C	3.50139000	-2.55231000	-0.15975000
C	2.29753600	-2.96029400	-0.97929400
H	2.05980500	-2.22542900	-1.75558000
H	1.38828900	-3.09705300	-0.38316200
H	2.49726900	-3.90912300	-1.48006400
C	-1.04714200	0.54571900	-0.39924700
C	-0.14505400	0.67768600	-1.45409500
F	0.45366000	-0.40973100	-1.99751600
C	0.29927900	1.88093100	-1.99138300
F	1.17479000	1.88564400	-3.00217200
C	-0.10936500	3.06993500	-1.41966200
F	0.35142800	4.23966200	-1.86520600
C	-0.96145700	3.01599000	-0.32749300
F	-1.30596800	4.14649700	0.29526200

C	-1.40419400	1.78641100	0.14183800
F	-2.18582100	1.84890400	1.23230000
C	-3.35757500	-0.60673700	0.40367300
C	-4.03706600	-0.20835000	1.56142200
F	-3.39759900	0.08828700	2.70731900
C	-5.41745800	-0.06986600	1.66235500
F	-5.97376600	0.30941200	2.81865200
C	-6.22194400	-0.31821600	0.56576800
F	-7.54699300	-0.18578900	0.64378500
C	-5.61301600	-0.69840000	-0.61507100
F	-6.35427700	-0.93562500	-1.70282700
C	-4.22998100	-0.82552100	-0.66280000
F	-3.76225300	-1.18278000	-1.87031600
C	-1.14355200	-3.89917100	-2.12069100
C	-1.14164200	-4.52275000	-0.89834700
C	-1.31830600	-3.49631900	0.06232200
C	-1.42243700	-2.25941200	-0.57365600
N	-1.32035200	-2.55356500	-1.93047800
H	-1.05229600	-4.29665800	-3.12261600
H	-1.02585500	-5.58280700	-0.71965800
H	-1.38260900	-3.64345200	1.13169600
C	-1.51843200	-1.70293500	-3.09475900
H	-1.74570300	-0.68545100	-2.79318100
H	-0.62474400	-1.70365600	-3.72328200
H	-2.36598900	-2.07444800	-3.67609500
H	1.18305300	-0.85842400	-0.09229900

P7_TS

P	-1.99795080	-0.31599845	-0.18938906
B	1.50418169	0.22227330	0.07971570
C	-1.04723835	0.30629597	-2.97339506
H	-1.76715282	-0.27707019	-3.55451948
C	-0.75753124	-0.24616214	-1.56129092
H	-0.39706722	-1.26690403	-1.66522309
C	0.41421438	0.67338615	-1.08300271
H	-0.08826106	1.50553097	-0.56608086
C	0.92371217	1.27632882	-2.43076659
H	1.99802228	1.47585896	-2.45443716
C	0.02960280	2.49348624	-2.72103481
H	0.35264651	3.01591684	-3.62763181
H	0.03203303	3.21591485	-1.89823742
C	-1.36526448	1.82316596	-2.92960680
H	-2.07364980	2.07639573	-2.13597550
H	-1.82038723	2.13752091	-3.87275150
C	0.39783129	0.31132354	-3.51114550
H	0.47003014	0.74045962	-4.51604667
H	0.86356675	-0.67359716	-3.50449950
C	-3.51219333	0.70924113	-0.46017430
C	-4.56304533	0.29179637	-1.30652515
C	-5.70739333	1.08301710	-1.40645155
H	-6.50768597	0.75585596	-2.06623119
C	-5.85476005	2.27183771	-0.69839287
C	-4.80321920	2.67624867	0.11826532
H	-4.88392894	3.61137350	0.66725104
C	-3.63686400	1.92375137	0.24918548
C	-4.51631857	-0.96657922	-2.13139097
H	-5.30148013	-0.94847671	-2.88959782
H	-4.65482847	-1.86036270	-1.51645332
H	-3.55979410	-1.06475065	-2.64633221
C	-7.11766190	3.08487587	-0.80027106
H	-7.84608358	2.76092496	-0.04961512
H	-7.58283263	2.97153111	-1.78218277
H	-6.91950433	4.14586422	-0.63210483
C	-2.53215846	2.47011044	1.11132828
H	-2.87826829	3.33720525	1.67714773
H	-1.68363677	2.78944926	0.49910135
H	-2.15003321	1.72701595	1.81515487
C	-2.58442533	-2.02260579	0.17487394
C	-3.10766427	-2.17332893	1.48309945
C	-3.54373070	-3.42303881	1.91269686
H	-3.94668718	-3.52035043	2.91821945
C	-3.46043949	-4.55046303	1.09934598
C	-2.90976860	-4.39491628	-0.16696473
H	-2.81747289	-5.26554849	-0.81232326
C	-2.46299785	-3.16118770	-0.65041529
C	-3.14918384	-1.03521147	2.47412515
H	-3.68399422	-0.16108405	2.09025335
H	-2.13020502	-0.71850886	2.73294784
H	-3.64421435	-1.35394668	3.39333610
C	-3.96517339	-5.88656291	1.57552734
H	-3.52946956	-6.70500006	0.99854309
H	-5.05341730	-5.94693239	1.47116197
H	-3.72856320	-6.04452361	2.63085971
C	-1.84309697	-3.18423671	-2.02966715
H	-2.23999341	-4.02909166	-2.59651526
H	-0.75570293	-3.31691427	-1.97229869
H	-2.02684561	-2.27929986	-2.60544673
C	2.73882429	-0.82177421	-0.26985096

F	4.04153269	0.05970341	1.51139887
C	3.88828789	-0.83961181	0.51967589
F	6.00021499	-1.68883080	1.16513881
C	4.92654817	-1.74752704	0.37587857
F	5.81925653	-3.62160118	-0.75042356
C	4.83718323	-2.73545889	-0.59419278
F	3.57300961	-3.75277023	-2.30284440
C	3.70077029	-2.79388054	-1.38300636
F	1.59125560	-2.04348574	-1.95978668
C	2.69272183	-1.85505288	-1.19501571
C	2.10591631	1.71466400	0.47929288
F	4.05640844	1.53731048	-0.87411115
C	3.26096443	2.26457613	-0.07448596
F	4.79200550	4.03047761	-0.43449508
C	3.66804957	3.58002364	0.12295229
F	3.27026826	5.69528793	1.09337117
C	2.89504419	4.43332892	0.89440009
F	0.94214828	4.74892662	2.18393244
C	1.71926750	3.95086271	1.44769499
F	0.18486134	2.24046968	1.75368123
C	1.36181632	2.63074285	1.21363839
C	0.68238491	-1.79437205	3.24845540
C	0.31857820	-2.70826214	2.26780877
C	0.35985189	-2.00523127	1.07054104
C	0.68465378	-0.63392880	1.29576606
N	0.91605758	-0.59040236	2.69094791
H	0.78950829	-1.93190248	4.31629023
H	0.05468222	-3.74416166	2.41928138
H	0.16592206	-2.41838144	0.08881937
C	1.47081409	0.50564170	3.47643931
H	1.80585715	0.09694585	4.43005141
H	2.33223319	0.92146347	2.95547888
H	0.73091429	1.28405891	3.65482211
H	-0.69629325	-0.23156391	0.95935985

P7_P

P	-2.15467100	-0.26720400	-0.23181200
B	1.57865000	-0.06675800	0.20375100
C	-0.93138100	-0.23930200	-2.92464200
H	-1.63286700	-0.89174800	-3.45095100
C	-0.77375600	-0.55708500	-1.41733400
H	-0.54395400	-1.61484600	-1.31272200
C	0.45654600	0.32605800	-0.99651900
H	0.01108000	1.25066500	-0.60775700
C	1.04624700	0.71556600	-2.39193500
H	2.12498100	0.87342500	-2.37746400
C	0.23668300	1.90841600	-2.92495900
H	0.66756900	2.29560700	-3.85417100
H	0.19679100	2.73433500	-2.20708400
C	-1.16134900	1.26893200	-3.18352100
H	-1.93982300	1.70081400	-2.55178000
H	-1.47766100	1.41486100	-4.22030700
C	0.54565000	-0.39057400	-3.33696600
H	0.70673000	-0.14173400	-4.39101700
H	0.95113500	-1.38129600	-3.13373700
C	-3.39716800	1.00588300	-0.65825400
C	-4.46647000	0.69776800	-1.52452200
C	-5.44003100	1.66595500	-1.76547300
H	-6.26156500	1.42228200	-2.43419000
C	-5.38340500	2.92942200	-1.18449600
C	-4.30242700	3.22225800	-0.35484400
H	-4.22765800	4.21208700	0.08817200
C	-3.30136300	2.29353200	-0.07589900
C	-4.58334100	-0.62331400	-2.23606200
H	-5.40600700	-0.59583100	-2.95317800
H	-4.76762100	-1.44912300	-1.54295600
H	-3.67158700	-0.84530200	-2.79875000
C	-6.45576100	3.95362900	-1.43581100
H	-6.02566800	4.95046100	-1.56228100
H	-7.14873100	4.00013200	-0.58897600
H	-7.03617000	3.71095900	-2.32866600
C	-2.16636400	2.73597800	0.81442100
H	-2.29073700	3.78741800	1.07955200
H	-1.19782600	2.63683700	0.31515100
H	-2.11690800	2.16623700	1.74657300
C	-2.99168000	-1.74488700	0.43659800
C	-3.54264900	-1.59659500	1.73214900
C	-4.18599800	-2.68451500	2.31615800
H	-4.60715500	-2.56815000	3.31150000
C	-4.29030900	-3.91534700	1.67130700
C	-3.71601100	-4.04369200	0.41052400
H	-3.76785400	-5.00119600	-0.10217100
C	-3.05987500	-2.98722400	-0.22447400
C	-3.40462300	-0.33327400	2.54338000
H	-3.77426800	0.54983600	2.01305900
H	-2.35360200	-0.16539600	2.81260300
H	-3.97019800	-0.41992800	3.47256000
C	-5.01443000	-5.06536200	2.31564100

H	-4.86821400	-5.06581500	3.39867200
H	-4.66890200	-6.02312600	1.91995300
H	-6.09139500	-4.99632700	2.12804900
C	-2.42013200	-3.28940800	-1.55728200
H	-2.93323800	-4.12764800	-2.03355400
H	-1.37217800	-3.58559300	-1.42437500
H	-2.44232200	-2.45239600	-2.25431000
C	2.87720500	-0.95980400	-0.31430900
F	4.56030500	0.27270700	0.84794200
C	4.20492400	-0.76118600	0.05888400
F	6.50297000	-1.33112000	0.08821700
C	5.25349500	-1.59938900	-0.30168500
F	5.98843300	-3.55269600	-1.40942800
C	4.99757200	-2.73285200	-1.05428600
F	3.40798200	-4.11557200	-2.11169800
C	3.68943500	-3.00934600	-1.41568800
F	1.43017100	-2.53329900	-1.35633700
C	2.68119600	-2.14131400	-1.02395600
C	1.98894900	1.49158200	0.62755800
F	3.71174100	1.77448000	-0.99704700
C	2.91063100	2.28993200	-0.04657800
F	3.97147600	4.35339100	-0.52153100
C	3.06024600	3.65685200	0.16264000
F	2.35262200	5.62297600	1.26452500
C	2.23735300	4.30914800	1.06536300
F	0.43635200	4.17888400	2.58666500
C	1.28064300	3.57204900	1.74477600
F	0.18969600	1.58549300	2.18442500
C	1.18335000	2.20801700	1.51110600
C	0.77451700	-1.86069900	3.50689200
C	-0.05931300	-2.57681700	2.68599200
C	0.09118800	-2.01959400	1.38754400
C	0.98735500	-0.95755700	1.43682500
N	1.40901300	-0.90507400	2.75442500
H	0.98504700	-1.95113900	4.56341100
H	-0.68474800	-3.41126500	2.97208900
H	-0.35517600	-2.42059500	0.49071500
C	2.39643700	-0.01293700	3.33297400
H	2.89045800	-0.52750700	4.15989100
H	3.15151600	0.24681700	2.59219000
H	1.93836600	0.90421000	3.71614900
H	-1.50238500	0.26571800	0.87854300

P8_TS

C	-1.24937000	-0.39959200	-0.91321900
H	-1.26170500	0.32895200	-1.72814300
H	-1.14296600	-1.37142100	-1.40538400
P	-2.86636400	-0.47987700	-0.05215600
B	0.01586400	-0.15239000	0.17423600
C	-3.68263300	1.21312500	-0.08869000
C	-2.59090000	2.23513300	0.25698200
H	-1.85526200	2.33310200	-0.54465300
H	-3.05964200	3.21394200	0.40937800
H	-2.05622600	1.97557400	1.17771000
C	-4.74992800	1.27049600	1.01496800
H	-5.17963400	2.27844100	1.04654500
H	-5.56730000	0.56576300	0.85057200
H	-4.30993900	1.05946100	1.99472300
C	-4.29029100	1.58985100	-1.44242500
H	-5.16911000	0.98656000	-1.68394700
H	-4.61054000	2.63825500	-1.41207700
H	-3.56271500	1.48663600	-2.25357000
C	-3.89548600	-1.78293500	-0.94419700
C	-3.26106900	-3.13853700	-0.59367000
H	-2.26556800	-3.24556700	-1.03405300
H	-3.18323000	-3.28234900	0.48936500
H	-3.88385500	-3.94226400	-1.00226800
C	-5.33530600	-1.77877100	-0.41804300
H	-5.36837500	-1.86403600	0.67316000
H	-5.87784400	-0.87717900	-0.71287800
H	-5.87294500	-2.63608600	-0.83905500
C	-3.89155800	-1.62295300	-2.46971100
H	-4.40062800	-2.48329300	-2.92101300
H	-4.41548600	-0.72179700	-2.79244200
H	-2.87435900	-1.58976900	-2.86918400
C	1.40881100	-0.96779600	-0.17756600
C	1.71937000	-1.66977000	-1.33638100
C	2.43379500	-0.92846300	0.76828300
C	2.93494900	-2.31563000	-1.54067100
C	3.65984900	-1.56036500	0.61140700
C	3.91263600	-2.26238700	-0.55975500
F	0.82298200	-1.80188700	-2.32919000
F	3.16538500	-2.98671200	-2.67016500
F	4.59279200	-1.49482800	1.56137600
F	2.24805300	-0.25919300	1.91698700
F	5.07971000	-2.87744000	-0.73847600
C	0.53464900	1.41909900	0.05870900
C	0.74332300	2.39745100	1.02510200

C	0.92150000	1.82520800	-1.22535000
C	1.22985800	3.67431800	0.75641700
C	1.40951000	3.08404700	-1.54403900
C	1.56647600	4.02628800	-0.53849400
F	0.84300500	0.96530000	-2.25685400
F	1.74366900	3.38728500	-2.79828400
F	2.03488800	5.24054200	-0.81504800
F	1.37599300	4.55830500	1.74454900
F	0.45097400	2.18235200	2.31810500
C	-0.36812000	-2.33122100	3.29933300
C	-0.64760700	-1.13446900	3.94907600
C	-0.72253300	-0.16586700	2.95574500
C	-0.51133300	-0.74910400	1.67291800
N	-0.28108100	-2.12040900	1.97918000
H	-0.25472700	-3.32850700	3.70497600
H	-0.79659100	-1.00540900	5.01018900
H	-0.97737000	0.87036800	3.10679900
C	-0.04564800	-3.20101700	1.03264000
H	-0.38557900	-2.90005100	0.04403600
H	1.01890800	-3.44278100	0.97284700
H	-0.60724900	-4.08275300	1.34941100
H	-1.81105600	-0.74117000	1.37266700

P8_P

C	-1.10929700	-0.69158500	-0.80501600
H	-0.98867400	-0.23546700	-1.78970400
H	-1.08965500	-1.77338000	-0.96920200
P	-2.79051000	-0.34138500	-0.24903100
B	0.12928500	-0.32309100	0.28767900
C	-3.59930500	1.08813300	-1.12417400
C	-2.53982200	2.18478800	-1.29481300
H	-1.72852900	1.87584100	-1.95746200
H	-3.02276500	3.06015600	-1.74224400
H	-2.11279900	2.49289700	-0.33484300
C	-4.73273700	1.64683300	-0.25164600
H	-5.14485600	2.53164100	-0.74869000
H	-5.55065500	0.93999300	-0.10705900
H	-4.36655000	1.96064900	0.73091300
C	-4.12905800	0.66674800	-2.49812300
H	-4.98452700	-0.00931500	-2.41667000
H	-4.46324700	1.56151500	-3.03402500
H	-3.35614700	0.18573400	-3.10682800
C	-3.78187600	-1.91484000	-0.22383100
C	-3.10298700	-2.81603000	0.82013800
H	-2.08996200	-3.09497900	0.51903000
H	-3.04036300	-2.34516700	1.80622900
H	-3.69013200	-3.73652700	0.91007900
C	-5.22923000	-1.64875500	0.20079400
H	-5.28777800	-1.09234200	1.14202000
H	-5.79006700	-1.10963000	-0.56726200
H	-5.72714400	-2.61200900	0.35461100
C	-3.75177000	-2.61348400	-1.58887000
H	-4.23764300	-3.58950900	-1.48226100
H	-4.29061800	-2.05880000	-2.35947000
H	-2.73211700	-2.79213000	-1.94189300
C	1.58235500	-0.88073400	-0.26959600
C	1.85270800	-1.50425900	-1.48045000
C	2.69936800	-0.70013100	0.54608100
C	3.11516700	-1.95038000	-1.85537000
C	3.97751100	-1.12793700	0.21592000
C	4.18749700	-1.76258300	-0.99990100
F	0.86714700	-1.75872500	-2.36410000
F	3.30056800	-2.56183900	-3.02906700
F	5.00251400	-0.93372100	1.04873200
F	2.56599400	-0.08978600	1.73326200
F	5.40549900	-2.18451500	-1.33992600
C	0.39747200	1.31568600	0.33539200
C	0.60656200	2.13650500	1.44357800
C	0.60682900	1.97768000	-0.87848300
C	0.88255500	3.50000800	1.36009300
C	0.88123800	3.32920500	-1.01378500
C	1.01487700	4.10745900	0.12488800
F	0.53194400	1.30055200	-2.04430900
F	1.02258000	3.87953100	-2.22283200
F	1.27868000	5.40989500	0.03079100
F	1.04349600	4.22183100	2.47167800
F	0.57430100	1.65917800	2.69276100
C	-0.57480500	-2.79029200	3.11909000
C	-1.15918000	-1.69236300	3.70031600
C	-0.96981400	-0.61478000	2.79723000
C	-0.25963600	-1.05691800	1.68645000
N	-0.04495100	-2.41193500	1.91272400
H	-0.48202800	-3.81169000	3.46062600
H	-1.65088300	-1.66501900	4.66262500
H	-1.28369900	0.40574700	2.96296600
C	0.60030200	-3.37228600	1.03502500
H	0.25002500	-3.26132100	0.00496900
H	1.68884100	-3.26916000	1.04290800

H	0.34059500	-4.37654700	1.37559000
H	-2.76674800	0.06287700	1.09451000

P9_TS

C	0.44161600	-0.45494700	-1.07458500
H	0.56207500	0.18722000	-1.95061800
H	0.36090700	-1.48075700	-1.45156400
B	-0.90875900	-0.01717600	-0.19033300
C	-0.83190700	1.58537000	0.09507600
C	-0.57953000	2.17761900	1.33995500
C	-0.98240200	2.47357300	-0.98622900
C	-0.53741300	3.56042800	1.52016300
C	-0.91816200	3.85570500	-0.83239900
C	-0.70937800	4.40820700	0.43169000
C	-2.28212100	-0.46182200	-0.95915500
C	-3.38383500	0.38758300	-1.13591900
C	-2.43167500	-1.77134800	-1.44406800
C	-4.55603600	-0.03329500	-1.76554200
C	-3.58946600	-2.20600100	-2.07949600
C	-4.66326800	-1.33287300	-2.24606400
P	1.90488100	-0.36837000	0.03738000
C	2.91730300	1.21787200	-0.36167500
C	4.39346400	1.06279300	0.03360700
H	4.93848500	0.33792700	-0.56982800
H	4.88040800	2.03269000	-0.11316900
H	4.50246100	0.79952300	1.09020000
C	2.80706600	1.58872800	-1.84723600
H	3.09422800	0.77898800	-2.52112400
H	1.78879600	1.90139800	-2.09353400
H	3.46761100	2.44229100	-2.04163500
C	2.38856500	2.40864800	0.44952200
H	1.43311300	2.76359200	0.07309900
H	2.28419900	2.17393500	1.51251900
H	3.10751500	3.23088300	0.35294900
C	3.02929500	-1.87090900	-0.36488900
C	4.14511400	-2.03468800	0.68452500
H	4.61456800	-3.01535400	0.54492700
H	4.93233000	-1.28863300	0.61358500
H	3.73338000	-2.00666200	1.69857400
C	3.60213300	-1.76562500	-1.78581400
H	4.27419000	-0.91819200	-1.92192600
H	4.17359600	-2.67475600	-2.00828200
H	2.79776000	-1.68775800	-2.52518200
C	2.23739200	-3.18787900	-0.31071700
H	1.94314800	-3.43755800	0.71075100
H	1.35666600	-3.19376900	-0.95828300
H	2.89873800	-3.98772500	-0.66154200
H	-3.32705400	1.41440800	-0.78089700
H	-5.38451300	0.66019700	-1.88130600
H	-5.57114700	-1.66533400	-2.74048600
H	-3.65905200	-3.22774300	-2.44272200
H	-1.61904800	-2.48423700	-1.30797000
H	-0.37602100	1.54000900	2.19791700
H	-0.34624900	3.97380000	2.50687500
H	-0.67139200	5.48550200	0.56102200
H	-1.04042000	4.50495000	-1.69491000
H	-1.17283500	2.06109300	-1.97658500
C	-0.83392000	-0.91499700	1.23081400
C	-0.50823300	-2.28660600	1.33049500
C	-1.10086000	-2.86168300	2.45601200
C	-1.85127400	-1.85307900	3.03074500
N	-1.70282600	-0.70857400	2.32671500
H	0.12834800	-2.79071000	0.62623000
H	-1.01146500	-3.87688600	2.81165300
H	-2.50711200	-1.88194200	3.89088600
C	-2.64319100	0.39339200	2.52145300
H	-2.89362400	0.82989700	1.55624300
H	-3.55165400	-0.01222900	2.96872100
H	-2.23750300	1.17051800	3.17107500
H	0.63126100	-0.59213200	1.27666200

P9_P

C	0.45104900	-0.35635300	-0.90605500
H	0.51147500	0.35581000	-1.73704300
H	0.37470000	-1.34784400	-1.35936700
B	-0.96943800	0.01152100	-0.04478900
C	-0.97979200	1.63944200	0.12707700
C	-0.81428100	2.26643300	1.37176800
C	-1.04465100	2.49502000	-0.98973000
C	-0.70327800	3.65232700	1.49903300
C	-0.94753300	3.87987700	-0.87975600
C	-0.76710200	4.46717700	0.37248400
C	-2.15853500	-0.57391700	-1.02411500
C	-3.17849600	0.18653700	-1.61349800
C	-2.21068400	-1.95865000	-1.26370800
C	-4.17552500	-0.38736700	-2.40414900
C	-3.19625300	-2.54867000	-2.04956100
C	-4.18736200	-1.75959000	-2.63148700

P	2.05159500	-0.31519000	-0.08076800
C	3.12251500	1.12614800	-0.57948900
C	4.47567400	1.05802600	0.13919200
H	5.09982300	0.22765600	-0.19830700
H	5.02080300	1.98513200	-0.06907300
H	4.35200400	0.98721600	1.22488000
C	3.31452100	1.20460400	-2.09759000
H	3.89871700	0.37247600	-2.49474700
H	2.35854100	1.24567600	-2.62773400
H	3.85469800	2.12922300	-2.32760500
C	2.38751900	2.38682800	-0.09675800
H	1.41795000	2.51759600	-0.58207900
H	2.22983800	2.37936800	0.98611500
H	3.00860600	3.25543700	-0.34160200
C	2.90516900	-1.98226900	-0.15641500
C	3.72505400	-2.17367800	1.12711400
H	4.24908800	-3.13356500	1.06908500
H	4.47678500	-1.39359700	1.26957500
H	3.08196800	-2.19810500	2.01272200
C	3.80384300	-2.07832100	-1.39636400
H	4.66981600	-1.41536500	-1.34815700
H	4.17860300	-3.10506500	-1.46724000
H	3.24738700	-1.86781100	-2.31574200
C	1.85986900	-3.10888500	-0.25807000
H	1.06091400	-3.03646300	0.48313600
H	1.40599500	-3.15029800	-1.25152600
H	2.38151800	-4.05892100	-0.09908500
H	-3.21671200	1.25828500	-1.43427500
H	-4.94925000	0.24162800	-2.83706400
H	-4.96230300	-2.21111400	-3.24418000
H	-3.19903500	-3.62493500	-2.20176100
H	-1.46295600	-2.59890000	-0.79423100
H	-0.76068200	1.64853400	2.26683500
H	-0.56697200	4.09684300	2.48161600
H	-0.68337400	5.54597900	0.46718300
H	-1.00304800	4.50190100	-1.76940900
H	-1.16279700	2.05898100	-1.98117900
C	-1.08592600	-0.72639800	1.40673000
C	-0.21796000	-1.46075600	2.21066500
C	-0.87462100	-1.82453200	3.41788100
C	-2.13909500	-1.30080100	3.33832000
N	-2.25859700	-0.64440700	2.14140800
H	0.80565200	-1.71415900	1.97961700
H	-0.46708100	-2.39134400	4.24336400
H	-2.96984600	-1.33713600	4.02910800
C	-3.49947800	-0.02330400	1.71649200
H	-4.09859300	0.19858600	2.60248200
H	-3.29493500	0.91097900	1.19132000
H	-4.06852000	-0.68115100	1.05308600
H	1.87683600	-0.06401000	1.28684300

P10_TS

P	2.32575800	-0.32616900	0.57761500
B	-1.85686000	-0.19105200	0.57717200
C	2.05180200	-0.06353700	2.39067200
H	1.93726500	1.02195200	2.46420400
H	2.95682500	-0.31729100	2.93748400
C	0.82191900	-0.76171200	3.01356400
H	1.11369000	-1.03260400	4.03421900
H	0.62606700	-1.71513900	2.51390600
C	-0.50229700	0.03496000	3.08321900
H	-0.84933300	-0.04003900	4.12031800
H	-0.31314900	1.09931900	2.92431700
C	-1.66084100	-0.46839100	2.19868200
H	-1.73299200	-1.55797100	2.34853000
H	-2.56785000	-0.07580300	2.66583000
C	3.39172800	1.11938200	0.13919400
C	4.64084700	1.32788700	0.77304700
C	5.37584700	2.47044300	0.46562100
H	6.33826300	2.61647700	0.95168900
C	4.91895200	3.43091700	-0.43542400
C	3.70046500	3.20270100	-1.05957800
H	3.32595700	3.92543500	-1.78133800
C	2.93444900	2.06338800	-0.80116200
C	5.24146300	0.38470000	1.78977200
H	4.88402100	0.61705200	2.79851100
H	5.01603600	-0.66099000	1.57529300
H	6.32779800	0.49544500	1.80057200
C	5.71813900	4.67760300	-0.70661900
H	5.55373200	5.41889400	0.08195200
H	6.78902700	4.46127500	-0.73638700
H	5.43128900	5.13386900	-1.65640800
C	1.68064800	1.90957700	-1.62490700
H	1.94330500	1.68025000	-2.66425000
H	1.02848700	1.11926700	-1.25950900
H	1.12001700	2.84819900	-1.62843500
C	3.30683100	-1.84955500	0.18788400
C	3.63387800	-2.88923700	1.09005100

C	4.26058400	-4.04016400	0.60451500
H	4.49660100	-4.83403700	1.30934300
C	4.59542800	-4.20919200	-0.73302500
C	4.28420900	-3.17242200	-1.60558200
H	4.53383600	-3.27217900	-2.65948300
C	3.65181600	-2.00667600	-1.17873300
C	3.39842600	-2.85395500	2.58289700
H	3.65714300	-3.82169100	3.01593500
H	4.02984300	-2.10517100	3.07187900
H	2.36359700	-2.64476300	2.84852000
C	5.29570500	-5.45272800	-1.21192600
H	5.01518200	-6.32133700	-0.61170600
H	5.05666500	-5.66418600	-2.25678200
H	6.38208100	-5.33693200	-1.13844500
C	3.36512400	-0.96426100	-2.23059300
H	3.50681700	-1.39103000	-3.22594200
H	2.33704200	-0.59732400	-2.16887700
H	4.03042200	-0.10103600	-2.13185700
C	-1.87223100	1.43523200	0.20243200
C	-0.76949400	2.24605100	0.45011800
F	0.37021700	1.71106400	0.94664700
C	-0.67970300	3.60267600	0.16935100
F	0.45988700	4.26477500	0.39205600
C	-1.76418400	4.24677000	-0.39993500
F	-1.71080500	5.54470700	-0.68985700
C	-2.89518800	3.49901400	-0.68705500
F	-3.94317000	4.08541800	-1.26582300
C	-2.92082900	2.13698200	-0.40300600
C	-3.34796500	-0.87253700	0.25720100
C	-4.47172000	-0.41121500	0.95119300
F	-4.37979000	0.69139500	1.71659200
C	-5.73282000	-0.97893000	0.88123400
F	-6.75090100	-0.47455100	1.58083100
C	-5.93877200	-2.07865000	0.05881300
F	-7.14131000	-2.64578200	-0.02451800
C	-4.87603300	-2.56128600	-0.68009300
F	-5.04936400	-3.60162800	-1.49981200
C	-3.62572800	-1.95339800	-0.57285800
F	-2.68638500	-2.49199700	-1.37596200
F	-4.03182100	1.51620200	-0.82721000
H	0.62746100	-0.61193500	0.09148500
C	0.03967200	-1.94318900	-2.22001400
C	0.31037600	-2.85880000	-1.21149200
C	-0.20113600	-2.29831200	-0.04360300
C	-0.71065600	-0.99442800	-0.30461800
N	-0.56854000	-0.86083100	-1.70077700
H	0.24904300	-2.00066500	-3.28063300
H	0.83137600	-3.79786900	-1.32447200
H	-0.15675900	-2.73545800	0.94492300
C	-1.23481000	0.14448400	-2.51522500
H	-2.31557300	0.08296400	-2.35642900
H	-0.89733300	1.14933100	-2.25990500
H	-1.01061600	-0.05825500	-3.56325900

P10_P

P	2.79032600	-0.36482600	0.55145700
B	-2.15972800	-0.37535100	0.43996300
C	2.00245500	-0.09265700	2.18449600
H	1.71379000	0.96095600	2.13364900
H	2.73254100	-0.18598500	2.98629300
C	0.76551800	-1.00156700	2.35983800
H	0.97773100	-1.75899500	3.12060500
H	0.59000500	-1.54764000	1.43370200
C	-0.54495400	-0.27140000	2.72293400
H	-0.69142100	-0.34708700	3.80733600
H	-0.44984500	0.80104900	2.52175200
C	-1.78341300	-0.83556100	1.99698700
H	-1.72553400	-1.93568100	2.02141500
H	-2.64151200	-0.59104500	2.62714800
C	3.60550100	1.16577800	0.01328900
C	4.74753100	1.59870200	0.73364800
C	5.33527000	2.80988800	0.39853900
H	6.21384000	3.13650800	0.94987000
C	4.82615700	3.61905300	-0.62122900
C	3.71386700	3.16996500	-1.31725200
H	3.30771200	3.78092400	-2.12016400
C	3.08444500	1.95068700	-1.03484200
C	5.35010000	0.79926000	1.86153500
H	4.69743100	0.79147100	2.74091600
H	5.55238100	-0.23600600	1.57130300
H	6.29754300	1.24339800	2.17185500
C	5.47975700	4.93336100	-0.94819500
H	4.97689200	5.43290200	-1.77871700
H	5.45667500	5.60388400	-0.08369900
H	6.52995000	4.78883300	-1.21968000
C	1.92126700	1.60047800	-1.93547600
H	2.27081000	1.55325500	-2.97221400
H	1.41600900	0.66301800	-1.71525500

H	1.17474400	2.39833600	-1.87997900
C	3.82871900	-1.84822900	0.32762200
C	4.27167300	-2.68000100	1.37946800
C	5.02620600	-3.80937000	1.05942800
H	5.36587700	-4.44887800	1.86986700
C	5.35380300	-4.14488200	-0.25102400
C	4.91306700	-3.30519600	-1.27043100
H	5.16214200	-3.54276000	-2.30150900
C	4.15725800	-2.16344900	-1.01457600
C	3.99502500	-2.43628800	2.84191200
H	4.46022100	-3.22288100	3.43809000
H	4.40552400	-1.48254300	3.18507000
H	2.92562400	-2.44995800	3.06145200
C	6.13891500	-5.38935500	-0.56037600
H	5.46288300	-6.21917100	-0.79224200
H	6.78807000	-5.24223500	-1.42691600
H	6.75587500	-5.69126600	0.28896300
C	3.72880100	-1.32188300	-2.19031300
H	4.06431800	-1.78491200	-3.12000400
H	2.63820200	-1.23136500	-2.24693200
H	4.15674000	-0.31546800	-2.14711500
C	-2.07808800	1.28427300	0.29791300
C	-0.83177400	1.90574400	0.28793800
F	0.29536800	1.15888700	0.38123700
C	-0.60730600	3.26665300	0.14397900
F	0.64008200	3.75668000	0.12843300
C	-1.69157200	4.11346100	-0.01513600
F	-1.51609000	5.42763700	-0.15908500
C	-2.96058100	3.56088000	-0.02937000
F	-4.02077600	4.35341600	-0.20515000
C	-3.12663500	2.18588400	0.11306800
C	-3.71336100	-0.93485100	0.17814600
C	-4.74514900	-0.65064500	1.07727000
F	-4.55364100	0.23616600	2.07286500
C	-6.01778200	-1.19512600	1.01830500
F	-6.94613000	-0.87242900	1.92551100
C	-6.33237000	-2.07821700	-0.00355100
F	-7.54970200	-2.62077100	-0.07951900
C	-5.36589700	-2.37412200	-0.94477300
F	-5.65348200	-3.20927800	-1.95010800
C	-4.09936800	-1.80070500	-0.84413200
F	-3.26502900	-2.14352900	-1.84220900
F	-4.39875400	1.77518600	-0.00497200
H	1.70138600	-0.58888400	-0.29632800
C	-0.13129600	-1.43726600	-2.63933600
C	0.22977100	-2.47105000	-1.80755800
C	-0.42051600	-2.23030200	-0.56600700
C	-1.15494100	-1.05396100	-0.65056600
N	-0.95694800	-0.59166900	-1.93926600
H	0.11182500	-1.23660500	-3.67410100
H	0.86683300	-3.30585800	-2.06826800
H	-0.39727900	-2.88623100	0.29388400
C	-1.68277800	0.48727500	-2.58409900
H	-2.72543300	0.48392100	-2.25746400
H	-1.24897200	1.46933500	-2.36744400
H	-1.66178700	0.32524600	-3.66340500

P11_TS

P	1.78726200	-0.62145000	0.04033200
B	-1.48267500	-0.34811300	0.03401000
C	0.76074400	-0.80844500	1.59698500
H	0.76390100	0.16193000	2.09039200
C	1.35101200	-1.84985100	2.56677100
H	2.40498400	-1.65086400	2.77960200
H	1.31479300	-2.83923600	2.08960200
C	0.56242700	-1.87942600	3.87780900
H	0.70034800	-0.91691100	4.39078200
H	0.97315200	-2.65119700	4.53813600
C	-0.92724200	-2.10538500	3.63461200
H	-1.08547200	-3.11802700	3.23862300
H	-1.47680900	-2.04428900	4.58075900
C	-1.47236900	-1.09193800	2.62924800
H	-2.54396700	-1.25405600	2.48199700
H	-1.37372300	-0.08231000	3.05044500
C	-0.72680700	-1.15980000	1.28017400
H	-0.71875300	-2.22242400	1.00770500
C	3.23926200	-1.78081200	0.11242700
C	3.25692100	-2.93071400	-0.71017300
C	4.37337400	-3.76441000	-0.71167700
H	4.36730900	-4.64258600	-1.35367900
C	5.49254200	-3.50319800	0.07543100
C	5.46268500	-2.37227200	0.88203800
H	6.32082500	-2.14423000	1.51026100
C	4.36835700	-1.50331600	0.91514200
C	2.10290000	-3.31440700	-1.59458500
H	2.41286300	-4.06494400	-2.32545400
H	1.29334800	-3.75021400	-1.00277000
H	1.68984500	-2.46180300	-2.13798600

C	6.68328300	-4.42275000	0.04604800
H	6.40199300	-5.43535600	0.35157600
H	7.09817200	-4.49269000	-0.96427100
H	7.47325300	-4.07337000	0.71429700
C	4.49694900	-0.30588400	1.82045000
H	3.53803000	0.01793900	2.21989100
H	5.14647800	-0.54788600	2.66548200
H	4.93577600	0.54632500	1.29083200
C	2.59937000	1.02493400	-0.19894600
C	3.21115100	1.16089000	-1.46836100
C	3.93081300	2.31728600	-1.77106500
H	4.40136200	2.39693600	-2.74817000
C	4.04102800	3.37101100	-0.87298700
C	3.38147700	3.24876200	0.34680700
H	3.42241400	4.07784200	1.04986900
C	2.65683300	2.11100100	0.70815600
C	3.08310000	0.11970300	-2.55452400
H	3.48741000	0.50669500	-3.49212700
H	3.62260100	-0.80252500	-2.31667600
H	2.03173100	-0.13951200	-2.72608300
C	4.79414000	4.62762400	-1.21590900
H	5.40519100	4.49360300	-2.11142900
H	4.09917100	5.45289400	-1.40341900
H	5.44939100	4.93294800	-0.39506600
C	1.96341500	2.18129800	2.04972600
H	0.87815400	2.09898900	1.94849500
H	2.28822500	1.40919300	2.75276300
H	2.17095900	3.14653200	2.51664400
C	-3.12368100	-0.62007700	0.00481900
C	-4.09316200	0.31022400	-0.36459100
F	-3.76462300	1.58907000	-0.64168700
C	-5.44850500	0.02824100	-0.49352800
F	-6.30751300	0.98478900	-0.85619000
C	-5.90699200	-1.25345700	-0.24756400
F	-7.20129800	-1.54931600	-0.36879700
C	-4.99223100	-2.22464500	0.12427200
F	-5.40754500	-3.47056800	0.36775100
C	-3.64941200	-1.89266300	0.23738100
F	-2.84666300	-2.90946500	0.60606600
C	-1.15179200	1.26953700	0.16285200
C	-1.76321400	2.03619600	1.16028900
F	-2.69766100	1.48482500	1.95185700
C	-1.52570800	3.38644000	1.36747400
F	-2.16351600	4.05269800	2.33219600
C	-0.62409500	4.05647400	0.55049700
F	-0.37444200	5.35187700	0.73908400
C	-0.00639400	3.35895700	-0.47101300
F	0.85719200	3.97438200	-1.28008700
C	-0.28383700	2.00496700	-0.63556300
F	0.34789400	1.42661900	-1.67727300
C	-1.48412900	-1.65100700	-3.53127400
C	-1.21867100	-2.83707800	-2.86152300
C	-0.93606900	-2.47183600	-1.54784700
C	-0.97192200	-1.05923300	-1.40001300
N	-1.35692800	-0.60992300	-2.68862900
H	-1.76895900	-1.48661700	-4.56273800
H	-1.23270500	-3.82928000	-3.28802100
H	-0.71153200	-3.15412100	-0.74225800
C	-1.79271200	0.72356700	-3.09320300
H	-2.19910500	1.26032000	-2.23785700
H	-0.97466100	1.29713300	-3.53011700
H	-2.58891400	0.61287400	-3.83092300
H	0.43240000	-0.89718700	-1.04039000

P11_p

P	-1.90913500	-0.61322800	-0.16725200
B	1.58442700	-0.56327200	0.20499100
C	-0.63923200	-1.03794700	-1.44979800
H	-0.46480700	-0.09378000	-1.96855200
C	-1.30908700	-2.01819600	-2.43701700
H	-2.30729400	-1.66445300	-2.72674200
H	-1.46156000	-2.98209200	-1.93248700
C	-0.44339800	-2.21775700	-3.67433500
H	-0.35873400	-1.26325900	-4.21303100
H	-0.92413800	-2.92991200	-4.35329800
C	0.94009000	-2.69377400	-3.25698900
H	0.85492700	-3.69032000	-2.80223100
H	1.58958400	-2.79959900	-4.13283800
C	1.57973900	-1.73579100	-2.25159000
H	2.55965600	-2.13389200	-1.99920500
H	1.75005600	-0.76356200	-2.73204200
C	0.76610400	-1.51543700	-0.94958300
H	0.62849500	-2.51232200	-0.50729900
C	-3.31113400	-1.79195000	-0.07597600
C	-3.14085900	-2.93854000	0.73004800
C	-4.20568600	-3.82163000	0.89778100
H	-4.05731300	-4.70273200	1.51695400
C	-5.43545000	-3.61212800	0.28138100

C	-5.57278400	-2.49378800	-0.53730800
H	-6.51684200	-2.32911800	-1.05142700
C	-4.54347600	-1.57285600	-0.73349600
C	-1.82573500	-3.26377200	1.37776300
H	-1.84419400	-4.26618200	1.80959400
H	-1.01826400	-3.23724900	0.64176700
H	-1.55642000	-2.56579400	2.17807800
C	-6.58446700	-4.56042200	0.48503900
H	-7.29528000	-4.15286300	1.21172100
H	-7.12925000	-4.72533500	-0.44823700
H	-6.24066400	-5.52667300	0.86017000
C	-4.83238400	-0.41815100	-1.65995600
H	-4.00865000	-0.22138000	-2.34839300
H	-5.71208100	-0.64898000	-2.26405600
H	-5.03576400	0.50617500	-1.11131900
C	-2.56343600	1.08546600	-0.20529800
C	-3.09263600	1.55380000	1.02220900
C	-3.59964600	2.84984500	1.08513700
H	-3.98865300	3.21111700	2.03343700
C	-3.60872800	3.69369400	-0.02350600
C	-3.10536800	3.20125400	-1.22452400
H	-3.11086900	3.84142000	-2.10336900
C	-2.58129800	1.91330600	-1.34884500
C	-3.09637800	0.73296000	2.28980700
H	-3.67182700	1.24636100	3.06180300
H	-3.54491000	-0.25544800	2.14732400
H	-2.08257200	0.59416200	2.68028900
C	-4.09931400	5.10983000	0.08585800
H	-4.50303500	5.46732200	-0.86424800
H	-4.87094900	5.20776700	0.85294200
H	-3.26672800	5.76516200	0.36210200
C	-2.06874100	1.54305000	-2.72233900
H	-1.00799200	1.79585600	-2.83128000
H	-2.18752000	0.48837100	-2.97361600
H	-2.61194900	2.11782600	-3.47572500
C	3.20443700	-0.37149400	-0.20658100
C	3.93131900	0.80500000	-0.37004800
F	3.34792400	2.01701800	-0.27057500
C	5.30585300	0.86225500	-0.58537500
F	5.91987400	2.04208300	-0.72344400
C	6.04119900	-0.30613000	-0.64271300
F	7.35859700	-0.27512900	-0.85018300
C	5.37972800	-1.51230400	-0.47402600
F	6.06565800	-2.65811700	-0.52549400
C	4.01034800	-1.51493800	-0.25650300
F	3.46277300	-2.73509900	-0.11624800
C	0.85004100	0.93438500	0.33713300
C	0.78965400	1.79445400	-0.75903000
F	1.30475900	1.39699600	-1.94071900
C	0.24722800	3.06798400	-0.74289300
F	0.18514200	3.80133100	-1.85917600
C	-0.29991300	3.55818400	0.43306000
F	-0.86559400	4.76473700	0.46441400
C	-0.28944200	2.75099900	1.55444900
F	-0.87475300	3.16663400	2.68157200
C	0.26544900	1.47644600	1.48140100
F	0.16597500	0.77058500	2.62490200
C	2.39771300	-1.71335000	3.76492600
C	1.75109600	-2.85299800	3.35717200
C	1.31339500	-2.60899300	2.02823200
C	1.68228000	-1.32680100	1.64589100
N	2.36299700	-0.80908000	2.73809000
H	2.88300100	-1.46275300	4.69844600
H	1.61873300	-3.75578100	3.93732400
H	0.84200000	-3.34079000	1.39072100
C	3.01240200	0.48271500	2.84739800
H	2.76482000	1.11124200	1.99488300
H	2.68416300	0.99219700	3.75794700
H	4.10095500	0.36568000	2.87926200
H	-1.27434500	-0.72627000	1.07465500

P12_TS

P	2.26681100	-0.85094700	-0.54319900
C	1.81035500	-1.01822100	-2.31667600
H	2.67061700	-0.72740400	-2.93128300
H	1.66477400	-2.09443500	-2.47342000
C	0.53664700	-0.26175300	-2.72659000
H	0.66043800	0.81122300	-2.54766500
H	0.45502200	-0.37397300	-3.81494400
C	-0.76324700	-0.75350800	-2.07505700
H	-0.84920700	-1.82941600	-2.26323500
H	-1.57909000	-0.30962800	-2.65661400
B	-1.09056000	-0.42677800	-0.47924100
C	3.56335000	-2.09943800	-0.25730800
C	3.33617300	-3.12042700	0.66937200
H	2.39859900	-3.15527700	1.21889000
C	4.31009100	-4.09384000	0.88587600
H	4.13174700	-4.88427700	1.60775200

C	5.50930100	-4.04781500	0.18196000
H	6.26730000	-4.80599700	0.35138500
C	5.74277600	-3.02647200	-0.73916800
H	6.68138600	-2.98730300	-1.28258000
C	4.77502500	-2.05303000	-0.95707600
H	4.96383100	-1.24776300	-1.66282500
C	3.11245500	0.75060400	-0.35488800
C	3.07561200	1.73688000	-1.34572500
H	2.61277900	1.54066300	-2.30705200
C	3.62986600	2.99152500	-1.10117700
H	3.58473500	3.75634000	-1.87037200
C	4.23213300	3.26550600	0.12338800
H	4.65389100	4.24722700	0.31358800
C	4.29633900	2.27535500	1.10264100
H	4.76780500	2.48301700	2.05778900
C	3.73380500	1.02612900	0.86993400
H	3.76599900	0.26523700	1.64612400
C	-0.42879400	1.04266600	-0.06136700
C	-0.72679700	2.14850300	-0.86165100
F	-1.55168900	2.00060800	-1.91217700
C	-0.23833900	3.42881900	-0.65675900
F	-0.55935300	4.42523000	-1.48597800
C	0.58781800	3.67583200	0.43090200
F	1.07936200	4.89493100	0.64778400
C	0.89536100	2.63101600	1.28012000
F	1.67921700	2.83956100	2.34119700
C	0.40739300	1.35659200	1.00647600
F	0.84867100	0.40520100	1.85981500
C	-2.73741200	-0.40891200	-0.28719200
C	-3.49543200	-1.48110100	-0.76137400
F	-2.89649700	-2.52621800	-1.35864800
C	-4.87612600	-1.57117300	-0.65492900
F	-5.52954500	-2.63278800	-1.13465500
C	-5.58094600	-0.54658900	-0.04285100
F	-6.90734000	-0.61173200	0.07301900
C	-4.88223900	0.53744800	0.45797000
F	-5.54024700	1.52895100	1.06508800
C	-3.49814900	0.58246400	0.32739400
F	-2.91592200	1.66396200	0.88426600
C	-0.55157100	-1.68179300	0.47972300
C	-0.30673100	-3.02164500	0.07892200
C	-0.43146100	-3.87945900	1.17046000
C	-0.81282700	-3.07855400	2.24215000
N	-0.88832800	-1.79766700	1.84291400
H	0.00083400	-3.30387800	-0.92022300
H	-0.27135000	-4.94723400	1.19751900
H	-1.04563500	-3.34824600	3.26462000
C	-1.43506500	-0.75640800	2.70452900
H	-2.39787000	-1.08869500	3.09986300
H	-0.75333900	-0.54558400	3.53049100
H	-1.59488900	0.15489100	2.13401100
H	0.78445900	-1.31228300	0.26238200

P12-P

P	2.46962200	-0.76194200	-0.63047500
C	1.76154900	-1.06688400	-2.27294900
H	2.57008100	-0.81844900	-2.97234900
H	1.63092300	-2.15395500	-2.32247600
C	0.45183600	-0.35095700	-2.64594700
H	0.54952000	0.72547900	-2.47850200
H	0.37152400	-0.47392200	-3.73298700
C	-0.82683100	-0.88669000	-1.98896900
H	-0.86354200	-1.96937800	-2.15820700
H	-1.64722400	-0.48099300	-2.59233500
B	-1.18802700	-0.57280400	-0.39134400
C	3.79625000	-1.93947200	-0.32748800
C	3.58496900	-2.98022400	0.58097400
H	2.63750600	-3.06310000	1.10888800
C	4.59562700	-3.91462600	0.79192700
H	4.43943700	-4.72306100	1.49816500
C	5.80023200	-3.80782700	0.10285600
H	6.58433800	-4.53925700	0.27007100
C	6.00815500	-2.76535300	-0.80034100
H	6.95143200	-2.68304500	-1.32988500
C	5.00865500	-1.82459100	-1.01662500
H	5.17484800	-1.00128900	-1.70744100
C	3.12249500	0.91168000	-0.48029300
C	2.96454600	1.85430500	-1.49972400
H	2.48850500	1.58578400	-2.43709100
C	3.42015100	3.15632900	-1.30550500
H	3.28869000	3.89388900	-2.09014200
C	4.02613500	3.51228100	-0.10407400
H	4.36291000	4.53236200	0.04931900
C	4.19650300	2.56600900	0.90625600
H	4.66414000	2.84593000	1.84404900
C	3.74811500	1.26433600	0.72201200
H	3.86399200	0.53177200	1.51709200
C	-0.48200100	0.87140600	0.06309900

C	-0.69279900	2.00268000	-0.72942200
F	-1.48319900	1.91090800	-1.81311600
C	-0.14254300	3.25102700	-0.48976300
F	-0.36521000	4.27386100	-1.31985300
C	0.65710400	3.43712700	0.62980400
F	1.21921500	4.62142600	0.87309200
C	0.87757700	2.36600200	1.47292000
F	1.66159500	2.50975500	2.54624600
C	0.32333600	1.12509300	1.16840900
F	0.67946900	0.13896700	2.02285500
C	-2.84532200	-0.43436800	-0.29209600
C	-3.63825500	-1.46085000	-0.80769900
F	-3.06696800	-2.53686300	-1.37528100
C	-5.02575800	-1.47006000	-0.77601000
F	-5.71596900	-2.49214000	-1.29121000
C	-5.70145100	-0.40530900	-0.20075700
F	-7.03530800	-0.38987800	-0.16038800
C	-4.96813200	0.63598900	0.33892100
F	-5.59828700	1.66785400	0.91013600
C	-3.57874600	0.59832300	0.28415600
F	-2.96650600	1.64257000	0.88018400
C	-0.76626200	-1.82964000	0.55027300
C	-0.07811100	-2.99512100	0.22780500
C	0.00558100	-3.82335500	1.38113800
C	-0.64140500	-3.14719000	2.38836400
N	-1.10158800	-1.96154800	1.88320700
H	0.29723200	-3.24176700	-0.75776500
H	0.46767600	-4.79798900	1.46202200
H	-0.82513700	-3.41297800	3.42032700
C	-1.89329300	-1.02999300	2.66360400
H	-2.95026500	-1.31682700	2.66283200
H	-1.52823100	-1.01796300	3.69313200
H	-1.80859800	-0.02350300	2.25558700
H	1.52020400	-1.01236400	0.36596700

P13-TS

P	-2.71492000	-0.35563300	0.15030100
B	1.25642300	-0.29108800	0.52736200
C	-2.33445100	0.51950100	1.71462700
H	-1.74966800	1.40384200	1.44334700
H	-3.26585400	0.89175500	2.15303500
C	-1.53462100	-0.36085400	2.70655900
H	-2.07178800	-0.33627600	3.65963500
H	-1.55124500	-1.41476900	2.39676000
C	-0.07269900	0.06436600	2.95094300
H	0.14149100	-0.13280900	4.00811600
H	0.00625600	1.15438100	2.83660400
C	1.01072600	-0.65343100	2.12322200
H	0.86375600	-1.73097000	2.25808400
H	1.96504100	-0.44433500	2.62448300
C	-3.82771700	0.74762300	-0.79077700
C	-4.81759000	0.20834700	-1.61995200
H	-4.95379000	-0.86773500	-1.67792900
C	-5.64360800	1.04722900	-2.36092100
H	-6.41195200	0.61869000	-2.99660000
C	-5.48829900	2.42929200	-2.28549100
H	-6.13384600	3.08168700	-2.86478300
C	-4.50251800	2.97069400	-1.46493900
H	-4.37226800	4.04640900	-1.40117700
C	-3.67350400	2.13699200	-0.72081500
H	-2.90766400	2.57975800	-0.09386600
C	-3.74297400	-1.78666100	0.62581600
C	-4.50219700	-1.80017200	1.80126300
H	-4.48637500	-0.94837600	2.47486300
C	-5.28064900	-2.90892200	2.11810900
H	-5.86387400	-2.91344600	3.03319700
C	-5.31282800	-4.00879900	1.26311800
H	-5.92007500	-4.87262700	1.51426700
C	-4.56075000	-4.00284700	0.09172600
H	-4.57917200	-4.86032100	-0.57318600
C	-3.77148300	-2.89919300	-0.22204100
H	-3.16573100	-2.90070700	-1.12443500
C	1.14114500	1.35501000	0.42692800
C	0.12976900	2.09359100	-0.16568400
F	-0.83048500	1.48149200	-0.89987800
C	-0.01031100	3.47510200	-0.06833200
F	-1.04368400	4.09444700	-0.65299300
C	0.91476600	4.19955800	0.65874600
F	0.80358600	5.52184200	0.77924900
C	1.96842200	3.52103500	1.25756200
F	2.87867100	4.20271000	1.95561400
C	2.05473700	2.14341700	1.13185800
F	3.10896700	1.56939500	1.73312500
C	2.77354100	-0.80148400	0.06821000
C	3.68957800	-0.07053900	-0.68481600
F	3.40564500	1.18061700	-1.10383000
C	4.93523100	-0.54193300	-1.08189800
F	5.75743900	0.22880100	-1.79982400

C	5.32566000	-1.82137600	-0.72708300
F	6.51574700	-2.29662000	-1.09611900
C	4.45234600	-2.60264500	0.01153400
F	4.80285100	-3.84499700	0.35707900
C	3.21925500	-2.08553400	0.38530000
F	2.44033700	-2.92932300	1.08650100
C	0.26198000	-1.17912900	-0.45494400
C	-0.14138700	-2.51894300	-0.18736600
C	-0.45561900	-3.16784200	-1.37818800
C	-0.18730000	-2.25525700	-2.39598100
N	0.26927100	-1.11029400	-1.86410200
H	-0.26121400	-2.91823600	0.81111800
H	-0.83565100	-4.17089800	-1.50667100
H	-0.28577100	-2.36283600	-3.46889600
C	0.71874000	0.01269900	-2.67555800
H	1.23628600	-0.37581300	-3.55434400
H	-0.12467400	0.62971100	-2.99023700
H	1.41617100	0.62088700	-2.10143700
H	-1.09559600	-0.84360600	-0.23729900

P13_P

P	2.78377900	-0.40984800	-0.39035700
B	-1.18283900	-0.34036200	-0.26728300
C	2.42039200	0.57404500	-1.87365400
H	1.92670800	1.49582500	-1.54735300
H	3.40028400	0.85340200	-2.27840400
C	1.59781900	-0.16810300	-2.94523100
H	1.85704000	0.30541300	-3.89813800
H	1.94323300	-1.20709200	-3.02643600
C	0.07013600	-0.13021200	-2.79881600
H	-0.31443300	-0.46028800	-3.77043400
H	-0.22944100	0.92089200	-2.71683700
C	-0.58181900	-1.00305500	-1.69503200
H	0.11735900	-1.82059200	-1.46037500
H	-1.41826200	-1.51486800	-2.17903400
C	3.66650700	0.55408200	0.84377100
C	3.45699100	0.23539800	2.18991600
H	2.75577800	-0.55062400	2.46696400
C	4.13647800	0.94802700	3.17119200
H	3.97443500	0.70960600	4.21709200
C	5.01173300	1.97185200	2.81320000
H	5.53527100	2.52791800	3.58421900
C	5.20916600	2.29314700	1.47304300
H	5.88037600	3.09950600	1.19726700
C	4.53487100	1.58618600	0.48143300
H	4.68429200	1.84816800	-0.56235700
C	3.76720600	-1.84898000	-0.85795000
C	5.15939500	-1.75829200	-0.95275700
H	5.66872400	-0.83000600	-0.70754100
C	5.89436200	-2.87131600	-1.34494100
H	6.97488900	-2.80564100	-1.41706500
C	5.24448500	-4.06981800	-1.63483100
H	5.82276900	-4.93806200	-1.93402100
C	3.85886000	-4.16200200	-1.53360800
H	3.35453800	-5.09746600	-1.75105500
C	3.11471400	-3.05243300	-1.14393100
H	2.03422400	-3.12289900	-1.05596100
C	-1.02559900	1.29870200	-0.39817900
C	0.02838000	2.04064000	0.11222500
F	0.98433400	1.44881300	0.86995000
C	0.23500700	3.39706700	-0.11881500
F	1.29567600	4.02050200	0.40616900
C	-0.65721000	4.08834000	-0.91806900
F	-0.48871500	5.38854400	-1.16184800
C	-1.72422100	3.39836500	-1.47709700
F	-2.58180000	4.04259500	-2.27194800
C	-1.87157200	2.04320000	-1.22166000
F	-2.88999100	1.43812700	-1.85173400
C	-2.77613900	-0.78877600	-0.05723200
C	-3.82095000	0.00640100	0.40436200
F	-3.63533000	1.31791200	0.67005800
C	-5.11026200	-0.45257900	0.64669800
F	-6.06090400	0.38197400	1.08025800
C	-5.40977500	-1.78786600	0.44234200
F	-6.64046600	-2.25120700	0.67043600
C	-4.40494300	-2.63599000	0.00521600
F	-4.66640200	-3.93416000	-0.18125700
C	-3.13341400	-2.12792500	-0.22157100
F	-2.21513900	-3.03246500	-0.61036000
C	-0.46954500	-0.99940300	1.05101700
C	0.16298500	-2.23680700	1.16063700
C	0.53198400	-2.45873200	2.51506200
C	0.09791300	-1.35902100	3.21647300
N	-0.50797200	-0.49815200	2.33816500
H	0.28124600	-2.94186400	0.34900200
H	1.02242200	-3.32792600	2.93098000
H	0.15102500	-1.11856300	4.26959300
C	-1.11227300	0.74752100	2.77485900

H	-1.59165900	0.59139800	3.74395000
H	-0.36375400	1.53935400	2.87407400
H	-1.87339200	1.06512800	2.06380000
H	1.59827600	-0.89983200	0.20674800

P14-TS

P	2.93176800	-0.78339400	-0.29675400
B	-0.67219500	-0.23988300	-0.30995600
C	2.43906900	0.37852100	-1.64358100
H	2.32686300	1.33085900	-1.11865100
H	3.26774400	0.52307800	-2.34713600
C	1.13099700	0.08043900	-2.42347400
H	1.40083200	-0.44452000	-3.34719400
H	0.75539000	1.05778300	-2.75102400
C	-0.02848500	-0.70186700	-1.76361500
H	0.25280700	-1.75792700	-1.72726900
H	-0.83963200	-0.67243900	-2.50303700
C	3.63806300	-2.35135900	-1.08593500
C	4.85827200	-2.12435200	-1.98947800
H	5.03750700	-3.03933100	-2.56643100
H	5.77047400	-1.90879900	-1.43793200
H	4.68631900	-1.31788100	-2.71021000
C	3.95419100	-3.34267900	0.04301500
H	3.05648200	-3.56477700	0.63104700
H	4.72341900	-2.98129800	0.72904700
H	4.31356100	-4.28310600	-0.39115300
C	2.55404000	-2.98879800	-1.96760600
H	2.26883700	-2.34822600	-2.80614900
H	1.65960600	-3.25066700	-1.40308700
H	2.95731700	-3.91888000	-2.38419200
C	4.27084900	0.10052300	0.74537600
C	4.23084800	1.62349300	0.54169000
H	3.27120600	2.07374200	0.78685400
H	4.50109100	1.90073100	-0.48187600
H	4.97687100	2.07093500	1.20873800
C	5.72287100	-0.31396800	0.47013200
H	6.36197700	0.22970000	1.17615200
H	6.04295000	-0.03512700	-0.53706700
H	5.91337600	-1.37724900	0.62248100
C	3.93080700	-0.24181600	2.20521600
H	3.97388200	-1.32241600	2.38266900
H	2.92850000	0.10232700	2.47413300
H	4.65221900	0.23884300	2.87647100
C	-0.46911900	1.39050700	-0.22043400
C	0.49562100	2.05787700	0.51426300
F	1.33321800	1.35663700	1.31466900
C	0.70672800	3.43162500	0.48919300
F	1.66982300	3.98424900	1.23469800
C	-0.07611200	4.21754800	-0.33825500
F	0.10601700	5.53624000	-0.39724300
C	-1.05492500	3.60623700	-1.10961700
F	-1.81557200	4.34662100	-1.91838700
C	-1.22269700	2.23131400	-1.04012000
F	-2.18375500	1.71653900	-1.82326000
C	-2.28365400	-0.62734800	-0.21122900
C	-2.77147000	-1.83444000	-0.71035700
F	-1.94802800	-2.70050900	-1.33146200
C	-4.09201200	-2.25061500	-0.60605200
F	-4.47874200	-3.42439500	-1.11383700
C	-5.01380800	-1.44036800	0.03605800
F	-6.28707500	-1.81927100	0.15313100
C	-4.58633100	-0.23368400	0.56278300
F	-5.45465700	0.56050800	1.19543500
C	-3.25384100	0.13705500	0.43159700
F	-2.94265500	1.32612400	0.99043300
C	-0.14306400	-2.08838700	2.98674900
C	0.03535500	-3.15061200	2.10428100
C	0.07656000	-2.58468400	0.83748100
C	0.00456200	-1.16229200	0.91954500
N	-0.18484800	-0.93389600	2.30595100
H	-0.25617400	-2.09429200	4.06326700
H	0.11087700	-4.19464500	2.37037600
H	0.18579800	-3.12507900	-0.09127100
C	-0.54865000	0.31725200	2.96260200
H	-1.13550400	0.93064600	2.28076900
H	-1.16771000	0.08017700	3.82879400
H	0.33229400	0.86960200	3.29011400
H	1.33408300	-1.03960000	0.56323600

P14_P

P	3.15409000	-0.95512600	-0.36469400
B	-0.85989600	-0.35158500	0.02223100
C	2.21812100	0.18143900	-1.43713300
H	1.93694100	1.00903700	-0.78284400
H	2.93067500	0.58025000	-2.16955700
C	0.96778500	-0.38284000	-2.13530100
H	1.29743900	-1.05355300	-2.93924900
H	0.51229200	0.46896100	-2.65052700

C	-0.09023300	-1.09518300	-1.27270900
H	0.33581700	-2.03940900	-0.91251100
H	-0.85659400	-1.41131400	-1.98998400
C	3.80204300	-2.41148800	-1.32780900
C	4.37566100	-1.92672500	-2.66576300
H	4.76912500	-2.79467900	-3.20505400
H	5.19685200	-1.21550000	-2.53946000
H	3.60688100	-1.46798800	-3.29483300
C	4.87146300	-3.16626700	-0.52770000
H	4.49382500	-3.50027000	0.44372300
H	5.77981200	-2.58031500	-0.37307500
H	5.14940800	-4.05833900	-1.09830600
C	2.63816600	-3.38202800	-1.58671100
H	1.85172600	-2.94955600	-2.20665000
H	2.19233100	-3.74152400	-0.65449300
H	3.04065100	-4.25104900	-2.11829900
C	4.42335600	0.01327700	0.59505100
C	3.74700900	1.30358500	1.08673400
H	2.83521400	1.10123200	1.65432800
H	3.51737500	1.99036600	0.26757800
H	4.44563900	1.81484000	1.75684600
C	5.62970100	0.37745100	-0.27712800
H	6.29050400	1.02574300	0.30781200
H	5.33668500	0.93298800	-1.17376500
H	6.21125300	-0.49664600	-0.58029700
C	4.85082400	-0.80094100	1.82591600
H	5.40747200	-1.70300500	1.56988000
H	3.98865300	-1.08022600	2.44008700
H	5.50570100	-0.17295600	2.43875300
C	-0.42021000	1.24691200	0.03618000
C	0.36494400	1.88593700	0.98918300
F	0.72096900	1.27235600	2.13610700
C	0.88512400	3.16923900	0.83740300
F	1.68840400	3.68584900	1.77448400
C	0.59951200	3.89361800	-0.30596900
F	1.09611700	5.11965500	-0.47712800
C	-0.21742800	3.32237400	-1.27319300
F	-0.50845400	4.00730600	-2.38250300
C	-0.70194900	2.03925400	-1.07665800
F	-1.46806200	1.53974000	-2.06306700
C	-2.50172200	-0.51670700	-0.18800500
C	-3.01932700	-1.79052800	-0.42518900
F	-2.19223700	-2.84734600	-0.51249300
C	-4.36888300	-2.07066100	-0.58387200
F	-4.78675700	-3.31939500	-0.81574200
C	-5.29295600	-1.04095200	-0.49501300
F	-6.59708400	-1.28533800	-0.64175500
C	-4.83952200	0.24253100	-0.24652900
F	-5.71330400	1.25011900	-0.14782300
C	-3.47571300	0.47194900	-0.09856200
F	-3.14441700	1.75164200	0.16895300
C	-0.62570800	-1.62261300	3.62561000
C	0.16221300	-2.58749000	3.04909800
C	0.22122800	-2.27748800	1.66282800
C	-0.51891000	-1.12537700	1.41589900
N	-1.03497500	-0.75996100	2.64378700
H	-0.93721400	-1.47416200	4.65055000
H	0.62871200	-3.41892400	3.55929500
H	0.70112100	-2.88605600	0.90741800
C	-1.93869900	0.34229100	2.90853800
H	-1.92978800	1.04943000	2.08090900
H	-2.96419900	-0.01863000	3.04156600
H	-1.62783000	0.86652900	3.81601000
H	2.25453300	-1.44745200	0.60042300

P15-TS

P	1.17554500	-0.67188400	0.22218000
B	-1.54312200	-0.38635300	0.25077400
C	-0.34258500	-1.06010300	-0.72231800
H	-0.34731600	-2.14282000	-0.52349500
C	-0.48504400	-0.90290300	-2.23472200
H	-0.55710700	0.14835200	-2.51156700
H	-1.45716700	-1.34598200	-2.48081600
C	0.60406900	-1.58974300	-3.05216900
H	0.72625800	-2.63523600	-2.74644800
H	1.56459600	-1.08135500	-2.93872000
H	0.35426700	-1.58183200	-4.11618800
C	1.95299700	0.96376700	0.01906000
C	1.87583800	1.77607300	-1.10825000
F	1.31835400	1.33186900	-2.23091800
C	2.36764400	3.07368400	-1.10931700
F	2.25204100	3.83573500	-2.18944400
C	2.96954500	3.57989800	0.03603900
F	3.44092700	4.81564400	0.04346400
C	3.08965600	2.78473600	1.16882700
F	3.66827500	3.26685500	2.26022900
C	2.58241400	1.49490800	1.14602100
F	2.69469200	0.75791700	2.24924000

C	2.54101800	-1.83728500	-0.11411800
C	3.53359400	-1.63037100	-1.07371600
F	3.51685200	-0.54516100	-1.84865500
C	4.56411400	-2.53799600	-1.27241900
F	5.49055000	-2.31495400	-2.19589300
C	4.62165100	-3.69012400	-0.49679600
F	5.60009900	-4.56199400	-0.68026600
C	3.65209600	-3.92717700	0.46837200
F	3.70897000	-5.02280700	1.21465200
C	2.63717200	-2.99885800	0.64965700
F	1.73258200	-3.24710600	1.59740000
C	-1.29647000	1.25219400	0.20403000
C	-1.69376700	1.98744300	-0.91546100
F	-2.40788500	1.39949600	-1.88487200
C	-1.40014800	3.32970000	-1.11871500
F	-1.80429600	3.95412400	-2.22123700
C	-0.67924800	4.02232400	-0.15572200
F	-0.35410500	5.29779000	-0.33642000
C	-0.27719300	3.35246300	0.98748000
F	0.45814800	3.98482400	1.90709900
C	-0.57945700	2.00383100	1.13091000
F	-0.03761300	1.42084800	2.22497400
C	-3.04981100	-0.82741800	-0.22968300
C	-4.17364400	-0.00822100	-0.14686500
F	-4.06535300	1.26784800	0.27353500
C	-5.46239300	-0.41585200	-0.46753200
F	-6.48708900	0.42944500	-0.36137100
C	-5.68025600	-1.71638500	-0.89417900
F	-6.90599900	-2.12986800	-1.20327400
C	-4.60069300	-2.58116500	-0.98666900
F	-4.78624900	-3.83892500	-1.38636400
C	-3.33267300	-2.12531600	-0.65180300
F	-2.34670500	-3.03774900	-0.76004500
C	-1.97360700	-1.59768700	3.84754800
C	-1.44245500	-2.75990000	3.31488000
C	-1.07465400	-2.44630000	2.00577700
C	-1.32252200	-1.07201400	1.74769700
N	-1.91038200	-0.60658900	2.93385500
H	-2.40069200	-1.41367800	4.82441800
H	-1.34161700	-3.70844400	3.81942900
H	-0.63220600	-3.13468500	1.30055100
C	-2.55732500	0.68218900	3.15573000
H	-2.98171800	1.04895200	2.22193900
H	-3.36837200	0.53816200	3.87045300
H	-1.85254500	1.41364200	3.55156800
H	0.16577000	-0.89374800	1.56935600

P15_P

P	1.26096100	-0.67776400	0.25875900
B	-1.57578600	-0.41065300	0.38711100
C	-0.31401200	-1.17892300	-0.47149500
H	-0.29620400	-2.22593700	-0.13081900
C	-0.43666600	-1.19235800	-2.00136800
H	-0.56555200	-0.18004600	-2.38269800
H	-1.38145000	-1.70639600	-2.19819000
C	0.68278900	-1.90178300	-2.75886400
H	1.60778600	-1.31818400	-2.75634700
H	0.40157600	-2.04582500	-3.80483200
H	0.89011100	-2.88961600	-2.33191600
C	1.91632600	0.99580200	0.04859000
C	1.77624300	1.73695900	-1.11988200
F	1.23494500	1.18391200	-2.20078800
C	2.18112300	3.06034200	-1.19245200
F	1.99304700	3.76037400	-2.30084600
C	2.75110700	3.66191200	-0.07540900
F	3.12679800	4.92559000	-0.13241200
C	2.92866800	2.93777600	1.09765800
F	3.47310800	3.51326400	2.15772100
C	2.50837900	1.61724000	1.14914200
F	2.67802500	0.94287600	2.28278700
C	2.66267400	-1.78944200	-0.07421800
C	3.62541800	-1.54858300	-1.05366100
F	3.55014700	-0.46341000	-1.82441900
C	4.67083500	-2.43081300	-1.27984900
F	5.57144300	-2.18076600	-2.21897700
C	4.76932300	-3.58565400	-0.51080600
F	5.76199600	-4.43140900	-0.72008000
C	3.82482300	-3.85544500	0.47183700
F	3.92108700	-4.95485200	1.20317500
C	2.78930900	-2.95558300	0.67804000
F	1.89651600	-3.22832000	1.62549300
C	-1.32660100	1.21656300	0.20338200
C	-1.64599600	1.89968800	-0.97138000
F	-2.26031000	1.26287600	-1.97838500
C	-1.35165000	3.23800700	-1.20528600
F	-1.66794300	3.81428600	-2.36203700
C	-0.70787400	3.97318200	-0.22009200
F	-0.35202700	5.23635100	-0.43599900

C -0.37112200 3.34974900 0.97013700
F 0.32491200 4.02056000 1.89495800
C -0.66825100 2.00402000 1.14472500
F -0.16401000 1.45430300 2.26978900
C -3.02153100 -0.91819200 -0.23521100
C -4.15483200 -0.12335700 -0.38983100
F -4.11398100 1.20057100 -0.13910900
C -5.39961000 -0.60450900 -0.77928100
F -6.43636300 0.22391400 -0.90291100
C -5.56192900 -1.95621000 -1.03428900
F -6.74471000 -2.43762800 -1.40820500
C -4.47152400 -2.79979400 -0.88654600
F -4.60148500 -4.10551700 -1.12239900
C -3.25223500 -2.27069200 -0.48810000
F -2.25096800 -3.16477700 -0.36348800
C -2.11920500 -1.14178700 4.09799400
C -1.33242800 -2.22448300 3.80254200
C -0.98784000 -2.10508600 2.43107100
C -1.55777300 -0.94560000 1.91736900
N -2.26052400 -0.38390100 2.96471400
H -2.60015300 -0.84232900 5.01835600
H -1.05466000 -3.01727300 4.48129100
H -0.43007500 -2.84091500 1.86821700
C -3.01539100 0.85360800 2.94714300
H -3.40619700 1.04632100 1.94902300
H -3.85740700 0.76384900 3.63659500
H -2.39615300 1.70162500 3.25673800
H 1.07124300 -0.78735900 1.64016700

P16_TS

P 1.17748800 -0.67117800 0.19572500
B -1.56183000 -0.37335900 0.25541300
C -0.34996500 -0.85633400 -0.76321200
C -0.47145900 -1.22842000 -2.04232200
H -1.49609700 -1.31728300 -2.40902900
C 0.58096400 -1.52152300 -3.06229600
H 0.35864500 -0.96846800 -3.98041400
H 0.57643100 -2.58534000 -3.32430200
H 1.57894500 -1.23286300 -2.73950400
C 1.93792900 0.96991500 0.00251400
C 1.78645100 1.77863200 -1.12144600
F 1.19668800 1.31819500 -2.22023400
C 2.22913200 3.09409100 -1.13128800
F 2.03155300 3.86090000 -2.19598300
C 2.85197100 3.61814800 -0.00508100
F 3.26990000 4.87305200 -0.00705500
C 3.04009600 2.82662900 1.12175400
F 3.63483500 3.32991500 2.19471400
C 2.57937200 1.51913500 1.11252200
F 2.74683800 0.78153300 2.20872400
C 2.53518200 -1.84007400 -0.15834000
C 3.53203400 -1.62067500 -1.11156000
F 3.49556900 -0.54248800 -1.89685700
C 4.57976800 -2.50951800 -1.30057600
F 5.50465300 -2.27608100 -2.22289400
C 4.65724600 -3.65258500 -0.51317100
F 5.65296900 -4.50646200 -0.68484600
C 3.68980100 -3.89808600 0.45160600
F 3.76872700 -4.98214300 1.21197600
C 2.65539100 -2.98891500 0.62219100
F 1.76481500 -3.24069400 1.57901900
C -1.31647800 1.26591700 0.27965300
C -1.63687100 1.99353500 -0.86954800
F -2.26805900 1.38567700 -1.88094500
C -1.32169600 3.33084900 -1.06071900
F -1.64703300 3.95594400 -2.18933800
C -0.63435000 4.01435100 -0.06507400
F -0.26491100 5.27908600 -0.24314900
C -0.28404000 3.34190900 1.09291100
F 0.44660700 3.96049500 2.02609600
C -0.61400600 1.99780100 1.22939300
F -0.09987400 1.39596700 2.32599100
C -3.06049200 -0.80597500 -0.23791400
C -4.18126800 0.01912600 -0.17884700
F -4.07403600 1.29470400 0.23998400
C -5.46628300 -0.38976400 -0.51297000
F -6.49183800 0.45737600 -0.42915800
C -5.67996300 -1.69453900 -0.92920100
F -6.90245100 -2.10825600 -1.25202800
C -4.60265100 -2.56489400 -0.99343500
F -4.78890700 -3.82744100 -1.37889600
C -3.33710200 -2.11138200 -0.64398600
F -2.35428500 -3.02501600 -0.71227500
C -1.92502500 -1.84965400 3.74346800
C -1.36130900 -2.94961300 3.11814300
C -1.01346500 -2.52236700 1.83448400
C -1.30914700 -1.14479300 1.69072600
N -1.90112800 -0.78879200 2.90795500

H	-2.34602400	-1.75423500	4.73555300
H	-1.22766200	-3.93020200	3.54867700
H	-0.56656100	-3.12106800	1.05291700
C	-2.57732300	0.46548000	3.21588100
H	-3.39940000	0.25522400	3.90117500
H	-1.89348300	1.18031200	3.67427700
H	-2.99021100	0.89288900	2.30179600
H	0.18965100	-0.93975500	1.52904700

P16_P

C	-0.63913700	-1.76241000	-2.81847700
H	-1.61523400	-1.32548900	-2.61567100
H	-0.74308900	-2.85081100	-2.90033200
H	-0.32172100	-1.40182600	-3.80127200
C	0.41067300	-1.41768300	-1.81094300
H	1.43012600	-1.55511300	-2.17327400
C	0.32078700	-0.95667500	-0.55313200
P	-1.26870300	-0.68215400	0.22591300
C	-2.63949000	-1.82113600	-0.11846900
C	-3.62051900	-1.56881200	-1.07745800
F	-3.54560200	-0.48161100	-1.84668100
C	-4.67659600	-2.43973100	-1.28732700
F	-5.59203800	-2.17837100	-2.20988900
C	-4.77284600	-3.59260700	-0.51632400
F	-5.77925100	-4.42703900	-0.70589700
C	-3.81440900	-3.87117300	0.44848300
F	-3.91168300	-4.96829400	1.18460900
C	-2.76634000	-2.98357800	0.63980900
F	-1.87230500	-3.26252800	1.58174300
C	-1.93387800	0.99139200	0.04814200
C	-2.50055600	1.62303400	1.15475600
F	-2.65987000	0.95324400	2.29246900
C	-2.89650100	2.95087400	1.10636400
F	-3.41509400	3.54112500	2.17271200
C	-2.71746800	3.67003800	-0.06912300
F	-3.06353200	4.94337500	-0.12158400
C	-2.16416400	3.06050900	-1.18941400
F	-1.96369900	3.76256100	-2.29581600
C	-1.77896100	1.73105800	-1.12143600
F	-1.23948900	1.17491700	-2.20153900
B	1.58851600	-0.38944000	0.37847900
C	3.02777200	-0.89674500	-0.24772800
C	4.16144400	-0.10733100	-0.41806100
F	4.12560900	1.21779600	-0.17498900
C	5.40017800	-0.59757000	-0.81330600
F	6.44301900	0.22422700	-0.95061500
C	5.55236400	-1.95133300	-1.05953700
F	6.73120000	-2.44254900	-1.43945400
C	4.46000000	-2.78816200	-0.89680300
F	4.58584000	-4.09812200	-1.12297500
C	3.24532500	-2.25404500	-0.49005800
F	2.24492400	-3.13784000	-0.34104800
C	1.35397400	1.24609100	0.25330400
C	0.69748200	2.02896800	1.19596700
F	0.21396800	1.48161000	2.33072300
C	0.38508400	3.37076900	1.00951100
F	-0.30721000	4.04537100	1.93515800
C	0.70071700	3.98415800	-0.19068000
F	0.33385100	5.24433700	-0.41416900
C	1.32758600	3.24324700	-1.18351200
F	1.60554300	3.80980700	-2.35726500
C	1.62359000	1.90848200	-0.94533500
F	2.17894100	1.23891700	-1.96435400
C	1.54390400	-0.99446800	1.87570500
C	0.97052900	-2.18263000	2.30770900
C	1.30440700	-2.38571800	3.67264200
C	2.09073600	-1.32173600	4.04131200
N	2.23950500	-0.49599900	2.95819400
H	0.42051800	-2.86635900	1.67487300
H	1.02011800	-3.21675300	4.30247400
H	2.56470500	-1.07961000	4.98237300
C	3.01454500	0.72909100	3.00615000
H	3.85208000	0.59273300	3.69343100
H	3.41497800	0.96339600	2.01934500
H	2.40844200	1.57228400	3.35181200
H	-1.05897100	-0.81437000	1.60116400

P18_TS

P	-2.39268423	0.49113194	0.27584762
Si	3.95763601	1.31149396	-1.51185422
B	1.11935561	-0.20503422	0.42430018
C	-1.37760771	1.28853196	-1.04308227
H	-0.98786596	0.53429983	-1.72231957
H	-1.98874631	1.98686525	-1.61813438
C	-0.20645912	2.02711630	-0.35593897
H	-0.09328885	2.98979440	-0.85095235
H	-0.50649626	2.27587831	0.66796495
C	1.11589979	1.24180905	-0.37439397

C	2.13305612	1.78229799	-1.09178053	C	-3.41388672	-0.94203075	-0.26164083
C	4.19967891	1.75463248	-3.33713070	C	-4.07483230	-1.62162564	0.78665389
H	5.22370557	1.49605295	-3.62676436	C	-4.88028100	-2.71991204	0.49294689
H	4.04448997	2.81653472	-3.54110339	H	-5.38380509	-3.23116074	1.30989095
H	3.52011599	1.18453589	-3.97957875	C	-5.03877520	-3.18902015	-0.80588115
C	5.10504795	2.42960662	-0.50442056	C	-4.36101808	-2.52428937	-1.82342347
H	4.97938921	2.28900427	0.57261673	H	-4.45550947	-2.88714824	-2.84425477
H	4.94904243	3.48688034	-0.73389372	C	-3.54305634	-1.41948707	-1.58525422
H	6.14180167	2.18419019	-0.76023047	C	-3.91289208	-1.22760777	2.23393140
C	4.69849672	-0.42232452	-1.35832745	H	-4.41374493	-1.95087292	2.87989113
H	5.53483669	-0.45196149	-2.06611358	H	-4.33768902	-0.24061064	2.44319097
H	4.02212215	-1.23878142	-1.60709916	H	-2.85313599	-1.20635212	2.51379347
H	5.11464725	-0.59337295	-0.36407996	C	-5.86338979	-4.41271694	-1.09949041
C	1.90453635	3.07750076	-1.82536583	H	-6.63023560	-4.56830540	-0.33742562
C	1.30156960	3.07315247	-3.09012587	H	-5.22435527	-5.30167543	-1.11779613
H	0.98921915	2.12097643	-3.51365313	H	-6.35261200	-4.33739936	-2.07355246
C	1.11542823	4.25480809	-3.79876649	C	-2.82761492	-0.83788304	-2.78043316
H	0.64878947	4.22888555	-4.77910972	H	-1.75281680	-1.03775036	-2.73202498
C	1.53261485	5.46917940	-3.25605642	H	-2.96212602	0.24075956	-2.87174460
H	1.39344187	6.39239787	-3.80940785	H	-3.20218358	-1.29927788	-3.69599355
C	2.12683154	5.48901520	-1.99803918	C	2.61567095	-0.67595555	0.94930902
H	2.44813815	6.43107017	-1.56380036	F	3.07094064	1.50350026	1.80610236
C	2.31065168	4.30315869	-1.28895151	C	3.41501145	0.21264495	1.67532457
H	2.76263971	4.32025689	-0.30060896	F	5.31984214	0.77945228	2.95244455
C	-3.59475903	1.79465420	0.81077279	C	4.60571194	-0.13770635	2.29812450
C	-4.59870647	2.26910789	-0.07451283	F	6.18422587	-1.80928566	2.83853093
C	-5.47643401	3.25719298	0.36232440	C	5.05301237	-1.44940394	2.23627419
H	-6.23894749	3.61509921	-0.32565599	F	4.70805703	-3.64360269	1.45386090
C	-5.41786589	3.79742088	1.64605618	C	4.29821480	-2.37682671	1.53626801
C	-4.44760334	3.30629851	2.50612745	F	2.48050125	-2.94972374	0.23832497
H	-4.38452676	3.69752481	3.51881323	C	3.12522807	-1.97217875	0.90912749
C	-3.53690552	2.31916963	2.11718863	C	0.43040732	-1.36944411	-0.56483103
C	-4.79400241	1.76318478	-1.48211682	F	1.92079155	-0.82852393	-2.34332662
H	-5.66318377	2.24544984	-1.93230982	C	0.93454926	-1.59242705	-1.85295937
H	-4.95696954	0.68390104	-1.50763427	F	1.07640275	-2.76145923	-3.90136774
H	-3.93627168	1.98885159	-2.12086180	C	0.51738549	-2.60992956	-2.70158011
C	-6.37648714	4.87759472	2.06991278	F	-0.91202367	-4.45028042	-3.09924585
H	-7.40819178	4.60105708	1.83593191	C	-0.49007251	-3.47583816	-2.29698438
H	-6.16117263	5.81253882	1.54324274	F	-2.03785522	-4.08331658	-0.62647923
H	-6.30752201	5.07144727	3.14213728	C	-1.05111458	-3.29167949	-1.04734174
C	-2.51971738	1.90712339	3.14852355	F	-1.24097627	-2.17027446	0.94365106
H	-2.93203550	2.02617592	4.15347149	C	-0.59256875	-2.25661995	-0.23770168
H	-1.63335935	2.54384879	3.08398354	H	-1.07426759	0.15972152	1.32877724
H	-2.18038252	0.87748866	3.04356670	C	0.50682385	-0.45299323	4.13836623

C	0.59420316	0.92429241	4.01388339
C	0.50868045	1.18318381	2.64858741
C	0.33045600	-0.01636474	1.91084447
N	0.36688787	-1.01787949	2.92376093
H	0.54672266	-1.07185059	5.02572263
H	0.70793704	1.63444837	4.81919968
H	0.60828399	2.15982257	2.19905634
C	0.52495644	-2.46428460	2.78559125
H	0.88819787	-2.71564402	1.79304907
H	1.26955839	-2.79619324	3.51176857
H	-0.41719357	-2.98570496	2.96116548

P18_P

P	-2.68034000	0.29233500	0.12848100
Si	3.37341500	1.75392700	-2.00255400
B	1.48724400	-0.10983600	0.75232200
C	-1.36105200	1.20010000	-0.73245300
H	-0.88610400	0.50773400	-1.42741900
H	-1.80884800	2.00809700	-1.31539100
C	-0.32897700	1.72831400	0.29632600
H	-0.40470800	2.81726400	0.33814600
H	-0.57211700	1.35710100	1.29138600
C	1.08391600	1.25300900	-0.07778300
C	1.73207400	1.96429800	-1.03349700
C	3.06465700	2.45682700	-3.73587300
H	3.98938200	2.35357700	-4.31609000
H	2.79480300	3.51648900	-3.72771600
H	2.28137600	1.91471800	-4.27698800
C	4.72693800	2.85248800	-1.27545700
H	5.08820300	2.53060600	-0.29672300
H	4.37696100	3.88597200	-1.17941000
H	5.57995400	2.86186300	-1.96430200
C	4.10039000	0.04345600	-2.33729200
H	4.61965300	0.09658400	-3.30135300
H	3.35211200	-0.74700600	-2.40611200
H	4.84196700	-0.24331200	-1.58960400
C	1.09979400	3.23956100	-1.52846700
C	0.22003200	3.25944500	-2.61949300
H	-0.01668700	2.32419300	-3.12264200
C	-0.32959500	4.45376100	-3.07893800
H	-1.00488600	4.44580500	-3.92988800
C	-0.00553700	5.65837900	-2.45768600
H	-0.42811800	6.59088100	-2.81829000
C	0.87073300	5.65636500	-1.37514800
H	1.12920900	6.58944700	-0.88331700
C	1.41791300	4.45944300	-0.91724500
H	2.09588400	4.45537000	-0.06712400
C	-3.88072200	1.42179500	0.87887700
C	-4.83357400	2.07078000	0.06501600
C	-5.74257600	2.93952900	0.66205700
H	-6.48128100	3.43500500	0.03716100
C	-5.72545100	3.19066100	2.03330600
C	-4.76920300	2.54386500	2.81337900
H	-4.74563000	2.73234500	3.88332400
C	-3.83482800	1.65903900	2.27351000
C	-4.88880000	1.87540000	-1.42824000
H	-5.75368800	2.39244800	-1.84708500
H	-4.96604400	0.81959500	-1.70514300
H	-3.99836000	2.29116000	-1.91344000
C	-6.69519500	4.16056700	2.65023200
H	-7.64416000	4.17062700	2.10906100
H	-6.28793800	5.17679000	2.62157000
H	-6.89360800	3.91350600	3.69556000
C	-2.84775000	1.01449200	3.21918200
H	-3.04405200	1.35132000	4.23830300
H	-1.80722500	1.26985000	2.99339300
H	-2.92378100	-0.07736600	3.21958400
C	-3.37549500	-1.12678700	-0.75886800
C	-3.97524900	-2.11862400	0.05716100
C	-4.40398000	-3.30165100	-0.53271700
H	-4.84947600	-4.06720200	0.09700600
C	-4.25616100	-3.53935900	-1.89957800
C	-3.67543800	-2.54652000	-2.68116200
H	-3.55255300	-2.71523900	-3.74794600
C	-3.22830000	-1.33551500	-2.14614600
C	-4.11116100	-1.97258100	1.55188100
H	-4.71089400	-2.79074200	1.95377700
H	-4.59597600	-1.03343600	1.83628500
H	-3.13317600	-2.02132200	2.04501000
C	-4.66405300	-4.85864400	-2.49093100
H	-4.72058200	-4.80923600	-3.58012700
H	-5.63357400	-5.18428700	-2.10500000
H	-3.92777100	-5.62402100	-2.22501000
C	-2.58259900	-0.37193400	-3.11361800
H	-1.49985100	-0.53775800	-3.16898000
H	-2.75743700	0.67535700	-2.86660400
H	-2.98172300	-0.54312300	-4.11541700
C	3.07695200	-0.54702700	0.60027700

F	3.69736200	1.61849100	1.35760200
C	4.04762800	0.37368200	1.00349500
F	6.27381400	1.03916800	1.44827800
C	5.40471900	0.09343800	1.07828100
F	7.15869500	-1.47266000	0.83683600
C	5.86039600	-1.17767200	0.76076000
F	5.35285500	-3.37433900	0.08118500
C	4.93989700	-2.13750700	0.37500700
F	2.77943900	-2.81929100	-0.06058000
C	3.59186100	-1.80357000	0.29797700
C	0.46526900	-1.33881500	0.21242700
F	1.13470600	-0.95919600	-2.03356200
C	0.44255500	-1.68905700	-1.14017800
F	-0.23118600	-3.00635600	-2.98907500
C	-0.24847600	-2.76699000	-1.67453900
F	-1.63698700	-4.64572100	-1.31538000
C	-0.98563600	-3.58690900	-0.83462900
F	-1.82980100	-3.98381300	1.32503800
C	-1.05957300	-3.25815200	0.50605100
F	-0.60193000	-1.87705900	2.28858700
C	-0.37255400	-2.14434300	0.98547700
H	-2.04037100	-0.28466700	1.22395600
C	1.61282900	-0.16429500	4.58603700
C	1.26302000	1.15491100	4.43507600
C	1.10758900	1.37030400	3.03982600
C	1.35314300	0.18258200	2.36047300
N	1.67309300	-0.73817300	3.34411800
H	1.82388600	-0.74998000	5.47032800
H	1.13696500	1.87671300	5.23011200
H	0.88498400	2.31857500	2.56785700
C	2.09981700	-2.11135600	3.15981500
H	1.85718000	-2.46047300	2.15808500
H	3.18219400	-2.20511100	3.30187100
H	1.59152900	-2.75642800	3.88127200

P19_TS

B	0.76503000	-0.37474200	0.12251300
C	0.12655800	-1.06357700	1.49161000
C	0.90757800	-2.00931200	2.17704600
H	1.91796300	-2.20110900	1.82988500
C	0.45989200	-2.75572500	3.26239000
H	1.12262000	-3.47477700	3.73427700
C	-0.83027300	-2.56613000	3.73515700
H	-1.20629200	-3.12668200	4.58513700
C	-1.62716400	-1.61355600	3.11301100
H	-2.62096400	-1.41957100	3.51091500
C	-1.18224900	-0.86843100	2.01278700
C	-2.16883200	0.16671300	1.49790500
H	-1.67139500	1.12176400	1.31414000
H	-2.93606900	0.35858900	2.25439500
C	-4.32440100	-1.49459200	0.15833400
C	-5.24482500	-1.41599800	-1.07677000
H	-4.67397600	-1.73951100	-1.95886600
H	-6.04502600	-2.15422800	-0.93389400
C	-5.84752500	-0.03803800	-1.36360200
H	-6.42149700	0.31507200	-0.49840800
H	-6.56739400	-0.13546600	-2.18252800
C	-4.78975400	0.99437200	-1.76182800
H	-5.27497800	1.92695600	-2.07965900
H	-4.24277100	0.61331200	-2.63608100
C	-3.77129000	1.35056000	-0.66234800
C	-2.66765800	2.22987600	-1.27303700
H	-2.22494800	1.75375000	-2.15165100
H	-1.86409400	2.44441400	-0.55807700
H	-3.09781400	3.18784800	-1.58670000
C	-4.43498400	2.13624100	0.47477800
H	-3.70915300	2.41527500	1.24408600
H	-5.24886500	1.59323000	0.95810800
H	-4.85375900	3.06387800	0.06700200
C	-5.11633300	-1.27856800	1.45187500
H	-4.47559500	-1.39713500	2.32927800
H	-5.90619400	-2.03654600	1.51746900
H	-5.59291200	-0.29882800	1.51053300
C	-3.68221600	-2.88787200	0.21101800
H	-3.10759800	-3.09235600	-0.69869700
H	-4.46819800	-3.64733700	0.29901300
H	-3.01707500	-2.98609900	1.07530800
C	2.35732300	-0.78088900	-0.05879400
C	2.93690300	-1.47802300	-1.11218200
F	2.18849200	-1.99330000	-2.10462500
C	4.30565100	-1.70700900	-1.22548100
F	4.79387500	-2.38850600	-2.26342500
C	5.16380100	-1.22236000	-0.25306600
F	6.47524700	-1.43319700	-0.34422200
C	4.63832600	-0.51859800	0.82254800
F	5.45096200	-0.05218900	1.77111600
C	3.26850100	-0.31350500	0.89101600
F	2.81861700	0.36434400	1.95766300

C	0.80965600	1.28503900	0.12291300
C	0.53814100	2.11026000	1.21598300
F	0.17860700	1.59923400	2.40128800
C	0.61549500	3.49975100	1.18092700
F	0.30579800	4.22133900	2.25869900
C	1.04856600	4.13497600	0.02838300
F	1.13759700	5.46138900	-0.02200400
C	1.41510200	3.36183000	-1.06236400
F	1.88600600	3.94623500	-2.16460700
C	1.31823000	1.97856000	-0.97841100
F	1.78468400	1.30901300	-2.04764500
C	-0.11951000	-0.89602000	-1.19563400
C	-0.36827400	-0.28779300	-2.46389100
C	-0.89221700	-1.21914900	-3.34754100
C	-0.88755300	-2.43624300	-2.66571800
N	-0.40998100	-2.26368000	-1.42941600
H	-0.27267300	0.77176200	-2.64494600
H	-1.23382000	-1.05749400	-4.35850700
H	-1.19245000	-3.41791900	-3.00532500
C	-0.16454500	-3.36501200	-0.51287700
H	-0.42023800	-4.29398300	-1.02470200
H	0.89208400	-3.38062300	-0.23778900
H	-0.76159600	-3.26412300	0.39413800
H	-1.35269400	-0.53841100	-0.89820600
P	-2.95650700	-0.24560700	-0.11741800

P19_P

B	0.82924000	-0.35089800	0.07377700
C	0.10134200	-0.96619300	1.42517600
C	0.74463300	-1.94782700	2.19721900
H	1.73115700	-2.28413800	1.89350900
C	0.18343600	-2.52824600	3.32976100
H	0.73410800	-3.28625700	3.87851200
C	-1.06544100	-2.10946300	3.77279800
H	-1.50364000	-2.51440000	4.67920000
C	-1.73502300	-1.13859200	3.03984300
H	-2.68689400	-0.76145900	3.40664800
C	-1.19103900	-0.60623700	1.86575900
C	-2.01784000	0.44918500	1.15593100
H	-1.38353000	1.14345800	0.59710300
H	-2.59601900	1.03203500	1.88219500
C	-4.42434000	-1.42832100	0.37746900
C	-5.40630400	-1.54202300	-0.81396900
H	-4.85665300	-1.92480400	-1.68558800
H	-6.13687000	-2.31456000	-0.54281000
C	-6.12688700	-0.25113800	-1.21239000
H	-6.69149000	0.15344400	-0.36427500
H	-6.86808100	-0.49024800	-1.98072200
C	-5.17049600	0.80537000	-1.77189900
H	-5.73617500	1.66883400	-2.14370700
H	-4.64078600	0.38258400	-2.63688400
C	-4.12269900	1.34721900	-0.77617600
C	-3.12894300	2.24099900	-1.53363300
H	-2.64552500	1.70116400	-2.34978800
H	-2.34882400	2.64127300	-0.87811500
H	-3.68019800	3.09044500	-1.95155600
C	-4.77477000	2.17300600	0.34069100
H	-4.02541100	2.59525000	1.01562200
H	-5.49504100	1.61172100	0.93781900
H	-5.30803100	3.01134100	-0.11977700
C	-5.17442500	-1.14144800	1.68196000
H	-4.48866500	-1.01653800	2.52109600
H	-5.81405900	-2.00200200	1.90513400
H	-5.81673200	-0.26161300	1.62572200
C	-3.65198100	-2.75185700	0.50461300
H	-3.08452200	-2.96688700	-0.40716800
H	-4.37543800	-3.56086000	0.65456600
H	-2.96247900	-2.74340300	1.35297000
C	2.37317700	-0.93386400	-0.07106200
C	2.89604700	-1.64729900	-1.14451100
F	2.11540700	-2.04795700	-2.16109000
C	4.23572500	-2.01144100	-1.24786600
F	4.67144300	-2.70261800	-2.30341300
C	5.12382800	-1.64997800	-0.24881100
F	6.40991900	-1.98744500	-0.33357400
C	4.65576800	-0.93609800	0.84544200
F	5.49846800	-0.58211900	1.81770500
C	3.31208400	-0.59554600	0.90448200
F	2.92210800	0.09732600	1.98706600
C	1.06733900	1.29717900	0.11204400
C	0.79587500	2.16868800	1.16505100
F	0.21084900	1.73612900	2.29546600
C	1.07232500	3.53309900	1.13905900
F	0.75154500	4.30851600	2.17802500
C	1.68983500	4.08614100	0.03058200
F	1.95969700	5.38979600	-0.01603700
C	2.01924600	3.26003500	-1.03416100
F	2.62649500	3.77155200	-2.10610300

C	1.71901500	1.90613900	-0.96505200
F	2.10244500	1.17528200	-2.02210100
C	-0.09305500	-0.71017800	-1.22109500
C	-0.54850700	0.07934100	-2.26924900
C	-1.32678000	-0.71546000	-3.15197900
C	-1.33468900	-1.98883800	-2.62445800
N	-0.60336000	-1.97476300	-1.46238900
H	-0.31246000	1.12627600	-2.39839400
H	-1.79988000	-0.40770800	-4.07455100
H	-1.76100900	-2.91044000	-2.99615700
C	-0.27171700	-3.18181400	-0.72369400
H	-0.58593800	-4.04448900	-1.31435600
H	0.80742100	-3.23763200	-0.57135900
H	-0.76213500	-3.20793000	0.25187700
H	-2.58183500	-0.68333900	-1.21813000
P	-3.21405800	-0.11831400	-0.10610400

P21_TS

P	-1.95369300	0.23215600	0.65984300
C	-2.18435500	1.99784700	0.26801900
C	-3.25411100	2.77361800	0.72388600
C	-3.29837300	4.13220000	0.43182200
C	-2.29206500	4.72380400	-0.33109100
C	-1.20945500	3.98249900	-0.80073800
C	-1.16794200	2.63107900	-0.45304700
O	-0.13208100	1.84560000	-0.83685000
C	1.11595900	2.45670000	-0.98675100
C	2.20679600	1.64714600	-0.66862500
C	3.45028600	2.24905000	-0.86675700
C	3.57509300	3.57206900	-1.29349800
C	2.44743700	4.34251600	-1.54017100
C	1.17468000	3.78238800	-1.39829600
C	-0.12792300	4.49803700	-1.74519800
C	0.00714200	6.01922700	-1.67304800
C	-0.52078200	4.09190600	-3.18363100
B	1.96406700	0.16418800	-0.00688400
C	1.11001700	-0.82596000	-1.03822700
C	0.78149900	-2.12987100	-0.67533400
F	1.11651400	-2.58113500	0.54947800
C	0.16453900	-3.06477700	-1.48856900
F	-0.12902200	-4.28656300	-1.02914700
C	-0.20366000	-2.70116200	-2.77203400
F	-0.84189100	-3.56006200	-3.56734300
C	0.08028900	-1.41806300	-3.19733600
C	0.73797800	-0.52867300	-2.35167600
F	0.98633800	0.66033100	-2.92580500
F	-0.31718800	-1.02074900	-4.40959100
C	3.44017000	-0.49758800	0.34852000
C	4.01534000	-1.62804200	-0.23055100
F	3.41438400	-2.29603500	-1.22666400
C	5.24470300	-2.15857500	0.15686300
F	5.72231800	-3.25503100	-0.43408600
C	5.97946000	-1.53920600	1.15226900
F	7.15785900	-2.02992900	1.53079800
C	5.47391100	-0.38772700	1.73842600
C	4.24393100	0.10258700	1.32426900
F	3.84698900	1.23583100	1.92068800
F	6.17818600	0.24036700	2.68211400
C	-3.29200300	-0.11228300	1.88455300
C	-2.94321100	-0.30156200	3.23738100
C	-1.52471500	-0.16169400	3.71106100
C	-3.92935700	-0.63617500	4.16076200
C	-5.26398500	-0.78252100	3.78915100
C	-6.31139400	-1.13933100	4.81005200
C	-5.59430400	-0.56815900	2.45599800
C	-4.64156300	-0.23567700	1.48851900
C	-5.14896800	-0.03275900	0.07922600
C	-2.39311300	-0.92197400	-0.70655500
C	-2.73414800	-0.56630200	-2.02974700
C	-2.52912600	0.80317000	-2.62549700
C	-3.26874300	-1.54810400	-2.86487100
C	-3.46699400	-2.86323800	-2.45116000
C	-4.02135900	-3.89706200	-3.39270700
C	-3.05518600	-3.20735800	-1.16796700
C	-2.50460700	-2.26853700	-0.29579100
C	-2.01828000	-2.77467400	1.04375100
H	-4.04103900	2.31774900	1.31593700
H	-4.12606100	4.73326200	0.79188300
H	-2.35973900	5.78129100	-0.56405200
H	4.35427500	1.68061700	-0.67120900
H	4.56138100	4.00646400	-1.42249600
H	2.55788600	5.37477900	-1.85435200
H	0.77119700	6.36715200	-2.37155300
H	0.27697300	6.35162800	-0.66645200
H	-0.92796000	6.50361800	-1.96715700
H	0.22805100	4.46558700	-3.88854900
H	-0.55916100	3.00347000	-3.28265600
H	-1.49888100	4.50787300	-3.44766400

H	-1.47029600	-0.21950600	4.80031700
H	-0.88901600	-0.95525700	3.30310200
H	-1.08738100	0.79156900	3.40497900
H	-3.64473100	-0.78498300	5.19993600
H	-6.42717300	-0.33691100	5.54507100
H	-7.28213900	-1.31041800	4.34057400
H	-6.03156500	-2.04407000	5.35702900
H	-6.63299400	-0.65895200	2.14680800
H	-6.20602600	0.24112600	0.11033200
H	-5.05308900	-0.94988300	-0.51136200
H	-4.61483800	0.75725500	-0.45282200
H	-2.81696700	0.79181000	-3.67821300
H	-1.47491600	1.08838700	-2.57167400
H	-3.11610500	1.57801300	-2.12306600
H	-3.52982200	-1.27202300	-3.88348600
H	-4.58149300	-4.66509200	-2.85430800
H	-3.20109200	-4.38907200	-3.92385800
H	-4.67995000	-3.44363200	-4.13697500
H	-3.13292600	-4.24081800	-0.83785300
H	-1.62452500	-3.78560700	0.91975200
H	-1.20154400	-2.16605900	1.44450100
H	-2.82146300	-2.79734800	1.78751400
C	1.54523900	0.61732900	3.76547800
C	1.18812700	1.88988300	3.35298400
C	0.98444700	1.79650000	1.97913400
C	1.19209800	0.46537100	1.51526800
N	1.54403300	-0.22068100	2.71082700
H	1.77447800	0.24703400	4.75620100
H	1.10551700	2.76654400	3.97763100
H	0.74425000	2.63551600	1.34393200
C	1.85686500	-1.62956800	2.90117300
H	2.27938600	-1.74546700	3.90006000
H	0.95911500	-2.24702900	2.81826900
H	2.58096400	-1.96830000	2.16367400
H	-0.33519000	0.21330200	1.18592600

P21-P

P	-2.53316700	0.13676800	0.18205000
C	-2.51984500	1.84220900	-0.40368100
C	-3.74478600	2.51798100	-0.50422900
C	-3.78654500	3.82506300	-0.95892300
C	-2.60586800	4.45814000	-1.34014400
C	-1.37554200	3.81444200	-1.25725400
C	-1.33289800	2.51039500	-0.74455200
O	-0.15742600	1.84925500	-0.60781600
C	0.99912400	2.62617600	-0.36017600
C	2.04410200	2.02012200	0.33060900
C	3.08748300	2.88836300	0.68950000
C	3.11629600	4.22784100	0.33448500
C	2.09317300	4.75453700	-0.44873200
C	1.02217500	3.94830800	-0.82121900
C	-0.07206800	4.38869800	-1.78227200
C	-0.13611700	5.90380400	-1.96261400
C	0.20975500	3.73718500	-3.15629900
B	2.11639600	0.48095500	0.87760200
C	1.23344400	-0.64694000	0.02154600
C	0.73732100	-1.83415200	0.56333000
F	0.54372300	-1.97979300	1.88522700
C	0.43109800	-2.95838100	-0.19831400
F	-0.02235400	-4.07593400	0.38407900
C	0.55070800	-2.91482800	-1.57542600
F	0.25717400	-3.98718600	-2.31587300
C	0.93955600	-1.72778800	-2.17357800
C	1.27965300	-0.64586800	-1.37460800
F	1.73376900	0.43392200	-2.03641400
F	0.97791800	-1.63680300	-3.50781800
C	3.62327100	-0.18340900	0.60727200
C	4.53535400	0.21676600	-0.36993100
F	4.31588600	1.28424100	-1.14945400
C	5.72826800	-0.45219500	-0.63463800
F	6.56136000	0.00186600	-1.57632500
C	6.04557100	-1.60372100	0.06197700
F	7.18279600	-2.25638200	-0.18391500
C	5.15359200	-2.07383500	1.01362200
C	3.97765600	-1.37619100	1.24003300
F	3.14252700	-1.92520700	2.14043500
F	5.42358600	-3.19892200	1.68327800
C	-4.10374300	-0.10182900	1.06268500
C	-4.12797700	0.07315700	2.46140900
C	-2.92240800	0.52112000	3.24551300
C	-5.31916000	-0.17472900	3.14126800
C	-6.47748900	-0.57462000	2.47856600
C	-7.74088500	-0.87164300	3.23844500
C	-6.43440400	-0.69927400	1.09066400
C	-5.27079200	-0.47029500	0.35757500
C	-5.35020600	-0.62227400	-1.14474900
C	-2.36772700	-1.20650700	-1.02344400
C	-2.17861200	-1.01105600	-2.40684400

C	-1.83449600	0.31446100	-3.03794200
C	-2.26524800	-2.12406800	-3.24321300
C	-2.51830400	-3.40555400	-2.75837800
C	-2.56354100	-4.58651300	-3.68589000
C	-2.63410300	-3.57735300	-1.38027500
C	-2.55404500	-2.50622400	-0.49360800
C	-2.64026300	-2.81080100	0.98444900
H	-4.66233000	2.02229700	-0.20799500
H	-4.73447400	4.34710300	-1.02370300
H	-2.64943600	5.47382800	-1.71784800
H	3.91445100	2.48185700	1.26961000
H	3.94536900	4.85821600	0.64104100
H	2.13461400	5.78957900	-0.76894500
H	0.81497100	6.27696700	-2.34908500
H	-0.35617500	6.41972400	-1.02295000
H	-0.89901800	6.17194900	-2.69902000
H	1.13530400	4.14497700	-3.57425000
H	0.33869500	2.65462600	-3.06254100
H	-0.61190200	3.93745800	-3.85259400
H	-3.19807100	0.70491300	4.28549500
H	-2.12044800	-0.22595700	3.24691400
H	-2.50245900	1.45049900	2.84544000
H	-5.33994500	-0.04280900	4.21988600
H	-8.62612600	-0.61318600	2.65232300
H	-7.80167000	-1.93928100	3.47522300
H	-7.77537400	-0.32101500	4.18121600
H	-7.33912800	-0.97635900	0.55492700
H	-6.38801200	-0.50883100	-1.46557200
H	-5.00439900	-1.60921300	-1.46869500
H	-4.75860700	0.12384200	-1.68361300
H	-1.65620400	0.17577200	-4.10577100
H	-0.91795200	0.72320400	-2.60087700
H	-2.62815000	1.05957100	-2.92451000
H	-2.11225900	-1.98185900	-4.30987400
H	-3.22502400	-5.36754100	-3.30368800
H	-1.56057800	-5.01472600	-3.78292500
H	-2.90234900	-4.29811500	-4.68361000
H	-2.76318900	-4.57800000	-0.97532400
H	-2.37680500	-3.85553300	1.15298200
H	-1.93936000	-2.21269100	1.57775000
H	-3.64768300	-2.64695200	1.37774000
C	1.55117900	0.92332900	4.67378800
C	0.30094000	1.22308100	4.19383300
C	0.37101400	1.05690700	2.78662000
C	1.64499300	0.63856400	2.43048400
N	2.35663000	0.58600600	3.61219600
H	1.94913900	0.93057300	5.67868000
H	-0.54787000	1.54220600	4.78416000
H	-0.39627700	1.30922600	2.06629700
C	3.77034900	0.31613400	3.80430700
H	4.34485400	0.69110700	2.95712500
H	4.10424500	0.84505400	4.69913200
H	3.97020900	-0.75077800	3.92905400
H	-1.49354300	-0.00274400	1.11152800

P22-TS

P	2.34804800	0.78462100	1.60023500
C	3.37575200	-0.47191700	0.78441600
C	4.64682300	-0.88637700	1.19125400
C	5.31685300	-1.87222800	0.47362300
C	4.73725800	-2.43329300	-0.66513800
C	3.48315900	-2.02177600	-1.11458300
C	2.82350400	-1.04982800	-0.36169200
O	1.57929100	-0.61741500	-0.71851300
C	0.73029800	-1.55753500	-1.30039300
C	-0.62403400	-1.43669600	-0.98524800
C	-1.44266100	-2.40519600	-1.58124300
C	-0.93119600	-3.43009100	-2.37292400
C	0.43430400	-3.51138400	-2.62820800
C	1.29648400	-2.54990500	-2.10313300
C	2.79484800	-2.47678000	-2.39620500
C	3.02019300	-1.38303000	-3.46496800
C	3.35591500	-3.80051300	-2.91184600
B	-1.17117800	-0.23648600	-0.00186500
C	-0.74499200	1.22376500	-0.67727100
C	-0.84424800	1.38052800	-2.06478100
F	-1.33685000	0.39176000	-2.82420900
C	-0.52071200	2.54014800	-2.75431900
F	-0.64318400	2.59862300	-4.08209700
C	-0.08913000	3.65683000	-2.05485200
F	0.24102200	4.77846100	-2.69476100
C	-0.01334400	3.57751000	-0.67878600
C	-0.33767300	2.38754500	-0.03483800
F	-0.20225100	2.45446900	1.31002400
F	0.43149200	4.62255100	0.03066900
C	-2.82597300	-0.32878000	0.11897600
C	-3.75219400	0.62986400	-0.28169100
F	-3.37700000	1.77400700	-0.88019300

C	-5.12703100	0.50855300	-0.09182200
F	-5.95115900	1.47894100	-0.49690200
C	-5.64194100	-0.61929700	0.51886400
F	-6.95509700	-0.75262800	0.70710200
C	-4.76638500	-1.61239000	0.93269300
C	-3.40628500	-1.44328700	0.72895200
F	-2.62700200	-2.45525000	1.15490800
F	-5.24017400	-2.71441400	1.52146300
C	2.91880700	0.89346600	3.35181100
C	2.72501200	-0.43912200	4.07613200
C	2.13297700	2.01024000	4.04819000
C	2.85510400	2.38066500	0.77142200
C	4.20757900	2.88362900	1.28244400
C	2.91155000	2.21714400	-0.75358000
H	5.10323700	-0.45528000	2.07789300
H	6.29679900	-2.20502500	0.79858400
H	5.28002100	-3.19800400	-1.21120200
H	-2.51602800	-2.36368600	-1.42421400
H	-1.60332400	-4.16831900	-2.79954500
H	0.82004300	-4.31476600	-3.24685800
H	2.50814800	-1.65704700	-4.39240200
H	4.08904900	-1.26555000	-3.67205400
H	2.62414800	-0.41931300	-3.12972200
H	2.86791800	-4.08043700	-3.84871700
H	4.42371900	-3.70725100	-3.12858800
H	3.21512900	-4.61138400	-2.19061100
H	3.98600300	1.14561200	3.33961900
H	3.12552400	-0.36379700	5.09200100
H	3.23054700	-1.26864000	3.57335100
H	1.66004500	-0.67911200	4.15393700
H	2.46658400	2.10487600	5.08609000
H	1.06346600	1.77405200	4.05788100
H	2.26092800	2.98306200	3.56424800
H	2.07274900	3.10131000	1.03217900
H	4.49609000	3.77158200	0.71092600
H	4.18888000	3.16353000	2.33847900
H	4.98770300	2.12828500	1.13302600
H	3.01388000	3.20700100	-1.21056800
H	3.78836700	1.62602600	-1.03917800
H	2.02454200	1.73498400	-1.16988800
H	0.72233600	0.26350700	1.56114100
C	-0.61823600	-0.39023200	1.58595900
C	-1.23805400	0.33092000	2.64386000
C	-1.21167700	-0.40752600	3.82111700
C	-0.59585500	-1.61519600	3.49862400
N	-0.26688800	-1.61899700	2.19833900
H	-1.66992000	1.31426000	2.52526800
H	-1.58383400	-0.12330400	4.79477600
H	-0.35448700	-2.46227100	4.12772500
C	0.38929100	-2.76236300	1.58573600
H	0.56771200	-3.50652500	2.36323000
H	-0.24095000	-3.19475500	0.80810500
H	1.35130500	-2.47330200	1.15730000

P22-P

P	-2.95605400	-1.18496700	-1.31600800
C	-3.55600200	-0.83997900	0.33936900
C	-4.86307400	-1.19643400	0.69515700
C	-5.37798400	-0.81230200	1.92336600
C	-4.60850000	-0.01936900	2.77266700
C	-3.32128100	0.38619400	2.42974400
C	-2.78082100	-0.07070300	1.22061700
O	-1.51013000	0.24576900	0.84650500
C	-0.57495000	0.51167100	1.86755500
C	0.77217000	0.25943300	1.57822900
C	1.65542000	0.52103800	2.63538300
C	1.23193600	0.96002300	3.88446900
C	-0.11557400	1.20496900	4.11162100
C	-1.04252500	1.00481800	3.08940300
C	-2.50688600	1.40365400	3.20877500
C	-2.68840200	2.76902600	2.50615100
C	-2.97239100	1.53308000	4.65836300
B	1.31919600	-0.15941500	0.08315700
C	1.08058200	1.23983100	-0.78068900
C	1.77734500	2.39194700	-0.40213900
F	2.72262900	2.31354400	0.54685300
C	1.58658700	3.64717900	-0.95599400
F	2.29722400	4.69732600	-0.53573200
C	0.64050900	3.81445000	-1.95873600
F	0.42684500	5.01437900	-2.50069800
C	-0.07299100	2.71053700	-2.38108000
C	0.16097500	1.47007200	-1.79230100
F	-0.61215300	0.47925200	-2.28777600
F	-1.00316500	2.83334500	-3.33864900
C	2.93945000	-0.55282400	0.12860600
C	3.93553100	-0.05758600	-0.70899200
F	3.67658900	0.90118800	-1.61659900
C	5.25225400	-0.50634200	-0.70554600

F	6.15131700	0.02671300	-1.53924100
C	5.63369500	-1.51371700	0.16224800
F	6.89155200	-1.95945900	0.18024200
C	4.68148400	-2.05653600	1.00987400
C	3.38038100	-1.57818700	0.96490500
F	2.51807600	-2.18013400	1.80620000
F	5.02197700	-3.03610100	1.85398100
C	-3.71556800	-2.71718100	-1.96889500
C	-3.55290900	-3.89349400	-1.00578600
C	-3.09714600	-3.01727800	-3.33930600
C	-3.43366600	0.24871100	-2.37874900
C	-4.87123100	0.09797000	-2.88158100
C	-3.27359400	1.56122300	-1.60238500
H	-5.47080100	-1.78600900	0.01477600
H	-6.38086500	-1.10675400	2.21132100
H	-5.03506800	0.30686500	3.71494500
H	2.71867600	0.38100200	2.46942100
H	1.95598800	1.12815000	4.67558900
H	-0.44337400	1.56836400	5.07907900
H	-2.09121800	3.53187200	3.01491700
H	-3.74085700	3.07182200	2.52630500
H	-2.36012000	2.72126100	1.46293400
H	-2.39795000	2.30587000	5.17368800
H	-4.01849800	1.84748000	4.69968200
H	-2.86504300	0.59264500	5.20728000
H	-4.78086500	-2.48678100	-2.09037500
H	-4.02418600	-4.77730300	-1.44561900
H	-4.01512000	-3.70914000	-0.03334300
H	-2.49450800	-4.11455100	-0.85072500
H	-3.58167300	-3.89994200	-3.76580300
H	-2.02864400	-3.22777100	-3.23396500
H	-3.21880400	-2.19270700	-4.04832700
H	-2.73051700	0.23431000	-3.21682100
H	-5.14530400	1.00454900	-3.42839100
H	-5.00077600	-0.74943000	-3.55936200
H	-5.57521100	-0.00462200	-2.04757600
H	-3.36483300	2.39632600	-2.30081500
H	-4.06269500	1.65708400	-0.84975800
H	-2.30831500	1.64190900	-1.09884700
H	-1.55533200	-1.32721100	-1.27992000
C	0.67875700	-1.48762300	-0.61187500
C	0.87413100	-1.87586500	-1.93337000
C	0.45789400	-3.21897200	-2.09695300
C	0.01112800	-3.63792300	-0.86269300
N	0.14406000	-2.59799900	0.02273800
H	1.31495100	-1.24076800	-2.69153300
H	0.50920800	-3.82208700	-2.99344300
H	-0.35369900	-4.59834900	-0.52602800
C	-0.33700200	-2.70501700	1.39000100
H	0.27580100	-2.10835900	2.05927600
H	-1.38073700	-2.37708100	1.47138800
H	-0.27659200	-3.75071400	1.69937000

P23_TS

C	1.44286100	-2.19281000	2.03961300
H	2.31329000	-1.83328600	2.59883600
H	1.65114700	-3.22076400	1.72524000
H	0.59547400	-2.20955400	2.72580500
C	1.18356500	-1.28847900	0.85804800
C	0.00319200	-0.72455200	0.50862300
P	2.64788000	-0.86310000	-0.11855000
C	3.88914500	-0.10502700	0.97231300
C	4.75164800	-0.87515600	1.76252400
H	4.73716500	-1.95901700	1.68680800
C	5.62882500	-0.24868300	2.64197800
H	6.29611800	-0.84806500	3.25255900
C	5.64223100	1.14065000	2.74759400
H	6.32106700	1.62298900	3.44334500
C	4.78702400	1.91007100	1.96320500
H	4.78592300	2.99147000	2.04756800
C	3.92111600	1.28864400	1.07054600
H	3.26310000	1.88976400	0.44958600
C	3.31355800	-2.43653500	-0.72968400
C	4.65627400	-2.55183600	-1.10747900
H	5.33938100	-1.72122000	-0.95092600
C	5.11940600	-3.73271500	-1.67618800
H	6.16287600	-3.82005100	-1.96073600
C	4.24608500	-4.79919100	-1.88238100
H	4.61063400	-5.71992600	-2.32616600
C	2.90791100	-4.68432900	-1.51782200
H	2.22564100	-5.51300800	-1.67585800
C	2.43736400	-3.50731300	-0.94246400
H	1.39431700	-3.42147000	-0.64690600
B	-0.32493400	0.42394200	-0.67862000
C	-1.17583300	-1.16515300	1.31576200
F	-0.81316800	-3.43540400	0.74769400
C	-1.55615400	-2.50603500	1.35933700
F	-3.02697000	-4.22578700	2.03081400

C	-2.70344800	-2.93518200	2.01049900
F	-4.61236900	-2.40334600	3.28102100
C	-3.51444900	-2.00655700	2.64797800
F	-3.94489700	0.23232800	3.23103200
C	-3.17092500	-0.66141100	2.62221400
F	-1.74082600	1.04250000	1.93386000
C	-2.01640000	-0.26279400	1.96345400
C	0.10067300	1.92975100	-0.16349600
F	1.03779600	1.35647800	1.96078100
C	0.74991800	2.26891500	1.02044900
F	1.84902000	3.79777900	2.45333300
C	1.17792600	3.56157300	1.32288300
F	1.33190200	5.83395700	0.71213000
C	0.91958300	4.59822300	0.44347400
F	-0.00823700	5.29397100	-1.61276300
C	0.22922000	4.32256400	-0.72972500
F	-0.78802900	2.81949400	-2.16129800
C	-0.16134100	3.02157500	-0.98984600
C	-1.96410100	0.33331500	-0.99252400
F	-2.59103500	2.56112000	-0.35505300
C	-2.91311900	1.34582300	-0.81774800
F	-5.10652300	2.21744100	-0.87558500
C	-4.27770800	1.18878700	-1.05033400
F	-6.08268400	-0.20381200	-1.65806300
C	-4.78107000	-0.03861000	-1.44143800
F	-4.35062300	-2.31170100	-1.89415500
C	-3.89920600	-1.09871000	-1.57287200
F	-1.80213100	-2.01248900	-1.38508400
C	-2.54780100	-0.88834300	-1.34052200
H	1.75860900	-0.18740800	-1.29763200
C	0.58872200	0.03988000	-2.06131600
C	0.47491500	-1.20000200	-2.74146800
C	1.05024200	-1.11770700	-4.00677500
C	1.53514200	0.17350000	-4.12285800
N	1.25351000	0.87515800	-3.00563000
H	0.02304600	-2.07866000	-2.31315000
H	1.13201000	-1.89763500	-4.74867200
H	2.10441000	0.63067000	-4.92105100
C	1.91752100	2.15569200	-2.78031000
H	2.80382300	2.19770900	-3.41459600
H	1.26252200	2.99260500	-3.02115800
H	2.23525700	2.23179200	-1.73957000

P23-P

C	1.55653900	-1.97834700	2.02261700
H	0.66138700	-2.22006000	2.59400500
H	2.21695300	-1.40807800	2.68570600
H	2.06464200	-2.91654200	1.77486200
C	1.22236300	-1.15599900	0.79451900
C	0.00769400	-0.67435800	0.45235400
P	2.73954700	-0.73785200	-0.09605100
C	3.91679500	0.06406100	1.01959600
C	4.75071900	-0.67171600	1.86832400
H	4.74858700	-1.75795400	1.83252400
C	5.59220400	-0.00472200	2.75239400
H	6.24193800	-0.57410200	3.40868600
C	5.59827100	1.38811500	2.79626000
H	6.25255200	1.90343300	3.49214800
C	4.77146500	2.12201300	1.94909900
H	4.77226000	3.20619400	1.98376500
C	3.93494600	1.46176900	1.05639700
H	3.29894200	2.03742400	0.38760900
C	3.49872600	-2.25395000	-0.72094200
C	4.83352800	-2.23170900	-1.13465700
H	5.42452100	-1.32481100	-1.03851700
C	5.40229400	-3.38056200	-1.67434800
H	6.43671300	-3.36692000	-2.00097100
C	4.64447700	-4.54344900	-1.79306600
H	5.09136000	-5.43851500	-2.21386200
C	3.31588300	-4.56411600	-1.37366900
H	2.72804100	-5.47120400	-1.46610600
C	2.73579800	-3.41882400	-0.83879600
H	1.69538000	-3.42437300	-0.52415600
B	-0.40684600	0.42162500	-0.75901100
C	-1.12874800	-1.19192000	1.27255800
F	-0.65622000	-3.42675300	0.64912100
C	-1.44645700	-2.54908600	1.27828700
F	-2.83576400	-4.35148300	1.90680100
C	-2.57000800	-3.04648200	1.92144000
F	-4.49674200	-2.63794900	3.21046800
C	-3.41920600	-2.17372500	2.58593600
F	-3.94691400	0.02670700	3.23503400
C	-3.13515600	-0.81522000	2.59972500
F	-1.78182100	0.96969000	1.95238100
C	-2.00464200	-0.34650900	1.94721000
C	-0.08221500	1.96551600	-0.25544200
F	1.05662900	1.46911100	1.79373600
C	0.62892400	2.35530700	0.87571100

F	1.71369100	3.95734500	2.23852400
C	0.97817600	3.67525200	1.15808100
F	0.88703100	5.95902800	0.57250900
C	0.56126500	4.69263700	0.32016200
F	-0.60830600	5.32763000	-1.62701000
C	-0.20101200	4.36752500	-0.79379700
F	-1.20396600	2.79996400	-2.16087700
C	-0.49919700	3.03967400	-1.04135100
C	-2.05582300	0.23022000	-1.01650600
F	-2.82665900	2.35981800	-0.21716400
C	-3.07194700	1.15393500	-0.75318400
F	-5.32478800	1.86501500	-0.69556500
C	-4.42753800	0.91273300	-0.96112000
F	-6.14493300	-0.57195800	-1.61061400
C	-4.85115300	-0.32113400	-1.41826100
F	-4.27234400	-2.52421500	-2.02093800
C	-3.89840200	-1.30138200	-1.63543300
F	-1.73701400	-2.06541900	-1.55298500
C	-2.55909300	-1.00730200	-1.42429300
H	2.58933900	0.18932700	-1.12907900
C	0.50271300	0.03275100	-2.08636100
C	0.76891100	-1.27413900	-2.49234500
C	1.77931500	-1.28438300	-3.48053500
C	2.13347500	0.02441600	-3.67484400
N	1.37109300	0.81943100	-2.85077700
H	0.29903300	-2.14943900	-2.07588100
H	2.20385000	-2.14545900	-3.97774900
H	2.84420100	0.47226200	-4.35461800
C	1.60424200	2.25699300	-2.86639300
H	2.39886200	2.45432200	-3.58669300
H	0.71621300	2.80167000	-3.18229700
H	1.93291600	2.62438600	-1.89085300

P24_TS

P	-1.90514400	0.37746900	0.43314800
B	1.00238500	-0.73433300	0.62426900
C	-0.51315100	1.16198500	-0.50781500
C	0.71896700	0.61883400	-0.30803600
C	-3.10216100	1.69625200	0.90974100
C	-3.95260100	2.29790500	-0.05558900
C	-4.80527500	3.32257200	0.34017600
H	-5.44064900	3.78766800	-0.41040000
C	-4.87372800	3.77082100	1.65952500
C	-4.07745300	3.13384800	2.59877200
H	-4.13612600	3.43790300	3.64166000
C	-3.20092900	2.09709600	2.25742500
C	-4.01384600	1.88157500	-1.50346200
H	-4.45572400	2.68322100	-2.09919200
H	-4.62690700	0.98226100	-1.62350800
H	-3.02828100	1.67221700	-1.91753800
C	-5.80081300	4.89424100	2.03991800
H	-5.54577200	5.80862400	1.49600500
H	-5.74859600	5.10884400	3.10910000
H	-6.83684300	4.64594800	1.79138600
C	-2.44904000	1.47683500	3.41141400
H	-1.62675800	2.12105500	3.73330000
H	-2.02360300	0.49871000	3.19187900
H	-3.12756900	1.36301100	4.26144200
C	-2.95695900	-0.84291600	-0.47646800
C	-2.83627500	-1.20099100	-1.83557600
C	-3.69932300	-2.16858800	-2.35740000
H	-3.58548500	-2.44652100	-3.40328800
C	-4.66242400	-2.80975200	-1.58819500
C	-4.76345800	-2.44616100	-0.24879900
H	-5.49629100	-2.94185800	0.38312700
C	-3.93460000	-1.48413500	0.32506500
C	-1.83870600	-0.61494800	-2.80308700
H	-1.62017700	-1.34412100	-3.58764500
H	-0.89536100	-0.35499100	-2.33003900
H	-2.23658700	0.28479000	-3.28566500
C	-5.51702400	-3.90373800	-2.16832600
H	-4.98469600	-4.85839800	-2.11217200
H	-5.74688300	-3.71210700	-3.21914400
H	-6.45646300	-4.00858500	-1.62103100
C	-4.12400100	-1.20420400	1.79770900
H	-4.62589600	-0.24614000	1.96495800
H	-3.17219700	-1.18416800	2.33284600
H	-4.73385100	-1.99133000	2.24487200
C	-0.73654500	2.38738200	-1.33526100
C	-1.24764000	3.57102100	-0.78818100
H	-1.46412200	3.62441900	0.27350700
C	-1.46539700	4.68640400	-1.59022300
H	-1.86599500	5.59251000	-1.14701600
C	-1.16377400	4.64646800	-2.94849100
H	-1.33837200	5.51721500	-3.57237000
C	-0.61783200	3.48932200	-3.49736900
H	-0.35600500	3.45409900	-4.55001700
C	-0.40254400	2.37284800	-2.69675600

H	0.03501500	1.47598600	-3.12571500
C	1.88488700	1.43067500	-0.79476300
C	2.09296900	2.73414600	-0.32952700
F	1.23748600	3.30318700	0.52718200
C	3.19410600	3.49779000	-0.68145900
F	3.33900900	4.72914100	-0.19697100
C	4.15543900	2.96880300	-1.52982900
F	5.21565900	3.68949300	-1.87682800
C	3.99350100	1.67964000	-2.00906000
F	4.90281700	1.15351100	-2.82633200
C	2.87929800	0.93544300	-1.63835700
F	2.79821100	-0.29301600	-2.14254300
C	0.03579600	-2.00241900	0.12884900
C	0.10622200	-2.45450700	-1.18955800
F	0.95957100	-1.86990500	-2.03633900
C	-0.67295800	-3.46623800	-1.72460200
F	-0.58523800	-3.77662800	-3.01956400
C	-1.60585900	-4.10860900	-0.92427300
F	-2.40898100	-5.03968900	-1.43456100
C	-1.72252400	-3.71711300	0.39485700
F	-2.65250700	-4.26321200	1.18077300
C	-0.91731000	-2.69051800	0.87813300
F	-1.20142800	-2.33918600	2.15196800
C	2.60184200	-1.14955000	0.67601900
C	3.14290000	-2.38173500	0.30480900
F	2.36461200	-3.38920200	-0.12306200
C	4.49470300	-2.69960800	0.38624300
F	4.92514100	-3.90667000	0.01938300
C	5.39494400	-1.76226300	0.86392800
F	6.69040400	-2.04916000	0.95360700
C	4.91580800	-0.52558200	1.26492300
F	5.75528900	0.39364700	1.74218200
C	3.55608100	-0.25674600	1.17499800
F	3.19502300	0.96006600	1.61397600
H	-0.77082800	-0.10753500	1.67448200
C	0.59319000	-0.23172000	2.16717200
C	0.65688700	1.08573100	2.68017100
C	0.92999900	1.05490900	4.04762900
C	1.08576200	-0.28350300	4.37483200
N	0.88884600	-1.04407800	3.28116400
H	0.48626600	1.96951700	2.08154200
H	1.01062400	1.89087400	4.72618600
H	1.33693900	-0.74356000	5.32205000
C	1.19881000	-2.46917800	3.27766200
H	2.18362300	-2.62196300	3.72500000
H	0.44550300	-3.02760200	3.83611500
H	1.22801000	-2.84015800	2.25571200

P24_P

P	-2.05299600	0.37144100	0.29303100
B	1.10480100	-0.71991600	0.78179500
C	-0.53516900	1.15560100	-0.37661600
C	0.69939700	0.64674400	-0.11002900
C	-3.29542600	1.62043000	0.77111000
C	-4.10028200	2.22800200	-0.21645800
C	-5.01917100	3.20008800	0.17520900
H	-5.62423600	3.67949400	-0.59012900
C	-5.17899100	3.57577100	1.50486900
C	-4.39246200	2.94206700	2.46387400
H	-4.50921900	3.21477500	3.50971600
C	-3.45310200	1.96593500	2.13436600
C	-4.02799800	1.88813600	-1.68312400
H	-4.50871700	2.67451400	-2.26806800
H	-4.53573600	0.94183800	-1.89492800
H	-2.99772000	1.81053800	-2.03707300
C	-6.19019800	4.61608100	1.90501800
H	-7.08400100	4.14206300	2.32302500
H	-6.50038800	5.21768800	1.04827200
H	-5.78573500	5.28462800	2.66892900
C	-2.68023100	1.32763200	3.26048600
H	-1.59895400	1.40041300	3.13490500
H	-2.91304000	0.26178300	3.36094300
H	-2.93810300	1.80682400	4.20617800
C	-2.94870900	-0.91393100	-0.65787700
C	-2.74052300	-1.19414000	-2.02207800
C	-3.52036700	-2.18119400	-2.62967100
H	-3.34108300	-2.40213400	-3.67875300
C	-4.47418000	-2.91275100	-1.93352100
C	-4.65004300	-2.63240600	-0.57973100
H	-5.37018700	-3.21022100	-0.00620500
C	-3.91016500	-1.65375400	0.07737300
C	-1.70863300	-0.52278600	-2.88914600
H	-1.42867400	-1.19345400	-3.70458700
H	-0.79952400	-0.28449500	-2.34196200
H	-2.09806100	0.40028700	-3.33192500
C	-5.23756100	-4.02559600	-2.59604200
H	-6.24060700	-4.12569000	-2.17517600
H	-4.71166000	-4.97232200	-2.43812300

H	-5.32416600	-3.86414000	-3.67250100
C	-4.13808900	-1.48422100	1.55947500
H	-4.48633400	-0.47761900	1.80744000
H	-3.22601800	-1.68764500	2.13097600
H	-4.89170200	-2.19461700	1.90117000
C	-0.74801600	2.41845200	-1.15794900
C	-1.27974900	3.56578000	-0.55911000
H	-1.52238300	3.55789200	0.49840300
C	-1.47918900	4.72504800	-1.30274900
H	-1.89562100	5.60381900	-0.82126000
C	-1.13400700	4.76258700	-2.65018100
H	-1.29095200	5.66764600	-3.22807700
C	-0.56284600	3.64090000	-3.24704000
H	-0.26286800	3.66905300	-4.28955900
C	-0.36889800	2.48067400	-2.50653400
H	0.09652200	1.61503900	-2.96859700
C	1.82030100	1.53203400	-0.57433100
C	2.03503300	2.79405800	-0.01817500
F	1.20644200	3.28177800	0.90832100
C	3.10572300	3.59596300	-0.38081000
F	3.27581100	4.78680400	0.18817100
C	4.00098800	3.15401200	-1.34433300
F	5.02429300	3.91872500	-1.70843300
C	3.80542300	1.91658400	-1.93764000
F	4.63807700	1.49181600	-2.88515200
C	2.72824800	1.13028700	-1.55009200
F	2.56406800	-0.03033000	-2.18388200
C	0.08588800	-1.96114600	0.34514400
C	0.06292100	-2.41620900	-0.97301800
F	0.87229400	-1.84657100	-1.87454300
C	-0.75505300	-3.42936700	-1.44468300
F	-0.76884000	-3.75104100	-2.74000400
C	-1.62667500	-4.05847300	-0.56891400
F	-2.46638100	-4.99632600	-1.00560000
C	-1.65406200	-3.65227800	0.75292500
F	-2.55029100	-4.18155700	1.59072400
C	-0.81376700	-2.62664800	1.17607800
F	-0.98751800	-2.25673400	2.46208300
C	2.70382300	-1.14302400	0.55673300
C	3.19353900	-2.35051900	0.05531100
F	2.37213300	-3.35375600	-0.30169900
C	4.54366000	-2.66063900	-0.07095100
F	4.91798500	-3.84184900	-0.56526900
C	5.50092000	-1.74520300	0.32910600
F	6.79741700	-2.02061500	0.21073000
C	5.07923200	-0.54290100	0.87284500
F	5.97716300	0.35209100	1.28711400
C	3.71956100	-0.28130700	0.98973100
F	3.42703200	0.89452900	1.56423100
H	-1.65338700	-0.21806100	1.49585400
C	0.97849300	-0.29814200	2.33881200
C	0.66378700	0.91754900	2.92361400
C	0.83226500	0.80954700	4.33086900
C	1.25772200	-0.47219600	4.57293900
N	1.35515800	-1.12887700	3.37495100
H	0.40179600	1.81336100	2.37915800
H	0.67006300	1.57763500	5.07350600
H	1.50063500	-0.97859000	5.49652200
C	1.82656800	-2.49483000	3.26246200
H	2.91773900	-2.54248400	3.34417800
H	1.38047900	-3.10271000	4.05346400
H	1.53992100	-2.91547500	2.29878000

P25_TS

B	-1.56465600	-0.26161200	0.05672800
C	-0.13356800	-1.48702400	2.17548200
C	-1.26538400	-0.76461900	1.62560900
C	1.17525100	-1.66235600	1.56876800
C	-0.27359200	-2.09352800	3.47388900
C	2.08978600	-2.55145700	2.10084900
H	3.03514500	-2.71502200	1.59911500
C	-2.31662800	-0.53843000	2.50258500
H	-3.16318300	0.03840700	2.15019900
C	-1.40794500	-1.84296900	4.28146900
H	-1.46144500	-2.30525700	5.26301500
C	0.71544900	-2.96665300	3.99844500
H	0.52942500	-3.42107700	4.96796000
C	1.85669400	-3.24264100	3.30521700
H	2.59797100	-3.93666400	3.68567600
C	-2.39702800	-1.02693100	3.81882500
H	-3.26384000	-0.79209800	4.42811000
C	-3.20753600	-0.23203300	-0.21381900
C	-3.98446600	-1.36527800	0.05681100
C	-3.92957800	0.81267300	-0.78733600
C	-5.34307000	-1.46197400	-0.20921100
C	-5.29150900	0.75960000	-1.07435400
C	-6.00812600	-0.38653700	-0.78014500
C	-0.91316800	1.24523800	-0.20379000

C	-0.10361200	1.64896300	-1.25859200
C	-1.18146800	2.27989500	0.69794000
C	-0.64718200	3.55628000	0.61483200
C	0.42850300	2.92409400	-1.41124500
C	0.17857500	3.88357100	-0.45077100
P	1.73185400	-0.72701300	0.08958300
C	2.33786900	0.99176900	0.43335800
C	2.12792600	1.69751400	1.64061700
C	3.04472600	1.61928900	-0.62348600
C	2.62861500	2.99631300	1.75546400
C	3.52948400	2.91554900	-0.44117800
C	3.32737100	3.62959800	0.73325400
H	2.45414800	3.53290700	2.68508600
H	4.05666100	3.38868000	-1.26633700
C	3.25543900	-1.56703200	-0.50958100
C	4.49818600	-1.38832400	0.13729800
C	3.19054200	-2.30461400	-1.71112500
C	5.63748300	-1.96686100	-0.42835200
C	4.35814500	-2.85378000	-2.23558100
C	5.59335600	-2.69842300	-1.60996000
H	6.59206000	-1.83068000	0.07509000
H	4.30006400	-3.41508100	-3.16526800
C	1.40815300	1.15224700	2.84955900
H	0.34711400	0.97820300	2.65140900
H	1.83404600	0.20757300	3.19934100
H	1.47719900	1.87243200	3.66670200
C	3.78758100	5.05540800	0.87093000
H	2.99953200	5.73154100	0.52482300
H	4.00950100	5.30302100	1.91154700
H	4.68010100	5.24601500	0.27030900
C	3.31876600	1.00673100	-1.98143400
H	2.51655100	0.35346200	-2.32315300
H	3.41665600	1.80776200	-2.71611600
H	4.24643700	0.42559100	-1.97641100
C	4.70605300	-0.58537000	1.40201800
H	4.92212100	0.46162100	1.16401100
H	3.84019100	-0.59419100	2.06539600
H	5.55827700	-0.98924900	1.95406500
C	6.83398500	-3.32461300	-2.18937300
H	6.86271700	-4.39697400	-1.97084000
H	6.86101700	-3.21106000	-3.27613500
H	7.73783900	-2.87459200	-1.77345700
C	1.90450700	-2.50856300	-2.46953800
H	1.18907900	-3.09478100	-1.88783000
H	1.41147900	-1.56151200	-2.71867700
H	2.09519500	-3.04347300	-3.40186600
F	-3.32914900	1.96102400	-1.15554500
F	-5.90438700	1.80503800	-1.63263500
F	-7.31080300	-0.46135100	-1.04478700
F	-6.01519600	-2.57689100	0.08192300
F	-3.43647200	-2.44638100	0.62736000
F	-2.01762300	2.07860900	1.72656400
F	-0.91641400	4.46965500	1.54747800
F	0.73467100	5.09165800	-0.53422300
F	1.24240700	3.19617000	-2.43521600
F	0.30110100	0.77076800	-2.20765500
C	-1.60790100	-2.73634000	-2.78376900
C	-1.32069600	-3.61559200	-1.74961900
C	-0.99854100	-2.82104500	-0.64926000
C	-1.05364100	-1.44853800	-1.00307300
N	-1.45987600	-1.46764600	-2.35663200
H	-1.91634800	-2.93782900	-3.80164800
H	-1.34873300	-4.69354700	-1.80346500
H	-0.74745200	-3.17752900	0.33947900
C	-1.88851200	-0.34192600	-3.17921200
H	-2.06890600	0.53174000	-2.55765800
H	-1.13364700	-0.09375800	-3.92767800
H	-2.82417700	-0.60931300	-3.67503300
H	0.33282300	-1.04086200	-0.78657500

P25-P

C	-2.94375200	-2.03755900	3.53202000
C	-1.75285000	-2.32906000	4.12863600
C	-0.51372600	-2.07130600	3.48814900
C	-0.41828100	-1.47285200	2.17284200
C	-1.71914100	-1.12113800	1.61876900
C	-2.90447900	-1.42649800	2.27227800
H	0.50304100	-2.89187500	5.20682400
H	-3.89311800	-2.25125100	4.01001400
H	-1.72398600	-2.78384400	5.11508200
C	0.63677300	-2.45230700	4.22277300
C	0.90974900	-1.31360600	1.58186800
H	-3.85354800	-1.16921100	1.81222200
C	1.96470400	-1.72157600	2.38579000
C	1.86860200	-2.26704700	3.67920500
H	2.96814200	-1.66426400	1.98778000
H	2.77064400	-2.54684100	4.21445400
P	-2.11005000	-0.20701900	0.09885800

B	1.37548100	-0.83998000	0.03503100
C	3.05845400	-0.84917300	-0.12106100
C	3.83392800	0.04395600	0.62806300
F	3.25920800	0.77978100	1.59730200
C	5.19900400	0.22819500	0.47929000
F	5.86087800	1.10049700	1.24531100
C	5.88121700	-0.50969400	-0.47611500
F	7.19556300	-0.35658800	-0.63982900
C	5.17376500	-1.41504500	-1.24333900
C	3.80015200	-1.56682400	-1.05847600
F	3.24264000	-2.48757300	-1.85828400
F	5.81324700	-2.14699500	-2.16046100
C	1.12283500	0.78591000	-0.26603400
C	1.49000300	1.37076500	-1.48037700
F	1.93158700	0.61410400	-2.49798600
C	1.48730300	2.73868300	-1.72768300
F	1.79530700	3.21324400	-2.93894300
C	1.19701500	3.61367200	-0.69532200
F	1.15508100	4.93051800	-0.90718300
C	0.91935300	3.09874400	0.55791800
C	0.86687800	1.72319500	0.73624300
F	0.55058300	1.33205300	1.98142500
F	0.67634500	3.93325200	1.57472300
C	-3.69655800	-0.84944800	-0.55597800
C	-4.88055300	-0.08102000	-0.66632000
C	-5.07356200	1.31678800	-0.12498300
C	-5.98753100	-0.66461900	-1.28413900
C	-5.97807100	-1.96947100	-1.77169400
C	-7.20194300	-2.56041400	-2.41631800
C	-4.82266200	-2.72197500	-1.59478100
C	-3.68427900	-2.19718400	-0.98439000
C	-2.53420600	-3.13725100	-0.73614700
C	-2.11509600	1.60493900	0.20253700
C	-2.22944400	2.33082100	1.40901900
C	-2.40390900	1.70809100	2.77190000
C	-2.19845100	3.72076700	1.33683900
C	-2.04684000	4.40975500	0.13357900
C	-1.89278000	5.90434400	0.13043100
C	-1.97832300	3.66973000	-1.04226500
C	-2.00958700	2.27464800	-1.03618700
C	-1.95249800	1.55585400	-2.36089600
H	-6.14118500	1.54345900	-0.09851100
H	-4.68710100	1.42356200	0.89121000
H	-4.58805500	2.07937400	-0.74030800
H	-6.89620300	-0.07459500	-1.37716200
H	-6.93954500	-3.38933500	-3.07754600
H	-7.88910200	-2.94635900	-1.65577100
H	-7.74381900	-1.81043400	-2.99782800
H	-4.79864200	-3.75472400	-1.93373700
H	-2.54692300	-3.94647900	-1.46762100
H	-2.62627100	-3.57955200	0.26299100
H	-1.55455700	-2.66300200	-0.79656100
H	-2.46132600	2.49636500	3.52474100
H	-1.57000900	1.05347500	3.03177500
H	-3.32443700	1.11880100	2.83563600
H	-2.26137100	4.28754000	2.26259600
H	-1.95390600	6.31335400	-0.88010700
H	-0.91473500	6.16948500	0.54537100
H	-2.65556800	6.38509900	0.74921500
H	-1.87088800	4.18450500	-1.99405300
H	-1.93982400	2.27816000	-3.17881000
H	-1.04881600	0.94679700	-2.44841200
H	-2.81230300	0.89319600	-2.51282100
C	0.36111800	-3.82187900	-2.12801900
C	-0.01018000	-2.84389200	-3.01664400
C	0.23652800	-1.60758600	-2.37001300
C	0.74994800	-1.84008300	-1.09585500
N	0.79076900	-3.22140800	-0.97508300
H	0.34097600	-4.89985800	-2.20718600
H	-0.40197400	-2.99753900	-4.01215900
H	0.12123300	-0.63672600	-2.82720700
C	1.35072200	-3.98568000	0.12296800
H	2.34786600	-3.61837500	0.37845900
H	0.72055800	-3.93684500	1.01496200
H	1.43673000	-5.02640300	-0.19448500
H	-1.15404100	-0.52927100	-0.86866200

6. Coordinates of intermediates and transition states involved in Figure 4.

IM1

P	3.12339900	-0.42689600	0.43326100
C	2.91340400	-1.49395400	-1.04527900

C	3.93586700	-2.15676000	-1.73117300
C	3.66127300	-3.17928800	-2.65221000
C	2.36224700	-3.60741400	-2.90115900
C	1.31622100	-2.97198500	-2.22575000
C	1.62571400	-1.92639900	-1.35151200
O	0.50218600	-1.39847400	-0.78194900
C	-0.56545100	-2.14554100	-1.24026000
C	-0.12876200	-3.13021300	-2.13443000
C	-1.07517100	-3.99257300	-2.69336500
C	-2.40138700	-3.81895000	-2.32227700
C	-2.78493000	-2.80311300	-1.43191800
C	-1.88321300	-1.90377300	-0.85146000
B	-2.26038800	-0.69341400	0.18301800
C	-3.88305900	-0.61237800	0.43871700
C	-4.69383800	-0.35083000	-0.66937800
F	-4.14445600	-0.23903700	-1.88882900
C	-6.06964400	-0.18559300	-0.60660800
F	-6.78055800	0.05080500	-1.71033400
C	-6.70737000	-0.25898800	0.62286700
F	-8.02655000	-0.09938900	0.70947100
C	-5.95101200	-0.49884400	1.75625800
C	-4.57487800	-0.66300900	1.64135600
F	-3.92740200	-0.86783200	2.80253300
F	-6.54185500	-0.56092800	2.95220200
C	-1.85700100	0.80139600	-0.38813100
C	-2.14427000	1.93267000	0.37765200
F	-2.71412200	1.79564900	1.59113600
C	-1.89562800	3.23882300	-0.02146900
F	-2.15695400	4.26731000	0.79059000
C	-1.37302500	3.46711400	-1.28232500
F	-1.09757000	4.70418600	-1.69435800
C	-1.11392500	2.38676700	-2.10548800
C	-1.36448200	1.09578900	-1.65942900
F	-1.10476400	0.13648600	-2.55818800
F	-0.59159500	2.59794600	-3.31714800
C	2.68312600	1.33340400	0.07935300
C	2.75402100	2.16175300	1.22912600
C	3.26375300	1.66007000	2.55986900
C	2.35151800	3.49316700	1.15089900
C	1.85650600	4.04544000	-0.02802400
C	1.36621300	5.46867900	-0.06575500
C	1.79686700	3.22323600	-1.14854800
C	2.19135600	1.87985100	-1.12583300
C	2.04867100	1.11544200	-2.41981200
C	4.95834300	-0.46649500	0.67959700
C	5.41724600	-1.33528300	1.69665800
C	4.47931600	-2.21951300	2.48446500
C	6.77949400	-1.39815400	1.98713000
C	7.71360900	-0.62373600	1.30195800
C	9.17591100	-0.68153900	1.65597200
C	7.25132600	0.20888100	0.28705400
C	5.89608100	0.30055300	-0.04239700
C	5.50992000	1.20348200	-1.18493600
H	4.96851900	-1.88617900	-1.52921000
H	4.48912700	-3.65991100	-3.16332200
H	2.16785400	-4.42048900	-3.59331600
H	-0.78228000	-4.77222500	-3.38941600
H	-3.16208800	-4.47506000	-2.73346200
H	-3.84027300	-2.72112600	-1.18396500
H	3.15481200	2.43468700	3.32237800
H	2.71083600	0.77406400	2.89225000
H	4.32191500	1.38150100	2.51497500
H	2.41816100	4.11280100	2.04283400
H	1.34571800	5.85788300	-1.08610600
H	0.34840000	5.53796100	0.33412900
H	2.00166200	6.12007600	0.54083000
H	1.41732800	3.63301800	-2.08300600
H	1.88431600	1.81329300	-3.24337000
H	1.19256000	0.43434600	-2.39733500
H	2.92725000	0.51396700	-2.65783900
H	5.04710600	-2.85946800	3.16416600
H	3.88452400	-2.86481300	1.83099000
H	3.77410000	-1.62944400	3.07850400
H	7.12046900	-2.07384200	2.76864500
H	9.40531400	0.00747100	2.47617200
H	9.80190900	-0.40121200	0.80515900
H	9.46663800	-1.68426700	1.98034500
H	7.96626000	0.80204300	-0.27928800
H	6.39085100	1.45638200	-1.77982900
H	4.78394900	0.72127800	-1.84402000
H	5.06011200	2.13712300	-0.83361800
H	-0.31947200	-1.18093700	1.07707500
C	-1.53253200	-1.83244300	3.66806400
C	-1.12855500	-0.48054900	3.84601900
C	-0.97277100	0.03648000	2.59721100
C	-1.27596500	-0.97549600	1.58276200
N	-1.59838900	-2.13429000	2.39730100
H	-1.77605400	-2.56902500	4.42560300

H	-0.96937000	0.00182100	4.79887200
H	-0.59828500	1.02066300	2.34878500
C	-2.04988300	-3.41336300	1.87402700
H	-3.01885400	-3.28501800	1.38552800
H	-1.33328200	-3.79216300	1.14428600
H	-2.14261500	-4.11458600	2.70385900

TS1

P	3.16517300	-0.48314200	0.46638100
C	2.96358300	-1.52138800	-1.02681800
C	3.98888100	-2.15633800	-1.73332100
C	3.71594000	-3.15761800	-2.67851200
C	2.41815800	-3.58297600	-2.93904200
C	1.36923600	-2.96964300	-2.24672100
C	1.67907000	-1.95706000	-1.33673700
O	0.55607900	-1.44262500	-0.75362400
C	-0.51809600	-2.14640000	-1.26579400
C	-0.07908500	-3.11001200	-2.18151700
C	-1.02867700	-3.93694700	-2.78655900
C	-2.35776100	-3.75351800	-2.43297600
C	-2.74403600	-2.75389000	-1.52484800
C	-1.83973500	-1.88106300	-0.91094400
B	-2.21670000	-0.64271600	0.08930500
C	-3.77494900	-0.73125700	0.61052200
C	-4.80082100	-0.55429200	-0.31764500
F	-4.50226700	-0.37480400	-1.61482700
C	-6.14996900	-0.54923600	0.00675900
F	-7.07985200	-0.38264900	-0.93544500
C	-6.52862200	-0.71214800	1.33068400
F	-7.81646800	-0.71157500	1.66921800
C	-5.54973600	-0.87660600	2.29619700
C	-4.21076500	-0.87497300	1.92305900
F	-3.33141700	-1.01840400	2.93292800
F	-5.89761500	-1.02849200	3.57598000
C	-2.06298700	0.83836200	-0.62691500
C	-2.41428400	1.98519300	0.08665400
F	-2.84036600	1.87883200	1.35969000
C	-2.34158100	3.27579600	-0.41864700
F	-2.66017500	4.32842800	0.33863400
C	-1.91148000	3.46243900	-1.72163800
F	-1.76920600	4.69166700	-2.21985700
C	-1.56348000	2.35955200	-2.48115300
C	-1.65459000	1.08463400	-1.93556100
F	-1.30152800	0.08997100	-2.76151900
F	-1.08541500	2.53671300	-3.71542200
C	2.50497800	1.22422200	0.18993500
C	2.52930400	2.02333300	1.36597300
C	3.26554300	1.59444300	2.61212400
C	1.88534600	3.25766400	1.38461500
C	1.22422400	3.76008300	0.26355900
C	0.51725700	5.08890400	0.30985300
C	1.25707700	2.99353100	-0.89501500
C	1.87088200	1.73558400	-0.96395600
C	1.80164900	1.04602300	-2.30598000
C	5.00006200	-0.32814200	0.63091600
C	5.59373900	-1.10461200	1.65430400
C	4.79905600	-2.08152400	2.48915800
C	6.96026200	-0.98784400	1.89901600
C	7.76946300	-0.12466300	1.15982900
C	9.24106300	-0.01251500	1.45806300
C	7.17810700	0.60998900	0.13784800
C	5.81182300	0.52128400	-0.14725500
C	5.27743900	1.32720800	-1.30175100
H	5.01946900	-1.87637200	-1.53294800
H	4.54446300	-3.61951400	-3.20558000
H	2.22676800	-4.37215800	-3.65898900
H	-0.73519600	-4.69812900	-3.50251800
H	-3.12104300	-4.38634600	-2.87476500
H	-3.80206400	-2.65968900	-1.30104500
H	3.01356200	2.24884400	3.45038500
H	3.02488800	0.56349000	2.89366200
H	4.35019700	1.64030300	2.46592100
H	1.90751000	3.84785900	2.29882200
H	0.01692800	5.30770600	-0.63644800
H	-0.23920200	5.10445400	1.10059500
H	1.22014400	5.90233700	0.51718200
H	0.80092000	3.38983600	-1.79959700
H	1.60381100	1.78638900	-3.08542100
H	0.98889700	0.31601000	-2.34520600
H	2.72102000	0.52113900	-2.56466800
H	5.45467600	-2.59415200	3.19685200
H	4.31483500	-2.84068000	1.86650800
H	4.00618400	-1.58261400	3.05509000
H	7.40788700	-1.58920800	2.68779200
H	9.72969200	0.69806300	0.78770300
H	9.73934400	-0.98087900	1.34782400
H	9.40814900	0.32220200	2.48671000
H	7.79517700	1.26951600	-0.46821100

H	6.10052800	1.69996900	-1.91581600
H	4.63229000	0.71652300	-1.93823900
H	4.68894500	2.18591000	-0.96563800
H	-0.04101100	-0.29700000	0.90485100
C	-0.10583200	-1.42321800	3.20342300
C	0.19883500	-0.08309000	3.15525600
C	-0.45362700	0.42160800	2.00947800
C	-1.16091600	-0.65644100	1.36179500
N	-0.87826800	-1.76462000	2.14759600
H	0.21488000	-2.17810100	3.90954600
H	0.82321500	0.46870000	3.84011600
H	-0.44698200	1.44063300	1.64352400
C	-1.39177400	-3.10825800	1.91409700
H	-1.36486300	-3.65333800	2.85796700
H	-2.42187200	-3.05137500	1.56223500
H	-0.78726600	-3.62856900	1.16810000

IM2

P	3.07649900	-0.47212200	0.47088800
C	2.82114900	-1.54401200	-0.99349900
C	3.83604300	-2.18765300	-1.70885700
C	3.55503700	-3.17183600	-2.66742300
C	2.25114900	-3.56555600	-2.94148700
C	1.21331600	-2.94700000	-2.23975000
C	1.52685600	-1.96271300	-1.29635600
O	0.40094200	-1.44984600	-0.71025000
C	-0.67075800	-2.11493700	-1.27540400
C	-0.23638700	-3.05761200	-2.21259600
C	-1.19026300	-3.83395900	-2.87604200
C	-2.52518000	-3.62026600	-2.56376600
C	-2.91058100	-2.64291900	-1.63103900
C	-1.99614500	-1.83225000	-0.95336800
B	-2.34274500	-0.65833900	0.12968700
C	-3.93741400	-0.60904800	0.51095700
C	-4.85548900	-0.27678800	-0.48431900
F	-4.42942800	-0.06766600	-1.73941000
C	-6.21816300	-0.13925800	-0.26158200
F	-7.04689100	0.17549200	-1.25799100
C	-6.71839100	-0.32161300	1.01980700
F	-8.02219000	-0.19323500	1.25810900
C	-5.84594100	-0.63729600	2.04785800
C	-4.48872400	-0.76349100	1.77685000
F	-3.70690800	-1.04830300	2.83609500
F	-6.30913000	-0.80697000	3.28793800
C	-1.96641100	0.85101400	-0.41290700
C	-2.15058300	1.94647400	0.42737200
F	-2.60656500	1.75186500	1.68311400
C	-1.89761600	3.26278900	0.06914900
F	-2.06373000	4.26098400	0.94145900
C	-1.46020900	3.53181800	-1.21655400
F	-1.17509400	4.77839500	-1.59226900
C	-1.28072600	2.48395000	-2.10186600
C	-1.54131200	1.18097200	-1.69753400
F	-1.34494200	0.24421400	-2.63518900
F	-0.81291500	2.73650100	-3.32784100
C	2.60679300	1.28365200	0.11487400
C	2.74656500	2.12853900	1.24669300
C	3.32668700	1.64649700	2.55549000
C	2.35492300	3.46356800	1.17170300
C	1.81362100	4.00870700	0.01094100
C	1.34553500	5.43913400	-0.02542600
C	1.68352600	3.17090600	-1.09218600
C	2.05458500	1.82107900	-1.07047300
C	1.82969600	1.04525500	-2.34587400
C	4.91873300	-0.46662700	0.64701100
C	5.43451100	-1.28057600	1.68030000
C	4.54815800	-2.15157300	2.53982000
C	6.80796100	-1.29507100	1.92650900
C	7.69554600	-0.52429600	1.18002900
C	9.17003900	-0.51772000	1.48271900
C	7.17667700	0.25363700	0.14773800
C	5.81033000	0.29564100	-0.13820900
C	5.35936900	1.14637000	-1.29733500
H	4.87018800	-1.92313300	-1.50790500
H	4.37887500	-3.63850900	-3.19754900
H	2.04490600	-4.33382600	-3.67986800
H	-0.89537700	-4.57810800	-3.60925500
H	-3.29202600	-4.20925300	-3.05706000
H	-3.97223900	-2.51817200	-1.43494100
H	3.19440400	2.40542400	3.32982900
H	2.84314000	0.72332100	2.89451900
H	4.39795900	1.43329300	2.47342800
H	2.47444400	4.09312300	2.05107300
H	1.30134400	5.81980400	-1.04823100
H	0.34062400	5.52665600	0.40205000
H	2.00771600	6.08538100	0.55745900
H	1.27159400	3.57606400	-2.01494900
H	1.58996700	1.73411900	-3.15893800

H	0.98955900	0.35150100	-2.25529700
H	2.69968000	0.45737000	-2.64487800
H	5.15644000	-2.76763600	3.20628700
H	3.92333700	-2.81803000	1.93811300
H	3.87204000	-1.54864200	3.15545300
H	7.19322000	-1.92919900	2.72188000
H	9.43462400	0.34751200	2.10024500
H	9.76203300	-0.45688200	0.56562400
H	9.46945300	-1.41671100	2.02682200
H	7.85582100	0.84386000	-0.46420100
H	6.21031600	1.39255500	-1.93706700
H	4.61608700	0.62730400	-1.90760200
H	4.90661800	2.08452100	-0.96150600
H	0.33105500	0.27407000	1.27958100
C	-0.27410700	-2.20423500	2.99619400
C	0.33106500	-1.01800600	3.04077600
C	-0.34162800	-0.12884000	2.04863600
C	-1.38738400	-0.99491800	1.42097800
N	-1.30736300	-2.15955500	2.01355100
H	-0.11341100	-3.12250000	3.54376200
H	1.14830600	-0.73591900	3.68851700
H	-0.82590900	0.73559000	2.52099100
C	-2.14942900	-3.32639200	1.78006900
H	-2.49489900	-3.69979800	2.74560300
H	-3.00274100	-3.05142000	1.16575800
H	-1.56695600	-4.09731700	1.26978500

TS2

P	-2.71478500	-0.32202000	-0.15948400
C	-2.70705600	-1.30437400	1.36992500
C	-3.78718700	-1.87787300	2.04639700
C	-3.55735800	-2.80985300	3.06894000
C	-2.27296100	-3.23506200	3.41775400
C	-1.17573900	-2.69174800	2.74443500
C	-1.45344600	-1.72375800	1.77261700
O	-0.34535200	-1.26427200	1.16322000
C	0.70444300	-1.98338400	1.67802100
C	0.27572500	-2.86748400	2.67262300
C	1.25525800	-3.66212200	3.28010700
C	2.56658800	-3.51497100	2.84175300
C	2.92136800	-2.60586700	1.82546800
C	1.98578900	-1.78423000	1.19395800
B	2.13242100	-0.71608900	-0.04119300
C	3.69544500	-0.63356400	-0.56115100
C	4.67274500	-0.24360800	0.35455400
F	4.33004500	0.00532800	1.62943500
C	6.01356800	-0.07754200	0.03771700
F	6.89867600	0.28930400	0.96785400
C	6.43056300	-0.28757000	-1.26776500
F	7.71251600	-0.13414100	-1.60001000
C	5.49865800	-0.65962300	-2.22143500
C	4.16538100	-0.81502600	-1.85806500
F	3.33537500	-1.16425100	-2.85514700
F	5.88629800	-0.85808100	-3.48445600
C	1.75621100	0.83217600	0.39820200
C	1.82568800	1.84331700	-0.56023300
F	2.16966900	1.54359500	-1.82617500
C	1.54461500	3.18011700	-0.31124000
F	1.58133200	4.08873000	-1.29117000
C	1.21201200	3.56797300	0.97513100
F	0.90341500	4.83859900	1.24382700
C	1.14670100	2.61101500	1.97055700
C	1.42410400	1.28239400	1.67331300
F	1.32122500	0.44132200	2.71570800
F	0.76331000	2.96832800	3.20185200
C	-2.41755500	1.47389400	0.02037000
C	-2.45512400	2.18632400	-1.20232400
C	-2.82430800	1.55103900	-2.52303600
C	-2.19240700	3.55429900	-1.19626200
C	-1.89643000	4.24336000	-0.02182300
C	-1.59093700	5.71599200	-0.06323800
C	-1.85423700	3.51864000	1.16622700
C	-2.09594100	2.14307600	1.21796800
C	-1.97838300	1.46534900	2.56045200
C	-4.41887700	-0.58306700	-0.78150700
C	-4.63341700	-1.67678500	-1.64233900
C	-3.52533400	-2.62989500	-2.00645700
C	-5.91394600	-1.90144200	-2.14711400
C	-6.98946700	-1.08586700	-1.80659800
C	-8.36838700	-1.34019400	-2.35308200
C	-6.76519700	-0.04219100	-0.90955300
C	-5.50422200	0.22508700	-0.37582100
C	-5.38913900	1.34098600	0.63458100
H	-4.80472700	-1.62188800	1.76494900
H	-4.41103600	-3.23811500	3.58348900
H	-2.14032800	-3.99065100	4.18540300
H	1.00433300	-4.37175800	4.06196900
H	3.34438700	-4.12146400	3.29554800

H	3.96550700	-2.55679200	1.52360200
H	-2.45617100	2.16539000	-3.34736500
H	-2.40646200	0.54811700	-2.65254300
H	-3.91095200	1.46092100	-2.62828800
H	-2.21998700	4.09651800	-2.13915900
H	-1.50379000	6.13521900	0.94090700
H	-0.64496600	5.89190000	-0.58449500
H	-2.37229900	6.26176400	-0.60031200
H	-1.59645100	4.03216700	2.08995800
H	-1.85020900	2.21548200	3.34278800
H	-1.10895300	0.80026500	2.60258700
H	-2.86006600	0.86823900	2.80668000
H	-3.90718600	-3.44304100	-2.62705300
H	-3.07103200	-3.07577300	-1.11397000
H	-2.72352200	-2.13293800	-2.55771800
H	-6.07331000	-2.74247600	-2.81752600
H	-9.04613100	-1.67165600	-1.55971400
H	-8.35322300	-2.11076300	-3.12699900
H	-8.79389900	-0.42947700	-2.78436300
H	-7.60269600	0.58028100	-0.60187100
H	-6.38097200	1.59192500	1.01724700
H	-4.76525700	1.05775400	1.48814400
H	-4.95736800	2.24841400	0.20154200
H	-1.29227500	-0.67780700	-1.06543800
C	0.26195100	-2.48765600	-2.88355300
C	-0.40450600	-1.30928300	-2.98973000
C	0.01286100	-0.48789500	-1.87123600
C	1.08001200	-1.18059700	-1.22166800
N	1.14042900	-2.39407200	-1.81436500
H	0.20701800	-3.39656500	-3.46539900
H	-1.14090500	-1.05965600	-3.74183300
H	-0.06242700	0.59519300	-1.85049800
C	2.03317100	-3.48410500	-1.45527300
H	2.95508600	-3.08799300	-1.03271900
H	1.56417800	-4.14222300	-0.71838300
H	2.27105100	-4.05409200	-2.35501300

P

P	-3.00385700	-0.31015900	-0.06586600
C	-2.79709400	-1.34013700	1.38743600
C	-3.83193400	-1.89892900	2.14475500
C	-3.53892100	-2.83448000	3.14089900
C	-2.23241100	-3.26706900	3.38243900
C	-1.18874200	-2.73653300	2.62514800
C	-1.50674000	-1.76295600	1.66759400
O	-0.42844800	-1.30828300	1.00725300
C	0.65619400	-2.01561200	1.50076300
C	0.25251100	-2.90809900	2.50004500
C	1.22901000	-3.70131600	3.11218000
C	2.53915300	-3.53972000	2.68553800
C	2.88243800	-2.60749900	1.68839600
C	1.94968800	-1.78654500	1.04945600
B	2.19938600	-0.68741000	-0.14388200
C	3.79992800	-0.66292400	-0.55831300
C	4.73872700	-0.25951500	0.39037200
F	4.33910600	0.04111700	1.63920500
C	6.09862600	-0.13999400	0.14196700
F	6.94276400	0.24791200	1.10324400
C	6.58050400	-0.42327200	-1.12670000
F	7.88305300	-0.31289900	-1.39509200
C	5.69203200	-0.82488200	-2.10938100
C	4.33669600	-0.93280500	-1.81319900
F	3.55979200	-1.33040200	-2.83284700
F	6.14373000	-1.10159000	-3.33680200
C	1.86580500	0.85747300	0.35913800
C	2.06850000	1.91532500	-0.52801800
F	2.52350500	1.67529500	-1.76825400
C	1.83002200	3.24969600	-0.22255400
F	2.00791700	4.21163300	-1.13645100
C	1.39201700	3.58424900	1.04702900
F	1.12124700	4.85343600	1.36756200
C	1.19106200	2.57779800	1.97261400
C	1.43534300	1.25613600	1.62192200
F	1.20652800	0.37011700	2.60868700
F	0.71511100	2.88584100	3.18696600
C	-2.52347800	1.43462600	0.00449200
C	-2.55451200	2.10410600	-1.24216900
C	-3.01084800	1.45239100	-2.52475700
C	-2.16493600	3.43881800	-1.28640600
C	-1.75606400	4.12693400	-0.14474100
C	-1.29243900	5.55362200	-0.24721100
C	-1.74501700	3.44351400	1.06866900
C	-2.11269400	2.10016800	1.17619700
C	-2.00892200	1.45044700	2.53320600
C	-4.68597600	-0.54211900	-0.67996100
C	-4.91389700	-1.53354900	-1.65900000
C	-3.82410300	-2.44551700	-2.16338000
C	-6.20623700	-1.67704600	-2.15585400

C -7.26371200 -0.88288100 -1.70875200
C -8.63921400 -1.04236900 -2.29629600
C -7.01391900 0.05779400 -0.71157600
C -5.73832100 0.24622100 -0.17887000
C -5.54679700 1.27194100 0.91108600
H -4.86251700 -1.62069200 1.94365900
H -4.35282700 -3.25253400 3.72302800
H -2.04089800 -4.01727000 4.14308000
H 0.97203600 -4.41240100 3.89075300
H 3.32313500 -4.14048600 3.13630600
H 3.92876400 -2.53414400 1.40135300
H -2.66761000 2.03729400 -3.38021800
H -2.61123300 0.44140200 -2.64860800
H -4.10332900 1.38973400 -2.57443200
H -2.17609700 3.95427600 -2.24368000
H -1.24481000 6.03145500 0.73288900
H -0.29035400 5.59078600 -0.68681000
H -1.95734200 6.13627000 -0.89064200
H -1.40915300 3.96068500 1.96458400
H -1.85081000 2.21518500 3.29499700
H -1.15656700 0.76376600 2.57940700
H -2.90505900 0.88709600 2.80505200
H -4.24390800 -3.19780500 -2.83329500
H -3.32373500 -2.97255200 -1.34363500
H -3.04875900 -1.90328200 -2.71667600
H -6.39338900 -2.43667500 -2.91068900
H -8.71370500 -0.50303400 -3.24660500
H -9.40615800 -0.64592100 -1.62751800
H -8.86373100 -2.09268600 -2.49822100
H -7.83418100 0.66055200 -0.32953600
H -6.50198500 1.48117700 1.39704500
H -4.85306600 0.92800500 1.68545000
H -5.15703700 2.21473100 0.51414400
H -2.11597300 -0.87166200 -0.99969200
C 0.12345100 -2.32974500 -2.89755800
C -0.40182000 -1.06277700 -3.01730700
C 0.26448700 -0.25438900 -2.05225100
C 1.18930100 -1.03643100 -1.37387400
N 1.07010700 -2.30392600 -1.91070900
H -0.07349100 -3.23844800 -3.44942400
H -1.12314900 -0.74803700 -3.76073200
H 0.09608300 0.80170200 -1.87989900
C 1.84991300 -3.46800800 -1.53747400
H 2.86390600 -3.17045800 -1.26755400
H 1.40635200 -3.99639200 -0.68745200
H 1.90361400 -4.14543000 -2.39247700

7. Coordinates of intermediates and transition states involved in Figure 5

CH4

N1_TS

C -0.93139800 2.53104300 -0.02723600
C -0.02408800 1.55535600 -0.45292700
C 0.74295100 1.89280800 -1.58009600
C 0.56687200 3.09353700 -2.25905400
C -0.37247000 4.02419000 -1.81593000
C -1.11845300 3.74857300 -0.67668300
H 1.48696000 1.18368100 -1.93544700
H 1.16753500 3.31119800 -3.13688100
H -0.51246300 4.96147400 -2.34441900
H -1.84129400 4.46952600 -0.30333300
H -0.89206600 1.16890200 1.53404400
B 0.13761600 0.10730900 0.28146800
N -1.66459700 2.20513200 1.18376900
C -1.05980700 -0.99010200 0.00838300
C -1.16347400 -2.16064600 0.80887600
C -2.05253300 -0.84483300 -1.00023800
C -2.26813200 -3.00978700 0.71217100
C -3.14634200 -1.71627800 -1.06209500
C -3.29849300 -2.78820700 -0.19234700
C 1.69750400 -0.37113000 0.13713400
C 2.08490800 -1.41671500 -0.73954100
C 2.74613600 0.31490700 0.79933600
C 3.42449900 -1.80031400 -0.84655500
C 4.07411000 -0.09936300 0.67109600
C 4.43747300 -1.17567500 -0.12815100
C 1.11453900 -2.16435000 -1.63058200
H 0.52873200 -2.90513600 -1.07777700
H 0.39743200 -1.49621000 -2.11234000
H 1.66529800 -2.68997600 -2.41489300
C 5.86724300 -1.64124900 -0.22127400
H 6.08452400 -2.05921200 -1.20778600
H 6.56504900 -0.82070800 -0.03517300
H 6.07323000 -2.42254300 0.51838800

C	-1.99195300	0.17059600	-2.12907000
H	-2.22472200	1.19555200	-1.82513700
H	-1.00255200	0.20464700	-2.59018400
H	-2.70738100	-0.11215300	-2.90515300
C	-4.47921900	-3.71979000	-0.28334400
H	-5.31908300	-3.24464100	-0.79624800
H	-4.22208100	-4.62751800	-0.83940600
H	-4.81624200	-4.03010000	0.70948200
C	-0.08224200	-2.64416300	1.76003900
H	0.90707400	-2.27057200	1.49369200
H	-0.28546700	-2.36506200	2.79869500
H	-0.04095100	-3.73647600	1.72412800
H	3.68232200	-2.61248500	-1.52419400
H	-2.30892400	-3.88672900	1.35722500
H	-3.89487300	-1.56011100	-1.83706500
C	-1.54781400	3.22737500	2.23182700
H	-1.92488800	2.81351200	3.17074000
H	-0.49647100	3.49637500	2.35070500
H	-2.12378200	4.12521700	1.98235100
C	-3.06456100	1.80538900	0.97122900
H	-3.47749900	1.45985800	1.92266200
H	-3.65580600	2.65441300	0.60693500
H	-3.10370400	0.98653800	0.25423900
H	4.84892800	0.45016700	1.20379900
C	2.52904900	1.54191100	1.66213400
H	3.40774200	2.18996600	1.60706500
H	1.66433800	2.12636300	1.34252700
H	2.38777800	1.27788300	2.71551500
C	-0.15667200	0.33221700	2.27378000
H	-1.02952900	-0.17307900	2.70171500
H	0.13999700	1.16111200	2.92783400
H	0.67113100	-0.36794100	2.32468300

N1_P

C	-1.28936900	2.17998800	-0.06478600
C	-0.04621900	1.55162200	-0.18442000
C	0.82864600	2.21883900	-1.06405900
C	0.48924900	3.38254000	-1.74236200
C	-0.77228700	3.95517000	-1.58008900
C	-1.67795100	3.34318600	-0.72633500
H	1.81301500	1.78495100	-1.22212000
H	1.20950100	3.84594100	-2.40968100
H	-1.04680200	4.86168200	-2.10834500
H	-2.66773300	3.76984300	-0.58382500
H	-1.85715100	0.66791600	1.13645700
B	0.32627900	0.12908200	0.63584100
N	-2.29773100	1.55346600	0.83874700
C	-0.87086500	-0.94992400	0.15354300
C	-1.52414400	-1.83208200	1.05206500
C	-1.35256700	-0.98180700	-1.18638000
C	-2.68531000	-2.52589100	0.68149400
C	-2.48687500	-1.71737400	-1.53785200
C	-3.20994100	-2.45677300	-0.60270800
C	1.89229500	-0.32935900	0.33495300
C	2.29072300	-1.55545900	-0.26357100
C	2.95862600	0.54372900	0.69953100
C	3.63908500	-1.82604400	-0.53399900
C	4.28997800	0.24326600	0.40257600
C	4.65861400	-0.93634800	-0.23180800
C	1.34813200	-2.69021700	-0.62121000
H	0.70561300	-2.97380700	0.21394500
H	0.68954400	-2.45263600	-1.45982200
H	1.93101900	-3.57063200	-0.90368000
C	6.09587800	-1.22961500	-0.57609300
H	6.26422300	-2.30368700	-0.68883400
H	6.38242200	-0.75014200	-1.51851000
H	6.77304300	-0.85749800	0.19780000
C	-0.61747900	-0.31054800	-2.33020600
H	-0.98681000	0.70027800	-2.53651400
H	0.45233800	-0.23270400	-2.12375300
H	-0.74720900	-0.90268100	-3.24111300
C	-4.47134800	-3.18692100	-0.98548700
H	-5.33869100	-2.51721500	-0.97269200
H	-4.39711400	-3.60128500	-1.99448000
H	-4.67934200	-4.00734700	-0.29432900
C	-0.93146600	-2.21352100	2.39683900
H	0.14337100	-2.03042500	2.41902200
H	-1.37882400	-1.68298600	3.24335000
H	-1.09486700	-3.28210600	2.56435800
H	3.89624600	-2.77705800	-0.99699400
H	-3.15802500	-3.18167500	1.41200100
H	-2.80747300	-1.72321400	-2.57930300
C	-2.56508300	2.37811400	2.04900200
H	-3.21314200	1.81451600	2.72226400
H	-1.61545400	2.59834100	2.53123000
H	-3.05580100	3.30032300	1.73708100
C	-3.56408500	1.17064700	0.15039800
H	-4.15159300	0.55573100	0.83412900

H	-4.11173300	2.07467000	-0.11478200
H	-3.30585700	0.59224100	-0.73496000
H	5.06483600	0.95216900	0.69114500
C	2.76725400	1.83120000	1.48040800
H	3.64173800	2.47597700	1.35754700
H	1.88959600	2.40056100	1.17739300
H	2.66548000	1.62097100	2.55010000
C	0.16191200	0.51917700	2.26291600
H	-0.82157600	0.30904500	2.71137300
H	0.35960500	1.57990500	2.45315100
H	0.87390300	-0.03121900	2.88678900

N2_TS

C	0.65504800	2.61550300	-0.04478400
C	-0.21734400	1.61797100	0.39983200
C	-1.03059100	1.97017000	1.48843300
C	-0.96522900	3.22645800	2.08348900
C	-0.08096700	4.19072300	1.60228800
C	0.73683400	3.88449900	0.52170600
H	-1.73528100	1.23772100	1.87245100
H	-1.60872000	3.45786900	2.92684600
H	-0.02769900	5.17072200	2.06531300
H	1.43559100	4.62085400	0.13309400
H	0.74855100	1.12554600	-1.54624900
B	-0.19776500	0.14623900	-0.31457900
N	1.49518700	2.23150800	-1.16794500
C	1.15610600	-0.66909400	0.06717600
C	2.09976400	-1.05554500	-0.89537800
C	1.46111000	-0.99592800	1.40578000
C	3.29835300	-1.70591300	-0.60271200
C	2.66035900	-1.64634300	1.70655300
C	3.59216500	-2.00291000	0.73453700
C	1.63675900	-0.60455600	-0.24143100
C	1.84670100	-1.99824900	-0.14990700
C	2.79045100	0.19241200	-0.34675700
C	3.15590600	-2.49339300	-0.12516600
C	4.09294800	-0.29850800	-0.33648700
C	4.28164900	-1.68151500	-0.20974700
C	5.66656200	-2.27219200	-0.17913300
H	6.22615800	-2.02796900	-1.08906600
H	5.62796300	-3.35987000	-0.08783700
H	6.24823400	-1.88240200	0.66390900
C	4.87285000	-2.70182500	1.10875000
H	4.93217700	-2.86097700	2.18761200
H	4.95415200	-3.67856200	0.61889200
H	5.75121300	-2.12109400	0.80521600
C	1.42448800	3.16223600	-2.30173200
H	1.96246200	2.72749700	-3.14900600
H	0.37844700	3.31200700	-2.57547800
H	1.87591000	4.13048000	-2.05912200
C	2.89211400	1.98619200	-0.76462600
H	3.41707200	1.47449400	-1.57588200
H	3.39146800	2.93852800	-0.54532900
H	2.90326900	1.34957100	0.11932700
C	0.73132100	-3.01857100	-0.09372800
H	0.00084500	-2.87481700	-0.89379700
H	0.17131700	-2.96810800	0.84475500
H	1.14391500	-4.02662300	-0.18583600
C	0.50686000	-0.69692700	2.53727300
H	0.56866400	0.34978900	2.85144100
H	0.53041600	-0.88555000	2.24078700
H	0.73280900	-1.32161700	3.40567400
C	4.25931700	-2.07797700	-1.70231100
H	5.24073500	-1.61243700	-1.55526400
H	4.42682400	-3.16008500	-1.74187800
H	3.87945700	-1.76319400	-2.67733700
C	5.27244700	0.63099500	-0.45808800
H	5.88253600	0.39244600	-1.33668400
H	5.93265900	0.55979400	0.41385600
H	4.94461400	1.66915700	-0.54694600
H	-3.30085700	-3.56957500	-0.04478300
H	-2.66573600	1.27005700	-0.44615100
H	2.87279000	-1.89303700	2.74595000
H	1.90587700	-0.84101700	-1.94505100
C	-0.16465200	0.40269200	-2.24887200
H	0.53053100	0.66424200	-3.06496900
H	-1.09258700	0.94690800	-2.42880800
H	-0.33699500	-0.66491700	-2.38087800

N2_P

C	1.00125900	2.41225900	-0.03990600
C	-0.14404600	1.62851600	0.12914100
C	-1.07215600	2.20114700	1.02187100
C	-0.87373000	3.42549900	1.65045100
C	0.28855200	4.16042600	1.42396600
C	1.24724700	3.64111100	0.56669800
H	-1.97822300	1.64543900	1.24117900
H	-1.62803900	3.80770900	2.33166500

H	0.45022700	5.11629400	1.91030800
H	2.16853500	4.18800500	0.38080500
H	1.70556900	0.96416600	-1.23913000
B	-0.31276700	0.12235100	-0.59813000
N	2.06560500	1.88236600	-0.93668000
C	1.00088500	-0.74533800	-0.07985800
C	1.90150100	-1.30800200	-1.00136900
C	1.35198200	-0.88221700	1.28428200
C	3.09527500	-1.94913700	-0.65319000
C	2.53881600	-1.52776300	1.64066400
C	3.42847500	-2.05398400	0.70250400
C	-1.79573800	-0.51925400	-0.28863800
C	-2.10991900	-1.83411000	0.11725000
C	-2.89878600	0.30263200	-0.58870900
C	-3.45120000	-2.21919700	0.24590800
C	-4.23304400	-0.07471800	-0.46898800
C	-4.51962000	-1.37309200	-0.02613300
C	-5.94257200	-1.84295300	0.12856000
H	-6.48913800	-1.78795900	-0.81996200
H	-5.97801300	-2.87763900	0.47723300
H	-6.49454600	-1.22599000	0.84696900
C	4.69769600	-2.73917100	1.13770000
H	4.80156100	-2.72030100	2.22477400
H	4.71557000	-3.78650300	0.81620000
H	5.58446700	-2.26172300	0.70526000
C	2.25835300	2.72076300	-2.14925900
H	2.93444200	2.20279000	-2.83184100
H	1.28560900	2.86937200	-2.61575200
H	2.68457700	3.67906900	-1.85155800
C	3.34690300	1.60200000	-0.22834500
H	4.03169600	1.12266200	-0.92979300
H	3.76315500	2.54220500	0.13318300
H	3.12989400	0.92558600	0.59952300
C	-1.07049900	-2.89857200	0.39537700
H	-0.34047700	-2.97389500	-0.41385600
H	-0.50394500	-2.70018700	1.31044900
H	-1.55348500	-3.87289500	0.51033100
C	0.46285800	-0.36446900	2.39113900
H	0.65490600	0.69383300	2.60237500
H	-0.59369600	-0.45041900	2.12134700
H	0.63380100	-0.92561000	3.31451200
C	3.99925900	-2.52547300	-1.71279200
H	5.00190000	-2.08270100	-1.67616600
H	4.12973700	-3.60550200	-1.58219700
H	3.58940800	-2.35720600	-2.71135900
C	-5.34388500	0.88323300	-0.81441800
H	-5.98171100	0.48860000	-1.61376200
H	-5.99698900	1.07205400	0.04558900
H	-4.94233100	1.84279600	-1.14869100
H	-3.66883000	-3.23806800	0.56419800
H	-2.70270400	1.31496200	-0.94437300
H	2.78058700	-1.63052000	2.69827300
H	1.65642200	-1.26070300	-2.06276100
C	-0.29277400	0.29994200	-2.25133900
H	0.63144400	0.64223400	-2.74998900
H	-1.07779800	0.99490700	-2.57008000
H	-0.52555000	-0.66363900	-2.72090700

N3_TS

C	-0.35534600	2.91545700	0.28702900
C	-0.18782600	1.59466500	0.69130100
C	-0.25177900	1.33397100	2.06701200
C	-0.46473500	2.35593300	2.98386200
C	-0.63192200	3.67047300	2.54650800
C	-0.57615600	3.95544400	1.18800900
H	-0.13156200	0.31149100	2.41962700
H	-0.50574100	2.13071000	4.04483000
H	-0.80310900	4.46725200	3.26275400
H	-0.69957800	4.97437600	0.83026200
H	-0.14193400	1.68767200	-1.49609000
B	0.01103600	0.36095900	-0.32779200
N	-0.25176900	3.13201800	-1.13906600
C	1.46348400	-0.34055700	-0.22659900
C	1.69107400	-1.64890000	-0.65300300
C	2.59874100	0.33013800	0.22337600
C	2.92649500	-2.27517500	-0.59832800
C	3.85736600	-0.25725400	0.29450700
C	4.02074300	-1.57164800	-0.11278800
C	-1.30333800	-0.57692900	-0.17302400
C	-1.35787700	-1.70404300	0.64023700
C	-2.51950600	-0.19855600	-0.73052200
C	-2.51772100	-2.43999700	0.84721500
C	-3.70187000	-0.90297600	-0.55780000
C	-3.69791700	-2.04000600	0.23762600
C	-1.41555200	3.78724000	-1.74034900
H	-1.32964200	3.73040300	-2.82963200
H	-2.31869500	3.26519500	-1.42486700
H	-1.48257300	4.84413400	-1.45131900

C	1.01235200	3.77482300	-1.51661500
H	1.11089000	3.76022300	-2.60615600
H	1.05206000	4.81503400	-1.16839900
H	1.83638500	3.21865700	-1.06499900
F	-4.83190800	-0.49399100	-1.13270400
F	-4.81832500	-2.73064600	0.42478700
F	-2.50900300	-3.51631900	1.63113000
F	-0.26253000	-2.11987100	1.29354000
F	-2.59192800	0.92288400	-1.47695800
F	0.68182900	-2.36388500	-1.17301000
F	3.07553800	-3.53143400	-1.01284000
F	5.21661700	-2.14782700	-0.05890800
F	4.90376000	0.43767100	0.73743900
F	2.53919000	1.62026300	0.58854700
C	0.09189300	0.69265200	-2.26659600
H	1.09258000	0.95019300	-2.61989700
H	-0.01587100	-0.38984500	-2.30856500
H	-0.66878200	1.08544700	-2.95069300

N3_P

C	-1.32526600	2.50694500	0.35521100
C	-0.17272500	1.72478700	0.43935400
C	0.61125900	1.99701300	1.57403300
C	0.28212200	2.97897500	2.50027900
C	-0.87035200	3.74699500	2.34202900
C	-1.69505100	3.50308100	1.25366700
H	1.50603000	1.40450600	1.73696700
H	0.92806200	3.14753000	3.35618500
H	-1.12893200	4.51738700	3.06016800
H	-2.60957900	4.07513800	1.11892600
H	-1.80815200	1.47276100	-1.30907900
B	0.16750100	0.52043500	-0.65167100
N	-2.25801500	2.22460300	-0.76962600
C	1.69069700	-0.05466400	-0.41663900
C	2.06822100	-1.39078400	-0.31230900
C	2.76501500	0.83607500	-0.43885900
C	3.38849800	-1.81539000	-0.20082100
C	4.09689200	0.46121300	-0.32896300
C	4.41299800	-0.88348600	-0.20736900
C	-0.99491500	-0.62173800	-0.32404800
C	-0.98771900	-1.28461100	0.90552900
C	-2.09011100	-0.93339800	-1.11658300
C	-1.95744800	-2.19006200	1.30759000
C	-3.09904800	-1.82421100	-0.76099700
C	-3.02732200	-2.46694100	0.46225100
C	-3.57951000	1.70817300	-0.30896500
H	-4.11273400	1.30336800	-1.17088100
H	-3.39678200	0.92415100	0.42500200
H	-4.13948400	2.52658300	0.14329000
C	-2.39919000	3.37255800	-1.70868200
H	-2.99684900	3.05328800	-2.56409200
H	-2.88908400	4.19553200	-1.18919600
H	-1.40123800	3.66998500	-2.02820700
F	-4.14079800	-2.02656200	-1.57129700
F	-3.97733800	-3.32039800	0.83525200
F	-1.88614500	-2.79100600	2.49378400
F	0.00838400	-1.05639300	1.76976700
F	-2.30396900	-0.27264600	-2.28687100
F	1.15489100	-2.37468400	-0.32437900
F	3.67503800	-3.11374600	-0.09422600
F	5.68221500	-1.27185300	-0.09943200
F	5.06894800	1.37503700	-0.34772400
F	2.53934500	2.15681200	-0.57138800
C	0.19617900	1.02683200	-2.22607200
H	1.00581400	1.75429100	-2.34060800
H	0.43449000	0.17242500	-2.86855500
H	-0.68742500	1.48341700	-2.69337000

N4_TS

C	-2.50881400	-0.11222600	-0.36872600
C	-1.87770000	0.74958000	0.52978300
C	-2.30808700	2.08099300	0.53720400
C	-3.32113600	2.51541000	-0.31320000
C	-3.93565100	1.62379600	-1.19153300
C	-3.53034500	0.29326000	-1.22218500
H	-1.84083800	2.78099300	1.22369600
H	-3.64002100	3.55290900	-0.28965200
H	-4.72708800	1.96408200	-1.85145900
H	-3.99807400	-0.41021100	-1.90626700
H	-1.32559100	-1.33906200	0.96894300
B	-0.72093700	0.18451500	1.49240200
N	-2.00322300	-1.47400900	-0.33997500
C	0.73410100	0.09606100	0.78093900
C	1.38444700	1.29447200	0.49434800
C	1.43021300	-1.04724100	0.41083100
C	2.63337300	1.36684200	-0.10659300
C	2.68002500	-1.02675600	-0.19733200
C	3.28798900	0.19216800	-0.45464800

C	-3.04612500	-2.49325100	-0.18630300
H	-2.57088400	-3.46446300	-0.02139800
H	-3.66696800	-2.24290400	0.67620200
H	-3.68264000	-2.56380500	-1.07658900
C	-1.13389700	-1.76746200	-1.48991000
H	-0.63329600	-2.72495400	-1.33107400
H	-1.72076100	-1.80752200	-2.41655400
H	-0.38551200	-0.97917400	-1.57858400
F	0.79162100	2.45425000	0.80777400
F	3.20820500	2.54248800	-0.35588200
F	4.48367300	0.23627300	-1.03668000
F	3.29122400	-2.16300300	-0.53487800
F	0.89007100	-2.26968600	0.60611200
H	-0.66175500	0.79808700	2.52217200
C	-1.15274800	-1.46566700	2.26482500
H	-0.25829200	-1.42413200	2.88278700
H	-2.04170500	-1.22539600	2.84620500
H	-1.21633000	-2.52173700	1.96635700

N4_P

C	2.36087700	-0.20508400	0.40569500
C	1.93689000	0.69340900	-0.57159100
C	2.58219800	1.93999700	-0.52081800
C	3.56261700	2.23684200	0.42012900
C	3.94438900	1.28980000	1.37059500
C	3.33258300	0.04291000	1.36928500
H	2.28817400	2.69021000	-1.24899300
H	4.03345400	3.21525500	0.41979600
H	4.70693400	1.51893200	2.10714000
H	3.60906800	-0.70697500	2.10675600
H	1.05982400	-1.49957900	-0.41140700
B	0.77308300	0.35004300	-1.68072000
N	1.68088300	-1.52720900	0.41110800
C	-0.67290500	0.22145300	-0.87940100
C	-1.31900500	1.38400200	-0.45463900
C	-1.36059500	-0.94425200	-0.56998800
C	-2.54728700	1.39402400	0.19407300
C	-2.58355200	-0.99585700	0.08892200
C	-3.18970900	0.19181400	0.46445100
C	2.61046200	-2.66705500	0.18499500
H	2.02171300	-3.57851600	0.06857200
H	3.17891500	-2.46109200	-0.72106700
H	3.28076700	-2.75465900	1.03995000
C	0.82280200	-1.72949400	1.61230900
H	0.20949000	-2.61785000	1.45572000
H	1.46518700	-1.84636500	2.48546400
H	0.19122800	-0.84867600	1.72249700
F	-0.74795200	2.57596300	-0.65976800
F	-3.11804600	2.54006100	0.56605300
F	-4.36349900	0.18128400	1.09408000
F	-3.16119400	-2.16646400	0.37341600
F	-0.79778600	-2.15773800	-0.83984800
H	0.67347300	1.32167300	-2.39488100
C	1.13304200	-0.93838500	-2.63709600
H	0.33115300	-1.08809500	-3.36813400
H	2.04144600	-0.71150500	-3.20609700
H	1.29702300	-1.93521800	-2.19638600

N5_TS

B	0.22682300	-0.10710400	-0.05774700
C	-0.75152500	-0.24821400	1.22780200
C	-0.14876900	-0.29418800	2.49598600
H	0.93242200	-0.27807100	2.57136000
C	-0.88641900	-0.31253300	3.67088500
H	-0.37714500	-0.33870400	4.62897500
C	-2.27610600	-0.28525600	3.61409400
H	-2.86872100	-0.28662400	4.52309900
C	-2.90570000	-0.26511500	2.37775200
H	-3.98646600	-0.26012100	2.33215200
C	-2.15473700	-0.25949100	1.19575500
N	-2.78907700	-0.31919800	-0.11406400
C	-3.45119000	-1.66621800	-0.39539100
C	-3.87275400	-1.69851900	-1.87499800
H	-2.98006000	-1.72688200	-2.50973800
H	-4.40652600	-2.63847000	-2.04980900
C	-4.72526400	-0.50194400	-2.27904100
H	-5.67481100	-0.50206200	-1.73093200
H	-4.98032000	-0.56509900	-3.34121300
C	-3.94738700	0.77878600	-2.00354700
H	-4.52925300	1.66169700	-2.28839700
H	-3.04364000	0.78294100	-2.62442600
C	-3.54326700	0.93516800	-0.52833700
C	-4.78862800	1.27953100	0.31012400
H	-5.56840000	0.52079400	0.28925000
H	-5.21968000	2.19714600	-0.10162500
H	-4.52525100	1.48976900	1.34767700
C	-2.60708800	2.13929800	-0.39229100
H	-1.67867800	2.01434200	-0.95123600

H	-2.35431000	2.32898600	0.65154700
H	-3.11699700	3.02276200	-0.78722500
C	-4.67098800	-2.02755200	0.47375400
H	-4.40415300	-2.11625600	1.52697000
H	-5.01719000	-3.01358700	0.14932500
H	-5.51348700	-1.34542900	0.37617200
C	-2.41510900	-2.76612600	-0.14311400
H	-1.49113000	-2.59911800	-0.69265500
H	-2.83272200	-3.72223200	-0.47181400
H	-2.17337600	-2.84817100	0.91950100
C	0.87803300	1.38287100	-0.23575900
C	0.59122900	2.48966300	0.56517700
F	-0.30009200	2.42595400	1.56231100
C	1.19608700	3.73285500	0.40518800
F	0.86362100	4.74815900	1.20081300
C	2.15410900	3.91283300	-0.57844800
F	2.74179800	5.09305600	-0.74220500
C	2.49340100	2.84087000	-1.39046100
F	3.42087500	2.98732600	-2.33470600
C	1.86588200	1.61936000	-1.19626600
F	2.27775100	0.62488300	-2.00410200
C	1.41983700	-1.23892500	-0.02988100
C	2.57068800	-1.00650800	0.72725900
F	2.72038900	0.14378200	1.40825300
C	3.62258400	-1.90413400	0.84742600
F	4.68204800	-1.60828900	1.59715500
C	3.56916600	-3.11233000	0.17007200
F	4.56429800	-3.98744700	0.26884000
C	2.46144800	-3.38931900	-0.61296600
F	2.38931500	-4.53740800	-1.28417100
C	1.43149400	-2.45971100	-0.69976000
F	0.42236100	-2.81440100	-1.51817500
H	-1.45649200	-0.30453900	-0.86034500
C	-0.58854500	-0.26432000	-1.81332300
H	-0.88858000	0.67232300	-2.28394500
H	0.45812700	-0.41006900	-2.06832800
H	-1.07790600	-1.11095900	-2.29944000

N5_P

B	0.35086000	-0.13091100	-0.28285700
C	-0.67306800	-0.45481000	0.99668900
C	-0.03229100	-0.66991000	2.23489700
H	1.05401900	-0.64863400	2.24931500
C	-0.70254400	-0.87303900	3.42943900
H	-0.14373700	-1.02337700	4.34761700
C	-2.09507100	-0.87239500	3.44841800
H	-2.64217900	-1.02159900	4.37302900
C	-2.78192300	-0.68194900	2.26077800
H	-3.86320900	-0.68781300	2.26803300
C	-2.07398700	-0.48928600	1.06933900
N	-2.86650200	-0.33251000	-0.18713800
C	-3.67792000	-1.59411400	-0.62016100
C	-4.12833900	-1.33501500	-2.06471400
H	-3.23496200	-1.29081500	-2.70349500
H	-4.70227400	-2.20806800	-2.39013500
C	-4.92376500	-0.04801600	-2.23453100
H	-5.84593100	-0.08016000	-1.64298500
H	-5.22809600	0.06639100	-3.27856300
C	-4.05159400	1.13143200	-1.82195900
H	-4.58787800	2.07948200	-1.93155900
H	-3.18218600	1.18441500	-2.49126800
C	-3.55354500	1.06129400	-0.37111900
C	-4.69217700	1.34575900	0.60870900
H	-5.60356700	0.78250100	0.41396400
H	-4.94135700	2.40365200	0.48849300
H	-4.38506600	1.20812900	1.64530800
C	-2.47777400	2.12206200	-0.15311100
H	-1.58713900	1.93751700	-0.75752200
H	-2.17589300	2.18131200	0.89258700
H	-2.89333900	3.08793600	-0.45327600
C	-4.86717900	-1.92604200	0.28589200
H	-4.53289800	-2.35279600	1.23178400
H	-5.44660900	-2.69762800	-0.22876100
H	-5.53905200	-1.09592700	0.48891000
C	-2.74051600	-2.79937600	-0.60174000
H	-1.94067400	-2.71296200	-1.33463300
H	-3.33511800	-3.68146500	-0.85531100
H	-2.29190500	-2.95585200	0.38065600
C	0.92840500	1.42422900	-0.16344000
C	0.61669100	2.37723600	0.80273600
F	-0.20711800	2.09979700	1.83417900
C	1.09629900	3.68524000	0.79743500
F	0.72584200	4.54201100	1.75258000
C	1.96268100	4.09445300	-0.19955800
F	2.43780200	5.33866200	-0.22058300
C	2.33600200	3.17984700	-1.17411800
F	3.19085400	3.54212000	-2.13192400
C	1.82484000	1.89227600	-1.12876100

F	2.29498900	1.05566100	-2.06982500
C	1.60841700	-1.21222200	-0.15316100
C	2.93670000	-0.92773000	0.16111600
F	3.33447400	0.31601100	0.47218000
C	3.94509800	-1.88633100	0.21513500
F	5.19452300	-1.53286400	0.51985100
C	3.64783800	-3.21571000	-0.03264200
F	4.59864300	-4.14645400	0.02289200
C	2.33737900	-3.56185600	-0.32458600
F	2.01403800	-4.83873800	-0.54274100
C	1.37120100	-2.56761500	-0.37027800
F	0.11807600	-2.99515400	-0.62637000
H	-2.12482400	-0.29903000	-0.90048200
C	-0.25836100	-0.29931000	-1.81518000
H	-0.92777500	0.51088400	-2.14461400
H	0.56813500	-0.27344300	-2.52686300
H	-0.76336100	-1.25459000	-1.99727000

N6_TS

C	-1.89521900	0.89478900	-0.00002800
C	-1.08087200	-0.23925700	0.00004700
C	-1.61840300	-1.53199600	0.00011400
C	-2.99692700	-1.70489300	0.00009800
C	-3.83300300	-0.58881600	0.00002000
C	-3.28272100	0.68531700	-0.00003900
H	-0.97306600	-2.40130700	0.00017900
H	-3.41419100	-2.70673400	0.00014700
H	-4.91113500	-0.71836200	0.00000600
H	-3.93857800	1.55250600	-0.00009500
C	1.04684300	-0.29790400	1.30744100
C	1.04681900	-0.29814000	-1.30737900
C	2.48951300	0.23285600	1.24343000
C	2.48939300	0.23285700	-1.24347100
C	3.24058200	-0.22907000	-0.00005500
H	3.00513300	-0.08879900	2.15453400
H	2.47652800	1.32792900	1.25975200
H	3.00501100	-0.08864000	-2.15463200
H	2.47620200	1.32793200	-1.25967500
H	4.25312000	0.18604000	-0.00009400
H	3.35287500	-1.31946000	-0.00005800
N	0.35125200	0.04617600	0.00000700
C	1.07845100	-1.79300100	1.67049400
H	0.07295200	-2.19872300	1.78512500
H	1.63215800	-2.41037900	0.96372800
H	1.57508400	-1.88703700	2.64128600
C	0.29095600	0.40653900	2.44421600
H	0.86983000	0.30059200	3.36647500
H	0.14064200	1.47024100	2.25352400
H	-0.69079800	-0.04579400	2.60294400
C	1.07866600	-1.79331400	-1.67011000
H	0.07323500	-2.19927500	-1.78447900
H	1.57513200	-1.88743700	-2.64097900
H	1.63264700	-2.41044100	-0.96334200
C	0.29075800	0.40588800	-2.44429700
H	0.14020700	1.46959500	-2.25385000
H	0.86963000	0.29984800	-3.36654600
H	-0.69090200	-0.04669900	-2.60288600
B	-1.36962300	2.41175000	-0.00002500
H	0.18534300	1.49805600	-0.00012700
H	-1.70102900	2.98178200	1.01061200
H	-1.70123500	2.98196400	-1.01048200
C	0.46307000	2.82516600	-0.00041800
H	1.09367800	2.77974200	0.89009200
H	1.09252000	2.78038200	-0.89176800
H	0.04090500	3.82837900	0.00030300

N6_P

C	-1.89770100	0.84965700	-0.04067400
C	-1.08139700	-0.28332100	0.02458700
C	-1.54919800	-1.59986900	0.09525400
C	-2.91722200	-1.82475200	0.09265200
C	-3.78457800	-0.73259800	0.01690400
C	-3.27833200	0.55564600	-0.04817200
H	-0.86902700	-2.44029000	0.15587200
H	-3.29998200	-2.83834300	0.14733600
H	-4.85827800	-0.89603200	0.00899000
H	-3.96208300	1.39835200	-0.10371600
C	1.10354500	-0.18221400	1.35904900
C	1.10364700	-0.42867700	-1.29987800
C	2.49812000	0.44781400	1.21546200
C	2.46619700	0.27763000	-1.27677600
C	3.27084300	-0.01967700	-0.01442600
H	3.05150700	0.22621600	2.13388300
H	2.38682800	1.53820500	1.17201000
H	3.01467600	-0.02593700	-2.17427800
H	2.29976800	1.36078000	-1.35430400
H	4.23183100	0.50063700	-0.05935400
H	3.50197400	-1.08903000	0.05411400

N	0.37916900	-0.00312600	0.00090900
C	1.19640700	-1.63476700	1.82161500
H	0.20934400	-2.08911500	1.90900800
H	1.83224600	-2.26255900	1.19857800
H	1.63581400	-1.61939000	2.82309900
C	0.29032400	0.60308700	2.39274100
H	0.87347700	0.64960600	3.31690900
H	0.07285000	1.62196000	2.06064200
H	-0.66129600	0.11094000	2.60430400
C	1.27140700	-1.93776200	-1.47115800
H	0.30991900	-2.45004300	-1.49188000
H	1.73875800	-2.09186900	-2.44819200
H	1.91497800	-2.40525500	-0.72817500
C	0.25948800	0.08854600	-2.46821100
H	-0.01810100	1.13638000	-2.34007900
H	0.85076000	-0.00738100	-3.38317200
H	-0.65773400	-0.49294200	-2.58066600
B	-1.49749000	2.45132300	-0.00232300
H	0.35741200	1.02775200	-0.09248700
H	-1.75447900	2.78877900	1.14711900
H	-2.28831000	2.99830600	-0.74866100
C	0.01213800	3.03914800	-0.37298200
H	0.86178100	2.79176600	0.28831500
H	0.36706300	2.89320600	-1.40358500
H	-0.09056800	4.12323200	-0.26231300

N8_TS

C	3.50325800	-3.72941200	-1.49616900
H	3.43275700	-3.90139300	-2.57408100
H	3.59897900	-4.71258500	-1.02467600
C	4.70841300	-2.80985900	-1.13418900
H	5.46511500	-3.34017400	-0.54844700
H	5.20878500	-2.43664800	-2.03324400
C	4.66971500	-0.56104900	0.23096800
H	5.73840200	-0.37483800	0.19121700
C	3.82315700	0.33167700	0.93084200
H	4.26988900	1.17299900	1.45439600
C	2.45799300	0.15131500	0.99982100
C	0.38999500	-1.13614300	0.15670000
C	0.03841100	-2.31269900	-0.49641500
H	-1.01802400	-2.50880400	-0.65163800
C	0.94794900	-3.25510200	-1.04482000
H	0.56366900	-4.13821600	-1.54781700
C	4.10102300	-1.66541600	-0.34874800
C	1.80711400	-0.92724400	0.32984000
C	2.28846000	-2.99567900	-0.96949500
C	2.69988100	-1.83102800	-0.28702500
C	1.99104900	0.64192000	3.29647500
H	1.83471900	-0.42774900	3.44438800
H	1.33075000	1.20294600	3.96196500
H	3.03311500	0.88809300	3.52503400
C	1.82964200	2.44078300	1.69004800
H	2.79088900	2.78824100	2.08681700
H	1.02155200	2.95253000	2.22043000
H	1.75504000	2.67021500	0.62850000
C	-0.47719500	1.45840700	-0.27888600
C	-1.15403300	2.61898800	0.18976200
C	-0.77437100	3.89762500	-0.22176300
H	-1.31544600	4.75636600	0.17428500
C	0.24219500	4.10721400	-1.14884000
C	0.80264000	2.97434500	-1.72332000
H	1.53064700	3.09398800	-2.52457800
C	0.45260700	1.67542200	-1.32994900
C	-2.39376400	2.57544600	1.06648100
H	-2.17780500	2.76479000	2.12245200
H	-2.92016800	1.62380900	0.98988100
H	-3.08309100	3.35880500	0.73866100
C	0.67481200	5.49549700	-1.54231600
H	1.15178000	5.49820800	-2.52560300
H	1.39573700	5.90171200	-0.82447200
H	-0.17616000	6.18137400	-1.57240400
C	1.07678400	0.59088800	-2.19186600
H	1.08946400	0.93807500	-3.22949800
H	0.53336500	-0.35162900	-2.16262000
H	2.11400900	0.38553100	-1.90587500
C	-2.28965500	-0.64984900	0.14199400
C	-3.09879000	-0.27193400	-0.96432000
C	-4.39103700	-0.78338500	-1.11516000
H	-4.98179600	-0.46062600	-1.97063600
C	-4.94322900	-1.69941500	-0.22785300
C	-4.12691200	-2.14295200	0.80274100
H	-4.49866200	-2.90760300	1.48351700
C	-2.83099600	-1.65358200	0.98871800
C	-2.64635400	0.65536000	-2.07474500
H	-1.64122100	0.41595000	-2.42624600
H	-2.63497400	1.70502400	-1.76883600
H	-3.32736900	0.56256000	-2.92460100
C	-6.35569300	-2.19666300	-0.39121400

H	-6.63191700	-2.26423100	-1.44695600
H	-7.06818000	-1.51809600	0.09027000
H	-6.48423600	-3.18366100	0.06044000
C	-2.06617200	-2.31346700	2.12220200
H	-0.98685100	-2.24842500	1.99324400
H	-2.33298100	-3.37315400	2.16796800
H	-2.32087400	-1.87448500	3.09306200
B	-0.78484100	-0.01713900	0.39765200
N	1.67280200	0.98813500	1.89471500
H	0.34104200	0.64479000	1.78434900
C	-0.83380600	0.39148000	2.37558200
H	-0.72499100	1.36813300	2.86697200
H	-1.90496800	0.27200900	2.25791100
H	-0.49394600	-0.38402800	3.06471800

N_P8

C	4.17503400	-3.17825100	-1.45224900
H	4.6403600	-3.18391100	-2.53014500
H	4.17382100	-4.22210300	-1.12562200
C	5.24497700	-2.33969100	-0.69476500
H	5.74708000	-2.93156200	0.07679200
H	6.02508100	-1.96629000	-1.36449300
C	4.90523000	-0.12502000	0.66113300
H	5.95312300	0.03731300	0.89001000
C	3.93526100	0.80864300	1.08771100
H	4.26911500	1.69753900	1.61543300
C	2.59427200	0.63519200	0.83049200
C	0.68381300	-0.83213200	-0.18487200
C	0.53435500	-1.94842900	-1.00259000
H	-0.48034600	-2.23154500	-1.26746300
C	1.57341000	-2.77482100	-1.49482200
H	1.32892500	-3.62580000	-2.12479500
C	4.47111200	-1.20033700	-0.06704400
C	2.05833400	-0.49373300	0.12317200
C	2.86225800	-2.49517400	-1.13541000
C	3.09136000	-1.35760900	-0.33313200
C	1.78627000	2.11830000	2.66801400
H	1.67652100	1.21505000	3.26198400
H	0.97357400	2.81467300	2.88088400
H	2.74630100	2.59525500	2.86178800
C	1.94446000	2.97331900	0.38166000
H	2.89419900	3.41805200	0.68027300
H	1.11030300	3.66042900	0.53994700
H	1.97557600	2.67052500	-0.66154900
C	-0.95452400	1.42892200	-0.13529300
C	-1.76598500	2.33166200	0.60580200
C	-1.90069100	3.67468700	0.23249700
H	-2.53382300	4.32429000	0.83606300
C	-1.31059600	4.18498100	-0.91834200
C	-0.60927000	3.28496300	-1.71533900
H	-0.19580800	3.63285000	-2.66115400
C	-0.42274100	1.94773300	-1.34987800
C	-2.66283600	1.88005400	1.74517100
H	-2.24677800	2.09839300	2.73417000
H	-2.86555900	0.80981300	1.69382100
H	-3.61780000	2.40903400	1.67469200
C	-1.45081900	5.63472500	-1.30580700
H	-0.58375300	6.22066000	-0.98073100
H	-2.33785500	6.08164300	-0.85022300
H	-1.53065800	5.74714800	-2.39023700
C	0.31522500	1.09830100	-2.36716200
H	0.35279100	1.62212800	-3.32604100
H	-0.17580200	0.13589500	-2.52080200
H	1.34523800	0.86691500	-2.07299100
C	-2.00740600	-1.15045900	0.21194900
C	-3.12303700	-0.89384300	0.63009700
C	-4.21866900	-1.76582900	-0.6703470
H	-5.05557500	-1.5206600	-1.32230400
C	-4.27270700	-293600900	0.07324900
C	-3.15771100	3.23639500	0.84417100
H	-3.13772500	4.17029900	1.40540100
C	-2.05311800	2.38489900	0.92113300
C	-3.23487200	0.29238600	-1.56921300
H	-2.33725700	0.42361700	2.17499400
H	-3.40519700	1.23721700	-1.04562500
H	-4.07374800	0.13771500	-2.25291000
C	-5.47793300	-3.8398210	0.04158900
H	-6.16803600	-3.60723300	0.86003500
H	-.18946100	-4.88940700	0.14681400
H	-6.03110200	-3.73020000	-0.89497100
C	-0.92562700	-2.91613400	1.79172100
H	-0.85738300	-400173800	1.67247300
H	-1.11296000	-2.72021900	2.85321900
H	0.04594600	-2.49505900	1.54414300
B	-0.70330200	-0.12988300	0.44861600
N	1.71092800	1.75782900	1.22185600
H	0.73739800	1.45849300	1.02314200
C	-0.45898500	-0.02930100	2.11969600

H	-0.44663700	0.98894300	2.52534400
H	-1.26981300	-0.51867700	2.66480600
H	0.46700700	-0.51995300	2.4529380

N10_TS

C	4.46859600	-2.67261000	0.38755900
H	5.44146400	-3.06815200	0.65869000
C	4.00427700	-1.48120000	0.98160100
H	4.64611000	-0.95600200	1.68023900
C	2.77205400	-0.96593400	0.66372700
C	0.52993000	-1.17852300	-0.55965400
C	-0.12556400	-1.88864500	-1.55411900
H	-1.14512700	-1.60537200	-1.79939300
C	0.42550000	-2.98725900	-2.24229000
H	-0.15573500	-3.48491800	-3.01250100
C	3.69200100	-3.28960300	-0.55082900
C	1.88034400	-1.60884100	-0.26209800
C	1.66554800	-3.44761900	-1.89929900
C	2.41385700	-2.77881000	-0.90110200
C	2.83272300	0.57509400	2.58906500
H	2.56806600	-0.27200800	3.22196200
H	2.32730000	1.47561800	2.94831400
H	3.91245500	0.74655100	2.63772400
C	2.91590100	1.41253200	0.34631300
H	4.00418900	1.46780300	0.47667200
H	2.44741700	2.36052000	0.62206800
H	2.69101700	1.19084800	-0.69291100
C	-0.09942100	1.53887400	-0.13382800
C	-0.41817900	2.55931000	0.79353200
C	-0.03459600	3.88804100	0.57136700
H	-0.28304600	4.63630800	1.32321500
C	0.60317400	4.28761400	-0.59453300
C	0.80390800	3.31109100	-1.56719300
H	1.24196400	3.60248200	-2.52055500
C	0.46669900	1.97284500	-1.36335600
C	-1.29783500	2.34294600	2.01163100
H	-0.72977000	2.24222600	2.94196300
H	-1.93945200	1.46728600	1.90328600
H	-1.95003200	3.21250200	2.13437700
C	0.98982300	5.72347500	-0.83784200
H	1.99650600	5.79749900	-1.25906700
H	0.96377200	6.30300400	0.08796300
H	0.30499600	6.19812400	-1.54821700
C	0.71786600	1.05366500	-2.54149500
H	0.96541700	1.64650800	-3.42548700
H	-0.15955100	0.44693700	-2.77854400
H	1.53643300	0.34706700	-2.37068900
C	-1.99758900	-0.50275300	0.17407700
C	-3.02535800	0.26972400	-0.43385800
C	-4.36270200	-0.14202300	-0.37470400
H	-5.11981300	0.48501100	-0.84161800
C	-4.76024400	-1.32024400	0.24021300
C	-3.75508600	-2.11967200	0.76658600
H	-4.01903000	-3.07752500	1.21328000
C	-2.41069500	-1.74420500	0.73890500
C	-2.80496400	1.54867100	-1.22045200
H	-2.00945600	1.44794700	-1.96020300
H	-2.54195600	2.40024700	-0.58826200
H	-3.72235000	1.80363100	-1.75667400
C	-6.21075000	-1.71659600	0.32768700
H	-6.79406300	-1.26537600	-0.47902900
H	-6.64999000	-1.38752300	1.27566900
H	-6.32941500	-2.80201400	0.27111500
C	-1.47786600	-2.77594000	1.35315800
H	-1.84220800	-3.77782500	1.10960500
H	-1.46369300	-2.69691800	2.44611800
H	-0.45231700	-2.70623500	0.99813600
B	-0.40012300	-0.04458200	0.21058800
N	2.38536500	0.32942400	1.20597300
H	1.01088300	0.18573600	1.34186300
H	2.09787000	-4.32241600	-2.37658000
H	4.03709200	-4.19018300	-1.05119000
C	0.03320000	-0.30930200	2.11970800
H	0.52211100	-1.25719200	2.36113500
H	0.31935300	0.43521600	2.86863900
H	-1.03167600	-0.43709800	2.27327300

N10_P

C	4.28222600	-2.96217400	0.39625200
H	5.24106800	-3.40067300	0.64763300
C	3.94843900	-1.67889000	0.86946100
H	4.67834100	-1.12114900	1.44779500
C	2.72898700	-1.12498900	0.57285300
C	0.36991100	-1.28354900	-0.45154500
C	-0.36003100	-2.02450800	-1.36905800
H	-1.36531500	-1.68728000	-1.60297800
C	0.07816100	-3.21934000	-1.97494500
H	-0.57317700	-3.73496100	-2.67414900

C	3.39454000	-3.60854300	-0.41707400
C	1.71035500	-1.77938200	-0.19869900
C	1.29134800	-3.74802800	-1.63617200
C	2.13452500	-3.04296100	-0.74470500
C	2.87196000	0.58421200	2.40125700
H	2.40399300	-0.17057400	3.02712400
H	2.46769300	1.57239000	2.62757200
H	3.95306100	0.58638700	2.53627600
C	3.36554300	1.19769100	0.09169600
H	4.42086000	1.02653100	0.30592100
H	3.07128200	2.22712900	0.30428300
H	3.14530900	0.95906900	-0.94581700
C	0.03655500	1.47711300	-0.06581900
C	-0.18502200	2.56565200	0.82278900
C	0.33628300	3.83919800	0.56650900
H	0.13874700	4.63836500	1.28022300
C	1.03541000	4.13183000	-0.59944000
C	1.15486200	3.10692300	-1.53343500
H	1.62941800	3.32065000	-2.49014100
C	0.67910600	1.81399000	-1.29069300
C	-1.12553300	2.47393900	2.01181900
H	-0.60361000	2.35888200	2.96737600
H	-1.82302800	1.64225800	1.90653500
H	-1.70766500	3.39815200	2.07313600
C	1.60503200	5.50299800	-0.85792400
H	2.64645100	5.57202400	-0.52412500
H	1.03988700	6.27231000	-0.32592400
H	1.58795300	5.74268400	-1.92419600
C	0.83166200	0.84028300	-2.44336100
H	1.10795500	1.38290500	-3.35129100
H	-0.09809300	0.30129100	-2.63681700
H	1.58978200	0.06914500	-2.26962900
C	-2.10601700	-0.32093800	0.19451100
C	-3.01066200	0.53354800	-0.49172200
C	-4.38860900	0.28055800	-0.49311100
H	-5.04406300	0.96921100	-1.02366600
C	-4.94924700	-0.81930700	0.14003000
C	-4.06965300	-1.69881500	0.75715000
H	-4.46414900	-2.59929200	1.22720800
C	-2.69177000	-1.47350700	0.79255500
C	-2.60102400	1.75417700	-1.29453800
H	-1.78838800	1.53883200	-1.98959700
H	-2.26859200	2.58555400	-0.66685300
H	-3.45255000	2.10546800	-1.88306300
C	-6.43813400	-1.05035700	0.15494900
H	-6.89620000	-0.61645300	1.05054400
H	-6.67379300	-2.11814900	0.15262100
H	-6.91902300	-0.59266900	-0.71364000
C	-1.90770700	-2.57053100	1.49495000
H	-2.36372500	-3.54017600	1.27338700
H	-1.93311900	-2.44380800	2.58288200
H	-0.86415900	-2.62115000	1.19361000
B	-0.46280900	-0.06965400	0.37555900
N	2.56146700	0.29228200	0.97017700
H	1.56673700	0.54728400	0.80932100
H	1.63346400	-4.69691000	-2.03864400
H	3.64119600	-4.57858000	-0.83937000
C	-0.11786600	-0.28881400	2.01423800
H	0.48174700	-1.18681100	2.22118300
H	0.39293700	0.55412000	2.49008200
H	-1.03723300	-0.40459400	2.59349000

N11_TS

B	0.20083100	-0.25431200	-0.07694100
N	-1.94399600	-2.26088800	0.04569000
C	-0.85071000	-2.42997400	1.01921300
H	-0.11010000	-3.06367000	0.51221900
C	-0.19095600	-1.07802600	1.28475000
H	-0.94331100	-0.47519000	1.81514700
C	0.83095000	-1.45885900	2.36473600
H	1.71222400	-1.92731400	1.92131000
H	1.16790700	-0.58876800	2.93152300
C	0.08159900	-2.49438500	3.25120200
H	0.74119900	-3.31579000	3.54063400
H	-0.27250300	-2.03338000	4.17651800
C	-1.12410500	-3.00633800	2.41315100
H	-2.05882900	-2.59819000	2.81056300
H	-1.21255600	-4.09608700	2.41724600
C	-2.31831200	-3.52792800	-0.60866900
H	-2.74775700	-4.20937100	0.14350800
H	-1.39855900	-3.98796900	-0.98478400
C	-3.31620900	-3.31334800	-1.74261100
H	-3.58924100	-4.28773500	-2.15927300
H	-2.83484700	-2.74557500	-2.54787000
C	-4.54973800	-2.55749300	-1.25369300
H	-5.22881200	-2.35188100	-2.08591400
H	-5.09858100	-3.18168200	-0.53571200
C	-4.11935400	-1.26060900	-0.57195200

H	-3.64836800	-0.59083600	-1.29971600
H	-4.98252600	-0.73309400	-0.15486300
C	-3.13222500	-1.54875400	0.55438400
H	-2.80216200	-0.61671800	1.01985400
H	-3.62559300	-2.15930300	1.32666700
C	-0.63983800	1.13952800	-0.10445900
F	0.56568800	1.69592600	1.83988200
C	-0.39413600	2.01263900	0.95645600
F	-0.77095900	3.99574500	2.18843700
C	-1.06588800	3.20505100	1.15841600
F	-2.73227700	4.71185300	0.43750300
C	-2.06481300	3.57476100	0.26469900
F	-3.31556600	3.07575700	-1.66642400
C	-2.35666100	2.74340100	-0.80228200
F	-2.00263900	0.82037500	-2.03837100
C	-1.64125300	1.55898800	-0.96923400
C	1.80358800	-0.12496500	-0.32402800
F	1.87769300	2.25062200	-0.50370000
C	2.50475500	1.06823500	-0.48897900
F	4.49337600	2.30582500	-0.80475200
C	3.88521200	1.13045100	-0.65726900
F	5.94758000	0.00536500	-0.84805900
C	4.62958400	-0.03726200	-0.68262300
F	4.67441000	-2.39082500	-0.56883200
C	3.97936300	-1.25450400	-0.53872100
F	2.04090700	-2.48720600	-0.24664900
C	2.60381000	-1.26653500	-0.36746800
H	-1.07247700	-1.42837700	-0.81095600
C	-0.21797800	-1.18219900	-1.75926900
H	-1.09360300	-1.38809000	-2.38795600
H	0.20932800	-0.30072600	-2.23236600
H	0.46896600	-2.02465900	-1.82672300

N11_P

B	0.46264100	-0.51279800	-0.43708300
N	-2.37632800	-1.80000600	-0.09412500
C	-1.13637300	-2.44240100	0.50857400
H	-0.75169600	-3.04998600	-0.31816900
C	-0.05314900	-1.43206900	0.87667800
H	-0.48712400	-0.75137100	1.62583500
C	0.84920000	-2.34995500	1.72008000
H	1.38067800	-3.06736300	1.09208200
H	1.59585000	-1.77953900	2.27842800
C	-0.14606900	-3.09031600	2.63067500

H	0.25448400	-4.01717700	3.04725100
H	-0.42550100	-2.44726000	3.47107100
C	-1.38750200	-3.34472700	1.73598500
H	-2.31238000	-3.09885200	2.26588100
H	-1.46474300	-4.39349900	1.43627600
C	-3.23223200	-2.81896000	-0.78445600
H	-3.59587100	-3.49636900	-0.00438400
H	-2.57288200	-3.37734900	-1.45314500
C	-4.37828800	-2.15798500	-1.53582700
H	-4.96446800	-2.93988600	-2.02633400
H	-3.96440100	-1.51778500	-2.32528200
C	-5.23513200	-1.32196600	-0.58737200
H	-6.04118600	-0.82777800	-1.13487600
H	-5.70237800	-1.97664300	0.15980500
C	-4.35166400	-0.28476100	0.10278900
H	-3.97962700	0.42422400	-0.64326300
H	-4.91759000	0.29645900	0.83635300
C	-3.18542600	-0.94163300	0.83568900
H	-2.50482400	-0.20311100	1.26219900
H	-3.55385100	-1.59493300	1.63047900
C	-0.45295500	0.87093800	-0.26193300
F	0.71235100	1.35828000	1.73440500
C	-0.26244800	1.67209700	0.86912100
F	-0.77533800	3.50349900	2.27887000
C	-1.02405100	2.78540400	1.18456600
F	-2.84372200	4.19580600	0.64487400
C	-2.07915500	3.14607700	0.35193300
F	-3.39244100	2.64083000	-1.54264500
C	-2.33336400	2.37788100	-0.76843600
F	-1.95205000	0.51644300	-2.08872200
C	-1.52901400	1.27198900	-1.03833600
C	2.08744200	-0.18324500	-0.38315200
F	1.97113900	2.20477600	-0.39662000
C	2.69516400	1.07266200	-0.37058500
F	4.56755700	2.51734200	-0.32561800
C	4.07129300	1.27886300	-0.35249200
F	6.25182900	0.37128700	-0.36049200
C	4.93136800	0.19464000	-0.37774200
F	5.19142200	-2.14420400	-0.49222900
C	4.38872800	-1.07862800	-0.43607300
F	2.58981800	-2.50876100	-0.57175200
C	3.00879800	-1.23061200	-0.45251800
H	-2.00642300	-1.17249900	-0.82529900
C	0.22954000	-1.24043200	-1.89906200

H	-0.77625800	-1.61092300	-2.15147700
H	0.48260800	-0.54664000	-2.70793000
H	0.89533100	-2.10252500	-2.00047300

N12_TS

N	2.60190100	-1.41477500	-0.25568000
H	1.37398000	-0.97187000	-0.99620800
B	-0.11531500	-0.37569500	-0.10686700
C	2.09680700	-1.10025400	1.10878500
H	2.34501300	-0.04001200	1.26807100
C	0.56943100	-1.22395600	1.10097200
H	0.32770300	-2.28754700	0.97804600
C	3.88748200	-0.74922100	-0.54124900
H	4.66370100	-1.16033700	0.12483700
H	3.77506800	0.31220600	-0.31501000
C	4.30807100	-0.94590900	-1.99259300
H	3.57423000	-0.45853800	-2.64536600
H	5.26500700	-0.44105400	-2.15593600
C	4.41396300	-2.43346900	-2.32656300
H	5.23222200	-2.87554900	-1.74239400
H	4.65975600	-2.57926600	-3.38234300
C	3.10684700	-3.14358000	-1.97547200
H	2.31112400	-2.81598300	-2.65286500
H	3.20608800	-4.22594200	-2.10367700
C	2.70909900	-2.86481400	-0.52692400
H	1.74746300	-3.33057100	-0.29108600
H	3.46642300	-3.30616800	0.13771500
C	2.74650700	-1.90743700	2.23726000
H	2.51598200	-2.97238800	2.10761000
H	3.83832000	-1.80417600	2.21077100
C	2.19450800	-1.45848400	3.59168200
H	2.65365100	-2.04351400	4.39480100
H	2.46670100	-0.40886400	3.76721100
C	0.67201800	-1.59536200	3.61899900
H	0.27853300	-1.25703000	4.58316200
H	0.39974000	-2.65464400	3.51678100
C	0.03284900	-0.79849400	2.48110500
H	0.24989000	0.26716200	2.63811100
H	-1.05511200	-0.90037200	2.51270500
C	0.22334200	1.21828300	-0.00371000
C	-0.53518300	2.03609000	0.83450200
F	-1.56990500	1.52199300	1.51620200
C	-0.29901700	3.39161100	1.01764500
F	-1.06140200	4.10842000	1.84068300
C	0.74351100	4.00091200	0.33346600
F	0.99137100	5.29700800	0.49552500
C	1.52261200	3.23803300	-0.52099300
F	2.52933700	3.79891200	-1.19060900
C	1.24465600	1.88443900	-0.67152600
F	2.04778000	1.22679600	-1.53558100
C	-1.70926700	-0.65906500	-0.32018600
C	-2.48383000	0.19222000	-1.11371400
F	-1.90833900	1.23250800	-1.74157000
C	-3.84346400	0.03735000	-1.33642100
F	-4.50885900	0.89484600	-2.10777800
C	-4.50847700	-1.02922200	-0.74860000
F	-5.81129500	-1.20153900	-0.94485700
C	-3.79157600	-1.91405400	0.03828300
F	-4.40700800	-2.95310700	0.60116000
C	-2.42692800	-1.71940800	0.23174400
F	-1.83773800	-2.64500200	1.00641900
C	0.43140000	-0.93389200	-1.89058000
H	1.30568900	-0.86994400	-2.55012800
H	-0.21313200	-0.14311600	-2.26941600
H	-0.03242400	-1.91198700	-2.02311900

N12_P

N	2.75233900	-1.42253600	-0.21075500
H	2.20621300	-0.89742500	-0.90663800
B	-0.16812700	-0.37757700	-0.33785100
C	2.04063200	-1.10647900	1.09925600
H	2.30113900	-0.05391600	1.28171700
C	0.52042800	-1.21361600	0.93425800
H	0.28638600	-2.28109400	0.82470800
C	4.15239400	-0.89697100	-0.27733100
H	4.75190400	-1.47807700	0.42927600
H	4.11570400	0.14301800	0.04910100
C	4.69215500	-1.02326700	-1.69540200
H	4.09371800	-0.38511000	-2.35700600
H	5.71341100	-0.63322400	-1.71314300
C	4.64650700	-2.47530700	-2.17076700
H	5.33014300	-3.07995100	-1.56088000
H	4.98994500	-2.54803200	-3.20573200
C	3.22700700	-3.02741200	-2.04301300
H	2.55427900	-2.51208200	-2.73847000
H	3.19393100	-4.09064300	-2.29648900
C	2.69525700	-2.86198100	-0.62412400
H	1.65533500	-3.17969800	-0.53781200

H	3.30320800	-3.42322600	0.09121800
C	2.58640200	-1.96432200	2.24131700
H	2.33131800	-3.01580300	2.05793700
H	3.67876400	-1.89358100	2.31367700
C	1.94484100	-1.53644200	3.56463700
H	2.31915200	-2.17190700	4.37303800
H	2.25264100	-0.50846400	3.79781900
C	0.42161600	-1.59883200	3.46777000
H	-0.02686600	-1.24670100	4.40246800
H	0.10357900	-2.64138200	3.33283600
C	-0.07609400	-0.76671500	2.28695100
H	0.18883700	0.28682200	2.46033500
H	-1.16565800	-0.79885800	2.23406200
C	0.21013900	1.22846900	-0.10942500
C	-0.58091400	2.10985000	0.63086500
F	-1.71515100	1.68452600	1.20526900
C	-0.27720300	3.44890500	0.84183600
F	-1.09354200	4.22695400	1.55165600
C	0.89106100	3.98096500	0.31472100
F	1.20602500	5.25959800	0.51045000
C	1.73357900	3.15022300	-0.40331200
F	2.88054100	3.61966400	-0.90193700
C	1.37259800	1.82122500	-0.58679100
F	2.29776800	1.08617400	-1.27465400
C	-1.81353200	-0.61093900	-0.34512400
C	-2.62959400	0.25116700	-1.08064700
F	-2.08986100	1.32254900	-1.68934800
C	-3.99614100	0.09259400	-1.25163100
F	-4.70295300	0.97194200	-1.96434200
C	-4.62917100	-1.00299600	-0.68180800
F	-5.94044200	-1.18343600	-0.83434100
C	-3.87142300	-1.90545900	0.04216800
F	-4.45237000	-2.97544700	0.59150600
C	-2.50170300	-1.69819900	0.18496300
F	-1.87008500	-2.65427400	0.90098200
C	0.22543000	-0.88474300	-1.87019300
H	1.21627100	-0.66205000	-2.29002500
H	-0.45940000	-0.39963300	-2.57352200
H	0.05532200	-1.96588800	-1.97615300

N13_TS

N	2.28684500	-0.38420100	-1.05352100
H	0.85879600	-0.26911200	-1.53257200
B	-0.49445300	-0.34270200	-0.34826700
C	1.97691600	-0.56721000	0.40193000
H	1.88530200	0.45389000	0.79048500
C	0.59737800	-1.21418500	0.47240400
H	0.28503700	-1.27990600	1.52036000
H	0.62879800	-2.24425400	0.10443800
C	2.82449700	-1.60947800	-1.68571600
H	2.13089700	-2.43163200	-1.48237700
H	3.78544900	-1.86540500	-1.21611400
C	3.02735100	-1.43736100	-3.18942600
H	2.06044100	-1.30948500	-3.68833100
H	3.46586000	-2.35919600	-3.58375500
C	3.92602100	-0.23751400	-3.48381100
H	4.93416600	-0.43802000	-3.09759400
H	4.02004700	-0.08038000	-4.56213800
C	3.36610400	1.00811600	-2.79946100
H	4.03535600	1.86322200	-2.93444900
H	2.40115300	1.28460300	-3.23985700
C	3.18134100	0.76123200	-1.30625400
H	4.16108300	0.56349400	-0.84166000
H	2.74663400	1.63949300	-0.82023100
C	3.08675800	-1.23942800	1.19085700
C	4.11319700	-0.45844600	1.73031100
H	4.07974400	0.62158400	1.60652800
C	5.16119200	-1.03861300	2.43920700
H	5.94486600	-0.41285400	2.85414500
C	5.19352800	-2.41740700	2.62500700
H	6.00544500	-2.87504800	3.18093200
C	4.17080500	-3.20605000	2.10477400
H	4.18228000	-4.28057900	2.25724900
C	3.12572800	-2.62136800	1.39538700
H	2.32782800	-3.24782200	1.00804200
C	-2.01140900	-0.92747300	-0.31579100
C	-3.07987900	-0.16795100	-0.79676400
F	-2.84792900	1.02865500	-1.36500200
C	-4.40582600	-0.57244400	-0.75198900
F	-5.37494100	0.20390300	-1.23337200
C	-4.71743500	-1.80265600	-0.19081200
F	-5.98013500	-2.21216500	-0.12914100
C	-3.69647900	-2.59989400	0.29957300
F	-3.98110500	-3.78380900	0.83962900
C	-2.37992000	-2.15546400	0.22915800
F	-1.46614600	-2.99866400	0.73068700
C	-0.46166700	1.21559400	0.12315300
C	0.04304500	2.30653800	-0.57270900

F	0.56143700	2.15570200	-1.80974500
C	0.07293800	3.59935300	-0.06202900
F	0.57861700	4.60071900	-0.78115400
C	-0.42761300	3.84082200	1.20780200
F	-0.40751500	5.06871100	1.71733200
C	-0.94180800	2.78434900	1.94709300
F	-1.41887900	3.00156600	3.17046500
C	-0.94429200	1.51011200	1.39724200
F	-1.43764700	0.52442100	2.16211400
C	-0.21166000	-0.41431400	-2.24981800
H	-1.09476700	0.20672600	-2.38008900
H	-0.41660300	-1.45787000	-2.49085800
H	0.47184900	-0.01303800	-3.00840800

N13_P

N	-2.33999500	-0.18829100	0.76901000
H	-1.55996300	0.34706000	1.17457700
B	0.67334300	-0.32378100	0.75698800
C	-1.68643300	-1.01189300	-0.36028600
H	-1.54248200	-0.26052700	-1.14311400
C	-0.31102000	-1.49308800	0.08281800
H	0.14069700	-1.91105800	-0.82301500
H	-0.40289000	-2.33758000	0.77644300
C	-2.89695700	-1.00358100	1.89477000
H	-2.10585500	-1.68789300	2.20950500
H	-3.73060400	-1.58143600	1.48684300
C	-3.34998800	-0.08875400	3.02679100
H	-2.48230100	0.44661500	3.43160000
H	-3.74735900	-0.71313800	3.83168300
C	-4.39363000	0.91477200	2.53671800
H	-5.29696200	0.37819200	2.21889400
H	-4.68566200	1.58546100	3.34850400
C	-3.83430600	1.71815900	1.36178800
H	-4.59451800	2.38090300	0.93895400
H	-3.00377000	2.35131500	1.69332000
C	-3.34522000	0.79448800	0.25378000
H	-4.16156700	0.20344300	-0.17374900
H	-2.85125900	1.35213200	-0.54701600
C	-2.66006700	-2.04508900	-0.88938400
C	-3.47225600	-1.72542800	-1.98144200
H	-3.37863300	-0.74633200	-2.44594300
C	-4.37772100	-2.64922700	-2.49620700
H	-4.99271600	-2.38509900	-3.35013400
C	-4.47976800	-3.91233500	-1.92210300
H	-5.18113200	-4.63744000	-2.32166100
C	-3.66599000	-4.24882700	-0.84272800
H	-3.73041700	-5.23812600	-0.40152100
C	-2.75847700	-3.32484300	-0.33416600
H	-2.11327800	-3.60681400	0.49193900
C	2.25918600	-0.65756600	0.47585800
C	3.23914300	0.33214100	0.45558000
F	2.90190100	1.62475900	0.61627400
C	4.59545000	0.08118400	0.28956100
F	5.47726400	1.08295500	0.27165800
C	5.03325800	-1.22726200	0.15162300
F	6.32965000	-1.49459000	-0.00500800
C	4.10375600	-2.25444600	0.19135200
F	4.50798200	-3.52179800	0.07531200
C	2.75794400	-1.95140800	0.35943600
F	1.92926100	-3.01214100	0.42391800
C	0.31101100	1.13045900	0.02241500
C	-0.28268400	2.23107400	0.62674600
F	-0.68746400	2.16376100	1.92213600
C	-0.59817100	3.41937400	-0.02418200
F	-1.20971300	4.40993400	0.63010300
C	-0.29105600	3.55186700	-1.36851400
F	-0.57952900	4.67546200	-2.02126700
C	0.29907300	2.48262600	-2.03282800
F	0.57443000	2.58814300	-3.33201200
C	0.57590200	1.31436100	-1.33511000
F	1.10130400	0.30642600	-2.04776400
C	0.52563600	-0.29236600	2.39114100
H	1.14552000	0.49035600	2.84149600
H	0.88362000	-1.25264200	2.78350200
H	-0.48068700	-0.15300200	2.81016700

N15_TS

N	2.28071000	-0.21996200	-1.29707600
B	-0.44059200	-0.35013300	-0.42067100
C	2.08284500	-0.49352200	0.16310600
H	1.98162200	0.50055800	0.61407900
C	0.73526300	-1.20015100	0.29726700
H	0.50209000	-1.32385200	1.35985500
H	0.78025900	-2.21274900	-0.11778800
C	2.76205300	-1.38212000	-2.08123800
C	4.27498500	-1.58346400	-2.18005100
H	4.76563300	-0.75351500	-2.69442700
H	4.46489300	-2.48755200	-2.76476200

C	2.98050300	1.43022100	-3.04270700
H	3.50409600	2.38132000	-3.16481900
H	1.95062800	1.57500300	-3.37342700
C	3.02961200	1.01768200	-1.57659300
C	3.26696900	-1.16552500	0.83137400
C	4.34471900	-0.38333200	1.25906700
H	4.28466300	0.69898200	1.17013200
C	5.48509800	-0.96925500	1.79927800
H	6.31274700	-0.34603700	2.12259600
C	5.55803300	-2.35365200	1.93272800
H	6.44506600	-2.81467800	2.35480700
C	4.47855800	-3.14081300	1.54086000
H	4.52018400	-4.21864300	1.66171900
C	3.33990500	-2.55015900	0.99908400
H	2.50146100	-3.17532800	0.70812300
C	-1.93171700	-0.98448500	-0.31991700
C	-3.04381700	-0.26501100	-0.76134400
F	-2.87792400	0.94028400	-1.33403900
C	-4.35068800	-0.72098100	-0.67181400
F	-5.36417400	0.01691700	-1.12075600
C	-4.59473800	-1.96239700	-0.10144400
F	-5.83757100	-2.42207700	0.00024300
C	-3.52777600	-2.71876600	0.35568000
F	-3.74882300	-3.91188100	0.90529300
C	-2.23255200	-2.22446500	0.23880300
F	-1.26723900	-3.02920600	0.70515700
C	-0.44329800	1.19613400	0.08770300
C	-0.10767700	2.33651700	-0.62996700
F	0.26327200	2.24814200	-1.92284600
C	-0.11197600	3.61713700	-0.08892000
F	0.23350200	4.66796800	-0.83210700
C	-0.47727400	3.79424800	1.23653700
F	-0.48652300	5.00960300	1.77528000
C	-0.82573900	2.68706200	1.99822000
F	-1.17598100	2.84203900	3.27298700
C	-0.80035600	1.42785300	1.41524700
F	-1.14344700	0.39454700	2.19951900
H	4.74423100	-1.70820900	-1.20331600
H	3.46246500	0.70106200	-3.69854900
H	2.34910200	-1.28185400	-3.09258900
H	2.29975300	-2.27427000	-1.64867100
H	2.57726100	1.80645300	-0.96740700
H	4.07311700	0.90996500	-1.24830500
H	0.82136600	-0.17993700	-1.67239600
C	-0.26267300	-0.40804500	-2.34384000
H	-1.15104000	0.20674300	-2.46271900
H	0.39416200	-0.06207700	-3.15530900
H	-0.47117500	-1.46227100	-2.52525400

N15_P

N	-2.36255000	0.08536100	1.01778900
B	0.59867600	-0.36478300	0.79093800
C	-1.88804900	-0.78147500	-0.17609300
H	-1.73061900	-0.03731800	-0.96247300
C	-0.53903300	-1.40464100	0.15538500
H	-0.19875400	-1.83458800	-0.79083000
H	-0.65886500	-2.25623400	0.83689300
C	-2.95645500	-0.69957100	2.16205000
C	-4.46105700	-0.92054600	2.09563300
H	-5.01659500	0.01416800	2.19863100
H	-4.73554200	-1.55648300	2.94059500
C	-3.33599600	2.28771600	1.71833200
H	-3.88295600	3.14984800	1.33072900
H	-2.36204600	2.63813300	2.06757200
C	-3.17935600	1.26854200	0.60138200
C	-2.99754500	-1.70448100	-0.63314300
C	-3.96387500	-1.22499600	-1.52314000
H	-3.86668400	-0.21984800	-1.92697800
C	-5.03817600	-2.02063900	-1.90975700
H	-5.77834100	-1.63169400	-2.60129200
C	-5.15180100	-3.31817200	-1.41772200
H	-5.98793000	-3.94227600	-1.71544800
C	-4.17457200	-3.81971300	-0.56141200
H	-4.24422200	-4.83870300	-0.19496900
C	-3.10131500	-3.02112300	-0.17684200
H	-2.33951800	-3.42960100	0.47886000
C	2.12916800	-0.84846700	0.43564200
C	3.19543700	0.03466100	0.60119600
F	2.96834700	1.29160100	1.02377800
C	4.52453200	-0.29592400	0.37390600
F	5.49426200	0.60480700	0.54693200
C	4.84276400	-1.58447300	-0.02870600
F	6.11054600	-1.93111800	-0.24786400
C	3.82481000	-2.51132000	-0.18413800
F	4.11593900	-3.76077600	-0.55391500
C	2.50861600	-2.13195600	0.05632800
F	159451300	-3.11130000	-0.08612400
C	0.37452500	1.12765900	0.08077000

C	0.04794300	2.31093500	0.73192700
F	-0.15271100	2.32358700	2.07014000
C	-0.17868700	3.52524300	0.09190500
F	-0.53631300	4.60462700	0.79123500
C	-0.04743300	3.59842600	-1.28602800
F	-0.26034600	4.74588800	-1.92621100
C	0.28683400	2.44953900	-1.99312300
F	0.39862200	2.50097500	-3.31981300
C	0.48528700	1.26017200	-1.30316100
F	0.75548600	0.17774700	-2.05016700
H	-4.77752800	-1.42235500	1.18093700
H	-3.89558000	1.89338800	2.56970400
H	-2.69474600	-0.15427400	3.07204400
H	-2.41165400	-1.64558700	2.18481000
H	-2.64285400	1.71579900	-0.24047500
H	-4.13751300	0.88984000	0.23951700
H	-1.47645300	0.45763900	1.38657700
C	0.52456000	-0.33229100	2.43323400
H	1.29540200	0.32173500	2.85252600
H	-0.41459100	-0.01215500	2.91054000
H	0.72019500	-1.34602700	2.80484700

N16_TS

N	-2.28665700	0.05087400	1.08497900
H	-0.86151900	-0.02163700	1.54818600
B	0.46795700	-0.46808200	0.35998500
C	-2.04621500	-0.65191200	-0.22553800
H	-1.83844300	0.18325200	-0.90224000
C	-0.70616400	-1.46072800	-0.24873800
C	-3.06505500	-0.71625100	2.08851800
H	-2.53773700	-1.64645900	2.30542200
H	-4.04238900	-0.97896800	1.66269600
C	-3.30602400	0.06343100	3.38018800
H	-2.35841800	0.30051700	3.87811400
H	-3.86801600	-0.58340100	4.06119500
C	-4.06184900	1.35558500	3.09307800
H	-5.05392200	1.11461700	2.68894300
H	-4.21529900	1.93139800	4.01013500
C	-3.26808600	2.16116000	2.07081200
H	-3.81319000	3.06017300	1.76659400
H	-2.32311300	2.48940700	2.51113600
C	-2.98594800	1.33673500	0.81945100
H	-3.93739900	1.10359600	0.31408000
H	-2.37527600	1.91100700	0.11834300
C	-3.27464800	-1.33406900	-0.81473400
C	-3.86266900	-0.76473100	-1.94971000
H	-3.43051800	0.14102900	-2.36888000
C	-4.97760700	-1.33539400	-2.55758500
H	-5.40830200	-0.87119200	-3.43892400
C	-5.53350100	-2.49809100	-2.03560100
H	-6.40188600	-2.94915100	-2.50451400
C	-4.96460800	-3.08027300	-0.90646000
H	-5.38879600	-3.98861800	-0.49059500
C	-3.84847200	-2.50757800	-0.30452100
H	-3.43042200	-2.98441100	0.57442400
C	2.04678100	-0.95368000	0.42115700
C	2.99689500	-0.05840200	0.93660300
F	2.61957600	1.14107900	1.41691500
C	4.35999900	-0.30099300	1.01558600
F	5.17866900	0.61129400	1.53589400
C	4.86623000	-1.50083600	0.54111300
F	6.16735300	-1.76115500	0.60467300
C	3.98480800	-2.42038100	0.00285600
F	4.44071700	-3.57908600	-0.46965000
C	2.62246900	-2.13456600	-0.05075200
F	1.89514100	-3.11176500	-0.61450600
C	0.49101100	1.02905100	-0.30959300
C	0.12512700	2.22412100	0.30051700
F	-0.31339700	2.23537000	1.57359100
C	0.18606700	3.46708100	-0.31612000
F	-0.21389500	4.56197000	0.33021500
C	0.67854300	3.56228300	-1.60857800
F	0.74589300	4.74088300	-2.21956100
C	1.10944600	2.41006200	-2.24860100
F	1.61802300	2.48213800	-3.47688100
C	1.02121800	1.18905900	-1.59073900
F	1.51673200	0.13291100	-2.25179900
C	-0.49987200	-1.78991700	-1.74353800
H	-1.26304600	-2.50283100	-2.07498700
H	-0.59647600	-0.89718700	-2.36885900
H	0.47672900	-2.22698200	-1.93844300
C	-0.78199100	-2.79375200	0.52352800
H	0.20083000	-3.25451900	0.60383000
H	-1.15901200	-2.68379300	1.54270800
H	-1.42299900	-3.51128900	0.00292800
C	0.18377300	-0.34674300	2.26419200
H	0.43213400	-1.38649200	2.46972900
H	-0.55951200	-0.07071200	3.02537700

H	1.01155100	0.32255200	2.46807200

N16_P

N	-2.39321400	0.06740500	0.89467200
H	-1.54297200	0.50201100	1.27399800
B	0.62161000	-0.44100400	0.64670400
C	-1.88956400	-0.87823300	-0.21628800
H	-1.67993900	-0.15413400	-1.00752800
C	-0.50323200	-1.55941300	0.03535900
C	-3.09494000	-0.53829000	2.07299400
H	-2.45695900	-1.33106400	2.46101400
H	-4.02547000	-0.97317700	1.70076900
C	-3.37419100	0.53296800	3.12146500
H	-2.42521800	0.96422300	3.46449200
H	-3.83883700	0.04889000	3.98490000
C	-4.27462600	1.62715900	2.54969400
H	-5.25109300	1.19598500	2.29346400
H	-4.45153000	2.40585200	3.29569300
C	-3.62991300	2.22841400	1.30215000
H	-4.31470100	2.91849100	0.80048700
H	-2.74091200	2.80361300	1.57518200
C	-3.24864800	1.15157200	0.29395700
H	-4.13072500	0.64263800	-0.10829000
H	-2.68056400	1.57504800	-0.53766800
C	-3.03183500	-1.73767300	-0.73005900
C	-3.52098500	-1.47316100	-2.01418800
H	-3.07995100	-0.66412300	-2.59161900
C	-4.54298700	-2.23473100	-2.57217600
H	-4.89681000	-2.01118200	-3.57327500
C	-5.10348500	-3.28069000	-1.84672200
H	-5.90028000	-3.87914500	-2.27606500
C	-4.62999100	-3.55920000	-0.56776900
H	-5.05557100	-4.37810700	0.00319200
C	-3.60202600	-2.80038600	-0.01575800
H	-3.23978300	-3.05066300	0.97459600
C	2.24562600	-0.79576100	0.44668500
C	3.16010900	0.24107000	0.68151200
F	2.73032500	1.47403200	1.01367900
C	4.54095900	0.11761100	0.62034200
F	5.32714000	1.17159500	0.84606000
C	5.10445100	-1.11514400	0.33364200
F	6.42641400	-1.26194500	0.26676900
C	4.25938600	-2.18966000	0.12985800
F	4.76718800	-3.39664200	-0.12802200
C	2.87984500	-2.01279700	0.20323900
F	2.18798500	-3.15694900	0.02092600
C	0.40283200	1.02995700	-0.13938100
C	-0.13639200	2.17436300	0.43924200
F	-0.58104100	2.14723500	1.72648300
C	-0.31938100	3.39237600	-0.20268400
F	-0.89198500	4.41710700	0.43558800
C	0.09155600	3.52627300	-1.51875400
F	-0.07312400	4.67613000	-2.16784500
C	0.67408100	2.43453700	-2.14705900
F	1.08911100	2.54735400	-3.40767400
C	0.82455200	1.23723500	-1.45521300
F	1.43545200	0.25715300	-2.13669500
C	-0.16287800	-2.07213700	-1.38481600
H	-0.84707300	-2.87502600	-1.68667400
H	-0.23701000	-1.27696800	-2.13210700
H	0.84816200	-2.46640000	-1.43141100
C	-0.59282700	-2.78336700	0.96524600
H	0.40578800	-3.17746300	1.14893700
H	-1.01726400	-2.55380800	1.94749700
H	-1.17657400	-3.59419800	0.51619000
C	0.49597300	-0.31381300	2.28923800
H	0.87965800	-1.23882700	2.73414400
H	-0.50019000	-0.17267100	2.73551900
H	1.11427900	0.50330000	2.67236600

N17_TS

B	0.01043700	-0.35717900	-0.21479600
N	-2.62797300	-1.46734900	-0.76300800
H	-1.35729700	-0.93626000	-1.31043800
C	-2.35848200	-1.08825700	0.64232200
H	-2.62493100	-0.02180200	0.70856400
C	-0.84757300	-1.22273300	0.86204700
H	-0.60611100	-2.28738000	0.74701600
C	-3.90494400	-0.93903200	-1.31364200
H	-4.57811700	-0.68704600	-0.48519700
H	-3.71825800	-0.02790800	-1.88331200
C	-4.49546800	-2.07303200	-2.15356400
H	-4.07997400	-2.05199400	-3.16702600
H	-5.58209200	-2.00467700	-2.23281300
C	-4.00561200	-3.31990300	-1.41689400
H	-4.05752700	-4.23275700	-2.01375900
H	-4.58611000	-3.47136800	-0.49964900
C	-2.57435100	-2.92340600	-1.06842100

H	-1.91984200	-3.06989800	-1.93738800
H	-2.14512900	-3.48397200	-0.23713900
C	-3.19132900	-1.85149000	1.67705700
H	-3.00926900	-2.92948400	1.57521400
H	-4.26203300	-1.68835800	1.50233600
C	-2.80877500	-1.42890000	3.09737500
H	-3.05960900	-0.36926500	3.24158600
H	-3.39655700	-1.99561000	3.82635600
C	-1.31078700	-1.62611900	3.33133800
H	-1.06587200	-2.69344600	3.24308700
H	-1.04220900	-1.32086200	4.34791700
C	-0.49494600	-0.83371400	2.30917700
H	0.57570800	-0.97236900	2.48337800
H	-0.69936800	0.23611200	2.45489900
C	-0.26231700	1.24229300	-0.05790900
C	0.48239800	1.99166300	0.85279900
F	1.43628100	1.40041500	1.58778800
C	0.30244100	3.35158800	1.06392500
F	1.04923500	4.00680000	1.95031000
C	-0.66585300	4.03236400	0.33906700
F	-0.85318000	5.33562700	0.52369500
C	-1.43765100	3.33448300	-0.57546700
F	-2.37550100	3.96658100	-1.28050200
C	-1.22174100	1.97239700	-0.74713100
F	-2.01729100	1.36527800	-1.65068100
C	1.59833000	-0.70953100	-0.29676700
C	2.21552300	-1.81226500	0.29327500
F	1.52116400	-2.71228500	1.00870900
C	3.57947200	-2.07451100	0.20230900
F	4.09792400	-3.15032100	0.79312700
C	4.39986900	-1.21567300	-0.50875500
F	5.70287300	-1.45536200	-0.61044500
C	3.83955100	-0.10396600	-1.12122600
F	4.60825300	0.73389200	-1.81405400
C	2.47647200	0.11882200	-1.00173100
F	2.00926200	1.20645800	-1.63860100
C	-0.36541400	-0.79038600	-2.12057800
H	0.27151800	0.05642300	-2.36573500
H	0.16415000	-1.72833600	-2.28274100
H	-1.18150800	0.72030800	-2.85588400

N17_P

B	0.14132900	-0.39575100	-0.51859200
N	-2.73962300	-1.4075700	-0.68513500
H	-2.15469500	-0.8714700	-1.26353800
C	-2.12718400	-.28485000	0.70009100
H	-2.40307000	-0.26322400	1.00069700
C	-0.59973500	-1.8225000	0.61423600
H	-0.36239100	-2.4272200	0.36568600
C	-4.16761400	-0.9383400	-0.78757100
H	-4.59335300	-0.95235500	0.21666700
H	4.15087600	0.08703800	-1.15494500
C	-4.86182400	-.94643800	-1.69888000
H	-4.70884500	-1.68154700	-2.74969900
H	-5.93558600	-1.98753300	-1.51036200
C	-4.13288300	-3.25212100	-1.37052800
H	-4.27706700	-4.03071700	-2.12099300
H	-4.46262000	-3.64151900	-0.40155900
C	-2.67872800	-2.80421500	-1.29504600
H	-2.24033000	-2.69274500	-2.28880100
H	-2.02270900	-3.43572700	-0.69874600
C	-2.75090500	-2.28263600	1.67736800
H	-2.50815300	-3.30583000	1.36227400
H	-3.84451000	-2.20062200	1.70415300
C	-2.18053100	-2.05926100	3.08155600
H	-2.49649900	-1.07156700	3.44246800
H	-2.60352900	-2.79860800	3.76835900
C	-0.65452800	-2.12539200	3.06222900
H	-0.33360700	-3.14082100	2.79337000
H	-0.25998800	-1.92085500	4.06278300
C	-0.08549200	-1.13498300	2.04700800
H	1.00499000	-1.16778900	2.05710300
H	-0.36211100	-0.11628700	2.35474100
C	-0.35668400	1.15819700	-0.18591800
C	0.25932400	1.95533200	0.78215600
F	1.32632600	1.49996900	1.45551200
C	-0.15332000	3.23685200	1.12201900
F	0.49507400	3.93722600	2.05185400
C	-1.25642000	3.79425800	0.49050400
F	-1.67696800	5.01612400	0.81059700
C	-1.91924300	3.04925600	-0.46892900
F	-2.99834400	3.54371100	-1.08168500
C	-1.45175300	1.77678700	-0.77572900
F	-2.19170600	1.12659500	-1.72208800
C	1.79059900	-0.52271300	-0.40478600
C	2.48671700	-1.65611300	0.00445500
F	1.83780200	-2.74567200	0.46880300
C	3.87303500	-1.77685800	-0.01949900

F	4.46277300	-2.89727600	0.40514800
C	4.64117800	-0.73063900	-0.49883200
F	5.96967000	-0.82526800	-0.53837900
C	4.00242400	0.41630500	-0.94700700
F	4.72308000	1.43095700	-1.42792600
C	2.61773200	0.48600300	-0.90103500
F	2.07823700	1.61738900	-1.38839900
C	-0.09046900	-0.82147000	-2.10704300
H	0.56784300	-0.20316600	-2.72716000
H	0.23285800	-1.86085300	-2.25482700
H	-1.07072200	-0.71889100	-2.59460700

N18_TS

C	-2.99396300	-0.66800500	1.61816300
C	-2.17922000	-0.31806400	0.30526300
H	-2.09213000	0.77432100	0.30422700
C	-0.78104500	-0.90442000	0.54028200
H	-0.78009600	-1.93029000	0.16410600
C	-0.79426600	-1.03680600	2.07948300
H	0.08578800	-1.54153600	2.48495800
C	-1.09237000	0.33652100	2.73806700
H	-0.51929100	1.14070000	2.27668800
H	-0.83018000	0.33236000	3.79853500
C	-2.62371200	0.52833500	2.52573600
H	-2.87162000	1.48579100	2.05574100
H	-3.17846300	0.48002400	3.46722700
C	-2.14184400	-1.78924400	2.30474800
C	-4.48681100	-0.90879600	1.49478800
H	-4.72121800	-1.75469400	0.84041100
H	-5.00363200	-0.02410900	1.10886700
H	-4.91411900	-1.12583900	2.47975600
C	-2.25692400	-3.17508500	1.65924800
H	-2.37025800	-3.15779000	0.57845300
H	-3.13166200	-3.69831200	2.06014100
H	-1.37287700	-3.77735300	1.89507400
C	-2.45128600	-2.00661700	3.79463200
H	-3.50683800	-2.26138100	3.94151100
H	-2.22006700	-1.15248500	4.43071300
H	-1.86225800	-2.85400000	4.16029400
N	-2.58137900	-0.56109300	-1.10329600
H	-1.24208100	-0.29069600	-1.57958000
C	-2.85967800	-1.94197200	-1.54725000
H	-3.76486300	-2.32096200	-1.05146400
H	-2.02004500	-2.57396800	-1.25448300
C	-3.02971300	-2.01540100	-3.0659200
H	-3.26658500	-3.04972100	-3.3263300
H	-2.07562800	-1.77188300	-3.4664800
C	-4.11083600	-1.05945600	-3.56181700
H	-5.08637800	-1.37634100	-3.16932500
H	-4.17860100	-1.08863600	-4.65301700
C	-3.80754400	0.35294100	-3.0686900
H	-2.88778600	0.73058400	-3.5319940
H	-4.60929900	1.04593900	-3.34110200
C	-3.64977900	0.35903800	-1.55308100
H	-3.40027600	1.35814300	-1.1920500
H	-4.59997300	0.05419700	-1.0028900
B	0.34908200	-0.14672400	-032224100
C	1.78940400	-0.90569400	-0.40423500
F	1.13051200	-2.89315400	0.75732400
C	2.06801000	-2.16434600	0.12716900
F	3.50767600	-3.97764100	0.59667100
C	3.31796300	-2.77278900	0.05997000
F	5.56649700	-2.69103300	-0.63946800
C	4.36729100	-2.12326300	-0.56708700
F	5.14064200	-0.22888400	-1.73340700
C	4.14676000	-0.87050500	-1.12181500
F	2.75002900	0.90790500	-1.59798900
C	2.88568900	-0.30287600	-1.02779400
C	0.49255700	1.45133000	-0.04973200
F	2.17261000	1.06271200	1.57044500
C	1.41549600	1.92821100	0.88111500
F	2.48425700	3.66241900	2.08069000
C	1.58656600	3.27214100	1.17849500
F	0.94704200	5.51108100	0.80619700
C	0.80242800	4.21829200	0.53152400
F	-0.91481700	4.68794600	-1.01193500
C	-0.14187500	3.79743900	-0.39063500
F	-1.23169900	2.11131500	-1.55108600
C	-0.27446800	2.43894200	-0.65454900
C	-0.09577000	-0.30095300	-2.25114400
H	-0.76492600	0.12634400	-3.01176000
H	0.78891500	0.32346900	-2.37140200
H	0.13959100	-1.32799800	-2.53706000

N18_P

C	-2.52429000	1.22664700	-1.76963300
C	-2.00032500	0.61811800	-0.41587800
H	-2.02679700	-0.46869700	-0.53182800

C	-0.53808700	1.07459600	-0.27623200
H	-0.53206400	2.02346700	0.26872000
C	-0.26774100	1.46823400	-1.75112600
H	0.70049800	1.95145000	-1.88914400
C	-0.49449400	0.25385900	-2.68440000
H	-0.03428000	-0.64806100	-2.28458500
H	-0.05365000	0.42145400	-3.66967700
C	-2.04537200	0.14214600	-2.76908800
H	-2.41996400	-0.85098100	-2.49878500
H	-2.42196200	0.36229300	-3.77195900
C	-1.49502900	2.36164900	-2.09092500
C	-3.99506000	1.57759400	-1.90568700
H	-4.32419200	2.31223000	-1.16123300
H	-4.63620700	0.69321500	-1.82975900
H	-4.17444800	2.01655200	-2.89213600
C	-1.64612200	3.63782400	-1.25683900
H	-1.89141000	3.46677700	-0.21210100
H	-2.43812200	4.26911700	-1.67465200
H	-0.71379000	4.21124600	-1.28202600
C	-1.50995000	2.83130100	-3.55429600
H	-2.48635400	3.24908700	-3.82456300
H	-1.26459400	2.05491500	-4.27823100
H	-0.77437300	3.63277100	-3.67556100
N	-2.76561300	0.73089500	0.90618300
H	-2.12554100	0.20455900	1.51839700
C	-2.93275300	2.06200600	1.56969800
H	-3.64747800	2.63843200	0.97506700
H	-1.96279300	2.55727000	1.55167100
C	-3.40587200	1.86701300	3.00724800
H	-3.53915100	2.85446900	3.45740400
H	-2.60685100	1.36671000	3.56958200
C	-4.69241600	1.04602600	3.08277500
H	-5.51421000	1.60782100	2.62053800
H	-4.96852800	0.87132600	4.12568500
C	-4.50942800	-0.28061700	2.34686000
H	-3.76345800	-0.90414800	2.85603700
H	-5.44076000	-0.85283400	2.32309000
C	-4.05396100	-0.03885700	0.91499700
H	-3.86797600	-0.97382800	0.38419800
H	-4.78627900	0.55227700	0.36152600
B	0.45830100	0.08975800	0.61828400
C	2.03269400	0.60236600	0.53310000
F	1.53664500	2.89109000	0.03285900
C	2.43383600	1.91673100	0.30835800
F	4.04937500	3.63904300	0.12791800
C	3.75277500	2.35826900	0.36564400
F	6.02640600	1.86869700	0.76991100
C	4.75771500	1.46746500	0.69744100
F	5.36561400	-0.72109300	1.31741300
C	4.41690200	0.15088200	0.97078300
F	2.84453200	-1.51568400	1.23390700
C	3.08703800	-0.23599900	0.90128900
C	0.27263000	-1.49966300	0.15095500
F	2.13009500	-1.50827000	-1.32473600
C	1.11859500	-2.16499200	-0.73933600
F	1.83322900	-4.05399100	-1.97126500
C	0.97700000	-3.49288600	-1.11811800
F	-0.23737300	-5.51487900	-0.97286500
C	-0.07686900	-4.24275300	-0.61507400
F	-2.02002000	-4.31137800	0.72215800
C	-0.97206100	-3.63331600	0.24573900
F	-1.74739500	-1.79777900	1.41094400
C	-0.77107600	-2.30312500	0.59551200
C	0.14905600	0.25256800	2.24199700
H	-0.79855200	-0.12833800	2.65113600
H	0.91549400	-0.28498000	2.81210300
H	0.23160300	1.30907200	2.53560800

N19_TS

B	0.53224500	-0.16183900	0.17664100
C	-0.05112400	-1.00998800	1.43475500
C	0.87575400	-1.66713500	2.25791500
H	1.92838700	-1.64260100	1.99932100
C	0.52088000	-2.33890900	3.42655800
H	1.28803800	-2.82463200	4.02123400
C	-0.80591400	-2.37247000	3.82496500
H	-1.10325200	-2.88134300	4.73626500
C	-1.75556000	-1.73963600	3.02922800
H	-2.80222700	-1.76210900	3.32538400
C	-1.39941000	-1.08323700	1.85229500
C	-2.51785300	-0.41818100	1.09231700
H	-2.34825000	0.65823200	1.11437800
H	-3.46037600	-0.59420700	1.61901700
N	-2.63812600	-0.80538400	-0.33566500
C	-3.01206400	-2.27733100	-0.54003300
C	-3.20878400	-2.54011100	-2.04410300
H	-2.23733100	-2.44767100	-2.54639600
H	-3.51800500	-3.58484500	-2.15149300

C	-4.20353800	-1.60935200	-2.71417700
H	-5.20492000	-1.73935200	-2.28810100
H	-4.28650800	-1.84761400	-3.77881300
C	-3.71854200	-0.18182700	-2.53578100
H	-4.42509500	0.53268200	-2.97148400
H	-2.77611200	-0.05670000	-3.08151900
C	-3.51797300	0.22076600	-1.06447300
C	-2.83049500	1.59934800	-1.06942700
H	-1.78510000	1.54910500	-1.37302000
H	-2.88515000	2.10880500	-0.10431000
H	-3.35709400	2.23084000	-1.79041500
C	-4.90073000	0.43056800	-0.41686600
H	-4.83399000	0.63398200	0.65358700
H	-5.58759800	-0.40060400	-0.57115200
H	-5.35379900	1.31166900	-0.88065900
C	-4.26794300	-2.70708500	0.23602900
H	-4.15104200	-2.54416800	1.31021600
H	-4.39465800	-3.78432200	0.09295800
H	-5.18705700	-2.22420700	-0.09237400
C	-1.88076000	-3.22334300	-0.08422200
H	-0.88958300	-2.88547000	-0.38102100
H	-2.06193100	-4.19465000	-0.55522900
H	-1.87073100	-3.37735700	0.99401100
C	2.08092000	-0.59134800	-0.13354400
C	2.38748100	-1.89282100	-0.53316900
F	1.40541500	-2.80151800	-0.66538400
C	3.67147600	-2.34464100	-0.79489800
F	3.88867800	-3.60270500	-1.17565100
C	4.73933900	-1.47035100	-0.64368000
F	5.98100900	-1.87856600	-0.88512500
C	4.49460600	-0.17182300	-0.23010400
F	5.50967300	0.67485100	-0.06775200
C	3.18790400	0.23892100	0.01976600
F	3.05692800	1.50415800	0.43956600
C	0.31427800	1.45448800	0.21284400
C	-0.26752600	2.16072900	1.26519400
F	-0.68204000	1.54874000	2.38265500
C	-0.48495200	3.53689900	1.24984500
F	-1.06964300	4.13474700	2.28717600
C	-0.08153400	4.28486200	0.15839500
F	-0.27924700	5.59809100	0.12566200
C	0.55367600	3.64270700	-0.89617200
F	0.98909700	4.34853700	-1.93778200
C	0.75132700	2.27274300	-0.83549600

F	1.43987700	1.74976200	-1.86443200
H	-1.25078800	-0.64358400	-0.78966200
C	-0.24089200	-0.64070700	-1.63458700
H	-0.18708300	-1.69129000	-1.91686800
H	0.71264700	-0.19481900	-1.89686200
H	-0.91573500	-0.11216300	-2.31717200

N19_P

B	-0.57351600	-0.50205800	0.36924100
C	0.03491800	-1.50711400	-0.80853000
C	-0.73947900	-2.62166200	-1.17806900
H	-1.68288900	-2.78810000	-0.66752300
C	-0.38477800	-3.51732400	-2.18324800
H	-1.03884900	-4.35253400	-2.41557700
C	0.77860100	-3.31390900	-2.91477000
H	1.04632100	-3.96465200	-3.74060400
C	1.59902600	-2.25448200	-2.55420200
H	2.52024900	-2.08413600	-3.10832100
C	1.27196300	-1.40428300	-1.49154200
C	2.32158500	-0.37742900	-1.15296600
H	1.90054600	0.62360400	-1.13433300
H	3.12404600	-0.39627600	-1.88981900
N	2.98524600	-0.55504900	0.21141400
C	3.72732100	-1.91751700	0.40872700
C	4.47510200	-1.89106600	1.75196500
H	3.73465400	-1.93325000	2.56265400
H	5.04603700	-2.82299100	1.80414800
C	5.36510800	-0.68154200	1.96378100
H	6.14399100	-0.61775300	1.19547300
H	5.87836700	-0.75862000	2.92606100
C	4.47330000	0.54475600	1.94520200
H	5.03935000	1.46111000	2.13822700
H	3.73272700	0.45793700	2.75180100
C	3.73177800	0.75635700	0.61836400
C	2.69704500	1.85927700	0.86958600
H	1.94725500	1.54843300	1.60184200
H	2.18424400	2.20561000	-0.02917500
H	3.23301400	2.71511200	1.28936100
C	4.69486100	1.20760000	-0.48091200
H	4.17462100	1.45579600	-1.40739200
H	5.47746500	0.48135300	-0.69959200
H	5.18208100	2.12151900	-0.13156300
C	4.68914000	-2.20598900	-0.74143600
H	4.17253900	-2.26480400	-1.69966000

H	5.12512400	-3.19125100	-0.55499600
H	5.51209900	-1.49484500	-0.81887800
C	2.70349300	-3.06035900	0.51797600
H	1.87418400	-2.79449300	1.17755200
H	3.22679100	-3.90967100	0.96744200
H	2.29658000	-3.37776600	-0.43939800
C	-2.22373100	-0.72188100	0.44225100
C	-2.94430400	-1.29447200	1.48884000
F	-2.33530500	-1.80866200	2.57159500
C	-4.33272100	-1.40862800	1.50720100
F	-4.95010500	-1.96149700	2.55417000
C	-5.07482900	-0.95686400	0.43080900
F	-6.40288500	-1.06109800	0.43020600
C	-4.41019400	-0.40605900	-0.65488700
F	-5.10431000	0.01741400	-1.71303300
C	-3.02616300	-0.31195100	-0.62625800
F	-2.45946400	0.19827300	-1.73070600
C	-0.41475800	1.12551600	0.01585500
C	-0.22230800	1.67677500	-1.25316900
F	-0.04787200	0.89788300	-2.33194900
C	-0.12718300	3.04119200	-1.51357200
F	0.08556400	3.48047000	-2.75576700
C	-0.26587400	3.94867800	-0.47546100
F	-0.17953200	5.25798100	-0.70158900
C	-0.49350200	3.46443900	0.80312700
F	-0.63008400	4.31616000	1.82142600
C	-0.57612200	2.09210700	1.01064200
F	-0.78233400	1.73312300	2.28866200
H	2.18850900	-0.61352600	0.86222700
C	0.08467300	-0.86998500	1.84290400
H	0.12258500	-1.95488800	1.99127400
H	-0.50304200	-0.47002600	2.66703900
H	1.09738500	-0.48077300	2.04924000

N20_TS

B	-0.85467300	-0.12659800	0.16942100
C	0.07052300	-0.66033200	1.39337700
C	-0.42635400	-1.64250500	2.26046700
H	-1.42102700	-2.04414000	2.09616900
C	0.28864300	-2.11247900	3.36210300
H	-0.15340100	-2.86364900	4.00930700
C	1.55953500	-1.62322600	3.62276500
H	2.13360800	-1.98496200	4.46982400
C	2.08853700	-0.65034800	2.77817500
H	3.08258300	-0.25159900	2.96661000
C	1.35814700	-0.16088600	1.69871000
C	1.97013800	0.95874500	0.89916500
H	2.87613800	1.31814200	1.39918800
H	1.27875000	1.79588700	0.82806800
N	2.30308300	0.52127400	-0.48934700
C	2.56892000	1.69802800	-1.42493800
C	3.61297800	2.61742100	-0.78125500
H	3.90941100	3.36042000	-1.52816400
H	3.13651000	3.17646600	0.02988300
C	4.84545400	1.85860500	-0.26058200
C	4.74045500	0.34674000	-0.29558100
C	5.92168500	-0.38607500	-0.22635600
C	5.89623300	-1.77331300	-0.25448200
H	6.81941400	-2.34133800	-0.20342300
C	4.67765800	-2.42456700	-0.35089000
H	4.65401800	-3.50946100	-0.37040900
C	3.46454500	-1.72914200	-0.42413400
C	3.51033700	-0.32615000	-0.41280100
C	2.19144400	-2.54429300	-0.53332800
H	1.34966800	-1.89176300	-0.32864100
C	2.11857100	-3.65510100	0.51953500
H	1.12152800	-4.10207700	0.50615100
H	2.29707000	-3.25166900	1.51961200
H	2.84492600	-4.45167400	0.32815700
C	2.03567800	-3.11569200	-1.94676300
H	1.08226800	-3.64579600	-2.03495500
H	2.84303200	-3.82165800	-2.16954300
H	2.06408700	-2.32625100	-2.70535100
C	1.29900000	2.51859100	-1.68080700
H	0.84676900	2.90153800	-0.76339000
H	0.54053000	1.96361000	-2.23603400
H	1.58141800	3.38471300	-2.28608500
C	3.08727400	1.16923500	-2.77102000
H	2.45595800	0.36857400	-3.16609900
H	4.10572500	0.78415300	-2.70576300
H	3.07763200	1.98914500	-3.49409000
C	-2.16566600	-1.08482100	-0.07048100
C	-3.49309500	-0.66022400	0.01286200
F	-3.81474800	0.60274300	0.32103100
C	-4.58801700	-1.49614800	-0.19018600
F	-5.82573100	-1.01337700	-0.09953300
C	-4.39148900	-2.83535100	-0.47624100
F	-5.42618600	-3.64624500	-0.66831400

C	-3.09371100	-3.32057300	-0.54577200
F	-2.87633700	-4.61069100	-0.79788200
C	-2.03409500	-2.45204400	-0.33432200
F	-0.81375300	-3.02031100	-0.38224200
C	-1.24681100	1.46581600	0.21408100
C	-1.92307400	2.10275000	-0.83195500
F	-2.35850900	1.39782700	-1.89085800
C	-2.22728100	3.45364100	-0.86946700
F	-2.85648700	3.97987300	-1.91870200
C	-1.89170500	4.25640900	0.21216500
F	-2.17345500	5.55452000	0.20255600
C	-1.27867700	3.67385200	1.30725900
F	-0.97311300	4.41466300	2.37158400
C	-0.99442300	2.30929700	1.29712200
F	-0.41352500	1.85962400	2.41700500
H	6.86667200	0.14607900	-0.15307100
H	5.73366900	2.13982100	-0.83408400
H	5.05349700	2.16703100	0.77113400
H	1.01756600	-0.00644200	-0.90117600
C	-0.04360500	-0.44570200	-1.62070000
H	-0.89707200	0.05858900	-2.06184100
H	0.74526300	-0.20554200	-2.34934700
H	-0.15434500	-1.52065100	-1.70047400

N20_P

B	-1.04404500	0.00214300	-0.34165900
C	-0.09319700	-0.24269300	0.99556800
C	-0.55987500	-1.04047200	2.05540600
H	-1.52906800	-1.51840400	1.95480100
C	0.14463000	-1.22882800	3.23844500
H	-0.27284500	-1.85791300	4.01938300
C	1.37267600	-0.60101000	3.42904200
H	1.92836400	-0.72666900	4.35252700
C	1.86618200	0.20470800	2.41397700
H	2.82284100	0.70808900	2.54643800
C	1.15782300	0.37828200	1.21686100
C	1.81438600	1.25253400	0.17894300
H	2.44880300	2.00126200	0.65572300
H	1.09378200	1.74340200	-0.47205000
N	2.75910200	0.45372800	-0.74610100
C	3.44707000	1.30180900	-1.84755600
C	4.23222300	2.41035500	-1.14075300
H	4.91139200	2.83041600	-1.88801200
H	3.54405000	3.21648700	-0.87097800
C	5.02420200	1.94401100	0.10063600
C	4.89256400	0.47428500	0.42978600
C	5.89702300	-0.16970800	1.14863700
C	5.78132800	-1.51758400	1.46028000
H	6.56716800	-2.01508600	2.01889400
C	4.66082300	-2.23243000	1.05656200
H	4.57834000	-3.28082200	1.31824700
C	3.62476700	-1.63300000	0.33431700
C	3.78432800	-0.27520800	0.03335600
C	2.40220400	-2.43865600	-0.07850600
H	1.53049600	-1.77892800	-0.06438900
C	2.08072200	-3.55935200	0.91401600
H	1.10070900	-3.97563800	0.67791000
H	2.05015800	-3.17371500	1.93664600
H	2.81265300	-4.37212100	0.85876800
C	2.55006600	-3.00481200	-1.49678900
H	1.64452800	-3.55602100	-1.76418400
H	3.40758200	-3.68277500	-1.55705100
H	2.69409500	-2.22054300	-2.24906200
C	2.42246700	1.91256100	-2.79951300
H	1.66363800	2.51580900	-2.30100700
H	1.92398000	1.14788700	-3.40001100
H	2.97065900	2.56754100	-3.48258300
C	4.36864000	0.37868100	-2.65104600
H	3.82339500	-0.49460100	-3.02177700
H	5.23306600	0.03426400	-2.08290300
H	4.72625100	0.93598000	-3.52032200
C	-2.23945200	-1.14180600	-0.41514500
C	-3.60721900	-0.92617800	-0.56268700
F	-4.12052000	0.31264500	-0.58955300
C	-4.54356500	-1.94682800	-0.70827200
F	-5.83978900	-1.65997500	-0.84116500
C	-4.12760100	-3.26741400	-0.72725200
F	-5.00888300	-4.25703200	-0.86968000
C	-2.77408400	-3.54258100	-0.60227100
F	-2.34095500	-4.80629100	-0.62897300
C	-1.88356100	-2.48893700	-0.46116200
F	-0.58107200	-2.84464500	-0.36249800
C	-1.65021600	1.52605600	-0.09874600
C	-1.33363600	2.66982600	-0.81788500
F	-0.49879600	2.61618500	-1.88674700
C	-1.79264200	3.94808400	-0.51135900
F	-1.42342500	4.99767600	-1.25260400
C	-2.63326400	4.12348800	0.57231100

F	-3.09083000	5.33437300	0.88849100
C	-2.98909700	3.01552800	1.33194000
F	-3.80135300	3.16772100	2.37864000
C	-2.49306400	1.76829500	0.98854300
F	-2.88731800	0.74538200	1.76074700
H	6.77235000	0.39521000	1.45588700
H	6.08674400	2.16981600	-0.02046300
H	4.69821600	2.52218100	0.97369100
H	2.15152100	-0.22889100	-1.21999300
C	-0.27288200	-0.18580700	-1.78232600
H	-0.98611500	-0.09667300	-2.60970200
H	0.49778700	0.54980500	-2.00602700
H	0.16385200	-1.19171000	-1.86145400

N21_TS

B	-0.71972000	-0.10106700	-0.04396400
C	-0.05201800	-0.56869700	1.37722600
C	-0.76372200	-1.43098300	2.22391400
H	-1.73631500	-1.79612700	1.91494100
C	-0.29746200	-1.82485300	3.47491000
H	-0.89640300	-2.49357800	4.08508300
C	0.92729500	-1.36190200	3.93463100
H	1.31114800	-1.66843700	4.90226500
C	1.65546500	-0.49187100	3.13194500
H	2.61754200	-0.11881200	3.47378500
C	1.17445100	-0.08629300	1.88773200
C	2.01650500	0.88744200	1.10835400
H	2.97284900	1.05131100	1.61871200
H	1.50254800	1.85281900	1.03063700
N	2.25414700	0.43223800	-0.28395400
C	2.73939600	1.53650000	-1.14097100
C	4.19040300	1.87869200	-0.84888600
H	4.29920800	2.14614800	0.20980700
H	4.48750900	2.76053700	-1.42460000
C	5.10381500	0.70083400	-1.18327200
H	5.24573800	0.68699200	-2.27373900
C	4.45685700	-0.62254100	-0.80341400
C	5.19427600	-1.80647400	-0.92383400
H	6.21687200	-1.75172100	-1.28560800
C	4.65732000	-3.04430800	-0.60865100
H	5.26231300	-3.94028700	-0.71934900
C	3.33860700	-3.14981800	-0.16114800
C	2.58671200	-1.98514500	-0.05792600
H	1.55419300	-2.05910700	0.26368100
C	3.12770900	-0.73268900	-0.37094800
C	6.47428600	0.89481700	-0.52717400
H	6.88010000	1.87617700	-0.79072300
H	7.19848000	0.14091400	-0.84180700
H	6.38270800	0.84225600	0.56216500
C	2.73349400	-4.48010700	0.20357800
H	1.64299200	-4.44327100	0.14605700
H	3.00221300	-4.75981500	1.22739100
H	3.08981400	-5.27205400	-0.46000900
C	-0.89749100	1.52047000	-0.07213800
C	-1.52858000	2.11899500	1.02199100
F	-2.05224400	1.35051200	1.98250000
C	-1.68874600	3.48776000	1.16906100
F	-2.29724500	3.99312500	2.23963200
C	-1.21392600	4.33987000	0.17930800
F	-1.34853700	5.65627700	0.30450500
C	-0.60738300	3.79723000	-0.93984200
F	-0.14467100	4.59223100	-1.90551200
C	-0.48260800	2.41471100	-1.05079200
F	0.10641700	1.98866600	-2.18637800
C	-2.09150100	-0.93053900	-0.36868000
C	-3.33691900	-0.35609400	-0.61301000
F	-3.51298700	0.97102700	-0.59174700
C	-4.48185600	-1.09570300	-0.90109200
F	-5.63992300	-0.47902900	-1.12830600
C	-4.41327000	-2.47678800	-0.95700000
F	-5.49569500	-3.19688100	-1.23268400
C	-3.19626000	-3.10171100	-0.72055300
F	-3.11002100	-4.43002000	-0.76855800
C	-2.08291500	-2.32711200	-0.43563800
F	-0.94258300	-3.00839200	-0.20833800
H	0.95659100	-0.03209600	-0.81115800
H	2.08756600	2.39669900	-0.97999200
H	2.62168200	1.22321400	-2.18537400
C	0.17498100	-0.65102800	-1.67198800
H	-0.64029500	-0.25414000	-2.26969000
H	1.06600900	-0.42992000	-2.27991400
H	0.13042200	-1.73166100	-1.58524500

N21_P

B	-0.88131100	-0.43072600	-0.21411200
C	-0.19890100	-1.26596500	1.05995200
C	-0.85960200	-2.44609600	1.44599300
H	-1.81090600	-2.68267800	0.98223900

C	-0.36809300	-3.33922500	2.39335000
H	-0.94059500	-4.22832700	2.64026600
C	0.84658000	-3.09164200	3.01914700
H	1.24648800	-3.77433700	3.76146100
C	1.54643400	-1.94790300	2.66217700
H	2.50912400	-1.74341500	3.12543200
C	1.04380600	-1.05266500	1.71113400
C	1.93471300	0.12624600	1.41609100
H	2.88099900	0.04492000	1.95344800
H	1.46978600	1.07951700	1.66274700
N	2.29292300	0.19677000	-0.06246100
C	2.65088400	1.58220900	-0.51057000
C	4.01008100	1.96079200	0.03922600
H	3.99164600	1.91091600	1.13499600
H	4.21831800	3.00286100	-0.21839500
C	5.11214600	1.05463300	-0.51339800
H	5.32292400	1.37980300	-1.54225600
C	4.65419800	-0.39377600	-0.60010900
C	5.56419300	-1.40532100	-0.92121200
H	6.60405400	-1.14356400	-1.08900000
C	5.17618900	-2.73317200	-1.03744700
H	5.91846000	-3.48796500	-1.28121600
C	3.84684000	-3.11384600	-0.84800600
C	2.92080700	-2.11962500	-0.54158900
H	1.87766700	-2.37782900	-0.38862000
C	3.32742300	-0.79466700	-0.42419800
C	6.38969500	1.22243400	0.31334300
H	6.64963600	2.28224500	0.38763800
H	7.24188600	0.70427200	-0.12965700
H	6.24275900	0.83253500	1.32514400
C	3.39943800	-4.54473900	-0.98363300
H	2.69498500	-4.80737100	-0.19033500
H	4.24769400	-5.23046300	-0.93615000
H	2.89198100	-4.70135300	-1.94046300
C	-0.66312200	1.22359400	-0.17415200
C	-0.57647900	1.97236400	1.00134200
F	-0.63512400	1.36608700	2.19611900
C	-0.39071800	3.34847200	1.04896800
F	-0.28587500	3.98429200	2.21664000
C	-0.32401600	4.06782500	-0.13711300
F	-0.15295900	5.38709900	-0.11867400
C	-0.44895500	3.38812400	-1.33736700
F	-0.40309900	4.06052100	-2.48837600
C	-0.62420000	2.00530200	-1.33119500
F	-0.75051200	1.45640000	-2.54827000
C	-2.52516500	-0.69245700	-0.23821500
C	-3.44950300	0.09623500	0.44898900
F	-3.05043500	1.10145300	1.24466600
C	-4.82807500	-0.07877800	0.39251800
F	-5.64172400	0.73656400	1.06578300
C	-5.35760200	-1.11171100	-0.36211700
F	-6.67410300	-1.30034500	-0.42793700
C	-4.48919500	-1.95590300	-1.03380700
F	-4.97072000	-2.97910700	-1.74257000
C	-3.11935000	-1.73639200	-0.95184600
F	-2.36844200	-2.65259100	-1.58858600
H	1.42668000	-0.06490900	-0.56100700
H	1.85564400	2.24238500	-0.16222700
H	2.64645100	1.56718200	-1.60521900
C	-0.23064400	-1.03824100	-1.62387900
H	-0.91602100	-0.89929300	-2.46175800
H	0.70471300	-0.57664600	-1.98732700
H	-0.04017700	-2.11223700	-1.54535400

P1_TS

P	-1.12312300	0.03715700	-0.33618200
C	-0.72385200	-1.26102900	0.85179500
C	0.58321100	-1.75422500	0.69695900
B	1.60015400	-1.12658300	-0.37906500
C	-1.61058000	-1.77555700	1.80269900
C	-1.18680300	-2.80362300	2.63485700
C	0.10881600	-3.30832900	2.50481900
C	0.97909100	-2.79034400	1.55100700
O	2.89809800	-1.78068000	-0.42867600
O	1.86274800	0.30908700	-0.17568000
C	3.19681900	0.46686100	-0.37639500
C	3.92352200	1.63964100	-0.41965700
C	5.30744100	1.53633800	-0.63048000
C	5.91877900	0.29830900	-0.78791900
C	5.17421500	-0.89069900	-0.74001600
C	3.81429400	-0.78330000	-0.53080200
H	3.43467800	2.59892200	-0.28576800
H	5.90665500	2.44018800	-0.66914400
H	6.99027800	0.24611600	-0.95068200
H	5.63879800	-1.86310200	-0.85871300
C	-1.01809000	1.68496100	0.41838400
C	-2.85647900	-0.19086900	-0.83906500
C	-3.67896900	0.90532000	-1.11847900

C	-4.97763100	0.70365000	-1.57601600
C	-5.46187100	-0.58823200	-1.76199000
C	-4.64378100	-1.68293200	-1.49289900
C	-3.34478300	-1.48873000	-1.03561100
H	-3.30634700	1.91463300	-0.97114800
H	-5.61172600	1.55865800	-1.78670100
H	-6.47546800	-0.74239000	-2.11760900
H	-5.01790800	-2.69127300	-1.63664400
H	-2.71245000	-2.34496700	-0.81736700
C	-0.12581600	2.62310800	-0.10417900
C	-0.05343200	3.89479800	0.45771800
C	-0.86500900	4.22793900	1.53798600
C	-1.75465000	3.29135800	2.06297800
C	-1.83286200	2.02083700	1.50486200
H	0.52431900	2.34345500	-0.92637200
H	0.64264500	4.62265700	0.05393500
H	-0.80218600	5.21860300	1.97691600
H	-2.38252500	3.55036300	2.90907200
H	-2.52739600	1.28995600	1.91112000
H	-2.62379700	-1.38766100	1.87824000
H	-1.86152500	-3.21318200	3.37976900
H	0.43696800	-4.11428900	3.15465500
H	1.98646300	-3.18442700	1.45358100
H	0.04847600	-0.49286000	-1.35442400
C	1.01452200	-1.36698200	-2.12026800
H	1.87744900	-0.91915400	-2.62163300
H	0.15808400	-1.17650700	-2.78353300
H	1.11920500	-2.45504500	-2.10637700

P1_P

P	-1.60336300	-0.02213100	-0.55703100
C	-0.57593600	-1.21449900	0.30237800
C	0.79910300	-1.32300900	0.01207700
B	1.74642600	-0.37388200	-0.97983400
C	-1.23272500	-2.00956800	1.26043900
C	-0.51064500	-2.95207100	1.97142600
C	0.85115700	-3.10065000	1.70738000
C	1.47586500	-2.31063400	0.75194100
O	2.94250400	-1.19020600	-1.36158500
O	2.31752200	0.71656800	-0.09166300
C	3.64149400	0.47601400	0.01866800
C	4.57700400	1.18218700	0.74855300
C	5.91333400	0.74658000	0.70682500
C	6.27807200	-0.36107000	-0.04471400
C	5.32301300	-1.07954600	-0.78529600
C	4.01117000	-0.64812100	-0.74419200
H	4.27907400	2.04757300	1.33150200
H	6.66669500	1.28763000	1.27074800
H	7.31509400	-0.68149000	-0.06393400
H	5.59421600	-1.94675600	-1.37777800
C	-1.38166300	1.68146700	-0.01781400
C	-3.35015000	-0.44941600	-0.39269400
C	-4.12336000	0.04585200	0.66162300
C	-5.44633700	-0.36318600	0.79509500
C	-5.99605500	-1.25968600	-0.11908300
C	-5.22666600	-1.75166800	-1.17034200
C	-3.90296700	-1.34903700	-1.30918100
H	-3.69424500	0.74911800	1.36990400
H	-6.04829200	0.01882200	1.61264000
H	-7.02877600	-1.57495400	-0.01206500
H	-5.65578800	-2.44782100	-1.88278100
H	-3.30128100	-1.73690400	-2.12706800
C	-2.32924900	2.64866300	-0.37983900
C	-2.13758700	3.96842200	0.00497300
C	-1.00446700	4.32096700	0.73987600
C	-0.06481600	3.35812600	1.08979000
C	-0.24664200	2.02818000	0.71752300
H	-3.21100100	2.37147700	-0.95143800
H	-2.86705100	4.72254900	-0.27078400
H	-0.85476600	5.35508900	1.03347400
H	0.82351900	3.63420500	1.64710000
H	0.51245000	1.29033800	0.96025100
H	-2.29775500	-1.90098700	1.44171000
H	-1.00476600	-3.57049600	2.71323700
H	1.42642700	-3.84424100	2.25146700
H	2.53301500	-2.45222700	0.55151800
H	-1.37572700	-0.10075700	-1.93282200
C	1.08151400	0.28162900	\ -2.30976200
H	0.55368200	-0.43836400	-2.94972600
H	1.91586900	0.67263100	-2.90173200
H	0.42273700	1.14049100	-2.12671200

P2_TS

C	-1.35470700	-1.69685000	1.94466200
C	-0.57654900	-1.20619300	0.89383300
C	0.73239300	-1.65694500	0.64710200
C	1.24679000	-2.62041900	1.52242200
C	0.48806900	-3.11117200	2.58084500

C	-0.81310200	-2.65238300	2.79569100
H	-2.37171600	-1.34165900	2.09162300
H	2.25916400	-2.97837400	1.35406200
H	0.90709700	-3.86017900	3.24686100
H	-1.40112500	-3.04169600	3.62078500
B	1.61372300	-1.02843200	-0.55572800
P	-1.15702700	-0.01194000	-0.33428100
C	-2.91166800	-0.35144600	-0.66657800
C	-3.93507500	0.11388000	0.16771800
C	-3.22953700	-1.14745100	-1.77146600
C	-5.25773000	-0.22478900	-0.09795900
H	-3.69454500	0.74829700	1.01677800
C	-4.55441300	-1.48474700	-2.03305400
H	-2.43824800	-1.50111200	-2.42742100
C	-5.56715900	-1.02474500	-1.19658500
H	-6.04783400	0.13671600	0.55213600
H	-4.79539000	-2.10129800	-2.89265600
H	-6.60044400	-1.28510300	-1.40253400
C	-1.06703400	1.66873100	0.34525100
C	-1.87988500	2.68196100	-0.17269900
C	-0.13408600	1.96500800	1.34345400
C	-1.76744300	3.98074800	0.31357700
H	-2.60444700	2.45763400	-0.95092200
C	-0.02569300	3.26650500	1.82175600
H	0.51356300	1.17992000	1.72021600
C	-0.84110500	4.27377300	1.31102800
H	-2.40309200	4.76265200	-0.08902800
H	0.70049300	3.49315800	2.59588100
H	-0.75383800	5.28707300	1.68985600
O	2.96723100	-1.50549500	-0.67019400
O	1.67975400	0.41705700	-0.45739000
C	2.99297300	0.81886800	-0.83282900
C	3.32088700	2.12015800	-0.11434500
H	4.35829500	2.41963200	-0.29806400
H	2.66156400	2.91388500	-0.47816500
H	3.16686900	2.02054100	0.96218000
C	3.03534300	1.03168500	-2.34821800
H	2.87941200	0.08753300	-2.87736100
H	2.23333400	1.72138200	-2.62585900
H	3.99046900	1.45792500	-2.67053700
C	3.84017300	-0.41524200	-0.38848600
C	5.13714800	-0.60698800	-1.16218200
H	5.79397000	0.26236600	-1.04926100
H	5.66401400	-1.48592300	-0.78081300
H	4.93872900	-0.76539500	-2.22369000
C	4.12262300	-0.40117000	1.11629000
H	3.19679000	-0.23589600	1.67672000
H	4.52679200	-1.37580400	1.40390000
H	4.84822000	0.37056500	1.39183400
H	-0.09061200	-0.57506100	-1.41445800
C	0.74331400	-1.55801100	-2.15523000
H	1.66958000	-2.10196200	-2.36629500
H	0.58836400	-0.90441000	-3.02416300
H	-0.05168300	-2.31324200	-2.12207800

P3_TS

C	-1.38995100	0.70567000	0.24610600
C	-0.12493900	0.26761700	0.10901200
C	-1.90934800	1.49406600	1.39252200
C	-2.00091400	0.86738900	2.64114000
C	-2.36361800	2.80847100	1.24805100
C	-2.54068400	1.54535200	3.72852600
H	-1.65138600	-0.15767700	2.73764000
C	-2.90200600	3.48373300	2.34051600
H	-2.28567600	3.30188700	0.28438200
C	-2.99529700	2.85394900	3.57902400
H	-2.60959100	1.05109900	4.69231500
H	-3.24618900	4.50633000	2.22344200
H	-3.41807700	3.38288000	4.42718500
C	0.86931600	0.76477400	1.11306100
C	1.33351700	2.07590200	1.03909200
C	1.44675900	-0.05896600	2.07012500
C	2.35145000	2.54463500	1.85496400
C	2.45780900	0.38829600	2.91160700
C	2.91538500	1.69319800	2.79799000
F	1.02783800	-1.31889400	2.20960600
F	2.98214700	-0.42194800	3.82592800
F	3.88339000	2.13246600	3.59407100
F	2.78772000	3.79632500	1.74461300
F	0.82034700	2.90837700	0.12706500
B	0.55631500	-0.66527900	-1.06811000
P	-2.51587200	0.02531300	-0.99148700
C	-3.26716000	1.44258100	-1.85065700
C	-2.41372100	2.46561500	-2.28640400
C	-4.62731600	1.49155300	-2.16386100
C	-2.92721500	3.53337700	-3.01403000
H	-1.35290800	2.43114600	-2.04443600
C	-5.13332900	2.56442400	-2.89306500

H	-5.29103200	0.69803100	-1.83362100
C	-4.28699100	3.58484900	-3.31644200
H	-2.26403200	4.32599100	-3.34456000
H	-6.19194400	2.60169000	-3.12872100
H	-4.68526500	4.42005500	-3.88344700
C	-3.83620400	-0.85061200	-0.10434600
C	-4.70449100	-0.16474900	0.75492300
C	-3.94288100	-2.23680700	-0.24258300
C	-5.67091900	-0.86883600	1.46466400
H	-4.61880100	0.91283400	0.87088500
C	-4.91117900	-2.93592200	0.47170400
H	-3.26124800	-2.76885300	-0.90073700
C	-5.77321700	-2.25170000	1.32366300
H	-6.34023400	-0.33735900	2.13309000
H	-4.98783700	-4.01279100	0.36561100
H	-6.52718700	-2.79732600	1.88194700
C	0.64037600	-2.24356400	-0.69270400
C	-0.22765300	-2.89238200	0.18148700
C	1.53547500	-3.08774800	-1.34957200
C	-0.20898400	-4.26450100	0.40643300
C	1.59682500	-4.45856300	-1.15186800
C	0.71299800	-5.05363300	-0.26186700
C	1.97302400	0.09901700	-1.35663000
C	2.00895600	1.31223200	-2.03509200
C	3.16899200	-0.26094200	-0.74013800
C	3.14197900	2.10993300	-2.13239600
C	4.32534200	0.50422200	-0.80711900
C	4.31316700	1.69844200	-1.51529300
F	-1.07488800	-4.82289400	1.25216200
F	-1.16233900	-2.21095000	0.86688500
F	2.39500900	-2.57710700	-2.24585900
F	2.47935500	-5.20617000	-1.81124100
F	0.74539300	-6.36734100	-0.06253900
F	3.23306200	-1.36309500	0.02159000
F	5.43821900	0.11198100	-0.19092600
F	5.41091800	2.44344800	-1.59198900
F	3.10657600	3.26472600	-2.79424400
F	0.88933800	1.80597000	-2.59930000
H	-1.12765800	-0.62808800	-1.79911300
C	-0.19085100	-0.81470500	-2.77524800
H	0.80629400	-1.11858000	-3.10442300
H	-0.46596300	0.08141100	-3.33469900
H	-0.83853000	-1.65402800	-3.05405700

P3_P

C	-1.43649200	-0.24511000	0.10837000
C	-0.08416800	-0.18147000	0.04470800
C	-2.23260800	-0.54842900	1.34303900
C	-2.93193800	-1.74963900	1.49259900
C	-2.32167100	0.42437300	2.34483500
C	-3.70696300	-1.97360900	2.62775900
H	-2.84781400	-2.51801000	0.73020200
C	-3.09515400	0.19769700	3.47750300
H	-1.76922500	1.35316600	2.22590000
C	-3.79363800	-1.00022200	3.61849000
H	-4.23467700	-2.91521900	2.74093100
H	-3.15475200	0.95690300	4.25059800
H	-4.39666600	-1.17736300	4.50320000
C	0.55463900	-0.65399000	1.32743800
C	1.20325900	0.18724700	2.22870700
C	0.45326700	-1.99395000	1.71579800
C	1.82755900	-0.28168900	3.37716500
C	1.07567300	-2.49093500	2.85123400
C	1.78090200	-1.63296000	3.68198700
F	-0.30531900	-2.85575100	1.03167100
F	0.96457200	-3.77904600	3.16643800
F	2.36884200	-2.09612100	4.77909600
F	2.43203700	0.56666600	4.20376000
F	1.15682400	1.51200600	2.07220500
B	0.91592800	0.37688500	-1.18687100
P	-2.53944700	0.04173800	-1.27898200
C	-4.00707700	0.94439100	-0.74730200
C	-3.99963800	2.33810300	-0.86794100
C	-5.09544500	0.28824500	-0.16197400
C	-5.08762300	3.07092900	-0.40354900
H	-3.14593800	2.84891000	-1.30303300
C	-6.17581400	1.03081800	0.29834100
H	-5.09370600	-0.79288500	-0.05970000
C	-6.17205000	2.41925500	0.17624000
H	-5.08427800	4.15152200	-0.49611700
H	-7.01954600	0.52553600	0.75572400
H	-7.01858500	2.99496100	0.53631600
C	-3.00536700	-1.53794200	-2.02169200
C	-2.11727600	-2.61502300	-1.92439200
C	-4.20195100	-1.66013000	-2.73511700
C	-2.43845500	-3.81814300	-2.54582300
H	-1.18995800	-2.51572000	-1.36440200
C	-4.51177900	-2.86861400	-3.34912100

H	-4.88877100	-0.82141700	-2.80528900
C	-3.63144600	-3.94441600	-3.25351500
H	-1.75332000	-4.65576000	-2.47336100
H	-5.44045700	-2.96980200	-3.90045900
H	-3.87718900	-4.88619500	-3.73342000
C	2.30996600	-0.50520700	-1.27514600
C	2.30801600	-1.88860100	-1.15718300
C	3.51944300	0.00358300	-1.75209400
C	3.41328300	-2.70711700	-1.35560400
C	4.65607200	-0.76468900	-1.96474600
C	4.60924200	-2.13527100	-1.75383100
C	1.17272300	1.96659700	-0.77748900
C	0.24464100	2.95781100	-1.07332500
C	2.28163200	2.45110400	-0.08242900
C	0.39518000	4.30800900	-0.78483300
C	2.49252300	3.78830300	0.22130200
C	1.54308700	4.73342600	-0.14002900
F	3.31958800	-4.02928400	-1.19587900
F	1.15736900	-2.53422100	-0.86917600
F	3.63317000	1.29911000	-2.08232600
F	5.78407100	-0.20117900	-2.39910300
F	5.68744300	-2.88937200	-1.95888600
F	3.19867100	1.60406100	0.40545800
F	3.58371900	4.16993800	0.88434800
F	1.71717700	6.01958700	0.15503900
F	-0.56143300	5.18140300	-1.11439000
F	-0.94094300	2.64717900	-1.66391600
H	-1.97439100	0.83959200	-2.26367900
C	0.35016400	0.24531600	-2.73192000
H	1.21304800	0.29104000	-3.40405500
H	-0.32336100	1.02818000	-3.08426700
H	-0.11874300	-0.73101100	-2.92382000

P4_TS

C	-0.04656500	-0.19512800	-1.40558400
H	-0.52328200	-0.31702100	-2.38173100
H	0.56759300	0.70643500	-1.42692400
C	0.80219700	-1.41140800	-0.98572700
H	0.13361500	-2.27999800	-0.91445200
H	1.48547500	-1.64483600	-1.80986800
P	-1.22685000	-0.04656300	-0.01513800
B	1.71861300	-1.28768600	0.36964000
C	-2.01718800	1.58951600	0.23960700
C	-2.71183000	1.73931300	1.46328700
C	-1.93847500	2.68458800	-0.64702500
C	-3.33099500	2.95094800	1.75870700
C	-2.57209500	3.87977700	-0.30049100
C	-3.27804100	4.03564900	0.88783700
H	-3.86342700	3.05006600	2.70173900
H	-2.50862200	4.71775200	-0.99085600
C	-2.52747300	-1.31865900	-0.25526500
C	-3.52113300	-1.18811600	-1.24687400
C	-2.51009800	-2.46008800	0.57786700
C	-4.48448200	-2.18892300	-1.37520300
C	-3.49339600	-3.43390500	0.40657000
C	-4.48879900	-3.31781500	-0.56121600
H	-5.25115000	-2.08100600	-2.13875800
H	-3.47911300	-4.31028700	1.05029800
C	-3.58071900	-0.01715800	-2.19422000
H	-3.68563800	0.93353700	-1.66484200
H	-2.67428500	0.03942600	-2.80464900
H	-4.42713000	-0.12213900	-2.87537900
C	-5.52025100	-4.40102300	-0.73582400
H	-6.38791400	-4.03801100	-1.29074700
H	-5.09961200	-5.24742700	-1.28840500
H	-5.86282600	-4.77884700	0.23081700
C	-1.47412800	-2.67072800	1.65733600
H	-0.45402400	-2.67866500	1.26117200
H	-1.52359700	-1.88237200	2.41522700
H	-1.64450900	-3.62491000	2.15945200
C	-1.19294000	2.66202100	-1.95953000
H	-0.11183300	2.73480300	-1.80732900
H	-1.38389900	1.75454900	-2.53274100
H	-1.49376400	3.51465200	-2.57124200
C	-3.98098000	5.32682100	1.21274300
H	-5.02786500	5.28710300	0.89417900
H	-3.97127300	5.52193400	2.28774500
H	-3.51144000	6.17216100	0.70488500
C	-2.79519100	0.63021600	2.48279200
H	-3.30767200	-0.25277800	2.08699500
H	-1.79386300	0.31985700	2.80103600
H	-3.33773900	0.96669300	3.36796200
H	0.11267300	-0.42175500	0.97499300
H	1.82238100	-2.36737200	0.89587000
C	4.24682200	-0.95507700	0.98936500
C	3.20738700	-0.66691600	0.10263900
C	3.59629000	0.10835400	-0.98546100
C	4.89396500	0.56356400	-1.19463300

C	5.88391400	0.24457500	-0.28062000
C	5.55523800	-0.52160200	0.82910300
F	4.00148200	-1.68316800	2.09162400
F	6.49602600	-0.83505400	1.72073800
F	7.13314600	0.66885000	-0.46071100
F	5.19053800	1.30146800	-2.26662800
F	2.70884000	0.47052800	-1.93238200
C	1.18977300	-0.26079200	1.85222500
H	2.10912000	-0.60604300	2.32708800
H	0.41996300	-0.50887700	2.59523400
H	1.24820400	0.82861700	1.75389700

P4_P

C	0.07166800	-0.06563700	-1.02164100
H	-0.26563300	-0.06265600	-2.06343200
H	0.64758700	0.84773500	-0.84742300
C	0.87588200	-1.32137500	-0.62212600
H	0.17464200	-2.15897800	-0.49889000
H	1.46426900	-1.59473500	-1.50340100
P	-1.38432400	0.00179300	0.05653500
B	1.88947000	-1.16753800	0.68334600
C	-2.25789300	1.58632500	0.25567100
C	-3.03899500	1.72099700	1.42553100
C	-2.16060000	2.65584600	-0.66093300
C	-3.72456500	2.91420800	1.64365400
C	-2.87115400	3.82454400	-0.39237000
C	-3.65554100	3.97723700	0.74856600
H	-4.32795900	3.01207600	2.54239200
H	-2.80308000	4.64633800	-1.10099500
C	-2.51069200	-1.39167300	-0.24178600
C	-3.40646200	-1.36642800	-1.33144100
C	-2.46435100	-2.50512000	0.62676500
C	-4.26389600	-2.44451100	-1.51936900
C	-3.34886100	-3.55946900	0.38789100
C	-4.25493900	-3.54874100	-0.66727000
H	-4.95392200	-2.42549200	-2.35951700
H	-3.31926500	-4.42006700	1.05070300
C	-3.44588300	-0.22873800	-2.31954800
H	-3.65375000	0.73061400	-1.83699100
H	-2.49331500	-0.14367000	-2.85322900
H	-4.21998000	-0.40138600	-3.06882900
C	-5.19679400	-4.69943700	-0.90028800
H	-6.23678700	-4.36110700	-0.87798800
H	-5.02099300	-5.15151900	-1.88076600
H	-5.07318900	-5.47416600	-0.14149200
C	-1.51433600	-2.62957200	1.79692600
H	-0.46391000	-2.51177400	1.50947500
H	-1.72869400	-1.88791400	2.57488700
H	-1.62395400	-3.61431900	2.25332300
C	-1.31732100	2.62773000	-1.91312400
H	-0.25050200	2.67730700	-1.67932500
H	-1.48280400	1.73007400	-2.51064900
H	-1.55741200	3.48884500	-2.53855800
C	-4.38201100	5.26834200	1.01336100
H	-3.69222600	6.01862600	1.41255700
H	-4.81256500	5.67337100	0.09439000
H	-5.18409800	5.13092100	1.74108600
C	-3.14833600	0.63499700	2.46833300
H	-3.48432700	-0.31545200	2.04190600
H	-2.18473400	0.46479700	2.96317200
H	-3.86387500	0.92544400	3.23867100
H	-0.88257500	-0.25072700	1.32595700
H	2.08020300	-2.29356500	1.11182900
C	4.46701300	-0.80326100	1.04359500
C	3.36493100	-0.58798300	0.21466300
C	3.66666600	0.13620800	-0.93178700
C	4.93724600	0.59721900	-1.26191300
C	5.99431300	0.34300200	-0.40494200
C	5.75400800	-0.36341300	0.76538300
F	4.31350100	-1.46460200	2.20169600
F	6.76281700	-0.60772800	1.60807300
F	7.22324600	0.77706900	-0.69593300
F	5.14498000	1.28387900	-2.39190600
F	2.69895200	0.45671100	-1.82694100
C	1.39006100	-0.20364800	1.92848000
H	2.22033600	-0.07999200	2.63174300
H	0.56908900	-0.62087500	2.53355700
H	1.09452900	0.81919300	1.63449000

P5_TS

P	-1.49079600	-0.94219600	-0.15117500
B	1.59699100	-0.70119000	0.05508800
C	-0.43570000	-1.74249000	-1.42764400
C	0.99478900	-1.17939700	-1.40447100
C	-3.00854900	-1.98489700	-0.09111900
C	-5.12982000	-2.70288900	-1.00138800
C	-5.31886800	-3.60095500	0.04553400
C	-4.33022200	-3.68245900	1.02104300

C	-3.18025800	-2.89420800	0.97122300
C	-2.08099100	0.78344000	-0.42956100
C	-1.86087600	1.58280700	-1.56777400
C	-2.36553400	2.88749900	-1.58999700
C	-3.06944600	3.43725000	-0.52707100
C	-3.27338100	2.63636400	0.59472900
C	-2.79185500	1.33238800	0.66996200
C	3.22374200	-0.90671100	0.04160600
C	3.77846400	-2.15083000	-0.26587200
C	5.14019800	-2.40186800	-0.35073800
C	6.03529500	-1.36803000	-0.11824500
C	5.54309700	-0.11345700	0.19926600
C	4.16859500	0.09080500	0.27945900
C	1.13802400	0.83109800	0.41940100
C	1.33362200	1.84259000	-0.52115100
C	0.80264800	3.11725700	-0.41857700
C	0.05195800	3.45223200	0.69961000
C	-0.11024700	2.51376100	1.70209200
C	0.44719500	1.24883300	1.55129700
C	-6.54439500	-4.47450000	0.09933300
C	-2.15522500	-3.05740300	2.06611900
C	-1.07989800	1.17249300	-2.79330500
C	-3.54575300	4.86398600	-0.56031500
C	-3.01436600	0.57922800	1.96021300
F	2.98078500	-3.21135500	-0.50078300
F	5.59141200	-3.61991100	-0.65053200
F	7.34602800	-1.58091800	-0.19807400
F	6.38815000	0.89067200	0.43076400
F	3.80292900	1.33708300	0.60944800
F	2.05615500	1.59478000	-1.62832200
F	0.95254800	3.99850100	-1.40797900
F	-0.52084900	4.65094000	0.79405400
F	-0.86442400	2.79607100	2.76634700
F	0.18998900	0.39358200	2.56608300
H	-0.87768200	-1.81330500	-2.42466400
H	-0.42432300	-2.76883700	-1.03140400
H	1.05868300	-0.33160200	-2.08910600
H	1.63938700	-1.95095000	-1.83295300
H	-5.88890100	-2.62663100	-1.77694900
H	-4.45312900	-4.37894800	1.84694400
H	-3.80634100	3.04472300	1.44997100
H	-6.68218700	-4.90431800	1.09366600
H	-7.44300100	-3.90850400	-0.15892000
H	-6.46075500	-5.30069500	-0.61395800
H	-1.91945100	-2.10034500	2.54391200
H	-1.22203700	-3.47675200	1.67593600
H	-2.52393200	-3.73733000	2.83619700
H	-1.56723000	1.56467100	-3.68973400
H	-0.07061200	1.59743300	-2.75919600
H	-0.97972200	0.09810100	-2.91148100
H	-2.78417300	5.51831200	-0.12533400
H	-4.46512000	4.98951100	0.01646100
H	-3.72979700	5.19769600	-1.58404100
H	-3.48360200	1.23363600	2.69655200
H	-3.65943200	-0.29439900	1.82182500
H	-2.06478300	0.23856800	2.38444400
C	-3.99767400	-1.89413300	-1.09539100
C	-3.91119400	-0.95541200	-2.27380300
H	-4.18101200	0.06645900	-1.99026700
H	-2.90645000	-0.91617900	-2.69944500
H	-4.59428800	-1.28255400	-3.06017200
H	-2.18063700	3.49329800	-2.47495200
H	-0.06718300	-1.23714700	0.86942500
C	1.10003000	-1.82596800	1.40573900
H	0.26574400	-1.97526800	2.10501800
H	1.35859200	-2.81022000	1.01574900
H	1.86541100	-1.45307600	2.08841000

P5_P

P	-1.72590500	-0.85703900	-0.13810300
B	1.68268100	-0.97330000	0.34365700
C	-0.49185200	-1.88875700	-1.01304800
C	0.97384900	-1.44448200	-1.08371700
C	-3.26721800	-1.82268700	-0.10835500
C	-5.33690000	-2.51658500	-1.12715700
C	-5.63252800	-3.33152300	-0.03869000
C	-4.71741200	-3.38780100	1.01199400
C	-3.53347200	-2.65504200	1.00223300
C	-2.08418400	0.88322000	-0.51735300
C	-1.69582800	1.54577100	-1.69256100
C	-2.03908200	2.89233200	-1.84303400
C	-2.73604500	3.59463100	-0.86862000
C	-3.11733200	2.91230400	0.28835900
C	-2.80619000	1.57332800	0.49179000
C	3.32476100	-1.00406800	0.11815100
C	3.94635400	-2.18904800	-0.28150700
C	5.31278400	-2.33654300	-0.48008800
C	6.15138400	-1.25451500	-0.26508100

C	5.59631700	-0.05552000	0.14823500
C	4.22109800	0.04090200	0.33592800
C	1.14908400	0.57749800	0.62896000
C	1.36684500	1.57076400	-0.32933200
C	0.86066900	2.85782100	-0.26366400
C	0.10496400	3.23245800	0.83765800
C	-0.11529900	2.30766500	1.84085500
C	0.39751100	1.01867900	1.71307100
C	-6.88954800	-4.15896700	-0.00228200
C	-2.59149000	-2.79139700	2.17508000
C	-0.88763500	0.93136800	-2.80674000
C	-3.01606400	5.06380200	-1.01929700
C	-3.20648600	0.93999700	1.80362700
F	3.22118100	-3.30624700	-0.49993500
F	5.82349000	-3.50880700	-0.86664600
F	7.46728200	-1.36828500	-0.44742300
F	6.38621200	0.99770800	0.37150700
F	3.80164800	1.24569700	0.76365500
F	2.10585000	1.29923600	-1.42118600
F	1.03974600	3.71940600	-1.26854100
F	-0.43973800	4.44946000	0.90403800
F	-0.90116200	2.63100000	2.87505900
F	0.02587000	0.17115800	2.70255500
H	-0.93687600	-2.14111900	-1.98391700
H	-0.57240600	-2.80018100	-0.40149000
H	1.08776100	-0.67516800	-1.85112400
H	1.49624800	-2.32744200	-1.46364800
H	-6.03389100	-2.46777400	-1.96000200
H	-4.92913000	-4.02613300	1.86636800
H	-3.65107200	3.44701100	1.06963500
H	-7.35013400	-4.12943900	0.98837500
H	-7.61956500	-3.80504700	-0.73274900
H	-6.66614700	-5.20605700	-0.22972300
H	-2.44895900	-1.84325600	2.70598800
H	-1.60139000	-3.14488400	1.86963000
H	-2.98869200	-3.51158100	2.89159100
H	-1.31704900	1.20668400	-3.77384300
H	0.13659600	1.31832800	-2.77295900
H	-0.82635800	-0.15366800	-2.76113500
H	-3.96160900	5.33935300	-0.54670900
H	-3.04957500	5.35898600	-2.07000200
H	-2.21805800	5.63261000	-0.53228500
H	-3.78599000	1.64987500	2.39480600
H	-3.81803500	0.04326000	1.66162900
H	-2.32767800	0.67053700	2.39966800
C	-4.16781000	-1.75810200	-1.18805700
C	-3.93399000	-0.90090500	-2.40792900
H	-4.12193200	0.15590800	-2.19613100
H	-2.90958700	-0.98184100	-2.78407400
H	-4.60074000	-1.21074900	-3.21444900
H	-1.72168000	3.40668300	-2.74715400
H	-1.30118000	-0.82845500	1.18718400
C	1.34654800	-2.01104800	1.56043100
H	0.28189900	-2.09817500	1.81257400
H	1.69604800	-3.01566700	1.29777500
H	1.84614200	-1.72674600	2.49251900

P6_TS

P	1.77723200	-0.66937500	0.39595200
C	1.29961000	-0.94476600	2.16663000
H	2.19397800	-1.08551100	2.77690700
H	0.77031000	-0.06554700	2.53092900
C	0.35962000	-2.15694300	2.27514500
H	0.74823600	-2.98415500	1.66569300
H	0.41555000	-2.51778400	3.30842000
C	-1.11172000	-1.84535300	1.93964300
H	-1.50628200	-1.20642100	2.73247400
H	-1.66424500	-2.79149200	2.02823700
B	-1.49582400	-1.21435900	0.48271700
C	2.10274300	1.10626100	-0.01166000
C	2.18563100	2.18320600	0.90321700
C	2.03958600	2.08323000	2.40536500
H	2.47738200	2.96967800	2.86876400
H	2.53139300	1.21183300	2.83044800
H	0.98713200	2.05570800	2.70293800
C	2.37458700	3.47621800	0.40643700
H	2.42742800	4.29677200	1.11818500
C	2.47255200	3.75682400	-0.95175000
C	2.60392000	5.17107900	-1.44688100
H	3.15082800	5.21257400	-2.39154200
H	3.11946000	5.80104300	-0.71837000
H	1.60952900	5.59639100	-1.61382500
C	2.39315400	2.68765500	-1.83745500
H	2.45800400	2.87399000	-2.90648300
C	2.20738700	1.37857100	-1.39708200
C	2.08188200	0.30150500	-2.44624100
H	1.10471600	-0.19176000	-2.39026600
H	2.85782900	-0.46309200	-2.33884000

H	2.17314300	0.73473000	-3.44359000
C	3.38154200	-1.58212600	0.15950900
C	4.54550800	-1.23232100	0.89720800
C	4.62069300	-0.07423700	1.86031300
H	5.65379700	0.07463100	2.17917400
H	4.03106200	-0.25083600	2.76392900
H	4.27553600	0.85521400	1.40434700
C	5.71346800	-1.96913300	0.72887900
H	6.59268700	-1.68571300	1.30300200
C	5.80070600	-3.04424900	-0.15358300
C	7.08228300	-3.81979100	-0.29825000
H	7.91337800	-3.15670200	-0.55497800
H	6.99971500	-4.58142300	-1.07611700
H	7.34106000	-4.31930800	0.64026400
C	4.67200700	-3.35265400	-0.89493800
H	4.71400500	-4.16942800	-1.61192400
C	3.47045200	-2.64696600	-0.76286800
C	2.34918400	-3.11967500	-1.65647500
H	1.62929600	-2.34140700	-1.89606500
H	1.80788600	-3.95200500	-1.19506400
H	2.76347000	-3.49096300	-2.59678600
C	-1.16881600	0.37065100	0.18297600
C	-1.10774900	0.84036300	-1.12932100
F	-1.24311200	-0.01970100	-2.15646200
C	-0.87770500	2.16217500	-1.47653300
F	-0.73750100	2.51970300	-2.75256300
C	-0.75743500	3.11193900	-0.47349300
F	-0.50204200	4.38379700	-0.77523800
C	-0.87688700	2.71352300	0.84635800
F	-0.71223700	3.60768500	1.82385300
C	-1.07925400	1.37059700	1.14678300
F	-1.13011100	1.08155200	2.46127000
C	-3.12116600	-1.29519400	0.18835900
C	-3.98826300	-0.75511400	1.14030600
F	-3.49406900	-0.20590400	2.26207800
C	-5.36814400	-0.70813900	1.00330000
F	-6.13151800	-0.18718800	1.96384400
C	-5.95235000	-1.19930700	-0.15513900
F	-7.27341000	-1.16436000	-0.31191800
C	-5.13846400	-1.71431800	-1.14975300
F	-5.67932900	-2.17620200	-2.27759000
C	-3.75855700	-1.73851000	-0.96936700
F	-3.06151300	-2.23027100	-2.00858200
H	0.28812500	-1.44331100	-0.23516600
C	-0.66263200	-2.36886700	-0.65786800
H	-1.57789300	-2.94846300	-0.51182700
H	0.09191600	-3.09320100	-0.34463300
H	-0.59845100	-2.19042600	-1.72995600

P6_P

P	-1.98111800	-0.65551500	-0.34321900
C	-1.11627700	-1.38516600	-1.76973800
H	-1.88629700	-1.62611700	-2.50875100
H	-0.44992900	-0.63723300	-2.20082900
C	-0.28681000	-2.62838500	-1.36497100
H	-0.66060600	-3.06781400	-0.42995700
H	-0.46590000	-3.38665700	-2.13481300
C	1.20914700	-2.30007800	-1.26567900
H	1.50260400	-1.84120700	-2.21517100
H	1.74058600	-3.25957400	-1.23434500
B	1.72122900	-1.46080900	0.05447500
C	-2.23201600	1.14277300	-0.33892900
C	-2.08764900	1.96565500	-1.47849300
C	-1.70064300	1.48816400	-2.85816300
H	-1.97793900	2.24556500	-3.59330900
H	-2.18941100	0.55748300	-3.14615600
H	-0.61712900	1.34768700	-2.93455200
C	-2.26934900	3.33972000	-1.32629200
H	-2.14635200	3.97523700	-2.19954700
C	-2.57338100	3.92523900	-0.10040500
C	-2.68969000	5.41770800	0.03124200
H	-3.31549600	5.69557900	0.88169900
H	-3.10793000	5.86507900	-0.87322100
H	-1.69506900	5.84563300	0.18918700
C	-2.71301600	3.09331700	1.00633800
H	-2.93556800	3.52789300	1.97705900
C	-2.54896800	1.71364400	0.91629000
C	-2.65527500	0.90529700	2.18578200
H	-1.67558900	0.51723800	2.48751800
H	-3.35141800	0.06693100	2.08704300
H	-3.01213200	1.53756900	2.99959700
C	-3.52502300	-1.56680700	-0.02909400
C	-4.63827300	-1.36806300	-0.87517500
C	-4.61469500	-0.43836900	-2.06315200
H	-5.59402900	-0.41754200	-2.54350200
H	-3.89528200	-0.77106200	-2.81832800
H	-4.35706300	0.58596800	-1.78108100
C	-5.81393700	-2.06618000	-0.61334700

H	-6.67046800	-1.90793600	-1.26387200
C	-5.92302100	-2.95496200	0.45353100
C	-7.19682600	-3.71874100	0.69519100
H	-7.21021700	-4.63553800	0.09706900
H	-8.07157800	-3.12803500	0.41428700
H	-7.29381300	-4.00557600	1.74433800
C	-4.81319700	-3.13458000	1.27340300
H	-4.88145800	-3.82196200	2.11245400
C	-3.60838600	-2.46407900	1.06016700
C	-2.47068800	-2.75850500	2.00953100
H	-2.05572000	-1.85890300	2.47429100
H	-1.64679100	-3.27471600	1.50935100
H	-2.82059300	-3.40744600	2.81343500
C	1.16424900	0.12828000	0.12560800
C	0.75109900	0.76782800	1.29494000
F	0.53118300	0.06644400	2.42487600
C	0.47418300	2.12558800	1.40008000
F	0.01351500	2.63956300	2.54539600
C	0.60097800	2.93640600	0.28511200
F	0.30652900	4.23697500	0.35024200
C	0.97650700	2.35821200	-0.91608200
F	1.00676700	3.10200000	-2.02767700
C	1.24442100	0.99549700	-0.96424700
F	1.57555900	0.52431000	-2.18615400
C	3.36962800	-1.24956400	0.03240300
C	4.25421000	-1.64861700	-0.96632000
F	3.82460400	-2.27107900	-2.07793300
C	5.63156400	-1.45088200	-0.90920300
F	6.41887700	-1.86861700	-1.90483300
C	6.19049200	-0.81653400	0.18685300
F	7.50755000	-0.61886400	0.25995000
C	5.35725500	-0.38835400	1.20955700
F	5.87546900	0.22947700	2.27477500
C	3.99144300	-0.61086300	1.10787000
F	3.25484700	-0.15197000	2.13820200
H	-1.17346000	-0.93032100	0.76183600
C	1.33887700	-2.33867100	1.38379900
H	1.82796200	-2.00291200	2.30022300
H	1.63703000	-3.38182900	1.21376600
H	0.26399600	-2.35842700	1.60815600

P7_TS

P	-1.86088500	-0.20357800	-0.21455000
B	1.41610700	0.00070600	-0.29129800
C	-0.95064700	0.08851300	2.73872300
H	-1.71014900	0.72810800	3.19489800
C	-0.79408600	0.25081800	1.21275600
H	-0.58426400	1.30638200	1.03052600
C	0.48588300	-0.58899700	0.90531800
H	0.15168700	-1.59153900	0.61497900
C	1.12289300	-0.73582900	2.32265500
H	2.21395400	-0.79208000	2.32278600
C	0.41534800	-1.92272000	2.99977900
H	0.86520700	-2.13805100	3.97436300
H	0.47025300	-2.83128300	2.39606000
C	-1.04313100	-1.39493700	3.16481700
H	-1.75842000	-1.96030500	2.56374300
H	-1.37190400	-1.46004000	4.20584000
C	0.50775800	0.43004300	3.12125100
H	0.69522100	0.34409100	4.19638100
H	0.81578600	1.42420700	2.78187100
C	-2.74178700	-1.77483600	0.13965700
C	-3.86031100	-1.79715100	1.00334300
C	-4.49409100	-3.01015700	1.26003200
H	-5.35181300	-3.02150700	1.92877500
C	-4.05669200	-4.20675000	0.69383500
C	-2.95992900	-4.16427700	-0.15992700
H	-2.60952700	-5.08573700	-0.61822200
C	-2.29161800	-2.97312300	-0.45423200
C	-4.38364200	-0.55847400	1.68454400
H	-5.10860900	-0.82759100	2.45491300
H	-4.86977300	0.11848400	0.97597000
H	-3.57380600	-0.00295500	2.16637200
C	-4.74325000	-5.50681600	1.01891600
H	-4.43091600	-5.87128300	2.00276500
H	-4.50046400	-6.27813300	0.28517400
H	-5.82891300	-5.38377800	1.04413800
C	-1.12632500	-3.04265600	-1.41395100
H	-1.09769900	-4.02310800	-1.89330200
H	-0.16581600	-2.89688900	-0.90964600
H	-1.20593900	-2.28665600	-2.19854500
C	-3.10436100	1.02239200	-0.78605300
C	-3.72628400	0.70671200	-2.01815900
C	-4.68007600	1.57344200	-2.54879200
H	-5.15365000	1.31348000	-3.49249000
C	-5.02949400	2.76202600	-1.91600800
C	-4.37569200	3.07913900	-0.72968500
H	-4.61256300	4.01570700	-0.23030000

C	-3.41812000	2.24380500	-0.15231100
C	-3.37979800	-0.52846300	-2.81491700
H	-3.65812700	-1.45058700	-2.29586000
H	-2.30364900	-0.57441300	-3.01685700
H	-3.89612700	-0.51333700	-3.77622600
C	-6.08612600	3.66923700	-2.48818700
H	-6.14765500	3.56883900	-3.57416200
H	-5.88021200	4.71530300	-2.25008000
H	-7.07072400	3.42398500	-2.07666600
C	-2.75756100	2.73720800	1.10992100
H	-3.34206800	3.54965000	1.54547500
H	-1.75946400	3.13065400	0.89029400
H	-2.65702900	1.95492700	1.86170800
C	1.79763200	1.54911300	0.05726400
F	3.79764100	0.92986800	1.17237000
C	2.92154400	1.87972300	0.81184400
F	4.29643200	3.41682100	1.96963200
C	3.20241800	3.16669300	1.25297500
F	2.57054200	5.43679100	1.37635700
C	2.32660200	4.20057700	0.95064900
F	0.31137200	4.89095600	-0.06429100
C	1.18913600	3.92409700	0.20847900
F	-0.18851700	2.43118800	-0.90958900
C	0.96213500	2.62386600	-0.22382300
C	2.70637200	-0.93606400	-0.64599100
F	1.90180500	-2.92460300	0.40637200
C	2.85317200	-2.27418200	-0.29011800
F	4.02692000	-4.32621700	-0.24358300
C	3.96080900	-3.04953500	-0.62085900
F	6.06113600	-3.21407000	-1.67761800
C	4.99588000	-2.48930700	-1.34946700
F	5.88205900	-0.60512200	-2.44987300
C	4.90080300	-1.16115600	-1.74135800
F	3.75072300	0.84384700	-1.81421500
C	3.77714200	-0.43072900	-1.38730900
H	-0.35377100	-0.13872200	-1.14434000
C	0.66986300	0.03725100	-2.05020000
H	0.71298600	-0.94667800	-2.52042700
H	1.57765500	0.58381400	-2.31879100
H	-0.11219800	0.64106700	-2.52889700

P7_P

P	-1.85608700	0.54584300	0.16493700
B	1.59360700	0.37012300	-0.35978500
C	-0.66150000	1.63858700	2.64881500
H	-1.40856800	2.40315700	2.87893300
C	-0.50513400	1.30158100	1.14882900
H	-0.26284500	2.21401100	0.59848800
C	0.74829400	0.36381200	1.11169700
H	0.37446800	-0.66231500	1.22999300
C	1.38867000	0.69373700	2.50016700
H	2.47719900	0.61675400	2.50884400
C	0.68158100	-0.20476500	3.52951900
H	1.13068800	-0.09598100	4.52210600
H	0.73051400	-1.26148700	3.25221600
C	-0.77384000	0.36005700	3.51575900
H	-1.49700300	-0.35962200	3.12511000
H	-1.10643900	0.62928000	4.52219600
C	0.79753100	2.05660900	2.90776000
H	0.97827300	2.28920100	3.96225900
H	1.11783000	2.89687800	2.29424300
C	-3.03608400	-0.48475500	1.09470800
C	-4.07875500	0.11824300	1.82950300
C	-4.94941800	-0.69532500	2.54916800
H	-5.74916100	-0.22787800	3.11820300
C	-4.81945100	-2.08206700	2.56767900
C	-3.78363100	-2.65393000	1.83450500
H	-3.66372800	-3.73408800	1.84084400
C	-2.88365000	-1.89034600	1.09137400
C	-4.27871500	1.61034100	1.89102400
H	-5.14908200	1.85052600	2.50351200
H	-4.43261400	2.04448200	0.89953800
H	-3.41525900	2.10253800	2.34808300
C	-5.78704100	-2.93839600	3.33896200
H	-5.32191100	-3.87448000	3.65503500
H	-6.65340700	-3.19052900	2.71888700
H	-6.15497900	-2.41768200	4.22581600
C	-1.81002300	-2.62177200	0.32341500
H	-1.84536400	-3.68728900	0.55544100
H	-0.80313400	-2.26812600	0.56102400
H	-1.94666900	-2.51988900	-0.75882400
C	-2.71339900	1.61196900	-1.04117000
C	-3.30494000	0.94217800	-2.14148400
C	-3.95561500	1.69712200	-3.11137100
H	-4.41104000	1.18161000	-3.95331600
C	-4.03646600	3.08674200	-3.03901000
C	-3.44264900	3.71958000	-1.95381800
H	-3.49220900	4.80294400	-1.87912400

C	-2.77301000	3.01611500	-0.95021600
C	-3.24413800	-0.55458700	-2.33228000
H	-3.59631100	-1.09924100	-1.45098100
H	-2.22348900	-0.88065000	-2.56465600
H	-3.87565700	-0.84677000	-3.17235300
C	-4.74665900	3.86741600	-4.11177900
H	-4.26164400	3.71974500	-5.08100300
H	-4.74825500	4.93638200	-3.89135000
H	-5.78423700	3.53588900	-4.21150000
C	-2.13889000	3.83833400	0.14539400
H	-2.65324300	4.79745200	0.23138900
H	-1.08978400	4.04817700	-0.09014400
H	-2.17106300	3.35611600	1.12187400
C	3.20990800	0.65886300	-0.14249800
F	4.08239900	-1.33146900	-1.13668700
C	4.27867000	-0.14586700	-0.53534000
F	6.58422500	-0.63224100	-0.76420700
C	5.61690600	0.19673900	-0.36484400
F	7.22679300	1.75387700	0.38384000
C	5.95041500	1.40922900	0.21402200
F	5.22230200	3.44363900	1.15768000
C	4.93091600	2.26223600	0.60636000
F	2.68619400	2.77290500	0.80145800
C	3.61353900	1.87228800	0.41191400
C	1.34111700	-1.16783300	-0.93814000
F	2.47314900	-2.01718500	0.94982100
C	1.78070700	-2.25407000	-0.17704000
F	2.00139900	-4.56943700	0.28390300
C	1.54224300	-3.58279200	-0.48787800
F	0.52779500	-5.15629300	-1.93274200
C	0.79351400	-3.88779700	-1.62078800
F	-0.45897500	-3.11443900	-3.46316700
C	0.31497400	-2.85322500	-2.40387300
F	0.03938800	-0.60758400	-2.86194300
C	0.60356800	-1.53437700	-2.05478300
H	-1.19196200	-0.35974000	-0.66169600
C	1.07020200	1.51536500	-1.41059600
H	-0.00136500	1.49213100	-1.65447000
H	1.58528300	1.42250700	-2.37246300
H	1.28893500	2.52029800	-1.03087400

P8_TS

C	0.37876600	-1.18908800	0.73211800
H	0.49375200	-0.66082500	1.68155300
H	-0.16360600	-2.11413600	0.93801500
P	1.98016600	-1.58569500	-0.06631800
B	-0.39317500	-0.24853900	-0.38481800
C	3.44064200	-0.80884800	0.84700800
C	3.06377800	0.60232900	1.31806600
H	2.23524100	0.59724800	2.03160900
H	3.93468100	1.03413700	1.82389300
H	2.80471100	1.25937800	0.48587200
C	4.60483000	-0.67940100	-0.14746300
H	5.44855800	-0.18841700	0.35035300
H	4.95372000	-1.64622900	-0.51633500
H	4.31552900	-0.06866500	-1.00801000
C	3.87006400	-1.61552300	2.07729200
H	4.27483500	-2.59522300	1.81597800
H	4.66036900	-1.06503400	2.60071500
H	3.04000300	-1.75476400	2.77695800
C	2.11490400	-3.46721600	-0.11285800
C	1.02813700	-3.93445300	-1.09678300
H	0.02385200	-3.66119400	-0.76259800
H	1.18642900	-3.52077200	-2.09769200
H	1.06787000	-5.02670300	-1.17377500
C	3.47546800	-3.89799600	-0.67465900
H	3.69112700	-3.40678900	-1.62935800
H	4.29827100	-3.69287600	0.01425700
H	3.45946100	-4.97922700	-0.85227200
C	1.85767800	-4.14435400	1.23953600
H	1.81870200	-5.22940500	1.08776900
H	2.64082800	-3.94188500	1.97050900
H	0.89948800	-3.83869300	1.66869400
C	-2.01687000	-0.29346200	-0.21222800
C	-2.68060400	-1.51709700	-0.11991100
C	-2.86056800	0.81465800	-0.13108100
C	-4.04788800	-1.65768600	0.06647300
C	-4.23513700	0.72367400	0.06286500
C	-4.83561100	-0.52060000	0.16420200
F	-1.99439700	-2.67420900	-0.23109600
F	-4.60474700	-2.86591100	0.14122900
F	-4.97977900	1.82493600	0.13982900
F	-2.38673900	2.06250600	-0.24605300
F	-6.14851000	-0.62357400	0.34306200
C	0.25710500	1.24626800	-0.36699400
C	1.04216400	1.84793300	-1.34411400
C	0.10275800	2.00330100	0.79352500
C	1.62626300	3.10490100	-1.19515300

C	0.66238700	3.25421100	0.99029700
C	1.43770600	3.81183600	-0.01972900
F	-0.62815600	1.50587500	1.80365400
F	0.47089600	3.92192600	2.12657500
F	1.99021900	5.01012900	0.14126300
F	2.36751900	3.62375700	-2.17339500
F	1.31423800	1.22949300	-2.50789400
H	1.01049100	-1.00868700	-1.41081700
C	-0.19631800	-0.94130500	-2.08579200
H	0.57858000	-1.04611500	-2.86030000
H	-0.75410300	-1.87641900	-2.06071500
H	-0.79820700	-0.12743000	-2.49770500

P8_P

C	1.13262900	-0.68100700	0.43710200
H	1.26888600	0.01820300	1.26907100
H	0.89819800	-1.64308400	0.88825100
P	2.74386800	-0.88219400	-0.31590300
B	-0.12985000	-0.18125800	-0.59741700
C	3.79644700	0.63660500	-0.14836800
C	2.86698900	1.84631500	-0.34589500
H	2.14628400	1.94785700	0.46892200
H	3.48446200	2.75041500	-0.36696600
H	2.30737500	1.79800700	-1.28602900
C	4.87170200	0.65617100	-1.24405300
H	5.43950600	1.58785500	-1.15516700
H	5.57853500	-0.17122100	-1.16153600
H	4.42291000	0.63608000	-2.24180400
C	4.43422100	0.70141200	1.24444200
H	5.19052000	-0.07533900	1.38807100
H	4.92799800	1.67173500	1.35836400
H	3.68673400	0.61573100	2.03987900
C	3.51464300	-2.49026700	0.20177000
C	2.62953900	-3.57525800	-0.44115500
H	1.57969000	-3.49157800	-0.14699400
H	2.68686900	-3.54169400	-1.53360500
H	2.99174700	-4.55585700	-0.11593700
C	4.94589800	-2.63034900	-0.32710800
H	5.00111700	-2.46783200	-1.40838300
H	5.63853500	-1.94462600	0.16812200
H	5.29253100	-3.64966800	-0.12792300
C	3.49036800	-2.64179100	1.72837500
H	3.89056600	-3.62791300	1.98510400
H	4.10633500	-1.89169700	2.23019300
H	2.47485600	-2.58213500	2.12776700
C	-1.50448300	-1.06701100	-0.31835800
C	-1.62142600	-2.34018400	0.23017700
C	-2.72217500	-0.54985200	-0.77091600
C	-2.82052700	-3.03099500	0.37279700
C	-3.94483700	-1.19697800	-0.65346100
C	-3.99825500	-2.45275100	-0.06715400
F	-0.53737800	-3.03306500	0.66431900
F	-2.83832600	-4.24809100	0.92093100
F	-5.06251200	-0.62974600	-1.10762100
F	-2.76023800	0.64123100	-1.39030100
F	-5.15763000	-3.09635400	0.05525800
C	-0.44071400	1.37756700	-0.16318000
C	-0.33894200	2.50119100	-0.97538800
C	-0.82788100	1.65184500	1.14643300
C	-0.58666900	3.79671300	-0.53407400
C	-1.09055100	2.92482500	1.63558500
C	-0.97326800	4.01089700	0.78024700
F	-0.93655300	0.63946800	2.02826200
F	-1.44984200	3.11353200	2.90649400
F	-1.21307300	5.24522500	1.22011000
F	-0.45256100	4.83532900	-1.36116700
F	0.06334600	2.39454900	-2.25429000
H	2.62577900	-1.04382700	-1.70315200
C	0.20950800	-0.38410400	-2.17817300
H	1.05915700	0.19821900	-2.55675200
H	0.41044800	-1.44341100	-2.39859500
H	-0.64822300	-0.09168100	-2.79109300

P9_TS

C	0.13640900	-0.54202400	-0.79802400
H	0.13689800	0.14431900	-1.65006000
H	0.03726400	-1.55184500	-1.20410900
B	-1.08092800	-0.11562300	0.25591100
C	-1.21673000	1.49242500	0.29704600
C	-0.92187700	2.30131200	1.40041900
C	-1.59779100	2.15822000	-0.88275100
C	-0.97021800	3.69498100	1.33166400
C	-1.65669600	3.54477900	-0.96562700
C	-1.33048600	4.32325100	0.14570900
C	-2.45609000	-0.91216400	-0.04208800
C	-3.70912000	-0.28471500	-0.05000700
C	-2.44669500	-2.29690700	-0.27028200
C	-4.88610400	-0.99411700	-0.28617000

C	-3.61249000	-3.01981200	-0.50159500
C	-4.84232100	-2.36574800	-0.51474100
P	1.67159400	-0.37009100	0.17401700
C	2.75019800	0.99825000	-0.54667500
C	4.12617500	1.00779300	0.12971500
H	4.73234600	0.14194100	-0.14873100
H	4.66837200	1.90436900	-0.19102800
H	4.04191800	1.04189400	1.22073900
C	2.91895300	0.87737000	-2.06705700
H	3.43693500	-0.03533800	-2.36687000
H	1.95622100	0.91377300	-2.58272100
H	3.51338600	1.72882700	-2.41961700
C	2.04997700	2.33085000	-0.23921600
H	1.06519000	2.40281800	-0.70948100
H	1.91790200	2.48201800	0.83554100
H	2.67253600	3.14542400	-0.62754600
C	2.57172500	-2.02501900	0.23414700
C	3.51898600	-2.03327100	1.44491600
H	3.97446300	-3.02591200	1.53700300
H	4.32729700	-1.30620300	1.35585800
H	2.97284600	-1.82618200	2.37058800
C	3.33814300	-2.35190000	-1.05058700
H	4.19222100	-1.68679000	-1.20075300
H	3.72498400	-3.37611400	-0.98950500
H	2.68899900	-2.29002400	-1.93009600
C	1.52020100	-3.12201100	0.46653600
H	0.89574200	-2.91406300	1.33989000
H	0.87050500	-3.26285600	-0.40019600
H	2.04117900	-4.06900400	0.64642100
H	-3.76521400	0.78616700	0.13015100
H	-5.83964600	-0.47357000	-0.28938500
H	-5.75692800	-2.92108300	-0.69963500
H	-3.56424500	-4.09156500	-0.67269800
H	-1.49751500	-2.83180400	-0.25579300
H	-0.62743500	1.84193000	2.34138000
H	-0.72175800	4.28821200	2.20724000
H	-1.36704700	5.40668000	0.08628300
H	-1.95524600	4.02226200	-1.89468300
H	-1.85771400	1.56608400	-1.75949000
H	0.52074600	-0.26314600	1.39995100
C	-0.71201500	-0.67346300	2.00897300
H	-0.79255300	-1.76197200	2.00769000
H	-0.06628900	-0.39969500	2.85952200
H	-1.67843700	-0.24855900	2.29849700

P9_P

C	0.02795600	-0.75709600	-0.53382600
H	-0.13613800	-0.22843700	-1.47914400
H	-0.00229400	-1.82976500	-0.74641000
B	-1.18033700	-0.26041500	0.58241600
C	-1.09543900	1.37139000	0.58443800
C	-0.53155000	2.10520300	1.63912300
C	-1.51830700	2.12287900	-0.52799100
C	-0.36814400	3.49199200	1.58491500
C	-1.37058800	3.50459600	-0.59800600
C	-0.78151800	4.19797700	0.45999900
C	-2.60176000	-0.82099800	0.00723900
C	-3.78269500	-0.06475300	0.07369500
C	-2.73974200	-2.12824100	-0.48503000
C	-5.01440300	-0.56756000	-0.34134600
C	-3.96129500	-2.64696200	-0.90703600
C	-5.10958800	-1.86234900	-0.84235300
P	1.67641300	-0.36561200	0.03369200
C	2.59880300	0.74159400	-1.14650600
C	4.00930700	1.05129000	-0.63177500
H	4.66238300	0.17524700	-0.64389700
H	4.45886400	1.80539800	-1.28620000
H	3.99008400	1.46373400	0.38238600
C	2.66103400	0.13048400	-2.55280100
H	3.31104800	-0.74462600	-2.60561500
H	1.66850100	-0.15135900	-2.91532100
H	3.06187800	0.88466200	-3.23801300
C	1.79461000	2.05204100	-1.21096600
H	0.80861200	1.90625400	-1.65533300
H	1.64432500	2.50832200	-0.22674700
H	2.34842100	2.75860800	-1.83862100
C	2.63885500	-1.86482000	0.59410300
C	3.66126000	-1.44917900	1.66248400
H	4.19935400	-2.34224000	1.99766700
H	4.40076600	-0.73617300	1.29365000
H	3.16391300	-1.01407300	2.53467300
C	3.33775300	-2.53407800	-0.59655600
H	4.15090600	-1.92492100	-0.99855700
H	3.77061900	-3.48118800	-0.25820600
H	2.63506300	-2.76015300	-1.40538100
C	1.67225100	-2.87752300	1.22928400
H	1.14185900	-2.45969400	2.08589100
H	0.93067000	-3.25165000	0.51945700

H	2.26478600	-3.73144100	1.57480300
H	-3.73544500	0.94972700	0.46282800
H	-5.90295600	0.05470200	-0.27485700
H	-6.06557200	-2.25719700	-1.17336000
H	-4.01900200	-3.66454400	-1.28454900
H	-1.86693200	-2.77972700	-0.52908400
H	-0.21987300	1.58008500	2.54045500
H	0.07472600	4.02006800	2.42543200
H	-0.66160200	5.27623800	0.41165800
H	-1.71386800	4.04573500	-1.47592200
H	-1.98529600	1.59952700	-1.36238200
H	1.59725600	0.43337500	1.18819400
C	-0.94217400	-0.89163200	2.07090500
H	-1.05789400	-1.98260400	2.07601200
H	0.03138200	-0.66406200	2.53709900
H	-1.70080100	-0.49708700	2.75801000

P10_TS

P	-2.06551000	-0.07060600	-0.69120700
B	1.44429800	0.11321700	-0.39987500
C	-2.41378700	-0.16455700	-2.52705200
H	-3.07657900	0.68924500	-2.69239300
H	-2.98819900	-1.06218200	-2.75871900
C	-1.17939900	-0.01719900	-3.43315100
H	-1.52398600	0.44201200	-4.36568100
H	-0.78347800	-1.00142700	-3.70628800
C	-0.06285800	0.82414200	-2.79879700
H	0.49851400	1.32617500	-3.59055500
H	-0.52299300	1.63985200	-2.23125100
C	0.91730900	-0.03884600	-1.95738800
H	0.56647400	-1.07790300	-2.02748600
H	1.87008600	-0.06356400	-2.50451600
C	-3.18509800	1.33016700	-0.25245800
C	-4.59222600	1.17177500	-0.34125700
C	-5.41303500	2.25389900	-0.04262100
H	-6.49138100	2.12361500	-0.10734400
C	-4.90089400	3.49808600	0.33113800
C	-3.52328400	3.63988900	0.38251400
H	-3.09586000	4.60415300	0.64913400
C	-2.65031500	2.58341800	0.09397600
C	-5.27369300	-0.12686400	-0.70882100
H	-4.76419100	-0.66519500	-1.50959000
H	-5.32353700	-0.80614600	0.14844500
H	-6.29500100	0.07469300	-1.03831000
C	-5.82643700	4.64471500	0.63996600
H	-5.27112200	5.52711600	0.96365000
H	-6.41417900	4.91782800	-0.24162700
H	-6.53157600	4.37474900	1.43138600
C	-1.17581800	2.90247000	0.18932100
H	-0.96998200	3.86206400	-0.29356200
H	-0.86781700	2.99532200	1.23703200
H	-0.53174200	2.16474500	-0.28319500
C	-2.67939500	-1.57186700	0.18309000
C	-2.58516200	-2.84577900	-0.41389800
C	-3.07664900	-3.95717200	0.27287400
H	-2.99927400	-4.93303400	-0.20161800
C	-3.62354000	-3.86240500	1.54643500
C	-3.61432100	-2.61200700	2.15883500
H	-3.97286700	-2.52091400	3.18179600
C	-3.14155900	-1.46784500	1.51804500
C	-1.92333100	-3.12006900	-1.74610300
H	-1.40416100	-4.07943000	-1.69195600
H	-2.65043800	-3.18270800	-2.56280800
H	-1.17209600	-2.37312700	-2.00285900
C	-4.18095000	-5.06914000	2.25280800
H	-3.75539900	-5.99278300	1.85460700
H	-3.97476000	-5.02939000	3.32516000
H	-5.26749100	-5.12233800	2.12794300
C	-3.14524300	-0.18903000	2.32815600
H	-3.14626600	-0.43919500	3.39109700
H	-2.26728300	0.43197400	2.13598700
H	-4.02698300	0.42691400	2.12556500
C	2.23086700	1.49293300	0.01059500
C	2.13884900	2.70388700	-0.66824000
F	1.39319400	2.82014800	-1.77921400
C	2.77010600	3.87771500	-0.26681500
F	2.61139700	5.00199100	-0.96721900
C	3.56497500	3.87431200	0.86571100
F	4.17721600	4.98493500	1.26749100
C	3.71003800	2.69186600	1.57717300
F	4.47873600	2.66151800	2.66567200
C	3.05522900	1.55164500	1.13896600
C	2.35664500	-1.24701200	-0.23931800
C	3.74515200	-1.29685400	-0.31656100
F	4.46377500	-0.17413500	-0.45817600
C	4.47511900	-2.47984800	-0.26895300
F	5.80586100	-2.45929300	-0.34232400
C	3.81206700	-3.69242800	-0.15938400

F	4.49537800	-4.83453400	-0.12192300
C	2.42623800	-3.70073400	-0.10342500
F	1.76436300	-4.85712000	-0.02245600
C	1.74430100	-2.49356700	-0.14794100
F	0.39504000	-2.57902100	-0.12446500
F	3.26800300	0.44834700	1.87790000
H	-0.41507900	0.03612300	-0.09812100
C	0.34917500	-0.03570300	1.10482500
H	1.25710400	-0.18517700	1.69093900
H	-0.11159700	0.87269500	1.49901200
H	-0.26682200	-0.90926600	1.34256500

P10_P

P	2.79099300	-0.40929300	0.57217600
B	-1.96864200	0.01404400	0.44258800
C	2.13387500	-0.28330600	2.27058600
H	2.12809700	0.79993000	2.44534000
H	2.85562800	-0.70721400	2.97319700
C	0.70642900	-0.87359300	2.37435300
H	0.72792400	-1.70094900	3.09009800
H	0.41773400	-1.32003100	1.41909400
C	-0.38699500	0.13449100	2.77782400
H	-0.50875600	0.09216000	3.86803600
H	-0.06240300	1.15301400	2.55505800
C	-1.72262400	-0.16728000	2.08301400
H	-1.96829200	-1.21855200	2.30777100
H	-2.49286900	0.41603300	2.60044300
C	4.45614300	0.28561900	0.42875700
C	5.59887900	-0.49803400	0.67712700
C	6.85337000	0.09506600	0.55149800
H	7.73771100	-0.50736300	0.74211900
C	7.00214500	1.43368700	0.19861900
C	5.85327500	2.19141400	-0.02684000
H	5.95453700	3.24076700	-0.29259400
C	4.57361300	1.65277100	0.08097200
C	5.51708800	-1.94537100	1.09105600
H	4.95100900	-2.06284900	2.02095300
H	5.03602500	-2.56240600	0.32672300
H	6.51645100	-2.34514800	1.26941300
C	8.36403900	2.06254200	0.07754100
H	8.49013100	2.53077900	-0.90246800
H	8.49616400	2.84327600	0.83238200
H	9.15671600	1.32409800	0.20974900
C	3.38860500	2.55453900	-0.17370700
H	2.83637100	2.26600400	-1.07542000
H	2.66629200	2.56430300	0.64629200
H	3.72561700	3.58138100	-0.32017200
C	2.51228000	-1.95787600	-0.32425500
C	2.37135100	-3.21935300	0.27990600
C	2.03581800	-4.30807900	-0.52715400
H	1.91924400	-5.28205600	-0.05734300
C	1.83680000	-4.18532800	-1.89636900
C	2.00562900	-2.92684000	-2.47567300
H	1.86119500	-2.81135000	-3.54671200
C	2.33161900	-1.80650100	-1.72277000
C	2.55700300	-3.51351800	1.74851100
H	3.32575500	-4.28170600	1.87723100
H	2.85176600	-2.65150000	2.34043200
H	1.62607300	-3.90225600	2.17059300
C	1.42125500	-5.36348100	-2.73377800
H	0.33872900	-5.34474400	-2.89438100
H	1.90117900	-5.33920700	-3.71501900
H	1.67141600	-6.30771000	-2.24621500
C	2.43911100	-0.47696300	-2.43063200
H	2.47469700	-0.63278700	-3.50949400
H	1.55816100	0.14313300	-2.21942000
H	3.33763200	0.08229300	-2.14892400
C	-1.84799200	1.60679700	-0.07145400
C	-0.64088200	2.29437200	0.01185000
F	0.46237200	1.70039400	0.55992700
C	-0.39893100	3.58112600	-0.44523400
F	0.81770300	4.13594700	-0.31174900
C	-1.42312500	4.28147900	-1.05809600
F	-1.22994800	5.52163900	-1.50890300
C	-2.64894400	3.65220600	-1.20123500
F	-3.64705800	4.29417700	-1.81114900
C	-2.82922000	2.35354500	-0.73065000
C	-3.52384100	-0.55047600	0.29165000
C	-4.57705600	0.09931200	0.93823600
F	-4.35663100	1.28030300	1.54623300
C	-5.88044700	-0.36755900	0.99104700
F	-6.83455400	0.32057600	1.62601900
C	-6.19299200	-1.57128200	0.37420200
F	-7.43824700	-2.05110600	0.41426300
C	-5.19155800	-2.26387000	-0.28093300
F	-5.47050100	-3.42757300	-0.88030000
C	-3.89646300	-1.74940700	-0.30578400
F	-3.00714400	-2.51866900	-0.96348200

F	-4.04048800	1.84558200	-0.99284400
H	1.98877300	0.48027900	-0.12698500
C	-0.93494100	-0.81192600	-0.53627000
H	-1.26528700	-0.79209000	-1.58176400
H	0.06288500	-0.35390000	-0.52047000
H	-0.81144100	-1.86945200	-0.27074800

P11_TS

P	1.59992900	-0.65818300	-0.11550900
B	-1.46094200	-0.51315300	-0.22796000
C	0.60103800	-1.08848400	1.39716600
H	0.44102000	-0.16825000	1.96227400
C	1.27629400	-2.12278000	2.30351200
H	2.25469600	-1.76233400	2.64697800
H	1.47478700	-3.03462700	1.72360000
C	0.37678200	-2.45987000	3.49238600
H	0.24575000	-1.56067900	4.11053800
H	0.85816000	-3.21437900	4.12355500
C	-0.98854500	-2.94567400	3.00959600
H	-0.85619500	-3.88081800	2.44770200
H	-1.63246500	-3.17879000	3.86431400
C	-1.66396100	-1.90874000	2.10891100
H	-2.62236000	-2.30723400	1.76750300
H	-1.88876400	-1.01405600	2.69657800
C	-0.79074600	-1.54143900	0.89142600
H	-0.64551600	-2.49477600	0.35835100
C	3.01160300	-1.84434800	-0.28564800
C	2.79755700	-3.00286200	-1.06807600
C	3.82609300	-3.92709200	-1.23539000
H	3.63705600	-4.81612100	-1.83262100
C	5.08241200	-3.74277800	-0.66555000
C	5.28256900	-2.59827200	0.09589200
H	6.25346000	-2.43542500	0.55778900
C	4.28098300	-1.64550400	0.30285200
C	1.49190300	-3.28526200	-1.76774000
H	1.50912200	-4.27999100	-2.21672000
H	0.63593200	-3.24144200	-1.08995300
H	1.31748100	-2.56144100	-2.56993100
C	6.18709000	-4.74141400	-0.88683200
H	6.97235200	-4.63669600	-0.13520800
H	5.80636000	-5.76511200	-0.84748800
H	6.64503800	-4.59894500	-1.87100800
C	4.64954000	-0.45445600	1.15193100
H	3.89939000	-0.24866400	1.91614500
H	5.59775800	-0.64197700	1.65934900
H	4.75805000	0.45297000	0.55138800
C	2.27584800	1.05003000	-0.10493500
C	2.64325900	1.52976000	-1.38728900
C	2.99397400	2.86589100	-1.54470100
H	3.25014000	3.22748500	-2.53769500
C	2.98206800	3.76029800	-0.47483600
C	2.68711500	3.25651300	0.78534800
H	2.70133900	3.93020600	1.64024500
C	2.33774700	1.91855200	1.00307300
C	2.64125100	0.64106400	-2.61005100
H	3.06908200	1.17304600	-3.46177100
H	3.22170000	-0.27423300	-2.45652400
H	1.61872100	0.35601000	-2.87756000
C	3.21778200	5.22933900	-0.69435100
H	2.28665700	5.69862400	-1.02881600
H	3.53159900	5.72647000	0.22614000
H	3.97603500	5.40306300	-1.46178400
C	2.07125400	1.54303200	2.44609500
H	1.04625300	1.78804100	2.74824600
H	2.23587900	0.48831900	2.66122100
H	2.73890100	2.11872400	3.09217600
C	-3.08262800	-0.72165100	-0.34414900
C	-4.03248900	0.29850300	-0.33423800
F	-3.68188300	1.58576100	-0.20223300
C	-5.40194900	0.07750900	-0.45948500
F	-6.25217400	1.10321700	-0.43888600
C	-5.88106500	-1.21209600	-0.61334800
F	-7.18531800	-1.43934600	-0.73978800
C	-4.97861200	-2.26621200	-0.63284800
F	-5.41591300	-3.51684200	-0.77809300
C	-3.62479600	-1.99915100	-0.49975900
F	-2.81576200	-3.07671000	-0.51718600
C	-1.04201600	1.07760100	-0.10035400
C	-1.11829300	1.74055200	1.12263700
F	-1.50868400	1.07617800	2.22530600
C	-0.78200000	3.07002900	1.32766600
F	-0.80282500	3.59075800	2.55686700
C	-0.33698000	3.82953700	0.25939000
F	0.07273100	5.08362200	0.43748800
C	-0.26409100	3.23958100	-0.98877800
F	0.19284200	3.94394700	-2.02534800
C	-0.61571700	1.90422500	-1.14141000
F	-0.45178300	1.43650800	-2.39432900

H	0.13364200	-0.79764500	-1.15239300
C	-0.99833600	-1.09742700	-1.93783100
H	-1.29534900	-2.14472100	-1.91099700
H	-0.15196600	-1.02375800	-2.63470300
H	-1.75065100	-0.48518600	-2.43236700

P11_P

P	1.69337200	-0.65193400	-0.05567800
B	-1.65643700	-0.61416500	-0.57955400
C	0.43210800	-1.22006800	1.17070600
H	0.23336600	-0.33349300	1.77470700
C	1.07068800	-2.29873100	2.06777400
H	2.06115400	-1.99115800	2.42993500
H	1.23379500	-3.20530500	1.46965800
C	0.15164800	-2.61344600	3.24433200
H	0.05195100	-1.71534100	3.87037100
H	0.60184200	-3.39211200	3.86887600
C	-1.22063600	-3.03525400	2.73285000
H	-1.12114200	-3.98262400	2.18539000
H	-1.89713800	-3.22530800	3.57294500
C	-1.82150000	-1.97918100	1.80329800
H	-2.78757100	-2.34482500	1.46001800
H	-2.01758900	-1.06177300	2.37057000
C	-0.94329800	-1.64457900	0.57239900
H	-0.78846600	-2.60732900	0.05788400
C	3.04349700	-1.86562900	-0.27662100
C	2.82041100	-2.92530200	-1.18335300
C	3.81969000	-3.88043100	-1.36385200
H	3.63895800	-4.69677000	-2.05841000
C	5.02946100	-3.82101600	-0.67968300
C	5.22552800	-2.76911600	0.21150000
H	6.16063400	-2.71100200	0.76313800
C	4.26328600	-1.78453100	0.43097500
C	1.55033800	-3.06698000	-1.98341300
H	1.51724200	-4.04394200	-2.46783800
H	0.64763800	-2.96962700	-1.37450400
H	1.48952800	-2.30744800	-2.77141800
C	6.10420100	-4.85004900	-0.90607300
H	6.83736500	-4.48404300	-1.63202100
H	6.63976200	-5.07139200	0.02005600
H	5.68634500	-5.78071800	-1.29511700
C	4.60790200	-0.69111500	1.41262400
H	3.81154300	-0.51356300	2.13832900
H	5.50255700	-0.96902000	1.97233600
H	4.80415300	0.25813800	0.90624300
C	2.35664600	1.02909000	0.15535100
C	2.86407200	1.62741300	-1.02214800
C	3.35537400	2.92921900	-0.95731700
H	3.72703300	3.39087100	-1.86830200
C	3.36219900	3.65603700	0.23024000
C	2.88133400	3.03571600	1.37982900
H	2.88280900	3.58580800	2.31756500
C	2.37315700	1.73660600	1.37595400
C	2.85437100	0.94196900	-2.36830900
H	3.46784100	1.50461500	-3.07324500
H	3.25170000	-0.07719700	-2.32341600
H	1.83887500	0.90213800	-2.77892400
C	3.82767000	5.08501400	0.25965100
H	4.23227600	5.35279900	1.23814900
H	4.59173700	5.27066300	-0.49832600
H	2.97920700	5.74489000	0.05385400
C	1.85448700	1.22237200	2.70006600
H	0.78876400	1.45004700	2.81496100
H	1.98697600	0.14864200	2.84125300
H	2.38049700	1.72651500	3.51328900
C	-3.30489400	-0.56455800	-0.37218700
C	-4.10711900	0.56046800	-0.19883900
F	-3.58063900	1.79404900	-0.08636800
C	-5.49823500	0.52511500	-0.13400200
F	-6.19531200	1.65145400	0.03390600
C	-6.16300200	-0.68283900	-0.24741200
F	-7.49349000	-0.73538600	-0.18337700
C	-5.41761800	-1.83959500	-0.42078300
F	-6.03332800	-3.02054200	-0.51971300
C	-4.03521500	-1.74985500	-0.48345100
F	-3.39028500	-2.92768900	-0.62262400
C	-1.02985800	0.93279000	-0.48288400
C	-1.00295000	1.64006500	0.71930200
F	-1.50758400	1.07673100	1.83648200
C	-0.48260600	2.91260600	0.88673100
F	-0.43048400	3.47848600	2.09727800
C	0.05842000	3.57380300	-0.20546400
F	0.61831100	4.77622800	-0.06147400
C	0.04748600	2.93924800	-1.43238100
F	0.61921600	3.52141000	-2.49048100
C	-0.49553400	1.66118200	-1.54477900
F	-0.41328900	1.14847300	-2.78914400
H	1.06398800	-0.60628200	-1.29872100

C	-1.44998300	-1.23703500	-2.08810200
H	-1.76072100	-2.28671200	-2.10461300
H	-0.42581700	-1.20501300	-2.47652100
H	-2.05668900	-0.70866300	-2.83077000

P12_TS

P	-2.10074300	-1.10718300	0.41952700
C	-1.77058800	-1.40419400	2.19851400
H	-2.60327900	-1.03532600	2.80753200
H	-1.74829400	-2.49557000	2.30190100
C	-0.41137100	-0.82383100	2.60566400
H	-0.45176800	0.26962600	2.60583100
H	-0.23730400	-1.11497800	3.64806300
C	0.75352400	-1.31986300	1.73158000
H	0.67296700	-2.41327800	1.65232100
H	1.66727000	-1.15144700	2.31147200
B	1.05097200	-0.68200800	0.23234000
C	-3.30784600	-2.37192200	-0.08976600
C	-2.80499300	-3.62512800	-0.45857600
H	-1.73094100	-3.80016700	-0.46603900
C	-3.67326000	-4.65300700	-0.81198600
H	-3.27624800	-5.62138300	-1.09794100
C	-5.04816700	-4.43299800	-0.80683400
H	-5.72566500	-5.23243200	-1.08865600
C	-5.55430300	-3.18762300	-0.44236400
H	-6.62582500	-3.01666300	-0.43558400
C	-4.68970100	-2.15865300	-0.08205700
H	-5.08626700	-1.18837200	0.20208100
C	-2.93667400	0.49852800	0.26725900
C	-2.87203300	1.44384900	1.29402100
H	-2.39816900	1.19930100	2.23903500
C	-3.39547800	2.72017000	1.10099400
H	-3.32527700	3.45476100	1.89679900
C	-3.98592300	3.05803300	-0.11267000
H	-4.37871900	4.05765500	-0.26561600
C	-4.06228500	2.11414600	-1.13541900
H	-4.51216100	2.37738100	-2.08679700
C	-3.53431400	0.84185800	-0.95246200
H	-3.57317400	0.11792600	-1.76205200
C	0.44933700	0.82446600	0.02607100
C	0.73548600	1.80012600	0.97905400
F	1.49146600	1.47885900	2.04235500
C	0.29238600	3.11228700	0.90703100
F	0.57817100	3.98809400	1.87080800
C	-0.45488700	3.51389900	-0.19213800
F	-0.92030300	4.75842700	-0.27311400
C	-0.73958600	2.59510500	-1.18714800
F	-1.46564300	2.96172000	-2.24467900
C	-0.29212400	1.28627200	-1.05519900
F	-0.68985900	0.44315500	-2.03053900
C	2.67553500	-0.71388300	-0.04215600
C	3.41220600	-1.86599800	0.23769300
F	2.79324300	-2.96515500	0.70808900
C	4.78073100	-1.98649800	0.04613800
F	5.41302300	-3.12482500	0.33382100
C	5.49116500	-0.90699600	-0.45741200
F	6.80457400	-0.99574600	-0.64958500
C	4.81148100	0.25982600	-0.76344300
F	5.47647300	1.30383400	-1.25769400
C	3.43573700	0.33266500	-0.56198300
F	2.87630500	1.49979900	-0.91647500
H	-0.56175700	-1.31969900	-0.36838100
C	0.55273400	-1.79664900	-1.14045900
H	-0.36764000	-2.11413900	-1.65231300
H	1.05101000	-2.71359400	-0.82028000
H	1.10974900	-1.29919900	-1.93456400

P12_P

P	-2.37152000	-1.03464900	0.26221000
C	-1.62297600	-1.75263700	1.75045500
H	-2.45003700	-1.76193200	2.47345300
H	-1.43328200	-2.79906500	1.47821300
C	-0.34846200	-1.11523900	2.33655100
H	-0.45213900	-0.02734700	2.37307800
H	-0.32862200	-1.44735700	3.38211100
C	0.96101700	-1.50881700	1.64480900
H	1.02048400	-2.60563500	1.65411900
H	1.75744300	-1.17274800	2.31805700
B	1.28494200	-0.97745900	0.09878100
C	-3.75904200	-2.08294000	-0.21654000
C	-3.51128700	-3.17587100	-1.05313800
H	-2.51296600	-3.35112300	-1.44778000
C	-4.55343300	-4.03509800	-1.38464700
H	-4.36666500	-4.88112500	-2.03708400
C	-5.83254000	-3.80232100	-0.88468100
H	-6.64453400	-4.47232300	-1.14741700
C	-6.07787700	-2.71087300	-0.05411400
H	-7.07692700	-2.53088900	0.32805400

C	-5.04243000	-1.84568300	0.28249600
H	-5.23277900	-0.98430700	0.91739500
C	-2.95878300	0.64846800	0.48692700
C	-2.83429000	1.31387200	1.70773500
H	-2.41000100	0.81344000	2.57180600
C	-3.24546800	2.64149000	1.80934300
H	-3.13322400	3.16586000	2.75186000
C	-3.77589500	3.29422600	0.70157400
H	-4.07279600	4.33469800	0.77888500
C	-3.91046100	2.62470000	-0.51487200
H	-4.30486800	3.13990300	-1.38358900
C	-3.50510500	1.30188500	-0.62553600
H	-3.57539100	0.79312600	-1.58420600
C	0.56765400	0.50686800	-0.14293300
C	0.71926900	1.51566400	0.81142400
F	1.42987100	1.27687100	1.92645400
C	0.16365800	2.78336300	0.71341600
F	0.30137900	3.67624200	1.69589500
C	-0.56067800	3.11980100	-0.42144200
F	-1.14062000	4.31670400	-0.52702400
C	-0.70709900	2.17831600	-1.42388300
F	-1.46314500	2.46028300	-2.49197400
C	-0.14963100	0.91456100	-1.26216500
F	-0.43749800	0.04231500	-2.25730500
C	2.92576500	-0.77198700	-0.05221100
C	3.78950600	-1.77592300	0.38764600
F	3.29275200	-2.89448000	0.95060700
C	5.17260300	-1.72767800	0.28058600
F	5.93120300	-2.73149500	0.73004400
C	5.76918800	-0.62162200	-0.30582200
F	7.09581500	-0.54721800	-0.42126800
C	4.96297400	0.40071600	-0.77546100
F	5.51774000	1.47085200	-1.35205000
C	3.58039400	0.30360600	-0.64771300
F	2.89736500	1.34469800	-1.16076700
H	-1.49949800	-1.07827700	-0.82376000
C	0.88644100	-2.13559400	-0.99719100
H	-0.14611100	-2.51769700	-0.93130200
H	1.51743200	-3.00923800	-0.79721900
H	1.05074900	-1.84541300	-2.03890600

P13_TS

P	-2.55905500	-0.28983200	-0.23849800
B	0.84694500	0.10598200	-0.24409100
C	-2.70687200	-1.37476200	-1.71308200
H	-2.35041000	-2.36290500	-1.40240000
H	-3.76515900	-1.46821100	-1.97931400
C	-1.86505000	-0.86207800	-2.88488900
H	-2.05547400	-1.52554900	-3.73572900
H	-2.21520800	0.13461400	-3.18314900
C	-0.35966700	-0.82570600	-2.59534900
H	0.14953800	-0.76324300	-3.56241700
H	-0.07073400	-1.79821000	-2.18271300
C	0.12702000	0.35118700	-1.70770500
H	-0.65863600	1.11420800	-1.67258200
H	0.93250300	0.85147600	-2.26100300
C	-3.57111800	-1.09264000	1.04923000
C	-4.60696300	-0.42925800	1.71408500
H	-4.86613400	0.58864800	1.44027100
C	-5.30933800	-1.07294100	2.72974500
H	-6.11318200	-0.55168600	3.23934300
C	-4.98273500	-2.37612000	3.09202300
H	-5.53130100	-2.87428300	3.88470500
C	-3.94403400	-3.03786000	2.44086700
H	-3.67889400	-4.05093800	2.72483700
C	-3.23629900	-2.40017500	1.42822000
H	-2.41339200	-2.91762900	0.94347500
C	-3.40708800	1.25829200	-0.67758300
C	-4.70448000	1.26193200	-1.20440500
H	-5.24078700	0.32571100	-1.33816400
C	-5.31631300	2.46356100	-1.54151900
H	-6.32212800	2.46258500	-1.94868300
C	-4.63663800	3.66766100	-1.35770200
H	-5.11536700	4.60404200	-1.62571200
C	-3.34747800	3.67073300	-0.83582100
H	-2.81371500	4.60520500	-0.69926000
C	-2.73205300	2.46842300	-0.49485300
H	-1.71550200	2.47378100	-0.11153900
C	1.65896700	-1.30919700	-0.31949200
C	1.00749100	-2.52096900	-0.12673500
F	-0.32813200	-2.53361800	0.12303800
C	1.61230200	-3.76552800	-0.22016200
F	0.91233100	-4.88370700	-0.02072700
C	2.95934600	-3.83153400	-0.54246100
F	3.57209100	-5.00883000	-0.63820500
C	3.65501300	-2.65476800	-0.78018800
F	4.94496100	-2.70675200	-1.10772600
C	2.99768700	-1.43428800	-0.67816200

F	3.72928600	-0.34582300	-0.94965700
C	1.74887500	1.41452800	0.18038700
C	2.82627800	1.31550100	1.06334400
F	3.15189600	0.12345100	1.59175500
C	3.60239700	2.38600000	1.48068600
F	4.61794900	2.20449700	2.32373700
C	3.30151400	3.66106700	1.02379900
F	4.02581500	4.70654400	1.41373800
C	2.22336800	3.82835600	0.17239900
F	1.89583800	5.04790200	-0.25793900
C	1.47603100	2.72096300	-0.21788500
F	0.42211100	3.00756300	-1.00804300
H	-0.91717900	-0.10712900	0.30090400
C	-0.04101700	-0.03007400	1.40573300
H	0.93373300	-0.18743700	1.87243100
H	-0.65385500	-0.85528000	1.78593900
H	-0.43983600	0.91691500	1.78380700

P13_P

P	2.40995700	-0.73543400	-0.23013000
B	-1.32304900	-0.67872400	0.34897000
C	1.90760500	-0.34510400	-1.93066000
H	1.40667500	0.63087300	-1.89572100
H	2.85746200	-0.22068000	-2.46592100
C	1.03878600	-1.40416400	-2.63236100
H	1.15403100	-1.22316800	-3.70658600
H	1.45631900	-2.40143200	-2.44042200
C	-0.44624000	-1.37325400	-2.26361000
H	-0.94143700	-2.06868000	-2.95078400
H	-0.84297300	-0.38807200	-2.51788800
C	-0.76225300	-1.76416100	-0.80182800
H	0.13045900	-2.27337300	-0.39733000
H	-1.49215100	-2.57455900	-0.82839200
C	3.48474700	0.56328700	0.40089000
C	3.78642800	0.55783600	1.76660100
H	3.37788600	-0.21132300	2.41629800
C	4.59472500	1.55632600	2.29382300
H	4.82488400	1.55888400	3.35351500
C	5.09093200	2.56088800	1.46607100
H	5.71263800	3.34583900	1.88384200
C	4.78092300	2.57263400	0.10938600
H	5.15398800	3.36475200	-0.53041000
C	3.97809900	1.57284000	-0.42972900
H	3.72219900	1.60297500	-1.48425900
C	3.25329300	-2.32267400	-0.16231200
C	4.51007600	-2.47852000	-0.75684500
H	4.99813600	-1.63584300	-1.24085700
C	5.14108700	-3.71487600	-0.70839200
H	6.11607400	-3.84258200	-1.16610900
C	4.52303100	-4.78749700	-0.06536900
H	5.02032400	-5.75122500	-0.02794200
C	3.27650900	-4.62861800	0.53302200
H	2.80016700	-5.46304300	1.03581000
C	2.63431900	-3.39461500	0.48750800
H	1.65908800	-3.26727300	0.95107000
C	-0.63227800	0.82402200	0.10218500
C	0.35094200	1.40305400	0.90031000
F	0.80688900	0.78403600	2.01478000
C	0.99809100	2.60592100	0.61948700
F	1.96479000	3.05980600	1.42223800
C	0.65514700	3.31455200	-0.51681500
F	1.27511900	4.45521500	-0.82051900
C	-0.36046700	2.82057000	-1.32811300
F	-0.73907200	3.51298800	-2.40310700
C	-0.97546700	1.62142500	-0.99628700
F	-1.98181700	1.25259500	-1.80452900
C	-2.98480600	-0.56367000	0.31589400
C	-3.73660600	0.60779100	0.41303000
F	-3.14674800	1.81429500	0.50082700
C	-5.12662900	0.65453000	0.44599600
F	-5.76298000	1.82665600	0.52234800
C	-5.85453600	-0.52206400	0.41728500
F	-7.18756800	-0.50206000	0.45535400
C	-5.16649400	-1.72269600	0.36397400
F	-5.84045200	-2.87668500	0.36416700
C	-3.77766700	-1.71515700	0.32971100
F	-3.21855600	-2.94305700	0.33722900
H	1.33252200	-0.85958400	0.64313100
C	-0.97870000	-1.31167500	1.82529700
H	-1.29428100	-0.67050100	2.65547500
H	0.08401900	-1.53889000	2.00535400
H	-1.51220900	-2.26364000	1.92724700
S			

P14_TS

P	-2.61356400	-1.06634500	0.02237000
B	0.65518200	-0.39683700	0.01964200
C	-2.26562100	-0.16538300	1.58276500
H	-2.11768900	0.86818600	1.25508200

H	-3.12702000	-0.16970700	2.26012700
C	-0.98069700	-0.64722800	2.28362700
H	-1.24484800	-1.43613900	2.99981600
H	-0.62001200	0.18969400	2.89146800
C	0.17663500	-1.15511200	1.39575000
H	0.01148200	-2.20331500	1.14776500
H	1.05514400	-1.17600400	2.05081900
C	-3.17305000	-2.82587900	0.45248600
C	-4.14322600	-2.86541500	1.64040700
H	-4.37234200	-3.91205200	1.87202300
H	-5.08663400	-2.35534400	1.44708700
H	-3.68806600	-2.42609800	2.53370300
C	-3.79017700	-3.48852600	-0.78896200
H	-3.09024900	-3.48084400	-1.63175800
H	-4.72223400	-3.02342800	-1.11137700
H	-4.00998700	-4.53541200	-0.55288900
C	-1.94617200	-3.67095100	0.83547900
H	-1.51092200	-3.35350300	1.78460200
H	-1.16770400	-3.64929300	0.06678000
H	-2.27238600	-4.71033200	0.95406000
C	-4.03450400	-0.13577600	-0.82396600
C	-3.83124600	1.37889400	-0.65853900
H	-2.88724100	1.71767300	-1.08126200
H	-3.90323200	1.69934700	0.38383300
H	-4.62899800	1.88648000	-1.21200000
C	-5.42439400	-0.45549700	-0.25845400
H	-6.15622700	0.18387000	-0.76531900
H	-5.48216600	-0.23348500	0.81186900
H	-5.73466000	-1.48892500	-0.41616500
C	-3.96820700	-0.44554300	-2.32834500
H	-4.08365700	-1.50767600	-2.5533430
H	-3.01694500	-0.10166200	-2.74571700
H	-4.77300800	0.09274000	-2.84157400
C	0.27137100	1.18142600	0.02428800
C	-0.45612200	1.87862000	-0.92653600
F	-0.93868100	1.25731700	-2.02635300
C	-0.79644400	3.22310500	-0.80970300
F	-1.55579400	3.80594200	-1.73956500
C	-0.38092400	3.93811900	0.29971600
F	-0.70256300	5.22136800	0.43704300
C	0.37734800	3.29492100	1.27146400
F	0.79997400	3.97317200	2.33666400
C	0.68242900	1.95299400	1.11183500
F	1.43761400	1.38930400	2.06730000
C	2.26898400	-0.66122200	-0.16495500
C	2.78811400	-1.95110500	-0.04677300
F	1.96548000	-2.99536300	0.17341500
C	4.13357200	-2.26617000	-0.16668600
F	4.55250400	-3.52593700	-0.04330800
C	5.04668000	-1.25332700	-0.42159600
F	6.34266500	-1.52970600	-0.54103900
C	4.58704400	0.04549300	-0.56009100
F	5.44798700	1.02988500	-0.81703400
C	3.22619800	0.31328800	-0.43853600
F	2.88177800	1.59749400	-0.61693400
H	-1.00680900	-0.98718100	-0.75539700
C	0.12134400	-1.19862300	-1.57578900
H	0.50199600	-2.21629500	-1.47564500
H	-0.75692500	-1.24562200	-2.23274400
H	0.80660300	-0.58420200	-2.16262000

P14_P

P	-3.28243400	-0.76893000	-0.03264400
B	0.64433200	-0.25521800	-0.21062800
C	-2.33111700	0.60142500	0.69326800
H	-1.86501200	1.09186900	-0.16637500
H	-3.07269300	1.29376100	1.11273600
C	-1.24208400	0.22500000	1.70908100
H	-1.70980900	-0.15393300	2.62766700
H	-0.77176800	1.17456200	1.98949700
C	-0.18153100	-0.73302100	1.15856800
H	-0.63589900	-1.71591700	0.97657400
H	0.53971300	-0.89830400	1.96885400
C	-4.10883300	-1.81617600	1.26433100
C	-4.72836500	-0.91381400	2.34132200
H	-5.18378900	-1.55230900	3.10500400
H	-5.51035300	-0.26104300	1.94494900
H	-3.97247100	-0.29668000	2.83447300
C	-5.17813700	-2.72487000	0.64172700
H	-4.76274200	-3.36203800	-0.14504600
H	-6.02656800	-2.16977000	0.23690400
H	-5.56073100	-3.38236700	1.42880600
C	-3.03577500	-2.72163200	1.89899700
H	-2.24579300	-2.16221800	2.40003900
H	-2.57094800	-3.38065800	1.15960800
H	-3.53201200	-3.35102300	2.64508000
C	-4.40617900	-0.07161700	-1.34681000
C	-3.63890200	1.02120500	-2.11598000

H	-2.70684100	0.65730300	-2.55466600
H	-3.40582200	1.88954800	-1.49615300
H	-4.28452900	1.35944200	-2.93293900
C	-5.65403600	0.56119000	-0.71520400
H	-6.23514200	1.03729000	-1.51117500
H	-5.39394400	1.34023800	0.00864800
H	-6.30155800	-0.16994600	-0.22665300
C	-4.78736300	-1.18277300	-2.33651200
H	-5.38038800	-1.97754700	-1.88185600
H	-3.89859400	-1.62518000	-2.79657500
H	-5.38786500	-0.73863200	-3.13676600
C	0.87809100	1.37743400	-0.06156200
C	0.23124200	2.36967900	-0.78644900
F	-0.66238800	2.05872900	-1.75989700
C	0.38207900	3.73604500	-0.56230700
F	-0.29229900	4.62486300	-1.29955000
C	1.22864500	4.16892500	0.44216800
F	1.38855800	5.47062100	0.68198000
C	1.89453800	3.22361900	1.21248700
F	2.70163500	3.62582300	2.19623100
C	1.70000400	1.87604000	0.95105200
F	2.35793400	1.02009200	1.74915900
C	2.08404800	-1.07781700	-0.25380700
C	2.10261200	-2.45977600	-0.07359100
F	0.95567600	-3.13332800	0.16782800
C	3.24256100	-3.24991600	-0.14030900
F	3.17148600	-4.57145600	0.05016500
C	4.46351500	-2.65600600	-0.41691600
F	5.57418700	-3.39146800	-0.48789800
C	4.50648000	-1.28748800	-0.62683800
F	5.67069100	-0.69672000	-0.90861000
C	3.33451700	-0.54169900	-0.55185000
F	3.48325700	0.77010700	-0.80205200
H	-2.40439900	-1.62772300	-0.70459200
C	-0.09739600	-0.68618500	-1.61112200
H	-0.20859800	-1.77949400	-1.64534500
H	-1.08697700	-0.24946500	-1.78165500
H	0.50007100	-0.40172900	-2.48493400

P15_TS

P	-1.17410200	-0.62082500	-0.43031600
B	1.50877000	-0.58955600	-0.51440700
C	0.31022600	-1.20974400	0.45404800
H	0.22672900	-2.27688200	0.18660000
C	0.47261600	-1.10024000	1.96788700
H	0.52339300	-0.05172100	2.26566500
H	1.44550700	-1.53490700	2.21555300
C	-0.62085100	-1.81605800	2.75490800
H	-1.59384200	-1.33813400	2.60885300
H	-0.40595900	-1.79892600	3.82624700
H	-0.69951300	-2.86420100	2.44594000
C	-1.76459700	1.06979800	-0.11324700
C	-1.70316900	1.75017800	1.10035400
F	-1.37634500	1.11644400	2.22272900
C	-1.98238300	3.10663100	1.19167600
F	-1.90124600	3.73324700	2.35829600
C	-2.36201000	3.80721500	0.05278700
F	-2.63147300	5.09875100	0.13345900
C	-2.47692400	3.14925300	-1.16532000
F	-2.84917100	3.81560100	-2.24835500
C	-2.18439600	1.79569100	-1.23098600
F	-2.29205600	1.19217100	-2.41244400
C	-2.60308000	-1.72187400	-0.20535200
C	-3.62006100	-1.52430200	0.72650300
F	-3.57786300	-0.48608800	1.56140600
C	-4.68880400	-2.40207400	0.83765000
F	-5.64313800	-2.19584900	1.73590500
C	-4.75236900	-3.50998600	0.00022800
F	-5.76656300	-4.35380900	0.10003000
C	-3.74992700	-3.74035300	-0.93405800
F	-3.81272800	-4.80037400	-1.72897200
C	-2.69523300	-2.84456100	-1.02616400
F	-1.74133300	-3.08408300	-1.92736800
C	1.55145200	1.03714700	-0.33578500
C	2.11725700	1.59005700	0.81342700
F	2.73982900	0.79837700	1.69847500
C	2.06814300	2.94228400	1.12823600
F	2.61639300	3.40105900	2.24979000
C	1.43555400	3.82104700	0.25881400
F	1.34814800	5.11407000	0.54992700
C	0.88203000	3.32713000	-0.91158300
F	0.24414800	4.14871300	-1.74871100
C	0.96790100	1.96801500	-1.18699500
F	0.36381300	1.57296900	-2.32677800
C	2.96590200	-1.27650300	-0.27755500
C	4.12026500	-0.65580600	-0.75577800
F	4.02426500	0.50333100	-1.42789700
C	5.39888700	-1.17435600	-0.61120800

F	6.45666100	-0.52560700	-1.09272200
C	5.56681700	-2.39130200	0.03472000
F	6.78043700	-2.91120300	0.18440800
C	4.45203400	-3.06401000	0.50807000
F	4.59254500	-4.24201400	1.11327500
C	3.18944900	-2.50709700	0.33419100
F	2.16803100	-3.25061800	0.79819200
H	-0.03309000	-0.70931600	-1.67500500
C	1.20504600	-1.02880700	-2.24952300
H	1.35495400	-2.10768200	-2.30627300
H	2.08845200	-0.48356300	-2.59405500
H	0.48122500	-0.76453300	-3.04022800

P15_P

P	1.16556300	-0.67506800	0.48490800
B	-1.65524400	-0.51004100	0.83563600
C	-0.43061400	-1.28526700	-0.07663600
H	-0.34583000	-2.30600500	0.33319000
C	-0.64835200	-1.38277200	-1.59337000
H	-0.84149200	-0.39190100	-2.00388300
H	-1.58163200	-1.93999700	-1.70661600
C	0.45033000	-2.07915000	-2.39201300
H	1.35395100	-1.46561000	-2.46106300
H	0.11640500	-2.26665100	-3.41536500
H	0.71647100	-3.04466500	-1.94704000
C	1.72880300	1.00781400	0.14380000
C	1.49839000	1.67245900	-1.05650100
F	0.94576300	1.03007700	-2.08032300
C	1.83205300	3.00778900	-1.22049000
F	1.56468400	3.63107700	-2.35760600
C	2.42065600	3.69943000	-0.16698900
F	2.73043300	4.97353400	-0.31002800
C	2.68749100	3.05353900	1.03500700
F	3.24778100	3.71491300	2.03428500
C	2.33808200	1.72025200	1.17883100
F	2.59442100	1.12182100	2.33967800
C	2.57243600	-1.77348500	0.14339800
C	3.49346800	-1.57659100	-0.88238300
F	3.37901100	-0.52996700	-1.69936200
C	4.53019300	-2.47073800	-1.10491300
F	5.39596500	-2.26874600	-2.08708200
C	4.65625700	-3.58833800	-0.28604400
F	5.64065600	-4.44431200	-0.49247900
C	3.74742700	-3.81500100	0.74135000
F	3.86899900	-4.88271300	1.51421700
C	2.71965900	-2.90592500	0.94099900
F	1.84579200	-3.13267700	1.92043500
C	-1.50475300	1.11004600	0.53306300
C	-1.94420400	1.70302300	-0.65241600
F	-2.61177400	0.97685700	-1.56138900
C	-1.72691400	3.03184500	-0.99449300
F	-2.15535900	3.51786200	-2.15728000
C	-1.04758200	3.85766200	-0.10921500
F	-0.76210200	5.11575400	-0.43545500
C	-0.60667000	3.33094900	1.09255600
F	0.11401500	4.08983400	1.92650200
C	-0.83633200	1.98978900	1.37800100
F	-0.23674500	1.54459500	2.50304000
C	-3.11465600	-1.10704700	0.35453200
C	-4.28625800	-0.35229900	0.33551200
F	-4.25625700	0.96492100	0.59251200
C	-5.54500300	-0.87717000	0.06826200
F	-6.61971000	-0.08848300	0.05783700
C	-5.68468200	-2.23437100	-0.17597400
F	-6.88176400	-2.75662500	-0.43258800
C	-4.55698000	-3.03966200	-0.14512900
F	-4.66490100	-4.35117500	-0.36433500
C	-3.32284400	-2.46555100	0.12852100
F	-2.28178200	-3.32801700	0.16405200
H	1.12724400	-0.68449900	1.87648400
C	-1.55009300	-0.92771500	2.41097100
H	-1.73639300	-2.00392300	2.51288400
H	-2.33247700	-0.41817100	2.98469700
H	-0.61343800	-0.71021300	2.93384300

P16_TS

C	0.24515400	-1.78143400	2.73332100
H	1.26624600	-1.49981500	2.47824300
H	0.20569800	-2.85718000	2.93647200
H	-0.01457900	-1.27241800	3.66714200
C	-0.74315600	-1.40989200	1.67504300
H	-1.79127200	-1.53612400	1.95642300
C	-0.52277400	-0.92789400	0.44795600
P	1.04285200	-0.69692900	-0.40695600
C	2.31017300	-1.95908300	-0.08415700
C	3.30096900	-1.85154800	0.88962600
F	3.32436800	-0.80363100	1.71442200
C	4.26702600	-2.83181200	1.06103500

F	5.19645500	-2.71037000	1.99990000
C	4.25222100	-3.95333500	0.23902300
F	5.16862900	-4.89479500	0.39432800
C	3.27307900	-4.09483100	-0.73715700
F	3.26260500	-5.16760700	-1.51690200
C	2.31990500	-3.09760300	-0.88843600
F	1.39264200	-3.24699500	-1.83271400
C	1.80914800	0.91801700	-0.08229000
C	2.46264100	1.54858900	-1.14150900
F	2.66304600	0.88625800	-2.28035800
C	2.89991900	2.86163600	-1.05751700
F	3.50812700	3.44100600	-2.08287300
C	2.67068500	3.57666100	0.11261400
F	3.05883500	4.83728200	0.20350500
C	2.02840000	2.97250000	1.18694900
F	1.78441200	3.66864500	2.28975900
C	1.61169700	1.65242000	1.08367200
F	0.99237000	1.11471200	2.13027300
B	-1.62536200	-0.36836400	-0.62857100
C	-3.12195200	-0.92649000	-0.35207900
C	-4.27328800	-0.14586600	-0.27678100
F	-4.21845300	1.18031500	-0.45347100
C	-5.53697700	-0.66989800	-0.02877400
F	-6.60006400	0.12894100	0.03856800
C	-5.69023800	-2.03713800	0.14198100
F	-6.89152000	-2.55349100	0.37933200
C	-4.57755600	-2.86164200	0.05584900
F	-4.71055000	-4.17881000	0.20492000
C	-3.33446000	-2.29574300	-0.19156800
F	-2.30273000	-3.15092300	-0.29020900
C	-1.43540500	1.25606800	-0.54730300
C	-0.72879500	2.05251200	-1.43761000
F	-0.22198000	1.53491200	-2.57564900
C	-0.38475000	3.37619100	-1.18307400
F	0.36942200	4.05821900	-2.04895900
C	-0.75500200	3.95804900	0.01671600
F	-0.36873300	5.19699500	0.30451800
C	-1.47343000	3.20985200	0.94276900
F	-1.82082700	3.75359300	2.10563900
C	-1.78959300	1.89350600	0.64416500
F	-2.44508900	1.20458700	1.58591300
H	-0.04731900	-0.71728900	-1.70518600
C	-1.29309700	-0.97314500	-2.29619800
H	-0.51323200	-0.84044600	-3.06703400
H	-1.52970500	-2.03650700	-2.26936700
H	-2.10375500	-0.38593800	-2.73655100

P16_P

C	-0.93872900	-1.96194100	-2.31715500
H	-1.65014500	-1.13599300	-2.38639200
H	-1.48150300	-2.87280500	-2.03779100
H	-0.53010100	-2.12464500	-3.31580000
C	0.17805400	-1.67125000	-1.35216600
H	1.16659700	-2.00414300	-1.66982400
C	0.15700800	-1.04147700	-0.16729500
P	-1.36313100	-0.49238800	0.55220500
C	-2.92945200	-1.28440600	0.09317600
C	-3.79194500	-0.81339800	-0.89329900
F	-3.47756600	0.27696100	-1.58900100
C	-4.97008700	-1.47435900	-1.20370600
F	-5.77534200	-1.01241400	-2.14858200
C	-5.30104600	-2.63683300	-0.51395800
F	-6.42178500	-3.27237400	-0.80282000
C	-4.45727700	-3.13527800	0.47165500
F	-4.77469700	-4.24475600	1.12005000
C	-3.28421500	-2.45415200	0.76376700
F	-2.48328300	-2.94230100	1.70633300
C	-1.52286200	1.29878600	0.47430100
C	-1.66411300	2.04923800	1.63941800
F	-1.88665200	1.44087900	2.80117400
C	-1.54868600	3.43202700	1.62577600
F	-1.67063700	4.13087300	2.74246100
C	-1.29549900	4.07815000	0.42252900
F	-1.16433000	5.38909600	0.39681600
C	-1.15433200	3.35187100	-0.75769100
F	-0.86800200	3.98034700	-1.88859000
C	-1.25522200	1.97317100	-0.71893300
F	-1.06433600	1.28517900	-1.84007900
B	1.47573700	-0.58407900	0.77897000
C	2.68774400	-1.62915800	0.42081000
C	3.98991200	-1.28312000	0.07078200
F	4.35210500	0.00149800	-0.06084400
C	4.99952400	-2.21327800	-0.15645200
F	6.22653900	-1.81294200	-0.49071700
C	4.72803100	-3.56581900	-0.02788500
F	5.68193100	-4.46998100	-0.24056100
C	3.44815000	-3.96582500	0.32772500
F	3.16693800	-5.26372100	0.45877500

C	2.47360700	-3.00196900	0.54720700
F	1.25835600	-3.46688900	0.89494700
C	1.76850700	0.96997900	0.29142900
C	1.58185100	2.11434700	1.06168900
F	1.18289200	2.04436600	2.34721200
C	1.71161000	3.41048700	0.56951400
F	1.42055800	4.45757800	1.35129500
C	2.03522900	3.61491800	-0.76079100
F	2.11773500	4.84701700	-1.25488600
C	2.24451800	2.50888900	-1.57435700
F	2.56205800	2.67869000	-2.85674300
C	2.10663900	1.23700700	-1.03671900
F	2.32543400	0.21891900	-1.88258300
H	-1.32434500	-0.75870800	1.91698200
C	1.18427400	-0.72179600	2.38009200
H	0.41400500	-0.06434700	2.79743300
H	0.91970800	-1.75592900	2.63137800
H	2.09564300	-0.48028900	2.93637500

P18_TS

P	2.14813100	0.68977700	-0.46469200
Si	-3.64092300	0.61122500	1.70563500
B	-1.14298800	-0.17913800	-0.73552000
C	1.17609400	1.82063400	0.62628600
H	0.85871600	1.29669800	1.52688600
H	1.78723700	2.67984500	0.91503500
C	-0.08716500	2.25512700	-0.15226500
H	-0.37806200	3.24517900	0.19961200
H	0.14792800	2.37341900	-1.21847300
C	-1.20546700	1.24203800	0.07500800
C	-2.16049100	1.56810800	0.97565400
C	-3.63382300	0.94802300	3.56614000
H	-4.47737700	0.42397800	4.02828200
H	-3.72150800	2.01147300	3.80063500
H	-2.71555400	0.57085100	4.02889900
C	-5.22179200	1.38469700	1.00826500
H	-5.20793200	1.47791500	-0.08139900
H	-5.36256900	2.38700000	1.42397300
H	-6.09226000	0.78178300	1.28739000
C	-3.73767600	-1.27389300	1.55052800
H	-4.03109900	-1.67037500	2.52802400
H	-2.81136400	-1.77059400	1.25812200
H	-4.51121900	-1.56136300	0.83283200
C	-2.15311600	2.92644000	1.61688000
C	-1.41395800	3.18675200	2.77727400
H	-0.83010400	2.38266000	3.22009800
C	-1.43592600	4.44647300	3.36804700
H	-0.85800400	4.63147100	4.26866100
C	-2.20237000	5.46817300	2.81110400
H	-2.22265400	6.44982000	3.27364300
C	-2.94251500	5.22142800	1.65792500
H	-3.53895100	6.01314300	1.21462200
C	-2.91941700	3.96043100	1.06757400
H	-3.48595800	3.76270000	0.16102800
C	3.48573400	1.76838800	-1.18426500
C	4.52652300	2.29131500	-0.38049400
C	5.51421100	3.08395200	-0.96825800
H	6.30877200	3.47264000	-0.33604900
C	5.51935400	3.39029300	-2.32360600
C	4.48691600	2.87819900	-3.10109700
H	4.46064700	3.10385900	-4.16443600
C	3.47644000	2.08304100	-2.56206500
C	4.64631300	2.05909500	1.10347100
H	5.52040900	2.58313500	1.49347000
H	4.75237500	0.99967100	1.34540700
H	3.77531700	2.44281500	1.64058100
C	6.58270800	4.26928400	-2.92536000
H	6.84824900	3.93483100	-3.93118200
H	7.48695800	4.27376100	-2.31309000
H	6.22808600	5.30203500	-3.00465800
C	2.40708900	1.60599200	-3.51011900
H	2.60160100	1.97541200	-4.51842600
H	1.41827100	1.96462900	-3.21357000
H	2.37518500	0.51327100	-3.56228100
C	3.01178800	-0.68967700	0.40427000
C	3.57081600	-1.65587300	-0.46580500
C	4.24274200	-2.75777700	0.05948200
H	4.65618600	-3.49246600	-0.62676000
C	4.37523600	-2.95106300	1.43064600
C	3.81200200	-1.99923600	2.27467100
H	3.88825000	-2.13958800	3.35035300
C	3.12795200	-0.87648500	1.80141800
C	3.43361100	-1.56732400	-1.96675000
H	3.91961700	-2.42136800	-2.44121500
H	3.89231900	-0.65591700	-2.36401800
H	2.37886700	-1.58329300	-2.26725500
C	5.04797400	-4.17772300	1.98267000
H	5.83215400	-4.53704600	1.31241400

H	4.31197000	-4.97881600	2.10090400
H	5.49017400	-3.98223200	2.96220700
C	2.53534500	0.02637500	2.85911500
H	1.46192000	-0.14730700	2.98102300
H	2.68222900	1.08370600	2.64794400
H	3.00359000	-0.18672700	3.82195800
C	-2.62603000	-0.62385700	-1.29162500
F	-3.15845300	1.63039900	-1.86042300
C	-3.49819500	0.33168700	-1.81601500
F	-5.55209200	1.01673600	-2.75610900
C	-4.75440900	0.03623900	-2.33066600
F	-6.37180600	-1.58690900	-2.89150600
C	-5.17791200	-1.28129400	-2.39200300
F	-4.71270200	-3.55258200	-1.98957700
C	-4.33037200	2.27719200	-1.93149200
F	-2.37079500	-2.96755900	-0.93999100
C	-3.09266400	-1.3465000	-1.40104100
C	-0.34199500	-1.39162500	0.04146900
F	-0.71235000	-0.56936900	2.26211900
C	-0.19335300	-1.48856000	1.42468400
F	0.65986700	-2.49064500	3.38848400
C	0.50592700	-2.50922500	2.06289000
F	1.83486900	-4.44896000	1.91012000
C	1.11942800	3.49656200	1.31589800
F	1.61454900	-4.37365900	-0.81324100
C	1.00025400	-3.45832700	-0.06421600
F	0.18991500	-2.50585300	-1.99503800
C	0.27182500	-2.43811500	-0.65244600
H	0.72162500	0.21097500	-1.48029800
C	-0.37373300	0.10421500	-2.39062500
H	-0.83260400	1.04743300	-2.69356100
H	-0.87953800	-0.74059000	-2.85038800
H	0.58160300	0.05141400	-2.92835600

P18_P

P	2.42148700	0.49213400	-0.34386100
Si	-3.63841700	1.31415900	1.59364500
B	-1.36457800	-0.11073800	-1.02243600
C	1.18093400	1.52761300	0.49925000
H	0.70568100	0.92039300	1.27036400
H	1.70647400	2.35268000	0.99000600
C	0.09176100	2.04088700	-0.48656400
H	0.00375600	3.11847900	-0.34216600
H	0.40113200	1.89322100	-1.52457500
C	-1.22124400	1.30404200	-0.19053900
C	-2.03741900	1.88408300	0.72110400
C	-3.47209200	1.87547400	3.39615900
H	-4.37038300	1.57294300	3.94527500
H	-3.36134700	2.95846800	3.49318100
H	-2.61266400	1.40289700	3.88424800
C	-5.11309600	2.25421000	0.87750100
H	-5.25496100	2.07800000	-0.19125200
H	-4.98756300	3.33111900	1.02807500
H	-6.02686100	1.95076900	1.39984400
C	-4.07791000	-0.52046500	1.70560200
H	-4.56228900	-0.68114600	2.67494400
H	-3.21531100	-1.18680600	1.64841700
H	-4.79026700	-0.80735600	0.92901500
C	-1.69503500	3.24659400	1.25661600
C	-0.83864000	3.41741100	2.35249600
H	-0.42502600	2.53562300	2.83872400
C	-0.53725000	4.68704100	2.83727100
H	0.12549700	4.79641800	3.69098500
C	-1.09192700	5.81532900	2.23698300
H	-0.86113400	6.80580800	2.61609100
C	-1.95037400	5.66130800	1.15161100
H	-2.38894800	6.53453600	0.67798200
C	-2.25034800	4.38966100	0.66896500
H	-2.91393500	4.26397500	-0.18313100
C	3.72710200	1.51316100	-1.09303900
C	4.72705900	2.07764800	-0.27082200
C	5.73018700	2.83992300	-0.86164400
H	6.50035400	3.27106800	-0.22691300
C	5.77383800	3.06455700	-2.23605600
C	4.77377000	2.50443500	-3.02492600
H	4.79040800	2.67279400	-4.09842500
C	3.74165600	1.73367400	-2.48885700
C	4.74977000	1.90370500	1.22692900
H	5.60213100	2.43294700	1.65521400
H	4.82545900	0.85230900	1.51840900
H	3.84855000	2.31876500	1.68963700
C	6.85629000	3.91541000	-2.84295800
H	7.81077300	3.76237300	-2.33403000
H	6.60117300	4.97640700	-2.75502200
H	6.98924500	3.69170200	-3.9032930
C	2.69757000	1.20265700	-3.44286500
H	2.97389000	1.45132300	-4.46839300
H	1.71388300	1.64253100	-3.25550300

H	2.58475700	0.11525700	-3.39130900
C	3.08560900	-0.92788400	0.57096000
C	3.66293600	-1.94706800	-0.22422200
C	4.16335400	-3.08453900	0.40224400
H	4.59071100	-3.87221100	-0.21249200
C	4.10935000	-3.24890300	1.78407700
C	3.53607600	-2.23367100	2.54393200
H	3.46999400	-2.35245700	3.62232600
C	3.01409900	-1.07247800	1.97355800
C	3.70687300	-1.89202300	-1.73201800
H	4.29781600	-2.72434400	-2.11617200
H	4.15853800	-0.96632600	-2.10206300
H	2.70165300	-1.98892300	-2.16096600
C	4.59992200	-4.51368800	2.43055800
H	4.94280900	-4.33244500	3.45144600
H	5.41686100	-4.96163300	1.86086400
H	3.78132200	-5.23850800	2.47305400
C	2.35688200	-0.09674000	2.92111300
H	1.27847900	-0.28299800	2.97804900
H	2.50974100	0.94744900	2.65035300
H	2.76131100	-0.23870600	3.92484200
C	-2.92056600	-0.63756500	-1.17287900
F	-3.59555400	1.53158300	-1.88424900
C	-3.89428900	0.24121700	-1.64827700
F	-6.09453900	0.80190100	-2.30801300
C	-5.21239200	-0.11532000	-1.90170100
F	-6.86286100	-1.80453200	-1.98493600
C	-5.60777900	-1.43367100	-1.73306900
F	-5.02691800	-3.63676700	-1.14366500
C	-4.66978600	-2.36058700	-1.30706700
F	-2.55586100	-2.92244500	-0.58171500
C	-3.36932800	-1.94930300	-1.03615600
C	-0.43162200	-1.30225000	-0.29496000
F	-0.89660700	-0.60645200	1.94718700
C	-0.35596300	-1.49623700	1.08561500
F	0.34208400	-2.64318600	3.03798100
C	0.27312800	-2.57113100	1.70462700
F	1.52620700	-4.6426600	1.50110000
C	0.89738300	-3.5409600	0.93256800
F	1.59180200	-4.23027900	-1.20470900
C	0.90632600	-3.37258300	-0.44225400
F	0.35712100	-2.22749700	-2.35461800
C	0.25801100	-2.28421100	-1.01128400
H	1.74594600	-0.07636900	-1.42406400
C	-0.91258700	0.14574000	-2.59053300
H	-1.34940400	1.08731100	-2.94564000
H	-1.27310700	-0.64808800	-3.25026200
H	0.16798300	0.20345100	-2.76815200

P19_TS

C	3.64194800	-0.37499500	1.10662600
C	4.77246100	-0.75128000	1.84854700
C	4.64211400	-1.14511600	3.18756200
C	3.40339400	-1.22504900	3.83294500
C	2.25614900	-0.84823900	3.13444200
C	2.45756200	-0.38405800	1.82700500
O	1.30103700	-0.03562300	1.23996800
C	0.28209800	-0.41889800	2.07292000
C	0.79734400	-0.87507500	3.29451300
C	-0.12864700	-1.31931800	4.24164000
C	-1.47213900	-1.31098200	3.87728400
C	-1.90526000	-0.90879200	2.60103100
C	-1.02496600	-0.44138600	1.61601600
B	-1.39549300	-0.06507100	0.05355200
C	-1.93331000	1.48415500	0.00395600
C	-3.13899100	1.79268500	0.63523500
F	-3.87518200	0.80518200	1.16694600
C	-3.66704400	3.06933100	0.73395900
F	-4.83002400	3.28459900	1.34736100
C	-2.96852200	4.13712200	0.18519400
F	-3.45053000	5.37447200	0.27225800
C	-1.76717400	3.89300700	-0.45568800
C	-1.28524800	2.58960900	-0.53244200
F	-0.10363000	2.46692600	-1.18705900
F	-1.07580500	4.90122000	-0.99018700
C	-2.44119500	-1.17309900	-0.53671500
C	-3.62750300	-0.92009200	-1.22103400
F	-4.02303000	0.33281600	-1.48394600
C	-4.47406300	-1.92207500	-1.68537800
F	-5.60255200	-1.61271200	-2.32443900
C	-4.13713800	-3.25157500	-1.48969300
F	-4.93173600	-4.22276800	-1.93390700
C	-2.95353600	-3.55959500	-0.83450500
C	-2.14393800	-2.52818400	-0.38180900
F	-0.99684800	-2.89850400	0.21698400
F	-2.60368000	-4.83423700	-0.65522400
H	5.75588100	-0.75365200	1.39001200
H	5.53542700	-1.43104500	3.73247300

H	3.34498700	-1.59370900	4.85189100
H	0.18442100	-1.68465700	5.21406300
H	-2.21104600	-1.66397600	4.58957100
H	-2.96183500	-0.99145700	2.36556700
C	4.18795200	1.62837300	-1.03626300
C	4.21093400	-1.30788600	-1.72232200
H	1.43664100	-0.05935900	-0.99783700
C	4.24402500	1.76257200	-2.57061000
C	4.24464400	-0.72217100	-3.15071900
C	4.96592700	0.62169400	-3.29556600
H	4.72823100	2.72060300	-2.80047300
H	3.21779800	1.83347600	-2.95749200
H	4.71432500	-1.47353500	-3.79892100
H	3.21258000	-0.60587900	-3.51165800
H	5.03015100	0.86923400	-4.35966200
H	6.00025300	0.53809900	-2.94091400
P	3.36935600	-0.00984500	-0.67571500
C	3.29266100	-2.53904100	-1.69999200
H	3.18610900	-2.93939100	-0.68628200
H	2.29541200	-2.30471900	-2.08458300
H	3.72082100	-3.32736600	-2.32933300
C	5.60812800	-1.74140500	-1.26869900
H	5.56563500	-2.23035900	-0.29236700
H	6.00072900	-2.47071200	-1.98705800
H	6.32284100	-0.91815500	-1.21148100
C	3.26636300	2.70780300	-0.44973800
H	3.18385200	2.60637100	0.63787500
H	3.68469000	3.69823900	-0.66184900
H	2.25928300	2.66703300	-0.87451100
C	5.57745600	1.78225000	-0.41025600
H	6.30193200	1.05899500	-0.78912700
H	5.95952200	2.78416900	-0.63846300
H	5.53009200	1.6241200	0.67764000
C	0.02911300	-0.27000800	-0.98917800
H	0.48698700	0.61858900	-0.54199600
H	0.34473500	-1.26427900	-0.67809900
H	-0.23405900	-0.16159100	-2.03746500

P19_P

C	3.19437900	-1.66671900	0.16929700
C	4.26596600	-2.57109500	0.07510400
C	4.01630100	-3.93592500	-0.06583000
C	2.71533500	-4.44869900	-0.12812300
C	1.63295700	-3.58133600	-0.01421100
C	1.91390000	-2.21266600	0.15794500
O	0.80361400	-1.47887500	0.29952400
C	-0.27484000	-2.35382700	0.18348300
C	0.17830900	-3.66089000	-0.01427800
C	-0.78155300	-4.66919200	-0.16474800
C	-2.11431700	-4.28738200	-0.11096300
C	-2.49899800	-2.94817600	0.09464500
C	-1.58593500	-1.90443500	0.25974400
B	-1.88297000	-0.35974300	0.71944700
C	-3.45729900	0.05157300	0.45538600
C	-4.01681700	-0.05861400	-0.81725900
F	-3.26558300	-0.50383800	-1.83594000
C	-5.33068000	0.26261000	-1.13023200
F	-5.79274700	0.12691400	-2.37629900
C	-6.16724600	0.73273200	-0.12908600
F	-7.43346800	1.05088900	-0.39957700
C	-5.66659200	0.86602000	1.15522800
C	-4.34193400	0.52818800	1.42131500
F	-3.96549700	0.69792600	2.69726600
F	-6.45936800	1.31776700	2.13106300
C	-0.92673900	0.69326900	-0.17318800
C	-0.45834000	1.90332900	0.34106600
F	-0.76968600	2.29993600	1.58541200
C	0.38892400	2.77656600	-0.34034400
F	0.86516900	3.88043100	0.25475100
C	0.78067500	2.47645200	-1.63293200
F	1.62388300	3.27847500	-2.29673400
C	0.34288500	1.29185800	-2.20256900
C	-0.47554800	0.43422500	-1.47316200
F	-0.75249500	-0.71487200	-2.09596500
F	0.76952700	0.96227700	-3.42762400
H	5.28992000	-2.21792500	0.12023800
H	4.85777300	-4.61605000	-0.13644700
H	2.55839900	-5.51473100	-0.25779900
H	-0.49661000	-5.70426100	-0.32125300
H	-2.88512400	-5.04226600	-0.23038400
H	-3.56105600	-2.71648500	0.13457000
C	3.42429700	0.77398300	1.97277900
C	4.44638400	0.93733000	-0.89037100
H	1.99383300	0.54501900	-0.16881500
C	3.55291100	2.30818000	1.85673500
C	4.37805800	2.44604600	-0.55091200
C	4.65160400	2.80678200	0.91260800
H	3.72373100	2.68719500	2.87178700

H	2.59064700	2.72492600	1.52938100
H	5.10393000	2.94209100	-1.20687200
H	3.39311300	2.83788200	-0.83642500
H	4.70356500	3.89620300	0.99238900
H	5.63408000	2.43322700	1.22578300
P	3.26134200	0.12168300	0.25942600
C	3.96061000	0.69284200	-2.32630600
H	3.90506600	-0.37587300	-2.55557700
H	2.98491200	1.14288200	-2.52061400
H	4.67940500	1.14840400	-3.01541700
C	5.87463600	0.39353000	-0.74775300
H	5.94741100	-0.62023200	-1.14774100
H	6.53994300	1.02828100	-1.34209800
H	6.24795600	0.39203800	0.27841800
C	2.13020300	0.40475600	2.71204200
H	1.99154600	-0.67944100	2.77088100
H	2.19076900	0.79465300	3.73335000
H	1.24279700	0.83626100	2.23895700
C	4.62577300	0.14025200	2.68491300
H	5.58595200	0.44859900	2.26527700
H	4.60798700	0.45615700	3.73266300
H	4.56964300	-0.95251600	2.66488500
C	-1.47566100	-0.36153100	2.30659900
H	-1.59466700	0.59037700	2.82290300
H	-2.09145700	-1.09588400	2.83792000
H	-0.43492000	-0.68740100	2.42830400

P20_TS

P	-2.69088200	-0.26393700	-0.02891500
C	-2.75250800	-0.61122900	1.76497900
C	-3.79065200	-1.12472400	2.54685200
C	-3.51313900	-1.64614400	3.81997700
C	-2.21254500	-1.75120600	4.32208500
C	-1.14977400	-1.27503500	3.54890600
C	-1.49651500	-0.66329700	2.34191000
O	-0.41731400	-0.26657800	1.63863000
C	0.68185100	-0.80406900	2.27083700
C	0.31534500	-1.36287100	3.50346300
C	1.33104300	-1.96232800	4.25179200
C	2.60596600	-2.00119800	3.69291300
C	2.87463900	-1.50956400	2.40446200
C	1.89598000	-0.89606600	1.61307600
B	2.02339900	-0.42026700	0.04587400
C	3.11412900	-1.34098500	-0.73985600
C	4.17825000	-0.88089100	-1.51163400
F	4.39081800	0.43005100	-1.68820200
C	5.07858500	-1.72382600	-2.15557100
F	6.07867600	-1.22083400	-2.87953300
C	4.92918400	-3.09725900	-2.05104500
F	5.77861000	-3.91679900	-2.66610600
C	3.87751400	-3.60856100	-1.30448300
C	3.00512600	-2.73111200	-0.67726100
F	1.99502300	-3.29466900	0.00946400
F	3.71219800	-4.92809500	-1.20207100
C	2.35155700	1.18832500	0.05507300
C	1.54021900	2.23424200	-0.36666200
F	0.37049300	2.00446400	-1.00068700
C	1.84633900	3.57910900	-0.18096700
F	1.00130300	4.52993100	-0.59634900
C	3.03325600	3.93028500	0.43622200
F	3.34912900	5.21092300	0.61742300
C	3.89595100	2.92570000	0.85736400
C	3.53768100	1.60182700	0.66364700
F	4.41632700	0.67816900	1.07987900
F	5.04928900	3.24610600	1.44336300
C	-2.58013800	1.51104500	-0.46776700
C	-2.48761900	1.75094900	-1.86087900
C	-2.65425300	0.66008800	-2.89347000
C	-2.22570500	3.04024600	-2.31350900
C	-2.01004200	4.09939300	-1.43627000
C	-1.64685100	5.46500000	-1.95213300
C	-2.10489900	3.84253000	-0.07361800
C	-2.39134000	2.57476600	0.43738100
C	-2.45823600	2.44199000	1.93986800
C	-4.25822600	-0.95716600	-0.66044400
C	-4.20220200	-2.23579200	-1.25394100
C	-2.91692300	-3.02607000	-1.32853000
C	-5.36915200	-2.78765700	-1.77393800
C	-6.58891000	-2.11184700	-1.71960600
C	-7.82902900	-2.72968400	-2.30979400
C	-6.62455400	-0.86335700	-1.10642900
C	-5.48119600	-0.26714500	-0.56874900
C	-5.61404300	1.08543100	0.08757200
H	-4.80298400	-1.15973900	2.15356700
H	-4.33545100	-2.03256000	4.41265100
H	-2.03804200	-2.23701400	5.27654200
H	1.13262900	-2.41377900	5.21826600
H	3.40997700	-2.47110200	4.25029100

H	3.87161700	-1.64313300	1.99335000
H	-2.38444000	1.03691000	-3.88139100
H	-2.00842200	-0.20195100	-2.68451200
H	-3.68281800	0.28916400	-2.93934900
H	-2.16049900	3.21473300	-3.38509300
H	-1.79769000	6.23077100	-1.18838300
H	-0.59179700	5.48604900	-2.24031600
H	-2.24115100	5.72862200	-2.83081900
H	-1.94369900	4.65728600	0.62820600
H	-2.53314200	3.43318400	2.39110700
H	-1.55590400	1.96671300	2.33710800
H	-3.31394400	1.85327500	2.27555500
H	-3.10043700	-4.01249200	-1.75792100
H	-2.47439200	-3.16773800	-0.33672300
H	-2.16752400	-2.52268200	-1.94913200
H	-5.32653600	-3.77382500	-2.23087500
H	-7.74825700	-2.79413000	-3.39928400
H	-8.71821300	-2.14304100	-2.07106300
H	-7.97722500	-3.74542700	-1.93287900
H	-7.57151400	-0.33319300	-1.03677800
H	-6.66313600	1.29560400	0.30491200
H	-5.05652800	1.13620800	1.02783800
H	-5.23038400	1.88303200	-0.55604300
H	-0.93344900	-0.68110200	-0.59701700
C	0.48531500	-0.78445300	-0.78789900
H	0.05277400	0.12764200	-0.36953700
H	0.59650900	-0.76621300	-1.86867500
H	0.28515000	-1.76462000	-0.35643300

P20_P

P	-2.76349700	-0.49153700	-0.19077800
C	-2.42898200	-1.53092000	1.22844600
C	-3.40950500	-2.08983300	2.06130200
C	-3.03235800	-2.85720500	3.16289000
C	-1.68756000	-3.09975700	3.46154300
C	-0.69896000	-2.58349300	2.62821100
C	-1.10480400	-1.82031700	1.51982100
O	-0.07118400	-1.36392900	0.79837200
C	1.08647300	-1.83193400	1.42720000
C	0.75221000	-2.57717500	2.56562600
C	1.78724500	-3.09583500	3.34776300
C	3.08458400	-2.81809200	2.94167100
C	3.35161600	-2.06575200	1.78520300
C	2.35656100	-1.54702500	0.94846500
B	2.52101100	-0.67813800	-0.43647300
C	4.11843400	-0.49725000	-0.81594800
C	4.79257100	0.69326900	-1.07592500
F	4.17071200	1.88256100	-0.99326900
C	6.13075900	0.76383300	-1.45200100
F	6.70921700	1.94529200	-1.68569000
C	6.86727500	-0.40004200	-1.59291400
F	8.15070300	-0.35243300	-1.95350900
C	6.24640800	-1.61690400	-1.35706100
C	4.90777800	-1.63631000	-0.98775500
F	4.38325700	-2.85901200	-0.78680000
F	6.93683700	-2.75291400	-1.49307500
C	1.80212100	0.78087000	-0.10493000
C	0.71498000	1.34297800	-0.76201700
F	0.06468600	0.69771200	-1.76031700
C	0.20030700	2.60224300	-0.46710900
F	-0.79680600	3.11701200	-1.19684400
C	0.70712000	3.32150900	0.59945900
F	0.18103400	4.50915900	0.92412200
C	1.76337700	2.78772200	1.32597100
C	2.28495300	1.55733800	0.95028200
F	3.34931500	1.13116800	1.64359200
F	2.27201700	3.47160800	2.35391400
C	-2.86375800	1.29717600	0.07001300
C	-3.42522500	2.05779600	-0.97945300
C	-3.74935500	1.49548900	-2.34526800
C	-3.62543400	3.42178700	-0.77673000
C	-3.22268200	4.05710400	0.39264300
C	-3.34972400	5.54501600	0.56309200
C	-2.59898800	3.29378700	1.37813100
C	-2.39848000	1.92268600	1.24989400
C	-1.60318700	1.22854200	2.32980700
C	-4.26684600	-1.15856100	-0.93865400
C	-4.12317700	-2.12222200	-1.95979800
C	-2.77695500	-2.61170400	-2.44075100
C	-5.27680700	-2.64151500	-2.53960200
C	-6.55140300	-2.24972800	-2.12918600
C	-7.77653300	-2.85195800	-2.76314000
C	-6.65839300	-1.31895400	-1.09917300
C	-5.53776800	-0.75828600	-0.48443800
C	-5.76473600	0.26670500	0.60448100
H	-4.46133500	-1.93919200	1.84121200
H	-3.80336300	-3.27796300	3.79844800
H	-1.42165200	-3.68995300	4.33253900

H	1.58424700	-3.68116400	4.23849000
H	3.91711300	-3.18958300	3.53064800
H	4.39033700	-1.87228400	1.53316900
H	-3.75508400	2.30859600	-3.07280400
H	-2.99247000	0.77736500	-2.67899500
H	-4.72407600	1.00031900	-2.38229700
H	-4.05064600	4.01143200	-1.58476100
H	-3.76703700	5.79726900	1.54128600
H	-2.35660700	6.00097300	0.49985900
H	-3.98040900	5.98680900	-0.21037400
H	-2.21520300	3.79138600	2.26466400
H	-1.26283200	1.96767400	3.05663800
H	-0.71003900	0.75386800	1.90622200
H	-2.17854800	0.46888700	2.86541100
H	-2.90803600	-3.43324500	-3.14583500
H	-2.15567400	-2.98165700	-1.61814000
H	-2.21098400	-1.82672700	-2.95489800
H	-5.17724000	-3.37958700	-3.33146300
H	-7.65754600	-2.93441500	-3.84610400
H	-8.66579500	-2.25324100	-2.55752300
H	-7.95233900	-3.85980200	-2.37414600
H	-7.64506300	-1.01499000	-0.75857600
H	-6.74699100	0.11147900	1.05510300
H	-5.01947700	0.22143400	1.40283600
H	-5.73374700	1.28435000	0.20107500
H	-1.69892800	-0.72516600	-1.06955300
C	1.81542300	-1.46594800	-1.68707300
H	1.87978700	-0.88788600	-2.61556800
H	2.32147900	-2.42041000	-1.85908100
H	0.75483300	-1.68554900	-1.51746500

P21_TS

P	1.88880400	0.08115700	-0.60344300
C	2.27621700	1.86475400	-0.41883300
C	3.47364700	2.47407700	-0.81226400
C	3.60382100	3.85671300	-0.75174500
C	2.55117200	4.65188200	-0.29717400
C	1.34362800	4.08274600	0.09690700
C	1.23702300	2.69401700	0.00396900
O	0.08008600	2.07201100	0.35124500
C	-1.09684000	2.75689300	0.06462900
C	-2.15903000	1.96237300	-0.35119400
C	-3.33058000	2.66200900	-0.66089100
C	-3.40436200	4.04991900	-0.56627900
C	-2.29933800	4.79146400	-0.15801900
C	-1.10951400	4.14493000	0.17336200
C	0.15253000	4.82729500	0.69310800
C	0.18766400	6.31831200	0.36470000
C	0.19551800	4.64236100	2.22737000
B	-1.92553600	0.38454000	-0.63138000
C	-1.33762800	-0.53715600	0.58026400
C	-1.11207600	-1.88828900	0.32199100
F	-1.21267200	-2.34139500	-0.94452900
C	-0.76882000	-2.81626400	1.29045200
F	-0.52323300	-4.09066200	0.97296100
C	-0.61041800	-2.39090400	2.60106100
F	-0.23003600	-3.25307900	3.54400400
C	-0.81499700	-1.05748700	2.91141500
C	-1.19349300	-0.16617400	1.91239200
F	-1.40606100	1.09480800	2.31398600
F	-0.61247500	-0.63063400	4.15906700
C	-3.33530700	-0.36447700	-1.02733200
C	-4.34515100	-0.33497400	-0.06325100
F	-4.13941800	0.32412400	1.08698100
C	-5.57672000	-0.95674400	-0.20800100
F	-6.50124500	-0.87767600	0.74893400
C	-5.83864900	-1.67503600	-1.36677100
F	-7.01115900	-2.28300100	-1.53234300
C	-4.86321500	-1.75477500	-2.34660400
C	-3.64207300	-1.11419700	-2.15763600
F	-2.75236500	-1.27128500	-3.15292900
F	-5.09637600	-2.44925200	-3.46117400
C	3.34859200	-0.49930500	-1.58810300
C	3.19544500	-0.80578700	-2.95382400
C	1.88251500	-0.64452800	-3.67207400
C	4.28017600	-1.29905300	-3.67730800
C	5.52648200	-1.49545000	-3.09089300
C	6.67711100	-2.06695800	-3.87556800
C	5.67140800	-1.15354500	-1.75035700
C	4.61688400	-0.65916300	-0.97981100
C	4.94271100	-0.32230000	0.45908500
C	2.07713100	-0.91711500	0.93974500
C	2.20672200	-0.40716600	2.24868700
C	2.02318000	1.04357500	2.61465400
C	2.48999700	-1.29402100	3.28844200
C	2.63673300	-2.66405800	3.08774100
C	2.90120900	-3.59432000	4.24000000
C	2.44865300	-3.15239100	1.79921100

C	2.15691500	-2.31182200	0.72443900
C	1.91133600	-2.97440600	-0.61327000
H	4.29425300	1.87111500	-1.18559800
H	4.53331200	4.32076400	-1.06337500
H	2.68017100	5.72839900	-0.25387200
H	-4.20257400	2.11323300	-1.00589100
H	-4.32840800	4.56018600	-0.81840600
H	-2.36867000	5.87262300	-0.10036100
H	-0.66400900	6.82683300	0.82265500
H	0.16064700	6.49345700	-0.71481900
H	1.08975200	6.78054100	0.77490700
H	-0.64968900	5.16418000	2.68599900
H	0.12276800	3.58406500	2.49411700
H	1.12771500	5.04669800	2.63496100
H	2.00848800	-0.81606000	-4.74253900
H	1.14497700	-1.36644000	-3.30617700
H	1.46458200	0.35693300	-3.54056300
H	4.14194900	-1.53487900	-4.72971000
H	7.63265600	-1.67752600	-3.51666200
H	6.70663500	-3.15694200	-3.77622400
H	6.58626200	-1.83426000	-4.93891600
H	6.64467600	-1.26964500	-1.27851300
H	6.01042000	-0.10717300	0.54469500
H	4.71122000	-1.15875000	1.12637000
H	4.39704200	0.54878100	0.82639400
H	2.11039500	1.16320100	3.69604700
H	1.02970900	1.39169700	2.31946500
H	2.76512600	1.69498600	2.14285500
H	2.58559900	-0.89452000	4.29500300
H	3.48487700	-4.46217800	3.92416800
H	1.95214200	-3.95787000	4.64565100
H	3.43877000	-3.08771300	5.04469700
H	2.50005400	-4.22409400	1.62049500
H	1.49972600	-3.97207500	-0.44999400
H	1.18295100	-2.42993200	-1.22187500
H	2.83401200	-3.06910400	-1.19421100
H	0.16502700	0.17249900	-1.21358800
C	-0.86396400	0.47283800	-2.11343800
H	-1.81313400	0.79018600	-2.56194600
H	-0.23746000	1.34741100	-2.34066300
H	-0.59514700	-0.39207100	-2.71852600

P21_P

P	-2.22562300	0.11005200	0.49055000
C	-2.40976900	1.88259100	0.25443900
C	-3.65293500	2.50822800	0.41230600
C	-3.75261300	3.87998200	0.24448500
C	-2.62121600	4.62443400	-0.09766500
C	-1.37361200	4.03131700	-0.26269300
C	-1.27768300	2.65028300	-0.04938000
O	-0.10814900	1.99593600	-0.16594200
C	1.07992400	2.71540800	0.09241500
C	2.16254000	1.95778800	0.53670400
C	3.30982800	2.70527300	0.82500200
C	3.35940200	4.08883600	0.67819300
C	2.25346800	4.78288600	0.20333700
C	1.08284900	4.09314500	-0.11433500
C	-0.12899400	4.73933700	-0.77660700
C	-0.19454600	6.24841400	-0.54356200
C	-0.03249500	4.46486800	-2.29560200
B	2.02277900	0.36819200	0.88544600
C	1.38582300	-0.55389400	-0.34565000
C	1.15407100	-1.91174500	-0.11613400
F	1.29222500	-2.42124700	1.12437600
C	0.80526000	-2.82742000	-1.09868700
F	0.55915200	-4.10832300	-0.79267600
C	0.65433400	-2.39459900	-2.40562600
F	0.28586300	-3.24943800	-3.36485500
C	0.85191600	-1.05552500	-2.69267400
C	1.23004600	-0.17956200	-1.67972000
F	1.44102900	1.08351600	-2.08635200
F	0.64027300	-0.60905400	-3.93592400
C	3.51174500	-0.31906200	1.07101500
C	4.44288400	-0.22719900	0.03600300
F	4.13125200	0.45980700	-1.07628800
C	5.69828300	-0.81708900	0.06374700
F	6.54734900	-0.68447200	-0.95895900
C	6.06658300	-1.56784900	1.17199900
F	7.26559500	-2.14962800	1.22207000
C	5.17210100	-1.70981000	2.21953400
C	3.92447000	-1.09589400	2.14807300
F	3.12033200	-1.30990300	3.20141900
F	5.51161300	-2.44061900	3.28558900
C	-3.62909800	-0.43265100	1.50455600
C	-3.45751000	-0.53068700	2.90056900
C	-2.17760500	-0.13168400	3.59068800
C	-4.51554800	-1.01391400	3.66631400
C	-5.73090800	-1.38600500	3.09466400

C	-6.84211100	-1.94273900	3.94338100
C	-5.88696300	-1.23344000	1.71900800
C	-4.86057400	-0.76167100	0.90088800
C	-5.14654800	-0.62380600	-0.57807100
C	-2.15522800	-0.97547000	-0.95711500
C	-2.22821700	-0.52166800	-2.28986600
C	-2.02905900	0.91636600	-2.69803500
C	-2.43390500	-1.46790300	-3.29146400
C	-2.54905900	-2.83096000	-3.01948000
C	-2.73862600	-3.82404700	-4.13209700
C	-2.38653800	-3.25585500	-1.70359200
C	-2.17625600	-2.35650700	-0.65961100
C	-1.94714300	-2.92462900	0.72337800
H	-4.52516700	1.92401500	0.68719600
H	-4.70879600	4.37351200	0.37527200
H	-2.72367800	5.69448400	-0.24461400
H	4.19026400	2.18420000	1.19144400
H	4.26822200	4.62917400	0.92421600
H	2.30695100	5.85813500	0.07366900
H	0.69895900	6.73047600	-0.94506300
H	-0.27211900	6.48854000	0.52086000
H	-1.04727900	6.68620800	-1.06985900
H	0.85056700	4.96686900	-2.70163000
H	0.07580600	3.39369600	-2.49001100
H	-0.92455800	4.83534000	-2.81219100
H	-2.31026100	-0.15377700	4.67313600
H	-1.34960100	-0.80569500	3.34658200
H	-1.86098800	0.87976800	3.31546600
H	-4.38556500	-1.09576400	4.74250200
H	-7.81713500	-1.77734600	3.48036900
H	-6.71546400	-3.02207100	4.07643500
H	-6.84731600	-1.48715200	4.93617900
H	-6.84121300	-1.48429800	1.26206500
H	-6.22403900	-0.53779400	-0.73286700
H	-4.79010900	-1.49339100	-1.14006900
H	-4.67801300	0.26114700	-1.01853900
H	-2.00518200	0.98746900	-3.78641100
H	-1.07072400	1.28763500	-2.32053000
H	-2.82012100	1.57783100	-2.33110500
H	-2.48213400	-1.12742500	-4.32244600
H	-3.26885000	-4.71351600	-3.78485300
H	-1.76031500	-4.13928300	-4.50696800
H	-3.29443600	-3.38739400	-4.96473600
H	-2.39115200	-4.31995200	-1.48069500

H	-1.48716500	-3.90993900	0.62840400
H	-1.25358300	-2.32175100	1.31946500
H	-2.88241500	-3.02773000	1.28301100
H	-1.05127000	-0.07234900	1.22729600
C	1.12701700	0.32958200	2.26208100
H	1.71578700	0.70699300	3.10426400
H	0.26698300	1.00964200	2.18018800
H	0.77187200	-0.66620400	2.55034500

P22_TS

P	2.02420900	-1.27274700	-1.40501100
C	3.22930800	0.06782400	-1.12522700
C	4.45962900	0.24852800	-1.76755500
C	5.23519600	1.36674400	-1.48134700
C	4.80391400	2.30927800	-0.54469400
C	3.59547300	2.15365200	0.12863600
C	2.83325500	1.02871000	-0.19216000
O	1.63008100	0.82750900	0.41802400
C	0.87330600	1.96839500	0.67044900
C	-0.50220700	1.84729100	0.47528800
C	-1.24233700	2.98528800	0.81527700
C	-0.63139000	4.15925700	1.24773700
C	0.75393700	4.23918900	1.35653200
C	1.53977400	3.12268100	1.07624800
C	3.05803400	3.05258600	1.23862700
C	3.36620100	2.36734300	2.58953300
C	3.70663400	4.43550800	1.21637400
B	-1.11613100	0.48105800	-0.15058700
C	-0.76113600	-0.84605600	0.73252800
C	-0.62888400	-0.79433200	2.11940200
F	-0.71135900	0.36868200	2.77473100
C	-0.44224000	-1.92038700	2.91488000
F	-0.32419700	-1.80522100	4.23690400
C	-0.36796600	-3.17303300	2.32521200
F	-0.15658900	-4.25824900	3.06542700
C	-0.50812700	-3.27706500	0.94998500
C	-0.71785400	-2.12951200	0.20103600
F	-0.89872600	-2.31760400	-1.12321700
F	-0.40651700	-4.46857200	0.35175100
C	-2.74557200	0.55955700	-0.42088500
C	-3.68916500	-0.34148400	0.08480100
F	-3.34437900	-1.38110600	0.85800100
C	-5.05912500	-0.25003700	-0.14818000
F	-5.88661800	-1.15590300	0.37064900

C	-5.56484500	0.78265500	-0.91679000
F	-6.86970900	0.88344200	-1.14779500
C	-4.68196000	1.71608000	-1.43692400
C	-3.32565600	1.58755500	-1.17529300
F	-2.56036300	2.56025800	-1.70956500
F	-5.13749300	2.72935500	-2.17217700
C	2.34398700	-1.87389800	-3.12765700
C	2.07643900	-0.77683900	-4.16209500
C	1.47483400	-3.10835800	-3.39369800
C	2.58463500	-2.65761800	-0.29319400
C	3.89692200	-3.29660600	-0.74993900
C	2.71029600	-2.13631000	1.14169000
H	4.80169000	-0.47252600	-2.50542400
H	6.18394200	1.50926100	-1.98727100
H	5.42814900	3.17268100	-0.33843900
H	-2.32469500	2.95930600	0.73721300
H	-1.23879100	5.02443600	1.49301100
H	1.21560400	5.16933600	1.67086900
H	2.96362800	2.96923100	3.40954200
H	4.44744400	2.26038400	2.72357200
H	2.90710300	1.37573800	2.63617200
H	3.32884100	5.04565400	2.04011500
H	4.78833100	4.35463200	1.35443200
H	3.50860900	4.95716900	0.27540900
H	3.40129100	-2.16115800	-3.18338100
H	2.34890400	-1.13832800	-5.15821400
H	2.65299200	0.13019700	-3.95990800
H	1.01638400	-0.50894300	-4.18395900
H	1.63624300	-3.46310900	-4.41587900
H	0.41335900	-2.86767600	-3.27999400
H	1.70221400	-3.93319200	-2.71197800
H	1.77757100	-3.39947800	-0.33951700
H	4.21271900	-4.04191100	-0.01324500
H	3.81000400	-3.80162500	-1.71534300
H	4.68981000	-2.54336600	-0.81931100
H	2.83707400	-2.97674000	1.83090000
H	3.58799700	-1.48715900	1.23140200
H	1.83580800	-1.55995100	1.44967300
H	0.47799800	-0.37589800	-1.26392300
C	-0.39422700	0.48796400	-1.88467400
H	0.54149100	0.97123500	-2.20278300
H	-0.71607300	-0.26009100	-2.61472600
H	-1.06972100	1.33468700	-2.00104800

P22_P

P	2.58510400	1.21842200	1.30993700
C	3.36824100	-0.37177300	1.08621200
C	4.62185700	-0.67692000	1.63032200
C	5.20535400	-1.90321400	1.35457600
C	4.56521700	-2.79974500	0.49385000
C	3.33430600	-2.51186900	-0.08601600
C	2.71857800	-1.30209900	0.26153500
O	1.49917100	-0.97275000	-0.21427100
C	0.60333500	-2.02741200	-0.50957700
C	-0.75675400	-1.76448700	-0.32624100
C	-1.59526000	-2.81885100	-0.71138000
C	-1.11178100	-4.03973300	-1.16765600
C	0.25663200	-4.25615400	-1.27773100
C	1.14615700	-3.23153000	-0.96172100
C	2.65407800	-3.33239700	-1.17245800
C	2.99247400	-2.66576100	-2.52661100
C	3.15085400	-4.77787100	-1.19008300
B	-1.29322500	-0.37162400	0.37137100
C	-0.79775000	0.91617500	-0.56302100
C	-0.80748100	0.83190900	-1.95924900
F	-1.22773500	-0.28372500	-2.56462100
C	-0.44487500	1.86891500	-2.81125800
F	-0.47798300	1.70900200	-4.13467000
C	-0.04250700	3.08627100	-2.27921600
F	0.35832800	4.07960200	-3.07297700
C	-0.04189600	3.23702100	-0.90436400
C	-0.43043700	2.17288600	-0.09781200
F	-0.39805600	2.44772500	1.23218900
F	0.39274000	4.38099900	-0.35115800
C	-2.95052500	-0.34298200	0.48378000
C	-3.80235200	0.63021100	-0.03923500
F	-3.34006400	1.64927400	-0.78652900
C	-5.18036200	0.65279900	0.15936400
F	-5.92388600	1.62726700	-0.37011800
C	-5.78352500	-0.33728000	0.91444300
F	-7.10108400	-0.33226500	1.11564400
C	-4.99065400	-1.33692600	1.45544900
C	-3.62021100	-1.31497000	1.23431100
F	-2.94267800	-2.33567600	1.79062800
F	-5.54883800	-2.30613800	2.18471200
C	2.90248600	1.91725400	2.96592100
C	2.33510300	0.96589100	4.02677500
C	2.26779500	3.31289400	3.04309500

C	3.10758200	2.36251400	-0.01945500
C	4.55520600	2.81046400	0.20009700
C	2.92356300	1.67395300	-1.37811500
H	5.12698700	0.03751100	2.27517100
H	6.16571800	-2.15897800	1.78742300
H	5.05435100	-3.73928400	0.25994600
H	-2.66927100	-2.67869500	-0.64861400
H	-1.80507100	-4.82988000	-1.43847800
H	0.62507300	-5.21476300	-1.62543400
H	2.49299900	-3.20624400	-3.33557800
H	4.07331600	-2.67649300	-2.70287500
H	2.64181300	-1.62947300	-2.54640800
H	2.68159300	-5.32921700	-2.00722100
H	4.22911100	-4.81444600	-1.36862300
H	2.92782600	-5.29113000	-0.25029300
H	3.99082300	1.99786900	3.07967600
H	2.52727500	1.37954000	5.02013100
H	2.79017300	-0.02638000	3.97173300
H	1.25326500	0.85240100	3.90552600
H	2.37923600	3.70033600	4.05887500
H	1.20051600	3.27024600	2.80738100
H	2.73968500	4.02339900	2.35892900
H	2.42566400	3.21877300	0.05630300
H	4.86045900	3.45472900	-0.62821800
H	4.68587200	3.37338800	1.12864800
H	5.22953800	1.94725300	0.21553800
H	3.05838300	2.41249800	-2.17314700
H	3.67189100	0.88512200	-1.50861900
H	1.93185700	1.22636400	-1.48472900
H	1.21356200	1.00883700	1.21569500
C	-0.71542900	-0.35805600	1.91808600
H	0.35726600	-0.58999900	1.97742200
H	-0.90021000	0.57677500	2.45748200
H	-1.20230300	-1.15561700	2.48302200

P23_TS

C	-1.20233900	-2.50410500	-1.71108300
H	-0.32786500	-2.55867200	-2.36077500
H	-2.05235200	-2.19237300	-2.32768200
H	-1.41663600	-3.50739000	-1.32958300
C	-1.00789700	-1.51470900	-0.59463700
C	0.10813700	-0.80962300	-0.31099300
P	-2.38690800	-1.14317500	0.51576100
C	-3.75676600	-0.36824800	-0.39352100
C	-4.47892200	-1.05061200	-1.38091000
H	-4.27132000	-2.09867200	-1.57942900
C	-5.47143900	-0.38906800	-2.09514000
H	-6.02641700	-0.91764500	-2.86309400
C	-5.74677700	0.95251300	-1.83180100
H	-6.51340300	1.46855100	-2.40049400
C	-5.04640400	1.62964800	-0.83841800
H	-5.25864600	2.67378500	-0.63006500
C	-4.06288800	0.96598200	-0.10994200
H	-3.51628500	1.49077100	0.67001700
C	-2.96597200	-2.76907800	1.09609300
C	-4.31390000	-3.02342000	1.36401100
H	-5.06210800	-2.26155300	1.16422300
C	-4.69824200	-4.25785500	1.88017400
H	-5.74645200	-4.45321900	2.08179600
C	-3.74360300	-5.23842300	2.13565400
H	-4.04812900	-6.20023500	2.53523600
C	-2.39796700	-4.98505300	1.87730100
H	-1.65134700	-5.74768600	2.07274300
C	-2.00610300	-3.75435300	1.36216800
H	-0.95589000	-3.55968800	1.15098900
B	0.23993700	0.40673700	0.79728600
C	1.33960000	-1.10516400	-1.09813500
F	1.30180600	-3.38272000	-0.45816200
C	1.91281800	-2.37541400	-1.08889000
F	3.64208000	-3.87124500	-1.67923600
C	3.12557900	-2.64578500	-1.70805300
F	4.96347800	-1.87057600	-2.95674100
C	3.80486500	-1.62372000	-2.35711400
F	3.91852400	0.63916100	-2.99475500
C	3.26705800	-0.34342400	-2.38012400
F	1.58772800	1.14842200	-1.75688700
C	2.05300800	-0.10294700	-1.75383300
C	-0.60400900	1.73422800	0.36782300
F	-1.05114500	1.07437700	-1.88645700
C	-1.17792500	1.95320400	-0.88445700
F	-2.53854000	3.17981900	-2.37810400
C	-1.96074500	3.06565000	-1.18351800
F	-2.97020400	5.07213100	-0.47528100
C	-2.19279600	4.2470400	-0.21415600
F	-1.79420000	4.80750200	1.97297300
C	-1.61088800	3.87608200	1.03902400
F	-0.25226200	2.70370800	2.50058100
C	-0.82988000	2.75864800	1.29059500

C	1.81462900	0.66697700	1.12995100
F	1.87005700	2.96107200	0.50601300
C	2.49639400	1.86619600	0.94920000
F	4.46446000	3.17625200	0.98493300
C	3.86272100	2.00482600	1.18016200
F	5.90581800	1.03397200	1.83792500
C	4.60163000	0.91555300	1.61233200
F	4.66611800	-1.36389200	2.20968500
C	3.96709400	-0.30533500	1.80363800
F	2.04754400	-1.61153000	1.73316800
C	2.60553000	-0.39702700	1.56388800
H	-1.30039300	-0.26456200	1.53829200
C	-0.36293500	-0.11199600	2.51292600
H	0.34859000	0.57038700	2.97291300
H	-1.29467900	0.11644800	3.04933700
H	-0.07808800	-1.14548900	2.71452700

P23_P

C	1.45813200	-2.51800100	1.50866200
H	0.62044600	-2.64265900	2.19585200
H	2.35500700	-2.34327700	2.11025300
H	1.59286400	-3.46149400	0.96717100
C	1.19661500	-1.36927800	0.55905300
C	0.02212400	-0.75351600	0.29548200
P	2.67865900	-0.76334000	-0.23859700
C	4.08041700	-0.64221500	0.89375700
C	4.91311000	-1.74632900	1.11150600
H	4.74189800	-2.67537400	0.57432500
C	5.96599300	-1.64760700	2.01362300
H	6.61096400	-2.50272700	2.18510600
C	6.19220600	-0.45151600	2.69202600
H	7.01668600	-0.37643000	3.39349200
C	5.36596600	0.64706000	2.47319600
H	5.54097200	1.57867400	3.00012600
C	4.30633700	0.55783000	1.57626400
H	3.65652900	1.41446600	1.43142600
C	3.15317300	-1.84465400	-1.60323800
C	4.40957300	-1.70700900	-2.20345700
H	5.11668800	-0.96927000	-1.83377100
C	4.75096400	-2.52694500	-3.27157200
H	5.72103500	-2.42034700	-3.74492100
C	3.84603300	-3.48329700	-3.73105600
H	4.11582600	-4.12166300	-4.56599900
C	2.59961900	-3.62307800	-3.12714300
H	1.89806900	-4.36677100	-3.48879700
C	2.24380000	-2.80236500	-2.06049800
H	1.26637000	-2.89531800	-1.59267000
B	-0.30446900	0.46857200	-0.81127500
C	-1.15409400	-1.28812900	1.03852600
F	-0.93422800	-3.45312700	0.11474200
C	-1.61154100	-2.59233800	0.88250700
F	-3.18083700	-4.29942200	1.32707600
C	-2.77381400	-3.04360700	1.49345100
F	-4.61568800	-2.59765600	2.88763600
C	-3.50805900	-2.17501900	2.28944200
F	-3.77830100	-0.03372400	3.23136700
C	-3.07804700	-0.86476400	2.46573900
F	-1.53449800	0.82693900	1.99975400
C	-1.91531400	-0.44092800	1.84113800
C	0.34617000	1.92247100	-0.37875900
F	1.36088600	1.26909300	1.68286200
C	1.11763500	2.20831400	0.74025400
F	2.54408700	3.58034300	2.03505200
C	1.74807200	3.42851400	0.96823800
F	2.17293000	5.63962200	0.26717600
C	1.57685900	4.46589400	0.07019600
F	0.58044200	5.24589900	-1.91382800
C	0.77380400	4.25538100	-1.04376400
F	-0.58621400	2.89626700	-2.32435900
C	0.19144400	3.01228700	-1.24019600
C	-1.94563200	0.56187700	-0.96099600
F	-2.25101500	2.74149200	-0.04914100
C	-2.76090800	1.63070800	-0.60028200
F	-4.86772700	2.68810800	-0.38527800
C	-4.14561700	1.62763600	-0.74829000
F	-6.10318200	0.48990900	-1.41187500
C	-4.77967000	0.51152600	-1.26835100
F	-4.60942400	-1.68531900	-2.10780800
C	-4.01643900	-0.59097900	-1.62657300
F	-1.96854900	-1.66366700	-1.78488500
C	-2.63964900	-0.53482100	-1.47098600
H	2.47285100	0.54005400	-0.69682900
C	0.28847200	0.07709400	-2.30249000
H	-0.21244800	0.68840300	-3.05791900
H	1.36085800	0.26443600	-2.44486000
H	0.10331500	-0.96747500	-2.56550800

P24_TS

P	1.82896900	0.55675500	-0.56049900	C	0.37397100	2.28257400	1.30218600
B	-0.88027000	-0.89018100	-0.88618300	C	0.70474000	3.56692400	0.85539900
C	0.36227600	1.09260600	0.40080700	H	0.94333100	3.73330000	-0.18921800
C	-0.77907200	0.43136700	0.08196900	C	0.70895100	4.64034400	1.74153200
C	2.82576200	2.07052900	-0.89912900	H	0.96999700	5.62945100	1.37909000
C	3.69118400	2.64308900	0.05719100	C	0.37240500	4.45284600	3.07891700
C	4.40436100	3.79424200	-0.28241100	H	0.38023200	5.29276100	3.76598400
H	5.06323200	4.23376400	0.46272600	C	0.00635000	3.18561900	3.52592300
C	4.28744900	4.40420700	-1.52699500	H	-0.28112300	3.03210300	4.56108100
C	3.40845500	3.84058600	-2.44919200	C	0.00284500	2.11143900	2.64360100
H	3.27949200	4.31210400	-3.42056100	H	-0.30368500	1.12512400	2.98385800
C	2.67783600	2.69045800	-2.15936100	C	-2.05663700	1.09322800	0.49365500
C	3.85180600	2.11058100	1.45642600	C	-2.40846600	2.29459600	-0.11983500
H	4.33249400	2.86205700	2.08609800	F	-1.59285000	2.84505100	-1.02485000
H	4.46248100	1.20336000	1.47732700	C	-3.60601400	2.94160700	0.14287200
H	2.88117300	1.87917800	1.90336900	F	-3.90724800	4.08399400	-0.46911000
C	5.08924300	5.63039100	-1.87354200	C	-4.49902100	2.38221500	1.04678200
H	4.51815300	6.30746300	-2.51340000	F	-5.64893100	2.99157700	1.31315600
H	5.99949200	5.35416600	-2.41566600	C	-4.18447500	1.18329900	1.67098700
H	5.38980000	6.17462800	-0.97562100	F	-5.03643800	0.64234100	2.53866600
C	1.71397300	2.17794600	-3.19860400	C	-2.97963400	0.55464900	1.38452700
H	0.68840200	2.18256100	-2.81718000	F	-2.72306700	-0.59970900	1.99973800
H	1.95552500	1.15393200	-3.50061300	C	0.10974100	-2.11121300	-0.44299200
H	1.74237600	2.80650900	-4.09029500	C	0.24119100	-2.45737900	0.90239200
C	3.01428300	-0.65520600	0.17035900	F	-0.50785600	-1.83946400	1.82350800
C	3.09431900	-1.06132000	1.51510100	C	1.13525500	-3.40614900	1.37098100
C	4.06363300	-1.99898300	1.88444000	F	1.26068900	-3.64287200	2.67874600
H	4.10340700	-2.31320400	2.92557700	C	1.97830100	-4.05117300	0.47870200
C	4.94102400	-2.56499400	0.96789300	F	2.89909900	-4.90383600	0.92143100
C	4.83648600	-2.16497600	-0.36366300	C	1.86863700	-3.77114400	-0.87057900
H	5.49704000	-2.60705000	-1.10556300	F	2.68409300	-4.35775600	-1.74600100
C	3.89271400	-1.23063600	-0.78155400	C	0.92884100	-2.84099500	-1.29965500
C	2.18161700	-0.57308900	2.60967100	F	0.90403000	-2.64198600	-2.63304200
H	1.87398300	-1.41490800	3.23525000	C	-2.44863700	-1.34058700	-1.05392100
H	1.28112300	-0.10999000	2.21834100	C	-2.99738100	-2.47282400	-0.45146500
H	2.69252300	0.15150000	3.25244900	F	-2.24240100	-3.33372500	0.24017200
C	5.92233800	-3.62688100	1.38359700	C	-4.35045300	-2.79706900	-0.50694600
H	6.21974500	-3.50568600	2.42764600	F	-4.80175000	-3.89966200	0.08690700
H	6.82012700	-3.60531600	0.76157100	C	-5.23071300	-1.96943500	-1.18234600
H	5.46340800	-4.61473100	1.27839400	F	-6.52346800	-2.26733400	-1.24971500
C	3.81466000	-0.91335400	-2.25681900	C	-4.74159300	-0.82280800	-1.79078600
H	3.93366400	0.15666700	-2.45635500	F	-5.57037600	-0.00400300	-2.43614400
H	2.85351300	-1.23566100	-2.67487900	C	-3.38674600	-0.53888900	-1.71041400
H	4.60134200	-1.44333400	-2.79652600	F	-3.01120400	0.61477400	-2.29390200

H	0.64926300	-0.14426400	-1.74078200
C	-0.38957900	-0.33217200	-2.59804300
H	-0.87417100	0.62286500	-2.77226600
H	0.49780300	-0.39889200	-3.24090700
H	-0.98555100	-1.15179000	-3.00251700

P24_P

P	1.96105300	0.49143200	-0.39318200
B	-1.06311400	-0.82518400	-1.14497800
C	0.37655100	1.03496600	0.30534800
C	-0.79036700	0.47776200	-0.12074900
C	2.98722700	1.94228600	-0.79889400
C	3.89340800	2.48634300	0.13331400
C	4.66890900	3.57765700	-0.25457200
H	5.36443000	4.00178100	0.46530600
C	4.56709900	4.14948800	-1.52031100
C	3.63106400	3.62118700	-2.40702400
H	3.50809700	4.07649500	-3.38640300
C	2.83120700	2.52997100	-2.07348500
C	4.02020200	1.98905900	1.54990500
H	4.58509000	2.70808300	2.14576700
H	4.52948700	1.02292200	1.60592600
H	3.03341100	1.88849900	2.01324800
C	5.44536400	5.30431300	-1.92020500
H	6.37081800	4.93887600	-2.37720100
H	5.72135300	5.91010700	-1.05431700
H	4.94772700	5.94715800	-2.64952400
C	1.80681600	2.05410800	-3.07235400
H	0.80617000	1.99174500	-2.63182500
H	2.05517900	1.06480300	-3.47469900
H	1.75601200	2.74467900	-3.91537100
C	3.01710200	-0.77782000	0.37138000
C	2.92557100	-1.19148000	1.71449600
C	3.80864900	-2.17123600	2.16793200
H	3.72487000	-2.50417200	3.19914700
C	4.75795300	-2.75994000	1.33743300
C	4.81776600	-2.34427000	0.00937600
H	5.53483400	-2.80857200	-0.66258800
C	3.96437500	-1.36738900	-0.49942700
C	1.90096800	-0.66575600	2.68413600
H	1.84297400	-1.32528400	3.55178700
H	0.90676000	-0.62485800	2.23799600
H	2.15211900	0.33791700	3.04099400
C	5.64225800	-3.86683100	1.84050400
H	6.59715000	-3.88656000	1.31084200
H	5.14688200	-4.82916300	1.67853600
H	5.83728700	-3.76506000	2.91023400
C	4.05798800	-1.04724300	-1.97331300
H	4.16873400	0.02431900	-2.16624600
H	3.17296800	-1.40655100	-2.51202300
H	4.92245000	-1.55218800	-2.40602000
C	0.43219100	2.20172700	1.24614000
C	0.83903000	3.47658700	0.83530300
H	1.11056500	3.65008800	-0.20014100
C	0.86391700	4.53488900	1.73965300
H	1.18269900	5.51626100	1.40372100
C	0.46661100	4.34212000	3.05907600
H	0.48621600	5.16971400	3.76069600
C	0.01959700	3.08771000	3.46833400
H	-0.32152600	2.93428600	4.48697100
C	0.00109100	2.02804200	2.56884600
H	-0.37351400	1.05572900	2.87807600
C	-1.98825000	1.28325700	0.27651200
C	-2.22130500	2.51501700	-0.33046400
F	-1.34768300	2.99458200	-1.22428800
C	-3.35449400	3.26645800	-0.06555800
F	-3.55387400	4.43160900	-0.67769400
C	-4.28995900	2.79218000	0.84475400
F	-5.37919900	3.50422600	1.11300700
C	-4.07674000	1.57826600	1.48204200
F	-4.95958000	1.13075200	2.37148100
C	-2.93670200	0.84106200	1.19034400
F	-2.75280800	-0.30805700	1.84071900
C	0.00778200	-2.01607000	-0.72785400
C	0.07941900	-2.45454500	0.59483500
F	-0.75356400	-1.92552500	1.50575100
C	0.96487400	-3.41097900	1.06212300
F	1.02401700	-3.72027400	2.36020400
C	1.86345000	-3.98885200	0.17701600
F	2.76768000	-4.86810000	0.60965400
C	1.84381000	-3.59540500	-1.14852300
F	2.75790000	-4.07311600	-1.99788900
C	0.92434500	-2.63852600	-1.57035500
F	1.03168700	-2.29781600	-2.87068000
C	-2.63758700	-1.35070000	-0.99192300
C	-3.06682400	-2.56866300	-0.45921800
F	-2.20411700	-3.49992400	-0.02170100
C	-4.40063900	-2.94934800	-0.34269300

F	-4.71708100	-4.13096300	0.18917100
C	-5.40265200	-2.10291300	-0.78166300
F	-6.68334300	-2.44678700	-0.67014500
C	-5.04176800	-0.89079000	-1.34588100
F	-5.98287900	-0.05266100	-1.78509700
C	-3.69746900	-0.55907100	-1.44910800
F	-3.47000600	0.63922000	-2.02126700
H	1.64186700	-0.05298400	-1.63357300
C	-0.86140700	-0.29048800	-2.68346800
H	-1.37198900	0.66104700	-2.84823200
H	0.18109200	-0.15382800	-2.97543700
H	-1.27736500	-1.01217300	-3.39460100

P25_TS

C	-2.45334400	-2.39723700	3.36113500
C	-1.32829700	-3.15963700	3.51735900
C	-0.23048600	-3.04945100	2.62838000
C	-0.21304300	-2.07149900	1.57683000
C	-1.42668400	-1.30378500	1.43661200
C	-2.50340300	-1.50267600	2.28066000
H	0.77723600	-4.70299800	3.58693200
H	-3.30555800	-2.50671100	4.02282300
H	-1.26799200	-3.90074600	4.30998400
C	0.82849100	-3.98277600	2.77529500
C	0.96077700	-1.98942900	0.72144600
H	-3.43419400	-0.97674100	2.08353500
C	1.90466700	-2.98787200	0.88586600
C	1.85693100	-3.98605900	1.88352200
H	2.77334800	-2.99451700	0.23566900
H	2.65569600	-4.71744400	1.95214900
P	-1.76870200	-0.39534400	-0.10954700
B	1.36622900	-0.83593400	-0.37842000
C	2.99266300	-0.86380100	-0.73823900
C	3.84685900	-0.55351900	0.32908800
F	3.33577000	-0.35017100	1.55048500
C	5.22569800	-0.45088600	0.23699200
F	5.95853800	-0.15266400	1.30889100
C	5.84234100	-0.66584300	-0.98692700
F	7.16316000	-0.57207100	-1.10547400
C	5.05488300	-0.98504900	-2.07690900
C	3.67032100	-1.07992600	-1.93892900
F	3.04283900	-1.41356300	-3.08030600
F	5.62188600	-1.20894700	-3.26244700
C	1.12045300	0.72279100	0.07910600
C	1.15369900	1.76749500	-0.84511800
F	1.31521000	1.51272500	-2.15759900
C	0.99649500	3.10615300	-0.51849300
F	0.94215700	4.04140600	-1.46729100
C	0.86200100	3.46440300	0.81188300
F	0.65304100	4.73481600	1.15017200
C	0.89496900	2.47708100	1.78005900
C	1.01511200	1.14417500	1.40652000
F	0.98437600	0.27343700	2.42346000
F	0.72561900	2.80640500	3.06176400
C	-3.33965000	-1.17793600	-0.69798800
C	-4.59373000	-0.52921800	-0.78649500
C	-4.91595200	0.82879400	-0.20188000
C	-5.65467900	-1.19716200	-1.40423900
C	-5.53850600	-2.48411700	-1.91709600
C	-6.69079300	-3.15128600	-2.61976300
C	-4.32197300	-3.13580200	-1.74776300
C	-3.22930500	-2.52005200	-1.13944600
C	-2.02002400	-3.38942500	-0.88518300
C	-2.01721300	1.42700900	-0.00774300
C	-2.12072700	2.20762600	1.16063500
C	-2.11404300	1.64970900	2.56298200
C	-2.23842100	3.59502000	1.03413600
C	-2.24198500	4.23823000	-0.19828000
C	-2.24851300	5.73918000	-0.29518400
C	-2.16163800	3.44829800	-1.34290600
C	-2.04140700	2.06266200	-1.27311800
C	-1.91834200	1.30153400	-2.57302900
H	-5.99963100	0.92899500	-0.11180500
H	-4.48505000	0.96031900	0.79313400
H	-4.55262200	1.65591000	-0.81814200
H	-6.61399400	-0.68879900	-1.47079000
H	-6.66616400	-4.23454400	-2.47961100
H	-7.64897400	-2.77744000	-2.25149200
H	-6.65118100	-2.95635200	-3.69651100
H	-4.21672400	-4.16398700	-2.08660200
H	-2.06369100	-3.79548300	0.13226200
H	-1.06350100	-2.87380100	-0.96396500
H	-2.00585300	-4.22977300	-1.58235300
H	-2.04703800	2.46716700	3.28301000
H	-1.27773000	0.97348600	2.74883000
H	-3.03380000	1.09570500	2.77495600
H	-2.28931000	4.19250800	1.94136000
H	-2.81894400	6.08103600	-1.16226100

H	-1.22123000	6.10208500	-0.40524800
H	-2.67036300	6.19545500	0.60293300
H	-2.16001700	3.92494300	-2.32031500
H	-2.08515600	1.97461600	-3.41583500
H	-0.91391500	0.88204200	-2.68560100
H	-2.64228700	0.48386900	-2.64987600
H	-0.31284900	-0.81163500	-1.08888500
C	0.59111700	-1.27054500	-2.00389200
H	1.06912900	-2.25066600	-2.03713700
H	1.06117900	-0.57635900	-2.69019500
H	-0.40474300	-1.46370200	-2.42988500

P25_P

C	-1.97447400	-2.85014200	3.01779700
C	-1.03175100	-3.78265100	2.67405600
C	-0.18399300	-3.60914000	1.55353200
C	-0.18487900	-2.38071300	0.80194200
C	-1.26910100	-1.48569500	1.13137100
C	-2.13530000	-1.73656900	2.18124000
H	0.61381100	-5.60815700	1.73703200
H	-2.63745000	-3.00053400	3.86205600
H	-0.94379100	-4.70720600	3.23873300
C	0.63118400	-4.69459700	1.15015900
C	0.79556300	-2.18720100	-0.23667400
H	-2.99218700	-1.08878500	2.34418800
C	1.45713400	-3.33790800	-0.63919700
C	1.38041000	-4.58350800	0.01403700
H	2.17344300	-3.24909100	-1.45311400
H	1.96997500	-5.42027300	-0.34723600
P	-1.91799500	-0.30739200	-0.07093200
B	1.41702500	-0.83309300	-0.98214200
C	3.08210700	-0.83605500	-0.77628900
C	3.80073900	-1.49808500	0.22329300
F	3.19728800	-2.27470600	1.13615300
C	5.18196900	-1.40745300	0.37497400
F	5.80102700	-2.09267300	1.34003800
C	5.91847800	-0.58971400	-0.46486300
F	7.24006300	-0.48563800	-0.32631700
C	5.25137700	0.13718200	-1.43770700
C	3.87377400	0.01116200	-1.55715800
F	3.31104800	0.82268600	-2.47176100
F	5.93130300	0.96615100	-2.23365400
C	1.10115900	0.64618800	-0.29846000
C	0.90167300	1.82104600	-1.02254600
F	0.64998300	1.79130100	-2.34274600
C	0.89718800	3.08946600	-0.45348900
F	0.63018700	4.17276500	-1.18987800
C	1.10818000	3.22867000	0.90719800
F	1.01642500	4.43002400	1.48109100
C	1.32793800	2.09711700	1.67634800
C	1.31753900	0.85210900	1.06446300
F	1.53382500	-0.19953000	1.87085100
F	1.47070300	2.21246300	3.00080400
C	-3.57567000	-0.91340900	-0.54005300
C	-4.76514200	-0.39558000	0.01230700
C	-4.83574600	0.73773800	1.01118800
C	-5.97932800	-0.94964300	-0.39879200
C	-6.05317300	-1.98246500	-1.32686400
C	-7.37559000	-2.57571400	-1.73250700
C	-4.86221700	-2.47395300	-1.85754400
C	-3.62156400	-1.96583300	-1.48392200
C	-2.38740100	-2.54731900	-2.13136300
C	-1.98729500	1.47818900	0.24031200
C	-1.79317800	2.08953100	1.49292900
C	-1.49147600	1.35964800	2.78021600
C	-1.88134500	3.48206000	1.56140100
C	-2.11926800	4.27340100	0.44383700
C	-2.05404600	5.77215700	0.53250200
C	-2.32866400	3.63816000	-0.77991700
C	-2.26824700	2.25521000	-0.91222900
C	-2.45596000	1.65910100	-2.28848900
H	-5.82644500	0.75689800	1.46904000
H	-4.10362300	0.64803300	1.81646600
H	-4.66360200	1.70714500	0.53327700
H	-6.89863600	-0.55489600	0.02643600
H	-7.42906700	-2.71066200	-2.81585000
H	-7.51211100	-3.55961200	-1.27279400
H	-8.20851300	-1.94159400	-1.42274300
H	-4.89695900	-3.28191200	-2.58443400
H	-2.65037700	-3.44654700	-2.69022500
H	-1.60850400	-2.82129900	-1.41306800
H	-1.94210100	-1.84109500	-2.84114100
H	-1.06921600	2.06180800	3.50073200
H	-0.77439000	0.54775400	2.65829800
H	-2.40042100	0.93907000	3.22607400
H	-1.70081100	3.96237300	2.51987100
H	-2.70833600	6.24707900	-0.20189600
H	-1.02721100	6.09370200	0.32903800

H	-2.32587400	6.12711000	1.52881200
H	-2.50843200	4.23886700	-1.66762500
H	-2.68176300	2.45035800	-3.00436000
H	-1.53997700	1.16697200	-2.63229500
H	-3.27566600	0.93484500	-2.32056300
H	-1.13265200	-0.46000300	-1.21078600
C	1.02400500	-0.93685400	-2.57287800
H	1.57521400	-0.25618200	-3.21869400
H	-0.03951700	-0.73543000	-2.76802700
H	1.19966900	-1.95559800	-2.93669900

C6H6

N1_TS

C	0.36399700	2.76585100	-0.24051900
C	0.59764200	1.52299400	-0.83251500
C	1.37498100	1.55448800	-2.00402300
C	1.83210100	2.74143500	-2.56293000
C	1.55492800	3.96185600	-1.94682600
C	0.82324400	3.97227000	-0.76704100
H	1.63264400	0.61200500	-2.48204700
H	2.42081600	2.71745600	-3.47471600
H	1.91735900	4.89157500	-2.37303300
H	0.61207400	4.90835900	-0.25619000
H	-0.28964500	1.39264800	1.25889200
B	0.14125300	0.06670000	-0.24633600
N	-0.38667600	2.73993000	0.99762900
C	-1.28402000	-0.55018100	-0.76942000
C	-1.77263300	-1.77012000	-0.21640900
C	-2.12914500	0.08235600	-1.71993700
C	-3.03312100	-2.26197600	-0.54337300
C	-3.40005200	-0.43961400	-2.00667300
C	-3.88379400	-1.59947600	-1.42265500
C	1.51346800	-0.82686300	-0.27566900
C	1.67705400	-1.96006900	-1.11525800
C	2.67522300	-0.39345100	0.41634200
C	2.87298000	-2.68477400	-1.10979200
C	3.84454600	-1.15714100	0.41737800
C	3.95832100	-2.33040900	-0.31812200
C	0.63930900	-2.43485700	-2.11346500
H	-0.14140900	-3.04652500	-1.65354700
H	0.13395200	-1.60637200	-2.61377200
H	1.12844400	-3.04428400	-2.87806100
C	5.21014300	-3.16739300	-0.27948600
H	5.39105300	-3.65363900	-1.24171000
H	6.08541500	-2.56140900	-0.03152500
H	5.13034200	-3.95593400	0.47658400
C	-1.75068800	1.28242400	-2.57243600
H	-1.51084000	2.18456500	-2.00708500
H	-0.87681500	1.06761600	-3.19293800
H	-2.57759900	1.52452200	-3.24427900
C	-5.25520900	-2.13759200	-1.73584000
H	-5.74165900	-1.55260200	-2.51992400
H	-5.20240200	-3.17763100	-2.07195300
H	-5.89912200	-2.11640000	-0.85056800
C	-1.00264100	-2.62632100	0.77049500
H	0.06253600	-2.41141400	0.79689800
H	-1.39579100	-2.49065400	1.78404100
H	-1.13057700	-3.68264500	0.51265900
H	2.95642400	-3.55434400	-1.75948400
H	-3.36105600	-3.19700300	-0.09012300
H	-4.02375000	0.07896300	-2.73191600
C	0.19275700	3.49872000	2.11136300
H	-0.23535500	3.11967100	3.04429100
H	1.27345700	3.35373900	2.12423900
H	-0.02666900	4.56943500	2.02531300
C	-1.80761100	3.06489900	0.80503000
H	-2.33699900	2.92594000	1.75120400
H	-1.91763900	4.10366100	0.47011200
H	-2.22837700	2.39298900	0.05367000
H	4.70220900	-0.80450000	0.98830100
C	2.79346700	0.95550200	1.10149200
H	3.11608500	1.71236100	0.37632400
H	1.86073200	1.30889700	1.53658600
H	3.54212500	0.91389700	1.89855900
C	0.38896400	-0.38149500	2.73110000
C	-0.10539800	-0.94060600	3.90925900
C	-1.46983400	-0.92969900	4.17451200
C	-2.34173000	-0.37675000	3.23655000
C	-1.83816400	0.15195400	2.05564700
C	-0.45601900	0.17992900	1.75815900
H	1.45803400	-0.43686900	2.56234800
H	0.58379100	-1.38698500	4.61963300
H	-1.85412800	-1.35728400	5.09567100
H	-3.41222600	-0.37597100	3.41798600
H	-2.54337800	0.52580700	1.31821100

N1_P

C	-0.05234500	2.55181500	-1.02016700

C	0.32086800	1.21001900	-1.11443700
C	1.05681300	0.93074400	-2.28833600
C	1.34606200	1.87589900	-3.26034800
C	0.92013400	3.19692500	-3.11092200
C	0.21473800	3.53906100	-1.96987400
H	1.41777600	-0.08493700	-2.42543500
H	1.91207900	1.58690500	-4.14055900
H	1.14157700	3.94569800	-3.86369600
H	-0.12066600	4.56234900	-1.81958400
H	-0.80556000	2.15035200	0.80722400
B	0.09082800	-0.06843800	-0.03747100
N	-0.81649300	2.98144400	0.17522800
C	-1.23034400	-1.02664100	-0.42049800
C	-1.57405100	-2.05497500	0.50712600
C	-2.13556500	-0.85462600	-1.49871100
C	-2.73508000	-2.81143900	0.36803000
C	-3.30465800	-1.62757800	-1.59523300
C	-3.63404300	-2.60757500	-0.67377200
C	1.59487200	-0.79802700	-0.05056300
C	1.86390300	-2.11248900	-0.51574200
C	2.73834700	-0.05726600	0.36371200
C	3.14230100	-2.67262900	-0.39917900
C	3.99690600	-0.65221200	0.48085000
C	4.21904100	-1.97929400	0.13578600
C	0.85260400	-2.99043900	-1.22707200
H	0.17875000	-3.50910300	-0.53973800
H	0.22129400	-2.42263500	-1.91289700
H	1.38258700	-3.75253400	-1.80508600
C	5.56812100	-2.62627200	0.31191500
H	5.72950900	-3.41303300	-0.42976800
H	6.37486000	-1.89447500	0.21511300
H	5.65878800	-3.08554400	1.30235500
C	-1.92866900	0.08926500	-2.67120700
H	-1.80761100	1.13798000	-2.39154200
H	-1.03850400	-0.17657600	-3.24778200
H	-2.78456600	0.02690800	-3.34815400
C	-4.88563500	-3.43629100	-0.80277200
H	-5.46792900	-3.42346100	0.12376700
H	-5.52394100	-3.06662500	-1.60928700
H	-4.64587200	-4.48249200	-1.01942200
C	-0.72272700	-2.42495000	1.70881100
H	0.33171500	-2.18318300	1.57737300
H	-1.06864500	-1.90845900	2.61152700
H	-0.80319600	-3.50026700	1.89601900
H	3.29745000	-3.69029100	-0.75426200
H	-2.94322800	-3.58795500	1.10364400
H	-3.97372700	-1.45774500	-2.43718600
C	-0.18811100	4.08169300	0.95209300
H	-0.69456000	4.14593700	1.91722900
H	0.86514900	3.84208700	1.09410500
H	-0.28956100	5.02112300	0.40812500
C	-2.24797300	3.24644400	-0.12453000
H	-2.77477200	3.41897000	0.81603000
H	-2.31758100	4.11962400	-0.77440800
H	-2.65512700	2.36830100	-0.62740600
H	4.83400500	-0.04812400	0.82873300
C	2.73334300	1.44421100	0.59881400
H	2.92382900	1.97041500	-0.34476400
H	1.78981900	1.81678600	0.99186300
H	3.52426500	1.72266900	1.30216600
C	0.55410100	0.51848700	2.62604400
C	0.14092800	0.91150900	3.89751500
C	-1.18788000	1.25164700	4.13887200
C	-2.10146100	1.15194500	3.09448000
C	-1.67346800	0.73400700	1.83279600
C	-0.32335900	0.43902000	1.52739500
H	1.59045200	0.22161700	2.49516900
H	0.86291600	0.94232300	4.70872000
H	-1.50993100	1.56000300	5.12868700
H	-3.15408500	1.36241800	3.26521200
H	-2.42272900	0.59712600	1.05342000

N2_TS

C	-0.86962400	2.51149900	-0.71137600
C	-0.05570600	1.43195500	-1.08942900
C	0.60907700	1.58596000	-2.31643100
C	0.47909300	2.73024100	-3.09826700
C	-0.32132600	3.78642800	-2.67086000
C	-1.00180600	3.67564100	-1.46439800
H	1.25454500	0.78323000	-2.66155900
H	1.01038400	2.80088900	-4.04252300
H	-0.42015400	4.68428500	-3.27231800
H	-1.64263600	4.48317600	-1.12030800
H	-0.81206500	1.30555300	1.06199600
B	0.04236300	0.09497100	-0.15035100
N	-1.62232100	2.34546900	0.52754300
C	-1.34755000	-0.75067900	-0.27744600
C	-2.31082900	-0.82215800	0.73909400

C	-1.66484200	-1.43196600	-1.47409900
C	-3.51084800	-1.52649000	0.64609000
C	-2.86245700	-2.14551900	-1.57264200
C	-3.79252000	-2.21684000	-0.53876300
C	1.45197800	-0.72123900	-0.27373900
C	1.62882100	-2.10381600	-0.04516400
C	2.61761500	0.00927900	-0.56248200
C	2.90883700	-2.66104300	-0.15817500
C	3.89106700	-0.54004500	-0.67171100
C	4.04124500	-1.91961300	-0.47369000
C	5.39330300	-2.57396700	-0.57963900
H	6.10168200	-2.14880800	0.14036400
H	5.32540600	-3.64778500	-0.39159000
H	5.83059600	-2.43243600	-1.57448900
C	-5.06623100	-3.00563100	-0.69226600
H	-5.11930200	-3.47831000	-1.67544500
H	-5.14305800	-3.79362500	0.06529000
H	-5.95032600	-2.36817800	-0.57683400
C	-1.50903000	3.46731500	1.47051900
H	-1.96528100	3.16832300	2.41805700
H	-0.45884900	3.70031500	1.64366300
H	-2.02361100	4.35939300	1.09600800
C	-3.04486800	2.06854000	0.24426800
H	-3.54143100	1.73918800	1.16104400
H	-3.52900700	2.98166300	-0.12523500
H	-3.12020700	1.28290800	-0.50525400
C	0.51369400	-3.05387600	0.33043000
H	-0.15547300	-2.63214200	1.08218300
H	-0.11303000	-3.31211100	-0.52960300
H	0.93389800	-3.98186300	0.72724500
C	-0.75004600	-1.42166900	-2.67577400
H	-0.82395600	-0.47145900	-3.21545500
H	0.29760900	-1.55094400	-2.38879500
H	-1.01847100	-2.22170800	-3.37066800
C	-4.48607900	-1.53598000	1.79474800
H	-5.46130500	-1.13178800	1.49922600
H	-4.66486800	-2.55231500	2.16345600
H	-4.11290500	-0.93897100	2.63104500
C	5.08225200	0.32637100	-0.98921400
H	5.83954300	0.27458900	-0.19860000
H	5.57255900	0.01170900	-1.91760300
H	4.78573600	1.37158600	-1.10378900
H	3.02438100	-3.72942000	0.01858700
H	2.53404800	1.08625100	-0.69810900
H	-3.07740100	-2.67263600	-2.50113500
H	-2.15097500	-0.27213900	1.66419300
C	1.01276900	-0.13041600	3.75271600
C	0.16780700	-0.17990800	2.65033300
C	0.26830300	0.74933000	1.59661900
C	1.24908300	1.75407600	1.73513100
C	2.09049900	1.82069000	2.83764400
C	1.97577300	0.87048800	3.85071100
H	0.92310200	-0.87722000	4.53532800
H	-0.55711800	-0.98458700	2.59426000
H	1.36858400	2.48154300	0.93377700
H	2.84245700	2.60080800	2.90472700
H	2.63660900	0.90816200	4.71137700

N2_P

C	-1.06941600	2.09408400	-1.16979300
C	0.04106200	1.24000900	-1.13452700
C	0.91760800	1.42975600	-2.21935400
C	0.70810100	2.37429700	-3.21820900
C	-0.41374200	3.19871700	-3.18815000
C	-1.32158200	3.05114500	-2.14922600
H	1.79404100	0.79270700	-2.28236500
H	1.42371400	2.46519100	-4.02964800
H	-0.58499700	3.93946700	-3.96159600
H	-2.21234200	3.67304000	-2.11380100
H	-1.70789900	1.25309200	0.55126300
B	0.24978600	0.07137300	0.05004100
N	-2.10566200	1.94208300	-0.10596300
C	-1.10149600	-0.88498900	-0.07562800
C	-2.02996000	-1.02381500	0.97112800
C	-1.45348700	-1.52083300	-1.29086800
C	-3.23589500	-1.72553900	0.87722200
C	-2.65383900	-2.23031800	-1.38925000
C	-3.56016700	-2.34458800	-0.33515200
C	1.70606300	-0.68972700	-0.09531900
C	1.98311300	-2.07218300	-0.04238400
C	2.82944000	0.15607300	-0.16343600
C	3.31182800	-2.51342300	-0.12721400
C	4.14838900	-0.27538500	-0.24686700
C	4.39803600	-1.65580300	-0.24297400
C	5.80391600	-2.18906600	-0.33331600
H	6.42122000	-1.83880000	0.50191900
H	5.81050600	-3.28139700	-0.32042700
H	6.30034400	-1.85952900	-1.25321400

C	-4.84119600	-3.12119700	-0.49294800
H	-4.93023500	-3.53229200	-1.50083300
H	-4.89176400	-3.95479200	0.21637600
H	-5.72031100	-2.49405000	-0.30496500
C	-2.37726200	3.18723500	0.66504200
H	-3.05792500	2.94141600	1.48141600
H	-1.43703200	3.56005500	1.06430500
H	-2.83819100	3.92051000	0.00417500
C	-3.37111600	1.35822800	-0.64664100
H	-4.03404500	1.12920600	0.18894000
H	-3.82475800	2.09473800	-1.31074800
H	-3.12561300	0.44429800	-1.18750400
C	0.93002900	-3.14718800	0.12946200
H	0.20625800	-2.89881800	0.90870000
H	0.35569700	-3.31915900	-0.78666400
H	1.40755700	-4.09320400	0.39956200
C	-0.57273000	-1.46458500	-2.51829000
H	-0.77116900	-0.56311200	-3.10929800
H	0.48720900	-1.44822400	-2.25234800
H	-0.75776100	-2.33061100	-3.16047800
C	-4.16749800	-1.81398300	2.05893300
H	-5.15890000	-1.40694000	1.82588400
H	-4.32219700	-2.85319600	2.36991500
H	-3.76734800	-1.26596300	2.91524100
C	5.28380300	0.71208100	-0.32673300
H	5.98711200	0.58530700	0.50418200
H	5.86085900	0.58835500	-1.25065500
H	4.91087300	1.73844000	-0.29725900
H	3.50286800	-3.58523300	-0.09143700
H	2.66104900	1.23342400	-0.13572400
H	-2.89321500	-2.71926000	-2.33314000
H	-1.81315000	-0.55664500	1.93118300
C	0.59143200	0.62198000	3.95489300
C	0.39850900	0.05481100	2.70114700
C	0.36765600	0.82076400	1.51894000
C	0.60577700	2.19427200	1.68080700
C	0.80905600	2.78155100	2.93335700
C	0.78684300	1.99810800	4.07985500
H	0.60392000	-0.00936500	4.83889800
H	0.28588300	-1.02552000	2.62505000
H	0.67206000	2.82745900	0.79583400
H	0.99918500	3.84939600	3.00686800
H	0.94155000	2.44564900	5.05689000

N3_TS

C	1.15242100	2.55562300	-0.98133700
C	0.32312600	1.45162900	-1.22438900
C	-0.16431200	1.31586800	-2.53146800
C	0.13005800	2.24112900	-3.52554600
C	0.92494800	3.34923200	-3.23865200
C	1.44233600	3.50621000	-1.95912700
H	-0.79191900	0.46189700	-2.77040000
H	-0.26813400	2.10422700	-4.52586700
H	1.14847100	4.08021200	-4.00886400
H	2.08209200	4.35362900	-1.72729500
H	0.80087900	1.71383400	1.04069600
B	0.00504300	0.37720500	-0.05637600
N	1.75339700	2.64662700	0.34284600
C	-1.41843200	-0.40631800	-0.20360500
C	-1.62149400	-1.71896800	0.22321500
C	-2.57009800	0.22314600	-0.67782500
C	-2.84294900	-2.37609300	0.14880500
C	-3.81071000	-0.39501900	-0.76945600
C	-3.94874800	-1.71088100	-0.35724200
C	1.29117700	-0.62997400	0.06263000
C	1.45097000	-1.56269100	-0.96442300
C	2.31630000	-0.64388200	0.99926800
C	2.51243600	-2.44899500	-1.05484300
C	3.40670700	-1.50780000	0.94985700
C	3.50567300	-2.42213700	-0.08424000
C	3.18264700	2.28999400	0.29756700
H	3.56235300	2.17755900	1.31436500
H	3.30249000	1.35096900	-0.24256400
H	3.74993000	3.07129700	-0.22444600
C	1.58261600	3.94557000	1.00512500
H	1.92336200	3.85417100	2.03979100
H	2.16430600	4.73272100	0.51073100
H	0.52966900	4.22730600	1.00368000
F	4.35482100	-1.44760900	1.88532600
F	4.53796500	-3.25759100	-0.15286000
F	2.59160500	-3.31887300	-2.05962700
F	0.52527500	-1.64233400	-1.93127700
F	2.33539100	0.23162200	2.02881300
F	-0.61998000	-2.42650800	0.76774700
F	-2.96145100	-3.63509900	0.56974600
F	-5.12851600	-2.32067700	-0.42991100
F	-4.86616300	0.27093700	-1.23804100
F	-2.53989700	1.51128600	-1.05389500

C	-1.36080600	0.66715700	3.60299700
C	-0.36809300	0.48493700	2.65172900
C	-0.30836400	1.28976200	1.49431500
C	-1.27034500	2.31584300	1.36453300
C	-2.25775800	2.51211000	2.31949700
C	-2.30670900	1.67895800	3.43625500
H	-1.40441100	0.02121900	4.47357000
H	0.34675000	-0.31859200	2.78344100
H	-1.25968500	2.93106800	0.46864000
H	-2.99619800	3.29634600	2.19071700
H	-3.08597400	1.81687800	4.17972900

N3_P

C	1.35541000	-2.07251100	1.45960000
C	0.28807800	-1.17441000	1.36550800
C	-0.36787200	-0.93770800	2.58600000
C	-0.00899300	-1.56574000	3.77170900
C	1.04404000	-2.47773600	3.79614100
C	1.74369900	-2.72845100	2.62492300
H	-1.18784600	-0.22653100	2.59612800
H	-0.55435600	-1.34490400	4.68363200
H	1.32635400	-2.97856800	4.71562000
H	2.58462500	-3.41657500	2.63145200
H	1.72713000	-1.80202200	-0.52321200
B	-0.11294100	-0.37473700	-0.02467000
N	2.20442400	-2.29021200	0.25314000
C	-1.61810500	0.28766700	0.05691500
C	-1.96361000	1.54015300	-0.44416900
C	-2.70515100	-0.46968200	0.49170500
C	-3.26226900	2.03416900	-0.46557500
C	-4.01813800	-0.01698400	0.49652000
C	-4.30042900	1.25133800	0.01384200
C	1.07274700	0.78131100	-0.15257800
C	1.05761500	1.87290600	0.71948700
C	2.18946700	0.75084300	-0.97792800
C	2.02606000	2.86396600	0.74916100
C	3.19702200	1.71150900	-0.98372900
C	3.11066500	2.78657400	-0.11696500
C	3.55616400	-1.67691200	0.42839100
H	4.06647700	-1.66684700	-0.53469900
H	3.42237100	-0.66211700	0.79948400
H	4.10875600	-2.27035400	1.15671400
C	2.31970700	-3.71755100	-0.16596300
H	2.86664300	-3.75161100	-1.10907000
H	2.85986700	-4.26871000	0.60259000
H	1.31842500	-4.11970600	-0.30041600
F	4.24824200	1.58203900	-1.79638500
F	4.05982900	3.71801400	-0.09530500
F	1.93712000	3.88150700	1.60364300
F	0.05378900	2.00512400	1.59624100
F	2.41973000	-0.30568500	-1.80226000
F	-1.02967000	2.35506400	-0.96453800
F	-3.51878700	3.24879800	-0.95301100
F	-5.55123800	1.70816500	0.00244500
F	-5.00602700	-0.79414700	0.94442900
F	-2.51883300	-1.72602500	0.93487500
C	-0.41323600	-1.65117400	-3.74703500
C	-0.16756000	-0.85682700	-2.63376700
C	-0.24289200	-1.36443800	-1.32567400
C	-0.62123200	-2.70937700	-1.19574800
C	-0.86785600	-3.51968200	-2.30596400
C	-0.75553600	-2.99402000	-3.58778100
H	-0.34219300	-1.22383800	-4.74286400
H	0.07830100	0.19384100	-2.77817200
H	-0.76088200	-3.12353800	-0.19811700
H	-1.16453400	-4.55559400	-2.16553000
H	-0.94955900	-3.61684800	-4.45553600

N4_TS

C	-2.69919300	-1.14187600	-0.10176100
C	-1.46414300	-1.18958500	-0.75110200
C	-1.07263800	-2.44140500	-1.25022300
C	-1.87211900	-3.56945900	-1.11134400
C	-3.10118100	-3.48408500	-0.45713200
C	-3.51599800	-2.26115200	0.05350400
H	-0.11529700	-2.52877500	-1.75931600
H	-1.53721500	-4.52157600	-1.51132900
H	-3.72725300	-4.36347500	-0.34611000
H	-4.46754700	-2.17083100	0.57231100
H	-1.81164800	0.89618800	-0.04313900
B	-0.49433700	0.07193900	-1.00463400
N	-3.08584000	0.14788300	0.42258000
C	0.99441100	-0.19075100	-0.45776800
C	2.12325300	0.00924500	-1.24499600
C	1.23721900	-0.68703400	0.81966900
C	3.41279300	-0.26419400	-0.79922300
C	2.50401500	-0.96290100	1.30932200
C	3.60224100	-0.75343000	0.48439700

C	-4.25395900	0.74020500	-0.22111500
H	-4.33799300	1.78703900	0.08730000
H	-4.12232600	0.69765300	-1.30454600
H	-5.18270200	0.21567500	0.04326500
C	-3.14032700	0.20533100	1.88131300
H	-3.19499200	1.25324600	2.19404800
H	-4.00952500	-0.33316000	2.28374800
H	-2.22794100	-0.24062000	2.28240100
F	2.00899500	0.48074100	-2.49053000
F	4.46565500	-0.06183400	-1.59093300
F	4.82949900	-1.01477000	0.92728500
F	2.68093400	-1.42112500	2.54909800
F	0.20446600	-0.88150600	1.66442600
H	-0.52247100	0.42730300	-2.14747500
C	-0.88354600	4.08295000	-0.88938600
C	-1.22765700	2.75885200	-1.13390800
C	-0.95488000	1.75316200	-0.19204000
C	-0.36909800	2.13067800	1.03041800
C	-0.02253000	3.45181400	1.27948600
C	-0.27507800	4.42795400	0.31573900
H	-1.08086400	4.84456700	-1.63672100
H	-1.69174700	2.48569600	-2.07731300
H	-0.17808000	1.37139300	1.78349600
H	0.44425900	3.72469200	2.22042500
H	0.00045900	5.46064300	0.50684800

N4_P

C	-2.29768800	-1.30786300	-0.05355100
C	-1.29783400	-1.06719900	-0.99595800
C	-1.11829300	-2.11959600	-1.91334400
C	-1.86239500	-3.29141400	-1.88060400
C	-2.85269400	-3.46923900	-0.91383300
C	-3.07458100	-2.46225400	0.01328300
H	-0.35838000	-1.99163500	-2.67944300
H	-1.67883400	-4.07083800	-2.61370800
H	-3.44345500	-4.37823800	-0.88304700
H	-3.83731400	-2.58312200	0.77853100
H	-1.91708000	0.51828300	0.70236700
B	-0.32631800	0.25495200	-1.14695100
N	-2.54241100	-0.26492900	0.97559600
C	1.16533300	-0.02598500	-0.53321600
C	2.24143000	0.72699100	-1.00491200
C	1.49785500	-0.93108300	0.46548700
C	3.54425500	0.58229400	-0.54676200
C	2.78563500	-1.11458600	0.95836400
C	3.81998700	-0.35063800	0.44420900
C	-3.92118000	0.29203700	0.94241700
H	-3.95303800	1.16916100	1.59102900
H	-4.14158500	0.58073800	-0.08411700
H	-4.62422000	-0.46687500	1.28682600
C	-2.13039300	-0.68128500	2.34407500
H	-2.20703800	0.18384500	3.00533200
H	-2.79052500	-1.47991700	2.68473600
H	-1.10344800	-1.03859800	2.29003400
F	2.04237000	1.65733600	-1.94550400
F	4.53315200	1.33024800	-1.03921600
F	5.06378900	-0.50248700	0.89849200
F	3.02729200	-2.00836200	1.92158100
F	0.54628400	-1.70305800	1.04141200
H	-0.20581100	0.42130000	-2.34173900
C	-2.67165600	3.37453600	-0.58600600
C	-2.04993900	2.25591900	-1.13348600
C	-0.95255300	1.62658400	-0.51208300
C	-0.51040500	2.20119700	0.69356500
C	-1.13049400	3.31866200	1.25920700
C	-2.21780700	3.90751300	0.62219900
H	-3.50765800	3.83917300	-1.10167100
H	-2.41072600	1.85137000	-2.07769700
H	0.34602000	1.75966000	1.20111200
H	-0.75432100	3.73455300	2.18975800
H	-2.69959300	4.78024800	1.05207800

N5_TS

B	-0.37095300	-0.08206600	-0.19334500
C	0.63672500	-0.50454700	-1.40134400
C	-0.00335900	-0.84311700	-2.60743500
H	-1.08797700	-0.77446400	-2.65253900
C	0.67945600	-1.24988700	-3.74231900
H	0.13450500	-1.49341300	-4.64867100
C	2.06690500	-1.34604900	-3.70374500
H	2.62663800	-1.66691500	-4.57634500
C	2.73721200	-1.02068200	-2.53426000
H	3.81611700	-1.09167700	-2.51226800
C	2.04148300	-0.60069600	-1.39074100
N	2.76817100	-0.24918000	-0.17232400
C	3.61945200	-1.35186700	0.44143400
C	4.00983800	-0.87437000	1.85032500
H	3.09419300	-0.75412000	2.44571900

H	4.59895900	-1.66569900	2.32612700
C	4.76999000	0.44393600	1.83381100
H	5.71743300	0.33107900	1.29261400
H	5.02954600	0.74535800	2.85327500
C	3.89278400	1.50911800	1.18715800
H	4.42334100	2.46529200	1.11445800
H	3.02233600	1.67537000	1.82969200
C	3.41701500	1.13373000	-0.22717900
C	4.59997000	1.30072500	-1.20403900
H	5.54337500	0.90650900	-0.82794900
H	4.74728900	2.37497600	-1.35087200
H	4.38926300	0.87214000	-2.18453300
C	2.36343600	2.13822300	-0.69320700
H	1.42203400	2.01819000	-0.16261700
H	2.15664300	2.03509100	-1.75973500
H	2.73426900	3.15081800	-0.50828100
C	4.88281100	-1.77257900	-0.34339700
H	4.62224000	-2.42539400	-1.17855200
H	5.51114500	-2.36037400	0.33333500
H	5.48963500	-0.95556300	-0.72313300
C	2.79280500	-2.62812400	0.58125400
H	1.96119600	-2.49915300	1.26813200
H	3.44127400	-3.41122400	0.98539400
H	2.40706500	-2.96647300	-0.38170300
C	-0.99652600	1.42611700	-0.30055900
C	-0.81993100	2.31018000	-1.36236000
F	-0.07676700	1.99164700	-2.43051700
C	-1.37333100	3.58929600	-1.39963300
F	-1.14860900	4.38825300	-2.44292200
C	-2.15818000	4.02919300	-0.34972700
F	-2.69469000	5.24530300	-0.36856400
C	-2.38490500	3.17907900	0.72611900
F	-3.15716500	3.57902000	1.73645700
C	-1.82048500	1.91639800	0.71795000
F	-2.11907300	1.12888000	1.75992300
C	-1.55865000	-1.22952300	-0.10902600
C	-2.93869600	-1.01615300	-0.18204300
F	-3.47673700	0.20533500	-0.27868600
C	-3.88395800	-2.04053400	-0.19947900
F	-5.18139100	-1.74685900	-0.27009200
C	-3.47964300	-3.36231300	-0.16849000
F	-4.36995100	-4.34867500	-0.18449100
C	-2.12123700	-3.63747700	-0.12767500
F	-1.69307100	-4.89949700	-0.10913900

C	-1.21963800	-2.58552200	-0.11957100
F	0.07498800	-2.94825100	-0.09386400
H	1.47090000	-0.15029300	0.71132600
C	0.50135200	-0.04463000	1.52418100
C	0.21743900	-1.16407600	2.34156400
C	0.04035000	-1.06152100	3.71444500
C	0.16734300	0.17525600	4.34120200
C	0.45768600	1.30694600	3.58184700
C	0.61064500	1.19085500	2.20950000
H	0.11506400	-2.14312900	1.89213900
H	-0.19784600	-1.94626400	4.29532400
H	0.03376600	0.25916600	5.41548600
H	0.55130700	2.27728700	4.05812700
H	0.80911700	2.09873600	1.64874800

N5_P

B	0.46751600	-0.06955800	-0.04492900
C	-0.48921800	-0.48533600	1.27885800
C	0.24995600	-0.76397300	2.44984300
H	1.33065400	-0.66663000	2.39841900
C	-0.31240300	-1.13414500	3.65863700
H	0.32429800	-1.32828400	4.51585600
C	-1.69510600	-1.25725400	3.76674900
H	-2.16147200	-1.55612600	4.69935500
C	-2.47591700	-0.98538400	2.65703200
H	-3.55055100	-1.07649000	2.73147100
C	-1.87667100	-0.60302300	1.45046100
N	-2.78775100	-0.29827200	0.31179400
C	-3.61463900	-1.49916500	-0.24290800
C	-4.11901300	-1.03044100	-1.61534300
H	-3.25013300	-0.86435400	-2.26947200
H	-4.69275000	-1.85224300	-2.05555500
C	-4.95310000	0.24463800	-1.54190300
H	-5.85697100	0.07680000	-0.94381800
H	-5.29152000	0.52195200	-2.54420300
C	-4.11354300	1.37963200	-0.96217700
H	-4.70444300	2.29692500	-0.86863400
H	-3.29073200	1.59664100	-1.65254600
C	-3.52705100	1.07789000	0.42497300
C	-4.62040100	1.15077800	1.49403500
H	-5.55184500	0.66196700	1.21329400
H	-4.84746700	2.21312500	1.61963100
H	-4.28511600	0.78418700	2.46365500
C	-2.46718800	2.11530100	0.78648400

H	-1.58709700	2.04077500	0.14718100
H	-2.13961100	2.01637300	1.82190700
H	-2.90402800	3.10808200	0.64729200
C	-4.76550300	-1.96029200	0.65485800
H	-4.39527800	-2.48218600	1.53766500
H	-5.33771200	-2.68800800	0.07210200
H	-5.45518400	-1.18100900	0.96477000
C	-2.67468600	-2.68682900	-0.41615300
H	-1.85427600	-2.46168700	-1.09428300
H	-3.25674300	-3.50799300	-0.84448000
H	-2.25910000	-3.01630300	0.53729300
C	1.04762600	1.47538400	0.14968600
C	0.83681200	2.34788500	1.21353600
F	0.11664000	1.99475800	2.29512900
C	1.32046100	3.65517200	1.25787200
F	1.06051400	4.43270600	2.31204300
C	2.06955800	4.14594100	0.20561800
F	2.54382200	5.38970200	0.23081800
C	2.32213300	3.31747900	-0.88050000
F	3.05344600	3.76813900	-1.90043700
C	1.81910900	2.02740500	-0.87951400
F	2.13104000	1.27777400	-1.94413900
C	1.71954700	-1.17632400	-0.00379900
C	3.09146500	-0.91975400	0.01070800
F	3.58868300	0.32449100	0.00335900
C	4.06862700	-1.91249100	0.06814400
F	5.35961500	-1.58032400	0.07864900
C	3.70353000	-3.24460100	0.13623500
F	4.62529300	-4.20275000	0.19748700
C	2.35376400	-3.56113200	0.14787300
F	1.96554600	-4.83536400	0.23013100
C	1.42096200	-2.53758200	0.08861000
F	0.13313100	-2.94009400	0.12275400
H	-2.12633200	-0.12407000	-0.46455900
C	-0.23420200	-0.12582100	-1.55730100
C	-0.23871200	-1.28222600	-2.36141100
C	-0.77303200	-1.30994700	-3.64788200
C	-1.32919200	-0.16185200	-4.20617100
C	-1.32112500	1.01027700	-3.45859200
C	-0.78032500	1.01760400	-2.17200400
H	0.19779700	-2.19992400	-1.98311200
H	-0.74025100	-2.23227300	-4.22059300
H	-1.73718100	-0.17619400	-5.21194100
H	-1.71427600	1.93158100	-3.88008500
H	-0.74069000	1.97284600	-1.65399600

N6_TS

C	-1.27161100	-1.80256900	-0.17093500
C	-1.66576500	-0.46804700	-0.00896200
C	-3.01301300	-0.11849600	0.14768700
C	-3.99337000	-1.10266000	0.15095600
C	-3.62718800	-2.43858300	0.00359500
C	-2.28859200	-2.77058700	-0.15076500
H	-3.30819700	0.91621500	0.26344600
H	-5.03565700	-0.82493800	0.27110500
H	-4.38569900	-3.21580400	0.01182100
H	-2.00728700	-3.81474900	-0.26366500
C	-0.49655800	1.36479100	-1.25192300
C	-0.37758600	1.21374000	1.33225100
C	0.85571400	2.09909300	-1.22813400
C	0.99885900	1.89792800	1.26532000
C	1.11852800	2.84887200	0.07575400
H	0.87805500	2.78737000	-2.08073100
H	1.66057400	1.37500800	-1.38185800
H	1.15797800	2.43589600	2.20662400
H	1.78045600	1.13283700	1.19603000
H	2.12205800	3.28488900	0.05031100
H	0.42244700	3.68854300	0.18496100
N	-0.58515000	0.51504000	0.00148100
C	-1.62617100	2.39139600	-1.44749800
H	-2.61126200	1.93066600	-1.36629500
H	-1.57119800	3.24344700	-0.77189700
H	-1.53764500	2.78308900	-2.46576300
C	-0.56214000	0.42353300	-2.46276200
H	-0.21578900	0.96298000	-3.34958000
H	0.04820400	-0.46958100	-2.33096400
H	-1.58746600	0.08970100	-2.64281800
C	-1.43518100	2.25373400	1.74892700
H	-2.43436900	1.82066200	1.79522000
H	-1.18247900	2.57676600	2.76425500
H	-1.46306900	3.14604600	1.12719800
C	-0.39740400	0.14342700	2.43159500
H	0.13668900	-0.75663400	2.13752200
H	0.06700300	0.54909900	3.33524300
H	-1.42212200	-0.15138400	2.67115000
B	0.22282300	-2.32696400	-0.45191700
H	0.54032900	-0.50559500	-0.09577500
H	0.38077900	-2.56454600	-1.62574500

H	0.50227600	-3.25843400	0.25838700
C	3.69861700	-1.28754900	1.22128000
C	4.46673400	-0.93666000	0.11276000
C	3.85009000	-0.76326900	-1.12345100
C	2.47312200	-0.91601200	-1.23650900
C	1.65479400	-1.23072000	-0.13313300
C	2.32299700	-1.43084200	1.09318400
H	4.17456600	-1.46243200	2.18091500
H	5.54199200	-0.81969400	0.20851100
H	4.44245300	-0.52097600	-2.00005100
H	2.02040500	-0.80277600	-2.21709500
H	1.75865700	-1.74923500	1.96333900

N6_P

C	-1.30293400	-1.73011000	-0.33309100
C	-1.63647200	-0.41286600	0.01560500
C	-2.91944000	0.00270000	0.38434500
C	-3.94725800	-0.92710200	0.43224600
C	-3.67171400	-2.25607600	0.11389500
C	-2.38758600	-2.62929900	-0.25399900
H	-3.12706500	1.03567600	0.63219800
H	-4.94599800	-0.61603800	0.71962500
H	-4.46271500	-2.99907500	0.15604700
H	-2.18638100	-3.66693600	-0.50336200
C	-0.52882200	1.56060200	-1.20387900
C	-0.11557300	1.14674000	1.38787200
C	0.86810200	2.20077700	-1.24900400
C	1.29259300	1.72239800	1.18099600
C	1.34399700	2.78181100	0.08108500
H	0.84659800	2.97297000	-2.02557300
H	1.58545100	1.43895700	-1.57325600
H	1.62566400	2.13998100	2.13686400
H	1.98034700	0.89969800	0.93645400
H	2.37257400	3.13610300	-0.03335800
H	0.74688500	3.65747200	0.36107100
N	-0.52748600	0.58121600	0.00055600
C	-1.62747400	2.62148300	-1.15234200
H	-2.61218000	2.18177300	-0.99127200
H	-1.45536500	3.41054400	-0.42243600
H	-1.64721000	3.09511700	-2.13805800
C	-0.74977900	0.71356500	-2.45841100
H	-0.52847500	1.33582800	-3.33017800
H	-0.10649900	-0.16860400	-2.48119300
H	-1.78469500	0.37009100	-2.52718800
C	-1.05980000	2.19877300	1.97136300
H	-2.07052300	1.81397500	2.10273200
H	-0.67641200	2.43017200	2.96955400
H	-1.09694400	3.13395900	1.41867800
C	-0.07354300	-0.02752100	2.36561000
H	0.49765900	-0.86348300	1.96446400
H	0.41780300	0.31436700	3.28101600
H	-1.07838300	-0.37431600	2.61555900
B	0.09964400	-2.36058300	-0.95297200
H	0.28124200	-0.02772200	-0.22119200
H	-0.00441200	-2.25340300	-2.16663900
H	0.08330900	-3.53922700	-0.65744800
C	3.36766700	-1.63718700	1.16075100
C	4.13149300	-0.83918200	0.30741000
C	3.63098600	-0.52888000	-0.95282400
C	2.36856300	-0.99084200	-1.33646500
C	1.54914600	-1.76080900	-0.49202800
C	2.11386300	-2.08696100	0.75693800
H	3.75921000	-1.91936100	2.13445800
H	5.11226100	-0.48806700	0.61368600
H	4.22737200	0.06016300	-1.64516600
H	2.01761200	-0.77154300	-2.34426300
H	1.54735400	-2.73674800	1.42271100

N8_TS

C	4.70857400	-2.51068100	-1.83081700
H	4.87895100	-2.52377900	-2.91209900
H	4.86405300	-3.53281600	-1.47371200
C	5.65466100	-1.49853700	-1.12185100
H	6.21839100	-1.98002400	-0.31638300
H	6.38892700	-1.06938600	-1.80975000
C	5.00679400	0.73975300	0.08076500
H	6.02219900	1.06972600	0.27680200
C	3.91219100	1.56117700	0.43779300
H	4.11762200	2.53981200	0.86220600
C	2.60113400	1.19080000	0.22455600
C	0.93871700	-0.67314900	-0.52715300
C	0.93854900	-1.81893300	-1.32069600
H	-0.01871500	-2.27547200	-1.54676400
C	2.07470300	-2.49215700	-1.82990200
H	1.93995000	-3.38998400	-2.42702100
C	4.73241300	-0.43745500	-0.55965800
C	2.25157900	-0.08145800	-0.34156300
C	3.31512000	-2.01518000	-1.51209600

C	3.38626400	-0.81282900	-0.77940500
C	1.60075500	2.86296000	1.74151700
H	1.57088500	2.12918200	2.54654800
H	0.72865400	3.51714900	1.81048800
H	2.49911600	3.48467200	1.83641800
C	1.58739400	3.19298200	-0.63370600
H	2.48083100	3.82206700	-0.54402000
H	0.68735200	3.81212200	-0.56206600
H	1.59903000	2.69391600	-1.60334700
C	-0.93997100	1.52436400	-0.30422900
C	-1.68234900	2.44402200	0.50629800
C	-2.44348200	3.45848100	-0.07868800
H	-3.00139300	4.13307500	0.56778800
C	-2.48753000	3.65352500	-1.45433300
C	-1.72690500	2.80324600	-2.25325500
H	-1.73704900	2.94795600	-3.33137400
C	-0.97596000	1.76070000	-1.71964300
C	-1.69620800	2.45579800	2.01952900
H	-0.81378700	2.01604200	2.47567900
H	-2.55324100	1.88651100	2.39216500
H	-1.80624700	3.48553900	2.37265600
C	-3.33168400	4.73900400	-2.06354500
H	-2.84660800	5.17264400	-2.94163100
H	-3.53380800	5.53700100	-1.34571500
H	-4.29456700	4.33099100	-2.38842400
C	-0.27711300	0.88132400	-2.72728100
H	-0.44204400	1.26690100	-3.73614100
H	-0.67246300	-0.13844000	-2.67464100
H	0.79801600	0.80084500	-2.55737700
C	-1.72125400	-1.35666900	-0.18495400
C	-2.94189000	-1.16563100	-0.87904700
C	-3.81333400	-2.23885300	-1.13345900
H	-4.73385300	-2.03684900	-1.67783900
C	-3.55797600	-3.53187000	-0.71918600
C	-2.39166500	-3.72640700	0.01181000
H	-2.16703800	-4.72251300	0.39087200
C	-1.50286800	-2.69210400	0.29787300
C	-3.47534600	0.15421300	-1.40126500
H	-3.02071500	0.43295500	-2.35534600
H	-3.33525400	0.98098500	-0.70692500
H	-4.54924500	0.05971200	-1.58048500
C	-4.49163400	-4.67272900	-1.02750600
H	-5.42814700	-4.31105500	-1.45921000
H	-4.73128300	-5.24317400	-0.12507600

H	-4.04085300	-5.36993200	-1.74157400
C	-0.34609900	-3.13254700	1.18396000
H	-0.10929700	-4.18012500	0.97590200
H	-0.63445500	-3.05782100	2.23866800
H	0.56505100	-2.55517900	1.06104700
B	-0.50994400	-0.26670800	0.21294300
N	1.55701200	2.18014000	0.43920200
H	0.23415900	1.57283500	0.15911800
C	-0.41966700	-0.30182000	1.84841100
C	0.76802600	-0.25172300	2.59129800
C	0.78192700	-0.32069300	3.98573700
C	-0.40750100	-0.46487300	4.69156500
C	-1.60331400	-0.56206300	3.98254600
C	-1.59826100	-0.48969400	2.59375500
H	1.72327900	-0.18670600	2.07909200
H	1.72894100	-0.28026800	4.51752900
H	-0.40208800	-0.52421200	5.77574100
H	-2.54108000	-0.70281600	4.51280400
H	-2.54228000	-0.58529700	2.06120100

N8_P

C	4.03179200	-3.38513100	-1.82971100
H	4.21839900	-3.39674300	-2.90824700
H	3.95433400	-4.42833400	-1.51038200
C	5.15860700	-2.62894000	-1.06956300
H	5.59281600	-3.24936300	-0.27925000
H	5.98001300	-2.33685000	-1.73014900
C	4.99493500	-0.36410200	0.22905000
H	6.05248500	-0.27886300	0.45505400
C	4.10173400	0.66097600	0.61591000
H	4.50675600	1.54809100	1.09327400
C	2.75290000	0.57839700	0.36795700
C	0.70937700	-0.80218200	-0.51585500
C	0.49407400	-1.90571300	-1.34329700
H	-0.53273100	-2.14099100	-1.59941400
C	1.46509900	-2.79473400	-1.85570700
H	1.15036900	-3.62481100	-2.48223400
C	4.47602800	-1.41795700	-0.47129400
C	2.11761900	-0.53507300	-0.28116900
C	2.77431100	-2.60853100	-1.50908700
C	3.08493000	-1.47827800	-0.72992500
C	2.11941300	2.42183700	1.96601400
H	1.91188200	1.67367500	2.72978500
H	1.39975000	3.23937900	2.02954300

H	3.13118300	2.81386500	2.06055900
C	2.20765800	2.79695400	-0.45881900
H	3.23483300	3.14862200	-0.35796700
H	1.48811300	3.60920500	-0.35011800
H	2.06484900	2.29976800	-1.41645400
C	-0.77244300	1.54552100	-0.37819400
C	-1.00557200	2.71216200	0.41288500
C	-1.05247500	3.99141300	-0.16641300
H	-1.25101400	4.84681100	0.47737600
C	-0.88318800	4.20704100	-1.52711500
C	-0.66564800	3.08105200	-2.31245400
H	-0.55350400	3.20350600	-3.38891800
C	-0.61873700	1.79195200	-1.77642200
C	-1.29639200	2.71176900	1.90413800
H	-0.59213100	2.13542800	2.50437500
H	-2.28040000	2.28068000	2.10712000
H	-1.30514300	3.74032400	2.27654300
C	-0.97371100	5.58399300	-2.13163500
H	-0.21720300	5.72808900	-2.90807500
H	-0.84095500	6.36032700	-1.37407700
H	-1.95196800	5.74005700	-2.59783800
C	-0.49026100	0.68524300	-2.80215000
H	-0.80775100	1.05518000	-3.78090100
H	-1.11437400	-0.17141600	-2.54145400
H	0.53614900	0.31949200	-2.89804900
C	-2.06223300	-0.95830200	-0.21691000
C	-3.24600200	-0.47130600	-0.84017000
C	-4.30710700	-1.33093800	-1.15466300
H	-5.18447200	-0.91309100	-1.64617000
C	-4.29266100	-2.68529900	-0.85664700
C	-3.18506500	-3.15489100	-0.16626600
H	-3.15496700	-4.20104100	0.13566700
C	-2.11102500	-2.32931700	0.17767200
C	-3.53278000	0.98349800	-1.17052800
H	-3.01279400	1.33443800	-2.06562900
H	-3.25857700	1.65737300	-0.35784900
H	-4.60400200	1.10345400	-1.35167800
C	-5.44644000	-3.58261400	-1.22363100
H	-6.35311000	-3.30609200	-0.67546600
H	-5.22243600	-4.62727400	-0.99374800
H	-5.67913000	-3.51320100	-2.29077400
C	-1.07284200	-3.02189900	1.04567700
H	-1.01007100	-4.08070000	0.77513100
H	-1.37874200	-2.96380000	2.09725800

H	-0.07536200	-2.59895000	0.98329200
B	-0.68749800	-0.06379100	0.17348400
N	1.95698600	1.79424400	0.62439300
H	0.95512900	1.53340500	0.52539200
C	-0.61303200	-0.20565300	1.82964400
C	0.53944100	-0.44174500	2.59339400
C	0.51538000	-0.57354100	3.98632000
C	-0.68754500	-0.49495300	4.67491800
C	-1.86291700	-0.31261300	3.94582200
C	-1.81647800	-0.18304700	2.56379900
H	1.49842500	-0.57962400	2.10224400
H	1.44110300	-0.76301500	4.52423300
H	-0.71640400	-0.60212200	5.75499300
H	-2.82043400	-0.28138700	4.45859400
H	-2.75279600	-0.06453700	2.02277700

N10_TS

C	5.22239900	-1.35648700	-0.27497500
H	6.25140800	-1.62592000	-0.06288600
C	4.21815300	-1.59767400	0.68458700
H	4.48288400	-2.08376800	1.61806800
C	2.91078800	-1.25471600	0.44252800
C	1.15423300	-0.12734400	-1.02669600
C	0.96057900	0.42258200	-2.28758700
H	-0.02257700	0.81225500	-2.53248200
C	1.94886100	0.52998000	-3.28191900
H	1.69570900	0.96664300	-4.24311000
C	4.86705500	-0.81115500	-1.47511800
C	2.48781900	-0.61411700	-0.76731600
C	3.22453400	0.11926400	-3.01313500
C	3.52311000	-0.44236600	-1.75139100
C	1.52485300	-3.04496600	1.23938800
H	1.08888500	-3.18546000	0.24933500
H	0.79170400	-3.31588700	2.00133500
H	2.40924200	-3.68495500	1.33965300
C	2.28742100	-1.40611700	2.82458100
H	3.01345900	-2.15036000	3.16926800
H	1.38382100	-1.48549300	3.43608500
H	2.71219800	-0.40771200	2.93573100
C	-0.35181000	1.76115100	-0.11651200
C	-1.28794100	2.53437300	-0.85647500
C	-1.30011100	3.93120400	-0.75558700
H	-2.04596300	4.48170400	-1.32704400
C	-0.40305700	4.63979700	0.02996900

C	0.58798400	3.89952400	0.66210400
H	1.36357300	4.41907800	1.22302300
C	0.64597300	2.50900700	0.56569400
C	-2.31536800	1.99205300	-1.83625500
H	-2.03657700	1.04532900	-2.29522500
H	-2.46043700	2.72704300	-2.63291800
H	-3.28847300	1.83296200	-1.36159800
C	-0.48358400	6.13685100	0.17258000
H	0.50849600	6.57633600	0.30683300
H	-1.08685000	6.41679000	1.04300800
H	-0.94412800	6.59436200	-0.70681200
C	1.90175000	1.89252400	1.14685000
H	2.69393800	1.87698100	0.39009600
H	1.77143700	0.86809000	1.48421700
H	2.26274400	2.48185900	1.99586800
C	-1.55767900	-0.76008100	-0.70128700
C	-2.86695000	-0.46553700	-0.20660800
C	-3.98879800	-1.13459000	-0.68439300
H	-4.96647100	-0.86092600	-0.28961100
C	-3.90545500	-2.14391900	-1.64020500
C	-2.63745100	-2.48647900	-2.07382800
H	-2.53013000	-3.29730900	-2.79221700
C	-1.47666200	-1.83577700	-1.62283300
C	-3.16585800	0.53846500	0.89124900
H	-2.44620900	1.34984100	0.95970700
H	-3.18908900	0.03601800	1.86504100
H	-4.15639900	0.97489300	0.73216000
C	-5.14051800	-2.82910600	-2.16268800
H	-4.88468600	-3.71703900	-2.74578100
H	-5.71653600	-2.15885200	-2.80923000
H	-5.79953100	-3.13543100	-1.34475900
C	-0.20479800	-2.42227600	-2.21225800
H	-0.38717000	-3.46416100	-2.48985300
H	0.64484400	-2.39759200	-1.53416300
H	0.11418300	-1.89089100	-3.11410500
B	-0.26083900	0.10074300	-0.15504000
N	1.90607100	-1.63045800	1.42021000
H	0.70034300	-0.84238300	1.24865300
H	4.02188800	0.22520900	-3.74296300
H	5.61163900	-0.64054800	-2.24762500
C	-0.46535400	0.48128700	2.75535900
C	-1.14606900	0.26424000	3.95279600
C	-1.80509000	-0.93876100	4.17872400
C	-1.80364200	-1.91539400	3.18183400
C	-1.14859500	-1.67571200	1.98164500
C	-0.44534600	-0.47618300	1.72561600
H	0.02428300	1.43800400	2.61976000
H	-1.15744900	1.04212900	4.71001400
H	-2.32703000	-1.11162000	5.11494700
H	-2.33017800	-2.85271300	3.33282500
H	-1.20134600	-2.42831600	1.19984000

N10_P

C	5.25459700	-0.75102800	-0.97687500
H	6.32493900	-0.91677100	-0.93813700
C	4.42527700	-1.23555300	0.05297300
H	4.86084200	-1.80551900	0.86795200
C	3.07453500	-1.01015500	0.01256100
C	0.98102000	0.05110700	-1.04037800
C	0.57596200	0.67836600	-2.21456100
H	-0.47072900	0.94706100	-2.30443400
C	1.40602100	1.02312300	-3.29479400
H	0.97521200	1.51318800	-4.16234400
C	4.67019000	-0.10380500	-2.02820700
C	2.39143400	-0.28414000	-1.01601900
C	2.74878900	0.77748200	-3.22466100
C	3.26957300	0.12614500	-2.08612900
C	2.15501700	-3.12772800	0.82625300
H	1.72351700	-3.28606100	-0.16139200
H	1.51000300	-3.54885200	1.59708200
H	3.15486800	-3.55970800	0.87131600
C	2.72020600	-1.39310500	2.46438000
H	3.63418700	-1.95395700	2.65845100
H	1.92028600	-1.72014400	3.13138800
H	2.89677400	-0.32543600	2.57977200
C	-0.61091500	1.73527900	0.11072300
C	-1.68443300	2.47024600	-0.46622900
C	-1.79635300	3.85378000	-0.27531900
H	-2.64521500	4.36838800	-0.72340000
C	-0.87352700	4.59599600	0.44721900
C	0.23672200	3.90969700	0.92025400
H	1.02687000	4.46132600	1.42851300
C	0.38849500	2.53463800	0.73314500
C	-2.77538300	1.89833700	-1.35656300
H	-2.46632600	1.02927800	-1.93505000
H	-3.10174300	2.67377200	-2.05538600
H	-3.65395400	1.58926500	-0.78205400
C	-1.05674000	6.07147600	0.68893700

H	-1.59962000	6.25388800	1.62279100
H	-1.62867100	6.53676000	-0.11820700
H	-0.09380300	6.58391700	0.76450900
C	1.74328700	1.99813800	1.15186200
H	2.46924600	2.13778500	0.34254500
H	1.72439000	0.93732900	1.38459000
H	2.11710300	2.53164400	2.03211900
C	-1.64685700	-0.83525000	-0.57999600
C	-2.92460700	-0.73033700	0.05000300
C	-4.03552500	-1.41656500	-0.43383300
H	-4.99356000	-1.27969000	0.06615000
C	-3.96185600	-2.27640300	-1.52635000
C	-2.70691100	-2.47365700	-2.07613900
H	-2.59588300	-3.19011100	-2.88887100
C	-1.56381300	-1.80013800	-1.61552900
C	-3.18104900	0.08827200	1.30183500
H	-2.57289600	0.98930500	1.36646300
H	-2.97038000	-0.50977400	2.19654400
H	-4.23389000	0.38253400	1.34603400
C	-5.18625000	-2.97085500	-2.06283000
H	-4.91500000	-3.81286000	-2.70476200
H	-5.79940900	-2.28497400	-2.65708200
H	-5.81569300	-3.34865900	-1.25182800
C	-0.28130400	-2.26671300	-2.28461000
H	-0.41917000	-3.28762300	-2.65226200
H	0.56900200	-2.26656800	-1.60195000
H	0.01410900	-1.64481000	-3.13465700
B	-0.38133800	0.06593200	0.03938800
N	2.27435600	-1.65844200	1.06408600
H	1.31405200	-1.25728300	1.00270100
H	3.42777200	1.07181300	-4.01923400
H	5.27456200	0.25373100	-2.85709800
C	-0.22070800	0.20352500	2.79291700
C	-0.35782300	-0.34414900	4.06844100
C	-0.59212800	-1.70663400	4.23238000
C	-0.70764700	-2.50546500	3.09603800
C	-0.59012200	-1.93519200	1.82864200
C	-0.32201000	-0.56395200	1.61614200
H	-0.08485400	1.27754800	2.71478600
H	-0.29627800	0.30376500	4.93814000
H	-0.70728900	-2.13424300	5.22359700
H	-0.92616200	-3.56586600	3.19301800
H	-0.75199400	-2.57265100	0.96005000

N11_TS

B	0.04477700	0.06015000	0.41994700
N	-2.24743600	-2.06080200	0.46754100
C	-1.30594000	-1.95212200	1.59910400
H	-0.47452300	-2.62870200	1.36855600
C	-0.79865300	-0.50249100	1.68668100
H	-1.71989700	0.09302500	1.70549000
C	-0.20839200	-0.37403500	3.11595000
H	0.87860100	-0.27261700	3.12065800
H	-0.60996100	0.51794100	3.60477200
C	-0.64551500	-1.66341300	3.86213200
H	0.18922800	-2.37057800	3.88702800
H	-0.94501000	-1.47408700	4.89553300
C	-1.78613300	-2.25758100	3.02157200
H	-2.72043900	-1.71954700	3.22167100
H	-1.95646200	-3.32130600	3.21347700
C	-2.32951400	-3.40867000	-0.12048700
H	-2.83884600	-4.08436500	0.58634000
H	-1.31214600	-3.78389200	-0.25880400
C	-3.07073000	-3.37465800	-1.45467800
H	-3.12155400	-4.38975700	-1.86032000
H	-2.49004600	-2.76815500	-2.16183300
C	-4.46780700	-2.77936200	-1.28601100
H	-4.96821900	-2.69302900	-2.25488200
H	-5.07746800	-3.45099500	-0.66672700
C	-4.36819700	-1.41671600	-0.60422800
H	-3.84604200	-0.70841200	-1.25738400
H	-5.36153500	-1.00413400	-0.40321700
C	-3.60174800	-1.52913100	0.70942500
H	-3.52145400	-0.55286100	1.18815100
H	-4.13791600	-2.20401800	1.39627600
C	-0.35640700	1.63122600	0.24558500
F	1.75339900	2.50178700	0.91792700
C	0.48273900	2.70259800	0.54711100
F	0.93533900	5.00870900	0.79524600
C	0.07749400	4.03265200	0.50371300
F	-1.63164800	5.61198700	0.11727700
C	-1.23035100	4.34516600	0.16792400
F	-3.38529200	3.58684400	-0.42141100
C	-2.11748900	3.31582600	-0.10977700
F	-2.59109700	1.05161600	-0.29538700
C	-1.66510800	2.00686100	-0.04395600
C	1.63664800	-0.24852700	0.30189700
F	1.85717500	1.33308500	-1.44732300

C	2.41546400	0.41096700	-0.65278500
F	4.44734700	0.83572300	-1.78439500
C	3.76076800	0.15766500	-0.86573300
F	5.68598100	-1.08607300	-0.30381700
C	4.39551900	-0.82449500	-0.11664600
F	4.24416600	-2.50532900	1.52659700
C	3.66300200	-1.53687000	0.81756600
F	1.66735800	-2.04582300	1.86520200
C	2.31457600	-1.24468700	0.99490500
H	-1.40049900	-1.19532100	-0.42659900
C	0.74405700	-2.33338600	-2.77207800
C	0.30512900	-2.01629600	-1.49874100
C	-0.47809700	-0.86633800	-1.22940600
C	-0.81944400	-0.06916400	-2.33798400
C	-0.37568300	-0.37040400	-3.62271400
C	0.40742700	-1.49801300	-3.84132100
H	1.35303000	-3.21588700	-2.93963900
H	0.60210700	-2.65433900	-0.66795500
H	-1.44072100	0.80798500	-2.19961900
H	-0.64009900	0.27658000	-4.45264400
H	0.75778100	-1.73102500	-4.84222700

N11_P

B	-0.45512100	-0.34849700	-0.09905800
N	2.44262200	-1.40578300	-0.71182600
C	1.30672300	-1.77461100	-1.65124600
H	0.99198700	-2.75135100	-1.28124400
C	0.09385500	-0.83596000	-1.61306500
H	0.41123600	0.08444300	-2.12910400
C	-0.74793300	-1.57057000	-2.67512900
H	-1.16702900	-2.49237300	-2.26816400
H	-1.58491100	-0.96011300	-3.02200700
C	0.25703500	-1.91059100	-3.80440400
H	0.03217400	-2.87178600	-4.27204400
H	0.23027000	-1.15543100	-4.59350800
C	1.66127100	-1.91726400	-3.13414000
H	2.23309800	-1.05427800	-3.48402600
H	2.23947400	-2.81830600	-3.35976700
C	3.21033700	-2.62408300	-0.28513100
H	3.62982100	-3.06118800	-1.19765400
H	2.47455300	-3.31023200	0.14144500
C	4.29371200	-2.26748900	0.72248500
H	4.82114900	-3.18639100	0.99306500
H	3.81813000	-1.88692800	1.63482000
C	5.24932700	-1.22001100	0.15506700
H	6.01162000	-0.95950400	0.89335200
H	5.77069000	-1.63245800	-0.71874900
C	4.45681500	0.02276300	-0.24686600
H	4.04318700	0.49369900	0.64755600
H	5.10009800	0.76447800	-0.72929000
C	3.35165200	-0.33273400	-1.23591600
H	2.72092400	0.52384400	-1.48877700
H	3.79355700	-0.73330700	-2.15159100
C	0.47317700	1.03112800	0.15005600
F	-0.74037700	2.09522100	-1.57802000
C	0.25200800	2.13862100	-0.67870600
F	0.70382100	4.31963900	-1.47359200
C	0.98977200	3.31105600	-0.65213000
F	2.78505200	4.53813600	0.27667900
C	2.05005700	3.42968800	0.23994200
F	3.38779200	2.41022400	1.89405900
C	2.33418800	2.36382400	1.07114600
F	2.03196100	0.18482800	1.76056800
C	1.56134300	1.20638000	0.99472100
C	-2.07429500	0.01951700	-0.06527000
F	-1.89914300	2.23939500	0.80523900
C	-2.65169300	1.20901000	0.38009900
F	-4.48723600	2.61326000	0.87743100
C	-4.02207100	1.43581000	0.45511800
F	-6.22262900	0.62679800	0.17409400
C	-4.90682600	0.43086300	0.10463200
F	-5.21984600	-1.79138900	-0.61297900
C	-4.39362500	-0.79086400	-0.29973500
F	-2.62201100	-2.20877300	-0.69215900
C	-3.01775400	-0.96698800	-0.35672000
H	1.98176500	-1.05308000	0.14196700
C	-0.20715400	-3.72254800	2.01289400
C	-0.27719300	-2.82907100	0.94085000
C	-0.28554100	-1.43652300	1.11977500
C	-0.29749300	-0.99392700	2.45516300
C	-0.23728800	-1.86739400	3.53352800
C	-0.17178900	-3.24358100	3.31620000
H	-0.20677100	-4.79264400	1.82450800
H	-0.39493800	-3.24359900	-0.05672600
H	-0.36499600	0.07538900	2.65090200
H	-0.24651000	-1.47743300	4.54713900
H	-0.12147200	-3.93123900	4.15476500

N12_TS

N	2.60890100	-1.37364900	0.34345900
H	1.34827700	-1.16194800	-0.51118300
B	-0.12020700	-0.11422100	0.28900000
C	2.16150800	-0.61466200	1.54553600
H	2.47620800	0.42280600	1.35735300
C	0.62393100	-0.61551200	1.65191600
H	0.31362000	-1.64083900	1.89732200
C	3.91428600	-0.91231900	-0.16733400
H	4.70418900	-1.18970200	0.55103600
H	3.89280800	0.17468900	-0.23954000
C	4.21533000	-1.53976800	-1.52360700
H	3.44055200	-1.22447100	-2.23279400
H	5.17284900	-1.15926600	-1.89194500
C	4.23520500	-3.06391700	-1.41343800
H	5.07717200	-3.36933400	-0.77757700
H	4.38979800	-3.52350200	-2.39392800
C	2.92541400	-3.55065700	-0.79369200
H	2.09991700	-3.35967500	-1.48820000
H	2.95479300	-4.62960100	-0.61172500
C	2.65564400	-2.83740100	0.53058300
H	1.70889400	-3.16600800	0.96714400
H	3.45233900	-3.09214600	1.24611500
C	2.83090900	-1.07632600	2.84648600
H	2.52999800	-2.10910900	3.06445900
H	3.92245700	-1.07226800	2.73717400
C	2.40530400	-0.19281600	4.01973600
H	2.88287800	-0.54044000	4.94144100
H	2.75447800	0.83447200	3.84831800
C	0.88389000	-0.19183100	4.15906600
H	0.57763800	0.45939500	4.98440100
H	0.54038900	-1.20518500	4.40797000
C	0.22288100	0.25985800	2.85712200
H	0.51974600	1.29946700	2.65924700
H	-0.86475200	0.26472700	2.96618600
C	0.36171500	1.40101900	-0.10653300
C	-0.30932400	2.49322200	0.45032300
F	-1.35853700	2.29990000	1.26233100
C	0.04096500	3.81829200	0.23068700
F	-0.64991800	4.80486900	0.79791100
C	1.12365900	4.11513600	-0.58479600
F	1.47949100	5.37737500	-0.80346400
C	1.83159600	3.07287500	-1.15756700
F	2.88131400	3.32321000	-1.94055200
C	1.43928300	1.76356600	-0.90426800
F	2.19410800	0.82008200	-1.51122400
C	-1.74838800	-0.24450100	0.25788400
C	-2.49013300	0.36560700	-0.75495500
F	-1.87427400	1.11866900	-1.68041100
C	-3.86387000	0.23573700	-0.90026900
F	-4.50308100	0.84521900	-1.89837200
C	-4.57193900	-0.54585700	0.00020400
F	-5.88920500	-0.68803000	-0.11942200
C	-3.88439800	-1.18574900	1.01912500
F	-4.54412100	-1.95416100	1.88638300
C	-2.50698600	-1.02993600	1.12279200
F	-1.92947200	-1.72685000	2.11957300
C	0.14185800	-0.90462400	-2.42097300
C	0.24069500	-1.33865200	-1.08393800
C	-0.34171200	-2.59242800	-0.77372600
C	-0.99720300	-3.35344900	-1.72576100
C	-1.08839700	-2.88230200	-3.03819300
C	-0.51957000	-1.66025600	-3.38320500
H	0.57807000	0.04264000	-2.71289200
H	-0.32848600	-2.94093700	0.25637200
H	-1.44996200	-4.30120800	-1.45347200
H	-1.60892500	-3.46906300	-3.78908500
H	-0.59588400	-1.29348100	-4.40143100

N12_P

N	2.75183800	-1.03448100	0.56078200
H	2.18996300	-0.86106600	-0.28905800
B	-0.22994300	-0.18203200	0.08990500
C	1.98799400	-0.32669900	1.67582200
H	2.20285800	0.73616100	1.49810900
C	0.46941000	-0.53906900	1.56403000
H	0.27441400	-1.59106000	1.81383000
C	4.11609500	-0.46082600	0.31601000
H	4.73627500	-0.73440900	1.17439300
H	4.01044600	0.62269300	0.27778300
C	4.68808900	-1.01926600	-0.98038400
H	4.04677800	-0.69989000	-1.81173300
H	5.67397200	-0.57522900	-1.14341800
C	4.76447500	-2.54558000	-0.93086600
H	5.49333700	-2.85104500	-0.16859100
H	5.11627300	-2.93859700	-1.88808500
C	3.38819400	-3.12005500	-0.59586600
H	2.68490800	-2.90740900	-1.40988400

H	3.42591600	-4.20737300	-0.48379400
C	2.84165300	-2.52445600	0.69528700
H	1.84046800	-2.89329200	0.91931200
H	3.50330300	-2.72969100	1.54166100
C	2.54686400	-0.72765600	3.04251000
H	2.33697300	-1.78992100	3.21939000
H	3.63467400	-0.59233300	3.08539800
C	1.87241100	0.08729700	4.14909200
H	2.26304000	-0.22742800	5.12163200
H	2.13380500	1.14688400	4.02756700
C	0.35480600	-0.07076000	4.08205800
H	-0.12132800	0.54847700	4.84919500
H	0.08349400	-1.11290100	4.29794600
C	-0.15847000	0.30576000	2.69367900
H	0.06848000	1.36672200	2.51205700
H	-1.24539400	0.21497100	2.65090300
C	0.10213500	1.42143100	-0.20631900
C	-0.75504700	2.45230500	0.19252800
F	-1.91385300	2.17857400	0.80760100
C	-0.48846300	3.80484600	0.02127200
F	-1.36216100	4.72457500	0.42737800
C	0.70457700	4.20535500	-0.56324700
F	0.98191600	5.49673500	-0.72445300
C	1.60870200	3.23369200	-0.95271200
F	2.78218900	3.57576500	-1.49037000
C	1.28471300	1.89625800	-0.75906400
F	2.26863600	1.03060500	-1.13926800
C	-1.86672000	-0.44492800	0.10940500
C	-2.65194700	0.07566600	-0.91987800
F	-2.09237800	0.87960600	-1.83992900
C	-4.00503600	-0.17888800	-1.07863400
F	-4.69124800	0.36422200	-2.08533200
C	-4.64730200	-1.02226900	-0.18251000
F	-5.94642900	-1.28654400	-0.31480000
C	-3.91363900	-1.59002700	0.84399400
F	-4.50631900	-2.41741500	1.70828700
C	-2.55695400	-1.30111300	0.95959700
F	-1.92877400	-1.94218200	1.97184000
C	0.63262500	-0.88738100	-2.40118300
C	0.20706100	-1.23040000	-1.10913300
C	-0.02446600	-2.60352300	-0.88131000
C	0.17610000	-3.57065900	-1.85994300
C	0.62695400	-3.19570500	-3.12652700
C	0.85030300	-1.84945100	-3.39202300
H	0.77211000	0.15817100	-2.65774900
H	-0.41820600	-2.91500000	0.08687200
H	-0.03038600	-4.61480500	-1.64210400
H	0.78241700	-3.94295100	-3.89844300
H	1.17334100	-1.53698800	-4.38090700

N13_TS

N	2.33646000	-0.77581800	-0.68886700
H	0.86633600	-0.76347500	-1.14933200
B	-0.42921500	-0.14338400	0.14757400
C	2.12494500	-0.28608700	0.71168600
H	2.13974500	0.80586400	0.61801300
C	0.71729700	-0.68906400	1.16400200
H	0.54144800	-0.24792800	2.15273400
H	0.65929400	-1.76989300	1.32119900
C	2.71760400	-2.20041900	-0.77351100
H	2.01481500	-2.78490300	-0.17340800
H	3.71448800	-2.33732300	-0.32854600
C	2.72625600	-2.68019300	-2.22391700
H	1.71330500	-2.61650700	-2.63854500
H	3.01964000	-3.73452300	-2.24192100
C	3.67896100	-1.83240100	-3.06588400
H	4.70971500	-1.99118200	-2.72163100
H	3.63918300	-2.13629800	-4.11594500
C	3.31981400	-0.35526700	-2.91336100
H	4.03121700	0.28074200	-3.44891000
H	2.32579800	-0.16512200	-3.33531000
C	3.30911500	0.03862500	-1.44124000
H	4.31247200	-0.10724800	-1.00829800
H	3.03978200	1.08989600	-1.32250000
C	3.25977100	-0.65542400	1.65328600
C	4.35924700	0.20133500	1.76198600
H	4.36418600	1.13170100	1.19866400
C	5.43407900	-0.10833900	2.59007600
H	6.27452100	0.57445100	2.66315300
C	5.42256900	-1.28669900	3.33008300
H	6.25614900	-1.53154900	3.98032200
C	4.32960400	-2.14472400	3.24002700
H	4.30834800	-3.05975500	3.82337200
C	3.25702000	-1.83031300	2.41034100
H	2.40610900	-2.50336500	2.36282800
C	-1.97371700	-0.47992100	0.52821600
C	-3.02454200	0.13253300	-0.15340000
F	-2.76717500	1.03027400	-1.11810600

C	-4.36450800	-0.13883100	0.08655900
F	-5.31970500	0.48032600	-0.60693500
C	-4.70483500	-1.06926800	1.05703300
F	-5.98220100	-1.34833100	1.30371500
C	-3.69708800	-1.71340400	1.75858300
F	-4.00838500	-2.61772100	2.68727600
C	-2.36837700	-1.41224800	1.48281300
F	-1.45771100	-2.10322400	2.18955200
C	-0.22740700	1.46834300	-0.05233500
C	0.41759900	2.16067700	-1.06854500
F	0.94857300	1.50667700	-2.12689300
C	0.59269400	3.53984500	-1.08768400
F	1.22420600	4.12536300	-2.10535200
C	0.11466800	4.30107800	-0.03451700
F	0.27322200	5.62124900	-0.02855100
C	-0.51929500	3.66231100	1.02213700
F	-0.96785100	4.37472500	2.05296000
C	-0.66710300	2.28217900	0.99485300
F	-1.25490100	1.73070400	2.06716800
C	-1.30801600	-3.28076000	-2.00943400
C	-0.55072100	-2.48705600	-1.16533300
C	-0.31266300	-1.11676300	-1.43379300
C	-0.87100400	-0.59489000	-2.61861700
C	-1.63901200	-1.38368300	-3.46839900
C	-1.85745300	-2.72447300	-3.16676600
H	-1.48702200	-4.32316200	-1.76748100
H	-0.17787900	-2.91841500	-0.24015900
H	-0.71510000	0.44468300	-2.87382900
H	-2.07045300	-0.95111200	-4.36496300
H	-2.46090800	-3.33848100	-3.82847400

N13_P

N	-2.51646000	-0.58095600	0.51960200
H	-1.72689200	-0.36895900	1.15218500
B	0.55336100	-0.20205800	0.05864600
C	-1.98767400	-0.21037800	-0.87354300
H	-1.95592400	0.88427600	-0.83504800
C	-0.56409700	-0.73127100	-1.05110800
H	-0.25841000	-0.39977500	-2.05117700
H	-0.57347600	-1.82386200	-1.11196300
C	-2.82665000	-2.03328600	0.71890400
H	-1.96864700	-2.60229300	0.36028900
H	-3.69773500	-2.25973100	0.09757300
C	-3.09152800	-2.29805000	2.19599300
H	-2.17820100	-2.08031800	2.76431100
H	-3.29683400	-3.36496400	2.32147600
C	-4.24965700	-1.44345900	2.70995500
H	-5.18051700	-1.73851900	2.20846000
H	-4.39466400	-1.60489000	3.78115700
C	-3.96550600	0.03246000	2.42984200
H	-4.81077300	0.66307400	2.71890100
H	-3.09706400	0.36585000	3.01182100
C	-3.67723800	0.26079000	0.95212800
H	-4.52484000	-0.02487900	0.32165600
H	-3.41339100	1.29964000	0.75073200
C	-2.99312900	-0.60595900	-1.93663400
C	-3.93443500	0.32843400	-2.37820400
H	-3.92464500	1.33339500	-1.96179500
C	-4.86678200	-0.00285300	-3.35730300
H	-5.58382200	0.73863100	-3.69424600
C	-4.86747100	-1.27951500	-3.91038300
H	-5.58926700	-1.54061100	-4.67748000
C	-3.92764700	-2.21619600	-3.48693400
H	-3.91301900	-3.20868200	-3.92542800
C	-2.99413700	-1.88033000	-2.51196200
H	-2.25109900	-2.61142100	-2.20825000
C	2.08450600	-0.65184900	-0.35400100
C	3.15780500	-0.10665000	0.34978900
F	2.92830000	0.83307200	1.28347800
C	4.48146700	-0.46694000	0.15334200
F	5.46014400	0.10090800	0.86035200
C	4.78640000	-1.44065100	-0.78832500
F	6.05094200	-1.80893500	-0.99195300
C	3.75760500	-2.02700000	-1.50489900
F	4.02836000	-2.97258300	-2.40801700
C	2.44426100	-1.62828900	-1.27274100
F	1.51267400	-2.27877800	-2.00076700
C	0.44661300	1.45743500	0.07114400
C	-0.43539700	2.20851800	0.83956000
F	-1.30689300	1.59543600	1.69168500
C	-0.56676600	3.59116700	0.79736000
F	-1.45816400	4.21272900	1.57329000
C	0.21999900	4.31365100	-0.08296600
F	0.11436200	5.63789100	-0.15887200
C	1.10007100	3.62519200	-0.90455100
F	1.84301500	4.29786200	-1.78153800
C	1.18354800	2.24018100	-0.82329400
F	2.02092500	1.67225000	-1.70093200

C	0.31547600	-3.12549800	2.66933100
C	0.35830500	-2.36503300	1.50583100
C	0.37534900	-0.95508700	1.51605400
C	0.38582900	-0.35332300	2.78323700
C	0.33325400	-1.10291900	3.96179200
C	0.29010500	-2.49187100	3.91190800
H	0.31544500	-4.21029900	2.61016400
H	0.42465700	-2.88244800	0.54790800
H	0.46276500	0.72652000	2.86350300
H	0.34761600	-0.59442600	4.92161000
H	0.25930600	-3.07552000	4.82674900

N14_TS

B	0.42888900	-0.14344900	-0.14781800
N	-2.33661500	-0.77573000	0.68887700
H	-0.86675100	-0.76360000	1.14897600
C	-2.12528000	-0.28565700	-0.71157500
H	-2.13986900	0.80626600	-0.61754000
C	-0.71780000	-0.68871200	-1.16430400
H	-0.54213300	-0.24728900	-2.15293300
H	-0.65998000	-1.76951000	-1.32183500
C	-2.71777900	-2.20034200	0.77339800
H	-2.01518700	-2.78477100	0.17302000
H	-3.71479400	-2.33709100	0.32868800
C	-2.72616500	-2.68027100	2.22377000
H	-3.01922700	-3.73468800	2.24176100
H	-1.71321200	-2.61628000	2.63834400
C	-3.67900100	-1.83263400	3.06573700
H	-4.70962600	-1.99099700	2.72089000
H	-3.63982300	-2.13689400	4.11571100
C	-3.31938700	-0.35556300	2.91367800
H	-4.03030500	0.28056800	3.44972200
H	-2.32512000	-0.16595800	3.33528500
C	-3.30897700	0.03872200	1.44166000
H	-3.03942700	1.08995300	1.32311700
H	-4.31243300	-0.10686800	1.00887100
C	-3.26037100	-0.65447300	-1.65303900
C	-4.35923200	0.20303700	-1.76195000
H	-4.36356600	1.13348000	-1.19875000
C	-5.43425100	-0.10604400	-2.59002300
H	-6.27419400	0.57733900	-2.66329000
C	-5.42356600	-1.28458900	-3.32974100
H	-6.25728400	-1.52898000	-3.97997500
C	-4.33124800	-2.14341300	-3.23940200
H	-4.31066800	-3.05863500	-3.82247100
C	-3.25845400	-1.82957200	-2.40977200
H	-2.40802500	-2.50322300	-2.36206000
C	1.97334100	-0.48016500	-0.52855300
C	3.02425700	0.13202100	0.15315500
F	2.76702100	1.02965600	1.11798700
C	4.36417700	-0.13958000	-0.08680800
F	5.31948400	0.47939000	0.60669900
C	4.70433700	-1.07011700	-1.05724000
F	5.98165100	-1.34955500	-1.30377700
C	3.69648000	-1.71398900	-1.75888400
F	4.00762200	-2.61840300	-2.68753200
C	2.36782800	-1.41251000	-1.48319500
F	1.45701200	-2.10326200	-2.18997200
C	0.22742400	1.46832800	0.05217300
C	-0.41679000	2.16083500	1.06876300
F	-0.94809200	1.50691300	2.12702700
C	-0.59081800	3.54013300	1.08838900
F	-1.22143400	4.12583500	2.10651100
C	-0.11267600	4.30130700	0.03523900
F	-0.27026900	5.62159400	0.02970500
C	0.52035100	3.66236700	-1.02187200
F	0.96894200	4.37472600	-2.05270900
C	0.66722100	2.28213000	-0.99500300
F	1.25432800	1.73054700	-2.06765100
C	0.87051400	-0.59564600	2.61844800
C	0.31257700	-1.11707100	1.43325200
C	0.55065100	-2.48728300	1.16442700
C	1.30763200	-3.28131100	2.00850400
C	1.85666900	-2.72547400	3.16624300
C	1.63816200	-1.38478400	3.46825100
H	0.71444000	0.44379400	2.87407200
H	0.17795500	-2.91836100	0.23905500
H	1.48663600	-4.32364500	1.76625700
H	2.45985400	-3.33974100	3.82795500
H	2.06924700	-0.95254800	4.36515100

N14_P

B	0.55345200	-0.20204700	0.05853600
N	-2.51626200	-0.58131500	0.51950600
H	-1.72670900	-0.36937400	1.15212200
C	-1.98753500	-0.21037100	-0.87358400
H	-1.95582100	0.88427800	-0.83481000
C	-0.56394000	-0.73112300	-1.05138900

H	-0.25834200	-0.39930100	-2.05137300
H	-0.57319900	-1.82369300	-1.11257100
C	-2.82631600	-2.03371100	0.71860500
H	-1.96825400	-2.60261000	0.35996600
H	-3.69735500	-2.26016100	0.09721500
C	-3.09118900	-2.29863500	2.19566200
H	-3.29628200	-3.36559700	2.32107700
H	-2.17790600	-2.08076300	2.76401800
C	-4.24949800	-1.44430700	2.70966000
H	-5.18028300	-1.73949000	2.20809800
H	-4.39451800	-1.60589500	3.78083800
C	-3.96557700	0.03169200	2.42974300
H	-4.81100700	0.66212800	2.71871800
H	-3.09732200	0.36518000	3.01193500
C	-3.67715200	0.26024700	0.95211200
H	-3.41339700	1.29915900	0.75091000
H	-4.52466300	-0.02541800	0.32151600
C	-2.99306400	-0.60573200	-1.93669000
C	-3.93468000	0.32860800	-2.37773000
H	-3.92508000	1.33339600	-1.96091100
C	-4.86713500	-0.00253400	-3.35676500
H	-5.58444400	0.73889300	-3.69326100
C	-4.86761600	-1.27898500	-3.91034800
H	-5.58949500	-1.53997500	-4.67740200
C	-3.92745900	-2.21558600	-3.48746700
H	-3.91265100	-3.20788800	-3.92636700
C	-2.99384200	-1.87986200	-2.51253900
H	-2.25055600	-2.61087700	-2.20926600
C	2.08469400	-0.65158100	-0.35405100
C	3.15791900	-0.10641100	0.34986600
F	2.92831100	0.83310100	1.28374500
C	4.48163000	-0.46651000	0.15337400
F	5.46021600	0.10133200	0.86050900
C	4.78668400	-1.44001600	-0.78845800
F	6.05126800	-1.80810200	-0.99219400
C	3.75796200	-2.02633600	-1.50516800
F	4.02886400	-2.97169500	-2.40848500
C	2.44457700	-1.62782500	-1.27296200
F	1.51307100	-2.27824300	-2.00114800
C	0.44641600	1.45742900	0.07138800
C	-0.43560500	2.20828300	0.84001000
F	-1.30698100	1.59494200	1.69209200
C	-0.56716600	3.59091500	0.79799600
F	-1.45857100	4.21224900	1.57409500
C	0.21941800	4.31363200	-0.08229500
F	0.11356200	5.63786800	-0.15798200
C	1.09951000	3.62541100	-0.90407900
F	1.84230800	4.29828200	-1.78102500
C	1.18313800	2.24040400	-0.82304300
F	2.02048200	1.67266300	-1.70083400
C	0.38568500	-0.35400500	2.78314200
C	0.37542100	-0.95546300	1.51579400
C	0.35872800	-2.36540600	1.50523600
C	0.31593300	-3.12615900	2.66855000
C	0.29029100	-2.49285300	3.91126300
C	0.33314700	-1.10389200	3.96149700
H	0.46242100	0.72582300	2.86365600
H	0.42534800	-2.88255200	0.54718900
H	0.31619300	-4.21094400	2.60911900
H	0.25954500	-3.07671300	4.82597300
H	0.34731500	-0.59565900	4.92145500

N15_TS

N	-2.37029100	-0.74533000	1.04311700
B	0.36071900	-0.15747700	-0.04332100
C	-2.23996200	-0.34804300	-0.39558000
H	-2.24883300	0.74820700	-0.37715300
C	-0.86327900	-0.79890600	-0.89570000
H	-0.75794500	-0.48181800	-1.94100200
H	-0.80992600	-1.89074300	-0.92234700
C	-2.65128000	-2.18463100	1.25991000
C	-4.11930200	-2.59774400	1.36977800
H	-4.59315900	-2.19470800	2.26821800
H	-4.16536800	-3.68786500	1.43758300
C	-2.98377200	-0.03422000	3.33548500
H	-3.59174300	0.67836700	3.89775100
H	-1.93157100	0.15797000	3.56073000
C	-3.24464000	0.12884100	1.84402300
C	-3.42633200	-0.78871500	-1.23501600
C	-4.59258000	-0.01605400	-1.22352900
H	-4.60144000	0.92485900	-0.67813900
C	-5.73483200	-0.43206100	-1.89955700
H	-6.63051300	0.18049400	-1.87738700
C	-5.72357900	-1.62891600	-2.61173200
H	-6.61212100	-1.95712600	-3.14129800
C	-4.55959300	-2.39111200	-2.65731400
H	-4.53726800	-3.31422700	-3.22774800
C	-3.41843700	-1.97146700	-1.97805300

H	-2.51480900	-2.57100400	-2.03265000
C	1.86338600	-0.62398500	-0.45919500
C	2.98239800	0.02059700	0.06818200
F	2.82874400	1.05737600	0.90759500
C	4.29131600	-0.34953600	-0.20808100
F	5.31572100	0.30945400	0.33303000
C	4.52951500	-1.41868500	-1.05878200
F	5.77546400	-1.79346500	-1.33749800
C	3.45247500	-2.09671900	-1.60706600
F	3.66374200	-3.12924100	-2.42381700
C	2.15833700	-1.69296700	-1.30085000
F	1.18044700	-2.42364000	-1.86403000
C	0.23295100	1.47372300	-0.07655400
C	-0.38631500	2.31774300	0.83517200
F	-0.94172900	1.82190100	1.96464600
C	-0.50925100	3.69296700	0.67459700
F	-1.12032600	4.43073400	1.60176800
C	-0.00409900	4.29024400	-0.46834400
F	-0.11508400	5.60267700	-0.64993800
C	0.60393600	3.49450200	-1.42879300
F	1.07839900	4.04727900	-2.54285500
C	0.69888600	2.12511100	-1.22102900
F	1.25797800	1.41846700	-2.21380800
H	-4.70126000	-2.28762900	0.49919000
H	-3.23369100	-1.03804900	3.68943200
H	-2.11582400	-2.48867100	2.16817000
H	-2.18958100	-2.73124800	0.43360000
H	-3.03110300	1.15779400	1.55108300
H	-4.29975000	-0.06620700	1.60989500
H	-0.89435600	-0.60668700	1.41494000
C	1.82333400	-0.78548600	3.59921400
C	0.96248200	-0.15435200	2.70912300
C	0.31061500	-0.87022700	1.68325100
C	0.53502200	-2.26789400	1.63829700
C	1.38333600	-2.90662400	2.52657200
C	2.03796100	-2.15802000	3.50658600
H	2.33363600	-0.20708700	4.36221600
H	0.81428600	0.91447600	2.79673100
H	0.07063600	-2.84903000	0.84512900
H	1.55083600	-3.97607100	2.45338600
H	2.71692300	-2.64737800	4.19852000

N15_P

N	-2.58525100	-0.51137200	0.92042500
B	0.50861000	-0.15219300	0.13597800
C	-2.14465400	-0.26494000	-0.53779300
H	-2.11936300	0.82657200	-0.61110000
C	-0.73725400	-0.82494400	-0.73201800
H	-0.51766700	-0.70997400	-1.80042600
H	-0.75847600	-1.90721900	-0.57189700
C	-2.93044600	-1.95053500	1.23563100
C	-4.40005300	-2.32082400	1.09541600
H	-5.02040900	-1.84705800	1.85978500
H	-4.47781000	-3.40134900	1.23801000
C	-3.56883900	0.47631100	2.96346500
H	-4.23415100	1.26002800	3.33066300
H	-2.55464500	0.69697200	3.31513600
C	-3.60587900	0.45196600	1.44337200
C	-3.20774200	-0.78297400	-1.48532600
C	-4.33593000	-0.00245000	-1.75602900
H	-4.40624200	0.99979800	-1.33938100
C	-5.36326200	-0.48858900	-2.55907800
H	-6.23202400	0.12965200	-2.76073800
C	-5.26724600	-1.76264500	-3.11372400
H	-6.06565400	-2.14446500	-3.74154300
C	-4.13118500	-2.53301200	-2.88111800
H	-4.03794800	-3.51534000	-3.33261700
C	-3.10505000	-2.04431600	-2.07770800
H	-2.21375000	-2.64391800	-1.92159100
C	1.94283000	-0.86841900	-0.25583700
C	3.11725500	-0.33537600	0.27740200
F	3.05829000	0.79586100	1.00058600
C	4.37705900	-0.88349300	0.09952000
F	5.45763400	-0.31139700	0.63386200
C	4.50926100	-2.04883100	-0.64428600
F	5.70792900	-2.60139800	-0.82769400
C	3.37759600	-2.62609900	-1.19194100
F	3.48140600	-3.74954100	-1.90664800
C	2.13398600	-2.03204300	-0.98973400
F	1.09954700	-2.68234600	-1.56508700
C	0.53928100	1.46547800	-0.22146500
C	-0.32989100	2.38037900	0.36135900
F	-1.25230100	1.95749500	1.26981800
C	-0.37713600	3.73932900	0.08165500
F	-1.25807600	4.53221600	0.69725500
C	0.49586900	4.25788700	-0.86020100
F	0.47683200	5.55449800	-1.15974800
C	1.37649500	3.39671400	-1.49701400

F	2.20870600	3.87376900	-2.42161000
C	1.37669000	2.04281000	-1.17823000
F	2.24405500	1.29854600	-1.87629200
H	-4.80333900	-2.08424000	0.10899000
H	-3.89091600	-0.47173700	3.40192600
H	-2.57801300	-2.12686800	2.25433200
H	-2.31395200	-2.56085800	0.57526500
H	-3.34448000	1.43017800	1.04320300
H	-4.57931700	0.16104800	1.04658000
H	-1.71586400	-0.31858100	1.44802900
C	0.46435400	0.14946200	4.12506600
C	0.56566800	0.48625200	2.77624200
C	0.37754500	-0.45745700	1.75295500
C	0.13621300	-1.77863000	2.17416600
C	0.03037200	-2.13236100	3.51856100
C	0.18061700	-1.16037800	4.50442500
H	0.61692100	0.91221400	4.88314000
H	0.81284700	1.51095500	2.51395900
H	0.04647700	-2.56301300	1.42178200
H	-0.15476100	-3.16699700	3.79482200
H	0.10092200	-1.42420200	5.55441900

N16_TS

N	2.45496600	-0.69926000	-0.73161200
H	0.95764500	-0.81054500	-1.10463300
B	-0.38833700	-0.22561000	0.23470000
C	2.21616700	-0.17571500	0.65115600
H	2.08266600	0.89642900	0.46613600
C	0.84757200	-0.59960400	1.29203700
C	2.91945000	-2.09271000	-0.89396800
H	2.24878900	-2.76267000	-0.36021300
H	3.92627000	-2.20344300	-0.46482200
C	2.94258500	-2.49425600	-2.37009500
H	1.92283200	-2.44757100	-2.77259600
H	3.26970900	-3.53683900	-2.43687800
C	3.85745300	-1.57794800	-3.17632900
H	4.89510500	-1.71077100	-2.84197200
H	3.82472800	-1.83359600	-4.23947100
C	3.43352300	-0.13018500	-2.94736300
H	4.11946400	0.56640800	-3.43944000
H	2.43618700	0.03589100	-3.36973400
C	3.39863200	0.18361600	-1.45681800
H	4.40425900	0.05283000	-1.02422700
H	3.10142700	1.21901400	-1.29010200

C	3.44545800	-0.26439900	1.54957300
C	4.09502200	0.92639700	1.89307200
H	3.69615100	1.86913300	1.52565600
C	5.23166900	0.93173800	2.69588900
H	5.71050600	1.87283400	2.94691900
C	5.75006900	-0.26792200	3.17253000
H	6.63667200	-0.27209100	3.79811600
C	5.12007200	-1.46340400	2.84040400
H	5.51364300	-2.40565300	3.20850500
C	3.98081800	-1.46316100	2.03963500
H	3.51013300	-2.41015700	1.80486500
C	-1.95113700	-0.63876400	0.57609300
C	-2.94350300	-0.30774400	-0.35747500
F	-2.63408500	0.37373500	-1.47239500
C	-4.29130900	-0.60777600	-0.22255000
F	-5.15743500	-0.26766600	-1.17637200
C	-4.73791000	-1.24163900	0.92546400
F	-6.02492700	-1.53627400	1.08188700
C	-3.81643500	-1.53972400	1.91259600
F	-4.21916500	-2.11015900	3.04776700
C	-2.47329700	-1.22205500	1.73141100
F	-1.71000400	-1.49144900	2.80441900
C	-0.41044700	1.39038600	-0.10806600
C	0.23134000	2.06535800	-1.13957400
F	1.00481800	1.39768200	-2.02219900
C	0.15060800	3.43428300	-1.36866500
F	0.79867100	3.98326800	-2.39609600
C	-0.60529900	4.22145000	-0.51814300
F	-0.69805400	5.53337800	-0.70868600
C	-1.25763200	3.61328500	0.54419000
F	-1.97661300	4.34911800	1.38893800
C	-1.14973600	2.24064000	0.72449200
F	-1.79992000	1.75841700	1.79285000
C	0.70301400	0.32887600	2.52484600
H	1.48919600	0.10869500	3.25460900
H	0.79790700	1.38561900	2.25469000
H	-0.25996800	0.18896100	3.01476300
C	0.89003000	-2.04633300	1.85248300
H	-0.10238500	-2.46406400	1.99418500
H	1.44627800	-2.74749100	1.23288300
H	1.37777400	-2.05306000	2.83282800
C	-1.43070900	-3.00970600	-3.21354600
C	-1.24188200	-1.67125800	-3.54178700
C	-0.61858300	-0.81642300	-2.63997200

C	-0.16399400	-1.26128600	-1.38022400
C	-0.35699800	-2.63552100	-1.09471400
C	-0.98683800	-3.49308700	-1.98167900
H	-1.92157000	-3.67828000	-3.91444800
H	-1.58486100	-1.29092000	-4.49819400
H	-0.48145900	0.21812700	-2.92322500
H	-0.04143900	-3.03137500	-0.13735100
H	-1.13495700	-4.53547500	-1.71950900

N16_P

N	-2.58096200	-0.59318300	0.59602500
H	-1.75734800	-0.47692100	1.20805200
B	0.50588600	-0.33279900	-0.05942500
C	-2.08617100	-0.12128400	-0.78571800
H	-1.92925300	0.94287800	-0.58490400
C	-0.68194800	-0.63968600	-1.24514400
C	-3.02321200	-2.01651700	0.77539500
H	-2.24315900	-2.66275200	0.38635700
H	-3.93972100	-2.13904500	0.19315200
C	-3.24959300	-2.30534200	2.25503300
H	-2.29402100	-2.19543900	2.78212700
H	-3.54466000	-3.35447800	2.34989800
C	-4.29880600	-1.37426400	2.85739800
H	-5.27747300	-1.57160000	2.40101200
H	-4.39978800	-1.55595400	3.93046700
C	-3.89892100	0.07663800	2.59556500
H	-4.67046100	0.77205400	2.93801000
H	-2.97853300	0.31941200	3.14095300
C	-3.66668800	0.31400100	1.11028100
H	-4.55956400	0.09227500	0.51807200
H	-3.35788900	1.33940000	0.91112000
C	-3.22486500	-0.19253800	-1.78895200
C	-3.75151300	1.01236300	-2.26737500
H	-3.33602700	1.95082400	-1.90789700
C	-4.78273200	1.03166300	-3.20084400
H	-5.16730600	1.98057600	-3.56007800
C	-5.31547600	-0.16446000	-3.67102100
H	-6.12062500	-0.15620400	-4.39840900
C	-4.80398600	-1.37248100	-3.20620700
H	-5.20634700	-2.31086100	-3.57387500
C	-3.76635200	-1.38864900	-2.27820500
H	-3.37183000	-2.34304300	-1.95009900
C	2.08611500	-0.75011400	-0.45246100
C	3.04604500	-0.44329700	0.52238300
F	2.68877100	0.21179300	1.64055500
C	4.39944000	-0.73081400	0.43154700
F	5.23249000	-0.39623800	1.41822500
C	4.88809000	-1.35669200	-0.70404500
F	6.18286400	-1.64532600	-0.82059900
C	3.99924400	-1.65951300	-1.71776300
F	4.43939100	-2.23974500	-2.83637400
C	2.64924900	-1.34404400	-1.58008200
F	1.92400700	-1.64123800	-2.67899300
C	0.54045800	1.33513200	0.17830600
C	-0.26829800	2.06613100	1.04238100
F	-1.25030800	1.45133400	1.76219500
C	-0.21486600	3.44000800	1.24671600
F	-1.04630100	4.02978000	2.10878600
C	0.70040500	4.19033400	0.53144400
F	0.78036900	5.50772200	0.69554300
C	1.51369800	3.53781600	-0.38177600
F	2.37930700	4.24059200	-1.11000200
C	1.41217600	2.16130600	-0.54547100
F	2.20752000	1.66576500	-1.50346400
C	-0.38529900	0.25323300	-2.47739500
H	-1.07425300	0.03391000	-3.30121200
H	-0.48424200	1.31830200	-2.24131200
H	0.62904300	0.08672600	-2.83601100
C	-0.75083000	-2.09265900	-1.77096600
H	0.24444200	-2.50056700	-1.92028100
H	-1.27538500	-2.78843100	-1.11668100
H	-1.25366600	-2.12561100	-2.74396100
C	0.11373100	-2.90122900	3.63884900
C	0.18332200	-1.51913700	3.77493800
C	0.27263600	-0.70158200	2.64667800
C	0.28322400	-1.21319900	1.33597200
C	0.24746600	-2.61989800	1.24447000
C	0.15611300	-3.45058400	2.35894700
H	0.05401400	-3.54148800	4.51355500
H	0.19480000	-1.06924300	4.76364000
H	0.39125100	0.36379900	2.80649100
H	0.34764300	-3.08713400	0.26917400
H	0.14353700	-4.52891900	2.22747500

N17_TS

B	0.01774600	-0.18264200	0.16623100
N	-2.56292900	-1.66961700	-0.35048900
H	-1.27089200	-1.18188600	-0.99566600

C	-2.38505200	-0.97191700	0.95155000
H	-2.72863500	0.05736900	0.77226900
C	-0.88637300	-0.91565000	1.30676500
H	-0.55081500	-1.95231100	1.45008600
C	-3.83821100	-1.35692000	-1.05589800
H	-4.48039400	-0.77121400	-0.38947600
H	-3.63625700	-0.75057800	-1.93937900
C	-4.48593900	-2.70254600	-1.41731900
H	-4.20687800	-2.99254700	-2.43503100
H	-5.57583500	-2.65792400	-1.37046000
C	-3.87119200	-3.68091400	-0.41503000
H	-3.90661100	-4.72039900	-0.74838900
H	-4.37555800	-3.61149200	0.55360900
C	-2.44586400	-3.14656200	-0.31034300
H	-1.86455400	-3.46601600	-1.18572000
H	-1.90495300	-3.46876700	0.58194700
C	-3.24313500	-1.56095400	2.07946400
H	-2.97641200	-2.61546200	2.23055300
H	-4.30336100	-1.53448500	1.79711000
C	-3.02268600	-0.81860600	3.39814600
H	-3.35775100	0.22220500	3.29296300
H	-3.63488900	-1.27028800	4.18535900
C	-1.54269000	-0.83438600	3.77561900
H	-1.21769000	-1.87139600	3.93722700
H	-1.38332900	-0.30035300	4.71814900
C	-0.70596800	-0.20537000	2.66304100
H	0.35325700	-0.20034400	2.93542300
H	-1.00645700	0.84685200	2.56110700
C	-0.47026300	1.36610400	-0.03790100
C	0.12256000	2.38765500	0.70802600
F	1.11259700	2.11163300	1.56859800
C	-0.25472400	3.72156800	0.63573500
F	0.35980200	4.64044200	1.37813300
C	-1.28679900	4.09705000	-0.21210000
F	-1.66133400	5.36983900	-0.29959600
C	-1.92543600	3.12280700	-0.96040500
F	-2.92933000	3.45188300	-1.77387700
C	-1.51348400	1.80054200	-0.84428100
F	-2.20615300	0.91401000	-1.59160100
C	1.63860600	-0.28674100	0.32945600
C	2.30541100	-1.19825500	1.14425700
F	1.62826400	-2.04686500	1.94041600
C	3.68862700	-1.33135900	1.18680400
F	4.25668500	-2.22933800	1.99256300
C	4.47960800	-0.53102900	0.37797200
F	5.80485500	-0.64654100	0.40135600
C	3.86801100	0.38814100	-0.46076300
F	4.60947400	1.16044800	-1.25455800
C	2.48424400	0.48797500	-0.46717200
F	1.97046700	1.39101600	-1.31767000
C	1.02365600	-0.92496000	-3.59858500
C	0.13810700	-0.44255200	-2.64125800
C	-0.11585700	-1.15597100	-1.45418300
C	0.51545900	-2.41556600	-1.30979500
C	1.39090200	-2.90872300	-2.26144700
C	1.65404800	-2.15171200	-3.40569500
H	1.22973600	-0.34400500	-4.49140200
H	-0.34194100	0.51553900	-2.80339400
H	0.35690400	-2.97879800	-0.39188800
H	1.88068200	-3.86579100	-2.11546600
H	2.35306600	-2.52303100	-4.14910900

N17_P

B	-0.14885800	-0.19713500	0.01310100
N	2.80382300	-1.30980400	0.04119800
H	2.15118000	-1.02108100	0.78576700
C	2.24927800	-0.64267700	-1.21847100
H	2.51318800	0.41473400	-1.08555500
C	0.72065500	-0.77688400	-1.29038300
H	0.50724600	-1.84657100	-1.42515700
C	4.20179700	-0.85361400	0.40763400
H	4.61330600	-0.33733400	-0.46075600
H	4.12867200	-0.14505200	1.22975300
C	4.98137800	-2.12734700	0.73035600
H	4.87383500	-2.38030500	1.78937300
H	6.04480400	-2.01062700	0.51558100
C	4.29537800	-3.18768600	-0.13415500
H	4.49085500	-4.20763600	0.20079100
H	4.60994400	-3.10226300	-1.17844700
C	2.82636900	-2.82228500	0.02373800
H	2.43878400	-3.14982000	0.99025200
H	2.15723500	-3.18715200	-0.75419600
C	2.96560300	-1.18240700	-2.45941600
H	2.75731200	-2.25483800	-2.56635300
H	4.05354800	-1.06935500	-2.37425600
C	2.46611700	-0.47211300	-3.71994400
H	2.73447400	0.59131600	-3.66698500
H	2.97641900	-0.88610100	-4.59502300

C	0.95030600	-0.60531500	-3.84226300
H	0.68228200	-1.66192200	-3.97758700
H	0.59630900	-0.06998400	-4.72919800
C	0.27291600	-0.06611400	-2.58490700
H	-0.81320500	-0.13752600	-2.67467200
H	0.50389100	1.00550600	-2.49627700
C	0.16691200	1.43032900	0.10294900
C	-0.65166500	2.41463900	-0.45618100
F	-1.77817700	2.08330500	-1.10170700
C	-0.37667300	3.77612200	-0.41474100
F	-1.21192300	4.65340100	-0.96951400
C	0.78505900	4.22799400	0.19430200
F	1.06616500	5.52807900	0.24055200
C	1.65460000	3.29800600	0.73733400
F	2.79732000	3.69101300	1.30548900
C	1.32600500	1.95015300	0.66498800
F	2.26906000	1.11372400	1.18260500
C	-1.77080100	-0.48782200	-0.16443200
C	-2.36447200	-1.45496400	-0.96690900
F	-1.63507700	-2.20698500	-1.82252500
C	-3.72644300	-1.74554800	-0.96214300
F	-4.21858500	-2.68910500	-1.76918700
C	-4.56806200	-1.06010500	-0.10508800
F	-5.87402000	-1.32320300	-0.07970000
C	-4.02820100	-0.09516500	0.73495800
F	-4.82133900	0.56844600	1.57759900
C	-2.66601100	0.15349200	0.69357700
F	-2.21081800	1.08279400	1.55080800
C	0.54369200	-1.18073200	3.82929700
C	0.39774300	-0.41969000	2.66869600
C	0.14368700	-1.00716000	1.41944700
C	0.00085100	-2.40928400	1.41395500
C	0.13980500	-3.18098800	2.56407200
C	0.42551900	-2.56578000	3.78197300
H	0.73622800	-0.68511300	4.77638500
H	0.46230400	0.66204600	2.74650300
H	-0.25531600	-2.90534700	0.47693900
H	0.01093200	-4.25887600	2.51434700
H	0.53179600	-3.15796300	4.68564600

N18_TS

C	-3.02775600	-2.27332100	-0.55851800
C	-2.30831900	-1.02304700	0.09408100
H	-2.04726000	-1.38221600	1.09052200
C	-0.99985400	-0.83345100	-0.70458100
H	-1.23978300	-0.20828800	-1.55710900
C	-0.88610500	-2.25300900	-1.31348200
H	-0.07842900	-2.36888000	-2.03682300
C	-0.87846200	-3.31596300	-0.18359800
H	-0.24673500	-2.98768900	0.64946800
H	-0.47428700	-4.27298700	-0.52281300
C	-2.37905700	-3.43697400	0.22433300
H	-2.54075500	-3.33939800	1.30401500
H	-2.81006900	-4.39637500	-0.07648900
C	-2.30679900	-2.43572700	-1.92993900
C	-4.54403500	-2.27202500	-0.56081900
H	-4.94927000	-1.40156100	-1.08878900
H	-4.94447400	-2.26852600	0.45823400
H	-4.92706500	-3.17072400	-1.05647500
C	-2.73320500	-1.40073100	-2.97830000
H	-2.98929800	-0.42649600	-2.56276800
H	-3.62113300	-1.76033800	-3.50922700
H	-1.94127300	-1.25102200	-3.72044100
C	-2.47669100	-3.80874200	-2.59696600
H	-3.53623200	-4.04985000	-2.73887700
H	-2.01320800	-4.62969600	-2.04873300
H	-2.01656800	-3.78180200	-3.59020800
N	-2.90464000	0.27590400	0.43066700
H	-1.46974500	0.60784100	1.05403200
C	-3.33147500	1.20903100	-0.61719100
H	-4.29685000	0.88793800	-1.04376800
H	-2.59071200	1.21500800	-1.41457200
C	-3.46405100	2.61906300	-0.04334400
H	-3.78961600	3.29766300	-0.83761600
H	-2.47076300	2.95483000	0.28384000
C	-4.43365600	2.64838900	1.13632900
H	-5.44262800	2.40142600	0.77880800
H	-4.48514100	3.65048500	1.57224000
C	-4.00531700	1.62261800	2.18431100
H	-3.04436900	1.91722200	2.62529800
H	-4.73416200	1.56501700	2.99876000
C	-3.85495200	0.24193800	1.55083800
H	-3.47841600	-0.47657000	2.28750100
H	-4.83554000	-0.11879200	1.20370200
B	0.19313400	-0.02589800	0.09976300
C	0.71567200	1.38262700	-0.58685300
F	-0.72467600	1.50350800	-2.51056800
C	0.29531800	1.98516600	-1.77279700

F	0.40718000	3.64137900	-3.45359900
C	0.87792000	3.12691900	-2.31800800
F	2.52151900	4.81152200	-2.19419600
C	1.95361800	3.72275300	-1.68547400
F	3.45850000	3.72225800	0.12599900
C	2.42502800	3.16659000	-0.50476400
F	2.31439500	1.57186400	1.15700800
C	1.81187600	2.03043100	0.00134700
C	1.54067200	-0.94342500	0.28615000
F	1.59415000	-0.93547500	-2.08494400
C	2.11619200	-1.35808100	-0.91837800
F	3.71070600	-2.53434800	-2.20880700
C	3.23358700	-2.17349700	-1.01778900
F	4.94346000	-3.37361000	0.08145800
C	3.86850200	-2.59173500	0.14219800
F	3.99587200	-2.51833500	2.48967600
C	3.37689400	-2.16281900	1.36325400
F	1.91843000	-0.92605500	2.64213600
C	2.25482600	-1.33998400	1.41303200
C	-0.06556100	2.18901000	3.58037200
C	-0.22198500	1.87958500	2.23977200
C	-0.49794200	0.56498200	1.79057900
C	-0.61983000	-0.42365800	2.79937500
C	-0.47671300	-0.12539600	4.14681300
C	-0.19811000	1.18179100	4.53749000
H	0.15044500	3.20739700	3.88536700
H	-0.15667100	2.67792700	1.50476500
H	-0.78985200	-1.46024200	2.51915200
H	-0.56129800	-0.9073700	4.89030000
H	-0.07926900	1.41739000	5.59090900

N18_P

C	2.82834100	-2.02601600	1.12993600
C	2.25436200	-0.99412900	0.08922800
H	2.02953600	-1.53417500	-0.83476300
C	0.94424300	-0.44425900	0.73486700
H	1.23004000	0.49147900	1.22109000
C	0.76083900	-1.47807100	1.87907900
H	-0.00456100	-1.18683700	2.59259600
C	0.54909000	-2.87832200	1.25464400
H	-0.15016100	-2.84936300	0.41945100
H	0.15152000	-3.58629800	1.98616800
C	1.97442200	-3.27698500	0.78498300
H	2.02517800	-3.50959100	-0.28349300
H	2.36083700	-4.15366900	1.31080900
C	2.17450100	-1.61437000	2.49256800
C	4.31279000	-2.35644900	1.17280600
H	4.95362900	-1.49113600	1.37422400
H	4.65370300	-2.83500500	0.24910800
H	4.48455800	-3.07396800	1.98145000
C	2.71217100	-0.35550800	3.18570900
H	2.44161700	0.58169900	2.70671100
H	3.80410400	-0.39791900	3.28732200
H	2.30230600	-0.30471000	4.19871300
C	2.26995800	-2.71354700	3.56760700
H	3.28497600	-2.76607500	3.97783700
H	1.99582100	-3.71220700	3.23000600
H	1.60216300	-2.45575800	4.39541500
N	3.17154600	0.11083000	-0.47335000
H	2.51909700	0.63788400	-1.07912900
C	3.73529100	1.11402600	0.48385000
H	4.38081600	0.57458800	1.18097800
H	2.89648100	1.53740900	1.03273100
C	4.49118000	2.21379100	-0.25228600
H	4.88820300	2.90448000	0.49684300
H	3.78037400	2.77685800	-0.87039700
C	5.59804200	1.64329200	-1.13327200
H	6.34396800	1.13361900	-0.50912600
H	6.11555500	2.44563700	-1.66512300
C	4.98397200	0.65515500	-2.12109700
H	4.29786400	1.17936400	-2.79506800
H	5.75028300	0.17784400	-2.73890200
C	4.22733600	-0.44399100	-1.38909600
H	3.70971400	-1.10852200	-2.08458400
H	4.90839400	-1.03045600	-0.77431500
B	-0.32510300	0.08340200	-0.25616600
C	-1.19282800	1.27775600	0.50954900
F	-0.27400800	1.11923400	2.71316900
C	-1.13691200	1.68024200	1.83929600
F	-1.82047900	3.01417900	3.67159900
C	-1.94044100	2.67808100	2.38499700
F	-3.63945000	4.28731400	2.08807400
C	-2.86566200	3.32638900	1.58590500
F	-3.85640400	357878000	-0.53541300
C	-2.97237700	2.9645100	0.25036100
F	-2.30604100	1.66137100	-1.54460300
C	-2.15002100	1.6164000	-0.24602200
C	-1.52873200	-100470700	-0.65326700

F	-1.96945000	-1.33176600	1.65799800
C	-2.28087200	-1.5357900	0.37419900
F	-4.00403600	-2.95077100	1.24373900
C	-3.36875000	-.42449400	0.19459000
F	-4.84690900	-3.50397400	-1.29771300
C	-3.80603400	-2.69981800	-1.09172800
F	-3.56280900	-2.33054900	-3.40138700
C	-3.14412500	-2.11040000	-2.15314800
F	-1.56051500	-0.70601800	-3.02783100
C	-2.05748900	-1.27013100	-1.91773900
C	1.50419700	2.69631500	-2.61593400
C	0.75878400	2.12111500	-1.58263400
C	0.42590800	0.75453200	-1.55619600
C	0.93551900	-0.01300800	-2.62461200
C	1.68106400	0.54333700	-3.66160900
C	1.96291300	1.91053500	-3.66836800
H	1.71148000	3.76300900	-2.60218000
H	0.40791500	2.76360800	-0.77785900
H	0.71157800	-1.07652700	-2.66353500
H	2.02781200	-0.08694600	-4.47628300
H	2.52671700	2.35341700	-4.48409400

N19_TS

B	0.45578800	-0.32263400	0.30062700
C	-0.19870100	-0.72639800	1.74741300
C	0.51071100	-1.59846400	2.58849200
H	1.45056100	-2.01470800	2.23825900
C	0.08452500	-1.95583300	3.86470200
H	0.68626300	-2.62942700	4.46692800
C	-1.09175900	-1.41907700	4.36693300
H	-1.42819100	-1.65048300	5.37248000
C	-1.84833000	-0.59415800	3.54357000
H	-2.79626000	-0.20423900	3.90970300
C	-1.45220500	-0.28438600	2.24034200
C	-2.42771200	0.55792100	1.43742900
H	-1.92504800	1.47498900	1.13921200
H	-3.24829900	0.85857300	2.10004600
N	-2.96343500	-0.05899600	0.19380600
C	-3.91369500	-1.22893500	0.41862500
C	-4.24546200	-1.83115900	-0.95820200
H	-3.31864800	-2.23640100	-1.38998000
H	-4.92077100	-2.67850600	-0.79708300
C	-4.86160600	-0.83370000	-1.92579500
H	-5.84558800	-0.50736100	-1.57040400
H	-5.02871500	-1.30541300	-2.89908800
C	-3.92284900	0.35031500	-2.08041400
H	-4.37471200	1.12883700	-2.70548600
H	-3.01828800	0.01151500	-2.59831900
C	-3.50899000	1.00763400	-0.75120900
C	-2.41692900	2.02984200	-1.09969000
H	-1.46587500	1.55152200	-1.31979100
H	-2.25205400	2.77077500	-0.31336200
H	-2.73745100	2.57721100	-1.99064900
C	-4.68739600	1.82739400	-0.18132600
H	-4.53077200	2.09995600	0.86564300
H	-5.65114100	1.32917600	-0.26915300
H	-4.75721000	2.75941900	-0.74965200
C	-5.21064400	-0.86610700	1.17160800
H	-5.00895400	-0.24211100	2.04696600
H	-5.65592200	-1.79551400	1.53850700
H	-5.96277500	-0.37066900	0.56201800
C	-3.26919200	-2.35193400	1.25040100
H	-2.23097300	-2.53117100	0.99218100
H	-3.83353000	-3.26912100	1.05558100
H	-3.31596700	-2.15491100	2.32028900
C	2.02106900	-0.83271000	0.15888500
C	2.62475400	-1.58195700	-0.84553800
F	1.92392000	-2.07022800	-1.87958800
C	3.98821400	-1.86453800	-0.88397700
F	4.49289500	-2.60030800	-1.87558600
C	4.82382700	-1.36871800	0.10076600
F	6.12867500	-1.62908200	0.07581200
C	4.28123500	-0.58457600	1.10946800
F	5.07016500	-0.08286200	2.05953400
C	2.91889100	-0.32712200	1.10632600
F	2.46612200	0.46634800	2.08870600
C	0.60626300	1.28097600	-0.03216300
C	0.41822800	2.32200900	0.87797600
F	0.00523200	2.09083500	2.12999100
C	0.61926800	3.66433300	0.57006000
F	0.38632000	4.60759900	1.48202200
C	1.07807400	4.02130900	-0.68714400
F	1.27078900	5.29880200	-1.00044200
C	1.35269000	3.02210100	-1.60889200
F	1.83771800	3.33846700	-2.80911300
C	1.14382900	1.69661100	-1.25473300
F	1.50022800	0.79189900	-2.18366800
H	-1.56242900	-0.64688600	-0.39888500

C	-0.56926200	-1.19344900	-0.92818300
C	-0.51690400	-2.59067100	-0.65513600
C	-0.63597500	-0.84347300	-2.30316600
C	-0.56987700	-3.54954700	-1.65047800
H	-0.39773200	-2.90898700	0.37731700
C	-0.69474900	-1.79234600	-3.31195500
H	-0.68336400	0.20389900	-2.58225400
C	-0.67192100	-3.14712900	-2.98308200
H	-0.52139800	-4.60409500	-1.40081700
H	-0.75966400	-1.48367000	-4.34990100
H	-0.72307600	-3.89364400	-3.77056800

N19_P

B	0.64300800	-0.48442200	0.03078300
C	-0.01688800	-1.18943600	1.38343100
C	0.69195500	-2.25999200	1.95930200
H	1.64361300	-2.54417500	1.52038800
C	0.26419100	-2.96804000	3.07620700
H	0.87308000	-3.77839600	3.46576600
C	-0.91811600	-2.60822300	3.71092500
H	-1.25049300	-3.11298700	4.61203800
C	-1.66515800	-1.57764000	3.16141300
H	-2.59278500	-1.28246100	3.64824100
C	-1.26409600	-0.91079000	1.99496400
C	-2.21538600	0.16832700	1.53001200
H	-1.66888100	1.07935000	1.31947200
H	-2.93178200	0.38273600	2.32209400
N	-3.04580300	-0.07688800	0.26991100
C	-3.96386900	-1.33688700	0.28030800
C	-4.71046500	-1.39955500	-1.06427100
H	-3.98212200	-1.66969500	-1.84014700
H	-5.41383600	-2.23493500	-0.98666500
C	-5.42497600	-0.12461500	-1.47647100
H	-6.24528300	0.11290700	-0.78974200
H	-5.87918300	-0.26161000	-2.46180400
C	-4.41221700	1.00683900	-1.52915900
H	-4.88312000	1.94867200	-1.82819300
H	-3.64831500	0.77651400	-2.28468700
C	-3.70841700	1.26081000	-0.19030700
C	-2.61776100	2.31784100	-0.42097300
H	-1.74028200	1.91361300	-0.92840000
H	-2.29122700	2.79910400	0.50311100
H	-3.04507300	3.09613400	-1.05840600
C	-4.68786000	1.81936600	0.84547400
H	-4.24480500	1.90468200	1.83926300
H	-5.61964800	1.26329000	0.92004900
H	-4.94102200	2.83326200	0.52313600
C	-4.93449100	-1.32862300	1.46049700
H	-4.43026400	-1.13623100	2.40913600
H	-5.36562500	-2.33098400	1.52707900
H	-5.75946100	-0.62673500	1.34662100
C	-3.10052300	-2.60398500	0.33834500
H	-2.25886800	-2.54680900	-0.35420700
H	-3.73651100	-3.43365700	0.01499400
H	-2.72462900	-2.83003900	1.33356000
C	2.27349000	-0.77070200	-0.03931700
C	2.99069100	-1.21520300	-1.14738900
F	2.38256300	-1.52644200	-2.29917400
C	4.37656300	-1.36277200	-1.15363500
F	5.00334500	-1.81263700	-2.24281400
C	5.10992300	-1.03525600	-0.02624300
F	6.43514600	-1.17178400	-0.01736400
C	4.44706500	-0.55360800	1.09427300
F	5.14126400	-0.21562700	2.18314500
C	3.06739800	-0.42174500	1.05591400
F	2.48815400	0.08422800	2.15651000
C	0.63356100	1.17209700	-0.00303700
C	0.49646100	2.02143500	1.09372200
F	0.20815400	1.54053200	2.31514700
C	0.59213700	3.40852000	1.02019200
F	0.40170100	4.16008100	2.10622300
C	0.88984800	4.01169600	-0.19073000
F	0.98625000	5.33629000	-0.28150500
C	1.10342900	3.21183400	-1.30446300
F	1.43051700	3.77156000	-2.46935400
C	1.00318500	1.83218600	-1.17929400
F	1.30586000	1.12609300	-2.27837000
H	-2.35408200	-0.29360900	-0.47063300
C	-0.13339500	-1.19359200	-1.23374800
C	-0.04119700	-2.59421800	-1.36007100
C	-0.94076200	-0.55256100	-2.18266800
C	-0.70831700	-3.30353100	-2.34940600
H	0.58223800	-3.14070300	-0.65500500
C	-1.63693000	-1.25391900	-3.17627400
H	-0.99047700	0.53410100	-2.20158500
C	-1.53103400	-2.63553800	-3.25981400
H	-0.59402100	-4.38188200	-2.41252300
H	-2.23828000	-0.70869700	-3.90043600

H	-2.05808000	-3.18421200	-4.03407800

N20_TS

B	0.81667900	0.07296100	-0.09012100
C	-0.14525300	0.19162200	-1.39867900
C	0.30869500	-0.28459300	-2.63871200
H	1.22992500	-0.85659100	-2.67809400
C	-0.35795900	-0.02129400	-3.83173100
H	0.04202100	-0.40024900	-4.76739000
C	-1.53614700	0.71666800	-3.82091300
H	-2.07267100	0.92140500	-4.74177900
C	-2.01929400	1.19144000	-2.60651900
H	-2.94479300	1.76312800	-2.57884900
C	-1.33058600	0.96031800	-1.41353200
C	-1.90762200	1.56505900	-0.15184700
H	-2.63238800	2.33985700	-0.41773000
H	-1.13333200	2.02978100	0.45461500
N	-2.56238500	0.50971900	0.69078100
C	-3.11958300	1.01243000	2.02888700
C	-4.00164000	2.24690300	1.79370600
H	-4.58273400	2.40532300	2.70776700
H	-3.35242200	3.12288200	1.69056300
C	-4.92853200	2.12578600	0.57756800
C	-4.87167200	0.77962500	-0.10533300
C	-6.02013000	0.34132000	-0.75552600
C	-6.04558400	-0.88713100	-1.39849900
H	-6.94516400	-1.23294100	-1.89703500
C	-4.90108000	-1.66328200	-1.39438500
H	-4.90889700	-2.62096500	-1.90414500
C	-3.71865200	-1.25763800	-0.75998200
C	-3.71560100	-0.03010700	-0.08202800
C	-2.53259000	-2.19148300	-0.86948600
H	-1.64484300	-1.63109700	-0.58085100
C	-2.30057100	-2.64473500	-2.31620200
H	-1.34394000	-3.16441600	-2.39427200
H	-2.28083900	-1.78620100	-2.99089500
H	-3.08006900	-3.33588400	-2.65224300
C	-2.71246200	-3.40522200	0.04976900
H	-1.83770400	-4.06275000	-0.01015400
H	-3.58912700	-3.98568200	-0.25591800
H	-2.85765800	-3.10924100	1.09422500
C	-2.03718900	1.41924500	3.02856900
H	-1.27707000	2.07111500	2.59973200
H	-1.55792200	0.55164600	3.48137800
H	-2.53190900	1.97612900	3.83038500
C	-3.89963000	-0.14261400	2.67276700
H	-3.28037400	-1.04672900	2.67248700
H	-4.84209400	-0.36650700	2.17220500
H	-4.11559700	0.11851600	3.71258400
C	1.93102500	-1.13869000	-0.09851400
C	3.15428700	-1.02906800	0.56183000
F	3.49489100	0.11346000	1.17951800
C	4.07895600	-2.06155500	0.66062400
F	5.23275900	-1.87782000	1.30249700
C	3.79147700	-3.29458500	0.09651200
F	4.66248600	-4.29757100	0.18185000
C	2.57116400	-3.47222000	-0.53672400
F	2.25435800	-4.65953800	-1.05520200
C	1.68049800	-2.40949400	-0.60805500
F	0.48217100	-2.69640100	-1.15616900
C	1.59916800	1.55287000	-0.11674100
C	1.35065000	2.75934200	0.53011700
F	0.39502000	2.87988900	1.48812800
C	2.02144900	3.95549600	0.28789700
F	1.69909200	5.05277100	0.97435900
C	3.01452700	4.00271600	-0.67017700
F	3.66631200	5.13360600	-0.92506200
C	3.30869900	2.83913600	-1.36493600
F	4.25592100	2.84776100	-2.30050300
C	2.60882600	1.67574700	-1.08521600
F	2.96940100	0.61884900	-1.82982000
H	-6.90238300	0.97553400	-0.74370300
H	-5.96584300	2.31951500	0.86309700
H	-4.67832300	2.90130800	-0.15690200
H	-1.22643700	-0.06522500	1.05005000
C	0.01716100	-0.41305400	1.46919300
C	0.69303500	0.19522000	2.56412100
C	-0.26399300	-1.79775400	1.65384100
C	1.06694200	-0.50462200	3.70174900
H	0.96625900	1.23876700	2.51373600
C	0.03425900	-2.49395000	2.81301400
H	-0.71514700	-2.34600900	0.83984300
C	0.72132100	-1.84742900	3.84002900
H	1.62124700	-0.00125800	4.48682200
H	-0.23166600	-3.54245700	2.90105300
H	0.99527700	-2.39110400	4.73943900

N20_P

B	1.10387700	0.06564300	0.07270200
C	0.07987500	-0.03050400	-1.23032900
C	0.58818200	-0.59156400	-2.41859600
H	1.58011300	-1.03189800	-2.39949400
C	-0.10375700	-0.58592900	-3.62261900
H	0.34737300	-1.03192400	-4.50421700
C	-1.36349600	0.00344200	-3.70354700
H	-1.91025200	0.02980900	-4.64057100
C	-1.89794800	0.57665900	-2.56074900
H	-2.87341700	1.05793700	-2.60671000
C	-1.19983100	0.55675200	-1.34380500
C	-1.85044100	1.25414700	-0.18222800
H	-2.40604000	2.12849100	-0.52473900
H	-1.11137900	1.54705100	0.55576000
N	-2.88328800	0.40991300	0.59140600
C	-3.44018100	1.15575000	1.82752400
C	-4.28022200	2.32686600	1.32253600
H	-4.68660600	2.83466900	2.20259500
H	-3.62456100	3.05423300	0.83595900
C	-5.41031200	1.89973900	0.39327100
C	-5.09464500	0.69688600	-0.46865400
C	-6.05270100	0.29577500	-1.40173500
C	-5.89376900	-0.87613700	-2.12134300
H	-6.64043400	-1.17714800	-2.84883500
C	-4.78395800	-1.67999200	-1.89017700
H	-4.67557600	-2.60781900	-2.43911100
C	-3.79196700	-1.32042400	-0.97819400
C	-3.95955400	-0.09988600	-0.29768500
C	-2.63654100	-2.26978500	-0.70830000
H	-1.73521800	-1.67729000	-0.54452200
C	-2.30251700	-3.18620300	-1.88666500
H	-1.35696000	-3.69267300	-1.68394700
H	-2.18515100	-2.60821900	-2.80716500
H	-3.06865400	-3.95383900	-2.03891400
C	-2.93215500	-3.09860800	0.55060700
H	-2.08095900	-3.74615300	0.78341400
H	-3.81458500	-3.72667200	0.39167700
H	-3.12974200	-2.46977700	1.42779200
C	-2.30138700	1.68074600	2.69714200
H	-1.75348400	2.50064800	2.23055000
H	-1.59842700	0.89362400	2.97040100
H	-2.74969700	2.06274400	3.61864300
C	-4.25493000	0.13717500	2.62496400
H	-3.58957700	-0.63278800	3.03106700
H	-5.03603900	-0.34293100	2.03121800
H	-4.72658100	0.64664400	3.46901400
C	2.19492900	-1.18466800	0.05506700
C	3.51152300	-1.06640000	0.49449600
F	4.00341400	0.13058800	0.85121700
C	4.39243900	-2.13469400	0.60774600
F	5.64612600	-1.94271700	1.02122100
C	3.95979400	-3.41462000	0.29690100
F	4.78630900	-4.45452700	0.40369900
C	2.64997400	-3.59656400	-0.11692500
F	2.20429800	-4.82271400	-0.40400400
C	1.80951900	-2.49568900	-0.21490200
F	0.53789700	-2.77788300	-0.58519200
C	1.82295800	1.54262100	-0.17767700
C	1.39470400	2.75413600	0.35537300
F	0.36940300	2.81067000	1.24285500
C	1.94358900	3.99436400	0.04543300
F	1.46924000	5.10498700	0.61672500
C	2.97849900	4.07012500	-0.86889800
F	3.52064500	5.24424100	-1.18707000
C	3.43694700	2.89826400	-1.45395500
F	4.42660400	2.94927300	-2.34590600
C	2.85374600	1.68804700	-1.10970300
F	3.35121000	0.61227800	-1.73905300
H	-6.93593800	0.91357800	-1.53927400
H	-6.29261100	1.63388000	0.98850800
H	-5.71637600	2.73743700	-0.24056400
H	-2.34422600	-0.38377600	0.97171200
C	0.50759700	-0.14335000	1.58952100
C	1.03253900	0.53150200	2.70432000
C	-0.36935300	-1.20566500	1.87986900
C	0.67604700	0.20034800	4.01043000
H	1.76297100	1.32033400	2.54886400
C	-0.74189500	-1.54654500	3.18231000
H	-0.69624900	-1.84860200	1.06844400
C	-0.22655300	-0.83263600	4.26059000
H	1.11433800	0.74702600	4.84056500
H	-1.40704000	-2.39099600	3.35109800
H	-0.50017600	-1.0188300	5.27850600

N21_TS

B	-0.73195200	-0.28195800	0.26542300
C	-0.02794700	0.76325700	1.66283700
C	-0.60572700	-1.83721100	2.35716900

H	-1.51625200	-2.28293400	1.96747800
C	-0.06697800	-2.3789700	3.52181100
H	-0.56562600	-3.20901100	4.01226800
C	1.10190600	-1.84840900	4.04783900
H	1.53693300	2.24925200	4.95769700
C	1.71780900	0.79688700	3.37836800
H	2.64888800	-0.38999100	3.76495700
C	1.18282400	-0.25920100	2.20571200
C	1.97575600	0.86309900	1.56671100
H	2.89015700	1.03855900	2.14645500
H	1.40045200	1.78839400	1.58639700
N	2.33185600	0.60296900	0.14950600
C	2.36778500	1.81516100	-0.68320500
C	3.66814400	2.57984600	-0.49721700
H	3.81587400	2.79826500	0.56909100
H	3.62756300	3.54091400	-1.01929800
C	4.83159700	1.74213600	-1.02622700
H	4.77834900	1.76469600	-2.12496400
C	4.68796400	0.29008500	-0.58975200
C	5.75348400	-0.59979200	-0.75214000
H	6.67109100	-0.24329500	-1.21035400
C	5.68202800	-1.92230300	-0.33600300
H	6.53460400	-2.58066700	-0.47775900
C	4.52497200	-2.40847100	0.27161000
C	3.44921800	-1.53924400	0.42419600
H	2.54077500	-1.89815600	0.89365300
C	3.50948000	-0.21286800	-0.01075700
C	6.16609300	2.36057000	-0.60167500
H	6.18995600	3.41979600	-0.87476200
H	7.02156800	1.87843900	-1.07853300
H	6.29323600	2.28254500	0.48245800
C	4.40242000	-3.84185800	0.71802300
H	3.88228300	-3.90985800	1.67735200
H	5.38262100	-4.31218400	0.82266700
H	3.82690100	-4.42418300	-0.00964500
C	-0.97196600	1.32455300	0.04187000
C	-0.93892300	2.28041200	1.05982100
F	-0.61920000	1.94920900	2.31670500
C	-1.23006700	3.62742500	0.86647000
F	-1.14257100	4.48783700	1.87945300
C	-1.63904200	4.07039200	-0.38146300
F	-1.92571600	5.35242800	-0.58183500
C	-1.77462500	3.14863100	-1.40859700
F	-2.22088000	3.54031700	-2.60132300
C	-1.47643400	1.81382100	-1.16754300
F	-1.74270500	0.97446900	-2.18131500
C	-2.23119200	-0.91655600	0.04862100
C	-3.17535700	-0.57303200	1.02160200
F	-2.79733700	0.17289700	2.07095800
C	-4.50871500	-0.95033300	0.98414300
F	-5.34876600	-0.59398600	1.95528200
C	-4.96756500	-1.70002500	-0.09016600
F	-6.24330300	-2.07150100	-0.15704800
C	-4.08090900	-2.04646600	-1.09448300
F	-4.50830900	-2.75367200	-2.14108700
C	-2.74848300	-1.64699500	-1.01636400
F	-1.99233300	-2.00975000	-2.06054400
H	1.11504500	-0.26934800	-0.39940200
H	1.48869400	2.41642600	-0.43899300
H	2.27848900	1.49646800	-1.72997500
C	0.47017700	-0.52825700	-2.38366400
C	0.64783300	-1.39955300	-3.44679000
C	0.76822000	-2.76653900	-3.20177600
C	0.73429800	-3.25809300	-1.89715000
C	0.56734900	-2.37948900	-0.83957600
C	0.39907500	-0.98816200	-1.04715600
H	0.40914900	0.53581900	-2.58135700
H	0.69696300	-1.02114100	-4.46213600
H	0.89988500	-3.45325600	-4.03280700
H	0.83858300	-4.32232600	-1.71336700
H	0.53366200	-2.76593900	0.17545600

N21_P

B	0.92159800	-0.26066800	0.07366500
C	-0.10656300	-0.78372700	-1.11527900
C	0.19079700	-1.91696200	-1.88859300
H	1.12786400	-2.43784800	-1.71805600
C	-0.65867100	-2.41116900	-2.87627200
H	-0.36791800	-3.29059800	-3.44354300
C	-1.85717100	-1.76505500	-3.15693700
H	-2.51195900	-2.11680500	-3.94760500
C	-2.20034400	-0.65326800	-2.39856900
H	-3.14446000	-0.14374600	-2.58469400
C	-1.36568200	-0.19691400	-1.37446700
C	-1.88069000	0.94704300	-0.54870700
H	-2.52932600	1.59762400	-1.13697600
H	-1.07997000	1.52992200	-0.09040200
N	-2.73053600	0.47108500	0.64566300

C	-3.11541000	1.61311000	1.53932600
C	-4.12949700	2.50280300	0.84824000
H	-3.68236600	2.95489500	-0.04336700
H	-4.37142100	3.33453500	1.51589400
C	-5.41252500	1.74842300	0.47299500
H	-6.07177400	1.75024000	1.35177200
C	-5.15379900	0.28699100	0.14066200
C	-6.21037900	-0.53946100	-0.25345500
H	-7.20625800	-0.11475000	-0.33786400
C	-6.02238500	-1.88678500	-0.53211100
H	-6.86837100	-2.49071500	-0.84744300
C	-4.76391300	-2.47946800	-0.41038700
C	-3.70511900	-1.67784200	0.00739800
H	-2.70656400	-2.09649900	0.10225400
C	-3.90620400	-0.32644300	0.26312200
C	-6.13078000	2.48050400	-0.66551600
H	-6.28984600	3.52935400	-0.39896400
H	-7.10475300	2.04072500	-0.88802700
H	-5.52694700	2.44571400	-1.57785100
C	-4.52428700	-3.92924100	-0.73512300
H	-3.75199200	-4.02106300	-1.50484900
H	-5.43409500	-4.41024800	-1.09911800
H	-4.17745400	-4.47688900	0.14607400
C	1.41890900	1.31761200	-0.07529400
C	1.15806700	2.19786700	-1.12228400
F	0.32223100	1.86310500	-2.12058300
C	1.69996300	3.47683700	-1.21598900
F	1.38363200	4.27335000	-2.23960900
C	2.57388100	3.92411600	-0.23962200
F	3.10138000	5.14497400	-0.30946000
C	2.88620800	3.08324900	0.81944000
F	3.72771500	3.49515100	1.76869300
C	2.31825700	1.81791700	0.87035100
F	2.67794000	1.05502200	1.91519600
C	2.34077500	-1.09692800	0.00487200
C	3.11262500	-1.00316400	-1.15385000
F	2.65259400	-0.29544800	-2.19863800
C	4.35056200	-1.60806100	-1.31685500
F	5.03135600	-1.48652900	-2.45844000
C	4.88415500	-2.34344900	-0.26755600
F	6.07103700	-2.93554900	-0.39465400
C	4.16818000	-2.45329500	0.91282600
F	4.67590000	-3.14888500	1.93263800
C	2.92855200	-1.82967900	1.03052000
F	2.32587100	-1.97044000	2.21882800
H	-2.09578400	-0.14041200	1.18551700
H	-2.19408500	2.13943300	1.79210300
H	-3.51831900	1.16289000	2.45071000
C	-0.11827900	0.46951200	2.46886500
C	-0.93061700	0.22747300	3.58174900
C	-1.55377500	-1.00587900	3.74328000
C	-1.32792300	-2.00555700	2.79401800
C	-0.50926300	-1.75116900	1.69716000
C	0.11291800	-0.50334800	1.48549500
H	0.33632900	1.45179500	2.36619300
H	-1.07988200	1.01107800	4.32018900
H	-2.19130300	-1.19561000	4.60110600
H	-1.79285200	-2.98059600	2.91082600
H	-0.35106100	-2.53899200	0.96170300

P1_TS

P	-1.30458300	0.22853100	-0.15310900
C	-0.92086400	-0.06724600	1.59118300
C	0.41175200	-0.44595400	1.83633300
B	1.49754300	-0.52851500	0.65053700
C	-1.87217400	0.01564500	2.61189000
C	-1.49377900	-0.27256200	3.91702200
C	-0.17662500	-0.64700300	4.18787300
C	0.75973000	-0.73163300	3.16200100
O	2.83296500	-0.92229300	1.09245600
O	1.66170800	0.74994100	-0.07755900
C	3.00053000	0.89955100	-0.25027100
C	3.66282100	1.86709900	-0.97874700
C	5.06500100	1.80734200	-1.00878700
C	5.75509800	0.80983700	-0.32989600
C	5.07505400	-0.17091700	0.40853000
C	3.69594800	-0.10689100	0.43918200
H	3.10831000	2.63924800	-1.50097100
H	5.61559600	2.55327600	-1.57269500
H	6.83917900	0.78471600	-0.37089600
H	5.60116300	-0.95500300	0.94134100
C	-1.30411700	2.01304800	-0.47238100
C	-2.98496300	-0.38931400	-0.45853400
C	-4.12804300	0.39141400	-0.25502700
C	-5.39007900	-0.16238400	-0.44660600
C	-5.51745000	-1.49280400	-0.84001500
C	-4.38236700	-2.27212600	-1.04646400
C	-3.11739300	-1.72311100	-0.86037000

H	-4.02930700	1.43093300	0.04586100
H	-6.27503100	0.44511200	-0.28661700
H	-6.50333200	-1.92163300	-0.98824300
H	-4.48039300	-3.30659600	-1.35864700
H	-2.22945500	-2.32951700	-1.02619600
C	-1.97569400	2.52646200	-1.58777500
C	-1.91794800	3.88744100	-1.86701100
C	-1.18199200	4.73781800	-1.04482500
C	-0.50179300	4.22539900	0.05673500
C	-0.55848200	2.86598200	0.34675000
H	-2.54936900	1.86567500	-2.23230000
H	-2.44461300	4.28289100	-2.72950500
H	-1.13660400	5.79944900	-1.26549900
H	0.07863900	4.88419100	0.6941440
H	-0.01629600	2.46082600	1.19501800
H	-2.89866500	0.29254400	2.38448800
H	-2.22118200	-0.20983600	4.72015100
H	0.11638900	-0.87624900	5.20833200
H	1.78501800	-1.02170300	3.37366700
H	-0.05888800	-0.78822600	-0.61965000
C	0.52181600	-2.97636100	-0.11017400
C	0.68459100	-4.12543900	-0.87129200
C	1.32670300	-4.04929100	-2.10942700
C	1.80033200	-2.82602700	-2.58023600
C	1.62874900	-1.67814000	-1.81481200
C	0.99090200	-1.72331700	-0.55902700
H	0.02649900	-3.03575600	0.85835900
H	0.32247000	-5.08183200	-0.50624600
H	1.45697300	-4.94702800	-2.70654600
H	2.29943100	-2.77181200	-3.54286900
H	1.98552500	-0.71934000	-2.18464600

P1_P

P	-1.58669100	0.05587900	0.13596000
C	-0.63869500	1.48671800	-0.42923300
C	0.74696600	1.60871100	-0.17937800
B	1.73514500	0.52733200	0.60890100
C	-1.36648000	2.44687400	-1.15372400
C	-0.71981500	3.56872200	-1.65031900
C	0.64397400	3.72510600	-1.41628800
C	1.34795200	2.76642200	-0.69572100
O	2.98640700	1.23568200	1.00482300
O	2.18328400	-0.52485200	-0.36683200
C	3.51634500	-0.35718600	-0.52712400
C	4.36835900	-1.06249500	-1.35269900
C	5.72976700	-0.71129500	-1.34853500
C	6.19861000	0.31345900	-0.53884200
C	5.32760400	1.02996900	0.29967400
C	3.99113900	0.68212300	0.29258400
H	3.98890700	-1.86430200	-1.97767500
H	6.41984800	-1.25322500	-1.98747400
H	7.25370900	0.56829800	-0.54944000
H	5.68087700	1.83070500	0.94028000
C	-1.13666700	-1.47253600	-0.69553700
C	-3.35008600	0.32588400	-0.16404100
C	-3.95203000	-0.08945300	-1.35464300
C	-5.30227400	0.16859600	-1.56846700
C	-6.04810200	0.83428900	-0.59827500
C	-5.44927400	1.24391900	0.59094400
C	-4.09953500	0.99048300	0.81102000
H	-3.36843300	-0.61541200	-2.10520900
H	-5.77278100	-0.15355600	-2.49136900
H	-7.10164100	1.03095300	-0.76815800
H	-6.03259900	1.75674300	1.34811700
H	-3.63003400	1.30568200	1.73989900
C	-1.75670200	-2.66143800	-0.28920100
C	-1.41957700	-3.85320300	-0.91226900
C	-0.46799300	-3.85748700	-1.93512200
C	0.14344200	-2.67550100	-2.33372700
C	-0.18788300	-1.47084800	-1.71511900
H	-2.48330300	-2.65317400	0.51971100
H	-1.88873800	-4.77970300	-0.59882100
H	-0.19935200	-4.79306900	-2.41518400
H	0.89397600	-2.68379400	-3.11667900
H	0.32157800	-0.55392500	-1.98951100
H	-2.43123000	2.32695100	-1.32559300
H	-1.27810100	4.31434700	-2.20645400
H	1.15953200	4.60321700	-1.79474900
H	2.40724200	2.90595200	-0.50316200
H	-1.53545800	-0.13966100	1.52023100
C	0.42298500	0.69082000	2.87645700
C	-0.39278300	0.17295500	3.87828300
C	-0.68545500	-1.19409100	3.89795300
C	-0.11978200	-2.03228200	2.93949200
C	0.70470500	-1.49957400	1.94637900
C	0.96554200	-0.12678800	1.87165800
H	0.62336500	1.76063900	2.84809200
H	-0.80925700	0.82706900	4.63951700

H	-1.33556500	-1.60167300	4.66665800
H	-0.32693600	-3.09909100	2.96209000
H	1.12597600	-2.14668800	1.17996700

P2_TS

C	-1.45578000	0.23046500	2.71948400
C	-0.66643800	0.06213700	1.57813100
C	0.66366300	-0.39149100	1.63617300
C	1.17195600	-0.67982200	2.90994700
C	0.40244900	-0.50965800	4.05664900
C	-0.91211600	-0.04805000	3.96665300
H	-2.48726400	0.56294500	2.63070400
H	2.19203400	-1.04784800	2.98178100
H	0.82326900	-0.74205000	5.03099600
H	-1.50988800	0.08132400	4.86344100
B	1.58112000	-0.56932000	0.29419600
P	-1.28900500	0.26546300	-0.10843100
C	-3.00218900	-0.33844700	-0.14462000
C	-4.10281300	0.44987400	0.21061600
C	-3.19476200	-1.67641900	-0.50637600
C	-5.38108200	-0.09927100	0.20600200
H	-3.95746700	1.49271000	0.48029100
C	-4.47513400	-2.22150000	-0.50441100
H	-2.34077800	-2.28966100	-0.78661300
C	-5.56688200	-1.43355400	-0.14998300
H	-6.23300800	0.51407900	0.48108000
H	-4.62032900	-3.25941800	-0.78534000
H	-6.56569000	-1.85826700	-0.15322400
C	-1.30721500	2.02378300	-0.55473800
C	-2.18527900	2.49925200	-1.53476200
C	-0.37094900	2.88781000	0.02167000
C	-2.13856600	3.83511400	-1.92011700
H	-2.90724800	1.82836500	-1.99227100
C	-0.32579900	4.21994600	-0.37505200
H	0.32758800	2.50243900	0.75744700
C	-1.20949900	4.69598000	-1.34120700
H	-2.82592800	4.20202800	-2.67543500
H	0.40439800	4.88771400	0.07110700
H	-1.17255800	5.73728100	-1.64512300
O	2.94006100	-0.97068100	0.54797800
O	1.63597100	0.64964100	-0.50130800
C	3.00113800	0.94236100	-0.79298500
C	3.18416400	2.45577900	-0.77224600
H	4.23689600	2.72666000	-0.90810600
H	2.60476400	2.90311100	-1.58558700
H	2.83064900	2.88352600	0.16846800
C	3.35656900	0.41008100	-2.18275500
H	3.31902900	-0.68176000	-2.20211300
H	2.63462200	0.80167500	-2.90601500
H	4.35797600	0.72937300	-2.48838700
C	3.76381900	0.17128600	0.33547000
C	5.16197800	-0.29742500	-0.04676000
H	5.79818400	0.55032900	-0.32335600
H	5.62043400	-0.80500800	0.80632900
H	5.12604700	-1.00218700	-0.87873400
C	3.83867200	0.98754300	1.63055100
H	2.85297300	1.36856300	1.91401200
H	4.19684100	0.33843900	2.43459500
H	4.52901600	1.83144200	1.53518800
H	-0.16246900	-0.85511400	-0.64470800
C	0.79261400	-1.87589400	-0.67299400
C	0.99097500	-2.01268800	-2.06008100
C	0.90526700	-3.24643800	-2.69344800
C	0.61113100	-4.38636200	-1.94550700
C	0.39331400	-4.28379000	-0.57152000
C	0.47326300	-3.04296000	0.05036700
H	1.18090400	-1.11446900	-2.64252200
H	1.05781900	-3.32496300	-3.76569200
H	0.54399600	-5.35397800	-2.43414900
H	0.16405800	-5.17274700	0.00845100
H	0.30354000	-2.96741100	1.12269400

P2_P

C	-1.17179100	0.52116600	2.65651500
C	-0.44630700	0.15642800	1.51293000
C	0.79303200	-0.49765300	1.56055300
C	1.28183900	-0.78265900	2.84441000
C	0.58437300	-0.42840000	3.99339900
C	-0.64574800	0.23006100	3.90565500
H	-2.13655400	1.01758800	2.57389900
H	2.24200000	-1.28786100	2.91288400
H	0.99465500	-0.66400400	4.97140300
H	-1.18953500	0.50466100	4.80331800
B	1.61034300	-0.93682100	0.19527600
P	-1.16744700	0.52648000	-0.08870100
C	-2.86237400	-0.08373100	-0.15105000
C	-3.96028500	0.66460500	0.28344100
C	-3.02519400	-1.40856400	-0.57455600

C	-5.22608500	0.08754200	0.28461500
H	-3.82867500	1.69329700	0.60880400
C	-4.29547200	-1.97551800	-0.56606000
H	-2.16746200	-1.99509800	-0.90203700
C	-5.39175800	-1.22963700	-0.13965400
H	-6.08223500	0.66504400	0.61667700
H	-4.42301200	-3.00170300	-0.89373000
H	-6.38099400	-1.67576500	-0.13665900
C	-1.10202900	2.29077200	-0.45365900
C	-2.04386900	2.90187300	-1.28679600
C	-0.02440400	3.01929600	0.05739400
C	-1.91153700	4.25201500	-1.59455300
H	-2.87286400	2.32895000	-1.69191500
C	0.10211900	4.36691300	-0.26147000
H	0.71208000	2.51891000	0.67953100
C	-0.84138500	4.98182300	-1.08141700
H	-2.64113200	4.73218400	-2.23776800
H	0.94065700	4.93456900	0.12762500
H	-0.74070000	6.03436600	-1.32567900
O	3.01371900	-1.23995600	0.46892000
O	1.63618500	0.26112300	-0.70807200
C	2.98931900	0.51068500	-1.06786000
C	3.18582800	2.00944000	-1.26609100
H	4.23217800	2.24071700	-1.49325100
H	2.56878400	2.35351400	-2.10209300
H	2.89273200	2.57053300	-0.37599900
C	3.30285800	-0.22220000	-2.37585500
H	3.21302200	-1.30290100	-2.23500400
H	2.58445300	0.09277400	-3.13933200
H	4.31019800	0.00522400	-2.73850700
C	3.78313700	-0.10097200	0.13314700
C	5.19804000	-0.55338800	-0.21169400
H	5.79741400	0.27729600	-0.60086100
H	5.68685400	-0.93562800	0.68885400
H	5.17768900	-1.35609200	-0.95038600
C	3.83935400	0.86551200	1.32408300
H	2.84786100	1.26739400	1.55400000
H	4.19265500	0.31761500	2.20224600
H	4.52291200	1.70032800	1.13780200
H	-0.50390900	-0.18116700	-1.08615800
C	0.79607800	-2.20094100	-0.47499700
C	0.51735700	-2.25943400	-1.84993300
C	-0.23096100	-3.29274600	-2.41518900
C	-0.73182200	-4.31122400	-1.60649600
C	-0.47255400	-4.28197400	-0.23674800
C	0.27369700	-3.23973300	0.31115000
H	0.88638000	-1.45855300	-2.48790400
H	-0.42620100	-3.30234000	-3.48424100
H	-1.31566800	-5.11908100	-2.03799400
H	-0.85899600	-5.06939900	0.40460900
H	0.44543100	-3.22395300	1.38593000

P3_TS

C	1.51058200	-0.42621100	-0.22666500
C	0.15706100	-0.39497200	-0.29630500
C	2.37799900	-1.04689600	-1.27442100
C	3.07911700	-2.24129800	-1.07619800
C	2.54226400	-0.35667300	-2.48110400
C	3.92909400	-2.72831700	-2.06593000
H	2.95337300	-2.79595800	-0.15372500
C	3.39551500	-0.84325900	-3.46599900
H	1.99156100	0.56805400	-2.63489400
C	4.09569500	-2.02923000	-3.25826400
H	4.46007300	-3.66083200	-1.90292500
H	3.51379200	-0.29538700	-4.39533600
H	4.76251100	-2.41069400	-4.02476900
C	-0.39622800	-1.25885100	-1.40270000
C	-0.95713600	-0.76155500	-2.57582600
C	-0.26339400	-2.64929000	-1.33959000
C	-1.45898500	-1.58296300	-3.57736700
C	-0.75959800	-3.49462500	-2.32026400
C	-1.37343900	-2.95927800	-3.44367400
F	0.39951600	-3.22845900	-0.33409600
F	-0.62388800	-4.81308100	-2.20189500
F	-1.84331400	-3.75743200	-4.39580400
F	-1.98623200	-1.05098800	-4.67697100
F	-0.95219500	0.55024600	-2.82717100
B	-0.92177400	0.53059400	0.56815900
P	2.35696900	0.41109500	1.15632500
C	3.77756400	1.35117200	0.49625200
C	3.82080600	2.73432200	0.69531200
C	4.83544000	0.71563800	-0.17054700
C	4.89685600	3.47783800	0.21688000
H	3.01126900	3.23159800	1.21686900
C	5.90522700	1.46404200	-0.64685200
H	4.82246300	-0.36031600	-0.31700300
C	5.93580700	2.84470800	-0.45675300
H	4.91988600	4.55119900	0.37287100

H	6.71677900	0.96638500	-1.16755900
H	6.77365800	3.42495200	-0.83035200
C	3.09136900	-0.94838400	2.12901800
C	2.40938700	-2.16387500	2.27090600
C	4.28157900	-0.74273200	2.83472700
C	2.92081600	-3.15957000	3.09786800
H	1.49763100	-2.34547800	1.70450600
C	4.78780700	-1.74219600	3.65964800
H	4.81827700	0.19594000	2.73000800
C	4.10925100	-2.95050100	3.79393700
H	2.39232300	-4.10282700	3.19135500
H	5.71653800	-1.57589500	4.19584500
H	4.50714200	-3.72895500	4.43673500
C	-2.34910800	-0.19704500	0.92342000
C	-2.47136900	-1.55929900	1.17396700
C	-3.49902700	0.53342300	1.22758400
C	-3.64202700	-2.17935900	1.59217600
C	-4.69302900	-0.03673500	1.64648400
C	-4.77132200	-1.41048400	1.82110300
C	-1.11517600	1.92065000	-0.29483400
C	-0.19242700	2.95740200	-0.27468800
C	-2.17488200	2.14246200	-1.17522000
C	-0.29408800	4.13247700	-1.00808800
C	-2.32738200	3.29392200	-1.93298900
C	-1.37974000	4.30467300	-1.84889000
F	-3.67570100	-3.49618300	1.79923400
F	-1.39372700	-2.36071600	1.08441500
F	-3.49261300	1.87300000	1.15410400
F	-5.75581900	0.72567900	1.90066100
F	-5.90463100	-1.97671000	2.22622200
F	-3.10054500	1.19588200	-1.37354800
F	-3.36495700	3.42896600	-2.75664700
F	-1.50973100	5.41407600	-2.56959400
F	0.63781100	5.08103500	-0.90067300
F	0.89237500	2.87537500	0.52833600
H	0.77316500	0.98244700	1.79171900
C	-1.29826900	2.25736500	4.08844400
C	-1.28085600	1.13987600	4.92010300
C	-0.82543700	-0.09271200	4.44006800
C	-0.40890200	-0.19687400	3.12781400
C	-0.43185000	0.91188500	2.23518000
C	-0.87313100	2.14560900	2.77197000
H	-1.64930500	3.21122500	4.46758600
H	-1.61809300	1.22710200	5.94858800

H	-0.80635300	-0.95923700	5.09228100
H	-0.07280300	-1.15858600	2.74906700
H	-0.90130500	3.02395000	2.13613800

P3_P

C	1.33229300	-0.34555400	-0.36697300
C	-0.02197000	-0.29333200	-0.30751100
C	2.09828600	-0.60236200	-1.63457100
C	2.75549800	-1.80589300	-1.90717600
C	2.18910500	0.44802300	-2.55613900
C	3.49202800	-1.95122300	-3.08161500
H	2.67885000	-2.63436000	-1.21227300
C	2.92586600	0.30107900	-3.72537700
H	1.66907300	1.37851300	-2.34165700
C	3.58522100	-0.89851000	-3.98727000
H	3.98943600	-2.89334700	-3.28853800
H	2.98632200	1.12278900	-4.43150700
H	4.16086000	-1.01474000	-4.89992400
C	-0.66011000	-0.80036600	-1.57731300
C	-1.30396200	0.00059300	-2.51767600
C	-0.56066800	-2.15553400	-1.90639600
C	-1.90226600	-0.51803800	-3.65994000
C	-1.15320400	-2.70116600	-3.03375100
C	-1.84071300	-1.87821800	-3.91417000
F	0.16177900	-2.99113200	-1.15061700
F	-1.04004300	-4.00217000	-3.28997400
F	-2.40480900	-2.38617200	-5.00399500
F	-2.50190900	0.29407000	-4.52580900
F	-1.29431600	1.33093400	-2.40704500
B	-1.00724200	0.35919500	0.87995000
P	2.49975800	-0.02670700	0.98126400
C	3.88528300	1.00718700	0.45128900
C	3.97867800	2.30294400	0.96930900
C	4.85892500	0.53140900	-0.43580300
C	5.03786300	3.12291900	0.59016000
H	3.22202900	2.67611700	1.65214700
C	5.91052100	1.35842000	-0.80917200
H	4.79133700	-0.47476400	-0.83869500
C	5.99957000	2.65244500	-0.29814700
H	5.10705900	4.12886300	0.98955200
H	6.66020900	0.99141800	-1.50188300
H	6.82321200	3.29434100	-0.59349300
C	3.16829900	-1.61427300	1.54020800
C	2.42260800	-2.78525400	1.37256100

C	4.39628900	-1.64508100	2.20546200
C	2.91241300	-3.98609900	1.87516000
H	1.47328100	-2.75669200	0.84321800
C	4.87495600	-2.85028400	2.70984000
H	4.97662500	-0.73473200	2.32709700
C	4.13374800	-4.01744800	2.54549000
H	2.33827900	-4.89687700	1.74263300
H	5.82717000	-2.87677600	3.22879000
H	4.51086800	-4.95641900	2.93759100
C	-2.42749200	-0.43388500	1.12336000
C	-2.61008500	-1.80075000	0.96802900
C	-3.49062600	0.20356300	1.76576200
C	-3.77227000	-2.48319700	1.31163900
C	-4.67250300	-0.42635800	2.12384500
C	-4.82112700	-1.78611900	1.88539000
C	-1.21103200	1.91217400	0.33757700
C	-0.16088500	2.82310700	0.36128900
C	-2.36788300	2.42600900	-0.24972000
C	-0.23552400	4.14184900	-0.06831100
C	-2.50197200	3.73574400	-0.68859600
C	-1.42859900	4.60933600	-0.59182100
F	-3.87074900	-3.79935500	1.11290400
F	-1.60420100	-2.57355600	0.50620600
F	-3.39560900	1.50171600	2.09046200
F	-5.65734200	0.25415900	2.71099500
F	-5.94661000	-2.41195800	2.22436800
F	-3.41730900	1.63399000	-0.50026100
F	-3.64258400	4.15210900	-1.23681800
F	-1.53788000	5.86687600	-1.01317500
F	0.82843000	4.94531800	0.01562600
F	1.05969800	2.44906800	0.82111800
H	1.98118100	0.65385300	2.07980000
C	0.60627600	1.28384900	4.38786700
C	1.07613600	0.04038800	4.80955700
C	0.78723300	-1.08903200	4.04698200
C	0.08282300	-0.95666700	2.85268300
C	-0.31853900	0.29524400	2.35688800
C	-0.08840400	1.40214900	3.18487200
H	0.78645700	2.16531000	4.99671500
H	1.63394900	-0.04948800	5.73644800
H	1.11826700	-2.07040200	4.37602600
H	-0.14633000	-1.84870500	2.27243300
H	-0.43774100	2.38294000	2.87429700

P4_TS

C	-0.42515000	-0.47085800	-1.75975700
H	-0.96772600	-0.72169600	-2.67643000
H	0.13206100	0.45124500	-1.93824100
C	0.53141300	-1.59754500	-1.31645600
H	-0.07701300	-2.50011300	-1.16760700
H	1.17792100	-1.82937100	-2.17131200
P	-1.53390400	-0.18491900	-0.32319500
B	1.49463100	-1.37327300	-0.00450600
C	1.06248900	-0.06048600	1.20482000
C	1.21692300	-0.43965000	2.55260300
C	1.28916300	1.29870500	0.88356500
C	1.60446900	0.47763700	3.52415500
C	1.67993900	2.21595500	1.84466000
C	1.83790200	1.80434100	3.17058700
C	-2.17817900	1.52446600	-0.18331600
C	-2.59637400	1.88869800	1.11998100
C	-2.18355300	2.49727800	-1.20494700
C	-3.04159000	3.18428800	1.36260600
C	-2.63064300	3.78727300	-0.90724400
C	-3.06886300	4.15184700	0.36060500
H	-3.36413600	3.44853600	2.36706700
H	-2.63403700	4.53013200	-1.70199500
C	-2.88698200	-1.41950700	-0.32627600
C	-3.98274400	-1.37642200	-1.21281900
C	-2.79686600	-2.45871200	0.62763200
C	-4.96979800	-2.35627400	-1.11941700
C	-3.80782600	-3.41881700	0.67699500
C	-4.90380900	-3.38269500	-0.18020800
H	-5.81264500	-2.31806800	-1.80590300
H	-3.73285000	-4.21981800	1.40852800
C	-4.11530000	-0.32434100	-2.28329200
H	-4.05579600	0.68668600	-1.87176100
H	-3.32249300	-0.42979400	-3.03145500
H	-5.07048300	-0.42603800	-2.80192100
C	-5.99994600	-4.41038400	-0.08030300
H	-6.78518800	-4.07060300	0.60306700
H	-6.46397900	-4.58940500	-1.05307700
H	-5.61804000	-5.36027900	0.30042500
C	-1.64359800	-2.59046600	1.59633400
H	-0.69047100	-2.74137300	1.07955700
H	-1.53169700	-1.69762600	2.22009600
H	-1.80602200	-3.44475100	2.25669200
C	-1.70869800	2.25194800	-2.61689000

H	-0.62165900	2.36180800	-2.69356600
H	-1.96357000	1.25716300	-2.97914500
H	-2.15708600	2.98328000	-3.29269000
C	-3.57572900	5.54153300	0.64161800
H	-3.29557100	5.86732800	1.64645500
H	-3.17864300	6.26166600	-0.07710700
H	-4.66832800	5.57535000	0.57777600
C	-2.54560300	0.91828100	2.27484800
H	-3.12813500	0.01250900	2.07563100
H	-1.51060000	0.61633900	2.47895000
H	-2.94503400	1.38220600	3.17866100
H	-0.06528000	-0.39273500	0.68335300
H	1.48321400	-2.34496000	0.70565200
C	4.08761300	-1.42790900	0.41897600
C	3.02308400	-0.94981100	-0.34062100
C	3.37736300	-0.06311300	-1.35014200
C	4.68281500	0.32131400	-1.62133400
C	5.71102700	-0.19546000	-0.84571400
C	5.41162000	-1.07459400	0.18462900
F	3.86758600	-2.25994700	1.44474200
F	6.39564500	-1.56522900	0.94049200
F	6.97363500	0.15874900	-1.08139700
F	4.95792500	1.18101400	-2.60515400
F	2.41845200	0.50079200	-2.11538800
H	1.05497600	-1.47978500	2.82259500
H	1.73268300	0.15865200	4.55367400
H	2.14449500	2.52097900	3.92649700
H	1.86377800	3.25010600	1.57157800
H	1.16614900	1.62503700	-0.14849400

P4_P

C	0.03743000	-0.95989800	-0.81923200
H	-0.30581300	-1.29217600	-1.80460000
H	0.79481900	-0.19100900	-0.97140700
C	0.61024900	-2.10888700	0.04423300
H	-0.19122200	-2.58428800	0.62717900
H	0.95505800	-2.87983600	-0.64975300
P	-1.39333900	-0.16871000	-0.01418300
B	1.83487700	-1.61926200	1.03982300
C	1.26050100	-0.39116600	1.93818600
C	0.53337700	-0.65277600	3.11246900
C	1.27498600	0.94744600	1.50734600
C	-0.18420600	0.33759900	3.78208100
C	0.55254400	1.95066000	2.15561200
C	-0.19218700	1.64657500	3.29396400
C	-1.75778200	1.57844300	-0.39372000
C	-2.68350200	2.21584100	0.46459200
C	-1.13428200	2.30850000	-1.42848000
C	-2.96492800	3.56620100	0.26811700
C	-1.45704700	3.65739200	-1.57661300
C	-2.35963500	4.30831600	-0.74102500
H	-3.67842900	4.04987600	0.93037700
H	-0.97805400	4.21724000	-2.37627400
C	-2.85512600	-1.22624900	-0.24899700
C	-3.50948300	-1.25922600	-1.49970100
C	-3.30912900	-2.03105700	0.81784600
C	-4.60599200	-2.10028400	-1.65903400
C	-4.41475000	-2.85572700	0.60016200
C	-5.07414600	-2.90770300	-0.62311800
H	-5.10905800	-2.12790700	-2.62283300
H	-4.76577200	-3.47912000	1.41825300
C	-3.07098200	-0.42010000	-2.67501900
H	-3.13358900	0.65011300	-2.45792100
H	-2.03981200	-0.64153400	-2.96722800
H	-3.70120100	-0.62719000	-3.54126700
C	-6.26105500	-3.80843000	-0.83657700
H	-7.14285200	-3.22783800	-1.12270200
H	-6.06511300	-4.52311700	-1.64105600
H	-6.50157600	-4.37110700	0.06711700
C	-2.67955500	-2.04228300	2.19178100
H	-1.61842600	-2.30359000	2.16757100
H	-2.75553500	-1.06694900	2.68456500
H	-3.18756300	-2.77204500	2.82400800
C	-0.11379900	1.74771400	-2.39022500
H	0.87373200	1.68016500	-1.92253100
H	-0.37222700	0.75620600	-2.76248300
H	-0.02067600	2.41020000	-3.25231100
C	-2.65180100	5.77449600	-0.91416400
H	-1.85733900	6.37815300	-0.46382700
H	-2.70907900	6.04337200	-1.97175100
H	-3.59318600	6.05082400	-0.43498500
C	-3.36578200	1.51489600	1.61359500
H	-3.91689300	0.62628600	1.29210400
H	-2.63099700	1.21361800	2.36952700
H	-4.07513400	2.19072700	2.09357300
H	-1.16622500	-0.17689400	1.36191600
H	2.14873700	-2.54876600	1.75845300
C	4.25344500	-0.64085800	0.94119300

C	3.17038600	-1.13257400	0.20891500
C	3.39048000	-1.16201100	-1.16343000
C	4.57042100	-0.75027800	-1.77760200
C	5.61140900	-0.27495200	-0.99962900
C	5.45033000	-0.22004400	0.37861600
F	4.16876200	-0.55992400	2.27569500
F	6.44693600	0.23761000	1.14098100
F	6.75202800	0.12569600	-1.56422800
F	4.70371100	-0.80965500	-3.10721800
F	2.43736200	-1.60571900	-2.01667100
H	0.52504900	-1.67124000	3.49728800
H	-0.73651000	0.09384100	4.68666400
H	-0.75258500	2.42313600	3.80717000
H	0.56713600	2.96838600	1.77304400
H	1.86450800	1.20990400	0.62789100

P5_TS

P	1.83642700	-0.58371100	0.43221500
B	-1.31354100	-0.59468800	0.42817500
C	0.92427900	-1.09387000	1.95300400
C	-0.61331400	-0.88288700	1.88700500
C	3.39018000	-1.56823800	0.44540800
C	5.59924200	-2.05420000	1.30166300
C	5.76425000	-3.08947900	0.38403600
C	4.71934000	-3.35731300	-0.49422300
C	3.53430800	-2.61943300	-0.48412400
C	2.30713500	1.18697300	0.28612700
C	1.97861700	2.19922800	1.21574900
C	2.22552600	3.53008600	0.87693800
C	2.78098400	3.90257400	-0.34376800
C	3.14438200	2.88957700	-1.22373400
C	2.91346600	1.54421100	-0.94194600
C	-2.94340100	-0.78661300	0.40601500
C	-3.73984900	-1.21259200	1.46700300
C	-5.12044900	-1.36387800	1.38191000
C	-5.77396800	-1.06975900	0.19688500
C	-5.03255600	-0.62200700	-0.88598200
C	-3.65820600	-0.48589400	-0.75507700
C	-1.02277900	0.96648600	-0.04368000
C	-1.54954700	1.95975200	0.78894500
C	-1.40711900	3.32442200	0.57729800
C	-0.73073600	3.76882300	-0.54775900
C	-0.21047200	2.83558100	-1.42618500
C	-0.36415000	1.48118500	-1.15536000
C	7.04683100	-3.87693000	0.33227600
C	2.45396300	-3.00389200	-1.46586800
C	1.38193600	1.94277000	2.57772700
C	2.91301000	5.35162900	-0.72549800
C	3.29851200	0.53244200	-1.99272600
F	-3.21437800	-1.50408000	2.66731700
F	-5.81950900	-1.78587500	2.43629200
F	-7.09335700	-1.21190400	0.09888600
F	-5.64223100	-0.32448600	-2.03428500
F	-3.01366700	-0.02979100	-1.84342100
F	-2.23073600	1.61918100	1.89684000
F	-1.91102700	4.20430700	1.44322800
F	-0.55296000	5.07192800	-0.76220100
F	0.45638100	3.24224400	-2.50767600
F	0.24056000	0.66494500	-2.04324800
H	1.35312000	-0.71173100	2.87978300
H	1.15045100	-2.16867000	1.94238700
H	-0.89096000	-0.06275800	2.55218600
H	-1.06039300	-1.75951400	2.35865200
H	6.39928100	-1.83648800	2.00570000
H	4.82461300	-4.16590400	-1.21362200
H	3.58655500	3.15133900	-2.18193400
H	6.89134200	-4.85917700	-0.11922200
H	7.79860100	-3.35097400	-0.26533300
H	7.46371900	-4.01923900	1.33227500
H	2.07108500	-2.14520100	-2.02280000
H	1.59853100	-3.46171300	-0.95940300
H	2.83889200	-3.72971200	-2.18491200
H	1.17493300	2.89272400	3.07449700
H	0.44140000	1.39392100	2.52278400
H	2.06938400	1.37991300	3.21660600
H	3.70410000	5.50012700	-1.46378900
H	3.12623300	5.97671200	0.14500200
H	1.97203000	5.69959400	-1.16415300
H	3.63706200	1.04267000	-2.89619300
H	4.10043600	-0.12902900	-1.65228500
H	2.43999800	-0.08727400	-2.26507500
C	4.43607500	-1.28765300	1.35133600
C	4.34646000	-0.19741900	2.38952100
H	4.25157300	0.79135700	1.93219300
H	3.48093800	-0.34477900	3.04198500
H	5.23802600	-0.19903600	3.01915800
H	1.94633300	4.30518500	1.58765800
H	0.37735800	-1.22304000	-0.44031900

C	-0.78091800	-3.15103700	-0.06175900
C	-0.74581500	-1.87628200	-0.68935000
C	-0.96619100	-1.87316700	-2.08862600
C	-1.22760100	-3.04074200	-2.79547600
C	-1.28145700	-4.26423400	-2.13404500
C	-1.05600500	-4.31710100	-0.75784900
H	-0.61574300	-3.22178900	1.00845500
H	-0.93429300	-0.93828200	-2.62867800
H	-1.39758800	-2.99408800	-3.86621700
H	-1.49567600	-5.17432300	-2.68607800
H	-1.09547900	-5.26664800	-0.23383400

P5_P

P	-1.83476300	-0.58054800	-0.44915800
B	1.58155800	-0.64114300	-0.11257300
C	-0.62737900	-1.34181100	-1.58622100
C	0.83282800	-0.86895000	-1.57921800
C	-3.30163400	-1.64775700	-0.49970000
C	-5.35883100	-2.35054200	-1.53301000
C	-5.51077700	-3.36759600	-0.59487800
C	-4.53442200	-3.51209900	0.39107400
C	-3.42254800	-2.67645400	0.46255000
C	-2.30570700	1.17498000	-0.50035300
C	-1.97842900	2.06228200	-1.54020500
C	-2.39220600	3.39344900	-1.43483900
C	-3.09935500	3.86951100	-0.33833500
C	-3.41880400	2.96799100	0.67790700
C	-3.03888300	1.63195000	0.62576300
C	3.21608900	-0.49976000	-0.36419500
C	3.90804200	-1.46438000	-1.09809200
C	5.27859900	-1.44786000	-1.32196200
C	6.04607600	-0.43253500	-0.77478300
C	5.41995700	0.53567500	-0.00811500
C	4.04433500	0.47714200	0.18587100
C	0.97354200	0.80493300	0.47786600
C	1.17501000	1.95849700	-0.28915600
C	0.63802500	3.20441100	-0.01209500
C	-0.13479700	3.37350500	1.12637300
C	-0.34094600	2.28520300	1.95077600
C	0.19835200	1.04691200	1.60969900
C	-6.68336100	-4.31012700	-0.64973900
C	-2.40988300	-2.90562800	1.56147700
C	-1.16624100	1.71447500	-2.76285500
C	-3.45788600	5.32440300	-0.21724500
C	-3.37947400	0.74947800	1.80303700
F	3.26036900	-2.51676500	-1.63547700
F	5.86132100	-2.40286700	-2.05081700
F	7.36342000	-0.39366500	-0.97413800
F	6.14199300	1.51420500	0.54300700
F	3.54726700	1.44777100	0.97502700
F	1.92560800	1.90472400	-1.40561300
F	0.80901700	4.22414200	-0.85617600
F	-0.70409000	4.54894500	1.39654100
F	-1.14058400	2.40463600	3.01599400
F	-0.19123800	0.04345100	2.43335400
H	-1.08578000	-1.36197700	-2.58189800
H	-0.69424300	-2.37134900	-1.21031200
H	0.91446800	0.04347100	-2.17389400
H	1.36427400	-1.63528800	-2.15030600
H	-6.10837200	-2.22666900	-2.31053300
H	-4.63877500	-4.30415500	1.12854900
H	-3.96393800	3.31998000	1.54982400
H	-7.12262400	-4.44740600	0.34166000
H	-7.45962200	-3.94073900	-1.32252600
H	-6.36745700	-5.29498100	-1.00771200
H	-2.36767800	-2.06054400	2.25820400
H	-1.39373300	-3.06425800	1.18236200
H	-2.68164900	-3.79064300	2.13862600
H	-1.58907300	2.21124700	-3.63964500
H	-0.13884100	2.07494900	-2.64251600
H	-1.11666800	0.64923900	-2.97450200
H	-4.38816800	5.46039700	0.33873600
H	-3.56470600	5.79192300	-1.19828600
H	-2.66292900	5.84772800	0.32259900
H	-3.96218800	1.31422100	2.53161500
H	-3.96452500	-0.12702400	1.50888800
H	-2.47359100	0.40764200	2.31436400
C	-4.26801400	-1.48203700	-1.50849800
C	-4.18546400	-0.39709200	-2.55506500
H	-4.45020900	0.57959500	-2.13832300
H	-3.18335100	-0.30523700	-2.98535800
H	-4.87229800	-0.61460300	-3.37492600
H	-2.12412600	4.07941100	-2.23510600
H	-1.29704400	-0.78874900	0.81951200
C	1.16322000	-3.21262400	0.41761300
C	1.43655600	-1.92046600	0.89273100
C	1.74949100	-1.82351300	2.25928100
C	1.71893700	-2.92171900	3.11069100

C	1.39262800	-4.18382800	2.61475800
C	1.13264900	-4.32865100	1.25720800
H	1.01322800	-3.37181400	-0.64797700
H	2.03298000	-0.85447700	2.66525000
H	1.96023800	-2.79762300	4.16256800
H	1.36891000	-5.04527300	3.27524600
H	0.91763700	-5.31045900	0.84403700

P6_TS

P	-1.91274700	-0.44426100	-0.47731000
C	-1.33380100	-0.40784500	-2.23040900
H	-2.19004100	-0.46564400	-2.90759800
H	-0.78810000	0.51247300	-2.43019000
C	-0.37794500	-1.59553500	-2.41281200
H	-0.76060700	-2.46233500	-1.85363300
H	-0.41663200	-1.89964000	-3.46495400
C	1.08443600	-1.28667000	-2.04662500
H	1.46140300	-0.59315000	-2.80497100
H	1.64344400	-2.20474100	-2.23540900
B	1.50784200	-0.67749000	-0.57385700
C	-2.52161500	1.16212500	0.18556400
C	-2.53534200	2.40129600	-0.49269300
C	-1.99005200	2.62900800	-1.88270800
H	-2.15933600	3.66590800	-2.17890000
H	-2.45326300	1.99044200	-2.63808900
H	-0.91070400	2.45311000	-1.91627100
C	-3.04593400	3.52004800	0.16571900
H	-3.05234200	4.47173200	-0.36094000
C	-3.52046000	3.47068900	1.47357900
C	-4.01065700	4.71619200	2.16104400
H	-4.60653400	4.47516400	3.04393200
H	-4.61906400	5.32718300	1.48938400
H	-3.16187200	5.32744000	2.48467100
C	-3.46684400	2.25045800	2.13583300
H	-3.80433100	2.18808400	3.16758300
C	-2.96517700	1.10235400	1.52544800
C	-2.87345400	-0.15636700	2.35414800
H	-1.86218500	-0.57845800	2.30790300
H	-3.57611700	-0.92588500	2.01936600
H	-3.09042000	0.06642000	3.40034700
C	-3.43051000	-1.50802800	-0.54404600
C	-4.61628300	-1.04539500	-1.15992200
C	-4.77498200	0.31997600	-1.78326000
H	-5.65252800	0.33127300	-2.43250600
H	-3.91385200	0.60991600	-2.38609300
H	-4.90627000	1.09077000	-1.01796000
C	-5.74032800	-1.87418100	-1.17978200
H	-6.64465700	-1.50854600	-1.66058900
C	-5.74569200	-3.13752300	-0.60083800
C	-6.96496000	-4.01943700	-0.65353200
H	-6.84637600	-4.79577100	-1.41618200
H	-7.86065800	-3.44481700	-0.89838400
H	-7.12857600	-4.52207200	0.30313300
C	-4.57681200	-3.57032400	0.01698100
H	-4.55475500	-4.55140800	0.48606500
C	-3.42416400	-2.78657500	0.05449000
C	-2.21020800	-3.37225700	0.72480800
H	-1.71381700	-2.67248400	1.40311300
H	-1.47117500	-3.67961700	-0.01922000
H	-2.48129100	-4.26112900	1.29833300
C	1.19662000	0.95393000	-0.47424500
C	0.52569400	1.67603400	0.50594400
F	-0.08876700	1.04828500	1.52667100
C	0.41891000	3.06254900	0.54615100
F	-0.27491200	3.65594500	1.51713800
C	1.03334400	3.81950200	-0.43485500
F	0.94594600	5.14751300	-0.42598000
C	1.75267000	3.16505800	-1.42636400
F	2.38597900	3.87401200	-2.36030100
C	1.82244300	1.77741900	-1.42208600
F	2.59673800	1.24396800	-2.37975800
C	3.13012700	-0.79284700	-0.26948100
C	4.09658500	-1.38092900	-1.08454200
F	3.78707200	-1.93690900	-2.26611300
C	5.44893100	-1.44343300	-0.75942900
F	6.31265700	-2.03444000	-1.58653200
C	5.90254600	-0.88716300	0.42398800
F	7.19236600	-0.94262800	0.74832200
C	4.98896400	-0.27164100	1.26574300
F	5.40045500	0.28013600	2.40840800
C	3.65097300	-0.23529500	0.90085500
F	2.83612000	0.39413200	1.76788800
H	-0.38003300	-1.14027600	0.24175700
C	0.79996200	-1.72647600	0.67922900
C	0.91795000	-3.09113500	0.29713800
C	1.08880200	-4.12397500	1.20650100
C	1.11733400	-3.84750700	2.57178200
C	0.98631900	-2.53046700	3.00342600

C	0.84397000	-1.49983400	2.08274500
H	0.90202500	-3.34822000	-0.75614300
H	1.19457500	-5.14439900	0.85191200
H	1.24310600	-4.65033600	3.29194000
H	1.01121900	-2.30062600	4.06386300
H	0.77066500	-0.49392500	2.46505300

P6_P

P	2.17236200	-0.30429800	0.57529800
C	1.16926600	-0.69314300	2.03986200
H	1.85723500	-0.77930300	2.88491100
H	0.48706200	0.13356800	2.23650100
C	0.36936100	-1.99502100	1.81084900
H	0.77675600	-2.55209400	0.95829700
H	0.54676200	-2.63619900	2.68098400
C	-1.13556600	-1.74423600	1.63997900
H	-1.46483500	-1.15679700	2.50712700
H	-1.61857200	-2.71705800	1.76088200
B	-1.71593500	-1.08592400	0.24433400
C	2.56064000	1.44457800	0.28524600
C	2.36338000	2.46146700	1.24597600
C	1.89411600	2.25079500	2.66671800
H	2.18702200	3.10991500	3.27259800
H	2.31741200	1.36145200	3.13384300
H	0.80132500	2.18289100	2.71492000
C	2.57602000	3.78408200	0.85697100
H	2.41197200	4.56908100	1.59054500
C	2.96455000	4.13328300	-0.43250600
C	3.10235000	5.57463800	-0.83398000
H	3.86067600	5.70202700	-1.60943400
H	3.36472100	6.20282400	0.01980200
H	2.14761900	5.93013600	-1.23378800
C	3.17155300	3.11078600	-1.35511900
H	3.47057700	3.35876100	-2.36999300
C	2.97857000	1.77330600	-1.02609600
C	3.16217600	0.73973900	-2.10865100
H	2.19497300	0.33644900	-2.42933400
H	3.80094800	-0.08796600	-1.78624600
H	3.62436700	1.19598900	-2.98487100
C	3.60515400	-1.41507700	0.52638300
C	4.67642700	-1.21535700	1.42205500
C	4.70269500	-0.08729600	2.42418900
H	5.68532800	-0.02192300	2.89397300
H	3.97439100	-0.24871700	3.22655900
H	4.48839200	0.88041500	1.96241400
C	5.75048100	-2.09935400	1.38950700
H	6.57893000	-1.94210000	2.07568300
C	5.78994000	-3.17471300	0.50414000
C	6.94628300	-4.13777100	0.51254800
H	6.77204300	-4.93561700	1.24167600
H	7.87672900	-3.63601000	0.78756300
H	7.07966900	-4.60519700	-0.46524000
C	4.72225500	-3.34766100	-0.37287600
H	4.74470200	-4.17759800	-1.07427700
C	3.61916000	-2.49283200	-0.39063600
C	2.52742800	-2.77231100	-1.40041500
H	2.38682400	-1.94363100	-2.10128700
H	1.55117900	-2.97086300	-0.94753800
H	2.78984900	-3.65243900	-1.98901300
C	-0.97171900	0.37979300	-0.14381300
C	-0.37012300	0.71905300	-1.35600700
F	-0.01210500	-0.23433000	-2.24453100
C	-0.02369000	2.01265600	-1.73011200
F	0.58243000	2.24347400	-2.90033600
C	-0.23000900	3.05931400	-0.84756300
F	0.13263600	4.30245200	-1.17047100
C	-0.74375100	2.77568500	0.40800200
F	-0.83925100	3.74657800	1.32172400
C	-1.09466000	1.46864000	0.72011000
F	-1.56128300	1.28157100	1.97266400
C	-3.33022900	-0.67476700	0.44739600
C	-4.19315500	-1.34255400	1.31835400
F	-3.76632600	-2.35958000	2.08688500
C	-5.54192600	-1.04140500	1.47581000
F	-6.29061300	-1.72657300	2.34393700
C	-6.11272000	-0.02635700	0.72800800
F	-7.40307300	0.27876000	0.86473500
C	-5.31437300	0.66079200	-0.17056800
F	-5.83795400	1.63810200	-0.91531500
C	-3.97207300	0.32279400	-0.29133700
F	-3.30460300	1.04563500	-1.21794500
H	1.41298400	-0.70871600	-0.52113400
C	-1.66464000	-2.15468800	-0.99790100
C	-1.12996000	-3.44414300	-0.87438000
C	-1.12834900	-4.36106500	-1.92955100
C	-1.68578100	-4.01494600	-3.15289400
C	-2.25224700	-2.74848800	-3.30248400
C	-2.24021300	-1.84897800	-2.24480100

H	-0.71999900	-3.77080200	0.07862500
H	-0.70261600	-5.35067800	-1.78448600
H	-1.69471000	-4.72272700	-3.97642300
H	-2.70825900	-2.46591500	-4.24734700
H	-2.68565900	-0.86730700	-2.39224700

P7_TS

P	-2.08575300	0.00777200	0.10938000
B	1.39377800	-0.22699200	0.08544100
C	-1.29967900	-0.62141900	2.95143700
H	-2.09310100	-0.10667800	3.50053500
C	-0.96402300	-0.00454700	1.57744500
H	-0.68737300	1.03552800	1.72909900
C	0.30209500	-0.81781700	1.15396600
H	-0.09534700	-1.66951900	0.57992700
C	0.76951900	-1.43130400	2.51276100
H	1.85121800	-1.55952500	2.60179600
C	-0.05420000	-2.71747300	2.70083100
H	0.24714600	-3.25106200	3.60813900
H	0.05261600	-3.40510100	1.85583100
C	-1.50461800	-2.15419100	2.84007400
H	-2.14203500	-2.42691800	1.99444200
H	-1.99324800	-2.53407400	3.74123000
C	0.10411300	-0.54717700	3.58588500
H	0.13854000	-1.01131900	4.57707500
H	0.49734600	0.46604900	3.64947600
C	-3.56316100	-1.08929100	0.25755600
C	-4.69517200	-0.74017200	1.02644400
C	-5.80284300	-1.58915900	1.02510100
H	-6.67099500	-1.31107300	1.61784000
C	-5.83075800	-2.77476500	0.29783500
C	-4.70143100	-3.11049100	-0.44316200
H	-4.69512600	-4.03590500	-1.01397400
C	-3.56932300	-2.29766800	-0.47513300
C	-4.76775900	0.49844100	1.87945600
H	-5.69676200	0.50839000	2.45221900
H	-4.72485800	1.41230800	1.28171100
H	-3.94024700	0.52510500	2.59241800
C	-7.02949200	-3.68484000	0.33746700
H	-7.93720000	-3.13426100	0.59376400
H	-6.89248400	-4.46904700	1.08906500
H	-7.18377900	-4.17639100	-0.62599700
C	-2.37821700	-2.76999200	-1.26465200
H	-2.62855000	-3.66425800	-1.83838300
H	-1.54582600	-3.02276500	-0.60149600
H	-2.00919500	-2.00878600	-1.95625400
C	-2.68523300	1.69092200	-0.32261600
C	-3.06214600	1.82605800	-1.68284100
C	-3.42071000	3.07519400	-2.17962900
H	-3.70835900	3.16043400	-3.22492500
C	-3.39885000	4.21776300	-1.38293800
C	-3.01205200	4.07422400	-0.05554300
H	-2.98733400	4.95304100	0.58510400
C	-2.65595200	2.84012500	0.49738000
C	-3.03330500	0.66411800	-2.64704000
H	-3.63344000	-0.18184400	-2.29791900
H	-2.00489400	0.31132800	-2.79300800
H	-3.42034800	0.97197600	-3.62007900
C	-3.80634700	5.55664700	-1.93828900
H	-4.89658100	5.65648800	-1.94401100
H	-3.46129300	5.67790100	-2.96812000
H	-3.40073000	6.37516200	-1.33979500
C	-2.24132400	2.86419600	1.95140200
H	-2.74454400	3.68803800	2.46240200
H	-1.16219800	3.02947800	2.05654100
H	-2.48322700	1.94379200	2.47972800
C	2.46466900	0.96040200	0.48482200
F	3.87241400	0.34424100	-1.32504000
C	3.60679900	1.16255500	-0.29593400
F	5.59132600	2.30725300	-0.87947900
C	4.51865800	2.18841900	-0.09723300
F	5.16203600	4.10349200	1.12565300
C	4.30315200	3.10768000	0.92012200
F	2.91608000	3.86831400	2.66627300
C	3.16928900	2.98025100	1.70283200
F	1.17300400	1.94923000	2.22571400
C	2.28967600	1.93039400	1.46190700
C	2.17997900	-1.59930200	-0.38679000
F	4.05123900	-1.22808500	1.03645400
C	3.39401400	-2.00944300	0.16541200
F	5.16532300	-3.54715200	0.45715700
C	3.99908500	-3.23089800	-0.10500500
F	3.94153200	-5.30990300	-1.22241800
C	3.37783300	-4.13445700	-0.95371100
F	1.51672700	-4.65273100	-2.30707000
C	2.15294500	-3.79670300	-1.50519800
F	0.37426800	-2.33337800	-1.73793600
C	1.59306700	-2.56347200	-1.19827100

H	-0.56793500	-0.00847200	-0.92083900
C	0.94498900	1.27373500	-3.61953300
C	0.62407600	2.59493500	-3.31598400
C	0.28062400	2.94849900	-2.00862000
C	0.26426300	1.97545900	-1.02796700
C	0.56107000	0.60150400	-1.28395300
C	0.90737300	0.30089000	-2.62827100
H	1.22798900	1.00261400	-4.63132400
H	0.64633800	3.35141100	-4.09460200
H	0.02723000	3.97459600	-1.76242900
H	0.02182600	2.27082900	-0.00903200
H	1.15445200	-0.71760400	-2.89877600

P7_P

P	-2.16994000	-0.02740400	-0.19843000
B	1.56350900	0.03910200	0.12918500
C	-1.05754700	0.44299000	-2.87023700
H	-1.82581700	-0.07428900	-3.45329000
C	-0.83371100	-0.13019900	-1.44999500
H	-0.62016800	-1.19214700	-1.52711900
C	0.44095900	0.63973600	-0.95669200
H	0.05448600	1.50811500	-0.40746000
C	0.99279900	1.20669900	-2.30554600
H	2.08088500	1.29679700	-2.32364300
C	0.22971700	2.50890300	-2.59322700
H	0.62257300	3.01403900	-3.48143400
H	0.28544800	3.21241100	-1.75634100
C	-1.21857500	1.98454900	-2.84606600
H	-1.92624900	2.32819600	-2.08816800
H	-1.60299400	2.32669400	-3.81079400
C	0.38508000	0.30063400	-3.39202900
H	0.50223300	0.72209200	-4.39556500
H	0.74905100	-0.72506700	-3.38497300
C	-3.50940900	1.18305800	-0.47548800
C	-4.60884200	0.86613600	-1.29824000
C	-5.64118500	1.79473900	-1.42695700
H	-6.48459500	1.54954300	-2.06723600
C	-5.61889200	3.02105900	-0.77067800
C	-4.51003400	3.32273300	0.01782900
H	-4.46119300	4.28708200	0.51695800
C	-3.44758000	2.43658900	0.17908300
C	-4.71560500	-0.42339100	-2.07008200
H	-5.54769800	-0.37249900	-2.77399500
H	-4.87860500	-1.28166500	-1.41224000
H	-3.80983400	-0.61116400	-2.65245000
C	-6.75866300	3.99525900	-0.89875200
H	-6.39256300	5.02235000	-0.96721100
H	-7.41214900	3.93654900	-0.02251600
H	-7.36439300	3.78412700	-1.78215000
C	-2.26889400	2.88537000	1.00569200
H	-2.42552900	3.90574500	1.35788100
H	-1.34175600	2.87659700	0.42622600
H	-2.10118100	2.25760500	1.88674800
C	-2.89004400	-1.60769000	0.34987200
C	-3.41319800	-1.60537900	1.66578500
C	-3.96277000	-2.77796000	2.17140300
H	-4.35975400	-2.77407500	3.18349300
C	-3.99656000	-3.95781000	1.42923500
C	-3.45349600	-3.94035400	0.14972700
H	-3.45876700	-4.85354300	-0.44000600
C	-2.89241200	-2.79059600	-0.41408600
C	-3.32118700	-0.41104600	2.58334700
H	-3.72373100	0.50170200	2.13344600
H	-2.27451000	-0.23623400	2.86763900
H	-3.88047400	-0.60115100	3.50039200
C	-4.60755400	-5.20734400	2.00424000
H	-4.21718200	-5.40642000	3.00573000
H	-4.40159300	-6.07617800	1.37652700
H	-5.69327600	-5.10118700	2.09106600
C	-2.30103500	-2.94258700	-1.79704300
H	-2.79457700	-3.76525800	-2.31815700
H	-1.23332900	-3.18448600	-1.74158600
H	-2.40195000	-2.05223200	-2.41704700
C	2.63115400	-1.11946100	-0.39399600
F	4.23295300	-0.41842900	1.21202400
C	3.86380000	-1.27468100	0.24811700
F	5.93523500	-2.35426400	0.61997200
C	4.76937000	-2.28909000	-0.02469800
F	5.30257200	-4.24511900	-1.24035100
C	4.45052300	-3.25793800	-0.96611900
F	2.86696000	-4.12459200	-2.48283100
C	3.22260400	-3.18820800	-1.59803000
F	1.15002100	-2.22676600	-1.90948800
C	2.35692000	-2.14502300	-1.28732300
C	2.32546400	1.47288500	0.49795100
F	4.20225700	1.15763600	-0.93038000
C	3.51433500	1.92740000	-0.07069300
F	5.20566900	3.54390200	-0.42703000

C	4.05745800	3.18793400	0.15100800
F	3.88972300	5.30612000	1.18367300
C	3.38957600	4.09080000	0.96356400
F	1.49174400	4.57520900	2.27840200
C	2.17908800	3.71425800	1.52202200
F	0.46140900	2.19200600	1.79527600
C	1.68456600	2.44107800	1.26909500
H	-1.51399100	0.44364000	0.93995400
C	0.53412100	-1.04069600	3.83536900
C	-0.22393500	-2.18589300	3.59737700
C	-0.39702700	-2.61581000	2.28503300
C	0.16484600	-1.88872900	1.23767200
C	0.89402300	-0.70264900	1.43531400
C	1.08050000	-0.32100400	2.77388200
H	0.70901100	-0.70887700	4.85497200
H	-0.65331300	-2.74555600	4.42306800
H	-0.97061800	-3.51483500	2.07018100
H	0.05464600	-2.27943900	0.22614800
H	1.68563300	0.55160300	3.00027200

P8_TS

C	1.34731500	-0.40466600	-0.89853000
H	1.27211000	-1.40394700	-1.34243500
H	1.37118900	0.27989400	-1.74314800
P	2.93874700	-0.37125000	0.04198700
B	0.01006000	-0.19413100	0.07449900
C	4.00223900	-1.81017500	-0.58212900
C	3.17231300	-3.09739900	-0.45114200
H	2.28159500	-3.10120500	-1.08466900
H	3.79453600	-3.94416100	-0.76156900
H	2.87028900	-3.27078400	0.58655100
C	5.22688400	-1.99615400	0.32844700
H	5.71736700	-2.94251600	0.07241400
H	5.96752400	-1.20589100	0.21441300
H	4.93334200	-2.04461000	1.38181200
C	4.44057400	-1.64190100	-2.03941700
H	5.16210600	-0.82867900	-2.15267000
H	4.92690100	-2.56249600	-2.38508900
H	3.58751900	-1.44326800	-2.69648700
C	3.78363000	1.29008800	-0.26642300
C	3.02923000	2.28272100	0.63371600
H	1.95720700	2.28536600	0.43410200
H	3.18073600	2.04353200	1.69103200
H	3.40638300	3.29592100	0.45288700
C	5.24372900	1.25777600	0.20065000
H	5.35093100	0.81589200	1.19634100
H	5.88420500	0.71451500	-0.49886800
H	5.61522700	2.28788000	0.24879700
C	3.74144500	1.76926100	-1.72212200
H	4.25962900	2.73392700	-1.78756400
H	4.24707700	1.07693400	-2.39832000
H	2.72348100	1.92135500	-2.07617200
C	-0.56417000	1.34414800	0.25506600
C	-0.26085100	2.42329200	-0.57569300
C	-1.62155300	1.61440700	1.13111800
C	-0.87133100	3.67105000	-0.50196100
C	-2.25604300	2.84332900	1.25099200
C	-1.87749400	3.88891300	0.42344800
F	0.68078200	2.32900500	-1.53058300
F	-0.49861100	4.65150800	-1.32377000
F	-3.24024000	3.01390600	2.13292900
F	-2.11168900	0.64443000	1.91580700
F	-2.47492100	5.07291000	0.50909300
C	-1.29023900	-1.01104600	-0.53154100
C	-2.26163900	-1.72492200	0.17162600
C	-1.55537000	-0.88867500	-1.89749600
C	-3.36706200	-2.31726000	-0.43246700
C	-2.64546400	-1.45920600	-2.53944300
C	-3.56210800	-2.18827100	-1.79718900
F	-0.74034400	-0.15587100	-2.67600600
F	-2.82255400	-1.30376200	-3.85152500
F	-4.61775500	-2.74616200	-2.38480800
F	-4.24690000	-3.00182400	0.29954800
F	-2.19683100	-1.89039400	1.50051300
H	1.62045600	-0.59859200	1.36959500
C	0.15715700	-0.29256900	2.89144300
C	-0.17648700	-1.02068200	4.02347500
C	-0.19388500	-2.41263500	3.96200200
C	0.12498900	-3.08201200	2.77548900
C	0.44269900	-2.34478700	1.65373900
C	0.46264100	-0.91983500	1.65837100
H	0.21591300	0.79224400	2.94861200
H	-0.41355000	-0.51368300	4.95249300
H	-0.45086200	-2.98534100	4.84826700
H	0.10525400	-4.16598200	2.73928800
H	0.67659100	-2.85734000	0.72312400

P8_P

C	1.12348200	-0.42777200	-0.86966500
H	1.05626300	-1.48614700	-1.13675100
H	1.01479100	0.13425300	-1.79771200
P	2.83124100	-0.22074100	-0.31573900
B	-0.15516400	-0.17945100	0.24121900
C	3.93324200	-1.63473200	-0.83999300
C	3.13592300	-2.94393200	-0.71169800
H	2.35375400	-3.03046300	-1.46904300
H	3.83006100	-3.77786800	-0.85817400
H	2.68268000	-3.04955300	0.27844700
C	5.12720400	-1.72135200	0.12383200
H	5.73844000	-2.58537900	-0.15657300
H	5.76853800	-0.83991900	0.09282500
H	4.78788600	-1.87026700	1.15425700
C	4.40094400	-1.46827200	-2.28930600
H	5.08265600	-0.62239000	-2.40867500
H	4.93851900	-2.37274600	-2.59269300
H	3.55679700	-1.33779700	-2.97451900
C	3.48060600	1.47243300	-0.71930900
C	2.66174500	2.43243100	0.16098600
H	1.58972400	2.33599300	-0.02178900
H	2.84938800	2.25857100	1.22476200
H	2.95504100	3.45970700	-0.07980800
C	4.96222500	1.60825400	-0.34804300
H	5.15613000	1.31566300	0.68891700
H	5.61318100	1.02925400	-1.00832500
H	5.24295000	2.66160500	-0.45217300
C	3.27307500	1.82605300	-2.19825300
H	3.67180900	2.83250500	-2.36447800
H	3.80179200	1.14678600	-2.87096900
H	2.21592500	1.84409900	-2.46489100
C	-0.87470500	1.29991600	0.16769300
C	-0.77545800	2.25760000	-0.83479700
C	-1.83568200	1.59792500	1.13802600
C	-1.51329000	3.43651500	-0.86737400
C	-2.59671400	2.75854600	1.15134400
C	-2.43480500	3.68948300	0.13457600
F	0.09932700	2.11064200	-1.85524500
F	-1.34085800	4.31812400	-1.85403000
F	-3.48901500	2.98133100	2.11554000
F	-2.07390200	0.72452300	2.12392100
F	-3.15245600	4.81042800	0.12472100
C	-1.35356800	-1.22242600	-0.19874100
C	-2.12297400	-1.98706500	0.67704600
C	-1.74989800	-1.30242800	-1.53296600
C	-3.16750000	-2.80710500	0.25747400
C	-2.78189800	-2.10512100	-1.99590700
C	-3.49730100	-2.87098300	-1.08649300
F	-1.11992000	-0.55379800	-2.46061600
F	-3.09534500	-2.14387900	-3.29231700
F	-4.49096400	-3.65387200	-1.50229800
F	-3.85652000	-3.53042400	1.14110600
F	-1.89537900	-1.97905900	1.99490900
H	2.92582700	-0.27108400	1.08329800
C	0.92053700	0.50446300	2.56545700
C	1.76567200	0.24081700	3.64598700
C	2.21120500	-1.05552400	3.88571400
C	1.77418400	-2.08720700	3.05356200
C	0.92974100	-1.80612900	1.98279100
C	0.50031800	-0.50147900	1.68839500
H	0.60206200	1.53010800	2.38773200
H	2.07530500	1.05096000	4.30051400
H	2.87149300	-1.26574900	4.72129000
H	2.08558500	-3.11044300	3.24718800
H	0.59802800	-2.62492200	1.34409100

P9_TS

C	-0.64201200	0.01700700	-1.15374000
H	-0.64136400	-0.90676300	-1.74449800
H	-0.77272500	0.82990800	-1.87562000
B	0.78561800	0.09093800	-0.29030900
C	0.49996800	-0.78882700	1.31600000
C	0.68240100	-0.16725500	2.57231600
C	0.77364000	-2.17995600	1.26614000
C	1.16486900	-0.85435400	3.68022300
C	1.24873500	-2.88066800	2.36211500
C	1.45564700	-2.21233400	3.57154800
C	1.97897300	-0.73115800	-1.03344800
C	3.14753400	-1.11440900	-0.35334000
C	1.94787900	-0.99096400	-2.40900800
C	4.20887700	-1.73773600	-0.99753300
C	3.00821700	-1.61245100	-3.07172900
C	4.14282800	-1.99395200	-2.36730700
P	-2.07941000	-0.12503200	-0.03314900
C	-3.16653500	-1.50820100	-0.70263500
C	-4.47711300	-1.59587500	0.08628400
H	-5.13670600	-0.74874100	-0.11998100
H	-5.01205600	-2.50674600	-0.20621800

H	-4.29737400	-1.64398500	1.16521900
C	-3.46418200	-1.37928200	-2.20123000
H	-4.08947700	-0.51316700	-2.42643300
H	-2.54599400	-1.30166300	-2.78987300
H	-4.00081700	-2.27472600	-2.53795500
C	-2.36906300	-2.80177200	-0.46207600
H	-1.42667700	-2.81229100	-1.01892100
H	-2.14187500	-2.94291100	0.59954400
H	-2.96219400	-3.65800400	-0.80329600
C	-2.99415500	1.50797800	0.11523700
C	-3.82988100	1.48874100	1.40455000
H	-4.30282700	2.46856600	1.53710700
H	-4.62204000	0.73768600	1.38451400
H	-3.19802900	1.29649000	2.27686500
C	-3.88167200	1.83076900	-1.09018900
H	-4.73491600	1.15119300	-1.16688900
H	-4.27904200	2.84710000	-0.98153400
H	-3.31819200	1.79026900	-2.02773500
C	-1.92537000	2.59954900	0.25513500
H	-1.19406800	2.37216200	1.03838900
H	-1.37016400	2.75001700	-0.67206300
H	-2.41812400	3.54378700	0.51385400
H	3.22320500	-0.92304700	0.71646300
H	5.09255200	-2.02289000	-0.43350700
H	4.96919700	-2.48119800	-2.87582800
H	2.94342100	-1.79674500	-4.14054700
H	1.08117000	-0.69171100	-2.99442000
H	0.41764600	0.88330900	2.67330800
H	1.30401400	-0.34122800	4.62642300
H	1.83159900	-2.75635500	4.43318200
H	1.46836500	-3.94048500	2.28224900
H	0.63749800	-2.70212000	0.32154400
H	-0.74455000	-0.54378700	1.04109200
C	1.36078300	1.59526800	-0.03833000
C	2.22118900	1.95779900	1.01052600
C	1.17894500	2.57318200	-1.03170300
C	2.80607900	3.21917300	1.09983800
H	2.46450900	1.23135600	1.78050000
C	1.75699900	3.83756000	-0.96085900
H	0.58111900	2.33863500	-1.90968000
C	2.56694200	4.17544000	0.11900500
H	3.45775200	3.45086700	1.93770100
H	1.57984500	4.55738200	-1.75511400
H	3.01725400	5.16099800	0.18619100

P9-P

C	-0.27019200	-0.20019800	-1.02472000
H	-0.25151400	-1.21626100	-1.43490700
H	-0.19558000	0.48170600	-1.88158100
B	1.07349400	-0.09086100	0.05536600
C	0.47134800	-0.47661500	1.52545300
C	0.03523400	0.47591800	2.46193800
C	0.11922700	-1.81013300	1.80019200
C	-0.73724400	0.13455500	3.57423900
C	-0.65269000	-2.16891900	2.90491000
C	-1.09739700	-1.19220100	3.79486000
C	2.16735800	-1.18566900	-0.47068400
C	3.05507800	-1.80623100	0.42192100
C	2.34751200	-1.48298300	-1.82936800
C	4.04931200	-2.68078400	-0.00867000
C	3.33822600	-2.35416300	-2.27758100
C	4.19392300	-2.96312200	-1.36479500
P	-1.92930300	-0.00562300	-0.36812000
C	-3.06509800	-1.31422100	-1.04546700
C	-4.50212400	-1.11045600	-0.55190600
H	-4.96808700	-0.22008300	-0.98203400
H	-5.10215400	-1.97517500	-0.85457800
H	-4.55024300	-1.03956300	0.53972800
C	-3.01975300	-1.35045200	-2.57789800
H	-3.42662000	-0.44228400	-3.02844100
H	-2.00093100	-1.49462300	-2.94780000
H	-3.62338100	-2.19538100	-2.92597000
C	-2.53809900	-2.64855300	-0.48535000
H	-1.51578600	-2.86181000	-0.81010700
H	-2.55422700	-2.66324000	0.60873300
H	-3.18117900	-3.45546000	-0.85243100
C	-2.55596500	1.73916700	-0.48497900
C	-3.57964800	1.98044100	0.63382700
H	-3.90252700	3.02587100	0.59167000
H	-4.46943300	1.35420600	0.54020600
H	-3.13119700	1.80616200	1.61712300
C	-3.16352800	2.03170500	-1.86089000
H	-4.09579200	1.48701400	-2.03145200
H	-3.38861100	3.10168100	-1.92058800
H	-2.46564100	1.79586100	-2.67095200
C	-1.35147600	2.66662900	-0.26103500
H	-0.80315500	2.42657000	0.65392000
H	-0.63722900	2.62581200	-1.08528000

H	-1.72198500	3.69436100	-0.18097800
H	2.95901400	-1.59949000	1.48627100
H	4.71490500	-3.14260300	0.71563700
H	4.96705300	-3.64491100	-1.70622800
H	3.44454800	-2.55539200	-3.34027700
H	1.70426400	-1.01024100	-2.57148200
H	0.29953100	1.52085400	2.31580400
H	-1.05630900	0.90655600	4.26972000
H	-1.69975800	-1.46456400	4.65617500
H	-0.90524200	-3.21266400	3.07471200
H	0.45328100	-2.58747000	1.11420200
H	-1.96857000	-0.27124600	1.00858600
C	1.81417500	1.36056000	-0.01849500
C	2.54483900	1.85057200	1.07579400
C	1.90304600	2.11749800	-1.19717700
C	3.27531100	3.03553900	1.01755000
H	2.55147400	1.27906500	2.00140900
C	2.62719900	3.30473900	-1.27497800
H	1.40650000	1.76559100	-2.10163900
C	3.31184100	3.77788900	-0.15903400
H	3.82261100	3.37613600	1.89237200
H	2.66438000	3.85713100	-2.21014900
H	3.87717900	4.70345800	-0.21035400

P10_TS

P	2.19043500	-0.15957500	0.64992400
B	-1.59991100	-0.17003300	0.74652400
C	2.00629400	0.12245400	2.45322600
H	1.63783600	1.15208200	2.48939700
H	2.97618700	0.12662700	2.95060700
C	1.00301000	-0.81758600	3.15889300
H	1.44648300	-1.07967500	4.12577000
H	0.91685000	-1.76389000	2.61782700
C	-0.39177500	-0.22941500	3.41874600
H	-0.75713200	-0.70315300	4.33849800
H	-0.27630100	0.82609500	3.67103200
C	-1.52110800	-0.41813000	2.37822500
H	-1.87938200	-1.45172000	2.49860600
H	-2.35048100	0.18152000	2.77416800
C	3.39666500	1.12374100	0.09692900
C	4.72632900	1.12760400	0.59326600
C	5.59318300	2.14088100	0.19706700
H	6.61129300	2.13445200	0.58057400
C	5.20322800	3.16040700	-0.67171400
C	3.90888400	3.12624300	-1.16629700
H	3.58226800	3.89646700	-1.86157700
C	2.99881700	2.12458100	-0.81069400
C	5.28099000	0.08413100	1.53482000
H	4.99014300	0.27858600	2.57219900
H	4.95995400	-0.92513900	1.27253300
H	6.37209700	0.10430000	1.50315700
C	6.16750800	4.25123200	-1.05417700
H	7.09228300	3.83142400	-1.46025900
H	5.73656600	4.91865200	-1.80283300
H	6.43780400	4.85196500	-0.18048500
C	1.65826100	2.18487600	-1.50229300
H	1.77418200	1.90307800	-2.55517800
H	0.90577300	1.53366300	-1.05855200
H	1.27374200	3.20744500	-1.48434400
C	2.86125400	-1.81251600	0.17426700
C	3.18618200	-2.88307600	1.03361700
C	3.42683400	-4.14778900	0.48321600
H	3.66403000	-4.96514700	1.16149100
C	3.38916900	-4.39167100	-0.88141400
C	3.15222000	-3.30557400	-1.72224000
H	3.15881600	-3.45558700	-2.79975900
C	2.88631100	-2.03478200	-1.22867900
C	3.36063500	-2.79684900	2.53377800
H	4.24366700	-3.37446500	2.81996600
H	3.50318700	-1.78289000	2.89653200
H	2.50604400	-3.22952400	3.06209800
C	3.60027100	-5.77132800	-1.44499900
H	4.00603100	-6.45209200	-0.69391500
H	2.65230400	-6.18989900	-1.79955300
H	4.28548400	-5.74964600	-2.29656500
C	2.67444800	-0.93085100	-2.23516200
H	2.50411900	-1.35632200	-3.22650800
H	1.80573900	-0.31509400	-1.99413700
H	3.54540100	-0.26874200	-2.28446600
C	-1.40258100	1.37126300	0.16545600
C	-0.80676000	2.42481900	0.85201200
F	-0.21590000	2.22287800	2.04372300
C	-0.75373500	3.73813200	0.40011400
F	-0.15251000	4.68206900	1.12578500
C	-1.31758900	4.06014200	-0.82281300
F	-1.26123900	5.30555700	-1.28935700
C	-1.91816700	3.05499100	-1.56454300
F	-2.46791200	3.33975900	-2.74490200

C	-1.95727500	1.76147200	-1.05777000
C	-3.19267600	-0.54562600	0.41813900
C	-4.17062000	0.34295300	0.87435000
F	-3.81258900	1.47068200	1.51737800
C	-5.53722700	0.16164600	0.71797500
F	-6.40268700	1.06500700	1.18220400
C	-6.00129800	-0.97338600	0.07134000
F	-7.30814400	-1.17480000	-0.09107500
C	-5.08003700	-1.89316600	-0.39734100
F	-5.50146200	-2.99674000	-1.01993100
C	-3.71756800	-1.66714200	-0.21810400
F	-2.92947100	-2.63407400	-0.71818100
F	-2.59046700	0.87095500	-1.84131200
H	0.49115900	-0.52739500	0.17483700
C	-0.59032400	-1.27011300	-0.15367300
C	-0.38618300	-2.56858900	0.39376300
C	-0.18023400	-3.68973800	-0.39207200
C	-0.18287300	-3.56665200	-1.78097900
C	-0.37127600	-2.31799200	-2.37168400
C	-0.56207700	-1.20037200	-1.57309800
H	-0.42041600	-2.69226300	1.47150300
H	-0.02458700	-4.66003700	0.06877900
H	-0.02965200	-4.44373700	-2.40377700
H	-0.36752800	-2.21984300	-3.45283800
H	-0.68416900	-0.23327600	-2.05201700

P10_P

P	2.62998900	-0.29752500	0.54777600
B	-2.17848400	-0.23076200	0.42839200
C	1.91057500	-0.00174600	2.21094900
H	1.70158200	1.07153000	2.16286200
H	2.68959500	-0.14178000	2.95979300
C	0.61966400	-0.79447800	2.51608500
H	0.80271100	-1.41736400	3.39646300
H	0.41660900	-1.48547100	1.69715500
C	-0.64443100	0.06046200	2.77099500
H	-0.82448000	0.07201300	3.85278600
H	-0.46126500	1.10423600	2.50155900
C	-1.90462200	-0.46059700	2.05805900
H	-1.98577000	-1.53943600	2.26724300
H	-2.75295300	-0.01821000	2.58516900
C	3.60804200	1.18703200	0.16052700
C	4.75361900	1.44677800	0.95244500
C	5.46009100	2.62484200	0.74573600
H	6.34480000	2.81698400	1.34838200
C	5.06050500	3.56985900	-0.20038200
C	3.93970200	3.28849400	-0.96846500
H	3.61659000	4.00369800	-1.72119200
C	3.20125800	2.10950700	-0.82517800
C	5.24715300	0.51491700	2.03585400
H	4.71158300	0.68337000	2.97613500
H	5.15051000	-0.53959800	1.76574700
H	6.30390600	0.70355300	2.23331800
C	5.82090100	4.85803100	-0.36358800
H	5.55245300	5.36333300	-1.29298100
H	5.60024800	5.53870100	0.46461400
H	6.89940300	4.68042800	-0.36435500
C	2.06978100	1.92771100	-1.80868700
H	2.48541700	1.73192800	-2.80361400
H	1.37506600	1.12718000	-1.57168600
H	1.49318600	2.85320800	-1.86512300
C	3.57287100	-1.82989800	0.22742100
C	3.80310400	-2.83735900	1.18771300
C	4.47725900	-3.99318700	0.79307500
H	4.64926200	-4.76883800	1.53468200
C	4.92502300	-4.18962200	-0.50908900
C	4.70014300	-3.17485900	-1.43473400
H	5.05023700	-3.29954700	-2.45635500
C	4.03646800	-1.99745300	-1.09951200
C	3.37032600	-2.75211100	2.62939700
H	3.64931900	-3.66715600	3.15333800
H	3.85055700	-1.91979300	3.15316600
H	2.28940700	-2.63809400	2.71931700
C	5.60713000	-5.46852100	-0.91265600
H	4.88000800	-6.17139700	-1.33169600
H	6.36818700	-5.28800100	-1.67513200
H	6.08163800	-5.95260000	-0.05661900
C	3.86306400	-0.95971500	-2.18039300
H	4.10782700	-1.39436000	-3.15136600
H	2.83196500	-0.60226700	-2.23961900
H	4.51858300	-0.09862700	-2.01774900
C	-2.11086300	1.39456500	0.05492700
C	-0.89747900	2.07571900	0.12255300
F	0.24699500	1.40421300	0.43163700
C	-0.70622900	3.42535100	-0.12974600
F	0.51611500	3.97289500	-0.04215600
C	-1.79169000	4.19720700	-0.50935400
F	-1.64716200	5.49676300	-0.76688100

C	-3.02607300	3.58099900	-0.62713200
F	-4.08010500	4.29734300	-1.01865300
C	-3.15866800	2.21831400	-0.36468100
C	-3.70615900	-0.84555800	0.18802800
C	-4.78214800	-0.39196700	0.95697900
F	-4.62769800	0.66159500	1.78097800
C	-6.06247500	-0.91971100	0.91383400
F	-7.03576600	-0.42434100	1.68366100
C	-6.33539800	-1.96953600	0.04849500
F	-7.55774400	-2.50012300	-0.00854600
C	-5.31896300	-2.44665500	-0.75741900
F	-5.56147300	-3.44527900	-1.61257000
C	-4.04678600	-1.88107700	-0.67969000
F	-3.15609200	-2.41680500	-1.53051700
F	-4.39020200	1.74245900	-0.57999200
H	1.53310300	-0.36582500	-0.31953200
C	-1.10264000	-1.02867200	-0.52497700
C	-0.63509200	-2.30989700	-0.17895300
C	0.29447500	-3.00882500	-0.94406300
C	0.77530100	-2.45720200	-2.13132300
C	0.29489900	-1.21396900	-2.53504100
C	-0.61917300	-0.51873700	-1.73846700
H	-1.02211100	-2.78202200	0.72221900
H	0.63919300	-3.98816500	-0.62230000
H	1.49989700	-2.99583300	-2.73631300
H	0.63291200	-0.77977800	-3.47410400
H	-0.97317800	0.45157400	-2.08269300

P11_TS

P	1.79527500	-0.57090300	0.07818300
B	-1.42224700	-0.28560800	0.11409600
C	0.81228300	-0.63319700	1.66165300
H	0.78112200	0.38413900	2.04830100
C	1.44493400	-1.55343800	2.71888800
H	2.49088600	-1.28553400	2.90230300
H	1.45789900	-2.58095400	2.32906900
C	0.66122300	-1.50751000	4.03204100
H	0.77375800	-0.50751800	4.47413800
H	1.09245200	-2.21835100	4.74496900
C	-0.82357600	-1.78500900	3.80948800
H	-0.96337900	-2.82534700	3.48542900
H	-1.37173300	-1.66882500	4.75064900
C	-1.38980400	-0.85192300	2.74073700
H	-2.46538600	-1.00469700	2.63914600
H	-1.26233200	0.18572100	3.07657400
C	-0.66790900	-1.03161600	1.38578200
H	-0.66153900	-2.11473500	1.19481800
C	3.18827100	-1.79402300	0.16119200
C	3.04156700	-3.02366700	-0.51510700
C	4.08827500	-3.94325400	-0.52430100
H	3.95086100	-4.88700200	-1.04663500
C	5.29623100	-3.68624900	0.11577000
C	5.42764300	-2.47354600	0.78331700
H	6.35855000	-2.25440800	1.30158100
C	4.40914500	-1.51887200	0.81891100
C	1.76893700	-3.38768800	-1.22876300
H	1.86273400	-4.35637000	-1.72386900
H	0.93845200	-3.45528100	-0.52164100
H	1.49284600	-2.64318800	-1.98260800
C	6.43058800	-4.67502400	0.06746400
H	7.10076400	-4.45172500	-0.76914300
H	7.02473200	-4.64142500	0.98368200
H	6.06136500	-5.69420400	-0.06709700
C	4.71373600	-0.24336300	1.56459400
H	3.84964100	0.13612500	2.10941500
H	5.51363800	-0.42067400	2.28666000
H	5.03817600	0.54823100	0.88203200
C	2.64811800	1.02941500	-0.25966500
C	3.20652300	1.09730400	-1.55816300
C	3.89019600	2.24410300	-1.95590500
H	4.31479600	2.27899800	-2.95644800
C	4.01335500	3.35151500	-1.12472300
C	3.43417200	3.27945800	0.13715700
H	3.50587400	4.14116800	0.79682300
C	2.74944300	2.15123400	0.59389500
C	3.03774500	-0.00846700	-2.57380200
H	3.44185800	0.30455300	-3.53824600
H	3.54747000	-0.93114400	-2.27939400
H	1.97516100	-0.24115700	-2.71501600
C	4.69301700	4.61015500	-1.59183100
H	5.21015200	5.11112600	-0.76990500
H	5.41848300	4.40234200	-2.38155100
H	3.95368000	5.31134500	-1.99264300
C	2.15738100	2.26403800	1.98138800
H	1.06457000	2.33933000	1.94628200
H	2.41165200	1.42383700	2.63255000
H	2.52761100	3.17132200	2.46224300
C	-3.04205300	-0.57113800	0.04214900

C	-3.91312600	0.28661900	-0.62823500
F	-3.45129000	1.40555300	-1.21157600
C	-5.27513700	0.05612300	-0.77356000
F	-6.04578800	0.92917800	-1.42311700
C	-5.83110700	-1.10015400	-0.24907400
F	-7.13351800	-1.34456900	-0.37774800
C	-5.00594700	-2.00680800	0.39729400
F	-5.51654700	-3.13623500	0.89068500
C	-3.64932900	-1.73297600	0.51525000
F	-2.92064300	-2.69810800	1.11234500
C	-1.12392100	1.34068700	0.15739600
C	-1.79320200	2.10662600	1.12097400
F	-2.72224300	1.54140500	1.90719000
C	-1.60483800	3.46768900	1.31496600
F	-2.29018400	4.12133600	2.25184700
C	-0.70967000	4.15811500	0.50838200
F	-0.51309900	5.46314500	0.67825900
C	-0.04352500	3.46425600	-0.48523500
F	0.81361700	4.09703300	-1.28596600
C	-0.27131500	2.09885100	-0.63867800
F	0.40874100	1.54129000	-1.66110800
H	0.28723200	-0.82336400	-0.91857700
C	-1.59768700	-1.12642300	-3.79123800
C	-1.80149500	-2.50288600	-3.73069900
C	-1.60988200	-3.17461400	-2.52430300
C	-1.19968400	-2.47037800	-1.40413000
C	-0.95603200	-1.07070500	-1.41780700
C	-1.18944300	-0.42857400	-2.66228200
H	-1.77271600	-0.59197100	-4.71934200
H	-2.12388600	-3.04690700	-4.61341400
H	-1.79526700	-4.24183400	-2.45654400
H	-1.11865900	-3.01129300	-0.46767100
H	-1.07542500	0.64288200	-2.74458300

P11_P

P	-1.84384700	-0.56579700	-0.24513800
B	1.60912400	-0.50410100	0.15351600
C	-0.57587200	-0.87855800	-1.55614300
H	-0.41673400	0.10446300	-2.00177200
C	-1.22451900	-1.80019400	-2.61110200
H	-2.24119700	-1.47200100	-2.86358300
H	-1.32984200	-2.80626000	-2.18267700
C	-0.36563500	-1.85941900	-3.86894400
H	-0.33051600	-0.86054800	-4.32685000
H	-0.82295400	-2.53326800	-4.60082300
C	1.04446200	-2.30413900	-3.50602800
H	1.01062000	-3.34004500	-3.14131300
H	1.68512000	-2.30308000	-4.39421500
C	1.65332800	-1.41237400	-2.42316400
H	2.65302900	-1.78429900	-2.21632000
H	1.77158400	-0.39221400	-2.80962000
C	0.84096500	-1.35872700	-1.10179300
H	0.73149100	-2.40548900	-0.78257000
C	-3.17136800	-1.82077000	-0.20499700
C	-2.93913000	-2.97203300	0.57752400
C	-3.94775100	-3.92685500	0.69341600
H	-3.75831100	-4.81316300	1.29341000
C	-5.17532700	-3.77930800	0.05589700
C	-5.37584700	-2.64287900	-0.72446400
H	-6.32348300	-2.51974600	-1.24346400
C	-4.40567800	-1.65223600	-0.87071100
C	-1.62950200	-3.21333000	1.27958700
H	-1.57727600	-4.23466100	1.66044000
H	-0.78175600	-3.07148100	0.60658600
H	-1.47278800	-2.53874800	2.12998800
C	-6.26392300	-4.80752800	0.20850200
H	-7.02220500	-4.46002000	0.91740100
H	-6.76559800	-4.99334000	-0.74438000
H	-5.86676500	-5.75386100	0.58048800
C	-4.76073100	-0.46313100	-1.73212000
H	-3.95043000	-0.16934200	-2.40254000
H	-5.62508700	-0.70792200	-2.35208800
H	-5.01433000	0.41181300	-1.12621900
C	-2.58387000	1.09783400	-0.20488900
C	-3.18331300	1.45967200	1.02588300
C	-3.71642800	2.73909300	1.16387200
H	-4.15519800	3.01882200	2.11792700
C	-3.68264600	3.66851700	0.12831900
C	-3.12603600	3.27418600	-1.08434500
H	-3.10355300	3.98093200	-1.91019800
C	-2.57390800	2.00816200	-1.28378800
C	-3.23889500	0.54224500	2.22466800
H	-3.88028400	0.97840000	2.99160100
H	-3.64162900	-0.44450700	1.97684300
H	-2.24681300	0.41016000	2.66684100
C	-4.18979800	5.06973400	0.32492200
H	-4.58800700	5.48326500	-0.60423400
H	-4.96941500	5.10948500	1.08859900

H	-3.36411300	5.70969200	0.65099500
C	-2.00371700	1.75244800	-2.66160500
H	-0.93605400	1.99656500	-2.69980900
H	-2.13004000	0.72539300	-3.00790000
H	-2.50652500	2.40160700	-3.38110400
C	3.21586800	-0.21319700	-0.20980500
C	3.90960600	0.99452400	-0.19981700
F	3.29368400	2.16839700	0.03626200
C	5.28340400	1.11047000	-0.40230300
F	5.86577400	2.31190400	-0.38574700
C	6.04834200	-0.02238400	-0.60833400
F	7.36319800	0.06805000	-0.80580600
C	5.41858100	-1.25831000	-0.60513000
F	6.13262500	-2.36948200	-0.79721000
C	4.05019400	-1.32023100	-0.39574600
F	3.52354700	-2.55974000	-0.40483000
C	0.82201500	0.95189000	0.41724400
C	0.78425300	1.91952500	-0.58609900
F	1.34063000	1.65009000	-1.78385200
C	0.20900700	3.17359800	-0.46527600
F	0.16467500	4.00965100	-1.50668600
C	-0.39237500	3.53191800	0.73195700
F	-0.98887800	4.71750000	0.86182000
C	-0.42261000	2.60769400	1.75920100
F	-1.09241800	2.88080100	2.88288500
C	0.15768800	1.35498600	1.57625200
F	-0.06044700	0.49846900	2.59609700
H	-1.21923900	-0.70881200	0.99631700
C	2.57541800	-1.34266000	3.88022700
C	2.30561400	-2.70554400	4.00873900
C	1.80980800	-3.39592200	2.91173500
C	1.56424400	-2.72370100	1.71160100
C	1.76831400	-1.34830800	1.56163200
C	2.31901300	-0.69465700	2.68017400
H	2.99466400	-0.78973300	4.71606400
H	2.50293100	-3.22096600	4.94377400
H	1.63129500	-4.46613300	2.97519800
H	1.24510700	-3.32120600	0.86259500
H	2.55740200	0.36636400	2.60121400

P12_TS

P	-2.15792400	-0.83961700	0.68297000
C	-1.81994400	-0.87025500	2.48858700
H	-2.70843500	-0.55171300	3.04621600
H	-1.63759000	-1.92247500	2.73827900
C	-0.57874100	-0.03371900	2.84370000
H	-0.75384100	1.02102900	2.60764300
H	-0.46656400	-0.08086700	3.93341100
C	0.72287700	-0.51116200	2.18118400
H	0.85044800	-1.57098800	2.42135300
H	1.53979800	-0.01839900	2.71956000
B	1.03125500	-0.21980400	0.58364000
C	-3.20611900	-2.27238100	0.28898700
C	-2.82679200	-3.07887500	-0.78876600
H	-1.93086500	-2.83804200	-1.35729400
C	-3.59524500	-4.18983000	-1.13082100
H	-3.30021700	-4.81241400	-1.96891900
C	-4.73529500	-4.49900500	-0.39586800
H	-5.33162900	-5.36610700	-0.66109600
C	-5.11666600	-3.69769400	0.68050300
H	-6.00822700	-3.94003100	1.24953100
C	-4.35633400	-2.58545700	1.02178800
H	-4.65836700	-1.95368500	1.85309600
C	-3.17522000	0.63402100	0.36056400
C	-3.20828900	1.71104900	1.25146500
H	-2.72669600	1.64107500	2.22118400
C	-3.84867600	2.89406400	0.89144100
H	-3.85704200	3.73178700	1.58130800
C	-4.46624700	3.00569800	-0.35075700
H	-4.95128500	3.93364800	-0.63490800
C	-4.45816200	1.92484600	-1.23016300
H	-4.93637900	2.00967300	-2.20031400
C	-3.81098700	0.74599500	-0.88191700
H	-3.78264200	-0.08537500	-1.58139800
C	0.30231700	1.17624700	0.08108200
C	0.56165600	2.32329300	0.83544200
F	1.37440000	2.24426400	1.90169000
C	0.02655800	3.57690100	0.57699600
F	0.30732900	4.61348400	1.36703300
C	-0.80663700	3.74746900	-0.51926000
F	-1.34925000	4.93325500	-0.78336500
C	-1.07607200	2.65617800	-1.32460600
F	-1.87510000	2.78953100	-2.38435200
C	-0.53331000	1.41588500	-1.00693000
F	-0.93128100	0.41546000	-1.82250500
C	2.64911100	-0.16970200	0.31150300
C	3.49984200	-1.09978500	0.90948900
F	3.00107100	-2.05628400	1.71049700

C	4.87372100	-1.13359100	0.71526200
F	5.62731700	-2.04864200	1.32640000
C	5.46349100	-0.20509100	-0.12927500
F	6.77934600	-0.21701400	-0.32984600
C	4.66230300	0.72460100	-0.77178000
F	5.21152900	1.61064200	-1.60353000
C	3.28938900	0.71882000	-0.55053700
F	2.59299600	1.62467500	-1.25811300
H	-0.54925500	-1.10057800	-0.05654200
C	0.91901800	-4.06701100	-0.59431000
C	0.59395100	-2.93073600	0.13626100
C	0.60450400	-1.64208600	-0.43614200
C	0.96250500	-1.55708400	-1.80178300
C	1.29376300	-2.68437900	-2.53750700
C	1.26981500	-3.94432500	-1.93598200
H	0.89715900	-5.04309200	-0.12085300
H	0.29668100	-3.04571900	1.17542600
H	1.01723800	-0.58333700	-2.27744500
H	1.58067400	-2.58625500	-3.57955000
H	1.52789000	-4.82660800	-2.51371700

P12_P

P	2.38778000	-0.66739300	-0.68962300
C	1.74206500	-0.91885100	-2.36732600
H	2.56052800	-0.61687300	-3.03388200
H	1.63562300	-2.00518700	-2.46733200
C	0.41787000	-0.21807000	-2.72815800
H	0.49097700	0.85454600	-2.52845600
H	0.34220600	-0.31262400	-3.81840200
C	-0.84519400	-0.79368000	-2.07665000
H	-0.88856300	-1.86218800	-2.31615800
H	-1.67656300	-0.35818100	-2.64018700
B	-1.16770100	-0.55010000	-0.45883600
C	3.70992400	-1.84115400	-0.35750600
C	3.48907800	-2.86780400	0.56512300
H	2.53490700	-2.95050700	1.08078200
C	4.50132500	-3.79294600	0.80817500
H	4.33677800	-4.59058400	1.52448300
C	5.71638100	-3.69153700	0.13758100
H	6.50146200	-4.41550400	0.33006800
C	5.93516200	-2.66188000	-0.77794000
H	6.88693100	-2.58264100	-1.29215200
C	4.93401000	-1.73199300	-1.02659300
H	5.10739600	-0.91800100	-1.72632200
C	3.02937400	0.99999100	-0.46087300
C	2.81561000	2.00344000	-1.40979200
H	2.30796800	1.78700600	-2.34344800
C	3.24332400	3.30069000	-1.14215400
H	3.06273300	4.08543700	-1.86894000
C	3.87333400	3.59341700	0.06378100
H	4.18018900	4.61144100	0.27885500
C	4.10471200	2.58626500	0.99956400
H	4.59012400	2.81678500	1.94128500
C	3.68711400	1.28725200	0.74085500
H	3.84703900	0.50612200	1.47941300
C	-0.49408300	0.90806500	0.02016500
C	-0.79523000	2.04707400	-0.73456800
F	-1.58297700	1.93788800	-1.81664700
C	-0.32374900	3.32410500	-0.46939700
F	-0.62190000	4.34706900	-1.27302400
C	0.47606500	3.53524800	0.64506600
F	0.97952700	4.74218800	0.90352400
C	0.78055000	2.45825700	1.45454900
F	1.57371800	2.62459600	2.51755200
C	0.30952000	1.19073800	1.12169600
F	0.77056600	0.21120400	1.93288500
C	-2.81263700	-0.43202700	-0.27214500
C	-3.65220700	-1.34058300	-0.91622200
F	-3.13295900	-2.30278700	-1.69847600
C	-5.03534900	-1.34412700	-0.79927800
F	-5.77609500	-2.23974000	-1.45675800
C	-5.65121900	-0.40796100	0.01757800
F	-6.97807900	-0.39125300	0.14605500
C	-4.86521400	0.49974200	0.70692900
F	-5.43853100	1.39526900	1.51495400
C	-3.48244100	0.46329200	0.55713700
F	-2.80703000	1.35571300	1.30821500
H	1.40871000	-0.96084700	0.26072400
C	0.13200600	-4.08435800	0.85455300
C	-0.14765600	-2.99280800	0.02528600
C	-0.75090100	-1.81982200	0.49944200
C	-1.08183500	-1.81184100	1.86749500
C	-0.80072400	-2.87945000	2.70954100
C	-0.18507200	-4.02735800	2.20568100
H	0.59027000	-4.97743700	0.43698600
H	0.09725800	-3.08063300	-1.03171700
H	-1.57043100	-0.93046200	2.28016800
H	-1.06852600	-2.82578100	3.76082300

H	0.02514800	-4.86979100	2.85785200

P13_TS

P	2.66853400	0.14252300	-0.67532000
B	-0.95161600	0.02331100	-0.51780000
C	2.93041600	0.91096800	-2.33321000
H	3.05800900	1.97531800	-2.10486900
H	3.88193600	0.54892700	-2.73962400
C	1.76444800	0.75609600	-3.32465900
H	1.95158300	1.46074200	-4.14214200
H	1.75580000	-0.24234500	-3.77521200
C	0.41008100	1.04110400	-2.66480300
H	-0.28162100	1.43298400	-3.41699400
H	0.56533800	1.86616500	-1.96231800
C	-0.23786100	-0.18905000	-1.98082500
H	0.49176100	-1.01466300	-1.93277300
H	-0.99999500	-0.57637400	-2.66468300
C	3.89541000	0.93113900	0.41532800
C	3.45472800	1.75463800	1.45439100
H	2.39165100	1.90465700	1.61330500
C	4.38271300	2.38410000	2.28209500
H	4.03819400	3.02351600	3.08799300
C	5.74457000	2.19154700	2.07653900
H	6.46508400	2.68266000	2.72277900
C	6.18813400	1.36327600	1.04569600
H	7.25083400	1.20865600	0.88973700
C	5.26779600	0.73127600	0.21898700
H	5.61092100	0.06982200	-0.57266100
C	3.23384200	-1.58177500	-0.79156800
C	3.58905800	-2.25872000	0.38273600
H	3.60244200	-1.72765300	1.33109200
C	3.92284400	-3.60778400	0.33821500
H	4.19808800	-4.12416800	1.25215200
C	3.90216900	-4.29433800	-0.87397400
H	4.15973300	-5.34798000	-0.90561000
C	3.55145200	-3.62773400	-2.04427800
H	3.53593600	-4.15808700	-2.99062500
C	3.21665000	-2.27707100	-2.00460600
H	2.94104900	-1.76845600	-2.92293400
C	-1.46739300	1.57681800	-0.39245200
C	-0.65065300	2.64332400	-0.03796300
F	0.65273700	2.42506400	0.27866600
C	-1.04674600	3.97346800	-0.00253000
F	-0.19066400	4.93150400	0.35861300
C	-2.34356200	4.29666300	-0.36601700
F	-2.75645700	5.56168700	-0.34652600
C	-3.20087900	3.28044200	-0.76137500
F	-4.44633200	3.57525600	-1.13158400
C	-2.75119100	1.96576500	-0.77590200
F	-3.64119100	1.05657700	-1.19535300
C	-2.15018000	-1.05874400	-0.20890400
C	-3.09700200	-0.84327500	0.79189600
F	-3.09881600	0.31265700	1.47569400
C	-4.06588700	-1.76572800	1.15835500
F	-4.94959700	-1.48535800	2.11735900
C	-4.10430100	-2.99909200	0.52422700
F	-5.02242200	-3.90287100	0.86151400
C	-3.16651100	-3.28020600	-0.45562600
F	-3.16995200	-4.47176400	-1.05719300
C	-2.21777000	-2.32095100	-0.79114200
F	-1.30540100	-2.71125300	-1.70263700
H	1.07582100	0.07942500	0.08541200
C	0.40920900	-1.72217900	3.36458400
C	0.09419300	-0.36626400	3.32136400
C	-0.05725500	0.27396200	2.09691900
C	0.09027200	-0.41497600	0.87266400
C	0.41557800	-1.79466100	0.96220000
C	0.57442100	-2.43931100	2.17703400
H	0.52309800	-2.22226500	4.32163900
H	-0.04289300	0.18791700	4.24440900
H	-0.31871500	1.32718100	2.08923600
H	0.52521200	-2.36357800	0.03916100
H	0.81890300	-3.49664400	2.20497600

P13_P

P	2.78294700	-0.23179200	-0.47820700
B	-1.13386400	-0.17942300	-0.32256500
C	2.59099700	0.87375600	-1.90243800
H	2.19095200	1.81718000	-1.51735200
H	3.60675600	1.05704600	-2.27330200
C	1.70404400	0.28352900	-3.01373100
H	1.94243400	0.85075500	-3.91970200
H	2.00589000	-0.75273100	-3.21738000
C	0.19078200	0.36171700	-2.76956800
H	-0.26755200	0.22035000	-3.75407400
H	-0.04821400	1.39557000	-2.49437000
C	-0.44516400	-0.66066300	-1.78472400
H	0.26788700	-1.48872300	-1.63408900

H	-1.24996600	-1.13759700	-2.34702000
C	3.64225400	0.56528300	0.87844000
C	3.23187900	0.22867100	2.17105900
H	2.41195700	-0.46880500	2.32702200
C	3.87095200	0.81197200	3.25915600
H	3.54875700	0.56077900	4.26413300
C	4.90568200	1.72189100	3.05592300
H	5.39779200	2.17981100	3.90773900
C	5.30552900	2.06050500	1.76456500
H	6.10131900	2.78129900	1.61158300
C	4.67152900	1.48615000	0.66795900
H	4.96882200	1.76939800	-0.33840000
C	3.66718000	-1.71348300	-0.99985800
C	5.04974600	-1.66435600	-1.20566000
H	5.60331900	-0.75115500	-1.00133700
C	5.71892500	-2.79756900	-1.65047700
H	6.79135900	-2.76446500	-1.80929400
C	5.01223500	-3.97676700	-1.88289300
H	5.53846800	-4.86099900	-2.22747700
C	3.63826400	-4.02880900	-1.66730000
H	3.09213700	-4.94949500	-1.84129300
C	2.95934400	-2.89744700	-1.22338900
H	1.88885400	-2.93577700	-1.04483100
C	-1.20563500	1.46728900	-0.35964000
C	-0.12725100	2.26887100	-0.00812700
F	1.00241700	1.71732500	0.51471100
C	-0.05370500	3.64245600	-0.20267900
F	1.05051100	4.31800100	0.13455300
C	-1.12784100	4.29833700	-0.77956200
F	-1.09116600	5.61495000	-0.98052000
C	-2.23511100	3.55361700	-1.16139600
F	-3.27144800	4.16435300	-1.73593400
C	-2.24477800	2.17841500	-0.96038100
F	-3.32910700	1.53843000	-1.41660300
C	-2.63066000	-0.86929500	-0.12636500
C	-3.64336300	-0.28549500	0.63232600
F	-3.47184000	0.93826300	1.16331900
C	-4.86208500	-0.89078600	0.90623900
F	-5.79111600	-0.25924600	1.62808800
C	-5.10608200	-2.17400200	0.44047700
F	-6.26655000	-2.77898600	0.69703600
C	-4.11824900	-2.82157900	-0.28238300
F	-4.31805200	-4.07011000	-0.71486200
C	-2.91591600	-2.16998500	-0.53141700
F	-1.99284600	-2.90875000	-1.18601700
H	1.54228100	-0.64448700	0.02065200
C	0.54565300	-1.87400500	3.48033000
C	0.03656600	-0.57603100	3.45198100
C	-0.42506200	-0.02737700	2.25740500
C	-0.41499200	-0.74527300	1.04928300
C	0.06936000	-2.06439900	1.12070300
C	0.55886200	-2.62017000	2.30319900
H	0.90977400	-2.30364000	4.40870300
H	-0.00083200	0.01166200	4.36529800
H	-0.83067400	0.98169300	2.26454200
H	0.01768000	-2.68847700	0.22891300
H	0.92537200	-3.64309100	2.31099000

P14_TS

P	2.83409100	-0.76295700	-0.30114300
B	-0.58575600	-0.20885700	-0.41671200
C	2.47138200	0.41291800	-1.67554400
H	2.32316100	1.36613300	-1.16037400
H	3.34849900	0.54478000	-2.32071400
C	1.21059200	0.10605500	-2.51777300
H	1.51944500	-0.44535300	-3.41401400
H	0.84892400	1.07208000	-2.88878400
C	0.02394900	-0.64877000	-1.87610000
H	0.25320300	-1.71689400	-1.88507500
H	-0.79161300	-0.55442500	-2.59951400
C	3.54370900	-2.35276400	-1.04911500
C	4.72964100	-2.13053100	-1.99846000
H	4.95902400	-3.08114200	-2.49365200
H	5.63558400	-1.79848300	-1.49721800
H	4.48045900	-1.40903100	-2.78312200
C	3.90797000	-3.30665500	0.09881500
H	3.03967400	-3.49216400	0.74113600
H	4.72337500	-2.93987400	0.72468700
H	4.22384400	-4.26750600	-0.32316300
C	2.44811100	-3.04430100	-1.87498500
H	2.12526800	-2.43984300	-2.72564900
H	1.57942600	-3.29591200	-1.26687700
H	2.85856900	-3.98093500	-2.26850700
C	4.15328000	0.06924200	0.79908300
C	4.04877600	1.60091000	0.71263100
H	3.06767700	1.98514200	0.97879400
H	4.32182500	1.96698100	-0.28111600
H	4.76355800	2.02504600	1.42654100

C	5.61213900	-0.26403300	0.45708700
H	6.25151100	0.27305300	1.16710000
H	5.88256000	0.07618200	-0.54626900
H	5.85157100	-1.32382900	0.54824200
C	3.85367300	-0.38492300	2.23779000
H	3.93923500	-1.47043400	2.34834500
H	2.84813900	-0.09018400	2.54583700
H	4.57295600	0.07848100	2.92274600
C	-0.27399000	1.38386400	-0.18319600
C	0.52236200	2.01107700	0.75989900
F	1.20144400	1.28442300	1.67901200
C	0.73641600	3.38256400	0.82854400
F	1.54983100	3.88945300	1.75814600
C	0.12175300	4.21336500	-0.09244100
F	0.31256900	5.52923400	-0.05217500
C	-0.68901500	3.64492500	-1.06543400
F	-1.28379000	4.42275200	-1.96796300
C	-0.86239600	2.26782000	-1.09087200
F	-1.63759000	1.79062100	-2.07683700
C	-2.20666700	-0.45258500	-0.29271200
C	-2.89735600	-1.42902400	-1.00580100
F	-2.24673400	-2.24402500	-1.85614400
C	-4.26063900	-1.66369300	-0.87656500
F	-4.85887500	-2.61121900	-1.60058100
C	-5.00048000	-0.91013000	0.02173800
F	-6.30786100	-1.11933100	0.16248700
C	-4.35624500	0.05194900	0.78401300
F	-5.04689100	0.76806100	1.67237900
C	-2.99167700	0.25079900	0.61927000
F	-2.43390400	1.16511100	1.43243600
H	1.15245900	-0.98998200	0.43329300
C	-0.64793700	-3.64185800	1.50408700
C	-0.82889200	-3.26178200	2.83463900
C	-0.65325900	-1.93014300	3.19854500
C	-0.27587900	-0.98840200	2.24890100
C	-0.06508800	-1.32918300	0.89047300
C	-0.28336200	-2.69365500	0.56347100
H	-0.81365400	-4.67135100	1.20298200
H	-1.12122100	-3.99770100	3.57769500
H	-0.81658700	-1.62193700	4.22629600
H	-0.16786100	0.03848300	2.56834700
H	-0.22062100	-3.00480300	-0.47407000

P14_P

P	3.08401000	-0.63742900	-0.45329400
B	-0.79536600	-0.19072600	-0.25720900
C	2.36156000	0.53143900	-1.65152100
H	2.23398400	1.43128800	-1.04685500
H	3.14572000	0.76463800	-2.38273100
C	1.02964800	0.20653300	-2.38102100
H	1.28768600	-0.27767500	-3.33050600
H	0.63917500	1.19379000	-2.65653800
C	-0.10611300	-0.60216500	-1.71669400
H	0.17420800	-1.65858400	-1.71201700
H	-0.90999200	-0.55392800	-2.46226600
C	3.66295700	-2.23754300	-1.20536900
C	4.79805400	-2.03027700	-2.21672200
H	4.98120800	-2.98396300	-2.72178300
H	5.73372100	-1.71612000	-1.75656500
H	4.52146200	-1.30269600	-2.98717100
C	4.06105600	-3.19369200	-0.06848100
H	3.23179300	-3.33088200	0.63478200
H	4.94002100	-2.86752800	0.49010300
H	4.29381400	-4.16984000	-0.50687600
C	2.47158800	-2.87100200	-1.94211900
H	2.11620200	-2.25529400	-2.77144100
H	1.64056200	-3.06917300	-1.26318200
H	2.80804100	-3.82840900	-2.35369300
C	4.37005000	0.23332800	0.61641500
C	4.29465200	1.76187500	0.45344800
H	3.34116900	2.18528500	0.76125100
H	4.51252900	2.07112100	-0.57341200
H	5.06966200	2.19363400	1.09494700
C	5.81325000	-0.16826600	0.27646300
H	6.47125600	0.35511000	0.97731800
H	6.09725500	0.14564400	-0.73183300
H	6.00967200	-1.23477700	0.38541300
C	4.03640600	-0.16311200	2.06531800
H	4.10205800	-1.24571900	2.21781500
H	3.02903300	0.15688500	2.34615000
H	4.75548800	0.31757500	2.73667600
C	-0.67046800	1.45267800	-0.14417100
C	0.39743000	2.11242300	0.44536600
F	1.39544300	1.40593800	1.04704600
C	0.59520300	3.48805200	0.43984900
F	1.68079400	4.01878800	1.01831500
C	-0.32595700	4.29758400	-0.20041300
F	-0.16343200	5.61886800	-0.23532300

C	-1.41524200	3.69998100	-0.82140100
F	-2.30510900	4.46057000	-1.45794800
C	-1.55671800	2.31819400	-0.78929400
F	-2.61776800	1.83047400	-1.44419200
C	-2.37975800	-0.67211000	-0.21564700
C	-2.85974300	-1.80967800	-0.85521200
F	-2.04997200	-2.55749000	-1.63582400
C	-4.16246900	-2.28084000	-0.73329400
F	-4.55251400	-3.38108500	-1.38301900
C	-5.05683700	-1.60656100	0.08085200
F	-6.31146800	-2.03806000	0.21516500
C	-4.62474100	-0.47917600	0.76431400
F	-5.46980500	0.17327100	1.56571200
C	-3.31304600	-0.05354900	0.61340000
F	-2.96059900	1.02693600	1.33431800
H	2.05136200	-1.01966100	0.41694700
C	0.60886400	-3.15172800	1.94822200
C	0.84005200	-2.56222200	3.18975200
C	0.48915900	-1.22696700	3.37497500
C	-0.02732600	-0.48045000	2.31689000
C	-0.18877500	-1.01634800	1.02924200
C	0.09066600	-2.38972500	0.90138000
H	0.81230500	-4.20953800	1.79955500
H	1.24808900	-3.14442300	4.01037300
H	0.61266300	-0.76509700	4.35079300
H	-0.31047500	0.55367300	2.49697300
H	-0.13353500	-2.88422000	-0.04215300

P15_TS

P	1.17736600	-0.65888300	0.21635700
B	-1.50399500	-0.27029400	0.14317100
C	-0.31036900	-0.93649300	-0.81426200
H	-0.36180300	-2.02975800	-0.69443900
C	-0.37672900	-0.65863000	-2.31629700
H	-0.31776700	0.41251100	-2.51481400
H	-1.37723400	-0.97023300	-2.63503500
C	0.66769600	-1.40762400	-3.13810400
H	1.67743400	-1.04508600	-2.92913800
H	0.48853500	-1.27562400	-4.20815100
H	0.63376000	-2.48184800	-2.92431400
C	2.04928000	0.93647000	0.10498200
C	2.12853200	1.75464500	-1.01830000
F	1.69614700	1.32948000	-2.20123100
C	2.66408300	3.03285400	-0.95674100
F	2.71286600	3.79527800	-2.04210700
C	3.15977300	3.51084800	0.25041600
F	3.67680200	4.72604500	0.31596700
C	3.13135800	2.70598000	1.38216000
F	3.61226100	3.15810400	2.53211600
C	2.58500300	1.43529500	1.29414200
F	2.56253400	0.68966400	2.39591400
C	2.46971000	-1.90849200	-0.09751700
C	3.52756000	-1.73242600	-0.98927300
F	3.63324300	-0.61324900	-1.70538500
C	4.49305900	-2.70922600	-1.18574700
F	5.48451100	-2.51479900	-2.04574000
C	4.41244700	-3.90227800	-0.47764600
F	5.32693000	-4.84137600	-0.65956400
C	3.36988400	-4.11312600	0.41542000
F	3.29271700	-5.25241700	1.09110500
C	2.42144000	-3.11637700	0.59590400
F	1.43506700	-3.34323800	1.46516600
C	-1.25662900	1.36336800	0.14925500
C	-1.69321700	2.11163800	-0.94771800
F	-2.41831600	1.52776300	-1.91199000
C	-1.41696400	3.45864100	-1.13982400
F	-1.86221200	4.09477200	-2.21965700
C	-0.66823800	4.14354000	-0.19262100
F	-0.37441500	5.42815000	-0.35874400
C	-0.22168100	3.45902500	0.92456600
F	0.52480400	4.08728200	1.83745400
C	-0.52375200	2.10922600	1.06860500
F	0.02859400	1.52609800	2.15656900
C	-3.01873100	-0.67382000	-0.31282200
C	-4.11641800	0.10535000	0.04939300
F	-3.93472300	1.24612000	0.73432100
C	-5.43209300	-0.22716100	-0.24096000
F	-6.43358900	0.56851400	0.13193200
C	-5.70212000	-1.40917900	-0.91516700
F	-6.95522800	-1.75351100	-1.19765400
C	-4.64923100	-2.23439900	-1.27774300
F	-4.89061200	-3.38354900	-1.90875700
C	-3.34873600	-1.85827100	-0.96418100
F	-2.39011700	-2.73828500	-1.32049700
H	-0.00767900	-0.77851100	1.47469300
C	-1.92746500	-0.28390100	2.81456600
C	-2.44839600	-0.94074000	3.91950100
C	-2.42820300	-2.33305200	3.97627900

C	-1.90284000	-3.06767000	2.91343400
C	-1.40070000	-2.40410000	1.80666600
C	-1.37488200	-0.98648300	1.71456200
H	-1.98239700	0.79757200	2.78246000
H	-2.87654100	-0.37002000	4.73693200
H	-2.82886500	-2.84641000	4.84517400
H	-1.89562600	-4.15187000	2.94878700
H	-1.01228400	-2.99820300	0.98411000

P15_P

P	1.24812500	-0.66651800	0.22878200
B	-1.57715400	-0.36621300	0.32717700
C	-0.32080800	-1.12651000	-0.53286800
H	-0.31388500	-2.18957600	-0.24419100
C	-0.42643000	-1.06366100	-2.06379500
H	-0.55549000	-0.03189600	-2.38985400
H	-1.36636900	-1.57048700	-2.29512600
C	0.70159900	-1.72955900	-2.84744400
H	1.62853900	-1.15053900	-2.80093700
H	0.43257500	-1.81008100	-3.90340000
H	0.90085100	-2.74224300	-2.47868300
C	1.93399000	0.99903000	0.06996400
C	1.81574100	1.77891600	-1.07623400
F	1.27974600	1.26932200	-2.18034100
C	2.24365600	3.09673600	-1.10314100
F	2.08423200	3.83261900	-2.19228400
C	2.81335700	3.65289100	0.03733300
F	3.21452300	4.90957900	0.02307600
C	2.96846100	2.88961600	1.18849400
F	3.51381200	3.42243300	2.26986500
C	2.52744400	1.57541700	1.19393400
F	2.67618200	0.86180700	2.30620400
C	2.62447400	-1.80572100	-0.10990800
C	3.62169600	-1.55785800	-1.05167400
F	3.60973600	-0.43535400	-1.76995600
C	4.63628900	-2.47180000	-1.29356800
F	5.57094400	-2.21619400	-2.19668100
C	4.66513700	-3.66560500	-0.58030500
F	5.62653500	-4.54205400	-0.80677000
C	3.68267800	-3.94369600	0.36267900
F	3.71072000	-5.08300100	1.03638900
C	2.67957200	-3.01209800	0.58533700
F	1.74405800	-3.29239000	1.49031900
C	-1.31877700	1.26281000	0.18690800
C	-1.67000900	1.98819400	-0.95242300
F	-2.31840800	1.38742800	-1.96044600
C	-1.37786500	3.33274100	-1.14738100
F	-1.72433200	3.94990000	-2.27436900
C	-0.71178100	4.03357300	-0.15163500
F	-0.36939400	5.30652000	-0.32825600
C	-0.34926300	3.36907800	1.00835800
F	0.36516600	4.00888200	1.94147400
C	-0.64388500	2.01769900	1.14382100
F	-0.10182000	1.42749900	2.23022100
C	-3.02382700	-0.84038500	-0.30586600
C	-4.15497300	-0.02576400	-0.30644900
F	-4.07628800	1.25419500	0.09615500
C	-5.41812900	-0.45208800	-0.69536000
F	-6.45236400	0.38857600	-0.68087900
C	-5.60568000	-1.76793600	-1.09010800
F	-6.80932300	-2.19646200	-1.46337600
C	-4.52154500	-2.63085000	-1.08274500
F	-4.67805900	-3.90506300	-1.44434100
C	-3.27958200	-2.15568800	-0.68270000
F	-2.28482200	-3.07019700	-0.67950600
H	1.05339100	-0.80005900	1.60597300
C	-2.43534800	-0.09992300	2.78913900
C	-2.64749300	-0.51404900	4.09709100
C	-2.10469700	-1.71764600	4.54795600
C	-1.36957800	-2.49963900	3.66711000
C	-1.17108800	-2.07370500	2.35198400
C	-1.67678800	-0.85659000	1.87940800
H	-2.87773600	0.83672600	2.45335900
H	-3.23928500	0.10045900	4.76913000
H	-2.26491200	-2.04384800	5.57076300
H	-0.95579700	-3.44895300	3.99505200
H	-0.62487100	-2.73866800	1.68635800

P16_TS

C	-0.58050200	-1.29887700	-3.15576900
H	-1.58737200	-1.15675700	-2.76800200
H	-0.48396500	-2.31341500	-3.55683200
H	-0.45001800	-0.61096200	-3.99767500
C	0.48031400	-1.03754500	-2.13698600
H	1.49987300	-1.06627000	-2.52585000
C	0.36303900	-0.75281600	-0.83500200
P	-1.14991400	-0.65740700	0.15434100
C	-2.44841800	-1.88749600	-0.20845800

C	-3.48711900	-1.68675000	-1.11898600
F	-3.53814100	-0.57578900	-1.85552900
C	-4.48450400	-2.62987100	-1.31760300
F	-5.45179000	-2.41408800	-2.19971700
C	-4.46287700	-3.81127500	-0.58532100
F	-5.40849800	-4.71825400	-0.76814700
C	-3.44777000	-4.04211500	0.33351500
F	-3.43085200	-5.16694200	1.03682300
C	-2.46539300	-3.07875100	0.51553600
F	-1.52178300	-3.31922300	1.42426900
C	-1.98531000	0.95233400	0.01889900
C	-2.64241300	1.42673500	1.15410900
F	-2.77890000	0.63236000	2.21450300
C	-3.14763000	2.71520500	1.22730400
F	-3.75659900	3.14532200	2.32351200
C	-2.99028300	3.56525600	0.13908600
F	-3.45177300	4.80344700	0.19707300
C	-2.35314300	3.11676000	-1.01136500
F	-2.18748500	3.93968100	-2.03918400
C	-1.86476000	1.81844000	-1.06564200
F	-1.26173600	1.43450600	-2.18682500
B	1.54866500	-0.27933100	0.20451600
C	3.07111200	-0.63201500	-0.26325900
C	4.15346800	0.21476500	-0.02797000
F	3.97305400	1.40445400	0.56600900
C	5.46580400	-0.09428800	-0.36166300
F	6.45015000	0.76845100	-0.11234700
C	5.75105000	-1.31630200	-0.95112300
F	7.00051800	-1.63448100	-1.27697300
C	4.71586700	-2.20722400	-1.18642400
F	4.97127300	-3.39350500	-1.73888600
C	3.41895300	-1.85557900	-0.83292700
F	2.48396200	-2.79624200	-1.06239300
C	1.26007500	1.34549300	0.33129100
C	0.53150200	2.02759500	1.29950900
F	0.01725700	1.38622500	2.37186500
C	0.16806300	3.36766500	1.20286200
F	-0.58830500	3.93236300	2.14891000
C	0.51663100	4.09192300	0.07712800
F	0.12012200	5.35307600	-0.06103700
C	1.23827100	3.46281200	-0.92993500
F	1.56821400	4.13619200	-2.02906400
C	1.58397700	2.12842600	-0.78172400
F	2.24727900	1.57700100	-1.80573100
H	-0.01646200	-0.85626600	1.44365500
C	2.29382400	-1.41197800	3.91039300
C	1.87582700	-0.59873900	2.86870900
C	1.33751800	-1.13742000	1.67316000
C	1.26045600	-2.55255800	1.59695300
C	1.66128200	-3.37187200	2.63947200
C	2.18024000	-2.79841600	3.79996500
H	2.71076900	-0.97239300	4.81038900
H	1.98920000	0.47586600	2.96295900
H	0.86200700	-2.99885500	0.68934100
H	1.57627400	-4.44994900	2.55236200
H	2.49846900	-3.43335400	4.62136800

P16-P

C	-0.51892100	-1.46171100	-3.00760800
H	-1.52635400	-1.16341600	-2.72297300
H	-0.51837700	-2.52210100	-3.28253100
H	-0.25955000	-0.89999600	-3.91017300
C	0.51276700	-1.19781600	-1.95839400
H	1.54101200	-1.27924500	-2.31283100
C	0.39419700	-0.85319100	-0.66728300
P	-1.20193900	-0.65337800	0.11334800
C	-2.53630400	-1.83439000	-0.24316100
C	-3.52372200	-1.60120300	-1.20079500
F	-3.49216800	-0.49295300	-1.94226100
C	-4.54520700	-2.50861100	-1.43324200
F	-5.46538600	-2.26613300	-2.35481300
C	-4.59908900	-3.67981500	-0.68420800
F	-5.56805500	-4.55080600	-0.89763000
C	-3.63583500	-3.93786600	0.28311900
F	-3.69425200	-5.05068300	0.99771000
C	-2.62247400	-3.01376600	0.49618100
F	-1.72457900	-3.27447700	1.43890800
C	-1.93241900	0.99609700	0.00386600
C	-2.56911000	1.51905100	1.12936100
F	-2.76128500	0.74672700	2.19569600
C	-3.00222700	2.83460500	1.17098800
F	-3.59233300	3.31970700	2.25128700
C	-2.78789200	3.65277600	0.06669400
F	-3.17120000	4.91476500	0.10178000
C	-2.16944200	3.15071500	-1.07312500
F	-1.94303800	3.94416100	-2.10888100
C	-1.75121000	1.82865900	-1.09756500
F	-1.15382100	1.37619300	-2.19476400

B	1.61974500	-0.33148000	0.34443000
C	3.08539300	-0.74948400	-0.26565600
C	4.20011700	0.08594800	-0.25943000
F	4.09785500	1.36244000	0.14483900
C	5.47190700	-0.31992900	-0.64446300
F	6.49376600	0.53600600	-0.62084000
C	5.68009600	-1.62995200	-1.04732200
F	6.89166300	-2.03754600	-1.41921400
C	4.60963700	-2.51045400	-1.05150300
F	4.78972700	-3.77883500	-1.42383500
C	3.35771900	-2.06048200	-0.65264800
F	2.37790100	-2.98340600	-0.65540300
C	1.34332800	1.30230100	0.33676600
C	0.63167200	2.00618300	1.30304100
F	0.10560300	1.37875700	2.37491000
C	0.28021200	3.34731300	1.18366800
F	-0.46920100	3.94079800	2.12022100
C	0.60874200	4.04075500	0.03207200
F	0.19332100	5.29435800	-0.13367700
C	1.29431900	3.38228500	-0.98117700
F	1.58481000	4.02426200	-2.11077900
C	1.63470200	2.04821800	-0.80817600
F	2.24582100	1.46031400	-1.84435000
H	-0.98603200	-0.82171300	1.48301100
C	2.27124200	-1.03211900	4.15537600
C	2.20133400	-0.40755500	2.91592300
C	1.58803800	-1.02083100	1.81332900
C	1.07974300	-2.31084300	2.00963600
C	1.13079700	-2.94526500	3.25071300
C	1.72509500	-2.30367900	4.33128500
H	2.75089100	-0.52829000	4.98931100
H	2.63582700	0.58363700	2.79607400
H	0.65503000	-2.84644900	1.16257400
H	0.71542600	-3.94237400	3.36701700
H	1.77289700	-2.79048800	5.30043900

P19_TS

B	0.65031400	-0.41872800	0.21393100
C	0.03498500	-1.05262100	1.60921000
C	0.80280500	-2.05206700	2.23202200
H	1.75723400	-2.32198900	1.78878900
C	0.41545700	-2.73427300	3.38123300
H	1.06428700	-3.49603300	3.80209500
C	-0.79348200	-2.42115700	3.98369300
H	-1.11634800	-2.92410600	4.88965000
C	-1.58770400	-1.43979000	3.40422100
H	-2.53168800	-1.17684200	3.87639000
C	-1.20866700	-0.76481400	2.23726000
C	-2.21798600	0.24770000	1.72306500
H	-1.78326600	1.24509200	1.63839100
H	-3.06179700	0.33061900	2.41527700
C	-4.04498900	-1.57615400	0.20261100
C	-4.89034500	-1.57985400	-1.08696900
H	-4.23825100	-1.84041500	-1.93300600
H	-5.62002700	-2.39555900	-0.99650700
C	-5.60762500	-0.26872100	-1.41286600
H	-6.26150900	0.02584600	-0.58326600
H	-6.26575900	-0.43288100	-2.27212400
C	-4.63143300	0.85948400	-1.75251700
H	-5.18223600	1.74065600	-2.10754500
H	-3.99633500	0.53244700	-2.58836300
C	-3.72677500	1.31831400	-0.59269600
C	-2.68364100	2.30779800	-1.13165600
H	-2.09095300	1.86659600	-1.93876800
H	-1.99411800	2.63952700	-0.34780900
H	-3.19319700	3.19378900	-1.52786400
C	-4.54712400	2.02610800	0.49396800
H	-3.90833300	2.38856600	1.30440000
H	-5.32053100	1.39179700	0.93089100
H	-5.04405000	2.89807900	0.05216600
C	-4.93122400	-1.47931800	1.44866500
H	-4.33203300	-1.57528600	2.35880400
H	-5.65024400	-2.30709000	1.43994100
H	-5.49784200	-0.54770100	1.50960500
C	-3.25580800	-2.89386300	0.26962300
H	-2.65398900	-3.04457500	-0.63178800
H	-3.96059900	-3.73004200	0.35210200
H	-2.58911300	-2.92453300	1.13774800
C	2.27700300	-0.67723400	0.07732100
C	2.99642800	-1.21242800	-0.98797000
F	2.39438200	-1.68205900	-2.08918400
C	4.38719700	-1.29849500	-1.00914200
F	5.00940400	-1.83328100	-2.06114900
C	5.12664300	-0.81905100	0.05677200
F	6.45524400	-0.89628100	0.04848500
C	4.46133700	-0.25063800	1.13417900
F	5.15501700	0.22870100	2.16662400
C	3.07699600	-0.18138600	1.11238300

F	2.49895000	0.40798600	2.16938500
C	0.55838400	1.21295200	0.01655300
C	0.38062900	2.12714000	1.05951000
F	0.16975400	1.72107800	2.31801100
C	0.41896400	3.50927000	0.89718200
F	0.21331300	4.31614500	1.93741300
C	0.70555700	4.04808500	-0.34686500
F	0.75517800	5.36526800	-0.51933200
C	0.98581800	3.18827900	-1.39805500
F	1.33513400	3.68036500	-2.58617100
C	0.94985300	1.81680100	-1.18301300
F	1.38107700	1.06768200	-2.21084200
H	-1.18785300	-0.83713000	-0.61462000
P	-2.80220700	-0.18531000	0.03932000
C	-0.33103700	-0.92998500	-2.46106800
C	-0.15033300	-1.35301500	-1.12091300
C	0.00892300	-2.74797700	-0.92144100
C	0.05653000	-3.64000200	-1.98125000
C	-0.08748000	-3.17120500	-3.28673500
C	-0.29931800	-1.81324800	-3.52621600
H	-0.57050900	0.11208100	-2.65119800
H	0.11290200	-3.12430600	0.09195800
H	0.20212000	-4.69928500	-1.79637000
H	-0.05561800	-3.86824400	-4.11893800
H	-0.44711900	-1.45402600	-4.53921200

P19_P

B	0.84864900	-0.37263400	0.04949800
C	0.10055700	-0.98575800	1.39331700
C	0.67500700	-2.05348600	2.10246200
H	1.61093300	-2.47150300	1.74510100
C	0.11510200	-2.60436800	3.25162100
H	0.61343900	-3.42838400	3.75311400
C	-1.05194200	-2.06525200	3.77935900
H	-1.47162000	-2.43754700	4.70829100
C	-1.66968900	-1.02835400	3.09147800
H	-2.56728800	-0.57359100	3.50543800
C	-1.14265200	-0.53975600	1.89220800
C	-1.95099100	0.52529200	1.17991900
H	-1.32048400	1.16046300	0.54916400
H	-2.46962200	1.17181900	1.89664100
C	-4.37539400	-1.42945800	0.55729500
C	-5.36314500	-1.59688300	-0.62557900
H	-4.80725700	-1.98751000	-1.48996300
H	-6.06825000	-2.38317700	-0.32865900
C	-6.12934100	-0.34067800	-1.05370000
H	-6.71207700	0.05775900	-0.21508100
H	-6.85863200	-0.62623400	-1.81752500
C	-5.21797000	0.74574100	-1.63431800
H	-5.82037600	1.57932900	-2.01644900
H	-4.67171500	0.33267100	-2.49422300
C	-4.19336700	1.34021100	-0.64545900
C	-3.22830000	2.26657100	-1.40231200
H	-2.69616400	1.73635700	-2.19464700
H	-2.49068100	2.72513500	-0.73618400
H	-3.81099600	3.07446600	-1.85763900
C	-4.87879100	2.15115300	0.46201700
H	-4.15491700	2.56016800	1.17201200
H	-5.62548500	1.58595000	1.02034900
H	-5.39012200	2.99935700	-0.00526100
C	-5.11756900	-1.12328600	1.86242600
H	-4.42314200	-0.96519400	2.68909800
H	-5.73489100	-1.99220900	2.11324500
H	-5.77955200	-0.25928900	1.79344500
C	-3.57533700	-2.73435900	0.71371900
H	-3.01122100	-2.96864700	-0.19257300
H	-4.28955400	-3.54585900	0.89284800
H	-2.87789600	-2.69536000	1.55329300
C	2.37654900	-0.98962600	-0.07441600
C	2.91789800	-1.66150700	-1.16563100
F	2.18403400	-1.93273400	-2.25275800
C	4.24295200	-2.08596400	-1.22383300
F	4.70164700	-2.72955900	-2.29983000
C	5.09544500	-1.82849900	-0.16351900
F	6.36672600	-2.22597700	-0.20650800
C	4.61012800	-1.14733300	0.94393400
F	5.42154700	-0.88411800	1.97085600
C	3.28377100	-0.74100200	0.95708000
F	2.87763900	-0.07256400	2.04932100
C	1.13592000	1.26644600	0.09089900
C	0.91820100	2.14859900	1.14720200
F	0.32365200	1.74439100	2.28277900
C	1.26299100	3.49762600	1.11952800
F	0.99290500	4.28641500	2.16254700
C	1.89532900	4.02157900	0.00531400
F	2.22668500	5.31066600	-0.04334900
C	2.17211800	3.18257900	-1.06465600
F	2.79011800	3.66556700	-2.14342500

C	1.80676500	1.84563900	-0.99092200
F	2.13760200	1.09413200	-2.05344400
H	-2.61625600	-0.64502300	-1.10748700
P	-3.22587400	-0.08776500	0.01824500
C	-0.53062000	0.07574500	-2.24247500
C	-0.09001100	-0.79095100	-1.23287100
C	-0.54714300	-2.11949300	-1.32105100
C	-1.38892500	-2.55715300	-2.33971300
C	-1.82584100	-1.66305800	-3.32019900
C	-1.38083100	-0.34482100	-3.27019600
H	-0.21624600	1.11608200	-2.23407600
H	-0.22895900	-2.83058600	-0.56013700
H	-1.70421100	-3.59708700	-2.37420600
H	-2.47980800	-1.99627600	-4.12045100
H	-1.69034900	0.36077600	-4.03788500

P20_TS

P	-2.29623800	-0.48059200	0.05893300
C	-2.43024800	-0.91767900	1.83342500
C	-3.43693200	-1.57275800	2.55117500
C	-3.15784100	-2.08845700	3.82566300
C	-1.87912500	-2.05263600	4.39391000
C	-0.84231000	-1.44187700	3.68413600
C	-1.20797200	-0.85153500	2.46965400
O	-0.16544000	-0.31698600	1.81592400
C	0.95586700	-0.72244000	2.48962300
C	0.62458200	-1.34316400	3.70111000
C	1.69764100	-1.82199300	4.45862300
C	2.97757000	-1.68721100	3.92094600
C	3.20729100	-1.16323000	2.63586400
C	2.16790600	-0.68053900	1.83454100
B	2.07641400	-0.21215300	0.26348500
C	3.50802100	-0.45451800	-0.50916100
C	4.15046800	0.48145200	-1.31851800
F	3.62237100	1.69617100	-1.53577700
C	5.35116100	0.23807600	-1.97849200
F	5.91030700	1.18181200	-2.73715600
C	5.96334200	-0.99825300	-1.85639600
F	7.11086300	-1.25025700	-2.48310000
C	5.35806500	-1.97399200	-1.07931000
C	4.16018200	-1.68726700	-0.43897600
F	3.62931900	-2.69676400	0.27212900
F	5.92492500	-3.17625100	-0.96137400
C	1.64206700	1.37009000	0.25459200
C	0.67881900	1.97941900	-0.53620700
F	-0.00860200	1.26719600	-1.45581700
C	0.36874000	3.33370600	-0.48931900
F	-0.54208700	3.85383000	-1.31474800
C	1.03381600	4.15198200	0.40749700
F	0.74632900	5.45133500	0.48012400
C	2.02178900	3.60293000	1.21174000
C	2.31193000	2.24968900	1.10699900
F	3.32436700	1.80791400	1.86597000
F	2.69247700	4.38152400	2.06170500
C	-2.62479900	1.29027800	-0.30816900
C	-2.86343800	1.56750500	-1.67154600
C	-2.64963900	0.55043600	-2.76981400
C	-3.29758000	2.83978400	-2.03661000
C	-3.45778000	3.85782100	-1.10202900
C	-3.86211500	5.24364900	-1.52533200
C	-3.13241500	3.58351500	0.22340800
C	-2.70178200	2.32556700	0.64443700
C	-2.33145900	2.16389700	2.09769900
C	-3.78981500	-1.33391000	-0.61098300
C	-3.60620200	-2.54426600	-1.30424300
C	-2.23988000	-3.15293700	-1.44423600
C	-4.70832000	-3.19956400	-1.84750300
C	-5.99840100	-2.69253600	-1.71148300
C	-7.17612400	-3.39302000	-2.33586600
C	-6.16517600	-1.51696000	-0.98524400
C	-5.09144700	-0.81951100	-0.42572600
C	-5.41534800	0.43219700	0.35817200
H	-4.41932400	-1.71743300	2.11127800
H	-3.95596600	-2.58293300	4.36904600
H	-1.69912400	-2.53284300	5.35025500
H	1.54626700	-2.30885900	5.41630700
H	3.82550900	-2.04779300	4.49458500
H	4.22347400	-1.16582200	2.25015700
H	-2.44183500	1.06776400	-3.70882600
H	-1.79033100	-0.09733400	-2.56078900
H	-3.52635600	-0.08758400	-2.91942300
H	-3.48655600	3.04581100	-3.08789300
H	-4.40954400	5.75902000	-0.73273100
H	-2.96963100	5.83634600	-1.75260400
H	-4.48577200	5.22212600	-2.42195700
H	-3.19983100	4.38089500	0.95992400
H	-2.36566900	3.13510000	2.59492100
H	-1.31640800	1.76999900	2.20201800

H	-3.00986900	1.49053400	2.63026500
H	-2.27576800	-4.08681400	-2.01013900
H	-1.80851000	-3.37593400	-0.46307200
H	-1.54514200	-2.47864800	-1.95550300
H	-4.55358600	-4.13362400	-2.38250600
H	-8.09929000	-3.17752900	-1.79318400
H	-7.02944000	-4.47554800	-2.35170100
H	-7.31570200	-3.06363200	-3.37071800
H	-7.16892800	-1.12422300	-0.83884800
H	-6.45767800	0.39798800	0.68305900
H	-4.78855600	0.54034000	1.24702800
H	-5.27687600	1.33347300	-0.24765100
H	-0.47885000	-0.92346000	-0.36166800
C	0.95944400	-3.61416100	-2.25589800
C	0.85652500	-3.74513700	-0.87527000
C	0.86705200	-2.60796800	-0.07459100
C	0.98209400	-1.28822900	-0.59840600
C	1.04497100	-1.21960500	-2.02754200
C	1.02875800	-2.34238200	-2.83620400
H	0.98017000	-4.49909700	-2.88542200
H	0.79224300	-4.72934700	-0.42222100
H	0.82894000	-2.73508600	1.00594200
H	1.15544100	-0.24418800	-2.49098500
H	1.10338800	-2.24027700	-3.91422200

P20_P

P	-2.90769300	-0.38477900	0.06928700
C	-2.59669000	-0.84855400	1.76792400
C	-3.58776700	-1.20582800	2.69222400
C	-3.23312000	-1.56677900	3.99122400
C	-1.89513000	-1.61193300	4.39766200
C	-0.89169500	-1.30054200	3.48429700
C	-1.27461500	-0.91932500	2.18483600
O	-0.22395200	-0.66022100	1.39153500
C	0.91450600	-0.90944800	2.15388300
C	0.56120500	-1.27488800	3.45951000
C	1.58326000	-1.52623600	4.37878700
C	2.88916000	-1.39441100	3.92785100
C	3.17869000	-1.05085000	2.59632800
C	2.19565000	-0.81079600	1.63016500
B	2.41455200	-0.36393200	0.07068200
C	3.96807500	-0.62194700	-0.41690600
C	4.74186000	0.24995900	-1.17710300
F	4.27535500	1.46189100	-1.52539400
C	6.01540800	-0.05256200	-1.64528300
F	6.70637500	0.83662000	-2.36362400
C	6.56777800	-1.29395000	-1.37297300
F	7.78764000	-1.60381300	-1.81412200
C	5.82932700	-2.21284200	-0.64342600
C	4.55893600	-1.86654400	-0.20133500
F	3.88934900	-2.82408000	0.46264500
F	6.33820200	-3.42095600	-0.38663500
C	1.99959600	1.23551700	0.09162700
C	0.79362600	1.78014100	-0.33015400
F	-0.17555800	1.01646500	-0.88601000
C	0.47478200	3.13110900	-0.22303500
F	-0.69287600	3.59389000	-0.69007300
C	1.36808200	4.00282300	0.37240900
F	1.07265800	5.29904900	0.49003600
C	2.57421300	3.50620900	0.84819500
C	2.85147400	2.15566100	0.70553500
F	4.02866700	1.74037500	1.19363000
F	3.44237300	4.33061400	1.43726400
C	-3.03843500	1.36922700	-0.32928500
C	-3.02315100	1.65344900	-1.71318400
C	-2.68781100	0.61370700	-2.75607500
C	-3.28802000	2.95772600	-2.12038300
C	-3.54095000	3.97647500	-1.20507200
C	-3.76371500	5.38980100	-1.66735400
C	-3.50096500	3.67198600	0.15396500
C	-3.23611000	2.38816200	0.62392900
C	-3.14931000	2.18615800	2.11765000
C	-4.36230400	-1.30571700	-0.50052600
C	-4.14539500	-2.59457600	-1.03530100
C	-2.76933100	-3.19329900	-1.16523000
C	-5.24740900	-3.32611000	-1.47018900
C	-6.54530000	-2.82591700	-1.38479000
C	-7.71313100	-3.62898500	-1.89095900
C	-6.73069200	-1.56156500	-0.83072700
C	-5.66544500	-0.78061800	-0.38275900
C	-5.97984500	0.57411600	0.20668300
H	-4.63133500	-1.21013300	2.38944200
H	-4.01282600	-1.83331800	4.69579900
H	-1.64481900	-1.90611800	5.41182900
H	1.36431400	-1.80963200	5.40299400
H	3.70980900	-1.57129200	4.61556500
H	4.22106700	-0.97061100	2.29983000

H	-2.77377500	1.04532800	-3.75405000
H	-1.65471900	0.26458200	-2.63399800
H	-3.35374900	-0.25528200	-2.71833100
H	-3.27722400	3.18576600	-3.18300000
H	-4.41567800	5.93589200	-0.98211600
H	-2.80507900	5.91742600	-1.70697400
H	-4.20282300	5.41901000	-2.66675600
H	-3.65557000	4.46791300	0.87776500
H	-3.28762700	3.14304200	2.62285300
H	-2.17149300	1.79204600	2.40799400
H	-3.90225300	1.49363700	2.50425400
H	-2.83644100	-4.23678000	-1.47748700
H	-2.20574500	-3.16704000	-0.22616000
H	-2.16489900	-2.66881800	-1.91376700
H	-5.08420300	-4.31869800	-1.88200200
H	-7.82676500	-3.49656000	-2.97163900
H	-8.64571400	-3.31618600	-1.41718400
H	-7.56919600	-4.69539800	-1.70340700
H	-7.73903600	-1.16646600	-0.73782000
H	-7.04658400	0.64018900	0.42721100
H	-5.43925100	0.75647900	1.13991900
H	-5.72178500	1.38489400	-0.48117900
H	-1.79162000	-0.86487000	-0.62998100
C	0.29959100	-2.85705900	-2.97421800
C	0.46279300	-3.33383600	-1.67621500
C	1.06440100	-2.53452300	-0.70290400
C	1.54238400	-1.24944000	-0.99158600
C	1.38209800	-0.80351100	-2.31401100
C	0.76066700	-1.57797600	-3.28914900
H	-0.16383000	-3.47855100	-3.73557400
H	0.12591700	-4.33593700	-1.42006200
H	1.19573500	-2.93431200	0.30053600
H	1.76695800	0.17939300	-2.58450100
H	0.65746700	-1.19694400	-4.30179500

P21_TS

P	-1.93739100	0.29621600	0.57571900
C	-2.19155300	1.94546100	-0.17508200
C	-3.27245600	2.78833800	0.10354700
C	-3.32406000	4.06000000	-0.45617100
C	-2.31349200	4.50013500	-1.31031000
C	-1.21842600	3.69118900	-1.60756400
C	-1.17347400	2.43788200	-0.99522800
O	-0.12278000	1.60389700	-1.19599900
C	1.11901100	2.18427900	-1.44718800
C	2.20729700	1.46732200	-0.95166500
C	3.45201500	2.04478100	-1.21312800
C	3.57527400	3.26148600	-1.88450100
C	2.44738600	3.94691200	-2.31790700
C	1.17661800	3.40512700	-2.10920800
C	-0.12486300	4.01487100	-2.62109800
C	-0.00570300	5.51865800	-2.86750900
C	-0.48529500	3.31010100	-3.94867200
B	1.96320500	0.14307200	-0.03276100
C	1.12856000	-1.05882700	-0.80381700
C	0.86097500	-2.27127300	-0.16825700
F	1.23662800	-2.45989300	1.11474400
C	0.26792200	-3.37194600	-0.76230400
F	0.01627500	-4.47995300	-0.05780700
C	-0.11791300	-3.29742700	-2.08985100
F	-0.72676700	-4.32721900	-2.67757300
C	0.11645100	-2.12432800	-2.77989900
C	0.74268300	-1.05513700	-2.14560900
F	0.95307800	-0.00296600	-2.95463200
F	-0.29810600	-2.00466100	-4.04379100
C	3.43176900	-0.42814100	0.47819900
C	4.04887100	-1.59718400	0.03441400
F	3.48126500	-2.39146600	-0.88652300
C	5.29254800	-2.03838800	0.48085400
F	5.80917200	-3.17691200	0.01615700
C	6.00108100	-1.28594200	1.39988000
F	7.19336600	-1.68923800	1.83487700
C	5.45384200	-0.09317400	1.84920500
C	4.21221700	0.30675800	1.37657100
F	3.78498100	1.49763900	1.82451400
F	6.12928400	0.66415500	2.71606700
C	-3.31704900	0.22411300	1.81004600
C	-3.01121400	0.34821600	3.17911800
C	-1.59596800	0.51214700	3.65305400
C	-4.03227900	0.28245100	4.12381200
C	-5.36183700	0.09785500	3.75376100
C	-6.44699200	0.00460700	4.79339900
C	-5.64927700	-0.00826000	2.39743700
C	-4.66064500	0.04645000	1.41080100
C	-5.12618300	-0.08707400	-0.02091600
C	-2.35678000	-1.12341600	-0.52400400
C	-2.68406800	-1.05897500	-1.89583100
C	-2.48631200	0.16257000	-2.75588600

C	-3.19135200	-2.20113600	-2.51472300
C	-3.37557100	-3.40469400	-1.83675700
C	-3.90307500	-4.61961500	-2.54985800
C	-2.97438200	-3.46635600	-0.50675700
C	-2.45094100	-2.35624500	0.15745900
C	-1.97323700	-2.55873200	1.57774900
H	-4.06321800	2.45632000	0.76760900
H	-4.16188900	4.71091200	-0.23140800
H	-2.38672000	5.48863200	-1.75152700
H	4.35352600	1.54059800	-0.87871400
H	4.55988800	3.68282300	-2.06063100
H	2.55833000	4.90048000	-2.82293100
H	0.76252300	5.72085100	-3.61725100
H	0.25054600	6.05746000	-1.95066500
H	-0.94266000	5.91982500	-3.26312100
H	0.27751100	3.53023800	-4.70170000
H	-0.52010200	2.22511200	-3.81528700
H	-1.45813100	3.65425800	-4.31498400
H	-1.56016100	0.71610400	4.72544200
H	-1.02061400	-0.40100600	3.47291900
H	-1.08123000	1.33026300	3.14194800
H	-3.77826500	0.37868100	5.17694600
H	-6.42507900	-0.97207500	5.28746600
H	-6.31701100	0.76565200	5.56715300
H	-7.43580300	0.13178700	4.34789800
H	-6.68298400	-0.13673300	2.08462300
H	-6.18166200	0.18646200	-0.08717300
H	-5.01912800	-1.11750000	-0.37516300
H	-4.57007000	0.55410700	-0.70796500
H	-2.73864700	-0.07153300	-3.79165100
H	-1.44050000	0.48004000	-2.73051800
H	-3.10359700	1.00920900	-2.43912000
H	-3.43984700	-2.14753700	-3.57190100
H	-4.36469800	-5.32109800	-1.85143800
H	-3.08177100	-5.13728700	-3.05414000
H	-4.64183000	-4.34506400	-3.30668700
H	-3.04109700	-4.40985100	0.03017700
H	-1.63939000	-3.59109900	1.70169100
H	-1.11514900	-1.92123200	1.81704000
H	-2.76243700	-2.35597100	2.30901600
H	-0.20227200	0.38741500	1.06645400
C	1.62786100	0.60487100	3.87632900
C	1.43527700	-0.00336200	2.64308500
C	1.14788200	0.72424400	1.45263700
C	1.00238800	2.12968300	1.64803700
C	1.15634800	2.74630000	2.88076900
C	1.50041300	1.98635400	3.99807300
H	1.88754600	0.00296200	4.74170300
H	1.56331800	-1.07665600	2.57637000
H	0.78743900	2.76758700	0.79718800
H	1.04401000	3.82242200	2.96525500
H	1.66404100	2.46814900	4.95755600

P21_P

P	-2.53963400	-0.02213700	0.17250800
C	-2.71395400	1.60252600	-0.57064500
C	-3.98719300	2.11921800	-0.83376600
C	-4.11422700	3.41666400	-1.30157500
C	-2.97167600	4.19054600	-1.50085900
C	-1.69166500	3.70287500	-1.25169200
C	-1.57298600	2.39112700	-0.77375700
O	-0.38174400	1.81337800	-0.51383500
C	0.73659100	2.63604500	-0.27136000
C	1.84322100	2.00806200	0.30596400
C	2.89299500	2.87596700	0.63239000
C	2.85355200	4.24215500	0.37286600
C	1.76692000	4.78658700	-0.29498500
C	0.68812000	3.97732400	-0.65459300
C	-0.41867100	4.45933900	-1.58585500
C	-0.62422000	5.97456900	-1.51867600
C	-0.01539900	4.06922000	-3.02721900
B	1.90414000	0.40567200	0.67482900
C	1.58385200	-0.59812100	-0.61907100
C	1.67995200	-1.98517100	-0.46879300
F	1.97543300	-2.52225600	0.72748500
C	1.51961100	-2.90561100	-1.49706900
F	1.52711600	-4.22238200	-1.25435500
C	1.33755800	-2.45110200	-2.79028900
F	1.12491400	-3.31171600	-3.79125300
C	1.29603000	-1.08904000	-3.01618800
C	1.43069500	-0.20664200	-1.95038500
F	1.40314500	1.08652800	-2.31611100
F	1.06053600	-0.63261700	-4.25296100
C	3.47588200	-0.00250500	1.02405800
C	4.45215200	0.20264900	0.04664400
F	4.11350700	0.77136000	-1.12323500
C	5.78875700	-0.13708300	0.19410800
F	6.67177100	0.09807300	-0.78023700

C	6.20654600	-0.73534500	1.37451300
F	7.48512300	-1.07519100	1.54170200
C	5.27792200	-0.97537700	2.37250800
C	3.94531900	-0.61547900	2.18256700
F	3.12949300	-0.91141900	3.20266900
F	5.66557800	-1.55871400	3.50988600
C	-4.09966900	-0.31066600	1.04877100
C	-4.21699400	0.23979800	2.34337900
C	-3.13901700	1.09859600	2.95546700
C	-5.38632700	-0.00241200	3.05931300
C	-6.43620300	-0.74969800	2.52753300
C	-7.67093300	-1.02469300	3.34312100
C	-6.31494000	-1.22506200	1.22395700
C	-5.16675700	-1.01604700	0.45927500
C	-5.16017000	-1.53106200	-0.96268900
C	-2.11146600	-1.44383900	-0.86628600
C	-1.87842100	-1.34384300	-2.25329100
C	-1.81862400	-0.04164200	-3.01492800
C	-1.65507100	-2.52272900	-2.96463200
C	-1.60174200	-3.77091800	-2.34907100
C	-1.24695600	-5.01178900	-3.12020900
C	-1.81176400	-3.83369600	-0.97328000
C	-2.08171900	-2.69827000	-0.21388300
C	-2.39153200	-2.90270500	1.25133300
H	-4.86812600	1.51367900	-0.64465600
H	-5.09550100	3.83012400	-1.50445500
H	-3.08648800	5.20316700	-1.87147400
H	3.77448600	2.46599000	1.11588100
H	3.68780300	4.87485700	0.65866000
H	1.76735000	5.84009800	-0.55032900
H	0.29188900	6.49403500	-1.80475700
H	-0.91165900	6.29822300	-0.51431100
H	-1.39055000	6.29675100	-2.22813500
H	0.91363400	4.57899500	-3.29902600
H	0.15752200	2.99219400	-3.10358600
H	-0.80123500	4.35579700	-3.73473300
H	-3.48543900	1.51885000	3.90106300
H	-2.22601800	0.53188900	3.16716900
H	-2.87031600	1.93480600	2.30050500
H	-5.48247400	0.41595700	4.05814500
H	-8.51079200	-1.31587600	2.70925800
H	-7.48743900	-1.83920200	4.05108600
H	-7.96309600	-0.14609600	3.92338400
H	-7.14477200	-1.76712000	0.77708600
H	-6.18716600	-1.70636900	-1.28909100
H	-4.60983200	-2.47240300	-1.05837600
H	-4.70740700	-0.82066900	-1.66149300
H	-1.55708000	-0.24065100	-4.05503500
H	-1.04441500	0.61589800	-2.60768600
H	-2.76847700	0.50092700	-2.99661500
H	-1.45198200	-2.45191600	-4.03046000
H	-1.90074700	-5.84503000	-2.85002500
H	-0.21765400	-5.29837000	-2.88520300
H	-1.31210700	-4.84531200	-4.19672200
H	-1.75881700	-4.79631100	-0.46991500
H	-1.82681200	-3.75928500	1.62735300
H	-2.14024300	-2.04872200	1.88172600
H	-3.45814200	-3.11151900	1.39223400
H	-1.52188900	0.10135000	1.13380100
C	-0.33077900	-1.19338900	3.58339800
C	0.24604500	-0.98971200	2.32704000
C	0.96037400	0.17659800	2.00677700
C	0.96860500	1.17109300	3.00222500
C	0.38995000	0.98807900	4.25532600
C	-0.24423000	-0.21211400	4.56621000
H	-0.83099100	-2.13478000	3.79982100
H	0.17489800	-1.79290400	1.60015100
H	1.48335400	2.10764400	2.80428400
H	0.45422900	1.77815500	4.99844900
H	-0.67333600	-0.37483100	5.55045200

P22_TS

P	2.18300400	0.83226300	1.56183300
C	3.29142800	-0.43786800	0.87015900
C	4.50943600	-0.87633400	1.39905300
C	5.22343300	-1.88451200	0.75906700
C	4.74318100	-2.45096000	-0.42343900
C	3.54433000	-2.02610100	-0.99100300
C	2.83848300	-1.03143500	-0.31160900
O	1.64112600	-0.59020900	-0.79441200
C	0.83461600	-1.54015600	-1.41868100
C	-0.53549500	-1.42366400	-1.18268100
C	-1.32128900	-2.38775600	-1.82214000
C	-0.76167900	-3.40397800	-2.59295800
C	0.61706600	-3.49256900	-2.75616500
C	1.44967700	-2.54097900	-2.16839800
C	2.96794200	-2.48527000	-2.32652300
C	3.30243600	-1.40448900	-3.37987700

C	3.55503500	-3.82229500	-2.77432600
B	-1.10949600	-0.26853200	-0.18662600
C	-0.64609600	1.23075200	-0.68276300
C	-0.55114800	1.51622600	-2.04855200
F	-0.81825200	0.57633000	-2.96195700
C	-0.24084500	2.76857700	-2.56328300
F	-0.16886200	2.96169500	-3.87987800
C	-0.01010000	3.82944700	-1.69960200
F	0.31453700	5.03144900	-2.16945500
C	-0.11678600	3.60874200	-0.33847700
C	-0.43497700	2.33886400	0.12742000
F	-0.51937600	2.24484900	1.47503900
F	0.13638500	4.59735300	0.52673000
C	-2.75246700	-0.34451900	-0.06504900
C	-3.64634500	0.64593200	-0.47144600
F	-3.23109800	1.79603700	-1.02407000
C	-5.02998700	0.54153000	-0.34016100
F	-5.81983700	1.53572500	-0.74695700
C	-5.58603700	-0.59678300	0.21471500
F	-6.90459500	-0.71315700	0.34938800
C	-4.74416900	-1.61963500	0.62833400
C	-3.37370200	-1.47429200	0.47782800
F	-2.63350000	-2.51144900	0.89651800
F	-5.25867800	-2.72809400	1.16315700
C	2.56139100	0.91756300	3.36959900
C	2.22814700	-0.40903200	4.05673500
C	1.74426800	2.06070100	3.98337500
C	2.81841600	2.43628000	0.84643100
C	4.15743200	2.86457600	1.45054700
C	2.93666500	2.32259500	-0.67752200
H	4.88971800	-0.44406800	2.32090700
H	6.16220200	-2.23115000	1.17748700
H	5.32088600	-3.23115600	-0.90835000
H	-2.40102100	-2.34916900	-1.71713900
H	-1.40612900	-4.13862500	-3.06509700
H	1.03951300	-4.30009900	-3.34475800
H	2.87085500	-1.68252000	-4.34597100
H	4.38693800	-1.30321600	-3.48993700
H	2.88842000	-0.43546500	-3.08644600
H	3.14549200	-4.11087500	-3.74524900
H	4.63849700	-3.74341200	-2.89921300
H	3.33950400	-4.61746300	-2.05445000
H	3.63102000	1.12961300	3.48739700
H	2.51810800	-0.35997200	5.11100300

H	2.74403800	-1.25713900	3.59812100
H	1.15228100	-0.60252900	4.01435100
H	1.95273100	2.13337300	5.05495400
H	0.67327100	1.87012800	3.85831400
H	1.96677700	3.03063500	3.52930700
H	2.04923100	3.17846800	1.09404300
H	4.51420800	3.76402500	0.93887600
H	4.08770800	3.09500000	2.51653100
H	4.91220500	2.08236100	1.31098800
H	3.11410600	3.31508400	-1.10370400
H	3.78535700	1.68334200	-0.94376600
H	2.04017300	1.90223200	-1.13801300
H	0.52388100	0.09903400	1.33152500
C	-0.18738400	-2.75383000	2.79408400
C	-1.01995700	-2.26083600	3.79638400
C	-1.59552800	-0.99402400	3.66697500
C	-1.34099700	-0.23656600	2.53586800
C	-0.53173700	-0.70848300	1.46553500
C	0.03707900	-1.99758200	1.65325400
H	0.26457000	-3.73515600	2.89472000
H	-1.22238100	-2.85962700	4.67959400
H	-2.24589000	-0.60967600	4.44621000
H	-1.80167100	0.74167100	2.44543200
H	0.66584400	-2.41689900	0.87239300

P22_P

P	3.01135300	-0.86331300	-1.37530100
C	3.63392000	0.57454800	-0.51264200
C	4.98409200	0.91288000	-0.63825400
C	5.51806200	1.92923900	0.13841900
C	4.69532800	2.58372600	1.04864000
C	3.34458600	2.27157100	1.20484000
C	2.80911000	1.25440500	0.40585900
O	1.52521900	0.84434900	0.48768000
C	0.58651900	1.61161500	1.20510700
C	-0.76556300	1.30672000	0.97928600
C	-1.67043200	2.02493100	1.77159900
C	-1.27370200	2.97722600	2.70105000
C	0.07201500	3.26888100	2.84662400
C	1.03303600	2.59947100	2.08733700
C	2.50515200	2.95150900	2.27365900
C	2.97935000	2.45297100	3.65414000
C	2.69333500	4.47819500	2.18345500
B	-1.33235100	0.14979700	-0.05028300

C	-1.18988400	-1.25869400	0.81408100
C	-1.96710800	-1.45495400	1.95950700
F	-2.92358500	-0.57192700	2.27453500
C	-1.83977300	-2.53446900	2.81951500
F	-2.62112000	-2.64992500	3.89344800
C	-0.87917800	-3.50661700	2.56488800
F	-0.72462200	-4.54150400	3.38950800
C	-0.09107600	-3.37242000	1.43844000
C	-0.27387400	-2.27470700	0.60304400
F	0.55821600	-2.25488900	-0.46783000
F	0.86832500	-4.27002500	1.16307300
C	-2.91273100	0.44438900	-0.45113100
C	-3.92259100	-0.51079000	-0.51833900
F	-3.69238100	-1.78886100	-0.16696300
C	-5.21376900	-0.24511300	-0.96134700
F	-6.13406000	-1.21208000	-0.99199600
C	-5.54165000	1.03179900	-1.38551900
F	-6.77232700	1.30756900	-1.81745200
C	-4.56666800	2.01768000	-1.36456900
C	-3.29036200	1.70377500	-0.91789300
F	-2.39400200	2.70288800	-0.96095300
F	-4.86009500	3.24991000	-1.78740800
C	3.88193300	-1.09668400	-2.97112500
C	3.89146900	0.16726400	-3.83460500
C	3.22414700	-2.27690700	-3.69895700
C	3.32863900	-2.33514700	-0.30365100
C	4.77430600	-2.81789900	-0.45315600
C	3.02553100	-1.99326000	1.16114400
H	5.61507400	0.38031300	-1.34477600
H	6.56292100	2.20190600	0.04655400
H	5.12521700	3.35822000	1.67600500
H	-2.72946800	1.82100800	1.65599000
H	-2.01328700	3.49856500	3.30003000
H	0.38519200	4.02042900	3.56481200
H	2.39086700	2.92281400	4.44717900
H	4.03492800	2.69659700	3.81505800
H	2.85612600	1.36927900	3.73444700
H	2.12097300	4.98552100	2.96184900
H	3.74027900	4.75996800	2.32359000
H	2.35747300	4.85092300	1.21271900
H	4.91193800	-1.36284300	-2.70501500
H	4.45985300	-0.03542700	-4.74669000
H	4.35089000	1.01636300	-3.32414600
H	2.87424700	0.44406800	-4.11657200
H	3.75813000	-2.46868100	-4.63326900
H	2.18348300	-2.03878800	-3.94058900
H	3.24042800	-3.19665200	-3.10673300
H	2.62977100	-3.10573100	-0.64032000
H	4.94985900	-3.61582300	0.27308500
H	4.99100400	-3.21941900	-1.44642000
H	5.48574200	-2.01393600	-0.23246500
H	3.02201500	-2.91948100	1.74042800
H	3.79928600	-1.33223900	1.56447900
H	2.05733600	-1.50584500	1.29080800
H	1.64192400	-0.69459400	-1.62527400
C	0.48352200	1.30482100	-3.36611000
C	0.28082700	0.21603800	-4.21280600
C	-0.39370900	-0.90553000	-3.72841400
C	-0.85125200	-0.92730200	-2.41369700
C	-0.63788500	0.14206700	-1.52742700
C	0.02850000	1.26186100	-2.04796500
H	0.98373000	2.19619300	-3.73678200
H	0.62065900	0.24913900	-5.24478400
H	-0.58413700	-1.75095900	-4.38465300
H	-1.40196200	-1.79744500	-2.05929300
H	0.17706300	2.12955800	-1.40956300

P23_TS

C	-1.46446200	-2.08766500	-2.16293800
H	-0.55727000	-2.42631100	-2.66331600
H	-2.03103800	-1.47379400	-2.87355800
H	-2.07516600	-2.96384300	-1.92382500
C	-1.17287500	-1.25944900	-0.93771800
C	0.01990500	-0.72306800	-0.59714600
P	-2.59336300	-0.80092800	0.10856000
C	-3.84982700	0.04539100	-0.90656900
C	-4.56312700	-0.62291700	-1.90935500
H	-4.40097600	-1.68434200	-2.07603300
C	-5.48755100	0.07114900	-2.68186800
H	-6.03604300	-0.44775500	-3.46118700
C	-5.70563500	1.43027000	-2.45974900
H	-6.42137200	1.97022500	-3.07112700
C	-5.01384100	2.09410900	-1.45154900
H	-5.18436700	3.15046300	-1.27263700
C	-4.09744900	1.39916900	-0.66745800
H	-3.56565800	1.90923300	0.13051500
C	-3.35067200	-2.39047300	0.58131200
C	-4.69398800	-2.43398800	0.97386200

H	-5.30489000	-1.53712200	0.91447900
C	-5.25187500	-3.62289500	1.42968800
H	-6.29518100	-3.64813300	1.72722800
C	-4.47501400	-4.77754500	1.50380200
H	-4.91225100	-5.70470000	1.85986100
C	-3.13901100	-4.74096200	1.11624500
H	-2.53260100	-5.63967200	1.16234700
C	-2.57493900	-3.55343300	0.65806900
H	-1.53615000	-3.53660300	0.33739800
B	0.33066900	0.36892700	0.61337800
C	1.20210100	-1.12042100	-1.41395200
F	0.97415400	-3.38843400	-0.79305500
C	1.66147100	-2.43391800	-1.43373600
F	3.24550400	-4.06280200	-2.07973900
C	2.83242600	-2.79799400	-2.08271500
F	4.69750400	-2.16133600	-3.36978400
C	3.57656700	-1.82704900	-2.74039900
F	3.86642200	0.42417000	-3.36101900
C	3.14880500	-0.50504000	-2.73684600
F	1.60587100	1.11085700	-2.04709600
C	1.97397200	-0.17215000	-2.07828500
C	-0.33138900	1.84334800	0.30197400
F	-1.21517000	1.35733100	-1.87395000
C	-1.02594300	2.20649900	-0.85322000
F	-2.29036200	3.71509000	-2.16223600
C	-1.59257100	3.46475200	-1.05356300
F	-2.00286200	5.64642200	-0.26228100
C	-1.45518600	4.44692200	-0.09003600
F	-0.53170200	5.10508200	1.97601600
C	-0.72251900	4.16184100	1.05423600
F	0.60769000	2.74001600	2.28773900
C	-0.17408100	2.89915500	1.20696200
C	1.96230800	0.47040700	0.87169500
F	2.22567900	2.76518900	0.23180700
C	2.74647400	1.61016500	0.66284600
F	4.79554600	2.78151000	0.62308600
C	4.12745900	1.64862100	0.83409500
F	6.13307500	0.52621700	1.36473100
C	4.81414300	0.50503100	1.20311400
F	4.73052100	-1.79989800	1.68732000
C	4.09855100	-0.66802000	1.37620000
F	2.13985100	-1.86509600	1.30346100
C	2.72222300	-0.65625200	1.19922700
H	-1.46885600	-0.21599500	1.35927000
C	-0.42029400	-0.28542300	2.10669500
C	-0.21859500	-1.66970300	2.36426100
C	-0.23922300	-2.19362600	3.64569000
C	-0.49202200	-1.35391800	4.72960000
C	-0.75525700	-0.00034900	4.52059500
C	-0.72846100	0.51812900	3.23564200
H	-0.04244200	-2.33156400	1.52406200
H	-0.06810500	-3.25327900	3.80297100
H	-0.51073700	-1.75961300	5.73672200
H	-0.99376300	0.64374000	5.36033100
H	-0.99508600	1.55729300	3.09397400

P23_P

C	-1.44261600	-1.97194100	-2.18066100
H	-0.52421500	-2.34092200	-2.63444500
H	-1.96624200	-1.36461800	-2.92786500
H	-2.07690700	-2.83637800	-1.95323400
C	-1.14529700	-1.14010900	-0.95103000
C	0.05320800	-0.66668900	-0.54745600
P	-2.65747100	-0.63602200	-0.11961300
C	-3.88548200	0.02741600	-1.26853600
C	-4.68099000	-0.83619000	-2.02971700
H	-4.59736600	-1.91248300	-1.90331200
C	-5.58948800	-0.31005200	-2.94099400
H	-6.20684800	-0.97681600	-3.53330000
C	-5.70667800	1.07081200	-3.09088400
H	-6.41521900	1.47726200	-3.80511300
C	-4.92407300	1.93058300	-2.32512500
H	-5.01103600	3.00563900	-2.43876500
C	-4.01428300	1.41149500	-1.41041100
H	-3.41415200	2.08427600	-0.80597000
C	-3.42427300	-2.05891500	0.68784400
C	-4.68337900	-1.90848500	1.27535900
H	-5.19885700	-0.95262400	1.23254100
C	-5.27446300	-2.99074400	1.91666600
H	-6.24635100	-2.87560600	2.38416700
C	-4.61919300	-4.21981500	1.95289700
H	-5.08525600	-5.06428200	2.45011600
C	-3.37115500	-4.37138600	1.35333100
H	-2.86286300	-5.32916300	1.38404600
C	-2.76376900	-3.28920800	0.72470300
H	-1.77412700	-3.39164100	0.28688900
B	0.40022700	0.38712300	0.71763000
C	1.22653700	-1.16678600	-1.31844600

F	0.82541200	-3.39987100	-0.65584100
C	1.59911800	-2.50678200	-1.28692300
F	3.09419700	-4.24931500	-1.83603500
C	2.76548300	-2.96139700	-1.88510800
F	4.70109500	-2.48248400	-3.13328600
C	3.58811300	-2.05872700	-2.54616800
F	4.02973200	0.14895600	-3.23327400
C	3.24242800	-0.71331500	-2.59837600
F	1.77182900	1.01160100	-2.01768800
C	2.07237200	-0.28740400	-1.98709000
C	-0.11761700	1.92329400	0.35477900
F	-1.14129300	1.49955000	-1.77202900
C	-0.82619700	2.34338900	-0.76628600
F	-2.05081500	3.92655500	-2.02786000
C	-1.30615400	3.63921700	-0.95294700
F	-1.48082000	5.85857000	-0.17409900
C	-1.03204900	4.61607600	-0.01527900
F	0.02285800	5.19945600	2.00670200
C	-0.27685100	4.27213800	1.09871100
F	0.91346200	2.72441900	2.32714300
C	0.15759400	2.96506200	1.24703100
C	2.05488000	0.37872000	0.93347800
F	2.51095700	2.62023500	0.21563200
C	2.93399600	1.43540100	0.67963700
F	5.07907500	2.42098800	0.58531000
C	4.31516200	1.35829400	0.83847700
F	6.22111500	0.08091900	1.39173500
C	4.90222800	0.17225400	1.24304500
F	4.62025800	-2.09642100	1.81641400
C	4.08668100	-0.92467600	1.46758800
F	2.02317500	-1.93711100	1.47023600
C	2.71429900	-0.79339100	1.30854500
H	-2.41844300	0.38555800	0.80244500
C	-0.39825500	-0.15941900	2.07285100
C	-0.54741300	-1.53664400	2.31811900
C	-1.25478600	-2.03904100	3.40957000
C	-1.87316700	-1.17445200	4.30343800
C	-1.76296300	0.19559200	4.08604400
C	-1.03951300	0.68374300	3.00034700
H	-0.10935100	-2.24755900	1.62658200
H	-1.33658700	-3.11439800	3.54392100
H	-2.43343200	-1.55841600	5.15036000
H	-2.23849100	0.89540300	4.76738400
H	-0.99744800	1.75954600	2.88592400

P24_TS

P	-1.88612300	0.40011300	0.43301100
B	0.97535100	-0.75481400	0.60112300
C	-0.51256200	1.12495300	-0.56871600
C	0.71676900	0.57717500	-0.35453800
C	-3.07636800	1.74310900	0.85947500
C	-3.97909000	2.26194600	-0.10506300
C	-4.85520400	3.27660300	0.26740500
H	-5.53326500	3.67764500	-0.48273100
C	-4.89532400	3.79260800	1.56222400
C	-4.03230300	3.24728700	2.50171100
H	-4.05807000	3.61421500	3.52520300
C	-3.12692200	2.23041800	2.18107100
C	-4.06507800	1.77616200	-1.52964700
H	-4.53594200	2.54043900	-2.15174200
H	-4.66148800	0.86044400	-1.59626200
H	-3.08311400	1.56812400	-1.95336200
C	-5.85343300	4.89799400	1.91758500
H	-5.62047700	5.80792500	1.35623100
H	-5.80936600	5.13602100	2.98217800
H	-6.88173100	4.61793800	1.67160100
C	-2.25803900	1.73772800	3.31225700
H	-1.36926900	2.36635200	3.41733900
H		-1.91510800	0.71003700
3.18913500			
H	-2.80946000	1.79721600	4.25375200
C	-2.95681300	-0.86025800	-0.39125800
C	-2.85373400	-1.30085100	-1.72807000
C	-3.71351100	-2.30871200	-2.17148100
H	-3.61261200	-2.65487300	-3.19831600
C	-4.65652600	-2.91017500	-1.34590400
C	-4.74910300	-2.45412700	-0.03510400
H	-5.47128000	-2.91067800	0.63734300
C	-3.92182300	-1.44865300	0.46303500
C	-1.88467100	-0.75693800	2.74852200
H	-1.70539000	-1.50954100	-3.52089700
H	-0.91981300	-0.49732900	-2.31972400
H	-2.28720600	0.13537500	-3.24098300
C	-5.49763200	-4.05653500	-1.83800600
H	-6.41938800	-4.15232200	-1.26000100
H	-4.93810200	-4.99227100	-1.74158900
H	-5.75925100	-3.93289900	-2.89171600
C	-4.11608300	-1.05678800	1.90969100

H	-4.68364300	-0.12494400	1.99787200
H	-3.16640500	-0.92411800	2.43153100
H	-4.66558800	-1.84308600	2.43051400
C	-0.74166200	2.30744800	-1.45386800
C	-1.26298400	3.51549100	-0.97340100
H	-1.47691200	3.62800100	0.08383600
C	-1.49212100	4.58129500	-1.83729100
H	-1.89827800	5.50808100	-1.44486900
C	-1.19382100	4.46643100	-3.19208300
H	-1.37681600	5.29849200	-3.86436500
C	-0.64069100	3.28410800	-3.67606500
H	-0.38337400	3.19019400	-4.72618100
C	-0.41308900	2.21702200	-2.81374600
H	0.02782400	1.29929000	-3.19246800
C	1.88497600	1.36679500	-0.87417200
C	2.07815300	2.69412100	-0.47112600
F	1.21595400	3.29232900	0.36020900
C	3.17055400	3.45399400	-0.85481800
F	3.29928200	4.70851100	-0.42876100
C	4.14122300	2.89744600	-1.67453100
F	5.19220100	3.61470000	-2.05385500
C	3.99728000	1.58455000	-2.08946400
F	4.91538000	1.03044400	-2.87727000
C	2.89125600	0.84388600	-1.68684700
F	2.83379600	-0.40821900	-2.13106800
C	0.01002700	-2.03419200	0.15578300
C	0.12124200	-2.52200000	-1.14847900
F	0.97718500	-1.93559700	-1.99214900
C	-0.61467200	-3.56791600	-1.67676200
F	-0.48468200	-3.90809100	-2.96068100
C	-1.54789700	-4.21522500	-0.88137600
F	-2.31871000	-5.17671800	-1.38476600
C	-1.71075000	-3.78661600	0.42017900
F	-2.64668400	-4.33483400	1.19666100
C	-0.95108600	-2.72135800	0.89632300
F	-1.31703600	-2.32799300	2.13398300
C	2.56463100	-1.18452500	0.69850800
C	3.08497200	-2.44811500	0.40646200
F	2.30764500	-3.46081200	-0.00278900
C	4.43038500	-2.78379600	0.52597100
F	4.84119400	-4.01573400	0.22777400
C	5.34423200	-1.84096000	0.96239700
F	6.63203200	-2.14754600	1.08451100
C	4.88622100	-0.57246000	1.27876000
F	5.73725400	0.35914200	1.70883300
C	3.53504100	-0.28311900	1.14993800
F	3.19787600	0.96939100	1.50349500
H	-0.63779600	-0.14659500	1.63322300
C	0.80033100	1.10004300	2.64435400
C	0.58209200	-0.23666800	2.24469000
C	0.81148300	-1.22718000	3.23764600
C	1.21623600	-0.90087500	4.51933600
C	1.40573400	0.43817300	4.87336300
C	1.19770200	1.43923800	3.93227100
H	0.64164400	1.89339300	1.92034500
H	0.71845900	-2.27553200	2.97692200
H	1.39808000	-1.68557700	5.24626400
H	1.72448100	0.69345900	5.87949000
H	1.35423500	2.48085600	4.19390600

P24_P

P	-2.03384500	0.39792600	0.26144700
B	1.11316800	-0.77623400	0.82314800
C	-0.48582900	1.10639900	-0.41207500
C	0.73247000	0.58598000	-0.09385500
C	-3.23218200	1.70995400	0.67487900
C	-4.04726700	2.27073600	-0.33072600
C	-4.95554400	3.26615300	0.02642300
H	-5.57124000	3.70944200	-0.75215500
C	-5.09188500	3.71106600	1.33757900
C	-4.28021500	3.13635500	2.31331800
H	-4.36656100	3.47459600	3.34281200
C	-3.34917500	2.14339600	2.01530900
C	-3.98318300	1.86754400	-1.78171800
H	-4.45712800	2.63386300	-2.39801400
H	-4.49953700	0.91827600	-1.95560500
H	-2.95265200	1.76654700	-2.13198900
C	-6.09920200	4.76875600	1.69985700
H	-7.00303900	4.31102400	2.11457600
H	-6.39266500	5.35311000	0.82541500
H	-5.70055700	5.45105700	2.45440000
C	-2.50997400	1.59608300	3.14112700
H	-1.44208800	1.71248700	2.95101100
H	-2.68711300	0.52865100	3.31547200
H	-2.73911500	2.12074700	4.06987300
C	-2.97247500	-0.89868900	-0.62446100
C	-2.77759500	-1.24558200	-1.97444700
C	-3.58942700	-2.23441600	-2.53475100

H	-3.42065600	-2.50886900	-3.57299600
C	-4.56209600	-2.90327100	-1.80224900
C	-4.72614200	-2.55411300	-0.46287000
H	-5.46307700	-3.08147700	0.13712700
C	-3.9556900	-1.56893800	0.14754700
C	-1.72856900	-0.64098700	-2.86864100
H	-1.44929100	-1.36178600	-3.64024000
H	-0.82255300	-0.38445200	-2.32497700
H	-2.09955100	0.26036000	-3.36844100
C	-5.36089200	-4.02197500	-2.41080100
H	-6.35755900	-4.08655600	-1.96849900
H	-4.85078600	-4.97325400	-2.22996900
H	-5.46468300	-3.89605600	-3.49058500
C	-4.17715800	-1.32054300	1.62038100
H	-4.47334600	-0.28756500	1.82481800
H	-3.27891600	-1.54853800	2.20338700
H	-4.96852400	-1.97466500	1.98828200
C	-0.65576200	2.32898500	-1.26638900
C	-1.16578600	3.52351800	-0.74580800
H	-1.42530500	3.58520700	0.30589800
C	-1.32396400	4.64036300	-1.56161300
H	-1.72306000	5.55751900	-1.14090300
C	-0.95900200	4.58677300	-2.90302100
H	-1.08259200	5.45857300	-3.53741200
C	-0.40933400	3.41670400	-3.42237300
H	-0.09398600	3.37420200	-4.45981100
C	-0.25624800	2.29914600	-2.61039800
H	0.19450300	1.39584200	-3.01133700
C	1.86639000	1.44758400	-0.57380100
C	2.08833900	2.71683700	-0.03952900
F	1.25685100	3.22329200	0.87757200
C	3.16385100	3.50615800	-0.41324400
F	3.34252900	4.70431100	0.13739400
C	4.05293900	3.04387400	-1.37339300
F	5.08055700	3.79610900	-1.75059400
C	3.84572400	1.80066900	-1.95089700
F	4.67024500	1.35976700	-2.89768600
C	2.76433800	1.02655700	-1.54996700
F	2.58141100	-0.13648000	-2.17443000
C	0.05096800	-1.98568900	0.41434300
C	0.02706800	-2.47292100	-0.89341200
F	0.84566300	-1.93455600	-1.80729800
C	-0.80659000	-3.48145600	-1.34657200
F	-0.81387500	-3.83864500	-2.63307700

C	-1.70529900	-4.06437300	-0.46583300
F	-2.56222800	-4.99310600	-0.88881000
C	-1.74679900	-3.61222300	0.83992000
F	-2.67989400	-4.07918400	1.67470300
C	-0.88867500	-2.59414700	1.24610200
F	-1.11219000	-2.15501900	2.50169300
C	2.69211900	-1.24482900	0.55843500
C	3.15469700	-2.46216800	0.05621000
F	2.32029400	-3.45284900	-0.30023300
C	4.50049000	-2.78474100	-0.09632000
F	4.85208000	-3.97043200	-0.59654400
C	5.47620900	-1.88029900	0.28086900
F	6.76686800	-2.17168700	0.13773300
C	5.08050100	-0.66877700	0.82479100
F	5.99663900	0.21867900	1.21544000
C	3.72754300	-0.39538000	0.96779800
F	3.45194200	0.78809100	1.54081400
H	-1.67633400	-0.15829800	1.49170500
C	0.81900000	0.81526100	2.97600600
C	1.07147600	-0.44173600	2.42473400
C	1.41658800	-1.46356800	3.32584300
C	1.46022100	-1.25626100	4.69730500
C	1.17536600	0.00530200	5.22233600
C	0.86373000	1.04268400	4.35467400
H	0.61296400	1.65605400	2.31895800
H	1.65300900	-2.45231100	2.93373900
H	1.72232100	-2.07505700	5.36099200
H	1.21399500	0.17588400	6.29379200
H	0.66654600	2.03858000	4.74342400

P25_TS

C	-2.58639600	-1.60857500	3.90438600
C	-1.36941300	-1.88524500	4.45726300
C	-0.16951600	-1.74630300	3.71656200
C	-0.15459200	-1.26743300	2.35529200
C	-1.47325400	-0.95583000	1.82449100
C	-2.61810700	-1.15976300	2.57707100
H	0.94748700	-2.48249100	5.41083100
H	-3.50754000	-1.73672400	4.46228400
H	-1.29333900	-2.24422600	5.48002800
C	1.02047800	-2.13784800	4.38340400
C	1.14409900	-1.17233000	1.68275600
H	-3.58643700	-0.96077200	2.12831100
C	2.23743900	-1.62853900	2.39825600

C	2.20894600	-2.10128000	3.72616300
H	3.20708200	-1.63086700	1.91586000
H	3.12943000	-2.41878300	4.20551300
P	-1.80829900	-0.45132300	0.09074500
B	1.52660800	-0.61540400	0.16524000
C	3.16157000	-0.58647700	-0.13863100
C	3.93707500	0.31855700	0.59913700
F	3.37393600	1.02453200	1.59091300
C	5.29395000	0.53251600	0.41483200
F	5.95443200	1.40944600	1.17098900
C	5.96711400	-0.18561100	-0.56305800
F	7.27055600	-0.00511200	-0.75948300
C	5.25861200	-1.10161800	-1.31725700
C	3.89423500	-1.28710300	-1.09571600
F	3.34138200	-2.21592000	-1.88791900
F	5.88466900	-1.81584700	-2.25392900
C	1.09339300	0.95775000	-0.06063900
C	1.13519300	1.59873700	-1.29739700
F	1.52883400	0.93511800	-2.40158300
C	0.79485000	2.92728400	-1.50583000
F	0.74705500	3.42891500	-2.74129100
C	0.47211100	3.72118700	-0.41873700
F	0.08989000	4.98543100	-0.58895000
C	0.53484000	3.17157900	0.84923500
C	0.84364100	1.82713700	1.00477400
F	0.87085100	1.40366700	2.27576300
F	0.20506600	3.91541800	1.90651200
C	-3.44066900	-1.25020900	-0.33596200
C	-4.70552500	-0.61202600	-0.30126500
C	-4.98844700	0.77196500	0.24023000
C	-5.82818900	-1.32080400	-0.74138200
C	-5.76950300	-2.63500800	-1.18829600
C	-6.99832700	-3.34895300	-1.68418800
C	-4.53163000	-3.26600000	-1.15700700
C	-3.37878900	-2.60197500	-0.74585400
C	-2.08951100	-3.37627100	-0.75043600
C	-2.12287400	1.35400700	-0.14175300
C	-2.32248600	2.31192000	0.88135600
C	-2.21234700	2.02742300	2.36063000
C	-2.65719600	3.61639700	0.52221400
C	-2.79834800	4.02145000	-0.80451900
C	-3.11461000	5.45223500	-1.14511900
C	-2.57780300	3.07279500	-1.79471700
C	-2.23156500	1.75303400	-1.49002500
C	-1.94231100	0.84912200	-2.66822300
H	-6.06216800	0.87490900	0.40976200
H	-4.48814000	0.94734400	1.19444000
H	-4.67672600	1.56762900	-0.44168200
H	-6.79351700	-0.82093600	-0.71089800
H	-7.08250100	-3.26130000	-2.77241800
H	-6.96158600	-4.41385400	-1.44189300
H	-7.90626600	-2.92685200	-1.24737400
H	-4.45326900	-4.30735100	-1.46079000
H	-2.28332500	-4.44311600	-0.88080200
H	-1.53590000	-3.25239800	0.18536600
H	-1.44125000	-3.05610700	-1.57095300
H	-2.19555700	2.97022100	2.90962900
H	-1.31287800	1.47012700	2.61934900
H	-3.06381600	1.44305600	2.72465200
H	-2.80206500	4.34808700	1.31347600
H	-3.55053900	5.53719900	-2.14294700
H	-2.19704000	6.04792600	-1.12522300
H	-3.81086200	5.88676800	-0.42369400
H	-2.65670100	3.36191800	-2.84047100
H	-2.57348500	1.12659300	-3.51551000
H	-0.90014500	0.98559000	-2.98178200
H	-2.10366500	-0.21289500	-2.47192300
H	-0.24972600	-1.19742400	-0.56216200
C	0.66142000	-1.70340500	-2.36840800
C	0.81082000	-1.81203800	-0.96049800
C	1.01592300	-3.12836200	-0.46601300
C	1.12854400	-4.22691500	-1.30446200
C	1.00363100	-4.06513400	-2.68246800
C	0.75253700	-2.80008900	-3.21169300
H	0.42493200	-0.74224500	-2.80165000
H	1.11700400	-3.27978700	0.60345600
H	1.30795400	-5.21149500	-0.88472500
H	1.08477500	-4.92425000	-3.34176600
H	0.62715800	-2.67113400	-4.28184200

P25_P

C	-2.58990900	-1.69684400	3.82795900
C	-1.40644600	-2.21626000	4.26747000
C	-0.22895600	-2.16870600	3.48109600
C	-0.17995400	-1.52342200	2.18797200
C	-1.47048600	-0.99266100	1.76926800
C	-2.60775700	-1.11624300	2.55475200
H	0.81038100	-3.28423600	5.00780800

H	-3.49610700	-1.75181500	4.42001000
H	-1.34449600	-2.70424800	5.23645100
C	0.90533000	-2.81463300	4.03328800
C	1.09613900	-1.48923300	1.48393500
H	-3.56322900	-0.76157300	2.17742400
C	2.13226600	-2.18344900	2.09063200
C	2.06909500	-2.84642800	3.33090200
H	3.08692700	-2.22207900	1.57844300
H	2.94821500	-3.35122200	3.71864600
P	-1.94396600	-0.28088100	0.16545300
B	1.51399800	-0.81901900	0.01014900
C	3.17422300	-0.68389500	-0.15846200
C	3.91792700	0.02261000	0.79254000
F	3.31086200	0.50222600	1.89100100
C	5.27770900	0.27700800	0.70137600
F	5.91570900	0.95365000	1.65959600
C	5.97673400	-0.17376900	-0.40907300
F	7.28353800	0.05822600	-0.52492900
C	5.29241200	-0.86501400	-1.39210000
C	3.92434500	-1.10041000	-1.25909200
F	3.37948200	-1.76145600	-2.28696700
F	5.94654600	-1.30130600	-2.47073200
C	1.12034100	0.79601800	-0.08271500
C	1.26320400	1.53814700	-1.25850200
F	1.68581400	0.95277300	-2.39242500
C	1.01825500	2.90001000	-1.36506900
F	1.05971500	3.50680500	-2.55343300
C	0.70271700	3.62502600	-0.22732300
F	0.41028500	4.92403400	-0.30653100
C	0.67402800	2.96921200	0.98879100
C	0.87840000	1.59503700	1.03745000
F	0.79351900	1.07212600	2.27190500
F	0.34986100	3.64233600	2.09682600
C	-3.53141100	-1.05693000	-0.32825400
C	-4.77669000	-0.38559200	-0.33487100
C	-5.02749000	1.01821600	0.17142200
C	-5.89520700	-1.07912200	-0.79995000
C	-5.83738600	-2.40071900	-1.23131600
C	-7.07393300	-3.11698900	-1.70379300
C	-4.61173500	-3.05359800	-1.16436600
C	-3.45712800	-2.41597000	-0.71571800
C	-2.17903000	-3.21019300	-0.67968400
C	-2.09199800	1.52775500	-0.02805600
C	-2.25293400	2.42899500	1.05157400

C	-2.19266100	2.04671700	2.51106400
C	-2.47435100	3.77226000	0.76340400
C	-2.53577400	4.26281600	-0.54092100
C	-2.71171500	5.73280900	-0.80241700
C	-2.37450500	3.35840300	-1.58181000
C	-2.15280400	1.99633800	-1.35702500
C	-1.96906600	1.14674400	-2.59484000
H	-6.10263600	1.16766400	0.28549400
H	-4.56856700	1.19399200	1.14735200
H	-4.64951700	1.78755200	-0.50711100
H	-6.85205100	-0.56308200	-0.81232600
H	-6.82945400	-3.87260000	-2.45370500
H	-7.56498400	-3.62658400	-0.86845000
H	-7.79456300	-2.41972400	-2.13660400
H	-4.54314400	-4.09495900	-1.46873700
H	-2.39369600	-4.27202000	-0.81053100
H	-1.63612400	-3.09610900	0.26294700
H	-1.50594400	-2.91322100	-1.48748300
H	-2.17331100	2.95267600	3.11780500
H	-1.30484000	1.46241700	2.75303800
H	-3.06649300	1.46205200	2.81585700
H	-2.58063400	4.46648600	1.59278800
H	-3.11342100	5.91504000	-1.80130600
H	-1.74077200	6.23228300	-0.73054700
H	-3.37948000	6.18917600	-0.06798000
H	-2.40467600	3.71395500	-2.60914200
H	-2.64724100	1.49576100	-3.37683000
H	-0.94730000	1.26849600	-2.97349200
H	-2.15785000	0.08047000	-2.45988800
H	-1.01025800	-0.72119100	-0.77385200
C	0.41003400	-1.53036600	-2.40483000
C	0.97080300	-1.85709600	-1.15965700
C	1.17780000	-3.23193500	-0.93985200
C	0.88691700	-4.20025100	-1.89575300
C	0.35041000	-3.83443000	-3.12816100
C	0.10513100	-2.48838400	-3.37309600
H	0.22829100	-0.49560200	-2.65868700
H	1.59934000	-3.56013600	0.00592300
H	1.08468200	-5.24617600	-1.67820700
H	0.12864700	-4.58488200	-3.88069100
H	-0.31351300	-2.17246700	-4.32490400

propylene

N1_TS

C	0.90288000	-2.37595300	-0.74652500
C	-0.01097800	-1.33334800	-0.92619700
C	-0.82481400	-1.43402100	-2.06829900
C	-0.67455800	-2.45309400	-3.00065700
C	0.29031700	-3.43993400	-2.80898600
C	1.07190200	-3.41375400	-1.66110200
H	-1.58574500	-0.67536100	-2.23113000
H	-1.31024400	-2.47955200	-3.88011000
H	0.42195500	-4.23358700	-3.53690800
H	1.80781900	-4.19347000	-1.49017000
H	1.00863400	-1.50835000	1.12038100
B	-0.15600300	-0.03157200	0.02668800
N	1.66836000	-2.34628100	0.49499100
C	0.95447900	1.16154900	-0.12965700
C	0.99425400	2.24300500	0.79491200
C	1.91373100	1.21395300	-1.18127000
C	2.00618500	3.20439600	0.74216600
C	2.91421300	2.19207800	-1.19107200
C	3.00548800	3.18156500	-0.22107400
C	-1.73372000	0.37403500	0.09114600
C	-2.23890000	1.48126600	-0.64256700
C	-2.69023600	-0.44033900	0.73854300
C	-3.60306300	1.77836900	-0.63143100
C	-4.04781700	-0.10703500	0.73535500
C	-4.52811700	1.01358100	0.07301800
C	-1.37630600	2.37644400	-1.50920000
H	-0.77715700	3.07615600	-0.91965300
H	-0.67370500	1.80520300	-2.12208600
H	-2.01085500	2.96000100	-2.18110300
C	-5.98824600	1.38315900	0.10098700
H	-6.18403000	2.14789900	0.86021900
H	-6.31349400	1.78785600	-0.86129600
H	-6.61151300	0.51683100	0.33641500
C	1.90695700	0.32055800	-2.41112800
H	2.16028500	-0.72365500	-2.21135100
H	0.92876700	0.31165100	-2.89777300
H	2.63076500	0.70048900	-3.13575400
C	4.11809000	4.19630700	-0.22724300
H	4.53238400	4.32510300	-1.23028700
H	3.76760100	5.16940400	0.12679200
H	4.93669300	3.88465700	0.43059300
C	-0.04744400	2.49758800	1.87128800
H	-1.00729700	2.02818000	1.66012600
H	0.29105700	2.14773100	2.85178200
H	-0.21809100	3.57493800	1.95403100
H	-3.95518000	2.63366400	-1.20560600
H	1.99776000	4.00812300	1.47730400
H	3.63505000	2.19153300	-2.00643100
C	1.71796100	-3.65995900	1.16853600
H	2.17146700	-3.53040300	2.14890500
H	0.69975800	-4.03452300	1.28588300
H	2.30270300	-4.37140800	0.57771700
C	3.01859800	-1.75829800	0.36397600
H	3.46718400	-1.71326600	1.35925300
H	3.63564400	-2.37513400	-0.29753700
H	2.92862400	-0.74735700	-0.03543900
H	-4.74888800	-0.75921100	1.25421700
C	-2.33490500	-1.72088300	1.46132500
H	-3.14660000	-2.44561300	1.35559600
H	-1.42258900	-2.17792800	1.07397400
H	-2.19648500	-1.54342300	2.53334900
C	0.44377600	-0.51314600	2.06891200
H	1.27781400	0.18025400	2.20322900
H	-0.48566100	0.03686400	2.21639800
C	1.55607200	-1.69042800	3.98657100
H	2.39985900	-1.00425700	3.96540500
C	0.52913300	-1.55332000	3.14127300
H	-0.29435800	-2.26338900	3.22199200
H	1.57198900	-2.47086000	4.74107600

N1_P1

C	-1.35112500	1.94765400	-0.70528600
C	-0.08371200	1.34986000	-0.66065400
C	0.80861700	1.87931800	-1.61312100
C	0.47807400	2.90706900	-2.48805600
C	-0.79379300	3.47566700	-2.46258400
C	-1.72516800	2.98092100	-1.56165600
H	1.80356000	1.44464300	-1.66749200
H	1.21639600	3.26751000	-3.19789700
H	-1.06027800	4.28329300	-3.13558300
H	-2.72976300	3.39562300	-1.53422600
H	-2.00422300	0.59109400	0.64444400
B	0.32746500	0.14809500	0.44319700
N	-2.42658800	1.41849800	0.18750100
C	-0.85845800	-1.03378500	0.22473200
C	-1.50453500	-1.71396800	1.29009900
C	-1.32048800	-1.37504000	-1.07966900
C	-2.64564800	-2.49981500	1.07082100

C	-2.42961000	-2.20109500	-1.26972100
C	-3.15110400	-2.73176900	-0.20162800
C	1.88262100	-0.41896000	0.21684000
C	2.22962300	-1.76359300	-0.10493800
C	2.99274900	0.46902700	0.32983300
C	3.55511600	-2.12451400	-0.38766600
C	4.29826700	0.07290200	0.03132700
C	4.60795900	-1.22499600	-0.35030500
C	1.27430000	-2.94488800	-0.13656700
H	0.64807500	-2.99933000	0.75406400
H	0.60372800	-2.93766400	-0.99890500
H	1.85322100	-3.87057300	-0.18523300
C	6.02367900	-1.64455500	-0.64941300
H	6.04693600	-2.55141200	-1.25915600
H	6.56444500	-0.85891600	-1.18450500
H	6.57750600	-1.85185800	0.27274200
C	-0.58335000	-0.95655600	-2.33766400
H	-0.96505100	-0.02240100	-2.76576800
H	0.48203100	-0.81412400	-2.14336000
H	-0.69286900	-1.73672500	-3.09685800
C	-4.38688100	-3.56334600	-0.42853900
H	-5.24383400	-2.93735800	-0.70143600
H	-4.23771100	-4.27828400	-1.24255600
H	-4.65834300	-4.12290500	0.46976200
C	-0.91667300	-1.79307400	2.68796500
H	0.15762100	-1.60426000	2.67035400
H	-1.36809000	-1.09618400	3.40103100
H	-1.07367800	-2.80237700	3.07919400
H	3.76663900	-3.16122100	-0.64362200
H	-3.11462600	-2.98929200	1.92371400
H	-2.73356800	-2.44617800	-2.28739700
C	-2.87840100	2.37277300	1.24062500
H	-3.62132800	1.86788800	1.86073200
H	-2.02599700	2.68082400	1.84375000
H	-3.32801000	3.23705500	0.75055900
C	-3.60140100	0.90227600	-0.57864000
H	-4.18768900	0.26538700	0.08638500
H	-4.19624800	1.74958200	-0.91986500
H	-3.23568100	0.31856400	-1.41964000
H	5.09981100	0.80531700	0.11541500
C	2.87542100	1.88759900	0.84050800
H	3.78425900	2.44981300	0.60845800
H	2.03463400	2.43626900	0.41971800
H	2.75140400	1.90009100	1.92705000
C	0.12437300	0.88583900	1.95965100
H	-0.88291600	0.63791800	2.33326500
H	0.78910500	0.41798900	2.69569800
C	0.40023800	2.99622400	3.32345900
H	0.49112500	2.43117200	4.24867500
C	0.24564600	2.38517800	2.14816400
H	0.17344500	3.01963200	1.26201500
H	0.46673800	4.07732500	3.40047800

N2_TS2

C	-0.56738800	2.62283800	-0.34759700
C	0.26542700	1.57115100	-0.73556900
C	1.04049500	1.80997800	-1.88371500
C	0.96272000	3.00158900	-2.59653600
C	0.10207400	4.01533500	-2.18051200
C	-0.66531300	3.82813000	-1.03847500
H	1.72768700	1.03830800	-2.21819100
H	1.57685300	3.14197700	-3.48083000
H	0.03266400	4.94440400	-2.73662000
H	-1.33887000	4.61044300	-0.69998300
H	-0.73129700	1.33567300	1.30325500
B	0.24215600	0.13608700	0.02465400
N	-1.34732800	2.38871700	0.86408100
C	-1.07243800	-0.70859600	-0.39857400
C	-2.13991800	-0.92471500	0.48179700
C	-1.21046000	-1.25201600	-1.69463500
C	-3.29868300	-1.62795500	0.15371000
C	-2.36392300	-1.96766300	-2.02537000
C	-3.41438700	-2.16866800	-1.13292000
C	1.68508800	-0.58737600	0.10172200
C	1.89508100	-1.97770700	0.24581200
C	2.83484800	0.22124600	0.10788200
C	3.20223300	-2.46627700	0.33952200
C	4.13674100	-0.26228000	0.21061300
C	4.32637300	-1.64647000	0.31724100
C	5.71103600	-2.22942000	0.42074900
H	6.24350400	-1.84043100	1.29592700
H	5.67446500	-3.31777200	0.50354700
H	6.31677500	-1.97630800	-0.45660600
C	-4.63717400	-2.94994400	-1.53683500
H	-4.55330400	-3.30686200	-2.56564400
H	-4.78717300	-3.82064100	-0.88870800
H	-5.54522100	-2.34065700	-1.46412700
C	-1.18188100	3.46636400	1.85669600

H	-1.78669900	3.23227600	2.73332900
H	-0.12868400	3.52930700	2.13837900
H	-1.50476400	4.42745000	1.44691700
C	-2.77977000	2.15642000	0.57949900
H	-3.25541100	1.75992300	1.48054700
H	-3.25592000	3.09995200	0.28853800
H	-2.86874200	1.42936800	-0.22574000
C	0.77601300	-2.99210800	0.32369200
H	-0.00677500	-2.69249500	1.02611700
H	0.28137800	-3.13241400	-0.64241900
H	1.17075300	-3.95930800	0.64515800
C	-0.13603200	-1.11144400	-2.74624600
H	-0.17934400	-0.12938200	-3.22920000
H	0.86389600	-1.22045400	-2.31480600
H	-0.25716900	-1.86996500	-3.52413100
C	-4.39819400	-1.80000500	1.16986200
H	-5.34634400	-1.38125600	0.81314200
H	-4.58356000	-2.85761100	1.38891300
H	-4.14106600	-1.30237700	2.10899700
C	5.31429300	0.67773000	0.20959300
H	5.89774600	0.59189600	1.13332600
H	5.99916000	0.46273400	-0.61842900
H	4.98604100	1.71512200	0.11371100
H	3.34837200	-3.54023500	0.44384200
H	2.70922600	1.30036000	0.03649600
H	-2.44407900	-2.39182200	-3.02532300
H	-2.08091800	-0.51701500	1.48588800
C	0.11471500	0.41627200	2.09206000
H	1.10571700	0.86116400	2.21600100
C	-1.57577700	-0.08728100	3.91861100
H	-1.74439600	-1.10118500	3.56333200
C	-0.71388000	0.73082200	3.30905400
H	-0.56591000	1.71642700	3.74997000
H	-2.11493200	0.21980000	4.80873400
H	0.23534300	-0.66755700	2.06806600

N2_P2

C	-1.06070400	2.30672100	-0.43032600
C	0.14534600	1.58867000	-0.36512100
C	1.12989800	2.11999200	-1.22620400
C	0.94514300	3.23813300	-2.02974000
C	-0.27418300	3.90891500	-2.03555200
C	-1.29251000	3.42590400	-1.22962100
H	2.08124200	1.60444100	-1.28933200
H	1.75510300	3.57799000	-2.66805600
H	-0.43682800	4.78015000	-2.66081400
H	-2.26057400	3.91939300	-1.23201700
H	-1.90419200	1.09195200	0.97790500
B	0.36745300	0.14202500	0.48225000
N	-2.24463700	1.84414500	0.35800500
C	-0.87232500	-0.81539500	-0.03638600
C	-1.83012400	-1.32046400	0.85665500
C	-1.07863200	-1.12908900	-1.40204900
C	-2.94420100	-2.07912900	0.48165000
C	-2.17477600	-1.90842600	-1.78204800
C	-3.12182700	-2.38365800	-0.87304500
C	1.88256500	-0.48220500	0.24506200
C	2.24434400	-1.81498100	-0.04999100
C	2.96025700	0.38772500	0.50581900
C	3.60059000	-2.16359000	-0.13771400
C	4.30657100	0.04931800	0.42402600
C	4.63949100	-1.26721600	0.07454600
C	6.07775500	-1.70018700	-0.03634300
H	6.61195300	-1.56598800	0.91131900
H	6.14936800	-2.75382200	-0.31604800
H	6.61706600	-1.11393700	-0.78914800
C	-4.29101100	-3.21435000	-1.33423800
H	-4.27282700	-3.35051400	-2.41774500
H	-4.28255300	-4.20694100	-0.87024300
H	-5.24760100	-2.74999500	-1.06786800
C	-2.83326100	2.90307400	1.22769800
H	-3.49337600	2.42181700	1.94930800
H	-2.03446600	3.42936400	1.74380200
H	-3.39818100	3.59565000	0.60507100
C	-3.31230300	1.24814100	-0.50372200
H	-4.08331200	0.83128500	0.14636100
H	-3.72299700	2.04152200	-1.12863800
H	-2.87245600	0.46183500	-1.11386600
C	1.25668000	-2.94577900	-0.24849600
H	0.46009900	-2.93014900	0.49762200
H	0.77151800	-2.90929000	-1.22924800
H	1.77459700	-3.90600800	-0.17451000
C	-0.14263500	-0.65101000	-2.48797300
H	-0.39006400	0.36770300	-2.81040800
H	0.89396800	-0.63338500	-2.14175800
H	-0.20715000	-1.30233300	-3.36443000
C	-3.92412200	-2.56922800	1.51669000
H	-4.94353500	-2.22487500	1.30553100

H	-3.96292900	-3.66394500	1.54491800
H	-3.64750700	-2.21800300	2.51378000
C	5.38211100	1.06414400	0.71405900
H	6.01899500	0.74806000	1.54829200
H	6.04224000	1.21021800	-0.14882600
H	4.94692400	2.03262600	0.97206000
H	3.85234000	-3.19717300	-0.37225000
H	2.73051700	1.41276900	0.80103500
H	-2.29995900	-2.15480700	-2.83610600
H	-1.70417800	-1.12325800	1.91894100
C	0.34709600	0.35456000	2.15849100
H	1.35129600	0.71131100	2.41196400
C	-1.63058800	1.00240200	3.62326300
H	-1.86419300	-0.02469300	3.89388000
C	-0.59794100	1.29433300	2.82288100
H	-0.39467000	2.35221900	2.62806800
H	-2.23960000	1.77809400	4.07993900
H	0.26088800	-0.64236600	2.61059700

N3_TS

C	-1.02259100	2.75563200	0.44185900
C	-0.15843200	1.71466400	0.77616700
C	0.45324500	1.77981500	2.03475500
C	0.21349300	2.84212000	2.89935400
C	-0.64382000	3.87615600	2.52563500
C	-1.26782400	3.83681900	1.28454900
H	1.12714600	0.98411600	2.33903900
H	0.70001200	2.86873000	3.86927000
H	-0.82714500	4.70647700	3.19967200
H	-1.94321100	4.63260000	0.98139900
H	-0.78716400	1.50314000	-1.36528400
B	0.04373900	0.47104200	-0.23619600
N	-1.66096500	2.62509500	-0.85512200
C	1.53440000	-0.14350800	-0.11460800
C	1.84516200	-1.49956100	-0.04827100
C	2.63828200	0.70938600	-0.09512900
C	3.14399800	-1.98126800	0.06397300
C	3.95194100	0.27105900	0.00269800
C	4.20555000	-1.08996400	0.09096000
C	-1.19995300	-0.56454400	-0.06448600
C	-1.26137600	-1.31200600	1.11172700
C	-2.29112200	-0.73243700	-0.90678000
C	-2.29612100	-2.17784800	1.42613200
C	-3.35655500	-1.58464100	-0.63439300
C	-3.35844700	-2.31576500	0.54119700
C	-3.10751800	2.38136400	-0.73642900
H	-3.51138600	2.10911900	-1.71334400
H	-3.27474400	1.56247800	-0.03540600
H	-3.62087500	3.27680400	-0.36350900
C	-1.39296700	3.76497400	-1.74178900
H	-1.79345300	3.54550100	-2.73613400
H	-1.86225300	4.68496200	-1.37299400
H	-0.31423200	3.92057600	-1.81037300
F	-4.37211800	-1.68984100	-1.49129300
F	-4.36565500	-3.13578700	0.82391800
F	-2.28781100	-2.86859900	2.56365500
F	-0.26263100	-1.21689400	2.00004800
F	-2.39475000	-0.03559500	-2.06055200
F	0.88701000	-2.42845400	-0.11890300
F	3.37730300	-3.29104900	0.13134300
F	5.45452300	-1.53335400	0.19165000
F	4.96314200	1.13866900	0.01024900
F	2.46718400	2.03555100	-0.22236200
C	0.17007100	1.00105800	-2.12784100
H	1.09909700	1.56736800	-2.04567000
H	-0.46328400	1.58346600	-2.81991300
C	1.53726500	-0.80348800	-3.18855000
H	2.46280000	-0.27464000	-2.97180800
C	0.35518200	-0.31973800	-2.81956500
H	-0.54899100	-0.87308400	-3.05313500
H	1.61882000	-1.74999400	-3.71175500

N3_P

C	1.09629700	2.70345600	-0.51618900
C	0.41513700	1.50938600	-0.71768200
C	0.16428600	1.21711500	-2.07407200
C	0.53855100	2.06397200	-3.10618000
C	1.19755700	3.26472100	-2.83387700
C	1.48143000	3.59256300	-1.51832400
H	-0.34047900	0.28311400	-2.30775200
H	0.31675900	1.79237900	-4.13340100
H	1.48837100	3.93306600	-3.63672000
H	1.99239900	4.52235900	-1.28418300
H	1.20468900	2.19948100	1.40813500
B	-0.00807200	0.35954900	0.40515900
N	1.36912800	3.07610900	0.89350600
C	-1.52999800	-0.14075200	0.06679600
C	-2.00300000	-1.43425500	0.26901500

C	-2.51793800	0.79185800	-0.23471000
C	-3.33215400	-1.80023700	0.10501100
C	-3.86007400	0.47631200	-0.40559700
C	-4.26869000	-0.83807500	-0.24326200
C	1.18805900	-0.75110700	0.16121200
C	1.14219700	-1.79751800	-0.75810200
C	2.43798000	-0.57867900	0.74512800
C	2.20420700	-2.66059200	-1.00016800
C	3.53229300	-1.40649900	0.53883400
C	3.40871400	-2.47282400	-0.33774800
C	2.77298000	3.48127700	1.17096400
H	2.89611500	3.57695700	2.25072100
H	3.43133500	2.70603300	0.78410900
H	2.97480300	4.43734800	0.68972200
C	0.37277900	4.06996900	1.38720800
H	0.48836000	4.18773500	2.46603400
H	0.55159800	5.01639200	0.87522400
H	-0.61953900	3.69341200	1.13909800
F	4.69417300	-1.16838800	1.15164300
F	4.43880100	-3.28540600	-0.56246900
F	2.08505100	-3.65246100	-1.88160600
F	0.05062900	-2.00211100	-1.51084900
F	2.67468400	0.50036600	1.54384100
F	-1.17307200	-2.40272000	0.67992700
F	-3.72208000	-3.06061000	0.30132800
F	-5.54960500	-1.16992300	-0.39892400
F	-4.75298900	1.42271600	-0.70612900
F	-2.20480200	2.10247400	-0.33852500
C	-0.15202900	0.77411600	2.03025100
H	-0.92502500	1.54971100	2.13354500
H	0.75576600	1.14451300	2.53521500
C	-1.76239800	-0.56563800	3.42044800
H	-2.53433400	0.19461100	3.32166700
C	-0.56755100	-0.40894000	2.85718200
H	0.17435000	-1.20156200	2.95719600
H	-2.01762100	-1.45926000	3.98008400

N4_TS

C	-2.77615600	-0.35985200	-0.09043700
C	-1.69047600	-0.71201300	-0.88712400
C	-1.68907500	-2.01406000	-1.40636900
C	-2.72037000	-2.90506900	-1.13315800
C	-3.79941600	-2.51174000	-0.34048800
C	-3.83220600	-1.22588400	0.18433500
H	-0.86209400	-2.32894600	-2.03820200
H	-2.69102100	-3.90965600	-1.54366700
H	-4.60867200	-3.20437400	-0.13364300
H	-4.66426200	-0.90509100	0.80598400
H	-1.52986000	1.37818700	-0.28350100
B	-0.50130900	0.29881500	-1.26882800
N	-2.72021900	0.98912200	0.44573100
C	0.92230100	-0.00632600	-0.57871400
C	2.11236900	0.27647700	-1.24415000
C	1.07484200	-0.58618500	0.67584900
C	3.36769100	-0.01234000	-0.72414100
C	2.30611800	-0.89080500	1.23897900
C	3.46266000	-0.60904800	0.52538600
C	-3.79814200	1.85257700	-0.05312700
H	-3.59716500	2.88211500	0.25806600
H	-3.82294200	1.79910900	-1.14336200
H	-4.77429400	1.54214000	0.33928400
C	-2.66955100	1.00288600	1.91657200
H	-2.49195100	2.02444600	2.25786600
H	-3.61336300	0.63528500	2.33865700
H	-1.85468300	0.35939900	2.24793300
F	2.08073400	0.87602800	-2.44222500
F	4.47610000	0.27595400	-1.40546500
F	4.65435000	-0.89294100	1.04336900
F	2.38774300	-1.44102800	2.45047100
F	-0.00533400	-0.83134300	1.44057900
H	-0.41220300	0.36289400	-2.46313100
C	-0.74637900	2.20100600	-0.97013000
H	-1.56234800	2.67136000	-1.53284700
C	0.04420000	2.63007100	1.39541400
H	0.02906700	1.57457000	1.65080600
C	-0.32362100	3.06420100	0.19327600
H	-0.27774200	4.13328600	-0.01432300
H	0.38305800	3.31892600	2.16226300
H	0.08747500	2.20501600	-1.67653800

N4_P

C	2.56767000	-0.45517000	-0.28312600
C	1.59806900	-1.07768700	0.50060800
C	1.64711800	-2.48465600	0.43657200
C	2.57566100	-3.18296900	-0.32413300
C	3.53356400	-2.49598400	-1.06879000
C	3.53220700	-1.10929500	-1.04661800
H	0.91622800	-3.03540200	1.02139800

H	2.56112900	-4.26844100	-0.33344500
H	4.27165700	-3.03086200	-1.65658200
H	4.27049100	-0.55489900	-1.61960700
H	1.69504300	1.33412300	0.12402600
B	0.41836600	-0.41275800	1.44869800
N	2.60254700	1.02935900	-0.26838000
C	-0.96375800	-0.23146300	0.57377100
C	-2.19713700	-0.61954600	1.09486100
C	-1.03332800	0.27025200	-0.71790500
C	-3.39432600	-0.52600600	0.39262200
C	-2.19679700	0.38846900	-1.46442200
C	-3.39493800	-0.02233500	-0.89994600
C	3.65873500	1.53249700	0.65301300
H	3.60599700	2.62148600	0.69160100
H	3.47767500	1.10541000	1.63878500
H	4.62554400	1.20184800	0.27227400
C	2.71445000	1.66861200	-1.61032100
H	2.41400000	2.71262700	-1.50640400
H	3.74804100	1.61101600	-1.95039200
H	2.05230000	1.14956900	-2.29825200
F	-2.28539700	-1.10480600	2.33884800
F	-4.54378100	-0.91405500	0.94730000
F	-4.53310600	0.07936300	-1.58544000
F	-2.17702700	0.90143400	-2.69809800
F	0.09411100	0.74134300	-1.31689600
H	0.18237100	-1.28782100	2.25115500
C	0.76862700	0.90458800	2.40346100
H	1.63114900	0.64664700	3.03262000
C	0.25708100	2.95321800	0.95946600
H	-0.55303400	2.46968100	0.42320200
C	0.94686700	2.32259000	1.91826300
H	1.71125300	2.91433800	2.43042400
H	0.44357200	3.99877100	0.73095900
H	-0.07485200	0.92998600	3.10774500

N5-TS

B	0.41784000	-0.06511700	0.06475900
C	-0.57313100	-0.39694800	1.29129400
C	0.05192800	-0.58558900	2.53505000
H	1.13866700	-0.59388600	2.57448300
C	-0.65928800	-0.72314400	3.71699700
H	-0.13552200	-0.85767000	4.65789100
C	-2.04891600	-0.66990700	3.68514700
H	-2.62746400	-0.75222700	4.59951000
C	-2.70082000	-0.52126000	2.46887100
H	-3.78113500	-0.49625100	2.45357500
C	-1.97775000	-0.40934300	1.27440700
N	-2.65746400	-0.36272400	-0.02456200
C	-3.46372200	-1.63633900	-0.32835200
C	-3.97293300	-1.54380200	-1.77479800
H	-3.11025700	-1.55752300	-2.44872700
H	-4.54906800	-2.45165900	-1.98147900
C	-4.79361900	-0.29264500	-2.04322900
H	-5.69346300	-0.27074900	-1.41675900
H	-5.13602600	-0.27900500	-3.08240800
C	-3.91034300	0.91522800	-1.76955600
H	-4.44691600	1.85330100	-1.94800600
H	-3.06747500	0.89686200	-2.47245500
C	-3.36419800	0.96383700	-0.33331100
C	-4.49874600	1.35458100	0.63092800
H	-5.37007900	0.70377300	0.59115700
H	-4.83793500	2.35378200	0.34257900
H	-4.14497600	1.42274500	1.66040800
C	-2.34494100	2.09917300	-0.24689700
H	-1.51155700	1.96380200	-0.93613800
H	-1.95809900	2.19975900	0.76766500
H	-2.84438500	3.03442000	-0.51476300
C	-4.64941700	-1.94642300	0.61067100
H	-4.30356400	-2.31027900	1.57892200
H	-5.21562700	-2.76069300	0.14904200
H	-5.34304400	-1.12500100	0.77297900
C	-2.53560800	-2.84563500	-0.22825200
H	-1.74545100	-2.80990500	-0.97418200
H	-3.12904400	-3.74597600	-0.41338800
H	-2.08599200	-2.93157700	0.76307200
C	0.85586100	1.51809200	-0.00960900
C	0.53768800	2.47720800	0.95398900
F	-0.17566700	2.17752600	2.04889700
C	0.88588500	3.82161800	0.85581100
F	0.51601400	4.67842700	1.80629600
C	1.61678800	4.26953900	-0.22991800
F	1.95471500	5.54942300	-0.33857500
C	1.99541000	3.35555100	-1.20212700
F	2.72705600	3.75098100	-2.24176800
C	1.62631800	2.02824900	-1.06023900
F	2.10357200	1.19915000	-2.00789600
C	1.69878100	-1.08566600	0.05437200
C	3.04715100	-0.71899400	0.10748500

F	3.43486100	0.56101200	0.15555500
C	4.09348000	-1.63601700	0.15420500
F	5.35390900	-1.21026900	0.20488100
C	3.82500000	-2.99429600	0.17065900
F	4.81543900	-3.87770600	0.21774700
C	2.50423400	-3.41481600	0.15015100
F	2.21420300	-4.71428000	0.18607100
C	1.49434000	-2.46526900	0.10974000
F	0.24564900	-2.95449300	0.12102300
H	-1.43521700	-0.39229500	-0.81481300
C	-0.43169100	-0.35389400	-1.72975000
H	-0.97348000	0.43019900	-2.27626100
H	0.59018100	0.00879500	-1.84142800
C	-0.94037700	-1.76718300	-3.73977900
H	-1.46415800	-0.94950400	-4.23075900
C	-0.41160500	-1.62691500	-2.52577200
H	0.11655400	-2.46890400	-2.08539400
H	-0.86473300	-2.70156400	-4.28538700

N5_P

B	-0.45325200	-0.08524100	0.25744900
C	0.57225600	-0.30469600	-1.04980600
C	-0.11759100	-0.46779400	-2.27168700
H	-1.20026900	-0.54250300	-2.23119000
C	0.48860000	-0.48614200	-3.51548400
H	-0.11257900	-0.60245300	-4.41162100
C	1.86729300	-0.32341700	-3.60860500
H	2.36815300	-0.30398700	-4.57052400
C	2.60347200	-0.19403100	-2.44321800
H	3.67621300	-0.08336800	-2.51117100
C	1.96456800	-0.21709500	-1.19795600
N	2.84296700	-0.16652400	0.01064800
C	3.77321100	-1.42295200	0.18143500
C	4.41405200	-1.32118100	1.57403500
H	3.63203400	-1.48338900	2.32313800
H	5.11574800	-2.15592800	1.66567600
C	5.10162100	0.00830500	1.85497300
H	5.94952700	0.16433300	1.17813600
H	5.51102500	0.00152800	2.86911400
C	4.08520600	1.13617300	1.71751100
H	4.53514800	2.10794200	1.94426500
H	3.27566500	0.98561100	2.44595000
C	3.47225300	1.23308900	0.31558400
C	4.51352200	1.70188700	-0.70210400
H	5.42966000	1.11585100	-0.72212300
H	4.79126600	2.71690500	-0.40420700
H	4.09608000	1.77004100	-1.70646100
C	2.34520400	2.26269900	0.30379400
H	1.49499800	1.96711200	0.91894000
H	1.98494200	2.44443300	-0.70836400
H	2.74266300	3.19868600	0.70572000
C	4.84392800	-1.58889800	-0.90097500
H	4.40885900	-1.89124500	-1.85289800
H	5.48632600	-2.40866300	-0.56811000
H	5.48266700	-0.72252800	-1.05695300
C	2.87538600	-2.65612000	0.13807600
H	2.12092900	-2.63017100	0.92418500
H	3.50954700	-3.53232200	0.30190500
H	2.37668900	-2.76450200	-0.82665100
C	-1.08956800	1.46523600	0.13861300
C	-0.74297500	2.45480500	-0.78340100
F	0.14249600	2.23767900	-1.77790400
C	-1.24391300	3.75533200	-0.77391600
F	-0.83560600	4.63626600	-1.69057100
C	-2.16718400	4.13242700	0.18215500
F	-2.66291200	5.36767400	0.20413100
C	-2.57219700	3.18973600	1.11492800
F	-3.47688700	3.51537000	2.03852400
C	-2.03978400	1.91049400	1.06777300
F	-2.54635300	1.06657200	1.98488500
C	-1.64396100	-1.23911400	0.07466400
C	-3.01691400	-1.05156000	-0.09425300
F	-3.56235800	0.16815000	-0.19809800
C	-3.93512000	-2.09209700	-0.22287500
F	-5.23292500	-1.82531500	-0.37277500
C	-3.50029500	-3.40574700	-0.22216300
F	-4.36424600	-4.41035700	-0.35032000
C	-2.14163500	-3.65360900	-0.10508600
F	-1.68350100	-4.90691800	-0.13375500
C	-1.27001500	-2.58245700	0.02274700
F	0.03665000	-2.91516600	0.07738600
H	2.17373000	-0.29007900	0.78620000
C	0.09481200	-0.17173300	1.83931400
H	0.81618900	0.62015900	2.09546200
H	-0.79674300	0.11124400	2.40510300
C	1.48076700	-1.50267300	3.46218300
H	1.99867200	-0.61056900	3.81310300
C	0.55276300	-1.43636000	2.50431900

H	0.03374200	-2.35435600	2.23728600
H	1.72882100	-2.43654600	3.95732300

N6_TS

C	-1.48274600	1.44904200	-0.17323100
C	-1.34119100	0.08497600	0.09720900
C	-2.44457800	-0.71180700	0.42364400
C	-3.71606400	-0.15062000	0.45903400
C	-3.88489900	1.20057300	0.16380200
C	-2.77940800	1.98254200	-0.14452300
H	-2.32139500	-1.76198500	0.65718600
H	-4.56996000	-0.76942400	0.71530400
H	-4.87765500	1.64026900	0.18096000
H	-2.91321000	3.03890800	-0.36252300
C	0.68221600	-0.75824800	1.31358200
C	0.20046800	-1.39520300	-1.18278300
C	2.13279800	-1.20136400	1.06004400
C	1.68285000	-1.79285800	-1.29621300
C	2.26091000	-2.30271400	0.01699800
H	2.55699600	-1.51217800	2.02081400
H	2.71470600	-0.33091300	0.73218800
H	1.76299900	-2.54843400	-2.08458000
H	2.27189400	-0.93306700	-1.63607900
H	3.31178300	-2.57810100	-0.11445400
H	1.73724100	-3.20959600	0.34267300
N	0.02505700	-0.42272300	-0.01920000
C	-0.04876200	-1.82774300	2.14047000
H	-1.04927400	-1.49475400	2.42006400
H	-0.12416800	-2.79561000	1.64378300
H	0.51385900	-1.98114400	3.06621600
C	0.72094300	0.52039500	2.15907600
H	1.17878500	0.28466900	3.12432500
H	1.31646400	1.30490600	1.68462600
H	-0.28307700	0.91169300	2.34110000
C	-0.64726700	-2.68008600	-1.10631300
H	-1.70892300	-2.46490700	-1.22823600
H	-0.35049000	-3.32482600	-1.93902300
H	-0.50729700	-3.24953600	-0.18773300
C	-0.21705800	-0.67679200	-2.47172800
H	0.38284200	0.21575800	-2.65602900
H	-0.07169100	-1.35940700	-3.31420900
H	-1.26971700	-0.38650900	-2.44364300
B	-0.24513500	2.44240700	-0.35851400
H	0.61377300	0.88044400	-0.42485300
H	0.05406700	2.93670600	0.69748500
H	-0.40253200	3.24660400	-1.23887200
C	1.46151800	1.82095400	-0.98644000
H	2.07737400	0.91332100	-0.94950000
H	1.28967800	2.00362000	-2.04824100
C	3.44430000	2.69458200	0.27756800
H	3.86796100	1.69893300	0.38778600
C	2.29915500	2.90176200	-0.36997700
H	1.91086400	3.91326400	-0.46231200
H	3.99926800	3.51398300	0.72144300

N6_P

C	-1.00582300	1.73705300	-0.07435900
C	-1.28520100	0.37475700	0.08964100
C	-2.56301500	-0.15358400	0.29448500
C	-3.65069300	0.70693500	0.31627200
C	-3.43830800	2.07281100	0.12702600
C	-2.15256100	2.55920500	-0.05907500
H	-2.71729000	-1.21561800	0.44129500
H	-4.64909300	0.31328800	0.47458900
H	-4.28225600	2.75629200	0.13100200
H	-1.99853900	3.62619100	-0.19106300
C	0.36727400	-1.14024200	1.34850000
C	-0.08928900	-1.41702600	-1.27697600
C	1.69482000	-1.86855300	1.08787100
C	1.28989500	-2.08743100	-1.34596300
C	1.65038800	-2.84904000	-0.07644500
H	1.97849900	-2.36770500	2.01992500
H	2.46957500	-1.11591000	0.88736000
H	1.28871700	-2.74602200	-2.22012300
H	2.05082600	-1.31788600	-1.53179500
H	2.62717300	-3.32632500	-0.19448000
H	0.93016300	-3.65347600	0.11362500
N	-0.11021900	-0.53581000	0.00101700
C	-0.66167300	-2.06145400	1.99906100
H	-1.57664700	-1.52057400	2.24237900
H	-0.91098500	-2.94391600	1.40957600
H	-0.22875000	-2.41071200	2.94056400
C	0.63259500	0.04041300	2.28407700
H	1.05483500	-0.35407900	3.21267000
H	1.34113700	0.75282800	1.85165900
H	-0.28523800	0.58389100	2.51822600
C	-1.20156600	-2.46498000	-1.33459100
H	-2.18438500	-2.00207900	-1.41801600

H	-1.03838700	-3.04818500	-2.24545500
H	-1.20255600	-3.16280600	-0.49820200
C	-0.25654100	-0.47305200	-2.46928600
H	0.48557200	0.32840100	-2.45498200
H	-0.11713200	-1.05569400	-3.38431000
H	-1.25094000	-0.02328400	-2.48930600
B	0.45319200	2.49987800	-0.08434300
H	0.63577400	0.15201700	-0.19504700
H	0.68477000	2.71418800	1.09633400
H	0.28416400	3.57020700	-0.63745900
C	1.82429800	1.84622700	-0.79202500
H	2.12459400	0.79728900	-0.59695700
H	1.73962600	1.92518600	-1.88621500
C	4.07290600	2.21288200	0.28720500
H	4.21041600	1.14936700	0.47729500
C	2.98890000	2.66964800	-0.33799200
H	2.88652200	3.74302600	-0.50047000
H	4.85697800	2.87596900	0.63743500

N8_TS

C	-3.85241200	3.79175300	-1.00536200
H	-3.78329300	4.18046000	-2.02517200
H	-4.04299400	4.64890500	-0.35162300
C	-4.97300300	2.71825100	-0.86600700
H	-5.79486700	3.06299600	-0.23171400
H	-5.40741200	2.46870100	-1.83924100
C	-4.75705800	0.27709800	0.08829600
H	-5.80339200	0.00919500	-0.01970500
C	-3.84834000	-0.64562700	0.65875200
H	-4.23445100	-1.59208800	1.02571300
C	-2.50545100	-0.36617100	0.80120200
C	-0.54406800	1.20240500	0.21064600
C	-0.28789700	2.50195000	-0.21651500
H	0.74919800	2.80207800	-0.32684100
C	-1.27009300	3.45426200	-0.59561700
H	-0.95863700	4.44084300	-0.92694500
C	-4.27974800	1.51086500	-0.26998400
C	-1.93991600	0.85711400	0.33631900
C	-2.58361000	3.07662600	-0.59254800
C	-2.90043600	1.77887500	-0.13534100
C	-2.07205600	-1.09747900	3.02896600
H	-1.95996100	-0.04620800	3.29909400
H	-1.42080700	-1.71486800	3.64611900
H	-3.11665000	-1.39439800	3.16069300
C	-1.75668700	-2.69172200	1.21987900
H	-2.75090800	-3.09253000	1.44256000
H	-1.01624100	-3.23766200	1.80505700
H	-1.53923100	-2.79336900	0.15784500
C	0.53068900	-1.19984100	-0.69904800
C	1.43791800	-2.28469600	-0.51600000
C	1.17934000	-3.54050200	-1.06573400
H	1.87959900	-4.35139200	-0.87106600
C	0.09119500	-3.77342500	-1.90412100
C	-0.68108900	-2.67153100	-2.24287000
H	-1.47081500	-2.78668700	-2.98426900
C	-0.47374700	-1.40264300	-1.68132800
C	2.81343100	-2.13509900	0.10813100
H	3.16832900	-3.09559200	0.49280000
H	2.88594100	-1.39829600	0.90199700
H	3.51709000	-1.81371200	-0.66916900
C	-0.19424900	-5.14517900	-2.45669200
H	0.73154700	-5.68362400	-2.67680600
H	-0.78339700	-5.08785400	-3.37530100
H	-0.75952000	-5.74921800	-1.73852800
C	-1.31921000	-0.31317700	-2.32182600
H	-1.31951800	-0.47751700	-3.40400800
H	-0.94426700	0.69261900	-2.14166900
H	-2.36263800	-0.34845700	-1.98951100
C	2.15485100	0.93650100	0.00429700
C	2.87197600	0.86197100	-1.22091200
C	4.14597000	1.42087500	-1.33748900
H	4.66991100	1.33058300	-2.28771400
C	4.75858400	2.10950800	-0.29472600
C	4.01065100	2.29686000	0.85799800
H	4.41966000	2.90329000	1.66507900
C	2.73147100	1.75173200	1.01051000
C	2.31047200	0.25858700	-2.49424400
H	1.26404200	0.53391100	-2.64338700
H	2.35925200	-0.83387700	-2.51242200
H	2.87694100	0.63343300	-3.35085300
C	6.15515300	2.65727900	-0.43203400
H	6.90170800	1.87200200	-0.27284000
H	6.34445200	3.44666200	0.29971900
H	6.32319100	3.06909900	-1.43109100
C	2.01107000	2.15526700	2.28500900
H	2.21821800	3.20885400	2.49438600
H	2.35206700	1.57986400	3.15242400
H	0.93062100	2.03813400	2.20565800

B	0.71288900	0.16589100	0.18718700
N	-1.67948600	-1.26326400	1.60509400
H	-0.45324300	-0.88344600	1.58151300
C	0.84166200	-0.63632500	2.17880800
H	1.82604400	-0.22936100	1.96527100
C	0.97078000	-2.41265800	3.97030800
H	0.73198300	-1.70461400	4.76154300
C	1.06314500	-2.02909400	2.69421900
H	1.32114300	-2.78829000	1.95712300
H	1.15402300	-3.44013100	4.26882400
H	0.45352100	-0.00069900	2.98520700

N8_P

C	4.06376100	-3.10415400	-1.73215200
H	4.23419400	-3.09349700	-2.81343000
H	4.04807900	-4.15344500	-1.42447000
C	5.16214900	-2.29522600	-0.98192500
H	5.61627500	-2.89109300	-0.18380900
H	5.97328700	-1.98351400	-1.64577200
C	4.90568500	0.00682100	0.24497900
H	5.96279200	0.16548200	0.42999000
C	3.96535800	1.00074500	0.61277500
H	4.33006100	1.93064200	1.03681800
C	2.61974600	0.81523800	0.42410800
C	0.64002500	-0.71235600	-0.32397700
C	0.46192200	-1.80172100	-1.17941800
H	-0.55770500	-2.09489500	-1.40426500
C	1.46762000	-2.63068200	-1.72882300
H	1.18254300	-3.46082100	-2.36963200
C	4.43275100	-1.10475600	-0.39533700
C	2.03735000	-0.36363800	-0.14598000
C	2.77140600	-2.39734800	-1.38640500
C	3.04003900	-1.25472200	-0.61029000
C	2.04381200	2.88615700	1.74330400
H	2.22496800	2.28358200	2.62993800
H	1.18128900	3.54107900	1.87923600
H	2.91889800	3.48392000	1.49027900
C	1.67630900	2.75473100	-0.67244700
H	2.66809900	3.17805700	-0.83419600
H	0.91098900	3.52635300	-0.58080800
H	1.41832400	2.06555200	-1.47354800
C	-1.10856600	1.42824100	-0.08300900
C	-1.35236400	2.60076400	0.69009200
C	-1.57587500	3.85143700	0.09036900
H	-1.76133200	4.71213600	0.73078800
C	-1.60914700	4.02428100	-1.28621600
C	-1.44060900	2.87922800	-2.05795800
H	-1.51342400	2.95956500	-3.14162800
C	-1.20914800	1.62217700	-1.49470500
C	-1.50408000	2.61094000	2.19957400
H	-0.63469100	2.23177600	2.73347000
H	-2.35652400	2.00094600	2.51250300
H	-1.68672200	3.63046900	2.54880000
C	-1.86909800	5.36715300	-1.91795600
H	-1.11497400	5.60399500	-2.67454000
H	-1.86451400	6.16444000	-1.17094700
H	-2.84306200	5.38197000	-2.41698000
C	-1.17116500	0.48002900	-2.48581700
H	-1.66026900	0.78351300	-3.41511700
H	-1.69596300	-0.39348700	-2.09397500
H	-0.15211200	0.16688400	-2.72815600
C	-2.03232000	-1.20119300	0.33886200
C	-3.37768100	-0.82387300	0.04995500
C	-4.39229900	-1.77767600	-0.08855700
H	-5.39889100	-1.43476200	-0.32142100
C	-4.17490900	-3.13811800	0.07739500
C	-2.89461500	-3.51387500	0.44897100
H	-2.68798200	-4.56576500	0.64604200
C	-1.85142000	-2.59315300	0.59718900
C	-3.87596800	0.60530100	-0.07605700
H	-3.57708400	1.09029400	-1.00860800
H	-3.52063900	1.23670300	0.73898000
H	-4.96827900	0.60825300	-0.04078300
C	-5.27501600	-4.14711700	-0.12469300
H	-6.24742900	-3.73493300	0.15826300
H	-5.10011700	-5.04797500	0.46946300
H	-5.34263900	-4.45329300	-1.17439700
C	-0.57147700	-3.27193800	1.08032900
H	-0.28350200	-4.06753000	0.38400300
H	-0.76542100	-3.74619700	2.04941200
H	0.28958100	-2.62203900	1.19058400
B	-0.74460200	-0.12072900	0.51074300
N	1.73053700	1.97653900	0.60817300
H	0.77571600	1.60409600	0.75919400
C	-0.49934700	-0.25589500	2.19544400
H	-1.40553700	0.13667800	2.66395300
C	1.85653700	-0.44996900	3.10525500
H	2.05744100	-1.36331500	2.54973000

C	0.68875300	0.19069000	3.00057600
H	0.57752700	1.08394200	3.62108800
H	2.65319700	-0.08659400	3.74905600
H	-0.56390400	-1.32846500	2.36922700

N10_TS

C	3.82030400	-3.35744800	0.00705700
H	4.68535000	-3.94706900	0.29048600
C	3.65952500	-2.05574400	0.53360200
H	4.42466100	-1.64707700	1.18376200
C	2.56063700	-1.30934400	0.19371800
C	0.27857300	-1.09294300	-0.90705300
C	-0.51538900	-1.63868800	-1.90268500
H	-1.47436400	-1.17784600	-2.11288500
C	-0.22095300	-2.83436900	-2.59140900
H	-0.90711300	-3.19407400	-3.35210300
C	2.91206200	-3.83358000	-0.89564700
C	1.51630900	-1.80200200	-0.65522200
C	0.87454700	-3.57214100	-2.23921300
C	1.76810200	-3.07242000	-1.26082600
C	3.36303800	0.56276100	1.62469900
H	3.26637200	-0.04031800	2.52303500
H	3.09155300	1.59591800	1.85032500
H	4.39683500	0.54073300	1.26304000
C	2.78378400	0.88693500	-0.65705900
H	3.82321500	0.65495700	-0.91480200
H	2.67005600	1.95033200	-0.44875800
H	2.13249100	0.60246200	-1.47785800
C	0.03706600	1.64750900	-0.23245300
C	0.47783500	2.58605200	0.74043000
C	1.00585200	3.82507100	0.35969800
H	1.33673100	4.51165400	1.13699000
C	1.10128100	4.22469300	-0.96865900
C	0.56297800	3.36268500	-1.91493200
H	0.54224900	3.66799000	-2.95973900
C	0.02777000	2.11927200	-1.56980100
C	0.34004400	2.40232400	2.23965000
H	0.99788800	1.64000800	2.66119100
H	-0.68330000	2.13036600	2.51520300
H	0.56980600	3.34459200	2.74308300
C	1.72421200	5.54028600	-1.35530800
H	2.80313400	5.43468300	-1.51282200
H	1.57910200	6.29186000	-0.57501200
H	1.29398600	5.92376400	-2.28393800
C	-0.61503900	1.35068300	-2.70149500
H	-0.83936800	2.02892200	-3.52867100
H	-1.55562700	0.89491500	-2.37861000
H	0.02411700	0.55162000	-3.08630500
C	-2.04942800	-0.20298200	0.21561800
C	-3.02810800	0.83821900	0.18696800
C	-4.39513900	0.54836500	0.25458400
H	-5.10255000	1.37422500	0.22125500
C	-4.88767600	-0.74283500	0.37186700
C	-3.94864300	-1.75759900	0.44384400
H	-4.29133000	-2.78469500	0.56456500
C	-2.57152500	-1.52320900	0.37423500
C	-2.74229800	2.32771900	0.10240400
H	-2.33434100	2.63180000	-0.86419100
H	-2.03883600	2.66758100	0.86160500
H	-3.67494700	2.87692900	0.25029000
C	-6.36668300	-1.02408500	0.40797500
H	-6.77136100	-1.12652200	-0.60465100
H	-6.91121400	-0.21274300	0.89808000
H	-6.58032800	-1.95283400	0.94319600
C	-1.79527200	-2.83096300	0.52304400
H	-2.15661100	-3.55191000	-0.21770900
H	-2.00152700	-3.25549000	1.51231000
H	-0.71970000	-2.77911900	0.39877900
B	-0.42156500	0.08422300	0.05837100
N	2.44391800	0.09240000	0.56657000
H	1.18884000	0.04784500	1.04086900
H	1.08314400	-4.53352000	-2.69994600
H	3.04801900	-4.80859200	-1.35501400
C	0.07381800	-0.52072100	1.90533700
H	-0.69650000	0.08855500	2.37153500
H	-0.38366100	-1.46787900	1.64067300
C	1.07029400	-0.44314800	4.22544300
H	0.36957700	0.32086100	4.55259700
C	1.05545200	-0.91434800	2.97749100
H	1.76201800	-1.70275100	2.71299200
H	1.76995400	-0.82208100	4.96387300

N10-P

C	4.20786500	-2.95607900	-0.16356800
H	5.16351800	-3.43093800	0.02699800
C	3.98868700	-1.62465900	0.25141700
H	4.80109600	-1.07144100	0.70754000
C	2.76777800	-1.04192700	0.04621800

C	0.28951300	-1.17114400	-0.62319600
C	-0.50610300	-1.84159500	-1.54378800
H	-1.52192300	-1.49289500	-1.68961500
C	-0.15232300	-3.02276100	-2.22723100
H	-0.86561600	-3.47544400	-2.90968700
C	3.22752600	-3.59236200	-0.86974500
C	1.64188000	-1.70143900	-0.54809900
C	1.03778800	-3.63583000	-1.95349400
C	1.96761900	-2.98003900	-1.11469800
C	3.53573300	1.03200300	1.26491300
H	3.41668600	0.48604400	2.19996300
H	3.21352400	2.06846500	1.37605600
H	4.56509000	1.00489200	0.90835800
C	2.84756200	1.10146900	-1.07794200
H	3.87561300	0.91053500	-1.38828900
H	2.65021600	2.16641300	-0.94622700
H	2.14277100	0.67557300	-1.78931600
C	-0.04816700	1.52724900	-0.01239200
C	0.51211100	2.52783400	0.83496700
C	0.92435500	3.77504700	0.34026400
H	1.34531500	4.49903200	1.03631600
C	0.78299300	4.13303300	-0.99367800
C	0.18647400	3.19247600	-1.82533500
H	0.01974700	3.44557500	-2.87130100
C	-0.22540500	1.93762400	-1.36852700
C	0.66510100	2.38071100	2.33686000
H	1.21372700	1.48784100	2.63737100
H	-0.31208600	2.33164700	2.82757300
H	1.19077600	3.24851400	2.74517700
C	1.19630300	5.48910700	-1.50331600
H	1.63683700	5.42086100	-2.50182000
H	1.92464800	5.95878600	-0.83749200
H	0.33260800	6.15842700	-1.57324500
C	-0.93134200	1.09285900	-2.40543800
H	-1.29339800	1.73096700	-3.21563700
H	-1.78887000	0.57425500	-1.97367200
H	-0.27425800	0.33707400	-2.84512100
C	-2.16866000	-0.28129400	0.36486300
C	-3.13140700	0.76646900	0.32293100
C	-4.50112200	0.49503200	0.22144300
H	-5.19458700	1.33320300	0.17653100
C	-5.01160400	-0.79472100	0.19822800
C	-4.09467300	-1.82309400	0.35695700
H	-4.45548000	-2.84988400	0.41886900
C	-2.72003700	-1.59313000	0.46317200
C	-2.80861000	2.24430200	0.45255000
H	-2.42514000	2.68811700	-0.47033400
H	-2.06614200	2.43206800	1.22948000
H	-3.71759200	2.78673600	0.72535000
C	-6.48436100	-1.06520300	0.03339300
H	-6.79535900	-1.93603000	0.61728800
H	-6.73341300	-1.26831300	-1.01402500
H	-7.08273200	-0.20798700	0.35290700
C	-1.94243200	-2.87754700	0.73853900
H	-2.11852400	-3.60920600	-0.05766000
H	-2.31391100	-3.31938800	1.67037800
H	-0.86650800	-2.76108400	0.82905300
B	-0.50071100	-0.05277000	0.43813300
N	2.64951900	0.41497000	0.24204500
H	1.67011300	0.61531200	0.51596300
H	1.30151400	-4.59787200	-2.38322000
H	3.39639500	-4.58611000	-1.27514000
C	-0.21073400	-0.51286800	2.05207300
H	-0.54612900	0.27970600	2.72468700
H	-0.94758100	-1.30377000	2.21440300
C	1.75574700	-0.75199200	3.65200600
H	1.45525100	0.09093800	4.27065100
C	1.05768900	-1.11712600	2.57010300
H	1.39968800	-2.01002800	2.04395500
H	2.62656600	-1.31396100	3.97926300

N11_TS

B	0.05070800	-0.18540500	0.06638300
N	-1.93543300	-2.29606500	0.25257000
C	-0.99236600	-2.24028500	1.40483900
H	-0.16776400	-2.90420500	1.12808900
C	-0.44656600	-0.81517500	1.51344100
H	-1.29084100	-0.19388900	1.85906700
C	0.46571400	-0.96864200	2.74759900
H	1.41832500	-1.42628200	2.46478800
H	0.68552400	-0.00990300	3.21798500
C	-0.32729600	-1.93192300	3.69304600
H	0.33268400	-2.69273100	4.11714000
H	-0.76850000	-1.38548400	4.53027700
C	-1.44886600	-2.58847200	2.82525000
H	-2.41572000	-2.12218500	3.04296800
H	-1.54285600	-3.66304900	3.00469400
C	-2.11061900	-3.68581500	-0.26587200

H	-2.58243400	-4.28374100	0.52682700
H	-1.11007100	-4.09165800	-0.44397500
C	-2.96102500	-3.72414700	-1.53474800
H	-3.08482000	-4.76776500	-1.84027200
H	-2.43216700	-3.21488100	-2.35027700
C	-4.31959200	-3.05494100	-1.29990500
H	-4.90810000	-3.04913300	-2.22151500
H	-4.88667200	-3.63183800	-0.55637500
C	-4.10927400	-1.62611700	-0.78930600
H	-3.64124100	-1.01052100	-1.56498300
H	-5.06750500	-1.15307000	-0.55253300
C	-3.24697000	-1.61421100	0.47453500
H	-3.04118100	-0.58976800	0.79601000
H	-3.77116300	-2.13280100	1.28773600
C	-0.66640000	1.28865800	-0.02468400
F	0.75238600	1.96457900	1.77669100
C	-0.28282800	2.25266200	0.91255800
F	-0.43362200	4.39032500	1.97709300
C	-0.86952300	3.49778100	1.03701300
F	-2.52769000	5.05959600	0.31007400
C	-1.92421600	3.83827200	0.20063900
F	-3.40906800	3.22601800	-1.56061500
C	-2.36050800	2.92023300	-0.73458200
F	-2.26282600	0.82175900	-1.77864400
C	-1.73495900	1.67957900	-0.82110500
C	1.68188100	-0.14305200	-0.06977700
F	1.84832700	2.18237200	-0.68802000
C	2.44536300	0.96935100	-0.42297700
F	4.50773100	2.06952100	-0.93651600
C	3.82328600	0.93739200	-0.58747700
F	5.86569300	-0.30112800	-0.58253600
C	4.51007700	-0.25211000	-0.41591900
F	4.45321100	-2.58728300	0.07264600
C	3.80036500	-1.39380900	-0.08555300
F	1.78137500	-2.50780400	0.35485100
C	2.42643000	-1.31014500	0.07570800
H	-1.22946200	-1.59863100	-0.53434400
C	-0.40183600	-1.16903900	-1.37217800
H	-1.39111400	-1.21036300	-1.85949700
C	1.30271600	-0.93198900	-3.23315700
H	1.65832500	-1.94875400	-3.08945300
C	0.33129200	-0.41236100	-2.48303100
H	0.00076200	0.60961100	-2.65081800
H	1.78411600	-0.35276000	-4.01255000
H	0.06104000	-2.16849000	-1.37025000

N_P11

B	-0.00978800	-0.08692900	-0.09671900
N	-3.12780600	-1.30671400	0.10843900
C	-2.10447800	-1.39846700	1.23177400
H	-1.63286600	-2.36647900	1.05376300
C	-1.05015800	-0.29077100	1.19297100
H	-1.61975000	0.65013100	1.27284600
C	-0.43324000	-0.47142200	2.58942600
H	0.21386300	-1.35303800	2.59956400
H	0.17952100	0.38565900	2.87739300
C	-1.64180200	-0.68774100	3.52105400
H	-1.39731300	-1.29047100	4.39830800
H	-2.01227500	0.27502600	3.88382500
C	-2.72738100	-1.36561500	2.64143700
H	-3.65314200	-0.77984400	2.65470700
H	-2.98100300	-2.36971700	2.99241700
C	-4.10963900	-2.43560100	0.16268000
H	-4.71286900	-2.27860600	1.06217700
H	-3.53051000	-3.35351300	0.28965600
C	-4.97541400	-2.46766100	-1.08945600
H	-5.68025600	-3.29884200	-1.00073900
H	-4.34274000	-2.67787800	-1.96150100
C	-5.70405600	-1.13760500	-1.28067600
H	-6.27944200	-1.14958000	-2.20958000
H	-6.41931000	-0.99230600	-0.46096200
C	-4.69622500	0.01055400	-1.29341300
H	-4.05031600	-0.06509200	-2.17819900
H	-5.19784400	0.97973700	-1.35695800
C	-3.82030900	0.01696400	-0.04604900
H	-3.04808900	0.78137100	-0.12481400
H	-4.40731800	0.18021300	0.86195700
C	0.28933500	1.54103800	-0.02601100
F	2.11869000	1.29347300	1.46440500
C	1.28294900	2.09291300	0.78090100
F	2.46619000	3.90889500	1.73231700
C	1.48623400	3.45612400	0.94895900
F	0.82086500	5.66872100	0.45718800
C	0.64994400	4.35626600	0.30473500
F	-1.20957300	4.71066100	-1.10051800
C	-0.37529700	3.86687900	-0.48703400
F	-1.59415200	2.11255400	-1.37702000
C	-0.53175500	2.49218000	-0.62079300

C	1.38491000	-0.97362600	-0.04090600
F	2.66135800	0.70995100	-1.12956200
C	2.57545300	-0.52592800	-0.61814600
F	4.82934800	-0.78411100	-1.28631600
C	3.72984000	-1.28951900	-0.72441300
F	4.83144100	-3.34724600	-0.35265800
C	3.73526200	-2.59560300	-0.25782600
F	2.54112800	-4.36594700	0.74431800
C	2.57602100	-3.10614300	0.29917000
F	0.35622800	-2.90825400	0.90034300
C	1.44937800	-2.29580800	0.37858800
H	-2.56441300	-1.41787300	-0.74511900
C	-0.67704200	-0.54861500	-1.55720400
H	-1.66788700	-0.09968400	-1.71860300
C	0.92708500	-1.01220700	-3.43564300
H	0.95033500	-2.07586000	-3.20700300
C	0.16694200	-0.17415700	-2.73418400
H	0.18458500	0.88839800	-2.97822300
H	1.56236100	-0.66788100	-4.24467300
H	-0.78280200	-1.64908100	-1.54782700

N12_TS

N	2.22540200	-1.79839100	0.11423200
H	1.13111100	-1.14242800	-0.72413000
B	-0.19682500	-0.21906600	0.01791900
C	1.81608100	-1.19101000	1.40760200
H	2.29167800	-0.19810800	1.41799200
C	0.30174200	-0.98183900	1.37961200
H	-0.16517000	-1.97004500	1.44247100
C	3.64189100	-1.52202800	-0.19498900
H	4.27964600	-2.02201500	0.55240500
H	3.80114400	-0.44634300	-0.11077200
C	4.01900000	-2.01572000	-1.58569700
H	3.43016700	-1.46919800	-2.33290200
H	5.07021600	-1.77775400	-1.77416400
C	3.77258700	-3.51911600	-1.70003400
H	4.45164200	-4.04349800	-1.01438700
H	3.99689900	-3.87776400	-2.70870400
C	2.32726200	-3.84568700	-1.32477500
H	1.64779300	-3.47434000	-2.09841100
H	2.17628500	-4.92843000	-1.27305100
C	1.95801400	-3.25311800	0.03671100
H	0.89731100	-3.41714000	0.25098100
H	2.54380600	-3.77152400	0.80984100
C	2.27578300	-1.94327700	2.66096800
H	1.81283800	-2.93774600	2.68441800
H	3.36281000	-2.08928400	2.65334500
C	1.84369300	-1.18322200	3.91750600
H	2.16395600	-1.72776500	4.81131900
H	2.34491500	-0.20618600	3.94367400
C	0.32905800	-0.97339300	3.92219600
H	0.02730100	-0.41270500	4.81294800
H	-0.17053600	-1.95010300	3.97503100
C	-0.11997800	-0.23464500	2.65966000
H	0.33471100	0.76461600	2.67019100
H	-1.20384800	-0.08346400	2.66565500
C	0.42467100	1.28659200	-0.04331900
C	-0.15050500	2.32933700	0.68541500
F	-1.27029300	2.11904400	1.39376400
C	0.36803600	3.61466800	0.74606200
F	-0.23948800	4.55840700	1.46252100
C	1.54078500	3.90981000	0.06568300
F	2.06276800	5.13142700	0.11694300
C	2.16784600	2.90758400	-0.65507700
F	3.30803400	3.15939200	-1.29873600
C	1.60933600	1.63548700	-0.68596900
F	2.31879700	0.72260000	-1.38110400
C	-1.80869200	-0.38328800	-0.19164100
C	-2.69071600	0.63745200	-0.55314600
F	-2.23787800	1.84098400	-0.93062200
C	-4.07071600	0.48456200	-0.62288100
F	-4.84607000	1.50950100	-0.97004500
C	-4.63915100	-0.74997900	-0.35507600
F	-5.95498600	-0.91777900	-0.42611100
C	-3.80848300	-1.81795300	-0.05349100
F	-4.32495500	-3.02762700	0.15733100
C	-2.43641700	-1.61696100	0.00732600
F	-1.71070200	-2.73374700	0.21585800
C	0.27617800	-1.07218700	-1.73611000
H	1.19097500	-1.47110500	-2.19253100
H	-0.42076000	-1.91252400	-1.68467300
C	0.54000200	0.39111000	-3.74200700
H	1.50859300	-0.04585200	-3.97163400
C	-0.17022100	0.00437400	-2.68566100
H	-1.12509100	0.48368000	-2.49637500
H	0.17834700	1.16596100	-4.40916800

N12_P

N	2.54757100	-1.44563900	0.00548700

H	2.06873900	-0.83624300	-0.67011600
B	-0.36656900	-0.43173100	-0.30153300
C	1.64237900	-1.46999500	1.23797700
H	1.83325200	-0.49560200	1.70824700
C	0.15353100	-1.53452100	0.86148200
H	-0.04295200	-2.54569100	0.47383000
C	3.88499000	-0.81977800	0.27593900
H	4.38570800	-1.45456800	1.01255000
H	3.68644800	0.15503200	0.72886400
C	4.71673700	-0.68875600	-0.99491300
H	4.24868200	0.05076900	-1.65115200
H	5.69569300	-0.29182400	-0.71141800
C	4.85504500	-2.03013700	-1.71396100
H	5.44312100	-2.72202400	-1.09706100
H	5.39631100	-1.89879700	-2.65416200
C	3.47295500	-2.62824200	-1.97426700
H	2.91340200	-1.99204500	-2.67127500
H	3.54998400	-3.61623200	-2.43632200
C	2.69349300	-2.77422800	-0.67367900
H	1.68520300	-3.16163000	-0.83450200
H	3.22570500	-3.43050800	0.01989200
C	2.07195600	-2.57910200	2.20071000
H	1.86301600	-3.55601000	1.74739300
H	3.14718100	-2.53578700	2.41439100
C	1.27403000	-2.47509400	3.50242500
H	1.56988700	-3.28474800	4.17644500
H	1.52236500	-1.53166400	4.00625800
C	-0.22273900	-2.51513500	3.20596300
H	-0.79312300	-2.39842800	4.13314800
H	-0.48538300	-3.49738600	2.79003400
C	-0.60413100	-1.42629900	2.20353600
H	-0.40671700	-0.44328400	2.64974500
H	-1.67833300	-1.45900900	2.01704400
C	0.38354000	1.02042000	0.02108500
C	-0.02190800	1.85300700	1.06914700
F	-1.11359300	1.54981100	1.78654900
C	0.62463300	3.02456800	1.43853400
F	0.16860300	3.76465400	2.44755900
C	1.76334900	3.42902500	0.75296200
F	2.40835400	4.53827300	1.10533700
C	2.21846000	2.64688900	-0.29293300
F	3.33023700	2.97789600	-0.95587800
C	1.51625200	1.49390100	-0.62878000
F	2.08001100	0.78446000	-1.64849100
C	-2.01549500	-0.28571800	-0.27187000
C	-2.72472800	0.89383400	-0.49145500
F	-2.08448500	2.06084800	-0.66789100
C	-4.11097100	0.96717700	-0.57486800
F	-4.71702700	2.13731500	-0.78350100
C	-4.86733000	-0.18819800	-0.46407200
F	-6.19559200	-0.14108900	-0.55081700
C	-4.21624800	-1.39661900	-0.27382600
F	-4.92053900	-2.52659700	-0.17907000
C	-2.82982500	-1.41507600	-0.19639000
F	-2.28026300	-2.63820000	-0.03691200
C	-0.08847200	-1.00774200	-1.84124600
H	0.94157000	-1.30549700	-2.08089000
H	-0.67395900	-1.93848200	-1.89420700
C	0.20531500	0.40486500	-3.91303300
H	1.26010600	0.15405500	-4.00029300
C	-0.54816700	-0.08885700	-2.93261100
H	-1.59781800	0.20360000	-2.89309100
H	-0.19950700	1.08266700	-4.65718800

N14_TS

B	0.51700600	-0.15240400	0.13740200
N	-2.19338300	-0.73999500	0.83056600
H	-0.75977000	-0.42616400	1.29176100
C	-1.95731000	-0.46794400	-0.62052500
H	-2.02814200	0.62370900	-0.70384700
C	-0.51292200	-0.86169900	-0.90273400
H	-0.23575400	-0.50512800	-1.90230200
H	-0.39906400	-1.94635800	-0.92641100
C	-2.38846200	-2.17790800	1.12503700
H	-1.52138200	-2.72399700	0.73934100
H	-3.27945400	-2.53698100	0.58968400
C	-2.55911900	-2.44852300	2.62014400
H	-2.77216200	-3.51422600	2.74868200
H	-1.62128400	-2.25266000	3.15060800
C	-3.67972700	-1.59702000	3.21513200
H	-4.64110700	-1.91792700	2.79268600
H	-3.74026600	-1.74291900	4.29747000
C	-3.45549100	-0.12453500	2.87421900
H	-4.29110400	0.48874500	3.22489500
H	-2.55309500	0.25418300	3.37048900
C	-3.31213300	0.05622400	1.36829500
H	-3.12230700	1.10367100	1.12296700
H	-4.24600800	-0.25196200	0.87037900

C	-3.00757100	-1.05220500	-1.54926100
C	-4.12143900	-0.28290900	-1.89885800
H	-4.20573300	0.73276600	-1.51868500
C	-5.11103700	-0.78986400	-2.73644400
H	-5.96460300	-0.17359500	-2.99997400
C	-4.99754700	-2.08096600	-3.24295700
H	-5.76294900	-2.47851800	-3.90150700
C	-3.88979200	-2.85616000	-2.90951600
H	-3.78918600	-3.86039200	-3.30865100
C	-2.90321800	-2.34473500	-2.07219200
H	-2.04058800	-2.95801400	-1.82969800
C	1.99599500	-0.82100100	0.00984500
C	3.19077100	-0.12068800	-0.15442400
F	3.22156700	1.21542600	-0.06347700
C	4.42217600	-0.73112500	-0.36357100
F	5.52150800	0.00290600	-0.51863500
C	4.50553900	-2.11415500	-0.39089700
F	5.67404400	-2.71648200	-0.58459700
C	3.35583400	-2.86237500	-0.18693600
F	3.41798400	-4.19303900	-0.17360300
C	2.14776500	-2.20904200	0.01088500
F	1.09208700	-3.01126400	0.25983700
C	0.38090800	1.45756000	-0.04113500
C	-0.36929700	2.31944400	0.75224300
F	-1.03999800	1.86762700	1.83163300
C	-0.52724800	3.67493600	0.48424500
F	-1.25597000	4.44188800	1.29556700
C	0.06099100	4.22005700	-0.64484000
F	-0.08205600	5.51243500	-0.92312900
C	0.78438600	3.39490600	-1.49560200
F	1.33362500	3.89869600	-2.59887600
C	0.91091700	2.04905600	-1.18778900
F	1.58274300	1.29399700	-2.07035200
C	0.30311800	-0.52964600	2.07351100
H	-0.53123700	-0.49839700	2.78606000
H	0.65123100	-1.56532500	2.07046900
C	1.10463700	1.17119500	3.71970600
H	0.16016000	1.14373900	4.25708800
C	1.31763600	0.42791000	2.63750900
H	2.27455900	0.50362400	2.13025600
H	1.86792100	1.84167700	4.09941500

N14_P

B	0.72108100	-0.16211400	0.64754400
N	-2.71804800	-0.64035300	0.77756500
H	-2.11691200	-0.39549400	1.58720700
C	-1.80906200	-0.51475000	-0.46203000
H	-1.75442500	0.56491300	-0.62315800
C	-0.41317300	-1.06367300	-0.17347200
H	0.01384700	-1.24284500	-1.16602100
H	-0.49354500	-2.06487100	0.26956800
C	-3.22778700	-2.03315900	1.01177400
H	-2.35733100	-2.69021100	1.05670300
H	-3.81656600	-2.29572300	0.12970600
C	-4.07500900	-2.13387300	2.27604100
H	-4.46390700	-3.15501500	2.32831200
H	-3.44304900	-1.98406400	3.15507300
C	-5.21120600	-1.11282500	2.27979800
H	-5.92857900	-1.34929800	1.48340400
H	-5.75490100	-1.15664800	3.22699100
C	-4.64350700	0.28617700	2.04764500
H	-5.43806500	1.03563300	1.99678600
H	-3.98110800	0.57172000	2.87474100
C	-3.85778700	0.33406000	0.74595100
H	-3.42751400	1.31952500	0.56705000
H	-4.48332600	0.04898300	-0.10550400
C	-2.49928100	-1.11386200	-1.67550800
C	-3.32662000	-0.31649000	-2.47103200
H	-3.45431600	0.73522900	-2.22397300
C	-3.96443400	-0.84267800	-3.59186900
H	-4.59572500	-0.20596300	-4.20297800
C	-3.77643500	-2.17768400	-3.93390400
H	-4.26645000	-2.59050900	-4.80961000
C	-2.93729500	-2.97766700	-3.16065200
H	-2.76825400	-4.01381400	-3.43507600
C	-2.29860600	-2.44819700	-2.04476600
H	-1.61907800	-3.06813100	-1.46654200
C	2.22778400	-0.77872900	0.31469900
C	3.36529900	-0.05378800	0.67461400
F	3.22932500	1.18784800	1.17556800
C	4.66704600	-0.51051500	0.54621400
F	5.70098600	0.25437100	0.90239100
C	4.89084100	-1.78605800	0.04637900
F	6.13198700	-2.25310900	-0.08595400
C	3.80273200	-2.56269700	-0.30810200
F	3.99088200	-3.79900200	-0.77801200
C	2.51405600	-2.05447400	-0.15986300
F	1.53456000	-2.91594600	-0.51023600

C	0.58346400	1.40662800	0.11469800
C	-0.45545400	2.22896800	0.53433600
F	-1.39726500	1.74573300	1.39434400
C	-0.69101100	3.52716100	0.10468800
F	-1.74615200	4.21215400	0.55709900
C	0.17089000	4.08999500	-0.82151900
F	-0.01828400	5.33297700	-1.25863300
C	1.22029200	3.32101500	-1.30007100
F	2.04558000	3.83375400	-2.21196600
C	1.39478000	2.01787600	-0.84432300
F	2.41135300	1.36004700	-1.41512400
C	0.71224400	-0.33572300	2.31592100
H	0.94785600	-1.39600200	2.48230800
H	1.57775100	0.23443000	2.67625600
C	-1.27294700	-0.87213100	3.76149000
H	-1.12995900	-1.94337700	3.62473200
C	-0.44096100	0.01061900	3.19280700
H	-0.59954500	1.06495000	3.41177300
H	-2.06581100	-0.56097000	4.43663300

N15_TS

N	-2.19363000	-0.67363800	1.14489700
B	0.46108200	-0.17854600	0.20169000
C	-2.07063300	-0.53926700	-0.34144000
H	-2.16036300	0.53853100	-0.52380800
C	-0.64701200	-0.95423000	-0.69792700
H	-0.45248800	-0.69052700	-1.74475900
H	-0.52260800	-2.03621600	-0.63122400
C	-2.35899000	-2.07189100	1.61605700
C	-3.78599500	-2.61625800	1.70459400
H	-4.37254300	-2.09700600	2.46663000
H	-3.73524300	-3.66938700	1.99341700
C	-3.08469100	0.32434000	3.25872600
H	-3.82665400	1.03515800	3.63008100
H	-2.10113500	0.67657700	3.57524400
C	-3.17712200	0.24980800	1.73977300
C	-3.18180400	-1.21875400	-1.12125700
C	-4.39702200	-0.55109900	-1.30376300
H	-4.50226700	0.47006600	-0.94458400
C	-5.46832800	-1.17144300	-1.93943800
H	-6.40440200	-0.63757100	-2.06851300
C	-5.33398900	-2.47272400	-2.41678500
H	-6.16608000	-2.96053700	-2.91393600
C	-4.11980100	-3.13723600	-2.26900300
H	-4.00124000	-4.14434300	-2.65584100
C	-3.05131600	-2.51335100	-1.63046800
H	-2.11069800	-3.04527700	-1.52953000
C	1.95134100	-0.76429600	-0.04600100
C	3.08346500	0.02125600	-0.26013600
F	3.01255900	1.35584900	-0.22057100
C	4.34937000	-0.50175300	-0.49279500
F	5.38813000	0.30672200	-0.69479100
C	4.53587700	-1.87555200	-0.49260800
F	5.74165300	-2.39181600	-0.70745000
C	3.45031500	-2.70313900	-0.25059100
F	3.61097100	-4.02584900	-0.21937200
C	2.20081400	-2.13645300	-0.03519400
F	1.21558100	-3.01127100	0.25366400
C	0.25164300	1.42303800	-0.00467000
C	-0.38909100	2.31577500	0.84348100
F	-0.82353700	1.92746300	2.06370100
C	-0.65192700	3.64464400	0.52379400
F	-1.27701900	4.43559600	1.39557800
C	-0.27385900	4.13238200	-0.71489500
F	-0.51555300	5.39658200	-1.04737900
C	0.35806000	3.27942800	-1.61115100
F	0.71897800	3.73006200	-2.81016300
C	0.59120500	1.96308900	-1.24621400
F	1.18874900	1.18534200	-2.16060400
H	-4.31911200	-2.55102800	0.75501700
H	-3.28286100	-0.63885900	3.73582300
H	-1.89549200	-2.13940400	2.60781700
H	-1.75642300	-2.70507800	0.95854700
H	-2.97863500	1.24041900	1.32107000
H	-4.19298100	-0.03807000	1.43618800
H	-0.72687400	-0.40287000	1.48167600
C	0.39953900	-0.59744300	2.14478500
H	0.73112200	-1.63452700	2.07432700
H	-0.37827300	-0.60032400	2.92811900
C	2.71640900	-0.07401300	2.92965600
H	3.05231000	-1.08885900	2.72782400
C	1.46895400	0.31188800	2.67835900
H	1.17003300	1.33261900	2.89530000
H	3.44581500	0.61609800	3.33954300

N15_P

N	-2.44897100	-0.26966200	1.06755100
B	0.61263500	-0.22682500	0.57799300

C	-1.90804500	-0.81420700	-0.27778000
H	-1.88892200	0.08465300	-0.90269500
C	-0.47915300	-1.30278700	-0.08688100
H	-0.14583500	-1.58724000	-1.09266200
H	-0.46733900	-2.23089600	0.49324600
C	-2.77644100	-1.33695100	2.08550200
C	-4.20446100	-1.86282000	2.04427000
H	-4.93001300	-1.10914600	2.35781100
H	-4.26825400	-2.69124000	2.75378500
C	-3.83873600	1.51586700	2.17316900
H	-4.63383300	2.23441600	1.96307600
H	-2.96290100	2.07301100	2.50857600
C	-3.53673700	0.74685100	0.89652200
C	-2.88959700	-1.79068900	-0.89280800
C	-3.95233700	-1.30646000	-1.66166100
H	-4.03235900	-0.23837100	-1.85074300
C	-4.89820900	-2.17272000	-2.20201800
H	-5.71564500	-1.77865600	-2.79695900
C	-4.78278600	-3.54370400	-1.99004300
H	-5.51588100	-4.22411000	-2.41064300
C	-3.70773800	-4.04089000	-1.25760600
H	-3.59757400	-5.11052700	-1.11171700
C	-2.76356600	-3.17186000	-0.71829200
H	-1.92060300	-3.57484800	-0.16650300
C	2.13260800	-0.63288700	0.10517400
C	3.14741700	0.28706500	-0.14650800
F	2.89833400	1.60603000	-0.10705600
C	4.46079400	-0.06333600	-0.43286500
F	5.38030300	0.87483400	-0.66926900
C	4.82095300	-1.40165400	-0.45981400
F	6.07612000	-1.76130700	-0.72675300
C	3.85713400	-2.36077300	-0.19433000
F	4.18458600	-3.65556900	-0.20044800
C	2.55616900	-1.95853800	0.08164100
F	1.69335200	-2.95761800	0.36646900
C	0.18684800	1.27551800	0.01471900
C	-0.47850700	2.25706300	0.73399500
F	-0.78333800	2.05924000	2.04689700
C	-0.95007600	3.45584000	0.20558400
F	-1.61608000	4.32102000	0.97491900
C	-0.74320200	3.72323300	-1.13636800
F	-1.18977200	4.85290000	-1.67995100
C	-0.07750000	2.78178100	-1.91399700
F	0.11471600	3.02095200	-3.21005600
C	0.35860600	1.59921500	-1.33366800
F	0.96435600	0.72820700	-2.15116000
H	-4.48797000	-2.24116500	1.06195400
H	-4.18274700	0.86787400	2.98230600
H	-2.56106300	-0.90027100	3.06392000
H	-2.04970100	-2.13458600	1.92269600
H	-3.18372500	1.43180500	0.12047100
H	-4.41142200	0.21672700	0.51435800
H	-1.63730100	0.22849600	1.45793400
C	0.65802000	-0.35679600	2.23598000
H	0.94721000	-1.39719900	2.44021000
H	-0.31118000	-0.21494000	2.74207300
C	2.88206000	0.21166400	3.25325900
H	3.22993400	-0.81447100	3.15396900
C	1.65408800	0.56234400	2.87550700
H	1.35414200	1.60483600	2.97756400
H	3.58376900	0.93319500	3.65871700

N16_TS

N	-2.36146600	-0.27788500	0.97478900
H	-0.94980400	-0.42206400	1.40628200
B	0.44233300	-0.40218600	0.09678600
C	-2.08458700	-0.40947900	-0.49272700
H	-1.85836400	0.62022200	-0.78786100
C	-0.74322200	-1.16552800	-0.77107700
C	-3.15475000	-1.37287800	1.58927200
H	-2.66051900	-2.32373800	1.39586600
H	-4.14544500	-1.40116100	1.11428100
C	-3.34403500	-1.17656000	3.09114900
H	-2.37183300	-1.19518600	3.59422300
H	-3.91468800	-2.02837200	3.47445700
C	-4.05380200	0.14172700	3.38054400
H	-5.07012300	0.10649200	2.96659700
H	-4.14828700	0.30743300	4.45756200
C	-3.27157200	1.27395300	2.72237500
H	-3.79075000	2.23125800	2.83049600
H	-2.29553100	1.38233500	3.20600300
C	-3.06666600	1.00369500	1.23637200
H	-4.04367700	0.96383300	0.72724000
H	-2.49092000	1.81051900	0.77926700
C	-3.29479000	-0.78870500	-1.34014600
C	-3.80867700	0.18218600	-2.20778700
H	-3.33726100	1.16167700	-2.24181000
C	-4.89783800	-0.07819600	-3.03431400

H	-5.26824500	0.69618700	-3.69834300
C	-5.50517700	-1.32855700	-3.00793300
H	-6.35417700	-1.54065900	-3.64962700
C	-5.01376500	-2.30718700	-2.14889300
H	-5.47993400	-3.28687800	-2.11670500
C	-3.92306100	-2.04220400	-1.32597500
H	-3.57045800	-2.82557100	-0.66611700
C	2.01439800	-0.91369200	0.03237200
C	2.98905000	-0.20038100	0.75050800
F	2.64685400	0.84834100	1.52297100
C	4.34802700	-0.47729600	0.74593300
F	5.18881700	0.25333800	1.47440100
C	4.82939900	-1.51204900	-0.03956300
F	6.12601500	-1.79492000	-0.07024300
C	3.92799500	-2.23375000	-0.79915500
F	4.36050600	-3.21883200	-1.58351100
C	2.57102700	-1.92237800	-0.75863400
F	1.83411700	-2.67601100	-1.58864500
C	0.49378600	1.21988200	-0.14144700
C	0.07275900	2.22502800	0.72320800
F	-0.45899600	1.92241100	1.92318600
C	0.17327000	3.58419400	0.45230300
F	-0.27205200	4.48152800	1.33087700
C	0.75480700	4.00155900	-0.73393700
F	0.86604400	5.29593500	-1.01315200
C	1.22893100	3.04554500	-1.61941300
F	1.81320600	3.42194100	-2.75434300
C	1.10218000	1.69856900	-1.30451900
F	1.63640800	0.84483800	-2.18982500
C	-0.52387800	-0.99530500	-2.29106000
H	-1.28273500	-1.56690400	-2.83648400
H	-0.62565000	0.05067800	-2.59894100
H	0.45525500	-1.34484500	-2.61037400
C	-0.84381900	-2.67429500	-0.47471700
H	0.10045800	-3.17563400	-0.67162100
H	-1.09903900	-2.89553600	0.56166100
H	-1.58507300	-3.14495600	-1.12724200
C	0.14419300	-0.71869400	2.07911700
H	-0.49579300	-0.30354500	2.87289700
H	1.02484800	-0.09358100	2.19616000
C	-0.00174400	-2.79879100	3.47882400
H	-0.58332900	-2.30069700	4.25025200
C	0.41902600	-2.15785200	2.38947100
H	1.02477100	-2.70553100	1.66941400
H	0.24183100	-3.84213900	3.64867300

N16_P

N	-2.44457000	-0.00263000	0.79145400
H	-1.60972100	0.43648200	1.19539400
B	0.58177200	-0.41981800	0.42337400
C	-1.93856600	-0.67794100	-0.50051800
H	-1.74664400	0.20640300	-1.11515000
C	-0.54462800	-1.38300400	-0.41250800
C	-2.99946700	-0.87780100	1.87624800
H	-2.25100100	-1.64021400	2.09415200
H	-3.90114100	-1.34640100	1.47561600
C	-3.32571200	-0.04873600	3.11459000
H	-2.40498700	0.39732200	3.51156700
H	-3.70552500	-0.73057600	3.88096400
C	-4.34088300	1.04650400	2.79249900
H	-5.28817900	0.58651100	2.48266600
H	-4.54959200	1.64834400	3.68046000
C	-3.80500100	1.92817700	1.66698200
H	-4.55783900	2.65191100	1.34126700
H	-2.93621700	2.49928300	2.00716000
C	-3.41492700	1.09669700	0.45210600
H	-4.28251200	0.60362300	0.00304300
H	-2.93213700	1.71379100	-0.31013700
C	-3.07844000	-1.42247600	-1.17383400
C	-3.59991700	-0.89641600	-2.36125000
H	-3.17439000	0.01866800	-2.76655100
C	-4.64009900	-1.52726200	-3.03726700
H	-5.02178200	-1.10024200	-3.95883800
C	-5.18500000	-2.70293500	-2.53074100
H	-5.99673700	-3.19906600	-3.05241600
C	-4.67505400	-3.24374900	-1.35420400
H	-5.08470500	-4.16666900	-0.95685400
C	-3.62943900	-2.61461700	-0.68470200
H	-3.23640600	-3.06885400	0.21700700
C	2.20649200	-0.76630900	0.18836200
C	3.12657200	0.18920700	0.64477400
F	2.70206900	1.33610200	1.21105600
C	4.50729200	0.06944800	0.57437800
F	5.29571500	1.04393000	1.02985900
C	5.06604200	-1.07494100	0.02885100
F	6.38685700	-1.21766000	-0.05424800
C	4.21544700	-2.06664200	-0.42232300
F	4.71880300	-3.18639500	-0.94457500

C	2.83575800	-1.89848400	-0.32759700
F	2.14100000	-2.95623300	-0.79199200
C	0.41342500	1.16363500	-0.13486000
C	-0.19098400	2.21597300	0.54634200
F	-0.71947400	2.02265400	1.78959700
C	-0.35237500	3.50849000	0.06256600
F	-0.99665500	4.42536800	0.78897800
C	0.16227900	3.82905000	-1.18185400
F	0.02795100	5.05576300	-1.67856300
C	0.81869600	2.84034800	-1.90062400
F	1.33835600	3.13039600	-3.09164100
C	0.93604100	1.55972800	-1.37074000
F	1.62049700	0.69552500	-2.13305900
C	-0.19614900	-1.52966000	-1.91513100
H	-0.88262600	-2.23155400	-2.40424600
H	-0.26537900	-0.57730900	-2.44900800
H	0.81362700	-1.90718600	-2.05047000
C	-0.63413400	-2.80783500	0.16794100
H	0.36350300	-3.22005200	0.30402900
H	-1.11167300	-2.86196900	1.14752300
H	-1.16611800	-3.47948100	-0.51559900
C	0.43615800	-0.49052000	2.09041500
H	-0.53111100	-0.20746400	2.52393200
H	1.12915300	0.26843600	2.47707200
C	0.11580700	-2.40476600	3.69267800
H	-0.78614600	-1.95742200	4.11047200
C	0.80112200	-1.80160900	2.72125600
H	1.70880100	-2.28199600	2.35192500
H	0.43240800	-3.35119000	4.11823100

N17_TS

B	0.08946500	-0.25427500	0.02689500
N	-2.62989800	-1.33835700	-0.28556800
H	-1.44938000	-0.96571100	-0.97482900
C	-2.28707500	-0.74580100	1.03162600
H	-2.51038800	0.32669700	0.93280700
C	-0.77719600	-0.92027200	1.21685900
H	-0.58849600	-1.99923100	1.25067200
C	-3.92868700	-0.86542000	-0.84688700
H	-4.51688200	-0.40716700	-0.04346800
H	-3.74874100	-0.10769700	-1.60935900
C	-4.63313900	-2.10803600	-1.39320900
H	-4.32028600	-2.28683000	-2.42617700
H	-5.71981800	-2.00465700	-1.37189100
C	-4.10474300	-3.22420800	-0.49146500
H	-4.22552200	-4.22254200	-0.91698300
H	-4.60849600	-3.20062900	0.48149500
C	-2.63946000	-2.82973000	-0.33107600
H	-2.06073600	-3.14278900	-1.20629500
H	-2.16249800	-3.25087600	0.55419600
C	-3.10251100	-1.29453300	2.20653100
H	-2.97826500	-2.38346500	2.26677300
H	-4.17121100	-1.10097900	2.05372700
C	-2.63169000	-0.68063800	3.52762900
H	-2.82038300	0.40140000	3.51409800
H	-3.21503000	-1.09289500	4.35694500
C	-1.13714700	-0.92666000	3.73587000
H	-0.95036400	-2.00701700	3.80275200
H	-0.80811600	-0.48682000	4.68298400
C	-0.33228500	-0.34340700	2.57500400
H	0.73769700	-0.52074500	2.72034300
H	-0.47127700	0.74668900	2.56836800
C	-0.06531800	1.36253300	-0.01382500
C	0.83595300	2.17604300	0.67355100
F	1.85966100	1.62875800	1.34459900
C	0.73839000	3.55942100	0.72926200
F	1.63225500	4.27704500	1.40615900
C	-0.31004300	4.19771600	0.08021400
F	-0.42560800	5.52078100	0.12910200
C	-1.24211200	3.43553100	-0.60443400
F	-2.26871400	4.02218500	-1.21894000
C	-1.10092500	2.05350000	-0.62762800
F	-2.06285100	1.38022600	-1.29100700
C	1.62416400	-0.76245300	-0.11267200
C	2.18884400	-1.83015100	0.58596400
F	1.48012000	-2.55752000	1.46454300
C	3.51727900	-2.22407800	0.45415400
F	3.98520600	-3.25314000	1.15853500
C	4.35454600	-1.54225600	-0.41156000
F	5.62373500	-1.90748400	-0.55226900
C	3.84794400	-0.47087300	-1.13322000
F	4.63528000	0.19724800	-1.97309800
C	2.52041700	-0.11056400	-0.96598500
F	2.10731500	0.93500400	-1.70167600
C	-0.45981300	-0.93104100	-1.89454500
H	0.04936200	-1.89845600	-1.90108800
H	0.26481500	-0.15251900	-2.13282500
C	-1.87310900	-1.93983400	-3.71687400

H	-1.45141900	-2.92917800	-3.55394200
C	-1.48449500	-0.88674700	-2.99851500
H	-1.91570900	0.08901600	-3.21043900
H	-2.60121400	-1.83989900	-4.51547800

N17_P

B	0.14454200	-0.28799500	-0.33676000
N	-2.82174900	-1.39640400	-0.40589400
H	-2.26355600	-0.91605700	-1.12392300
C	-2.22443700	-0.97048600	0.92661600
H	-2.52119400	0.08040400	1.02534300
C	-0.69638400	-1.07932000	0.87453700
H	-0.47073300	-2.15179300	0.80153600
C	-4.25052300	-0.95573400	-0.62929100
H	-4.68338900	-0.72388200	0.34505100
H	-4.22934000	-0.05105000	-1.23543200
C	-4.93763300	-2.14954500	-1.28539400
H	-4.77665900	-2.12484100	-2.36785200
H	-6.01222200	-2.15224200	-1.09618800
C	-4.20482000	-3.34452300	-0.67121200
H	-4.33989900	-4.27128900	-1.23108900
H	-4.53741300	-3.51361800	0.35829900
C	-2.75421200	-2.87937100	-0.69369000
H	-2.32273600	-2.99006900	-1.68907300
H	-2.09498900	-3.36143400	0.02490300
C	-2.85305600	-1.76797300	2.07308900
H	-2.61779900	-2.83379900	1.95929700
H	-3.94596700	-1.67476200	2.07300000
C	-2.29108700	-1.29776000	3.41713700
H	-2.58598700	-0.25367000	3.58647400
H	-2.73471200	-1.88907700	4.22397800
C	-0.76739100	-1.39875700	3.42154700
H	-0.46517100	-2.45100300	3.33361300
H	-0.36939900	-1.02994100	4.37241800
C	-0.18409700	-0.60693200	2.25369800
H	0.90701200	-0.64692600	2.27285200
H	-0.45069100	0.45242600	2.38151900
C	-0.09118900	1.34329200	-0.08290800
C	0.82073200	2.20081500	0.53945400
F	2.00033600	1.74991300	0.98708200
C	0.59307100	3.55221300	0.77333800
F	1.52364300	4.30196800	1.36242100
C	-0.61710300	4.12380400	0.41140300
F	-0.85392500	5.41462000	0.63335800
C	-1.58141900	3.31666800	-0.16628100
F	-2.77426700	3.81847400	-0.49539900
C	-1.29460300	1.97439100	-0.37890500
F	-2.33667000	1.26095400	-0.89207700
C	1.75859300	-0.70274900	-0.22379300
C	2.32059500	-1.81146500	0.40232100
F	1.58339000	-2.66560900	1.14579200
C	3.67190100	-2.14954300	0.33521800
F	4.12623100	-3.23268300	0.97041000
C	4.54117100	-1.36735800	-0.40116300
F	5.83394800	-1.67680600	-0.48470700
C	4.03862100	-0.25390700	-1.06068400
F	4.85578800	0.51481500	-1.78242900
C	2.68839200	0.03493400	-0.96316600
F	2.27459000	1.11657000	-1.64888100
C	-0.15410200	-0.75587400	-1.94238500
H	-0.20026500	-1.85668900	-1.97927700
H	0.77469700	-0.49272500	-2.46109400
C	-2.13177900	-0.86297100	-3.53086000
H	-2.14459600	-1.95143300	-3.54610200
C	-1.21998000	-0.18242700	-2.82560100
H	-1.21566900	0.90265400	-2.92218600
H	-2.84293900	-0.36084200	-4.17929000

N18_TS

C	-2.83621300	-0.85976100	1.85252200
C	-2.08231800	-0.50157000	0.50340200
H	-2.15291100	0.58832700	0.41388900
C	-0.62257400	-0.87499900	0.76882300
H	-0.51057500	-1.92371900	0.51162400
C	-0.60482000	-0.86875700	2.31515200
H	0.34312500	-1.20818100	2.74419200
C	-1.09770000	0.49207900	2.87427700
H	-0.64797400	1.33625800	2.35524100
H	-0.84057200	0.60164800	3.93030800
C	-2.63950400	0.44606800	2.65812200
H	-3.01709900	1.31795200	2.11402700
H	-3.18715700	0.39733700	3.60375300
C	-1.83069100	-1.78787400	2.62000000
C	-4.28116600	-1.31677900	1.77408700
H	-4.40099200	-2.23428600	1.18937200
H	-4.91970100	-0.54494600	1.33232000
H	-4.66659700	-1.51492000	2.77993700
C	-1.74720800	-3.23005500	2.10564800

H	-1.82165300	-3.33553600	1.02665800
H	-2.56103200	-3.82190300	2.53831700
H	-0.80389400	-3.68981300	2.42087800
C	-2.10684100	-1.91898000	4.12792200
H	-3.11142300	-2.31811100	4.30708600
H	-2.01094800	-0.98926900	4.68773500
H	-1.39701900	-2.63275200	4.55848100
N	-2.43502200	-0.91623600	-0.87670800
H	-1.11743800	-0.49663000	-1.40508900
C	-2.52779800	-2.36332300	-1.16969100
H	-3.34383200	-2.80656000	-0.58167700
H	-1.59110900	-2.83574300	-0.86714100
C	-2.76185300	-2.64608900	-2.65626400
H	-2.90095500	-3.72531500	-2.77258100
H	-1.86455500	-2.38479400	-3.22736400
C	-3.96108000	-1.87868800	-3.20694800
H	-4.88171700	-2.25191400	-2.73870700
H	-4.05934300	-2.04234300	-4.28384700
C	-3.80824900	-0.39368200	-2.88723300
H	-2.94665700	0.02950100	-3.41894700
H	-4.68881800	0.17068900	-3.20860300
C	-3.62367500	-0.19857200	-1.38795000
H	-3.50194700	0.85821800	-1.14542100
H	-4.51754500	-0.57036800	-0.86532800
B	0.40077900	-0.08731500	-0.20053300
C	1.89588600	-0.74803700	-0.24688000
F	1.05537500	-2.99174300	-0.24097100
C	2.09394500	-2.13279900	-0.19162900
F	3.42997000	-4.07637500	-0.08726000
C	3.33745300	-2.74867100	-0.14614500
F	5.68556200	-2.54101200	-0.13926500
C	4.48545800	-1.97341400	-0.17687200
F	5.44376100	0.16572000	-0.36281300
C	4.35693900	-0.59879800	-0.28432800
F	3.08880400	1.29932300	-0.52844400
C	3.09110700	-0.02702100	-0.33088500
C	0.29689900	1.52459500	-0.06919800
F	1.85194800	1.48966700	1.70797300
C	1.04423900	2.19289300	0.89953200
F	1.71790700	4.13312600	2.07081000
C	0.97263600	3.55789200	1.12893200
F	-0.00888900	5.63450900	0.58914600
C	0.08836500	4.32494300	0.38074500
F	-1.59014900	4.41886200	-1.26798500
C	-0.71008200	3.70504900	-0.56554300
F	-1.46353900	1.80625500	-1.65156700
C	-0.59947900	2.33278500	-0.75897500
C	-0.01271800	-0.49972600	-2.18501200
H	-0.86176800	-0.56993300	-2.88107000
C	0.50874700	1.28491600	-3.86016700
H	-0.47128400	1.18911200	-4.32146000
C	0.86950800	0.53861000	-2.81895700
H	1.85566900	0.69039500	-2.39135300
H	1.18047100	2.02548700	-4.28048500
H	0.43848000	-1.49607800	-2.19065600

N19_TS

B	-0.55081200	-0.45854700	-0.02227800
C	0.02355000	-1.37641900	-1.22093200
C	-0.75040700	-2.44582400	-1.69046500
H	-1.68911800	-2.67510600	-1.19687500
C	-0.38000700	-3.22954500	-2.78186600
H	-1.01856300	-4.04690700	-3.10159300
C	0.78454300	-2.93395800	-3.47453500
H	1.06773100	-3.49960400	-4.35621700
C	1.59706100	-1.90492700	-3.00866500
H	2.52108400	-1.67700100	-3.53473300
C	1.26165500	-1.16875700	-1.87149900
C	2.27192900	-0.15651400	-1.39064400
H	1.80937200	0.82740300	-1.34717500
H	3.08679300	-0.09447500	-2.11482400
N	2.84795900	-0.40888700	-0.02406000
C	3.70789500	-1.69132400	0.03434400
C	4.51776800	-1.73809000	1.34232600
H	3.81912800	-1.88462700	2.17357600
H	5.14871900	-2.63127000	1.29189300
C	5.33166900	-0.49198800	1.62722900
H	6.07877700	-0.30974400	0.84506700
H	5.88186700	-0.60921100	2.56555900
C	4.34488600	0.65437100	1.73944700
H	4.84753500	1.59719200	1.97856000
H	3.64829400	0.43928000	2.55773200
C	3.53869300	0.89253400	0.45409100
C	2.47272400	1.93991100	0.80252900
H	1.73684800	1.54119200	1.49875000
H	1.95122400	2.34221500	-0.06854100
H	2.97540900	2.77844000	1.29273900
C	4.46134300	1.52596900	-0.60268000

H	3.93412500	1.77953600	-1.52391600
H	5.33120100	0.91937300	-0.85273800
H	4.83574000	2.46442000	-0.18409300
C	4.65319500	-1.81387100	-1.17143100
H	4.10352200	-2.03602200	-2.08759400
H	5.32615000	-2.65591700	-0.98938700
H	5.26975800	-0.93213700	-1.34186000
C	2.84207900	-2.96458100	0.06652300
H	2.26012600	-3.02142400	0.98598600
H	3.53193200	-3.81424100	0.06929700
H	2.17845800	-3.08091800	-0.78729200
C	-2.14430500	-0.81456700	0.27845100
C	-2.74111600	-1.42368900	1.37846600
F	-2.00816000	-1.85868500	2.41804600
C	-4.11292300	-1.63135600	1.49435300
F	-4.61466400	-2.21749000	2.58017800
C	-4.95664500	-1.22826400	0.47377500
F	-6.26882400	-1.41974600	0.56800400
C	-4.41321500	-0.62528800	-0.65278100
F	-5.20814200	-0.23933900	-1.64912800
C	-3.04177700	-0.43600700	-0.72362700
F	-2.57557300	0.12812900	-1.84696300
C	-0.52532200	1.16301500	-0.15625200
C	-0.39414500	1.86852200	-1.35024600
F	-0.17004100	1.23349100	-2.50611100
C	-0.45167700	3.25669000	-1.42937200
F	-0.29896200	3.87476400	-2.59924900
C	-0.68721000	4.00027300	-0.28265800
F	-0.75079300	5.32620000	-0.34104200
C	-0.87102200	3.34217400	0.92548700
F	-1.11909600	4.03755700	2.03373900
C	-0.80929800	1.95697800	0.95227200
F	-1.02288500	1.37589900	2.14707400
H	1.73343800	-0.61113900	0.66668400
C	0.59232500	-1.00618200	1.47182500
H	0.59462800	-2.09570100	1.41158200
H	-0.47035700	-0.77052500	1.64394500
C	1.10847300	-0.56032100	2.80634900
H	0.93901900	0.48042700	3.07508100
C	1.72917200	-1.35161500	3.68402700
H	1.87603500	-2.41195700	3.49177800
H	2.08438200	-0.96770800	4.63444600

N19_P

B	-0.60649400	-0.64338800	0.29948500
C	0.06209300	-1.71325000	-0.76302700
C	-0.63025800	-2.90457100	-1.03489400
H	-1.60069000	-3.05422200	-0.57007200
C	-0.15520100	-3.89854600	-1.88381700
H	-0.74665400	-4.79326400	-2.05413100
C	1.05318400	-3.71960100	-2.54584900
H	1.41762500	-4.45039300	-3.26013000
C	1.78671800	-2.57348900	-2.27566400
H	2.72059300	-2.41402800	-2.80950400
C	1.34818500	-1.61368800	-1.35274400
C	2.28232600	-0.43635700	-1.15718400
H	1.72881700	0.49411900	-1.19809800
H	3.02201800	-0.42467300	-1.95719100
N	3.10236400	-0.31956500	0.14277700
C	4.11786400	-1.48112700	0.38358400
C	5.03871400	-1.14112000	1.56918300
H	4.46233200	-1.20766900	2.49993500
H	5.78617700	-1.93917400	1.61542600
C	5.68616700	0.22858900	1.49732900
H	6.32396300	0.32084800	0.61070400
H	6.33216000	0.38241600	2.36596100
C	4.57100600	1.25851700	1.48735400
H	4.96731300	2.27859700	1.48435500
H	3.97270500	1.15587600	2.40200300
C	3.63622100	1.15338100	0.27606100
C	2.44029500	2.07318900	0.54937000
H	1.79644700	1.67348000	1.33914400
H	1.83718200	2.27985500	-0.33582800
H	2.83973000	3.02872700	0.90024800
C	4.34977500	1.62563500	-0.99252800
H	3.70454000	1.59918900	-1.87141800
H	5.27115500	1.08531200	-1.21009800
H	4.61972800	2.67227300	-0.82812300
C	4.94849100	-1.74730400	-0.87230000
H	4.34284100	-2.16887800	-1.67271400
H	5.70393300	-2.49344300	-0.61372100
H	5.47146600	-0.86752300	-1.24735200
C	3.36523600	-2.75843300	0.77840700
H	2.82769000	-2.62643600	1.72078300
H	4.12022600	-3.53335600	0.94146900
H	2.67113700	-3.11276900	0.01912700
C	-2.24787600	-0.84499000	0.40452900
C	-2.98916500	-0.90649500	1.57968400

F	-2.39098900	-0.88410600	2.78408100
C	-4.37887700	-0.98631400	1.61586200
F	-5.02460500	-1.04927100	2.78228700
C	-5.09459700	-0.99380700	0.43086000
F	-6.42448900	-1.07305000	0.44369600
C	-4.40767500	-0.91265100	-0.77280000
F	-5.08382400	-0.90608600	-1.92334400
C	-3.02310500	-0.82935000	-0.75694400
F	-2.42240800	-0.71612000	-1.95226100
C	-0.56140200	0.94376200	-0.21168700
C	-0.38942100	1.36984200	-1.53095800
F	-0.07064400	0.50591000	-2.50964300
C	-0.47616700	2.69767900	-1.94305900
F	-0.26555700	3.02752100	-3.21880000
C	-0.80086300	3.68110100	-1.02320600
F	-0.89744500	4.95516600	-1.39661000
C	-1.04935900	3.30959800	0.28997200
F	-1.40514700	4.23223600	1.18447700
C	-0.95348900	1.97143700	0.64769200
F	-1.30146700	1.69443400	1.91378100
H	2.41882000	-0.39648700	0.90994300
C	0.15920900	-1.09177500	1.73055200
H	1.10981700	-1.57103400	1.46876600
H	-0.44923300	-1.93620800	2.08038700
C	0.42164300	-0.20627500	2.90562000
H	-0.42768700	0.30973600	3.33823400
C	1.62093300	-0.04050500	3.47209200
H	2.49337200	-0.59282800	3.12065400
H	1.77394500	0.59763700	4.33646500

N20_TS

B	-1.02737300	-0.03423100	0.17494400
C	0.00734800	-0.26661400	1.41516100
C	-0.37720000	-1.14575700	2.43853800
H	-1.36020700	-1.60509300	2.38671300
C	0.43710300	-1.44869600	3.52373500
H	0.08703200	-2.13860200	4.28543400
C	1.69285500	-0.86165800	3.62962200
H	2.34306800	-1.08474800	4.46943600
C	2.10198200	0.02624700	2.64370900
H	3.08281600	0.49210500	2.71340100
C	1.28740200	0.32825500	1.54547600
C	1.87214900	1.31112700	0.54685700
H	2.58371600	1.95397100	1.07279400
H	1.09043100	1.95832600	0.14223500
N	2.60022400	0.66608300	-0.61619400
C	2.96863200	1.66503100	-1.71328400
C	3.85265200	2.76124200	-1.11574100
H	4.14135100	3.44564400	-1.91973700
H	3.25473800	3.35507200	-0.41700200
C	5.09728600	2.19761000	-0.43542400
C	4.96338800	0.77695800	0.07523400
C	6.09931700	0.21483700	0.65929200
C	6.10361700	-1.10576400	1.06980300
H	6.98695300	-1.53833400	1.52806300
C	4.96686600	-1.87879100	0.87401300
H	4.97750700	-2.91633900	1.18590600
C	3.80562300	-1.36165900	0.29594300
C	3.80920100	-0.00418500	-0.09169600
C	2.62316500	-2.29026400	0.07944200
H	1.72268100	-1.73699200	0.33649300
C	2.60853500	-3.51223800	1.00121700
H	1.63764400	-4.00535700	0.91110400
H	2.74484800	-3.22003100	2.04636800
H	3.37817200	-4.24324600	0.73168600
C	2.55419600	-2.73441100	-1.38636000
H	1.71215800	-3.41748600	-1.53243000
H	3.47438800	-3.25969000	-1.66249700
H	2.42594500	-1.89020200	-2.07150700
C	1.73792900	2.32597600	-2.34214100
H	1.12026200	2.87677500	-1.62976300
H	1.10878900	1.61410000	-2.87632100
H	2.10133600	3.05472400	-3.07213800
C	3.68354200	0.88514700	-2.82149000
H	3.01878000	0.11140300	-3.21974000
H	4.60413500	0.40771100	-2.48199800
H	3.92945600	1.56971600	-3.63793500
C	-2.13089100	-1.19450300	-0.04625800
C	-3.49447700	-0.97188000	-0.23677800
F	-4.01467400	0.26118200	-0.19224400
C	-4.41341800	-1.98598500	-0.49084700
F	-5.70353400	-1.69786900	-0.65425600
C	-3.98430400	-3.29955300	-0.57408500
F	-4.84749100	-4.27973500	-0.82157500
C	-2.63599000	-3.57831400	-0.40160400
F	-2.19489200	-4.83261200	-0.49546600
C	-1.75931500	-2.53798200	-0.13980200
F	-0.46416800	-2.89581200	-0.00373100

C	-1.64832400	1.47826300	0.22114000
C	-1.69724200	2.41449100	-0.80358700
F	-1.20429100	2.12463700	-2.02597700
C	-2.23462000	3.69042800	-0.66772700
F	-2.22166500	4.54229300	-1.69314500
C	-2.79469300	4.06442900	0.54175000
F	-3.32299900	5.27513100	0.69470200
C	-2.80486800	3.15541100	1.59169700
F	-3.36139300	3.49251400	2.75325200
C	-2.24340100	1.90043300	1.41127200
F	-2.33551800	1.05310000	2.44285100
H	6.98573100	0.83341900	0.77618500
H	5.93103100	2.19561900	-1.14758200
H	5.41089000	2.85068200	0.38593700
H	1.51513800	-0.00625400	-1.18727800
C	0.16361900	-0.31230600	-1.33932500
H	0.44334600	-1.15751900	-0.73164300
H	0.16172900	0.71818200	-1.01030700
C	-0.57865300	-1.85188600	-3.13623300
H	-0.30355200	-2.72577300	-2.55132800
C	-0.40760300	-0.61563400	-2.66134000
H	-0.72361800	0.23054700	-3.26688600
H	-1.00423300	-2.02605200	-4.11783000

N20_P

B	1.00838700	0.03534600	0.14212500
C	-0.00213100	-0.13803000	-1.16097300
C	0.43299300	-0.88054000	-2.27368500
H	1.40672500	-1.35720600	-2.22621900
C	-0.31029200	-1.01966900	-3.43960700
H	0.08512500	-1.60740900	-4.26306300
C	-1.54955700	-0.39682300	-3.55720700
H	-2.13711400	-0.48388600	-4.46532400
C	-2.01372700	0.35440100	-2.48831000
H	-2.97976400	0.85163200	-2.56275100
C	-1.26597100	0.48124800	-1.30887200
C	-1.90517700	1.30011300	-0.21543100
H	-2.58001900	2.04372100	-0.64226600
H	-1.17607700	1.79592500	0.42225200
N	-2.79613500	0.44302800	0.71262000
C	-3.43191300	1.22225300	1.89393400
C	-4.24716600	2.36708000	1.28786300
H	-4.87714400	2.75746300	2.09220100
H	-3.56724100	3.17944800	1.01643400
C	-5.11479900	1.96468400	0.07588500
C	-4.98179900	0.52308300	-0.36226900
C	-6.01165300	-0.07770500	-1.08301700
C	-5.90289500	-1.40009500	-1.49011200
H	-6.70725700	-1.86388900	-2.05140700
C	-4.76514700	-2.13352000	-1.17795800
H	-4.68932300	-3.16140500	-1.51229200
C	-3.70407800	-1.57812900	-0.45726100
C	-3.85498700	-0.24330300	-0.06188000
C	-2.46713600	-2.40578500	-0.14229100
H	-1.59420900	-1.74844900	-0.16931600
C	-2.19910900	-3.48288100	-1.19680900
H	-1.21051600	-3.91057800	-1.02620700
H	-2.21670800	-3.05330600	-2.20222100
H	-2.93064000	-4.29602100	-1.14101000
C	-2.54746000	-3.03376800	1.25547600
H	-1.63212800	-3.59808300	1.45332900
H	-3.40422700	-3.71136900	1.32840600
H	-2.64983500	-2.28341100	2.04806600
C	-2.36264300	1.77734300	2.83167600
H	-1.64201900	2.42793000	2.33599400
H	-1.82075900	0.98138100	3.34940000
H	-2.87949900	2.37458000	3.58818300
C	-4.31449800	0.25143100	2.68431900
H	-3.74583700	-0.62890100	2.99834300
H	-5.19353700	-0.07937200	2.13068000
H	-4.64923600	0.76456000	3.58916600
C	2.18046300	-1.12678100	0.13845600
C	3.54444100	-0.93125900	0.33367200
F	4.06185000	0.30230800	0.42910500
C	4.46626300	-1.96534100	0.46756400
F	5.76164900	-1.69781600	0.64276600
C	4.03626900	-3.28139200	0.42895800
F	4.90362900	-4.28524300	0.55674900
C	2.68413700	-3.53689200	0.26027500
F	2.23641800	-4.79569600	0.23194800
C	1.80896000	-2.46907000	0.12996000
F	0.50319300	-2.80704600	0.00456200
C	1.62282200	1.56089000	-0.05659200
C	1.30785700	2.69015800	0.68489300
F	0.47443200	2.61352000	1.75635100
C	1.77113800	3.97366400	0.41005800
F	1.40527000	5.00569000	1.17637300
C	2.61068400	4.17124900	-0.67058400

F	3.07090600	5.38728000	-0.96033800
C	2.96218200	3.07958200	-1.45542900
F	3.77083600	3.25379300	-2.50096200
C	2.46373000	1.82580800	-1.14035800
F	2.84929500	0.82172600	-1.94044900
H	-6.90051100	0.50084100	-1.31753700
H	-6.17038300	2.15528000	0.28614800
H	-4.86393600	2.60838500	-0.77582500
H	-2.16655300	-0.26377400	1.11702400
C	0.26393100	-0.20843200	1.60716300
H	-0.21920800	-1.19856900	1.58850200
H	-0.50178000	0.54552600	1.78282900
C	1.55701400	-1.19911200	3.52269500
H	1.15198100	-2.19171800	3.33471500
C	1.19663000	-0.15587900	2.77740500
H	1.63641300	0.81927400	2.98925300
H	2.27468100	-1.10466200	4.33093700

N21_TS

B	0.71577800	-0.52447300	-0.02685600
C	0.06202800	-1.47373100	-1.16322700
C	0.63439900	-2.73013100	-1.40755300
H	1.52203300	-3.01895800	-0.85213200
C	0.11784200	-3.63041300	-2.33611900
H	0.60481100	-4.58855200	-2.48783900
C	-1.01338500	-3.29345400	-3.06742000
H	-1.42345400	-3.97645100	-3.80418700
C	-1.62964800	-2.07061300	-2.82500700
H	-2.53987500	-1.81205500	-3.36023100
C	-1.11891700	-1.17535700	-1.88437700
C	-1.91674500	0.08363900	-1.63285600
H	-2.86547100	0.03987200	-2.17735400
H	-1.37828400	0.96379500	-1.98219500
N	-2.20764300	0.28733100	-0.17601900
C	-2.26580300	1.72718700	0.19300500
C	-3.60792000	2.34601600	-0.14690900
H	-3.83179200	2.19675200	-1.21194900
H	-3.56870400	3.42597000	0.02463200
C	-4.69991700	1.71396200	0.70889000
H	-4.53141800	2.03705100	1.74711300
C	-4.56896800	0.19878000	0.66683500
C	-5.63873400	-0.61000800	1.06818600
H	-6.54706100	-0.13661300	1.42582300
C	-5.58405500	-1.99358400	1.01023900
H	-6.44123100	-2.58222200	1.32474700
C	-4.43775900	-2.63400300	0.53748300
C	-3.35724400	-1.84634200	0.15581500
H	-2.46205100	-2.33382300	-0.21217800
C	-3.40265700	-0.44941500	0.22903900
C	-6.07951000	2.21148400	0.27134700
H	-6.08126900	3.30432000	0.22638200
H	-6.87165800	1.91227100	0.96008000
H	-6.32807700	1.82640500	-0.72231800
C	-4.34924000	-4.13614100	0.46720600
H	-5.31231900	-4.57536800	0.19610800
H	-4.05695300	-4.55379800	1.43596200
H	-3.60635100	-4.45120300	-0.26931600
C	0.83635800	1.07643800	-0.28896400
C	0.87172500	1.68804300	-1.54027500
F	0.69652400	0.98185600	-2.66418800
C	1.04792700	3.05852500	-1.71217100
F	1.03411100	3.59536900	-2.93082800
C	1.25076800	3.86795200	-0.60429700
F	1.42334900	5.17725300	-0.75193200
C	1.29658200	3.29336900	0.65952000
F	1.53513500	4.05313700	1.72641900
C	1.11596700	1.92344900	0.78269600
F	1.24536400	1.40503400	2.00948800
C	2.26728100	-0.94097300	0.35808100
C	3.15619600	-0.93643100	-0.72388500
F	2.69499200	-0.67279700	-1.95389000
C	4.51875400	-1.17144400	-0.62252700
F	5.29421000	-1.16098500	-1.70497500
C	5.07542500	-1.40396500	0.62725200
F	6.37910900	-1.62969400	0.75448900
C	4.25112200	-1.38959800	1.73831100
F	4.76465000	-1.59501600	2.95009200
C	2.88763800	-1.14869400	1.58834500
F	2.20035100	-1.13766900	2.74647800
H	-1.12212900	-0.25211200	0.52263100
H	-1.44881300	2.23012300	-0.32329700
H	-2.07063100	1.79762900	1.26905100
C	-0.48803900	-0.95783100	1.49125200
H	-0.99858900	-1.89596900	1.25348200
H	0.47737000	-1.26356700	1.87519000
C	-0.63087400	0.18027400	3.75016300
H	0.43945400	0.11598500	3.91264400
C	-1.19758700	-0.27361900	2.63432300

H	-2.28127000	-0.19700700	2.53540500
H	-1.22984300	0.62997100	4.53538500

N21_P

B	-0.92036200	-0.26187000	-0.33451600
C	0.11517200	-0.89957500	0.79063700
C	-0.20898300	-2.08952200	1.46474000
H	-1.14460400	-2.58664000	1.22857200
C	0.60163200	-2.66328300	2.44189000
H	0.28621700	-3.58163900	2.92871800
C	1.78939300	-2.04624000	2.81821200
H	2.41106400	-2.45845100	3.60654800
C	2.16384300	-0.88391800	2.15778600
H	3.10036300	-0.39236400	2.41538400
C	1.36711800	-0.34859000	1.14180300
C	1.92710100	0.82404300	0.39493100
H	2.62049100	1.40390100	1.00689800
H	1.15434300	1.47602700	-0.01582500
N	2.72807500	0.34307200	-0.82437000
C	3.02797300	1.45337100	-1.78428400
C	4.00915400	2.42418700	-1.15437400
H	3.54879300	2.88801400	-0.27532400
H	4.19739300	3.23737100	-1.86059100
C	5.33639300	1.75202000	-0.76809400
H	5.99000300	1.77020500	-1.65073200
C	5.16874600	0.28602300	-0.39941600
C	6.27920400	-0.47844300	-0.02949400
H	7.25405000	-0.00166100	0.01615500
C	6.16827900	-1.82983500	0.27219000
H	7.05318000	-2.38617600	0.56759700
C	4.93945700	-2.48848600	0.19596100
C	3.82622300	-1.74855300	-0.19474400
H	2.84930100	-2.22057800	-0.26276300
C	3.95177600	-0.39112900	-0.46817000
C	6.01837500	2.55195800	0.34673100
H	6.12581000	3.59912300	0.04961600
H	7.01249800	2.16501200	0.57899800
H	5.41851300	2.51457400	1.26152600
C	4.78820600	-3.94712800	0.53420400
H	4.00584400	-4.08167900	1.28699700
H	5.71919900	-4.36315000	0.92368400
H	4.49894900	-4.52602400	-0.34777600
C	-1.33940800	1.33332400	-0.08677100
C	-1.02959200	2.13828500	1.00825200
F	-0.24880700	1.68408800	2.00385400
C	-1.46166200	3.45201300	1.16109800
F	-1.09747800	4.16553800	2.22964800
C	-2.27875600	4.02081100	0.19897000
F	-2.70207000	5.27741400	0.32609800
C	-2.64490800	3.26019800	-0.90105500
F	-3.44012300	3.78437700	-1.83535500
C	-2.18119300	1.95566300	-1.01244900
F	-2.61921400	1.28350800	-2.09082200
C	-2.36715700	-1.05779900	-0.21925400
C	-3.12355900	-0.88878800	0.94247400
F	-2.63433200	-0.14760100	1.94921400
C	-4.36758900	-1.46399600	1.15656500
F	-5.03273700	-1.25913400	2.29501100
C	-4.91949300	-2.26830500	0.16889200
F	-6.11029300	-2.83779600	0.34974900
C	-4.20859600	-2.48225400	-0.99927900
F	-4.71804700	-3.26397400	-1.95386100
C	-2.96175500	-1.88423200	-1.16586900
F	-2.34346800	-2.17941200	-2.32562600
H	2.11702200	-0.34033100	-1.30930200
H	2.07691400	1.92738500	-2.03599700
H	3.44298400	0.98481700	-2.68161800
C	-0.28091800	-0.36524000	-1.86953000
H	-1.10419400	-0.28966600	-2.58312600
H	0.35834500	0.50369100	-2.10299300
C	1.57710700	-1.73344600	-2.91016900
H	1.99008800	-0.87021100	-3.43465700
C	0.46893600	-1.62734000	-2.16584000
H	0.06797700	-2.53683400	-1.71693300
H	2.07075500	-2.68469200	-3.08034500

P1_TS

P	1.26379300	0.00573400	0.23171300
C	0.88734300	-1.08183900	-1.16381800
C	-0.42213100	-1.59524300	-1.12839500
B	-1.45869600	-1.13432100	0.00658300
C	1.80599900	-1.45188200	-2.14854400
C	1.41232700	-2.34231600	-3.14004900
C	0.11482200	-2.85711600	-3.13358900
C	-0.78863100	-2.48701200	-2.14210300
O	-2.79123700	-1.69852100	-0.08468200
O	-1.62273700	0.31685800	0.10335300
C	-2.96162300	0.54861900	0.15357500

C	-3.61591000	1.75511000	0.27938500
C	-5.02008600	1.72634000	0.29446600
C	-5.71521500	0.52846500	0.18576100
C	-5.03905800	-0.69563600	0.05496600
C	-3.66094100	-0.65891700	0.03841500
H	-3.05713800	2.68075300	0.36339200
H	-5.56770300	2.65798500	0.39272400
H	-6.80015700	0.53443600	0.20018600
H	-5.56878300	-1.63772200	-0.03179600
C	1.11924300	1.74186800	-0.27618100
C	3.00055400	-0.25207100	0.70279500
C	4.04811900	0.39932700	0.03982500
C	5.36662000	0.13920000	0.39796500
C	5.64832600	-0.76706400	1.41830800
C	4.61081500	-1.41297300	2.08455600
C	3.28984700	-1.15477700	1.73036500
H	3.82906000	1.11765900	-0.74564200
H	6.17520900	0.64621700	-0.11831300
H	6.67818500	-0.96622500	1.69662600
H	4.82738700	-2.11346100	2.88421700
H	2.48178900	-1.65367400	2.25826600
C	1.75607800	2.73738100	0.47242900
C	1.60238600	4.07428700	0.12184900
C	0.80857200	4.42185800	-0.96871300
C	0.16922500	3.43148600	-1.71020400
C	0.32122000	2.09157600	-1.36878700
H	2.37508800	2.46868900	1.32417600
H	2.09961300	4.84422300	0.70264900
H	0.68754800	5.46580100	-1.23959100
H	-0.45134900	3.70089900	-2.55835500
H	-0.18695400	1.31734400	-1.93417500
H	2.81849700	-1.05638500	-2.13386700
H	2.11329000	-2.63832500	-3.91397800
H	-0.18926200	-3.55330100	-3.90978700
H	-1.79898900	-2.88627200	-2.14042800
H	0.11775900	-0.75521900	1.10650700
C	-0.82750200	-1.79316100	1.69101700
H	0.15767700	-2.17433500	2.00131800
C	-2.76366400	-1.27052300	3.18473300
H	-3.36532500	-2.05364300	2.73054500
C	-1.50795100	-1.05883000	2.79410000
H	-0.94516600	-0.24485600	3.25446900
H	-3.23606500	-0.65773800	3.94499900
H	-1.37853500	-2.70089600	1.42311000

P1_P

P	-1.25598700	-0.04999500	-0.39002900
C	-0.82608500	-1.21172800	0.91451800
C	0.46151500	-1.77070400	0.95872400
B	1.58023300	-1.52599300	-0.20255300
C	-1.80164500	-1.46831000	1.89234100
C	-1.48657600	-2.29530600	2.95934300
C	-0.21085900	-2.85865200	3.03858300
C	0.73579800	-2.60051700	2.05520000
O	2.93380300	-1.41070500	0.40960000
O	1.32232800	-0.14609600	-0.80532100
C	2.38656600	0.62260000	-0.45538500
C	2.58372400	1.96122000	-0.72695200
C	3.76167600	2.56058200	-0.25040900
C	4.69358500	1.81852300	0.46337600
C	4.48932500	0.45567000	0.73219200
C	3.32756300	-0.13419700	0.26602800
H	1.83661300	2.52526200	-1.27895800
H	3.94234000	3.61248500	-0.44743200
H	5.60033800	2.29669600	0.82086600
H	5.21399600	-0.13112600	1.28551500
C	-0.80495800	1.65149200	-0.01071500
C	-3.05572100	-0.08825100	-0.61191600
C	-3.89686900	0.84907800	-0.00668800
C	-5.27597700	0.74374100	-0.16567200
C	-5.81406100	-0.29052400	-0.92654000
C	-4.97702600	-1.22622600	-1.53113400
C	-3.59953100	-1.12728500	-1.37429300
H	-3.47492800	1.65932900	0.58113000
H	-5.92958100	1.47190500	0.30287100
H	-6.88932200	-0.36770900	-1.05088700
H	-5.39690500	-2.03070400	-2.12547900
H	-2.94662200	-1.86051800	-1.84241300
C	-1.21397400	2.68630600	-0.85893900
C	-0.84290100	3.99379100	-0.56927100
C	-0.07778600	4.26591000	0.56529800
C	0.32666600	3.23390200	1.40512600
C	-0.02866600	1.91914500	1.11666800
H	-1.81886500	2.47296100	-1.73657200
H	-1.15056500	4.80009600	-1.22659700
H	0.21075000	5.28818400	0.78736700
H	0.94292800	3.44422700	2.27218200
H	0.31660400	1.10318900	1.74518700

H	-2.79371700	-1.03124800	1.82082700
H	-2.22879000	-2.49950300	3.72377100
H	0.04001300	-3.50114300	3.87773200
H	1.73075200	-3.03173500	2.12809600
H	-0.77316600	-0.45070700	-1.63611800
C	1.50511800	-2.67332600	-1.35372900
H	0.47042000	-2.73931600	-1.73102600
C	3.53706200	-3.05185900	-2.78885300
H	3.81239100	-3.94902300	-2.24018900
C	2.44019300	-2.36469400	-2.47895700
H	2.21436300	-1.45867300	-3.04359300
H	4.20281000	-2.73775800	-3.58636700
H	1.74078700	-3.65316700	-0.91859600

P2_TS

C	-1.60522000	-1.04339600	2.44013700
C	-0.73826200	-0.75337400	1.38486000
C	0.62164400	-1.11143000	1.41094300
C	1.09363800	-1.75434800	2.55963900
C	0.24285500	-2.03816500	3.62436700
C	-1.10625000	-1.68493100	3.56790500
H	-2.65822400	-0.78015800	2.37834900
H	2.14467600	-2.02770300	2.60242400
H	0.62823200	-2.54080800	4.50682100
H	-1.76585100	-1.91399200	4.39877500
B	1.60139400	-0.72298500	0.20529200
P	-1.25246900	-0.01444600	-0.18287400
C	-2.92289800	-0.61480300	-0.56780500
C	-4.07005200	-0.02097900	-0.02748700
C	-3.04748300	-1.73501700	-1.39539500
C	-5.32486500	-0.55083200	-0.30810500
H	-3.98052700	0.86034300	0.60224300
C	-4.30585500	-2.26190300	-1.67200600
H	-2.15828800	-2.19253900	-1.82182800
C	-5.44287700	-1.67097800	-1.12879700
H	-6.21184100	-0.08901600	0.11324700
H	-4.39578500	-3.13057400	-2.31580100
H	-6.42380700	-2.08107000	-1.34679800
C	-1.35608400	1.79172600	-0.04501900
C	-2.16590800	2.51211100	-0.92923800
C	-0.57106000	2.46992200	0.89219900
C	-2.19803200	3.90153800	-0.86760700
H	-2.77383600	1.98950700	-1.66270400
C	-0.60647900	3.85915900	0.94477300
H	0.07688600	1.90722600	1.55607900
C	-1.41895600	4.57567900	0.06890700
H	-2.82919100	4.45667200	-1.55386500
H	0.00440600	4.38337900	1.67255900
H	-1.44403600	5.65967900	0.11544600
O	2.95491600	-1.10482100	0.28234700
O	1.52708100	0.65451100	-0.15760200
C	2.79478900	1.02517000	-0.70064800
C	3.01925200	2.50367400	-0.41279600
H	4.02417900	2.81364700	-0.71827000
H	2.28995100	3.09605700	-0.97313900
H	2.88919600	2.72261500	0.64905900
C	2.76781300	0.78601700	-2.21006500
H	2.65365100	-0.27686400	-2.43470800
H	1.91655900	1.32916700	-2.63131700
H	3.68307400	1.14876500	-2.68807600
C	3.76419900	0.05261200	0.05361400
C	4.99168100	-0.35614900	-0.74819200
H	5.58034800	0.52111000	-1.03766100
H	5.62467200	-1.00777900	-0.13961600
H	4.69673800	-0.90653900	-1.64387300
C	4.17172900	0.58812400	1.42668600
H	3.28572800	0.88299800	1.99792600
H	4.67964400	-0.21061000	1.97364300
H	4.84851800	1.44455500	1.35075000
H	-0.06533200	-0.71044100	-0.96706800
C	0.78012700	-1.96492500	-1.35054200
H	0.36876200	-1.65777100	-2.32650800
H	0.04371500	-2.62512900	-0.86777100
C	2.61619800	-2.85091700	-2.81944900
H	2.21587200	-2.35458800	-3.70087100
C	2.01516200	-2.75050400	-1.63071400
H	2.45853400	-3.26437500	-0.78028900
H	3.52461500	-3.43017100	-2.94944700

P2_P

C	-1.37691200	-1.59462100	1.87990500
C	-0.53606300	-1.11649100	0.85841600
C	0.84085400	-1.37833800	0.80719200
C	1.35203300	-2.16097300	1.85560100
C	0.54456300	-2.63697100	2.87996400
C	-0.82584400	-2.35552800	2.89808600
H	-2.44254600	-1.37783200	1.87498700
H	2.41712100	-2.37827500	1.84284200

H	0.97667000	-3.23515200	3.67753900
H	-1.45624400	-2.73212500	3.69674700
B	1.81387800	-0.88352500	-0.42127300
P	-1.26526800	-0.05649100	-0.38779000
C	-2.99335000	-0.56595300	-0.61326700
C	-4.06338000	0.29105800	-0.35267600
C	-3.23158700	-1.87308700	-1.05553000
C	-5.36930600	-0.15843000	-0.53772500
H	-3.87948600	1.30317300	-0.00515800
C	-4.53566100	-2.31318300	-1.24167200
H	-2.39771900	-2.54602500	-1.24261500
C	-5.60419700	-1.45512700	-0.98249100
H	-6.20117000	0.50704000	-0.33245700
H	-4.71950900	-3.32525000	-1.58624300
H	-6.62225900	-1.80219500	-1.12643200
C	-1.27531800	1.68400500	0.07110300
C	-1.49478900	2.65841800	-0.90510500
C	-1.08480500	2.04244800	1.40603700
C	-1.51510200	3.99974700	-0.54131000
H	-1.63037300	2.37371100	-1.94527400
C	-1.10837400	3.38821500	1.76171900
H	-0.89847200	1.27607400	2.15335500
C	-1.31933400	4.36321900	0.79005500
H	-1.67058200	4.76179800	-1.29751800
H	-0.94906500	3.67381200	2.79600900
H	-1.32666000	5.41176900	1.06944000
O	3.18074700	-0.63290700	0.03659900
O	1.31097400	0.45875400	-0.89337900
C	2.41964300	1.35225900	-0.89079300
C	1.94100000	2.77645900	-0.63949600
H	2.79500600	3.45339700	-0.52603200
H	1.34550900	3.12415500	-1.48997600
H	1.32163200	2.84002400	0.25845200
C	3.10837100	1.27603700	-2.25718300
H	3.55799200	0.29034100	-2.39890900
H	2.35810300	1.43296000	-3.03772000
H	3.88720900	2.03830100	-2.36314500
C	3.32173200	0.75808400	0.23508500
C	4.79720400	1.12263800	0.12060300
H	4.94256400	2.20852900	0.13996000
H	5.34519800	0.68814600	0.96160700
H	5.22195500	0.72141300	-0.80118200
C	2.80146600	1.14603700	1.62637800
H	1.71902100	0.99473700	1.69210800
H	3.28112400	0.50207200	2.36908300
H	3.02411000	2.19067500	1.86884500
H	-0.65659900	-0.19650900	-1.63458200
C	1.77097000	-2.01697100	-1.61484000
H	2.38305200	-1.65311500	-2.44931200
H	0.74004000	-2.12046700	-1.99505100
C	3.48581800	-3.84173800	-1.38364500
H	4.20928900	-3.29160600	-1.97973500
C	2.26860000	-3.34945400	-1.15844600
H	1.58573900	-3.93192600	-0.53567300
H	3.80581100	-4.79396200	-0.97228400

P3_TS

C	-1.46834700	-0.41237600	0.33537200
C	-0.11816600	-0.32729100	0.27730300
C	-2.24205400	-0.91219200	1.50432900
C	-3.05940300	-2.04631000	1.44245100
C	-2.18228100	-0.17951400	2.69514000
C	-3.80023500	-2.43605000	2.55457500
H	-3.10046800	-2.63015200	0.52822100
C	-2.92575600	-0.57019600	3.80375200
H	-1.54605600	0.70123700	2.73640800
C	-3.73859900	-1.69856500	3.73472600
H	-4.42286000	-3.32343400	2.50087700
H	-2.87211000	0.00856500	4.72017300
H	-4.31932500	-2.00463100	4.59887100
C	0.59434000	-1.07224600	1.37878700
C	1.38894500	-0.48445800	2.36211000
C	0.41940400	-2.45839600	1.49855700
C	2.05236100	-1.22077500	3.33625700
C	1.07543800	-3.21692700	2.45601600
C	1.90780300	-2.59748100	3.37679400
F	-0.42876000	-3.11876300	0.70717700
F	0.88998000	-4.53294900	2.51247400
F	2.53410400	-3.31470900	4.30148500
F	2.79651800	-0.60076000	4.24751500
F	1.48150800	0.84270800	2.46728000
B	0.83904700	0.51318400	-0.79143000
P	-2.32731500	0.20549500	-1.12717400
C	-3.74736800	1.23546000	-0.64172300
C	-3.76184600	2.59628400	-0.96198500
C	-4.82922100	0.67285100	0.04940500
C	-4.84501500	3.38772800	-0.58869800
H	-2.91879800	3.04033200	-1.48122200

C	-5.90595900	1.46980700	0.42015800
H	-4.82825900	-0.38440600	0.29767000
C	-5.91442000	2.82667500	0.10165600
H	-4.84853500	4.44388400	-0.83660300
H	-6.73837000	1.02984600	0.95941200
H	-6.75710500	3.44560400	0.39264500
C	-3.01424000	-1.25744200	-1.96930100
C	-2.24761400	-2.42869700	-2.01932400
C	-4.23436100	-1.19668800	-2.64917300
C	-2.71232900	-3.53154200	-2.72813400
H	-1.30089500	-2.48311200	-1.48972800
C	-4.69005800	-2.30434800	-3.35863800
H	-4.83170800	-0.29026500	-2.61735900
C	-3.93281600	-3.47218600	-3.39704300
H	-2.11827200	-4.43914700	-2.75372400
H	-5.64080100	-2.25383400	-3.87939700
H	-4.29260600	-4.33512900	-3.94800000
C	2.07512300	-0.36052300	-1.40193100
C	2.04609300	-1.74381200	-1.54198100
C	3.21691600	0.22812900	-1.95833400
C	3.07679900	-2.50264400	-2.08627900
C	4.27087100	-0.48049800	-2.51259900
C	4.20704400	-1.86629700	-2.56772700
C	1.23480500	1.91880800	-0.07348700
C	0.26923900	2.89856800	0.12782500
C	2.48629600	2.23359300	0.45422000
C	0.51831100	4.13766700	0.70341900
C	2.78649400	3.45512900	1.03720100
C	1.79713400	4.42285800	1.15213400
F	2.96760900	-3.82745600	-2.16825400
F	0.96381200	-2.44263200	-1.16647900
F	3.32433600	1.56574700	-2.02790800
F	5.32939500	0.15502600	-3.00958800
F	5.20345700	-2.56616400	-3.09759800
F	3.45590700	1.31087600	0.49950000
F	4.00355800	3.69645900	1.52096400
F	2.06894400	5.59640600	1.71424300
F	-0.45859900	5.03498500	0.83795200
F	-1.00922600	2.65990700	-0.22277700
H	-0.84862300	0.74663100	-1.70752900
C	0.22438100	1.15792300	-2.55357400
H	0.08569800	0.30773200	-3.23357500
C	-0.05170900	3.57696300	-3.10402700
H	0.99709600	3.76551000	-2.88687400
C	-0.57062900	2.35559700	-2.98942100
H	-1.62538100	2.20092900	-3.21930800
H	-0.65422000	4.42339600	-3.41656700
H	1.26098000	1.47446200	-2.63637000

P3_P

C	-1.44929600	-0.30581500	0.14217100
C	-0.09346400	-0.27205700	0.12393300
C	-2.28606300	-0.69880000	1.32349400
C	-3.03134800	-1.88094300	1.34612300
C	-2.36611000	0.18208100	2.40755800
C	-3.84522200	-2.17538200	2.43769700
H	-2.95669600	-2.58036600	0.51829400
C	-3.17705400	-0.11548700	3.49670800
H	-1.77616100	1.09521300	2.38656700
C	-3.92306300	-1.29271300	3.51066400
H	-4.41235300	-3.10058800	2.45157600
H	-3.22797900	0.57204600	4.33463300
H	-4.55645500	-1.52502400	4.36061600
C	0.48769000	-0.89052300	1.37420000
C	1.13340900	-0.17223400	2.37853100
C	0.31482500	-2.25608200	1.62733900
C	1.67443700	-0.77741400	3.50610400
C	0.85330800	-2.88716700	2.73823100
C	1.54997300	-2.14571800	3.68129900
F	-0.43071900	-3.01580700	0.81875700
F	0.67800100	-4.19405100	2.91757100
F	2.06071500	-2.73586300	4.75581000
F	2.27611200	-0.03876000	4.43402100
F	1.17085900	1.16140200	2.34984800
B	0.95895800	0.40608100	-0.99728900
P	-2.51052800	0.15293200	-1.23403400
C	-4.00942500	0.97134300	-0.65092000
C	-4.02304000	2.36860300	-0.58385900
C	-5.11461000	0.23047200	-0.21761000
C	-5.14657800	3.01856100	-0.08173900
H	-3.15937300	2.94492700	-0.89955600
C	-6.22995300	0.89039200	0.28289800
H	-5.09985800	-0.85407700	-0.26579300
C	-6.24544700	2.28224600	0.35055300
H	-5.15856700	4.10169900	-0.02762600
H	-7.08665500	0.31734600	0.62078500
H	-7.11845400	2.79374900	0.74291100
C	-2.95100700	-1.32110900	-2.18476500

C	-2.09441300	-2.42738000	-2.16451200
C	-4.10023300	-1.32278100	-2.98265900
C	-2.40008500	-3.53813000	-2.94595900
H	-1.20091400	-2.42185500	-1.54443100
C	-4.39404000	-2.43858600	-3.75813300
H	-4.76283900	-0.46204600	-2.99276800
C	-3.54537900	-3.54365100	-3.73835300
H	-1.73994400	-4.39841200	-2.93311200
H	-5.28479900	-2.44491300	-4.37696200
H	-3.77936200	-4.41330500	-4.34369300
C	2.34535600	-0.47901500	-1.15522200
C	2.36791800	-1.86641400	-1.12939100
C	3.53277100	0.07986200	-1.63432400
C	3.48228700	-2.64886300	-1.41393000
C	4.67411900	-0.64926900	-1.93320400
C	4.65579400	-2.03207100	-1.80927300
C	1.23010800	1.91164100	-0.35953800
C	0.25119800	2.89528000	-0.41651000
C	2.37322400	2.32426100	0.32687500
C	0.38800300	4.19456300	0.05073700
C	2.57201800	3.61157000	0.80621200
C	1.57419000	4.56451600	0.65990600
F	3.41464900	-3.97965600	-1.33780100
F	1.23809800	-2.55401300	-0.86227800
F	3.61231500	1.39972800	-1.87752900
F	5.77816200	-0.03860200	-2.36380500
F	5.73955600	-2.75129200	-2.09439800
F	3.33461900	1.44690500	0.64492500
F	3.69894200	3.92959200	1.44357300
F	1.74260300	5.80055100	1.12433200
F	-0.61697600	5.06816900	-0.06885600
F	-0.96986300	2.60888800	-0.93239300
H	-1.92215700	1.07207600	-2.09329000
C	0.42559300	0.46244600	-2.60579200
H	-0.36060600	-0.28553500	-2.78462000
C	0.95724500	2.72393500	-3.55104400
H	2.00340900	2.61039100	-3.27736500
C	0.07353600	1.76621900	-3.27693400
H	-0.96174300	1.93906500	-3.58186700
H	0.66651500	3.64325000	-4.04859900
H	1.26744300	0.06494900	-3.18010100

P4_TS

C	-0.05836900	-1.06262800	-1.39047000
H	-0.65594600	-1.18737000	-2.29877100
H	0.81455600	-0.45215800	-1.62213500
C	0.40658900	-2.38612200	-0.75372400
H	-0.46191500	-3.02362500	-0.54006700
H	1.00306200	-2.93985500	-1.48985500
P	-1.03811600	-0.30815800	-0.02399000
B	1.38766600	-2.19143100	0.54105900
C	-1.21451400	1.51984800	0.04583100
C	-2.01884900	2.00723700	1.10066700
C	-0.59322100	2.43759700	-0.82731100
C	-2.23605500	3.37944900	1.22041500
C	-0.83891100	3.80053100	-0.66048500
C	-1.66169900	4.29585900	0.34648600
H	-2.86696100	3.73805100	2.03057900
H	-0.36395700	4.49955900	-1.34493500
C	-2.73419500	-0.97023600	-0.31071200
C	-3.53909900	-0.47755300	-1.35856600
C	-3.23314800	-1.98872200	0.53051300
C	-4.81406600	-1.01628300	-1.54511100
C	-4.51360900	-2.49070400	0.30595800
C	-5.32033000	-2.02116300	-0.72782800
H	-5.42850100	-0.63202100	-2.35617600
H	-4.89166600	-3.27210900	0.96114100
C	-3.10611300	0.63171200	-2.28565200
H	-3.17224800	1.60719900	-1.79366500
H	-2.07326700	0.51099100	-2.62102200
H	-3.74520800	0.65171300	-3.17061400
C	-6.68900600	-2.60412200	-0.96146100
H	-7.20992000	-2.77606200	-0.01603400
H	-7.30260600	-1.94360400	-1.57770200
H	-6.61541200	-3.56811000	-1.47523200
C	-2.44145400	-2.55856700	1.68115400
H	-1.54024200	-3.07183700	1.33526700
H	-2.12219000	-1.77925700	2.37958200
H	-3.04450100	-3.27954800	2.23640400
C	0.35158000	2.04369700	-1.93429800
H	1.28478200	1.64690000	-1.52253900
H	-0.07018600	1.29062700	-2.60370700
H	0.60656800	2.91801600	-2.53569300
C	-1.88390400	5.77579600	0.51218700
H	-1.13472300	6.20526200	1.18531800
H	-1.80562800	6.29710000	-0.44469500
H	-2.86833700	5.98273300	0.93862300
C	-2.65345100	1.10539500	2.13255100

H	-3.47494600	0.51309200	1.71778400
H	-1.92084200	0.40620800	2.55072300
H	-3.04822100	1.70044900	2.95807700
H	0.06418500	-1.10110000	1.09548200
H	1.76529900	-3.26430900	0.93017100
C	3.74909300	-1.76483000	-0.49936000
C	2.62923400	-1.23026800	0.12928700
C	2.65501500	0.14851400	0.28489900
C	3.69630900	0.96101500	-0.13895300
C	4.79677000	0.37884300	-0.75236400
C	4.82411900	-0.99640500	-0.93345400
F	3.82370300	-3.08273100	-0.72865600
F	5.87477300	-1.56342400	-1.52784900
F	5.80844100	1.13502800	-1.17538100
F	3.63389300	2.28616800	0.01123100
F	1.60231800	0.77526900	0.85707500
C	0.78054200	-1.65438700	2.24361900
H	0.19030400	-0.79587600	2.59795600
H	0.24443800	-2.54960400	2.56504400
C	2.50637200	-0.44989500	3.60260900
H	1.88912500	0.44290300	3.65931700
C	2.10002400	-1.53511300	2.94774000
H	2.75145200	-2.40470600	2.88355500
H	3.47497300	-0.41387300	4.08958100

P4_P

C	-0.15072700	0.03409000	-1.16482500
H	-0.51766800	-0.00406500	-2.19586500
H	0.35625500	0.99043900	-1.01635000
C	0.76599600	-1.15085600	-0.79239200
H	0.14164100	-2.05064800	-0.70006700
H	1.37057800	-1.33951500	-1.68650900
P	-1.58430700	0.00771400	-0.05467200
B	1.79584200	-0.97895300	0.50995500
C	-2.59645200	1.50964900	0.12112300
C	-3.35976100	1.60182700	1.30799500
C	-2.61870200	2.55798300	-0.82258500
C	-4.14462700	2.73313900	1.51504700
C	-3.42470700	3.66574300	-0.56260700
C	-4.19167500	3.77634400	0.59451300
H	-4.73349400	2.79944000	2.42624500
H	-3.44786600	4.47216700	-1.29131300
C	-2.57729000	-1.49602500	-0.27610600
C	-3.51300700	-1.59168800	-1.32693900
C	-2.37837700	-2.57569700	0.61349700
C	-4.25804100	-2.75867300	-1.45544600
C	-3.15214800	-3.72392000	0.43258600
C	-4.09477000	-3.83551000	-0.58406300
H	-4.98076200	-2.83344600	-2.26457400
H	-3.00399700	-4.55922900	1.11155600
C	-3.70926500	-0.49071300	-2.33852300
H	-4.00520600	0.45360800	-1.87224500
H	-2.78912800	-0.31690600	-2.90676700
H	-4.48301000	-0.76803100	-3.05607600
C	-4.91062300	-5.08775400	-0.76110900
H	-5.97964100	-4.85741700	-0.77369000
H	-4.66947400	-5.57370000	-1.71129300
H	-4.72312000	-5.80255100	0.04193300
C	-1.37606000	-2.56871500	1.74525900
H	-0.35181600	-2.37755800	1.40566800
H	-1.62240000	-1.81592400	2.50294200
H	-1.38094600	-3.53894600	2.24398400
C	-1.80875200	2.56642700	-2.09642200
H	-0.74014200	2.67407700	-1.89287900
H	-1.94515500	1.65658100	-2.68431500
H	-2.11021300	3.40903600	-2.72047800
C	-5.02170300	5.00384200	0.85603900
H	-4.42311800	5.76698900	1.36373900
H	-5.39004900	5.43836100	-0.07592600
H	-5.87777800	4.77441700	1.49409500
C	-3.34332900	0.53893300	2.37992000
H	-3.58225700	-0.45350700	1.98500100
H	-2.36210700	0.48427600	2.86664100
H	-4.07666700	0.77384100	3.15245400
H	-1.03585900	-0.14793400	1.21162500
H	1.91909200	-2.07913100	1.01880200
C	4.44428700	-0.99658300	0.59368800
C	3.28445100	-0.51007000	-0.00628300
C	3.51659800	0.43224500	-0.99854200
C	4.77595300	0.86117300	-1.39990800
C	5.89532200	0.33320500	-0.77768300
C	5.72671300	-0.60350300	0.23201900
F	4.36698500	-1.88782000	1.59365100
F	6.80105800	-1.11251700	0.84350700
F	7.11970800	0.72380400	-1.14114500
F	4.91777100	1.77372600	-2.36866400
F	2.47417600	1.00950600	-1.64761300
C	1.32325400	0.08678500	1.70335400

H	0.98688900	1.06160800	1.30585400
H	0.47894500	-0.34096300	2.27212500
C	3.02810500	1.49932800	2.90851400
H	2.72952300	2.39981200	2.37621000
C	2.41842800	0.33558000	2.69122900
H	2.76987600	-0.54788600	3.22611200
H	3.85264200	1.59194000	3.60795000

P5_TS

P	-1.57305800	-0.75528100	-0.40544400
B	1.49441000	-0.65719800	-0.28805000
C	-0.60409900	-1.38022700	-1.85277600
C	0.89503300	-1.00294200	-1.77183600
C	-2.99788600	-1.90862700	-0.24267800
C	-4.99429100	-2.94927500	-1.13923600
C	-5.11782600	-3.76467500	-0.01215600
C	-4.18079700	-3.61805600	0.99883200
C	-3.12486100	-2.70232900	0.91351400
C	-2.25370700	0.95919900	-0.51845600
C	-2.05607700	1.83860700	-1.60714100
C	-2.51211200	3.15495200	-1.51004100
C	-3.15798100	3.64355000	-0.37952300
C	-3.36992400	2.75868600	0.67175700
C	-2.93085500	1.43557900	0.63096200
C	3.13071800	-0.79193000	-0.24136000
C	3.80053400	-1.88064200	-0.80763800
C	5.18012700	-2.02556200	-0.84177300
C	5.98019700	-1.04318700	-0.27975100
C	5.37547700	0.04969600	0.31785500
C	3.98773800	0.14940100	0.33434400
C	0.99977500	0.82913700	0.21409500
C	1.22662800	1.92412700	-0.62027000
C	0.74122000	3.20156500	-0.38226500
C	0.00545200	3.44448500	0.76739200
C	-0.19947700	2.40872700	1.66213400
C	0.30980400	1.14864000	1.37854000
C	-6.24122200	-4.76173200	0.08981300
C	-2.21728600	-2.64006000	2.12273300
C	-1.36841100	1.45906800	-2.89565700
C	-3.56096200	5.08947300	-0.28028300
C	-3.17224600	0.59471100	1.86037700
F	3.11243500	-2.90931100	-1.34220800
F	5.73620900	-3.09912400	-1.40207400
F	7.30420800	-1.15856800	-0.29937100
F	6.12504200	0.99267200	0.88655300
F	3.51391800	1.22222400	0.98370800
F	1.94819100	1.77394100	-1.74609100
F	0.94170800	4.18173300	-1.26286600
F	-0.52225900	4.64616300	0.99487700
F	-0.94663000	2.60556000	2.74990000
F	0.00318900	0.19418500	2.28087100
H	-1.03361500	-1.17013600	-2.83031400
H	-0.71159500	-2.46190700	-1.69410700
H	1.09575700	-0.15098300	-2.42258200
H	1.44623200	-1.83489700	-2.21421900
H	-5.72978000	-3.03342800	-1.93640200
H	-4.26563600	-4.22600600	1.89728100
H	-3.86954800	3.11039700	1.57091700
H	-6.25169700	-5.25080800	1.06585600
H	-7.20978900	-4.27656300	-0.06077500
H	-6.14147000	-5.53628700	-0.67670600
H	-1.48520200	-1.83414400	2.09988400
H	-1.67963700	-3.58773700	2.23693800
H	-2.82098000	-2.50709900	3.02592300
H	-1.90379700	0.66378400	-3.42148900
H	-1.32893600	2.32345900	-3.56057500
H	-0.34131300	1.12930700	-2.73077000
H	-4.35891100	5.23028500	0.45195700
H	-3.90163700	5.47477500	-1.24435200
H	-2.70296100	5.69013900	0.03702600
H	-3.62675100	1.20497100	2.64241300
H	-3.84070900	-0.24576800	1.65285700
H	-2.23727500	0.19671400	2.26223200
C	-3.96214000	-2.02868200	-1.27498700
C	-3.92510300	-1.18907900	-2.52849000
H	-3.84854900	-0.12271500	-2.29876800
H	-3.07858400	-1.46040700	-3.16583100
H	-4.83494300	-1.34408000	-3.11130100
H	-2.33589500	3.82529300	-2.34808100
H	-0.10605400	-1.22956600	0.46519500
C	1.00495900	-2.06039600	0.84131500
H	1.59820000	-2.70009300	0.18092400
C	1.31555600	-2.66899000	3.25145400
H	0.56155700	-3.45020300	3.19139800
C	1.63058000	-1.92069800	2.19788100
H	2.39258800	-1.15024900	2.30542300
H	1.80419700	-2.53223600	4.20997800
H	0.10726700	-2.68897100	0.95221600

P5_P

P	-1.80885200	-0.79427000	-0.28219200
B	1.62797800	-0.88338100	0.12060800
C	-0.57310700	-1.74586400	-1.23991400
C	0.88298000	-1.27127900	-1.31337000
C	-3.32573000	-1.79694000	-0.27790600
C	-5.40065100	-2.48263000	-1.29090700
C	-5.64480200	-3.37593800	-0.25239000
C	-4.70208000	-3.47360500	0.77088200
C	-3.54002000	-2.70674500	0.78226400
C	-2.20245100	0.95756100	-0.55173300
C	-1.83448900	1.69921700	-1.68572700
C	-2.19782500	3.04775900	-1.74648400
C	-2.89658000	3.67587300	-0.72381300
C	-3.25748700	2.91539600	0.39032100
C	-2.92520100	1.57108300	0.50564900
C	3.26107200	-0.86576100	-0.16090000
C	3.89196200	-1.99175200	-0.69371100
C	5.25445900	-2.08882500	-0.94433900
C	6.07953600	-1.01785800	-0.63987700
C	5.51577800	0.11964800	-0.08713800
C	4.14505000	0.16787400	0.14541200
C	1.07045800	0.63033600	0.52677400
C	1.27501200	1.68630100	-0.36632800
C	0.75539800	2.96134000	-0.22029400
C	-0.00665700	3.25682700	0.90024700
C	-0.21982000	2.26596400	1.83951100
C	0.30661200	0.99292600	1.63166400
C	-6.87627000	-4.24159400	-0.24024400
C	-2.56647700	-2.88959200	1.92327100
C	-1.03010200	1.16862700	-2.84495800
C	-3.20197700	5.14668500	-0.77932700
C	-3.30403100	0.84913700	1.77760200
F	3.18299100	-3.10125700	-0.99298100
F	5.77491000	-3.20248000	-1.46625400
F	7.39118000	-1.08653600	-0.86711700
F	6.29362800	1.15726800	0.22984200
F	3.71531100	1.30802600	0.71241100
F	2.01143700	1.49329800	-1.47684500
F	0.92853700	3.88592400	-1.16824900
F	-0.56421800	4.46096800	1.04471100
F	-1.01154200	2.51269500	2.88966000
F	-0.07542300	0.08019100	2.55532200
H	-1.03101000	-1.94560300	-2.21685800
H	-0.62647700	-2.69593800	-0.68655500
H	0.96543900	-0.44845300	-2.02735900
H	1.40850300	-2.11499900	-1.77023300
H	-6.12042600	-2.39935000	-2.10131300
H	-4.87331600	-4.17336900	1.58522000
H	-3.79302000	3.39179300	1.20731800
H	-7.32205600	-4.27560500	0.75706500
H	-7.62751400	-3.87428800	-0.94189100
H	-6.62541400	-5.26900800	-0.52233100
H	-2.44400100	-1.97478400	2.51474000
H	-1.57183100	-3.18829400	1.57623400
H	-2.92343700	-3.66920300	2.59757800
H	-1.44767300	1.53610100	-3.78594500
H	0.00128200	1.53079500	-2.77445200
H	-0.99131500	0.08240300	-2.89622900
H	-4.15524400	5.37371500	-0.29647000
H	-3.23514200	5.50959600	-1.80853800
H	-2.41719900	5.69615800	-0.25056100
H	-3.88457500	1.51255700	2.41952100
H	-3.90737700	-0.04356900	1.58418400
H	-2.41556700	0.55210500	2.34548800
C	-4.25489900	-1.68769300	-1.32906200
C	-4.07533100	-0.74423500	-2.49320900
H	-4.29218900	0.28957700	-2.20783100
H	-3.05704100	-0.76532200	-2.89309300
H	-4.74875100	-1.02195100	-3.30587900
H	-1.89726800	3.62311400	-2.61886900
H	-1.36046000	-0.83516700	1.03414100
C	1.34203600	-2.04496600	1.25665000
H	1.55488000	-3.00354900	0.75896400
C	1.77088600	-1.87821200	3.74296600
H	0.71008000	-1.86573700	3.98162600
C	2.19690900	-1.96172400	2.48441400
H	3.27312400	-1.96269500	2.30381600
H	2.46292600	-1.81043900	4.57614200
H	0.29070100	-2.08654000	1.57174300

P6_TS

P	1.88395100	-0.62343900	0.30566900
C	1.37914700	-1.00091200	2.03646900
H	2.25658200	-1.17647600	2.66295500
H	0.80530200	-0.17565400	2.45874100
C	0.47443000	-2.23415300	1.94583100

H	0.88528700	-2.94083000	1.20721200
H	0.51234500	-2.76735400	2.90227900
C	-0.98181200	-1.87724700	1.62758800
H	-1.40399600	-1.40553000	2.51926600
H	-1.51824000	-2.82562300	1.53416700
B	-1.40176600	-0.97675200	0.33067400
C	2.27315800	1.15136200	0.00564500
C	2.22102300	2.18518400	0.96742200
C	2.05171700	2.00316600	2.46116800
H	2.45801600	2.87769900	2.97396700
H	2.57280300	1.12777600	2.84279900
H	1.00025400	1.92291300	2.75599800
C	2.33991200	3.50891500	0.53102900
H	2.27041700	4.30282400	1.27195100
C	2.49893900	3.85037900	-0.80631400
C	2.47360000	5.28616700	-1.25177000
H	3.09608100	5.44323300	-2.13577200
H	2.81360900	5.95768700	-0.46026100
H	1.44640600	5.56401400	-1.50969900
C	2.61705000	2.81346300	-1.72864200
H	2.76717200	3.04977000	-2.77918600
C	2.50671000	1.47896900	-1.35277000
C	2.62537300	0.42756900	-2.42931300
H	1.70864600	-0.16655500	-2.49597900
H	3.46382900	-0.25160400	-2.24484000
H	2.77729700	0.89886700	-3.40167900
C	3.46512400	-1.57933600	0.09772900
C	4.63461400	-1.23761800	0.81843200
C	4.72664400	-0.08894300	1.79183700
H	5.75911500	0.03529400	2.12285900
H	4.12384100	-0.26939700	2.68653000
H	4.39905100	0.85333200	1.34933000
C	5.79367500	-1.99385000	0.63820900
H	6.68479300	-1.71544700	1.19566500
C	5.85024000	-3.08172100	-0.22547100
C	7.10539300	-3.89943000	-0.37107900
H	7.08672200	-4.75682600	0.30961400
H	7.99302900	-3.30850300	-0.13489100
H	7.20882800	-4.28810200	-1.38685400
C	4.69771000	-3.39981600	-0.93519800
H	4.71443600	-4.23897300	-1.62640100
C	3.51515200	-2.67426900	-0.79341200
C	2.34219000	-3.10691400	-1.63454400
H	2.03861700	-2.31824600	-2.32964700
H	1.47710400	-3.37012600	-1.02373600
H	2.60339800	-3.98455900	-2.22803600
C	-1.02642100	0.63541000	0.30253900
C	-0.70148200	1.36509100	-0.84365100
F	-0.39278300	0.73726300	-1.99296100
C	-0.61374300	2.74783500	-0.89523700
F	-0.29783900	3.37213600	-2.03167600
C	-0.80267600	3.48516200	0.26182500
F	-0.63466100	4.80618000	0.25132200
C	-1.10077800	2.81897400	1.43770000
F	-1.21532700	3.49736100	2.58167100
C	-1.21314000	1.43319600	1.42907400
F	-1.48033300	0.87533200	2.62418100
C	-3.05009100	-1.02217000	0.10521000
C	-3.97480100	-1.61531000	0.97011400
F	-3.60808400	-2.23036100	2.10297000
C	-5.35113900	-1.62120800	0.75530000
F	-6.16061300	-2.21813400	1.63017400
C	-5.88295300	-1.00770000	-0.36425400
F	-7.19495200	-1.00809000	-0.58287000
C	-5.01850300	-0.38915600	-1.25304500
F	-5.49835100	0.21674400	-2.33928700
C	-3.65516700	-0.40327200	-0.99465100
F	-2.90594200	0.22809700	-1.91918700
H	0.34509400	-1.22524900	-0.49662600
C	-0.79015100	-1.83318400	-1.19111700
H	0.12101500	-1.79285300	-1.80996100
C	-2.25405600	-3.83830400	-1.48050300
H	-2.97639800	-3.26292700	-2.05466700
C	-1.14435500	-3.27755200	-1.00702900
H	-0.46545200	-3.88912500	-0.41326400
H	-2.48176100	-4.88397300	-1.30329300
H	-1.47689200	-1.32553400	-1.86672800

P6_P

P	2.06305100	-0.56770800	0.48220900
C	1.17555000	-1.11373200	1.97410000
H	1.93432100	-1.29909900	2.73964000
H	0.53431300	-0.30066700	2.31656900
C	0.30923800	-2.36574800	1.69985100
H	0.65089200	-2.89695300	0.80065200
H	0.48729600	-3.05868500	2.52901400
C	-1.18199100	-2.01772100	1.59903000
H	-1.45240400	-1.46706300	2.50647700

H	-1.72434200	-2.96727800	1.66946900
B	-1.70943900	-1.30223300	0.20871800
C	2.37709900	1.21110000	0.30848300
C	2.28550900	2.13867600	1.36956500
C	1.91300900	1.80741600	2.79579800
H	2.25905800	2.60657600	3.45362900
H	2.34933900	0.87593400	3.15521300
H	0.82514000	1.74588900	2.91101700
C	2.50475900	3.48716800	1.08678900
H	2.42119000	4.20379100	1.89965800
C	2.79491200	3.94707800	-0.19383300
C	2.94633000	5.41561100	-0.47395500
H	3.68340500	5.59685400	-1.25943600
H	3.24552700	5.96457200	0.42112900
H	1.98663500	5.81817800	-0.81241800
C	2.88165800	3.01263300	-1.22247100
H	3.09304900	3.34985900	-2.23356900
C	2.67934600	1.65388500	-1.00214200
C	2.73256700	0.72781600	-2.19211300
H	1.74005600	0.32290300	-2.42260700
H	3.42598800	-0.10500700	-2.03779400
H	3.06441800	1.27623000	-3.07440400
C	3.56172300	-1.56862500	0.23857700
C	4.69441000	-1.33966000	1.04985600
C	4.72053900	-0.31142000	2.15305900
H	5.70619600	-0.28472300	2.61993400
H	3.99915800	-0.55034600	2.94131500
H	4.49597300	0.69326200	1.78445700
C	5.84088300	-2.09875500	0.83237200
H	6.71347600	-1.91617700	1.45447500
C	5.90124900	-3.07669300	-0.15731800
C	7.14394900	-3.90163200	-0.35501900
H	7.11247200	-4.79589900	0.27572100
H	8.04006000	-3.33816600	-0.08632700
H	7.23889000	-4.23149700	-1.39174500
C	4.77141400	-3.28656900	-0.94275200
H	4.80290100	-4.04456600	-1.72117700
C	3.59409500	-2.55861600	-0.77094200
C	2.43087000	-2.89031200	-1.67696700
H	2.06080000	-2.02213200	-2.23162700
H	1.58426700	-3.30678600	-1.12402700
H	2.73731900	-3.63678200	-2.41099800
C	-1.09598400	0.25163300	-0.01896900
C	-0.66397000	0.76861700	-1.24105500
F	-0.49621000	-0.03871900	-2.30454200
C	-0.33452600	2.09940700	-1.46897700
F	0.12993300	2.48992200	-2.66055000
C	-0.41696100	3.01087400	-0.42993000
F	-0.07125000	4.28775900	-0.61013000
C	-0.80368600	2.55578200	0.81934400
F	-0.79672300	3.39363400	1.86242900
C	-1.12949900	1.21501000	0.98915000
F	-1.47292700	0.87122400	2.24860200
C	-3.35107100	-1.05155200	0.25737000
C	-4.22793200	-1.45614500	1.26211200
F	-3.79902300	-2.12252700	2.34808600
C	-5.60038500	-1.22009200	1.23961700
F	-6.37677400	-1.64470700	2.24054600
C	-6.16466400	-0.53938100	0.17510700
F	-7.47684000	-0.30606600	0.13554200
C	-5.34118900	-0.10633500	-0.85318700
F	-5.86339700	0.55228800	-1.89076500
C	-3.97975000	-0.36645700	-0.78671900
F	-3.25727300	0.08588500	-1.82599400
H	1.23673200	-0.90750100	-0.59007900
C	-1.31809500	-2.36357400	-1.01437900
H	-1.35380100	-3.35619900	-0.53426300
C	-1.72317200	-2.43131300	-3.50464200
H	-0.67026600	-2.27227400	-3.72906700
C	-2.16021700	-2.47085900	-2.24841900
H	-3.22683300	-2.62798300	-2.07796800
H	-2.39590400	-2.53274100	-4.34996200
H	-0.27563500	-2.23207700	-1.33498900

P7_TS

P	-1.82908400	-0.41736900	-0.12814100
B	1.37362000	-0.07765100	0.17046000
C	-0.77608500	-1.11023100	-2.94408100
H	-1.46425100	-1.88285000	-3.29513500
C	-0.64937900	-0.99791400	-1.41147100
H	-0.32333200	-1.96653200	-1.02943100
C	0.52389600	0.01844900	-1.24482400
H	0.05472200	1.01191800	-1.20619600
C	1.20462000	-0.04449400	-2.65275300
H	2.28372100	0.11302400	-2.64475200
C	0.41739500	0.94310100	-3.53351100
H	0.86203100	1.01476000	-4.53115400
H	0.39177500	1.94947400	-3.10526200

C	-0.99384900	0.27649500	-3.59901200
H	-1.75739300	0.86529200	-3.08403800
H	-1.32522800	0.15247600	-4.63354300
C	0.71173200	-1.37078600	-3.26397700
H	0.90501400	-1.42139000	-4.34054700
H	1.09932900	-2.27201500	-2.78939600
C	-3.04531600	0.78968200	-0.80685700
C	-4.11528000	0.35548600	-1.62480300
C	-5.02537300	1.29313000	-2.10661700
H	-5.84162300	0.95032800	-2.73819700
C	-4.91740500	2.65110300	-1.81338100
C	-3.85486700	3.06177700	-1.01741600
H	-3.74438300	4.11733000	-0.78213000
C	-2.91646300	2.16230600	-0.50619600
C	-4.29983300	-1.08056300	-2.03931200
H	-5.13962900	-1.16974100	-2.73055500
H	-4.48963300	-1.73422800	-1.18400900
H	-3.40775600	-1.44979500	-2.55199600
C	-5.93451800	3.63407200	-2.32875500
H	-5.54548000	4.65411300	-2.30934600
H	-6.83981400	3.60956300	-1.71365200
H	-6.22805500	3.39593200	-3.35412900
C	-1.79743000	2.72351400	0.33173100
H	-2.01136800	3.75817500	0.60846800
H	-0.84890600	2.71941800	-0.21639900
H	-1.64634400	2.15197400	1.24893800
C	-2.75471200	-1.74205600	0.74794400
C	-3.34062300	-1.32677600	1.97104700
C	-4.00798100	-2.25664900	2.76381100
H	-4.45792200	-1.92183800	3.69558000
C	-4.10386700	-3.59874000	2.40399600
C	-3.49854100	-3.99586600	1.21757000
H	-3.54970100	-5.04149600	0.92308800
C	-2.82203400	-3.10316800	0.38176700
C	-3.24760600	0.09583900	2.47024300
H	-3.72761500	0.80344300	1.78674500
H	-2.20382100	0.41006800	2.58718500
H	-3.73423300	0.18672900	3.44283100
C	-4.85155100	-4.57851900	3.26886500
H	-4.58516400	-5.60854800	3.02310800
H	-5.93218300	-4.46997600	3.13139500
H	-4.63773800	-4.41128900	4.32757500
C	-2.19313000	-3.68553600	-0.86087300
H	-2.66817300	-4.63746900	-1.10625400
H	-1.12552900	-3.88375800	-0.71269300
H	-2.29043400	-3.02872300	-1.72412200
C	2.77727400	-0.90781500	-0.03238500
F	4.23024200	0.78516400	0.81331400
C	4.05482100	-0.40448200	0.22583000
F	6.41291300	-0.53840900	0.20940500
C	5.23146600	-1.09153000	-0.05734400
F	6.28951800	-3.03782500	-0.86738000
C	5.17502000	-2.36558200	-0.59786600
F	3.84393500	-4.16980300	-1.32511400
C	3.93349000	-2.93471900	-0.83320300
F	1.63017500	-2.86905100	-0.75766900
C	2.78924400	-2.20704700	-0.53992000
C	1.53280000	1.44718200	0.73892300
F	2.89600400	1.91989200	-1.13053000
C	2.25918100	2.35691400	-0.03175200
F	3.09642400	4.51248400	-0.52881200
C	2.37308800	3.70838000	0.24880300
F	1.78913400	5.52463600	1.63959300
C	1.71047400	4.22734000	1.35489000
F	0.24947000	3.86265400	3.16498200
C	0.94886900	3.37857700	2.13733200
F	0.00538300	1.32389400	2.57432800
C	0.87193400	2.02618500	1.81472600
H	-0.32460800	-0.31750500	0.86328900
C	0.60939500	-1.14647900	1.56468000
H	-0.35480900	-1.17491500	2.09761600
H	0.66545200	-2.11818800	1.06748400
C	1.31291800	-0.83071600	3.95250400
H	0.28376200	-0.71487900	4.28196900
C	1.62262900	-1.02291500	2.67322000
H	2.66791200	-1.12376800	2.39641800
H	2.08438900	-0.77075300	4.71253600

P7_P

P	-2.00443900	-0.47579100	-0.21951300
B	1.50209000	-0.09268600	0.25748100
C	-0.78796400	-0.87277500	-2.89463000
H	-1.44046900	-1.65148600	-3.29970900
C	-0.62779600	-0.89288300	-1.35645400
H	-0.30498800	-1.88413000	-1.03084000
C	0.52347400	0.13295000	-1.09918400
H	0.03059600	1.09562400	-0.91092800
C	1.12801000	0.27844500	-2.53367000

H	2.19518900	0.50232500	-2.53344900
C	0.25884200	1.30413800	-3.28253700
H	0.67638300	1.52998000	-4.26879500
H	0.16778500	2.24756600	-2.73573600
C	-1.10272900	0.55141500	-3.41621500
H	-1.90853000	1.04359000	-2.86689800
H	-1.42215800	0.49253800	-4.46043700
C	0.70102800	-1.00374600	-3.27362500
H	0.85164900	-0.92563300	-4.35527700
H	1.15944300	-1.92106200	-2.91178800
C	-3.32121700	0.62637300	-0.82421100
C	-4.37182100	0.11632000	-1.61409400
C	-5.38658700	0.97731500	-2.02177200
H	-6.19313200	0.58264200	-2.63438500
C	-5.39000500	2.32625100	-1.67395200
C	-4.33171500	2.81166400	-0.90970400
H	-4.31044500	3.86608600	-0.64753600
C	-3.28629600	1.99749600	-0.47314100
C	-4.41470600	-1.31949600	-2.06739800
H	-5.29030800	-1.49551000	-2.69396500
H	-4.45719000	-2.01509800	-1.22429900
H	-3.53092700	-1.56134800	-2.66657600
C	-6.51469600	3.23138800	-2.09855900
H	-6.17196400	4.26198700	-2.21153100
H	-7.31181000	3.22697400	-1.34812600
H	-6.94956400	2.90449100	-3.04549700
C	-2.17356200	2.65202000	0.31463900
H	-2.38639900	3.71472000	0.44006800
H	-1.21036300	2.57353200	-0.20033900
H	-2.04271000	2.22963700	1.31601900
C	-2.67249300	-1.86039100	0.75585000
C	-3.20220700	-1.52152800	2.02515900
C	-3.68761100	-2.53815800	2.84110100
H	-4.09529900	-2.27587100	3.81396400
C	-3.66018100	-3.87591500	2.45143800
C	-3.13455800	-4.18273900	1.20152700
H	-3.10712100	-5.22036200	0.87835800
C	-2.62900500	-3.20667600	0.34083500
C	-3.23862900	-0.10694700	2.55096200
H	-3.73123300	0.58109700	1.85666100
H	-2.22807300	0.27049200	2.74697200
H	-3.78772800	-0.07359300	3.49275700
C	-4.16128400	-4.95527800	3.37233400
H	-3.39660200	-5.20599400	4.11425300
H	-4.40408300	-5.86637700	2.82200000
H	-5.05194300	-4.62930100	3.91464100
C	-2.05154400	-3.69147900	-0.96796200
H	-2.53543800	-4.62577600	-1.25961400
H	-0.97949300	-3.89469300	-0.86748100
H	-2.17927500	-2.98282900	-1.78495300
C	2.96101500	-0.76972800	-0.13038000
F	4.36738900	0.81365600	0.96168600
C	4.22396900	-0.30368200	0.23515500
F	6.58624400	-0.43902900	0.29105200
C	5.41302000	-0.95490000	-0.07874500
F	6.51027900	-2.79930500	-1.06513500
C	5.38090100	-2.16107900	-0.76045600
F	4.08530400	-3.88000000	-1.72651500
C	4.15137500	-2.70149400	-1.10125300
F	1.83968400	-2.64952700	-1.07011900
C	2.99690500	-2.00930800	-0.76374600
C	1.62332500	1.46827300	0.80553400
F	3.15172600	2.04410700	-0.91335900
C	2.37568700	2.42202500	0.11706200
F	3.11921100	4.62980700	-0.30926900
C	2.36123400	3.78251300	0.38845100
F	1.46729800	5.57734600	1.64131800
C	1.51926500	4.27133800	1.37912400
F	-0.16398800	3.82511300	2.97057800
C	0.71435800	3.37985900	2.06534500
F	-0.12688700	1.26573300	2.42692900
C	0.78450500	2.02291100	1.76431500
H	-1.38674800	0.31031500	0.74997800
C	0.88679000	-1.13258800	1.40191700
H	-0.13374900	-0.92357500	1.73996000
H	0.85791200	-2.13543900	0.94991700
C	1.36713300	-0.91963900	3.87130200
H	0.34584900	-0.62242400	4.09628000
C	1.74950600	-1.19939000	2.62687600
H	2.78976400	-1.48274100	2.45912600
H	2.06054000	-0.96841700	4.70459300

P8_TS

C	-0.49023700	-1.19362700	-0.90409700
H	-0.65692300	-0.65057600	-1.83805700
H	0.02819200	-2.12068100	-1.15301100
P	-2.04450700	-1.56992100	-0.00611700
B	0.33714000	-0.25385000	0.16496200

C	-3.55963000	-0.80910600	-0.85071000
C	-3.20441600	0.55817100	-1.45053100
H	-2.44360900	0.48568100	-2.23275000
H	-4.11054200	0.97064700	-1.90833500
H	-2.86458700	1.26765700	-0.69612000
C	-4.62568500	-0.59510400	0.23507800
H	-5.50948900	-0.12688600	-0.21276700
H	-4.94353100	-1.53289500	0.69772000
H	-4.25061700	0.06531100	1.02295300
C	-4.11368500	-1.67593400	-1.98641600
H	-4.50040900	-2.63452200	-1.63662500
H	-4.94521700	-1.14175600	-2.46063700
H	-3.35629300	-1.86149500	-2.75428300
C	-2.17850500	-3.45294100	0.06531500
C	-1.01220500	-3.92347200	0.95208800
H	-0.03780600	-3.65936400	0.53121100
H	-1.08091600	-3.51051000	1.96292900
H	-1.05255400	-5.01517100	1.03527300
C	-3.48599900	-3.86586800	0.75310100
H	-3.59926200	-3.37677400	1.72642900
H	-4.36850500	-3.64084100	0.15002600
H	-3.47267300	-4.94832600	0.92356300
C	-2.04446900	-4.14728700	-1.29676800
H	-1.97326800	-5.22914500	-1.13377900
H	-2.89840700	-3.96970000	-1.95018400
H	-1.13754200	-3.83569800	-1.82307300
C	1.94363100	-0.28513600	-0.08157700
C	2.61051200	-1.49438100	-0.26947400
C	2.78095300	0.83059000	-0.07651400
C	3.97952900	-1.61224700	-0.46827000
C	4.15347600	0.76369700	-0.28108400
C	4.76005500	-0.46677400	-0.48123900
F	1.93750600	-2.66458500	-0.21975700
F	4.54295500	-2.80807900	-0.63907900
F	4.89475700	1.87036400	-0.26750000
F	2.29791300	2.05522200	0.16082200
F	6.07304100	-0.54853300	-0.67085400
C	-0.35216100	1.22523500	0.19631900
C	-1.21791600	1.74975100	1.15033700
C	-0.17242100	2.04058500	-0.92031500
C	-1.82322700	2.99953600	1.04152600
C	-0.75849400	3.28560400	-1.08141400
C	-1.59338400	3.77376200	-0.08331900
F	0.60836100	1.61133900	-1.92301600
F	-0.53605000	4.01191100	-2.17448500
F	-2.16852900	4.96528800	-0.21012900
F	-2.63567800	3.44041200	2.00153200
F	-1.56935200	1.04658000	2.24729400
H	-0.97039800	-0.97231400	1.27376900
C	0.27462400	-0.97610000	1.92740600
H	-0.52496400	-1.29896700	2.61980800
C	2.33663700	-0.09174600	3.04176400
H	2.92870500	-0.93659300	2.69721300
C	1.04880300	0.03204200	2.73517600
H	0.49077900	0.88791400	3.10043700
H	2.84330400	0.65394000	3.64495700
H	0.84940900	-1.88565200	1.73853700

P8_P

C	0.46248500	1.22210100	-0.63761100
H	0.49019900	0.71576000	-1.60714300
H	-0.01171000	2.19329300	-0.78444700
P	2.14223200	1.54305600	-0.10247200
B	-0.41448200	0.26657600	0.45446900
C	3.48232900	0.79943900	-1.16433100
C	3.01088300	-0.57497000	-1.66252500
H	2.12148300	-0.51524600	-2.29503400
H	3.81876300	-1.00839700	-2.26147200
H	2.80424200	-1.25849500	-0.83591900
C	4.74029300	0.58783300	-0.30762500
H	5.51012300	0.12051700	-0.93035200
H	5.15215200	1.51878800	0.08577200
H	4.53748100	-0.08585900	0.53094100
C	3.78620200	1.69525900	-2.37066700
H	4.27448400	2.62784800	-2.07740400
H	4.46934000	1.15989100	-3.03774800
H	2.88290500	1.93355900	-2.94180000
C	2.33776900	3.36072900	0.25273300
C	1.31265300	3.68597100	1.35357000
H	0.28596800	3.48660400	1.03398900
H	1.50175500	3.12216900	2.27120900
H	1.39301700	4.75265900	1.58741800
C	3.74735800	3.68388300	0.76036500
H	4.03096100	3.05851400	1.61302400
H	4.50258200	3.57685100	-0.02275200
H	3.76537200	4.72658400	1.09375700
C	2.01227200	4.20966400	-0.98576900
H	2.01669800	5.26204700	-0.68367100

H	2.74595600	4.09238800	-1.78459300
H	1.01963200	3.98955900	-1.38736700
C	-1.98104600	0.28823300	-0.04507400
C	-2.63667800	1.50034800	-0.25158700
C	-2.82091100	-0.82127500	-0.11642300
C	-3.98659800	1.62541800	-0.55154600
C	-4.17650200	-0.74984900	-0.41471200
C	-4.76703300	0.48374500	-0.63874100
F	-1.96910600	2.67521500	-0.13521600
F	-4.53462300	2.82729600	-0.74662700
F	-4.91790800	-1.85687500	-0.47376900
F	-2.35309800	-2.05512100	0.12365200
F	-6.06517200	0.57238400	-0.92355400
C	0.30973700	-1.21252300	0.34557900
C	1.19743900	-1.75177800	1.27155200
C	0.16842200	-1.98247200	-0.80940000
C	1.85432200	-2.97086400	1.10578900
C	0.79120900	-3.20193100	-1.02316000
C	1.64507300	-3.70574200	-0.04907900
F	-0.58479400	-1.52260100	-1.82221300
F	0.60254200	-3.88142400	-2.15430600
F	2.26311400	-4.87018900	-0.23110100
F	2.68231500	-3.42756500	2.04690400
F	1.50500000	-1.09738400	2.41089100
H	2.35492300	0.92919600	1.14613100
C	-0.46462300	0.86647700	1.98909300
H	0.53249700	1.03716200	2.41488300
C	-2.49682900	0.10620100	3.25816100
H	-3.07287100	0.96858500	2.92840200
C	-1.22303200	-0.05068100	2.90218500
H	-0.68704600	-0.93365500	3.24897900
H	-3.00896200	-0.61819900	3.88284700
H	-0.97661000	1.83624500	1.96048200

P9_TS

C	-0.26630100	-0.71043100	-0.98951600
H	-0.18648900	-1.77787700	-1.21953300
H	-0.30550000	-0.18101300	-1.94651300
B	0.97748600	-0.14655800	-0.05833300
C	2.39858000	-0.80508400	-0.46263700
C	3.57704700	-0.05062000	-0.56572000
C	2.51289100	-2.17829600	-0.72953700
C	4.79441300	-0.62710700	-0.92385500
C	3.72136100	-2.76998700	-1.08320100
C	4.87143600	-1.99099000	-1.18611000
P	-1.74648100	-0.42639100	0.04732000
C	-2.63464600	-2.08900500	0.18030700
C	-3.92425700	-1.95461300	0.99929000
H	-4.69557600	-1.38450800	0.47619300
H	-4.33080700	-2.95419100	1.19216300
H	-3.73795000	-1.47697600	1.96689100
C	-2.93137900	-2.73836600	-1.17790900
H	-3.69962800	-2.20728900	-1.73937600
H	-2.03425200	-2.80235400	-1.80012800
H	-3.29144400	-3.76054700	-1.01044500
C	-1.68357900	-3.02001300	0.95278900
H	-0.72975400	-3.16071500	0.43496900
H	-1.47557700	-2.64693100	1.95938800
H	-2.15536700	-4.00417800	1.04967300
C	-2.89404200	0.91374100	-0.63095100
C	-3.68233600	1.48950100	0.55516800
H	-4.34264300	2.28636300	0.19405900
H	-4.30397900	0.74051200	1.05195900
H	-3.00297900	1.92120300	1.29627800
C	-3.84993700	0.39585800	-1.71112100
H	-4.57484700	-0.32493500	-1.32747500
H	-4.41534800	1.24611400	-2.10991500
H	-3.30504200	-0.06078400	-2.54331800
C	-2.06350300	2.04820100	-1.24605400
H	-1.39512400	2.51762100	-0.52340300
H	-1.46702300	1.71080200	-2.09730300
H	-2.76059800	2.81202700	-1.61061400
H	3.54044400	1.01573900	-0.35719700
H	5.68505200	-0.00914200	-0.99306600
H	5.81766900	-2.44477300	-1.46492100
H	3.76729500	-3.83718100	-1.28023700
H	1.63122000	-2.81419000	-0.65375800
H	-0.49848800	-0.29495000	1.20312900
C	0.95490700	1.46421700	0.00077300
C	0.54126100	2.23769400	1.09383000
C	1.29049400	2.16392900	-1.17165900
C	0.45808400	3.62952600	1.02379700
H	0.27950200	1.74829900	2.02774600
C	1.20993800	3.54971600	-1.25706600
H	1.62081800	1.60027400	-2.04353100
C	0.78759400	4.29141200	-0.15420300
H	0.13424700	4.19528500	1.89282300
H	1.47405300	4.05408600	-2.18215900

H	0.72199900	5.37357400	-0.21356800
C	0.74910300	-0.83259400	1.75820500
H	-0.04727400	-0.94868100	2.51318000
C	1.83221900	0.18227600	3.77819600
H	0.91833600	0.09632400	4.36240000
C	1.89479300	-0.22283000	2.51057200
H	2.83136300	-0.11565500	1.96850200
H	2.69112500	0.62258400	4.27313800
H	0.96751200	-1.86208700	1.45551400

P9_P

C	0.24576100	0.92144200	-0.59865100
H	0.24780900	2.01394200	-0.51789900
H	0.16462400	0.66737600	-1.66160800
B	-1.08446800	0.29629200	0.27280200
C	-2.43576500	0.84841400	-0.45674200
C	-3.66113800	0.18368700	-0.26653500
C	-2.49005700	2.01788000	-1.22866000
C	-4.85625600	0.65204800	-0.80437300
C	-3.67807500	2.50055800	-1.77763200
C	-4.87074800	1.81789500	-1.56747400
P	1.84678000	0.37454500	-0.00975800
C	2.87669000	1.83225400	0.53009600
C	4.27527700	1.40326100	0.98754400
H	4.89793100	1.06387400	0.15617100
H	4.77294000	2.26776500	1.43945400
H	4.23397500	0.61277100	1.74384300
C	2.98599000	2.88083600	-0.58610800
H	3.54338900	2.51775500	-1.45143400
H	2.00479800	3.22088100	-0.92583600
H	3.51917200	3.75087000	-0.18840800
C	2.13798100	2.45404600	1.72829800
H	1.11664700	2.75374600	1.48035700
H	2.08989400	1.76565800	2.57711300
H	2.68744800	3.34653700	2.04546100
C	2.73432900	-0.81620200	-1.13652000
C	3.73353800	-1.65309300	-0.32242000
H	4.18588900	-2.39184900	-0.99225700
H	4.54147900	-1.06349200	0.11309900
H	3.22272800	-2.20087900	0.47582800
C	3.44085600	-0.05920600	-2.26699500
H	4.28128000	0.53978600	-1.90661800
H	3.83770800	-0.78976600	-2.97912100
H	2.75065700	0.59352800	-2.81196000
C	1.70355500	-1.78413900	-1.73640500
H	1.20995500	-2.38246100	-0.96723600
H	0.93295200	-1.27464400	-2.31854000
H	2.24055500	-2.46370500	-2.40755500
H	-3.67045800	-0.73325400	0.31997700
H	-5.77932600	0.10584700	-0.62995600
H	-5.79950500	2.18718900	-1.99209000
H	-3.67006800	3.41027400	-2.37213600
H	-1.58036700	2.58469800	-1.41917500
H	1.69489300	-0.36161200	1.17585000
C	-0.99306700	-1.33103300	0.22800800
C	-0.37618500	-2.08676500	1.23844100
C	-1.46014800	-2.05564200	-0.88293700
C	-0.19583600	-3.46817200	1.13663200
H	-0.04892900	-1.58673200	2.14839800
C	-1.30067500	-3.43374100	-0.99653300
H	-1.96729300	-1.51500500	-1.68013900
C	-0.65440600	-4.14838000	0.01221500
H	0.28554300	-4.01393000	1.94399100
H	-1.67792700	-3.95431800	-1.87274200
H	-0.52519700	-5.22345600	-0.07129100
C	-1.00404200	0.91191200	1.80714000
H	-0.05564900	0.67659600	2.31905700
C	-2.01087900	-0.21977300	3.82317400
H	-1.03648800	-0.50885400	4.21287400
C	-2.12730500	0.47594400	2.69289400
H	-3.12681600	0.74016900	2.34572100
H	-2.87789200	-0.52903200	4.39783500
H	-1.04208800	2.00991600	1.71457000

P10_TS

P	2.60477100	-0.69484700	0.65673900
B	-1.88459100	-0.34248200	0.77685800
C	2.42521200	-1.28477800	2.41234300
H	2.86661700	-0.60511900	3.14187600
H	3.01638500	-2.20720900	2.43391300
C	0.94893900	-1.58291200	2.72350200
H	0.88423700	-2.05945600	3.70805500
H	0.58703600	-2.33859400	2.01421300
C	0.05164700	-0.33393100	2.68800300
H	0.11284100	0.15987000	3.66464700
H	0.47469300	0.39061100	1.98429600
C	-1.42171800	-0.60666000	2.31853500
H	-1.65070700	-1.64977200	2.57766900

H	-2.07695000	-0.00194900	2.95666000
C	2.55073900	1.11493000	0.36077500
C	2.41496500	2.12507000	1.33850700
C	2.04700000	3.41211100	0.93590000
H	1.91982200	4.17518900	1.69915900
C	1.84585500	3.75302300	-0.39697800
C	2.06027400	2.76384500	-1.35543500
H	1.95621500	3.01233500	-2.40934000
C	2.40885400	1.46023500	-1.01039800
C	2.69020600	1.92666600	2.80942100
H	1.89024800	1.38095900	3.31417300
H	3.62885400	1.39156000	2.97175000
H	2.77804400	2.89851100	3.29806300
C	1.43869300	5.14475600	-0.80106200
H	0.41253900	5.16240900	-1.17911200
H	1.48908300	5.83161400	0.04547800
H	2.08882700	5.52285300	-1.59489800
C	2.66960500	0.47659400	-2.12507300
H	3.71588700	0.15598900	-2.13323300
H	2.04437400	-0.41790400	-2.04304200
H	2.44276500	0.93326200	-3.09027700
C	4.14257300	-1.43260300	0.00711700
C	5.41794800	-0.89229800	0.25990500
C	6.53104200	-1.49607000	-0.32382500
H	7.51647100	-1.07940600	-0.12904700
C	6.41682000	-2.62213200	-1.13677300
C	5.14834000	-3.16004100	-1.34621500
H	5.04485900	-4.05238000	-1.95884000
C	4.00445700	-2.58709000	-0.79223600
C	5.61968100	0.29493700	1.16655500
H	6.68326500	0.50252800	1.29641700
H	5.13792100	1.19534900	0.77458700
H	5.19611500	0.09905700	2.15759400
C	7.63103300	-3.23493500	-1.78315700
H	8.52964700	-3.05501500	-1.18893600
H	7.51047200	-4.31322400	-1.90934600
H	7.79768100	-2.80269200	-2.77516800
C	2.66383600	-3.23293100	-1.06204800
H	2.79935200	-4.16726100	-1.60966300
H	2.12669500	-3.46532200	-0.13626600
H	2.00845800	-2.58710900	-1.65761100
C	-2.06016400	1.20307000	0.28657300
C	-1.48486400	2.29413600	0.93951900
F	-0.91474800	2.16563500	2.15155400
C	-1.44021800	3.57884600	0.41086500
F	-0.84858700	4.57086000	1.08434600
C	-2.00401200	3.82634400	-0.82891500
F	-1.94957700	5.04856600	-1.36296000
C	-2.61862000	2.78714500	-1.51096400
F	-3.17659200	3.01460300	-2.69988200
C	-2.63992900	1.51847700	-0.94619600
C	-3.11003600	-1.33966100	0.40283800
C	-4.43798400	-0.91814900	0.36568300
F	-4.74562900	0.37025800	0.56568600
C	-5.51183500	-1.76929100	0.13683700
F	-6.75856500	-1.29915200	0.10853100
C	-5.28288900	-3.12382600	-0.05278500
F	-6.29803300	-3.95623100	-0.27190200
C	-3.98255600	-3.60154900	-0.00159000
F	-3.74120400	-4.90333200	-0.16598000
C	-2.94380200	-2.71209600	0.23741100
F	-1.71181400	-3.26554300	0.30688600
F	-3.26405500	0.57876900	-1.66505100
H	1.02797300	-1.03547900	-0.08148900
C	-0.35199600	-0.83835700	-0.31194600
H	-0.00971600	0.15993200	-0.05677200
H	-0.35725800	-1.66081500	0.39087900
C	-0.55391000	-0.22197400	-2.71205300
H	-0.15896000	0.77536000	-2.52398400
C	-0.68950700	-1.10410200	-1.71866900
H	-1.10369200	-2.08925100	-1.92753900
H	-0.86154800	-0.45300800	-3.72513700

P10_P

P	-2.92012600	-0.74910600	-0.62220500
B	1.83890500	-0.59179800	-0.47918600
C	-2.50989700	-1.54563900	-2.22905000
H	-2.78789000	-0.89975000	-3.06378400
H	-3.17485800	-2.41680000	-2.25392300
C	-1.02474900	-1.93914600	-2.27828100
H	-0.87492000	-2.59978000	-3.13935700
H	-0.77638800	-2.53258200	-1.38962100
C	-0.11025900	-0.71860700	-2.37051200
H	-0.20129700	-0.31046500	-3.38651700
H	-0.50515300	0.05793700	-1.70738900
C	1.35795800	-0.99160900	-2.00651100
H	1.55016400	-2.06553400	-2.15120900
H	2.01206600	-0.48379900	-2.72550200

C	-2.56396300	0.99140100	-0.28394100
C	-2.37237700	1.98290400	-1.26647500
C	-1.97821800	3.25264300	-0.84997300
H	-1.80603700	4.01388600	-1.60476100
C	-1.77955400	3.57151100	0.49122800
C	-2.01397600	2.58004500	1.44369500
H	-1.88310200	2.81202600	2.49778300
C	-2.39979200	1.28976800	1.09179200
C	-2.53166900	1.76116600	-2.74882200
H	-1.63028800	1.30241700	-3.16381600
H	-3.39013200	1.13472900	-3.00165200
H	-2.67242100	2.72050900	-3.24927500
C	-1.32432400	4.94366400	0.90500400
H	-1.82141100	5.25753800	1.82611600
H	-0.24566500	4.95570300	1.08706500
H	-1.53054000	5.67936200	0.12559200
C	-2.59201100	0.27060100	2.18992500
H	-3.53706800	-0.27411700	2.09845600
H	-1.76002500	-0.44553000	2.21026300
H	-2.59065000	0.77007900	3.15993200
C	-4.56853300	-1.29423500	-0.09624100
C	-5.71512800	-0.50539500	-0.29513700
C	-6.94323700	-0.99845400	0.14707400
H	-7.83290400	-0.39335400	-0.00717200
C	-7.06173700	-2.23662700	0.77087100
C	-5.90868200	-3.00205200	0.94829800
H	-5.98657300	-3.97436300	1.42871500
C	-4.65801200	-2.56065900	0.52856300
C	-5.67844700	0.84675700	-0.96153900
H	-6.69120100	1.17242000	-1.20415200
H	-5.22110300	1.60212500	-0.31594700
H	-5.10897300	0.82606300	-1.89489700
C	-8.39746700	-2.75500200	1.23153300
H	-8.68241200	-3.64487300	0.66242800
H	-8.36162100	-3.03937000	2.28671700
H	-9.18042300	-2.00541000	1.10448600
C	-3.45837200	-3.44651600	0.77652500
H	-3.78367200	-4.41750700	1.15172800
H	-2.86973400	-3.62876500	-0.12888600
H	-2.78414900	-3.01429800	1.52525300
C	2.05886100	1.03505300	-0.22325500
C	1.41747600	2.06636200	-0.90440500
F	0.70569000	1.83975700	-2.03256400
C	1.44022700	3.39949300	-0.51001800
F	0.76719900	4.33235400	-1.20455100
C	2.15104500	3.76803700	0.61764300
F	2.14509400	5.04083600	1.03422500
C	2.84978100	2.79491900	1.31420300
F	3.54895100	3.13212900	2.40057100
C	2.79937400	1.47739000	0.87655900
C	3.25142800	-1.40667900	-0.25573900
C	4.45514700	-0.92574700	-0.76679000
F	4.49266900	0.26601300	-1.38734700
C	5.66368300	-1.60378600	-0.68379400
F	6.78130800	-1.07855800	-1.19405200
C	5.70498800	-2.84809000	-0.07200000
F	6.85407900	-3.52126800	0.01624900
C	4.53409100	-3.38370300	0.43829800
F	4.55039000	-4.58703100	1.02110700
C	3.34896000	-2.66451000	0.32678000
F	2.25701100	-3.27823700	0.83366000
F	3.51603800	0.60896400	1.60333200
H	-2.04708700	-1.40605300	0.24654700
C	0.65283200	-0.98696100	0.63622200
H	-0.17883300	-0.29507100	0.43190100
H	0.31132000	-2.01490100	0.44721300
C	0.73450200	0.18646800	2.85851100
H	0.15426300	1.02740900	2.47876000
C	1.03288000	-0.85109200	2.07280100
H	1.65265300	-1.65106000	2.47659700
H	1.10061800	0.26008200	3.87738100

P11_TS

P	1.66800100	-0.65111400	-0.04446700
B	-1.37812700	-0.47493800	-0.04513800
C	0.73135900	-1.03076500	1.51697300
H	0.58912400	-0.09240400	2.05607700
C	1.43550900	-2.03962900	2.43019700
H	2.42550100	-1.67230200	2.73034700
H	1.61313800	-2.96678800	1.86815700
C	0.57931900	-2.34417100	3.65896000
H	0.48029300	-1.43179400	4.26360700
H	1.07821400	-3.09037500	4.28632600
C	-0.80829200	-2.82449800	3.23924400
H	-0.71069600	-3.77468900	2.69593600
H	-1.42258900	-3.02708000	4.12304000
C	-1.50299800	-1.79918700	2.34022600
H	-2.48796800	-2.17939500	2.06226000

H	-1.67013700	-0.88078400	2.91110100
C	-0.67750400	-1.48873800	1.07392300
H	-0.56085500	-2.46173900	0.56941700
C	3.06291400	-1.86025200	-0.22324700
C	2.83106100	-3.02979500	-0.98073100
C	3.85489500	-3.95917100	-1.15776300
H	3.65039400	-4.85654900	-1.73681800
C	5.12220200	-3.77049300	-0.61757700
C	5.33808100	-2.61810600	0.12947400
H	6.31648300	-2.45475200	0.57559900
C	4.34380400	-1.66070200	0.34342200
C	1.50794000	-3.32748500	-1.63863400
H	1.50129000	-4.34325900	-2.03795700
H	0.67076300	-3.23843200	-0.94272100
H	1.32407600	-2.64291800	-2.47305600
C	6.22601800	-4.76813600	-0.84719400
H	5.82462600	-5.76996400	-1.01571300
H	6.81632000	-4.49448100	-1.72776900
H	6.90647100	-4.80771800	0.00661000
C	4.73322100	-0.46535500	1.17794100
H	3.97491600	-0.21908700	1.92138800
H	5.66435000	-0.67453100	1.70822700
H	4.88438600	0.42633100	0.56266700
C	2.39899500	1.03455800	-0.07546400
C	2.81075400	1.45477300	-1.36515000
C	3.18746300	2.77836900	-1.56698400
H	3.47694800	3.09437900	-2.56631100
C	3.16597200	3.71487600	-0.53538000
C	2.83543400	3.26596400	0.73707200
H	2.85210800	3.97190000	1.56552900
C	2.45512100	1.94547500	1.00046200
C	2.87006400	0.50765200	-2.54075200
H	3.16424900	1.04538400	-3.44367900
H	3.59274000	-0.29700500	-2.37063500
H	1.89639100	0.04549300	-2.73162600
C	3.43587200	5.16993700	-0.80323900
H	2.51225500	5.65417600	-1.13728500
H	3.77741900	5.68594300	0.09686300
H	4.18558400	5.30145100	-1.58708600
C	2.17490600	1.63380900	2.45713200
H	1.13900400	1.85916700	2.73615100
H	2.37173500	0.59694900	2.72787800
H	2.81536400	2.26151900	3.08136300
C	-3.00257700	-0.64371000	-0.06277400
C	-3.92479000	0.39894500	-0.11506100
F	-3.53917100	1.68352200	-0.10027500
C	-5.29974400	0.20729300	-0.21816800
F	-6.12269600	1.25463200	-0.26408500
C	-5.81350500	-1.07575000	-0.28674700
F	-7.12380700	-1.27640000	-0.39448300
C	-4.93848500	-2.15220700	-0.24220700
F	-5.40879800	-3.39755500	-0.31493900
C	-3.57889000	-1.91264200	-0.12996700
F	-2.79591700	-3.01062500	-0.13102700
C	-0.91090900	1.10912900	0.01347700
C	-1.06713500	1.83206000	1.19283000
F	-1.54875500	1.21986800	2.28740000
C	-0.74592900	3.17170000	1.35125900
F	-0.85834600	3.75726200	2.54489500
C	-0.25158900	3.88293500	0.27037800
F	0.11903700	5.15421500	0.40251600
C	-0.09002400	3.22905000	-0.93755300
F	0.41462400	3.88611200	-1.98316100
C	-0.39182300	1.87632300	-1.02976900
F	-0.07239400	1.32108400	-2.21705900
H	0.10891200	-0.76578600	-1.02863500
C	-1.03041100	-1.15046200	-1.79391700
H	-1.24404200	-2.20729600	-1.62398400
H	-0.12655000	-1.14036900	-2.42614100
C	-3.01944500	-1.25977200	-3.29619100
H	-3.09243100	-2.33562800	-3.15412700
C	-2.08127200	-0.53907500	-2.68936800
H	-2.04118400	0.53384100	-2.84885100
H	-3.75345700	-0.79550200	-3.94630000

P11_P

P	-1.78735400	-0.65324800	-0.03936000
B	1.60087300	-0.54917500	0.37818100
C	-0.54858700	-1.12306700	-1.32845100
H	-0.38758500	-0.20478800	-1.89522300
C	-1.18952900	-2.17512500	-2.25583100
H	-2.19535500	-1.87319600	-2.57741200
H	-1.31802100	-3.10984300	-1.69356800
C	-0.29819400	-2.42165100	-3.46880300
H	-0.22628100	-1.49494700	-4.05567700
H	-0.75386800	-3.17773800	-4.11660900
C	1.09091800	-2.84677500	-3.00995300
H	1.01684500	-3.81900600	-2.50356800

H	1.74996300	-2.98909700	-3.87296200
C	1.70138500	-1.82509000	-2.04909400
H	2.67715400	-2.19658900	-1.74167400
H	1.87808600	-0.88185700	-2.58000800
C	0.84977800	-1.54802000	-0.78549100
H	0.71648200	-2.53117800	-0.30540100
C	-3.08911300	-1.91808200	0.17934600
C	-2.81767800	-2.98891500	1.05951600
C	-3.78825500	-3.97304500	1.24222700
H	-3.57122700	-4.79825500	1.91551700
C	-5.01412700	-3.93168800	0.58591400
C	-5.25733500	-2.86812500	-0.27978500
H	-6.20575500	-2.82340900	-0.80953400
C	-4.32616400	-1.85474100	-0.49949100
C	-1.52555800	-3.11335600	1.82665700
H	-1.46279100	-4.09228100	2.30398500
H	-0.64204500	-2.99849200	1.19326600
H	-1.45460200	-2.35779000	2.61785600
C	-6.05735700	-4.99211600	0.81466800
H	-6.79481900	-4.65086300	1.54831200
H	-6.59392800	-5.22289500	-0.10861500
H	-5.61074600	-5.91301800	1.19478400
C	-4.72004000	-0.74940300	-1.44865100
H	-3.93792300	-0.52433900	-2.17660700
H	-5.61002300	-1.04416000	-2.00719500
H	-4.94474900	0.17815900	-0.91451900
C	-2.51369000	1.01013000	-0.15608200
C	-3.01503400	1.53238400	1.05997300
C	-3.55165100	2.81738200	1.06940800
H	-3.91852900	3.22147100	2.00915500
C	-3.61125600	3.59955800	-0.08107300
C	-3.13685200	3.05229700	-1.26998400
H	-3.17960500	3.64633500	-2.17954100
C	-2.58411900	1.77352800	-1.34092900
C	-2.95365500	0.78394200	2.37109700
H	-3.55936800	1.29846700	3.11819100
H	-3.33002000	-0.24072000	2.28837600
H	-1.92761700	0.74725900	2.75402600
C	-4.12597900	5.01085900	-0.03187400
H	-4.57013400	5.30691800	-0.98476600
H	-4.87155700	5.13667700	0.75607900
H	-3.29467900	5.69116200	0.17730300
C	-2.07865100	1.34405200	-2.69972700
H	-1.02347200	1.61170000	-2.82428100
H	-2.18021800	0.27554700	-2.89385400
H	-2.63752200	1.87156100	-3.47526800
C	3.22564200	-0.41626700	0.07248400
C	3.97558600	0.74183500	-0.10747200
F	3.40286200	1.95871800	-0.15757800
C	5.36322400	0.76255600	-0.23740800
F	6.00654100	1.91971400	-0.41372900
C	6.07858400	-0.41922700	-0.18102600
F	7.40557200	-0.41941900	-0.30713500
C	5.38651900	-1.60731700	0.00388300
F	6.05082300	-2.76445800	0.06205200
C	4.00763900	-1.57152300	0.12714100
F	3.41445400	-2.77242400	0.29868300
C	0.90510200	0.97192800	0.39971100
C	0.81933700	1.73585100	-0.76564000
F	1.31482000	1.24513400	-1.91970500
C	0.25209900	2.99591500	-0.85626500
F	0.15182700	3.61901400	-2.03490200
C	-0.28621200	3.58259400	0.27938900
F	-0.88375700	4.77267700	0.21024500
C	-0.22885200	2.88577200	1.47079100
F	-0.80085300	3.39051900	2.56735700
C	0.35803600	1.62300800	1.50373200
F	0.30466300	1.03868000	2.71988000
H	-1.11851900	-0.63819600	1.18420700
C	1.48280400	-1.30691000	1.86167000
H	1.62230100	-2.38147900	1.69639400
H	0.47502800	-1.18857000	2.28109100
C	3.49379200	-1.61134000	3.33170600
H	3.65736400	-2.61769300	2.95213800
C	2.47177400	-0.87273400	2.90105200
H	2.35867300	0.13627700	3.29617400
H	4.20645200	-1.23438600	4.05841400

P12_TS

P	2.19080200	-0.95874400	-0.54338300
C	1.86414900	-1.20772400	-2.33395700
H	2.69122300	-0.79982300	-2.92554500
H	1.87566300	-2.29572500	-2.47130000
C	0.49511300	-0.65535000	-2.74484000
H	0.49696300	0.43740600	-2.68928700
H	0.35538500	-0.90050100	-3.80405800
C	-0.67409400	-1.23052600	-1.92790800
H	-0.56889400	-2.32491400	-1.91511100

H	-1.58109400	-1.04686600	-2.51272500
B	-0.98893300	-0.67963200	-0.40175200
C	3.46023000	-2.20204000	-0.12665400
C	3.04118500	-3.53395600	-0.01404700
H	1.99501800	-3.79200500	-0.16713700
C	3.95724900	-4.53862700	0.27788400
H	3.62119600	-5.56678600	0.36192200
C	5.30046600	-4.22112300	0.46499300
H	6.01577100	-5.00344100	0.69691000
C	5.72437600	-2.90056900	0.34983900
H	6.77164500	-2.65195200	0.48754200
C	4.81129300	-1.89256900	0.05272700
H	5.15194800	-0.86649600	-0.04174400
C	2.96491300	0.67240800	-0.34303100
C	2.88993900	1.63337200	-1.35457200
H	2.44989700	1.38951800	-2.31592700
C	3.35555600	2.92585000	-1.12505200
H	3.27610300	3.67156200	-1.90943700
C	3.90642200	3.26267900	0.10727400
H	4.25714400	4.27340500	0.28749900
C	3.99626700	2.30305900	1.11420700
H	4.41283000	2.56521800	2.08091200
C	3.51889600	1.01649500	0.89723000
H	3.55917300	0.28269200	1.69765000
C	-0.42460300	0.83692100	-0.13507300
C	-0.79354100	1.82945600	-1.04230700
F	-1.53961300	1.50352900	-2.11226800
C	-0.44708200	3.16721500	-0.92114500
F	-0.81643700	4.05653400	-1.84303300
C	0.28525600	3.57823200	0.18377200
F	0.64531400	4.85210100	0.31990200
C	0.65532500	2.63999600	1.13165800
F	1.36399000	3.01573400	2.19773200
C	0.30625900	1.30768000	0.94973400
F	0.78045300	0.45794500	1.87858900
C	-2.61327300	-0.71023700	-0.11143200
C	-3.39792500	-1.77337800	-0.56308200
F	-2.81910400	-2.82767900	-1.16805500
C	-4.77385200	-1.85712000	-0.40805700
F	-5.44933300	-2.90861700	-0.87071600
C	-5.44531700	-0.83166200	0.24124200
F	-6.76385700	-0.88641200	0.40392100
C	-4.71845000	0.23884100	0.73336400
F	-5.34224200	1.22116800	1.38285900
C	-3.33808000	0.27564000	0.55906600
F	-2.73120800	1.33356300	1.12071600
H	0.63290500	-1.28385400	0.19563600
C	-0.46776000	-1.97089500	0.84832700
H	0.42202300	-2.61476500	0.93586400
H	-1.17566900	-2.65402100	0.36928300
C	-0.33804700	-2.16342700	3.33550900
H	0.45335800	-2.90652100	3.26875400
C	-0.87692500	-1.62165800	2.24674200
H	-1.65883900	-0.87262600	2.35824000
H	-0.66645500	-1.88286300	4.33025700

P12_P

P	2.42666200	-0.93881400	-0.57201600
C	1.72524700	-1.42515100	-2.16979400
H	2.50975900	-1.20918500	-2.90665000
H	1.63289800	-2.51671800	-2.11062400
C	0.36937500	-0.80258900	-2.55257000
H	0.41625000	0.28739400	-2.47566300
H	0.26010800	-1.02042900	-3.62200000
C	-0.84738200	-1.34872000	-1.79306000
H	-0.79545400	-2.44626200	-1.83250500
H	-1.70610000	-1.09057100	-2.42160900
B	-1.21341100	-0.87588900	-0.22472700
C	3.78490200	-2.03772800	-0.13920000
C	3.53540800	-3.11184000	0.72030800
H	2.54824900	-3.25204700	1.15461600
C	4.56410700	-3.99764900	1.02595900
H	4.37659900	-4.82867300	1.69708400
C	5.82980500	-3.80996500	0.47692800
H	6.63065300	-4.50081200	0.71923900
C	6.07773000	-2.73593200	-0.37671400
H	7.06728300	-2.59076100	-0.79644500
C	5.05740100	-1.84487600	-0.68657100
H	5.25165600	-0.99526200	-1.33629600
C	3.02602300	0.75547000	-0.55751400
C	2.79009000	1.62317400	-1.62656300
H	2.27929000	1.28003100	-2.51986500
C	3.20338800	2.94963300	-1.53457100
H	3.00471400	3.63097800	-2.35474000
C	3.84029200	3.40410700	-0.38395600
H	4.13607200	4.44487800	-0.30699700
C	4.08730000	2.53280200	0.67636600
H	4.57145900	2.89145400	1.57773400

C	3.68537100	1.20657500	0.59271500
H	3.85543100	0.53343800	1.42914400
C	-0.49210200	0.58678400	0.10244900
C	-0.73923000	1.66262700	-0.75503000
F	-1.52350200	1.48151300	-1.83117600
C	-0.22304400	2.93874400	-0.59045700
F	-0.46597400	3.90362900	-1.48046000
C	0.57473900	3.21008700	0.51375800
F	1.11730700	4.41790800	0.67670000
C	0.84078300	2.19218800	1.40955400
F	1.65494600	2.41201000	2.44755400
C	0.32022800	0.92179500	1.17905100
F	0.75279000	-0.02226800	2.04426400
C	-2.85973700	-0.66842800	-0.13751000
C	-3.70838800	-1.64107100	-0.66703300
F	-3.19301800	-2.73243200	-1.26682500
C	-5.09494500	-1.58878300	-0.61460700
F	-5.83836300	-2.56147800	-1.14819000
C	-5.70949900	-0.51341800	0.00891200
F	-7.03893900	-0.43542400	0.07257700
C	-4.91848500	0.47364500	0.57206700
F	-5.49124300	1.51117300	1.18764800
C	-3.53264700	0.37170900	0.50028700
F	-2.86311500	1.36434000	1.11433300
H	1.48011600	-1.13129200	0.43296300
C	-0.89236700	-2.10964700	0.83903400
H	0.16680300	-2.41128800	0.89791100
H	-1.39827200	-2.97107400	0.37748800
C	-0.74339200	-2.14468500	3.36268000
H	0.31622700	-2.39440800	3.35231800
C	-1.42489500	-1.97414300	2.23210000
H	-2.48335700	-1.71780500	2.30233900
H	-1.20771200	-2.02894700	4.33651100

P13_TS

P	-2.21448800	-0.48311000	0.22966100
B	0.95097900	-0.85026800	-0.03709000
C	-1.91200200	-0.18222700	2.02720100
H	-1.21358000	0.65664500	2.11761700
H	-2.87243000	0.12757700	2.45574800
C	-1.35757800	-1.40893900	2.76709900
H	-1.47327800	-1.22169200	3.84033800
H	-1.98502300	-2.28059500	2.53891100
C	0.10795200	-1.73722700	2.46734800
H	0.36797900	-2.61923200	3.06302500
H	0.74034200	-0.93130300	2.84423600
C	0.39080200	-2.02199500	0.97843300
H	-0.53107900	-2.45488200	0.56333100
H	1.09849400	-2.85038500	0.91056000
C	-3.09958200	0.99635200	-0.36346100
C	-3.83766000	0.90874200	-1.55078900
H	-3.93137000	-0.04898800	-2.05772100
C	-4.45631500	2.03707800	-2.07379000
H	-5.02799700	1.96124200	-2.99298600
C	-4.33741000	3.26493100	-1.42363000
H	-4.81145100	4.14769700	-1.83988900
C	-3.61008800	3.35722500	-0.24283700
H	-3.51108800	4.31071900	0.26527500
C	-2.99856500	2.22516300	0.29204100
H	-2.43643300	2.31255200	1.21685900
C	-3.51744100	-1.76967700	0.26079500
C	-4.80935200	-1.43583600	0.68439100
H	-5.04872400	-0.40442700	0.93086600
C	-5.79274800	-2.41460800	0.77493800
H	-6.79240500	-2.14561600	1.10014200
C	-5.49438500	-3.73549900	0.44684100
H	-6.26251600	-4.49878400	0.51890000
C	-4.21263100	-4.07461000	0.02534800
H	-3.97720300	-5.10161500	-0.23379300
C	-3.22666400	-3.09549600	-0.06997500
H	-2.23293300	-3.36296200	-0.41441500
C	0.77479400	0.74165300	0.33079800
C	0.14184300	1.70839300	-0.44533500
F	-0.47538400	1.37383200	-1.59248200
C	0.08009900	3.05728700	-0.10958500
F	-0.58560200	3.91827600	-0.87751800
C	0.70952000	3.50213800	1.04094200
F	0.65251000	4.78512900	1.38771100
C	1.41291200	2.59249300	1.82006600
F	2.06055400	3.01183800	2.90504400
C	1.44940900	1.25798800	1.43996700
F	2.21421500	0.45194000	2.19456400
C	2.56272800	-0.97749900	-0.36223000
C	3.17691600	-0.25945900	-1.39205500
F	2.45141700	0.55317300	-2.18524000
C	4.53667100	-0.28897600	-1.66826800
F	5.03837700	0.40645500	-2.68838400
C	5.37772000	-1.03004700	-0.85233800

F	6.68563800	-1.07197800	-1.08956600
C	4.83577900	-1.70814400	0.22643200
F	5.63328400	-2.38759300	1.05019100
C	3.46508500	-1.65599900	0.45933300
F	3.06545500	-2.28259200	1.57570900
H	-0.77770400	-0.83475400	-0.67697500
C	0.18111200	-1.30915500	-1.71452200
H	0.54493400	-2.33944700	-1.66413700
H	0.87239500	-0.68946700	-2.27926100
C	-1.71099600	-2.40105100	-2.97505100
H	-1.33654000	-3.39804600	-2.75625200
C	-1.08311000	-1.31000000	-2.54017700
H	-1.47811800	-0.32731700	-2.79387700
H	-2.60692100	-2.33325400	-3.58378900

P13_P

P	-2.53507900	0.67020400	-0.24594800
B	1.21221700	0.75850600	0.36472200
C	-1.92338800	0.50568200	-1.94784200
H	-1.33004300	-0.41566000	-1.98643500
H	-2.83762300	0.34916800	-2.53497200
C	-1.13315500	1.70827200	-2.49758800
H	-1.21911400	1.64983600	-3.58793400
H	-1.63196700	2.64022700	-2.19956200
C	0.34414700	1.74919200	-2.10229400
H	0.79814500	2.56008100	-2.68110500
H	0.82645500	0.83422000	-2.45904400
C	0.60125200	1.96223300	-0.59573300
H	-0.32722000	2.35730300	-0.14481600
H	1.29647800	2.80041000	-0.48397600
C	-3.49325900	-0.78644600	0.20528800
C	-3.94403400	-0.89610600	1.52472200
H	-3.71392800	-0.12049300	2.25031600
C	-4.67833900	-2.01133000	1.90786700
H	-5.02568000	-2.10151900	2.93114200
C	-4.94968400	-3.01778600	0.98379500
H	-5.51269000	-3.89345600	1.28924800
C	-4.48969700	-2.91455000	-0.32575900
H	-4.68709200	-3.70660000	-1.03973200
C	-3.76449200	-1.79567600	-0.72274400
H	-3.39569900	-1.73094700	-1.74164400
C	-3.57690700	2.13068900	-0.10164000
C	-4.87532500	2.10216900	-0.62082700
H	-5.26785700	1.19057500	-1.06503500
C	-5.66905200	3.23980200	-0.54449900
H	-6.67754000	3.22279800	-0.94325000
C	-5.16996100	4.39723500	0.05151100
H	-5.79284100	5.28374800	0.11016900
C	-3.88102400	4.42140000	0.57651700
H	-3.49855200	5.32144400	1.04528000
C	-3.07658400	3.28760500	0.50278700
H	-2.07019200	3.30336000	0.91330800
C	0.55275400	-0.74922500	0.04587800
C	-0.37368700	-1.42320700	0.83925100
F	-0.88703800	-0.85175600	1.95349200
C	-0.91225800	-2.67270600	0.54589000
F	-1.84765200	-3.20814000	1.33372400
C	-0.51732500	-3.33343000	-0.60324100
F	-1.05252400	-4.51046400	-0.92708700
C	0.43654000	-2.73692400	-1.41762100
F	0.84872700	-3.36574300	-2.51846400
C	0.94913400	-1.49037700	-1.07426800
F	1.87307000	-1.00941700	-1.92177300
C	2.84066900	0.55782300	0.17004700
C	3.53067000	-0.31230400	1.01185000
F	2.85911000	-1.03593100	1.92864700
C	4.90487200	-0.50633500	0.98319900
F	5.49743900	-1.35416300	1.82871900
C	5.66685000	0.18932400	0.05682100
F	6.98909600	0.02158200	0.00309300
C	5.03200100	1.05309700	-0.81957600
F	5.74999300	1.71960900	-1.72865000
C	3.65202600	1.21100500	-0.75078600
F	3.13252000	2.04893600	-1.66955900
H	-1.51438300	0.83088500	0.69111100
C	0.95414900	1.22419500	1.93957500
H	1.18353900	0.41472700	2.63845400
H	-0.11192900	1.46775200	2.08976000
C	2.85170000	2.41513800	3.07301000
H	3.19908700	1.48954900	3.52740600
C	1.76449600	2.42932000	2.30349500
H	1.45900600	3.37904000	1.85885300
H	3.43355300	3.31066700	3.26627700

P14_TS

P	2.92179300	-0.33866800	-0.12109900
B	-0.45076000	-0.04424600	-0.10589900
C	2.45743700	1.15583400	-1.08768500

H	2.08839000	1.85015100	-0.32506900
H	3.30695500	1.63603400	-1.58401900
C	1.30691500	0.84408100	-2.06296000
H	1.72546200	0.51185200	-3.02134700
H	0.78847600	1.78655500	-2.27386600
C	0.29501500	-0.20170800	-1.56553800
H	0.71002300	-1.20286100	-1.66360300
H	-0.55048500	-0.18037000	-2.26411800
C	3.83367200	-1.52931500	-1.28896700
C	4.78502000	-0.78561100	-2.23649100
H	5.26890100	-1.51878600	-2.89220000
H	5.57060200	-0.23517200	-1.71882000
H	4.23708600	-0.08745900	-2.87662600
C	4.59316900	-2.58068700	-0.46555700
H	3.92251000	-3.10988000	0.21912400
H	5.42048800	-2.16407500	0.11005300
H	5.01503700	-3.32135500	-1.15340900
C	2.82611800	-2.29928000	-2.15895200
H	2.30750800	-1.64736400	-2.86450400
H	2.08478200	-2.83280000	-1.56051400
H	3.38485300	-3.03697600	-2.74574900
C	4.11365600	0.28150400	1.22151500
C	3.57128500	1.59666400	1.80794100
H	2.54388300	1.52353500	2.16443400
H	3.62341200	2.41818800	1.08900700
H	4.19916400	1.86761000	2.66365800
C	5.53331300	0.58213700	0.72222700
H	6.10625500	1.01514200	1.55034100
H	5.52581500	1.31569200	-0.08989600
H	6.07176700	-0.30453500	0.38519700
C	4.15582600	-0.75758600	2.35473100
H	4.56392700	-1.71757700	2.03475600
H	3.15841300	-0.93476700	2.76851100
H	4.79145600	-0.37890800	3.16298600
C	-0.98315800	1.49812900	-0.07365900
C	-0.41691400	2.56069600	0.61579100
F	0.59813200	2.34979300	1.48484500
C	-0.80904100	3.88814700	0.47719400
F	-0.21352000	4.84750900	1.18622600
C	-1.81685500	4.20551000	-0.41686500
F	-2.20943000	5.46653200	-0.57479000
C	-2.40412500	3.18741100	-1.15687400
F	-3.36233800	3.47705500	-2.03489700
C	-1.97368500	1.88076300	-0.98207800
F	-2.56049000	0.95867600	-1.75927500
C	-1.56661100	-1.23094500	-0.01666000
C	-1.17417500	-2.55944500	-0.15519800
F	0.13533200	-2.86589900	-0.29740000
C	-2.03992500	-3.64326100	-0.09429100
F	-1.58546500	-4.88946400	-0.23338200
C	-3.38914600	-3.41370700	0.12624700
F	-4.24431700	-4.43106100	0.18771500
C	-3.83125400	-2.11131900	0.30499200
F	-5.11948200	-1.88147500	0.55696100
C	-2.92386200	-1.06016700	0.24839100
F	-3.42529200	0.14959100	0.52035200
H	1.20557000	-0.42935300	0.46819200
C	0.33996800	-0.43818000	1.61940500
H	0.50877100	-1.51572300	1.71695300
H	1.20486100	0.07760900	2.04623300
C	-1.60989500	-0.90188600	3.12484900
H	-1.50761500	-1.97444900	2.97558200
C	-0.81081600	-0.03508000	2.51058200
H	-0.93804200	1.02982800	2.67848700
H	-2.40109500	-0.56605200	3.78646700

P14_P

P	3.34505700	-0.74115000	-0.06651300
B	-0.60694800	-0.18756900	-0.00967600
C	2.44761000	0.69735700	-0.72929300
H	1.97026800	1.15150900	0.14258400
H	3.21702800	1.39371900	-1.08816400
C	1.38050200	0.41129400	-1.79803700
H	1.87008900	0.09593900	-2.72914000
H	0.92693500	1.38440600	-2.01885800
C	0.29647100	-0.57599300	-1.35461100
H	0.74193600	-1.56933100	-1.21629800
H	-0.38857500	-0.68979400	-2.20449600
C	4.21583300	-1.68740000	-1.41165000
C	4.88849800	-0.70364500	-2.38002900
H	5.38464800	-1.27892400	-3.16795900
H	5.64740400	-0.08758500	-1.89067400
H	4.15921000	-0.04588700	-2.86088400
C	5.25002700	-2.65913800	-0.82667500
H	4.79627600	-3.35780200	-0.11717400
H	6.08446500	-2.15125700	-0.33943000
H	5.66068400	-3.24924500	-1.65203400
C	3.15910200	-2.51993200	-2.16219400

H	2.39760600	-1.90630300	-2.64368400
H	2.65653000	-3.23030100	-1.49898100
H	3.67581400	-3.09193300	-2.93987900
C	4.41526100	-0.17428500	1.35267400
C	3.62033400	0.85069800	2.18404200
H	2.67766100	0.44678900	2.56142300
H	3.40424600	1.76693400	1.63075300
H	4.23766500	1.12095700	3.04675300
C	5.69079400	0.50121400	0.83052300
H	6.23926700	0.90432100	1.68782300
H	5.46385300	1.34005000	0.16485100
H	6.35700900	-0.19021200	0.30958400
C	4.74976000	-1.37256400	2.25423800
H	5.34903000	-2.13357000	1.75257400
H	3.84072200	-1.83991700	2.64490600
H	5.32675700	-1.00714300	3.10959300
C	-0.84886700	1.44691800	-0.07764800
C	-0.20069600	2.40988000	0.68427000
F	0.68710900	2.05888900	1.65356900
C	-0.35231900	3.78399000	0.51760100
F	0.31824300	4.64203700	1.29283000
C	-1.19504700	4.25661500	-0.47239000
F	-1.35519200	5.56649700	-0.65926400
C	-1.85947400	3.34276200	-1.28063300
F	-2.66214400	3.78309800	-2.25091600
C	-1.66733600	1.98544700	-1.07247400
F	-2.32354600	1.16355900	-1.90547200
C	-2.02343900	-1.03454300	-0.05495800
C	-2.02096800	-2.40309000	-0.31094500
F	-0.86714400	-3.04409600	-0.60839300
C	-3.14442400	-3.21809700	-0.25122800
F	-3.05447700	-4.52686100	-0.51125600
C	-4.36624200	-2.66249800	0.09063200
F	-5.46173200	-3.42122800	0.15282900
C	-4.42814300	-1.30749700	0.37434200
F	-5.59460500	-0.75666400	0.71952900
C	-3.27255400	-0.53800300	0.30853700
F	-3.42749800	0.75354300	0.64079800
H	2.42301200	-1.63981500	0.48955400
C	0.09020300	-0.69599300	1.41048900
H	0.23511000	-1.78440400	1.32492300
H	1.07788100	-0.24317400	1.55692600
C	-1.50164800	-1.28558000	3.26460100
H	-1.53613100	-2.32933300	2.95844100
C	-0.73847200	-0.40317900	2.62115800
H	-0.74929600	0.63604200	2.95032600
H	-2.12795700	-0.99704200	4.10237500

P15_TS

P	1.13578700	-0.69309100	0.33267900
B	-1.52197000	-0.43750600	0.37070400
C	-0.36860800	-1.17475300	-0.57763200
H	-0.36045900	-2.23202100	-0.26358900
C	-0.52315500	-1.15000700	-2.09565600
H	-0.64709200	-0.12866000	-2.45489700
H	-1.46729500	-1.66470000	-2.31139400
C	0.61430900	-1.83734400	-2.84536300
H	1.54796100	-1.27577600	-2.74743800
H	0.38889200	-1.91361800	-3.91191300
H	0.78096900	-2.85164900	-2.46527500
C	1.90043500	0.93056100	0.03668900
C	1.75959300	1.69985400	-1.11241400
F	1.15755200	1.20406000	-2.19046600
C	2.21234300	3.01023400	-1.17473600
F	2.01872400	3.73559800	-2.26833200
C	2.84199900	3.57150600	-0.07053000
F	3.27207600	4.82097700	-0.12096500
C	3.02425900	2.81977900	1.08403600
F	3.62501400	3.35440000	2.13760400
C	2.55016800	1.51736700	1.12381600
F	2.71196300	0.82244700	2.24926700
C	2.47626600	-1.90172800	0.09819100
C	3.51712200	-1.75955400	-0.81723900
F	3.57627100	-0.69556600	-1.61825900
C	4.50975200	-2.71991600	-0.94968200
F	5.48797100	-2.56345900	-1.83222200
C	4.46998700	-3.85830300	-0.15281100
F	5.41041500	-4.78121300	-0.27433600
C	3.44050300	-4.03527000	0.76381000
F	3.40308700	-5.12418500	1.52048500
C	2.46410200	-3.05650400	0.87823000
F	1.47969900	-3.24478400	1.76016100
C	-1.30219000	1.18601800	0.32460900
C	-1.67989700	1.88536900	-0.82379100
F	-2.36185100	1.25531100	-1.79049800
C	-1.38885900	3.22182300	-1.05592400
F	-1.75970900	3.81431200	-2.18654100
C	-0.69505800	3.94115500	-0.09118600

F	-0.34915100	5.20521400	-0.30720800
C	-0.32164600	3.30678300	1.08123500
F	0.40071600	3.96298500	1.99218800
C	-0.62944900	1.96313400	1.26094000
F	-0.11870300	1.41121600	2.38449500
C	-3.03142200	-0.92271600	0.01685100
C	-4.15169900	-0.09284200	0.07715700
F	-4.03243400	1.19765800	0.40824400
C	-5.44637800	-0.52916300	-0.17171200
F	-6.47221600	0.31669600	-0.09851400
C	-5.67464300	-1.86199500	-0.48047200
F	-6.90827800	-2.29546200	-0.71524000
C	-4.60007400	-2.73587100	-0.53316700
F	-4.79482600	-4.02310600	-0.81594500
C	-3.32219700	-2.25158400	-0.28421900
F	-2.33907700	-3.17281800	-0.32157400
H	-0.06621700	-0.73273500	1.55658000
C	-1.34106500	-1.09588500	2.09310800
H	-1.62347500	-2.13396200	1.89357800
H	-0.48476100	-1.20167700	2.79256600
C	-3.58488000	-0.82384100	3.16259400
H	-3.91618400	-1.78854500	2.78521400
C	-2.36767900	-0.35522800	2.90564000
H	-2.06413200	0.61216400	3.29406400
H	-4.29198900	-0.25394200	3.75548600

P15_P

P	1.24362200	-0.69348800	0.40123000
B	-1.59567500	-0.47217600	0.60001700
C	-0.33913500	-1.26942400	-0.23897500
H	-0.29155200	-2.29465800	0.16745100
C	-0.48487300	-1.35528400	-1.76586700
H	-0.65789400	-0.36066400	-2.17759800
H	-1.41235400	-1.91060300	-1.92421100
C	0.64703500	-2.05188800	-2.51691700
H	1.55951500	-1.44785700	-2.53395100
H	0.36270800	-2.22480500	-3.55762300
H	0.88085900	-3.02519500	-2.07069600
C	1.86967200	0.97333300	0.09201600
C	1.69066500	1.66088600	-1.10367500
F	1.13806700	1.05299100	-2.14834200
C	2.06965300	2.98731600	-1.23954200
F	1.84557200	3.63522200	-2.37202700
C	2.65278300	3.64604400	-0.16217000
F	3.00230200	4.91232000	-0.27983000
C	2.87005100	2.97541200	1.03626100
F	3.42573900	3.60366700	2.05910700
C	2.47465800	1.65201800	1.15193100
F	2.68131400	1.02952600	2.31031700
C	2.63508000	-1.82821200	0.12793400
C	3.61347200	-1.64687100	-0.84666900
F	3.56765900	-0.59174300	-1.65932900
C	4.63919400	-2.56393400	-1.02095200
F	5.55910800	-2.37728000	-1.95559700
C	4.69637400	-3.68824700	-0.20336300
F	5.67033300	-4.56560400	-0.36303100
C	3.73047600	-3.89864300	0.77448200
F	3.78860100	-4.97167700	1.54737400
C	2.71423800	-2.96742600	0.92595200
F	1.78636800	-3.17682900	1.85821500
C	-1.36566100	1.14794900	0.36469600
C	-1.71915700	1.79433400	-0.82131300
F	-2.35607000	1.12201600	-1.79036800
C	-1.43772800	3.12558500	-1.10329200
F	-1.78080700	3.66558500	-2.26995300
C	-0.77533200	3.89408500	-0.15559700
F	-0.41934200	5.14786700	-0.42390000
C	-0.41360200	3.31118200	1.04675800
F	0.30095100	4.01037600	1.93573000
C	-0.70541200	1.97099000	1.26989800
F	-0.17412300	1.45843500	2.40371600
C	-3.04174200	-0.99822400	0.02265600
C	-4.18332200	-0.19938200	-0.02232600
F	-4.11290600	1.11044100	0.25467900
C	-5.45067500	-0.67599300	-0.33323000
F	-6.49719200	0.14920900	-0.36363600
C	-5.63192900	-2.02490500	-0.59668100
F	-6.83965500	-2.49936700	-0.89329300
C	-4.53633400	-2.87184100	-0.54232200
F	-4.68369900	-4.17648000	-0.78059000
C	-3.29104800	-2.34466000	-0.22752900
F	-2.28260000	-3.24452900	-0.16428000
H	1.13406300	-0.69541700	1.79006100
C	-1.61219200	-0.95187200	2.18117500
H	-1.83627400	-2.02723100	2.18146000
H	-0.64919700	-0.83444300	2.69426300
C	-3.85556000	-0.72867000	3.28767100
H	-4.13645000	-1.74046300	3.00192500

C	-2.65831400	-0.23425000	2.97895900
H	-2.41990800	0.78660500	3.27789800
H	-4.59407300	-0.14586300	3.82799900

P16_TS

C	0.45791000	1.66724000	-2.89774600
H	1.46962000	1.42991500	-2.56840500
H	0.39484300	2.73603400	-3.13006800
H	0.27989000	1.12790400	-3.83365400
C	-0.58449100	1.28473100	-1.89660000
H	-1.61617400	1.36225600	-2.24734300
C	-0.41934700	0.84377000	-0.64556100
P	1.10732700	0.70081000	0.29882700
C	2.26724400	2.07806800	0.05556000
C	3.34943800	2.07533700	-0.82160000
F	3.57675500	1.02467700	-1.61036400
C	4.20238900	3.16395500	-0.93229800
F	5.22314600	3.14006300	-1.77962300
C	3.97594200	4.29047500	-0.14990200
F	4.78219400	5.33452800	-0.25097900
C	2.90080300	4.32803600	0.73011800
F	2.68862700	5.40578700	1.47275400
C	2.06751800	3.22349600	0.82402000
F	1.04490600	3.27422800	1.67837500
C	2.01494200	-0.84351300	0.00161200
C	2.70655700	-1.39533600	1.08034300
F	2.85145800	-0.68603100	2.19913700
C	3.23330600	-2.67659300	1.03840500
F	3.87751800	-3.18081900	2.08099700
C	3.05845300	-3.44183700	-0.10920500
F	3.53969300	-4.67238800	-0.16039500
C	2.38029600	-2.91702500	-1.20234500
F	2.18937200	-3.66163600	-2.28345100
C	1.87440100	-1.62532000	-1.14185200
F	1.22152500	-1.16841500	-2.20626700
B	-1.54463500	0.25978000	0.38653800
C	-3.05060100	0.71060900	0.01430400
C	-4.15424000	-0.13884400	-0.01776800
F	-4.02147000	-1.44417600	0.24057100
C	-5.44470100	0.29785800	-0.28970200
F	-6.46280200	-0.56036800	-0.30873900
C	-5.67735200	1.64469500	-0.52767700
F	-6.90770500	2.07743000	-0.78293200
C	-4.61504100	2.53528500	-0.48801000
F	-4.82257800	3.83478700	-0.69808800
C	-3.34015100	2.05379100	-0.21958800
F	-2.36320800	2.97565400	-0.15221100
C	-1.23867000	-1.35157700	0.39642300
C	-0.48382200	-2.05853300	1.32402400
F	-0.01295900	-1.46013400	2.44111500
C	-0.05193500	-3.36703600	1.13604900
F	0.73411300	-3.95795400	2.03960700
C	-0.36900100	-4.02655100	-0.03855400
F	0.09790100	-5.25001000	-0.26499000
C	-1.12340700	-3.36962400	-1.00375600
F	-1.41765300	-3.98467400	-2.14552700
C	-1.53116800	-2.06555900	-0.76888700
F	-2.21165200	-1.46531500	-1.75174400
H	-0.07516100	0.68120000	1.53690700
C	-1.36071200	0.98257700	2.07183300
H	-1.67021900	2.00879300	1.85961400
C	-3.58665300	0.64868100	3.16441600
H	-3.95377300	1.59659900	2.77782100
C	-2.35625100	0.21879200	2.90318800
H	-2.01665200	-0.73299800	3.30036900
H	-4.26831100	0.06273700	3.77128100
H	-0.50534800	1.11906900	2.76769800

P16_P

C	-1.01966600	-1.96061500	-2.44260300
H	-1.79177700	-1.18788100	-2.46513800
H	-1.47806700	-2.90868300	-2.13731300
H	-0.65847400	-2.09162200	-3.46367900
C	0.12562300	-1.58994400	-1.54024600
H	1.11888400	-1.81643100	-1.92825100
C	0.12233500	-0.99774800	-0.33539800
P	-1.39479600	-0.60627600	0.48543100
C	-2.91366200	-1.51479600	0.08704700
C	-3.86346100	-1.08528400	-0.83609700
F	-3.67223900	0.04214600	-1.51705000
C	-5.00618100	-1.82329900	-1.09995100
F	-5.89514000	-1.39952800	-1.98543900
C	-5.21381200	-3.02192300	-0.42439700
F	-6.30049600	-3.73079600	-0.66788900
C	-4.28174000	-3.47995700	0.50002000
F	-4.48459700	-4.62160400	1.13831200
C	-3.14550500	-2.72224000	0.74556800
F	-2.25918900	-3.16921200	1.63029300

C	-1.71820000	1.16415700	0.47194100
C	-1.87276700	1.86257500	1.66720300
F	-1.96973800	1.20066300	2.81697500
C	-1.89980800	3.24945300	1.69517300
F	-2.03357700	3.89910300	2.83986600
C	-1.77323100	3.95247600	0.50372300
F	-1.77682700	5.27029000	0.51568000
C	-1.62012000	3.27848800	-0.70579800
F	-1.45196300	3.96534300	-1.82614700
C	-1.58046300	1.89596300	-0.70940400
F	-1.37786300	1.26427600	-1.86134100
B	1.46200700	-0.45232700	0.52347900
C	2.73459600	-1.36829000	0.05993400
C	3.99369800	-0.88213000	-0.27772000
F	4.22124400	0.43607600	-0.36909500
C	5.09285200	-1.69698800	-0.52480800
F	6.27280800	-1.16574300	-0.84538400
C	4.96177600	-3.07301200	-0.42730500
F	6.00347800	-3.86921400	-0.65811200
C	3.73047300	-3.61045800	-0.08427500
F	3.58579700	-4.93294600	0.02173200
C	2.66130300	-2.75733700	0.15311100
F	1.50415500	-3.35328800	0.50354200
C	1.57717800	1.13653700	0.07782400
C	1.32274100	2.23991400	0.88637200
F	1.00723700	2.10316400	2.19169200
C	1.30170700	3.55335000	0.42564700
F	0.96273500	4.55027000	1.25173500
C	1.52185300	3.81706100	-0.91520300
F	1.45450500	5.06108500	-1.37983300
C	1.78912400	2.75477900	-1.76948200
F	2.00404200	2.98320600	-3.06368300
C	1.81001300	1.46446100	-1.25969600
F	2.07747600	0.49171600	-2.14334600
H	-1.25726400	-0.90339900	1.83754200
C	1.30310900	-0.68090900	2.15154400
H	1.07150300	-1.74206600	2.31980000
C	3.44195400	-1.21557000	3.35770500
H	3.27174400	-2.28612300	3.26497100
C	2.56297000	-0.33351700	2.88733000
H	2.77430300	0.72971300	2.99585300
H	4.35877700	-0.90251500	3.84621600
H	0.48751000	-0.09144300	2.59201400

P19_TS

P	1.22957900	0.60329500	-0.11286900
B	-1.76901600	0.39093400	-0.01426600
C	0.32852600	0.57737100	-1.72109800
H	0.22965100	-0.48098500	-1.98045200
C	-1.03956000	1.14453500	-1.29797500
H	-1.70568700	1.08175700	-2.16673600
H	-0.92294700	2.21724500	-1.09117000
C	0.99810700	1.32079300	-2.87065600
H	0.34800800	1.26872800	-3.74838900
H	1.96505100	0.88451700	-3.13120000
H	1.13822000	2.37921500	-2.62897500
C	2.36518900	2.02396600	-0.13093700
C	3.56856500	2.01376900	-0.83543400
C	4.42064400	3.10717600	-0.83989400
C	4.06109700	4.25178300	-0.13498600
C	2.86198100	4.29918700	0.56486800
C	2.03165700	3.18640200	0.55840600
C	2.28991200	-0.84919500	0.12511300
C	2.67237300	-1.72183300	-0.88973400
C	3.33088900	-2.91144900	-0.61226000
C	3.63637600	-3.23152700	0.70586200
C	3.29616600	-2.36444000	1.73758200
C	2.63300300	-1.18692900	1.43361300
C	-1.39249700	-1.20852200	-0.05021100
C	-1.81335500	-1.95211000	-1.15607900
C	-1.47431400	-3.27608100	-1.38151800
C	-0.65594400	-3.93390600	-0.46827800
C	-0.21274600	-3.24859600	0.64783100
C	-0.59752100	-1.92317500	0.83531900
C	-3.37182000	0.68912900	-0.07483100
C	-3.84662400	1.97946200	-0.29932100
C	-5.19240700	2.31958500	-0.33865000
C	-6.14581400	1.33071400	-0.14984700
C	-5.72900300	0.03021900	0.08725800
C	-4.37070400	-0.25984600	0.12896700
F	3.92373400	0.93693300	-1.53694300
F	5.56463700	3.07493600	-1.50987600
F	4.86402400	5.30247200	-0.13679800
F	2.52371200	5.39601600	1.22779700
F	0.88335900	3.25340000	1.23186100
F	2.40936400	-1.43518400	-2.16379100
F	3.68244400	-3.73337100	-1.59235700
F	4.26636900	-4.36137800	0.97835900

F	3.60155500	-2.67082800	2.99014600
F	2.31157700	-0.35982900	2.42710900
F	-2.58664500	-1.36432800	-2.07929500
F	-1.91110000	-3.92107100	-2.45971900
F	-0.30088600	-5.19799400	-0.67099300
F	0.59703000	-3.84586700	1.52670700
F	-0.07874900	-1.33656300	1.93839200
F	-2.98305000	3.00254700	-0.45322800
F	-5.57109100	3.57962700	-0.55271000
F	-7.44145300	1.62816700	-0.19092800
F	-6.63205100	-0.92934600	0.28662800
F	-4.06361000	-1.53323700	0.40942300
H	-0.17129700	0.75022300	0.87069300
C	-1.38370400	1.14447500	1.61550600
H	-1.73201300	2.15971500	1.40312800
H	-0.42891100	1.31470200	2.14787400
C	-3.39396000	0.92379000	3.09246500
H	-3.80044200	1.86848200	2.73841500
C	-2.24246500	0.44351300	2.63285000
H	-1.87029000	-0.50552400	3.00649900
H	-3.97278900	0.38236600	3.83305200

P19_P

B	-0.85552300	-0.52768500	0.73852600
C	-0.15799100	-1.84135400	0.02006300
C	-0.66996600	-3.07482500	0.47483300
H	-1.40734600	-3.05627500	1.27486300
C	-0.32666800	-4.30286500	-0.06959500
H	-0.76569000	-5.21435300	0.32451600
C	0.54994700	-4.35577300	-1.15199000
H	0.79259200	-5.29873600	-1.63078600
C	1.11094600	-3.17279400	-1.60384400
H	1.79794800	-3.19520300	-2.44763800
C	0.80347900	-1.94067900	-1.00382400
C	1.63628400	-0.76132700	-1.46911600
H	1.05227600	0.12616200	-1.71791800
H	2.23250900	-1.02913100	-2.34842200
C	4.02181400	-1.62746000	0.26310800
C	5.15436900	-0.97704600	1.09122500
H	4.74175800	-0.66028100	2.05952000
H	5.87385600	-1.77614700	1.30820600
C	5.86230000	0.21255900	0.44435900
H	6.27295800	-0.06467400	-0.53384100
H	6.72018200	0.48779100	1.06473400
C	4.93951700	1.42422800	0.32227700
H	5.50153800	2.30296300	-0.01799300
H	4.55071900	1.67252100	1.31942900
C	3.75106300	1.25274900	-0.64392700
C	2.79920400	2.44466100	-0.49493600
H	2.47278300	2.57417000	0.54066800
H	1.91253800	2.34083300	-1.12928100
H	3.32552700	3.35490700	-0.80192800
C	4.21982700	1.17252600	-2.10398800
H	3.37360300	1.09628600	-2.79251400
H	4.89947300	0.34106500	-2.29933600
H	4.75385400	2.09811700	-2.34236200
C	4.57124800	-2.29486300	-1.00542900
H	3.78116300	-2.83178300	-1.53423300
H	5.32439900	-3.02954500	-0.70171900
H	5.04941000	-1.60111400	-1.69954500
C	3.34351100	-2.69677600	1.13458600
H	3.01279300	-2.27433100	2.08424300
H	4.08899200	-3.47170400	1.34565600
H	2.48938800	-3.16702400	0.64178700
C	-2.49882800	-0.60392300	0.44358000
C	-3.39327000	0.12836200	1.22545200
F	-2.94190000	0.92469400	2.21121700
C	-4.77124900	0.12776100	1.05047000
F	-5.56208700	0.84538300	1.85216100
C	-5.32477200	-0.62201300	0.02470300
F	-6.64380700	-0.64001900	-0.16509100
C	-4.48304400	-1.34057700	-0.80778000
F	-4.99632200	-2.04979500	-1.81608700
C	-3.10820800	-1.30922400	-0.59621300
F	-2.38849400	-2.00524000	-1.48793600
C	-0.50159600	0.99306800	0.14105100
C	-0.91071500	1.33335700	-1.15323700
F	-1.27993300	0.35784600	-1.99954000
C	-0.94788200	2.62382600	-1.66146800
F	-1.35402100	2.86128700	-2.90878500
C	-0.56078700	3.68211600	-0.84649300
F	-0.57849500	4.92992500	-1.30909900
C	-0.11842300	3.40748300	0.43476500
F	0.34946800	4.39347700	1.20412800
C	-0.08303600	2.09004400	0.88644900
F	0.47841400	1.93692500	2.10115500
H	2.11072500	0.01376900	1.01073500
P	2.82350300	-0.27911800	-0.16900600

C	-0.63154500	-0.63974100	2.38832500
H	-1.37175600	-1.37248900	2.74111500
C	1.61367300	-0.43506200	3.54629200
H	1.43478500	0.59159300	3.85120500
C	0.70267700	-1.12442200	2.85068100
H	0.93468500	-2.15895000	2.58840700
H	2.54711400	-0.89022600	3.87179400
H	-0.87438300	0.29454700	2.89170200

P20_TS

P	-2.70554700	-0.19425600	0.04195700
C	-2.77845000	-0.49725200	1.84488700
C	-3.82231200	-0.99124900	2.63227800
C	-3.56136400	-1.45845000	3.92929900
C	-2.26869900	-1.52665600	4.45660800
C	-1.19916800	-1.07320700	3.67974800
C	-1.52964300	-0.51916600	2.44003500
O	-0.43925700	-0.14332800	1.74012200
C	0.64985000	-0.64402100	2.41638000
C	0.26712800	-1.14661800	3.66750400
C	1.27528000	-1.69715500	4.46228000
C	2.56155800	-1.74807800	3.93036600
C	2.84816100	-1.32375900	2.62216500
C	1.87429700	-0.76706100	1.78419000
B	2.02911100	-0.39000500	0.20053800
C	3.08587500	-1.37562800	-0.54054400
C	4.09404100	-0.98301500	-1.41760400
F	4.29091200	0.30789000	-1.71096600
C	4.94875500	-1.87881600	-2.05118400
F	5.90142900	-1.44017000	-2.87411400
C	4.80285900	-3.23942900	-1.83263200
F	5.60703400	-4.10955600	-2.43843400
C	3.79919700	-3.68510600	-0.98471900
C	2.97273100	-2.75604500	-0.36890300
F	1.99110600	-3.25894100	0.40086500
F	3.63260900	-4.99228700	-0.77860200
C	2.37286200	1.21030000	0.11748000
C	1.54086000	2.26742200	-0.22874300
F	0.29013600	2.06025100	-0.69989400
C	1.89478400	3.60820400	-0.11449200
F	1.02685300	4.56656700	-0.45641500
C	3.14571200	3.94546400	0.36877300
F	3.50305300	5.22153400	0.49272600
C	4.02239700	2.93005100	0.73041800
C	3.61856700	1.61115100	0.60520800
F	4.50556300	0.67775100	0.98115800
F	5.23023800	3.23572700	1.20229500
C	-2.67096300	1.57496300	-0.43580200
C	-2.61469700	1.79663600	-1.83314700
C	-2.86556700	0.70629400	-2.84783200
C	-2.33562400	3.07616400	-2.30746800
C	-2.09894600	4.14618200	-1.44968000
C	-1.71443600	5.49960600	-1.98178000
C	-2.19801400	3.91433800	-0.08229500
C	-2.47927000	2.65541500	0.45062500
C	-2.53464900	2.54938500	1.95555500
C	-4.22272800	-0.99119900	-0.58488600
C	-4.08116800	-2.27397800	-1.15084500
C	-2.74537500	-2.97710900	-1.22243000
C	-5.21273300	-2.91479000	-1.65172600
C	-6.47282100	-2.32145900	-1.60672900
C	-7.67679400	-3.01724400	-2.18483700
C	-6.58992200	-1.05985000	-1.02595300
C	-5.48791900	-0.37712200	-0.51065400
C	-5.69738800	0.98950500	0.09460700
H	-4.82809000	-1.05442700	2.22613200
H	-4.38961200	-1.82954600	4.52346000
H	-2.10417100	-1.96892400	5.43375900
H	1.06354200	-2.10333500	5.44587100
H	3.35993800	-2.17812800	4.52663100
H	3.85234500	-1.46804300	2.23273300
H	-2.59234800	1.05568000	-3.84563700
H	-2.26865900	-0.19066100	-2.64962500
H	-3.91870200	0.40769800	-2.86426100
H	-2.29047800	3.23453300	-3.38258200
H	-2.06144700	6.29874700	-1.32254600
H	-0.62498600	5.57574600	-2.04960700
H	-2.12814900	5.66787700	-2.97882500
H	-2.03506800	4.74098200	0.60528500
H	-2.61539700	3.54830600	2.38838900
H	-1.62468100	2.09072400	2.35466000
H	-3.38306500	1.95968000	2.30766200
H	-2.86688300	-3.98036300	-1.63473200
H	-2.28861900	-3.07491200	-0.23162100
H	-2.03293000	-2.43601200	-1.85685700
H	-5.10678100	-3.90601100	-2.08591300
H	-7.82741300	-2.72299000	-3.22871100
H	-8.58471300	-2.75830400	-1.63515300

H	-7.55652300	-4.10256900	-2.16112700
H	-7.57013900	-0.59203100	-0.96586400
H	-6.75740800	1.15396800	0.29796000
H	-5.14829000	1.10891700	1.03372400
H	-5.35045900	1.77927100	-0.57904900
H	-0.95825600	-0.65576900	-0.47080600
C	0.44717800	-0.80806400	-0.66575600
H	0.10872200	0.16379700	-0.30486400
H	0.27700000	-1.71044800	-0.07885800
C	0.44771600	-0.01084800	-3.03918700
H	0.11272700	0.98321300	-2.75362100
C	0.63192600	-0.96929300	-2.13064900
H	0.98192600	-1.94887900	-2.45328600
H	0.63988700	-0.19267500	-4.09088400

P20_P

P	-2.92725800	-0.37444600	0.00622500
C	-2.66383200	-0.78211600	1.73038300
C	-3.65523800	-1.13929800	2.65488000
C	-3.30098200	-1.49231700	3.95526000
C	-1.96293000	-1.53886500	4.36568500
C	-0.95718600	-1.23730000	3.45248300
C	-1.34388800	-0.85840800	2.15312600
O	-0.29365500	-0.63490400	1.35032200
C	0.84893400	-0.90383900	2.10182900
C	0.49722400	-1.23985500	3.41585400
C	1.52404800	-1.49490800	4.32853400
C	2.82705000	-1.38892200	3.86115000
C	3.11029400	-1.07029700	2.52188600
C	2.12318100	-0.83699300	1.55634100
B	2.32179500	-0.40403200	-0.01700700
C	3.85871800	-0.64373300	-0.54827000
C	4.52967500	0.23110700	-1.39989800
F	3.97149000	1.39951100	-1.76301000
C	5.78227100	-0.02013100	-1.94560400
F	6.36703200	0.87017300	-2.75262000
C	6.42727700	-1.21150400	-1.65384100
F	7.62747800	-1.47694800	-2.17227800
C	5.80138300	-2.12662000	-0.82344000
C	4.54727500	-1.83113400	-0.30185500
F	4.01354400	-2.78123300	0.48495100
F	6.40406400	-3.28478100	-0.53843700
C	1.91834600	1.20585300	0.03135900
C	0.73902400	1.79156200	-0.40595000
F	-0.21775000	1.07527800	-1.05174800
C	0.41583200	3.13359600	-0.22800800
F	-0.74757900	3.61953900	-0.68559700
C	1.30159000	3.96824000	0.42791300
F	1.01086900	5.25878600	0.60704600
C	2.49614700	3.43815300	0.89826400
C	2.76553800	2.09291400	0.69903700
F	3.92953800	1.64701100	1.19349200
F	3.36105500	4.22588400	1.54027500
C	-3.25393400	1.34460700	-0.43858400
C	-3.10467500	1.63627800	-1.81439600
C	-2.67740600	0.60665000	-2.83296000
C	-3.33108900	2.94123000	-2.23987700
C	-3.66956600	3.95740600	-1.35035900
C	-3.81662900	5.37647900	-1.82406300
C	-3.80336100	3.63907100	-0.00127800
C	-3.59762200	2.35078600	0.48608300
C	-3.73760100	2.12988700	1.97240000
C	-4.08764900	-1.56846300	-0.69518900
C	-3.54050200	-2.78784300	-1.15855200
C	-2.06389400	-3.10729300	-1.09703900
C	-4.41300000	-3.72613100	-1.70174700
C	-5.78653200	-3.49588800	-1.79333600
C	-6.69098700	-4.53621000	-2.39759100
C	-6.29243100	-2.29019400	-1.31472600
C	-5.46757100	-1.31006900	-0.76249600
C	-6.09408000	-0.02364500	-0.27916800
H	-4.69994600	-1.14763600	2.35567100
H	-4.08209100	-1.75437000	4.66017900
H	-1.71663500	-1.83197100	5.38120900
H	1.31196200	-1.75713300	5.35989200
H	3.65161900	-1.56444500	4.54467900
H	4.15101600	-1.00458300	2.21817400
H	-2.71104600	1.03842700	-3.83400100
H	-1.64475100	0.28903700	-2.64921100
H	-3.31921500	-0.27986300	-2.83366000
H	-3.21221400	3.17327300	-3.29500900
H	-4.51050200	5.93897500	-1.19568800
H	-2.84367600	5.87704100	-1.77862000
H	-4.16591600	5.41902900	-2.85792700
H	-4.05802700	4.42575300	0.70408200
H	-4.03291900	3.06318900	2.45397400
H	-2.79789600	1.80041000	2.42347900
H	-4.49241500	1.37730600	2.21750900

H	-1.89254300	-4.14999500	-1.36652300
H	-1.63890100	-2.95519600	-0.09852700
H	-1.47729300	-2.49603000	-1.79358100
H	-4.00753600	-4.66860100	-2.06140100
H	-6.47268800	-4.66414000	-3.46207900
H	-7.74171600	-4.25805100	-2.29791500
H	-6.54452500	-5.50664300	-1.91569600
H	-7.36181800	-2.10303900	-1.36839000
H	-7.17163800	-0.15394800	-0.16448300
H	-5.69606800	0.29814700	0.68829800
H	-5.92261300	0.79580200	-0.98459900
H	-1.69371100	-0.64603500	-0.58784300
C	1.35093600	-1.26794700	-1.05032400
H	1.49814400	-0.89549500	-2.07378500
H	0.30246300	-1.08687900	-0.79720900
C	1.95360900	-3.49862400	-2.05339700
H	2.13000300	-3.05873300	-3.03234100
C	1.57379700	-2.74573800	-1.02117400
H	1.43188700	-3.22790900	-0.05096900
H	2.12525600	-4.56580100	-1.95335200

P21_TS

P	-2.03802700	0.08995800	0.51459600
C	-2.42029800	1.83293900	0.08777300
C	-3.64441600	2.47325700	0.30427500
C	-3.76869400	3.83784600	0.06750000
C	-2.68102000	4.57839600	-0.39339800
C	-1.44334000	3.97718300	-0.61358500
C	-1.34331200	2.61320300	-0.33999500
O	-0.16292600	1.95335800	-0.50848500
C	1.01162200	2.67472000	-0.31480200
C	2.09466800	1.93295500	0.15070100
C	3.25959600	2.67555400	0.37656800
C	3.31689500	4.04885800	0.15358100
C	2.20319000	4.73127800	-0.32539200
C	1.01804500	4.04328000	-0.58115900
C	-0.22290600	4.65996200	-1.21934200
C	-0.27470900	6.17783700	-1.04900900
C	-0.19187700	4.31817200	-2.72697500
B	1.92483000	0.36592700	0.55921700
C	1.29810100	-0.63532800	-0.58444600
C	1.06760100	-1.97211000	-0.26087100
F	1.20985600	-2.38243800	1.01779200
C	0.69748100	-2.94467200	-1.17556900
F	0.44911500	-4.19786800	-0.78526200
C	0.52892200	-2.58893600	-2.50473600
F	0.14433400	-3.49721900	-3.40128100
C	0.73708600	-1.27363100	-2.88154300
C	1.13395700	-0.33579200	-1.93418800
F	1.35484300	0.89724500	-2.41081900
F	0.52331300	-0.90643100	-4.14643800
C	3.38110200	-0.32796800	0.89053400
C	4.33874100	-0.27055700	-0.12642100
F	4.05236900	0.36932300	-1.27023700
C	5.59469400	-0.85385700	-0.04845500
F	6.46336400	-0.74612900	-1.05393600
C	5.93818500	-1.57253500	1.08838100
F	7.13478400	-2.14716000	1.18624800
C	5.01343400	-1.69377200	2.11106200
C	3.76520600	-1.08967800	1.98917200
F	2.92971200	-1.31640600	3.01873700
F	5.31640300	-2.39910600	3.20208900
C	-3.50859900	-0.34807400	1.55053800
C	-3.34244500	-0.37584500	2.95003500
C	-2.03946200	0.00111900	3.61225200
C	-4.40518800	-0.75783200	3.76582500
C	-5.64272500	-1.11449200	3.23676600
C	-6.76585800	-1.56977100	4.13035600
C	-5.80381700	-1.04258800	1.85689100
C	-4.76958500	-0.66283900	0.99740000
C	-5.09754200	-0.59562700	-0.47736100
C	-2.19194800	-1.07122700	-0.90937000
C	-2.31073900	-0.71064500	-2.26767600
C	-2.13332100	0.69623300	-2.78193700
C	-2.57372500	-1.70956400	-3.20500100
C	-2.70389500	-3.05169000	-2.85386100
C	-2.95441100	-4.10495200	-3.89870800
C	-2.50676300	-3.39433400	-1.52039400
C	-2.23542200	-2.43433300	-0.54392100
C	-1.97871400	-2.93082700	0.86155300
H	-4.49260000	1.90807600	0.67661200
H	-4.72044300	4.32864300	0.23985300
H	-2.80590500	5.63932900	-0.58275600
H	4.14098700	2.17159000	0.76322500
H	4.23671400	4.59041400	0.34957900
H	2.26298800	5.79948800	-0.50365800
H	0.59789800	6.64338200	-1.51271500
H	-0.30466400	6.46388700	0.00650400

H	-1.15348200	6.59104600	-1.55110000
H	0.67187000	4.79788200	-3.19716000
H	-0.10162500	3.23852600	-2.87921500
H	-1.10634100	4.66971400	-3.21582400
H	-2.13868500	-0.02314000	4.69891800
H	-1.23575100	-0.69036400	3.33831000
H	-1.73028100	1.01859300	3.34046000
H	-4.26084000	-0.77595600	4.84356800
H	-6.73299000	-1.06203300	5.09731600
H	-7.73919400	-1.37897700	3.67281100
H	-6.69427500	-2.64570900	4.32043200
H	-6.77348200	-1.28138200	1.42524200
H	-6.17434400	-0.46044200	-0.60365700
H	-4.80697400	-1.51405900	-0.99741900
H	-4.59193900	0.23299300	-0.97834300
H	-2.14805700	0.69359500	-3.87340400
H	-1.16998400	1.10541200	-2.46355800
H	-2.91820200	1.37394300	-2.43208900
H	-2.66392600	-1.42584800	-4.25099300
H	-2.00142300	-4.46221300	-4.30054700
H	-3.54118100	-3.70748700	-4.73017700
H	-3.48433400	-4.96292500	-3.47848900
H	-2.53661600	-4.44113000	-1.22615300
H	-1.54959400	-3.93404000	0.81741000
H	-1.26198800	-2.30065800	1.39812800
H	-2.89809200	-2.96944300	1.45457500
H	-0.12642900	0.38153800	1.25618600
C	0.99175700	0.36681000	2.02549100
H	0.92403100	-0.62272300	2.47378200
H	-0.04963500	0.79492600	2.21800600
C	1.87670900	1.08639300	4.25849200
H	1.73343200	0.09070400	4.66561600
C	1.57806700	1.38072400	2.99742100
H	1.74033800	2.37893000	2.60308700
H	2.30988100	1.83628100	4.91176000

P21_P

P	-2.28218000	0.09294000	0.39943000
C	-2.44206300	1.85784800	0.09268500
C	-3.67719700	2.49811800	0.25924600
C	-3.77882500	3.86062800	0.03003600
C	-2.65725400	4.58277400	-0.38292600
C	-1.41831200	3.97539400	-0.55928800
C	-1.31810400	2.60563800	-0.28348700
O	-0.15065900	1.94655700	-0.40964000
C	1.04248000	2.67647600	-0.20264000
C	2.13644700	1.94394900	0.25621000
C	3.28538000	2.70924900	0.49133500
C	3.32672700	4.08447400	0.28197000
C	2.20995200	4.75197900	-0.20666400
C	1.03852000	4.04392000	-0.47315700
C	-0.18784700	4.65292400	-1.14297200
C	-0.24970500	6.17270400	-0.99449100
C	-0.12615600	4.29274700	-2.64601300
B	2.01090600	0.37397200	0.69107500
C	1.31851500	-0.59800500	-0.47149000
C	1.04764800	-1.93576800	-0.17438800
F	1.18139100	-2.38772800	1.08853700
C	0.66282500	-2.88801200	-1.10785800
F	0.38754300	-4.14505600	-0.73692400
C	0.50990200	-2.51601200	-2.43323800
F	0.11417300	-3.40739100	-3.34689800
C	0.73874200	-1.19786300	-2.78591800
C	1.15499400	-0.28482600	-1.82148300
F	1.39605100	0.94828700	-2.29490600
F	0.52767900	-0.80534500	-4.04685000
C	3.48868200	-0.33164600	0.86531000
C	4.42544500	-0.22355300	-0.16340000
F	4.12310000	0.48688800	-1.26354800
C	5.67357900	-0.82905100	-0.14687800
F	6.53075900	-0.67270900	-1.15949900
C	6.02159600	-1.62671800	0.93433300
F	7.21214100	-2.22617000	0.97243000
C	5.11164200	-1.80465100	1.96295500
C	3.87213500	-1.17321100	1.90544700
F	3.04191000	-1.45491800	2.91943800
F	5.42535600	-2.59612200	2.99257400
C	-3.63761900	-0.36018200	1.51522300
C	-3.39251600	-0.36094800	2.90428100
C	-2.07364200	0.05964600	3.50443000
C	-4.41859800	-0.76677200	3.75487300
C	-5.66793100	-1.15455100	3.27461300
C	-6.74300200	-1.62695100	4.21606300
C	-5.89362100	-1.09842200	1.90108400
C	-4.90382500	-0.70681300	1.00037400
C	-5.26253600	-0.66772000	-0.46894100
C	-2.28992400	-1.05283400	-1.00117000
C	-2.37096400	-0.65118400	-2.35018500

C	-2.16027600	0.76689100	-2.81917400
C	-2.59309900	-1.63516900	-3.31133800
C	-2.71629600	-2.98497800	-2.98476400
C	-2.91959700	-4.02008200	-4.05576000
C	-2.54817500	-3.35876500	-1.65392900
C	-2.32052300	-2.42109500	-0.64821800
C	-2.08380100	-2.93639200	0.75395000
H	-4.54267000	1.93219900	0.58822300
H	-4.72921000	4.36335400	0.16769700
H	-2.76036300	5.64518800	-0.57671100
H	4.17198200	2.20950300	0.87267600
H	4.23673200	4.63813800	0.49084100
H	2.25565000	5.82041800	-0.38551600
H	0.63469100	6.63175200	-1.44068200
H	-0.30601400	6.47301500	0.05592800
H	-1.11390600	6.57900100	-1.52731800
H	0.74354300	4.77564800	-3.10104500
H	-0.01544800	3.21295500	-2.78247800
H	-1.03247500	4.62899200	-3.16116800
H	-2.16063700	0.13465600	4.58910400
H	-1.27419600	-0.65796300	3.28998800
H	-1.73719800	1.03377900	3.13403600
H	-4.23469800	-0.77310500	4.82617300
H	-7.73787300	-1.46323500	3.79652800
H	-6.63555600	-2.69927800	4.40901200
H	-6.68163400	-1.11155100	5.17710300
H	-6.87440200	-1.36275400	1.51293100
H	-6.34555500	-0.57941500	-0.57635900
H	-4.94221200	-1.57813900	-0.98574500
H	-4.80787300	0.17819700	-0.99334400
H	-2.15854300	0.79452500	-3.90975400
H	-1.18859600	1.13890900	-2.47712900
H	-2.93368500	1.45401400	-2.46241400
H	-2.64689900	-1.33577700	-4.35478000
H	-3.45415700	-4.89114000	-3.67061800
H	-1.94564800	-4.35719600	-4.42287900
H	-3.47677100	-3.61277500	-4.90218600
H	-2.56095900	-4.41300600	-1.38869800
H	-1.66774100	-3.94345800	0.69474400
H	-1.35283000	-2.33598400	1.30632900
H	-3.00816300	-2.97299800	1.33889400
H	-1.08103700	-0.09536500	1.08884700
C	1.12724900	0.42356100	2.09191200
H	0.83521900	-0.56858500	2.45325800
H	0.21037700	0.97724900	1.83331000
C	1.93179100	0.76528200	4.45864000
H	1.65554600	-0.24498000	4.74975400
C	1.74998500	1.20030900	3.21319300
H	2.06174800	2.21585500	2.96429600
H	2.38702200	1.39124400	5.21990700

P22_TS

P	2.09926700	1.25434900	1.35013500
C	3.30894700	-0.02374700	0.88961000
C	4.58239800	-0.21843900	1.42856500
C	5.34278500	-1.31334900	1.02755700
C	4.84875700	-2.20704200	0.07576200
C	3.59210000	-2.02923100	-0.50188400
C	2.84708400	-0.93701800	-0.06292800
O	1.59682800	-0.70909100	-0.55438400
C	0.83243200	-1.81806100	-0.88946800
C	-0.53790400	-1.71149900	-0.64316900
C	-1.29094100	-2.81877400	-1.05014500
C	-0.69787000	-3.95870000	-1.58559200
C	0.68264200	-4.02837400	-1.74552600
C	1.48116700	-2.93651300	-1.40909300
C	2.99250900	-2.86286000	-1.63145100
C	3.24451900	-2.10262900	-2.95348600
C	3.63190800	-4.24808700	-1.71612000
B	-1.14258900	-0.41191700	0.10172700
C	-0.81886700	0.98922000	-0.65758600
C	-0.79864000	1.05333100	-2.05170800
F	-0.94430900	-0.05186800	-2.78999700
C	-0.68257900	2.24216800	-2.76275800
F	-0.67518900	2.23919200	-4.09495500
C	-0.56362300	3.44261100	-2.07786400
F	-0.40983200	4.58643500	-2.73978700
C	-0.59659400	3.43064900	-0.69261800
C	-0.74081200	2.22292800	-0.02600300
F	-0.81137800	2.30295600	1.32047400
F	-0.43985100	4.56706400	-0.00530200
C	-2.77080600	-0.51929000	0.37655400
C	-3.72851600	0.40330800	-0.05804800
F	-3.40265600	1.51523300	-0.73340300
C	-5.09751000	0.26706100	0.16042100
F	-5.93961300	1.19848800	-0.28421100
C	-5.58690300	-0.83472200	0.83801300
F	-6.89056900	-0.97620300	1.05252500

C	-4.68833100	-1.78790000	1.29201200
C	-3.33440200	-1.61106200	1.04971800
F	-2.54412300	-2.58909100	1.53419700
F	-5.12670000	-2.86236600	1.94576000
C	2.54441300	1.85308000	3.05005400
C	2.45304800	0.76271900	4.11930600
C	1.63002400	3.03444200	3.40010600
C	2.52014200	2.70114300	0.24726500
C	3.84803300	3.36243500	0.62552200
C	2.55510100	2.25458200	-1.21695100
H	4.97032800	0.46865600	2.17582500
H	6.32657900	-1.47287100	1.45531900
H	5.46310500	-3.05066500	-0.22142300
H	-2.37043100	-2.79977300	-0.94001700
H	-1.31509500	-4.80273700	-1.87570700
H	1.13025800	-4.93190100	-2.14555200
H	2.79376100	-2.64838600	-3.78766900
H	4.31936000	-1.99786500	-3.13375900
H	2.79683600	-1.10511600	-2.91633300
H	3.21755600	-4.80891200	-2.55704000
H	4.70746900	-4.16467900	-1.89346800
H	3.46789300	-4.82066000	-0.79847200
H	3.58201600	2.20506600	3.00306200
H	2.75481800	1.18085300	5.08450000
H	3.10662900	-0.08625800	3.90082300
H	1.42965100	0.38993700	4.22308200
H	0.58123600	2.72284700	3.41158200
H	1.72049000	3.86176500	2.69054400
H	1.88332300	3.41419600	4.39415700
H	1.70559800	3.42040600	0.38812000
H	4.09220900	4.12324200	-0.12221900
H	3.81708200	3.85602900	1.59983100
H	4.66219500	2.62884500	0.62801500
H	2.61477900	3.13455700	-1.86489900
H	3.44021500	1.63650700	-1.40258100
H	1.67548000	1.67362600	-1.49599700
H	0.56508000	0.39477200	1.28754400
C	-0.33136200	-0.55933700	1.89379200
H	-0.41471600	0.23534600	2.64875300
H	-1.28156100	-1.06973800	2.02123700
C	0.59634300	-2.89852200	2.08526200
H	-0.30537100	-3.35234900	1.68621500
C	0.71315800	-1.58053800	2.24240600
H	1.65991100	-1.21028000	2.62905000
H	1.41951000	-3.55983500	2.33700500

P22_P

P	2.76893000	1.15185300	1.11126500
C	3.39429300	-0.48929500	0.80462200
C	4.66369700	-0.88437300	1.24366100
C	5.14707400	-2.13614100	0.90010100
C	4.39299600	-2.95742100	0.05868900
C	3.14274300	-2.57917100	-0.42044400
C	2.61291700	-1.35955100	0.02362800
O	1.37218800	-0.95666600	-0.32179100
C	0.42403500	-1.93561800	-0.69787000
C	-0.92035000	-1.61757500	-0.47738900
C	-1.82443700	-2.60158400	-0.89134700
C	-1.41878300	-3.81679000	-1.43341000
C	-0.06799800	-4.08812600	-1.59978900
C	0.88448900	-3.13361500	-1.24311700
C	2.37424300	-3.31049800	-1.51021200
C	2.70443100	-2.60453800	-2.84645300
C	2.78368600	-4.78025800	-1.61062200
B	-1.38258200	-0.28337400	0.36152400
C	-0.81623000	1.07374500	-0.41158300
C	-0.79827400	1.14285100	-1.80984500
F	-1.27587100	0.12963800	-2.53762800
C	-0.35468100	2.24067000	-2.53447300
F	-0.35914400	2.23023200	-3.86809700
C	0.12612700	3.35638400	-1.86160400
F	0.63388700	4.39151200	-2.53191200
C	0.10274400	3.35541500	-0.47974900
C	-0.39110300	2.24508700	0.19995300
F	-0.38013800	2.36906600	1.55001100
F	0.62170400	4.39047400	0.20240800
C	-3.04148700	-0.18079000	0.42936200
C	-3.85438900	0.73481100	-0.23930800
F	-3.34601000	1.67307400	-1.05803300
C	-5.24148600	0.77999800	-0.12423000
F	-5.94484700	1.69530900	-0.79476600
C	-5.89694300	-0.13105500	0.68436600
F	-7.22345400	-0.10285400	0.80438600
C	-5.14500200	-1.07435900	1.36694800
C	-3.76470700	-1.07506000	1.22167800
F	-3.12168600	-2.03847800	1.90473300
F	-5.75107200	-1.97030500	2.14908100
C	3.27466500	1.85292100	2.71959900

C	2.82968800	0.94102400	3.86712000
C	2.66334700	3.25924800	2.82725700
C	3.26817900	2.26783000	-0.25457900
C	4.75359300	2.62079500	-0.14094000
C	2.94229200	1.59611000	-1.59338100
H	5.25932800	-0.21660800	1.86109700
H	6.11690100	-2.46479300	1.25504200
H	4.80893800	-3.91194900	-0.24516200
H	-2.88722900	-2.41408600	-0.77548500
H	-2.15821600	-4.55564600	-1.72625300
H	0.24247200	-5.03892900	-2.01785200
H	2.15267700	-3.08243600	-3.66109300
H	3.77748700	-2.66227100	-3.05857800
H	2.40820100	-1.55154800	-2.81069100
H	2.25553700	-5.26626900	-2.43299600
H	3.85044400	-4.87135500	-1.83239600
H	2.56403700	-5.32166800	-0.68609000
H	4.36928000	1.92475100	2.70872700
H	3.11650200	1.39954400	4.81722100
H	3.29529500	-0.04583600	3.80741400
H	1.74310200	0.80751300	3.86851600
H	2.86826800	3.66111200	3.82266800
H	1.57807600	3.22849200	2.68781100
H	3.08082900	3.95271200	2.09237700
H	2.65491500	3.16864000	-0.13039900
H	5.04182400	3.22777700	-1.00293400
H	4.98239500	3.19317300	0.76234200
H	5.37099900	1.71591700	-0.15150800
H	3.03630900	2.33224400	-2.39657600
H	3.64333100	0.77770500	-1.78709300
H	1.92726600	1.19082300	-1.61446400
H	1.37952000	1.05720900	1.12709400
C	-0.77101900	-0.45028100	1.92486900
H	0.05626800	0.23587800	2.13008600
H	-1.55689500	-0.15451900	2.63295300
C	0.92647400	-2.14166500	2.71212300
H	1.69133300	-1.38234100	2.85819000
C	-0.29616800	-1.82740700	2.28395600
H	-1.01240900	-2.63525000	2.15346000
H	1.21241600	-3.16491300	2.93514400

P23_TS

C	0.88455600	2.59612000	-1.78549000
H	0.79028400	3.57868300	-1.31289000
H	0.09922900	2.50004900	-2.53908300
H	1.85577700	2.57229100	-2.29013600
C	0.78937700	1.49125000	-0.76401600
C	-0.31643300	0.78625200	-0.42594400
P	2.19013500	1.20639200	0.35200500
C	3.53988200	0.34567800	-0.51076000
C	3.42001700	-0.03463900	-1.84915600
H	2.52496700	0.22478500	-2.40894100
C	4.42501300	-0.78960300	-2.45159100
H	4.31676400	-1.10000700	-3.48572000
C	5.54643000	-1.16896000	-1.72022600
H	6.31826300	-1.77243100	-2.18630600
C	5.67237200	-0.78900800	-0.38407500
H	6.53862300	-1.09631200	0.19201900
C	4.67240400	-0.03669800	0.22117300
H	4.75601400	0.23125100	1.27214500
C	2.79076500	2.88787700	0.72925900
C	4.09273400	3.31562100	0.46300100
H	4.80074900	2.64366100	-0.01199800
C	4.48129100	4.61041200	0.79966500
H	5.49400900	4.93822100	0.58845600
C	3.57513400	5.48211100	1.39496700
H	3.88060100	6.49084200	1.65303400
C	2.27230300	5.06114000	1.65629300
H	1.56038800	5.74067000	2.11308400
C	1.88107900	3.76785700	1.33073700
H	0.85814400	3.44708200	1.51862700
B	-0.27129600	-0.45236800	0.67732600
C	-1.61472600	1.26341200	-0.99251000
F	-1.24725600	3.37135800	0.03464000
C	-2.02847400	2.56705200	-0.69752000
F	-3.59639800	4.32711200	-0.77712600
C	-3.25266700	3.08078700	-1.09265300
F	-5.30609900	2.75237300	-2.19656000
C	-4.12914200	2.27773100	-1.80866200
F	-4.59756700	0.19598800	-2.79469400
C	-3.76273700	0.97642800	-2.11451500
F	-2.25994700	-0.77837100	-2.01668200
C	-2.52835800	0.48548100	-1.70382000
C	0.92464700	-1.51842000	0.30611600
F	0.02561200	-1.73408000	-1.86591400
C	0.97881500	-2.04914700	-0.98400300
F	1.99452200	-3.30556300	-2.70784500
C	1.98490900	-2.88198900	-1.44431600

F	4.03785100	-3.97029900	-1.03721900
C	3.02927800	-3.22410900	-0.59728800
F	4.05374400	-2.98313000	1.50794500
C	3.02791600	-2.73183900	0.69434700
F	2.14826300	-1.39489000	2.35086000
C	1.98820000	-1.90920000	1.11368600
C	-1.73695600	-1.14562100	0.85409900
F	-1.04529800	-3.35547600	0.26633900
C	-2.00767300	-2.49180400	0.61115200
F	-3.45507900	-4.34938500	0.44869600
C	-3.27776800	-3.05461500	0.70367700
F	-5.57518800	-2.78990300	1.15969700
C	-4.35709600	-2.26833300	1.06574000
F	-5.15965000	-0.14505700	1.70424400
C	-4.14335700	-0.92452600	1.33611600
F	-2.74984800	0.89946400	1.54397800
C	-2.86242600	-0.40750700	1.22801200
H	1.07868100	0.44624300	1.51363200
C	-0.00830400	0.32280400	2.39094000
H	-0.73833500	1.12715000	2.29491700
H	0.85853000	0.81484800	2.86945400
C	-1.54135200	-0.61268500	4.12129500
H	-2.23214300	0.21653200	3.99042800
C	-0.42750600	-0.70974600	3.40201800
H	0.24433400	-1.55001800	3.54818700
H	-1.80227200	-1.36625400	4.85667600

P23_P

C	-1.60196700	-2.20515200	-1.85131800
H	-0.77869400	-2.27656600	-2.56216000
H	-2.49060900	-1.88852900	-2.40603500
H	-1.79400700	-3.20864100	-1.45390300
C	-1.26882200	-1.22376900	-0.74796200
C	-0.07589900	-0.65108800	-0.46770000
P	-2.70852300	-0.76171200	0.20839200
C	-4.14371000	-0.45327300	-0.84419300
C	-5.08154400	-1.46114400	-1.09185700
H	-4.98316300	-2.43020400	-0.61122500
C	-6.14379500	-1.21462000	-1.95516200
H	-6.87365900	-1.99396300	-2.14602100
C	-6.26825400	0.02884000	-2.57055400
H	-7.09847600	0.21795400	-3.24318200
C	-5.33049500	1.02983700	-2.32863800
H	-5.42266200	1.99743900	-2.80989200
C	-4.26479500	0.79398000	-1.46747900
H	-3.52969600	1.57471300	-1.30354000
C	-3.12250700	-2.07331200	1.37922600
C	-4.32158200	-2.04313900	2.09874100
H	-5.03428400	-1.23653900	1.94861300
C	-4.59945900	-3.05880200	3.00657400
H	-5.52387300	-3.03735900	3.57341100
C	-3.68943100	-4.09901800	3.18912000
H	-3.90971900	-4.88867400	3.90002800
C	-2.49805500	-4.12809800	2.46960000
H	-1.78832000	-4.93456500	2.61731600
C	-2.20700400	-3.11455300	1.56230300
H	-1.26705500	-3.11804800	1.01411600
B	0.30199800	0.46078900	0.73667600
C	1.05661500	-1.09518100	-1.33005300
F	0.78146500	-3.36052700	-0.70783300
C	1.47002700	-2.42439900	-1.37143400
F	2.96022900	-4.10768800	-2.09260700
C	2.59789900	-2.82786000	-2.07179900
F	4.42227600	-2.25565700	-3.44244800
C	3.34648300	-1.88265500	-2.75973500
F	3.68342700	0.35746500	-3.39514800
C	2.96489200	-0.54663300	-2.73686900
F	1.51346100	1.12158900	-1.99165700
C	1.83464000	-0.17147600	-2.02547400
C	-0.33063300	1.93915000	0.36079000
F	-1.22358100	1.43111100	-1.80146000
C	-1.03553600	2.29772400	-0.78310300
F	-2.37844700	3.76152800	-2.06499500
C	-1.64672300	3.53590400	-0.96648100
F	-2.11069000	5.69972700	-0.15136900
C	-1.52858000	4.51312100	0.00491900
F	-0.65557400	5.15682300	2.09476500
C	-0.79590700	4.22778700	1.15016800
F	0.46769900	2.76706400	2.42163900
C	-0.23098800	2.97038000	1.29668600
C	1.93667400	0.48677100	0.87754700
F	2.32678000	2.77359200	0.34268300
C	2.79163400	1.56731600	0.69888700
F	4.94348700	2.55415800	0.66860300
C	4.17388600	1.48057100	0.85040900
F	6.07442800	0.17023600	1.33143900
C	4.75436000	0.26901700	1.18641400
F	4.48949200	-2.02390800	1.67114600

C	3.94464100	-0.84545200	1.36341900
F	1.84013400	-1.82852100	1.35428600
C	2.57526500	-0.70695800	1.21213900
H	-2.46929600	0.44403300	0.87342800
C	-0.32923000	-0.03882300	2.21778000
H	-0.71292900	-1.06290500	2.14131800
H	-1.18913300	0.59883300	2.47886000
C	0.91831700	-1.09226300	4.12438300
H	0.53856900	-2.07934500	3.86984800
C	0.59011900	-0.02082700	3.40642900
H	1.00445200	0.94579800	3.68462400
H	1.58591300	-1.02604000	4.97739600

P24_TS

P	-1.83978700	0.52324700	0.55282700
B	0.90592300	-0.84616100	0.70741700
C	-0.41999600	1.13988700	-0.42511700
C	0.76961200	0.56012500	-0.11128000
C	-2.90879300	2.01015200	0.82175300
C	-3.82273100	2.47171900	-0.15182800
C	-4.56220900	3.62617900	0.11354000
H	-5.25727900	3.97881400	-0.64457000
C	-4.42258600	4.35270900	1.29097400
C	-3.49139900	3.90309000	2.22278200
H	-3.33895800	4.46377900	3.14187700
C	-2.73894200	2.75106900	2.00854700
C	-3.99621200	1.84076300	-1.50714900
H	-4.58443900	2.49936000	-2.14892800
H	-4.50191700	0.87359400	-1.45043000
H	-3.02518800	1.69440300	-1.98854300
C	-5.25308300	5.57995300	1.55474500
H	-4.68783500	6.32654200	2.11774200
H	-6.13914900	5.32345600	2.14454700
H	-5.59576600	6.03515700	0.62290300
C	-1.75704800	2.33211700	3.06272100
H	-0.76265300	2.14516000	2.64090000
H	-2.10159100	1.41147500	3.54033300
H	-1.65723900	3.10047200	3.83202500
C	-2.95274600	-0.77160700	-0.14250200
C	-2.96878500	-1.25216300	-1.46635600
C	-3.80921400	-2.32431200	-1.78036900
H	-3.79084800	-2.70942600	-2.79858300
C	-4.61666200	-2.94440300	-0.83528200
C	-4.62062700	-2.42277300	0.45688500
H	-5.25481100	-2.88001100	1.21250400
C	-3.81110800	-1.35208700	0.82548300
C	-2.15104600	-0.69151400	-2.60210600
H	-1.80730100	-1.49642300	-3.25656000
H	-1.27515000	-0.14828000	-2.25948500
H	-2.75460200	-0.01304000	-3.21400400
C	-5.39776700	-4.18512900	-1.17154400
H	-6.32972300	-4.23749800	-0.60384400
H	-4.80101700	-5.06820700	-0.92061400
H	-5.63363800	-4.23278100	-2.23689200
C	-3.87552700	-0.87075300	2.25448800
H	-4.14223400	0.18989900	2.31361800
H	-2.91682600	-1.00808400	2.76467100
H	-4.63008100	-1.43546800	2.80514600
C	-0.52611100	2.29850100	-1.36355800
C	-0.97356500	3.57154500	-0.99051200
H	-1.23534500	3.77683600	0.04064300
C	-1.05516900	4.59296100	-1.93276000
H	-1.40469800	5.57285100	-1.62374500
C	-0.68383100	4.36815800	-3.25474700
H	-0.75440900	5.16756500	-3.98528200
C	-0.20311800	3.11619500	-3.62996900
H	0.11202700	2.93388900	-4.65229800
C	-0.12066400	2.09426500	-2.69176400
H	0.27496700	1.12237200	-2.97732000
C	1.98501300	1.34654100	-0.49946100
C	2.14650200	2.62605100	0.04389300
F	1.23052900	3.11994200	0.88314600
C	3.25271200	3.41801200	-0.21234600
F	3.35967900	4.62593400	0.33599900
C	4.26500800	2.93433700	-1.03021900
F	5.33308900	3.68050100	-1.28624500
C	4.15213100	1.66403600	-1.57128200
F	5.11796100	1.18633800	-2.35234300
C	3.03115000	0.88830600	-1.29630000
F	3.00224700	-0.32666200	-1.84050200
C	-0.00997900	-2.04037900	0.07142200
C	0.10125200	-2.32603900	-1.28919600
F	0.87075700	-1.55862800	-2.07207700
C	-0.55543800	-3.36602100	-1.92709400
F	-0.44521800	-3.52750300	-3.24816400
C	-1.41562100	-4.17409400	-1.20238600
F	-2.13558700	-5.11379700	-1.80963100
C	-1.58248500	-3.92942800	0.14800000

F	-2.45842300	-4.65106700	0.84865700
C	-0.88808700	-2.88501600	0.74567700
F	-1.19109500	-2.68392000	2.03829900
C	2.47483200	-1.28406200	0.91123900
C	3.01671300	-2.50666000	0.50596700
F	2.26474000	-3.46550400	-0.04913900
C	4.36059400	-2.84248800	0.64157600
F	4.79855400	-4.02900500	0.22600600
C	5.24356000	-1.94639900	1.21840100
F	6.52710700	-2.25622100	1.35971500
C	4.76037200	-0.72287200	1.65564500
F	5.58159300	0.15645500	2.22617600
C	3.41389900	-0.42902100	1.49926400
F	3.04721700	0.77878900	1.97111800
H	-0.72645600	-0.16993400	1.69703300
C	0.45883400	-0.62956800	2.60211800
H	1.25681200	0.09625800	2.73598700
C	-1.01132600	-1.40989200	4.53931500
H	-1.00225900	-2.45369600	4.24732800
C	-0.41306500	-0.45572100	3.82612900
H	-0.43537400	0.55269700	4.22418500
H	-1.52368700	-1.15770100	5.46235500
H	0.86203400	-1.63840700	2.69602900

P24_P

P	-2.00608500	0.38323400	0.37501800
B	1.22879000	-0.77218800	1.10914100
C	-0.40504500	1.09610700	-0.16190800
C	0.80589400	0.59649800	0.22181700
C	-3.29002300	1.68374100	0.53344900
C	-3.98796100	2.13463000	-0.60733600
C	-4.99355900	3.08787700	-0.44704100
H	-5.51774600	3.43784900	-1.33281800
C	-5.33908600	3.60506200	0.79619400
C	-4.62728900	3.15812000	1.90619600
H	-4.86208800	3.56245200	2.88761800
C	-3.60797900	2.21424000	1.80385700
C	-3.70199700	1.66627200	-2.00930000
H	-4.08357200	2.39631400	-2.72561900
H	-4.18017300	0.70297300	-2.21245200
H	-2.63102100	1.56600700	-2.19540800
C	-6.45385500	4.60486700	0.94461300
H	-7.38289400	4.10098000	1.23041500
H	-6.63788400	5.13589300	0.00844900
H	-6.22568600	5.33881500	1.72107900
C	-2.87086500	1.83706400	3.05904900
H	-1.81040000	2.09300400	2.98819100
H	-2.92474200	0.76678600	3.27703800
H	-3.28592000	2.37053600	3.91605500
C	-2.83216600	-0.99352500	-0.50193600
C	-2.58109700	-1.36568400	-1.83434500
C	-3.34480400	-2.39082600	-2.39843900
H	-3.13274200	-2.68161400	-3.42464000
C	-4.31562400	-3.07618400	-1.67987100
C	-4.52525300	-2.71101000	-0.34986600
H	-5.25698300	-3.25547900	0.24127900
C	-3.80800100	-1.68674300	0.25963700
C	-1.50660100	-0.77113600	-2.70649900
H	-1.00085000	-1.57342200	-3.24937900
H	-0.74582600	-0.24902600	-2.13260000
H	-1.93150700	-0.08083400	-3.44272300
C	-5.06013000	-4.23245900	-2.28725000
H	-6.06734500	-4.31938200	-1.87359500
H	-4.52574000	-5.16250600	-2.06964000
H	-5.13461100	-4.13260100	-3.37214600
C	-4.07240600	-1.41295600	1.72128300
H	-4.38841000	-0.37940400	1.89471400
H	-3.18734100	-1.61442200	2.33694800
H	-4.86738000	-2.06767000	2.08030500
C	-0.53126500	2.33586700	-1.00180100
C	-1.06424400	3.52013200	-0.48380700
H	-1.37775100	3.55906000	0.55438200
C	-1.17596600	4.65549000	-1.28187000
H	-1.59355100	5.56504400	-0.86265600
C	-0.74301800	4.62813600	-2.60362400
H	-0.82873900	5.51435500	-3.22407000
C	-0.17558400	3.46511700	-3.12029900
H	0.19006400	3.44247200	-4.14168700
C	-0.06826900	2.32989200	-2.32567800
H	0.40135400	1.43348400	-2.72164000
C	1.92244400	1.51641700	-0.17245500
C	2.09542800	2.73660700	0.47446700
F	1.23477100	3.11493600	1.42695600
C	3.14592600	3.58859600	0.17492600
F	3.28678900	4.74452900	0.81961200
C	4.05749500	3.22831200	-0.80892700
F	5.06650200	4.03843800	-1.11115600
C	3.89759200	2.02998500	-1.48959100
F	4.75164300	1.69297200	-2.45273400

C	2.83607000	1.19513300	-1.16726700
F	2.68306000	0.07398300	-1.87460800
C	0.17563300	-1.97160000	0.67648800
C	0.17361000	-2.44125500	-0.63796900
F	0.99674000	-1.87826100	-1.53522400
C	-0.60588700	-3.48819600	-1.09761000
F	-0.57957400	-3.85361300	-2.38289900
C	-1.49140100	-4.10676300	-0.22741000
F	-2.30616800	-5.06753800	-0.66311100
C	-1.55745800	-3.66784000	1.08161000
F	-2.47018600	-4.18303600	1.91060000
C	-0.73124800	-2.62854000	1.50166700
F	-0.92102100	-2.24619500	2.77927600
C	2.81250500	-1.24961100	0.78781200
C	3.22913000	-2.49816300	0.31148400
F	2.36354000	-3.49090200	0.04573900
C	4.55450200	-2.86323200	0.09309800
F	4.84446100	-4.07691300	-0.37769800
C	5.57579400	-1.97569400	0.37690100
F	6.84767200	-2.30960700	0.17587200
C	5.23800400	-0.73900900	0.89677300
F	6.19173600	0.14019200	1.20779800
C	3.90122800	-0.42490000	1.10567600
F	3.72321500	0.78933500	1.66714900
H	-1.77232500	-0.08840600	1.67234400
C	1.24739700	-0.46349400	2.73887900
H	2.19589300	0.03980400	2.94675300
C	-0.52559400	0.08137700	4.44255300
H	-0.45123700	-0.90235700	4.89757400
C	0.21028400	0.40056400	3.37700200
H	0.13611400	1.40937200	2.96211700
H	-1.19829600	0.79176000	4.91400000
H	1.29646100	-1.42597200	3.26068700

P25_TS

C	-2.66770100	-3.49057500	2.68281400
C	-1.53087500	-3.62228200	3.42597800
C	-0.33030900	-2.95798500	3.06333500
C	-0.26525500	-2.08562500	1.92206500
C	-1.54138700	-1.85293700	1.26592300
C	-2.66098900	-2.58858700	1.60328100
H	0.71361100	-3.87282000	4.71700500
H	-3.57054200	-4.03851800	2.92937600
H	-1.50856700	-4.27200000	4.29656700
C	0.81742000	-3.22698000	3.84977700
C	1.03838600	-1.56566900	1.52188900
H	-3.57831700	-2.45203100	1.04238000
C	2.11133000	-1.92538700	2.31870500
C	2.02271600	-2.70733800	3.48781900
H	3.10677900	-1.63173600	2.02211100
H	2.91960400	-2.91867100	4.06100000
P	-1.73302100	-0.59541000	-0.05520400
B	1.40125800	-0.84292500	0.08762700
C	2.99978600	-0.71510300	-0.35953300
C	3.88681900	-0.02979100	0.47981700
F	3.46367400	0.45063200	1.66102000
C	5.21669000	0.23728300	0.18509200
F	5.99075100	0.86636800	1.06874800
C	5.72394100	-0.12650100	-1.05105200
F	6.99153300	0.12030800	-1.36812600
C	4.87222900	-0.72861000	-1.95995000
C	3.54594300	-0.97726800	-1.61911500
F	2.81385100	-1.45994400	-2.63324800
F	5.31643700	-1.04097200	-3.17724400
C	0.99314900	0.74071400	-0.09765800
C	0.85175500	1.30674500	-1.36126000
F	0.80906800	0.50095300	-2.44239700
C	0.69178000	2.66342100	-1.59086500
F	0.47570800	3.12840100	-2.82361900
C	0.68582100	3.53210300	-0.50879300
F	0.46257700	4.83267200	-0.68861200
C	0.84003700	3.02309300	0.76922200
C	0.98400000	1.65255800	0.95459900
F	1.06530500	1.24373700	2.22563900
F	0.78753000	3.84812900	1.81633700
C	-3.31423400	-0.96812400	-0.93892900
C	-4.57162600	-0.63727600	-0.37984600
C	-4.77416800	-0.02002600	0.98546400
C	-5.72646600	-0.85840000	-1.13242700
C	-5.68992500	-1.38069600	-2.42060700
C	-6.95556300	-1.63140000	-3.19618700
C	-4.44420900	-1.68116100	-2.96161300
C	-3.25936700	-1.48527300	-2.25264500
C	-1.97236700	-1.83071900	-2.95962000
C	-2.01713700	1.09848900	0.61552300
C	-1.96733400	1.47141000	1.98036500
C	-1.74981600	0.52380800	3.13745000
C	-2.11300400	2.82025300	2.30304200

C	-2.31766100	3.81052900	1.34459800
C	-2.34292900	5.26552200	1.72395800
C	-2.41639800	3.41407600	0.01766000
C	-2.25649000	2.08224400	-0.37059000
C	-2.34799400	1.79203800	-1.85287200
H	-5.76965800	-0.27437500	1.35676500
H	-4.04415300	-0.35767400	1.72080000
H	-4.69978700	1.07103300	0.93313300
H	-6.68771300	-0.60392600	-0.69130600
H	-7.75360400	-0.95507600	-2.88174300
H	-6.79426200	-1.50051400	-4.26875100
H	-7.30728800	-2.65613700	-3.03767800
H	-4.38596500	-2.07436900	-3.97375000
H	-2.15391200	-1.94711200	-4.02970700
H	-1.56140800	-2.77692700	-2.59493900
H	-1.20391500	-1.06424500	-2.83040100
H	-1.86486800	1.07067900	4.07505000
H	-0.74421400	0.10110000	3.12188200
H	-2.45676900	-0.30981100	3.14751900
H	-2.04423700	3.11017600	3.34869700
H	-2.95825400	5.84906900	1.03524100
H	-1.32417600	5.66563800	1.68575400
H	-2.72174300	5.40914400	2.73849400
H	-2.57311400	4.16547300	-0.75346100
H	-2.09955400	2.69182600	-2.42008700
H	-1.65581600	1.00913400	-2.17636300
H	-3.35673200	1.47347100	-2.13621400
H	-0.11084700	-1.10742700	-0.65581700
C	0.71539500	-2.17415200	-1.07562800
H	0.77030800	-1.92427700	-2.13131600
C	2.28413400	-4.04277600	-1.67692400
H	2.23878100	-3.79885000	-2.73305100
C	1.65709000	-3.32119900	-0.75369200
H	1.72845900	-3.60584000	0.29150000
H	2.87856800	-4.90411800	-1.39002600
H	-0.25688500	-2.65172500	-0.88664200

P25_P

C	-2.56778700	-3.40742800	2.74293300
C	-1.40652300	-3.53500400	3.44621300
C	-0.20242700	-2.91758000	3.01810400
C	-0.13685000	-2.07923200	1.84483900
C	-1.44023200	-1.85889100	1.21994900
C	-2.57099900	-2.54943800	1.63093700
H	0.85026100	-3.83530800	4.66205100
H	-3.47454200	-3.92361800	3.03650200
H	-1.36734600	-4.15204300	4.33986500
C	0.95401600	-3.21404300	3.77731200
C	1.16529800	-1.61176100	1.39786500
H	-3.50890100	-2.41475200	1.10503300
C	2.24582100	-2.00389000	2.17588000
C	2.16846400	-2.75785200	3.35993700
H	3.24096700	-1.74921700	1.83951800
H	3.07454800	-2.99629000	3.90776800
P	-1.92029600	-0.58066800	0.02301000
B	1.51241500	-0.94951600	-0.08267900
C	3.14022800	-0.66009600	-0.37562200
C	3.96063100	0.05245600	0.50463000
F	3.47486000	0.48374500	1.68228600
C	5.28909200	0.38064400	0.27086400
F	6.00000500	1.03973400	1.18915500
C	5.86960100	0.03818800	-0.93914100
F	7.13963700	0.34740200	-1.19584300
C	5.09297100	-0.61079700	-1.88213800
C	3.76480100	-0.92035500	-1.59916100
F	3.10698700	-1.46069700	-2.63497000
F	5.61446600	-0.91422700	-3.07271000
C	0.97928200	0.62768800	-0.24605200
C	0.74622300	1.21968300	-1.48559100
F	0.60951600	0.44615400	-2.58425200
C	0.55509800	2.57935300	-1.68933400
F	0.24791300	3.05404100	-2.90090200
C	0.60294100	3.43667000	-0.60194900
F	0.34110000	4.73677400	-0.74876300
C	0.81923200	2.90879200	0.65936800
C	0.97750000	1.53685000	0.81481800
F	1.06521800	1.11952700	2.08610900
F	0.79353500	3.72047400	1.72107000
C	-3.49323100	-0.99114800	-0.80452000
C	-4.72986400	-0.58499100	-0.25782400
C	-4.89165900	0.06573400	1.09676500
C	-5.88719400	-0.78527500	-1.01005900
C	-5.86055900	-1.36635700	-2.27427300
C	-7.12755800	-1.58445300	-3.05660200
C	-4.62958300	-1.77434400	-2.78279300
C	-3.44065100	-1.60650400	-2.07616000
C	-2.16365200	-2.09955800	-2.70977800
C	-2.08159900	1.12003800	0.63949900

C	-1.96828900	1.48551000	2.00014800
C	-1.72805400	0.52540200	3.14084400
C	-2.06538200	2.83827800	2.31834600
C	-2.26901500	3.82568600	1.35603200
C	-2.21140400	5.28320700	1.71713700
C	-2.43797700	3.42815500	0.03459400
C	-2.34265600	2.09201400	-0.35416100
C	-2.52641900	1.79043800	-1.82632200
H	-5.90349300	-0.11180000	1.46700300
H	-4.19394000	-0.32531800	1.84105100
H	-4.73561800	1.14771300	1.03970900
H	-6.83984000	-0.47251400	-0.58928000
H	-6.95926700	-1.44092100	-4.12640100
H	-7.49393600	-2.60624000	-2.91448900
H	-7.91530300	-0.90007400	-2.73501700
H	-4.58859400	-2.24142200	-3.76359100
H	-2.38455800	-2.58295300	-3.66237800
H	-1.65433300	-2.83209000	-2.07734600
H	-1.44829800	-1.29382300	-2.91115700
H	-1.79302300	1.06839700	4.08496800
H	-0.73308900	0.08206200	3.07810100
H	-2.45498100	-0.29074500	3.17428300
H	-1.94360700	3.13377200	3.35702100
H	-2.86677000	5.88191900	1.08064900
H	-1.18567400	5.63864500	1.57294000
H	-2.48342500	5.45101300	2.76127900
H	-2.59687400	4.18253400	-0.73231900
H	-2.34356900	2.69713800	-2.40614000
H	-1.83279500	1.03684000	-2.21094400
H	-3.54353400	1.44305800	-2.03730700
H	-1.00715000	-0.52125800	-1.02148300
C	0.95100400	-2.10867300	-1.13244500
H	0.94086900	-1.81678000	-2.18379900
C	2.25102400	-4.11915500	-1.95469500
H	2.25590300	-3.76605400	-2.98171000
C	1.68115800	-3.41512000	-0.97891200
H	1.72073200	-3.81677300	0.03481300
H	2.74634600	-5.06440100	-1.75500000
H	-0.08285900	-2.34448600	-0.85345000

Methylbenzene

N4_TS

C	1.23779700	1.26976500	1.24431200
C	0.66103000	1.43219800	-0.01633500
C	0.63496900	2.73125700	-0.53881400
C	1.15998000	3.80549400	0.17359800
C	1.73827700	3.60446300	1.42642600
C	1.78204700	2.32527600	1.97023600
H	0.19934200	2.89443000	-1.52033100
H	1.12730900	4.80413300	-0.25093000
H	2.15459400	4.44178200	1.97725600
H	2.23121100	2.15597600	2.94519000
H	0.91917800	-0.69901200	0.40425800
B	0.09499700	0.16922700	-0.83608100
N	1.22217400	-0.09372500	1.73519500
C	-1.45743900	-0.19046300	-0.53767500
C	-2.41155500	0.65536100	-1.10265500
C	-1.98276800	-1.23334100	0.21436800
C	-3.77997100	0.48563400	-0.95025600
C	-3.34534000	-1.44274400	0.39962800
C	-4.25179100	-0.57599300	-0.18958900
C	2.51360300	-0.56496600	2.25235000
H	2.47207200	-1.65207500	2.36778400
H	3.30126500	-0.31341300	1.54060200
H	2.74067100	-0.11971800	3.22982100
C	0.14542000	-0.31145600	2.71298500
H	0.05503300	-1.37927700	2.92342000
H	0.35736100	0.23005000	3.64416300
H	-0.79473600	0.05352300	2.29649100
F	-2.01277500	1.69724600	-1.84506200
F	-4.64152700	1.32461700	-1.52235700
F	-5.55942700	-0.75614300	-0.02601700
F	-3.78061900	-2.46402800	1.13765500
F	-1.16846800	-2.11694500	0.82867500
H	0.27316900	0.32059000	-2.01391100
C	5.37054200	-1.11941300	-1.44112900
C	4.52558300	-0.06590700	-1.77657400
C	3.15591500	-0.15287100	-1.53776600
C	2.60667600	-1.30074900	-0.95759000
C	3.46779700	-2.35345200	-0.62755300
C	4.83524800	-2.26897600	-0.86609200
H	6.43751500	-1.04505300	-1.62494100
H	4.93285700	0.83591200	-2.22276100
H	2.51521600	0.68338000	-1.79432200
H	3.05524900	-3.25227000	-0.17479400
H	5.48146900	-3.09977100	-0.60056500
C	1.13510500	-1.43176500	-0.64736800
H	0.55113800	-1.72742300	-1.52059500

H	0.97847600	-2.26865900	0.04511100

N4_P4

C	-1.54821600	-1.77256600	-0.19617800
C	-0.62473700	-1.03120000	-0.92673800
C	-0.08460300	-1.73209600	-2.02493100
C	-0.42588900	-3.03867200	-2.33768700
C	-1.35407700	-3.72959600	-1.55615700
C	-1.92487300	-3.08775800	-0.46958800
H	0.63773000	-1.20673400	-2.64462500
H	0.02946300	-3.52748500	-3.19338300
H	-1.62800000	-4.75242100	-1.79079300
H	-2.64432700	-3.61096600	0.15475700
H	-1.84956100	-0.14884200	0.93399500
B	-0.04806500	0.49685400	-0.70137200
N	-2.15227600	-1.12748400	1.00015100
C	1.53463900	0.41853500	-0.28204700
C	2.43324100	1.38965100	-0.71901900
C	2.09434700	-0.54663600	0.54523600
C	3.78323100	1.39623400	-0.38917400
C	3.43650500	-0.58806600	0.90204300
C	4.29087200	0.39466200	0.42622900
C	-3.64243200	-1.10896100	0.98319800
H	-3.99109700	-0.53619800	1.84235400
H	-3.96273500	-0.62189100	0.06169300
H	-4.00973300	-2.13319300	1.03200000
C	-1.60468600	-1.66978500	2.27593800
H	-2.00104800	-1.07944700	3.10420400
H	-1.91413900	-2.71107200	2.37076200
H	-0.51908200	-1.60235100	2.22934100
F	2.00792900	2.39802000	-1.49285400
F	4.59548900	2.35392800	-0.84165600
F	5.58286500	0.38149400	0.75469100
F	3.90551000	-1.55241200	1.69911800
F	1.32178200	-1.52868500	1.06717100
H	-0.09444600	0.99451900	-1.80793900
C	-5.03674900	2.21762900	-0.06457400
C	-4.26199500	1.88993700	-1.17661500
C	-2.89072000	1.68116300	-1.04665700
C	-2.25474500	1.81064300	0.19714900
C	-3.05042300	2.14414100	1.30313000
C	-4.42367100	2.34701800	1.17874600
H	-6.10539500	2.37616700	-0.16849700
H	-4.72923100	1.78997100	-2.15186700
H	-2.29304200	1.41227900	-1.91219200
H	-2.57562100	2.25669200	2.27653000
H	-5.01266000	2.61184300	2.05206300
C	-0.77023200	1.54954700	0.35259100
H	-0.24017300	2.49475400	0.17489500
H	-0.56153600	1.32845700	1.41491500

N5_TS

B	0.50028100	-0.29694100	0.23225300
C	-0.19488200	-1.07404600	1.47037900
C	0.66330700	-1.41849000	2.53093700
H	1.70834800	-1.13441700	2.47691200
C	0.22433700	-2.07665700	3.66858500
H	0.92497400	-2.31149100	4.46342600
C	-1.11582800	-2.43202500	3.78110200
H	-1.48076900	-2.95324000	4.66019100
C	-1.99116100	-2.11200400	2.75472000
H	-3.03057100	-2.39382300	2.84471700
C	-1.54333400	-1.43434300	1.61210600
N	-2.45757100	-1.10804000	0.52118600
C	-3.02005500	-2.33577600	-0.19122900
C	-3.71790000	-1.83118900	-1.46318800
H	-2.97274500	-1.37751600	-2.12501900
H	-4.12793500	-2.69746900	-1.99350400
C	-4.80565600	-0.80369400	-1.17137000
H	-5.61372300	-1.26192900	-0.58780400
H	-5.25490700	-0.46196900	-2.10908600
C	-4.20535500	0.38909600	-0.43345000
H	-4.98893500	1.10219300	-0.15390400
H	-3.52665300	0.91971400	-1.10522000
C	-3.45628600	0.00516900	0.85395600
C	-4.49673400	-0.29142100	1.95183300
H	-5.29353200	-0.96617800	1.64214300
H	-4.97204700	0.66372100	2.19541600
H	-4.04188700	-0.65918200	2.87127000
C	-2.64460700	1.19775800	1.35969400
H	-1.78069000	1.38826700	0.72535600
H	-2.28979900	1.03470400	2.37890300
H	-3.27599600	2.09107300	1.34558300
C	-4.00256200	-3.20731500	0.61624300
H	-3.48952200	-3.76657100	1.39961700
H	-4.42417800	-3.94355100	-0.07505000
H	-4.83390300	-2.66603400	1.05899700
C	-1.86713900	-3.26800800	-0.57142200

H	-1.10656500	-2.78402100	-1.17671100
H	-2.27658200	-4.10104200	-1.15040900
H	-1.38272900	-3.68210300	0.31623900
C	0.92404000	1.24948100	0.50970100
C	0.65247900	1.96313300	1.67613100
F	-0.00408400	1.40916100	2.70194400
C	1.03504500	3.28781300	1.86867200
F	0.73746200	3.91503100	3.00514500
C	1.74754600	3.94974100	0.88208400
F	2.12327000	5.21207500	1.05464200
C	2.07053000	3.27498500	-0.28732200
F	2.76188100	3.89264300	-1.24200200
C	1.66545800	1.95874400	-0.43918700
F	2.00281600	1.37594000	-1.60275500
C	1.83805800	-1.13345400	-0.27660700
C	3.07289300	-0.90177300	0.33804000
F	3.19054500	-0.00415100	1.33294800
C	4.24902300	-1.56252700	0.00994000
F	5.38202200	-1.28109300	0.64977200
C	4.24113300	-2.51612600	-0.99558700
F	5.35595400	-3.15782000	-1.32829000
C	3.04823300	-2.78760700	-1.64282400
F	3.00745400	-3.69608700	-2.61542100
C	1.89504300	-2.10363700	-1.27470600
F	0.80175100	-2.43679700	-1.98593600
H	-1.43122600	-0.51597800	-0.36256000
C	-2.63508900	3.19080600	-3.40541300
C	-1.98485200	3.43628200	-2.20108800
C	-1.32740800	2.41207500	-1.52294300
C	-1.30905700	1.10653600	-2.03038200
C	-1.94311600	0.88702400	-3.26192600
C	-2.60437500	1.90631500	-3.93887200
H	-3.15020800	3.99164800	-3.92545600
H	-1.98601700	4.43532100	-1.77628700
H	-0.84355500	2.64875300	-0.58391700
H	-1.91510600	-0.10785800	-3.69989200
H	-3.09017900	1.69485600	-4.88629300
C	-0.64382100	-0.09553600	-1.37615300
H	0.41185100	-0.00089900	-1.65009600
H	-0.90268100	-0.98008400	-1.96028200

N5_P

B	0.65186400	-0.16222500	-0.06561200
C	-0.14849300	-0.84036500	1.25093400
C	0.73209700	-1.11365200	2.32422400
H	1.78385800	-0.88866400	2.17696900
C	0.34681400	-1.61148000	3.55559400
H	1.09007000	-1.78268500	4.32755700
C	-0.99798200	-1.87558500	3.79670500
H	-1.33578000	-2.26098400	4.75262200
C	-1.91156300	-1.63043200	2.78762300
H	-2.95740700	-1.82713300	2.97102600
C	-1.48984300	-1.13291500	1.54673100
N	-2.56949600	-0.89795300	0.53729700
C	-3.24885000	-2.20196200	-0.00809800
C	-4.20753300	-1.79804400	-1.13945300
H	-3.61465600	-1.48318100	-2.00263100
H	-4.74515500	-2.70364800	-1.43800700
C	-5.17163500	-0.67298100	-0.79077000
H	-5.86885100	-0.97767500	-0.00136900
H	-5.77795700	-0.43291000	-1.66904600
C	-4.37004300	0.55347200	-0.37386300
H	-5.02836300	1.39876600	-0.14961700
H	-3.72537300	0.86808200	-1.20328800
C	-3.50727200	0.31436900	0.86992500
C	-4.38802800	0.13450200	2.10750700
H	-5.09750200	-0.68894200	2.04828300
H	-4.97274200	1.05519300	2.19242600
H	-3.79985700	0.04677200	3.02012700
C	-2.60091000	1.51377500	1.12328300
H	-1.83484800	1.61491800	0.35248700
H	-2.11074300	1.44678700	2.09324800
H	-3.21754300	2.41684400	1.09980000
C	-3.98179700	-3.03510600	1.04577900
H	-3.29300200	-3.48206200	1.76166400
H	-4.46075500	-3.85550900	0.50472900
H	-4.76675600	-2.50401200	1.58199100
C	-2.13306500	-3.06304100	-0.58777200
H	-1.59277800	-2.53760600	-1.37708500
H	-2.58464200	-3.95738500	-1.02591300
H	-1.41433200	-3.36900800	0.17316900
C	1.04181900	1.41747000	0.28952300
C	0.68157700	2.14406400	1.42245100
F	0.02524300	1.58080900	2.45492900
C	0.95399500	3.49949900	1.60694600
F	0.54318400	4.12093400	2.71482100
C	1.65957800	4.19553300	0.64466000
F	1.93410700	5.48860400	0.80062600

C	2.09285200	3.51381200	-0.48526100
F	2.80695500	4.15499000	-1.41086900
C	1.78819500	2.17005800	-0.62605400
F	2.29163100	1.58476300	-1.72508700
C	2.04459200	-1.08246800	-0.21778300
C	3.36469400	-0.66101900	-0.06214500
F	3.66440400	0.58067000	0.35102800
C	4.47283800	-1.47892800	-0.26803800
F	5.70583000	-1.00028700	-0.09682500
C	4.29634900	-2.80202300	-0.63324800
F	5.34448800	-3.59945300	-0.82871100
C	3.00516300	-3.28641400	-0.77427100
F	2.80383700	-4.56411200	-1.10459400
C	1.93494800	-2.43078200	-0.55892100
F	0.71693900	-2.99870300	-0.68584800
H	-2.05823600	-0.54949800	-0.28960900
C	-3.14281600	1.98740900	-3.58574000
C	-2.29817000	2.63492200	-2.68665600
C	-1.29495300	1.93316200	-2.02211400
C	-1.10514700	0.55617000	-2.22458300
C	-1.95534800	-0.06646500	-3.15184100
C	-2.96173700	0.62771200	-3.82092700
H	-3.92021000	2.53782100	-4.10558700
H	-2.41265600	3.69940000	-2.50527700
H	-0.64234100	2.47004600	-1.34194800
H	-1.79815400	-1.12035300	-3.37470400
H	-3.59347300	0.10614900	-4.53434200
C	0.03203100	-0.24478400	-1.62084300
H	0.90147700	0.01439700	-2.23404200
H	-0.14783800	-1.29595600	-1.86289900

N11_TS

B	0.22142500	-0.02444500	0.30827400
N	-2.22279400	-1.63742100	0.40147700
C	-1.26834400	-1.90267600	1.49467400
H	-0.65955400	-2.74321300	1.13892600
C	-0.31482400	-0.71517700	1.71288000
H	-0.93825300	0.06823500	2.16935500
C	0.48565800	-1.26832200	2.90507600
H	1.18403600	-2.04481800	2.58103500
H	1.06241000	-0.49881300	3.41802400
C	-0.59680600	-1.89292800	3.81858100
H	-0.21408800	-2.76426300	4.35488200
H	-0.91763300	-1.17028800	4.57371300
C	-1.78900100	-2.25784800	2.89409900
H	-2.66318800	-1.65282100	3.15016800
H	-2.08275800	-3.30787100	2.97417700
C	-2.76613000	-2.88998400	-0.16495600
H	-3.36427900	-3.40178800	0.60665500
H	-1.91879100	-3.53482400	-0.41583900
C	-3.63009900	-2.62296200	-1.39233700
H	-4.01084400	-3.57628100	-1.77130100
H	-3.01037300	-2.18456600	-2.18547900
C	-4.77072700	-1.66583700	-1.05428200
H	-5.36404400	-1.44040000	-1.94484600
H	-5.44296900	-2.14375300	-0.32909200
C	-4.19493800	-0.38517700	-0.45248900
H	-3.61017300	0.13944100	-1.21286900
H	-4.99259300	0.29108600	-0.12970900
C	-3.31672600	-0.70798000	0.75343900
H	-2.87808900	0.20001300	1.17592300
H	-3.94465300	-1.17303100	1.52849500
C	-0.69880300	1.30691600	0.04807800
F	0.20125600	2.10744300	2.07994900
C	-0.63000900	2.28576500	1.04043500
F	-1.27486000	4.33910800	2.02258400
C	-1.38177600	3.45216400	1.03540700
F	-2.99643500	4.79155300	-0.04316100
C	-2.25317300	3.68925100	-0.01889400
F	-3.16693500	2.96786800	-2.06795100
C	-2.33449500	2.76395900	-1.04742400
F	-1.66489400	0.79814700	-2.06513400
C	-1.54460700	1.62079200	-1.00653900
C	1.77657200	0.42213900	0.01134100
F	1.08782000	1.48787200	-2.00516200
C	2.05535400	1.21486600	-1.11148000
F	3.47170900	2.47374000	-2.51909700
C	3.30125600	1.73735600	-1.42344800
F	5.57583400	1.97933900	-0.85398400
C	4.37309400	1.48825400	-0.57982500
F	5.18149400	0.42862500	1.35804200
C	4.16730400	0.70120700	0.53896700
F	2.83449100	-0.58004500	1.89567200
C	2.89858300	0.19682100	0.80664500
H	-1.10552400	-1.15933600	-0.44015000
C	1.85028200	-5.13863900	-1.06037700
C	0.86078400	-4.87976200	-2.00400400
C	0.25134400	-3.62922400	-2.04903000

C	0.60823300	-2.60981300	-1.15765500
C	1.60585300	-2.88683700	-0.21632300
C	2.21890100	-4.13578800	-0.16869400
H	2.32792500	-6.11207300	-1.01874200
H	0.56146500	-5.65009500	-2.70791800
H	-0.51954000	-3.43582300	-2.79256800
H	1.90271000	-2.13057400	0.50043500
H	2.98772800	-4.32256700	0.57457300
C	-0.08953200	-1.27386500	-1.25398400
H	-0.92183100	-1.37784700	-1.96578900
H	0.50064000	-0.53242300	-1.78684400

N11_P

B	-0.15414700	0.08760100	-0.24540700
N	-3.33355400	1.21816900	0.05315400
C	-2.39698800	1.48295400	-1.11962000
H	-1.90255700	2.41404200	-0.83207200
C	-1.37122700	0.36313300	-1.34433500
H	-1.96414600	-0.56122300	-1.35685700
C	-0.93760100	0.57375800	-2.82353300
H	0.09894800	0.90993400	-2.89504600
H	-0.99793100	-0.37321300	-3.36700100
C	-1.90171800	1.62574100	-3.42436900
H	-1.41776800	2.60620200	-3.43039800
H	-2.20750600	1.40056100	-4.44811400
C	-3.09987300	1.66880200	-2.46316900
H	-3.76762900	0.82046100	-2.65957400
H	-3.67920900	2.59333600	-2.54671200
C	-4.24270400	2.38110100	0.30710900
H	-4.94311900	2.41746000	-0.53241800
H	-3.62363200	3.28071300	0.28278400
C	-4.97396300	2.21935600	1.63276700
H	-5.62531400	3.08565800	1.77597400
H	-4.24394200	2.23054100	2.45235200
C	-5.76971500	0.91442700	1.65751700
H	-6.24525500	0.77661000	2.63154400
H	-6.57197100	0.96330400	0.91041600
C	-4.84820300	-0.26281200	1.34242000
H	-4.11208600	-0.39272100	2.14676300
H	-5.40833500	-1.19929500	1.27903500
C	-4.10548300	-0.07001700	0.02491300
H	-3.39278500	-0.87798200	-0.13113600
H	-4.79175000	-0.01231200	-0.82441600
C	0.19697500	-1.50858200	-0.51097100
F	2.02802900	-1.09628300	-1.97038600
C	1.20892300	-1.96327400	-1.35599700
F	2.43963000	-3.66106300	-2.45270800
C	1.44280700	-3.30320800	-1.64251700
F	0.82027200	-5.56538200	-1.36784500
C	0.61815600	-4.27632600	-1.09900400
F	-1.26330200	-4.79422400	0.22640800
C	-0.43249800	-3.88243200	-0.28671800
F	-1.71514200	-2.24112900	0.71884200
C	-0.62020500	-2.52974800	-0.03629800
C	1.18020700	1.05003300	-0.40564700
F	2.57674400	-0.56048500	0.63822300
C	2.41660600	0.66334400	0.12033200
F	4.67817600	1.04300000	0.69841100
C	3.53721500	1.48024300	0.16515300
F	4.52723500	3.57494200	-0.30037000
C	3.46448100	2.77201200	-0.33666900
F	2.16571300	4.45036500	-1.36509600
C	2.26739300	3.21199400	-0.87329100
F	0.04846200	2.88833100	-1.42462600
C	1.17416900	2.35179200	-0.88935500
H	-2.69827500	1.13880700	0.85841600
C	2.26831100	-0.54059200	4.33146400
C	2.17407700	0.75017400	3.81966700
C	1.19917300	1.05801300	2.87526600
C	0.31090600	0.08267100	2.41057800
C	0.40661100	-1.20630700	2.94637400
C	1.37417600	-1.51557100	3.89597800
H	3.02918900	-0.78393100	5.06588000
H	2.86138000	1.52086400	4.15520000
H	1.13583300	2.06729600	2.47301100
H	-0.28986800	-1.97004300	2.61153500
H	1.43311200	-2.52378400	4.29465700
C	-0.67698900	0.38752300	1.31893400
H	-0.90532800	1.46831600	1.37201900
H	-1.59490600	-0.17666400	1.53956100

N12_TS

N	1.52827300	-2.43251100	0.39603100
H	0.63190500	-1.54232700	-0.40100100
B	0.11047800	0.14972800	0.24515100
C	1.76619800	-1.46815500	1.50616000
H	2.60393200	-0.84006900	1.16610200
C	0.53351100	-0.56637100	1.64669400

H	-0.28342300	-1.19915600	2.01557900
C	2.79457900	-2.88666600	-0.21967100
H	3.36379700	-3.46705800	0.52478200
H	3.38058700	-2.00543300	-0.48010400
C	2.54799300	-3.75499200	-1.44859900
H	2.05251800	-3.15624300	-2.22254800
H	3.51414800	-4.06595000	-1.85794700
C	1.68818700	-4.96547200	-1.09064900
H	2.25115800	-5.61480600	-0.40670700
H	1.46065100	-5.55742300	-1.98177400
C	0.40346300	-4.50037800	-0.40928200
H	-0.22366800	-3.95508400	-1.12257000
H	-0.18607900	-5.35339000	-0.05916100
C	0.72072900	-3.61074100	0.78945700
H	-0.19973700	-3.25966500	1.26290000
H	1.27919300	-4.20145500	1.53107100
C	2.17051400	-2.11535100	2.83510100
H	1.35487500	-2.75651800	3.19181500
H	3.05233000	-2.75405900	2.70251100
C	2.43319400	-1.04637000	3.89670900
H	2.71178100	-1.52197100	4.84241900
H	3.28571800	-0.42551100	3.58997400
C	1.20064200	-0.16003400	4.07399000
H	1.39261500	0.61438700	4.82379800
H	0.36573600	-0.76748800	4.44903200
C	0.80305200	0.48196200	2.74496500
H	1.62168000	1.14026500	2.42231900
H	-0.07522000	1.11896900	2.87733400
C	1.28423600	1.16178700	-0.27945800
C	1.31864700	2.47805700	0.18460900
F	0.37722900	2.91817400	1.03247800
C	2.29217900	3.40051700	-0.17219100
F	2.26585300	4.63825700	0.31724300
C	3.30334500	3.02370800	-1.04473900
F	4.24763100	3.88986200	-1.39746600
C	3.31758100	1.72911600	-1.53674300
F	4.28007100	1.34110500	-2.37282300
C	2.32087900	0.84214300	-1.14699400
F	2.41543000	-0.39767900	-1.67459000
C	-1.35014900	0.87710200	0.23089300
C	-1.71889700	1.71832300	-0.82204000
F	-0.87623000	1.91905400	-1.85189900
C	-2.93357100	2.38147200	-0.90951000
F	-3.20901500	3.16307500	-1.95144700
C	-3.86306600	2.22857200	0.10818500
F	-5.03501000	2.85155500	0.05130400
C	-3.55381000	1.40894800	1.18059100
F	-4.43595400	1.24105400	2.16469800
C	-2.32309200	0.76460200	1.22323900
F	-2.14026600	-0.01893100	2.30139700
C	-3.97093900	-3.22149400	-1.46360000
C	-3.26050700	-2.91690300	-2.62216800
C	-2.06136000	-2.21715600	-2.54033700
C	-1.54356900	-1.80487000	-1.30665600
C	-2.27087400	-2.11285300	-0.15462800
C	-3.47209700	-2.81497800	-0.23075400
H	-4.90596500	-3.76894000	-1.52268200
H	-3.63908500	-3.22604500	-3.59138900
H	-1.50965400	-1.98536800	-3.44835300
H	-1.90922900	-1.79144000	0.81622000
H	-4.01858400	-3.04016300	0.67969800
C	-0.21467100	-1.08284300	-1.28847900
H	0.47808000	-1.62060400	-1.95270500
H	-0.28198200	-0.13232500	-1.82118500

N12_P

N	2.37982800	-0.33172200	0.81948100
H	1.94528000	-0.21962900	-0.10554800
B	-0.59063100	-0.30560600	-0.22207300
C	1.22516600	-0.48547800	1.83160100
H	1.03018100	0.54960100	2.13886300
C	-0.08346500	-1.02655100	1.22986100
H	0.07433800	-2.09655500	1.04330100
C	3.14531700	0.92438000	1.14088800
H	3.53897900	0.77735200	2.15214300
H	2.41554800	1.73582800	1.16462500
C	4.26976000	1.20651200	0.15784900
H	3.85078800	1.42387000	-0.83120400
H	4.78312500	2.11305200	0.49203100
C	5.21957500	0.01503400	0.06967700
H	5.71025400	-0.14530600	1.03866400
H	6.00602600	0.20630000	-0.66466100
C	4.41100600	-1.21579200	-0.32643200
H	3.96589100	-1.05509000	-1.31233700
H	5.03299000	-2.11232700	-0.40445700
C	3.30382700	-1.51395400	0.67921300
H	2.69296500	-2.36824300	0.37484100
H	3.73292400	-1.70319200	1.66525400

C	1.71269600	-1.25500100	3.06047000
H	1.87291800	-2.30697800	2.79320100
H	2.66790000	-0.85613700	3.42624700
C	0.66316300	-1.17970600	4.16827300
H	1.00447100	-1.75116500	5.03690500
H	0.55353800	-0.13657200	4.49331300
C	-0.67410500	-1.69553300	3.65052600
H	-1.44108600	-1.60372000	4.42653500
H	-0.58321400	-2.76490100	3.41637100
C	-1.10403200	-0.93678600	2.39403200
H	-1.27183300	0.11626300	2.64949400
H	-2.06475600	-1.32856300	2.06663600
C	0.07405600	1.23182200	-0.32119300
C	-0.34513800	2.25736200	0.53098400
F	-1.31500800	2.02449500	1.42651800
C	0.15707600	3.55146300	0.52158700
F	-0.31007600	4.47163600	1.36289800
C	1.15700700	3.89255300	-0.38207400
F	1.66320400	5.12203000	-0.40131700
C	1.62653900	2.91871700	-1.24542200
F	2.63284300	3.18499100	-2.08322300
C	1.08626900	1.63729300	-1.18297600
F	1.73112600	0.72825000	-1.96128900
C	-2.24831300	-0.19317700	-0.28745600
C	-3.03433900	0.89595300	-0.65591600
F	-2.49391600	2.09276600	-0.94558700
C	-4.42127100	0.84927000	-0.78112500
F	-5.10143800	1.94239200	-1.13104500
C	-5.09617800	-0.33755300	-0.55792900
F	-6.42121300	-0.39946900	-0.67532600
C	-4.36417100	-1.46665400	-0.21895700
F	-4.98590400	-2.62902100	-0.01071000
C	-2.98654600	-1.36548200	-0.11158800
F	-2.34280900	-2.51704100	0.18632700
C	2.73005300	-4.37622900	-1.66646400
C	2.90770700	-3.22914000	-2.43144000
C	1.96081700	-2.20327300	-2.40171300
C	0.80685000	-2.29703100	-1.61438100
C	0.61973600	-3.49134700	-0.89256700
C	1.56722100	-4.50779800	-0.90498800
H	3.46613100	-5.17361000	-1.68979900
H	3.78746100	-3.12829600	-3.06165800
H	2.10499600	-1.32379900	-3.02347200
H	-0.30804800	-3.62334800	-0.34359800
H	1.38845300	-5.41673200	-0.33801400
C	-0.26257000	-1.23659600	-1.59809500
H	-0.12449200	-0.57334100	-2.45744700
H	-1.19091300	-1.77761800	-1.80292500

N13_TS

N	-2.31005900	-0.77451800	0.98779700
H	-0.80274800	-0.50175300	1.14989800
B	0.20148600	-0.18934200	-0.22269700
C	-2.36868100	-0.52987500	-0.48998800
H	-2.47149100	0.55808600	-0.57784500
C	-1.00196900	-0.91273300	-1.04402100
H	-0.93029800	-0.56769200	-2.08264000
H	-0.87585000	-1.99628200	-1.07593000
C	-2.50047100	-2.20315600	1.33351100
H	-1.75158800	-2.78794600	0.78818100
H	-3.49208100	-2.52664200	0.98591400
C	-2.39743500	-2.46460300	2.83577300
H	-1.37522900	-2.28914900	3.18563000
H	-2.61250600	-3.52338700	3.01002600
C	-3.36853900	-1.57862000	3.61406100
H	-4.39942200	-1.86552800	3.36772900
H	-3.24255500	-1.72280000	4.69093300
C	-3.15314200	-0.11598200	3.23148500
H	-3.88742200	0.52963500	3.72284500
H	-2.16247500	0.22300400	3.55703800
C	-3.27782200	0.06371400	1.72314500
H	-4.29808000	-0.20539600	1.40481000
H	-3.10027000	1.10516700	1.44153900
C	-3.57510900	-1.14034100	-1.18147600
C	-4.74545100	-0.38712400	-1.31093000
H	-4.76228300	0.63509400	-0.93964400
C	-5.87891700	-0.91798400	-1.92044600
H	-6.77575600	-0.31379500	-2.01330900
C	-5.85596000	-2.21785800	-2.41615300
H	-6.73590500	-2.63563000	-2.89445000
C	-4.69366300	-2.97666900	-2.30366600
H	-4.66417000	-3.98798400	-2.69656700
C	-3.56309000	-2.44094500	-1.69487700
H	-2.66129200	-3.04174900	-1.62446700
C	1.65499300	-0.71561400	-0.69221700
C	2.75165900	0.11089700	-0.93810200
F	2.65804200	1.43927400	-0.81321000
C	4.00957900	-0.36776500	-1.28193000

F	5.01785400	0.47384500	-1.49840100
C	4.21851600	-1.73402000	-1.38848800
F	5.41756100	-2.20899900	-1.71125100
C	3.16470900	-2.59975600	-1.14030500
F	3.35251600	-3.91818900	-1.20708600
C	1.92443800	-2.07838200	-0.80129200
F	0.98544800	-2.99213600	-0.47787900
C	-0.05771200	1.41780400	-0.26435800
C	-0.62290000	2.21594000	0.72115000
F	-0.87809400	1.72132100	1.95471100
C	-0.96018900	3.55351200	0.54424600
F	-1.49537600	4.25217400	1.54524300
C	-0.74647600	4.15061100	-0.68676600
F	-1.06500300	5.42639600	-0.88136800
C	-0.19727300	3.39628200	-1.71576200
F	0.00925800	3.95227400	-2.90697700
C	0.11794900	2.06550800	-1.48824700
F	0.62464500	1.38456200	-2.52512500
C	4.41332300	0.43976400	2.57156200
C	4.09438400	-0.90189400	2.37458300
C	2.78042700	-1.27271500	2.11528800
C	1.76084700	-0.31702600	2.03591900
C	2.08358700	1.01869500	2.28068100
C	3.40083200	1.39238000	2.53682100
H	5.43935000	0.73680400	2.76181100
H	4.86949100	-1.66094700	2.41690800
H	2.53929200	-2.31951700	1.94643900
H	1.30835600	1.77392600	2.27433200
H	3.63293800	2.43881900	2.70739000
C	0.35973600	-0.79187700	1.70458300
H	0.36245900	-1.88195800	1.61927200
H	-0.25022600	-0.53975500	2.58244600

N13_P

N	-2.57807900	0.25041600	-1.05313500
H	-1.73981800	-0.29441500	-1.29540500
B	0.37749500	0.33234200	-0.15175100
C	-2.24801000	0.87035900	0.31917400
H	-2.28851100	-0.00036200	0.98243900
C	-0.82163800	1.40397600	0.30556100
H	-0.63092600	1.72546100	1.33738800
H	-0.76358300	2.31593700	-0.29642300
C	-2.75487800	1.25145700	-2.15408100
H	-1.86936900	1.89016800	-2.14435600
H	-3.63265300	1.84950600	-1.89478500
C	-2.93444200	0.55155800	-3.49592300
H	-2.01403000	0.01246300	-3.75195400
H	-3.07471900	1.32053300	-4.26046300
C	-4.11692500	-0.41644300	-3.46013900
H	-5.04702200	0.14581700	-3.30643300
H	-4.20977500	-0.93810400	-4.41590400
C	-3.93039000	-1.42030900	-2.32371400
H	-4.80187700	-2.07382500	-2.22668800
H	-3.06618600	-2.06323300	-2.51997700
C	-3.72653800	-0.70948500	-0.99244500
H	-4.60490700	-0.12172600	-0.70899300
H	-3.50058800	-1.41670300	-0.18919600
C	-3.34191500	1.82749500	0.74772800
C	-4.38644600	1.35858400	1.54964800
H	-4.39345700	0.31598100	1.86007700
C	-5.40206300	2.21025800	1.97488100
H	-6.19936000	1.82906500	2.60436900
C	-5.38271400	3.55070000	1.60248900
H	-6.16910700	4.22012100	1.93536500
C	-4.34074300	4.03405200	0.81480900
H	-4.31167600	5.08216300	0.53528800
C	-3.32446400	3.18114300	0.39579700
H	-2.50367300	3.57619300	-0.19505400
C	1.82900200	0.85240900	0.42152200
C	2.85032900	0.00239000	0.84193900
F	2.63693800	-1.31788800	0.96039100
C	4.13841700	0.42509300	1.14119600
F	5.06666400	-0.44521700	1.54122200
C	4.46482300	1.76647800	1.01334500
F	5.69603000	2.19309300	1.29321400
C	3.49192000	2.65590400	0.58854700
F	3.78787300	3.95060000	0.44295500
C	2.21678200	2.18295400	0.30325300
F	1.35186600	3.11050600	-0.16386000
C	-0.05844700	-1.12384200	0.52648900
C	-0.70713800	-2.17424000	-0.10772000
F	-0.97028400	-2.12203500	-1.44858900
C	-1.19436400	-3.31819400	0.51779500
F	-1.83500400	-4.25416700	-0.18759200
C	-1.03500100	-3.45323800	1.88494800
F	-1.49658200	-4.52756500	2.52014100
C	-0.40181600	-2.43360700	2.58664600
F	-0.25495900	-2.53975800	3.90567000

C	0.05029700	-1.31037300	1.90867900
F	0.61240200	-0.36036600	2.66632800
C	4.41524000	-1.46406500	-2.80233400
C	4.26043800	-0.08027700	-2.79373600
C	3.01912800	0.48121600	-2.51322900
C	1.90669700	-0.31805300	-2.22430000
C	2.07645600	-1.70612600	-2.24875900
C	3.31494800	-2.27320700	-2.53501100
H	5.38305300	-1.90695900	-3.01447900
H	5.10861900	0.56469100	-3.00339900
H	2.90701900	1.56319400	-2.49748200
H	1.22872800	-2.35018000	-2.03724200
H	3.42189600	-3.35381700	-2.54106300
C	0.60023300	0.31262500	-1.80788600
H	0.61872700	1.36783900	-2.11684000
H	-0.20288300	-0.16242100	-2.38801500

N14_TS

B	-0.35750300	0.16894000	0.10302300
N	2.25489900	-0.87876700	-0.55589100
H	0.80642600	-0.72869900	-0.96555300
C	2.14952800	-0.11930600	0.73144100
H	2.27378700	0.92990600	0.43809500
C	0.71821900	-0.29911500	1.22590200
H	0.56496100	0.32450800	2.11490000
H	0.56382600	-1.32663600	1.56104200
C	2.47335300	-2.33073700	-0.36882200
H	1.68831800	-2.71447900	0.28986900
H	3.44007800	-2.48964700	0.13150500
C	2.46001500	-3.07541700	-1.70126000
H	2.64229000	-4.13623700	-1.50437100
H	1.46595600	-3.00551600	-2.15673000
C	3.51187200	-2.50601400	-2.65152400
H	4.51327800	-2.69029000	-2.23999800
H	3.46595700	-3.00607800	-3.62331900
C	3.30417200	-1.00062800	-2.80731100
H	4.09781800	-0.55437300	-3.41444900
H	2.35833100	-0.80057800	-3.32525300
C	3.28923700	-0.31563200	-1.44581800
H	3.09072200	0.75342200	-1.55709200
H	4.27434800	-0.43360200	-0.96571600
C	3.25290800	-0.42662200	1.72812900
C	4.42468500	0.33560900	1.71040200
H	4.51432600	1.15508100	1.00083900
C	5.46554800	0.07541200	2.59731300
H	6.36370700	0.68405500	2.57088000
C	5.34642200	-0.95628100	3.52331500
H	6.15252600	-1.15975800	4.22073700
C	4.18125800	-1.71828500	3.55849800
H	4.07631300	-2.51727900	4.28546200
C	3.14346000	-1.45377000	2.67018400
H	2.23688400	-2.04972900	2.71856600
C	-1.92450700	-0.00969600	0.50008600
C	-2.93092200	0.57518800	-0.27019800
F	-2.61534200	1.24562300	-1.39337700
C	-4.28398500	0.50301600	0.02763000
F	-5.18697900	1.08118700	-0.76158800
C	-4.68956700	-0.17200000	1.16921300
F	-5.97879500	-0.25771100	1.47851300
C	-3.73302100	-0.76662800	1.97631200
F	-4.10487900	-1.42215000	3.07457300
C	-2.38939700	-0.66781300	1.63634900
F	-1.54203800	-1.28489900	2.47610300
C	-0.04693500	1.71256500	-0.33071500
C	0.56667400	2.18255800	-1.48526400
F	0.96804900	1.33137100	-2.45339800
C	0.83237200	3.52529800	-1.73046800
F	1.42882800	3.89909000	-2.86176400
C	0.47720500	4.47373200	-0.78524800
F	0.72317500	5.76285900	-0.99652100
C	-0.12870500	4.05886900	0.39252700
F	-0.46660600	4.95523600	1.31600600
C	-0.36965500	2.70673100	0.59363800
F	-0.94010500	2.37090400	1.76043600
C	-1.99023100	-4.89268600	-1.46933100
C	-1.84318900	-4.19272300	-2.66460800
C	-1.31691800	-2.90651800	-2.65663500
C	-0.92673600	-2.28544100	-1.46304800
C	-1.08420300	-2.99854200	-0.27260200
C	-1.60994400	-4.28969600	-0.27607400
H	-2.39920900	-5.89775800	-1.46958400
H	-2.13857000	-4.64862300	-3.60442100
H	-1.20304400	-2.36689100	-3.59408300
H	-0.81358600	-2.54618100	0.67449800
H	-1.72425900	-4.82058400	0.66381500
C	-0.34479500	-0.89158900	-1.55087700
H	0.31678900	-0.85862000	-2.42832000
H	-1.09784000	-0.16536700	-1.86286500

N14_P

B	-0.57808600	-0.16370000	-0.23015600
N	2.45693400	-0.02562300	-0.41365700
H	1.68741100	0.20068500	-1.05819100
C	1.82349500	0.06376300	0.98592200
H	1.67201400	1.14238900	1.10242600
C	0.45799100	-0.61531000	0.99119600
H	0.02242900	-0.33524500	1.95598600
H	0.57949800	-1.70234700	1.03324400
C	2.92416200	-1.40125300	-0.78830600
H	2.08648900	-2.08309400	-0.62142300
H	3.73968300	-1.64899300	-0.10330500
C	3.39279700	-1.42909700	-2.23657500
H	3.72060500	-2.44732500	-2.46393800
H	2.54207800	-1.22048500	-2.89623700
C	4.50366900	-0.40767500	-2.47654800
H	5.38752400	-0.67829100	-1.88476800
H	4.80565900	-0.41130500	-3.52693800
C	4.02048000	0.98469000	-2.07081700
H	4.82064200	1.72453900	-2.16196600
H	3.20422200	1.30996000	-2.72529400
C	3.52826200	0.99721800	-0.62948800
H	3.09491600	1.96499400	-0.36032000
H	4.33019900	0.76033300	0.07679500
C	2.81046800	-0.38170000	2.04617400
C	3.58908500	0.57462500	2.70432700
H	3.46956700	1.62572000	2.45064800
C	4.49612300	0.20387900	3.69309100
H	5.08454000	0.96274300	4.19829000
C	4.63475600	-1.13652700	4.03822800
H	5.33666200	-1.43092100	4.81165200
C	3.85681400	-2.09851200	3.39816700
H	3.94967300	-3.14397200	3.67348000
C	2.94732600	-1.72432300	2.41456000
H	2.33254300	-2.48280200	1.93913700
C	-2.16282800	-0.41012600	0.17072100
C	-3.15669700	0.17249800	-0.61550600
F	-2.81598100	0.91813300	-1.68333500
C	-4.51852500	0.04134700	-0.38057600
F	-5.41026400	0.63408900	-1.17608700
C	-4.95016900	-0.71326000	0.69990200
F	-6.25106900	-0.85820400	0.94675400
C	-4.00768900	-1.32362300	1.51153000
F	-4.40368700	-2.06496800	2.54820500
C	-2.65566600	-1.15332600	1.23710800
F	-1.81544500	-1.80453800	2.06860400
C	-0.40039300	1.49827700	-0.35641000
C	0.24935000	2.22032100	-1.35059200
F	0.84895800	1.58444800	-2.39499100
C	0.41358700	3.60214900	-1.35763500
F	1.07154800	4.20173300	-2.35205100
C	-0.10352600	4.34840200	-0.31318200
F	0.03948100	5.67107000	-0.28825100
C	-0.74900200	3.68777000	0.72395100
F	-1.22652800	4.38638500	1.75215400
C	-0.87308100	2.30476700	0.68455800
F	-1.46435500	1.74723900	1.75195900
C	0.15261300	-5.26716500	-1.79828600
C	0.56880400	-4.46165400	-2.85572800
C	0.38565300	-3.08460200	-2.79377000
C	-0.20843200	-2.46173400	-1.68427000
C	-0.63127000	-3.29218600	-0.63907600
C	-0.45208200	-4.67430300	-0.69555000
H	0.29282100	-6.34265000	-1.83750700
H	1.03040500	-4.90650200	-3.73243200
H	0.69550300	-2.46797700	-3.63553500
H	-1.09758000	-2.86412700	0.23953300
H	-0.79049200	-5.28830200	0.13371400
C	-0.34510900	-0.94913100	-1.67900100
H	0.49843000	-0.57700500	-2.27382700
H	-1.20324800	-0.68584000	-2.31222800

N15_TS

N	2.37549200	-0.19338500	-0.77524400
B	-0.39630900	0.00649100	0.00631200
C	2.12019600	0.25410800	0.63247900
H	1.98119800	1.33881400	0.55428000
C	0.78548100	-0.35590300	1.05295100
H	0.51263900	0.03257300	2.03997900
H	0.88573500	-1.43812300	1.17679400
C	2.95933700	-1.55359300	-0.90392300
C	4.48262200	-1.67120100	-0.83588000
H	4.97094300	-1.14599100	-1.66073600
H	4.74638100	-2.72868500	-0.92251800
C	3.11995900	0.48782200	-3.07104000
H	2.11206600	0.41899200	-3.48427700
H	3.64785100	-0.44513000	-3.28205300

C	3.08536400	0.81471100	-1.58333100
C	3.28155500	0.02162600	1.58044900
C	4.31517500	0.96103100	1.64906300
H	4.23775300	1.88148500	1.07479300
C	5.43689500	0.73524500	2.44085500
H	6.23053100	1.47475000	2.47878800
C	5.53534900	-0.43482200	3.18973300
H	6.40896200	-0.61581600	3.80758300
C	4.49743400	-1.36191000	3.15632200
H	4.55695000	-2.26672400	3.75279300
C	3.37756300	-1.13207200	2.36254600
H	2.57397400	-1.86165400	2.35367700
C	-1.88250400	-0.53736300	0.39579200
C	-3.00828200	-0.13866500	-0.32707000
F	-2.86421600	0.61303000	-1.43301600
C	-4.31126800	-0.48285200	0.00388300
F	-5.33419200	-0.06859600	-0.74050900
C	-4.54032300	-1.25607600	1.13206300
F	-5.77766800	-1.59672700	1.47500000
C	-3.46031400	-1.68098200	1.88991600
F	-3.66222700	-2.43224900	2.97048000
C	-2.17376500	-1.31126800	1.51744800
F	-1.19090000	-1.78628300	2.29977500
C	-0.46984800	1.61618700	-0.25993200
C	-0.14293000	2.34221800	-1.39875500
F	0.31794900	1.72785500	-2.50632800
C	-0.24385000	3.72628300	-1.49046000
F	0.09080500	4.35367500	-2.61712700
C	-0.69872200	4.45152900	-0.40155500
F	-0.80621300	5.77422300	-0.47051600
C	-1.03466800	3.77847000	0.76477000
F	-1.47010700	4.45921300	1.82193400
C	-0.91204800	2.39668900	0.81059200
F	-1.24719100	1.80834500	1.96929000
H	4.89452700	-1.29574600	0.10178600
H	3.63901700	1.29039000	-3.60046500
H	2.60745600	-1.96329500	-1.85683900
H	2.49954900	-2.17502100	-0.12986300
H	2.56283100	1.76517100	-1.43946500
H	4.10903800	0.94567300	-1.20429000
H	0.91898300	-0.40570200	-1.15166800
C	0.04764500	-5.21016000	-1.97514900
C	0.26983400	-4.41421200	-3.09562000
C	0.20701300	-3.02971800	-2.98762200
C	-0.08050000	-2.40180000	-1.76853800
C	-0.30047000	-3.21497300	-0.65232000
C	-0.23649700	-4.60341600	-0.75690500
H	0.09676300	-6.29147500	-2.05147400
H	0.49220200	-4.87053900	-4.05514800
H	0.38549000	-2.41575700	-3.86781200
H	-0.51823100	-2.77814000	0.31389100
H	-0.41181800	-5.21072500	0.12570300
C	-0.11959100	-0.88987000	-1.74369000
H	0.39352800	-0.52373000	-2.64088700
H	-1.12063600	-0.49711200	-1.92069900

N15_P

N	-2.64115700	-0.22771700	0.56331800
B	0.63760700	-0.05926100	0.30104600
C	-1.90868200	0.30299900	-0.68712100
H	-1.75795700	1.36354900	-0.46313100
C	-0.55968500	-0.39511400	-0.80263700
H	-0.16962500	-0.10595100	-1.78671000
H	-0.72335200	-1.47370800	-0.87772800
C	-3.17800800	-1.63971800	0.41465700
C	-4.62733700	-1.74702700	-0.03457500
H	-5.32721900	-1.39766500	0.72845200
H	-4.83149800	-2.80777100	-0.19905200
C	-3.95145200	0.39655200	2.58000100
H	-4.68950500	1.10317100	2.96460200
H	-3.04394600	0.49577600	3.18253400
C	-3.65214600	0.72333100	1.12690700
C	-2.82481500	0.20221600	-1.89013000
C	-3.78944200	1.19098400	-2.10804300
H	-3.82582200	2.05768200	-1.45204200
C	-4.69420400	1.08630200	-3.15996800
H	-5.43729900	1.86183200	-3.31412100
C	-4.63353400	-0.00694200	-4.02029500
H	-5.33596500	-0.09192900	-4.84290300
C	-3.65192900	-0.97751400	-3.83564900
H	-3.58334700	-1.81744400	-4.51920600
C	-2.74953500	-0.87164600	-2.78094300
H	-1.97549600	-1.62333100	-2.65830500
C	2.05081900	-0.76316900	-0.21220700
C	3.24877200	-0.40299000	0.40945900
F	3.23946200	0.54964700	1.35861400
C	4.48737400	-0.95001400	0.11648900
F	5.58629200	-0.54018000	0.75193400

C	4.57918500	-1.93767800	-0.85498900
F	5.75532200	-2.48692000	-1.15377400
C	3.42569500	-2.34932900	-1.49713100
F	3.48413500	-3.31133400	-2.42135700
C	2.20731300	-1.75950300	-1.16957300
F	1.14691400	-2.27077100	-1.83625400
C	0.78373400	1.59852900	0.33222500
C	-0.08120800	2.39891300	1.07193500
F	-1.11272200	1.83841300	1.77096600
C	-0.03974100	3.78479200	1.13945000
F	-0.92966400	4.45829200	1.87394100
C	0.92601300	4.46149800	0.41378400
F	0.99542300	5.79058600	0.45077600
C	1.80406200	3.72756800	-0.36936700
F	2.72347700	4.36186300	-1.09572300
C	1.70949400	2.34042800	-0.40389400
F	2.57952800	1.73528500	-1.22341200
H	-4.82530700	-1.22066300	-0.97020900
H	-4.34646000	-0.61434800	2.71043700
H	-3.03607700	-2.12477900	1.38204800
H	-2.51608300	-2.13997800	-0.29088100
H	-3.21828700	1.71947300	1.04906200
H	-4.53711400	0.67856700	0.49174100
H	-1.88501000	-0.28748900	1.25980700
C	-1.77189100	-4.27641100	2.59927400
C	-1.89742800	-3.18921400	3.46002000
C	-1.17963000	-2.01933500	3.21264600
C	-0.31891100	-1.89765200	2.11075400
C	-0.19933400	-3.00989800	1.26313600
C	-0.91423200	-4.18025700	1.50383400
H	-2.32463200	-5.19172300	2.78503400
H	-2.54760200	-3.25039100	4.32804400
H	-1.26548100	-1.18209600	3.90271400
H	0.45541300	-2.95388500	0.39850600
H	-0.79896700	-5.02486100	0.83093800
C	0.43384400	-0.60930100	1.87512500
H	-0.01009000	0.14622700	2.53091200
H	1.44432600	-0.74026200	2.28054800

N16_TS

N	-2.36024100	0.81717500	-0.54891900
H	-0.91572500	0.97458700	-0.85730400
B	0.38812000	-0.00263700	0.19967100
C	-2.16209000	0.01900700	0.70103200
H	-2.05549000	-1.00230200	0.32330800
C	-0.77053900	0.31881300	1.35034500
C	-2.98175800	2.15524600	-0.38604700
H	-2.42948600	2.72171500	0.36158300
H	-4.00950000	2.02318700	-0.01912200
C	-3.02623800	2.91791800	-1.70698800
H	-2.00616500	3.08937600	-2.06729200
H	-3.46395600	3.90327300	-1.51916500
C	-3.83706200	2.14788300	-2.74544900
H	-4.88457700	2.09655200	-2.42044500
H	-3.82404200	2.66088300	-3.71152400
C	-3.27830500	0.73331400	-2.87510000
H	-3.90447000	0.11954300	-3.52999700
H	-2.28125400	0.76448800	-3.32839500
C	-3.19122100	0.05182900	-1.51394600
H	-4.19975400	-0.05591200	-1.08319500
H	-2.76967600	-0.94860700	-1.61713800
C	-3.37935900	-0.05601300	1.61996400
C	-3.98182500	-1.31051700	1.77789900
H	-3.56957000	-2.16330600	1.24331300
C	-5.08395600	-1.49616200	2.60646400
H	-5.52197900	-2.48392400	2.70800400
C	-5.61885000	-0.41675700	3.30074800
H	-6.47795000	-0.55227300	3.94974900
C	-5.04072600	0.84043500	3.15466500
H	-5.44971300	1.69237000	3.68858500
C	-3.93598200	1.01988700	2.32684700
H	-3.52006500	2.01537000	2.23480200
C	2.00798900	0.12010000	0.52400100
C	2.93108100	-0.28048500	-0.45421300
F	2.51742200	-0.68032200	-1.67251500
C	4.30859900	-0.30855800	-0.28955500
F	5.09803600	-0.69698600	-1.28816900
C	4.85686100	0.04972900	0.93050300
F	6.17089100	0.02334800	1.11919800
C	4.00121900	0.43446500	1.94684300
F	4.49255800	0.77919300	3.13534200
C	2.62720100	0.45271800	1.73036300
F	1.92346900	0.84518000	2.80384400
C	0.20809700	-1.50325700	-0.46144400
C	-0.30998600	-1.85056000	-1.70554100
F	-0.72434500	-0.90519900	-2.57247900
C	-0.43946800	-3.15525600	-2.16687900
F	-0.97347000	-3.39615700	-3.36339900

C	-0.00241300	-4.20536000	-1.37677700
F	-0.11950500	-5.46121700	-1.79458200
C	0.56309000	-3.92100800	-0.14360600
F	1.01028200	-4.91092400	0.62530800
C	0.66586900	-2.60042000	0.27555200
F	1.26601800	-2.42122500	1.46139700
C	-0.66822800	-0.66466900	2.53628000
H	-1.37677200	-0.37204200	3.31888200
H	-0.91957400	-1.68869800	2.24125900
H	0.32951600	-0.67289700	2.97055600
C	-0.71170500	1.74352200	1.92783300
H	0.18982400	1.88681100	2.51732600
H	-0.71376700	2.51849500	1.16089900
H	-1.55091400	1.91586700	2.60754500
C	1.62247600	5.42058600	-1.05721900
C	0.97189300	4.94777900	-2.19204900
C	0.53635800	3.62767600	-2.24467000
C	0.72395100	2.74346100	-1.17257900
C	1.39112800	3.23649400	-0.04578900
C	1.83173900	4.55587700	0.01211300
H	1.96591300	6.44870800	-1.00794500
H	0.80637200	5.60295800	-3.04159700
H	0.04934100	3.26552300	-3.14792500
H	1.56365600	2.59315700	0.80664100
H	2.34234400	4.90592400	0.90384700
C	0.25790600	1.31218300	-1.34598100
H	-0.36139200	1.28808700	-2.25390600
H	1.07325700	0.68542400	-1.69850000

N16_P

N	2.44535300	-0.15022500	0.01096900
H	1.79005300	-0.53704800	-0.68747400
B	-0.75767100	-0.44327700	0.07085200
C	1.57831900	0.33324300	1.20355600
H	1.26095200	1.31509800	0.84652300
C	0.23467100	-0.42731500	1.44745500
C	3.47390500	-1.21120400	0.30208000
H	2.95823200	-2.06938200	0.72773200
H	4.14618500	-0.78587800	1.04995400
C	4.23269400	-1.60801700	-0.95558000
H	3.53394400	-2.04053200	-1.67566600
H	4.93599900	-2.39920100	-0.67914800
C	4.94515000	-0.41443500	-1.58257200
H	5.71161900	-0.02777000	-0.89845800
H	5.45182200	-0.71398500	-2.50347600
C	3.91163000	0.67016900	-1.87025300
H	4.37791200	1.58402600	-2.24980500
H	3.20477700	0.32654100	-2.63277800
C	3.14192300	1.03616300	-0.61033700
H	3.80900800	1.42004100	0.16871300
H	2.38022500	1.78852800	-0.81432700
C	2.46391200	0.60670000	2.41027700
C	2.73961700	1.94035700	2.72924400
H	2.33075100	2.72987100	2.10283900
C	3.50706300	2.27690000	3.84115800
H	3.70014400	3.32031200	4.06729000
C	4.01532500	1.27532600	4.66050800
H	4.61139800	1.52981300	5.53056600
C	3.74822600	-0.05831700	4.36017100
H	4.13544500	-0.84696400	4.99698800
C	2.97886900	-0.39069600	3.24988500
H	2.78057200	-1.43529600	3.04153100
C	-2.39572700	-0.68262100	0.38574000
C	-3.30711400	-0.35703100	-0.62791000
F	-2.88063000	0.18706800	-1.78524500
C	-4.67827300	-0.56101900	-0.56668100
F	-5.46325000	-0.20698900	-1.58524500
C	-5.23102200	-1.15020800	0.55914600
F	-6.54298000	-1.36083500	0.64454200
C	-4.38389000	-1.52319000	1.58555500
F	-4.87959500	-2.10796900	2.67776800
C	-3.01371800	-1.29888900	1.47324400
F	-2.31277800	-1.73964000	2.53878000
C	-0.68024000	1.07994300	-0.61436800
C	0.10193400	1.40533600	-1.71083600
F	0.88704900	0.45397500	-2.28016900
C	0.21567000	2.66603500	-2.28369900
F	1.04958900	2.87223500	-3.30696900
C	-0.52476800	3.70940100	-1.75529100
F	-0.43934400	4.93387800	-2.26896700
C	-1.34621600	3.45308400	-0.66615200
F	-2.06834900	4.44207200	-0.14242900
C	-1.40771700	2.17188400	-0.12890700
F	-2.24178500	2.03585400	0.91176700
C	-0.40212200	0.45395500	2.55482400
H	0.13885400	0.34489000	3.50132700
H	-0.37818100	1.51533600	2.28658100
H	-1.44055500	0.19327200	2.73436900

C	0.44502200	-1.85445200	1.99449000
H	-0.42974000	-2.47657900	1.80959400
H	1.28655300	-2.36182700	1.52090100
H	0.61515900	-1.85881200	3.07666200
C	2.62586700	-4.09209200	-2.82118500
C	2.06910800	-3.02210100	-3.51718000
C	1.10615700	-2.21227100	-2.91719900
C	0.65736300	-2.44140600	-1.60435300
C	1.23500900	-3.52977100	-0.92651900
C	2.20368000	-4.33842500	-1.51667400
H	3.36453900	-4.73257300	-3.29198200
H	2.36570600	-2.82715400	-4.54349900
H	0.64912800	-1.41769700	-3.49656900
H	0.86096100	-3.78943600	0.05949600
H	2.60409400	-5.18416900	-0.96533800
C	-0.55452200	-1.72583400	-1.03244900
H	-1.16659200	-1.42702300	-1.89131300
H	-1.12414000	-2.53048700	-0.55190100

N19_TS

B	0.49180300	-0.11431300	0.23401800
C	-0.15334400	-0.33504200	1.70691900
C	0.45956000	-1.21166500	2.61307900
H	1.30631200	-1.80724600	2.28267100
C	0.05484300	-1.34146200	3.93933500
H	0.57088200	-2.03495800	4.59576800
C	-0.98067800	-0.55332100	4.42149300
H	-1.28394300	-0.60677400	5.46214400
C	-1.63916300	0.29577000	3.53939000
H	-2.47537600	0.89409900	3.89471900
C	-1.27317300	0.37390500	2.19605800
C	-2.12186900	1.24508600	1.30379400
H	-1.50142000	2.01504300	0.85047000
H	-2.86792600	1.76489900	1.91065700
N	-2.78265700	0.51100100	0.18347100
C	-3.83572800	-0.50369200	0.67259400
C	-4.55611500	-1.12024600	-0.53910900
H	-3.84662300	-1.76512800	-1.07024500
H	-5.33794200	-1.77704300	-0.14410000
C	-5.13665000	-0.11057600	-1.51121700
H	-5.92133300	0.49094100	-1.03785900
H	-5.60628100	-0.62565400	-2.35469300
C	-4.00219300	0.77034900	-2.00110400
H	-4.35642000	1.52099100	-2.71553300
H	-3.27487400	0.14375900	-2.53528800
C	-3.27429100	1.51562500	-0.87104200
C	-2.09286400	2.24847300	-1.53282400
H	-1.36434000	1.56765900	-1.97017900
H	-1.56744800	2.93285700	-0.86280700
H	-2.50139100	2.85219400	-2.34777200
C	-4.18786600	2.61253500	-0.29468500
H	-3.70962500	3.15180700	0.52599700
H	-5.15345200	2.24709000	0.04833100
H	-4.38078500	3.34183900	-1.08681400
C	-4.87715500	0.09039900	1.63602600
H	-4.41527700	0.58672200	2.49220700
H	-5.46041400	-0.74447700	2.03527900
H	-5.57934700	0.77834500	1.16777900
C	-3.18201600	-1.68791300	1.41247600
H	-2.24960200	-2.00339600	0.95210100
H	-3.87631200	-2.53221600	1.35790800
H	-2.99325900	-1.48047400	2.46407300
C	1.98171900	-0.82649900	0.09282100
C	2.47744500	-1.73974900	-0.83577700
F	1.70433400	-2.24337100	-1.81073300
C	3.79397900	-2.19420600	-0.84798800
F	4.18713400	-3.07840800	-1.76286600
C	4.69719400	-1.71602700	0.08472500
F	5.95644300	-2.14187000	0.08739000
C	4.26835500	-0.77795000	1.01335200
F	5.12288100	-0.29091100	1.91144100
C	2.95011900	-0.35014500	0.98555500
F	2.61674200	0.58001100	1.89133600
C	0.79107600	1.43143300	-0.23906300
C	0.85508800	2.52888700	0.62193800
F	0.54777300	2.41822600	1.91822500
C	1.20682600	3.81215300	0.21210900
F	1.22323200	4.81636400	1.08637100
C	1.55884200	4.04263000	-1.10783500
F	1.89924400	5.26099000	-1.51347600
C	1.56081900	2.97803600	-1.99682400
F	1.91642200	3.16759100	-3.26596800
C	1.20710100	1.71674100	-1.54127900
F	1.26842200	0.73535900	-2.46118700
H	-1.64234200	-0.19476100	-0.36543000
C	-1.64550100	-5.09991500	-1.26505800
C	-2.17478300	-4.22141800	-2.20441000
C	-1.86804900	-2.86428200	-2.14068600

C	-1.04310300	-2.35113100	-1.13543700
C	-0.50646300	-3.25065700	-0.20678900
C	-0.80495900	-4.60716300	-0.26992600
H	-1.87923300	-6.15846600	-1.31112400
H	-2.82082600	-4.59025000	-2.99506900
H	-2.27164400	-2.18815500	-2.89229400
H	0.14050800	-2.88169000	0.58296600
H	-0.37835400	-5.28343300	0.46422800
C	-0.73208200	-0.87791500	-1.09987400
H	0.28957100	-0.77522600	-1.48058500
H	-1.20506700	-0.38647100	-1.96109800

N19_P

B	0.83015200	-0.54640500	-0.56330700
C	0.00423000	-1.58660100	0.42754100
C	0.38045800	-2.93268300	0.23871100
H	1.02850900	-3.16898600	-0.60246000
C	0.06504800	-3.95614300	1.11947100
H	0.41380200	-4.96625700	0.92638000
C	-0.62719500	-3.66225100	2.29250500
H	-0.80448800	-4.42262100	3.04579000
C	-1.10342100	-2.37353900	2.46353200
H	-1.67142900	-2.13397900	3.35977200
C	-0.88077300	-1.37091800	1.50526700
C	-1.64676400	-0.08968800	1.70581900
H	-1.02371800	0.78133800	1.53812500
H	-2.04838100	-0.03716500	2.71731200
N	-2.85184900	0.11453600	0.76537900
C	-3.96291200	-0.98912900	0.85331700
C	-5.17476200	-0.56928500	0.00306900
H	-4.89972700	-0.65742100	-1.05486900
H	-5.94688400	-1.32216500	0.19002700
C	-5.69197800	0.83171600	0.26677800
H	-6.03732600	0.95087500	1.30035400
H	-6.55262700	1.03875300	-0.37534900
C	-4.56405700	1.79375400	-0.04950200
H	-4.87378800	2.83670900	0.06825200
H	-4.27289300	1.66592100	-1.10088000
C	-3.31727800	1.60340300	0.82374600
C	-2.22388000	2.48576100	0.21745400
H	-1.92939000	2.12689100	-0.77202100
H	-1.33290000	2.57510800	0.83903900
H	-2.63920900	3.48964600	0.09477100
C	-3.58064600	2.04749400	2.26337600
H	-2.67229300	2.03862500	2.86773200
H	-4.34597700	1.45954500	2.76893000
H	-3.93129800	3.08222600	2.22530100
C	-4.40370200	-1.24574200	2.29254000
H	-3.57373400	-1.50638300	2.94609000
H	-5.07059100	-2.11187400	2.26657400
H	-4.96200800	-0.41850900	2.73194300
C	-3.42608000	-2.28078200	0.21826700
H	-2.83698300	-2.06931200	-0.67662900
H	-4.29078900	-2.87389900	-0.09457800
H	-2.82304300	-2.88242900	0.89366400
C	2.46534100	-0.80839400	-0.30830900
C	3.39212200	-0.31893900	-1.23125700
F	2.97472300	0.36498000	-2.31266400
C	4.76907000	-0.44768400	-1.10998300
F	5.58575500	0.03811600	-2.04778100
C	5.29432000	-1.07623800	0.00801700
F	6.61176700	-1.21534200	0.15157500
C	4.42607200	-1.54268700	0.98057900
F	4.91561200	-2.12621300	2.07750600
C	3.05165500	-1.38894900	0.81881400
F	2.31857400	-1.83490400	1.84840000
C	0.78528800	1.08474400	-0.21859600
C	1.12801500	1.51173800	1.07006100
F	1.25962200	0.60503000	2.05351300
C	1.32679800	2.83256000	1.44612900
F	1.63645100	3.15118900	2.70361400
C	1.21886700	3.83159300	0.48647200
F	1.40516200	5.10738400	0.81620600
C	0.90787000	3.47318000	-0.81346200
F	0.79156000	4.41462300	-1.75111100
C	0.71482200	2.13054400	-1.13511400
F	0.39365300	1.92209400	-2.42475800
H	-2.47333800	-0.00780900	-0.18969100
C	-3.62106800	-1.67979400	-3.32819800
C	-2.72897500	-2.73779000	-3.16625100
C	-1.42137300	-2.49872900	-2.75275100
C	-0.95251600	-1.20091600	-2.49762800
C	-1.86994100	-0.14746600	-2.65465400
C	-3.18181100	-0.38293200	-3.07024200
H	-4.63635800	-1.86051900	-3.66737700
H	-3.05043100	-3.75490400	-3.37061300
H	-0.73205900	-3.33156600	-2.64267300
H	-1.51496000	0.87249900	-2.54138400

H	-3.85389900	0.45692900	-3.23305600
C	0.48482000	-0.95263800	-2.14006000
H	1.03869900	-1.87437400	-2.35612400
H	0.88179600	-0.20669900	-2.82670100

N20_TS

B	0.91011300	0.20404300	-0.40990800
C	-0.19245900	0.29339500	-1.60929400
C	0.15294900	-0.25540600	-2.85326500
H	1.13166700	-0.71276300	-2.96465500
C	-0.70049800	-0.23791700	-3.95088100
H	-0.38299600	-0.68182100	-4.88951100
C	-1.95403200	0.35115500	-3.83917300
H	-2.63505600	0.37648400	-4.68361400
C	-2.32435700	0.91482500	-2.62497500
H	-3.30693600	1.37172700	-2.52576500
C	-1.47456000	0.89511300	-1.51218200
C	-2.04906900	1.53082500	-0.25152000
H	-2.78379000	2.28280700	-0.55420700
H	-1.28571600	2.05633500	0.32700600
N	-2.73372000	0.53275700	0.65989400
C	-3.20936400	1.12898000	1.98603600
C	-4.08147500	2.35863400	1.71189700
H	-4.57432300	2.62804600	2.65117200
H	-3.43020100	3.20197900	1.46077800
C	-5.12056700	2.13750900	0.60066000
C	-5.06010800	0.78183800	-0.06951200
C	-6.20704300	0.32408400	-0.71465700
C	-6.21302600	-0.90553400	-1.35277000
H	-7.10713500	-1.26096900	-1.85454200
C	-5.06185900	-1.68011300	-1.34427200
H	-5.06714300	-2.63616700	-1.85417100
C	-3.88963600	-1.26254500	-0.70658200
C	-3.90526700	-0.01800500	-0.05415600
C	-2.66772700	-2.16107200	-0.74686500
H	-1.80164500	-1.51200700	-0.86778100
C	-2.63842200	-3.10225200	-1.95426600
H	-1.65029900	-3.56191200	-2.02238700
H	-2.82528600	-2.55620100	-2.88321400
H	-3.37331600	-3.90902200	-1.86376100
C	-2.53210400	-2.96393900	0.55155600
H	-1.68925200	-3.65773300	0.47694300
H	-3.44037600	-3.54904600	0.73008500
H	-2.36775300	-2.32126700	1.42300200
C	-2.04269400	1.55332600	2.87883700
H	-1.39891700	2.29930100	2.41461300
H	-1.43718200	0.70243600	3.19936000
H	-2.46772100	2.01256600	3.77595000
C	-3.98485600	0.04121200	2.74163700
H	-3.38271700	-0.86994700	2.81516600
H	-4.93873000	-0.21184700	2.27717300
H	-4.18119800	0.39587400	3.75689800
C	1.98622900	-1.00908300	-0.48956400
C	3.34494200	-0.90535800	-0.18675600
F	3.90251900	0.26903800	0.13113500
C	4.21808500	-1.98852900	-0.17569400
F	5.50547000	-1.81145200	0.11521400
C	3.74797100	-3.25911400	-0.46336900
F	4.56896500	-4.30458300	-0.45015000
C	2.40260200	-3.42481400	-0.75301700
F	1.91249100	-4.63921300	-1.00295200
C	1.57451700	-2.31391200	-0.76024200
F	0.26817500	-2.57312300	-0.98802200
C	1.60634300	1.68478200	-0.28558500
C	1.42362800	2.69102500	0.64821000
F	0.60207000	2.50698900	1.71136500
C	2.02096200	3.94612600	0.58637800
F	1.77962700	4.85453600	1.53172900
C	2.86172500	4.24281800	-0.47057400
F	3.44779800	5.43299000	-0.55572600
C	3.08433100	3.27544000	-1.44307400
F	3.89625500	3.54015900	-2.46420200
C	2.45866000	2.04515700	-1.33373500
F	2.73663200	1.15101300	-2.29230000
H	-7.09808800	0.94619100	-0.70754800
H	-6.13276600	2.27106500	0.99244200
H	-5.00145200	2.90912100	-0.17013600
H	-1.54460900	-0.17290400	1.04488800
C	1.83775800	-2.18987600	4.39896000
C	1.72684800	-0.80448400	4.32621600
C	1.07253700	-0.20338100	3.25484600
C	0.50743400	-0.97962400	2.23519500
C	0.63218800	-2.37303600	2.31654700
C	1.28915500	-2.97169800	3.38567700
H	2.35142900	-2.65631800	5.23302800
H	2.15881700	-0.18275900	5.10431900
H	1.00815800	0.87849500	3.20105100
H	0.21540500	-2.98846000	1.52367000

H	1.37517700	-4.05322900	3.42448100
C	-0.17393700	-0.36813900	1.07144300
H	-0.23427900	0.71166700	1.03418000
H	-0.49114900	-1.04693600	0.29183900

N20_P

B	0.81554700	0.15159800	-0.21482900
C	-0.32813100	0.07573700	-1.41469300
C	0.00578400	-0.51107000	-2.64890400
H	0.99478200	-0.94165200	-2.76612100
C	-0.85907100	-0.55106900	-3.73621500
H	-0.53731000	-1.01921100	-4.66208800
C	-2.12525800	0.01991900	-3.64502100
H	-2.80654500	0.01010900	-4.48940400
C	-2.49358600	0.61671500	-2.44909600
H	-3.47864300	1.07161900	-2.35718200
C	-1.62352300	0.64077400	-1.34956900
C	-2.16593600	1.29460600	-0.10367200
H	-2.90383800	2.05469500	-0.36567100
H	-1.38777000	1.74019300	0.51164900
N	-2.92639700	0.29761600	0.80152100
C	-3.45553500	0.90555200	2.12594200
C	-4.37289800	2.07136500	1.74936000
H	-4.92488500	2.33735400	2.65508500
H	-3.75650500	2.94111300	1.50496700
C	-5.35347500	1.76795100	0.59458400
C	-5.21696100	0.39360000	-0.02274700
C	-6.29789600	-0.16999100	-0.69763700
C	-6.18900900	-1.43124300	-1.26651700
H	-7.03381200	-1.86627700	-1.79017000
C	-4.99868300	-2.13995600	-1.16443400
H	-4.92266800	-3.11886600	-1.62311000
C	-3.88460600	-1.61871600	-0.50109500
C	-4.03744000	-0.34618200	0.06476100
C	-2.59106300	-2.41464000	-0.42374700
H	-1.75442600	-1.71356300	-0.47238500
C	-2.40230700	-3.35143500	-1.61949500
H	-1.38412800	-3.74275500	-1.60579600
H	-2.54767100	-2.81382900	-2.56041200
H	-3.08995400	-4.20297900	-1.58284900
C	-2.48903600	-3.19913600	0.89088200
H	-1.53956100	-3.74092900	0.91864600
H	-3.30872600	-3.91975500	0.97672200
H	-2.52473100	-2.55021000	1.77391200
C	-2.30984800	1.40166000	3.00424300
H	-1.68343500	2.15017100	2.51901900
H	-1.67708400	0.58057400	3.35162200
H	-2.75943900	1.87003400	3.88430300
C	-4.20727200	-0.19193600	2.88574100
H	-3.56539100	-1.06252600	3.05257300
H	-5.11759300	-0.51880900	2.38257600
H	-4.47934200	0.20632500	3.86631600
C	2.02347200	-0.94147000	-0.47940200
C	3.38992200	-0.70219100	-0.36377200
F	3.86018600	0.53557500	-0.15531900
C	4.35909900	-1.69873800	-0.42316800
F	5.65208200	-1.39250300	-0.30880000
C	3.97914700	-3.02035100	-0.59067900
F	4.89257300	-3.98895400	-0.64852700
C	2.62938600	-3.32029600	-0.68849800
F	2.23067500	-4.58913000	-0.82204000
C	1.70553400	-2.28887800	-0.62230400
F	0.40580600	-2.67548200	-0.64469000
C	1.34733300	1.72073200	-0.32254000
C	1.04163700	2.78376400	0.51545800
F	0.29966600	2.60341000	1.64321500
C	1.42974600	4.10349300	0.30272500
F	1.08429200	5.05918500	1.17020800
C	2.16566200	4.41871100	-0.82447200
F	2.54692800	5.67333400	-1.05816200
C	2.49538700	3.40249800	-1.71181700
F	3.19654500	3.68756800	-2.80942000
C	2.07983700	2.10706700	-1.44962100
F	2.43426900	1.18765000	-2.35802800
H	-7.22707500	0.38842900	-0.76620100
H	-6.38590000	1.88170000	0.93521200
H	-5.22033900	2.51616600	-0.19590900
H	-2.23186700	-0.41800700	1.05526400
C	3.32493900	-1.36717400	4.09875900
C	3.02396300	-0.02900200	3.86075200
C	2.01062200	0.31931600	2.97206500
C	1.27337400	-0.66292900	2.30127200
C	1.57944900	-2.00389200	2.56165800
C	2.59479200	-2.35609800	3.44486000
H	4.12088800	-1.63679700	4.78544300
H	3.58482100	0.75174100	4.36579000
H	1.78619700	1.36576900	2.79335800
H	1.01807200	-2.78118000	2.04628200

H	2.81649500	-3.40444700	3.62126700
C	0.22619600	-0.30066700	1.27900700
H	-0.40174700	0.47861700	1.70489300
H	-0.39349000	-1.19839200	1.12222400

N21_TS

B	1.09936800	-0.13725100	-0.53800600
C	0.49057000	-0.92076400	-1.81821800
C	1.30246900	-1.88148400	-2.44255200
H	2.29508600	-2.07335000	-2.05128600
C	0.90827000	-2.59874200	-3.56978500
H	1.58645300	-3.32386400	-4.00828600
C	-0.34777300	-2.38776500	-4.11708700
H	-0.67893600	-2.94724500	-4.98598500
C	-1.17911700	-1.43852000	-3.53215500
H	-2.16720300	-1.25948500	-3.94815700
C	-0.76950200	-0.70491200	-2.42071500
C	-1.71468800	0.35139000	-1.92352500
H	-2.65935800	0.29109000	-2.47250800
H	-1.29496800	1.34326200	-2.09671800
N	-2.01106100	0.25240400	-0.46448700
C	-2.55611100	1.55063200	0.02777600
C	-3.97632700	1.77438800	-0.46035700
H	-4.03670100	1.73369400	-1.55484700
H	-4.30613600	2.77492200	-0.16436000
C	-4.89566100	0.72261700	0.16296600
H	-4.99462800	0.97209200	1.23053600
C	-4.26658800	-0.66153600	0.09336100
C	-5.02386200	-1.79770000	0.39921900
H	-6.06695000	-1.67602400	0.67431100
C	-4.48111600	-3.07467100	0.37494000
H	-5.10263100	-3.92914500	0.62836600
C	-3.14270300	-3.27211200	0.02821900
C	-2.37063300	-2.15129000	-0.25973600
H	-1.31877200	-2.28045900	-0.49172400
C	-2.91543400	-0.86641600	-0.21066100
C	-6.28204700	0.78027800	-0.48252500
H	-6.66031500	1.80653600	-0.46034800
H	-7.01071600	0.15080400	0.03180800
H	-6.23095200	0.45616100	-1.52662600
C	-2.53750700	-4.64981200	-0.03514300
H	-2.98003800	-5.31150200	0.71343500
H	-1.45818800	-4.61091200	0.13065200
H	-2.70532800	-5.10183700	-1.01809200
C	1.19814000	1.47811700	-0.61136400
C	0.96282400	2.23941400	-1.75761900
F	0.69652000	1.66418100	-2.93840000
C	0.97544200	3.63219500	-1.78098700
F	0.70656200	4.28645500	-2.91002300
C	1.28532200	4.33615100	-0.62987100
F	1.30068400	5.66412000	-0.63125500
C	1.60291700	3.62822200	0.52129700
F	1.93667800	4.27558900	1.63625400
C	1.58683000	2.24388200	0.49198900
F	2.00688400	1.64546000	1.61745000
C	2.40595800	-0.89596000	0.05810500
C	3.66401700	-0.31400300	0.19832900
F	3.88655700	0.95278800	-0.17227700
C	4.77106500	-0.98804000	0.70609300
F	5.94550900	-0.36998500	0.81326600
C	4.64945400	-2.31187900	1.09194400
F	5.69483000	-2.97262600	1.57733600
C	3.42199100	-2.94685600	0.95474700
F	3.29392000	-4.22666400	1.30101400
C	2.34610200	-2.23955300	0.44259500
F	1.20491500	-2.93685700	0.30272300
H	-0.88708000	-0.01409200	0.19404800
H	-1.85803000	2.32824400	-0.29653400
H	-2.55648200	1.52877900	1.11684300
C	-2.90479700	1.32620900	4.04455800
C	-1.72975000	2.00115700	3.71874000
C	-0.79108800	1.41770200	2.87353400
C	-1.00502900	0.14419800	2.32208400
C	-2.16151800	-0.54656300	2.71187500
C	-3.10477400	0.03905600	3.55205300
H	-3.64004900	1.78618900	4.69698900
H	-1.54138200	2.99033700	4.12505600
H	0.11869200	1.95846800	2.64121400
H	-2.33247200	-1.55298600	2.34093500
H	-3.99682500	-0.51871300	3.82237400
C	-0.04588000	-0.46219400	1.32585900
H	0.94703500	-0.22709600	1.68966700
H	-0.15686300	-1.54560500	1.32798800

N21_P

B	0.62349400	-0.45962900	-0.23412400
C	-0.29742400	-1.16001200	-1.43067400
C	0.15463600	-2.43592600	-1.82698500

H	1.09758400	-2.79183100	-1.41592900
C	-0.52560200	-3.26098600	-2.70816600
H	-0.10869400	-4.22733100	-2.97488600
C	-1.74355800	-2.84739700	-3.24687800
H	-2.29346100	-3.47825700	-3.93716700
C	-2.24480400	-1.61569900	-2.86561700
H	-3.20870300	-1.28574700	-3.24625600
C	-1.54120100	-0.78616000	-1.97729200
C	-2.25371000	0.47185700	-1.57081700
H	-3.21191600	0.56348800	-2.08408300
H	-1.66725200	1.37413500	-1.73875200
N	-2.58079900	0.45594000	-0.07510000
C	-2.89359600	1.82898300	0.44540100
C	-4.25499100	2.26913800	-0.05188300
H	-4.25809300	2.29429900	-1.14788400
H	-4.43118300	3.29646100	0.27833800
C	-5.37301700	1.35720500	0.45919600
H	-5.57592500	1.63336400	1.50347400
C	-4.94761100	-0.10351300	0.47256900
C	-5.87553000	-1.10601300	0.76977600
H	-6.90576000	-0.82654300	0.96568900
C	-5.51637300	-2.44612800	0.83048800
H	-6.27192400	-3.19248000	1.05771900
C	-4.19858400	-2.84862000	0.61035900
C	-3.25385500	-1.86376900	0.33150600
H	-2.21933000	-2.14312300	0.15592200
C	-3.63297400	-0.52753300	0.26215600
C	-6.64831900	1.59264800	-0.35534600
H	-6.89193800	2.65873700	-0.36891500
H	-7.50797100	1.06237200	0.05760400
H	-6.50730300	1.25903300	-1.38787100
C	-3.77856000	-4.29275900	0.66925500
H	-3.22127900	-4.56592800	-0.23100700
H	-4.64281800	-4.95369100	0.75652100
H	-3.12388400	-4.47158200	1.52727600
C	0.34716300	1.16432500	0.05152500
C	0.46244500	2.06412600	-1.01104100
F	0.58864400	1.58973400	-2.26009000
C	0.42360500	3.44567500	-0.88030800
F	0.51836900	4.24169800	-1.94457800
C	0.29494200	4.00044500	0.38884000
F	0.26121700	5.32092800	0.54428700
C	0.19479000	3.15852800	1.48435800
F	0.05572500	3.67323300	2.70626800
C	0.22419900	1.77780500	1.29535600
F	0.08045600	1.04998100	2.41292900
C	2.22929700	-0.39752500	-0.63276900
C	2.80291800	-0.62372400	-1.88404600
F	2.05994600	-0.94438100	-2.95280900
C	4.16887300	-0.52207000	-2.13741000
F	4.64909300	-0.77866000	-3.35645000
C	5.03038700	-0.12972100	-1.12790000
F	6.33815600	-0.02210100	-1.35707200
C	4.50648900	0.17353400	0.12016900
F	5.31259200	0.59713000	1.09444700
C	3.13997200	0.05723100	0.32753500
F	2.70235200	0.45334400	1.53225000
H	-1.70606000	0.15210000	0.37749600
H	-2.09181700	2.48329200	0.10068500
H	-2.85741400	1.76689900	1.53733800
C	3.34991700	-2.54166200	3.96445600
C	3.44242800	-2.94839900	2.63579800
C	2.45322600	-2.59158700	1.72566800
C	1.35662300	-1.80990000	2.11072700
C	1.26772700	-1.43036500	3.45268600
C	2.25263400	-1.78766700	4.36906000
H	4.12105600	-2.81585400	4.67712000
H	4.28631500	-3.54750900	2.30703000
H	2.53385500	-2.91538600	0.69010400
H	0.41829700	-0.83993600	3.78165400
H	2.16183100	-1.47334100	5.40459500
C	0.29955300	-1.45625600	1.09281000
H	-0.01529300	-2.41502100	0.66289500
H	-0.58443200	-1.09085200	1.63958500

Methylbenzene

N4_TS

C	1.23779700	1.26976500	1.24431200
C	0.66103000	1.43219800	-0.01633500
C	0.63496900	2.73125700	-0.53881400
C	1.15998000	3.80549400	0.17359800
C	1.73827700	3.60446300	1.42642600
C	1.78204700	2.32527600	1.97023600
H	0.19934200	2.89443000	-1.52033100
H	1.12730900	4.80413300	-0.25093000
H	2.15459400	4.44178200	1.97725600
H	2.23121100	2.15597600	2.94519000
H	0.91917800	-0.69901200	0.40425800

B	0.09499700	0.16922700	-0.83608100
N	1.22217400	-0.09372500	1.73519500
C	-1.45743900	-0.19046300	-0.53767500
C	-2.41155500	0.65536100	-1.10265500
C	-1.98276800	-1.23334100	0.21436800
C	-3.77997100	0.48563400	-0.95025600
C	-3.34534000	-1.44274400	0.39962800
C	-4.25179100	-0.57599300	-0.18958900
C	2.51360300	-0.56496600	2.25235000
H	2.47207200	-1.65207500	2.36778400
H	3.30126500	-0.31341300	1.54060200
H	2.74067100	-0.11971800	3.22982100
C	0.14542000	-0.31145600	2.71298500
H	0.05503300	-1.37927700	2.92342000
H	0.35736100	0.23005000	3.64416300
H	-0.79473600	0.05352300	2.29649100
F	-2.01277500	1.69724600	-1.84506200
F	-4.64152700	1.32461700	-1.52235700
F	-5.55942700	-0.75614300	-0.02601700
F	-3.78061900	-2.46402800	1.13765500
F	-1.16846800	-2.11694500	0.82867500
H	0.27316900	0.32059000	-2.01391100
C	5.37054200	-1.11941300	-1.44112900
C	4.52558300	-0.06590700	-1.77657400
C	3.15591500	-0.15287100	-1.53776600
C	2.60667600	-1.30074900	-0.95759000
C	3.46779700	-2.35345200	-0.62755300
C	4.83524800	-2.26897600	-0.86609200
H	6.43751500	-1.04505300	-1.62494100
H	4.93285700	0.83591200	-2.22276100
H	2.51521600	0.68338000	-1.79432200
H	3.05524900	-3.25227000	-0.17479400
H	5.48146900	-3.09977100	-0.60056500
C	1.13510500	-1.43176500	-0.64736800
H	0.55113800	-1.72742300	-1.52059500
H	0.97847600	-2.26865900	0.04511100

N4_P

C	-1.54821600	-1.77256600	-0.19617800
C	-0.62473700	-1.03120000	-0.92673800
C	-0.08460300	-1.73209600	-2.02493100
C	-0.42588900	-3.03867200	-2.33768700
C	-1.35407700	-3.72959600	-1.55615700
C	-1.92487300	-3.08775800	-0.46958800
H	0.63773000	-1.20673400	-2.64462500
H	0.02946300	-3.52748500	-3.19338300
H	-1.62800000	-4.75242100	-1.79079300
H	-2.64432700	-3.61096600	0.15475700
H	-1.84956100	-0.14884200	0.93399500
B	-0.04806500	0.49685400	-0.70137200
N	-2.15227600	-1.12748400	1.00015100
C	1.53463900	0.41853500	-0.28204700
C	2.43324100	1.38965100	-0.71901900
C	2.09434700	-0.54663600	0.54523600
C	3.78323100	1.39623400	-0.38917400
C	3.43650500	-0.58806600	0.90204300
C	4.29087200	0.39466200	0.42622900
C	-3.64243200	-1.10896100	0.98319800
H	-3.99109700	-0.53619800	1.84235400
H	-3.96273500	-0.62189100	0.06169300
H	-4.00973300	-2.13319300	1.03200000
C	-1.60468600	-1.66978500	2.27593800
H	-2.00104800	-1.07944700	3.10420400
H	-1.91413900	-2.71107200	2.37076200
H	-0.51908200	-1.60235100	2.22934100
F	2.00792900	2.39802000	-1.49285400
F	4.59548900	2.35392800	-0.84165600
F	5.58286500	0.38149400	0.75469100
F	3.90551000	-1.55241200	1.69911800
F	1.32178200	-1.52868500	1.06717100
H	-0.09444600	0.99451900	-1.80793900
C	-5.03674900	2.21762900	-0.06457400
C	-4.26199500	1.88993700	-1.17661500
C	-2.89072000	1.68116300	-1.04665700
C	-2.25474500	1.81064300	0.19714900
C	-3.05042300	2.14414100	1.30313000
C	-4.42367100	2.34701800	1.17874600
H	-6.10539500	2.37616700	-0.16849700
H	-4.72923100	1.78997100	-2.15186700
H	-2.29304200	1.41227900	-1.91219200
H	-2.57562100	2.25669200	2.27653000
H	-5.01266000	2.61184300	2.05206300
C	-0.77023200	1.54954700	0.35259100
H	-0.24017300	2.49475400	0.17489500
H	-0.56153600	1.32845700	1.41491500

N5_TS

B	0.50028100	-0.29694100	0.23225300

C	-0.19488200	-1.07404600	1.47037900
C	0.66330700	-1.41849000	2.53093700
H	1.70834800	-1.13441700	2.47691200
C	0.22433700	-2.07665700	3.66858500
H	0.92497400	-2.31149100	4.46342600
C	-1.11582800	-2.43202500	3.78110200
H	-1.48076900	-2.95324000	4.66019100
C	-1.99116100	-2.11200400	2.75472000
H	-3.03057100	-2.39382300	2.84471700
C	-1.54333400	-1.43434300	1.61210600
N	-2.45757100	-1.10804000	0.52118600
C	-3.02005500	-2.33577600	-0.19122900
C	-3.71790000	-1.83118900	-1.46318800
H	-2.97274500	-1.37751600	-2.12501900
H	-4.12793500	-2.69746900	-1.99350400
C	-4.80565600	-0.80369400	-1.17137000
H	-5.61372300	-1.26192900	-0.58780400
H	-5.25490700	-0.46196900	-2.10908600
C	-4.20535500	0.38909600	-0.43345000
H	-4.98893500	1.10219300	-0.15390400
H	-3.52665300	0.91971400	-1.10522000
C	-3.45628600	0.00516900	0.85395600
C	-4.49673400	-0.29142100	1.95183300
H	-5.29353200	-0.96617800	1.64214300
H	-4.97204700	0.66372100	2.19541600
H	-4.04188700	-0.65918200	2.87127000
C	-2.64460700	1.19775800	1.35969400
H	-1.78069000	1.38826700	0.72535600
H	-2.28979900	1.03470400	2.37890300
H	-3.27599600	2.09107300	1.34558300
C	-4.00256200	-3.20731500	0.61624300
H	-3.48952200	-3.76657100	1.39961700
H	-4.42417800	-3.94355100	-0.07505000
H	-4.83390300	-2.66603400	1.05899700
C	-1.86713900	-3.26800800	-0.57142200
H	-1.10656500	-2.78402100	-1.17671100
H	-2.27658200	-4.10104200	-1.15040900
H	-1.38272900	-3.68210300	0.31623900
C	0.92404000	1.24948100	0.50970100
C	0.65247900	1.96313300	1.67613100
F	-0.00408400	1.40916100	2.70194400
C	1.03504500	3.28781300	1.86867200
F	0.73746200	3.91503100	3.00514500
C	1.74754600	3.94974100	0.88208400
F	2.12327000	5.21207500	1.05464200
C	2.07053000	3.27498500	-0.28732200
F	2.76188100	3.89264300	-1.24200200
C	1.66545800	1.95874400	-0.43918700
F	2.00281600	1.37594000	-1.60275500
C	1.83805800	-1.13345400	-0.27660700
C	3.07289300	-0.90177300	0.33804000
F	3.19054500	-0.00415100	1.33294800
C	4.24902300	-1.56252700	0.00994000
F	5.38202200	-1.28109300	0.64977200
C	4.24113300	-2.51612600	-0.99558700
F	5.35595400	-3.15782000	-1.32829000
C	3.04823300	-2.78760700	-1.64282400
F	3.00745400	-3.69608700	-2.61542100
C	1.89504300	-2.10363700	-1.27470600
F	0.80175100	-2.43679700	-1.98593600
H	-1.43122600	-0.51597800	-0.36256000
C	-2.63508900	3.19080600	-3.40541300
C	-1.98485200	3.43628200	-2.20108800
C	-1.32740800	2.41207500	-1.52294300
C	-1.30905700	1.10653600	-2.03038200
C	-1.94311600	0.88702400	-3.26192600
C	-2.60437500	1.90631500	-3.93887200
H	-3.15020800	3.99164800	-3.92545600
H	-1.98601700	4.43532100	-1.77628700
H	-0.84355500	2.64875300	-0.58391700
H	-1.91510600	-0.10785800	-3.69989200
H	-3.09017900	1.69485600	-4.88629300
C	-0.64382100	-0.09553600	-1.37615300
H	0.41185100	-0.00089900	-1.65009600
H	-0.90268100	-0.98008400	-1.96028200

N5_P

B	0.65186400	-0.16222500	-0.06561200
C	-0.14849300	-0.84036500	1.25093400
C	0.73209700	-1.11365200	2.32422400
H	1.78385800	-0.88866400	2.17696900
C	0.34681400	-1.61148000	3.55559400
H	1.09007000	-1.78268500	4.32755700
C	-0.99798200	-1.87558500	3.79670500
H	-1.33578000	-2.26098400	4.75262200
C	-1.91156300	-1.63043200	2.78762300
H	-2.95740700	-1.82713300	2.97102600
C	-1.48984300	-1.13291500	1.54673100
N	-2.56949600	-0.89795300	0.53729700

C	-3.24885000	-2.20196200	-0.00809800
C	-4.20753300	-1.79804400	-1.13945300
H	-3.61465600	-1.48318100	-2.00263100
H	-4.74515500	-2.70364800	-1.43800700
C	-5.17163500	-0.67298100	-0.79077000
H	-5.86885100	-0.97767500	-0.00136900
H	-5.77795700	-0.43291000	-1.66904600
C	-4.37004300	0.55347200	-0.37386300
H	-5.02836300	1.39876600	-0.14961700
H	-3.72537300	0.86808200	-1.20328800
C	-3.50727200	0.31436900	0.86992500
C	-4.38802800	0.13450200	2.10750700
H	-5.09750200	-0.68894200	2.04828300
H	-4.97274200	1.05519300	2.19242600
H	-3.79985700	0.04677200	3.02012700
C	-2.60091000	1.51377500	1.12328300
H	-1.83484800	1.61491800	0.35248700
H	-2.11074300	1.44678700	2.09324800
H	-3.21754300	2.41684400	1.09980000
C	-3.98179700	-3.03510600	1.04577900
H	-3.29300200	-3.48206200	1.76166400
H	-4.46075500	-3.85550900	0.50472900
H	-4.76675600	-2.50401200	1.58199100
C	-2.13306500	-3.06304100	-0.58777200
H	-1.59277800	-2.53760600	-1.37708500
H	-2.58464200	-3.95738500	-1.02591300
H	-1.41433200	-3.36900800	0.17316900
C	1.04181900	1.41747000	0.28952300
C	0.68157700	2.14406400	1.42245100
F	0.02524300	1.58080900	2.45492900
C	0.95399500	3.49949900	1.60694600
F	0.54318400	4.12093400	2.71482100
C	1.65957800	4.19553300	0.64466000
F	1.93410700	5.48860400	0.80062600
C	2.09285200	3.51381200	-0.48526100
F	2.80695500	4.15499000	-1.41086900
C	1.78819500	2.17005800	-0.62605400
F	2.29163100	1.58476300	-1.72508700
C	2.04459200	-1.08246800	-0.21778300
C	3.36469400	-0.66101900	-0.06214500
F	3.66440400	0.58067000	0.35102800
C	4.47283800	-1.47892800	-0.26803800
F	5.70583000	-1.00028700	-0.09682500
C	4.29634900	-2.80202300	-0.63324800
F	5.34448800	-3.59945300	-0.82871100
C	3.00516300	-3.28641400	-0.77427100
F	2.80383700	-4.56411200	-1.10459400
C	1.93494800	-2.43078200	-0.55892100
F	0.71693900	-2.99870300	-0.68584800
H	-2.05823600	-0.54949800	-0.28960900
C	-3.14281600	1.98740900	-3.58574000
C	-2.29817000	2.63492200	-2.68665600
C	-1.29495300	1.93316200	-2.02211400
C	-1.10514700	0.55617000	-2.22458300
C	-1.95534800	-0.06646500	-3.15184100
C	-2.96173700	0.62771200	-3.82092700
H	-3.92021000	2.53782100	-4.10558700
H	-2.41265600	3.69940000	-2.50527700
H	-0.64234100	2.47004600	-1.34194800
H	-1.79815400	-1.12035300	-3.37470400
H	-3.59347300	0.10614900	-4.53434200
C	0.03203100	-0.24478400	-1.62084300
H	0.90147700	0.01439700	-2.23404200
H	-0.14783800	-1.29595600	-1.86289900

N11_TS

B	0.22142500	-0.02444500	0.30827400
N	-2.22279400	-1.63742100	0.40147700
C	-1.26834400	-1.90267600	1.49467400
H	-0.65955400	-2.74321300	1.13892600
C	-0.31482400	-0.71517700	1.71288000
H	-0.93825300	0.06823500	2.16935500
C	0.48565800	-1.26832200	2.90507600
H	1.18403600	-2.04481800	2.58103500
H	1.06241000	-0.49881300	3.41802400
C	-0.59680600	-1.89292800	3.81858100
H	-0.21408800	-2.76426300	4.35488200
H	-0.91763300	-1.17028800	4.57371300
C	-1.78900100	-2.25784800	2.89409900
H	-2.66318800	-1.65282100	3.15016800
H	-2.08275800	-3.30787100	2.97417700
C	-2.76613000	-2.88998400	-0.16495600
H	-3.36427900	-3.40178800	0.60665500
H	-1.91879100	-3.53482400	-0.41583900
C	-3.63009900	-2.62296200	-1.39233700
H	-4.01084400	-3.57628100	-1.77130100
H	-3.01037300	-2.18456600	-2.18547900
C	-4.77072700	-1.66583700	-1.05428200

H	-5.36404400	-1.44040000	-1.94484600
H	-5.44296900	-2.14375300	-0.32909200
C	-4.19493800	-0.38517700	-0.45248900
H	-3.61017300	0.13944100	-1.21286900
H	-4.99259300	0.29108600	-0.12970900
C	-3.31672600	-0.70798000	0.75343900
H	-2.87808900	0.20001300	1.17592300
H	-3.94465300	-1.17303100	1.52849500
C	-0.69880300	1.30691600	0.04807800
F	0.20125600	2.10744300	2.07994900
C	-0.63000900	2.28576500	1.04043500
F	-1.27486000	4.33910800	2.02258400
C	-1.38177600	3.45216400	1.03540700
F	-2.99643500	4.79155300	-0.04316100
C	-2.25317300	3.68925100	-0.01889400
F	-3.16693500	2.96786800	-2.06795100
C	-2.33449500	2.76395900	-1.04742400
F	-1.66489400	0.79814700	-2.06513400
C	-1.54460700	1.62079200	-1.00653900
C	1.77657200	0.42213900	0.01134100
F	1.08782000	1.48787200	-2.00516200
C	2.05535400	1.21486600	-1.11148000
F	3.47170900	2.47374000	-2.51909700
C	3.30125600	1.73735600	-1.42344800
F	5.57583400	1.97933900	-0.85398400
C	4.37309400	1.48825400	-0.57982500
F	5.18149400	0.42862500	1.35804200
C	4.16730400	0.70120700	0.53896700
F	2.83449100	-0.58004500	1.89567200
C	2.89858300	0.19682100	0.80664500
H	-1.10552400	-1.15933600	-0.44015000
C	1.85028200	-5.13863900	-1.06037700
C	0.86078400	-4.87976200	-2.00400400
C	0.25134400	-3.62922400	-2.04903000
C	0.60823300	-2.60981300	-1.15765500
C	1.60585300	-2.88683700	-0.21632300
C	2.21890100	-4.13578800	-0.16869400
H	2.32792500	-6.11207300	-1.01874200
H	0.56146500	-5.65009500	-2.70791800
H	-0.51954000	-3.43582300	-2.79256800
H	1.90271000	-2.13057400	0.50043500
H	2.98772800	-4.32256700	0.57457300
C	-0.08953200	-1.27386500	-1.25398400
H	-0.92183100	-1.37784700	-1.96578900
H	0.50064000	-0.53242300	-1.78684400

N11_P

B	0.24605400	-0.20552000	0.04054700
N	-2.47088200	-1.85439200	0.19374400
C	-1.23796000	-2.24436600	0.99030200
H	-0.69445600	-2.88125600	0.28906600
C	-0.28772800	-1.09604400	1.36648600
H	-0.84743400	-0.41078100	2.02461300
C	0.60870000	-1.89485500	2.33484700
H	1.24166400	-2.59323700	1.77898000
H	1.26719700	-1.24834100	2.91504500
C	-0.38630200	-2.66517800	3.21696300
H	0.05882200	-3.53254800	3.70920400
H	-0.77038500	-2.00471400	4.00049100
C	-1.53780500	-3.06221000	2.26274700
H	-2.51130800	-2.82444000	2.70073900
H	-1.53964000	-4.13435900	2.04650400
C	-3.11269200	-3.06575600	-0.41754100
H	-3.46073100	-3.68399200	0.41623000
H	-2.32170200	-3.60389000	-0.94587500
C	-4.25803500	-2.68058300	-1.34224900
H	-4.68249900	-3.59727300	-1.76067700
H	-3.86169400	-2.09364400	-2.18028000
C	-5.30860900	-1.86625400	-0.58899700
H	-6.11562700	-1.56766000	-1.26228700
H	-5.75546000	-2.48199700	0.20220500
C	-4.64288900	-0.63300900	0.01716000
H	-4.29472500	0.01705800	-0.79103600
H	-5.34986900	-0.04759200	0.61179100
C	-3.47967400	-1.01629800	0.92767700
H	-2.95019500	-0.14276400	1.31128500
H	-3.83580500	-1.61738600	1.76718000
C	-0.95922000	0.95072600	-0.09576400
F	-0.18652000	1.93801900	1.91325300
C	-1.05576500	1.93682400	0.89226900
F	-2.02157300	3.86091400	1.87375200
C	-1.99971000	2.95290000	0.90001700
F	-3.87575700	3.96616600	-0.12183100
C	-2.94201600	3.01822800	-0.12081700
F	-3.85011600	2.03383400	-2.06300600
C	-2.90513000	2.06067300	-1.11739800
F	-2.06904600	0.10438100	-2.03207600
C	-1.93228500	1.06649400	-1.07398400

C	1.67972700	0.62352800	0.18350900
F	0.88959100	2.51035500	-1.06341000
C	1.88285400	1.87307900	-0.41265500
F	3.18811300	3.74840600	-1.02069800
C	3.09578800	2.55207600	-0.43590900
F	5.38983000	2.60384100	0.12558400
C	4.21526300	1.97601700	0.14107100
F	5.15829600	0.11764900	1.23418100
C	4.08877500	0.72204300	0.71259200
F	2.88037700	-1.16158100	1.20459900
C	2.85382400	0.08444000	0.70968800
H	-2.11833600	-1.27954900	-0.58503700
C	4.02211700	-3.46891700	-1.46847100
C	2.82125100	-4.04269600	-1.06481300
C	1.65104100	-3.28816800	-1.06536700
C	1.64365500	-1.94354900	-1.45207400
C	2.85571000	-1.39904800	-1.89730700
C	4.02998700	-2.14405600	-1.89664300
H	4.93816000	-4.05039900	-1.46303300
H	2.79200900	-5.08233300	-0.75214300
H	0.72002300	-3.76955700	-0.77171700
H	2.87556800	-0.36501000	-2.23393000
H	4.95541100	-1.68709900	-2.23435800
C	0.40304300	-1.08991200	-1.37417000
H	-0.46231600	-1.73683100	-1.59335500
H	0.42498300	-0.36669900	-2.19872200

N12_TS

N	1.52827300	-2.43251100	0.39603100
H	0.63190500	-1.54232700	-0.40100100
B	0.11047800	0.14972800	0.24515100
C	1.76619800	-1.46815500	1.50616000
H	2.60393200	-0.84006900	1.16610200
C	0.53351100	-0.56637100	1.64669400
H	-0.28342300	-1.19915600	2.01557900
C	2.79457900	-2.88666600	-0.21967100
H	3.36379700	-3.46705800	0.52478200
H	3.38058700	-2.00543300	-0.48010400
C	2.54799300	-3.75499200	-1.44859900
H	2.05251800	-3.15624300	-2.22254800
H	3.51414800	-4.06595000	-1.85794700
C	1.68818700	-4.96547200	-1.09064900
H	2.25115800	-5.61480600	-0.40670700
H	1.46065100	-5.55742300	-1.98177400
C	0.40346300	-4.50037800	-0.40928200
H	-0.22366800	-3.95508400	-1.12257000
H	-0.18607900	-5.35339000	-0.05916100
C	0.72072900	-3.61074100	0.78945700
H	-0.19973700	-3.25966500	1.26290000
H	1.27919300	-4.20145500	1.53107100
C	2.17051400	-2.11535100	2.83510100
H	1.35487500	-2.75651800	3.19181500
H	3.05233000	-2.75405900	2.70251100
C	2.43319400	-1.04637000	3.89670900
H	2.71178100	-1.52197100	4.84241900
H	3.28571800	-0.42551100	3.58997400
C	1.20064200	-0.16003400	4.07399000
H	1.39261500	0.61438700	4.82379800
H	0.36573600	-0.76748800	4.44903200
C	0.80305200	0.48196200	2.74496500
H	1.62168000	1.14026500	2.42231900
H	-0.07522000	1.11896900	2.87733400
C	1.28423600	1.16178700	-0.27945800
C	1.31864700	2.47805700	0.18460900
F	0.37722900	2.91817400	1.03247800
C	2.29217900	3.40051700	-0.17219100
F	2.26585300	4.63825700	0.31724300
C	3.30334500	3.02370800	-1.04473900
F	4.24763100	3.88986200	-1.39746600
C	3.31758100	1.72911600	-1.53674300
F	4.28007100	1.34110500	-2.37282300
C	2.32087900	0.84214300	-1.14699400
F	2.41543000	-0.39767900	-1.67459000
C	-1.35014900	0.87710200	0.23089300
C	-1.71889700	1.71832300	-0.82204000
F	-0.87623000	1.91905400	-1.85189900
C	-2.93357100	2.38147200	-0.90951000
F	-3.20901500	3.16307500	-1.95144700
C	-3.86306600	2.22857200	0.10818500
F	-5.03501000	2.85155500	0.05130400
C	-3.55381000	1.40894800	1.18059100
F	-4.43595400	1.24105400	2.16469800
C	-2.32309200	0.76460200	1.22323900
F	-2.14026600	-0.01893100	2.30139700
C	-3.97093900	-3.22149400	-1.46360000
C	-3.26050700	-2.91690300	-2.62216800
C	-2.06136000	-2.21715600	-2.54033700
C	-1.54356900	-1.80487000	-1.30665600

C	-2.27087400	-2.11285300	-0.15462800
C	-3.47209700	-2.81497800	-0.23075400
H	-4.90596500	-3.76894000	-1.52268200
H	-3.63908500	-3.22604500	-3.59138900
H	-1.50965400	-1.98536800	-3.44835300
H	-1.90922900	-1.79144000	0.81622000
H	-4.01858400	-3.04016300	0.67969800
C	-0.21467100	-1.08284300	-1.28847900
H	0.47808000	-1.62060400	-1.95270500
H	-0.28198200	-0.13232500	-1.82118500

N12_P

N	2.37982800	-0.33172200	0.81948100
H	1.94528000	-0.21962900	-0.10554800
B	-0.59063100	-0.30560600	-0.22207300
C	1.22516600	-0.48547800	1.83160100
H	1.03018100	0.54960100	2.13886300
C	-0.08346500	-1.02655100	1.22986100
H	0.07433800	-2.09655500	1.04330100
C	3.14531700	0.92438000	1.14088800
H	3.53897900	0.77735200	2.15214300
H	2.41554800	1.73582800	1.16462500
C	4.26976000	1.20651200	0.15784900
H	3.85078800	1.42387000	-0.83120400
H	4.78312500	2.11305200	0.49203100
C	5.21957500	0.01503400	0.06967700
H	5.71025400	-0.14530600	1.03866400
H	6.00602600	0.20630000	-0.66466100
C	4.41100600	-1.21579200	-0.32643200
H	3.96589100	-1.05509000	-1.31233700
H	5.03299000	-2.11232700	-0.40445700
C	3.30382700	-1.51395400	0.67921300
H	2.69296500	-2.36824300	0.37484100
H	3.73292400	-1.70319200	1.66525400
C	1.71269600	-1.25500100	3.06047000
H	1.87291800	-2.30697800	2.79320100
H	2.66790000	-0.85613700	3.42624700
C	0.66316300	-1.17970600	4.16827300
H	1.00447100	-1.75116500	5.03690500
H	0.55353800	-0.13657200	4.49331300
C	-0.67410500	-1.69553300	3.65052600
H	-1.44108600	-1.60372000	4.42653500
H	-0.58321400	-2.76490100	3.41637100
C	-1.10403200	-0.93678600	2.39403200
H	-1.27183300	0.11626300	2.64949400
H	-2.06475600	-1.32856300	2.06663600
C	0.07405600	1.23182200	-0.32119300
C	-0.34513800	2.25736200	0.53098400
F	-1.31500800	2.02449500	1.42651800
C	0.15707600	3.55146300	0.52158700
F	-0.31007600	4.47163600	1.36289800
C	1.15700700	3.89255300	-0.38207400
F	1.66320400	5.12203000	-0.40131700
C	1.62653900	2.91871700	-1.24542200
F	2.63284300	3.18499100	-2.08322300
C	1.08626900	1.63729300	-1.18297600
F	1.73112600	0.72825000	-1.96128900
C	-2.24831300	-0.19317700	-0.28745600
C	-3.03433900	0.89595300	-0.65591600
F	-2.49391600	2.09276600	-0.94558700
C	-4.42127100	0.84927000	-0.78112500
F	-5.10143800	1.94239200	-1.13104500
C	-5.09617800	-0.33755300	-0.55792900
F	-6.42121300	-0.39946900	-0.67532600
C	-4.36417100	-1.46665400	-0.21895700
F	-4.98590400	-2.62902100	-0.01071000
C	-2.98654600	-1.36548200	-0.11158800
F	-2.34280900	-2.51704100	0.18632700
C	2.73005300	-4.37622900	-1.66646400
C	2.90770700	-3.22914000	-2.43144000
C	1.96081700	-2.20327300	-2.40171300
C	0.80685000	-2.29703100	-1.61438100
C	0.61973600	-3.49134700	-0.89256700
C	1.56722100	-4.50779800	-0.90498800
H	3.46613100	-5.17361000	-1.68979900
H	3.78746100	-3.12829600	-3.06165800
H	2.10499600	-1.32379900	-3.02347200
H	-0.30804800	-3.62334800	-0.34359800
H	1.38845300	-5.41673200	-0.33801400
C	-0.26257000	-1.23659600	-1.59809500
H	-0.12449200	-0.57334100	-2.45744700
H	-1.19091300	-1.77761800	-1.80292500

N13_TS

N	-2.31005900	-0.77451800	0.98779700
H	-0.80274800	-0.50175300	1.14989800
B	0.20148600	-0.18934200	-0.22269700
C	-2.36868100	-0.52987500	-0.48998800

H	-2.47149100	0.55808600	-0.57784500
C	-1.00196900	-0.91273300	-1.04402100
H	-0.93029800	-0.56769200	-2.08264000
H	-0.87585000	-1.99628200	-1.07593000
C	-2.50047100	-2.20315600	1.33351100
H	-1.75158800	-2.78794600	0.78818100
H	-3.49208100	-2.52664200	0.98591400
C	-2.39743500	-2.46460300	2.83577300
H	-1.37522900	-2.28914900	3.18563000
H	-2.61250600	-3.52338700	3.01002600
C	-3.36853900	-1.57862000	3.61406100
H	-4.39942200	-1.86552800	3.36772900
H	-3.24255500	-1.72280000	4.69093300
C	-3.15314200	-0.11598200	3.23148500
H	-3.88742200	0.52963500	3.72284500
H	-2.16247500	0.22300400	3.55703800
C	-3.27782200	0.06371400	1.72314500
H	-4.29808000	-0.20539600	1.40481000
H	-3.10027000	1.10516700	1.44153900
C	-3.57510900	-1.14034100	-1.18147600
C	-4.74545100	-0.38712400	-1.31093000
H	-4.76228300	0.63509400	-0.93964400
C	-5.87891700	-0.91798400	-1.92044600
H	-6.77575600	-0.31379500	-2.01330900
C	-5.85596000	-2.21785800	-2.41615300
H	-6.73590500	-2.63563000	-2.89445000
C	-4.69366300	-2.97666900	-2.30366600
H	-4.66417000	-3.98798400	-2.69656700
C	-3.56309000	-2.44094500	-1.69487700
H	-2.66129200	-3.04174900	-1.62446700
C	1.65499300	-0.71561400	-0.69221700
C	2.75165900	0.11089700	-0.93810200
F	2.65804200	1.43927400	-0.81321000
C	4.00957900	-0.36776500	-1.28193000
F	5.01785400	0.47384500	-1.49840100
C	4.21851600	-1.73402000	-1.38848800
F	5.41756100	-2.20899900	-1.71125100
C	3.16470900	-2.59975600	-1.14030500
F	3.35251600	-3.91818900	-1.20708600
C	1.92443800	-2.07838200	-0.80129200
F	0.98544800	-2.99213600	-0.47787900
C	-0.05771200	1.41780400	-0.26435800
C	-0.62290000	2.21594000	0.72115000
F	-0.87809400	1.72132100	1.95471100
C	-0.96018900	3.55351200	0.54424600
F	-1.49537600	4.25217400	1.54524300
C	-0.74647600	4.15061100	-0.68676600
F	-1.06500300	5.42639600	-0.88136800
C	-0.19727300	3.39628200	-1.71576200
F	0.00925800	3.95227400	-2.90697700
C	0.11794900	2.06550800	-1.48824700
F	0.62464500	1.38456200	-2.52512500
C	4.41332300	0.43976400	2.57156200
C	4.09438400	-0.90189400	2.37458300
C	2.78042700	-1.27271500	2.11528800
C	1.76084700	-0.31702600	2.03591900
C	2.08358700	1.01869500	2.28068100
C	3.40083200	1.39238000	2.53682100
H	5.43935000	0.73680400	2.76181100
H	4.86949100	-1.66094700	2.41690800
H	2.53929200	-2.31951700	1.94643900
H	1.30835600	1.77392600	2.27433200
H	3.63293800	2.43881900	2.70739000
C	0.35973600	-0.79187700	1.70458300
H	0.36245900	-1.88195800	1.61927200
H	-0.25022600	-0.53975500	2.58244600

N13_P

N	-2.57807900	0.25041600	-1.05313500
H	-1.73981800	-0.29441500	-1.29540500
B	0.37749500	0.33234200	-0.15175100
C	-2.24801000	0.87035900	0.31917400
H	-2.28851100	-0.00036200	0.98243900
C	-0.82163800	1.40397600	0.30556100
H	-0.63092600	1.72546100	1.33738800
H	-0.76358300	2.31593700	-0.29642300
C	-2.75487800	1.25145700	-2.15408100
H	-1.86936900	1.89016800	-2.14435600
H	-3.63265300	1.84950600	-1.89478500
C	-2.93444200	0.55155800	-3.49592300
H	-2.01403000	0.01246300	-3.75195400
H	-3.07471900	1.32053300	-4.26046300
C	-4.11692500	-0.41644300	-3.46013900
H	-5.04702200	0.14581700	-3.30643300
H	-4.20977500	-0.93810400	-4.41590400
C	-3.93039000	-1.42030900	-2.32371400
H	-4.80187700	-2.07382500	-2.22668800
H	-3.06618600	-2.06323300	-2.51997700

C	-3.72653800	-0.70948500	-0.99244500
H	-4.60490700	-0.12172600	-0.70899300
H	-3.50058800	-1.41670300	-0.18919600
C	-3.34191500	1.82749500	0.74772800
C	-4.38644600	1.35858400	1.54964800
H	-4.39345700	0.31598100	1.86007700
C	-5.40206300	2.21025800	1.97488100
H	-6.19936000	1.82906500	2.60436900
C	-5.38271400	3.55070000	1.60248900
H	-6.16910700	4.22012100	1.93536500
C	-4.34074300	4.03405200	0.81480900
H	-4.31167600	5.08216300	0.53528800
C	-3.32446400	3.18114300	0.39579700
H	-2.50367300	3.57619300	-0.19505400
C	1.82900200	0.85240900	0.42152200
C	2.85032900	0.00239000	0.84193900
F	2.63693800	-1.31788800	0.96039100
C	4.13841700	0.42509300	1.14119600
F	5.06666400	-0.44521700	1.54122200
C	4.46482300	1.76647800	1.01334500
F	5.69603000	2.19309300	1.29321400
C	3.49192000	2.65590400	0.58854700
F	3.78787300	3.95060000	0.44295500
C	2.21678200	2.18295400	0.30325300
F	1.35186600	3.11050600	-0.16386000
C	-0.05844700	-1.12384200	0.52648900
C	-0.70713800	-2.17424000	-0.10772000
F	-0.97028400	-2.12203500	-1.44858900
C	-1.19436400	-3.31819400	0.51779500
F	-1.83500400	-4.25416700	-0.18759200
C	-1.03500100	-3.45323800	1.88494800
F	-1.49658200	-4.52756500	2.52014100
C	-0.40181600	-2.43360700	2.58664600
F	-0.25495900	-2.53975800	3.90567000
C	0.05029700	-1.31037300	1.90867900
F	0.61240200	-0.36036600	2.66632800
C	4.41524000	-1.46406500	-2.80233400
C	4.26043800	-0.08027700	-2.79373600
C	3.01912800	0.48121600	-2.51322900
C	1.90669700	-0.31805300	-2.22430000
C	2.07645600	-1.70612600	-2.24875900
C	3.31494800	-2.27320700	-2.53501100
H	5.38305300	-1.90695900	-3.01447900
H	5.10861900	0.56469100	-3.00339900
H	2.90701900	1.56319400	-2.49748200
H	1.22872800	-2.35018000	-2.03724200
H	3.42189600	-3.35381700	-2.54106300
C	0.60023300	0.31262500	-1.80788600
H	0.61872700	1.36783900	-2.11684000
H	-0.20288300	-0.16242100	-2.38801500

N14_TS

B	-0.35750300	0.16894000	0.10302300
N	2.25489900	-0.87876700	-0.55589100
H	0.80642600	-0.72869900	-0.96555300
C	2.14952800	-0.11930600	0.73144100
H	2.27378700	0.92990600	0.43809500
C	0.71821900	-0.29911500	1.22590200
H	0.56496100	0.32450800	2.11490000
H	0.56382600	-1.32663600	1.56104200
C	2.47335300	-2.33073700	-0.36882200
H	1.68831800	-2.71447900	0.28986900
H	3.44007800	-2.48964700	0.13150500
C	2.46001500	-3.07541700	-1.70126000
H	2.64229000	-4.13623700	-1.50437100
H	1.46595600	-3.00551600	-2.15673000
C	3.51187200	-2.50601400	-2.65152400
H	4.51327800	-2.69029000	-2.23999800
H	3.46595700	-3.00607800	-3.62331900
C	3.30417200	-1.00062800	-2.80731100
H	4.09781800	-0.55437300	-3.41444900
H	2.35833100	-0.80057800	-3.32525300
C	3.28923700	-0.31563200	-1.44581800
H	3.09072200	0.75342200	-1.55709200
H	4.27434800	-0.43360200	-0.96571600
C	3.25290800	-0.42662200	1.72812900
C	4.42468500	0.33560900	1.71040200
H	4.51432600	1.15508100	1.00083900
C	5.46554800	0.07541200	2.59731300
H	6.36370700	0.68405500	2.57088000
C	5.34642200	-0.95628100	3.52331500
H	6.15252600	-1.15975800	4.22073700
C	4.18125800	-1.71828500	3.55849800
H	4.07631300	-2.51727900	4.28546200
C	3.14346000	-1.45377000	2.67018400
H	2.23688400	-2.04972900	2.71856600
C	-1.92450700	-0.00969600	0.50008600
C	-2.93092200	0.57518800	-0.27019800

F	-2.61534200	1.24562300	-1.39337700
C	-4.28398500	0.50301600	0.02763000
F	-5.18697900	1.08118700	-0.76158800
C	-4.68956700	-0.17200000	1.16921300
F	-5.97879500	-0.25771100	1.47851300
C	-3.73302100	-0.76662800	1.97631200
F	-4.10487900	-1.42215000	3.07457300
C	-2.38939700	-0.66781300	1.63634900
F	-1.54203800	-1.28489900	2.47610300
C	-0.04693500	1.71256500	-0.33071500
C	0.56667400	2.18255800	-1.48526400
F	0.96804900	1.33137100	-2.45339800
C	0.83237200	3.52529800	-1.73046800
F	1.42882800	3.89909000	-2.86176400
C	0.47720500	4.47373200	-0.78524800
F	0.72317500	5.76285900	-0.99652100
C	-0.12870500	4.05886900	0.39252700
F	-0.46660600	4.95523600	1.31600600
C	-0.36965500	2.70673100	0.59363800
F	-0.94010500	2.37090400	1.76043600
C	-1.99023100	-4.89268600	-1.46933100
C	-1.84318900	-4.19272300	-2.66460800
C	-1.31691800	-2.90651800	-2.65663500
C	-0.92673600	-2.28544100	-1.46304800
C	-1.08420300	-2.99854200	-0.27260200
C	-1.60994400	-4.28969600	-0.27607400
H	-2.39920900	-5.89775800	-1.46958400
H	-2.13857000	-4.64862300	-3.60442100
H	-1.20304400	-2.36689100	-3.59408300
H	-0.81358600	-2.54618100	0.67449800
H	-1.72425900	-4.82058400	0.66381500
C	-0.34479500	-0.89158900	-1.55087700
H	0.31678900	-0.85862000	-2.42832000
H	-1.09784000	-0.16536700	-1.86286500

N14_P

B	-0.57808600	-0.16370000	-0.23015600
N	2.45693400	-0.02562300	-0.41365700
H	1.68741100	0.20068500	-1.05819100
C	1.82349500	0.06376300	0.98592200
H	1.67201400	1.14238900	1.10242600
C	0.45799100	-0.61531000	0.99119600
H	0.02242900	-0.33524500	1.95598600
H	0.57949800	-1.70234700	1.03324400
C	2.92416200	-1.40125300	-0.78830600
H	2.08648900	-2.08309400	-0.62142300
H	3.73968300	-1.64899300	-0.10330500
C	3.39279700	-1.42909700	-2.23657500
H	3.72060500	-2.44732500	-2.46393800
H	2.54207800	-1.22048500	-2.89623700
C	4.50366900	-0.40767500	-2.47654800
H	5.38752400	-0.67829100	-1.88476800
H	4.80565900	-0.41130500	-3.52693800
C	4.02048000	0.98469000	-2.07081700
H	4.82064200	1.72453900	-2.16196600
H	3.20422200	1.30996000	-2.72529400
C	3.52826200	0.99721800	-0.62948800
H	3.09491600	1.96499400	-0.36032000
H	4.33019900	0.76033300	0.07679500
C	2.81046800	-0.38170000	2.04617400
C	3.58908500	0.57462500	2.70432700
H	3.46956700	1.62572000	2.45064800
C	4.49612300	0.20387900	3.69309100
H	5.08454000	0.96274300	4.19829000
C	4.63475600	-1.13652700	4.03822800
H	5.33666200	-1.43092100	4.81165200
C	3.85681400	-2.09851200	3.39816700
H	3.94967300	-3.14397200	3.67348000
C	2.94732600	-1.72432300	2.41456000
H	2.33254300	-2.48280200	1.93913700
C	-2.16282800	-0.41012600	0.17072100
C	-3.15669700	0.17249800	-0.61550600
F	-2.81598100	0.91813300	-1.68333500
C	-4.51852500	0.04134700	-0.38057600
F	-5.41026400	0.63408900	-1.17608700
C	-4.95016900	-0.71326000	0.69990200
F	-6.25106900	-0.85820400	0.94675400
C	-4.00768900	-1.32362300	1.51153000
F	-4.40368700	-2.06496800	2.54820500
C	-2.65566600	-1.15332600	1.23710800
F	-1.81544500	-1.80453800	2.06860400
C	-0.40039300	1.49827700	-0.35641000
C	0.24935000	2.22032100	-1.35059200
F	0.84895800	1.58444800	-2.39499100
C	0.41358700	3.60214900	-1.35763500
F	1.07154800	4.20173300	-2.35205100
C	-0.10352600	4.34840200	-0.31318200
F	0.03948100	5.67107000	-0.28825100

C	-0.74900200	3.68777000	0.72395100
F	-1.22652800	4.38638500	1.75215400
C	-0.87308100	2.30476700	0.68455800
F	-1.46435500	1.74723900	1.75195900
C	0.15261300	-5.26716500	-1.79828600
C	0.56880400	-4.46165400	-2.85572800
C	0.38565300	-3.08460200	-2.79377000
C	-0.20843200	-2.46173400	-1.68427000
C	-0.63127000	-3.29218600	-0.63907600
C	-0.45208200	-4.67430300	-0.69555000
H	0.29282100	-6.34265000	-1.83750700
H	1.03040500	-4.90650200	-3.73243200
H	0.69550300	-2.46797700	-3.63553500
H	-1.09758000	-2.86412700	0.23953300
H	-0.79049200	-5.28830200	0.13371400
C	-0.34510900	-0.94913100	-1.67900100
H	0.49843000	-0.57700500	-2.27382700
H	-1.20324800	-0.68584000	-2.31222800

N15_TS

N	2.37549200	-0.19338500	-0.77524400
B	-0.39630900	0.00649100	0.00631200
C	2.12019600	0.25410800	0.63247900
H	1.98119800	1.33881400	0.55428000
C	0.78548100	-0.35590300	1.05295100
H	0.51263900	0.03257300	2.03997900
H	0.88573500	-1.43812300	1.17679400
C	2.95933700	-1.55359300	-0.90392300
C	4.48262200	-1.67120100	-0.83588000
H	4.97094300	-1.14599100	-1.66073600
H	4.74638100	-2.72868500	-0.92251800
C	3.11995900	0.48782200	-3.07104000
H	2.11206600	0.41899200	-3.48427700
H	3.64785100	-0.44513000	-3.28205300
C	3.08536400	0.81471100	-1.58333100
C	3.28155500	0.02162600	1.58044900
C	4.31517500	0.96103100	1.64906300
H	4.23775300	1.88148500	1.07479300
C	5.43689500	0.73524500	2.44085500
H	6.23053100	1.47475000	2.47878800
C	5.53534900	-0.43482200	3.18973300
H	6.40896200	-0.61581600	3.80758300
C	4.49743400	-1.36191000	3.15632200
H	4.55695000	-2.26672400	3.75279300
C	3.37756300	-1.13207200	2.36254600
H	2.57397400	-1.86165400	2.35367700
C	-1.88250400	-0.53736300	0.39579200
C	-3.00828200	-0.13866500	-0.32707000
F	-2.86421600	0.61303000	-1.43301600
C	-4.31126800	-0.48285200	0.00388300
F	-5.33419200	-0.06859600	-0.74050900
C	-4.54032300	-1.25607600	1.13206300
F	-5.77766800	-1.59672700	1.47500000
C	-3.46031400	-1.68098200	1.88991600
F	-3.66222700	-2.43224900	2.97048000
C	-2.17376500	-1.31126800	1.51744800
F	-1.19090000	-1.78628300	2.29977500
C	-0.46984800	1.61618700	-0.25993200
C	-0.14293000	2.34221800	-1.39875500
F	0.31794900	1.72785500	-2.50632800
C	-0.24385000	3.72628300	-1.49046000
F	0.09080500	4.35367500	-2.61712700
C	-0.69872200	4.45152900	-0.40155500
F	-0.80621300	5.77422300	-0.47051600
C	-1.03466800	3.77847000	0.76477000
F	-1.47010700	4.45921300	1.82193400
C	-0.91204800	2.39668900	0.81059200
F	-1.24719100	1.80834500	1.96929000
H	4.89452700	-1.29574600	0.10178600
H	3.63901700	1.29039000	-3.60046500
H	2.60745600	-1.96329500	-1.85683900
H	2.49954900	-2.17502100	-0.12986300
H	2.56283100	1.76517100	-1.43946500
H	4.10903800	0.94567300	-1.20429000
H	0.91898300	-0.40570200	-1.15166800
C	0.04764500	-5.21016000	-1.97514900
C	0.26983400	-4.41421200	-3.09562000
C	0.20701300	-3.02971800	-2.98762200
C	-0.08050000	-2.40180000	-1.76853800
C	-0.30047000	-3.21497300	-0.65232000
C	-0.23649700	-4.60341600	-0.75690500
H	0.09676300	-6.29147500	-2.05147400
H	0.49220200	-4.87053900	-4.05514800
H	0.38549000	-2.41575700	-3.86781200
H	-0.51823100	-2.77814000	0.31389100
H	-0.41181800	-5.21072500	0.12570300
C	-0.11959100	-0.88987000	-1.74369000
H	0.39352800	-0.52373000	-2.64088700

H -1.12063600 -0.49711200 -1.92069900

N15_P

N	-2.64115700	-0.22771700	0.56331800
B	0.63760700	-0.05926100	0.30104600
C	-1.90868200	0.30299900	-0.68712100
H	-1.75795700	1.36354900	-0.46313100
C	-0.55968500	-0.39511400	-0.80263700
H	-0.16962500	-0.10595100	-1.78671000
H	-0.72335200	-1.47370800	-0.87772800
C	-3.17800800	-1.63971800	0.41465700
C	-4.62733700	-1.74702700	-0.03457500
H	-5.32721900	-1.39766500	0.72845200
H	-4.83149800	-2.80777100	-0.19905200
C	-3.95145200	0.39655200	2.58000100
H	-4.68950500	1.10317100	2.96460200
H	-3.04394600	0.49577600	3.18253400
C	-3.65214600	0.72333100	1.12690700
C	-2.82481500	0.20221600	-1.89013000
C	-3.78944200	1.19098400	-2.10804300
H	-3.82582200	2.05768200	-1.45204200
C	-4.69420400	1.08630200	-3.15996800
H	-5.43729900	1.86183200	-3.31412100
C	-4.63353400	-0.00694200	-4.02029500
H	-5.33596500	-0.09192900	-4.84290300
C	-3.65192900	-0.97751400	-3.83564900
H	-3.58334700	-1.81744400	-4.51920600
C	-2.74953500	-0.87164600	-2.78094300
H	-1.97549600	-1.62333100	-2.65830500
C	2.05081900	-0.76316900	-0.21220700
C	3.24877200	-0.40299000	0.40945900
F	3.23946200	0.54964700	1.35861400
C	4.48737400	-0.95001400	0.11648900
F	5.58629200	-0.54018000	0.75193400
C	4.57918500	-1.93767800	-0.85498900
F	5.75532200	-2.48692000	-1.15377400
C	3.42569500	-2.34932900	-1.49713100
F	3.48413500	-3.31133400	-2.42135700
C	2.20731300	-1.75950300	-1.16957300
F	1.14691400	-2.27077100	-1.83625400
C	0.78373400	1.59852900	0.33222500
C	-0.08120800	2.39891300	1.07193500
F	-1.11272200	1.83841300	1.77096600
C	-0.03974100	3.78479200	1.13945000
F	-0.92966400	4.45829200	1.87394100
C	0.92601300	4.46149800	0.41378400
F	0.99542300	5.79058600	0.45077600
C	1.80406200	3.72756800	-0.36936700
F	2.72347700	4.36186300	-1.09572300
C	1.70949400	2.34042800	-0.40389400
F	2.57952800	1.73528500	-1.22341200
H	-4.82530700	-1.22066300	-0.97020900
H	-4.34646000	-0.61434800	2.71043700
H	-3.03607700	-2.12477900	1.38204800
H	-2.51608300	-2.13997800	-0.29088100
H	-3.21828700	1.71947300	1.04906200
H	-4.53711400	0.67856700	0.49174100
H	-1.88501000	-0.28748900	1.25980700
C	-1.77189100	-4.27641100	2.59927400
C	-1.89742800	-3.18921400	3.46002000
C	-1.17963000	-2.01933500	3.21264600
C	-0.31891100	-1.89765200	2.11075400
C	-0.19933400	-3.00989800	1.26313600
C	-0.91423200	-4.18025700	1.50383400
H	-2.32463200	-5.19172300	2.78503400
H	-2.54760200	-3.25039100	4.32804400
H	-1.26548100	-1.18209600	3.90271400
H	0.45541300	-2.95388500	0.39850600
H	-0.79896700	-5.02486100	0.83093800
C	0.43384400	-0.60930100	1.87512500
H	-0.01009000	0.14622700	2.53091200
H	1.44432600	-0.74026200	2.28054800

N16_TS

N	-2.36024100	0.81717500	-0.54891900
H	-0.91572500	0.97458700	-0.85730400
B	0.38812000	-0.00263700	0.19967100
C	-2.16209000	0.01900700	0.70103200
H	-2.05549000	-1.00230200	0.32330800
C	-0.77053900	0.31881300	1.35034500
C	-2.98175800	2.15524600	-0.38604700
H	-2.42948600	2.72171500	0.36158300
H	-4.00950000	2.02318700	-0.01912200
C	-3.02623800	2.91791800	-1.70698800
H	-2.00616500	3.08937600	-2.06729200
H	-3.46395600	3.90327300	-1.51916500
C	-3.83706200	2.14788300	-2.74544900
H	-4.88457700	2.09655200	-2.42044500

H	-3.82404200	2.66088300	-3.71152400
C	-3.27830500	0.73331400	-2.87510000
H	-3.90447000	0.11954300	-3.52999700
H	-2.28125400	0.76448800	-3.32839500
C	-3.19122100	0.05182900	-1.51394600
H	-4.19975400	-0.05591200	-1.08319500
H	-2.76967600	-0.94860700	-1.61713800
C	-3.37935900	-0.05601300	1.61996400
C	-3.98182500	-1.31051700	1.77789900
H	-3.56957000	-2.16330600	1.24331300
C	-5.08395600	-1.49616200	2.60646400
H	-5.52197900	-2.48392400	2.70800400
C	-5.61885000	-0.41675700	3.30074800
H	-6.47795000	-0.55227300	3.94974900
C	-5.04072600	0.84043500	3.15466500
H	-5.44971300	1.69237000	3.68858500
C	-3.93598200	1.01988700	2.32684700
H	-3.52006500	2.01537000	2.23480200
C	2.00798900	0.12010000	0.52400100
C	2.93108100	-0.28048500	-0.45421300
F	2.51742200	-0.68032200	-1.67251500
C	4.30859900	-0.30855800	-0.28955500
F	5.09803600	-0.69698600	-1.28816900
C	4.85686100	0.04972900	0.93050300
F	6.17089100	0.02334800	1.11919800
C	4.00121900	0.43446500	1.94684300
F	4.49255800	0.77919300	3.13534200
C	2.62720100	0.45271800	1.73036300
F	1.92346900	0.84518000	2.80384400
C	0.20809700	-1.50325700	-0.46144400
C	-0.30998600	-1.85056000	-1.70554100
F	-0.72434500	-0.90519900	-2.57247900
C	-0.43946800	-3.15525600	-2.16687900
F	-0.97347000	-3.39615700	-3.36339900
C	-0.00241300	-4.20536000	-1.37677700
F	-0.11950500	-5.46121700	-1.79458200
C	0.56309000	-3.92100800	-0.14360600
F	1.01028200	-4.91092400	0.62530800
C	0.66586900	-2.60042000	0.27555200
F	1.26601800	-2.42122500	1.46139700
C	-0.66822800	-0.66466900	2.53628000
H	-1.37677200	-0.37204200	3.31888200
H	-0.91957400	-1.68869800	2.24125900
H	0.32951600	-0.67289700	2.97055600

C	-0.71170500	1.74352200	1.92783300
H	0.18982400	1.88681100	2.51732600
H	-0.71376700	2.51849500	1.16089900
H	-1.55091400	1.91586700	2.60754500
C	1.62247600	5.42058600	-1.05721900
C	0.97189300	4.94777900	-2.19204900
C	0.53635800	3.62767600	-2.24467000
C	0.72395100	2.74346100	-1.17257900
C	1.39112800	3.23649400	-0.04578900
C	1.83173900	4.55587700	0.01211300
H	1.96591300	6.44870800	-1.00794500
H	0.80637200	5.60295800	-3.04159700
H	0.04934100	3.26552300	-3.14792500
H	1.56365600	2.59315700	0.80664100
H	2.34234400	4.90592400	0.90384700
C	0.25790600	1.31218300	-1.34598100
H	-0.36139200	1.28808700	-2.25390600
H	1.07325700	0.68542400	-1.69850000

N16_P

N	2.44535300	-0.15022500	0.01096900
H	1.79005300	-0.53704800	-0.68747400
B	-0.75767100	-0.44327700	0.07085200
C	1.57831900	0.33324300	1.20355600
H	1.26095200	1.31509800	0.84652300
C	0.23467100	-0.42731500	1.44745500
C	3.47390500	-1.21120400	0.30208000
H	2.95823200	-2.06938200	0.72773200
H	4.14618500	-0.78587800	1.04995400
C	4.23269400	-1.60801700	-0.95558000
H	3.53394400	-2.04053200	-1.67566600
H	4.93599900	-2.39920100	-0.67914800
C	4.94515000	-0.41443500	-1.58257200
H	5.71161900	-0.02777000	-0.89845800
H	5.45182200	-0.71398500	-2.50347600
C	3.91163000	0.67016900	-1.87025300
H	4.37791200	1.58402600	-2.24980500
H	3.20477700	0.32654100	-2.63277800
C	3.14192300	1.03616300	-0.61033700
H	3.80900800	1.42004100	0.16871300
H	2.38022500	1.78852800	-0.81432700
C	2.46391200	0.60670000	2.41027700
C	2.73961700	1.94035700	2.72924400
H	2.33075100	2.72987100	2.10283900

C	3.50706300	2.27690000	3.84115800
H	3.70014400	3.32031200	4.06729000
C	4.01532500	1.27532600	4.66050800
H	4.61139800	1.52981300	5.53056600
C	3.74822600	-0.05831700	4.36017100
H	4.13544500	-0.84696400	4.99698800
C	2.97886900	-0.39069600	3.24988500
H	2.78057200	-1.43529600	3.04153100
C	-2.39572700	-0.68262100	0.38574000
C	-3.30711400	-0.35703100	-0.62791000
F	-2.88063000	0.18706800	-1.78524500
C	-4.67827300	-0.56101900	-0.56668100
F	-5.46325000	-0.20698900	-1.58524500
C	-5.23102200	-1.15020800	0.55914600
F	-6.54298000	-1.36083500	0.64454200
C	-4.38389000	-1.52319000	1.58555500
F	-4.87959500	-2.10796900	2.67776800
C	-3.01371800	-1.29888900	1.47324400
F	-2.31277800	-1.73964000	2.53878000
C	-0.68024000	1.07994300	-0.61436800
C	0.10193400	1.40533600	-1.71083600
F	0.88704900	0.45397500	-2.28016900
C	0.21567000	2.66603500	-2.28369900
F	1.04958900	2.87223500	-3.30696900
C	-0.52476800	3.70940100	-1.75529100
F	-0.43934400	4.93387800	-2.26896700
C	-1.34621600	3.45308400	-0.66615200
F	-2.06834900	4.44207200	-0.14242900
C	-1.40771700	2.17188400	-0.12890700
F	-2.24178500	2.03585400	0.91176700
C	-0.40212200	0.45395500	2.55482400
H	0.13885400	0.34489000	3.50132700
H	-0.37818100	1.51533600	2.28658100
H	-1.44055500	0.19327200	2.73436900
C	0.44502200	-1.85445200	1.99449000
H	-0.42974000	-2.47657900	1.80959400
H	1.28655300	-2.36182700	1.52090100
H	0.61515900	-1.85881200	3.07666200
C	2.62586700	-4.09209200	-2.82118500
C	2.06910800	-3.02210100	-3.51718000
C	1.10615700	-2.21227100	-2.91719900
C	0.65736300	-2.44140600	-1.60435300
C	1.23500900	-3.52977100	-0.92651900
C	2.20368000	-4.33842500	-1.51667400
H	3.36453900	-4.73257300	-3.29198200
H	2.36570600	-2.82715400	-4.54349900
H	0.64912800	-1.41769700	-3.49656900
H	0.86096100	-3.78943600	0.05949600
H	2.60409400	-5.18416900	-0.96533800
C	-0.55452200	-1.72583400	-1.03244900
H	-1.16659200	-1.42702300	-1.89131300
H	-1.12414000	-2.53048700	-0.55190100

N19_TS

B	0.49180300	-0.11431300	0.23401800
C	-0.15334400	-0.33504200	1.70691900
C	0.45956000	-1.21166500	2.61307900
H	1.30631200	-1.80724600	2.28267100
C	0.05484300	-1.34146200	3.93933500
H	0.57088200	-2.03495800	4.59576800
C	-0.98067800	-0.55332100	4.42149300
H	-1.28394300	-0.60677400	5.46214400
C	-1.63916300	0.29577000	3.53939000
H	-2.47537600	0.89409900	3.89471900
C	-1.27317300	0.37390500	2.19605800
C	-2.12186900	1.24508600	1.30379400
H	-1.50142000	2.01504300	0.85047000
H	-2.86792600	1.76489900	1.91065700
N	-2.78265700	0.51100100	0.18347100
C	-3.83572800	-0.50369200	0.67259400
C	-4.55611500	-1.12024600	-0.53910900
H	-3.84662300	-1.76512800	-1.07024500
H	-5.33794200	-1.77704300	-0.14410000
C	-5.13665000	-0.11057600	-1.51121700
H	-5.92133300	0.49094100	-1.03785900
H	-5.60628100	-0.62565400	-2.35469300
C	-4.00219300	0.77034900	-2.00110400
H	-4.35642000	1.52099100	-2.71553300
H	-3.27487400	0.14375900	-2.53528800
C	-3.27429100	1.51562500	-0.87104200
C	-2.09286400	2.24847300	-1.53282400
H	-1.36434000	1.56765900	-1.97017900
H	-1.56744800	2.93285700	-0.86280700
H	-2.50139100	2.85219400	-2.34777200
C	-4.18786600	2.61253500	-0.29468500
H	-3.70962500	3.15180700	0.52599700
H	-5.15345200	2.24709000	0.04833100
H	-4.38078500	3.34183900	-1.08681400

C	-4.87715500	0.09039900	1.63602600
H	-4.41527700	0.58672200	2.49220700
H	-5.46041400	-0.74447700	2.03527900
H	-5.57934700	0.77834500	1.16777900
C	-3.18201600	-1.68791300	1.41247600
H	-2.24960200	-2.00339600	0.95210100
H	-3.87631200	-2.53221600	1.35790800
H	-2.99325900	-1.48047400	2.46407300
C	1.98171900	-0.82649900	0.09282100
C	2.47744500	-1.73974900	-0.83577700
F	1.70433400	-2.24337100	-1.81073300
C	3.79397900	-2.19420600	-0.84798800
F	4.18713400	-3.07840800	-1.76286600
C	4.69719400	-1.71602700	0.08472500
F	5.95644300	-2.14187000	0.08739000
C	4.26835500	-0.77795000	1.01335200
F	5.12288100	-0.29091100	1.91144100
C	2.95011900	-0.35014500	0.98555500
F	2.61674200	0.58001100	1.89133600
C	0.79107600	1.43143300	-0.23906300
C	0.85508800	2.52888700	0.62193800
F	0.54777300	2.41822600	1.91822500
C	1.20682600	3.81215300	0.21210900
F	1.22323200	4.81636400	1.08637100
C	1.55884200	4.04263000	-1.10783500
F	1.89924400	5.26099000	-1.51347600
C	1.56081900	2.97803600	-1.99682400
F	1.91642200	3.16759100	-3.26596800
C	1.20710100	1.71674100	-1.54127900
F	1.26842200	0.73535900	-2.46118700
H	-1.64234200	-0.19476100	-0.36543000
C	-1.64550100	-5.09991500	-1.26505800
C	-2.17478300	-4.22141800	-2.20441000
C	-1.86804900	-2.86428200	-2.14068600
C	-1.04310300	-2.35113100	-1.13543700
C	-0.50646300	-3.25065700	-0.20678900
C	-0.80495900	-4.60716300	-0.26992600
H	-1.87923300	-6.15846600	-1.31112400
H	-2.82082600	-4.59025000	-2.99506900
H	-2.27164400	-2.18815500	-2.89229400
H	0.14050800	-2.88169000	0.58296600
H	-0.37835400	-5.28343300	0.46422800
C	-0.73208200	-0.87791500	-1.09987400
H	0.28957100	-0.77522600	-1.48058500
H	-1.20506700	-0.38647100	-1.96109800

N19_P

B	0.83015200	-0.54640500	-0.56330700
C	0.00423000	-1.58660100	0.42754100
C	0.38045800	-2.93268300	0.23871100
H	1.02850900	-3.16898600	-0.60246000
C	0.06504800	-3.95614300	1.11947100
H	0.41380200	-4.96625700	0.92638000
C	-0.62719500	-3.66225100	2.29250500
H	-0.80448800	-4.42262100	3.04579000
C	-1.10342100	-2.37353900	2.46353200
H	-1.67142900	-2.13397900	3.35977200
C	-0.88077300	-1.37091800	1.50526700
C	-1.64676400	-0.08968800	1.70581900
H	-1.02371800	0.78133800	1.53812500
H	-2.04838100	-0.03716500	2.71731200
N	-2.85184900	0.11453600	0.76537900
C	-3.96291200	-0.98912900	0.85331700
C	-5.17476200	-0.56928500	0.00306900
H	-4.89972700	-0.65742100	-1.05486900
H	-5.94688400	-1.32216500	0.19002700
C	-5.69197800	0.83171600	0.26677800
H	-6.03732600	0.95087500	1.30035400
H	-6.55262700	1.03875300	-0.37534900
C	-4.56405700	1.79375400	-0.04950200
H	-4.87378800	2.83670900	0.06825200
H	-4.27289300	1.66592100	-1.10088000
C	-3.31727800	1.60340300	0.82374600
C	-2.22388000	2.48576100	0.21745400
H	-1.92939000	2.12689100	-0.77202100
H	-1.33290000	2.57510800	0.83903900
H	-2.63920900	3.48964600	0.09477100
C	-3.58064600	2.04749400	2.26337600
H	-2.67229300	2.03862500	2.86773200
H	-4.34597700	1.45954500	2.76893000
H	-3.93129800	3.08222600	2.22530100
C	-4.40370200	-1.24574200	2.29254000
H	-3.57373400	-1.50638300	2.94609000
H	-5.07059100	-2.11187400	2.26657400
H	-4.96200800	-0.41850900	2.73194300
C	-3.42608000	-2.28078200	0.21826700
H	-2.83698300	-2.06931200	-0.67662900
H	-4.29078900	-2.87389900	-0.09457800

H	-2.82304300	-2.88242900	0.89366400
C	2.46534100	-0.80839400	-0.30830900
C	3.39212200	-0.31893900	-1.23125700
F	2.97472300	0.36498000	-2.31266400
C	4.76907000	-0.44768400	-1.10998300
F	5.58575500	0.03811600	-2.04778100
C	5.29432000	-1.07623800	0.00801700
F	6.61176700	-1.21534200	0.15157500
C	4.42607200	-1.54268700	0.98057900
F	4.91561200	-2.12621300	2.07750600
C	3.05165500	-1.38894900	0.81881400
F	2.31857400	-1.83490400	1.84840000
C	0.78528800	1.08474400	-0.21859600
C	1.12801500	1.51173800	1.07006100
F	1.25962200	0.60503000	2.05351300
C	1.32679800	2.83256000	1.44612900
F	1.63645100	3.15118900	2.70361400
C	1.21886700	3.83159300	0.48647200
F	1.40516200	5.10738400	0.81620600
C	0.90787000	3.47318000	-0.81346200
F	0.79156000	4.41462300	-1.75111100
C	0.71482200	2.13054400	-1.13511400
F	0.39365300	1.92209400	-2.42475800
H	-2.47333800	-0.00780900	-0.18969100
C	-3.62106800	-1.67979400	-3.32819800
C	-2.72897500	-2.73779000	-3.16625100
C	-1.42137300	-2.49872900	-2.75275100
C	-0.95251600	-1.20091600	-2.49762800
C	-1.86994100	-0.14746600	-2.65465400
C	-3.18181100	-0.38293200	-3.07024200
H	-4.63635800	-1.86051900	-3.66737700
H	-3.05043100	-3.75490400	-3.37061300
H	-0.73205900	-3.33156600	-2.64267300
H	-1.51496000	0.87249900	-2.54138400
H	-3.85389900	0.45692900	-3.23305600
C	0.48482000	-0.95263800	-2.14006000
H	1.03869900	-1.87437400	-2.35612400
H	0.88179600	-0.20669900	-2.82670100

N20_TS

B	0.91011300	0.20404300	-0.40990800
C	-0.19245900	0.29339500	-1.60929400
C	0.15294900	-0.25540600	-2.85326500
H	1.13166700	-0.71276300	-2.96465500
C	-0.70049800	-0.23791700	-3.95088100
H	-0.38299600	-0.68182100	-4.88951100
C	-1.95403200	0.35115500	-3.83917300
H	-2.63505600	0.37648400	-4.68361400
C	-2.32435700	0.91482500	-2.62497500
H	-3.30693600	1.37172700	-2.52576500
C	-1.47456000	0.89511300	-1.51218200
C	-2.04906900	1.53082500	-0.25152000
H	-2.78379000	2.28280700	-0.55420700
H	-1.28571600	2.05633500	0.32700600
N	-2.73372000	0.53275700	0.65989400
C	-3.20936400	1.12898000	1.98603600
C	-4.08147500	2.35863400	1.71189700
H	-4.57432300	2.62804600	2.65117200
H	-3.43020100	3.20197900	1.46077800
C	-5.12056700	2.13750900	0.60066000
C	-5.06010800	0.78183800	-0.06951200
C	-6.20704300	0.32408400	-0.71465700
C	-6.21302600	-0.90553400	-1.35277000
H	-7.10713500	-1.26096900	-1.85454200
C	-5.06185900	-1.68011300	-1.34427200
H	-5.06714300	-2.63616700	-1.85417100
C	-3.88963600	-1.26254500	-0.70658200
C	-3.90526700	-0.01800500	-0.05415600
C	-2.66772700	-2.16107200	-0.74686500
H	-1.80164500	-1.51200700	-0.86778100
C	-2.63842200	-3.10225200	-1.95426600
H	-1.65029900	-3.56191200	-2.02238700
H	-2.82528600	-2.55620100	-2.88321400
H	-3.37331600	-3.90902200	-1.86376100
C	-2.53210400	-2.96393900	0.55155600
H	-1.68925200	-3.65773300	0.47694300
H	-3.44037600	-3.54904600	0.73008500
H	-2.36775300	-2.32126700	1.42300200
C	-2.04269400	1.55332600	2.87883700
H	-1.39891700	2.29930100	2.41461300
H	-1.43718200	0.70243600	3.19936000
H	-2.46772100	2.01256600	3.77595000
C	-3.98485600	0.04121200	2.74163700
H	-3.38271700	-0.86994700	2.81516600
H	-4.93873000	-0.21184700	2.27717300
H	-4.18119800	0.39587400	3.75689800
C	1.98622900	-1.00908300	-0.48956400
C	3.34494200	-0.90535800	-0.18675600

F	3.90251900	0.26903800	0.13113500
C	4.21808500	-1.98852900	-0.17569400
F	5.50547000	-1.81145200	0.11521400
C	3.74797100	-3.25911400	-0.46336900
F	4.56896500	-4.30458300	-0.45015000
C	2.40260200	-3.42481400	-0.75301700
F	1.91249100	-4.63921300	-1.00295200
C	1.57451700	-2.31391200	-0.76024200
F	0.26817500	-2.57312300	-0.98802200
C	1.60634300	1.68478200	-0.28558500
C	1.42362800	2.69102500	0.64821000
F	0.60207000	2.50698900	1.71136500
C	2.02096200	3.94612600	0.58637800
F	1.77962700	4.85453600	1.53172900
C	2.86172500	4.24281800	-0.47057400
F	3.44779800	5.43299000	-0.55572600
C	3.08433100	3.27544000	-1.44307400
F	3.89625500	3.54015900	-2.46420200
C	2.45866000	2.04515700	-1.33373500
F	2.73663200	1.15101300	-2.29230000
H	-7.09808800	0.94619100	-0.70754800
H	-6.13276600	2.27106500	0.99244200
H	-5.00145200	2.90912100	-0.17013600
H	-1.54460900	-0.17290400	1.04488800
C	1.83775800	-2.18987600	4.39896000
C	1.72684800	-0.80448400	4.32621600
C	1.07253700	-0.20338100	3.25484600
C	0.50743400	-0.97962400	2.23519500
C	0.63218800	-2.37303600	2.31654700
C	1.28915500	-2.97169800	3.38567700
H	2.35142900	-2.65631800	5.23302800
H	2.15881700	-0.18275900	5.10431900
H	1.00815800	0.87849500	3.20105100
H	0.21540500	-2.98846000	1.52367000
H	1.37517700	-4.05322900	3.42448100
C	-0.17393700	-0.36813900	1.07144300
H	-0.23427900	0.71166700	1.03418000
H	-0.49114900	-1.04693600	0.29183900

N20_P

B	0.81554700	0.15159800	-0.21482900
C	-0.32813100	0.07573700	-1.41469300
C	0.00578400	-0.51107000	-2.64890400
H	0.99478200	-0.94165200	-2.76612100
C	-0.85907100	-0.55106900	-3.73621500
H	-0.53731000	-1.01921100	-4.66208800
C	-2.12525800	0.01991900	-3.64502100
H	-2.80654500	0.01010900	-4.48940400
C	-2.49358600	0.61671500	-2.44909600
H	-3.47864300	1.07161900	-2.35718200
C	-1.62352300	0.64077400	-1.34956900
C	-2.16593600	1.29460600	-0.10367200
H	-2.90383800	2.05469500	-0.36567100
H	-1.38777000	1.74019300	0.51164900
N	-2.92639700	0.29761600	0.80152100
C	-3.45553500	0.90555200	2.12594200
C	-4.37289800	2.07136500	1.74936000
H	-4.92488500	2.33735400	2.65508500
H	-3.75650500	2.94111300	1.50496700
C	-5.35347500	1.76795100	0.59458400
C	-5.21696100	0.39360000	-0.02274700
C	-6.29789600	-0.16999100	-0.69763700
C	-6.18900900	-1.43124300	-1.26651700
H	-7.03381200	-1.86627700	-1.79017000
C	-4.99868300	-2.13995600	-1.16443400
H	-4.92266800	-3.11886600	-1.62311000
C	-3.88460600	-1.61871600	-0.50109500
C	-4.03744000	-0.34618200	0.06476100
C	-2.59106300	-2.41464000	-0.42374700
H	-1.75442600	-1.71356300	-0.47238500
C	-2.40230700	-3.35143500	-1.61949500
H	-1.38412800	-3.74275500	-1.60579600
H	-2.54767100	-2.81382900	-2.56041200
H	-3.08995400	-4.20297900	-1.58284900
C	-2.48903600	-3.19913600	0.89088200
H	-1.53956100	-3.74092900	0.91864600
H	-3.30872600	-3.91975500	0.97672200
H	-2.52473100	-2.55021000	1.77391200
C	-2.30984800	1.40166000	3.00424300
H	-1.68343500	2.15017100	2.51901900
H	-1.67708400	0.58057400	3.35162200
H	-2.75943900	1.87003400	3.88430300
C	-4.20727200	-0.19193600	2.88574100
H	-3.56539100	-1.06252600	3.05257300
H	-5.11759300	-0.51880900	2.38257600
H	-4.47934200	0.20632500	3.86631600
C	2.02347200	-0.94147000	-0.47940200
C	3.38992200	-0.70219100	-0.36377200

F	3.86018600	0.53557500	-0.15531900
C	4.35909900	-1.69873800	-0.42316800
F	5.65208200	-1.39250300	-0.30880000
C	3.97914700	-3.02035100	-0.59067900
F	4.89257300	-3.98895400	-0.64852700
C	2.62938600	-3.32029600	-0.68849800
F	2.23067500	-4.58913000	-0.82204000
C	1.70553400	-2.28887800	-0.62230400
F	0.40580600	-2.67548200	-0.64469000
C	1.34733300	1.72073200	-0.32254000
C	1.04163700	2.78376400	0.51545800
F	0.29966600	2.60341000	1.64321500
C	1.42974600	4.10349300	0.30272500
F	1.08429200	5.05918500	1.17020800
C	2.16566200	4.41871100	-0.82447200
F	2.54692800	5.67333400	-1.05816200
C	2.49538700	3.40249800	-1.71181700
F	3.19654500	3.68756800	-2.80942000
C	2.07983700	2.10706700	-1.44962100
F	2.43426900	1.18765000	-2.35802800
H	-7.22707500	0.38842900	-0.76620100
H	-6.38590000	1.88170000	0.93521200
H	-5.22033900	2.51616600	-0.19590900
H	-2.23186700	-0.41800700	1.05526400
C	3.32493900	-1.36717400	4.09875900
C	3.02396300	-0.02900200	3.86075200
C	2.01062200	0.31931600	2.97206500
C	1.27337400	-0.66292900	2.30127200
C	1.57944900	-2.00389200	2.56165800
C	2.59479200	-2.35609800	3.44486000
H	4.12088800	-1.63679700	4.78544300
H	3.58482100	0.75174100	4.36579000
H	1.78619700	1.36576900	2.79335800
H	1.01807200	-2.78118000	2.04628200
H	2.81649500	-3.40444700	3.62126700
C	0.22619600	-0.30066700	1.27900700
H	-0.40174700	0.47861700	1.70489300
H	-0.39349000	-1.19839200	1.12222400

N21_TS

B	1.09936800	-0.13725100	-0.53800600
C	0.49057000	-0.92076400	-1.81821800
C	1.30246900	-1.88148400	-2.44255200
H	2.29508600	-2.07335000	-2.05128600
C	0.90827000	-2.59874200	-3.56978500
H	1.58645300	-3.32386400	-4.00828600
C	-0.34777300	-2.38776500	-4.11708700
H	-0.67893600	-2.94724500	-4.98598500
C	-1.17911700	-1.43852000	-3.53215500
H	-2.16720300	-1.25948500	-3.94815700
C	-0.76950200	-0.70491200	-2.42071500
C	-1.71468800	0.35139000	-1.92352500
H	-2.65935800	0.29109000	-2.47250800
H	-1.29496800	1.34326200	-2.09671800
N	-2.01106100	0.25240400	-0.46448700
C	-2.55611100	1.55063200	0.02777600
C	-3.97632700	1.77438800	-0.46035700
H	-4.03670100	1.73369400	-1.55484700
H	-4.30613600	2.77492200	-0.16436000
C	-4.89566100	0.72261700	0.16296600
H	-4.99462800	0.97209200	1.23053600
C	-4.26658800	-0.66153600	0.09336100
C	-5.02386200	-1.79770000	0.39921900
H	-6.06695000	-1.67602400	0.67431100
C	-4.48111600	-3.07467100	0.37494000
H	-5.10263100	-3.92914500	0.62836600
C	-3.14270300	-3.27211200	0.02821900
C	-2.37063300	-2.15129000	-0.25973600
H	-1.31877200	-2.28045900	-0.49172400
C	-2.91543400	-0.86641600	-0.21066100
C	-6.28204700	0.78027800	-0.48252500
H	-6.66031500	1.80653600	-0.46034800
H	-7.01071600	0.15080400	0.03180800
H	-6.23095200	0.45616100	-1.52662600
C	-2.53750700	-4.64981200	-0.03514300
H	-2.98003800	-5.31150200	0.71343500
H	-1.45818800	-4.61091200	0.13065200
H	-2.70532800	-5.10183700	-1.01809200
C	1.19814000	1.47811700	-0.61136400
C	0.96282400	2.23941400	-1.75761900
F	0.69652000	1.66418100	-2.93840000
C	0.97544200	3.63219500	-1.78098700
F	0.70656200	4.28645500	-2.91002300
C	1.28532200	4.33615100	-0.62987100
F	1.30068400	5.66412000	-0.63125500
C	1.60291700	3.62822200	0.52129700
F	1.93667800	4.27558900	1.63625400
C	1.58683000	2.24388200	0.49198900

F	2.00688400	1.64546000	1.61745000
C	2.40595800	-0.89596000	0.05810500
C	3.66401700	-0.31400300	0.19832900
F	3.88655700	0.95278800	-0.17227700
C	4.77106500	-0.98804000	0.70609300
F	5.94550900	-0.36998500	0.81326600
C	4.64945400	-2.31187900	1.09194400
F	5.69483000	-2.97262600	1.57733600
C	3.42199100	-2.94685600	0.95474700
F	3.29392000	-4.22666400	1.30101400
C	2.34610200	-2.23955300	0.44259500
F	1.20491500	-2.93685700	0.30272300
H	-0.88708000	-0.01409200	0.19404800
H	-1.85803000	2.32824400	-0.29653400
H	-2.55648200	1.52877900	1.11684300
C	-2.90479700	1.32620900	4.04455800
C	-1.72975000	2.00115700	3.71874000
C	-0.79108800	1.41770200	2.87353400
C	-1.00502900	0.14419800	2.32208400
C	-2.16151800	-0.54656300	2.71187500
C	-3.10477400	0.03905600	3.55205300
H	-3.64004900	1.78618900	4.69698900
H	-1.54138200	2.99033700	4.12505600
H	0.11869200	1.95846800	2.64121400
H	-2.33247200	-1.55298600	2.34093500
H	-3.99682500	-0.51871300	3.82237400
C	-0.04588000	-0.46219400	1.32585900
H	0.94703500	-0.22709600	1.68966700
H	-0.15686300	-1.54560500	1.32798800

N21_P

B	0.62349400	-0.45962900	-0.23412400
C	-0.29742400	-1.16001200	-1.43067400
C	0.15463600	-2.43592600	-1.82698500
H	1.09758400	-2.79183100	-1.41592900
C	-0.52560200	-3.26098600	-2.70816600
H	-0.10869400	-4.22733100	-2.97488600
C	-1.74355800	-2.84739700	-3.24687800
H	-2.29346100	-3.47825700	-3.93716700
C	-2.24480400	-1.61569900	-2.86561700
H	-3.20870300	-1.28574700	-3.24625600
C	-1.54120100	-0.78616000	-1.97729200
C	-2.25371000	0.47185700	-1.57081700
H	-3.21191600	0.56348800	-2.08408300
H	-1.66725200	1.37413500	-1.73875200
N	-2.58079900	0.45594000	-0.07510000
C	-2.89359600	1.82898300	0.44540100
C	-4.25499100	2.26913800	-0.05188300
H	-4.25809300	2.29429900	-1.14788400
H	-4.43118300	3.29646100	0.27833800
C	-5.37301700	1.35720500	0.45919600
H	-5.57592500	1.63336400	1.50347400
C	-4.94761100	-0.10351300	0.47256900
C	-5.87553000	-1.10601300	0.76977600
H	-6.90576000	-0.82654300	0.96568900
C	-5.51637300	-2.44612800	0.83048800
H	-6.27192400	-3.19248000	1.05771900
C	-4.19858400	-2.84862000	0.61035900
C	-3.25385500	-1.86376900	0.33150600
H	-2.21933000	-2.14312300	0.15592200
C	-3.63297400	-0.52753300	0.26215600
C	-6.64831900	1.59264800	-0.35534600
H	-6.89193800	2.65873700	-0.36891500
H	-7.50797100	1.06237200	0.05760400
H	-6.50730300	1.25903300	-1.38787100
C	-3.77856000	-4.29275900	0.66925500
H	-3.22127900	-4.56592800	-0.23100700
H	-4.64281800	-4.95369100	0.75652100
H	-3.12388400	-4.47158200	1.52727600
C	0.34716300	1.16432500	0.05152500
C	0.46244500	2.06412600	-1.01104100
F	0.58864400	1.58973400	-2.26009000
C	0.42360500	3.44567500	-0.88030800
F	0.51836900	4.24169800	-1.94457800
C	0.29494200	4.00044500	0.38884000
F	0.26121700	5.32092800	0.54428700
C	0.19479000	3.15852800	1.48435800
F	0.05572500	3.67323300	2.70626800
C	0.22419900	1.77780500	1.29535600
F	0.08045600	1.04998100	2.41292900
C	2.22929700	-0.39752500	-0.63276900
C	2.80291800	-0.62372400	-1.88404600
F	2.05994600	-0.94438100	-2.95280900
C	4.16887300	-0.52207000	-2.13741000
F	4.64909300	-0.77866000	-3.35645000
C	5.03038700	-0.12972100	-1.12790000
F	6.33815600	-0.02210100	-1.35707200
C	4.50648900	0.17353400	0.12016900

F	5.31259200	0.59713000	1.09444700
C	3.13997200	0.05723100	0.32753500
F	2.70235200	0.45334400	1.53225000
H	-1.70606000	0.15210000	0.37749600
H	-2.09181700	2.48329200	0.10068500
H	-2.85741400	1.76689900	1.53733800
C	3.34991700	-2.54166200	3.96445600
C	3.44242800	-2.94839900	2.63579800
C	2.45322600	-2.59158700	1.72566800
C	1.35662300	-1.80990000	2.11072700
C	1.26772700	-1.43036500	3.45268600
C	2.25263400	-1.78766700	4.36906000
H	4.12105600	-2.81585400	4.67712000
H	4.28631500	-3.54750900	2.30703000
H	2.53385500	-2.91538600	0.69010400
H	0.41829700	-0.83993600	3.78165400
H	2.16183100	-1.47334100	5.40459500
C	0.29955300	-1.45625600	1.09281000
H	-0.01529300	-2.41502100	0.66289500
H	-0.58443200	-1.09085200	1.63958500

P1_TS

P	-1.26821700	0.28949700	-0.12315300
C	-0.97360200	2.05598600	-0.31446200
C	0.30631100	2.42628100	0.13971400
B	1.42990700	1.35588900	0.54732200
C	-1.91300200	2.99593200	-0.74638700
C	-1.57420500	4.34333000	-0.74434600
C	-0.30962900	4.73655500	-0.30301500
C	0.61602800	3.79200300	0.12910000
O	2.66665400	1.93600500	1.01109800
O	1.76203200	0.36917500	-0.46642700
C	3.11208700	0.22249600	-0.40266500
C	3.90841300	-0.68525300	-1.07174200
C	5.28660100	-0.64103600	-0.81489500
C	5.82489600	0.28022100	0.07808200
C	5.00863700	1.19900700	0.75381600
C	3.65382500	1.15328400	0.49341800
H	3.47167500	-1.40267000	-1.75779700
H	5.94259100	-1.34117300	-1.32170400
H	6.89449400	0.28907000	0.25924700
H	5.41357000	1.92134300	1.45353500
C	-1.06597100	-0.65813900	-1.64988300
C	-2.97364700	0.05157800	0.44932000
C	-3.70400100	-1.08149900	0.07586700
C	-4.97000700	-1.29860100	0.61108400
C	-5.51028200	-0.39532000	1.52323100
C	-4.78169400	0.72928200	1.90373400
C	-3.51618800	0.95393000	1.37262600
H	-3.28257400	-1.78879900	-0.63265800
H	-5.53531800	-2.17612500	0.31465300
H	-6.49763600	-0.56792500	1.93911800
H	-5.19836500	1.43408700	2.61575200
H	-2.95068100	1.83366900	1.66743300
C	-0.22699900	-1.77652800	-1.63440800
C	-0.09311400	-2.54415200	-2.78822400
C	-0.78473000	-2.19644300	-3.94558300
C	-1.61706800	-1.07794600	-3.95972800
C	-1.76022500	-0.30796300	-2.81158200
H	0.32402400	-2.03117800	-0.73168900
H	0.55569600	-3.41385300	-2.78033100
H	-0.67411000	-2.79652200	-4.84340900
H	-2.15044100	-0.80571300	-4.86448300
H	-2.40513100	0.56695900	-2.81757800
H	-2.90368600	2.67643000	-1.06103900
H	-2.29130400	5.08517100	-1.08114900
H	-0.04776900	5.79047900	-0.29771300
H	1.60055100	4.10304800	0.46517400
H	-0.09518700	0.18795900	0.93191600
C	0.58243900	-4.06993000	1.47798000
C	-0.54324400	-3.35156300	1.86950800
C	-0.44579200	-1.98681900	2.13237100
C	0.77566200	-1.30497500	2.01087700
C	1.90272800	-2.04848000	1.63090800
C	1.80745500	-3.41110200	1.36914800
H	0.51060300	-5.13254900	1.26939300
H	-1.50256700	-3.85018900	1.97290500
H	-1.33195800	-1.43698900	2.44503700
H	2.86164100	-1.54632300	1.53132200
H	2.69672100	-3.96142200	1.07610900
C	0.83868600	0.18233900	2.11771900
H	0.06771200	0.58812000	2.78333900
H	1.80947600	0.49289500	2.50841500

P1_P

P	2.16022800	-0.11589200	-0.39653700
C	1.50705400	1.48891100	0.06567300
C	0.12426900	1.75277600	-0.00261300

B	-1.14747400	0.73631600	-0.34202300
C	2.45773600	2.43843000	0.48595000
C	2.03691900	3.70312500	0.85849000
C	0.67739100	4.00908900	0.79659700
C	-0.24158300	3.05778200	0.37513500
O	-2.26621100	1.57734100	-0.85727800
O	-1.65006700	0.21465500	0.98461500
C	-2.93183300	0.63387500	1.10174400
C	-3.82457300	0.36537200	2.11923300
C	-5.11771000	0.90750700	2.01966800
C	-5.48211900	1.68664700	0.93081100
C	-4.56924500	1.95894600	-0.10223800
C	-3.29932800	1.42753700	0.00079700
H	-3.52850200	-0.25014500	2.96235900
H	-5.83947000	0.70859900	2.80568400
H	-6.48739600	2.09167200	0.87053500
H	-4.84171900	2.56047100	-0.96262100
C	1.82849600	-1.42918600	0.79079800
C	3.94265600	-0.03554100	-0.66950600
C	4.84131100	-0.27037900	0.37575800
C	6.20742100	-0.13871600	0.14939100
C	6.67433000	0.22267600	-1.11255000
C	5.77932100	0.45502200	-2.15408700
C	4.41185600	0.32748300	-1.93525300
H	4.47385300	-0.55960800	1.35638900
H	6.90770600	-0.32037500	0.95754800
H	7.74095200	0.32165100	-1.28556000
H	6.14456300	0.73370000	-3.13654600
H	3.71267800	0.51182600	-2.74664600
C	2.52414200	-2.64126400	0.68508500
C	2.23439600	-3.67231000	1.56804700
C	1.25222800	-3.49682300	2.54401400
C	0.56119800	-2.29371500	2.63972900
C	0.84620000	-1.24668000	1.76609200
H	3.28758900	-2.77569000	-0.07654500
H	2.76824600	-4.61354800	1.49278200
H	1.02386700	-4.30801200	3.22788800
H	-0.21251400	-2.16146600	3.38795400
H	0.27466400	-0.32524900	1.82418700
H	3.51562400	2.19547200	0.51135500
H	2.76005400	4.44330900	1.18431000
H	0.33530700	5.00052600	1.07888200
H	-1.29444600	3.31561200	0.32267500
H	1.65497100	-0.49075900	-1.64492700
C	-4.78111300	-2.32373100	-1.96485400
C	-3.96880700	-2.72962600	-0.90982100
C	-2.70710400	-2.16794500	-0.74182300
C	-2.23053400	-1.19035400	-1.62073000
C	-3.05931700	-0.78717800	-2.67158400
C	-4.32048000	-1.34783500	-2.84478200
H	-5.76722200	-2.75835500	-2.09561500
H	-4.32257500	-3.48105300	-0.20996800
H	-2.08355200	-2.46521500	0.09836300
H	-2.71341800	-0.00700300	-3.34434100
H	-4.94916300	-1.01714200	-3.66638400
C	-0.90400600	-0.52493600	-1.37386500
H	-0.22958800	-1.28621700	-0.96071900
H	-0.48137300	-0.17592000	-2.32740100

P5_P

P	-1.93986600	0.63631100	0.23459200
B	1.47418100	0.46982300	-0.06866900
C	-0.67135700	1.51937900	1.21523600
C	0.76279600	0.98325700	1.32550400
C	-3.39014800	1.73079400	0.17350900
C	-5.42309000	2.58275400	1.14672700
C	-5.60893000	3.44642800	0.07081300
C	-4.66084800	3.44170500	-0.95119800
C	-3.54685300	2.60527600	-0.92515300
C	-2.45833700	-1.08145200	0.51689800
C	-2.17101700	-1.82151800	1.67631800
C	-2.60751500	-3.14717800	1.74545100
C	-3.29904000	-3.75753500	0.70641500
C	-3.58556500	-2.99781000	-0.42879800
C	-3.17859700	-1.67407100	-0.55276100
C	3.06646300	0.09530400	0.14715300
C	3.82906500	0.29637900	1.29250500
C	5.18919000	0.01310400	1.37344900
C	5.84744000	-0.51280400	0.27376200
C	5.13083700	-0.75180400	-0.89033200
C	3.77553300	-0.45313600	-0.92190600
C	0.77688900	-0.97793500	-0.50786100
C	0.86782500	-2.05441300	0.37658100
C	0.27263000	-3.29160900	0.17304200
C	-0.42616200	-3.52104700	-1.00118500
C	-0.50714800	-2.50791100	-1.94155500
C	0.08429200	-1.27805700	-1.67640100
C	-6.78285300	4.38749200	0.02509300

C	-2.56704200	2.67711800	-2.07313100
C	-1.37023200	-1.31697100	2.85003100
C	-3.66856300	-5.21328900	0.77168400
C	-3.47598100	-0.95592600	-1.84814800
F	3.28290400	0.79527300	2.41408200
F	5.86498500	0.23894800	2.50251800
F	7.14943300	-0.79174600	0.33402700
F	5.74646600	-1.26925500	-1.95574400
F	3.14424000	-0.71195700	-2.08273300
F	1.52717500	-1.91955100	1.54320900
F	0.31469300	-4.23622900	1.11539700
F	-1.05856400	-4.67919100	-1.19990300
F	-1.23219600	-2.69408400	-3.04985300
F	-0.14575800	-0.32428000	-2.60827400
H	-1.13451700	1.75214900	2.18154800
H	-0.66625800	2.46764200	0.65922800
H	0.81027900	0.22260800	2.10811600
H	1.33122700	1.83805600	1.70861100
H	-6.14935600	2.57702200	1.95560700
H	-4.78860600	4.11382700	-1.79595100
H	-4.11639500	-3.45938500	-1.25719200
H	-7.13954400	4.52364200	-0.99834900
H	-7.61067500	4.01883900	0.63413100
H	-6.49841200	5.37229800	0.40921600
H	-2.51132000	1.73521600	-2.63014600
H	-1.55245600	2.91649100	-1.73906900
H	-2.86955600	3.45573400	-2.77444500
H	-0.36595100	-1.75142400	2.82229900
H	-1.25405700	-0.23600600	2.87618400
H	-1.84382400	-1.62843700	3.78463300
H	-4.57663900	-5.42054500	0.20144100
H	-3.81864300	-5.54163000	1.80217100
H	-2.85803600	-5.80951100	0.34119700
H	-4.07340600	-1.59684500	-2.49736600
H	-4.03264600	-0.02725200	-1.68861700
H	-2.55561900	-0.71991400	-2.39475800
C	-4.32929700	1.72163500	1.22291200
C	-4.21411500	0.81255600	2.42232100
H	-4.48033200	-0.21822300	2.16949600
H	-3.20158700	0.79623100	2.83623900
H	-4.88271500	1.15222700	3.21496800
H	-2.36576600	-3.72261300	2.63584100
H	-1.45276700	0.63488100	-1.07024000
C	3.07421200	5.40079900	0.11844000
C	1.69786700	5.25827900	-0.01539100
C	1.16239600	4.05583400	-0.47157000
C	1.97033100	2.95900000	-0.79695300
C	3.35769000	3.13488300	-0.67609200
C	3.90059500	4.33276700	-0.22453800
H	3.49952800	6.33433400	0.47215000
H	1.03716400	6.08605100	0.22513100
H	0.08418500	3.98092600	-0.60473400
H	4.02094500	2.32022000	-0.95073900
H	4.97868400	4.43361400	-0.14334700
C	1.37758900	1.64505000	-1.23760900
H	0.33599400	1.82572600	-1.53788100
H	1.88203400	1.29695900	-2.14502300

P6_TS

P	1.97149600	-0.60159100	0.38721800
C	1.48567400	-0.69804100	2.16172700
H	2.34749700	-0.94712600	2.78470900
H	1.07059800	0.25184900	2.49940200
C	0.39520000	-1.76899100	2.22950800
H	0.68931000	-2.61966700	1.59551300
H	0.36237000	-2.17365300	3.24734600
C	-0.99577800	-1.23663400	1.86205200
H	-1.33853400	-0.63448000	2.70712700
H	-1.66578400	-2.10041700	1.85617100
B	-1.31223200	-0.39083800	0.49966800
C	2.63602300	1.04835200	-0.09818200
C	2.81222400	2.15772000	0.75943500
C	2.70167900	2.14637900	2.26999800
H	3.30200100	2.96383200	2.67548500
H	3.06149800	1.22304700	2.71747800
H	1.67492500	2.30773100	2.61352700
C	3.12978500	3.39618300	0.19218600
H	3.23808900	4.25196800	0.85567500
C	3.27179900	3.58418100	-1.17700100
C	3.46961500	4.95805200	-1.75505700
H	2.49179100	5.40940900	-1.95284200
H	4.01985800	4.92128800	-2.69797700
H	4.00564400	5.61083600	-1.06225800
C	3.16044200	2.46503200	-1.99801500
H	3.29039800	2.57781300	-3.07155700
C	2.84641800	1.20912000	-1.48957800
C	2.72174000	0.06102300	-2.46108600
H	1.70488600	-0.34443600	-2.45337900

H	3.41975100	-0.74878700	-2.22618900
H	2.92779400	0.40201500	-3.47708400
C	3.36548200	-1.82390200	0.23713700
C	4.60780900	-1.62091900	0.88364900
C	4.93641500	-0.42032500	1.73523600
H	5.99406800	-0.43440600	2.00408100
H	4.36949400	-0.42181800	2.67044700
H	4.73346400	0.51870900	1.21814700
C	5.61477000	-2.57936600	0.74717200
H	6.56335000	-2.40824500	1.25035200
C	5.44947800	-3.73597700	-0.00417800
C	6.55933300	-4.74207900	-0.15326800
H	7.04703800	-4.63632400	-1.12762400
H	6.17570900	-5.76336100	-0.08753000
H	7.32107100	-4.60997500	0.61786200
C	4.22627600	-3.92035400	-0.63956000
H	4.06791600	-4.81478800	-1.23747200
C	3.19064100	-2.99225800	-0.53965600
C	1.92947400	-3.29643500	-1.30749400
H	1.75919200	-2.55606700	-2.09587400
H	1.04648200	-3.32074600	-0.66679700
H	2.00473600	-4.27285100	-1.78898300
C	-0.68070500	1.13831400	0.33537200
C	-0.28684800	1.74264200	-0.86312600
F	-0.12586600	1.02151600	-1.98831000
C	0.02255500	3.08726100	-1.00237700
F	0.38734800	3.58627100	-2.18513800
C	0.00755300	3.91157300	0.10997300
F	0.39373300	5.18190800	0.00879800
C	-0.34796400	3.37007900	1.33189400
F	-0.30093700	4.11826200	2.43661100
C	-0.68688900	2.02396100	1.41272900
F	-0.99254000	1.59621700	2.65149400
C	-2.95428100	-0.17412700	0.32718800
C	-3.92843700	-0.55151200	1.25654700
F	-3.62367300	-1.14766100	2.41718500
C	-5.29497300	-0.34546900	1.08145700
F	-6.15426700	-0.73986800	2.02088400
C	-5.76501500	0.27225300	-0.06299800
F	-7.06759300	0.47136700	-0.24280600
C	-4.84722600	0.68007600	-1.01716800
F	-5.26266900	1.27797800	-2.13435700
C	-3.49529200	0.46186100	-0.79546600
F	-2.69511800	0.89168400	-1.79218900
H	0.31015700	-1.03573400	-0.31154700
C	-3.43983700	-4.93527000	-0.93931500
C	-2.30972000	-4.89248300	-0.13012200
C	-1.49041500	-3.76592100	-0.12353400
C	-1.77818800	-2.66020100	-0.92808300
C	-2.91870000	-2.72066900	-1.73854100
C	-3.74245100	-3.84072700	-1.74490700
H	-4.08035600	-5.81103400	-0.93983800
H	-2.06357500	-5.73577500	0.50744000
H	-0.62808400	-3.75002700	0.53479100
H	-3.15932400	-1.87070900	-2.37268100
H	-4.62190200	-3.85670500	-2.38087700
C	-0.92849900	-1.40961100	-0.99557100
H	-0.01737400	-1.69635400	-1.54481700
H	-1.35621100	-0.71277200	-1.71025100

P6_P

P	-2.20681900	0.54990600	0.44022900
C	-1.21309200	0.90996700	1.92059200
H	-1.91286500	1.22406100	2.69986200
H	-0.72538400	-0.00851700	2.24828500
C	-0.13815400	1.98716300	1.63517500
H	-0.38369900	2.55523300	0.72849600
H	-0.19045700	2.71404500	2.45235600
C	1.26647700	1.37604300	1.54464100
H	1.41609500	0.77556200	2.44765500
H	1.97736200	2.20514100	1.63148900
B	1.66255700	0.56880100	0.17063000
C	-2.85727800	-1.13507200	0.25832400
C	-2.91280000	-2.07806200	1.30890300
C	-2.46619700	-1.83208000	2.73094200
H	-2.94114400	-2.56092000	3.38977600
H	-2.72340800	-0.83986800	3.10054800
H	-1.38362000	-1.96818400	2.82949200
C	-3.37056500	-3.36319500	1.01804500
H	-3.40103600	-4.09327500	1.82257300
C	-3.76041100	-3.74866800	-0.26124400
C	-4.17321700	-5.16476700	-0.54824300
H	-3.28859700	-5.75014500	-0.81688700
H	-4.87615800	-5.21388800	-1.38238000
H	-4.63270800	-5.63105400	0.32575700
C	-3.70313700	-2.79937000	-1.27818800
H	-3.99177400	-3.08008100	-2.28732800
C	-3.25910100	-1.50039700	-1.04914500

C	-3.16546200	-0.56618300	-2.23000600
H	-2.11949600	-0.37286000	-2.49498700
H	-3.66469500	0.38962000	-2.04460500
H	-3.63566700	-1.02164400	-3.10218300
C	-3.48470200	1.82340300	0.22122400
C	-4.62257900	1.82166800	1.05607900
C	-4.83544100	0.80880100	2.15399500
H	-5.80871200	0.96108500	2.62254100
H	-4.08222200	0.90673500	2.94263100
H	-4.80100600	-0.21772000	1.77933500
C	-5.59164400	2.80545800	0.87223500
H	-6.46594700	2.80421300	1.51786900
C	-5.47086000	3.78496900	-0.10960700
C	-6.54301400	4.82153000	-0.31105200
H	-7.19331900	4.54129400	-1.14578200
H	-6.10853000	5.79606500	-0.54530100
H	-7.16703600	4.92543400	0.57862300
C	-4.33706300	3.76870400	-0.91893800
H	-4.22425500	4.53108500	-1.68534500
C	-3.33323400	2.81110000	-0.78102300
C	-2.14214800	2.90852200	-1.70628600
H	-1.95859700	1.98793300	-2.26839100
H	-1.22268700	3.14582800	-1.16407900
H	-2.30336300	3.70593700	-2.43266100
C	0.74246500	-0.81987500	-0.10043500
C	0.20944500	-1.21784500	-1.32678200
F	0.16567900	-0.36322900	-2.37035800
C	-0.35962900	-2.46057400	-1.57948300
F	-0.89908300	-2.73176500	-2.77246300
C	-0.43542400	-3.39693500	-0.56226200
F	-1.00540900	-4.58548900	-0.77069300
C	0.03881200	-3.05115200	0.69241000
F	-0.11398900	-3.89776300	1.71623200
C	0.60736400	-1.79767000	0.88662000
F	1.01898800	-1.54961800	2.14746000
C	3.20448200	-0.04082200	0.24042500
C	4.11855500	0.12201500	1.27851000
F	3.79675900	0.78389300	2.40089400
C	5.42287100	-0.36558300	1.24654500
F	6.24668600	-0.16615800	2.27829200
C	5.87038700	-1.06352000	0.13800400
F	7.11515900	-1.53827900	0.09133200
C	5.00091500	-1.26356400	-0.92445800
F	5.41101500	-1.93795200	-2.00173500
C	3.71077100	-0.75823700	-0.84624400
F	2.93649200	-0.98757600	-1.92580900
C	3.94293300	5.19421000	-0.38232300
C	2.55896000	5.22367200	-0.24438600
C	1.80706400	4.07899700	-0.49549900
C	2.40459300	2.87427100	-0.88541200
C	3.79974500	2.87038100	-1.02907400
C	4.55803000	4.00965800	-0.78067000
H	4.53449500	6.08319300	-0.18866500
H	2.06076800	6.14113700	0.05557500
H	0.72458800	4.12519900	-0.39156900
H	4.29461000	1.95694200	-1.34811400
H	5.63649800	3.97247700	-0.90240600
C	1.59914200	1.62405100	-1.11490700
H	0.55944600	1.91281500	-1.32669800
H	1.95608900	1.13142900	-2.02276700
H	-1.34190300	0.73248500	-0.63967200

P7_TS

P	2.02255900	-0.50410600	0.10600900
B	-1.11901500	0.25830200	0.23486000
C	1.41351500	-0.56250100	3.12311500
H	2.07214000	-1.35134800	3.49568500
C	1.02329800	-0.68980900	1.63713400
H	0.52081800	-1.64748300	1.50724800
C	-0.03462400	0.44322500	1.46927600
H	0.53489600	1.33975200	1.18734400
C	-0.46383500	0.69962200	2.95347300
H	-1.50542100	0.99508000	3.08411700
C	0.56576600	1.70169100	3.50835800
H	0.31305700	1.99167600	4.53326300
H	0.62296300	2.61408800	2.90693500
C	1.88795200	0.87044100	3.47350100
H	2.60847100	1.26211100	2.75132700
H	2.37975700	0.86955200	4.45018800
C	-0.01319400	-0.56361000	3.71391900
H	-0.01805500	-0.41071300	4.79812800
H	-0.57455100	-1.46685000	3.47638100
C	3.48431800	0.57651100	0.41233400
C	4.60651400	0.08283500	1.11392200
C	5.69359900	0.92850200	1.33669900
H	6.55230100	0.53959800	1.87886300
C	5.71160700	2.24670500	0.89325400
C	4.59990200	2.71450100	0.19886100

H	4.59273600	3.73995600	-0.16304900
C	3.48872500	1.91045000	-0.05228300
C	4.69497200	-1.32128500	1.65324800
H	5.54622800	-1.41005800	2.33074100
H	4.81566000	-2.05664500	0.85264300
H	3.79568700	-1.58789000	2.21235200
C	6.88363700	3.14993000	1.17098900
H	7.13372900	3.75127000	0.29320100
H	7.76732600	2.57666700	1.45876700
H	6.65204500	3.84157400	1.98728500
C	2.33867200	2.52448600	-0.80730500
H	2.63576400	3.48458700	-1.23531900
H	1.48168400	2.70933500	-0.15066900
H	1.99504900	1.88413500	-1.62265900
C	2.64233700	-2.06470500	-0.64258500
C	3.14814200	-1.91082300	-1.95543000
C	3.61958500	-3.02681800	-2.64624900
H	4.01382900	-2.89091900	-3.65037500
C	3.58808200	-4.30158100	-2.09332700
C	3.05080800	-4.44108400	-0.81610900
H	2.99775900	-5.43293600	-0.37215800
C	2.57170800	-3.35828200	-0.07845500
C	3.18026400	-0.57611400	-2.66259900
H	3.84334900	0.13856800	-2.16473700
H	2.18320200	-0.12251400	-2.70563200
H	3.53229800	-0.70223100	-3.68791800
C	4.11950600	-5.49629900	-2.83971900
H	3.43096800	-6.34242000	-2.76869300
H	5.07917800	-5.81896800	-2.42326300
H	4.27351600	-5.26758600	-3.89628400
C	1.98113700	-3.67177400	1.27575200
H	2.31157200	-4.65890000	1.60481300
H	0.88589000	-3.69245000	1.23789000
H	2.26774600	-2.95077800	2.04118500
C	-2.55987300	-0.33270600	0.71700600
F	-3.89020200	1.32358400	-0.36668300
C	-3.79754100	0.22068700	0.38511400
F	-6.15929300	0.24773400	0.40550400
C	-5.01593500	-0.33775500	0.75551800
F	-6.20127200	-2.06567200	1.83822800
C	-5.04336800	-1.51529800	1.48512300
F	-3.83708000	-3.28415200	2.47373900
C	-3.84356500	-2.12077200	1.82134700
F	-1.54438900	-2.25619500	1.68377100
C	-2.65522100	-1.52445200	1.43012500
C	-1.18082200	1.69646800	-0.54467900
F	-2.26942400	2.57853800	1.35512000
C	-1.70412600	2.78484300	0.15614900
F	-2.21295900	5.07797800	0.42495700
C	-1.68758000	4.09268900	-0.30038000
F	-1.06123700	5.62433300	-1.98528100
C	-1.10184000	4.37555800	-1.52861400
F	0.05792400	3.58471700	-3.42105300
C	-0.55355600	3.33891300	-2.26127300
F	0.02819800	1.12181200	-2.52402500
C	-0.59891700	2.04358500	-1.75479100
H	0.39230800	-0.30305000	-0.65091300
C	-4.52494600	-1.83414500	-2.92160700
C	-3.99316700	-2.73772900	-2.00435600
C	-2.74171600	-2.50687000	-1.44507300
C	-2.00177000	-1.36398400	-1.77168100
C	-2.52552300	-0.48813400	-2.72408700
C	-3.77845200	-0.71927700	-3.28695500
H	-5.50562500	-2.00335600	-3.35389000
H	-4.55364300	-3.62303800	-1.72000500
H	-2.33802500	-3.21030500	-0.72073800
H	-1.95869800	0.37804400	-3.03669900
H	-4.17187000	-0.01604200	-4.01410300
C	-0.66792100	-1.14641800	-1.07055100
H	-0.45612000	-2.02108900	-0.44858700
H	0.07505000	-1.16893700	-1.88223500

P7_P

P	-2.26630100	-0.51368000	-0.20009700
B	1.23635800	0.29495800	-0.13878500
C	-1.47102000	-0.08265300	-3.01635200
H	-2.08666600	-0.83922400	-3.51255500
C	-1.05449000	-0.43733600	-1.57129400
H	-0.57259600	-1.41416500	-1.56281700
C	-0.00007300	0.66217300	-1.21822600
H	-0.55764500	1.45925800	-0.70929200
C	0.32638700	1.24104000	-2.63078700
H	1.34442500	1.62376600	-2.71538600
C	-0.77327600	2.26187200	-2.96889100
H	-0.55766200	2.77767200	-3.91013300
H	-0.88558100	3.02397400	-2.19125800
C	-2.03676200	1.35587300	-3.11060200
H	-2.79341700	1.56989100	-2.35215900

H	-2.51745100	1.49520000	-4.08294100
C	-0.05914000	0.11391400	-3.60767600
H	-0.09793400	0.46008100	-4.64570500
H	0.56192400	-0.77752000	-3.54634500
C	-3.84299800	0.37369200	-0.40262200
C	-4.90393700	-0.20955800	-1.12039400
C	-6.10617500	0.48731700	-1.22908700
H	-6.92346100	0.03457500	-1.78436300
C	-6.28263200	1.74188000	-0.65458800
C	-5.21154300	2.30667800	0.03766600
H	-5.32994300	3.29354000	0.47821700
C	-3.98693000	1.65851700	0.17903200
C	-4.78224800	-1.54591700	-1.80544400
H	-5.70878900	-1.79099400	-2.32686600
H	-4.56908400	-2.35217100	-1.09782500
H	-3.98361900	-1.52879600	-2.55422700
C	-7.58718200	2.48251200	-0.77407300
H	-7.43230200	3.47477600	-1.20678000
H	-8.04316400	2.62278300	0.21045800
H	-8.29599500	1.94113500	-1.40322400
C	-2.88269900	2.39025100	0.90945200
H	-3.24244000	3.36628600	1.23804100
H	-2.01559000	2.56364500	0.26267100
H	-2.52387300	1.86067400	1.79696800
C	-2.49686800	-2.14754100	0.56644300
C	-2.84480700	-2.13466900	1.93898000
C	-2.99984100	-3.34798500	2.60156300
H	-3.27001700	-3.33683900	3.65444700
C	-2.81132600	-4.56969500	1.95856700
C	-2.46600100	-4.55467100	0.61176400
H	-2.31649100	-5.49872000	0.09360500
C	-2.29387800	-3.36973600	-0.10556200
C	-3.02913200	-0.86233300	2.73137200
H	-3.74260800	-0.17784500	2.26144200
H	-2.07731600	-0.33144500	2.85839200
H	-3.40539200	-1.09626600	3.72806000
C	-2.94969900	-5.86520100	2.71124500
H	-2.02069800	-6.09552100	3.24243300
H	-3.16271900	-6.69641400	2.03595700
H	-3.74880500	-5.80697100	3.45394200
C	-1.87303400	-3.50817700	-1.55001300
H	-2.22618500	-4.46295700	-1.94483500
H	-0.78054900	-3.49990600	-1.63732400
H	-2.26153300	-2.71947400	-2.19406600
C	2.63442300	-0.24325300	-0.83116000
F	4.09125800	1.09328600	0.49615100
C	3.91503600	0.13822400	-0.42701500
F	6.27539200	-0.02210000	-0.47034100
C	5.08475300	-0.45244700	-0.89003000
F	6.12729800	-2.09250500	-2.23516300
C	5.01651800	-1.51284600	-1.78036600
F	3.66846900	-3.02317000	-2.99346400
C	3.77101100	-1.97275900	-2.17286700
F	1.46544400	-1.94574000	-2.02243000
C	2.63645300	-1.34440400	-1.67855200
C	1.39294600	1.78333100	0.58568200
F	2.82557100	2.62164800	-1.11678900
C	2.08181700	2.83667300	-0.01885400
F	2.73166100	5.10307100	-0.21896000
C	2.03253700	4.15733900	0.40952200
F	1.13485700	5.76785900	1.89032300
C	1.21984800	4.50262300	1.47982000
F	-0.35603100	3.81102400	3.09598800
C	0.48038400	3.50851600	2.09710300
F	-0.22923900	1.32288400	2.28833600
C	0.58676400	2.20021300	1.64041700
H	-1.64825400	0.21234200	0.81421400
C	4.27032300	-2.04902300	3.31141000
C	3.90985300	-2.82273800	2.21144700
C	2.78946900	-2.48563000	1.45803700
C	2.00885300	-1.36823000	1.77519100
C	2.37835200	-0.60772100	2.89022500
C	3.49481900	-0.94309100	3.65020300
H	5.14566400	-2.30611200	3.89923400
H	4.50257100	-3.69064400	1.93762200
H	2.51455800	-3.09167100	0.59703400
H	1.77719100	0.25562400	3.16191800
H	3.76371500	-0.33458800	4.50852000
C	0.85210800	-0.95234300	0.90421100
H	0.56499700	-1.81861400	0.28919400
H	0.00156900	-0.74037200	1.55887800

P8_TS

C	-1.05606600	-1.19167900	-1.02078000
H	-1.37787400	-0.60174600	-1.88376000
H	-0.67295400	-2.14000100	-1.40055300
P	-2.44550200	-1.45600700	0.15815200
B	0.02716500	-0.31153800	-0.14508400

C	-4.00727100	-0.65221300	-0.56102900
C	-3.90090100	0.86384300	-0.34367000
H	-3.06284900	1.29584600	-0.89741400
H	-4.81934600	1.33312600	-0.71433500
H	-3.79105200	1.11752800	0.71522100
C	-5.25951800	-1.14661900	0.17344900
H	-6.10939900	-0.52637700	-0.13156200
H	-5.50472400	-2.18046400	-0.08017300
H	-5.15927500	-1.06322600	1.26017400
C	-4.16066900	-0.90797000	-2.06655600
H	-4.21009100	-1.96844200	-2.31751600
H	-5.09317100	-0.44031900	-2.40439300
H	-3.34560300	-0.45848500	-2.63836400
C	-2.69527700	-3.32054800	0.36729300
C	-1.33308000	-3.94587700	0.71005900
H	-0.56003000	-3.73945000	-0.03214100
H	-0.97764800	-3.61006900	1.68810100
H	-1.46089300	-5.03239600	0.76697000
C	-3.61580600	-3.60384400	1.56649100
H	-3.28334400	-3.06278700	2.45832400
H	-4.65845700	-3.34998500	1.37974800
H	-3.57478000	-4.67539400	1.79271500
C	-3.23753500	-3.99225500	-0.89756400
H	-3.23209300	-5.07960800	-0.75909200
H	-4.26741100	-3.69625300	-1.11083100
H	-2.61921600	-3.76341400	-1.77172600
C	1.56756200	-0.46029100	-0.64089500
C	2.11182600	-1.70617700	-0.93981000
C	2.48571200	0.58995400	-0.69767200
C	3.43956000	-1.91864900	-1.28489600
C	3.82202700	0.42665400	-1.03800600
C	4.30530300	-0.83801100	-1.33705300
F	1.36548900	-2.82625700	-0.82836100
F	3.88530300	-3.14833600	-1.54374400
F	4.64466800	1.47302700	-1.07085600
F	2.12772200	1.84048200	-0.38358000
F	5.58300500	-1.01218400	-1.66106900
C	-0.52074400	1.22447500	-0.08888100
C	-1.19391700	1.86233700	0.94294500
C	-0.43287300	1.97851000	-1.26060900
C	-1.70533600	3.15356500	0.85825500
C	-0.93158900	3.26360500	-1.40096400
C	-1.57693700	3.86097600	-0.32374500
F	0.17007700	1.44929900	-2.33538900
F	-0.79955400	3.92849800	-2.54668900
F	-2.07208200	5.08973100	-0.43375100
F	-2.36208500	3.68342200	1.89005300
F	-1.45195600	1.22733000	2.10723400
H	-1.13509000	-0.84339700	1.19137400
C	3.90058500	0.74252900	2.97981000
C	3.84052000	-0.58235300	2.55348300
C	2.62770700	-1.12757700	2.15153000
C	1.45307300	-0.36430900	2.15198400
C	1.51682300	0.94896400	2.61850900
C	2.73327500	1.49649700	3.02235600
H	4.84626500	1.17815300	3.28502000
H	4.73862400	-1.19177500	2.52958100
H	2.58891900	-2.15810300	1.80644600
H	0.62002700	1.55155100	2.67491500
H	2.76347600	2.52487300	3.36816600
C	0.18476500	-1.02575300	1.63004100
H	0.40753500	-2.06506200	1.38280900
H	-0.48543500	-1.06834400	2.50638000

P8_P

C	-0.99916400	-1.26151900	-0.67120800
H	-1.17215500	-0.78488200	-1.64204100
H	-0.68239100	-2.28652700	-0.87012900
P	-2.58629400	-1.37101900	0.16066000
B	0.17669900	-0.33928800	0.13302500
C	-3.97814300	-0.74970800	-0.91159900
C	-3.78566200	0.77280700	-1.02009800
H	-2.81184300	1.04216300	-1.43976400
H	-4.55954900	1.17043300	-1.68458400
H	-3.88979000	1.26326400	-0.04716900
C	-5.34631800	-1.02712500	-0.27778400
H	-6.10875900	-0.50203300	-0.86219000
H	-5.59938100	-2.09004500	-0.28816200
H	-5.40615200	-0.65756800	0.75076800
C	-3.90779500	-1.37370500	-2.31219100
H	-4.03952200	-2.45773800	-2.29615600
H	-4.71510800	-0.94894800	-2.91741800
H	-2.96457100	-1.14725400	-2.81449900
C	-2.84952800	-3.05924500	0.90112300
C	-1.52530500	-3.49827200	1.54886400
H	-0.69760400	-3.55299200	0.83798700
H	-1.23309800	-2.82892400	2.36132900
H	-1.67624100	-4.49703500	1.97147300

C	-3.92138900	-3.00866500	2.00025800
H	-3.67232300	-2.27021600	2.76842800
H	-4.91891400	-2.79244700	1.61736500
H	-3.95941600	-3.98897400	2.48608100
C	-3.23769600	-4.06034100	-0.19449900
H	-3.25258100	-5.06440000	0.24102700
H	-4.23255300	-3.86218800	-0.60217800
H	-2.51453900	-4.06754500	-1.01649600
C	1.62206100	-0.63099200	-0.59716300
C	2.06762200	-1.92644800	-0.84548300
C	2.59912900	0.34422300	-0.79374300
C	3.34091500	-2.24733600	-1.30044000
C	3.88402200	0.07687000	-1.24787800
C	4.26005600	-1.23210300	-1.50860500
F	1.26933600	-2.99597700	-0.60221100
F	3.68498000	-3.51807800	-1.52315900
F	4.76480400	1.06263400	-1.41772700
F	2.34397200	1.62854600	-0.51165000
F	5.48801400	-1.51100400	-1.94225300
C	-0.39004700	1.19966400	-0.08706100
C	-1.19001800	1.89759100	0.81034000
C	-0.24981700	1.84634600	-1.31910800
C	-1.75333000	3.14641500	0.56072300
C	-0.78581200	3.09015900	-1.62013400
C	-1.54793300	3.75267000	-0.66551800
F	0.41424600	1.24468700	-2.31797400
F	-0.59613800	3.64659300	-2.81568800
F	-2.08746000	4.93847500	-0.93616400
F	-2.53089100	3.73098400	1.47442800
F	-1.54010000	1.36259700	2.00491300
C	3.74345100	1.46051400	3.41532600
C	3.95257500	0.22434900	2.81025300
C	2.87828800	-0.48926900	2.28896700
C	1.57061200	0.01082600	2.34614700
C	1.37808600	1.24900800	2.96921800
C	2.44929300	1.96465400	3.49723200
H	4.57868400	2.02095400	3.82272600
H	4.95472900	-0.18865600	2.74497700
H	3.05419200	-1.45555500	1.82062300
H	0.37491300	1.65192000	3.05361200
H	2.26887200	2.92280700	3.97541600
C	0.43287500	-0.75535500	1.71149700
H	0.70256000	-1.81884000	1.71923700
H	-0.46463300	-0.64653300	2.33277300
H	-2.61496600	-0.50172500	1.26469500

P9_TS

C	1.03010800	-0.85834300	1.12068200
H	1.01328900	-1.94630900	1.24171100
H	1.32579700	-0.43204200	2.08393400
B	-0.41451900	-0.22772700	0.62684700
C	-1.68352600	-0.91965600	1.33463900
C	-2.83038700	-0.19172800	1.68433500
C	-1.72046700	-2.30173700	1.57500500
C	-3.95239300	-0.80494800	2.23703700
C	-2.83788900	-2.93162000	2.11583900
C	-3.96267800	-2.18016400	2.45056400
P	2.17827100	-0.42255900	-0.23335200
C	3.02502700	-2.02102500	-0.77826400
C	4.03072400	-1.74071200	-1.90239900
H	4.90270500	-1.18030000	-1.55829800
H	4.39172600	-2.69398100	-2.30511100
H	3.56666600	-1.18542400	-2.72439600
C	3.69551100	-2.78673900	0.37034400
H	4.57087900	-2.27468100	0.76862800
H	2.99717000	-2.96563400	1.19337800
H	4.02503400	-3.76403600	-0.00187500
C	1.92062300	-2.92313100	-1.35745100
H	1.14270400	-3.15017300	-0.62210700
H	1.44424900	-2.47709400	-2.23437100
H	2.36977800	-3.87331600	-1.66735000
C	3.45030000	0.88862000	0.26128200
C	3.83319000	1.65486200	-1.01442500
H	4.55871200	2.43616400	-0.76030300
H	4.28653900	1.01137400	-1.77322800
H	2.95267700	2.13776100	-1.44919200
C	4.69946400	0.29873500	0.92704700
H	5.27679100	-0.34633200	0.26257800
H	5.35291000	1.12687700	1.22509400
H	4.44558000	-0.26360700	1.83094000
C	2.83645700	1.89017600	1.25068300
H	1.99051500	2.43178400	0.82925200
H	2.51669600	1.41299800	2.18051700
H	3.61327500	2.62144900	1.50348400
H	-2.85478100	0.87736200	1.49297700
H	-4.82549100	-0.20984600	2.48831600
H	-4.83770000	-2.66387800	2.87422100
H	-2.83305100	-4.00571700	2.27750800

H	-0.86128200	-2.91660700	1.31077000
H	0.67104300	-0.23417200	-1.00496800
C	-0.39081800	1.38027500	0.68583400
C	-0.18716600	2.23361700	-0.40493000
C	-0.48568600	1.99295000	1.94805500
C	-0.08490800	3.61742600	-0.25887700
H	-0.09178900	1.81101500	-1.39957800
C	-0.37814900	3.37014600	2.11241600
H	-0.64804000	1.36799500	2.82517200
C	-0.17631700	4.19219200	1.00427700
H	0.07006300	4.24368600	-1.13298600
H	-0.45431400	3.80534400	3.10474100
H	-0.09531700	5.26794400	1.12663700
C	-4.45855100	0.71748200	-2.71671400
C	-4.42456100	-0.33061500	-1.80280600
C	-3.20934800	-0.81799900	-1.32996300
C	-1.99551400	-0.25906100	-1.74798600
C	-2.04809500	0.76779300	-2.69891300
C	-3.25970500	1.25835400	-3.17263900
H	-5.40671000	1.09797000	-3.08303900
H	-5.34868500	-0.78069300	-1.45283700
H	-3.20510700	-1.64898100	-0.63166100
H	-1.12233800	1.17847700	-3.09450400
H	-3.26515800	2.05894800	-3.90609100
C	-0.67029100	-0.80853200	-1.27317000
H	-0.76372100	-1.85709700	-0.97706400
H	-0.06251300	-0.85990200	-2.19187900

P9_P

C	-0.92724100	-0.85271900	-0.80691900
H	-0.84335200	-1.94495700	-0.81400100
H	-1.15443400	-0.53253400	-1.82968300
B	0.56182200	-0.18259500	-0.29715300
C	1.66555400	-0.57393700	-1.42966800
C	2.88678200	0.12381800	-1.48208500
C	1.53161900	-1.64025000	-2.32960600
C	3.90377100	-0.21952600	-2.36642600
C	2.54174000	-1.99896300	-3.22368900
C	3.73571800	-1.28775000	-3.24610700
P	-2.33815200	-0.45754500	0.21971100
C	-3.11430900	-2.00713800	0.90911500
C	-4.34535000	-1.69082200	1.76642500
H	-5.18633100	-1.32779900	1.17073100
H	-4.66761400	-2.61142800	2.26418500
H	-4.12165900	-0.95482000	2.54529900
C	-3.48203800	-2.99498600	-0.20675300
H	-4.28596200	-2.62635700	-0.84618700
H	-2.62255200	-3.23725000	-0.83688100
H	-3.82571600	-3.92557800	0.25681500
C	-2.04095900	-2.64508200	1.80854400
H	-1.12979300	-2.89212900	1.25873300
H	-1.76563600	-1.99135300	2.64145500
H	-2.44707900	-3.57255500	2.22539900
C	-3.54650600	0.73963100	-0.54724300
C	-4.29435000	1.48882600	0.56600900
H	-4.96945700	2.21412300	0.10008100
H	-4.89629400	0.83505400	1.19928900
H	-3.59260000	2.04588400	1.19478800
C	-4.52239100	-0.00336100	-1.46786400
H	-5.20320200	-0.65652000	-0.91623800
H	-5.13083900	0.73700200	-1.99689900
H	-3.99635300	-0.59861800	-2.22164800
C	-2.76801800	1.77941700	-1.36743600
H	-2.09712200	2.37278200	-0.74340000
H	-2.18146400	1.33126200	-2.17227800
H	-3.50107200	2.45519200	-1.82173500
H	3.04454500	0.95321800	-0.79508700
H	4.83280600	0.34363800	-2.36585000
H	4.52611300	-1.56051100	-3.93905400
H	2.39243900	-2.83375700	-3.90329700
H	0.61372400	-2.22519200	-2.34658200
C	0.36480900	1.43492200	-0.16800300
C	-0.07242900	2.07001000	1.00731500
C	0.52806900	2.27362100	-1.28590100
C	-0.35480900	3.43682200	1.06760100
H	-0.18659900	1.48540500	1.91866600
C	0.25943800	3.63904900	-1.24416600
H	0.87979800	1.83487800	-2.21705700
C	-0.19305600	4.22956800	-0.06451800
H	-0.68574000	3.88262700	2.00196400
H	0.40185000	4.24668700	-2.13357300
H	-0.40327900	5.29410100	-0.02607800
C	4.98261500	-0.02589600	2.46597100
C	4.73704000	-1.09644100	1.60909900
C	3.43942500	-1.38416500	1.20148700
C	2.35085400	-0.61499300	1.63391000
C	2.61587000	0.45289400	2.49832000
C	3.91375500	0.74716800	2.90884000

H	5.99474600	0.20173300	2.78537700
H	5.56006300	-1.70817600	1.25166000
H	3.25898500	-2.20678700	0.51356500
H	1.79390400	1.06681700	2.85502800
H	4.08814300	1.58361000	3.57935800
C	0.96361300	-0.91779300	1.13651900
H	0.89296700	-2.00386000	0.97951600
H	0.24184200	-0.67686500	1.93425800
H	-1.91989500	0.20762200	1.38282900

P10_TS

B	1.45049100	0.26568100	-0.10401500
C	0.58352800	0.65616000	-1.44171400
C	1.37119800	0.84911700	-2.59385700
H	2.41750400	0.54916200	-2.56909000
C	0.86909200	1.44657400	-3.74024400
H	1.50996100	1.57307400	-4.60736800
C	-0.43907700	1.93829900	-3.76035900
H	-0.81347600	2.47123400	-4.62812200
C	-1.25696900	1.72290000	-2.66450600
H	-2.27937200	2.08841100	-2.66784100
C	-0.77119900	1.02599000	-1.54930300
C	2.22117300	-1.16579700	-0.15744800
C	1.82407400	-2.23166400	-0.95749500
F	0.71627200	-2.13977300	-1.71120700
C	2.46022000	-3.46738400	-0.97982400
F	2.01295100	-4.44595900	-1.76627200
C	3.55821900	-3.68129700	-0.16299100
F	4.18303600	-4.85434800	-0.16665100
C	3.98577400	-2.66119300	0.67569800
F	5.03013400	-2.85466400	1.47858700
C	3.31560300	-1.44808700	0.66138200
F	3.77396800	-0.51326700	1.51139700
C	2.43587900	1.57516600	0.03944300
C	3.77724400	1.62776400	-0.33186500
F	4.39937200	0.54547400	-0.82319800
C	4.54502500	2.78534100	-0.27322100
F	5.82671500	2.76560800	-0.63521600
C	3.96718700	3.97269900	0.14956800
F	4.68747600	5.08896900	0.21035200
C	2.62218900	3.98539300	0.48783200
F	2.03703000	5.12166500	0.86499300
C	1.89897500	2.80433200	0.40906000
F	0.58246000	2.89544500	0.68578100
H	-0.27832800	0.32166000	0.71079100
C	-3.18228800	1.60102400	0.11789800
C	-4.30139400	1.65927200	-0.74324700
C	-3.15103000	2.41934600	1.26947800
C	-5.31121700	2.58624600	-0.47672800
C	-4.18981300	3.32144900	1.49318400
C	-5.27052600	3.43596100	0.62318600
C	-2.56275700	-1.19747900	-0.56939100
C	-2.43530400	-1.91949100	-1.78197500
C	-3.30164700	-1.76128700	0.50051100
C	-3.03144500	-3.18118800	-1.86661500
C	-3.89104700	-3.01490700	0.34473700
C	-3.75917400	-3.75030600	-0.82743200
P	-1.79017900	0.43385800	-0.17985600
C	-6.35650800	4.44996900	0.86580000
H	-6.07817300	5.41981700	0.44088800
H	-7.29699500	4.14268300	0.40321600
H	-6.52863800	4.59658400	1.93472600
C	-2.05686600	2.34110700	2.30551200
H	-1.86459500	1.30769900	2.61247300
H	-1.12145300	2.76199900	1.93369900
H	-2.34494500	2.90465500	3.19507100
C	-4.52767700	0.72845900	-1.91089300
H	-4.94696600	-0.22332500	-1.56739700
H	-5.23340200	1.17705600	-2.61353200
H	-3.61360100	0.49258000	-2.45253600
H	-6.16427500	2.63171500	-1.14979100
H	-4.15453700	3.94794600	2.38119900
H	-4.45563300	-3.43067500	1.17611000
H	-2.92747600	-3.73503700	-2.79650200
C	-4.35755200	-5.12577400	-0.95948000
H	-5.23354100	-5.23998700	-0.31684700
H	-4.65667200	-5.33000400	-1.99024600
H	-3.63142100	-5.89252000	-0.66989400
C	-3.48792300	-1.07516900	1.83280600
H	-4.13770000	-0.19801100	1.75532000
H	-3.93522000	-1.76845700	2.54739500
H	-2.52933900	-0.75425100	2.25366200
C	-1.76686600	-1.43281700	-3.05007900
H	-2.35930200	-0.65420800	-3.54076100
H	-0.76879000	-1.03097700	-2.89747000
H	-1.68727500	-2.26669000	-3.75015600
C	-1.07568200	-3.22004700	3.64176800
C	-0.69409500	-2.08786300	4.35974200

C	-0.10935200	-1.01126100	3.70178400
C	0.10522200	-1.04247700	2.31837000
C	-0.28420800	-2.18074500	1.61003500
C	-0.86830400	-3.26224500	2.26721100
H	-1.53513500	-4.06055900	4.15186200
H	-0.85456100	-2.04195000	5.43222700
H	0.18347300	-0.12723900	4.26329500
H	-0.17585200	-2.21513800	0.53100500
H	-1.17892400	-4.12865400	1.69164200
C	0.71654000	0.17216100	1.64935300
H	0.34782500	1.09034500	2.11784400
H	1.77898600	0.18493000	1.91180200

P10_P

P	-2.80376500	-0.39982200	-0.45597600
B	2.49065900	-0.72632300	-0.21705500
C	-1.82108300	-0.81675000	-1.95974500
H	-1.66815900	0.16852900	-2.41245200
H	-2.45681800	-1.39009500	-2.63852300
C	-0.44829300	-1.46742800	-1.69029000
H	-0.47747400	-2.53001400	-1.94884500
H	-0.21844100	-1.41354300	-0.62670300
C	0.69939000	-0.79304500	-2.44082400
H	0.69970600	-1.13759500	-3.48420500
H	0.53190100	0.28639000	-2.47842800
C	2.05690500	-1.08084200	-1.79157000
H	2.25845700	-2.15969100	-1.89724900
H	2.79809700	-0.59898300	-2.44057300
C	-3.34930500	1.31032600	-0.68397800
C	-4.09833700	1.63281900	-1.83385200
C	-4.47814600	2.95891500	-2.02762300
H	-5.06613900	3.21134100	-2.90660000
C	-4.12330400	3.96547400	-1.13115500
C	-3.39557000	3.61337300	0.00320100
H	-3.11935900	4.38350600	0.71835900
C	-3.00452000	2.30076300	0.25761000
C	-4.52618000	0.61135200	-2.86315500
H	-3.73868200	0.44037700	-3.60372300
H	-4.78988200	-0.35336500	-2.41926000
H	-5.40417900	0.97276800	-3.40144400
C	-4.49970200	5.39935900	-1.38880400
H	-4.70763600	5.92724200	-0.45554800
H	-3.67732500	5.92234300	-1.88706300
H	-5.37897100	5.47057000	-2.03242000
C	-2.28447600	1.99753100	1.54507600
H	-2.84812600	1.29937400	2.17375200
H	-1.30653600	1.54614300	1.37009100
H	-2.12997000	2.91487000	2.11439200
C	-4.20877000	-1.52447700	-0.10573200
C	-4.09704700	-2.89538300	-0.43281600
C	-5.19882200	-3.72943600	-0.24691300
H	-5.09801500	-4.78226500	-0.49788100
C	-6.40514100	-3.26194900	0.26081600
C	-6.47332000	-1.92300100	0.63512200
H	-7.38889100	-1.54849000	1.08554000
C	-5.40653800	-1.03916200	0.47784600
C	-2.83113600	-3.54102500	-0.93673200
H	-2.84301500	-4.60470100	-0.69054500
H	-2.73029900	-3.46185000	-2.02360000
H	-1.94123800	-3.11204300	-0.47628600
C	-7.59396400	-4.17109100	0.41286500
H	-7.28525000	-5.21321300	0.51661400
H	-8.19180600	-3.89754600	1.28501600
H	-8.24150900	-4.10166600	-0.46707000
C	-5.62154500	0.36375700	0.99960400
H	-6.49396400	0.36853800	1.65494100
H	-4.77211400	0.72044500	1.58410200
H	-5.79750600	1.08676400	0.19898000
C	2.21384000	0.83970600	0.30131900
C	1.04128100	1.56014100	0.09731300
F	-0.04589800	0.98724000	-0.49324200
C	0.82727100	2.87599800	0.49282100
F	-0.32268900	3.50352600	0.19964600
C	1.79891400	3.53540300	1.22303700
F	1.61024500	4.79108500	1.62980800
C	2.96502500	2.85266600	1.52829400
F	3.90941000	3.45269500	2.25336000
C	3.13863800	1.54798700	1.07873500
C	4.14090500	-0.96703400	-0.35878200
C	4.91205400	-0.03500100	-1.05726500
F	4.33625900	1.09385500	-1.51598500
C	6.26434300	-0.16615300	-1.32744200
F	6.92871200	0.78346300	-1.99307600
C	6.93138000	-1.30716300	-0.90372300
F	8.23269800	-1.46457100	-1.15343900
C	6.21882700	-2.27815900	-0.22537500
F	6.83538700	-3.39287500	0.18003500
C	4.85909300	-2.09518900	0.02374700

F	4.27510500	-3.12323700	0.66972400
F	4.29024300	0.98364700	1.46757200
H	-1.91244100	-0.41833900	0.62619900
C	-1.88289700	-1.48157500	3.05674500
C	-0.92414300	-0.47890700	3.19988100
C	0.28325000	-0.53863400	2.50572700
C	0.58934000	-1.61482600	1.65517600
C	-0.38349600	-2.62516900	1.54739400
C	-1.59902000	-2.56503700	2.22559300
H	-2.81982100	-1.43476300	3.60364400
H	-1.11323000	0.35878700	3.86618300
H	1.02246600	0.24386100	2.65312400
H	-0.15629300	-3.49163500	0.92926800
H	-2.32010600	-3.37193300	2.11774300
C	1.92564300	-1.75806200	0.98200200
H	2.67206500	-1.68727000	1.78407400
H	2.00036900	-2.78377400	0.60678300

P11_TS

P	-1.91274000	-0.61059300	0.02426000
B	1.16348400	-0.33363900	-0.29527700
C	-1.13342400	-0.81482600	-1.65280100
H	-1.06527400	0.16767100	-2.12092600
C	-1.89299200	-1.76747000	-2.58404700
H	-2.91037900	-1.40318300	-2.76907600
H	-2.00130100	-2.74089600	-2.08763700
C	-1.14919000	-1.95927600	-3.90524700
H	-1.12095400	-1.00496200	-4.44956400
H	-1.69334000	-2.66974600	-4.53644700
C	0.27880200	-2.43281600	-3.64987700
H	0.25492700	-3.42409200	-3.17627800
H	0.81745000	-2.54511600	-4.59681500
C	1.01891000	-1.45445900	-2.73818700
H	2.04833500	-1.79151600	-2.59774400
H	1.07993600	-0.48300900	-3.24166900
C	0.31842600	-1.27896700	-1.37503500
H	0.25327600	-2.28675300	-0.93854000
C	-3.15897400	-1.96846100	0.22252900
C	-2.77660400	-3.14632600	0.90480800
C	-3.69480300	-4.18048000	1.06733200
H	-3.38065800	-5.08173800	1.58880900
C	-4.99765300	-4.09279200	0.58292300
C	-5.35898900	-2.93196800	-0.08821600
H	-6.36674300	-2.84437300	-0.48749800
C	-4.47507500	-1.86578900	-0.27938300
C	-1.40053500	-3.33518600	1.48939300
H	-1.31585000	-4.31825400	1.95600800
H	-0.61891100	-3.25958800	0.72816200
H	-1.19107700	-2.58082300	2.25426900
C	-5.98491000	-5.20412100	0.82116500
H	-6.39941300	-5.13672200	1.83268000
H	-6.81739100	-5.15791600	0.11627200
H	-5.50639100	-6.18209800	0.72872700
C	-5.02826600	-0.66414000	-1.00709700
H	-4.30082400	-0.20452800	-1.67514400
H	-5.89264200	-0.96216100	-1.60422000
H	-5.35104400	0.11077500	-0.30435300
C	-2.85088400	0.94653500	0.32342200
C	-3.28576600	1.10815100	1.66042700
C	-3.97010200	2.26365100	2.02790300
H	-4.29796900	2.37149700	3.05927300
C	-4.22030700	3.29024500	1.12229200
C	-3.78248900	3.11887500	-0.18526600
H	-3.96444900	3.90890400	-0.91012000
C	-3.10200700	1.97451100	-0.60842500
C	-3.01068300	0.08041900	2.73200900
H	-3.33584100	0.45234900	3.70505200
H	-3.52308100	-0.86740200	2.54031300
H	-1.93695300	-0.12868300	2.79824400
C	-4.87891400	4.56887200	1.56405300
H	-4.16700600	5.18593800	2.12243600
H	-5.22802600	5.15423600	0.71111800
H	-5.72925200	4.37090000	2.22126900
C	-2.66582500	1.95644700	-2.05436500
H	-1.58503700	2.11241400	-2.14447300
H	-2.90911700	1.02147700	-2.56635000
H	-3.15700900	2.76642500	-2.59688900
C	2.76281800	-0.65803600	-0.36658800
C	3.77696500	0.29523000	-0.26532000
F	3.49942000	1.60120500	-0.16039900
C	5.13053100	-0.01847400	-0.21899500
F	6.04273400	0.94619400	-0.11143800
C	5.53138800	-1.34308900	-0.27581400
F	6.82155800	-1.66293700	-0.22835500
C	4.56554900	-2.33236100	-0.37074300
F	4.92530400	-3.61613000	-0.40733100
C	3.22659000	-1.97207900	-0.40824900
F	2.35674200	-3.00283900	-0.42799100

C	0.79737300	1.26243700	-0.44781900
C	1.23782000	1.95534200	-1.58113700
F	2.05254600	1.34941700	-2.45692500
C	0.94010300	3.28166000	-1.85305800
F	1.41471500	3.87565300	-2.94639400
C	0.15010800	4.00213200	-0.96368600
F	-0.15962300	5.27142300	-1.21244600
C	-0.29390200	3.37905900	0.18790200
F	-1.04946400	4.03969000	1.06351000
C	0.04498000	2.04801300	0.41861000
F	-0.40586300	1.55772200	1.59313900
H	-0.25006700	-0.60503600	0.78812700
C	4.50974100	0.41376600	3.57584800
C	4.29822100	-0.92956800	3.27284300
C	3.12332900	-1.32243500	2.64356200
C	2.14020400	-0.38965500	2.28993100
C	2.34456900	0.94690800	2.63601100
C	3.52336000	1.34260800	3.26535500
H	5.43058600	0.73027000	4.05458000
H	5.05127600	-1.67250100	3.51755900
H	2.97395300	-2.36970700	2.39110800
H	1.59241300	1.69066500	2.41192700
H	3.66955700	2.39077700	3.50662300
C	0.91586700	-0.90345400	1.54676700
H	1.01122300	-1.98435700	1.42775200
H	0.08395200	-0.74842100	2.25317700

P11_P

B	1.73801300	2.41158900	-1.06605100
C	0.21784300	2.62711400	-1.59293600
C	-0.19830600	3.71566300	-2.37717800
H	0.54570100	4.45839600	-2.65211300
C	-1.51003200	3.86131800	-2.81587800
H	-1.78334100	4.71774700	-3.42619900
C	-2.48559400	2.91972900	-2.47873000
H	-3.50981600	3.04045400	-2.81524000
C	-2.13146500	1.82792100	-1.69942800
H	-2.88069100	1.09488600	-1.41146000
C	-0.79777000	1.69855800	-1.28763400
H	0.44679400	0.80868900	0.80148000
C	-1.82170200	-0.35825200	0.53586600
C	-2.61128700	-1.33653900	-0.09830700
C	-2.19719400	0.15654700	1.79528600
C	-3.75198200	-1.80138700	0.55774700
C	-3.34284000	-0.34461400	2.40839700
C	-4.12858400	-1.32924300	1.81162600
C	0.66230500	-1.06897800	-0.90991300
C	0.91320600	-1.26176200	-2.28363300
C	1.17094100	-1.96237200	0.05545100
C	1.62138700	-2.39947200	-2.66321800
C	1.88050700	-3.08003600	-0.38581900
C	2.10568000	-3.32232500	-1.73671500
P	-0.31922800	0.30887400	-0.25165800
C	-5.34613700	-1.87016400	2.51301700
H	-6.03323900	-2.34254700	1.80799400
H	-5.05950900	-2.62233400	3.25499400
H	-5.88258200	-1.07717400	3.03960500
C	-1.39984900	1.22367800	2.50433700
H	-1.21062900	2.09153600	1.86268700
H	-1.93907200	1.57337600	3.38625300
H	-0.42692400	0.84271200	2.83640400
C	-2.29315400	-1.89984100	-1.46243000
H	-1.48171000	-2.63218600	-1.41625600
H	-3.17521400	-2.39233500	-1.87614300
H	-1.99006100	-1.11986100	-2.16727800
H	-4.36597700	-2.55215000	0.06632100
H	-3.63346500	0.05276400	3.37787100
H	2.27201700	-3.77386300	0.35376600
H	1.81362700	-2.56100800	-3.72121400
C	2.88943500	-4.52462800	-2.19249400
H	3.91012000	-4.23706200	-2.46390700
H	2.95252300	-5.27929600	-1.40584500
H	2.43265100	-4.98199200	-3.07386000
C	1.01394000	-1.74619000	1.54214700
H	-0.03626000	-1.74365800	1.85221200
H	1.52152800	-2.54162300	2.09013800
H	1.47074400	-0.79937600	1.85513400
C	0.51407700	-0.27809100	-3.35334400
H	-0.55002400	-0.02877400	-3.34080200
H	1.06308900	0.65940100	-3.21627700
H	0.75508200	-0.68407100	-4.33738300
H	1.96519200	1.20510500	-1.12819900
H	2.51344300	2.98222500	-1.80946100
C	3.65912500	0.09439400	3.30090000
C	2.64581300	0.95356800	3.71747500
C	2.08912700	1.86376900	2.81904200
C	2.51273500	1.93890400	1.48336300
C	3.53111700	1.05913600	1.08494700

C	4.10100200	0.15743800	1.97993100
H	4.09945200	-0.61407900	3.99559100
H	2.29215600	0.92318700	4.74430300
H	1.31393900	2.54799000	3.15946700
H	3.86724800	1.09004000	0.05346900
H	4.89111300	-0.50667500	1.64128000
C	1.89819500	2.92421200	0.51581200
H	0.93974900	3.25830000	0.94413900
H	2.52748500	3.82387600	0.51753400

P12_TS

P	2.12270500	0.96013800	0.49659500
C	1.75997700	1.27956300	2.25741900
H	2.64517300	1.11071100	2.88086800
H	1.49663400	2.34287600	2.31781400
C	0.55649500	0.43475200	2.70238700
H	0.81772800	-0.62862700	2.69546100
H	0.36878000	0.68420900	3.75312400
C	-0.73662500	0.65614000	1.90055400
H	-0.98988000	1.72475000	1.92184000
H	-1.51435000	0.17907100	2.49962500
B	-0.94612500	0.06170200	0.38437100
C	3.09910700	2.35320500	-0.14684200
C	3.06138600	2.60850400	-1.52323600
H	2.47242300	1.96993800	-2.17873900
C	3.77152000	3.67958000	-2.05688900
H	3.73754500	3.87215200	-3.12393700
C	4.51066300	4.50928400	-1.21813100
H	5.05669500	5.35027200	-1.63306900
C	4.54596200	4.26538700	0.15291200
H	5.12293200	4.91237700	0.80549500
C	3.84393200	3.19057300	0.68905700
H	3.87796600	3.00338500	1.75848800
C	3.21024400	-0.49946700	0.39806800
C	3.27183600	-1.42519700	1.44347800
H	2.75330200	-1.24071100	2.37808200
C	3.99435000	-2.60551000	1.28875700
H	4.02517300	-3.32487400	2.10090700
C	4.65553900	-2.87109400	0.09358700
H	5.19879100	-3.80171200	-0.03165300
C	4.61448900	-1.94050100	-0.94223100
H	5.12746400	-2.14186000	-1.87657100
C	3.89477500	-0.76073100	-0.79447500
H	3.85680900	-0.04868800	-1.61370500
C	-0.10474000	-1.32451600	0.11624800
C	-0.20591400	-2.35440500	1.05017900
F	-0.97322500	-2.19486000	2.14069800
C	0.46429400	-3.56708200	0.94687700
F	0.34563800	-4.49584900	1.89636700
C	1.26802200	-3.80599100	-0.15724300
F	1.94498100	-4.94654400	-0.26614800
C	1.38100000	-2.82879900	-1.13319900
F	2.16120500	-3.03420200	-2.19536400
C	0.70098000	-1.62922900	-0.97645900
F	0.91974800	-0.71032900	-1.93953000
C	-2.53360500	-0.22525700	0.05622700
C	-3.62159300	0.21086800	0.81394400
F	-3.46576000	0.99550700	1.88743600
C	-4.94585400	-0.08496200	0.50190000
F	-5.93563700	0.36448500	1.27370800
C	-5.23973500	-0.83051700	-0.62758900
F	-6.50060200	-1.11027800	-0.94430100
C	-4.19935600	-1.26260400	-1.43688700
F	-4.46014900	-1.95646700	-2.54456800
C	-2.89456300	-0.95283500	-1.08107100
F	-1.94746100	-1.35414300	-1.94784300
H	0.55571100	0.93781600	-0.23039200
C	-2.36022300	5.05205400	0.39296200
C	-3.16372700	4.07617000	-0.18859100
C	-2.60289100	2.89108500	-0.65661000
C	-1.22993400	2.65277000	-0.54727700
C	-0.42966400	3.65649600	0.01352600
C	-0.98681600	4.84099800	0.48392400
H	-2.79747000	5.97437700	0.76115300
H	-4.23312400	4.23674100	-0.28310000
H	-3.23646100	2.14135500	-1.12184200
H	0.64941100	3.52004100	0.06428300
H	-0.34503900	5.60368500	0.91435000
C	-0.61719500	1.35820000	-0.99272100
H	-1.22169800	0.81668700	-1.72003800
H	0.27408500	1.59796200	-1.58864300

P12_P

P	2.51904300	1.00515200	0.26251700
C	1.64995700	1.55587600	1.75487600
H	2.47094300	1.81508800	2.43689900
H	1.17268500	2.50236800	1.47172500
C	0.62777200	0.60906800	2.41372400

H	0.99791200	-0.42045000	2.38345000
H	0.62683000	0.89066400	3.47381000
C	-0.80319100	0.67876400	1.86884700
H	-1.16715500	1.70651200	2.00350700
H	-1.39313600	0.07899400	2.56414500
B	-1.11481100	0.19171600	0.32137900
C	3.62883700	2.33282400	-0.24612900
C	3.07154100	3.44920100	-0.88079200
H	2.00606500	3.48660500	-1.09966900
C	3.89212000	4.51228800	-1.23899700
H	3.46635600	5.37748600	-1.73507600
C	5.25813100	4.45996300	-0.96602100
H	5.89605000	5.29031700	-1.24991900
C	5.80995000	3.34715000	-0.33654400
H	6.87420200	3.30896100	-0.13126800
C	4.99731300	2.27708700	0.02595900
H	5.42426600	1.40143200	0.50661600
C	3.44304900	-0.51566700	0.49464300
C	3.60096300	-1.09605000	1.75505400
H	3.18965700	-0.62099600	2.63997800
C	4.27505300	-2.31000200	1.86852000
H	4.38314600	-2.77429300	2.84230800
C	4.78966500	-2.93144000	0.73464700
H	5.29356700	-3.88766600	0.82582000
C	4.64574400	-2.34255700	-0.52155000
H	5.02499300	-2.83952700	-1.40734400
C	3.97275200	-1.13464500	-0.64504700
H	3.81840700	-0.70089100	-1.63033000
C	-0.12555300	-1.11104100	-0.04804300
C	0.01704300	-2.15799900	0.86604300
F	-0.63446400	-2.12388200	2.03912600
C	0.83617000	-3.26654900	0.67894200
F	0.96476300	-4.19283500	1.63068100
C	1.53630200	-3.39894900	-0.50918000
F	2.37113400	-4.42169200	-0.70072800
C	1.39838900	-2.41807700	-1.47634900
F	2.12531900	-2.49255200	-2.59863300
C	0.58613900	-1.31942800	-1.22729600
F	0.59695200	-0.38155700	-2.20069800
C	-2.66536200	-0.35946400	0.14875400
C	-3.72684600	-0.09122300	1.01013700
F	-3.55024200	0.61820000	2.13546900
C	-5.03838700	-0.49148500	0.77157100
F	-6.00686800	-0.20073200	1.64348600
C	-5.34571800	-1.18436900	-0.38756200
F	-6.59679200	-1.57367800	-0.63354100
C	-4.33256200	-1.45883500	-1.29415100
F	-4.61075000	-2.11180300	-2.42543700
C	-3.03909700	-1.04116300	-1.01025200
F	-2.12736500	-1.29897100	-1.96868000
C	-3.63387500	4.74702900	0.18598600
C	-4.11115200	3.64105900	-0.51332800
C	-3.26024200	2.58881700	-0.83511800
C	-1.90885000	2.60336100	-0.46218500
C	-1.44851200	3.73182900	0.22631900
C	-2.29224000	4.79014700	0.55140900
H	-4.29835400	5.56751000	0.43652700
H	-5.15339200	3.59796400	-0.81474900
H	-3.64551100	1.73794800	-1.39108500
H	-0.39661500	3.78637800	0.50599400
H	-1.90022800	5.64948500	1.08752000
C	-0.99532400	1.44984500	-0.77686200
H	-1.22406100	1.07174800	-1.77827700
H	0.02304200	1.86280600	-0.84041700
H	1.65369100	0.81903500	-0.81437100

P13_TS

P	2.54815200	-0.56361100	-0.26173300
B	-0.70128400	-0.24505100	-0.61750900
C	2.85809600	0.38446400	-1.80689600
H	2.43314100	1.38645500	-1.67139700
H	3.94200800	0.49148400	-1.92666600
C	2.22088300	-0.29277000	-3.02342000
H	2.62449300	0.19272200	-3.91888200
H	2.54442100	-1.34063100	-3.06952200
C	0.69433900	-0.21662900	-3.07164600
H	0.39839800	-0.62246900	-4.04522400
H	0.40503600	0.83805000	-3.10455000
C	-0.09287100	-0.98546600	-1.98165300
H	0.47889300	-1.88694200	-1.71722800
H	-0.97069600	-1.38760000	-2.49279300
C	3.65571500	0.17836500	0.98406600
C	4.21318400	-0.61926900	1.98866600
H	4.04257700	-1.69237900	1.98769300
C	4.99857500	-0.04506000	2.98365800
H	5.42911500	-0.67273900	3.75704100
C	5.23000600	1.32751700	2.98630000
H	5.84160600	1.77412500	3.76349300

C	4.67180900	2.12789000	1.99263600
H	4.83959800	3.19979500	1.99398500
C	3.88648300	1.55827200	0.99687300
H	3.44326700	2.19940300	0.24011500
C	3.29488600	-2.20178600	-0.57251200
C	4.65392800	-2.31285100	-0.89314900
H	5.27545600	-1.42158500	-0.93051700
C	5.21442600	-3.55939100	-1.14243500
H	6.26742800	-3.63819100	-1.39233200
C	4.42490500	-4.70696300	-1.06665900
H	4.86479600	-5.68014700	-1.25950100
C	3.07743600	-4.60577400	-0.73949500
H	2.46186500	-5.49686600	-0.67686800
C	2.51239900	-3.35589700	-0.49245800
H	1.45858000	-3.28252100	-0.23883300
C	-0.36304000	1.35279000	-0.57781000
C	0.47861100	2.04822600	0.27928000
F	1.05847100	1.43611700	1.33593100
C	0.80905300	3.39283700	0.13276400
F	1.66254700	3.97387300	0.98052400
C	0.25836200	4.11931300	-0.90866000
F	0.56110700	5.40390500	-1.07149100
C	-0.61816500	3.48467800	-1.78060900
F	-1.15919500	4.16740700	-2.78718100
C	-0.90270500	2.14121500	-1.59497400
F	-1.74180600	1.57773200	-2.47521800
C	-2.29923200	-0.57929100	-0.52704800
C	-3.30054900	0.32637600	-0.18531200
F	-3.01667300	1.61067400	0.06884700
C	-4.63866100	-0.02335900	-0.04462000
F	-5.54559600	0.89386000	0.28805400
C	-5.02989200	-1.33679600	-0.24790000
F	-6.30554300	-1.69067200	-0.11150700
C	-4.07130700	-2.28092500	-0.58160100
F	-4.42009500	-3.55870300	-0.74347100
C	-2.74859700	-1.88582000	-0.70859300
F	-1.86273300	-2.87967800	-0.92991500
H	0.84172900	-0.62418800	0.22827900
C	-3.53939000	-0.28258100	3.70495000
C	-2.60478700	0.71263300	3.43997400
C	-1.51581200	0.46011800	2.60911800
C	-1.35122000	-0.79346300	2.01628400
C	-2.27666200	-1.79818400	2.31934300
C	-3.36407800	-1.54755000	3.14859700
H	-4.39273700	-0.07728000	4.34288000
H	-2.72323400	1.69905100	3.87726500
H	-0.79773000	1.24722900	2.42169700
H	-2.15637500	-2.78177500	1.87113500
H	-4.07859600	-2.33930300	3.35213000
C	-0.21295700	-1.11842600	1.06208000
H	0.68508800	-1.04754400	1.69374100
H	-0.28737600	-2.16603500	0.75439400

P13_P

P	2.87514100	-0.66453600	-0.28432300
B	-0.99927600	-0.54573800	-0.24788400
C	2.56145100	-0.09197100	-1.98285400
H	2.02609000	0.86310300	-1.90419400
H	3.56265900	0.11770100	-2.37935500
C	1.80256700	-1.07526600	-2.89366300
H	2.04993100	-0.78741800	-3.92112900
H	2.20238200	-2.08714500	-2.74980100
C	0.28379600	-1.07268700	-2.70994500
H	-0.12303000	-1.72369100	-3.49138500
H	-0.08634100	-0.07383900	-2.95052700
C	-0.20112500	-1.54942900	-1.32211600
H	0.65754800	-2.00747400	-0.80274600
H	-0.85629000	-2.40754300	-1.47399600
C	4.09130500	0.41606400	0.49242100
C	4.82986000	-0.06541400	1.58029700
H	4.69332400	-1.08592300	1.92647800
C	5.74396700	0.76814600	2.21151500
H	6.31540300	0.39878900	3.05592000
C	5.92397400	2.07489400	1.75998900
H	6.63644400	2.72407400	2.25794400
C	5.19239600	2.55214200	0.67723700
H	5.32439800	3.57143700	0.33206600
C	4.27494300	1.72446800	0.03818100
H	3.69650300	2.11064000	-0.79596700
C	3.50313700	-2.34756200	-0.25697100
C	4.72005000	-2.64980100	-0.87838200
H	5.29603000	-1.87018700	-1.37078100
C	5.19585100	-3.95452200	-0.85197800
H	6.13652300	-4.19654900	-1.33429800
C	4.46426000	-4.94929300	-0.20283600
H	4.83960400	-5.96706000	-0.18413700
C	3.25816100	-4.64549200	0.42158900
H	2.69249300	-5.42074600	0.92623400

C	2.76935000	-3.34209000	0.39614700
H	1.82125600	-3.10291600	0.87217500
C	-0.29997300	0.97363000	-0.32027800
C	0.61506900	1.53463100	0.56773200
F	0.99618900	0.89068700	1.70036200
C	1.25439800	2.76144300	0.38950400
F	2.13387500	3.20838800	1.29120700
C	0.99743600	3.50620300	-0.74707500
F	1.62120100	4.66599400	-0.95526900
C	0.06951100	3.02194000	-1.66085000
F	-0.21870700	3.73635800	-2.74786800
C	-0.55141800	1.80401600	-1.42058600
F	-1.46110300	1.44562300	-2.33856400
C	-2.62578200	-0.45437700	-0.55931600
C	-3.40588800	0.68951600	-0.38361400
F	-2.84513700	1.87137900	-0.07067400
C	-4.79138600	0.72204200	-0.48461000
F	-5.45738700	1.86697200	-0.31744000
C	-5.48755100	-0.44521400	-0.74969500
F	-6.81729800	-0.43824800	-0.84921000
C	-4.77353600	-1.62192800	-0.89493200
F	-5.41902700	-2.77240800	-1.11020600
C	-3.38857400	-1.60007600	-0.78527900
F	-2.81323600	-2.82243900	-0.83291200
C	-4.00887300	0.05471300	3.93608800
C	-2.84250200	0.79973900	3.78836700
C	-1.82360100	0.35777200	2.94856100
C	-1.94133600	-0.84252500	2.23840400
C	-3.11625600	-1.58614000	2.40963400
C	-4.14041800	-1.14487700	3.24117700
H	-4.80604100	0.40462900	4.58415000
H	-2.72588200	1.73658900	4.32537000
H	-0.92221300	0.95200700	2.84247200
H	-3.22874300	-2.51934000	1.86174400
H	-5.04358800	-1.73908800	3.34477700
C	-0.90479500	-1.28967800	1.24070200
H	0.09551800	-1.20609600	1.68729800
H	-1.06393600	-2.35858300	1.04111900
H	1.71408300	-0.69751100	0.48463200

P14_TS

P	3.12161500	-0.41812600	-0.02745800
B	-0.20246500	-0.02118100	-0.43682700
C	2.86984800	1.09179800	-1.04728500
H	2.46328200	1.81982800	-0.33913400
H	3.80116000	1.50759100	-1.44546700
C	1.82292000	0.84127600	-2.15119000
H	2.33476500	0.50612300	-3.06221600
H	1.36887200	1.80718400	-2.40139100
C	0.71868400	-0.17159100	-1.80344400
H	1.10086700	-1.18654800	-1.88776400
H	-0.04042000	-0.09093200	-2.59212300
C	4.13757300	-1.64198500	-1.07242300
C	5.24356400	-0.93650900	-1.86993100
H	5.79441500	-1.69038000	-2.44454300
H	5.96192300	-0.40677800	-1.24462400
H	4.81978800	-0.22599500	-2.58590300
C	4.72747800	-2.73328900	-0.16650900
H	3.94324000	-3.24332800	0.40256200
H	5.47539200	-2.35813100	0.53273500
H	5.21665200	-3.48253600	-0.79848000
C	3.23197300	-2.35958500	-2.08602300
H	2.83975100	-1.67881800	-2.84414600
H	2.39626400	-2.87165900	-1.60542600
H	3.83811600	-3.11099000	-2.60463100
C	4.15188700	0.13922400	1.46939800
C	3.60067800	1.47154000	2.00502000
H	2.54265800	1.42958400	2.26335100
H	3.74890400	2.29384700	1.30063600
H	4.15139000	1.71838300	2.91931700
C	5.63678000	0.37710700	1.16315100
H	6.11766200	0.76823800	2.06728000
H	5.76882700	1.12275700	0.37274200
H	6.17301300	-0.52900500	0.87842300
C	3.99602600	-0.90969900	2.58324600
H	4.39009300	-1.88829300	2.30458600
H	2.94543000	-1.03394200	2.86283700
H	4.54187400	-0.57187800	3.47139600
C	-0.67668800	1.53920700	-0.46366700
C	-0.11283100	2.59559600	0.23605300
F	0.84187100	2.37087400	1.17076000
C	-0.44805400	3.93289800	0.05751100
F	0.13278200	4.87837800	0.79712900
C	-1.37918800	4.27380500	-0.90751600
F	-1.71691000	5.54515200	-1.10471400
C	-1.94469700	3.26533900	-1.67605000
F	-2.81981400	3.57338600	-2.63103600
C	-1.57596000	1.94685600	-1.45363600

F	-2.12271700	1.04192400	-2.27920000
C	-1.36071700	-1.16488300	-0.53312200
C	-1.00762300	-2.50261800	-0.68666900
F	0.29580800	-2.86286500	-0.70286300
C	-1.91702100	-3.55001000	-0.74659200
F	-1.50110200	-4.80882700	-0.89531700
C	-3.27092300	-3.27340600	-0.63377800
F	-4.16447500	-4.25781200	-0.68613700
C	-3.67721600	-1.96164900	-0.44221600
F	-4.97115200	-1.68726000	-0.28477000
C	-2.72863300	-0.94867900	-0.37953500
F	-3.20211800	0.26567900	-0.08405300
H	1.30309100	-0.45870500	0.28521800
C	-3.33027500	-0.15515900	3.65219600
C	-2.56406000	0.97916200	3.40779900
C	-1.38387000	0.88929300	2.67459500
C	-0.95502500	-0.33607200	2.15832700
C	-1.71379300	-1.47610700	2.44668200
C	-2.89232100	-1.38915500	3.17870300
H	-4.25321100	-0.08246300	4.21811800
H	-2.88166600	1.94357000	3.79144200
H	-0.78292600	1.77770800	2.52359100
H	-1.38077700	-2.44412900	2.07948900
H	-3.46707400	-2.28787600	3.37973300
C	0.34510400	-0.47222700	1.37811500
H	1.08059800	0.15175900	1.88741700
H	0.66489100	-1.51690300	1.46553800

P14_P

P	3.51758200	-0.82150300	-0.08592000
B	-0.40075900	-0.07754500	-0.38532100
C	2.76195700	0.70404600	-0.72684100
H	2.22251700	1.11696300	0.12895400
H	3.59519300	1.38246600	-0.95184000
C	1.79517200	0.55909800	-1.91271000
H	2.36050200	0.28730200	-2.81405200
H	1.41477000	1.57018500	-2.09792700
C	0.62384200	-0.39607200	-1.66256800
H	1.00339600	-1.42131800	-1.56850400
H	0.02702100	-0.40064100	-2.58375200
C	4.45887100	-1.74138900	-1.40157800
C	5.28182600	-0.74709200	-2.23332300
H	5.81364600	-1.30468000	-3.01061500
H	6.02786600	-0.21658200	-1.63601700
H	4.64314700	-0.01229300	-2.73073400
C	5.36992700	-2.81120400	-0.78315100
H	4.80696400	-3.51877900	-0.16686500
H	6.17981200	-2.38745300	-0.18619300
H	5.82585400	-3.37840300	-1.60068000
C	3.43598900	-2.45582100	-2.30477000
H	2.77376700	-1.76410200	-2.82548400
H	2.81813900	-3.16180800	-1.74199600
H	3.99461900	-3.02252900	-3.05678100
C	4.47501400	-0.40510300	1.45928700
C	3.65555600	0.61268800	2.27509700
H	2.65348000	0.24929700	2.51724600
H	3.55915900	1.57673700	1.77167300
H	4.18621000	0.78019500	3.21766800
C	5.83162600	0.22141300	1.10800000
H	6.31137100	0.54211700	2.03797100
H	5.72076700	1.10790400	0.47537100
H	6.50779600	-0.47976100	0.61386200
C	4.65203900	-1.67302600	2.30931400
H	5.26207800	-2.43471800	1.82236500
H	3.68489000	-2.11092200	2.57417400
H	5.15399500	-1.39311300	3.24075800
C	-0.55531300	1.57140900	-0.33947700
C	0.09063400	2.45395900	0.51626800
F	0.89894800	2.00538900	1.51774800
C	0.01777200	3.84204300	0.43322100
F	0.68210600	4.60994200	1.30229000
C	-0.73313300	4.42115200	-0.57303600
F	-0.81529900	5.74660200	-0.68383700
C	-1.38873400	3.59444900	-1.47672000
F	-2.10450600	4.13496600	-2.46351600
C	-1.27765300	2.21834400	-1.34686200
F	-1.92060900	1.49200500	-2.27315500
C	-1.84725400	-0.82795900	-0.65870900
C	-1.89178700	-2.15876100	-1.06267100
F	-0.74954300	-2.83377900	-1.33052600
C	-3.05435900	-2.91232900	-1.16682900
F	-3.00788200	-4.19011900	-1.55883900
C	-4.26913900	-2.33020400	-0.84502100
F	-5.40148500	-3.02916800	-0.93720700
C	-4.28661700	-1.01094000	-0.41997900
F	-5.44727500	-0.43759700	-0.09540600
C	-3.09492800	-0.30233000	-0.32901300
F	-3.21156300	0.95377600	0.12870900

C	-3.00977500	-1.03108700	4.00628000
C	-2.28740800	0.14850000	3.85217800
C	-1.25859500	0.23023300	2.91753300
C	-0.92362100	-0.86613300	2.11490900
C	-1.65191400	-2.04949600	2.29290200
C	-2.68473400	-2.13388800	3.22067800
H	-3.81699300	-1.09052500	4.72918800
H	-2.52933400	1.01596900	4.45905500
H	-0.70604000	1.15727800	2.80857300
H	-1.40845400	-2.91340000	1.67781800
H	-3.23751000	-3.06244700	3.32820000
C	0.12420600	-0.77517000	1.03517900
H	1.01205200	-0.27502300	1.43781300
H	0.41740600	-1.80355600	0.77200700
H	2.49914700	-1.68823400	0.33226800

P15-TS

P	1.34182400	-0.72234800	0.20968000
B	-1.27643500	-0.34596800	-0.05521200
C	-0.05718000	-1.10465600	-0.89546400
H	-0.13630200	-2.17174900	-0.63039300
C	-0.01683200	-1.01682400	-2.41789800
H	-0.07472900	0.02037200	-2.74590100
H	-0.93441100	-1.50113900	-2.77272500
C	1.19765600	-1.70131100	-3.03789900
H	2.11983500	-1.16886800	-2.78541500
H	1.12052500	-1.72669400	-4.12759200
H	1.29025600	-2.73439700	-2.68380800
C	2.22784700	0.85712600	0.04463100
C	2.24875800	1.67808900	-1.07739400
F	1.71430200	1.27407000	-2.22711100
C	2.79323000	2.95427600	-1.03886200
F	2.75390600	3.73173700	-2.11319400
C	3.35415700	3.42704500	0.14087500
F	3.87688800	4.64090200	0.18353800
C	3.37166100	2.62182100	1.27405600
F	3.91119300	3.06921500	2.39897500
C	2.80818200	1.35706600	1.21164800
F	2.81530100	0.60984300	2.31541600
C	2.63383700	-2.00242400	0.13019000
C	3.80873400	-1.90992300	-0.61422700
F	4.05232800	-0.84049000	-1.37173400
C	4.75435100	-2.92559100	-0.61350200
F	5.86593000	-2.81271500	-1.32909800
C	4.52761000	-4.07234700	0.13854800
F	5.42273800	-5.04673300	0.14077600
C	3.35947300	-4.20237000	0.87980200
F	3.14176000	-5.30052000	1.59098000
C	2.43632300	-3.16784100	0.86825000
F	1.32007300	-3.31178800	1.58617300
C	-0.97012100	1.26476700	0.00510100
C	-1.17836000	2.02237400	-1.15007300
F	-1.78090600	1.46635900	-2.20984400
C	-0.79057300	3.34606300	-1.29795600
F	-1.00560900	3.99503400	-2.43802800
C	-0.16667100	3.99295400	-0.23862600
F	0.26530800	5.24218500	-0.36889600
C	0.03926700	3.30120000	0.94244600
F	0.68644100	3.88257500	1.95475200
C	-0.36038100	1.97301900	1.03500000
F	-0.02361100	1.36384700	2.19434600
C	-2.75849200	-0.72413000	-0.59232300
C	-3.83977900	0.15894800	-0.56184600
F	-3.68047600	1.42979400	-0.17432400
C	-5.13901500	-0.20521800	-0.88817800
F	-6.12727600	0.68406800	-0.82816200
C	-5.40905400	-1.51095500	-1.26983700
F	-6.64688000	-1.87933000	-1.58261000
C	-4.37175000	-2.42844300	-1.31894500
F	-4.61006700	-3.69036200	-1.67475400
C	-3.08831100	-2.01918500	-0.98254100
F	-2.15110800	-2.98824200	-1.00271200
H	-0.01485900	-0.70786600	1.26632000
C	-5.23893200	-0.09599800	3.09757200
C	-4.95864900	-1.37978300	2.63514300
C	-3.67634000	-1.69956900	2.20453600
C	-2.65457200	-0.74415500	2.21807600
C	-2.93421900	0.52837400	2.71635000
C	-4.21965700	0.84882000	3.14699400
H	-6.24099400	0.16198500	3.42353300
H	-5.73965300	-2.13320700	2.60308200
H	-3.46637100	-2.69819700	1.82920000
H	-2.15079500	1.27422000	2.77290800
H	-4.42214200	1.84805600	3.51914800
C	-1.29660400	-1.13975800	1.66129800
H	-1.29581700	-2.20309400	1.40286600
H	-0.61539800	-1.05819000	2.52956300

P15_P

P	-1.50521600	0.67698200	0.38332000
B	1.34992400	0.50004200	0.22305300
C	-0.02081400	1.28703300	-0.43155600
H	-0.03264100	2.30342700	-0.00249000
C	-0.06242400	1.40502700	-1.96422900
H	0.10248200	0.42606200	-2.41522600
H	0.81370800	2.00398400	-2.22040200
C	-1.30126900	2.06486900	-2.56476200
H	-2.18414600	1.42193800	-2.49536600
H	-1.14307000	2.26914200	-3.62639000
H	-1.52259800	3.01923100	-2.07346900
C	-2.14108400	-0.99165000	0.10132100
C	-2.07765800	-1.64874900	-1.12335300
F	-1.63230600	-1.01336600	-2.20158800
C	-2.46466200	-2.97310000	-1.25550300
F	-2.36364000	-3.58818200	-2.42346000
C	-2.93441500	-3.66182200	-0.14243500
F	-3.29522700	-4.92529900	-0.25721100
C	-3.03074000	-3.02388500	1.08904200
F	-3.48123100	-3.68173700	2.14437800
C	-2.63344800	-1.70058600	1.19782700
F	-2.72072600	-1.10817800	2.38639100
C	-2.93834100	1.79075200	0.29655200
C	-4.02579300	1.60068900	-0.55240600
F	-4.06882100	0.55338100	-1.37491700
C	-5.07823400	2.50241000	-0.59462300
F	-6.10136100	2.30648000	-1.41256500
C	-5.05266000	3.62167900	0.23104600
F	-6.05152700	4.48451900	0.19688500
C	-3.97796900	3.84178400	1.08522300
F	-3.95651100	4.91009600	1.86681800
C	-2.93698000	2.92582600	1.10558400
F	-1.90423300	3.14546400	1.91769900
C	1.12824800	-1.12014800	-0.03566000
C	1.39938800	-1.72979900	-1.26231200
F	1.92576900	-1.01775800	-2.26786600
C	1.14037100	-3.06433200	-1.55248900
F	1.41451200	-3.56624400	-2.75366000
C	0.57993800	-3.87674200	-0.57658100
F	0.26652900	-5.14194900	-0.84121500
C	0.28811000	-3.32907900	0.66104300
F	-0.33332100	-4.06916700	1.58668300
C	0.55329100	-1.98495200	0.89032900
F	0.07988600	-1.51883500	2.07239400
C	2.68851600	1.09246200	-0.52871200
C	3.84780900	0.33919100	-0.70849300
F	3.86200300	-0.96985300	-0.42519500
C	5.05624800	0.86651900	-1.14541000
F	6.12320800	0.08321200	-1.29387800
C	5.15868800	2.22425900	-1.40508600
F	6.30926900	2.74992100	-1.81911500
C	4.04441400	3.02743600	-1.22230500
F	4.11810300	4.33932700	-1.45549100
C	2.85937400	2.45027100	-0.78428700
F	1.83724400	3.31752300	-0.59266100
H	-1.23209500	0.66287300	1.75005800
C	5.34280400	-0.51302400	3.34331200
C	5.25050100	0.75361900	2.77330200
C	4.02610700	1.22345700	2.30889600
C	2.86634300	0.44274100	2.39024300
C	2.97461700	-0.82071500	2.98179700
C	4.19653200	-1.29386500	3.45191100
H	6.29627000	-0.88372000	3.70548900
H	6.13334100	1.38073300	2.69186900
H	3.96549200	2.21627200	1.86765800
H	2.08775000	-1.43674200	3.08922400
H	4.25166500	-2.27879000	3.90594300
C	1.56255800	0.93585500	1.80818700
H	1.57070200	2.03512100	1.82370900
H	0.74900300	0.62075500	2.47000400

P18_TS

P	2.28945800	0.74485900	-0.17263400
Si	-3.24250400	-0.37423000	2.39876800
B	-0.97575700	-0.21252700	-0.39714800
C	1.41617600	1.30642600	1.35252600
H	1.18628700	0.45672200	1.99339100
H	2.02851000	2.01647700	1.91385800
C	0.09384900	1.94224900	0.87153200
H	-0.17397100	2.75627800	1.54514100
H	0.26727300	2.40410600	-0.10521900
C	-1.00677300	0.88607000	0.81323400
C	-1.87657100	0.85225100	1.84999600
C	-2.94904200	-0.70797400	4.23670100
H	-3.71194800	-1.40901000	4.59212600
H	-3.01204100	0.19895400	4.84237500
H	-1.97054000	-1.16732400	4.40831200

C	-4.92100200	0.48375100	2.26485800	C	4.24770000	-2.66194300	1.24233000
H	-5.14356300	0.84845800	1.25910100	H	4.40790400	-3.18689000	2.18143100
H	-4.96901400	1.33608200	2.94904700	C	3.55119100	-1.44797300	1.27464300
H	-5.70948600	-0.21935700	2.55433600	C	3.57866500	-0.67793900	-2.49103700
C	-3.39287800	-2.08613000	1.60572600	H	4.02766100	-1.27773100	-3.28424500
H	-3.73989500	-2.77313800	2.38489600	H	4.01920500	0.32325800	-2.53342900
H	-2.46353600	-2.49013200	1.19795600	H	2.50829700	-0.59628400	-2.70977100
H	-4.14133800	-2.08628300	0.80900900	C	5.34992500	-4.59663200	0.05222100
C	-1.79600200	1.90338700	2.92064800	H	4.63244500	-5.33953900	-0.30996000
C	-0.90355300	1.78060900	3.99331000	H	5.66715000	-4.89630600	1.05303400
H	-0.26152400	0.90398400	4.04450700	H	6.21869100	-4.61882900	-0.61052300
C	-0.84627700	2.75195800	4.98765500	C	3.10967500	-0.98795400	2.64780100
H	-0.14878700	2.64028700	5.81233200	H	2.07883300	-1.28218400	2.86870300
C	-1.68548900	3.86316100	4.92984600	H	3.18460300	0.08843300	2.78287200
H	-1.64341000	4.62000500	5.70645800	H	3.74119300	-1.46010100	3.40320000
C	-2.57924400	3.99476900	3.87065100	C	-2.48303400	-0.61964700	-0.92003000
H	-3.23476400	4.85862000	3.81702100	F	-3.43546900	1.52379700	-0.43041900
C	-2.63582900	3.02146800	2.87516700	C	-3.56241500	0.27013400	-0.87875100
H	-3.32451500	3.12241400	2.04023600	F	-5.82101600	0.85761300	-1.21765700
C	3.46364200	2.12871600	-0.58421700	C	-4.84261300	-0.04203400	-1.32336200
C	4.55698600	2.44578400	0.25898700	F	-6.31118200	-1.58466100	-2.33880400
C	5.41035800	3.49259500	-0.09041500	C	-5.09712500	-1.27773900	-1.89269200
H	6.24705900	3.71924700	0.56594300	F	-4.25961300	-3.36965900	-2.57635500
C	5.23179800	4.25007400	-1.24288600	C	-4.05116300	-2.17947000	-2.01333200
C	4.15941500	3.92558400	-2.06457400	F	-1.85997600	-2.78714500	-1.69920000
H	3.99811300	4.49508500	-2.97636000	C	-2.79339700	-1.83619700	-1.53402700
C	3.27713800	2.88740900	-1.76153800	C	-0.02436500	-1.54481200	-0.05863400
C	4.87448500	1.71968700	1.54115500	F	-0.34235300	-1.42616100	2.32456000
H	5.80536000	2.10103000	1.96393000	C	0.15326800	-2.04593900	1.23625300
H	4.99023400	0.64566700	1.38586300	F	1.03236100	-3.54829000	2.83014800
H	4.09511500	1.87175400	2.29277100	C	0.86963300	-3.19697000	1.55265900
C	6.15226800	5.39419600	-1.57347100	F	2.23989000	-4.98156500	0.84577400
H	7.16791700	5.19882800	-1.22183800	C	1.49502200	-3.91805300	0.55587700
H	5.80766600	6.31564500	-1.09311100	F	1.99396600	-4.12309600	-1.73219300
H	6.18792600	5.57530200	-2.64994100	C	1.37552500	-3.47046300	-0.74777300
C	2.16272400	2.63628500	-2.74402800	F	0.62499100	-1.99763400	-2.32072000
H	2.20591800	3.35995400	-3.55952600	C	0.64303900	-2.32409100	-1.01455300
H	1.17776200	2.73322700	-2.28361900	H	0.75025600	0.57664900	-1.14890700
H	2.23713800	1.63922800	-3.18877200	C	-3.24973300	3.42825100	-3.57505900
C	3.30980700	-0.77449300	0.05622800	C	-3.22991700	2.10965400	-4.02097000
C	3.80368700	-1.33233300	-1.14979700	C	-2.29457600	1.21712500	-3.51143800
C	4.49182800	-2.54028500	-1.12248800	C	-1.36069700	1.60039600	-2.53723700
H	4.84322800	-2.96395400	-2.05997100	C	-1.39460700	2.93022200	-2.10691700
C	4.70378300	-3.23872000	0.06415200	C	-2.32389300	3.83205900	-2.62116400

H	-3.98009400	4.12962900	-3.96452700
H	-3.94496100	1.77155500	-4.76436400
H	-2.29902900	0.18622900	-3.85869200
H	-0.71308900	3.28929700	-1.34665600
H	-2.32690100	4.85485100	-2.25804300
C	-0.39756800	0.52987400	-2.05547500
H	-0.65164800	-0.40097700	-2.55590900
H	0.55693600	0.73283700	-2.56792200

P18_P

P	2.61096700	0.57556000	-0.16912000
Si	-3.09809000	0.17685700	2.58443700
B	-1.25239000	-0.23498000	-0.64271700
C	1.38480200	1.20896600	1.01796300
H	1.04382400	0.37907400	1.63649000
H	1.87694200	1.93987700	1.66633900
C	0.16925100	1.82311200	0.26629900
H	0.02166000	2.83851000	0.63840900
H	0.39510100	1.91346900	-0.79715000
C	-1.05723600	0.92987500	0.49840100
C	-1.74840200	1.15724000	1.64105700
C	-2.52323500	0.14331900	4.39126400
H	-3.25749900	-0.41818100	4.97882700
H	-2.43008400	1.13963100	4.82915200
H	-1.56022300	-0.36866000	4.49522300
C	-4.72356200	1.13554400	2.54846500
H	-5.17299900	1.16026100	1.55310300
H	-4.57457200	2.16813300	2.87872300
H	-5.43474600	0.66472500	3.23569900
C	-3.47779900	-1.63504400	2.19649000
H	-3.80691300	-2.09705100	3.13401700
H	-2.61730400	-2.19755400	1.83237700
H	-4.29036300	-1.74006000	1.47495300
C	-1.43301500	2.39026800	2.44203600
C	-0.46801200	2.40871700	3.45734500
H	0.05501000	1.48707100	3.70526100
C	-0.18957000	3.57786500	4.16117600
H	0.55947800	3.56980500	4.94770400
C	-0.87650900	4.75302900	3.86577400
H	-0.66271800	5.66381500	4.41587300
C	-1.84374900	4.74844700	2.86347400
H	-2.38440000	5.65969900	2.62628600
C	-2.12044900	3.57844800	2.16080000
H	-2.86253600	3.56846300	1.36596100
C	3.68130100	1.91620100	-0.76500100
C	4.71176700	2.40164400	0.06995000
C	5.53082700	3.42360700	-0.40075200
H	6.32595600	3.79385600	0.24129700
C	5.35983800	3.98140100	-1.66607600
C	4.33476700	3.48905700	-2.46900400
H	4.18842900	3.91420700	-3.45834200
C	3.47997800	2.46846800	-2.05129000
C	4.95554400	1.86925800	1.46015800
H	5.81504100	2.36826500	1.90981300
H	5.15296800	0.79368500	1.46128200
H	4.09772400	2.05650500	2.11455300
C	6.24119900	5.10556700	-2.13903400
H	7.24885500	5.01830600	-1.72694200
H	5.83372400	6.06932400	-1.81734200
H	6.31167300	5.12217600	-3.22846200
C	2.38577000	2.04437300	-3.00424200
H	2.53785200	2.52120500	-3.97336400
H	1.39650700	2.35036300	-2.64895000
H	2.35456500	0.96461300	-3.17640300
C	3.52184700	-0.92445400	0.28608700
C	4.10153100	-1.63717800	-0.79166600
C	4.75494700	-2.83688100	-0.52808400
H	5.18428300	-3.38974000	-1.35913700
C	4.84891500	-3.35693800	0.76083200
C	4.27283400	-2.63716900	1.80334800
H	4.32591100	-3.03475300	2.81343300
C	3.60278300	-1.43011400	1.60187000
C	3.98574300	-1.19219700	-2.22906800
H	4.61604600	-1.81659200	-2.86317600
H	4.29720800	-0.15295700	-2.37106100
H	2.95707800	-1.30386500	-2.59243500
C	5.50081000	-4.68812300	1.00838900
H	4.76134600	-5.48307400	0.87228800
H	5.88838500	-4.75918700	2.02662400
H	6.31960800	-4.86668100	0.30818700
C	2.96978200	-0.80025500	2.82050000
H	1.92169600	-1.10727200	2.91400500
H	3.00978700	0.28889900	2.81867900
H	3.48607800	-1.14722900	3.71717500
C	-2.77484100	-0.86475100	-0.69858700
F	-3.78975700	1.27636000	-0.40249300
C	-3.90049200	-0.03797300	-0.64602300
F	-6.22513700	0.37302200	-0.74577600

C	-5.20371300	-0.48257400	-0.83210200
F	-6.68112500	-2.26399900	-1.30894100
C	-5.44042700	-1.81874500	-1.11369700
F	-4.55963800	-3.97091700	-1.48489700
C	-4.35858200	-2.68102200	-1.20422400
F	-2.09637400	-3.11393700	-1.10887300
C	-3.07301300	-2.19224800	-1.00169600
C	-0.11179400	-1.45841400	-0.36205800
F	-0.41310300	-1.43092100	2.01565200
C	0.12150600	-1.99160600	0.91023500
F	1.11897100	-3.49232900	2.44504700
C	0.92233000	-3.09632400	1.18382500
F	2.36020200	-4.79440500	0.38730100
C	1.57441400	-3.74334300	0.15062900
F	2.14453600	-3.76145200	-2.13171500
C	1.44027800	-3.23008700	-1.12748900
F	0.65035500	-1.69101200	-2.62606900
C	0.63238200	-2.12062200	-1.34585000
C	-3.95817600	3.39361000	-3.29288300
C	-4.12865500	2.06352300	-3.66522500
C	-3.16602200	1.11577200	-3.33272800
C	-2.02002400	1.45664100	-2.60226200
C	-1.85903600	2.80371100	-2.25351000
C	-2.81181000	3.75942500	-2.59342500
H	-4.70650600	4.13666000	-3.54899300
H	-5.01287400	1.76153500	-4.21858200
H	-3.30843500	0.07795500	-3.62643500
H	-0.98177500	3.12085100	-1.69699900
H	-2.65968100	4.79518200	-2.30368700
C	-1.05324600	0.37693800	-2.19277200
H	-1.18871300	-0.45803800	-2.88502800
H	-0.02455400	0.71996200	-2.36491700
H	1.86181100	0.19884200	-1.28489100

P21_TS

P	-2.08516100	0.22353000	0.35505200
C	-2.35477000	1.65422800	-0.74968000
C	-3.50941600	2.44288400	-0.78753300
C	-3.53266200	3.60302700	-1.55337900
C	-2.40996100	3.99320400	-2.28351300
C	-1.24052900	3.23701600	-2.26111600
C	-1.24272400	2.08419700	-1.47543900
O	-0.12848100	1.31220800	-1.36921700
C	1.09685900	1.97662400	-1.40744700
C	2.10439800	1.42495600	-0.62451400
C	3.29217000	2.16574800	-0.59590100
C	3.44319400	3.35422100	-1.30410700
C	2.40714100	3.83720100	-2.09647400
C	1.20341000	3.13924300	-2.16894300
C	0.02477600	3.52016300	-3.05976700
C	0.09020200	4.97190700	-3.52976900
C	0.03485400	2.58530300	-4.29085100
B	1.85952800	0.12324400	0.30924400
C	1.14895800	-1.19594600	-0.34895200
C	0.84053500	-2.26766200	0.48637700
F	0.99105600	-2.13039600	1.82269600
C	0.37580600	-3.49544600	0.04433100
F	0.04654600	-4.46099000	0.90726100
C	0.19189200	-3.69121700	-1.31568600
F	-0.29139500	-4.84754100	-1.76927700
C	0.48244700	-2.66006400	-2.19269200
C	0.97017000	-1.45182700	-1.70587200
F	1.26020400	-0.53453700	-2.63746200
F	0.25110700	-2.82013400	-3.49699800
C	3.33812500	-0.47238900	0.78817400
C	4.17126400	-0.87654400	-0.26302700
F	3.75763200	-0.72826400	-1.52930300
C	5.42799300	-1.43906400	-0.10076300
F	6.16013100	-1.79134700	-1.15673500
C	5.92062500	-1.64373900	1.18073700
F	7.12251300	-2.18391500	1.36500900
C	5.13819200	-1.27815700	2.26068500
C	3.88185200	-0.71632100	2.04510600
F	3.21691000	-0.41705000	3.18487300
F	5.58380700	-1.46965800	3.50284000
C	-3.57281500	0.31029900	1.45898300
C	-3.42092700	0.74222100	2.79195500
C	-2.09015700	1.14879400	3.37149400
C	-4.52871900	0.77460100	3.63810200
C	-5.79489000	0.39147300	3.20763200
C	-6.97148700	0.39907900	4.14696100
C	-5.93393900	-0.00956400	1.88280600
C	-4.85710500	-0.06120800	0.99473700
C	-5.17132700	-0.51236400	-0.41460700
C	-2.33645400	-1.40997100	-0.46936400
C	-2.47668400	-1.63067000	-1.85690000
C	-2.21407800	-0.58536900	-2.91208600
C	-2.84965200	-2.90070600	-2.29653700

C	-3.07277700	-3.96381400	-1.42294000
C	-3.44641200	-5.32508300	-1.94333800
C	-2.85393700	-3.74368000	-0.06791900
C	-2.47263200	-2.49384000	0.42406500
C	-2.19381900	-2.39634400	1.90747700
H	-4.37784800	2.16403500	-0.20069600
H	-4.42939600	4.21241800	-1.57693500
H	-2.45046100	4.90288400	-2.87336900
H	4.11659400	1.81722900	0.01940400
H	4.37599000	3.90523600	-1.24285500
H	2.53910100	4.75799900	-2.65437100
H	0.99914200	5.14077400	-4.11182700
H	0.08112900	5.66844200	-2.68601500
H	-0.75359400	5.20125900	-4.18633200
H	0.93799100	2.76433700	-4.88170300
H	0.03715600	1.53557800	-3.98375100
H	-0.84359100	2.76960800	-4.91781400
H	-2.22780100	1.64825600	4.33207900
H	-1.46493600	0.26706100	3.56033900
H	-1.54037800	1.83895600	2.72586000
H	-4.39368200	1.11111200	4.66320000
H	-7.04719400	-0.55596800	4.67683100
H	-6.87282600	1.18602000	4.89815900
H	-7.90842700	0.55247800	3.60691200
H	-6.91887300	-0.29055400	1.51680800
H	-6.22485700	-0.31946200	-0.62951100
H	-4.99139800	-1.58538000	-0.53655500
H	-4.57589000	0.00497100	-1.16879900
H	-2.28358000	-1.03971400	-3.90195500
H	-1.20725800	-0.17202000	-2.80609000
H	-2.92575200	0.24480900	-2.86953000
H	-2.95595400	-3.06570200	-3.36600700
H	-4.10721100	-5.25024800	-2.81022300
H	-3.94629500	-5.92014000	-1.17601000
H	-2.54534000	-5.86244800	-2.25350400
H	-2.95375200	-4.56983100	0.63227700
H	-1.80362600	-3.35229600	2.26304600
H	-1.43308000	-1.64384700	2.13878800
H	-3.09517400	-2.15047300	2.47775500
H	-0.33139500	0.42635800	1.01977300
C	1.60777400	4.90238200	2.49034600
C	0.54962100	4.50928100	1.67569000
C	0.31247400	3.15886300	1.44172200
C	1.12092300	2.17433300	2.01911700
C	2.17645100	2.58103400	2.84000500
C	2.41841000	3.93238900	3.07206400
H	1.80039100	5.95545200	2.66759700
H	-0.08855100	5.25266700	1.20790300
H	-0.50706700	2.87303200	0.78688000
H	2.81576000	1.82977600	3.29271800
H	3.24656800	4.22621700	3.70944100
C	0.92121800	0.71280400	1.72220700
H	-0.00231200	0.37937500	2.24052500
H	1.51552300	0.09050500	2.39326700

P21_P

P	-2.39109000	-0.26653700	-0.26843200
C	-2.45019300	-1.65599800	0.87272400
C	-3.62132800	-2.41607700	0.98748700
C	-3.65572600	-3.49712400	1.85327700
C	-2.53094700	-3.81020700	2.61881700
C	-1.35530000	-3.07199200	2.53037700
C	-1.31768100	-2.00733900	1.62063600
O	-0.21062400	-1.25597700	1.46145300
C	1.04165500	-1.86881500	1.69995300
C	2.12604200	-1.35238400	0.99157500
C	3.33387900	-2.02561600	1.20904100
C	3.43813600	-3.12404000	2.05597100
C	2.32534600	-3.57674400	2.75461000
C	1.09695200	-2.93386100	2.59807100
C	-0.14106200	-3.25364900	3.42763100
C	-0.09301500	-4.64818300	4.04963300
C	-0.23289000	-2.19936800	4.55588000
B	1.95332800	-0.22517000	-0.17438300
C	1.08785200	1.11939400	0.31532200
C	0.75438900	2.10968900	-0.61155000
F	0.97910700	1.91437200	-1.92643800
C	0.21341900	3.34628000	-0.28608900
F	-0.11946700	4.22563300	-1.23976600
C	-0.03193000	3.65217300	1.04187600
F	-0.57040200	4.82711100	1.37950900
C	0.25904000	2.70524900	2.00719400
C	0.82337900	1.48840200	1.63645800
F	1.09944700	0.67494300	2.66845300
F	-0.03659900	2.95395600	3.28736200
C	3.39763900	0.46770800	-0.56057100
C	4.21921800	0.95255400	0.45921600
F	3.84540300	0.80530400	1.74104800

C	5.41968900	1.61471200	0.24461400
F	6.16522100	2.03892500	1.26826700
C	5.83639200	1.85400200	-1.05732600
F	6.98393300	2.49089900	-1.29298000
C	5.03723200	1.43531800	-2.10773300
C	3.84272400	0.77267100	-1.84128000
F	3.11223900	0.45839900	-2.92216700
F	5.41258000	1.68184200	-3.36548200
C	-3.66148700	-0.58474800	-1.52286700
C	-3.28198100	-1.28892400	-2.68582100
C	-1.89076800	-1.83494500	-2.89579500
C	-4.24184700	-1.49478300	-3.67380200
C	-5.55207600	-1.03840500	-3.53878400
C	-6.55498200	-1.24766600	-4.64143700
C	-5.90649300	-0.38647900	-2.36005000
C	-4.98880400	-0.14808100	-1.33686000
C	-5.48646600	0.54661700	-0.08868700
C	-2.59808300	1.41253700	0.37124900
C	-2.77735900	1.71982400	1.73582500
C	-2.51718000	0.74566200	2.85794400
C	-3.14694400	3.02117900	2.06758900
C	-3.31843700	4.01781500	1.10704800
C	-3.68364800	5.41873400	1.51149600
C	-3.04772900	3.70494400	-0.22259600
C	-2.67530500	2.42064600	-0.61571700
C	-2.34551200	2.19599600	-2.07420400
H	-4.48791200	-2.17262200	0.38155500
H	-4.55534600	-4.09604500	1.93673300
H	-2.58019200	-4.64903700	3.30499700
H	4.21556300	-1.69694200	0.66624600
H	4.39219300	-3.62715800	2.17723100
H	2.41781400	-4.42188000	3.42778400
H	0.77475600	-4.73723800	4.70630100
H	-0.03430500	-5.42866800	3.28530200
H	-0.97483700	-4.82529100	4.67154000
H	0.62488400	-2.30612800	5.22637100
H	-0.20836700	-1.18597800	4.14447500
H	-1.15541800	-2.33065600	5.13161700
H	-1.86537400	-2.47243800	-3.78032900
H	-1.15112300	-1.04080000	-3.04439600
H	-1.54914900	-2.43857800	-2.04778600
H	-3.95638700	-2.03615900	-4.57218200
H	-7.57806000	-1.17268700	-4.26758000
H	-6.42736800	-0.49082300	-5.42188400
H	-6.42723000	-2.22669000	-5.10921500
H	-6.93357700	-0.05688300	-2.22382800
H	-6.57125800	0.44210900	-0.02248500
H	-5.24750200	1.61485300	-0.09714900
H	-5.05771200	0.12715300	0.82661500
H	-2.59942700	1.26207600	3.81541600
H	-1.50013100	0.34643400	2.78294100
H	-3.21509500	-0.09719800	2.86420400
H	-3.28032000	3.26943100	3.11731100
H	-4.19711100	5.94496700	0.70410500
H	-2.77295000	5.97521200	1.75199700
H	-4.32443000	5.42274400	2.39594600
H	-3.09862000	4.48595900	-0.97709300
H	-2.00818800	3.13708600	-2.51119000
H	-1.52714200	1.48161700	-2.21463500
H	-3.21189100	1.83904200	-2.63991700
H	-1.15207200	-0.31595200	-0.91170000
C	2.93545500	-4.51221300	-3.30432200
C	2.48731700	-4.57212700	-1.98745100
C	1.93040500	-3.45121200	-1.38007200
C	1.81740400	-2.22949800	-2.06247500
C	2.24696200	-2.19937400	-3.39544400
C	2.80260200	-3.31960800	-4.00761400
H	3.37095800	-5.38639700	-3.77829800
H	2.56499700	-5.49944000	-1.42706100
H	1.56855100	-3.52997800	-0.35869100
H	2.14376400	-1.28067200	-3.96207400
H	3.12971000	-3.25757100	-5.04154600
C	1.17428100	-1.02358800	-1.41364600
H	0.26254000	-1.41003700	-0.93949000
H	0.87092300	-0.32382100	-2.19957100

P22_TS

P	-2.74421400	1.81280700	0.91265100
C	-3.70116500	0.89138800	-0.34223100
C	-4.93979100	1.33849600	-0.81923700
C	-5.67090600	0.58199300	-1.72402700
C	-5.19388700	-0.66399800	-2.12505500
C	-3.97554100	-1.15314200	-1.66207500
C	-3.21569400	-0.33746900	-0.81461900
O	-1.97345200	-0.75648800	-0.40438100
C	-1.23504100	-1.55923200	-1.29352300
C	0.15883700	-1.43242900	-1.30113400
C	0.83340600	-2.27931700	-2.19449900

C	0.16887400	-3.15614200	-3.03940700
C	-1.21877200	-3.23639700	-3.00196900
C	-1.94148500	-2.44731300	-2.11206000
C	-3.44880000	-2.55606800	-1.92192700
C	-3.71455500	-3.39707500	-0.65111200
C	-4.14367600	-3.22361600	-3.10767400
B	1.08398300	-0.50111200	-0.33499100
C	1.45273800	-1.42181000	0.97187300
C	2.53028800	-2.30747900	0.92602700
F	3.33786700	-2.32569200	-0.14425400
C	2.84067300	-3.20446400	1.93706500
F	3.89119200	-4.01549400	1.82860900
C	2.03743400	-3.26728900	3.06815000
F	2.31526300	-4.12598200	4.04531000
C	0.94012100	-2.42866700	3.15796000
C	0.67292100	-1.55365800	2.11314800
F	-0.44933600	-0.80118000	2.27535500
F	0.13931000	-2.47622300	4.22366000
C	2.35458500	0.17377800	-1.09033300
C	3.56833400	0.47001100	-0.47291100
F	3.79620500	0.10328600	0.79389800
C	4.59265500	1.17681300	-1.09095800
F	5.73294500	1.42446000	-0.44699000
C	4.41720500	1.64560500	-2.38363300
F	5.38287500	2.33337800	-2.98877200
C	3.21548500	1.40556100	-3.03333500
C	2.21932600	0.69304400	-2.37788200
F	1.05386500	0.57345400	-3.03518200
F	3.02050500	1.87284800	-4.26750200
C	-2.95675000	3.60527400	0.50563700
C	-2.26309600	3.88662200	-0.83054300
C	-2.33598100	4.43938900	1.63131800
C	-3.65144000	1.49929700	2.50973900
C	-5.05141300	2.10761200	2.55898200
C	-3.69170100	-0.00916000	2.77372900
H	-5.32741200	2.29614600	-0.48375700
H	-6.61985200	0.94821000	-2.10014000
H	-5.78941100	-1.26542800	-2.80307700
H	1.91866400	-2.23153800	-2.22819500
H	0.73038800	-3.77972300	-3.72746400
H	-1.73470800	-3.92479500	-3.66190000
H	-3.31197200	-4.40558000	-0.78385200
H	-4.79041800	-3.46534200	-0.46017400
H	-3.23249400	-2.94775700	0.22160200
H	-3.77244500	-4.24221800	-3.24012200
H	-5.21868000	-3.30301500	-2.92603800
H	-3.98131800	-2.66884900	-4.03650700
H	-4.02412000	3.84377800	0.42134600
H	-2.39610800	4.93645000	-1.10790600
H	-2.66275400	3.26627100	-1.63786600
H	-1.18641700	3.69832500	-0.74545100
H	-2.38255400	5.50200900	1.37497600
H	-1.28140200	4.17307800	1.77369100
H	-2.85604800	4.30242200	2.58419300
H	-3.01824000	1.97437800	3.27013500
H	-5.49952800	1.92568000	3.54106300
H	-5.04358200	3.18868300	2.39207700
H	-5.69952700	1.64421100	1.80857500
H	-4.14006200	-0.20083600	3.75310400
H	-4.30560500	-0.51234100	2.01893300
H	-2.69582200	-0.45809800	2.75964100
H	-1.02177500	1.29398300	0.54471600
C	2.33161600	4.01218400	2.26365500
C	1.67904700	2.99187400	2.95006400
C	0.98751700	2.00455800	2.25327400
C	0.94588100	2.02088100	0.85455700
C	1.59850900	3.05458100	0.17294400
C	2.28786300	4.04025300	0.87177400
H	2.87123900	4.77981100	2.80826100
H	1.70755000	2.96139100	4.03473200
H	0.47370900	1.21482700	2.79358300
H	1.57197500	3.07511700	-0.91469900
H	2.79413300	4.83075400	0.32687700
C	0.17531100	0.99839900	0.10067700
H	-0.35569300	0.20207100	0.62999300
H	-0.12860300	1.23026700	-0.91985000

P22_P

P	3.11703700	0.77622100	1.30231100
C	3.65244800	-0.87019700	0.74023400
C	4.91032900	-1.39222500	1.03853400
C	5.30123300	-2.59661200	0.45576600
C	4.44679500	-3.23358100	-0.44389800
C	3.18176000	-2.72633300	-0.76385800
C	2.77828500	-1.55480100	-0.12016800
O	1.54519200	-0.97884400	-0.29802300
C	0.59298900	-1.55586700	-1.19004400
C	-0.68122100	-0.96766200	-1.19566700

C	-1.52295200	-1.42630600	-2.22261100
C	-1.16215100	-2.44958200	-3.09446400
C	0.06993600	-3.07586100	-2.95246000
C	0.98347900	-2.63565400	-1.99014500
C	2.30726400	-3.38740800	-1.82212600
C	3.08537700	-3.38869600	-3.15940200
C	2.00333400	-4.84347500	-1.39775100
B	-1.20234800	0.12835700	-0.07595700
C	-0.50797700	1.58937800	-0.47097500
C	-0.61767500	2.06884000	-1.78177100
F	-1.37093300	1.37668400	-2.69988600
C	-0.04635600	3.24818800	-2.23659200
F	-0.18437700	3.63881000	-3.54060000
C	0.67819900	4.04123500	-1.35949200
F	1.29203800	5.18583500	-1.79161700
C	0.77607800	3.64313200	-0.04064200
C	0.18046600	2.45730700	0.36954300
F	0.33376500	2.19261500	1.73257400
F	1.52091600	4.38683500	0.85199500
C	-2.84944900	0.29515300	-0.08881300
C	-3.49699800	1.51653700	0.11096800
F	-2.78074100	2.69896600	0.19183400
C	-4.86892600	1.65934600	0.27196400
F	-5.42351100	2.89830300	0.46515100
C	-5.67935100	0.53920400	0.24558800
F	-7.03302100	0.65428400	0.41233100
C	-5.09568200	-0.70169600	0.06005400
C	-3.72034400	-0.80226200	-0.09814400
F	-3.24591900	-2.08441300	-0.25541700
F	-5.88214600	-1.82365000	0.05016800
C	3.65685400	1.18723700	3.04411500
C	2.93610800	0.23072500	4.00334200
C	3.31058200	2.65940400	3.31345100
C	3.71977200	2.05151100	0.06144700
C	5.24500100	2.16600000	0.14957400
C	3.25086700	1.65261200	-1.34364800
H	5.57834200	-0.86562400	1.71278300
H	6.26904600	-3.02435000	0.68407900
H	4.77568400	-4.14994200	-0.92239500
H	-2.49566300	-0.96500000	-2.33621800
H	-1.85142700	-2.77521600	-3.86452600
H	0.32736900	-3.91183700	-3.59432200
H	2.49362600	-3.86489500	-3.94483100
H	4.02567600	-3.94106700	-3.06336600
H	3.31006100	-2.36471900	-3.47113100
H	1.40171500	-5.34092100	-2.16284400
H	2.92836400	-5.41454300	-1.26602400
H	1.44275600	-4.85800000	-0.45887500
H	4.74101500	1.03630700	3.09924300
H	3.24159300	0.44877800	5.03034300
H	3.16871500	-0.81678500	3.79150000
H	1.85076200	0.36395400	3.93708500
H	3.51381800	2.88689100	4.36352400
H	2.25180900	2.85867500	3.11942700
H	3.90331300	3.34359500	2.69958400
H	3.24949800	2.99178700	0.36906600
H	5.58930500	2.91146100	-0.57271700
H	5.58517700	2.48006200	1.14150200
H	5.72124100	1.21218000	-0.10272700
H	3.48892100	2.46101800	-2.04162600
H	3.76887000	0.74734600	-1.67882200
H	2.17435900	1.46161200	-1.38746800
H	1.71282000	0.75620500	1.33494800
C	-2.66196500	-4.06327800	2.64033600
C	-3.27425300	-2.85356800	2.96820200
C	-2.66598600	-1.64584600	2.62666500
C	-1.44686600	-1.61843200	1.93375500
C	-0.83255500	-2.84302100	1.63400300
C	-1.43223500	-4.05220200	1.97965300
H	-3.13566600	-5.00335700	2.89917100
H	-4.22539800	-2.85006400	3.48966900
H	-3.14574000	-0.70511900	2.88508700
H	0.12101800	-2.83964400	1.11249700
H	-0.94429000	-4.98824500	1.72806800
C	-0.84298700	-0.31376900	1.49025300
H	-1.18963200	0.48537800	2.15559700
H	0.24559700	-0.38351800	1.60657600

P23-TS

C	1.42336900	3.00008100	-1.34930900
H	2.49922500	3.15368700	-1.46618100
H	1.02354200	3.89501600	-0.86477400
H	0.97168500	2.92675200	-2.34318300
C	1.13044700	1.75931600	-0.54168400
C	0.01264600	0.99596700	-0.58726200
P	2.32816800	1.15148700	0.66321400
C	3.73361900	0.34573700	-0.15973500
C	4.04465100	0.57208100	-1.50494400

H	3.46330000	1.27500100	-2.09181200
C	5.06941600	-0.14803200	-2.10878600
H	5.29288400	0.01326200	-3.15789800
C	5.78665700	-1.09432900	-1.37952600
H	6.57349500	-1.66578900	-1.86065200
C	5.48338600	-1.32006000	-0.03949700
H	6.02941900	-2.06880300	0.52510100
C	4.45430800	-0.60672600	0.56946100
H	4.19358600	-0.80793200	1.60597200
C	2.94634600	2.62729000	1.52595600
C	4.29716500	2.80169400	1.83476000
H	5.02754400	2.06489600	1.51293000
C	4.70592500	3.92788600	2.54388600
H	5.75693300	4.06378500	2.77709100
C	3.77288000	4.87884300	2.94711600
H	4.09522000	5.75609500	3.49844700
C	2.42427700	4.70696700	2.64103800
H	1.69584800	5.44886300	2.95150400
C	2.00815400	3.58454000	1.93430900
H	0.95602600	3.45590700	1.68486600
B	-0.18261100	-0.40878900	0.24490200
C	-1.06480900	1.39272400	-1.53781500
F	-1.25543500	3.53715300	-0.55647200
C	-1.64804400	2.65934800	-1.48399400
F	-3.19564200	4.26856100	-2.24984400
C	-2.67074900	3.04877500	-2.33535100
F	-4.13541100	2.50798000	-4.09729700
C	-3.15995900	2.14817600	-3.27172900
F	-3.09972500	-0.00292100	-4.22224100
C	-2.62823300	0.86847800	-3.33485800
F	-1.16583300	-0.75315300	-2.54675600
C	-1.60761900	0.50290900	-2.46699000
C	0.91857300	-1.52521700	-0.21690300
F	1.35400700	-0.53006000	-2.35336100
C	1.58698300	-1.48659600	-1.44314600
F	3.18814300	-2.27278100	-2.99222100
C	2.58247000	-2.38793300	-1.81027800
F	3.95340600	-4.21824700	-1.24578200
C	2.96935900	-3.38198200	-0.93178500
F	2.65979000	-4.45013900	1.14506800
C	2.31937500	-3.48626700	0.29022800
F	0.71098700	-2.78099800	1.78472900
C	1.31479200	-2.58436200	0.60231500
C	-1.71196400	-0.88874200	0.29839300
F	-1.29854200	-3.17489900	-0.22193800
C	-2.15140000	-2.19444200	0.09964300
F	-3.84296400	-3.84535600	0.05537500
C	-3.48015000	-2.57754900	0.24621700
F	-5.70189500	-1.99348100	0.76373200
C	-4.43153600	-1.63662200	0.60520900
F	-4.94697300	0.59732700	1.15377200
C	-4.04665900	-0.31685000	0.79905900
F	-2.39535500	1.31011800	0.84933000
C	-2.71612000	0.02370300	0.62669100
H	1.08315700	0.22324300	1.33545300
C	-3.68443800	-0.73317300	4.27249600
C	-3.19691000	0.55774600	4.09485600
C	-1.96023700	0.75761900	3.48905200
C	-1.19458700	-0.31945200	3.03107100
C	-1.67914200	-1.61503400	3.24979400
C	-2.91342400	-1.81772400	3.85691800
H	-4.65224400	-0.89433000	4.73609700
H	-3.78013300	1.41262100	4.42187400
H	-1.59450300	1.76926400	3.33559100
H	-1.09974300	-2.46833200	2.91830600
H	-3.27760700	-2.83028400	4.00185400
C	0.12739400	-0.04187100	2.36115600
H	0.39051200	0.98663500	2.64023300
H	0.88810200	-0.71133700	2.77542400

P23_P

C	-1.62401100	-3.12371600	-0.74455000
H	-0.74604700	-3.58787400	-1.19272700
H	-2.43889400	-3.18243700	-1.47226400
H	-1.91075600	-3.72042000	0.13019300
C	-1.32773400	-1.68843500	-0.35956300
C	-0.12413900	-1.07111700	-0.35564500
P	-2.82343800	-0.82088900	0.12821200
C	-4.23784400	-1.17384800	-0.94192100
C	-4.98575000	-2.33537500	-0.71097600
H	-4.72399000	-3.00103900	0.10707100
C	-6.07377400	-2.62988500	-1.52265300
H	-6.65010400	-3.53126100	-1.34421600
C	-6.42460400	-1.76289400	-2.55556100
H	-7.27876200	-1.99090800	-3.18478000
C	-5.68624300	-0.60484900	-2.78068800
H	-5.96126400	0.07050600	-3.58350600
C	-4.58755600	-0.30397100	-1.97987900

H	-4.00804100	0.59139100	-2.17841100
C	-3.30218700	-1.32586700	1.79678000
C	-4.55921300	-0.96698700	2.29509600
H	-5.25824000	-0.41144400	1.67545200
C	-4.91323300	-1.33128200	3.58816300
H	-5.88322700	-1.04752200	3.98193500
C	-4.02150000	-2.05960400	4.37446200
H	-4.30146400	-2.34388600	5.38352600
C	-2.77504400	-2.42309900	3.87374400
H	-2.08345500	-2.98922900	4.48794400
C	-2.40567600	-2.05439400	2.58294600
H	-1.42728400	-2.32212500	2.19125000
B	0.32031600	0.46624500	0.18834700
C	1.02269800	-1.94249600	-0.75094400
F	0.70878600	-3.39313800	1.08714400
C	1.43879700	-3.01435100	0.03079800
F	2.98936900	-4.72185100	0.53954300
C	2.61467100	-3.70462100	-0.23115400
F	4.52376500	-3.98068800	-1.57765000
C	3.39982500	-3.32615100	-1.31180200
F	3.75746900	-1.90670200	-3.15594600
C	3.00396900	-2.26752000	-2.12218800
F	1.47153500	-0.56501900	-2.60069800
C	1.82279100	-1.60043100	-1.83793600
C	-0.30874200	1.71598900	-0.68757100
F	-1.71794100	0.45314500	-2.15164200
C	-1.27285400	1.64685200	-1.68430700
F	-2.86484500	2.56556000	-3.17274900
C	-1.89006000	2.74797300	-2.27053100
F	-2.07350800	5.09325300	-2.44842900
C	-1.49968100	4.02213100	-1.90535100
F	-0.06067200	5.37631000	-0.63116100
C	-0.48311300	4.15935200	-0.96791600
F	1.11403800	3.25417900	0.42255000
C	0.08987800	3.02957000	-0.40303400
C	1.97412200	0.51857600	0.18635800
F	2.21612000	1.99004100	-1.67500800
C	2.76284900	1.21637900	-0.72822300
F	4.83669600	1.84231100	-1.67664500
C	4.15202100	1.14276500	-0.77243300
F	6.15591400	0.23902900	0.08508300
C	4.82878700	0.32764700	0.11959900
F	4.71768600	-1.21354300	1.90147700
C	4.09662800	-0.40153500	1.04421000
F	2.08398600	-1.07319500	1.95161800
C	2.71430400	-0.28380200	1.05395800
H	-2.65016400	0.56454300	0.09803500
C	0.84059100	4.10271900	3.96919200
C	1.67687200	2.98891400	3.95600600
C	1.31030300	1.84257500	3.25912000
C	0.10245400	1.78521000	2.55630400
C	-0.74121100	2.89985900	2.60568500
C	-0.37582500	4.05165600	3.29487900
H	1.13033400	4.99836900	4.50919600
H	2.62000800	3.01250500	4.49373400
H	1.95891600	0.97126200	3.26620200
H	-1.69360700	2.86552100	2.07890100
H	-1.04230600	4.90882100	3.30861400
C	-0.27706100	0.57404400	1.75072500
H	0.01809800	-0.32713300	2.29562800
H	-1.37239600	0.56136000	1.72673400

P24_P

P	2.16441900	-0.48012400	0.37196800
B	-0.97131300	0.83872100	0.77893600
C	0.66371700	-0.98086600	-0.52616200
C	-0.56168800	-0.45907900	-0.22767800
C	3.12978100	-1.95874400	0.81998500
C	4.14544300	-2.46024200	-0.01824700
C	4.85752300	-3.58208700	0.40512100
H	5.63524200	-3.97758300	-0.24325800
C	4.59248100	-4.21994600	1.61424900
C	3.55794100	-3.72515500	2.40665800
H	3.31092200	-4.22929600	3.33746100
C	2.81468400	-2.60814400	2.03336000
C	4.46457700	-1.88645200	-1.37566000
H	5.06336800	-2.59947800	-1.94500000
H	5.02295500	-0.94881400	-1.30471700
H	3.55085400	-1.70083300	-1.94909300
C	5.40023200	-5.41009700	2.05697600
H	4.78701400	-6.11604000	2.62148500
H	6.22197200	-5.09301600	2.70710300
H	5.83541000	-5.93385600	1.20339100
C	1.67170800	-2.16898100	2.91279900
H	0.74579300	-2.07348800	2.33643100
H	1.86524800	-1.20297100	3.39461500
H	1.49570400	-2.90030400	3.70274900
C	3.32204400	0.78924700	-0.23296100

C	3.35600900	1.27914100	-1.55229600
C	4.30235500	2.25199100	-1.87672400
H	4.31420100	2.64162600	-2.89133900
C	5.19504000	2.76326100	-0.94038900
C	5.13530700	2.26877000	0.36093400
H	5.80862800	2.66979300	1.11375100
C	4.21797600	1.29260900	0.74223900
C	2.41493800	0.83500600	-2.63885300
H	2.72174100	-0.12049200	-3.07634300
H	2.39593200	1.57914300	-3.43717000
H	1.39505600	0.72410600	-2.27331200
C	6.14524200	3.87044900	-1.30285600
H	5.65705200	4.83594700	-1.13781400
H	6.43615200	3.81716500	-2.35406500
H	7.04709200	3.83965300	-0.68782300
C	4.18846600	0.88659700	2.19742600
H	4.26030200	-0.19611600	2.33517100
H	3.27251500	1.23904800	2.68355800
H	5.02711600	1.34421000	2.72335800
C	0.86471400	-2.07352100	-1.53950400
C	1.27139700	-3.36525800	-1.18438300
H	1.43076400	-3.61604000	-0.14205000
C	1.44035300	-4.34711700	-2.15757900
H	1.75642300	-5.34201200	-1.86142000
C	1.18783500	-4.06245800	-3.49547000
H	1.31907700	-4.82992200	-4.25124400
C	0.73860200	-2.79387500	-3.85643300
H	0.50752900	-2.56937000	-4.89251000
C	0.57635400	-1.81131500	-2.88724100
H	0.19599800	-0.83199700	-3.16460200
C	-1.66149700	-1.24322800	-0.87684300
C	-1.93877300	-2.53817200	-0.44338200
F	-1.15180300	-3.13002900	0.46498200
C	-3.01641600	-3.26507300	-0.92146600
F	-3.25905300	-4.49228600	-0.46692800
C	-3.83499100	-2.71097300	-1.89560300
F	-4.86535500	-3.40071300	-2.37282900
C	-3.55445200	-1.44466600	-2.38797900
F	-4.31329700	-0.92228400	-3.34814100
C	-2.47577500	-0.73279300	-1.88201800
F	-2.22289000	0.46603800	-2.40946700
C	0.22409900	1.97780100	0.53098800
C	0.39097800	2.52305500	-0.74344600
F	-0.39246700	2.09360400	-1.74423100
C	1.31758400	3.49756600	-1.07293500
F	1.45698800	3.91417900	-2.33379200
C	2.16583200	3.98824200	-0.09060600
F	3.10720100	4.88130300	-0.39454600
C	2.05872800	3.48377200	1.19153700
F	2.92424400	3.86546900	2.13473900
C	1.10585100	2.50723100	1.46962900
F	1.16175700	2.03259900	2.73470600
C	-2.46310500	1.49795100	0.40740600
C	-2.68498000	2.86760700	0.21980500
F	-1.68795500	3.76835500	0.28862400
C	-3.92637700	3.44834800	-0.02086500
F	-4.02581500	4.76744100	-0.19494600
C	-5.06073300	2.65939400	-0.06915400
F	-6.25708700	3.19548700	-0.29750000
C	-4.91863400	1.30013300	0.14524600
F	-5.99101500	0.50764700	0.13502800
C	-3.65982300	0.76513500	0.39169900
F	-3.68845800	-0.55541800	0.63475600
C	-4.49113700	-1.73009100	3.78730700
C	-4.46182100	-0.33852200	3.82064300
C	-3.32750600	0.34796000	3.39903900
C	-2.20399300	-0.33419400	2.91796100
C	-2.23757000	-1.73099900	2.92554100
C	-3.36823100	-2.42440500	3.34531700
H	-5.37710500	-2.26757100	4.10949900
H	-5.32555100	0.21521800	4.17624300
H	-3.31442000	1.43545800	3.42074100
H	-1.37603700	-2.28719800	2.57179400
H	-3.37274000	-3.51032800	3.32327200
C	-1.02096900	0.43320200	2.39559900
H	-0.99852000	1.39084600	2.92498900
H	-0.10295700	-0.08499600	2.69498100
H	1.72667200	0.01714500	1.59849100

P25_TS

C	-2.59963900	-1.71979700	3.78199400
C	-1.42735200	-2.34840600	4.10324200
C	-0.30336200	-2.30381700	3.24205800
C	-0.31502700	-1.51295200	2.04117100
C	-1.59081300	-0.92373100	1.70958500
C	-2.68175500	-1.05607600	2.54754600
H	0.78730700	-3.69666300	4.48217100
H	-3.46636200	-1.77686500	4.43125100

H	-1.34601300	-2.93141000	5.01683300
C	0.81751000	-3.10900300	3.56897300
C	0.89804900	-1.43575500	1.24734800
H	-3.64426800	-0.66769700	2.22678300
C	1.90568200	-2.31903500	1.59276000
C	1.88283800	-3.16447200	2.72067300
H	2.79872400	-2.35000800	0.97661800
H	2.72936100	-3.81192000	2.92463300
P	-1.93359300	-0.37225100	0.00177400
B	1.25835500	-0.42536300	0.00959500
C	2.90230900	-0.16873500	-0.19107100
C	3.73852300	-0.02529500	0.92702400
F	3.26064800	-0.13320300	2.17269200
C	5.09755500	0.26151400	0.86386700
F	5.81763100	0.34231700	1.98205800
C	5.69850300	0.49389700	-0.36106200
F	6.99524900	0.77464700	-0.44378900
C	4.90703700	0.45632700	-1.49487000
C	3.55156200	0.17012600	-1.38262600
F	2.88888300	0.28775900	-2.55177000
F	5.43561600	0.73009400	-2.68768300
C	0.77472200	1.14004600	0.13199100
C	0.59666100	1.99623800	-0.95660000
F	0.49587000	1.50677500	-2.20598200
C	0.44688200	3.36896900	-0.84783500
F	0.22493700	4.12153300	-1.92644500
C	0.46640000	3.95938800	0.40601000
F	0.23385900	5.26428000	0.53122600
C	0.64778700	3.16252300	1.52161000
C	0.80365200	1.78949500	1.36584100
F	0.94126100	1.09833300	2.50449400
F	0.60463100	3.70179500	2.74073100
C	-3.52662900	-1.24402000	-0.41344100
C	-4.81265800	-0.65602800	-0.36508200
C	-5.13882200	0.72652900	0.15764300
C	-5.91050800	-1.40893600	-0.79575800
C	-5.80201900	-2.71887200	-1.24622500
C	-7.00395500	-3.48057500	-1.73697400
C	-4.54001500	-3.30272900	-1.22100200
C	-3.41323300	-2.59363800	-0.81506800
C	-2.09688700	-3.32334700	-0.79031400
C	-2.30472900	1.41420000	-0.22434800
C	-2.38768400	2.38963600	0.79026800
C	-2.27263700	2.11652200	2.27275000

C	-2.62034500	3.71648300	0.41829200
C	-2.75139900	4.11608200	-0.90722700
C	-2.86090400	5.57252200	-1.26621300
C	-2.69297300	3.13179400	-1.89036800
C	-2.47208400	1.79221600	-1.57832500
C	-2.43524700	0.80015500	-2.71782000
H	-6.20345900	0.77430900	0.39565600
H	-4.58496500	0.97203200	1.06507500
H	-4.92113800	1.51248400	-0.57143500
H	-6.89469700	-0.94758500	-0.75749700
H	-7.08301400	-3.41481300	-2.82709200
H	-6.93468000	-4.53944900	-1.47603600
H	-7.92781300	-3.08053200	-1.31326800
H	-4.42351300	-4.34164900	-1.52102700
H	-2.25447400	-4.40249100	-0.84740500
H	-1.54537300	-3.11874400	0.13212300
H	-1.46011200	-3.05154900	-1.63765400
H	-2.13750000	3.06086200	2.80489700
H	-1.43889900	1.46607400	2.53522400
H	-3.18121300	1.64487600	2.66469200
H	-2.66192800	4.47155000	1.20093100
H	-3.39661700	6.13427400	-0.49746300
H	-3.37044300	5.71338300	-2.22201800
H	-1.85645700	5.99966700	-1.35413800
H	-2.79350700	3.41569500	-2.93512400
H	-2.45290100	1.32788200	-3.67282500
H	-1.52472300	0.19453100	-2.69827700
H	-3.29220800	0.11948100	-2.68802700
H	-0.34322900	-0.87654800	-0.78740700
C	2.95948200	-4.99750500	-1.98998600
C	3.29894100	-3.87008800	-2.73121200
C	2.58359100	-2.68612800	-2.57787100
C	1.51547300	-2.59546800	-1.67652500
C	1.18234100	-3.74038300	-0.94441900
C	1.89495300	-4.92601800	-1.09771700
H	3.51641700	-5.92148900	-2.10759300
H	4.12334400	-3.90799700	-3.43639200
H	2.85382100	-1.82164300	-3.17369900
H	0.37869900	-3.70450300	-0.22099200
H	1.61581400	-5.79423200	-0.50878500
C	0.73775900	-1.29408000	-1.58852100
H	1.11629300	-0.55982700	-2.28764300
H	-0.24062300	-1.52654100	-2.04245900

P25_P

C	-2.24626200	-1.82829100	3.80823600
C	-1.10723900	-2.58268000	3.90082200
C	-0.11820500	-2.57370000	2.88865800
C	-0.19920500	-1.67941900	1.75838500
C	-1.46783000	-0.98998400	1.65643700
C	-2.44455400	-1.08989700	2.63479000
H	0.96666200	-4.16671900	3.86130600
H	-3.01019300	-1.85508200	4.57641900
H	-0.95350800	-3.24132500	4.75146600
C	0.94326900	-3.50481100	3.00058800
C	0.91863200	-1.60178300	0.84634300
H	-3.41398900	-0.62346800	2.48008100
C	1.86862100	-2.60469800	0.99160400
C	1.88679000	-3.56776300	2.01850500
H	2.71831400	-2.60145000	0.31580900
H	2.68728300	-4.29945000	2.05428200
P	-2.13265800	-0.34215100	0.11089200
B	1.38743700	-0.48451300	-0.28999000
C	2.98568000	-0.05209700	-0.00642100
C	3.67060500	-0.13644100	1.21086800
F	3.11047100	-0.66008900	2.31069000
C	4.97498100	0.31309500	1.40134000
F	5.56593800	0.17673200	2.59058700
C	5.65875600	0.92203500	0.36360100
F	6.90539100	1.36023700	0.53362600
C	5.01334700	1.07922500	-0.85207000
C	3.71194500	0.61807200	-0.99369500
F	3.15266700	0.88052400	-2.19281000
F	5.63466500	1.68734800	-1.86526300
C	0.72958900	1.04504100	-0.17204500
C	0.42485700	1.89439900	-1.23628200
F	0.30389300	1.43363800	-2.49509600
C	0.16361800	3.25358200	-1.09980900
F	-0.18373600	3.99322800	-2.15719800
C	0.19984100	3.83564600	0.15417700
F	-0.13644200	5.11690400	0.31065500
C	0.50113500	3.04597800	1.25218900
C	0.74977600	1.69396800	1.06586500
F	1.02617200	0.99825100	2.17949800
F	0.47474700	3.57708100	2.47832200
C	-3.63313600	-1.31941000	-0.25673000
C	-4.94070200	-0.85405100	-0.00200400
C	-5.28793200	0.49877200	0.57928800
C	-6.01120400	-1.69505100	-0.31483600
C	-5.83540500	-2.96148000	-0.86227200
C	-7.01103500	-3.85096200	-1.16587000
C	-4.53375100	-3.39804400	-1.09770100
C	-3.42752600	-2.60624700	-0.80425300
C	-2.05538700	-3.15027600	-1.11612600
C	-2.49552200	1.41592100	-0.13703300
C	-2.54446700	2.38208700	0.88510500
C	-2.28704700	2.11221500	2.34901000
C	-2.85323800	3.69684400	0.52657400
C	-3.07886700	4.08036900	-0.79033300
C	-3.25509900	5.52792300	-1.15376600
C	-3.04494000	3.09639800	-1.77841200
C	-2.75811900	1.76751000	-1.48576400
C	-2.70699400	0.77891300	-2.62861000
H	-6.32717500	0.49265700	0.91234200
H	-4.66949700	0.76776700	1.43831800
H	-5.17208500	1.29878100	-0.15812400
H	-7.02006000	-1.34088700	-0.11902500
H	-6.88419000	-4.35410900	-2.12757000
H	-7.11285900	-4.62674200	-0.40048600
H	-7.94276100	-3.28276800	-1.19381000
H	-4.37033900	-4.38569500	-1.52175700
H	-2.11342300	-4.22009300	-1.32395200
H	-1.34188100	-3.01226800	-0.29671100
H	-1.63400500	-2.66972500	-2.00709800
H	-2.07915600	3.05486100	2.85774100
H	-1.43736500	1.45163400	2.52260100
H	-3.16078100	1.66301900	2.83511500
H	-2.86254900	4.45461600	1.30602900
H	-3.90173900	5.64774600	-2.02573800
H	-2.27579100	5.95205100	-1.39868200
H	-3.67184500	6.10216100	-0.32387000
H	-3.21072300	3.37812800	-2.81476700
H	-2.94731300	1.28655800	-3.56340400
H	-1.70486700	0.35366800	-2.74423800
H	-3.42091200	-0.04042000	-2.49991800
H	-1.21392600	-0.67313800	-0.88323100
C	3.60367900	-4.68035600	-2.57688400
C	4.11253700	-3.40577800	-2.81438400
C	3.32811300	-2.27976600	-2.58291800
C	2.01811600	-2.39297700	-2.09788400
C	1.51910600	-3.68337700	-1.88273500
C	2.29708400	-4.81366200	-2.11578500

H	4.21500800	-5.55858300	-2.75825000
H	5.12476500	-3.28595900	-3.18887800
H	3.72561700	-1.29224900	-2.79570300
H	0.50689000	-3.80082300	-1.50523700
H	1.88156200	-5.80074300	-1.93484900
C	1.20104600	-1.16110600	-1.80873600
H	1.42662600	-0.42627700	-2.57711500
H	0.14425500	-1.42887700	-1.95378600
